# An Analysis of Glucose Effectiveness in Subjects With or Without Type 2 Diabetes *via* Hierarchical Modeling

**DOI:** 10.3389/fendo.2021.641713

**Published:** 2021-03-29

**Authors:** Shihao Hu, Yuzhi Lu, Andrea Tura, Giovanni Pacini, David Z. D’Argenio

**Affiliations:** ^1^ Department of Biomedical Engineering, University of Southern California, Los Angeles, CA, United States; ^2^ Metabolic Unit, CNR Institute of Neuroscience, Padova, Italy; ^3^ Independent Researcher, Padova, Italy

**Keywords:** intravenous glucose tolerance test, glucose-insulin, minimal model, insulin sensitivity, EM algorithm

## Abstract

Glucose effectiveness, defined as the ability of glucose itself to increase glucose utilization and inhibit hepatic glucose production, is an important mechanism maintaining normoglycemia. We conducted a minimal modeling analysis of glucose effectiveness at zero insulin (*GEZI*) using intravenous glucose tolerance test data from subjects with type 2 diabetes (T2D, n=154) and non-diabetic (ND) subjects (n=343). A hierarchical statistical analysis was performed, which provided a formal mechanism for pooling the data from all study subjects, to yield a single composite population model that quantifies the role of subject specific characteristics such as weight, height, age, sex, and glucose tolerance. Based on the resulting composite population model, *GEZI* was reduced from 0.021 min^–1^ (standard error – 0.00078 min^–1^) in the ND population to 0.011 min^–1^ (standard error – 0.00045 min^–1^) in T2D. The resulting model was also employed to calculate the proportion of the non–insulin-dependent net glucose uptake in each subject receiving an intravenous glucose load. Based on individual parameter estimates, the fraction of total glucose disposal independent of insulin was 72.8% ± 12.0% in the 238 ND subjects over the course of the experiment, indicating the major contribution to the whole-body glucose clearance under non-diabetic conditions. This fraction was significantly reduced to 48.8% ± 16.9% in the 30 T2D subjects, although still accounting for approximately half of the total in the T2D population based on our modeling analysis. Given the potential application of glucose effectiveness as a predictor of glucose intolerance and as a potential therapeutic target for treating diabetes, more investigations of glucose effectiveness in other disease conditions can be conducted using the hierarchical modeling framework reported herein.

## Introduction

Glucose homeostasis is governed by the interaction of many processes, central among these are insulin secretion, insulin action, insulin clearance and glucose effectiveness. Glucose effectiveness, defined as the ability of glucose itself to increase glucose utilization and inhibit hepatic glucose production *via* mass action and other mechanisms (1), exerts an earlier temporal influence relative to insulin in maintaining normoglycemia. It has been shown in (2) that glucose effectiveness may be divided into two components: Basal Insulin Effect (*BIE*) and Glucose Effectiveness at Zero Insulin (*GEZI*). The latter measures the effect of glucose on its own removal in the absence of insulin and thus represents the theoretical insulin-independent glucose disappearance. In normal subjects, it has been reported that glucose effectiveness (independent of dynamic insulin) accounts for 45% to 65% of the total net glucose disposal following an intravenous glucose load (3). In patients with defective insulin action, the impact of insulin on glucose disposal is limited but it is partially compensated by the crucial contribution of glucose effectiveness in the attempt of restoring a good glucose tolerance (1). Given its central role in glucose homeostasis, glucose effectiveness impairment has been proposed as an important indicator of glucose intolerance and as a therapeutic target in the treatment of patients with impaired glucose regulation [Basu et al. (4), Pau et al. (5), Alford et al. (3)]. However, there have been only limited studies aimed at quantifying glucose effectiveness in subjects with normal and impaired glucose tolerance, and there are inconsistencies in those studies (1).

Glucose clamp experiments and the minimal model (MM) approach following an intravenous glucose tolerance test (IVGTT) have been used to quantify glucose effectiveness [Best et al. (6), Ader et al. (7), Dube et al. (1)]. While the glucose clamp method, which involves controlling insulin at near-basal level, is regarded as the gold standard for accessing insulin-mediated glucose disposal, it requires cumbersome experiments and trained research teams. In contrast, the MM analysis is based on a simpler IVGTT or an insulin modified IVGTT (IM-IVGTT) (8), and when coupled with a method for model-based statistical estimation, provides estimates of whole-body glucose disposal indices representing both glucose effectiveness and insulin sensitivity [Bergman et al. (9), Henriksen et al. (10)]. While the many applications of the MM reported in the literature have largely focused on questions related to insulin sensitivity, the MM has also been used to better understand the role of glucose effectiveness in glucose homeostasis in healthy and disease conditions. For example, Henriksen et al. (11) analyzed IVGTT data of 20 normoglycemic first degree relatives of type 2 diabetes (T2D) patients and another 20 matched subjects, where they observed an increased glucose effectiveness in the relatives. The study by Lorenzo et al. (12) assessed whether glucose effectiveness estimated *via* MM analysis in healthy participants could predict the future occurrence of T2D. More recently, Morettini et al. (13) analyzed results from a collection of previous studies in subjects with normal glucose tolerance, focusing on factors associated with differences in glucose effectiveness including body mass index. To explore pathogenic factors in type 2 diabetes, Taniguchi et al. (14) analyzed IM-IVGTT data from 11 healthy subjects and 9 T2D patients, and concluded that diminished glucose effectiveness is partially responsible for glucose intolerance. Similarly, Welch et al. (15) observed a decrease in glucose effectiveness in diabetic subjects based on MM analysis of 21 subjects. These studies using the MM to assess glucose effectiveness have involved either only non-diabetic (ND) subjects [e.g., Henriksen et al. (11), Lorenzo et al. (12), Morettini et al. (13)], or included only a small number of subjects with T2D [e.g., Taniguchi et al. (14) and Welch et al. (15)]. Moreover, these studies analyzed the data from subjects separately, which limits the ability of the analysis to define an overall composite model of the population that incorporates the role of anthropomorphic and pathophysiological factors on MM parameters.

To address these limitations, we conducted a MM analysis of glucose effectiveness using a large set of data obtained from previously conducted studies that included both ND subjects (n=343) and those with T2D (n=154). A hierarchical statistical analysis was performed using the MM, which provides a formal mechanism for a simultaneous modeling analysis of the data from all study subjects, yielding a single composite model that quantifies the role of subject specific characteristics such as weight, height, age, sex, and glucose tolerance status.

## Materials and Methods

### Clinical Study Data

This study involves a pooled analysis of data from previous studies, each performed following the Declaration of Helsinki and upon approval of the respective institutional ethics committees, in which subjects were administered either a regular intravenous glucose tolerance test (IVGTT) or an insulin-modified intravenous glucose tolerance test (IM-IVGTT). A total of 44 study groups was included in the analysis, comprising 497 different subjects as summarized in **Table 1**, which also summarizes sex, age and other anthropometric characteristics. Subjects with type 2 diabetes (T2D) and without diabetes (ND) (assessed by the guidelines of the American Diabetes Association) were incorporated in the analysis, including both obese (body mass index (BMI) > 28 *kg*/*m* ^2^) and non-obese subjects, but not subjects with other conditions that might alter glucose regulation. A standard IVGTT was performed in 268 subjects, while an IM-IVGTT was administered to 229 subjects.

**Table 1:** Summary of subject characteristics in the studies (mean ± SD).

Study No.	No. of subjects	Cohort	Sex (F/M/NA)	Age (yrs)	Weight (kg)	BMI (kg/m^2^)	Height (cm)	Study type	Reference
1	9	T2D	0/9/0	62.1 ± 5.16	73.1 ± 11.1	28.3 ± 4.48	161 ± 7.95	IM-IVGTT	Avogaro et al. (16)
2	9	ND	3/6/0	27.6 ± 9.44	68.3 ± 10.9	22.3 ± 3.39	175 ± 7.18	IM-IVGTT	Avogaro et al. (17)
3	8	ND	1/7/0	52.5 ± 2.98	85.8 ± 18.1	28.9 ± 6.7	173 ± 4.44	IM-IVGTT	Avogaro et al. (18)
4	8	T2D	1/7/0*^a^*	64.5 ± 6.26	88.4 ± 10.6	29.3 ± 2.54	173 ± 6.16	IM-IVGTT	Avogaro et al. (18)
5	6	T2D	0/6/0	57.0 ± 7.92	92.1 ± 8.45	29.2 ± 1.9	178 ± 5.05	IM-IVGTT	Ludvik et al. (19)
6	18	T2D	0/18/0	57.7 ± 8.11	88.3 ± 12	27.8 ± 2.72	178 ± 6.65	IM-IVGTT	Ludvik et al. (19)
7	11	ND	1/1/11	29.0 ± 0*^b^*	67.7 ± 5.88	22.5 ± 0*^b^*	173 ± 7.56*^d^*	IVGTT	Trojan et al. (20)
8	31	T2D	10/17/6	50.8 ± 12.9	85.8 ± 19.9	29.5 ± 6.9	171 ± 9.6	IM-IVGTT	O’Gorman et al. (21)
9	10	T2D	7/3/0	50.4 ± 7.24	78.8 ± 20.4	30.0 ± 6.49	162 ± 7.44	IM-IVGTT	Not published
10	2	ND	2/0/0	29.0 ± 9.9	100 ± 17.3	35.2 ± 8.67	170 ± 6.36	IM-IVGTT	Not published
11	2	T2D	2/0/0	36.0 ± 4.24	107 ± 15.3	34.0 ± 4.04	178 ± 2.12	IM-IVGTT	Not published
12	10	T2D	4/6/0	66.0 ± 4.71	64.3 ± 7.45	23.8 ± 0*^b^*	164 ± 9.45*^d^*	IVGTT	Viviani and Pacini (22)
13	6	ND	2/4/0	73.2 ± 7.33	63.0 ± 9.25	23.1 ± 0*^b^*	165 ± 12.2*^d^*	IVGTT	Viviani and Pacini (22)
14	11	ND	1/10/0	24.6 ± 7.21	71.5 ± 13.7	23.7 ± 0*^b^*	173 ± 17.7*^d^*	IVGTT	Viviani and Pacini (22)
15	23	T2D	6/17/0	28.4 ± 7.84	107 ± 20.3	34.8 ± 5.45	175 ± 11.3	IM-IVGTT	McQuaid et al. (23)
16	9	ND	5/4/0	35.2 ± 8.63	66.7 ± 5.24	23.0 ± 1.58	170 ± 5.57	IM-IVGTT	McQuaid et al. (23)
17	10	ND	7/3/0	18.6 ± 3.81	109 ± 14.5	35.8 ± 3.55	174 ± 5.36	IM-IVGTT	McQuaid et al. (23)
18	5	T2D	5/0/0	12.2 ± 1.86	64.8 ± 8.17	27.1 ± 2.94	155 ± 2.79	IM-IVGTT	McQuaid et al. (23)
19	2	ND	1/1/0	27.0 ± 12.7	69.5 ± 7.78	25.6 ± 5.68	166 ± 9.19	IVGTT	Not published
20	15	ND	7/8/0	38.9 ± 10.8	68.8 ± 12.3	24.3 ± 2.6	168 ± 10.6	IM-IVGTT	Pacini et al. (8)
21	10	ND	10/0/0	26.3 ± 2.58	57.0 ± 5.31	20.7 ± 2.3	166 ± 6.51	IM-IVGTT	Gennarelli et al. (24)
22	10	T2D	4/6/0	57.8 ± 8	69.0 ± 9.98	25.3 ± 1.8	165 ± 8.95	IVGTT	Not published
23	10	T2D	4/6/0	54.6 ± 11.2	68.9 ± 9.72	25.3 ± 1.64	165 ± 8.95	IVGTT	Not published
24	13	ND	1/1/13	68.3 ± 5.42	71.7 ± 8.73	24.6 ± 1.96	171 ± 5.33	IVGTT	Pacini et al. (25)
25	10	ND	1/1/10	26.7 ± 2	72.3 ± 9.71	22.9 ± 2.89	178 ± 5.87	IVGTT	Pacini et al. (25)
26	10	ND	2/8/0	36.1 ± 9.61	71.2 ± 7.1	23.8 ± 2.03	173 ± 3.35	IVGTT	Piccardo et al. (26)
27	10	ND	10/0/0	27.0 ± 0*^b^*	62.1 ± 0*^c^*	24.9 ± 0*^c^*	158 ± 0*^d^*	IVGTT	Ahrén and Pacini (27)
28	10	ND	10/0/0	63.0 ± 0*^b^*	68.0 ± 0*^c^*	25.2 ± 0*^c^*	164 ± 0*^d^*	IVGTT	Ahrén and Pacini (27)
29	10	ND	0/10/0	27.0 ± 0*^b^*	74.4 ± 0*^c^*	24.9 ± 0*^c^*	173 ± 0*^d^*	IVGTT	Ahrén and Pacini (27)
30	10	ND	0/10/0	63.0 ± 0*^b^*	78.6 ± 0*^c^*	25.2 ± 0*^c^*	177 ± 0*^d^*	IVGTT	Ahrén and Pacini (27)
31	9	ND	7/2/0	17.0 ± 2.24	54.2 ± 9.08	19.7 ± 2.5	165 ± 8.37	IVGTT	Cavallo-Perin et al. (28)
32	10	ND	2/8/0*^a^*	35.6 ± 4.7	75.3 ± 14.3	24.5 ± 3.18	175 ± 8.49	IVGTT	Cavallo-Perin et al. (29)
33	13	ND	10/3/0	13.3 ± 0.63	84.2 ± 10.2	32.5 ± 3.08	161 ± 6.57	IVGTT	Cerutti et al. (30)
34	4	ND	1/3/0	32.2 ± 11.2	75.8 ± 10.7	23.9 ± 1.06	178 ± 9.54	IM-IVGTT	Stingl et al. (31)
35	9	ND	6/4/1	43.9 ± 0*^b^*	65.7 ± 0 *^b^*	24.1 ± 0*^b^*	165 ± 0*^d^*	IVGTT	Handisurya et al. (32)
36	38	ND	38/0/0	31.5 ± 5.55	68.4 ± 13.3	25.0 ± 5.68	166 ± 5.15	IM-IVGTT	Tura et al. (33)
37	18	ND	9/9/0	44.9 ± 12.8*^b^*	114 ± 23.3	39.4 ± 3.57*^b^*	169 ± 12.6*^d^*	IVGTT	Kautzky-Willer et al. (34)
38	17	ND	10/7/0	33.5 ± 14.3	67.5 ± 13.1	23.0 ± 5.1	172 ± 11.6	IVGTT	Kautzky-Willer et al. (34)
39	7	ND	2/5/0	30.3 ± 6.52	70.0 ± 8.91	23.5 ± 0.835	172 ± 9.56	IVGTT	Kautzky-Willer et al. (35)
40	12	T2D	0/12/0	64.0 ± 5.88*^b^*	95 ± 19.6	28.6 ± 5.63*^b^*	182 ± 8.38*^d^*	IM-IVGTT	Schaller et al. (36)
41	17	ND	17/0/0	38.1 ± 7.85	84.3 ± 11.7	33.4 ± 4.05	159 ± 6.02	IVGTT	Basili et al. (37)
42	13	ND	13/0/0	42.7 ± 11.3	94.1 ± 12.4	37.4 ± 3.59	159 ± 9.86	IVGTT	Basili et al. (37)
43	11	ND	11/0/0	45.9 ± 7.61	111 ± 15.9	44.7 ± 5.82	158 ± 2.66	IVGTT	Romano et al. (38)
44	11	ND	11/0/0	48.2 ± 7.92	95.8 ± 9.46	38.1 ± 3.03	159 ± 3.88	IVGTT	Romano et al. (38)

The values in cells without superscripts are known.

a Individual values randomly assigned as per text.

b All subjects assigned as the mean value.

c Determine using anthropomorphic algorithm PopGen.

d Calculated as described in text.

**Figure 1:**
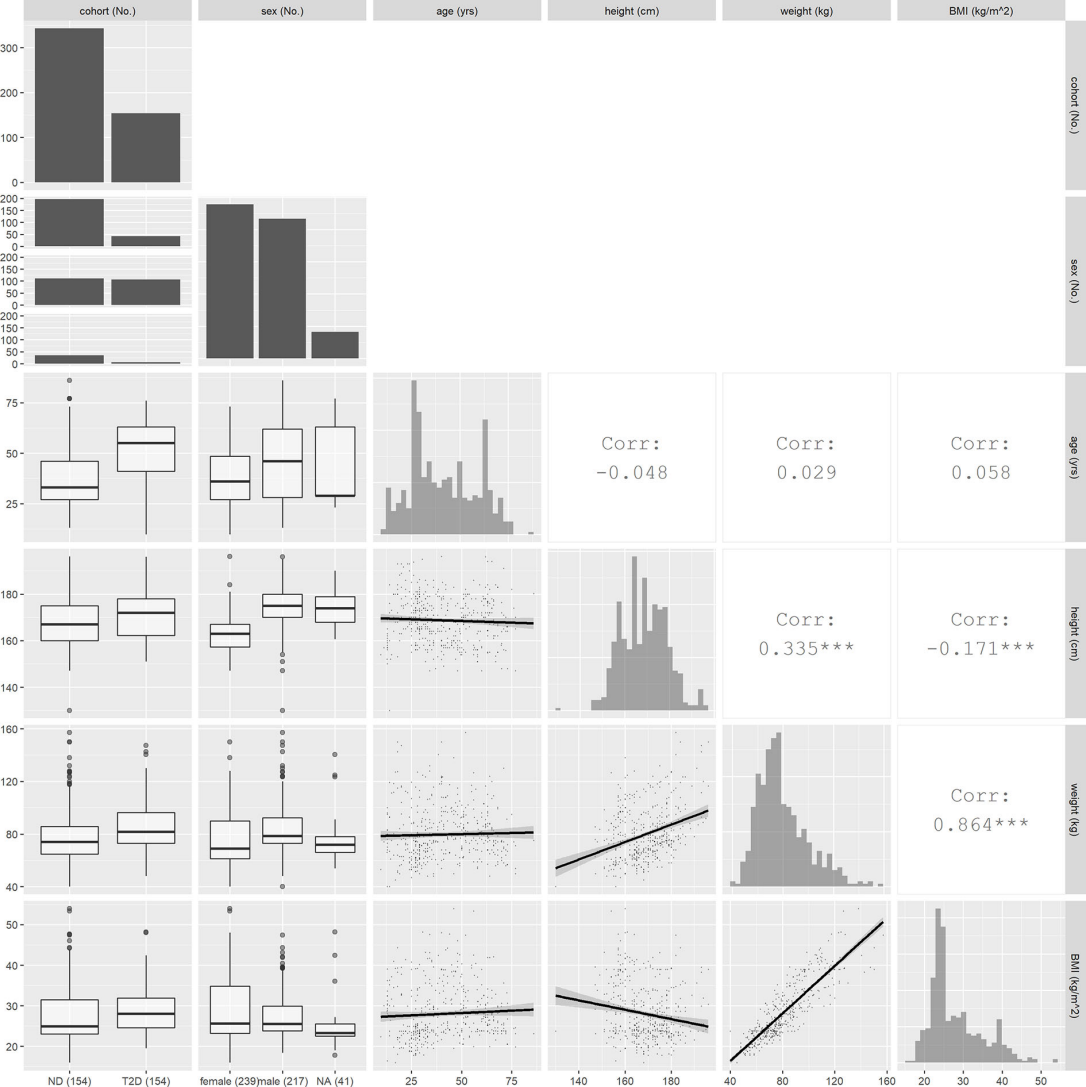
Overview of covariate values and relationships. Histograms plots for continuous covariates and bar graphs for discrete covariates are shown on the diagonal. In the lower triangle, the boxplots between continuous and discrete covariates and scatter plots between continuous covariates are displayed. In the upper triangle, the correlation coefficients between continuous covariates are shown.

Studies in which some of the characteristics are missing in individual subjects are noted in **Table 1**, with missing values imputed as follows. For studies in which only mean values of age, weight, height or BMI were reported (see table), each subject was assigned the corresponding mean value from that study. In 40 subjects from four of the study groups, the values of height, weight and BMI were missing, and only the mean BMI and its standard deviation (SD) were reported. For these subjects, we applied the virtual population anthropomorphic generator PopGen (39) to produce 40 virtual subjects, using the reported mean BMI ± 2SD as the required BMI range, the reported mean age, and the reported proportion of males (27). The resulting mean body weight of the virtual subjects in each group was then assigned to each of the 40 subjects, with the missing heights calculated as $H=\sqrt{weight/BMI}$ using the mean BMI value. The sex of 18 subjects from two study groups (see **Table 1**) was missing but the proportion of men and woman was reported, and the latter was used to randomly assign the sex of the subjects. For 41 subjects from five studies, no sex was provided, and the sex of these subjects was classified as not available (NA). After missing covariate imputation, characteristics of all 497 subjects are as summarized in **Table 2**, which includes sex, age, weight, height and body mass index. A graphic overview of investigated covariates is provided in **Figure 1**.

**Table 2:** Characteristics of study subjects.

Characteristic	No.	Mean ± SD	Minimum	Median	Maximum
Study type(IVGTT/IM-IVGTT)	268/229				
Cohort (ND/T2D)	343/154				
Sex (female/male/missing)	239/217/41				
Age (yrs)		41.4 ± 16.9	9.70	40.0	86.0
Weight (kg)		79.7 ± 19.9	40.0	75.0	157
Height (cm)		169 ± 10.1	130	168	196
BMI (kg/*m* ^2^)		28.0 ± 6.76	15.9	25.3	53.9

### Minimal Model

The following parameterization of the minimal model for glucose and insulin was used in the analysis [Bergman et al. (40), Araujo-vilar et al. (41)]:

(1) $$\frac{dG\left(t\right)}{dt}=-\left(GEZI+X\left(t\right)\right)*G\left(t\right)+\left(GEZI+X_{basal}\right)*G_{basal},\;G\left(0\right)=G_{basal}+\frac{Dose}{V}$$

(2) $$\frac{dX\left(t\right)}{dt}=-p2*X\left(t\right)+p2*S_{I}*I\left(t\right),\;X\left(0\right)=S_{I}*I_{basal}$$

where *Dose* denotes the glucose dose (mmol) at time zero, *G*(*t*) is the plasma glucose concentration (mmol/L), *G_basal_* is basal glucose concentration (measured glucose at the end point), *X*(*t*) is the remote insulin action (min^–1^), *I*(*t*) is the measured plasma insulin concentration (pmol/L) and *I_basal_* is the basal insulin concentration (measured insulin at the end point). The function *I*(*t*) was defined by linearly interpolating the measured insulin concentrations. Model parameters are: glucose effectiveness at zero insulin (*GEZI*, min^–1^), insulin sensitivity (*S_I_*, min^–1^/(pmol/L)), remote insulin action parameter (*p*2, min^–1^) and the volume of glucose distribution (*V*, L). For IVGTT, a glucose dose of 0.3 g/kg was administrated to the subjects at time zero. The study duration ranged between 180 min and 360 min, while the number of samples ranged from 12 to 30 for each subject. For IM-IVGTT, the same glucose dose was given and a short insulin infusion of between 0.03 to 0.05 U/kg was administered at 20 min. The duration of the IM-IVGTT studies ranged between 180 min and 240 min and number of samples ranged from 12 to 22. The glucose measurements obtained prior to 5 min were excluded from the analysis, since the one-compartment glucose kinetic model does not represent the initial phase of glucose disposal (42).

### Hierarchical Modeling Analysis

Hierarchical or population modeling, which is used widely in drug development, provides a formal basis for determining the distribution of model parameters in a population (central tendency and dispersion) and identifying relevant covariates that may explain aspects of the population parameter distribution [see Bonate (43)]. Notable applications of population modeling to the glucose-insulin system include the work of Agbaje et al. (44) using a Bayesian analysis and that of Denti et al. (45) using approximate maximum likelihood methods.

In this work, Eqs (1) and (2) define the first stage of the hierarchical framework, where the residual error (defined as the difference between the measured and predicted glucose concentrations) was assumed to be normally distributed with variance proportional to the predicted glucose concentration. For the second stage of the hierarchy, the vector of model parameters, logθ ≡ log [*GEZI S_I_ p*2 *V*], is assumed to follow a multivariate normal distribution, log*θ* ~ *N* (*μ*_log_ *_θ_*, Σ_log_ *_θ_*), with the population mean *μ*_log_ *_θ_*, covariance Σ_log_ *_θ_*, and the conditional mean for each subject *E*[log*θ*_i_], *i* = 1,…, n, are estimated from the pooled study data. The maximum likelihood estimates of *μ*_log_ *_θ_*, Σ_log_ *_θ_* and *E*[log*θ*_i_] were obtained using the expectation-maximization (EM) algorithm as applied to solve the nonlinear mixed effects hierarchical modeling problem by Schumitzky (46) and by Walker (47), and as implemented in the MLEM application in ADAPT (Version 5) software (48). The supplemental information contains details regarding the hierarchical modeling framework used in this work.

The following covariates were examined for their influence on model parameters: age, body weight, height, BMI, sex, and glucose tolerance (ND/T2D). We also included test type (IVGTT/IM-IVGTT) as a covariate since some previous studies indicated that there may be a difference in MM parameter estimates between IVGTT and IM-IVGTT experiments (8). Initially, covariate-parameter relationships were identified based on exploratory graphical analysis and mechanistic plausibility. Individual subject conditional mean estimates of model parameters were obtained from the hierarchical model without covariates. All identified covariates for each of the model parameters (*S_I_*, *GEZI*, etc.) were added one-by-one, based on their significance in the exploratory analysis, to generate new hierarchical models. The final explanatory covariates were selected based on estimate precision and objective function value (-2 log likelihood) improvement as accessed using the likelihood ratio test (*p*<0.05) (43). We tested the covariate model for *S_I_* initially, as the importance of *S_I_* for glucose tolerance has been well established and its relationship with BMI has been mentioned in previous studies. Covariate effects on *V* were then tested, as it was found to correlate with body weight in Denti et al. (45). After accounting for the effects on *S_I_* and *V*, we then tested the covariate model of *GEZI* to study its relationship with subject characteristics, as this has not been examined in a large population previously. For the continuous covariates considered (age, body weight, height, BMI), power models centered at their median values of the covariates were used. For the categorical covariates considered (sex, glucose tolerance and test type), changes in the covariate model parameters between levels were investigated.

## Results

**Table 3** (second column) presents the results of the population modeling analysis using the minimal model in Eqs. 1 and 2 without incorporating covariates in the stage 2 parameter distribution model. The table shows the typical values (TV) of the model parameters as a measure of the central tendency of the parameter population distribution $\left(TV=e^{\mu_{\log\theta }}\right)$, as well as the parameter inter-individual variability (IIV) as a measure of dispersion of the population distribution $IIV\equiv \{100\sqrt{{\left({\text{Σ}}_{\log\theta }\right)}_{ii}}\},i=1,\ldots ,4$. In the third column of **Table 3**, the corresponding results are presented from the population analysis that included the covariates determined to be significant, as described in MATERIALS AND METHODS.

**Table 3:** Population modeling results.

Parameter	Without covariates	With covariates
(Unit)	(RSE-CV%)	(RSE-CV%)
Typical values:		
*GEZI* (min^–1^)	0.0178 (3.37)	0.0210 (3.73)
*S_I_* (min^–1^/(pmol/L))	3.59e-5 (5.80)	6.26e-5 (6.33)
*p*2 (min^–1^)	0.0425 (3.62)	0.0420 (3.65)
*V* (L)	12.4 (1.87)	12.0 (1.56)
Inter-individual variabilities (CV%):		
*GEZI*	50.9 (4.65)	46.1 (5.09)
*S_I_*	113 (3.83)	83.8 (3.44)
*p*2	44.0 (7.79)	44.9 (7.47)
*V*	34.4 (3.48)	26.8 (3.11)
Covariate effects:		
T2D on *GEZI*		-0.473 (8.73)
T2D on *S_I_*		-0.479 (9.95)
BMI on *S_I_*		-2.14 (8.43)
IM-IVGTT on *S_I_*		-0.345 (19.4)
weight on *V*		0.865 (6.49)
Proportional error	0.0706 (0.352)	0.0706 (0.358)
-2 log likelihood	18674	18115

RSE, relative standard error.

Correlation between model parameters: GEZI and S_I_: -0.14; GEZI and p2: 0.77; GEZI and p2: -0.07; S_I_ and p2: -0.05; S_I_ and V: 0.14; p2 and V: -0.31.

**Figure 2:**
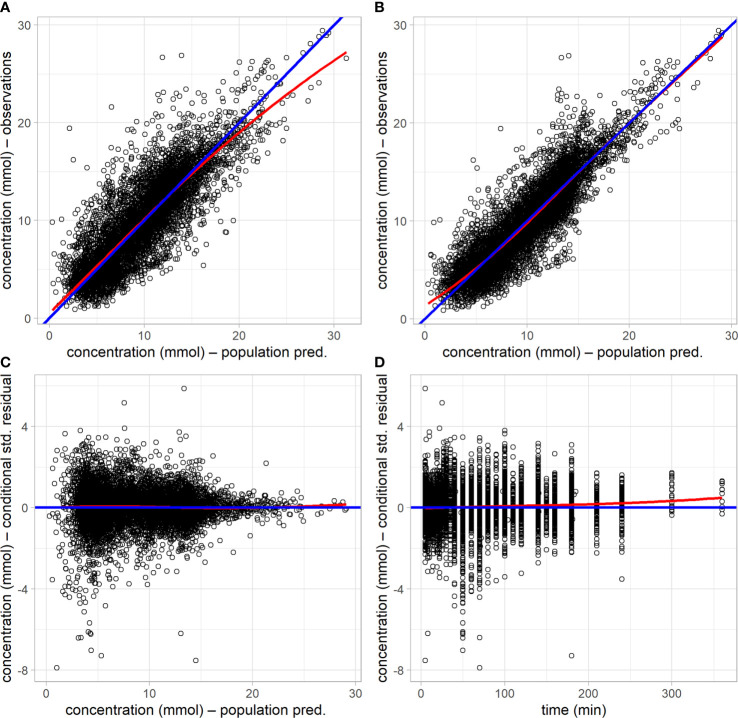
Goodness-of-fit plots of the base model without covariates and the final model with covariates. **(A)** observed glucose concentration versus population prediction from the base model. **(B)** observed glucose concentration versus population prediction from the final model. **(C)** conditional standardized residuals versus population prediction in the final model. **(D)** conditional standardized residual in the final model versus time. Blue lines are the lines of identity or zero value; red lines are loess smooth curves.

All model parameters were well estimated, with relative standard errors less than 20 CV%, and the model with covariates (final model) yielded a significant reduction in the log likelihood compared to the base model without covariates (likelihood ratio test, *p*<10^–6^). The upper row of the goodness-of-fit plots in **Figure 2** shows the population prediction of the base model (**Figure 2A**) and that of the final covariate model (**Figure 2B**), indicating an improved description of the observed data with the later. Plots of the resulting conditional standardized residuals from the final model versus the population predicted glucose concentration (**Figure 2C**) and versus time (**Figure 2D**), indicate that the final population model describes the observed glucose concentrations without significant bias.

### *GEZI* Is Decreased in T2D but Is Not Associated With Other Covariates

**Figure 3:**
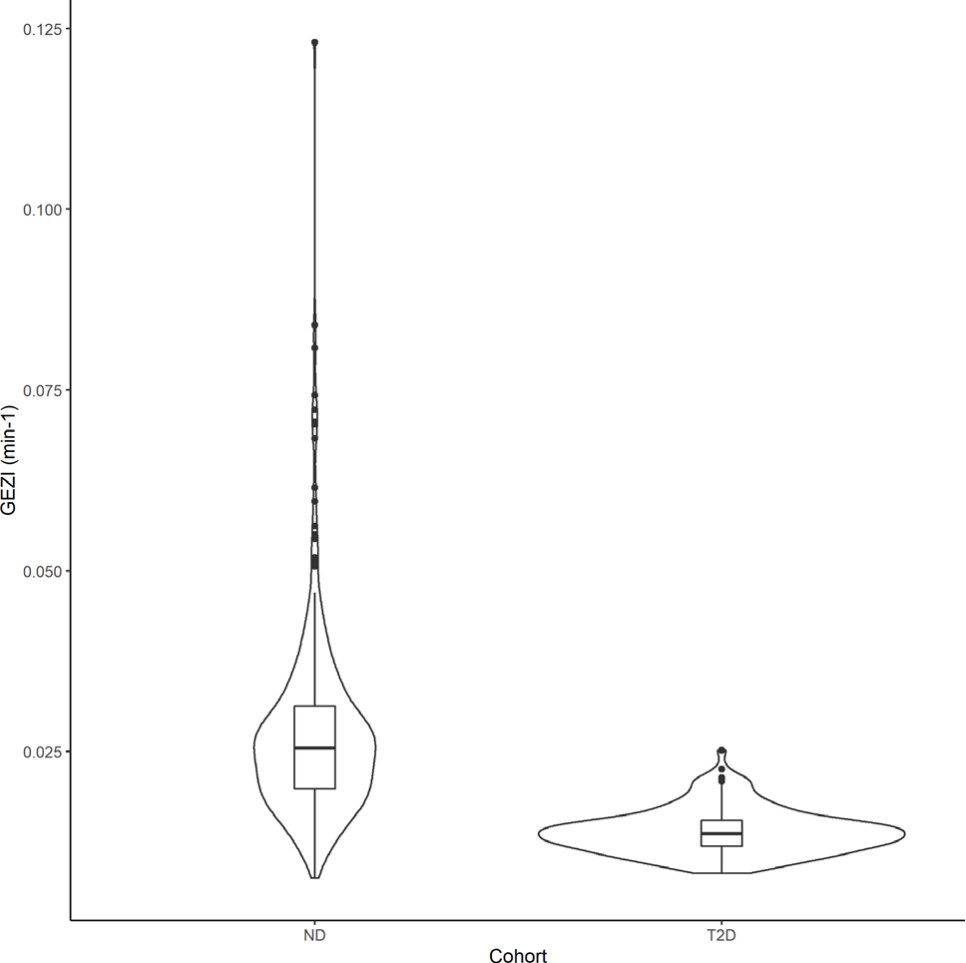
Violin plots showing the distribution of the individual subjects conditional mean estimates of *GEZI* in the ND and T2D cohorts. Boxplots were inserted for each cohort to indicate medians and interquartile ranges.

In the final population model, the typical value of *GEZI* depends on glucose tolerance category as follows: *GEZI*=0.0210*(1−0.473*T2*D*) (min^–1^), where *T*2*D*=1 for T2D subjects and *T*2*D*=0 for ND subjects. The distribution of *GEZI* in the ND population was estimated to have a typical value of 0.021 min^–1^ with inter-individual variability of ± 0.0097 min^–1^. The corresponding values in the T2D population were estimated to be 0.011 ± 0.0055 min^–1^. This 47% reduction in the value of *GEZI* in T2D subjects relative to ND subjects was found to be highly significant (*p*<10^–6^) *via* a likelihood ratio test. The distribution of individual subject conditional mean estimates of *GEZI* in ND and T2D subjects is shown in **Figure 3**. We also explored possible covariate models relating *GEZI* to *BMI* in both ND and T2D subjects, but no associations were found to be significant. Moreover, the variability in *GEZI* could not be further explained by subjects’ age, weight, height or sex; while a decreasing association between *GEZI* and age was noted, this was not statistically significant. No differences in *GEZI* were found between subjects that underwent an IVGTT versus an IM-IVGTT experiment.

### *S_I_* Decreases With BMI in Both ND and T2D

**Figure 4:**
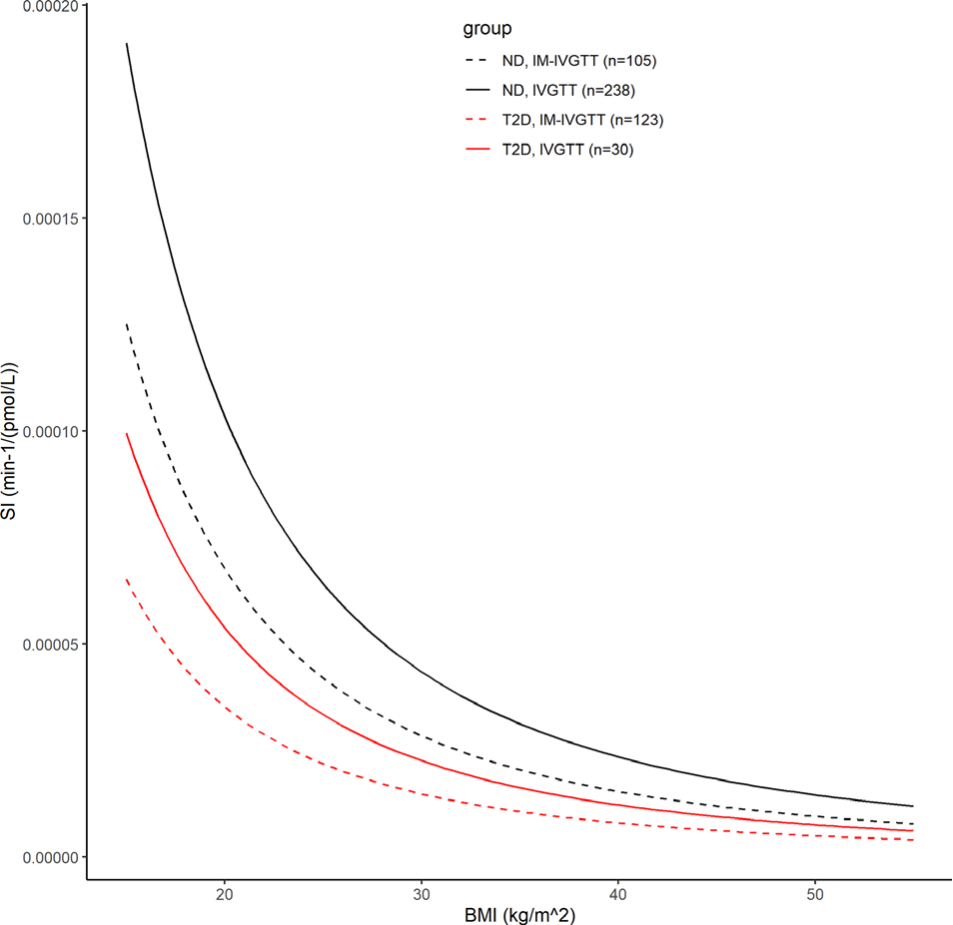
The black lines show the covariate model prediction of the typical value of *S_I_* versus BMI in ND subjects, with the solid line indicating subjects in IVGTT and dash line indicating IM-IVGTT. The red lines are the corresponding curves in T2D patients.

In the final population model, *S_I_* was found to be associated with BMI, *a priori* glucose tolerance status, and test type by the following model: *S_I_*=(6.26*e*–5)*(1–0.479*T2*D*)*(1–0.345*IM)*(*BMI*/25.3)^–2.14^, where *IM*=1 for IM-IVGTT and *IM*=0 for IVGTT. **Figure 4** shows the estimated relationships between the typical value of *S_I_* and *BMI* for both ND and T2D subjects from each of the two test types. In both ND and T2D groups, higher BMI values lead to decreased *S_I_* (with a power of -2.14). This is consistent with the conclusions in Bergman and Lovejoy (49), in which they reported a negative association between BMI and *S_I_*. For a given BMI, the population model shows a significant decrease (approximately 48%) in *S_I_*, between the T2D subjects versus those with ND. Our results indicated that IM-IVGTT is associated with significantly lower *S_I_* (approximately 35%) estimate when compared to IVGTT (*p*<10^–5^). The addition of weight, height, age or sex to the population model was not found to be significant.

### The Significant Non–Insulin-Dependent Contribution to Net Glucose Disposal Is Greater in ND Than T2D Following a Glucose Load

**Figure 5:**
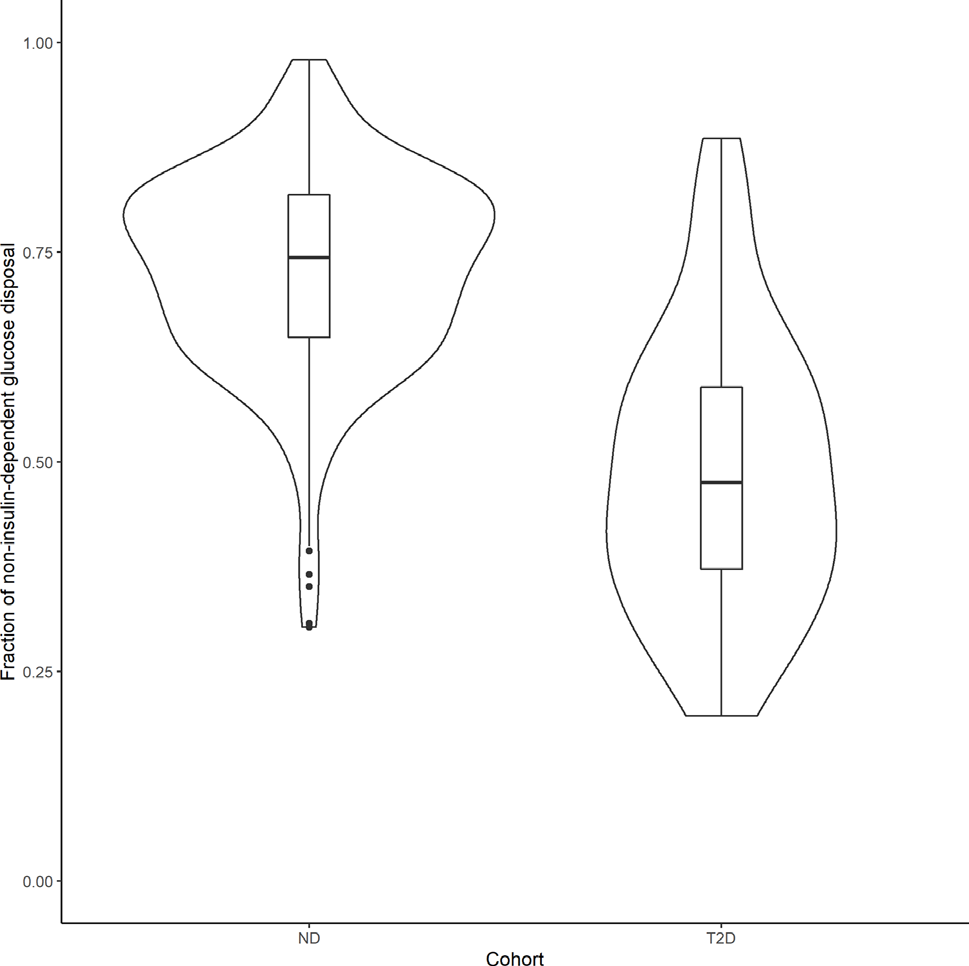
Violin plots showing the distribution of the fraction of non–insulin-dependent net glucose disposal in 238 ND subjects and 30 T2D patients that underwent an IVGTT test. Boxplots were inserted for each cohort to indicate medians and interquartile ranges.

In order to compare the relative contributions of non-insulin- and insulin-mediated pathways to net glucose disposal, we determined the proportion of glucose uptake due to glucose itself and that due to insulin, in ND subjects and those with T2D, in both the basal state and during an IVGTT experiment. Under basal conditions, the fraction of non–insulin-dependent glucose disposal can be calculated using the individual conditional mean estimates of *GEZI* and *S_I_*, together with the measured *I_basal_* of each subject: (*GEZI*/ (GEZI+*S_I_I_basal_*)). In the 343 ND subjects, the non–insulin-dependent pathway accounted for 88.5% ± 7.10% of the total net glucose disposal, and 89.0% ± 10.2% in the 154 T2D subjects. In the IVGTT experiment group (268 subjects), we calculated the net glucose disposal due to the two pathways during the course of the experiment, based on individual subject estimates. The non–insulin-dependent and insulin dependent glucose disposal (*GD*-*NID*, *GD*-*ID*) were calculated in each subject as follows: $GD-NID=\int_{0}^{T}\;GEZI*G(t)dt,\;GD-ID=\int_{0}^{T}X(t)*G(t)dt$ (*T* is the last measurement time in the subject’s IVGTT experiment). In the 238 ND subjects, the fraction of non–insulin-dependent glucose disposal (*GD*-*NID*/(*GD*-*NID* + *GD*-*ID*)) was 72.8% ± 12.0%, while it was 48.8% ± 16.9% in the 30 T2D subjects in the IVGTT group (**Figure 5**), and this difference was significant (*p*<10^–6^, unpaired two-samples Wilcoxon test). Our model based analysis indicates that the non–insulin-dependent route accounts for the large majority of the glucose disposal in the ND population, while it is reduced to approximately half of the total in the T2D population.

## Discussion

In this work, a hierarchical modeling analysis was conducted to develop a composite population minimal model of glucose-insulin dynamics in ND and T2D subjects, using data from IVGTT and IM-IVGTT studies. The resulting population model was used to quantify the role of subject characteristics (age, body weight, height, BMI, sex, glucose tolerance status) and test type (IVGTT/IM-IVGTT) on glucose effectiveness and other MM parameters. In the final composite population model, glucose tolerance status (ND, T2D) was a significant predictor of glucose effectiveness, as assessed by *GEZI*. The addition of other covariates did not further explain remaining inter-subject variability in *GEZI*, beyond that predicted by glucose tolerance status. Further analysis of the population model indicated that the relative contribution to the total net glucose disposal independent of insulin was significantly greater in ND compared to T2D subjects. As expected, a significant relation between insulin sensitivity, *S_I_*, and BMI was identified in both ND and T2D populations, albeit different in the two groups. Moreover, the estimated *S_I_*-BMI relations were found to depend on test type, with a lower population value of *S_I_* observed in IM-IVGTT versus IVGTT studies, after accounting for BMI.

Based on the composite population model (**Table 3**), glucose effectiveness, as assessed by *GEZI* in this study, is reduced from 0.021 min^–1^ (standard error – 0.00078 min^–1^) in the ND population to 0.011 min^–1^ (standard error – 0.00045 min^–1^) in T2D. Moreover, there is less inter-individual variability in GEZI in the T2D population (0.0051 min^–1^, standard error – 0.00026 min^–1^) relative to ND (0.0097 min^–1^, standard error – 0.00049 min^–1^). This result is qualitatively consistent with other studies that reported a reduction in glucose effectiveness, as assessed by *S_G_*, involving smaller numbers of T2D subjects Welch et al. (15) and Taniguchi et al. (14). We also examined potential associations between *GEZI* and both BMI and age, and while a negative relation was noted with each, neither age nor BMI was found to be significant, when considering all subjects, or separately within the ND and T2D populations. The lack of association with age is consistent with the conclusion in Pacini et al. (25) from their analysis of *S_G_* in 17 elderly healthy subjects. Morettini et al. (13) did, however, find a weak but statistically significant relation between *GEZI* and BMI based on a MM analysis of 204 healthy subjects. While we also noted a negative correlation between *GEZI* and BMI, this was not found to be statistically significant in our study.

In the study in healthy subjects following an IVGTT (50), it was found that the glucose disappearance constant *K_G_* was strongly correlated with *GEZI* and concluded that *GEZI* is a major determinant of glucose disappearance. Further analysis of the population model reported in this work, allows for the calculation of the relative contribution of insulin- and non-insulin-dependent pathways of total net glucose disposal in IVGTT experiments (see **Figure 5**). Although both *GEZI* and *S_I_* are reduced by a similar percent (47%) in T2D and ND subjects, the relative contribution of non–insulin-dependent glucose disposal is lower in T2D subjects (48.8%) compared to that in ND subjects (73.8%). This difference is not unexpected given that *GEZI* directly facilitates plasma glucose disappearance, while *S_I_* influences glucose disposal only indirectly *via* its effect on remote insulin action (*X*(*t*)). The average fraction of non–insulin-dependent glucose disposal in ND subjects found in this study, 72.8%, is somewhat higher than the range of 45% to 65% reported in Alford et al. (3), but consistent with the reported value of 71% following IVGTT in mice (51). While Best et al. (6) reported that the contribution of glucose effectiveness to glucose disposal is dominant in insulin-resistant subjects (83%) based on an oral glucose tolerance study, our results (**Figure 5**) indicate that the contribution of glucose effectiveness in T2D subjects following IVGTT can range widely (19.7% to 88.6%). Under basal conditions in both ND and T2D subjects, the population modeling analysis found that the fraction of net glucose disposal mediated by the non–insulin-dependent pathway was approximately 89% (see Results section). For comparison, Best et al. (6) reported that at basal insulin levels, glucose effectiveness can account for 60% to 75% of the glucose uptake based on the clamp approach, depending on the basal glucose concentration.

The population modeling results predicting a significantly lower *S_I_* in the T2D population compared to the ND population (approximately 47.9%, RSE=10%) were expected given the well-documented ability of *S_I_* to predict glucose tolerance (49). The negative association between *S_I_* and BMI quantified in the population model (see **Figure 4**) is also consistent with other studies in ND and T2D populations [Welch et al. (15), Morettini et al. (13)]. However, these results related to BMI should be interpreted cautiously, given the well-known challenges associated with using BMI as independent factor to explain differences in *S_I_*, without incorporating information related to fat distribution (52). The typical glucose distribution volume *V* was found to depend on body weight following a nonlinear relationship with coefficient and power estimated from the modeling analysis: *V*(*L*)=12.0*(*weight*/75)^0.865^ (weight in kg). The typical glucose distribution volume of 12 L is close to the reported range of 1.65-1.70 dl/kg in Denti et al. (45), but the confidence interval for the power estimate does not include 1. While no relation between *p*2 and any covariates was found to be significant, *p*2 was determined to be positively correlated with *GEZI* (*r*=0.77) (the ability to estimate all parameter correlations in the population is intrinsic to hierarchical modeling analysis). Thus, any association between *p*2 and glucose tolerance could be reflected in its correlation with *GEZI*. From our analysis, it was also concluded that there is a significant difference in the *S_I_* between subjects administered an IVGTT versus an IM-IVGTT. This results is consistent with the observation in Ward et al. (53) that the MM estimate of *S_I_* depends on the dose and duration of exogenous insulin administration in IM-IVGTT experiments. Since our study used previously collected data from various sites conducted over an extended period of time, the inability to retrieve all the details of the IM-IVGTT experiments precluded us from further exploring any potential effects of the insulin administration profiles on the estimation results.

In this study, a hierarchical modeling analysis was used to develop a composite population minimal model in a diverse collection of subjects who were assessed to either have or not have type 2 diabetes. This modeling analysis allows the complete study data to be used to simultaneously inform the estimation of the population distribution of model parameters (mean and covariance), which provides a mechanism for identifying explanatory subject specific factors (anthropomorphic, pathophysiological, treatment, study, etc.) and quantifying their effects on model parameters. Hierarchical modeling has been applied previously in MM studies, including by Agbaje et al. (44) who used a Bayesian framework to analyze results from IM-IVGTT experiments in 65 T2D subjects, and more recently by Denti et al. (45) using approximate maximum likelihood methods in a study of 204 healthy subjects after IM-IVGTT. An advantage of using hierarchical modeling as implemented in this study, is that it allows for a multivariate assessment of the relative contribution of subject specific characteristics. A limitation of the approach, beyond the additional computational difficulties associated with implementing the EM algorithm to obtain the analytically exact maximum likelihood estimates, is that the MM parameters are assumed to follow a defined distribution in the population (specifically, log[*GEZI S_I_ p*2 *V*] ~ *N* (*μ*_log_ *_θ_*, Σ_log_ *_θ_*)). Also, as with any multivariate analysis, identifying the explanatory covariates depends on the specific statistical procedure and the associated criteria for including and excluding covariates, which can be particularly challenging when covariates are not independent. While this work used glucose tolerance tests conducted at different sites, we did not find any systematic differences in model parameter estimates across study sites.

The role of glucose effectiveness as a predictor of glucose intolerance and diabetes has been suggested in several studies [Martin et al. (54), Lorenzo et al. (12)], which have reported that reduced glucose effectiveness may precede diabetes development even in normoglycemic subjects. Indeed, the modeling analysis in this study suggests *GEZI* may be a predictor of the dysregulated glucose tolerance. Also, glucose effectiveness may be a possible target for glucose-reducing therapies (55). Although the molecular mechanisms of glucose effectiveness in regulating glucose remains to be more fully elucidated, some studies have demonstrated that pharmacological intervention (5) and exercise (56) can improve glucose effectiveness and increase plasma glucose clearance. Recently, the development of sodium glucose cotransporter 2 inhibitors has provided a novel antidiabetic therapy independent of insulin action (57).

In summary, we have conducted a hierarchical minimal model analysis of the glucose-insulin response in ND and T2D subjects given an intravenous glucose load, which allowed us to quantify the influence of diabetes status, BMI and body weight on the glucose metabolic parameters, while accounting for the differences in the study type. The relative contribution of non–insulin-dependent net glucose disposal in ND and T2D populations was determined using the resulting population model, demonstrating the utility of this modeling approach to quantify the fraction of non–insulin-dependent glucose disposal based on an IVGTT. The novel finding that *GEZI* is markedly reduced in T2D, both in its absolute value and the relative contribution to net glucose disposal, represents a further indication of the extensive dysregulated glucose homeostasis induced by diabetes. Although this work was focused on MM analysis of ND and T2D subjects, the hierarchical modeling framework can be applied to investigate glucose effectiveness in populations with other accompanying disease conditions, and to investigate other possible explanatory covariates in future studies.

## Data Availability Statement

The raw data supporting the conclusions of this article will be made available by the authors, without undue reservation.

## Ethics Statement

The studies involving human participants were reviewed and approved by multiple institutions and approved by each institution’s respective ethics committees (**Table 1** in the manuscript lists each institution). Written informed consent to participate in this study was provided by the participants’ legal guardian/next of kin.

## Author Contributions

SH and DD’A participated in the study design, performed the modeling analysis, and drafted the manuscript. YL participated in data analysis and reviewed the manuscript. GP and AT provided the data, participated in the study design, and critically reviewed and revised the manuscript. GP was a visiting collaborator at the Biomedicine Simulations Resource at the University of Southern California over the course of this project. All authors contributed to the article and approved the submitted version.

## Funding

This work was supported by grants from National Institutes of Health/National Institute of Biomedical Imaging and Bioengineering (NIH/NIBIB) P41-EB001978 and the Alfred E. Mann Institute at USC (DD’A).

## Conflict of Interest

The authors declare that the research was conducted in the absence of any commercial or financial relationships that could be construed as a potential conflict of interest.

## References

[1] Dube S Errazuriz-Cruzat I Basu A Basu R. The forgotten role of glucose effectiveness in the regulation of glucose tolerance. Curr Diabetes Rep (2015) 15:605–10. 10.1007/s11892-015-0605-6 25869240

[2] Kahn SE Klaff LJ Schwartz MW Beard JC Bergman RN Taborsky GJJ. Treatment with a somatostatin analog decreases pancreatic B-cell and whole body sensitivity to glucose. J Clin Endocrinol Metab (1990) 71:994–1002. 10.1210/jcem-71-4-994 2205630

[3] Alford FP Henriksen JE Rantzau C Beck-Nielsen H. Glucose effectiveness is a critical pathogenic factor leading to glucose intolerance and type 2 diabetes: An ignored hypothesis. Diabetes Metab Res Rev (2018) 34:e2989. 10.1002/dmrr.2989 29451713

[4] Basu A Dalla Man C Basu R Toffolo G Cobelli C Rizza RA. Effects of type 2 diabetes on insulin secretion, insulin action, glucose effectiveness, and postprandial glucose metabolism. Diabetes Care (2009) 32:866–72. 10.2337/dc08-1826 PMC2671126 19196896

[5] Pau CT Keefe C Duran J Welt CK. Metformin improves glucose effectiveness, not insulin sensitivity: Predicting treatment response in women with polycystic ovary syndrome in an open-label, interventional study. J Clin Endocrinol Metab (2014) 99:1870–8. 10.1210/jc.2013-4021 PMC4010712 24606093

[6] Best JD Kahn SE Ader M Watanabe RM Ta-Chen N Bergman RN. Role of Glucose Effectiveness in the Determination of Glucose Tolerance. Diabetes Care (1996) 19:1018–30. 10.2337/diacare.19.9.1018 8875104

[7] Ader M Ni TC Bergman RN. Glucose effectiveness assessed under dynamic and steady state conditions. Comparability of uptake versus production components. J Clin Invest (1997) 99:1187–99. 10.1172/JCI119275 PMC507932 9077526

[8] Pacini G Tonolo G Sambataro M Maioli M Ciccarese M Brocco E. Insulin sensitivity and glucose effectiveness: Minimal model analysis of regular and insulin-modified FSIGT. Am J Physiol (1998) 274:592–9. 10.1152/ajpendo.1998.274.4.e592 9575818

[9] Bergman RN Prager R Volund A Olefsky JM. Equivalence of the insulin sensitivity index in man derived by the minimal model method and the euglycemic glucose clamp. J Clin Invest (1987) 79:790–800. 10.1172/JCI112886 3546379 PMC424201

[10] Henriksen JE Alford F Ward G Thye-Rønn P Levin K Hother-Nielsen O. Glucose effectiveness and insulin sensitivity measurements derived from the non-insulin-assisted minimal model and the clamp techniques are concordant. Diabetes Metab Res Rev (2010) 26:569–78. 10.1002/dmrr.1127 20830736

[11] Henriksen JE Alford F Handberg A Vaag A Ward GM Kalfas A. Increased glucose effectiveness in normoglycemic but insulin-resistant relatives of patients with non-insulin-dependent diabetes mellitus. A novel compensatory mechanism. J Clin Invest (1994) 94:1196–204. 10.1172/JCI117436 PMC295197 8083360

[12] Lorenzo C Wagenknecht LE Rewers MJ Karter AJ Bergman RN Hanley AJ. Disposition index, glucose effectiveness, and conversion to type 2 diabetes: The insulin resistance atherosclerosis study (IRAS). Diabetes Care (2010) 33:2098–103. 10.2337/dc10-0165 PMC2928371 20805282

[13] Morettini M Di Nardo F Ingrillini L Fioretti S Göbl C Kautzky-Willer A. Glucose effectiveness and its components in relation to body mass index. Eur J Clin Invest (2019) 49:e13099. 10.1111/eci.13099 30838644

[14] Taniguchi A Nakai Y Fukushima M Kawamura H Imura H Nagata I. Pathogenic factors responsible for glucose intolerance in patients with NIDDM. Diabetes (1992) 41:1540–6. 10.2337/diab.41.12.1540 1446794

[15] Welch S Gebhart SSP Bergman RN Phillips LS. Minimal model analysis of intravenous glucose tolerance test-derived insulin sensitivity in diabetic subjects. J Clin Endocrinol Metab (1990) 71:1508–18. 10.1210/jcem-71-6-1508 2229309

[16] Avogaro A Miola M Favaro A Gottardo L Pacini G Manzato E. Gemfibrozil improves insulin sensitivity and flow-mediated vasodilatation in type 2 diabetic patients. Eur J Clin Invest (2001) 31:603–9. 10.1046/j.1365-2362.2001.00856.x 11454015

[17] Avogaro A Watanabe RM Gottardo L De Kreutzenberg S Tiengo A Pacini G. Glucose tolerance during moderate alcohol intake: Insights on insulin action from glucose/lactate dynamics. J Clin Endocrinol Metab (2002) 87:1233–8. 10.1210/jcem.87.3.8347 11889193

[18] Avogaro A Watanabe RM Dall’Arche A De Kreutzenberg S Tiengo A Pacini G. Acute Alcohol Consumption Improves Insulin Action Without Affecting Insulin Secretion in Type 2 Diabetic Subjects. Diabetes Care (2004) 27:1369–74. 10.2337/diacare.27.6.1369 15161790

[19] Ludvik B Waldhäusl W Prager R Kautzky-Willer A Pacini G. Mode of action of Ipomoea Batatas (Caiapo) in type 2 diabetic patients. Metabolism (2003) 52:875–80. 10.1016/S0026-0495(03)00073-8 12870164

[20] Trojan N Pavan P Iori E Vettore M Marescotti MC Tiengo A. Effect of different times of administration of a single ethanol dose on insulin action, insulin secretion and redox state. Diabetic Med (1999) 16:400–7. 10.1046/j.1464-5491.1999.00060.x 10342340

[21] O’Gorman DJ Yousif O Dixon G McQuaid S Murphy E Rahman Y. In vivo and in vitro studies of GAD-antibody positive subjects with Type 2 diabetes: A distinct sub-phenotype. Diabetes Res Clin Pract (2008) 80:365–70. 10.1016/j.diabres.2007.12.009 18405999

[22] Viviani GL Pacini G. Reduced glucose effectiveness as a feature of glucose intolerance: Evidence in elderly type-2 diabetic subjects. Aging Clin Exp Res (1999) 11:169–75. 10.1007/bf03399659 10476312

[23] McQuaid S O’Gorman DJ Yousif O Yeow TP Rahman Y Gasparro D. Early-onset insulin-resistant diabetes in obese caucasians has features of typical type 2 diabetes, but 3 decades earlier. Diabetes Care (2005) 28:1216–8. 10.2337/diacare.28.5.1216 15855595

[24] Gennarelli G Rovei V Novi RF Holte J Bongioanni F Revelli A. Preserved insulin sensitivity and β-cell activity, but decreased glucose effectiveness in normal-weight women with the polycystic ovary syndrome. J Clin Endocrinol Metab (2005) 90:3381–6. 10.1210/jc.2004-1973 15755857

[25] Pacini G Valerio A Beccaro F Nosadini R Cobelli C Crepaldi G. Insulin sensitivity and beta-cell responsivity are not decreased in elderly subjects with normal OGTT. J Am Geriatr Soc (1988) 36:317–23. 10.1111/j.1532-5415.1988.tb02358.x 3280644

[26] Piccardo MG Pacini G Nardi E Rosa MS Vito RD. Beta-cell response and insulin hepatic extraction in noncirrhotic alcoholic patients soon after withdrawal. Metabolism (1994) 43:367–71. 10.1016/0026-0495(94)90106-6 8139486

[27] Ahrén B Pacini G. Age-related reduction in glucose elimination is accompanied by reduced glucose effectiveness and increased hepatic insulin extraction in man. J Clin Endocrinol Metab (1998) 83:3350–6. 10.1210/jc.83.9.3350 9745453

[28] Cavallo-Perin P Pacini G Cerutti F Bessone A Condo C Sacchetti L. Insulin resistance and hyperinsulinemia in Homozygous beta-Thalassemia. Metabolism (1995) 44:281–6. 10.1016/0026-0495(95)90155-8 7885270

[29] Cavallo-Perin P Bergerone S Gagnor A Comune M Giunti S Cassader M. Myocardial infarction before the age of 40 years is associated with insulin resistance. Metabolism (2001) 50:30–5. 10.1053/meta.2001.19501 11172471

[30] Cerutti F Sacchetti C Bessone A Rabbone I Cavallo-Perin P Pacini G. Insulin secretion and hepatic insulin clearance as determinants of hyperinsulinaemia in normotolerant grossly obese adolescents. Acta Paediatr (1998) 87:1045–50. 10.1080/080352598750031356 9825970

[31] Stingl H Schnedl WJ Krssak M Bernroider E Bischof MG Lahousen T. Reduction of hepatic glycogen synthesis and breakdown in patients with agenesis of the dorsal pancreas. J Clin Endocrinol Metab (2002) 87:4678–85. 10.1210/jc.2002-020036 12364458

[32] Handisurya A Pacini G Tura A Gessl A Kautzky-Willer A. Effects of T4 replacement therapy on glucose metabolism in subjects with subclinical (SH) and overt hypothyroidism (OH). Clin Endocrinol (2008) 69:963–9. 10.1111/j.1365-2265.2008.03280.x 18429948

[33] Tura A Grassi A Winhofer Y Guolo A Pacini G Mari A. Progression to type 2 diabetes in women with former gestational diabetes: time trajectories of metabolic parameters. PloS One (2012) 7:e50419. 10.1371/journal.pone.0050419 23185618 PMC3503894

[34] Kautzky-Willer A Pacini G Ludvik B Schernthaner G Prager R. β-Cell hypersecretion and not reduced hepatic insulin extraction is the main cause of hyperinsulinemia in obese nondiabetic subjects. Metabolism (1992) 41:1304–12. 10.1016/0026-0495(92)90100-O 1461136

[35] Kautzky-Willer A Thomaseth K Clodi M Ludvik B Waldhäusl W Prager R. β-Cell activity and hepatic insulin extraction following dexamethasone administration in healthy subjects. Metabolism (1996) 45:486–91. 10.1016/S0026-0495(96)90224-3 8609836

[36] Schaller G Kretschmer S Gouya G Haider DG Mittermayer F Riedl M. Alcohol acutely increases vascular reactivity together with insulin sensitivity in type 2 diabetic men. Exp Clin Endocrinol Diabetes (2010) 118:57–60. 10.1055/s-0029-1233453 19834876

[37] Basili S Pacini G Guagnano MT Manigrasso MR Santilli F Pettinella C. Insulin resistance as a determinant of platelet activation in obese women. J Am Coll Cardiol (2006) 48:2531–8. 10.1016/j.jacc.2006.08.040 17174194

[38] Romano M Guagnano MT Pacini G Vigneri S Falco A Marinopiccoli M. Association of Inflammation Markers with Impaired Insulin Sensitivity and Coagulative Activation in Obese Healthy Women. J Clin Endocrinol Metab (2003) 88:5321–6. 10.1210/jc.2003-030508 14602768

[39] McNally K Cotton R Hogg A Loizou G. Reprint of PopGen: A virtual human population generator. Toxicology (2015) 332:77–93. 10.1016/j.tox.2015.04.014 25921244

[40] Bergman RN Ider YZ Bowden CR Cobelli C. Quantitative estimation of insulin sensitivity. Am J Physiol (1979) 5:667–77. 10.1152/ajpendo.1979.236.6.e667 443421

[41] Araujo-Vilar D Rega-Liste CA Garcia-Estevez DA Sarmiento-Escalona F Mosquera-Tallon V Cabezas-Cerrato J. Minimal model of glucose metabolism: Modified equations and its application in the study of insulin sensitivity in obese subjects. Diabetes Res Clin Pract (1998) 39:129–41. 10.1016/S0168-8227(97)00126-5 9597383

[42] Vicini P Caumo A Cobelli C. Glucose effectiveness and insulin sensitivity from the minimal models: consequences of undermodeling assessed by Monte Carlo simulation. IEEE Trans Biomed Eng (1999) 46:130–7. 10.1109/10.740875 9932334

[43] Bonate P. Pharmacokinetic-Pharmacodynamic modeling and simulation. New York: Springer US (2011). 10.1007/978-1-4419-9485-1

[44] Agbaje OF Luzio SD Albarrak AI Lunn DJ Owens DR Hovorka R. Bayesian hierarchical approach to estimate insulin sensitivity by minimal model. Clin Sci (2003) 105:551–60. 10.1042/CS20030117 12790795

[45] Denti P Bertoldo A Vicini P Cobelli C. IVGTT glucose minimal model covariate selection by nonlinear mixed-effects approach. Am J Physiol Endocrinol Metab (2010) 298:E950–60. 10.1152/ajpendo.00656.2009 PMC2867373 20103736

[46] Schumitzky A. EM algorithms and two stage methods in pharmacokinetic population analysis. In: D’Argenio DZ, editor. Advanced methods of pharmacokinetic and pharmacodynamic systems analysis, vol. 2 . New York: Plenum Press (1995). p. 145–60.

[47] Walker S. An EM Algorithm for Nonlinear Random Effects Models. Biometrics (1996) 52:934–44. 10.2307/2533054

[48] D’Argenio DZ Alan S Wang X. ADAPT 5 User"s Guide: Pharmacokinetic/Pharmacodynamic Systems Analysis Software. Los Angeles: Biomedical Simulations Resources (2009).

[49] Bergman RN Lovejoy JC. The Minimal Model Approach and Determinants of Glucose Tolerance. Pennington Center Nutrition Series: Louisiana State University Press (1997).

[50] Kahn SE Prigeon RL McCulloch DK Boyko EJ Bergman RN Schwartz MW. The contribution of insulin-dependent and insulin-independent glucose uptake to intravenous glucose tolerance in healthy human subjects. Diabetes (1994) 43:587–92. 10.2337/diab.43.4.587 8138065

[51] Pacini G Thomaseth K Ahrén B. Contribution to glucose tolerance of insulin-independent vs. insulin-dependent mechanisms in mice. Am J Physiol Endocrinol Metab (2001) 281:693–703. 10.1152/ajpendo.2001.281.4.e693 11551845

[52] Klein S Allison DB Heymsfield SB Kelley DE Leibel RL Nonas C. Waist circumference and cardiometabolic risk: A consensus statement from shaping America’s health: Association for weight management and obesity prevention; NAASO, the obesity society; the American society for nutrition; and the American diabetes associat. Am J Clin Nutr (2007) 15:1197–202. 10.1093/ajcn/85.5.1197 17490953

[53] Ward GM Walters JM Barton J Alford FP Boston RC. Physiologic modeling of the intravenous glucose tolerance test in type 2 diabetes: A new approach to the insulin compartment. Metabolism (2001) 50:512–9. 10.1053/meta.2001.21029 11319711

[54] Martin B Warram J Krolewski A Bergman R Soeldner J Kahn C. Role of glucose and insulin resistance in development of type 2 diabetes mellitus: results of a 25-year follow-up study. Lancet (1992) 340:925–9. 10.1016/0140-6736(92)92814-V 1357346

[55] Ahrén B Pacini G. Glucose effectiveness: Lessons from studies on insulin-independent glucose clearance in mice. J Diabetes Invest (2020). 10.1111/jdi.13446 PMC8088998 33098240

[56] Karstoft K Clark MA Jakobsen I Knudsen SH van Hall G Pedersen BK. Glucose effectiveness, but not insulin sensitivity, is improved after short-term interval training in individuals with type 2 diabetes mellitus: a controlled, randomised, crossover trial. Diabetologia (2017) 60:2432–42. 10.1007/s00125-017-4406-0 28842722

[57] Seufert J. SGLT2 inhibitors - an insulin-independent therapeutic approach for treatment of type 2 diabetes: focus on canagliflozin. Diabetes Metab Syndr Obes (2015) 8:543–54. 10.2147/DMSO.S90662 PMC4644173 26609242

