# Supplementary material for: An Analysis of Glucose Effectiveness in Subjects With or Without Type 2 Diabetes via Hierarchical Modeling
Source: Front Endocrinol (Lausanne). 2021 Mar 29;12:641713. doi: 10.3389/fendo.2021.641713 (PMC8039510; doi:10.3389/fendo.2021.641713)
Supplement: Supplementary file 1 [file DataSheet_1.pdf]

An Analysis of Glucose Effectiveness in Subjects with or without Type 2 Diabetes via Hierarchical Modeling

Shihao Hu, Yuzhi Lu, Andrea Tura, Giovanni Pacini, David Z. D'Argenio

Supplemental Information

The material in this supplement is organized as follows:

S1. Overview of Hierarchical Modeling Approach and Solution via the EM Algorithm

S2. Complete set of MM Predictions of the Glucose-Time Measurements for all Subjects

S1. Overview of Hierarchical Modeling Approach and Solution via the EM Algorithm

S1.1 The Population Model and Estimation Problem

The dynamic systems model framework for an individual can be extended to a population of N individuals, for the case of no dynamic process error, as follows (also see D'Argenio et al. 2009 in the manuscript):

$$Y_i = h_i(\theta_i) + e_i, \quad i = 1, ..., N \qquad (1)$$

where θ_i represents the vector of system parameters for the ith individual, $h_i(\theta_i)$ is the vector of model outputs for the ith individual (constructed from the solution of the dynamic model – e.g., the glucose-insulin minimal model), e_i is the vector of associated output errors, and Y_i is the vector of all measurements for the ith individual. Assuming independence of output errors, it is further assumed that $e_i \sim N(0, G_i\left(h_i(\theta_i), \beta\right))$, where β represents the vector of parameters that are unique to the error variance model (assumed common across individuals) and $G_i\left(h_i(\theta_i), \beta\right)$ is a positive definite covariance matrix (D'Argenio et al. 2009). For convenience $G_i\left(\theta_i, \beta\right) \equiv G_i\left(h_i(\theta_i), \beta\right)$.

To account for the differences between individuals, the system parameters θ_i are assumed to be independent, identically distributed random vectors as follows:

$$\theta_i \sim_{i.i.d.} N(\mu, \Sigma) \text{ or } LN(\mu, \Sigma) \qquad (2)$$

This basic parametric population model can be extended to incorporate subject specific measured covariates to explain some of the inter-individual variability as follows:

$$\theta_i \sim_{i.i.d.} N(\mu_i, \Sigma) \text{ or } LN(\mu_i, \Sigma) \quad \text{where } \mu_i = v(c, r_i) \qquad (3)$$

In Eq. (3), $v(c,r_i)$ is a general (linear or nonlinear in r_i) user defined covariate model relating any measured covariates from the ith individual to the population mean for the ith individual (μ_i). The vector r_i is the collection of known, time-invariant covariates for the ith individual. The vector c represents the covariate model parameters (assumed common across individuals).

The population model can be summarized as follows using the hierarchical modeling framework:

Stage 1 - PK/PD system and observation model (intra-individual variation):

$$Y_i = h_i(\theta_i) + e_i, \quad i = 1,\ldots,N$$

$$e_i \sim N\left(0, G_i\left(\theta_i, \beta\right)\right)$$

Stage 2 - Parameter model (inter-individual variation):

$$\theta_i \sim_{i.i.d.} N(\mu, \Sigma) \text{ or } LN(\mu, \Sigma) \quad \text{without covariates}$$

$$\theta_i \sim_{i.i.d.} N(v(c, r_i), \Sigma) \text{ or } LN(v(c, r_i), \Sigma) \quad \text{with covariates}$$

The population estimation problem involves estimating the parameters (μ, Σ, β) or (c, Σ, β), as well as $(\theta_i,\, i = 1,\ldots,N)$, given all the population data $\{Y_1,\ldots,Y_N\}$.

S1.2 Maximum Likelihood Solution via the EM Algorithm

The expectation-maximization (EM) algorithm introduced in 1977 by Dempster, Laird and Rubin to solve an important class of maximum likelihood problems, has been applied widely to solve linear mixed effects models. Schumitzky 1995 (see citation in manuscript), applied the EM algorithm to solve the nonlinear mixed effects maximum likelihood estimation problem and suggested the use of sampling-based methods (including importance sampling) to calculate the

required integrals. In a 1996 paper, Walker also applied the EM algorithm to this problem and extended it to including the case of linear covariate models. In addition, he provided an error analysis for the maximum likelihood estimates and illustrated its application. (See D'Argenio et al, 2009 for further discussion.) The use of the EM algorithm with sampling-based methods now provides a powerful and computationally practicable method for solving the nonlinear maximum likelihood hierarchical modeling problems, without recourse to model linearization or analytic likelihood approximation (and thus without the resulting estimator bias). We present below the solution to the problem without covariates; see D'Argenio et al, 2009 for solution to the covariate problem.

S1.3 The Likelihood Function and the Estimation Problem

Given the problem statement above define:

$$p\left(Y_i \mid \theta_i, \beta\right) = N\left(h_i\left(\theta_i\right), G_i\left(\theta_i, \beta\right)\right)$$

$$p\left(\theta_i \mid \mu, \Sigma\right) = N\left(\mu, \Sigma\right)$$

The case when the systems parameters are distributed log normally is handled by transformation.

From the independence of the $\{Y_1, \ldots, Y_N\}$, the overall data likelihood function is then given by:

$$L\left(\mu, \Sigma, \beta\right) = \prod_{i=1}^{N} \int p\left(Y_i \mid \theta_i, \beta\right) p\left(\theta_i \mid \mu, \Sigma\right) d\theta_i \qquad (4)$$

Defining $\phi = \left(\mu, \Sigma, \beta\right)$, the maximum likelihood estimator of ϕ is then: $\phi_{ML} = \arg\max L(\phi)$.

S1.4 The EM Algorithm Solution

As shown by Schumitzky 1995 and also by Walker 1996, the EM algorithm given below solves (locally) the maximum likelihood estimation problem defined above. Given initial guesses for the population, error variance and individual subject parameters ($\mu^{(0)}$ $\Sigma^{(0)}$ $\beta^{(0)}$ $\theta_i^{(0)}$), the EM algorithm proceeds in two steps. In Step 1 (estimation or E step) the conditional mean and covariance for each individual's parameters are estimated, while Step 2 (Maximization-M step)

updates the population mean, covariance and error variance parameters. These two steps, defined below for the kth iteration, are then iterated until convergence.

Step 1

$$\bar{\theta}_i^{(k)} = E\left[\theta_i \mid Y_i,\ \phi^{(k)}\right] \tag{5}$$

$$\bar{\Omega}_i^{(k)} = E\left[\left(\theta_i - \bar{\theta}_i^{(k)}\right)\left(\theta_i - \bar{\theta}_i^{(k)}\right)^T \mid Y_i,\ \phi^{(k)}\right] \tag{6}$$

$$i = 1,\ \ldots,\ N$$

Step 2

$$\mu^{(k+1)} = \frac{1}{N}\sum_{i=1}^{N}\bar{\theta}_i^{(k)} \tag{7}$$

$$\Sigma^{(k+1)} = \frac{1}{N}\sum_{i=1}^{N}\left\{\left(\bar{\theta}_i^{(k)} - \mu^{(k+1)}\right)\left(\bar{\theta}_i^{(k)} - \mu^{(k+1)}\right)^T + \bar{\Omega}_i^{(k)}\right\} \tag{8}$$

$$\beta^{(k+1)} = \beta^{(k)} - H\left(\phi^{(k)}\right)^{-1}\frac{\partial\left(-\log L\left(\phi^{(k)}\right)\right)}{\partial\beta} \tag{9}$$

Letting $H(\phi^{(k)}) = \left\{h_{j1,j2}\right\},\ j1, j2 = 1,\ldots,q$ and $\partial\left(-\log L\left(\phi^{(k)}\right)\right)/\partial\beta = \left\{L_{\beta_{j1}}\right\},\ j1 = 1,\ldots,q$ and also defining $Y_i = \left\{y_{ij}\right\},\ \ h_i\left(\theta_i\right) = \left\{h_{ij}\left(\theta_i\right)\right\}$ and $G_i\left(\theta_i, \beta\right) = diag\left\{g_{ij}\left(\theta_i, \beta\right)\right\},\ \ j = 1,\ldots,m_i$ (m_i is the total number of observations for the ith subject), then terms in Eq. (4.9) are constructed from:

$$h_{j1,j2} = \frac{1}{2}\sum_{i=1}^{N}E\left[\sum_{j=1}^{m_i}\frac{1}{g_{ij}^2\left(\theta_i,\beta\right)}\left(\frac{\partial g_{ij}\left(\theta_i,\beta\right)}{\partial\beta_{j1}}\right)\left(\frac{\partial g_{ij}\left(\theta_i,\beta\right)}{\partial\beta_{j2}}\right) \mid Y_i,\ \phi^{(k)}\right]$$

$$L_{\beta_{j1}} = \frac{1}{2}\sum_{i=1}^{N}E\left[\sum_{j=1}^{m_i}\frac{1}{g_{ij}^2\left(\theta_i,\beta\right)}\left(\frac{\partial g_{ij}\left(\theta_i,\beta\right)}{\partial\beta_{j1}}\right)\left(g_{ij}\left(\theta_i,\beta\right) - \left(y_{ij} - h_{ij}\left(\theta_i\right)\right)^2\right) \mid Y_i,\ \phi^{(k)}\right]$$

The resulting sequence $\{\phi^{(k)}\}, k = 1,\ldots$ has the likelihood improving property $L(\phi^{k+1}) \geq L(\phi^k)$, and under sufficient conditions ϕ^k converges to a local maximum of the likelihood function in Eq. (4).

S1.5 The Conditional Mean and Covariance

The conditional mean and covariance in Eq. (5) and (6), as well as all of the other

expectations required above, are defined by the conditional distribution for θ_i :

$$p(\theta_i \,|\, Y_i,\, \mu,\, \Sigma,\, \beta) = \frac{p(Y_i \,|\, \theta_i,\, \beta)\, p(\theta_i \,|\, \mu,\, \Sigma)}{\int p(Y_i \,|\, \theta_i,\, \beta)\, p(\theta_i \,|\, \mu,\, \Sigma)\, d\theta_i}, \quad i = 1, \ldots, N \qquad (11)$$

All of the expectations required in the EM algorithm outlined above have the form $E\left[f(\theta_i) \right] = \int f(\theta_i)\, p(\theta_i \,|\, Y_i,\, \mu,\, \Sigma,\, \beta)\, d\theta_i$. Numerical approximation of these *p*-dimensional integrals is accomplished using importance sampling (see D'Argenio et al, 2009 for implementation details.)

S2. Complete set of MM Predictions of the Glucose-Time Measurements for all Subjects

Y(1) gf01101

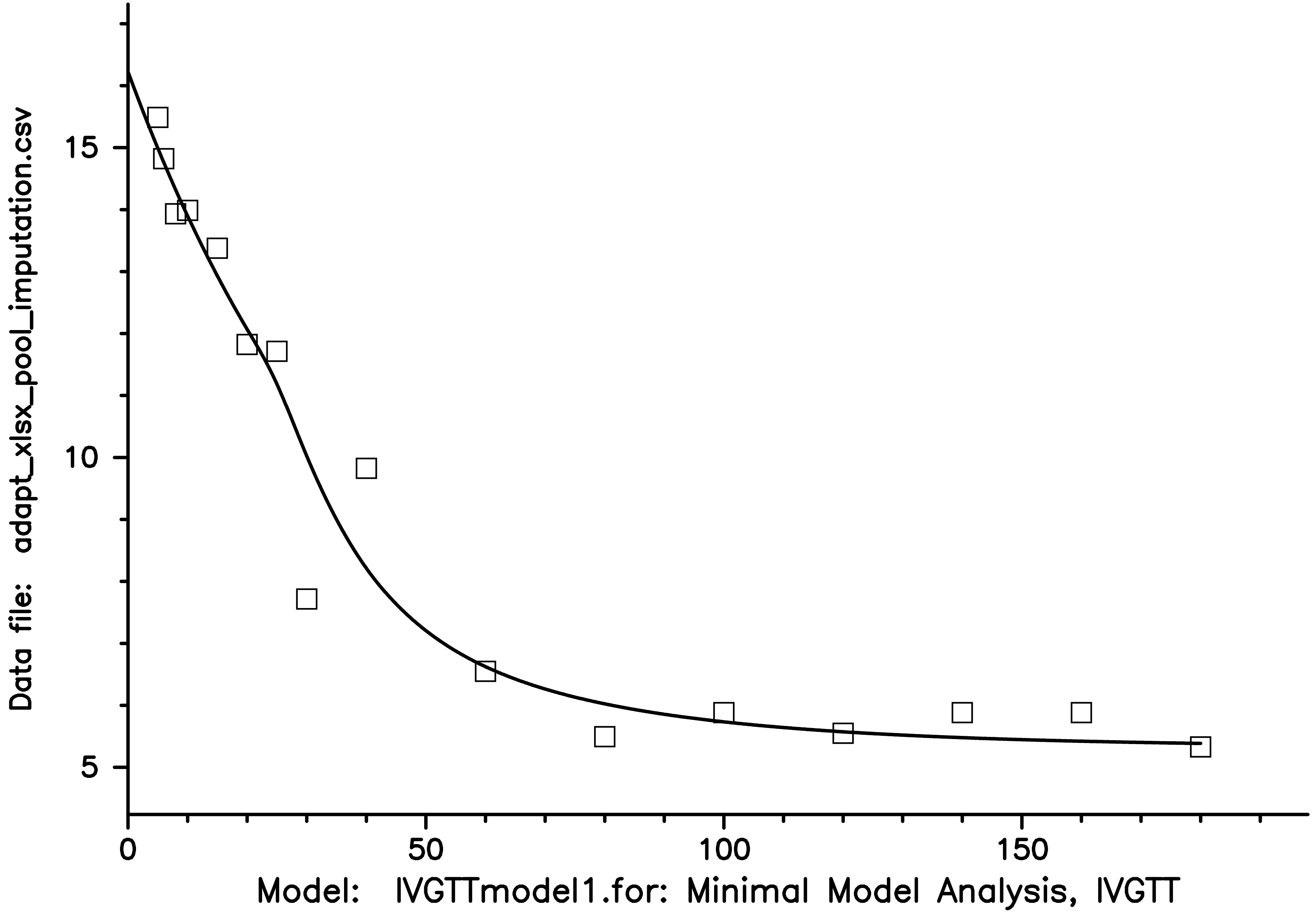

Data file: adapt_xlsx_pool_imputation.csv
15
10
5
0
50
100
150
Model: IVGTTmodel1.for: Minimal Model Analysis, IVGTT

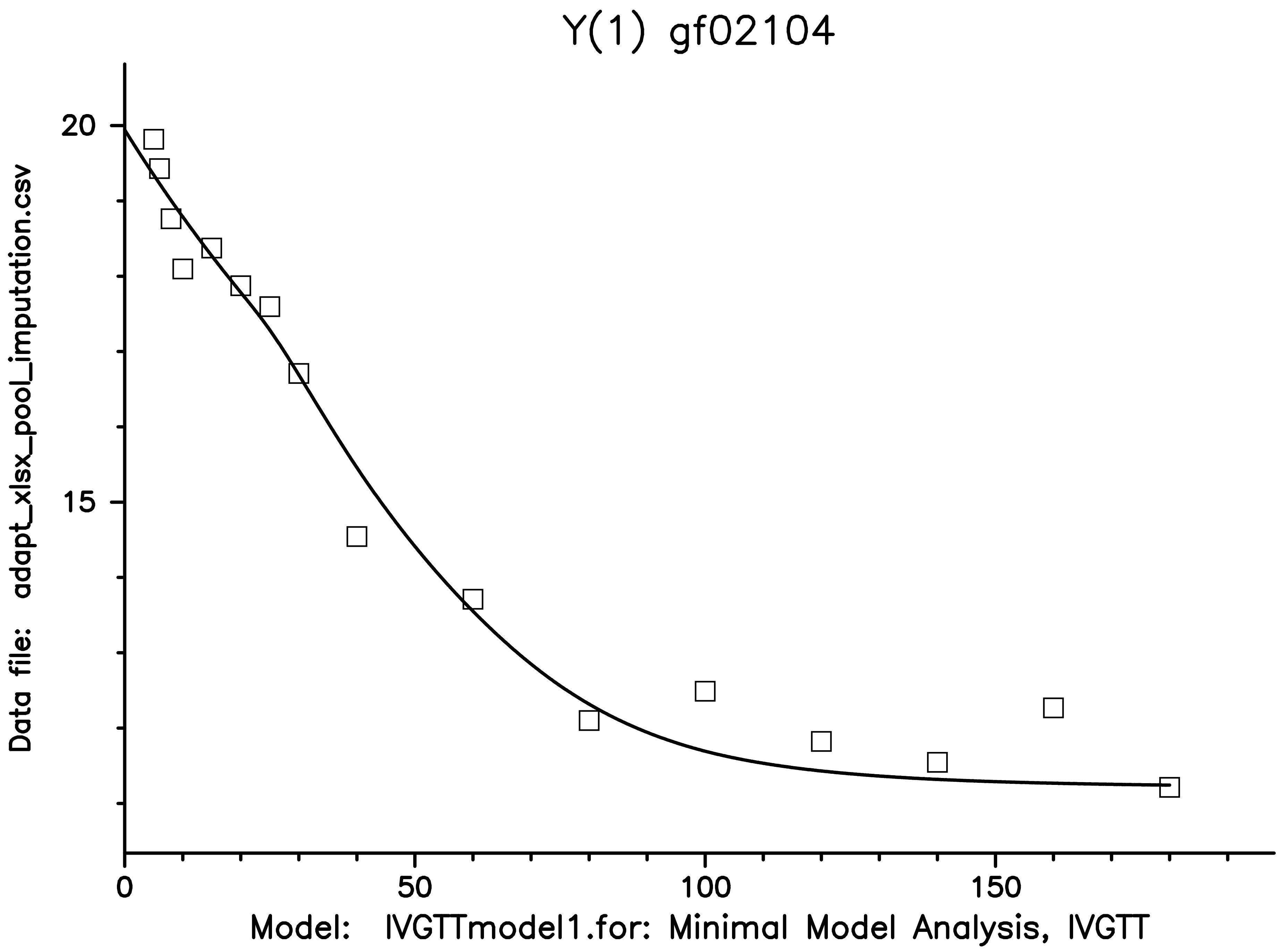
Y(1) gf02104
20
15
0
50
100
150
Data file: adapt_xlsx_pool_imputation.csv
Model: IVGTTmodel1.for: Minimal Model Analysis, IVGTT

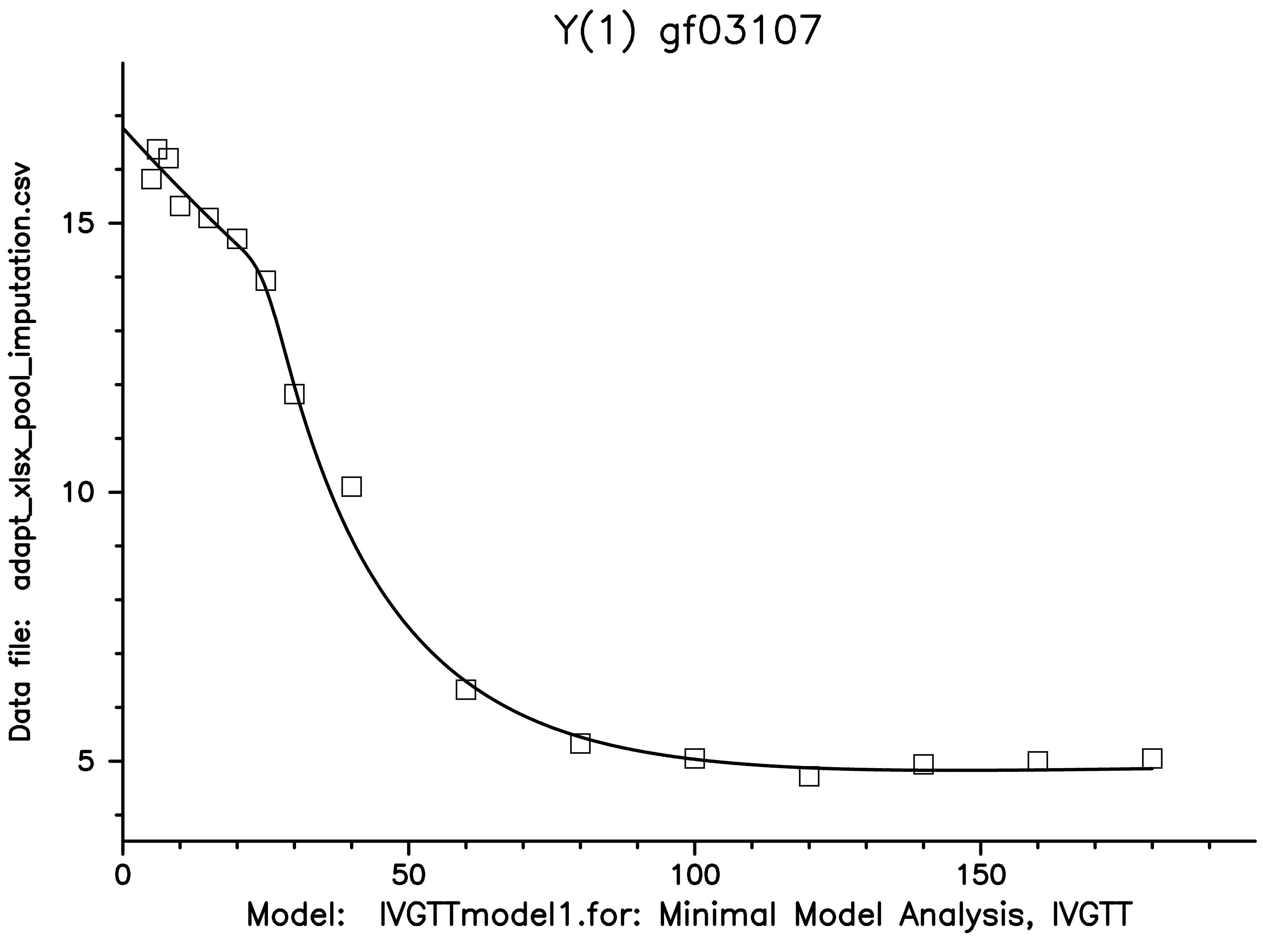
Y(1) gf03107
Data file: adapt_xlsx_pool_imputation.csv
15
10
5
0
50
100
150
Model: IVGTTmodel1.for: Minimal Model Analysis, IVGTT

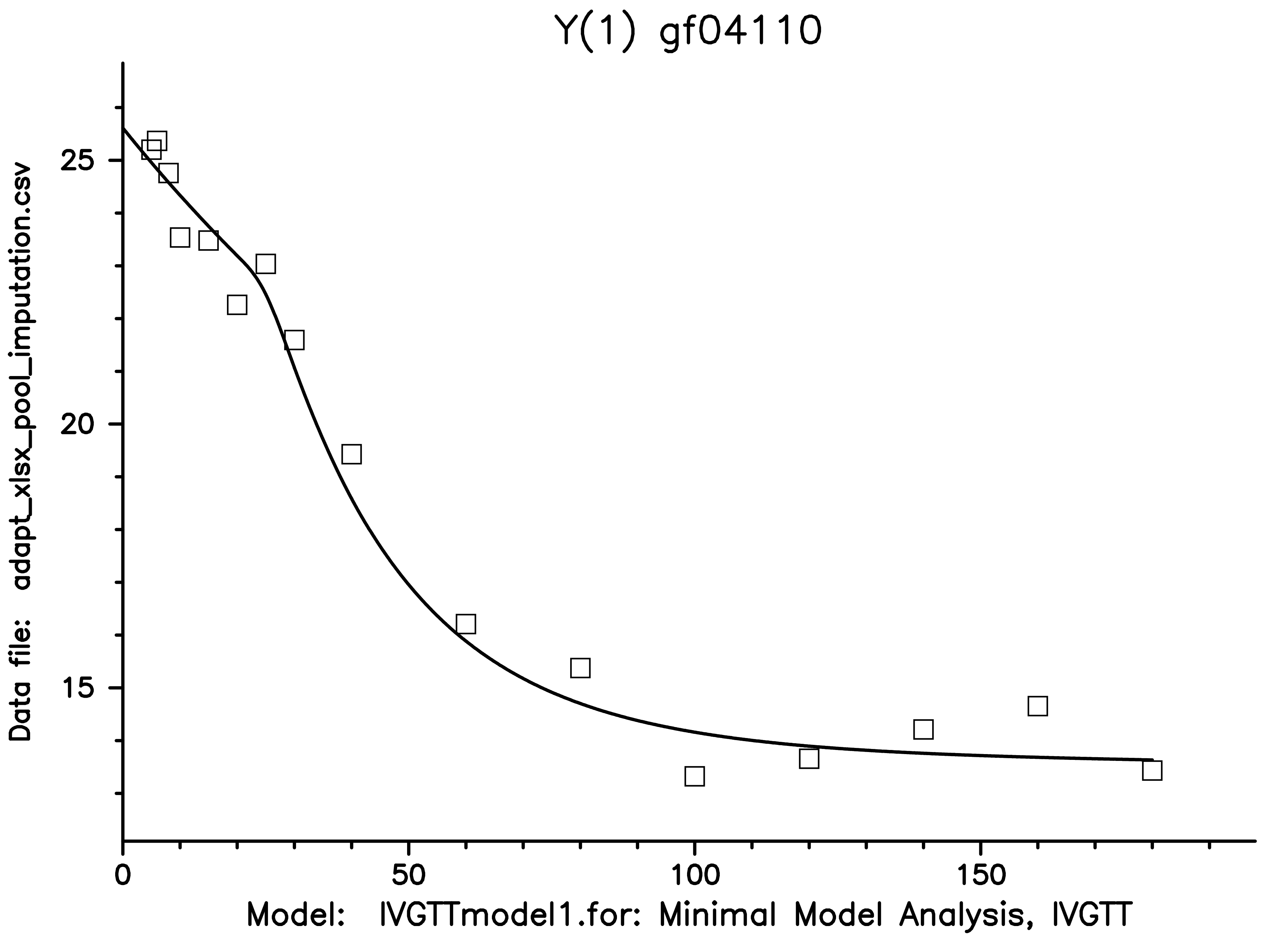

Y(1) gf04110
Data file: adapt_xlsx_pool_imputation.csv
25
20
15
0
50
100
150
Model: IVGTTmodel1.for: Minimal Model Analysis, IVGTT

Y(1) gf05113

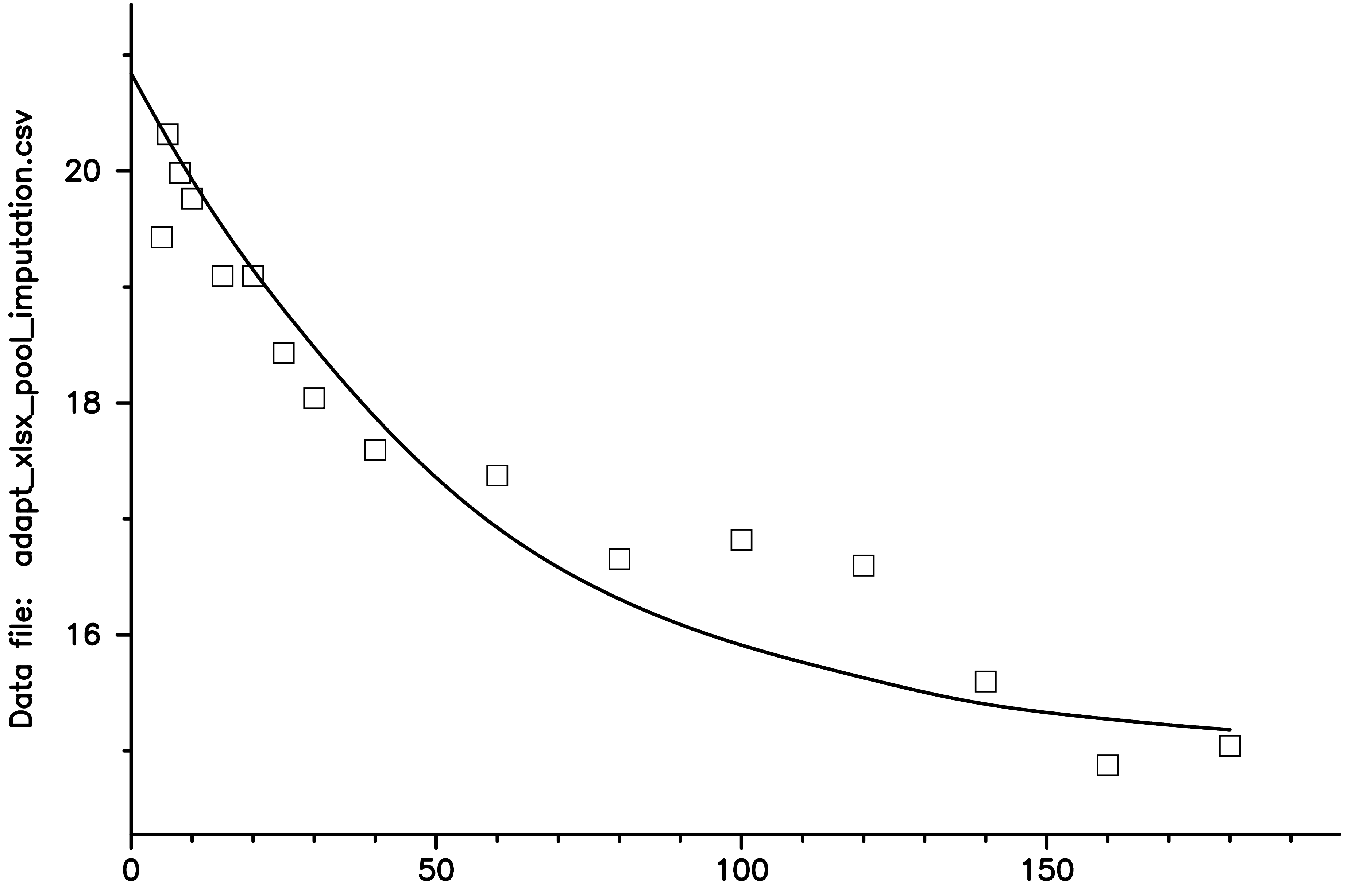

Model: IVGTTmodel1.for: Minimal Model Analysis, IVGTT

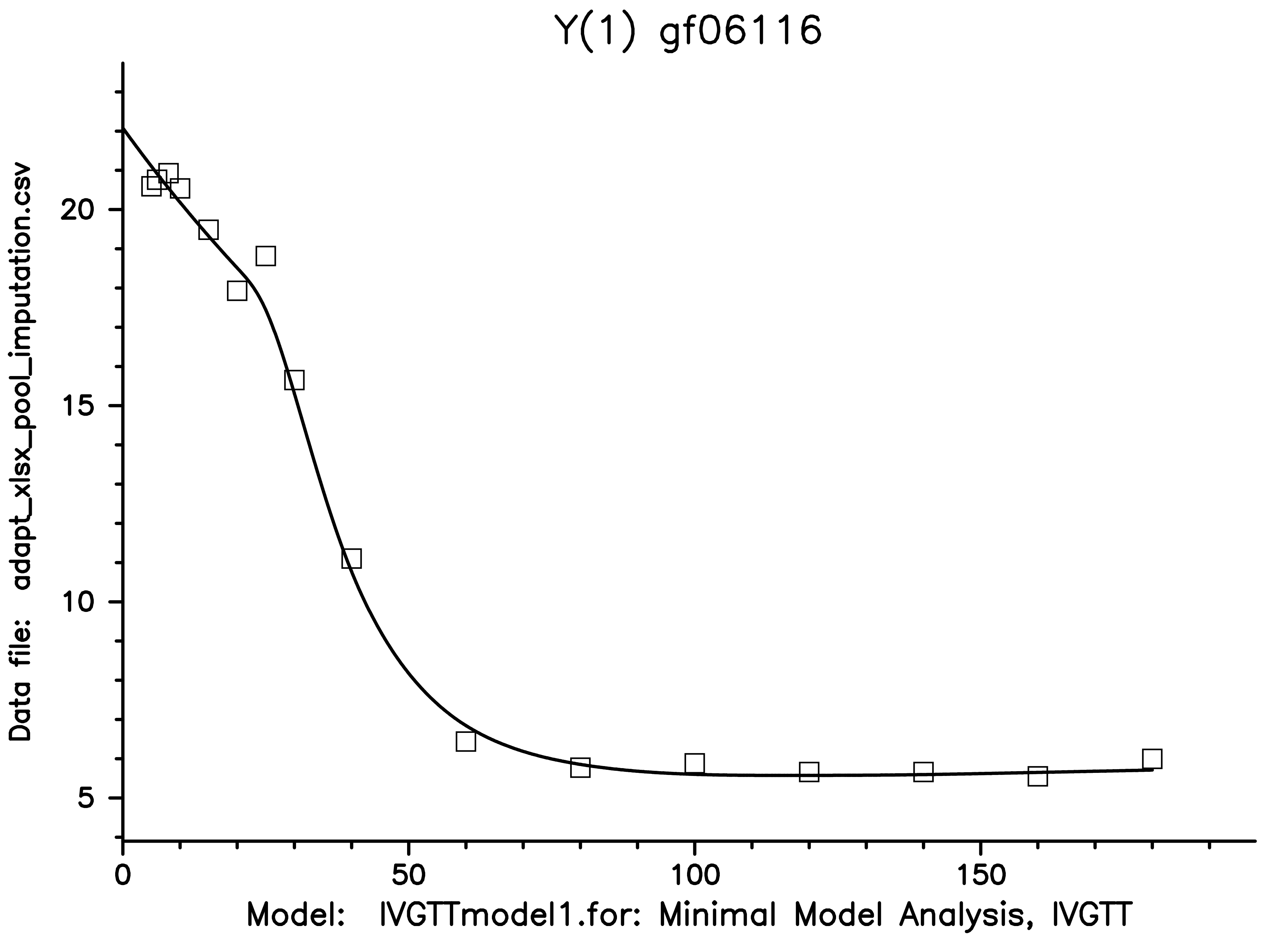

Y(1) gf06116
Data file: adapt_xlsx_pool_imputation.csv
20
15
10
5
0
50
100
150
Model: IVGTTmodel1.for: Minimal Model Analysis, IVGTT

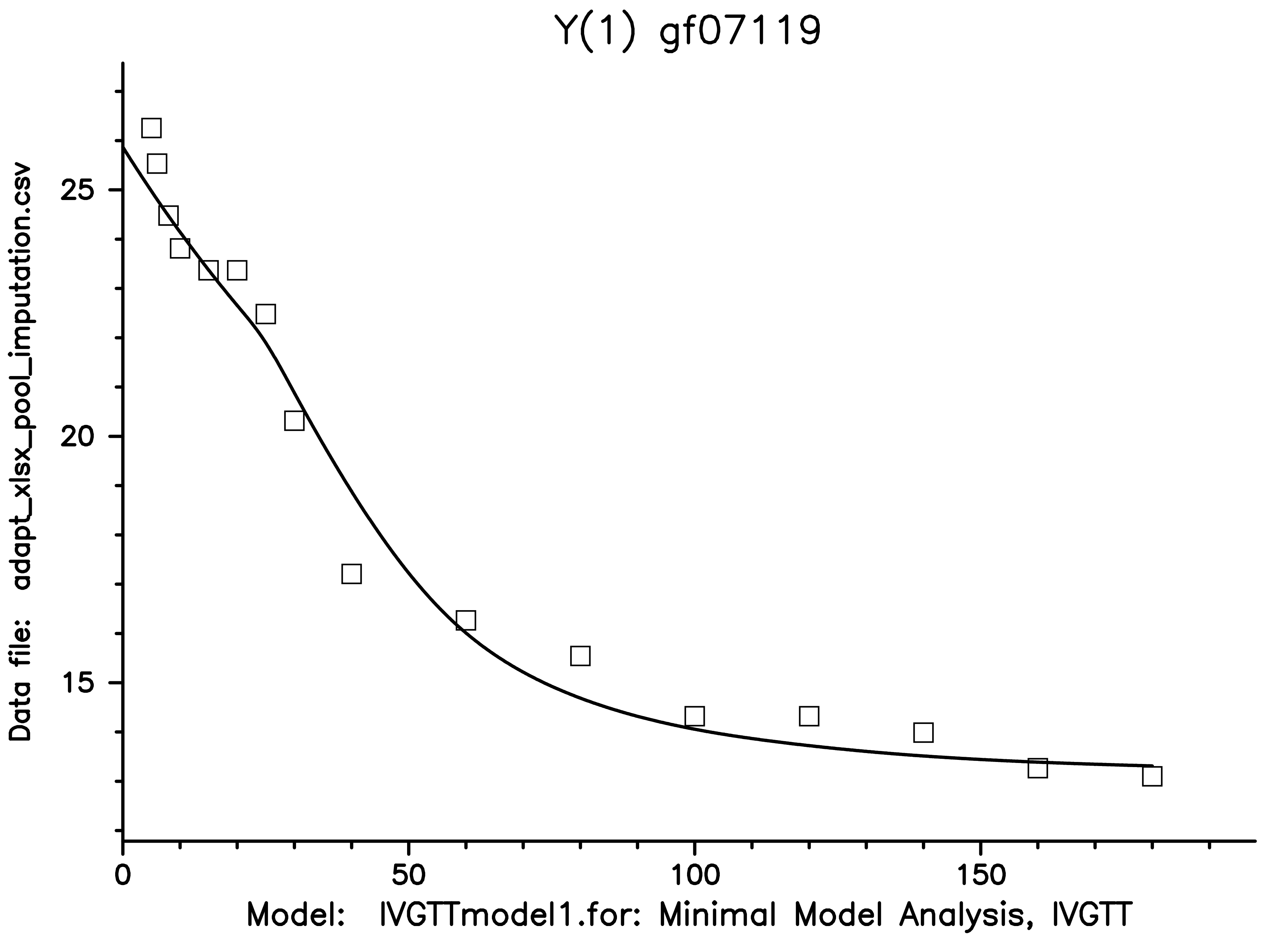
Y(1) gf07119
Data file: adapt_xlsx_pool_imputation.csv
25
20
15
0
50
100
150
Model: IVGTTmodel1.for: Minimal Model Analysis, IVGTT

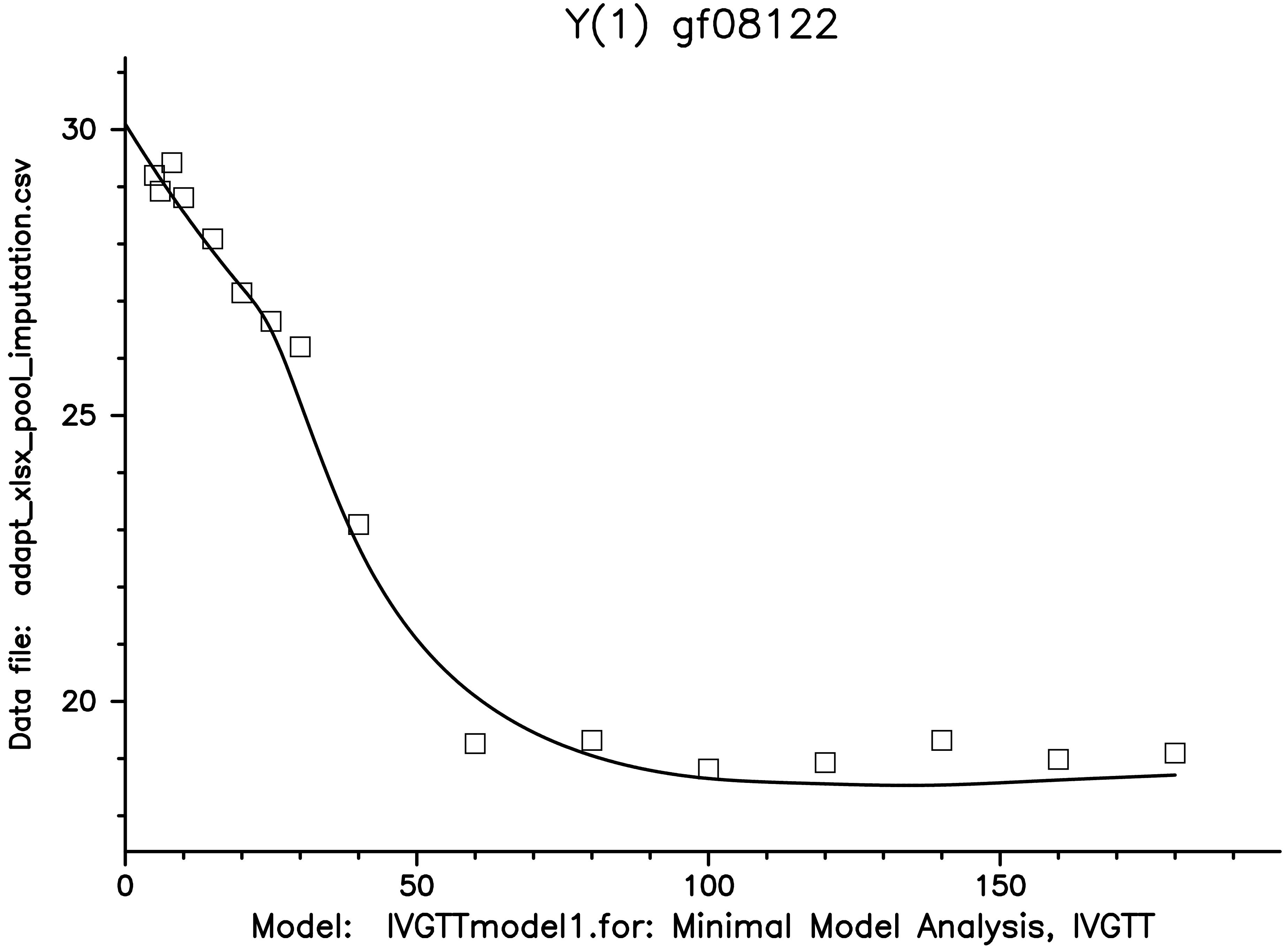

Y(1) gf08122
30
25
20
0
50
100
150
Data file: adapt_xlsx_pool_imputation.csv
Model: IVGTTmodel1.for: Minimal Model Analysis, IVGTT

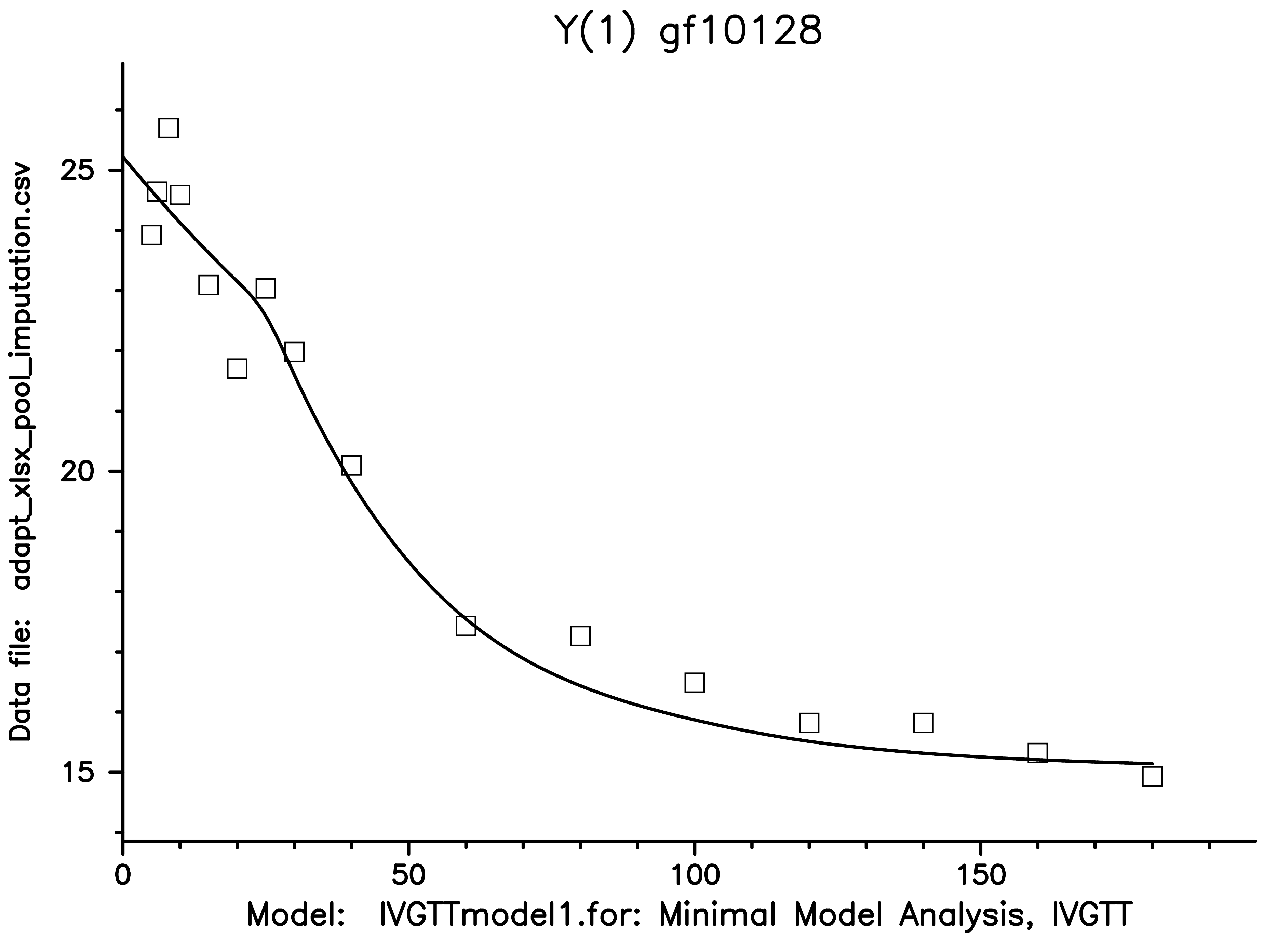
Y(1) gf10128
25
20
15
0
50
100
150
Model: IVGTTmodel1.for: Minimal Model Analysis, IVGTT
Data file: adapt_xlsx_pool_imputation.csv

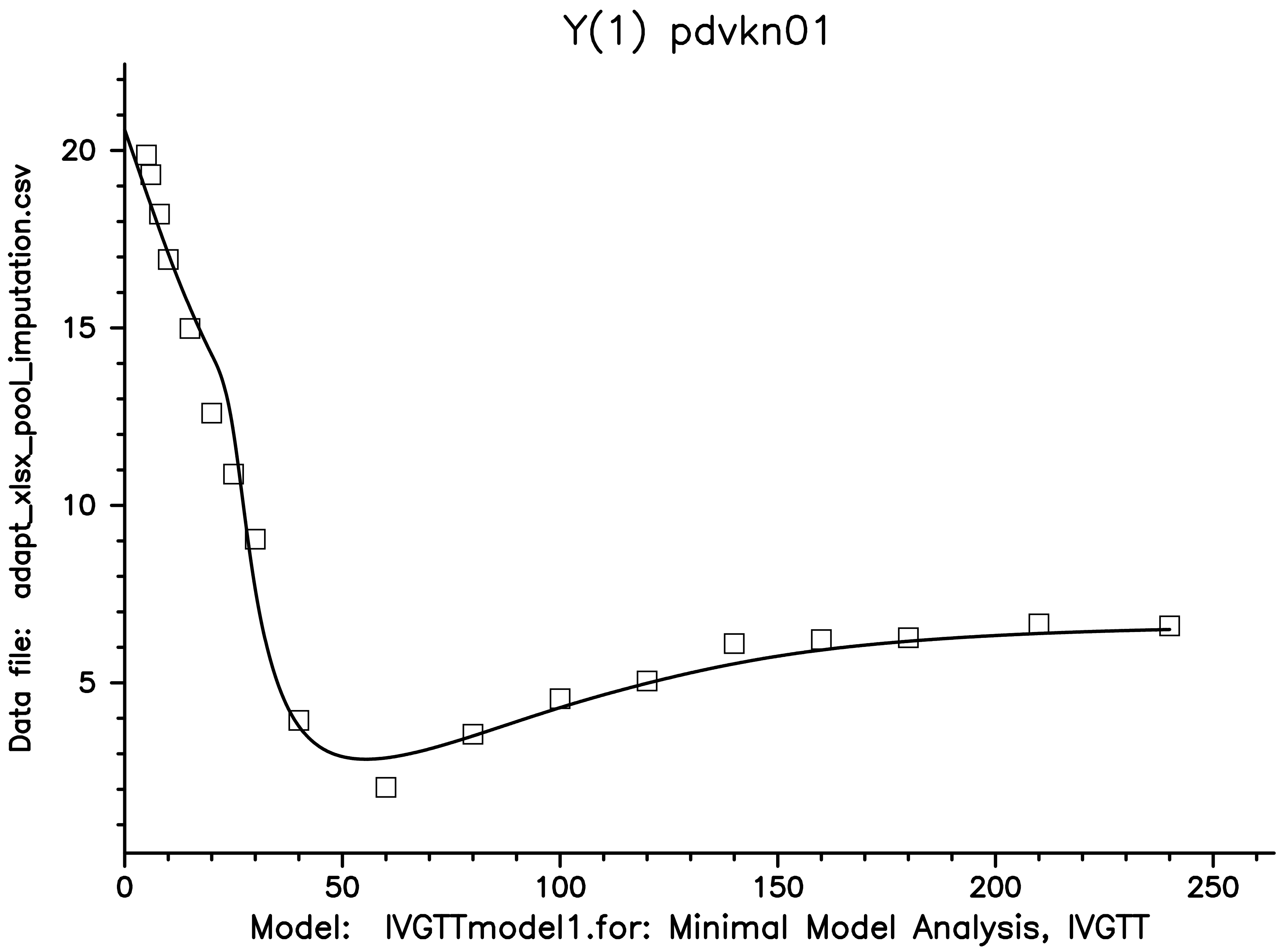
Y(1) pdvkn01
Data file: adapt_xlsx_pool_imputation.csv
20
15
10
5
0
50
100
150
200
250
Model: IVGTTmodel1.for: Minimal Model Analysis, IVGTT

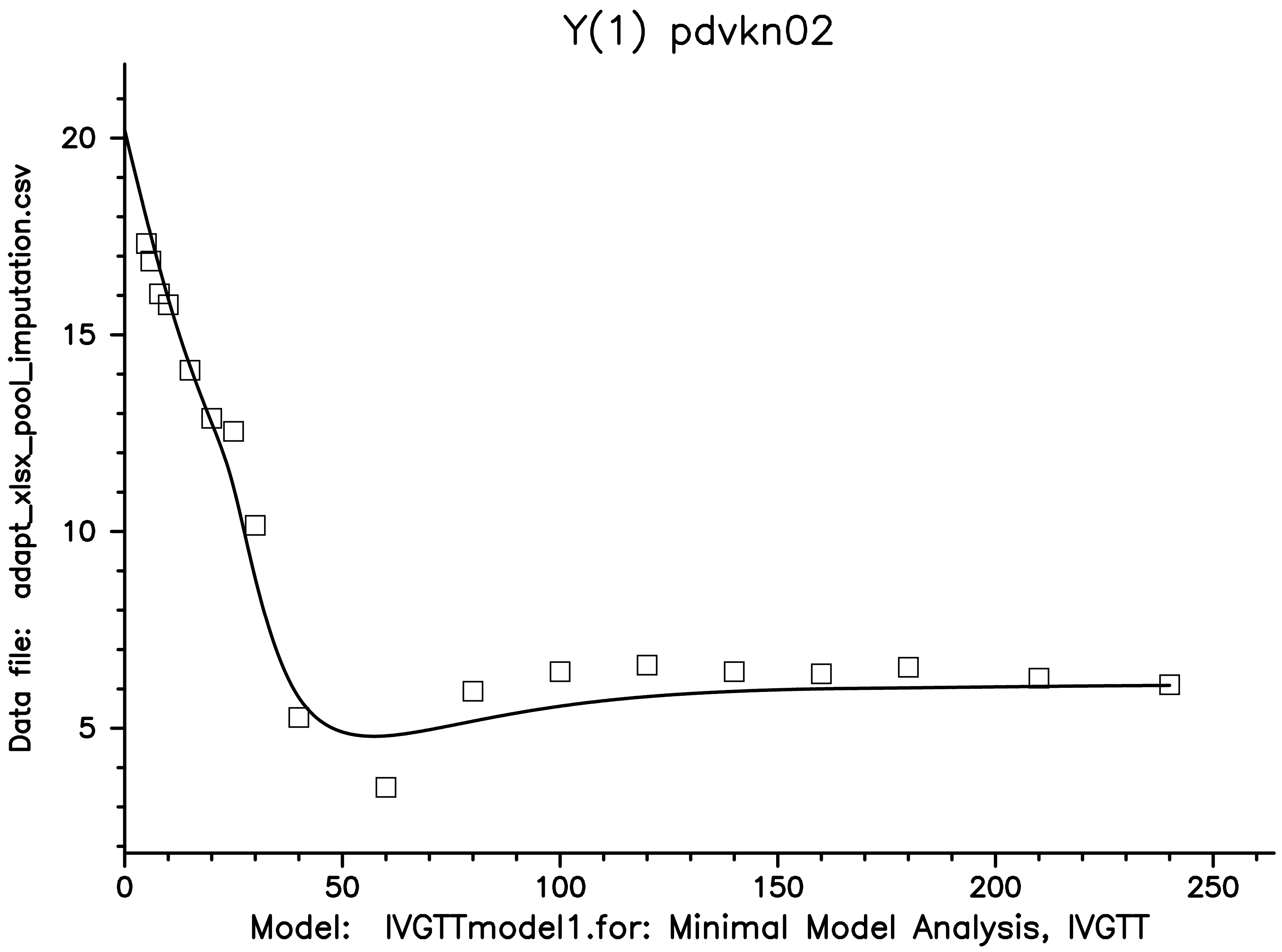
Y(1) pdvkn02
Data file: adapt_xlsx_pool_imputation.csv
Model: IVGTTmodel1.for: Minimal Model Analysis, IVGTT
20
15
10
5
0
50
100
150
200
250

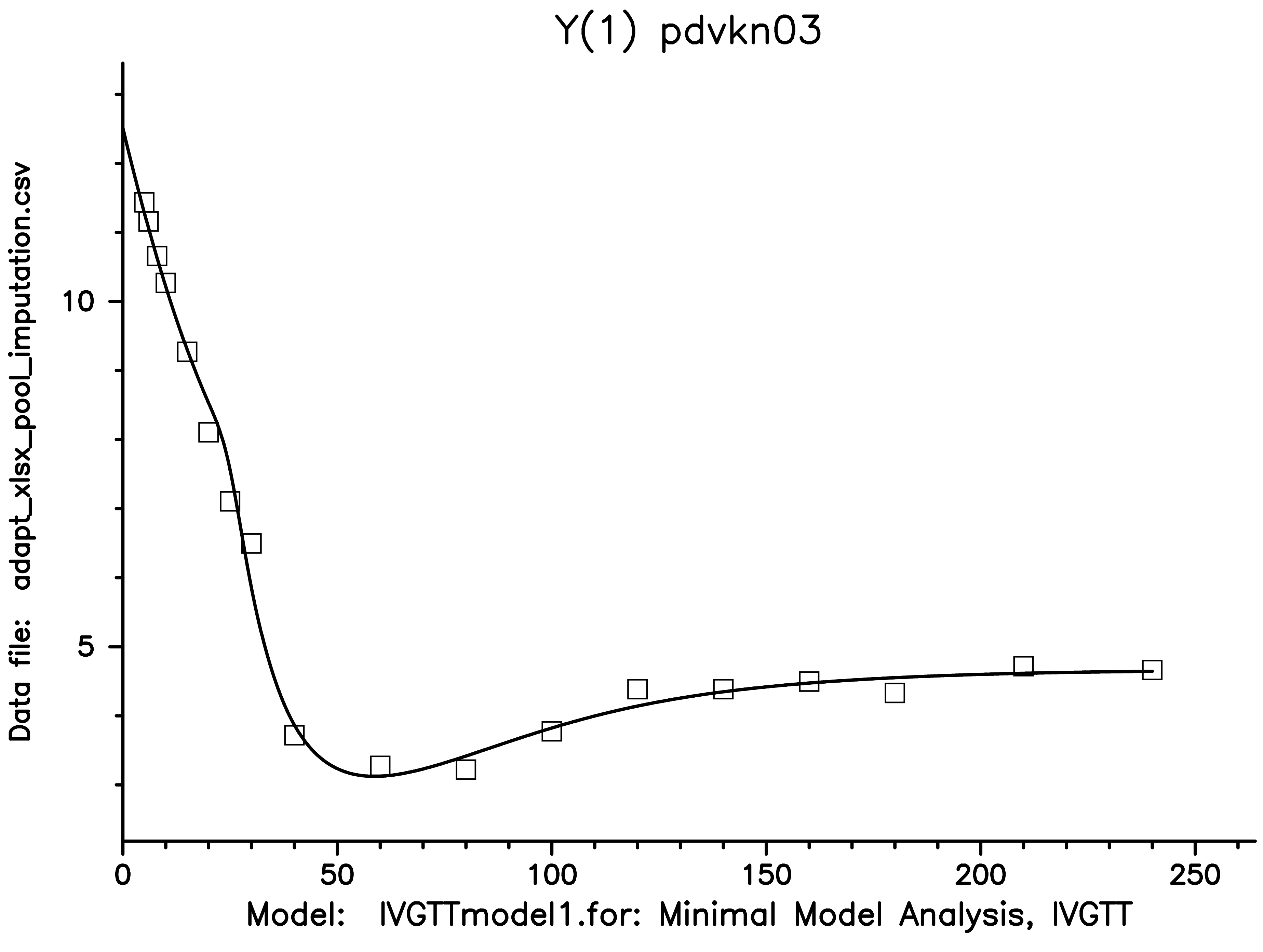
Y(1) pdvkn03
Data file: adapt_xlsx_pool_imputation.csv
10
5
0
50
100
150
200
250
Model: IVGTTmodel1.for: Minimal Model Analysis, IVGTT

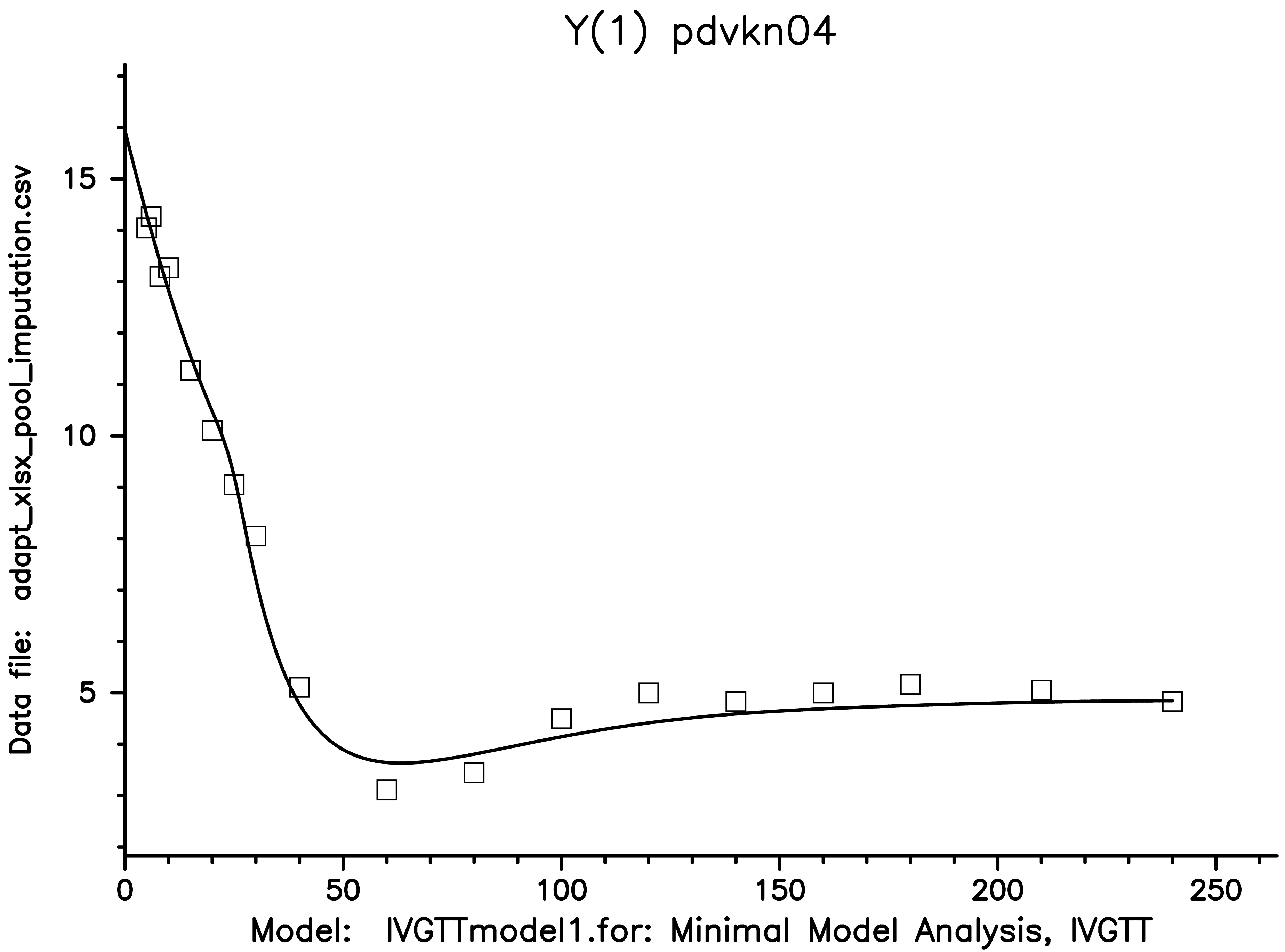
Y(1) pdvkn04
Data file: adapt_xlsx_pool_imputation.csv
15
10
5
0
50
100
150
200
250
Model: IVGTTmodel1.for: Minimal Model Analysis, IVGTT

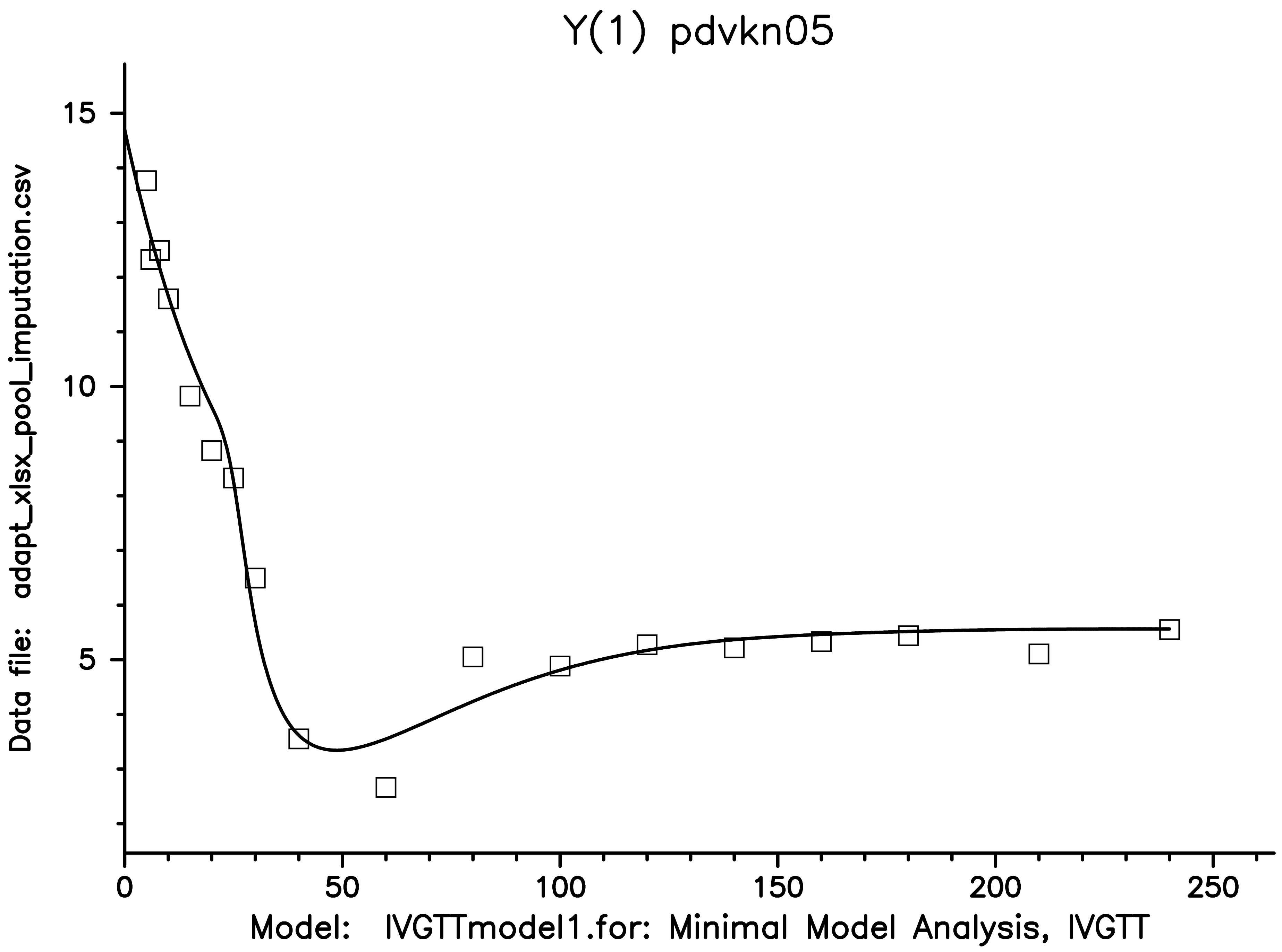
Y(1) pdvkn05
Data file: adapt_xlsx_pool_imputation.csv
Model: IVGTTmodel1.for: Minimal Model Analysis, IVGTT
15
10
5
0
50
100
150
200
250

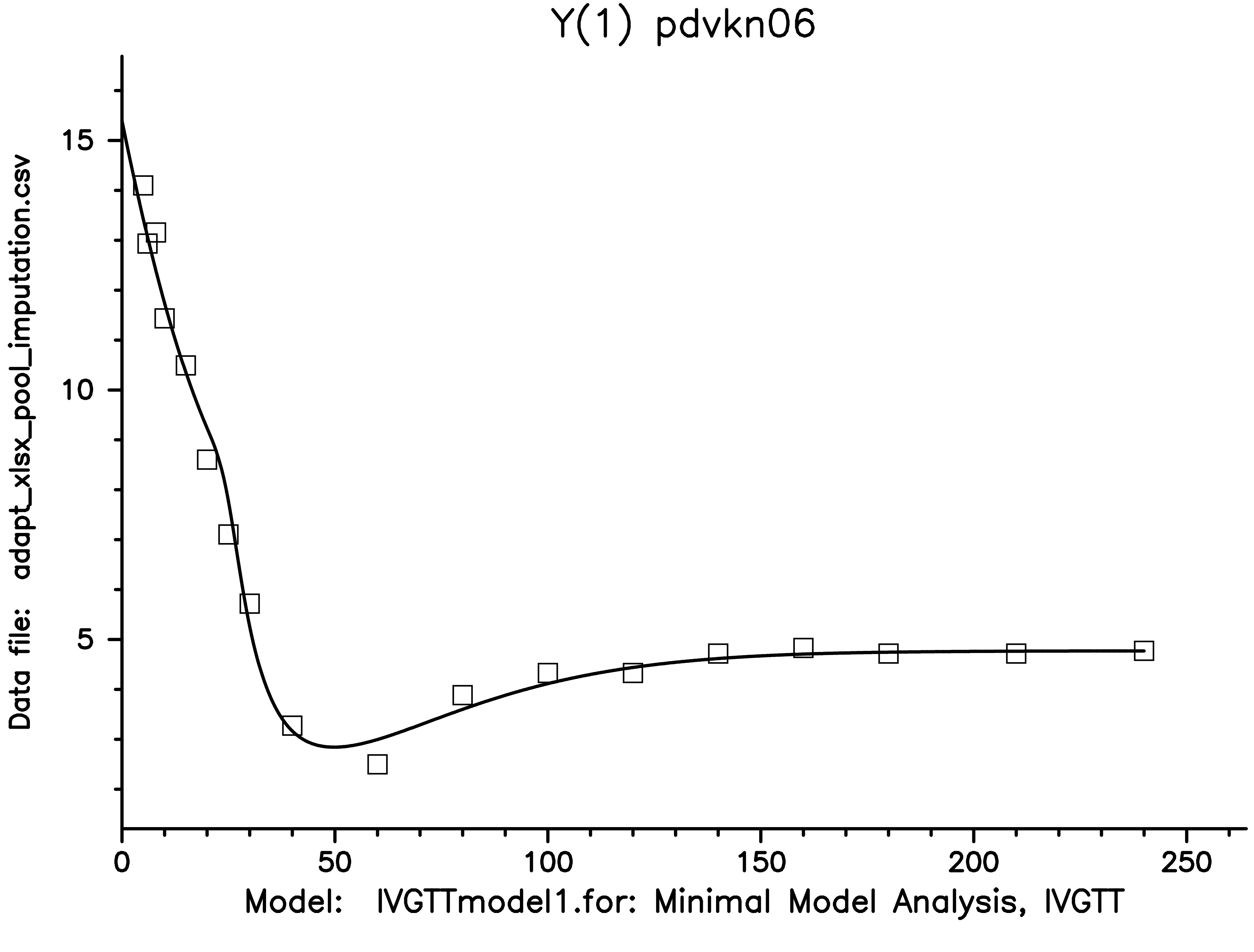

Y(1) pdvkn06
15
10
5
0
50
100
150
200
250
Data file: adapt_xlsx_pool_imputation.csv
Model: IVGTTmodel1.for: Minimal Model Analysis, IVGTT

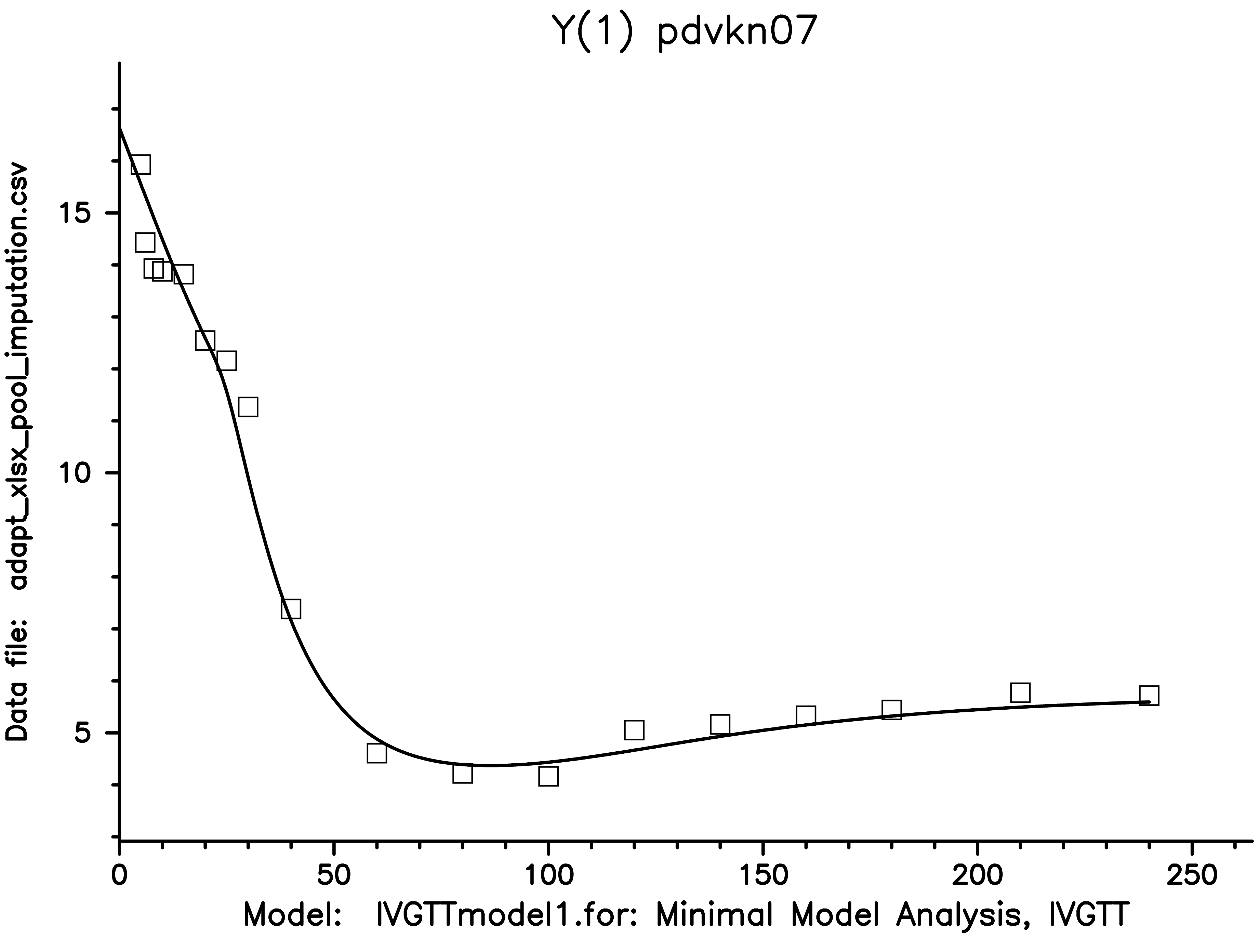
Y(1) pdvkn07
Data file: adapt_xlsx_pool_imputation.csv
Model: IVGTTmodel1.for: Minimal Model Analysis, IVGTT
15
10
5
0
50
100
150
200
250

Y(1) pdvkn08

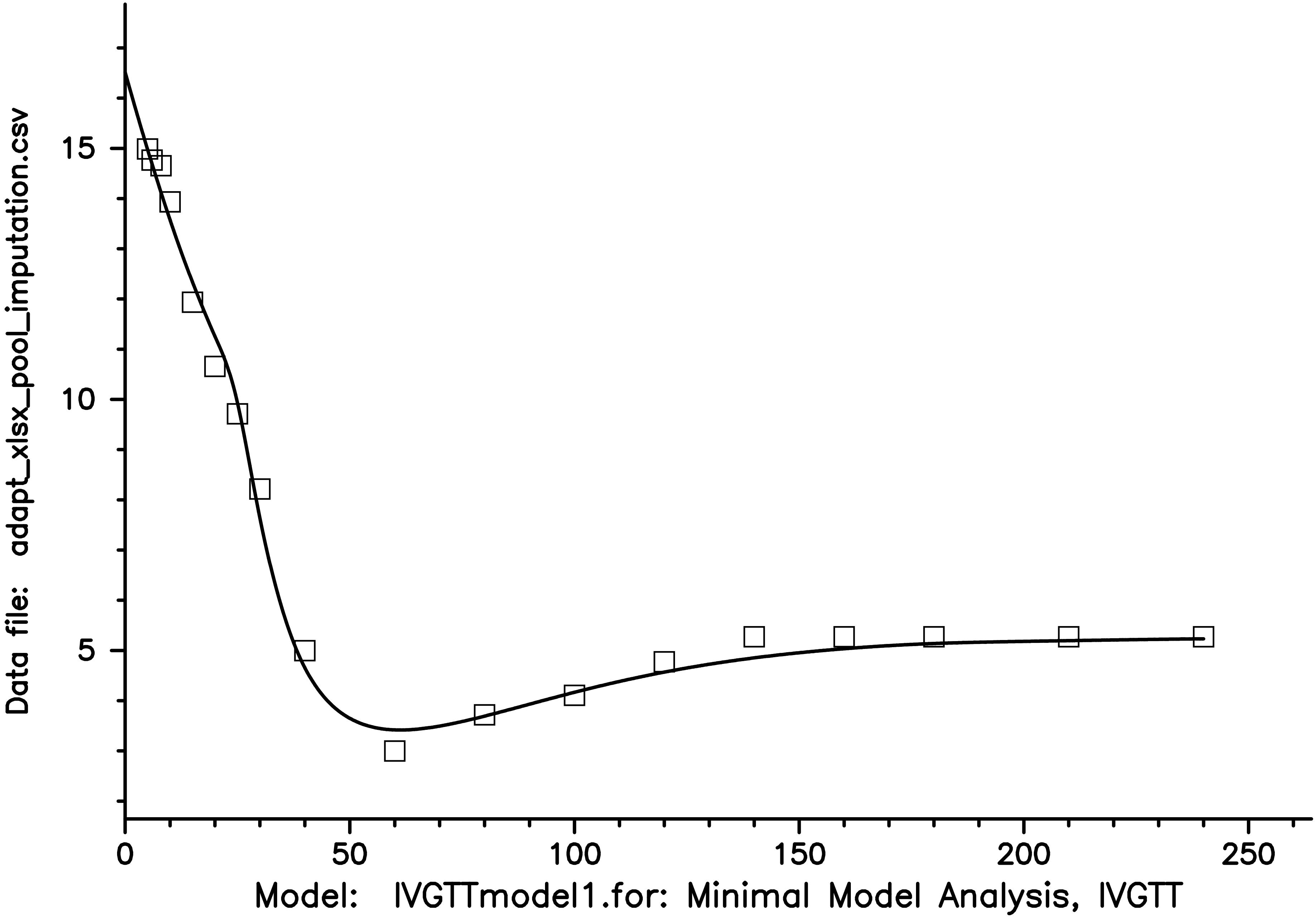

15
10
5
0
50
100
150
200
250
Data file: adapt_xlsx_pool_imputation.csv
Model: IVGTTmodel1.for: Minimal Model Analysis, IVGTT

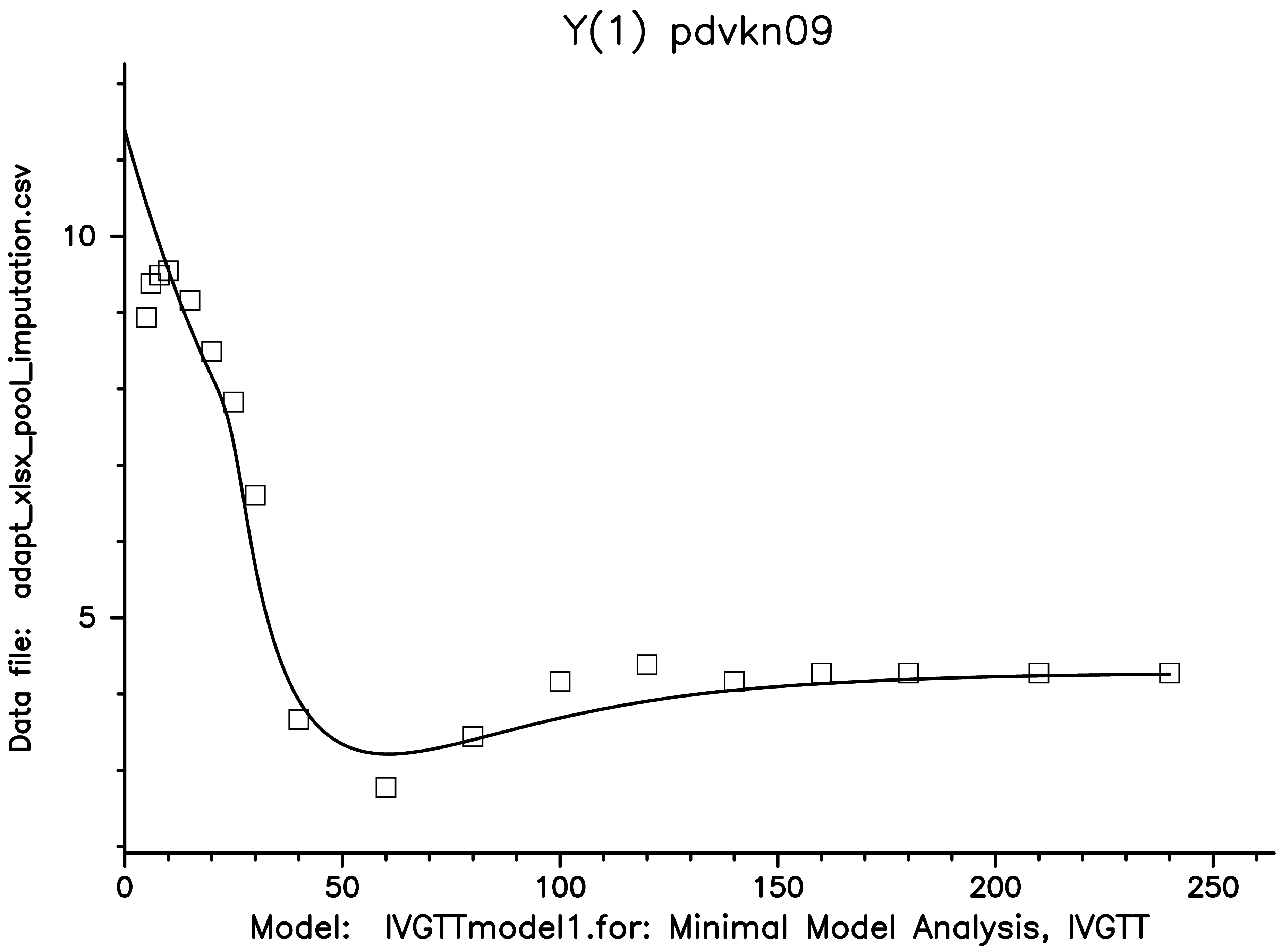
Y(1) pdvkn09
Data file: adapt_xlsx_pool_imputation.csv
10
5
0
50
100
150
200
250
Model: IVGTTmodel1.for: Minimal Model Analysis, IVGTT

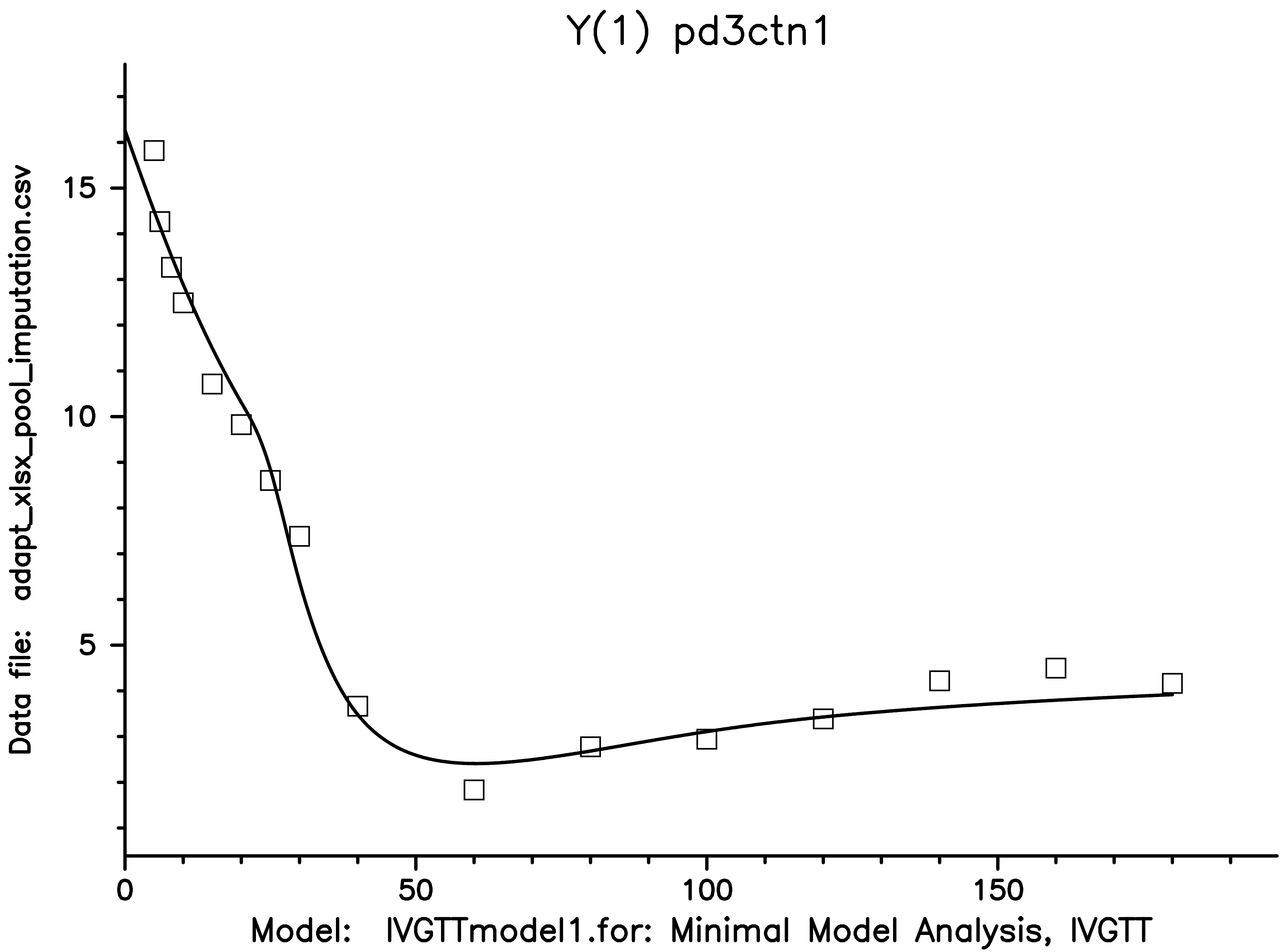
Y(1) pd3ctn1
Data file: adapt_xlsx_pool_imputation.csv
15
10
5
0
50
100
150
Model: IVGTTmodel1.for: Minimal Model Analysis, IVGTT

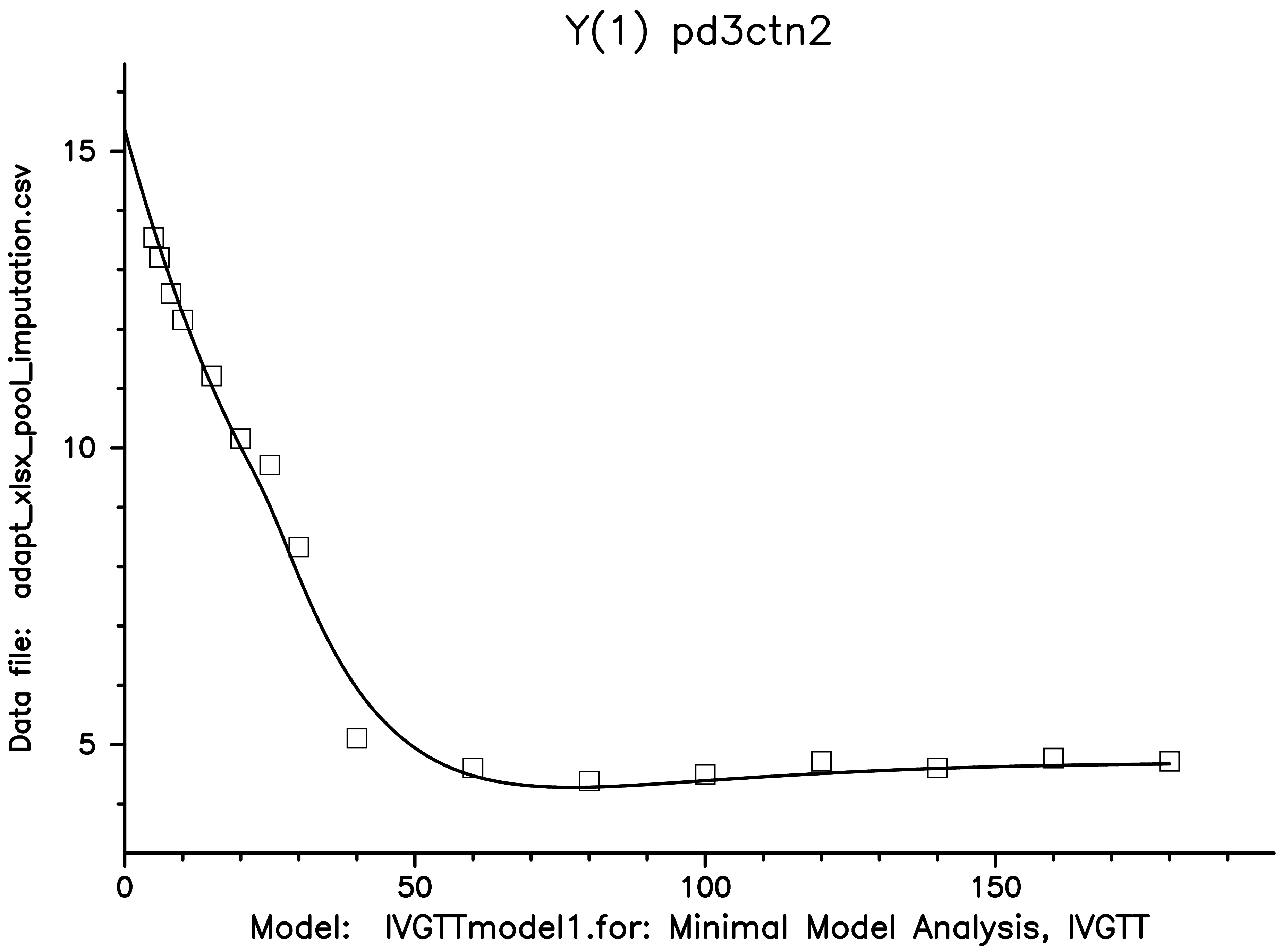

Y(1) pd3ctn2
Data file: adapt_xlsx_pool_imputation.csv
15
10
5
0
50
100
150
Model: IVGTTmodel1.for: Minimal Model Analysis, IVGTT

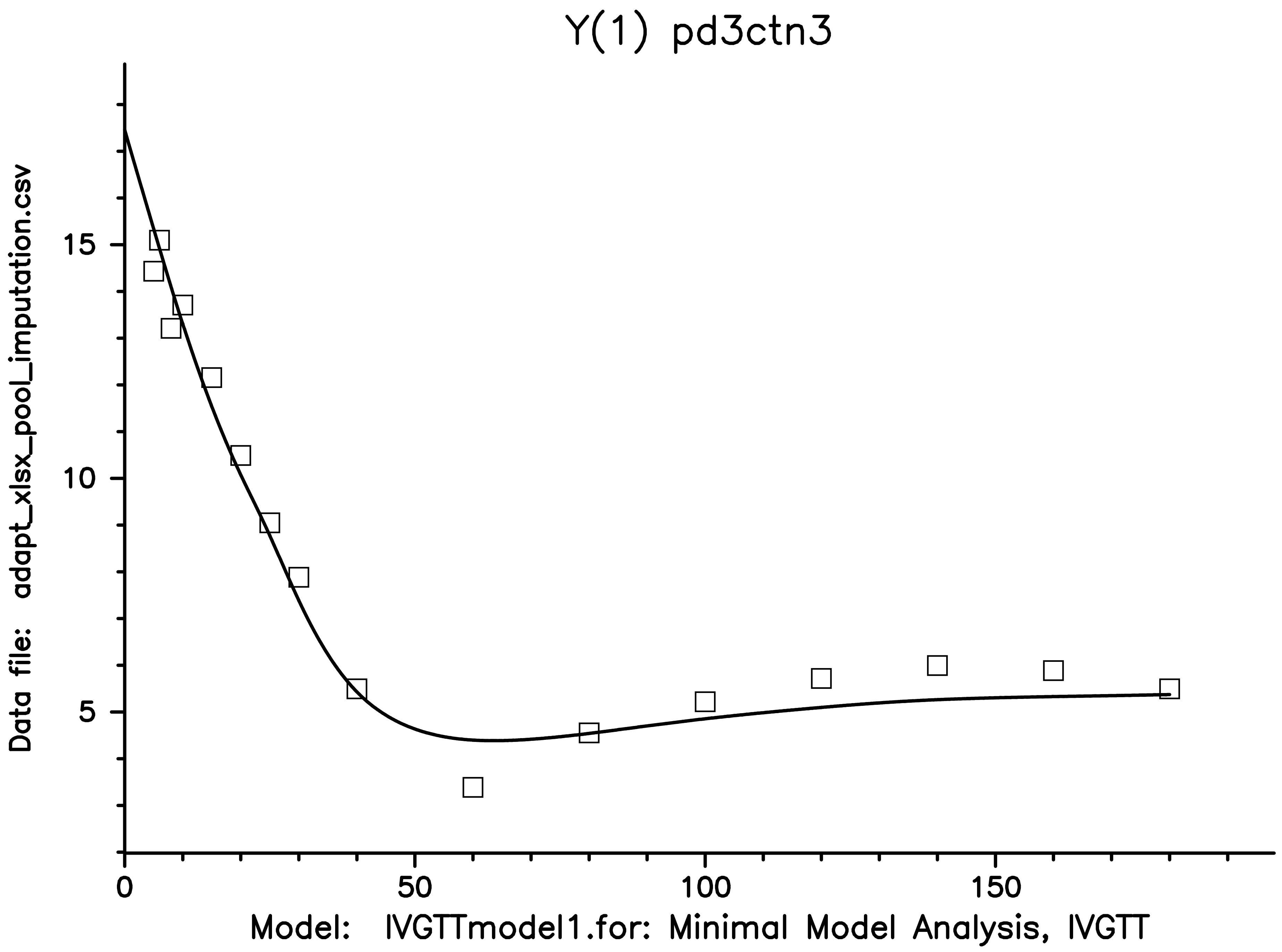
Y(1) pd3ctn3
Data file: adapt_xlsx_pool_imputation.csv
15
10
5
0
50
100
150
Model: IVGTTmodel1.for: Minimal Model Analysis, IVGTT

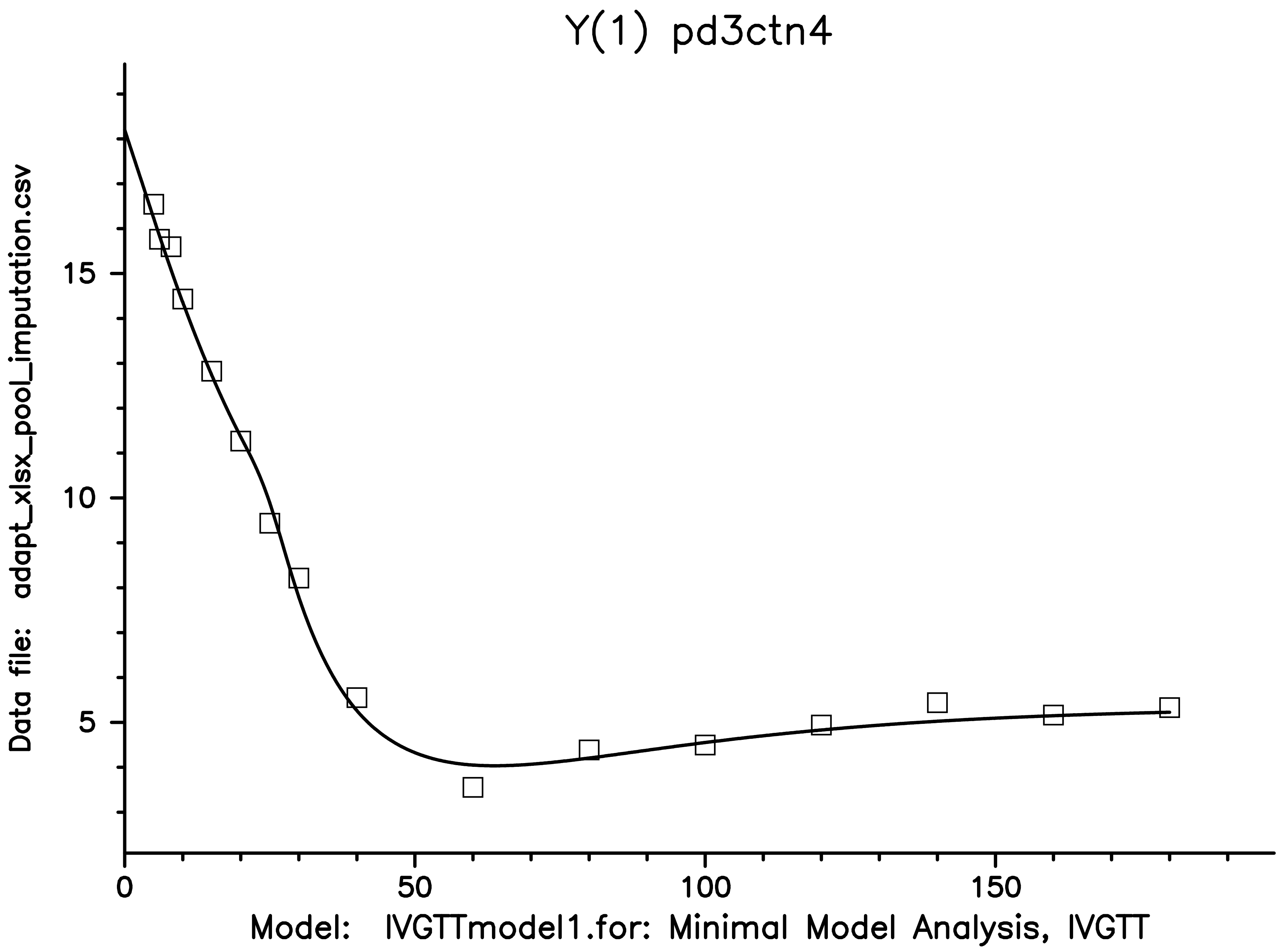
Y(1) pd3ctn4
Data file: adapt_xlsx_pool_imputation.csv
Model: IVGTTmodel1.for: Minimal Model Analysis, IVGTT
15
10
5
0
50
100
150

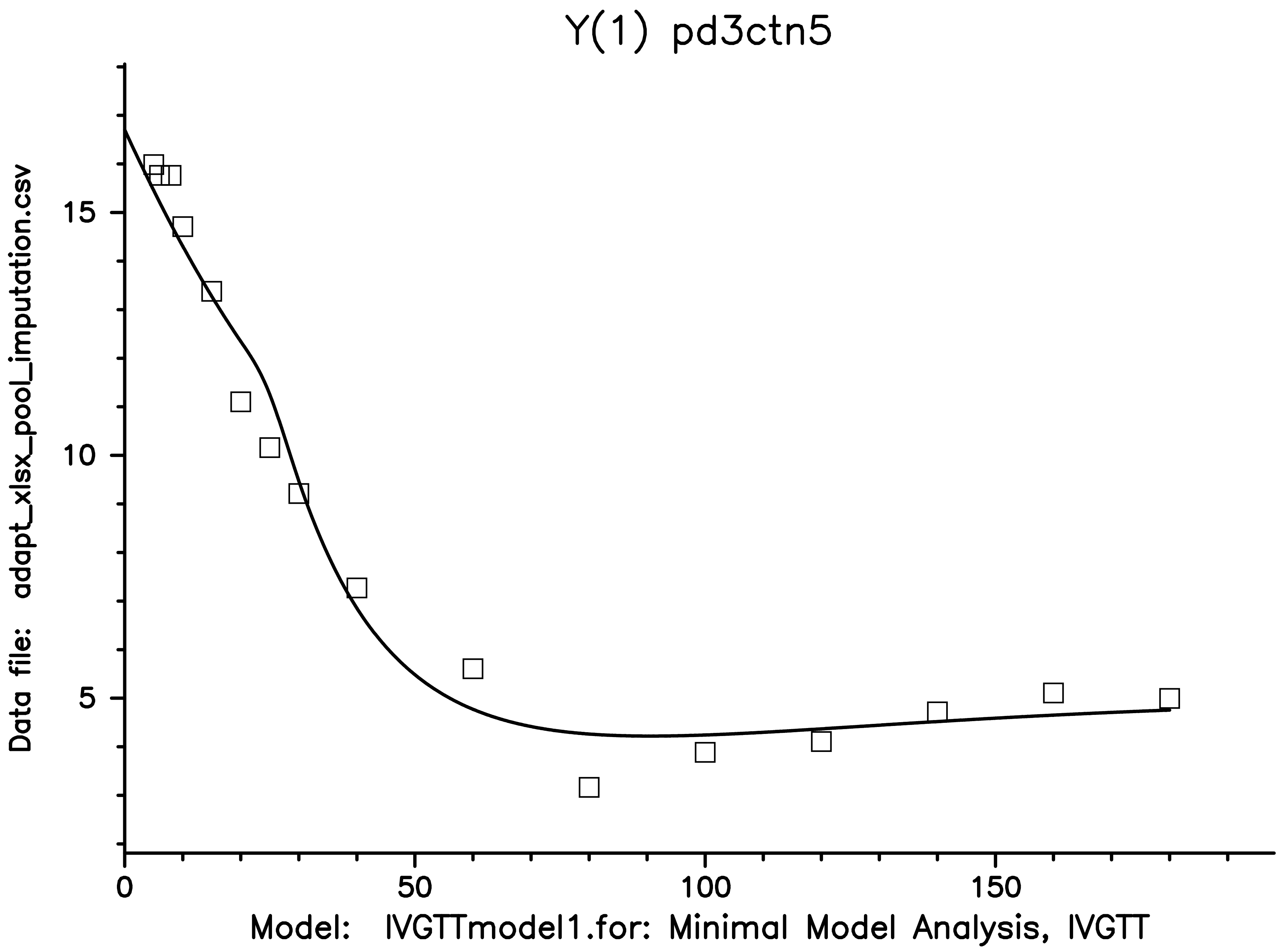
Y(1) pd3ctn5
Data file: adapt_xlsx_pool_imputation.csv
15
10
5
0
50
100
150
Model: IVGTTmodel1.for: Minimal Model Analysis, IVGTT

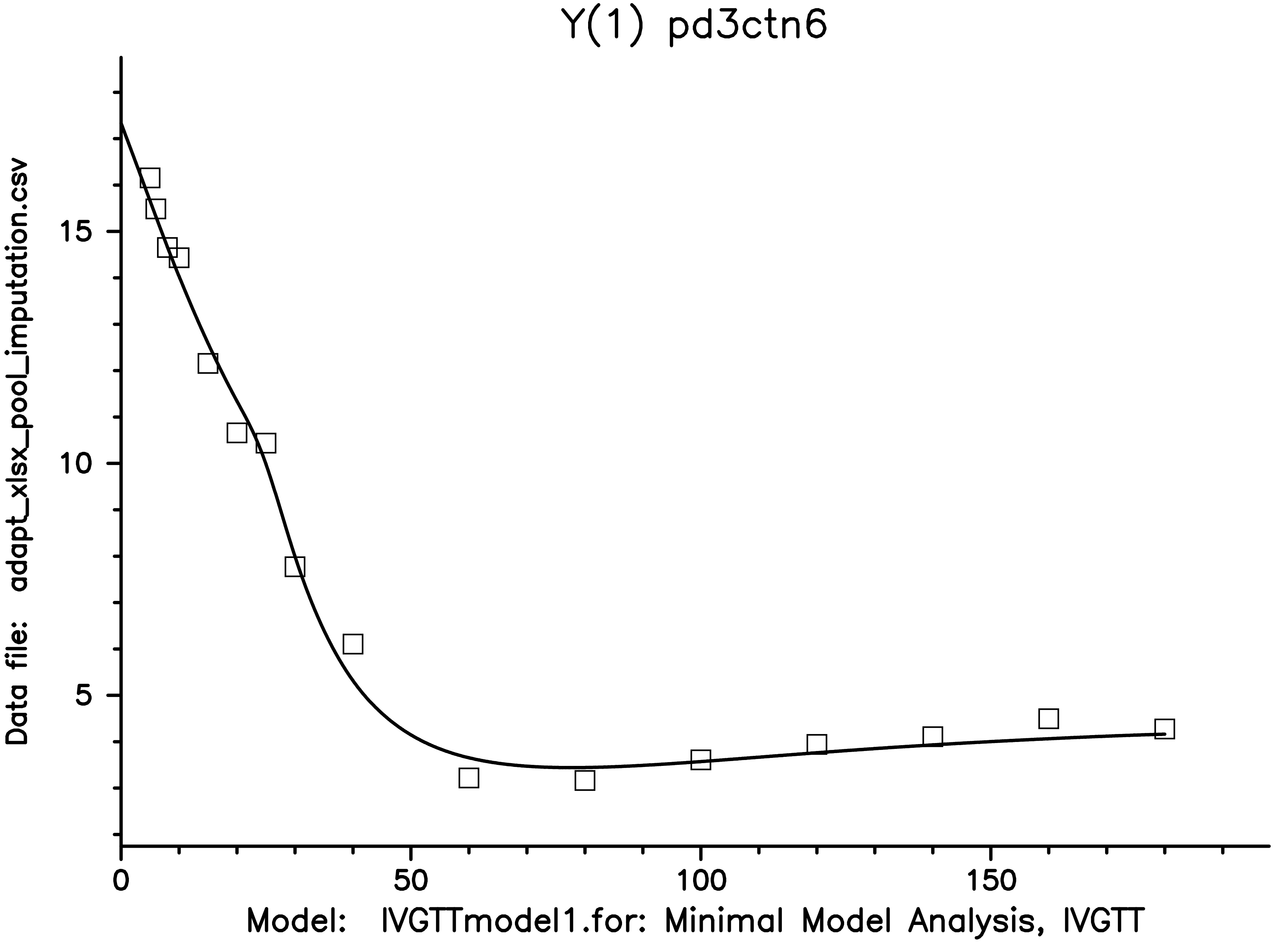
Y(1) pd3ctn6
Data file: adapt_xlsx_pool_imputation.csv
15
10
5
0
50
100
150
Model: IVGTTmodel1.for: Minimal Model Analysis, IVGTT

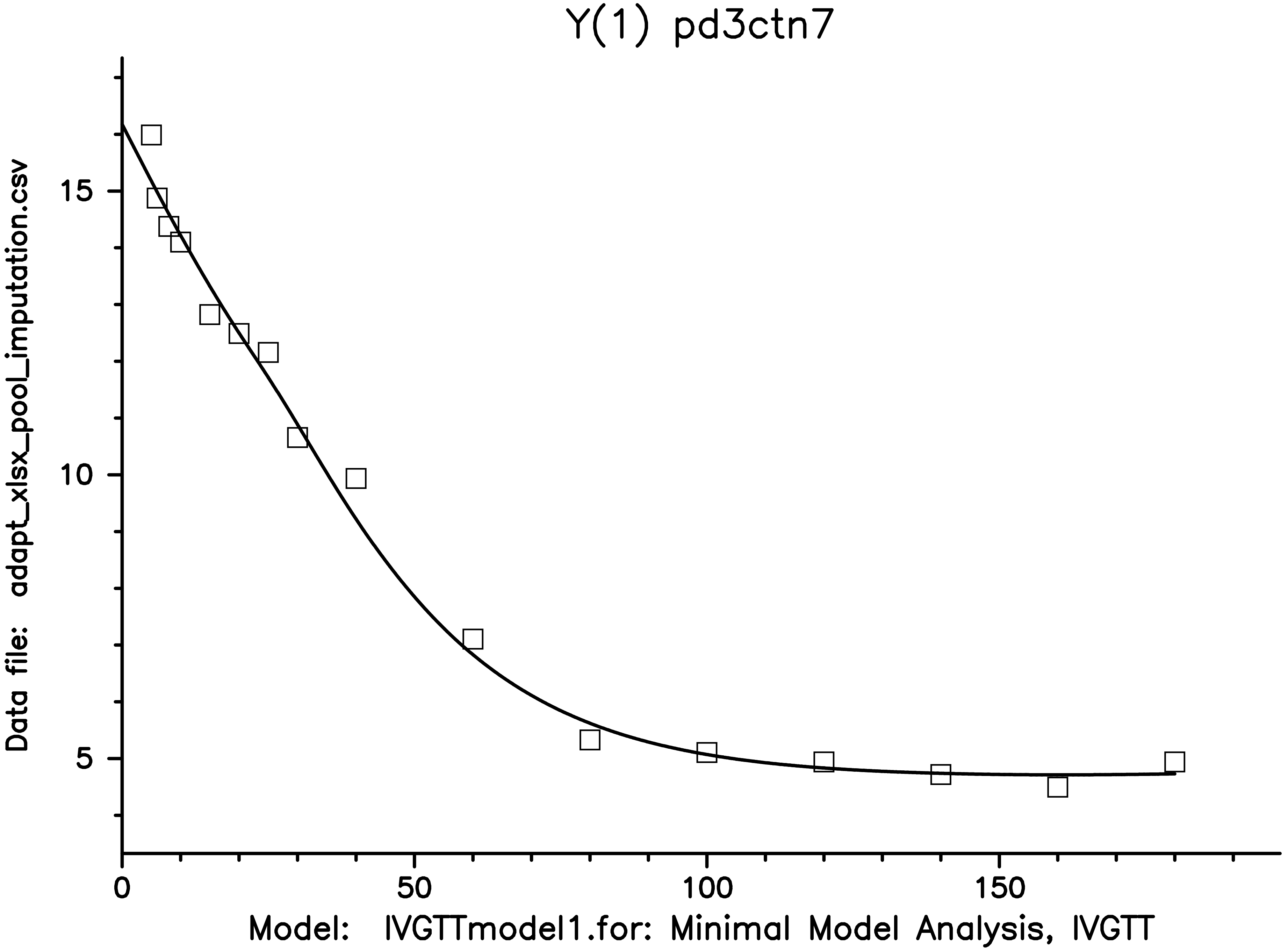
Y(1) pd3ctn7
Data file: adapt_xlsx_pool_imputation.csv
15
10
5
0
50
100
150
Model: IVGTTmodel1.for: Minimal Model Analysis, IVGTT

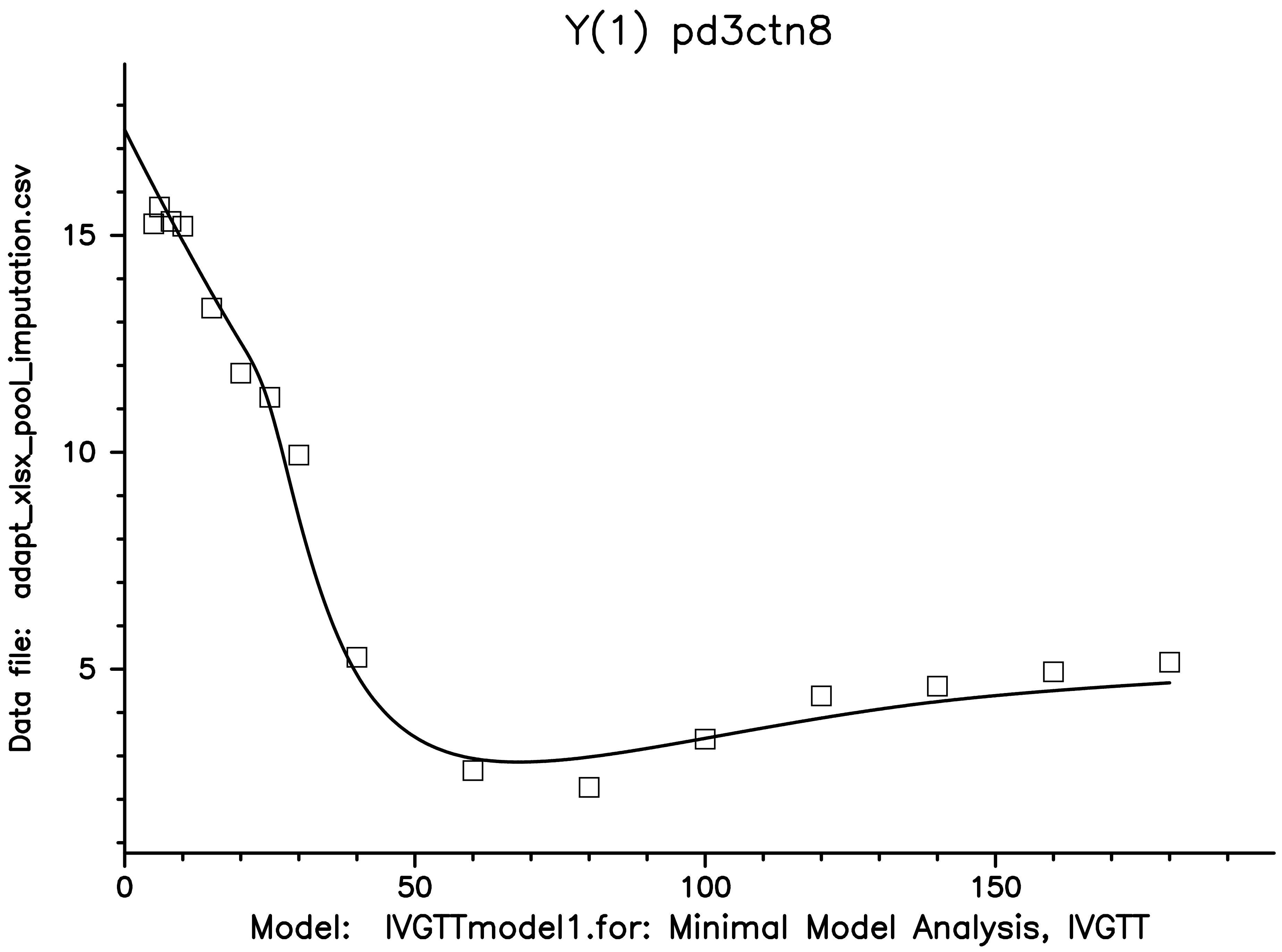

Y(1) pd3ctn8
Data file: adapt_xlsx_pool_imputation.csv
15
10
5
0
50
100
150
Model: IVGTTmodel1.for: Minimal Model Analysis, IVGTT

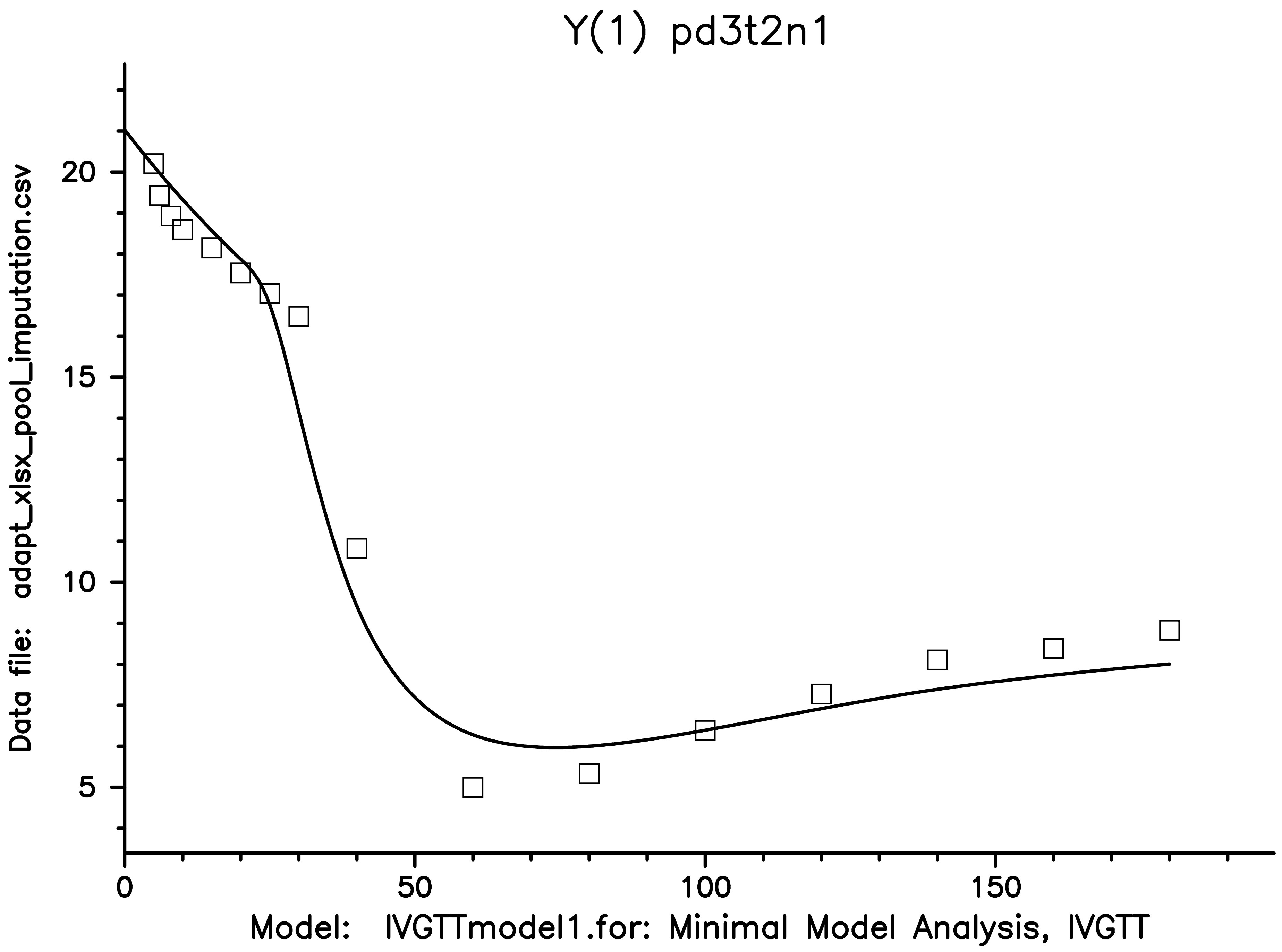
Y(1) pd3t2n1
Data file: adapt_xlsx_pool_imputation.csv
Model: IVGTTmodel1.for: Minimal Model Analysis, IVGTT
20
15
10
5
0
50
100
150

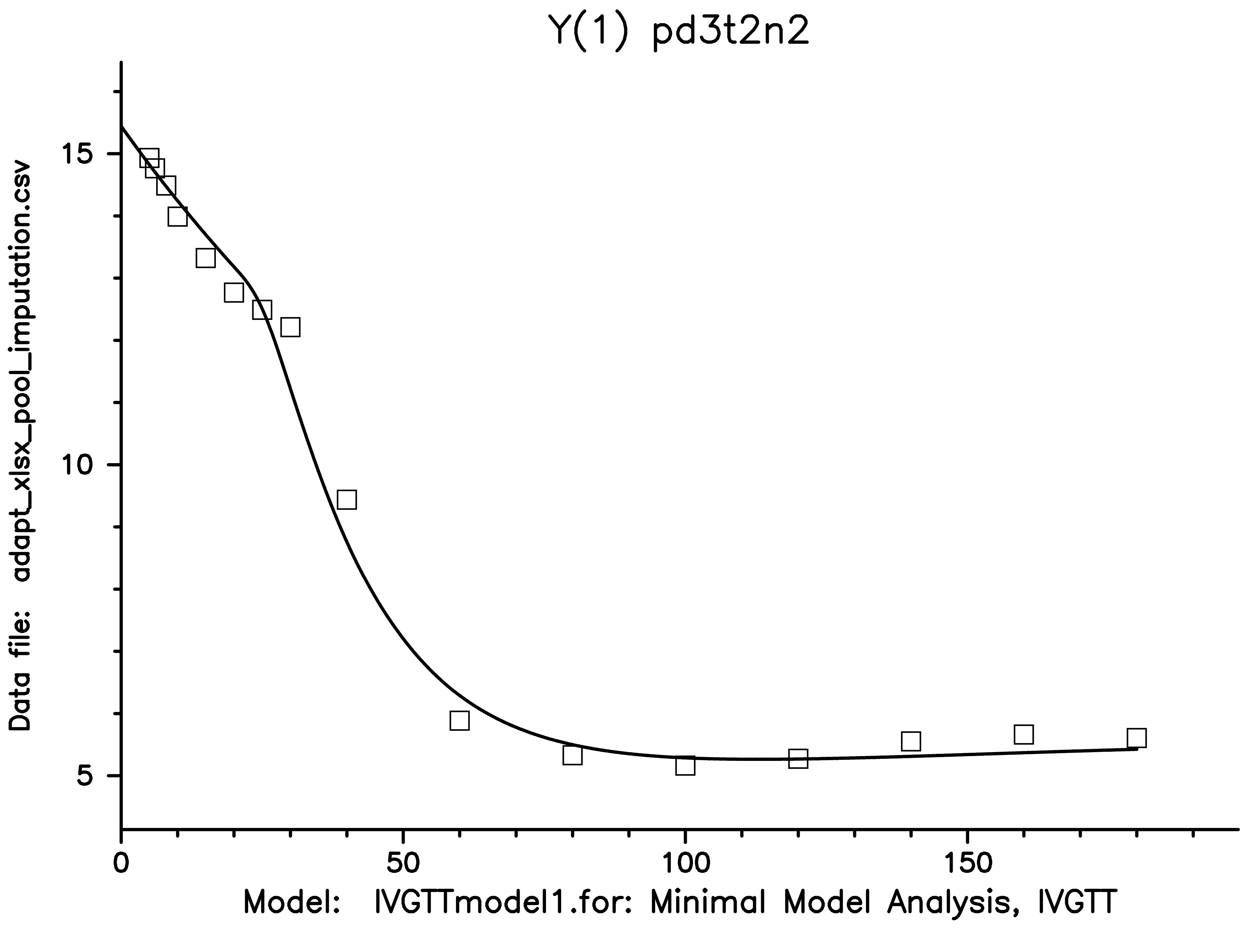
Y(1) pd3t2n2
15
10
5
0
50
100
150
Data file: adapt_xlsx_pool_imputation.csv
Model: IVGTTmodel1.for: Minimal Model Analysis, IVGTT

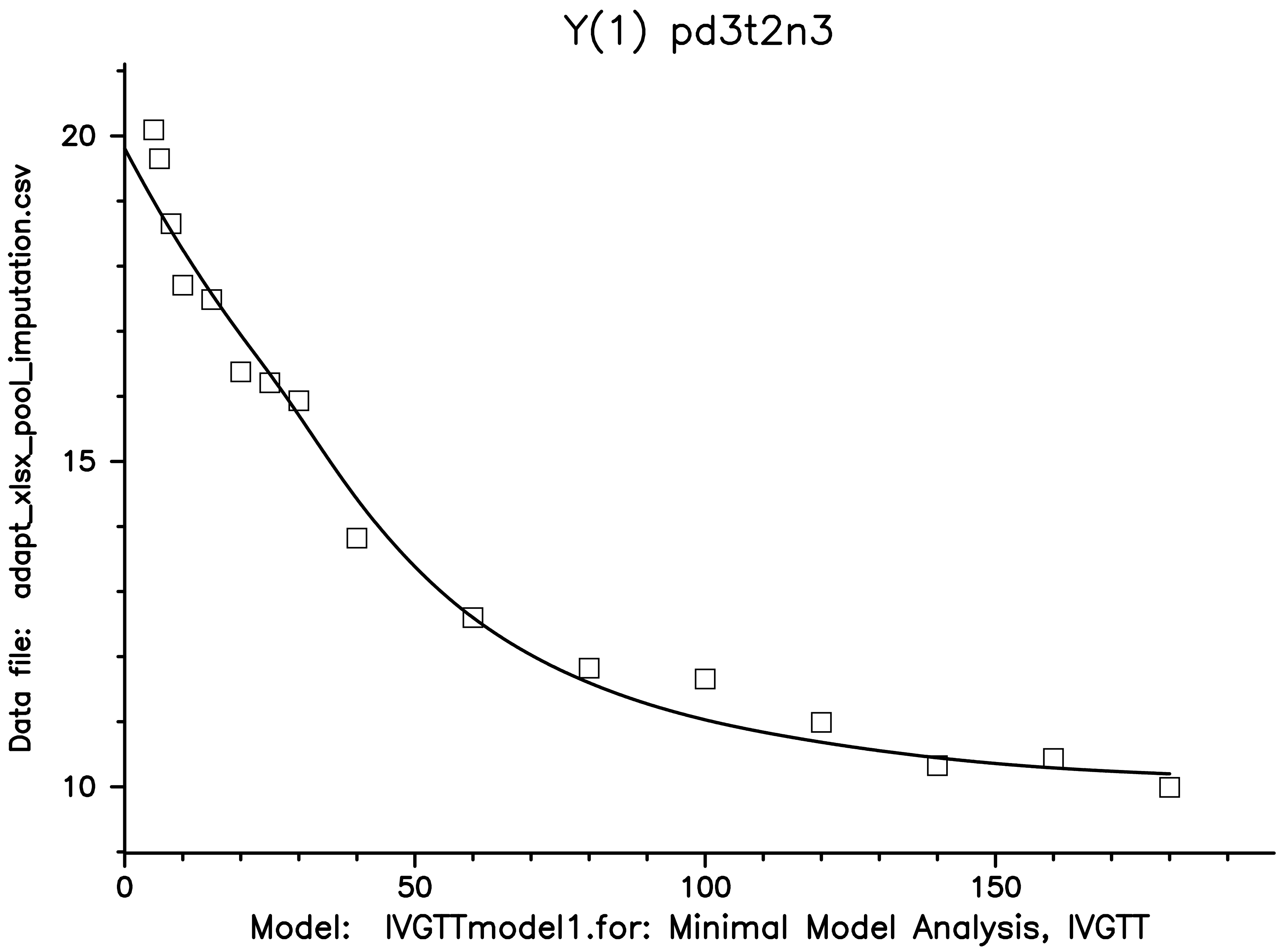

Y(1) pd3t2n3
20
15
10
0
50
100
150
Data file: adapt_xlsx_pool_imputation.csv
Model: IVGTTmodel1.for: Minimal Model Analysis, IVGTT

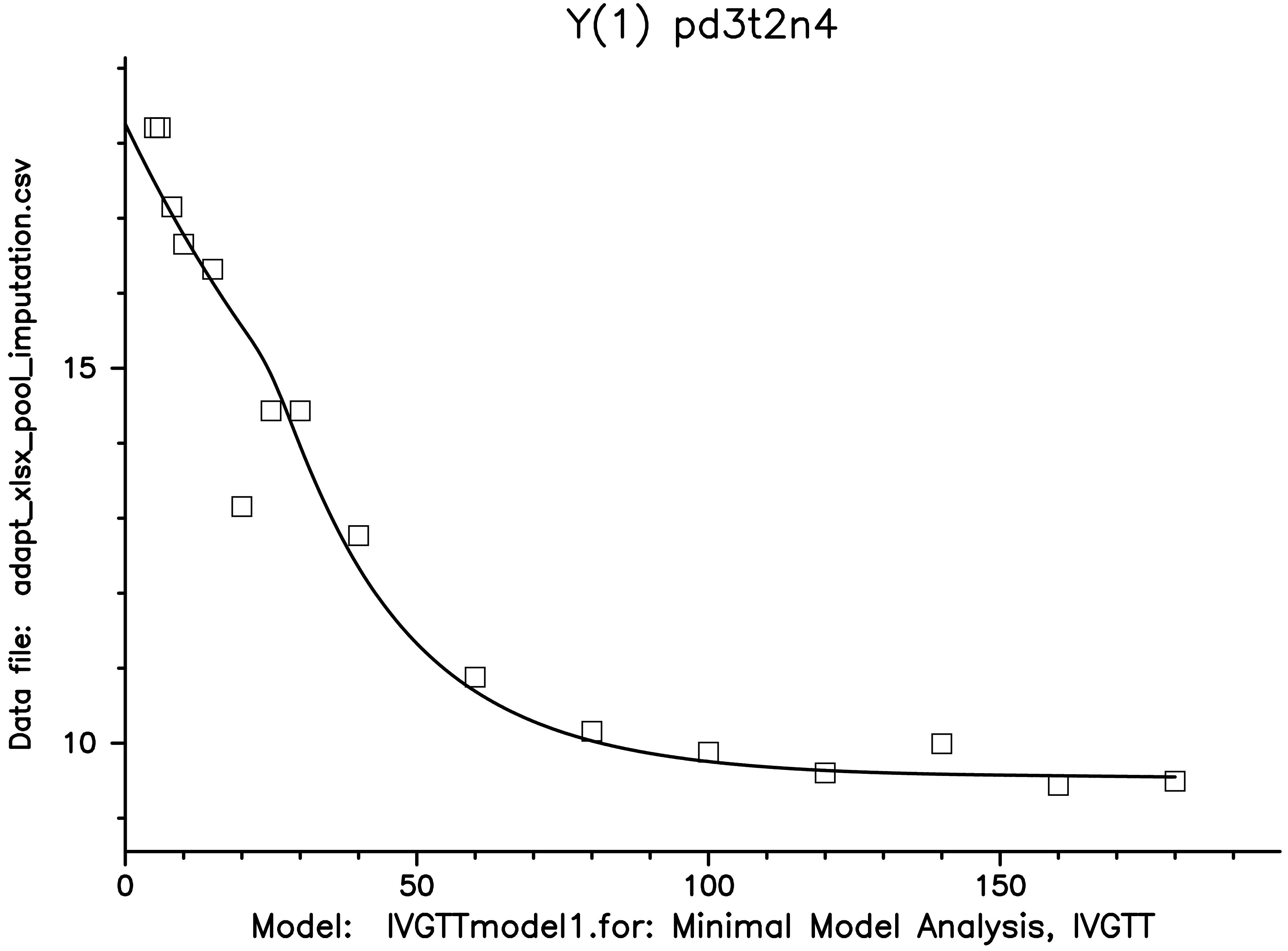
Y(1) pd3t2n4
Data file: adapt_xlsx_pool_imputation.csv
Model: IVGTTmodel1.for: Minimal Model Analysis, IVGTT
15
10
0
50
100
150

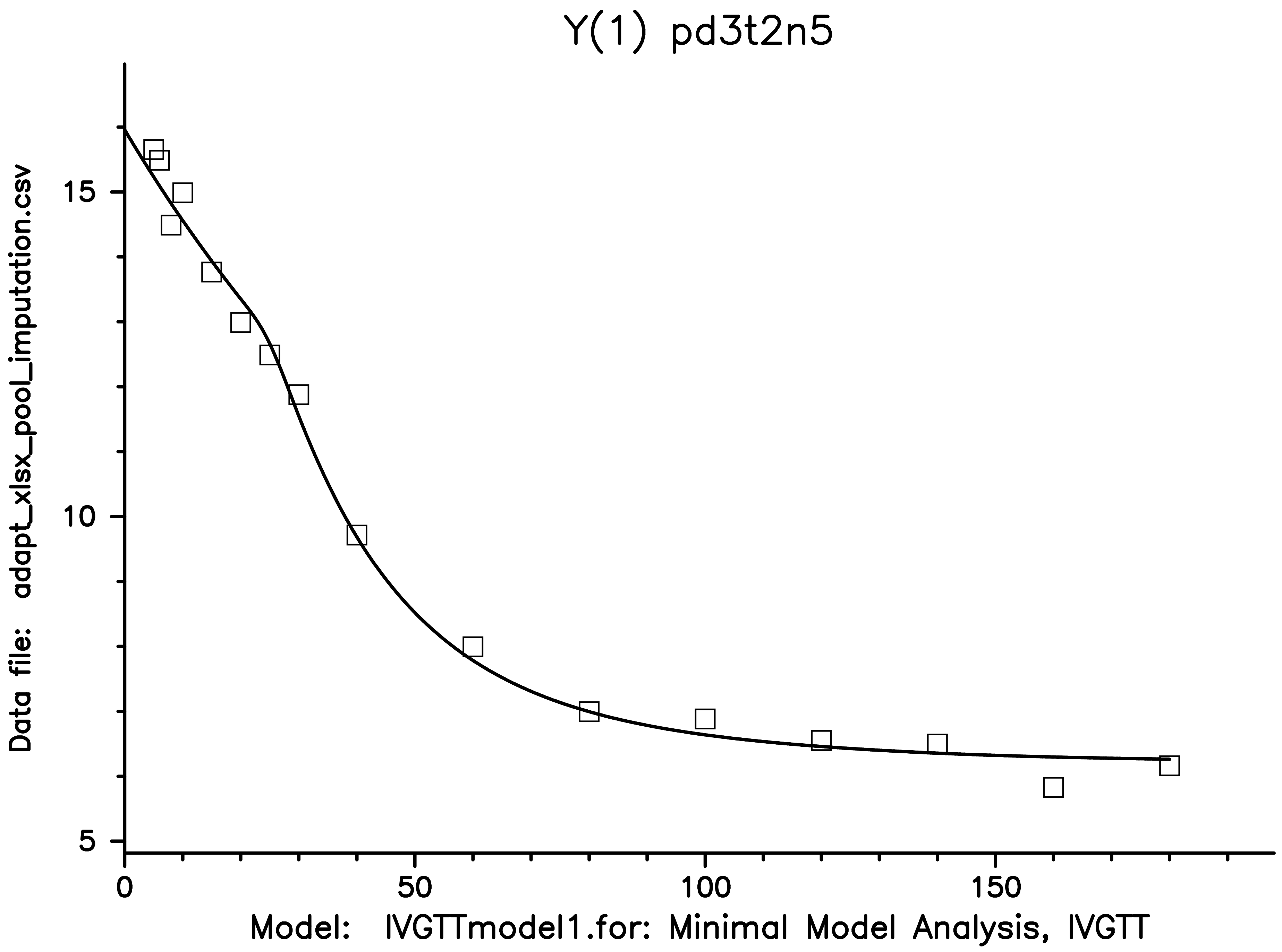

Y(1) pd3t2n5
Data file: adapt_xlsx_pool_imputation.csv
15
10
5
0
50
100
150
Model: IVGTTmodel1.for: Minimal Model Analysis, IVGTT

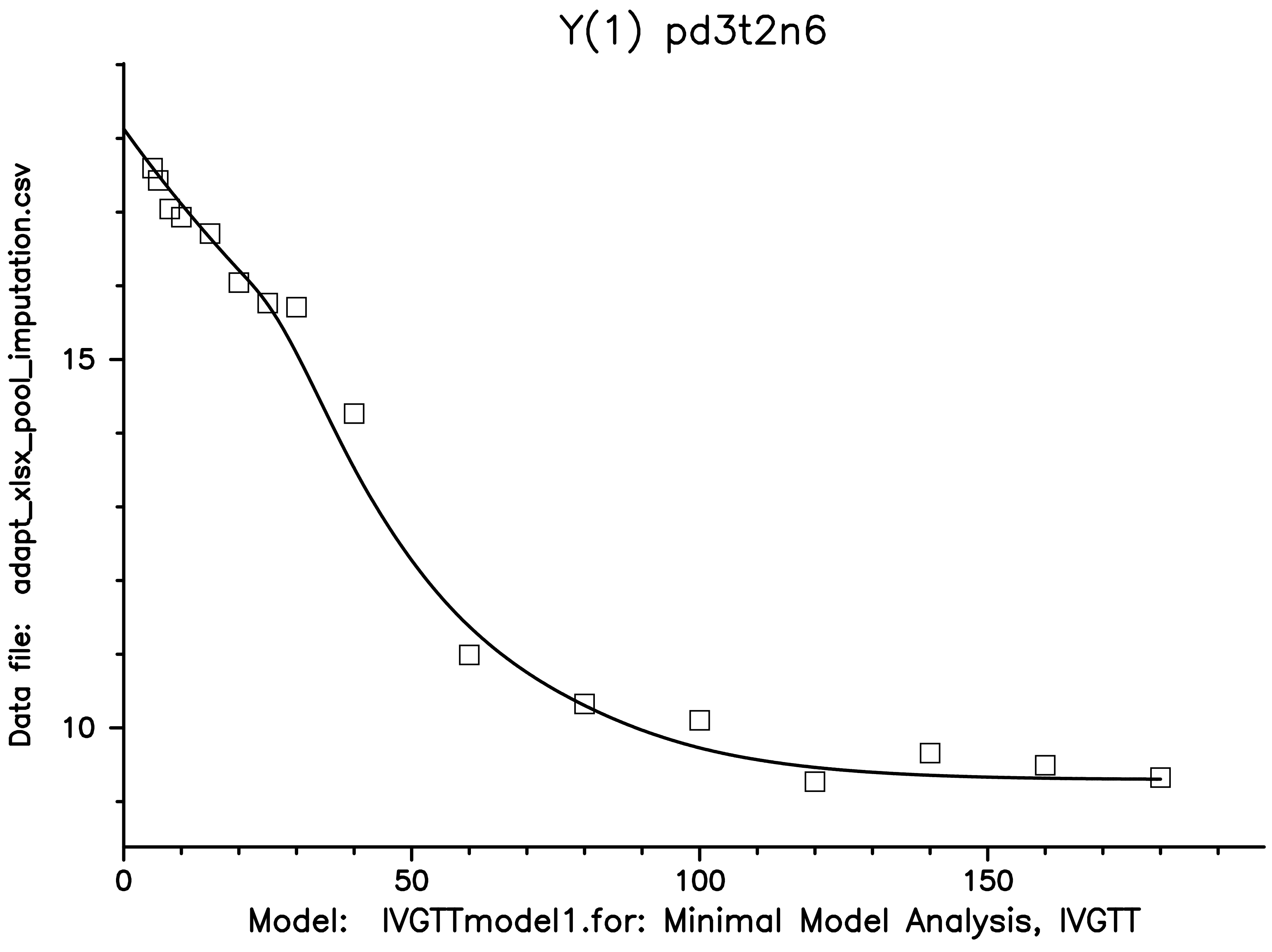
Y(1) pd3t2n6
Data file: adapt_xlsx_pool_imputation.csv
15
10
0
50
100
150
Model: IVGTTmodel1.for: Minimal Model Analysis, IVGTT

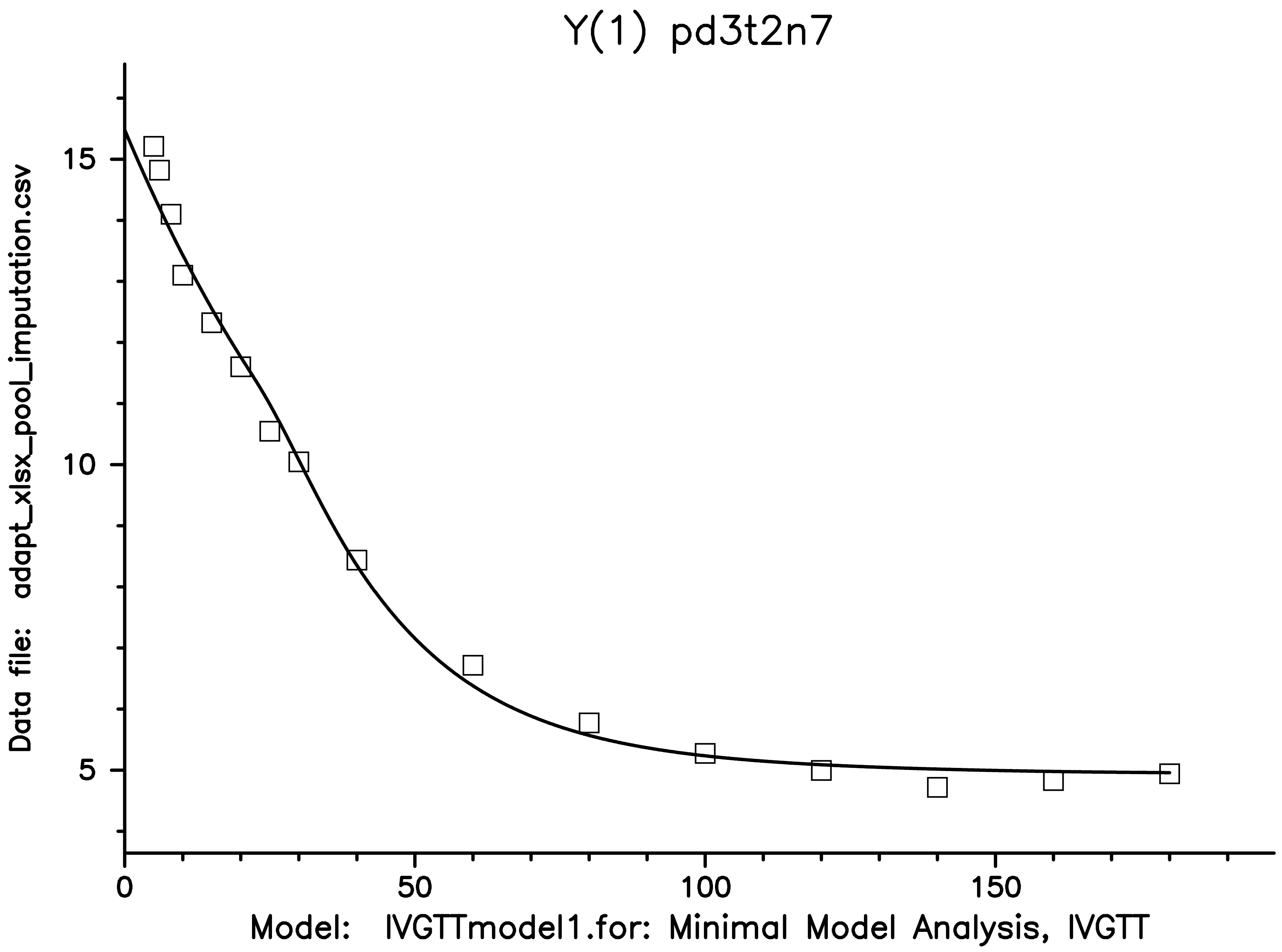
Y(1) pd3t2n7
Data file: adapt_xlsx_pool_imputation.csv
15
10
5
0
50
100
150
Model: IVGTTmodel1.for: Minimal Model Analysis, IVGTT

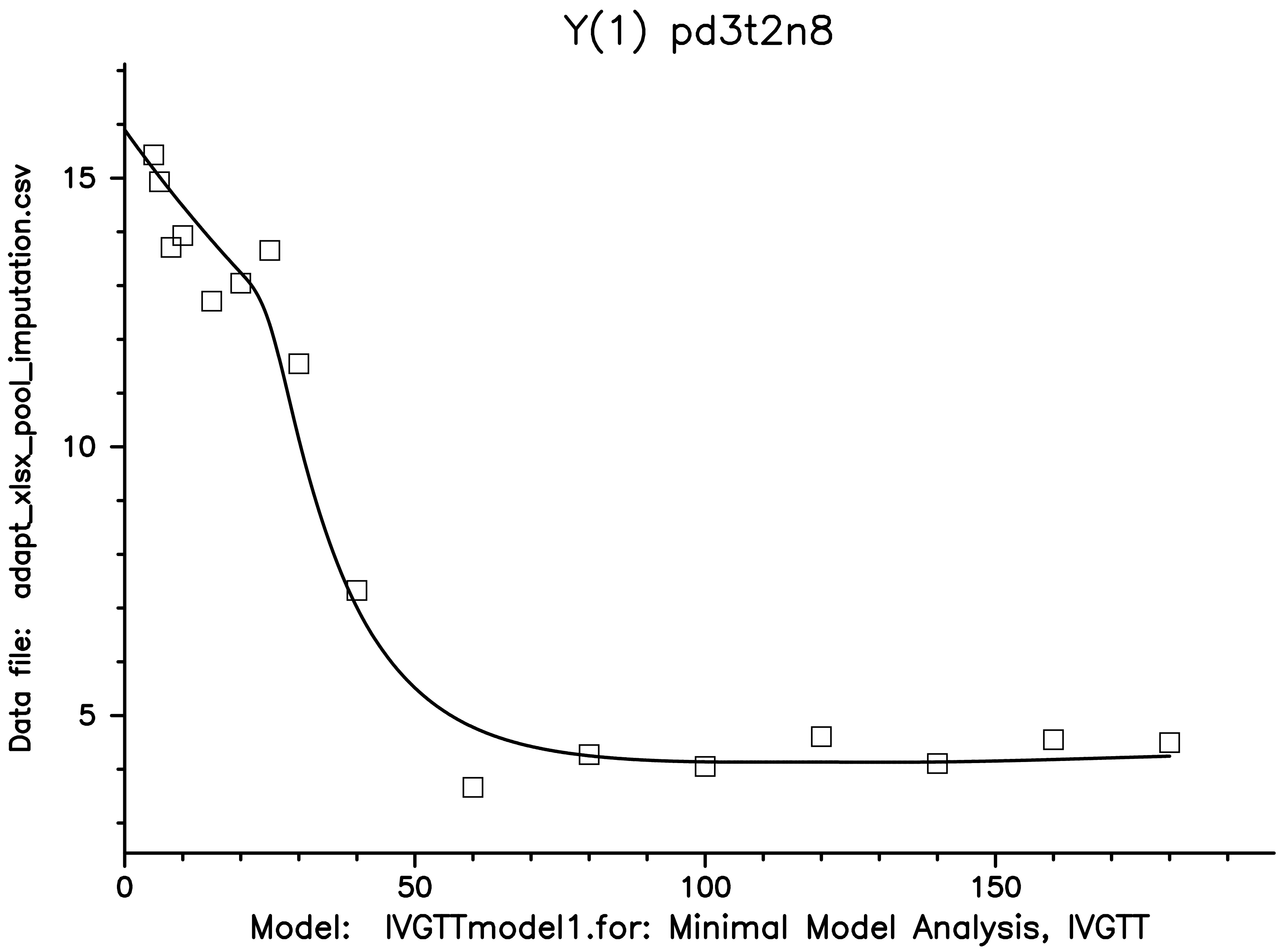

Y(1) pd3t2n8
Data file: adapt_xlsx_pool_imputation.csv
15
10
5
0
50
100
150
Model: IVGTTmodel1.for: Minimal Model Analysis, IVGTT

Y(1) cap0306

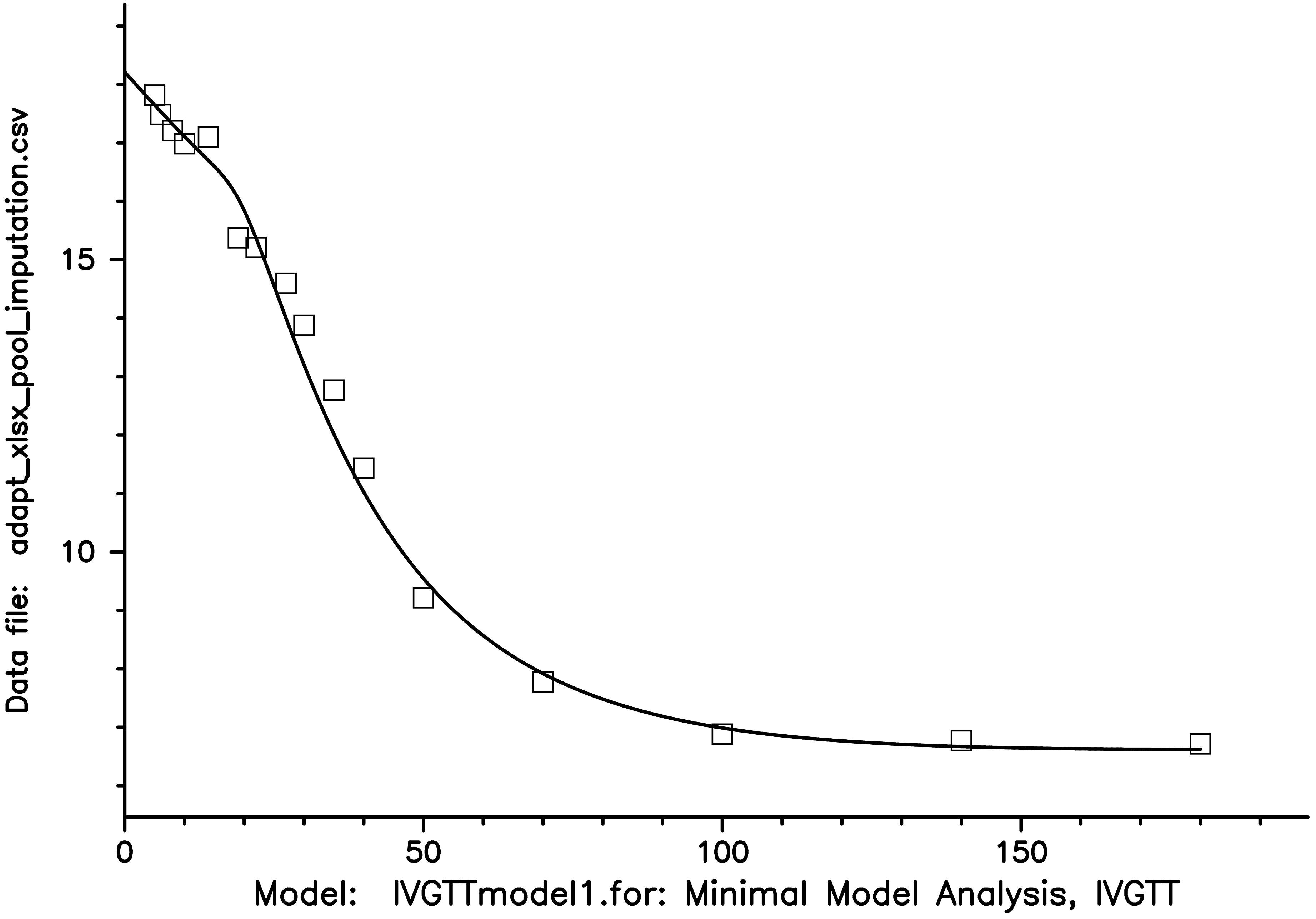

Data file: adapt_xlsx_pool_imputation.csv
15
10
0
50
100
150
Model: IVGTTmodel1.for: Minimal Model Analysis, IVGTT

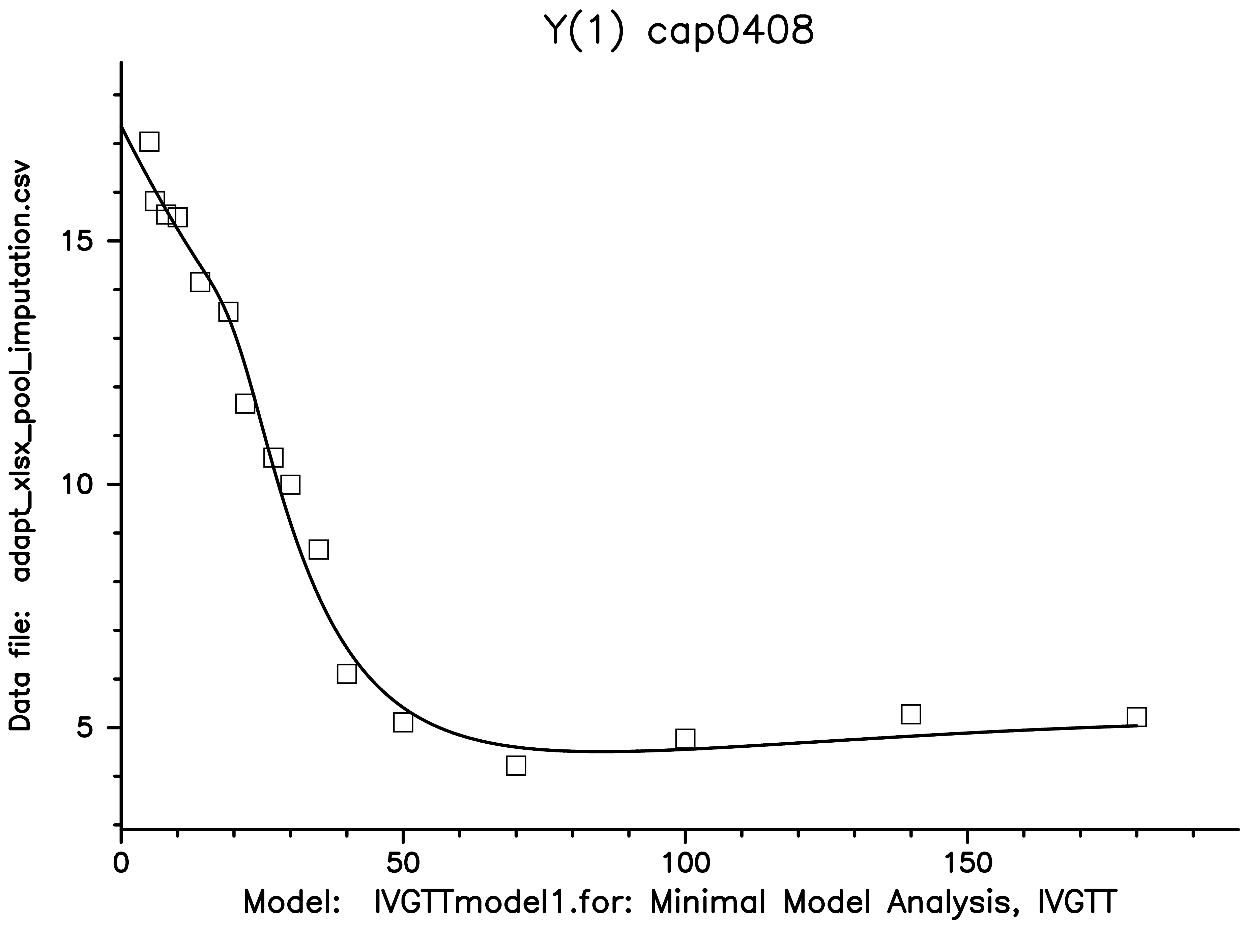

Y(1) cap0408
Data file: adapt_xlsx_pool_imputation.csv
15
10
5
0
50
100
150
Model: IVGTTmodel1.for: Minimal Model Analysis, IVGTT

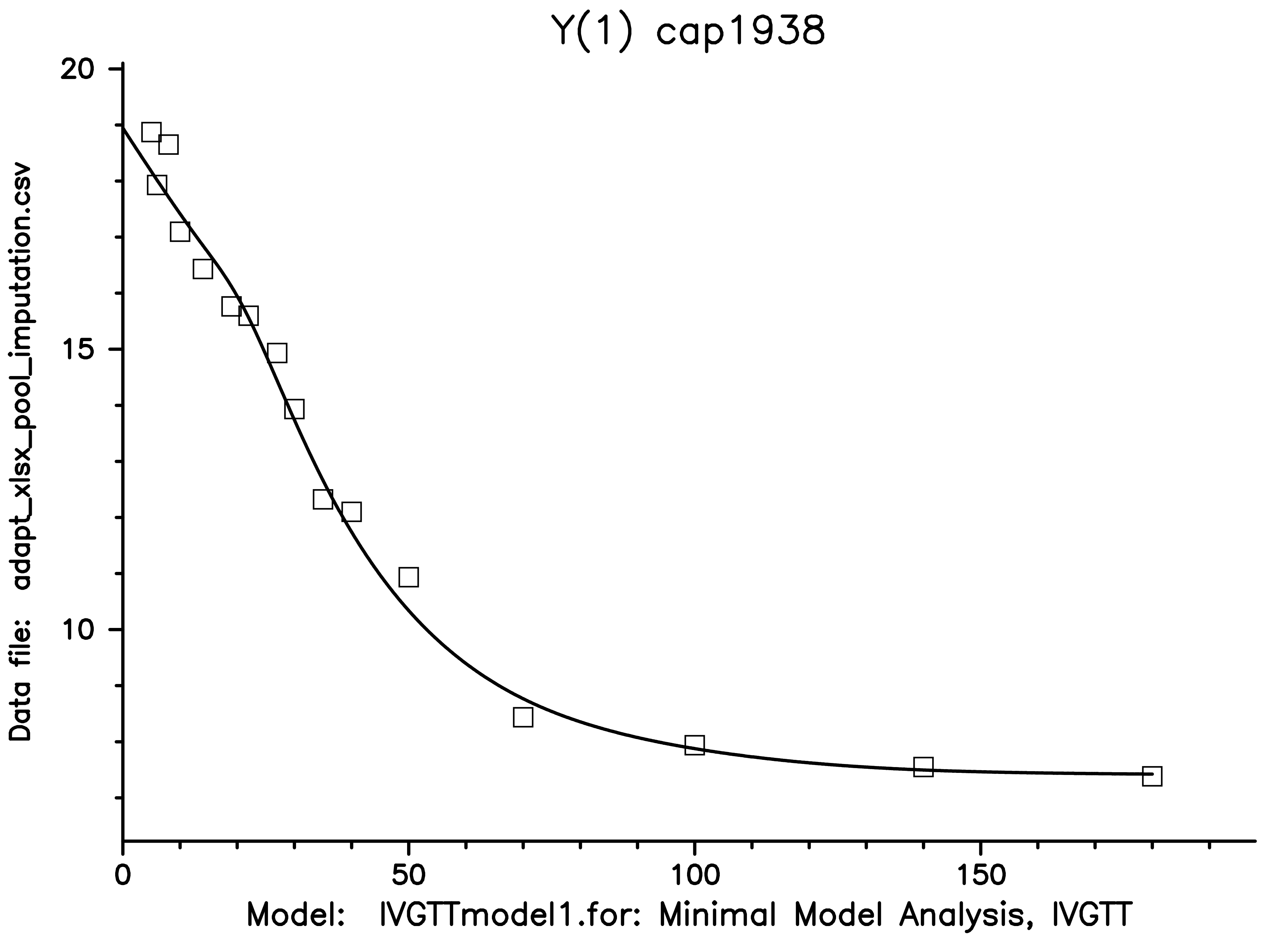
Y(1) cap1938
20
15
10
0
50
100
150
Data file: adapt_xlsx_pool_imputation.csv
Model: IVGTTmodel1.for: Minimal Model Analysis, IVGTT

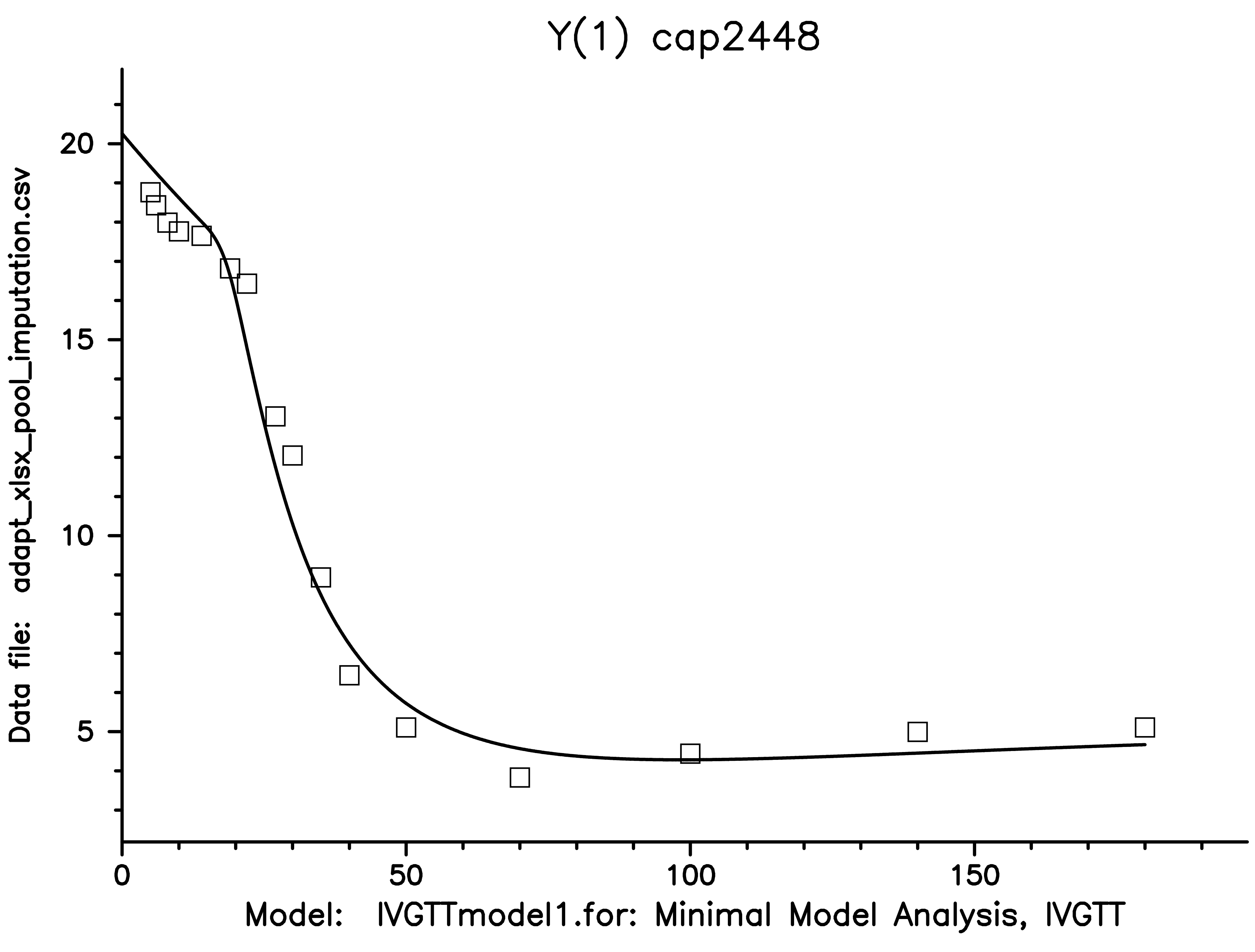

Y(1) cap2448
Data file: adapt_xlsx_pool_imputation.csv
20
15
10
5
0
50
100
150
Model: IVGTTmodel1.for: Minimal Model Analysis, IVGTT

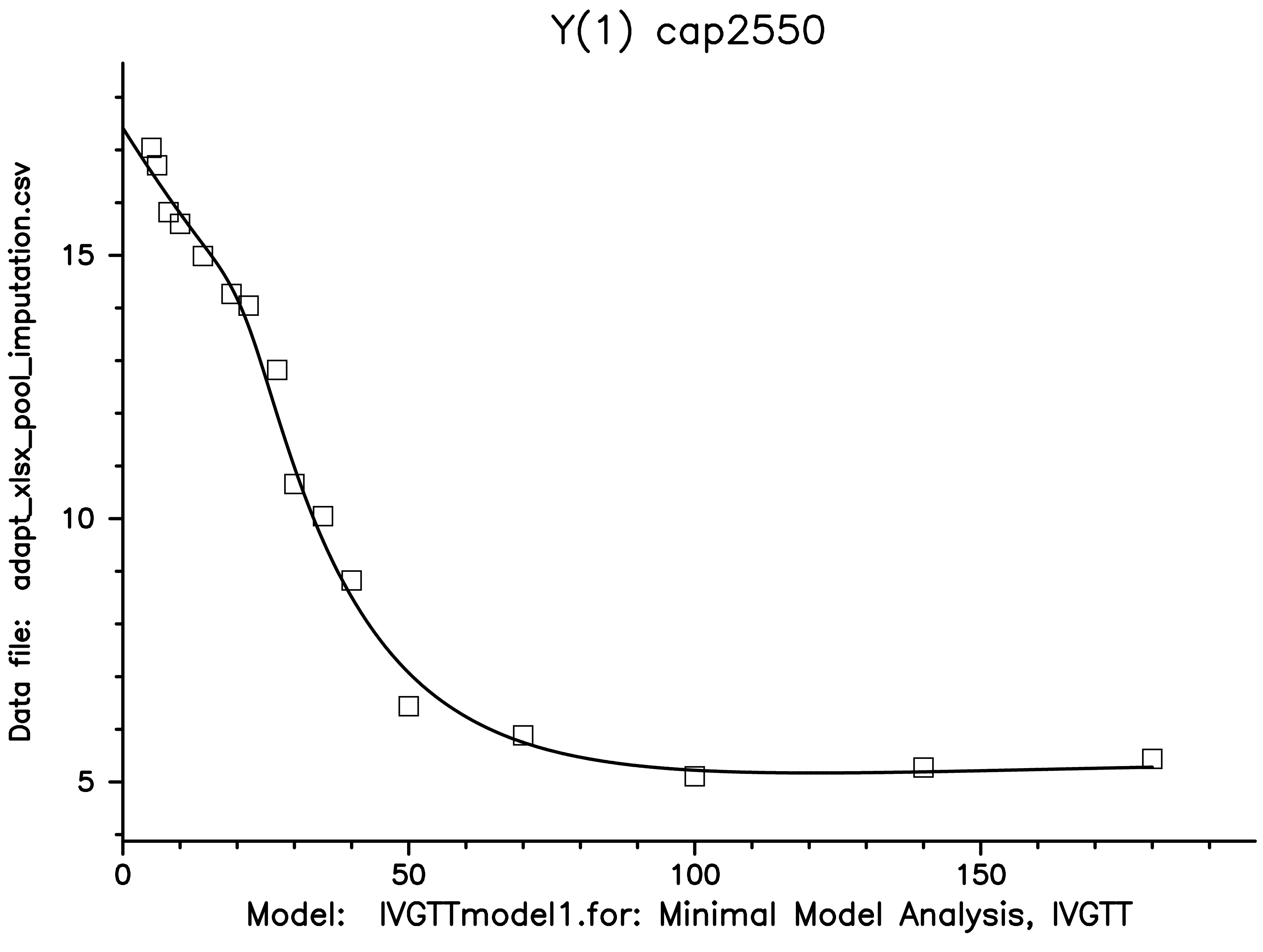
Y(1) cap2550
Data file: adapt_xlsx_pool_imputation.csv
15
10
5
0
50
100
150
Model: IVGTTmodel1.for: Minimal Model Analysis, IVGTT

Y(1) cap2958

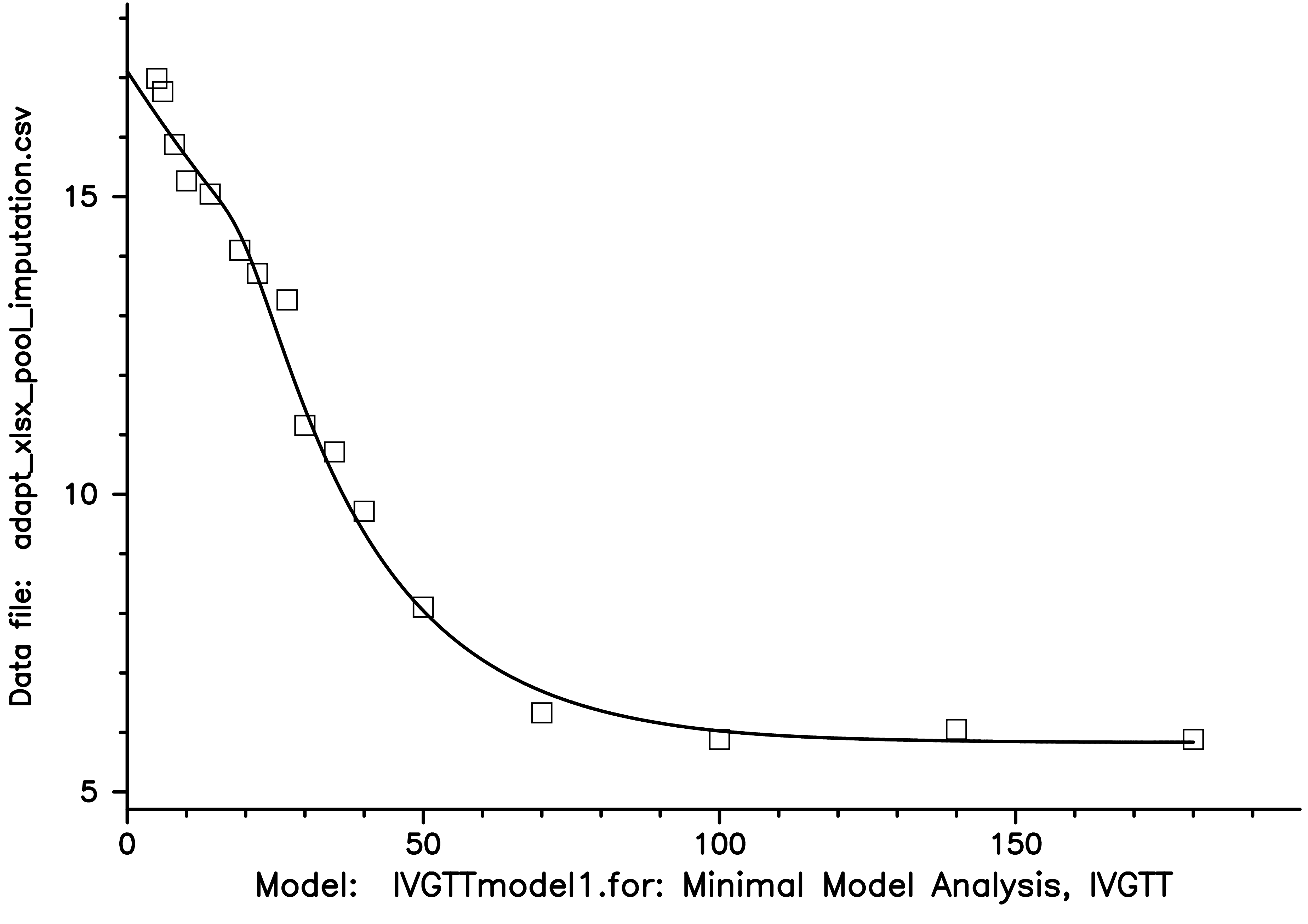
Data file: adapt_xlsx_pool_imputation.csv
15
10
5
0
50
100
150
Model: IVGTTmodel1.for: Minimal Model Analysis, IVGTT

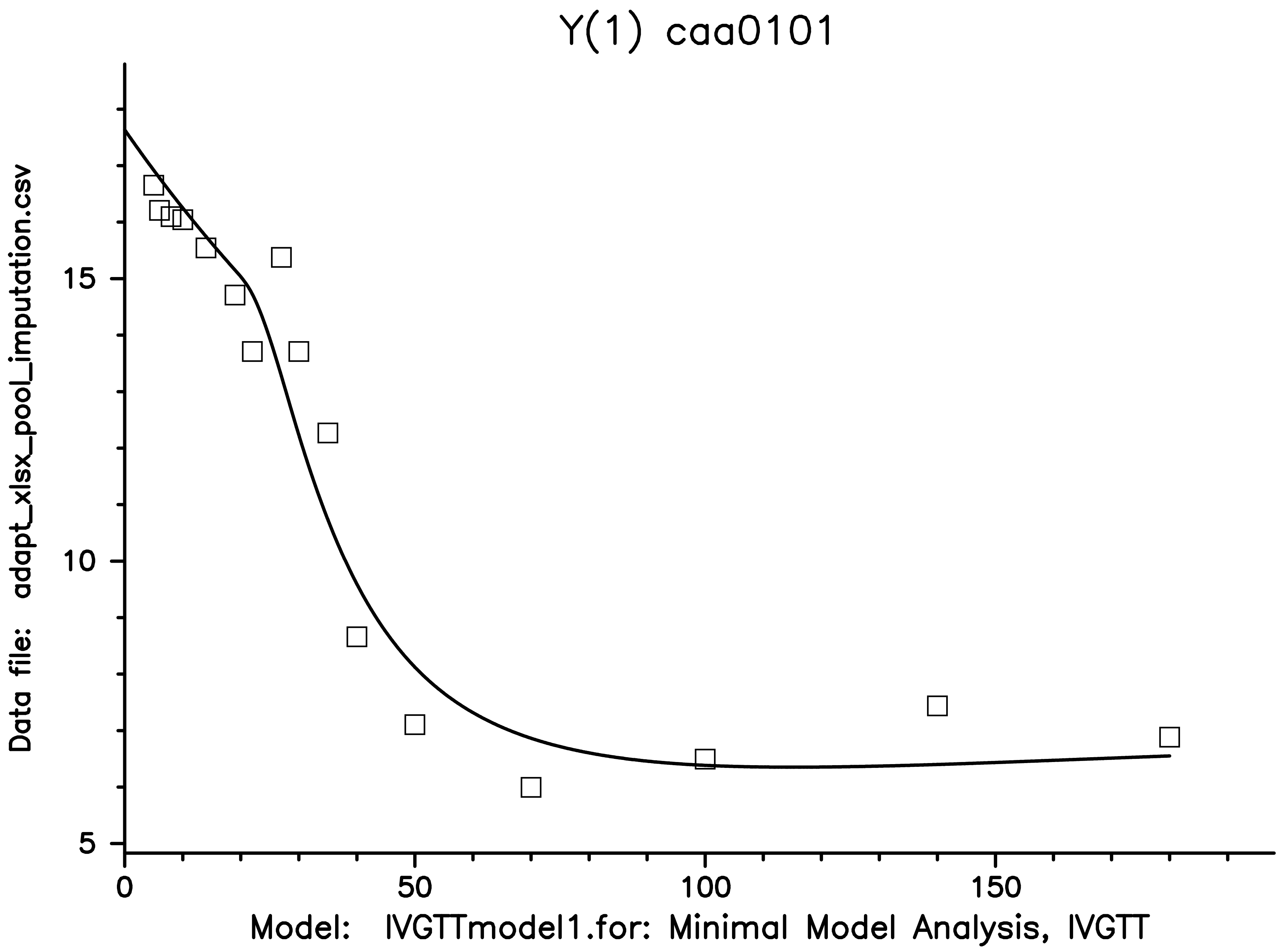
Y(1) caa0101
Data file: adapt_xlsx_pool_imputation.csv
15
10
5
0
50
100
150
Model: IVGTTmodel1.for: Minimal Model Analysis, IVGTT

Y(1) caa0203

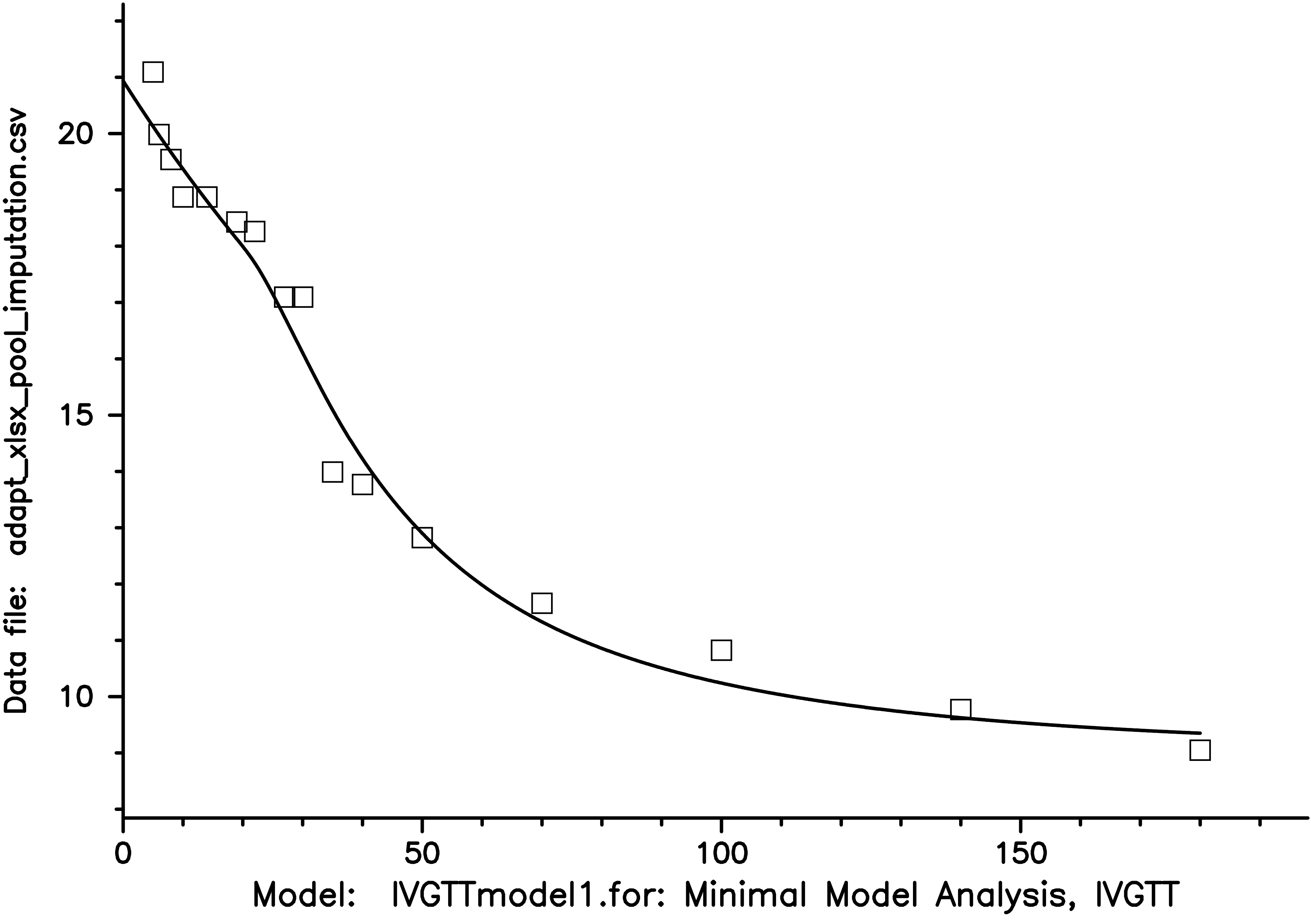

Data file: adapt_xlsx_pool_imputation.csv
20
15
10
0
50
100
150
Model: IVGTTmodel1.for: Minimal Model Analysis, IVGTT

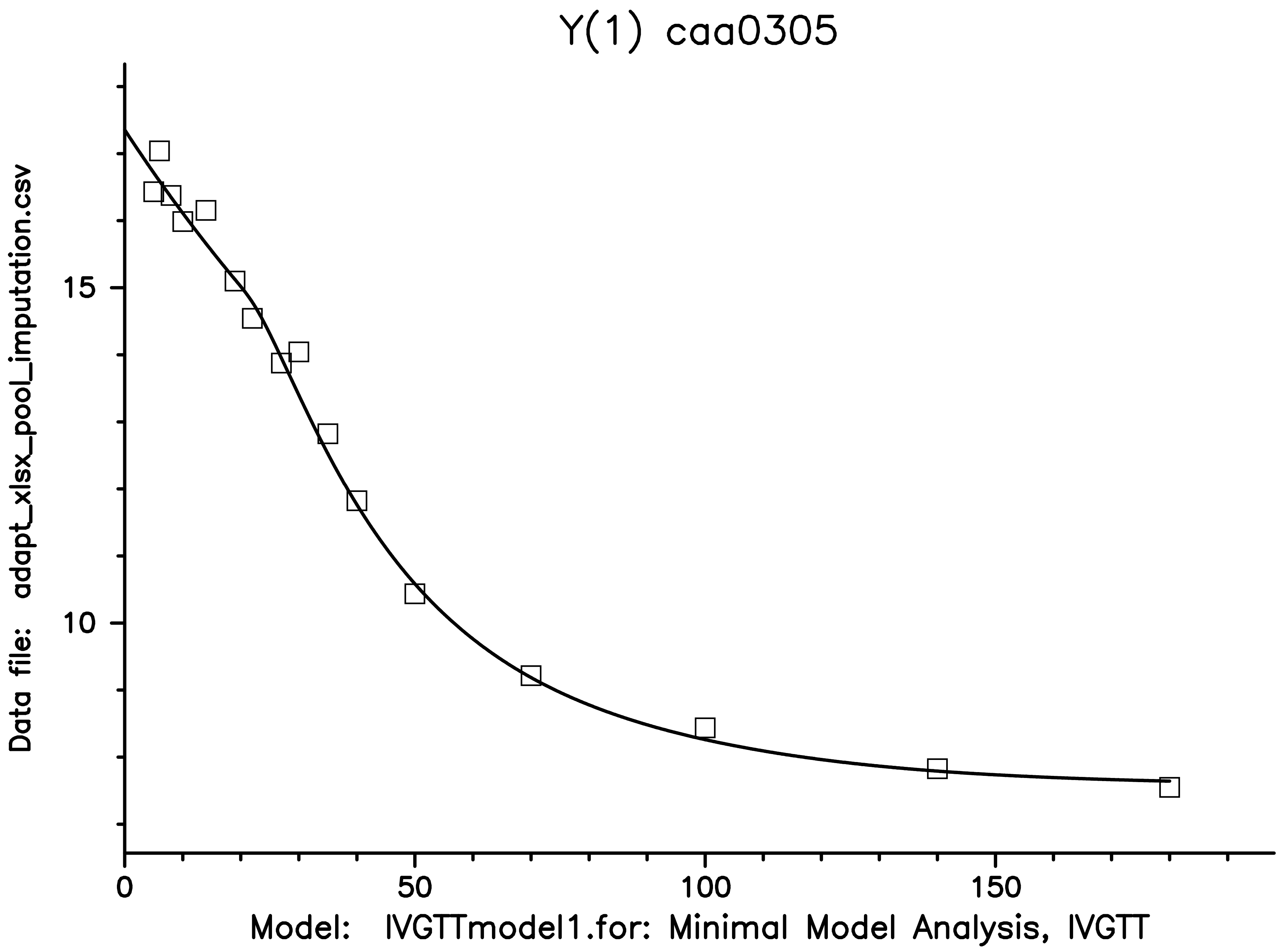
Y(1) caa0305
Data file: adapt_xlsx_pool_imputation.csv
15
10
0
50
100
150
Model: IVGTTmodel1.for: Minimal Model Analysis, IVGTT

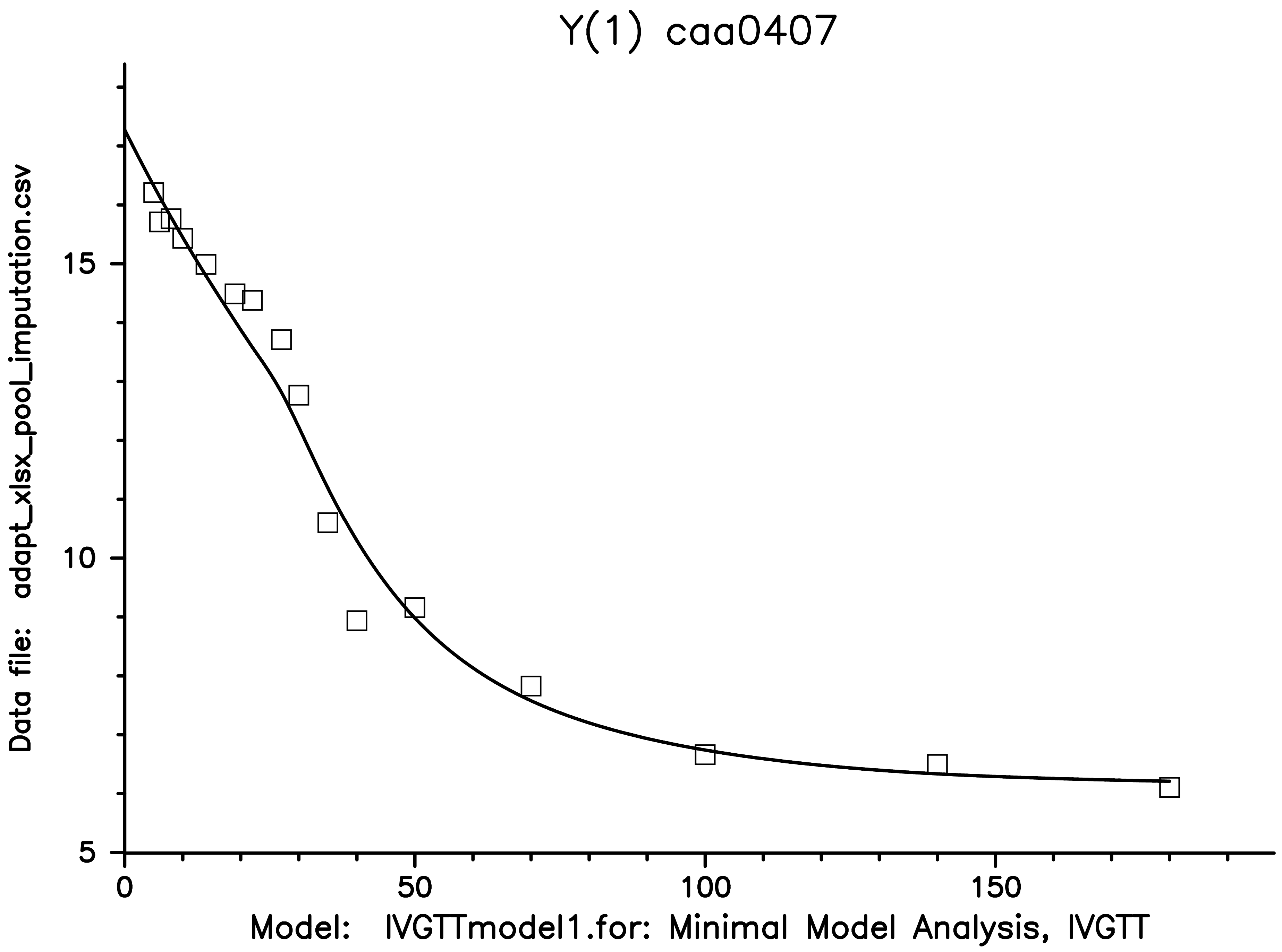
Y(1) caa0407
Data file: adapt_xlsx_pool_imputation.csv
15
10
5
0
50
100
150
Model: IVGTTmodel1.for: Minimal Model Analysis, IVGTT

Y(1) caa0509

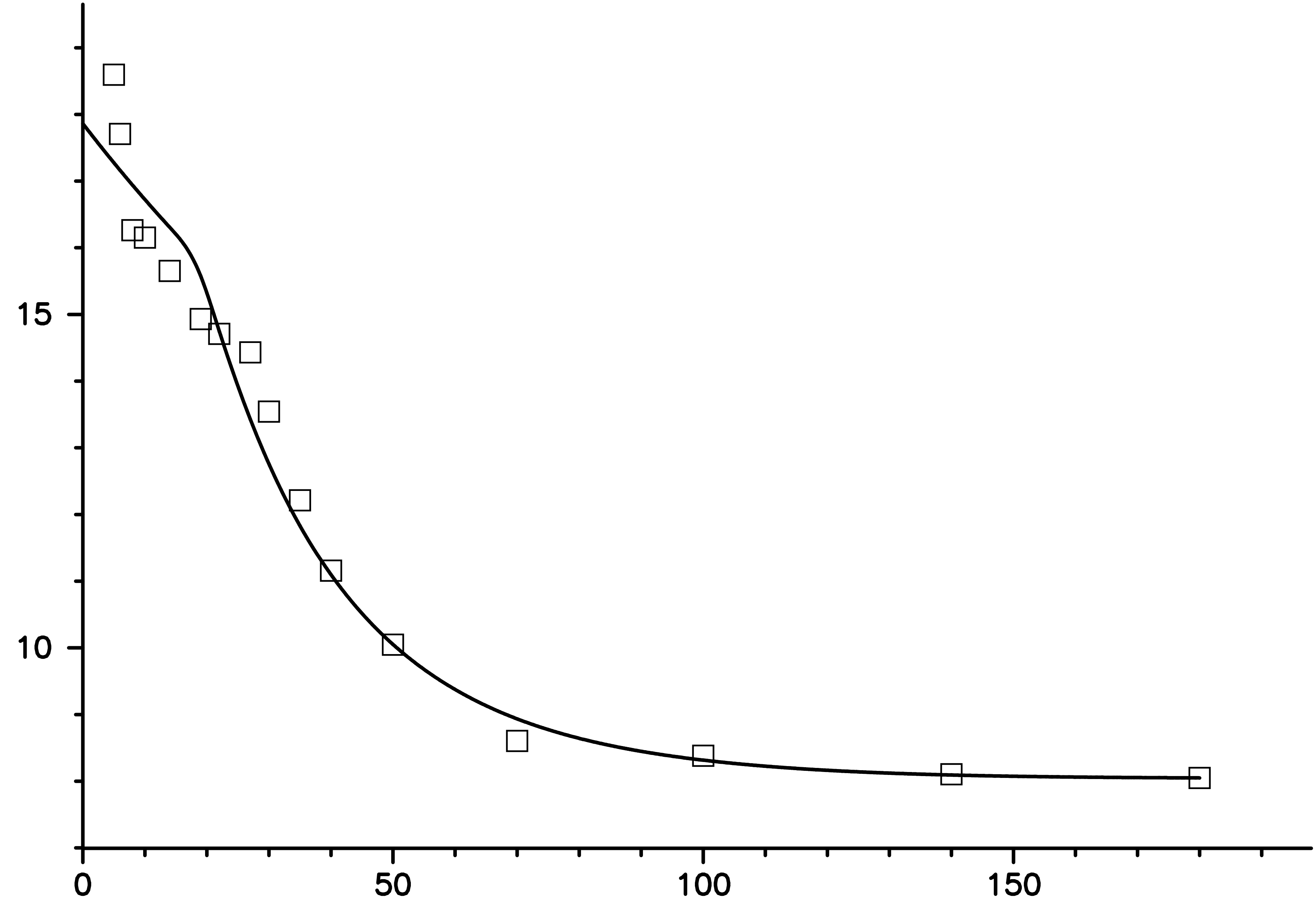

Data file: adapt_xlsx_pool_imputation.csv

Model: IVGTTmodel1.for: Minimal Model Analysis, IVGTT

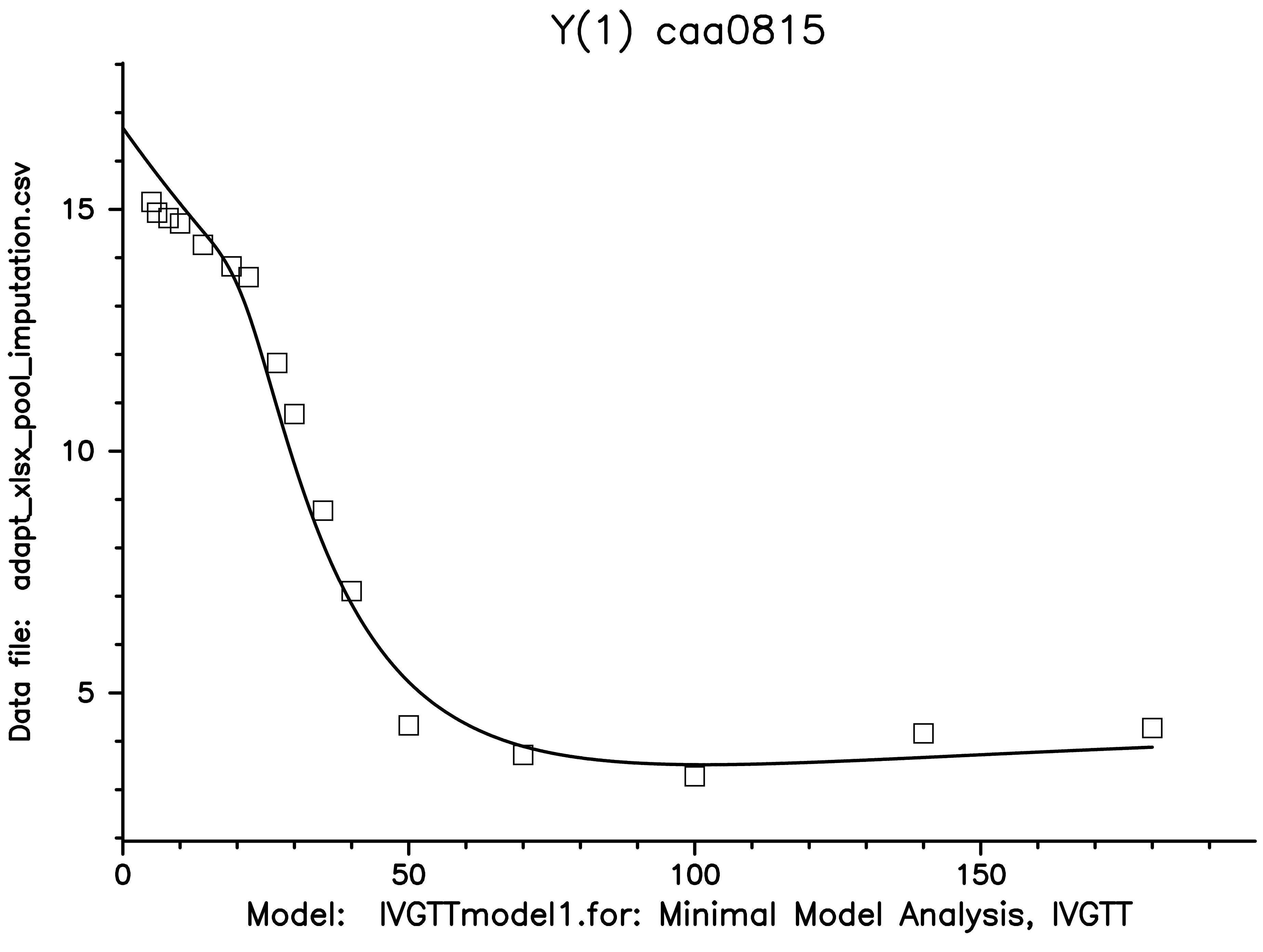
Y(1) caa0815
Data file: adapt_xlsx_pool_imputation.csv
15
10
5
0
50
100
150
Model: IVGTTmodel1.for: Minimal Model Analysis, IVGTT

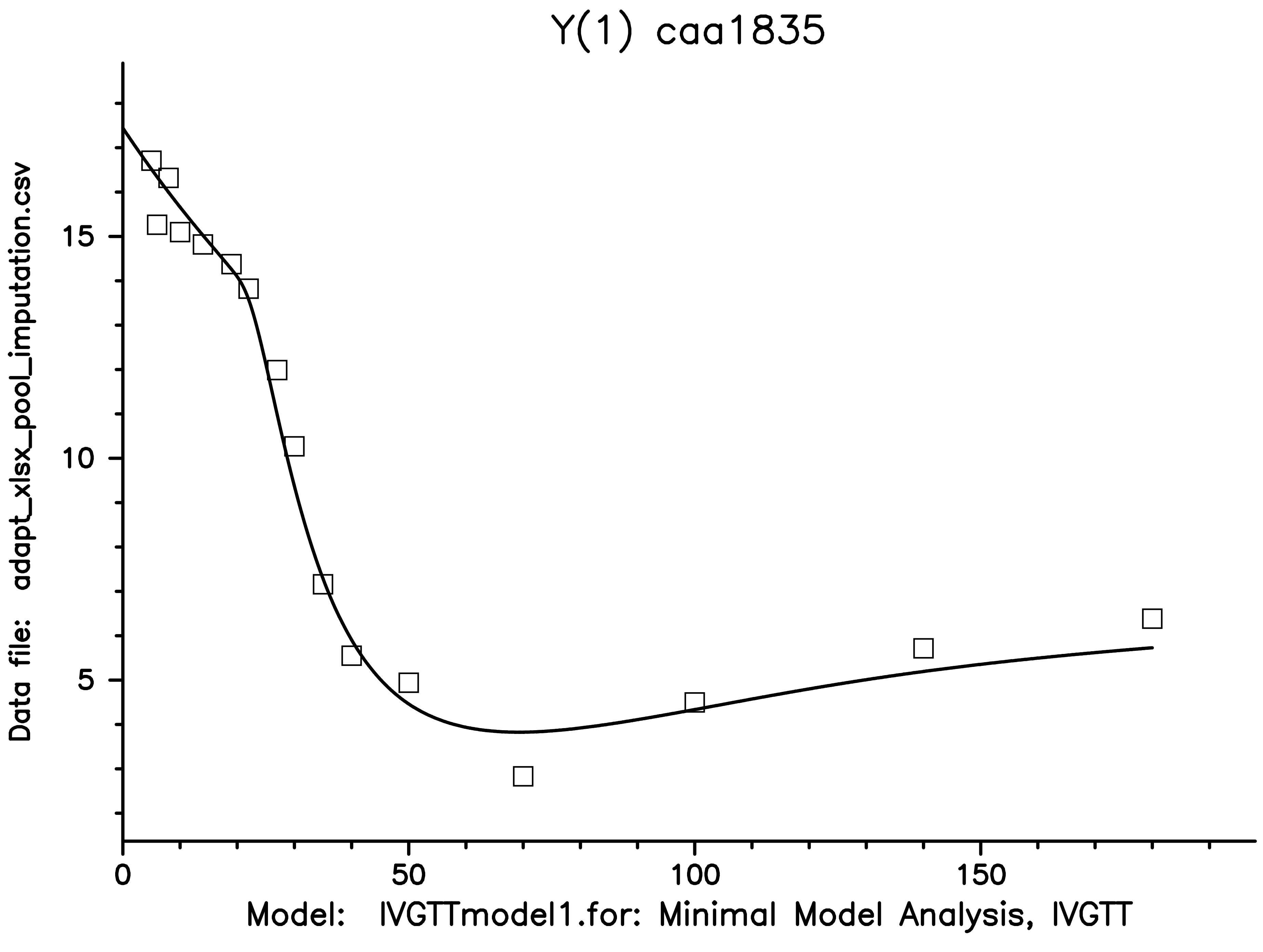
Y(1) caa1835
Data file: adapt_xlsx_pool_imputation.csv
15
10
5
0
50
100
150
Model: IVGTTmodel1.for: Minimal Model Analysis, IVGTT

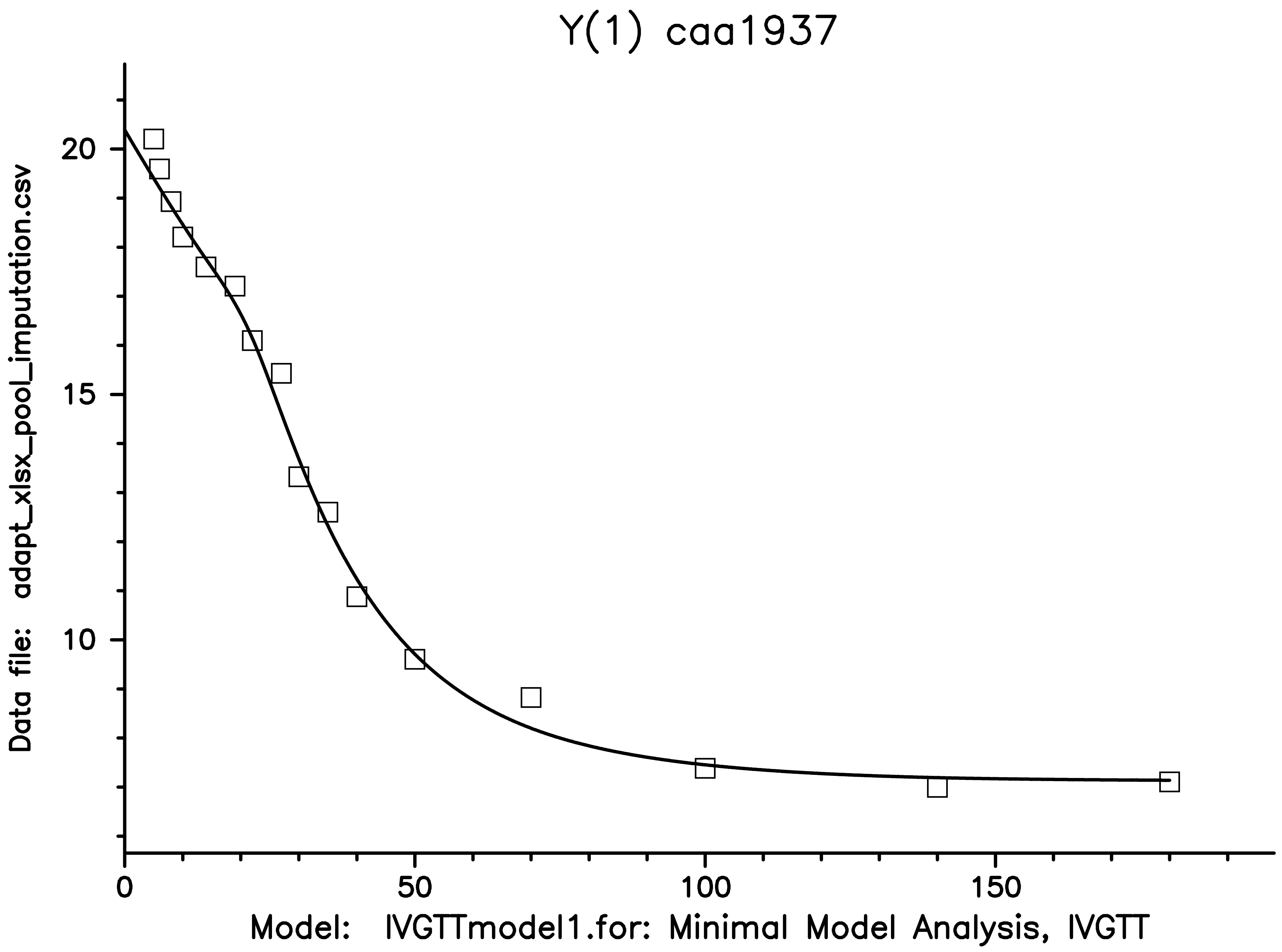
Y(1) caa1937
Data file: adapt_xlsx_pool_imputation.csv
Model: IVGTTmodel1.for: Minimal Model Analysis, IVGTT
20
15
10
0
50
100
150

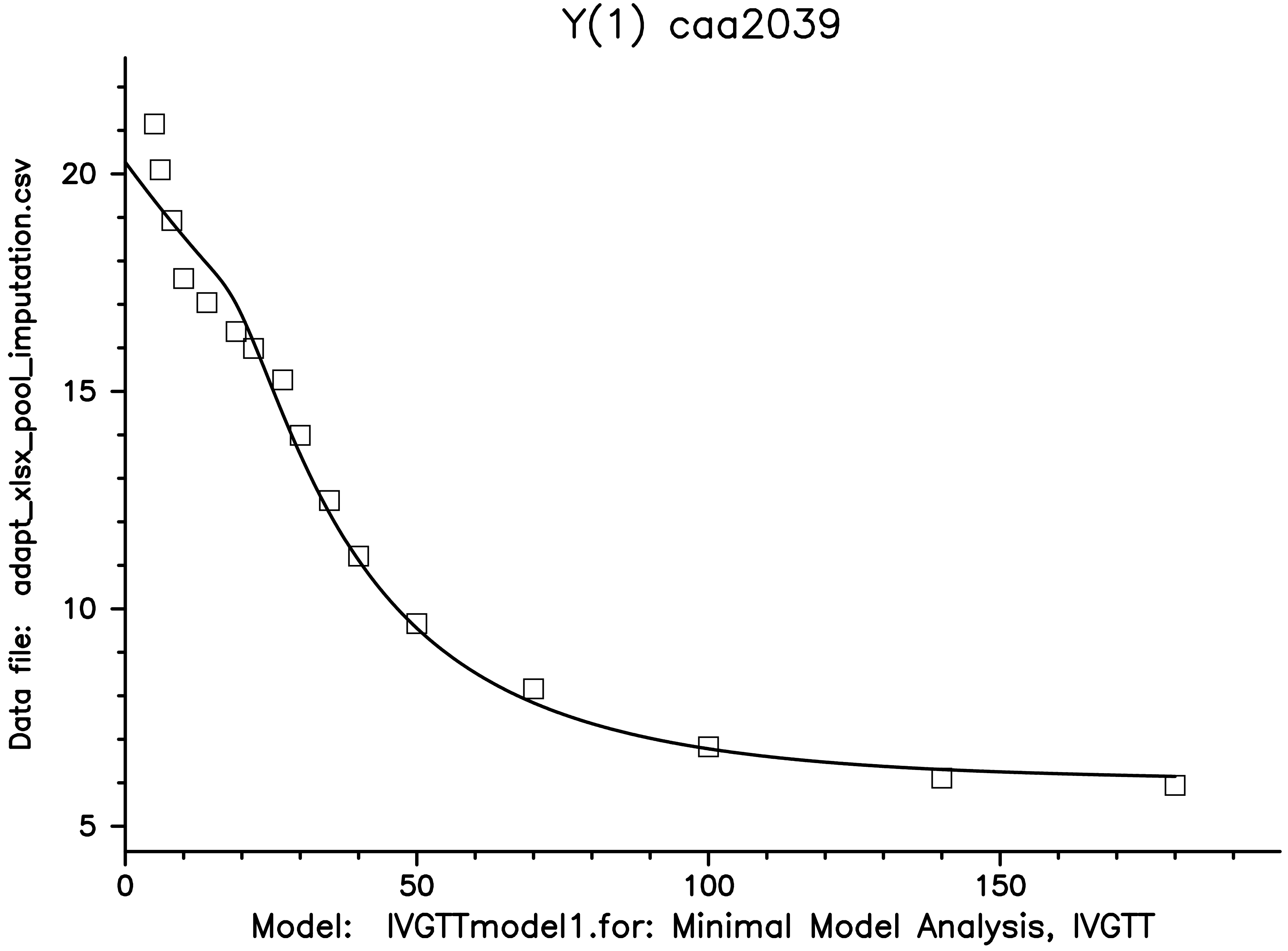

Y(1) caa2039
Data file: adapt_xlsx_pool_imputation.csv
20
15
10
5
0
50
100
150
Model: IVGTTmodel1.for: Minimal Model Analysis, IVGTT

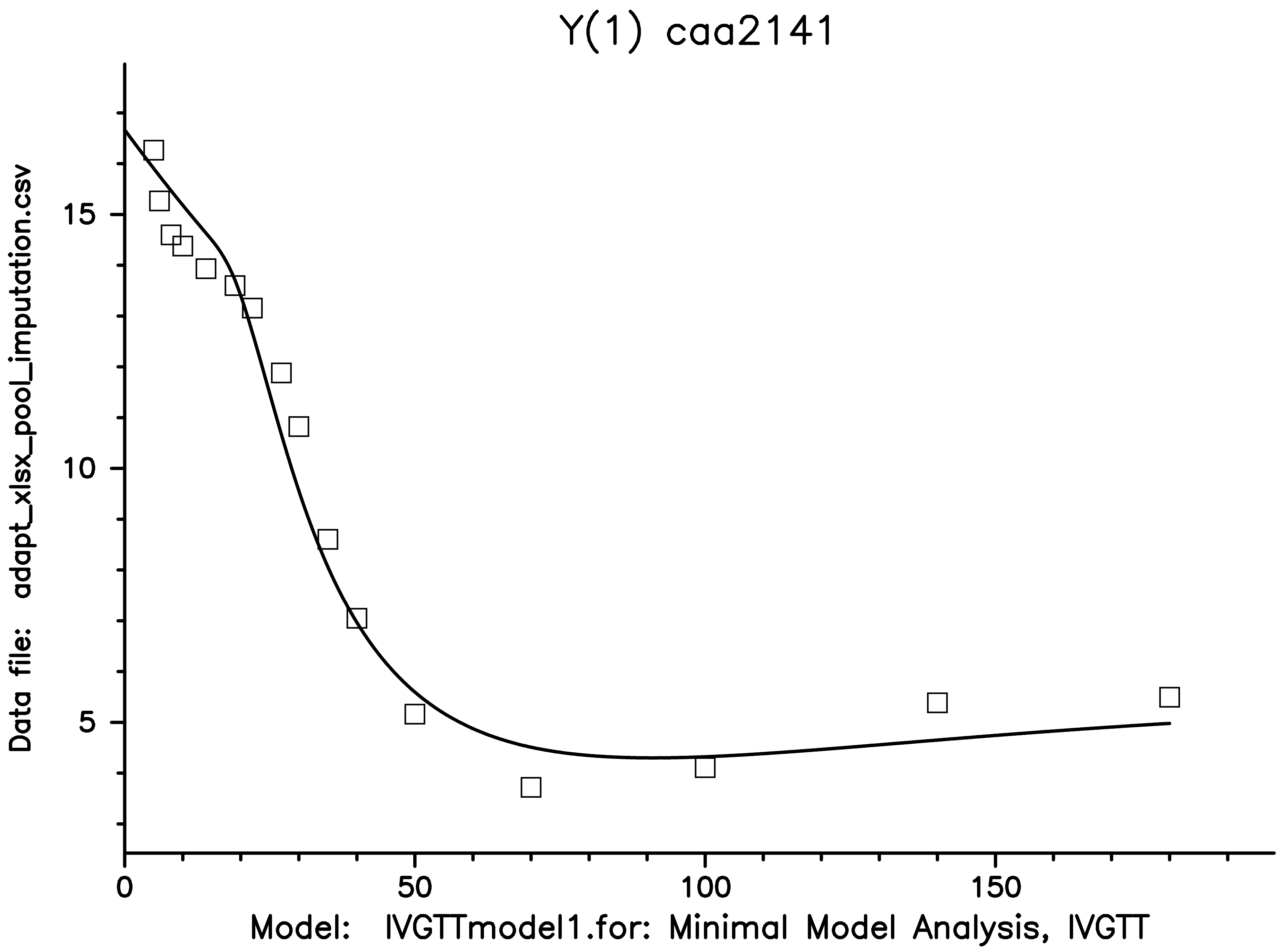
Y(1) caa2141
Data file: adapt_xlsx_pool_imputation.csv
15
10
5
0
50
100
150
Model: IVGTTmodel1.for: Minimal Model Analysis, IVGTT

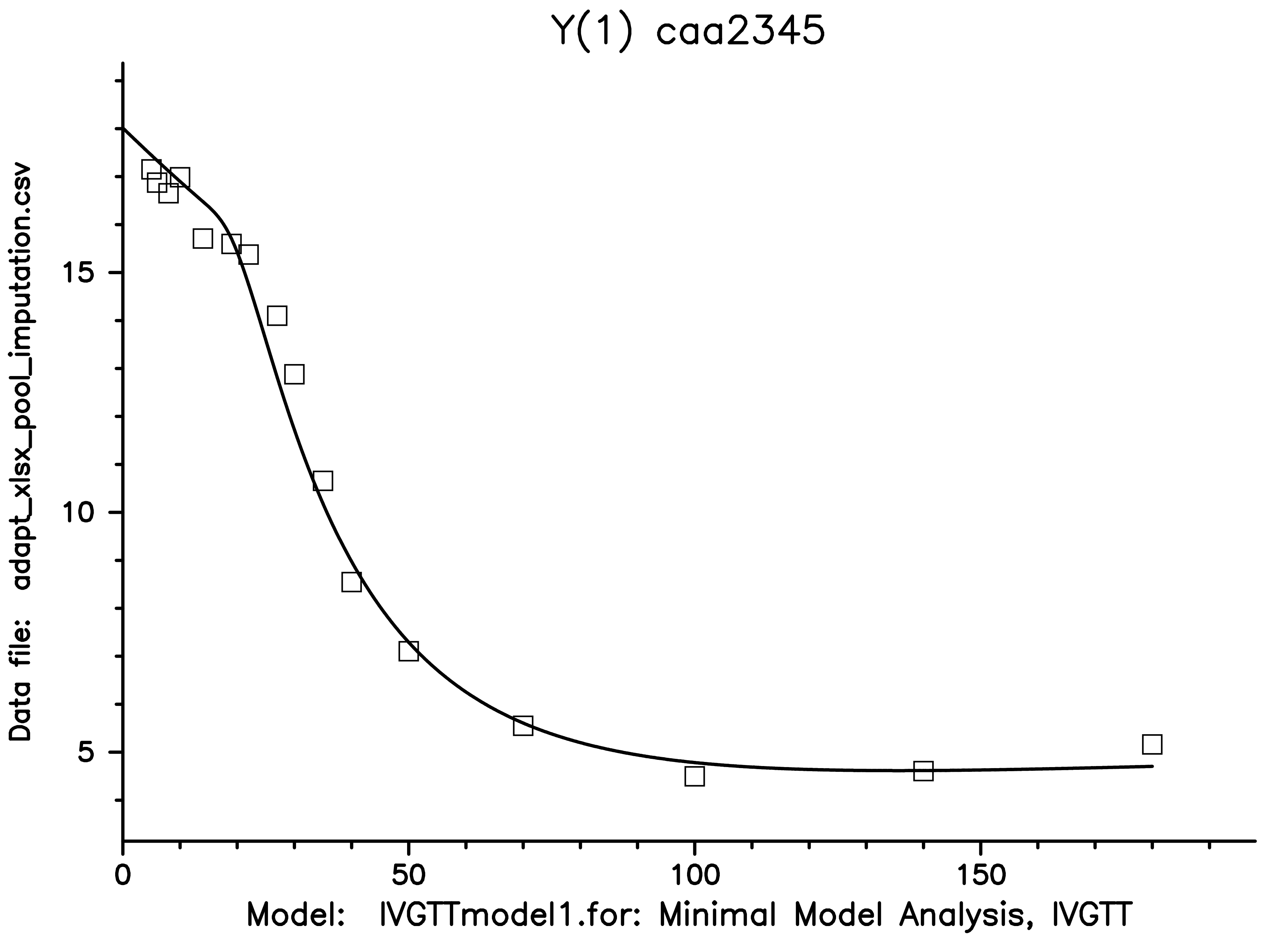
Y(1) caa2345
Data file: adapt_xlsx_pool_imputation.csv
15
10
5
0
50
100
150
Model: IVGTTmodel1.for: Minimal Model Analysis, IVGTT

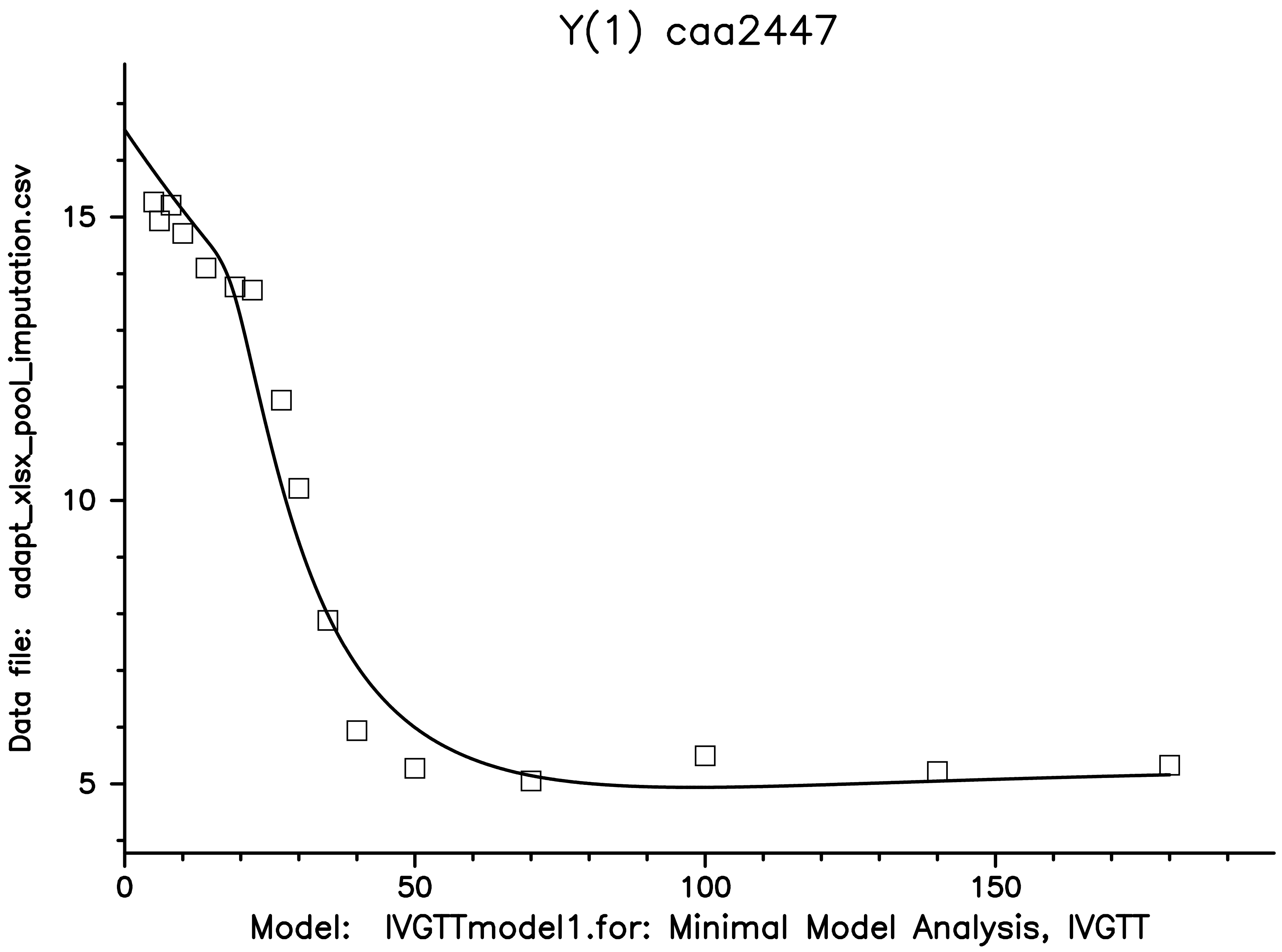
Y(1) caa2447
Data file: adapt_xlsx_pool_imputation.csv
15
10
5
0
50
100
150
Model: IVGTTmodel1.for: Minimal Model Analysis, IVGTT

Y(1) caa2549

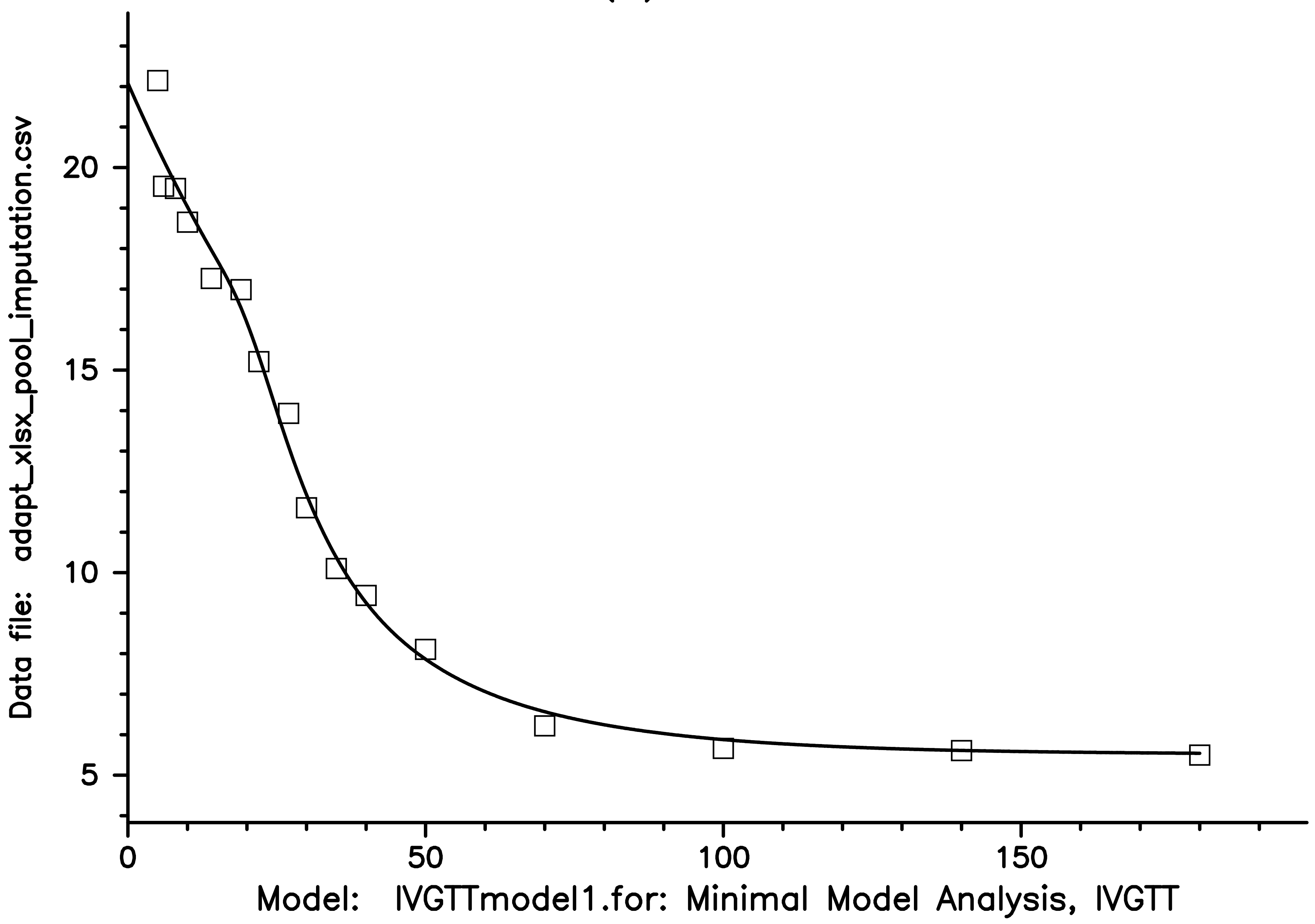

20
15
10
5
0
50
100
150
Data file: adapt_xlsx_pool_imputation.csv
Model: IVGTTmodel1.for: Minimal Model Analysis, IVGTT

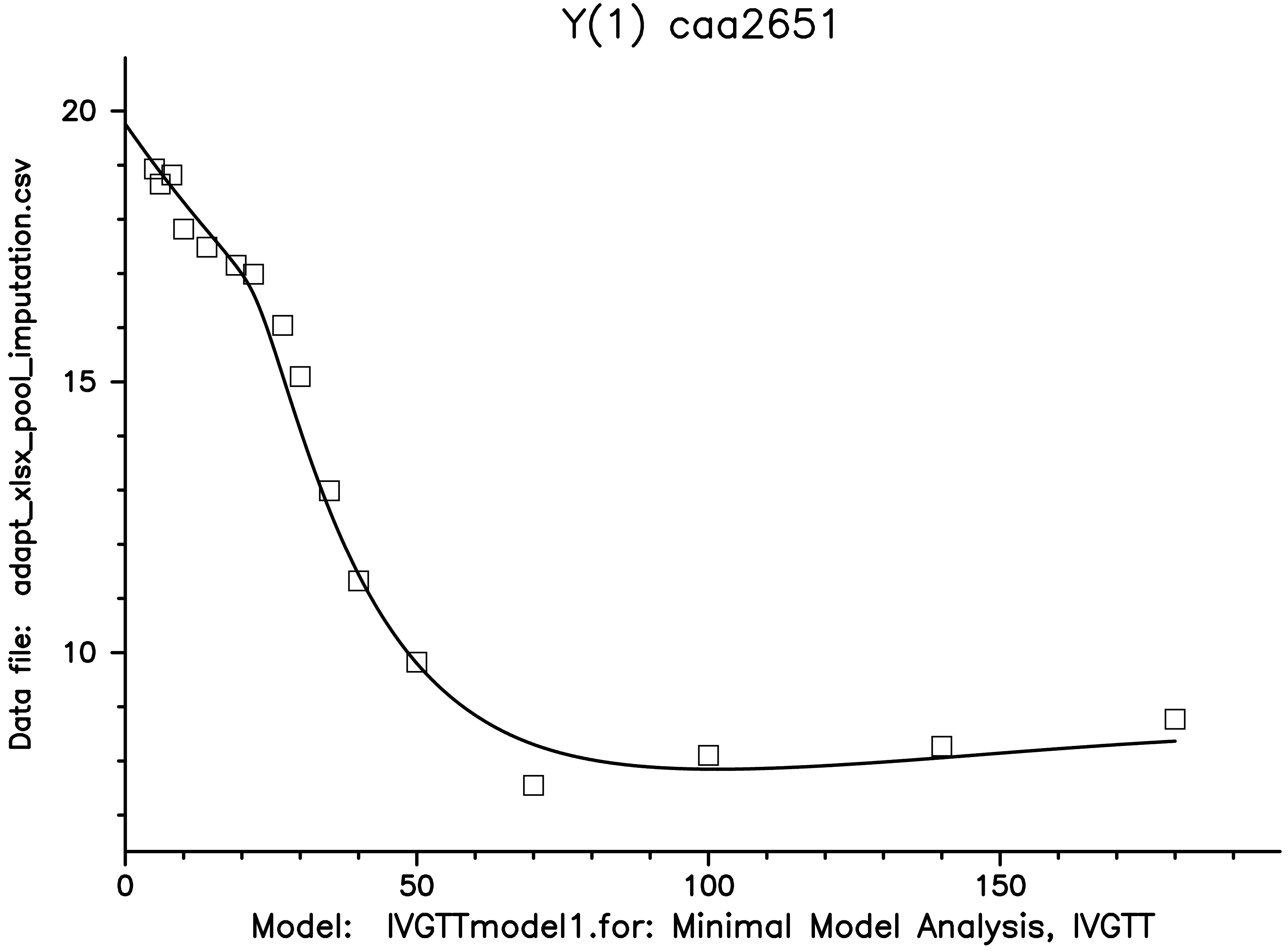
Y(1) caa2651
Data file: adapt_xlsx_pool_imputation.csv
Model: IVGTTmodel1.for: Minimal Model Analysis, IVGTT
20
15
10
0
50
100
150

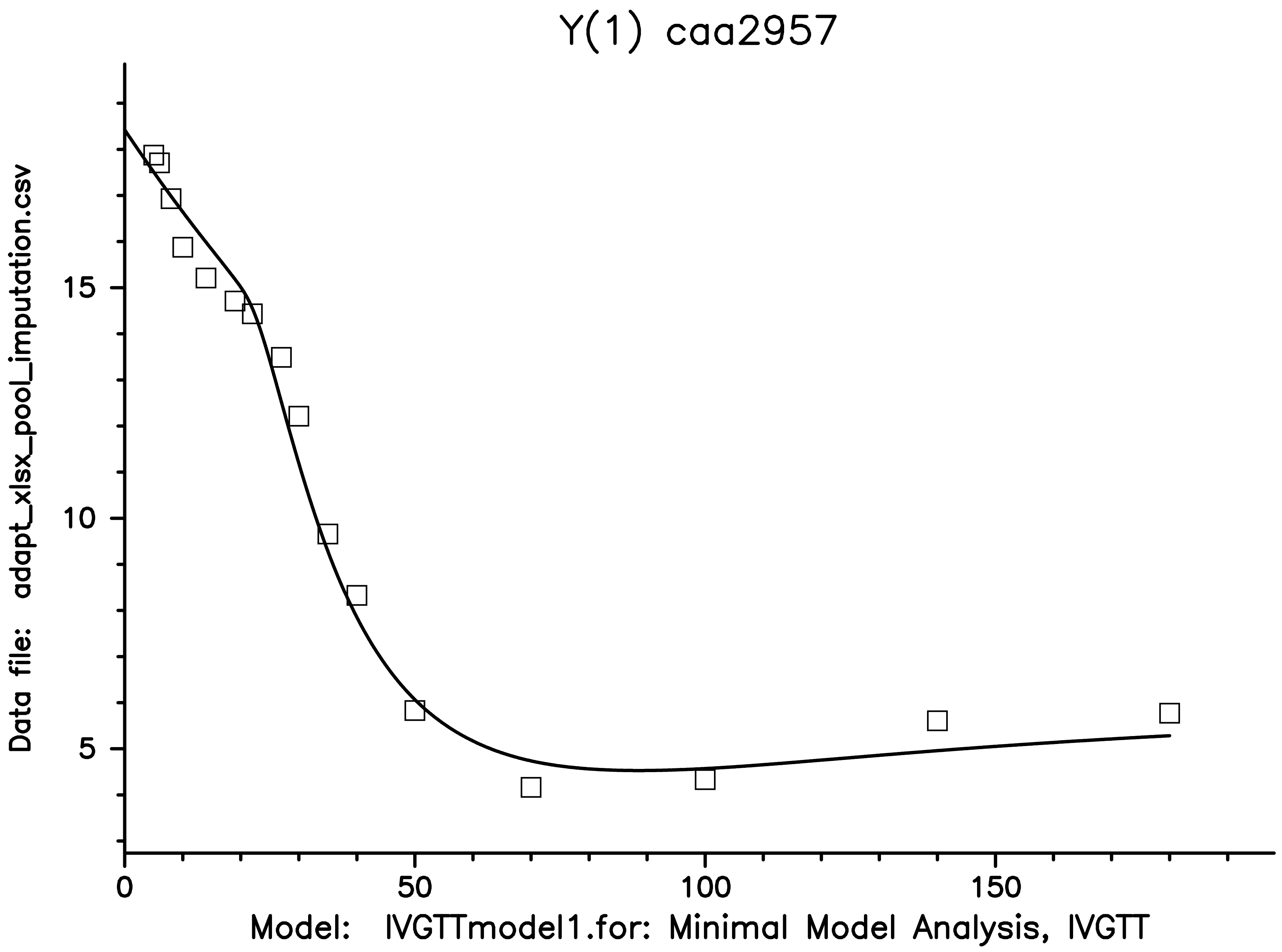

Y(1) caa2957
Data file: adapt_xlsx_pool_imputation.csv
Model: IVGTTmodel1.for: Minimal Model Analysis, IVGTT
0
50
100
150
5
10
15

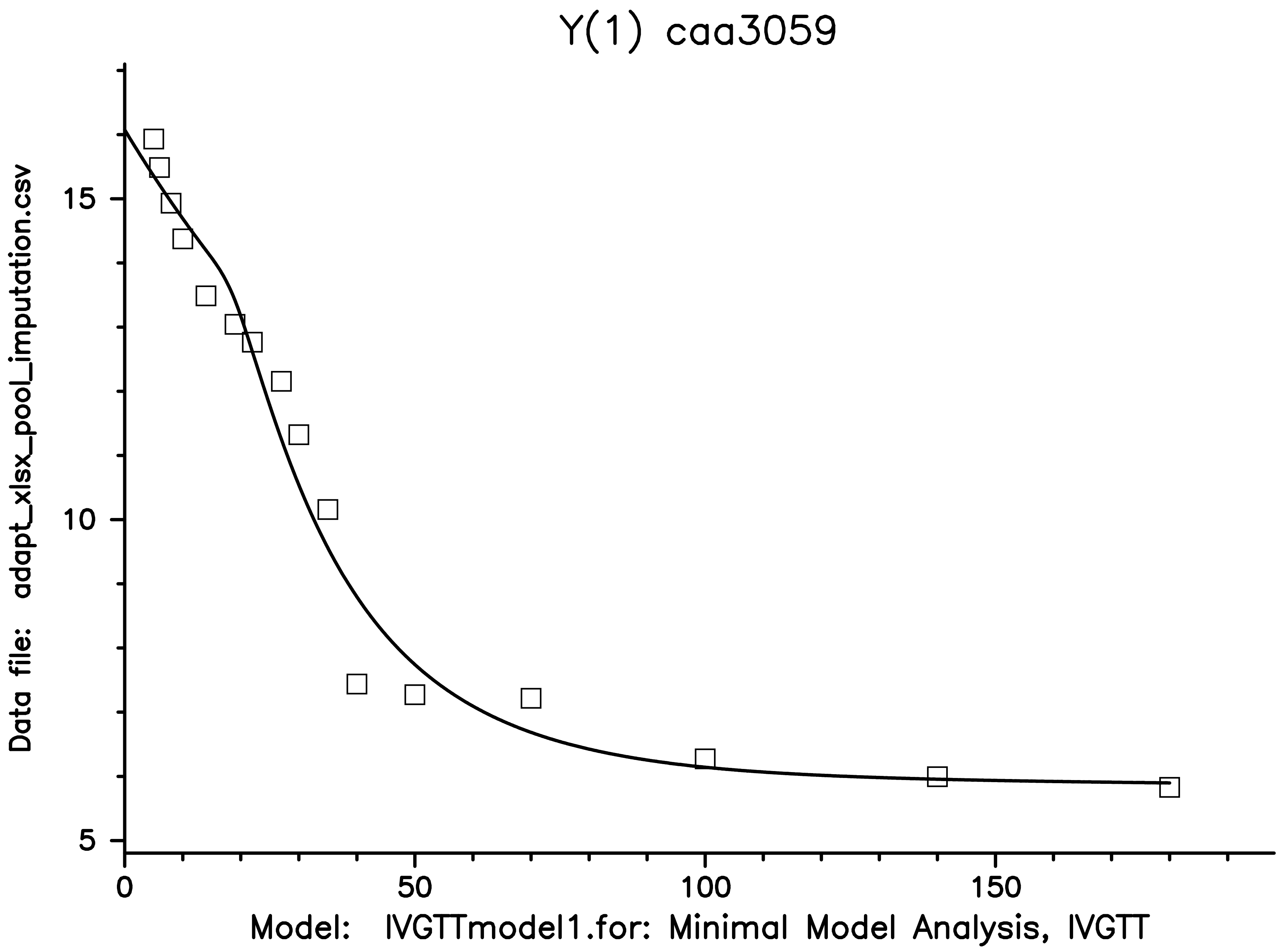
Y(1) caa3059
Data file: adapt_xlsx_pool_imputation.csv
15
10
5
0
50
100
150
Model: IVGTTmodel1.for: Minimal Model Analysis, IVGTT

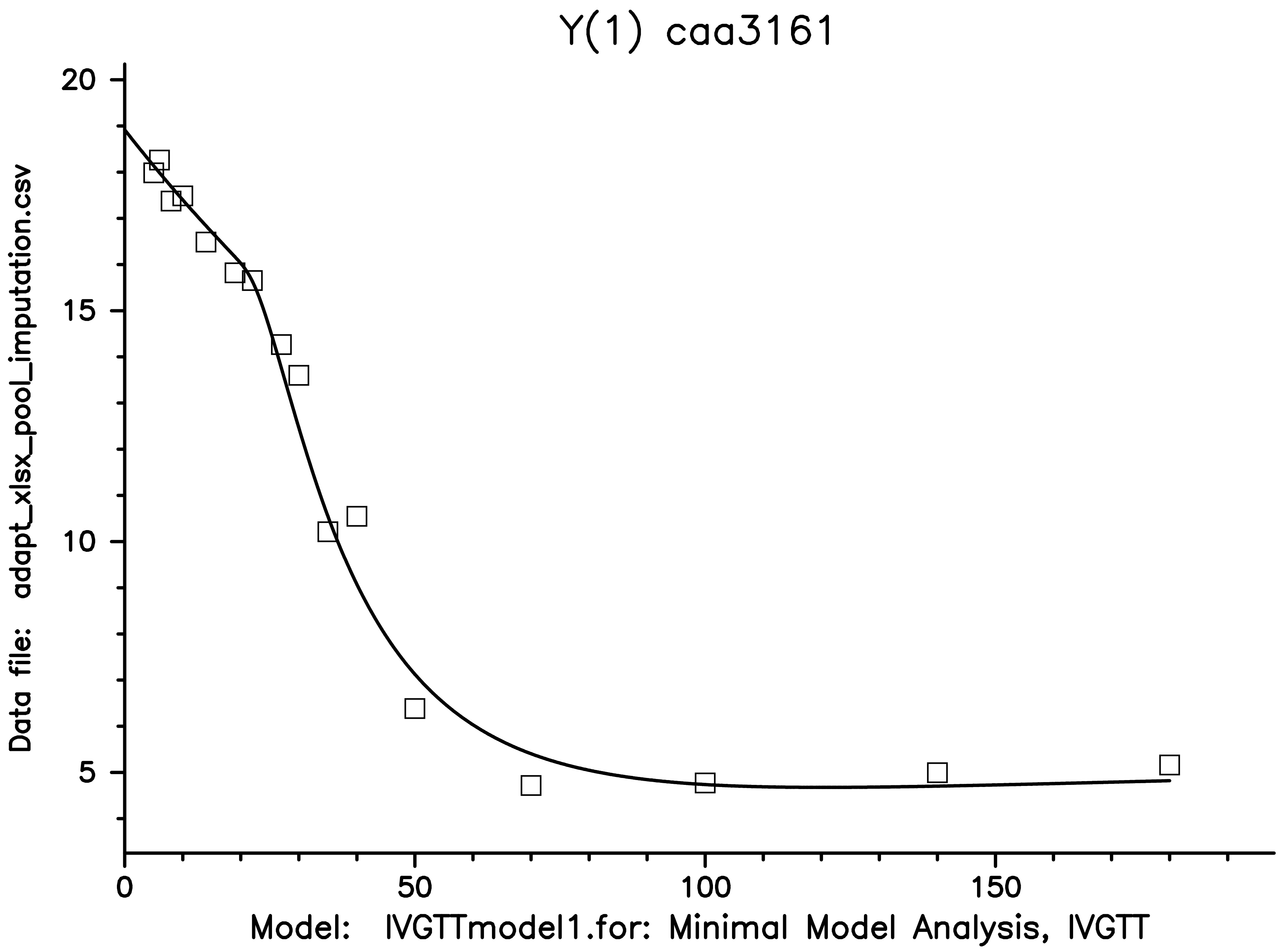

Y(1) caa3161
20
15
10
5
0
50
100
150
Data file: adapt_xlsx_pool_imputation.csv
Model: IVGTTmodel1.for: Minimal Model Analysis, IVGTT

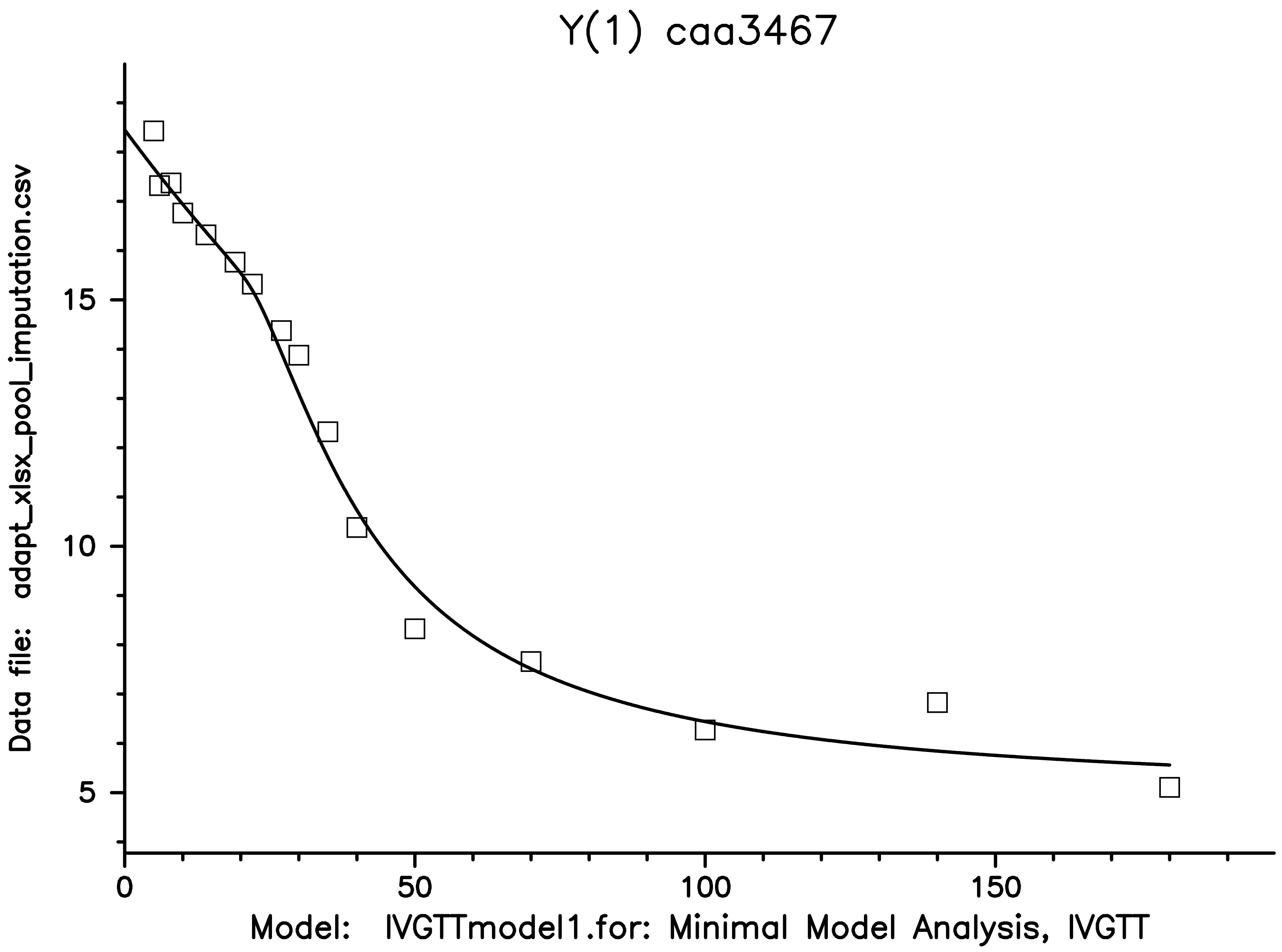
Y(1) caa3467
Data file: adapt_xlsx_pool_imputation.csv
15
10
5
0
50
100
150
Model: IVGTTmodel1.for: Minimal Model Analysis, IVGTT

Y(1) pdalb01

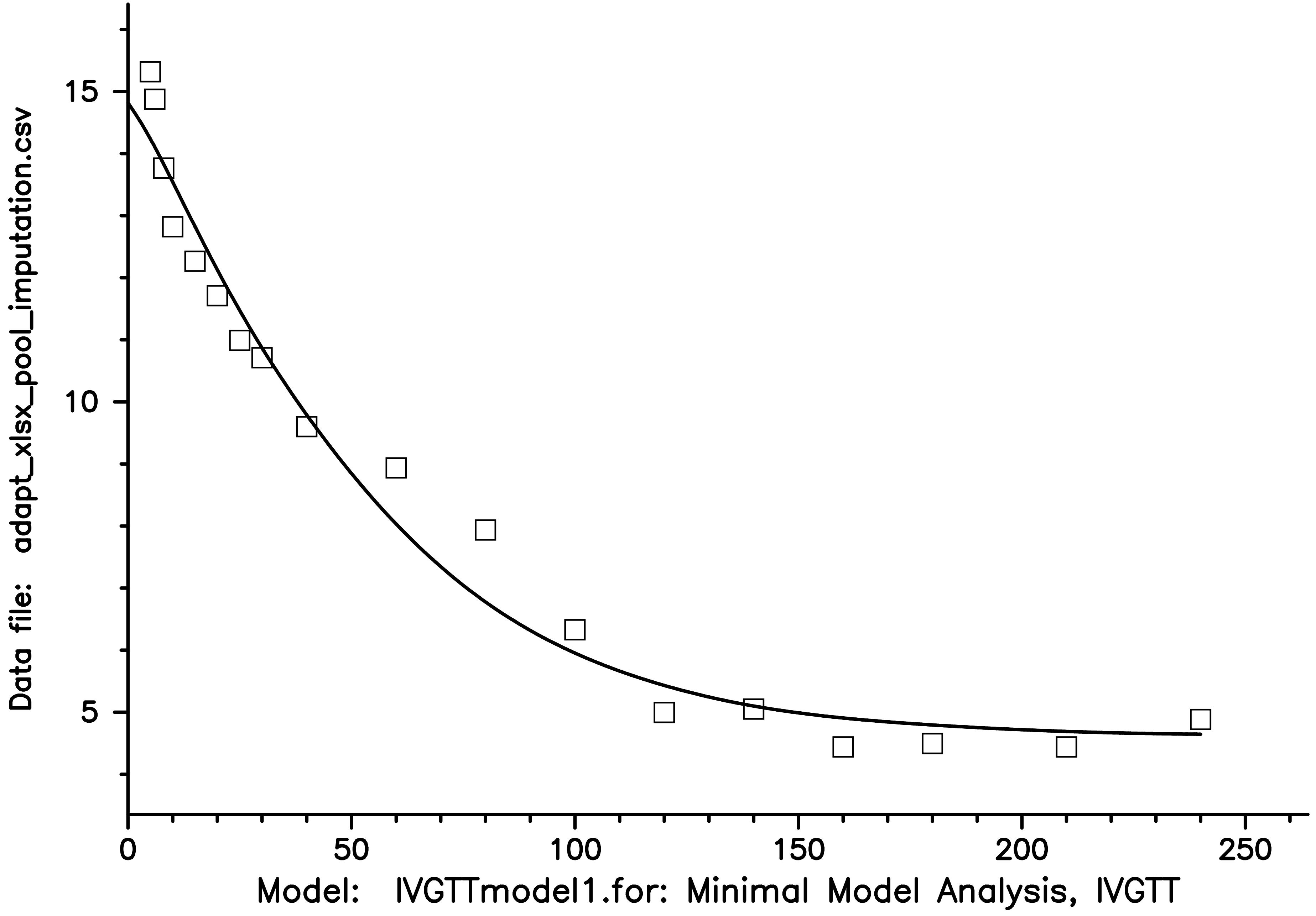

15
10
5
0
50
100
150
200
250
Data file: adapt_xlsx_pool_imputation.csv
Model: IVGTTmodel1.for: Minimal Model Analysis, IVGTT

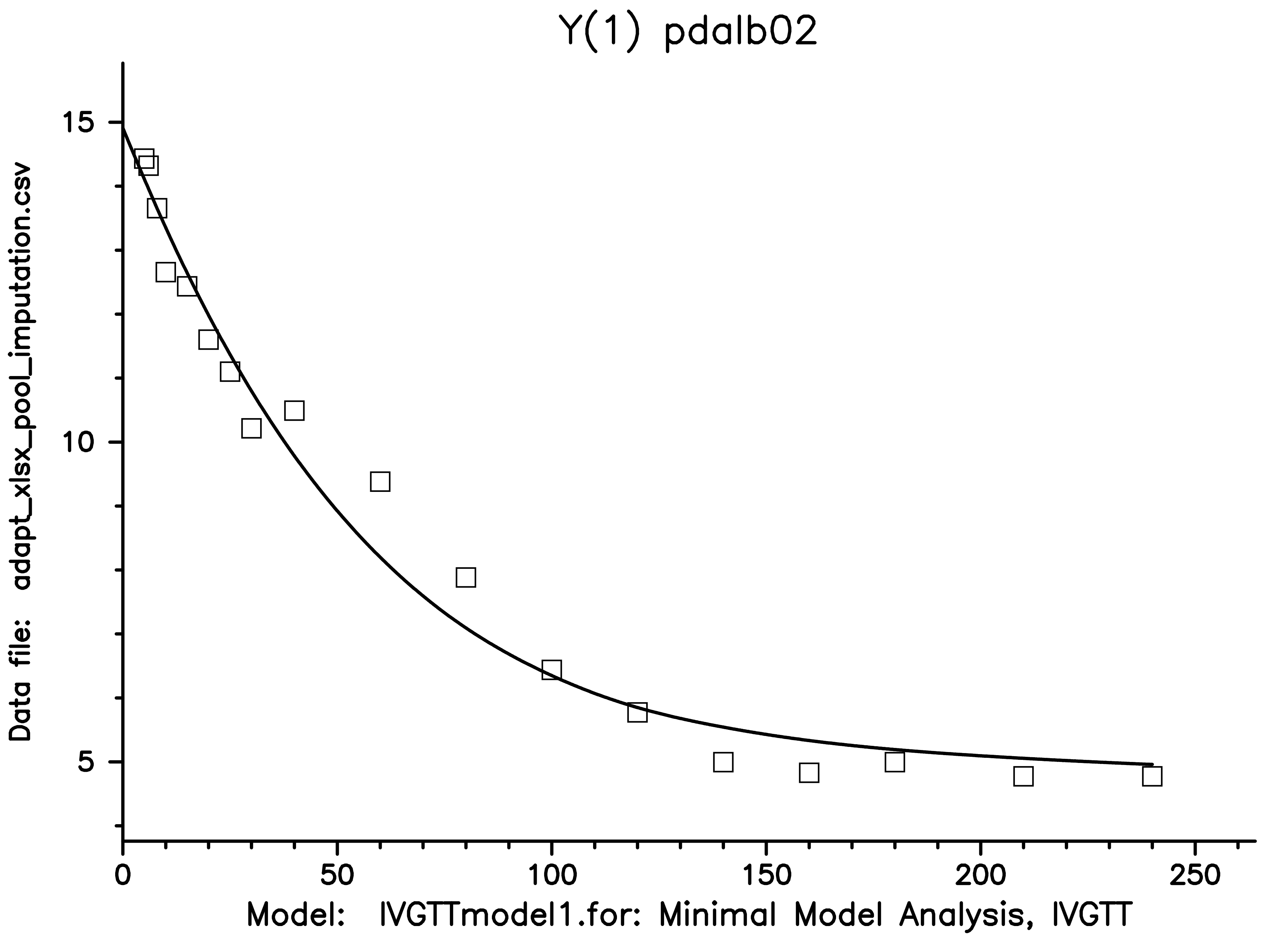

Y(1) pdalb02
Data file: adapt_xlsx_pool_imputation.csv
15
10
5
0
50
100
150
200
250
Model: IVGTTmodel1.for: Minimal Model Analysis, IVGTT

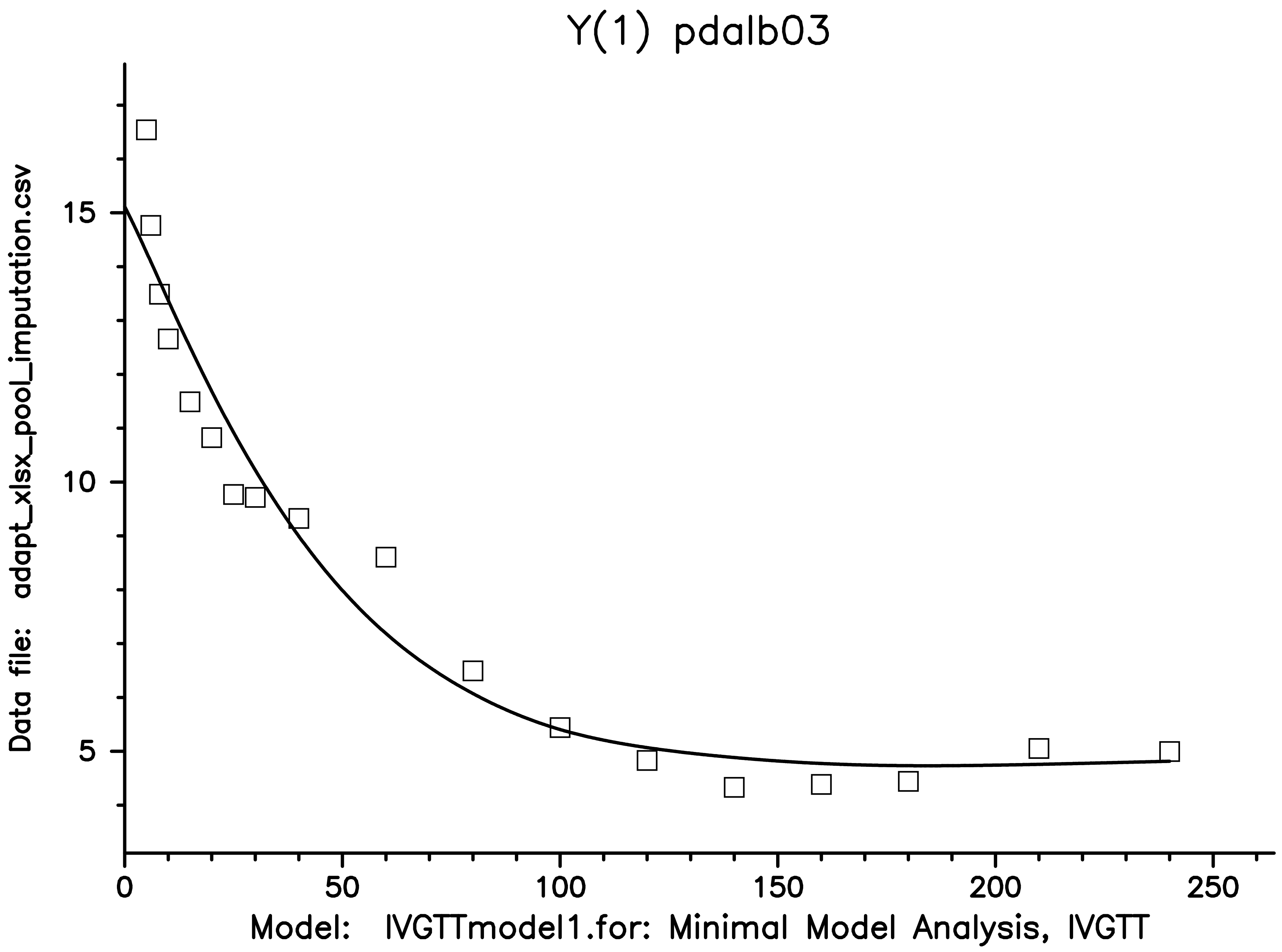
Y(1) pdalb03
Data file: adapt_xlsx_pool_imputation.csv
15
10
5
0
50
100
150
200
250
Model: IVGTTmodel1.for: Minimal Model Analysis, IVGTT

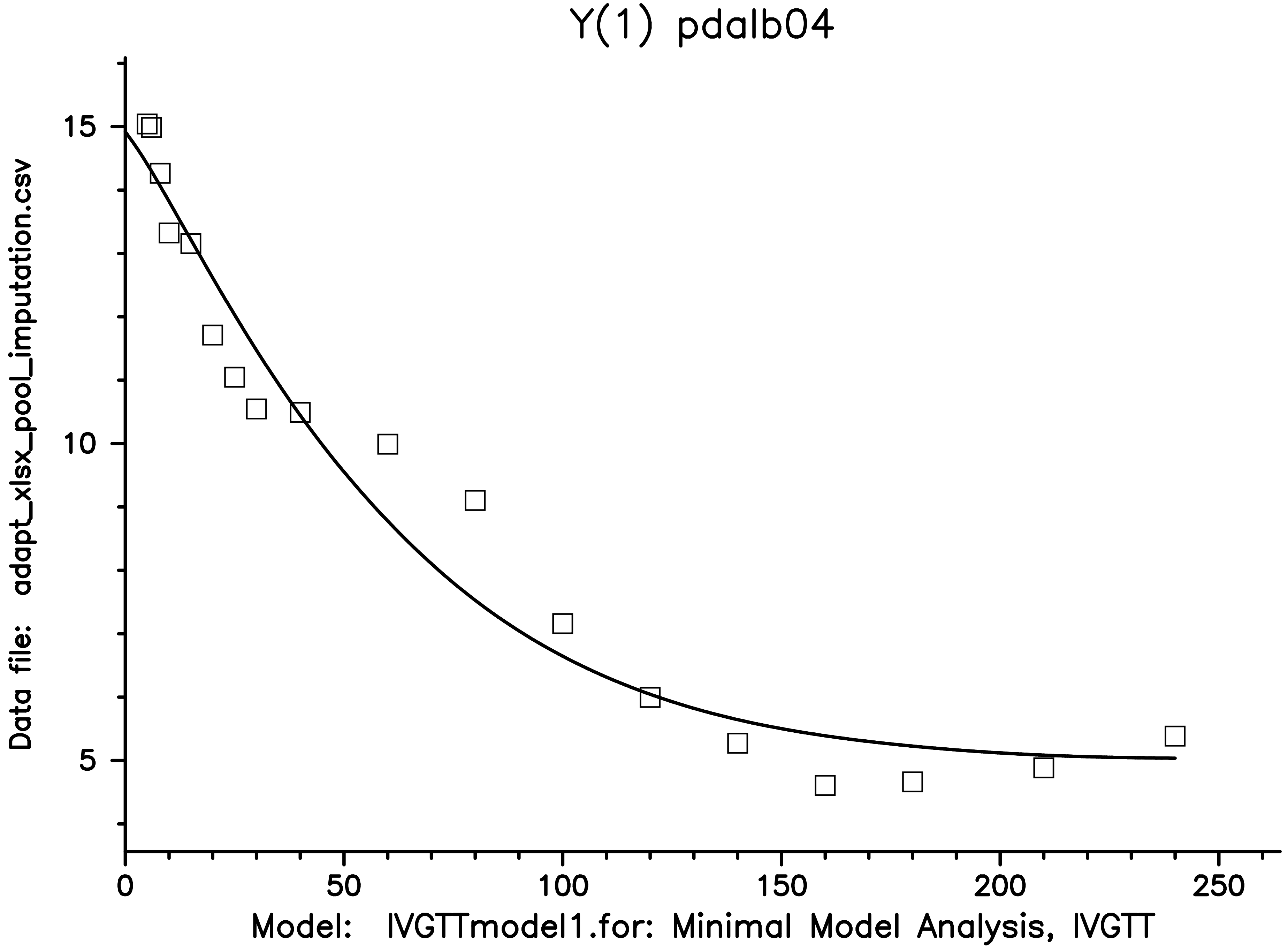
Y(1) pdalb04
Data file: adapt_xlsx_pool_imputation.csv
15
10
5
0
50
100
150
200
250
Model: IVGTTmodel1.for: Minimal Model Analysis, IVGTT

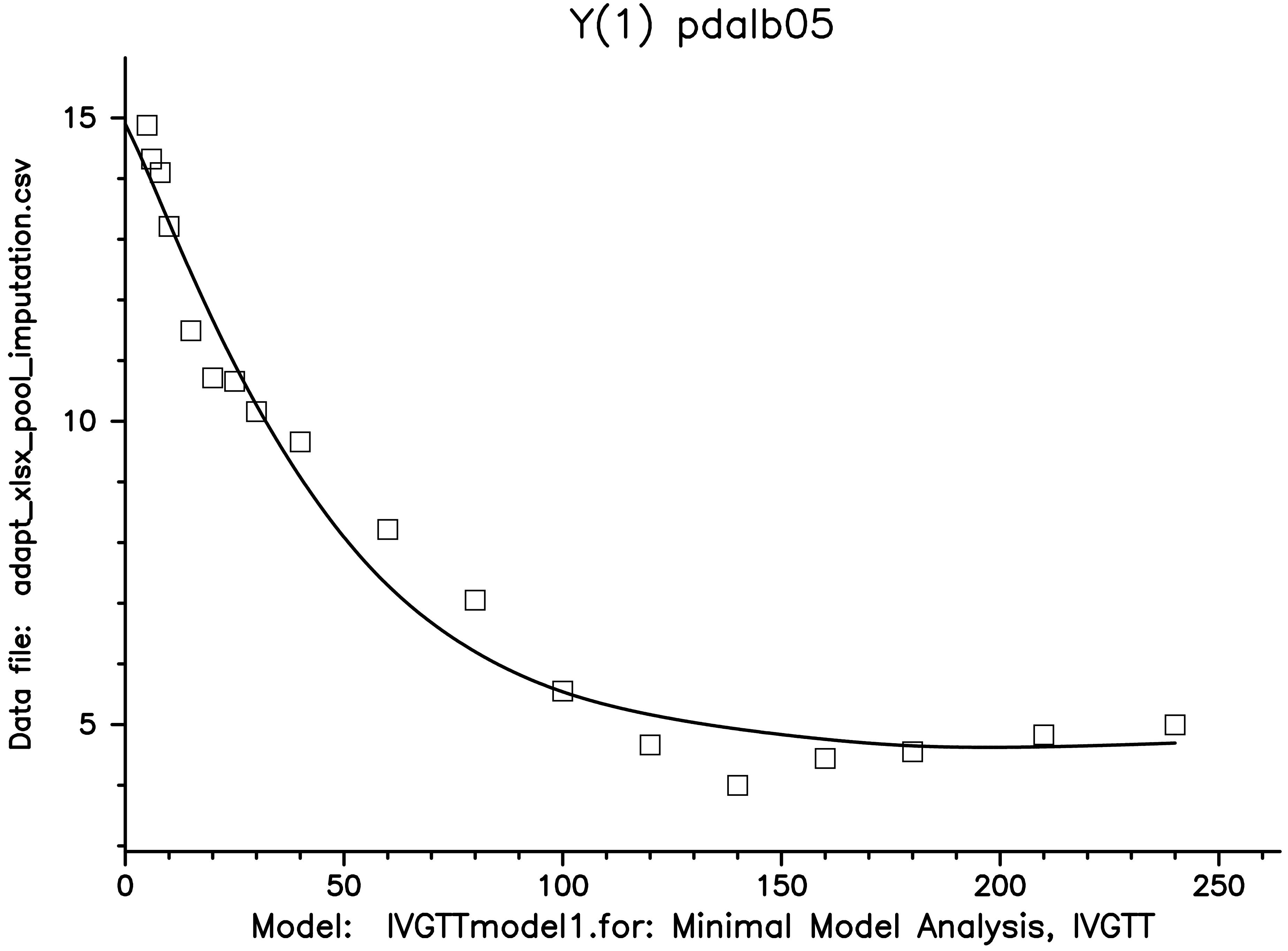
Y(1) pdalb05
Data file: adapt_xlsx_pool_imputation.csv
15
10
5
0
50
100
150
200
250
Model: IVGTTmodel1.for: Minimal Model Analysis, IVGTT

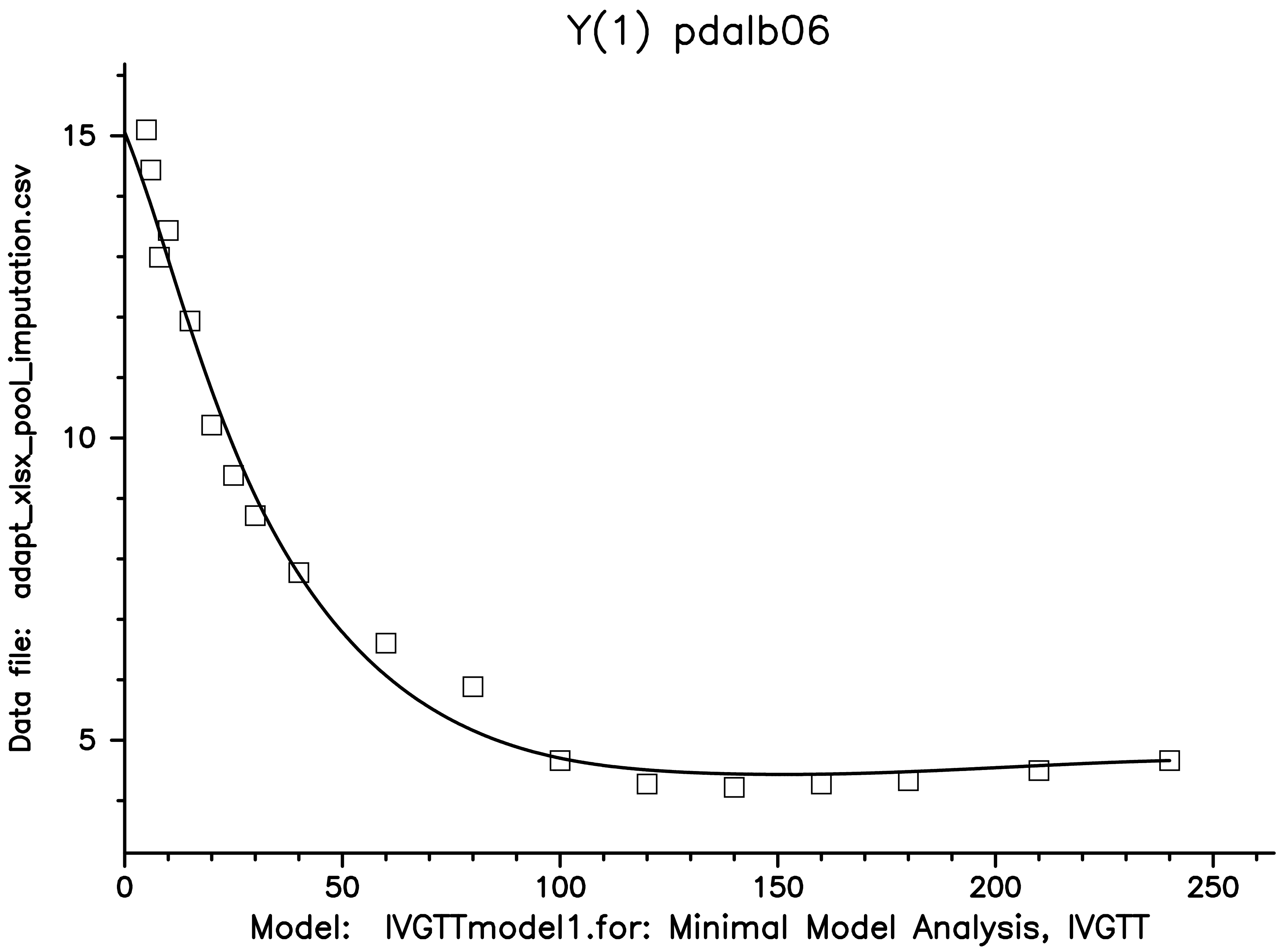
Y(1) pdalb06
Data file: adapt_xlsx_pool_imputation.csv
Model: IVGTTmodel1.for: Minimal Model Analysis, IVGTT
15
10
5
0
50
100
150
200
250

Y(1) pdalb07

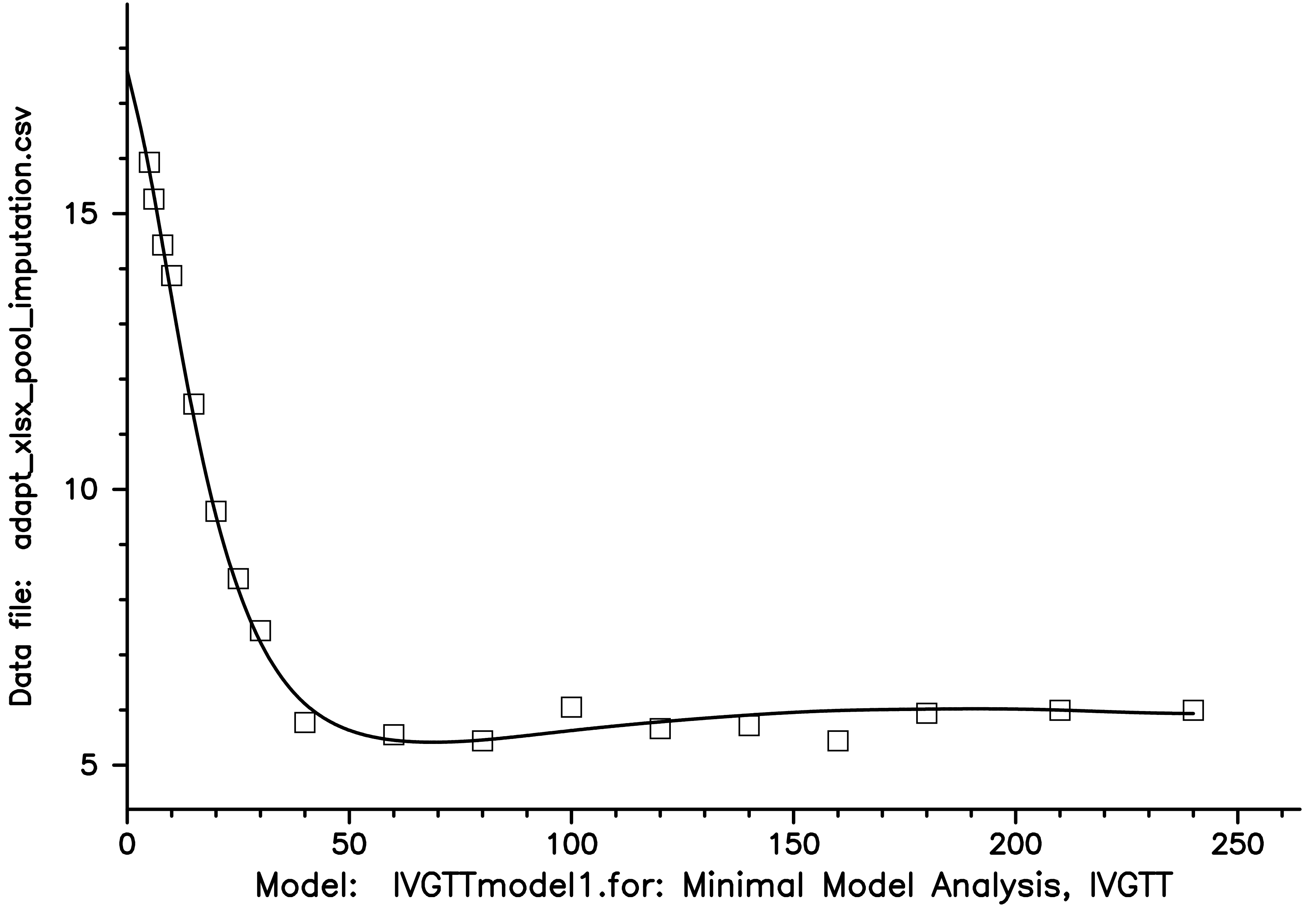
Data file: adapt_xlsx_pool_imputation.csv
15
10
5
0
50
100
150
200
250
Model: IVGTTmodel1.for: Minimal Model Analysis, IVGTT

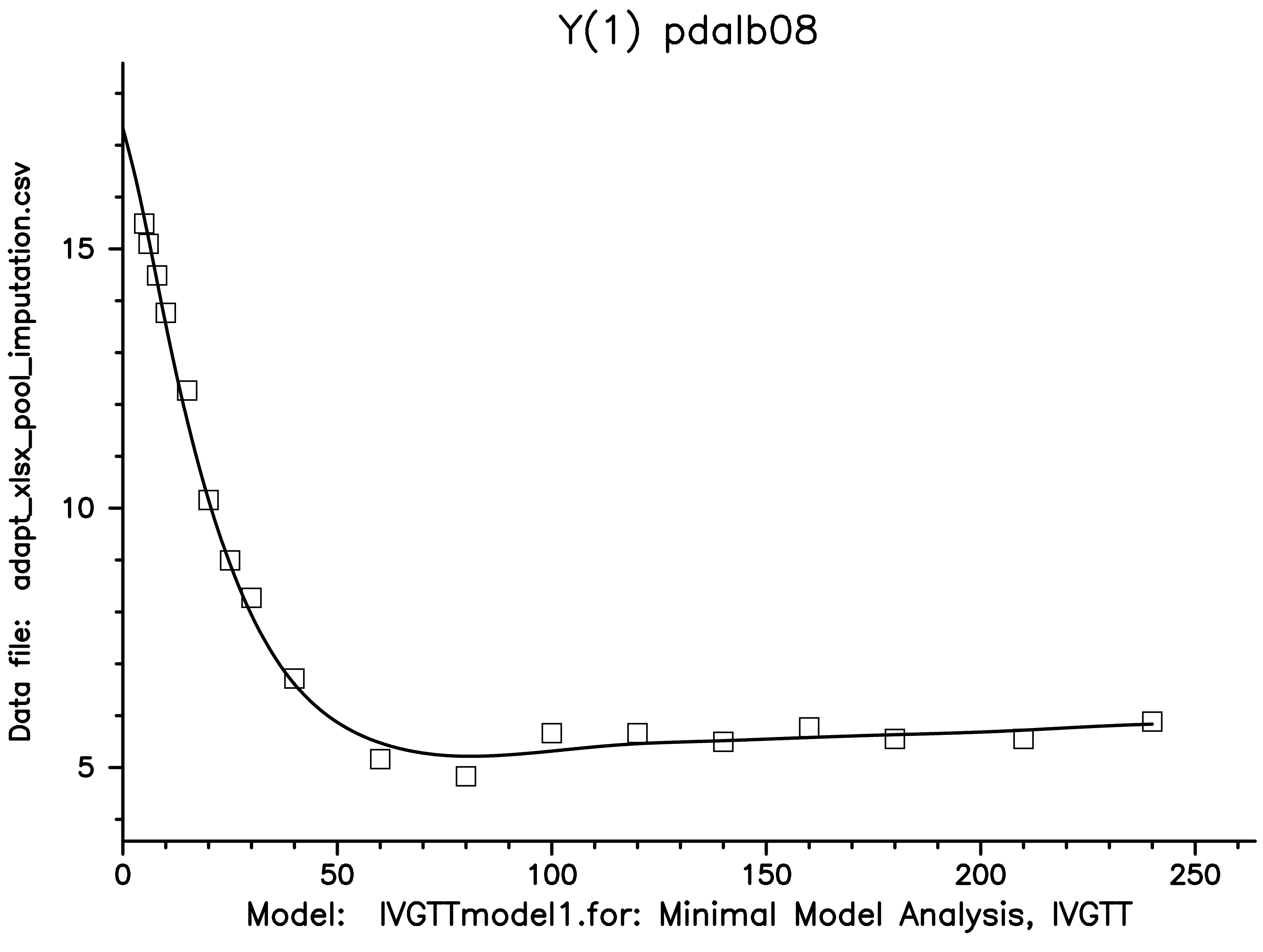
Y(1) pdalb08
Data file: adapt_xlsx_pool_imputation.csv
15
10
5
0
50
100
150
200
250
Model: IVGTTmodel1.for: Minimal Model Analysis, IVGTT

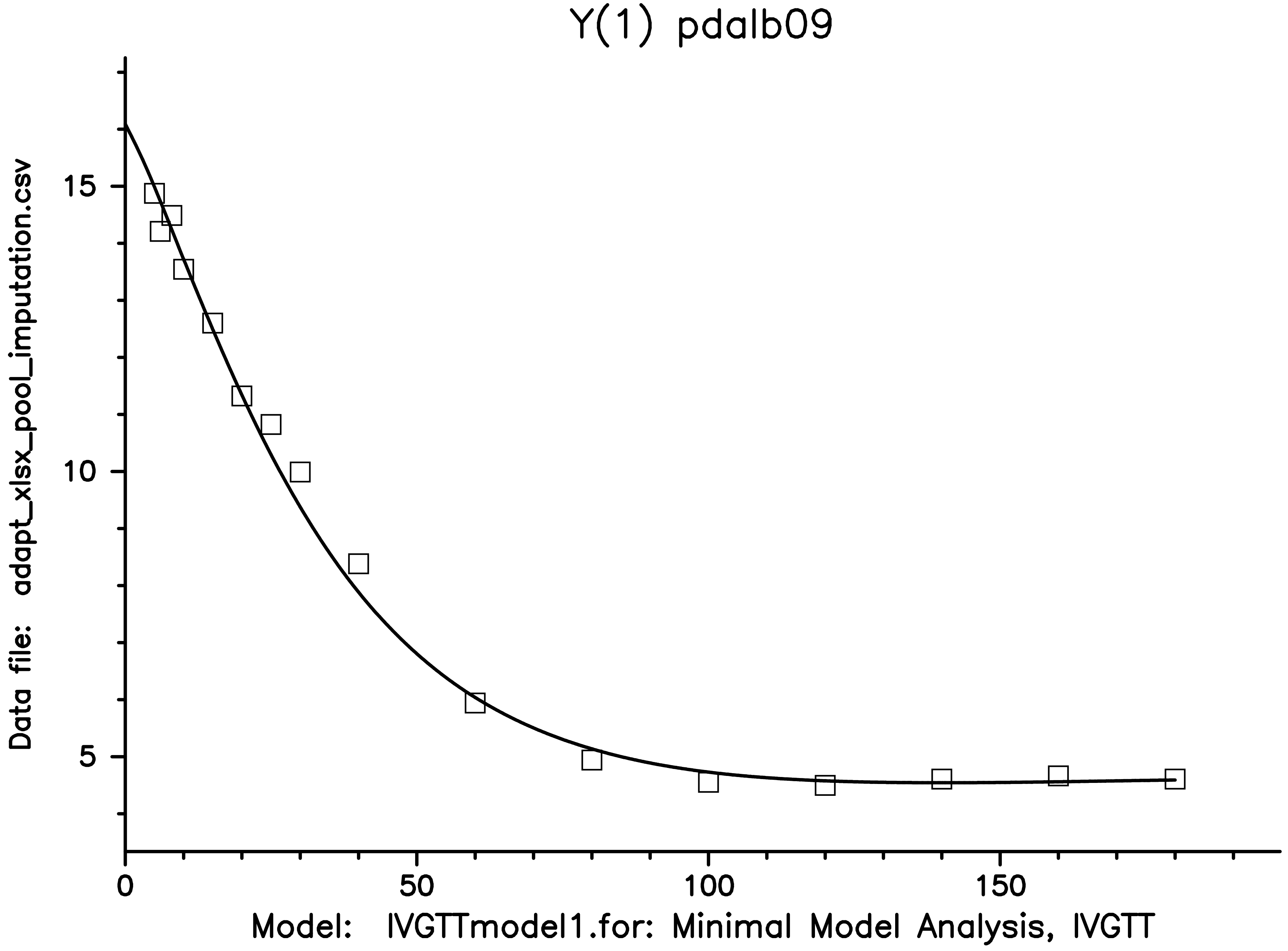

Y(1) pdalb09
Data file: adapt_xlsx_pool_imputation.csv
Model: IVGTTmodel1.for: Minimal Model Analysis, IVGTT
0
50
100
150
5
10
15

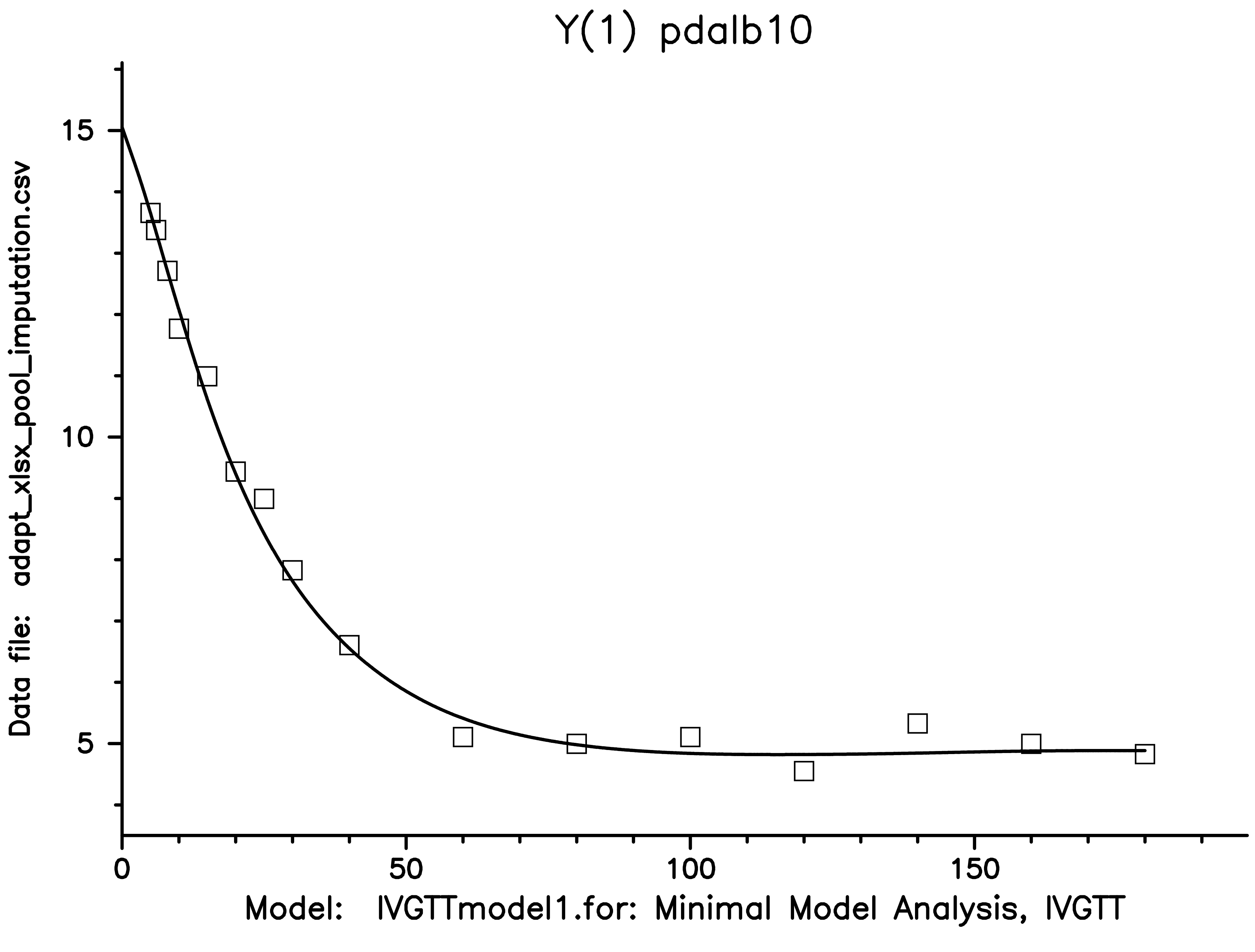

Y(1) pdalb10
Data file: adapt_xlsx_pool_imputation.csv
15
10
5
0
50
100
150
Model: IVGTTmodel1.for: Minimal Model Analysis, IVGTT

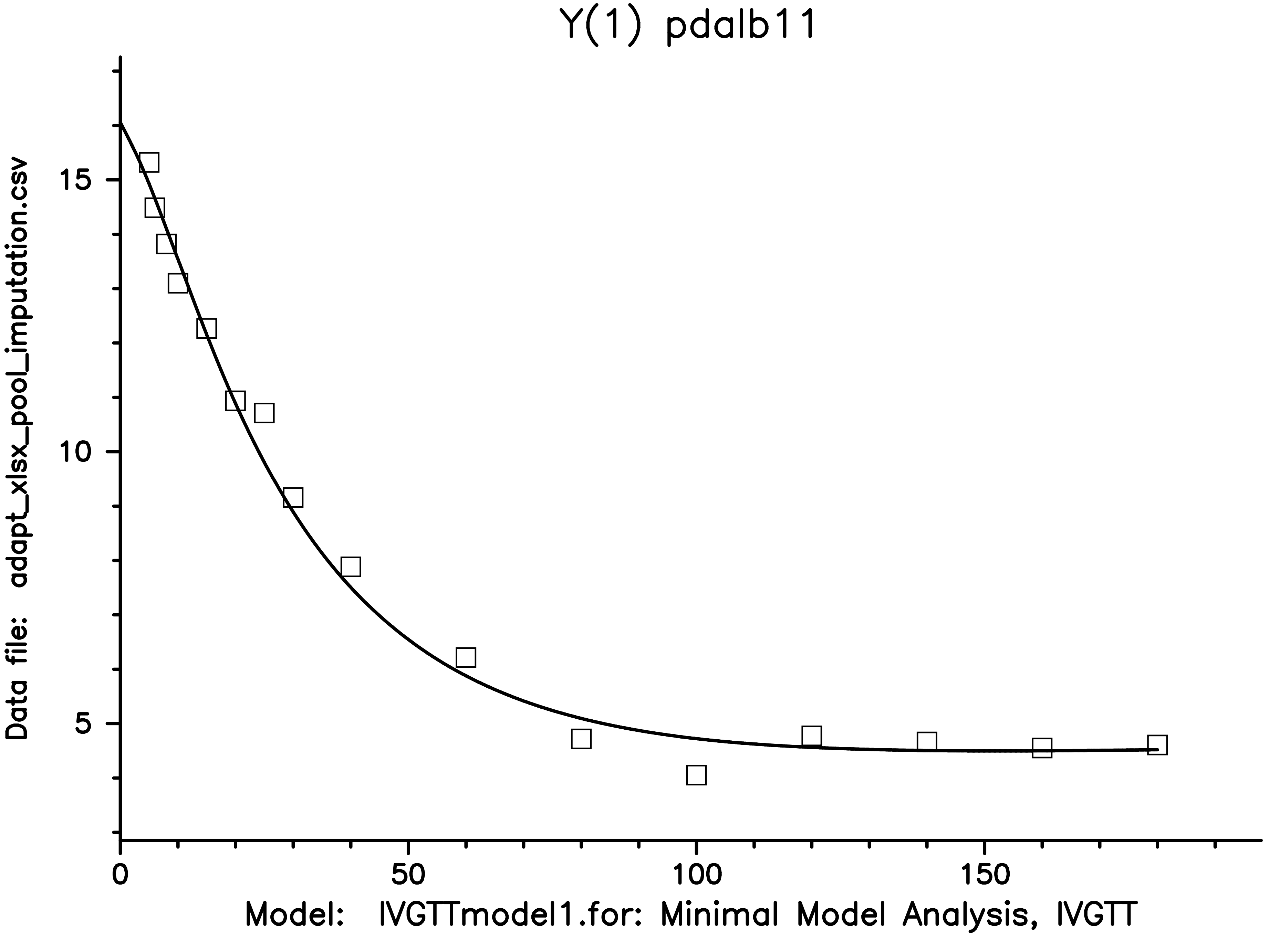

Y(1) pdalb11
Data file: adapt_xlsx_pool_imputation.csv
Model: IVGTTmodel1.for: Minimal Model Analysis, IVGTT
15
10
5
0
50
100
150

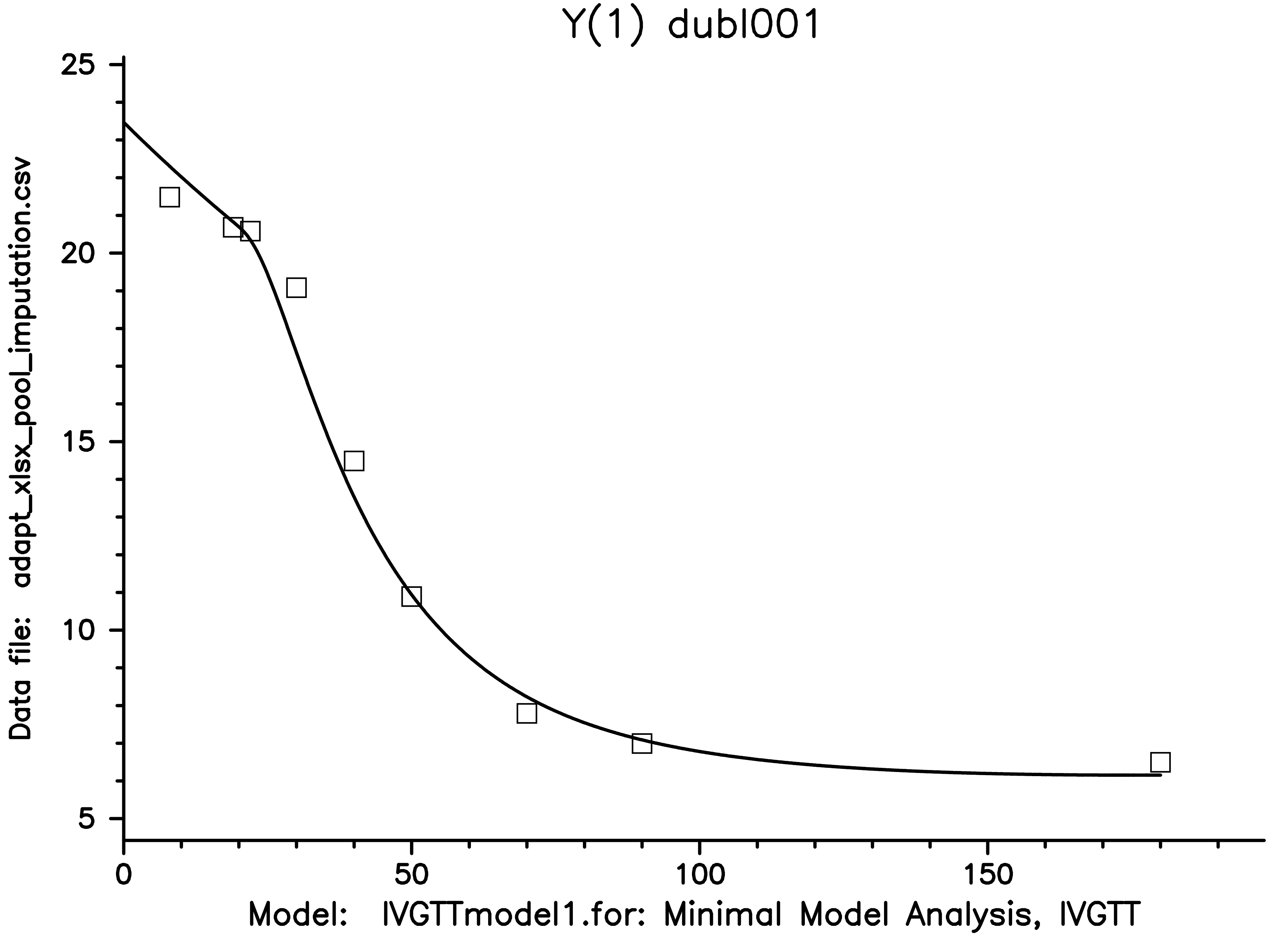
Y(1) dubl001
Data file: adapt_xlsx_pool_imputation.csv
25
20
15
10
5
0
50
100
150
Model: IVGTTmodel1.for: Minimal Model Analysis, IVGTT

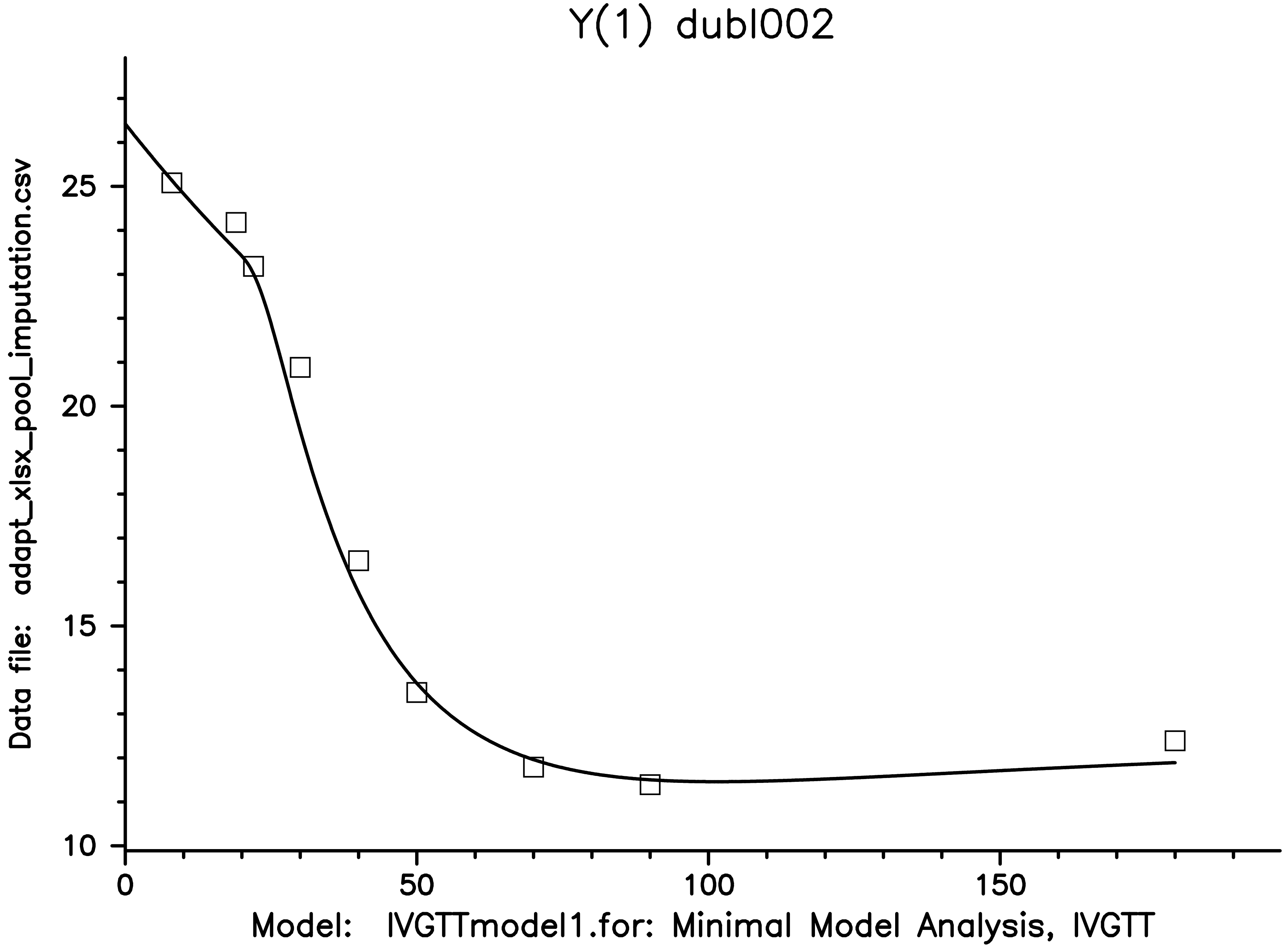
Y(1) dubl002
Data file: adapt_xlsx_pool_imputation.csv
25
20
15
10
0
50
100
150
Model: IVGTTmodel1.for: Minimal Model Analysis, IVGTT

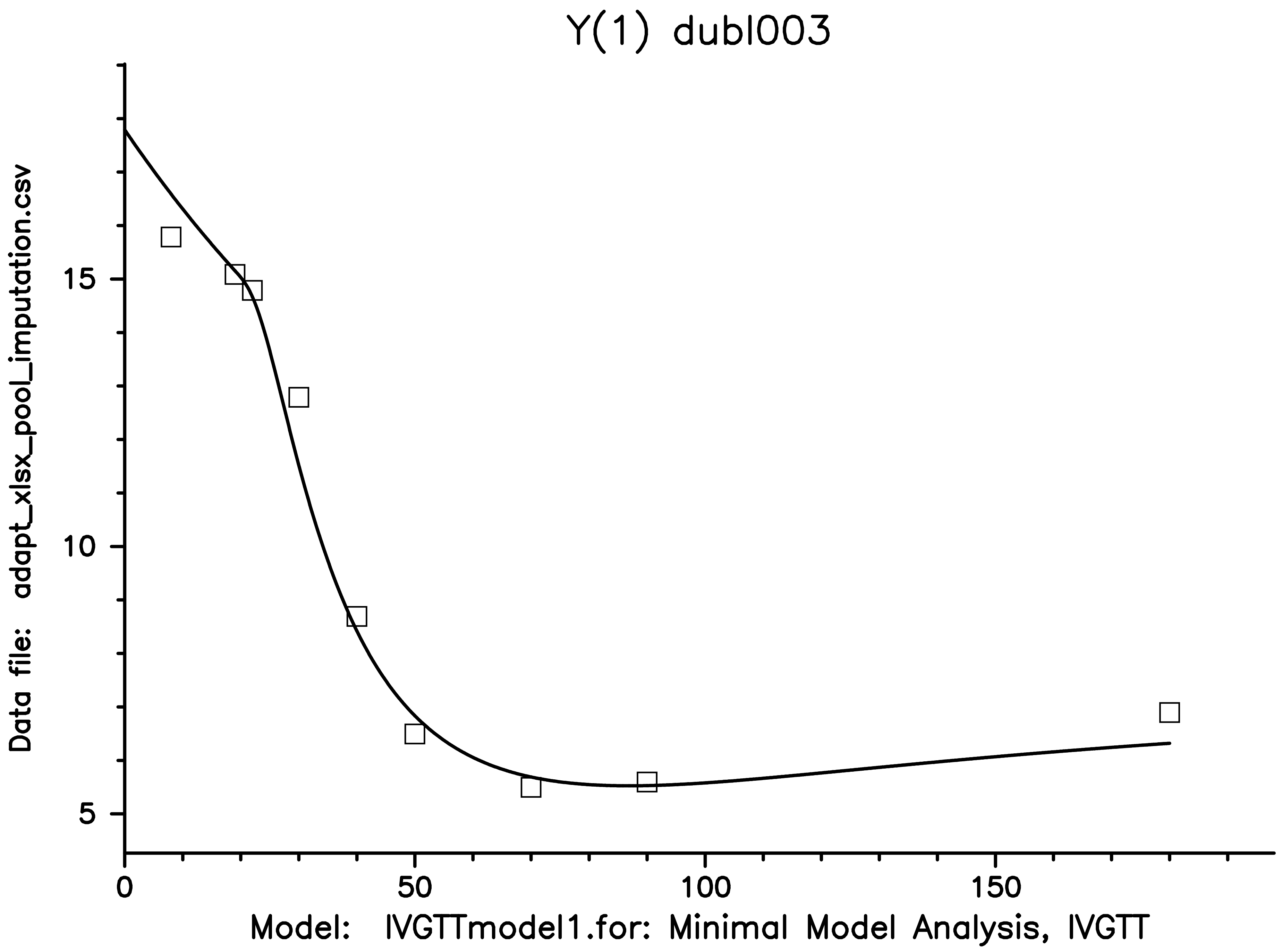

Y(1) dubl003
Data file: adapt_xlsx_pool_imputation.csv
15
10
5
0
50
100
150
Model: IVGTTmodel1.for: Minimal Model Analysis, IVGTT

Y(1) dubl004

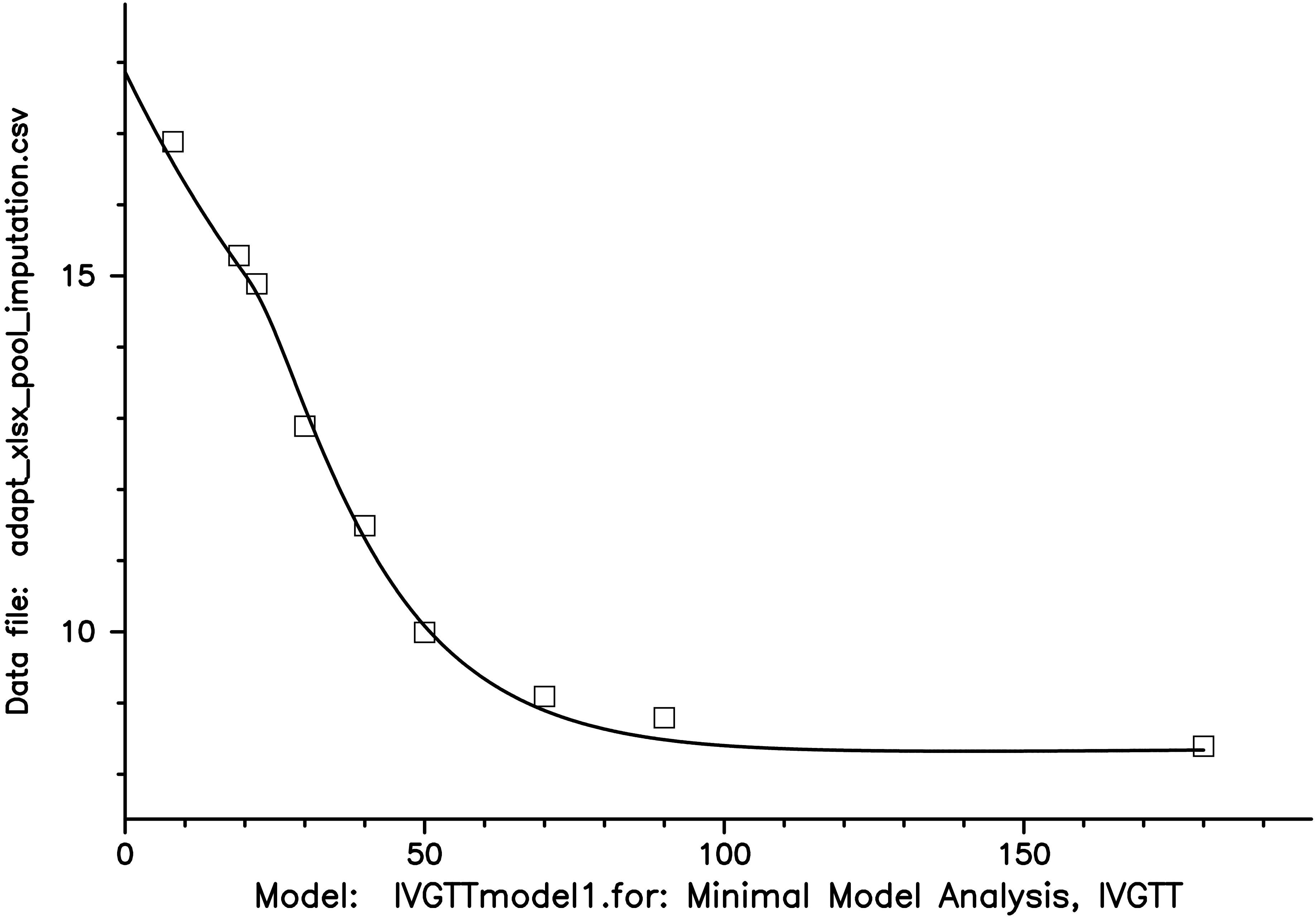

Data file: adapt_xlsx_pool_imputation.csv
15
10
0
50
100
150
Model: IVGTTmodel1.for: Minimal Model Analysis, IVGTT

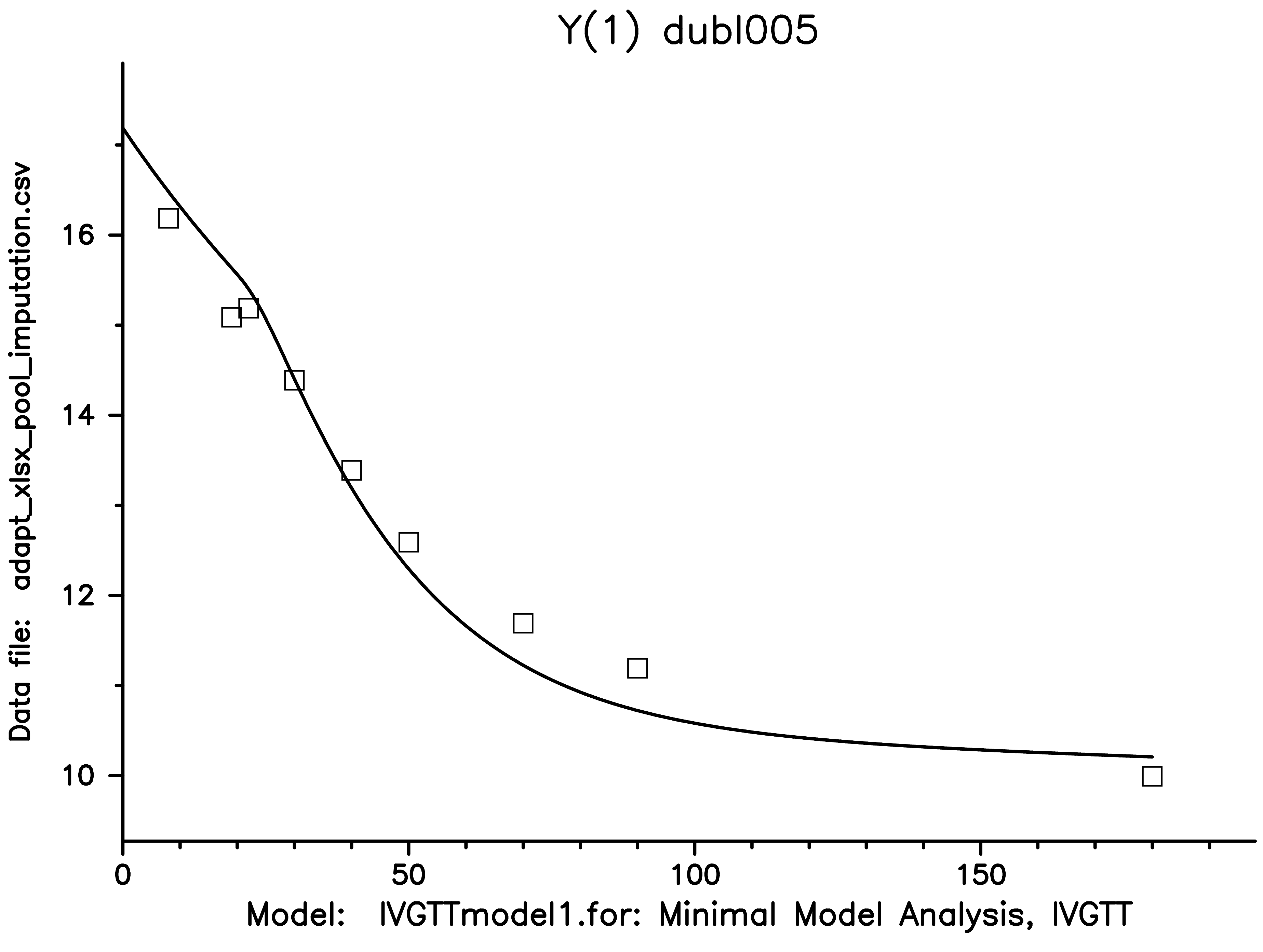
Y(1) dubl005
Data file: adapt_xlsx_pool_imputation.csv
16
14
12
10
0
50
100
150
Model: IVGTTmodel1.for: Minimal Model Analysis, IVGTT

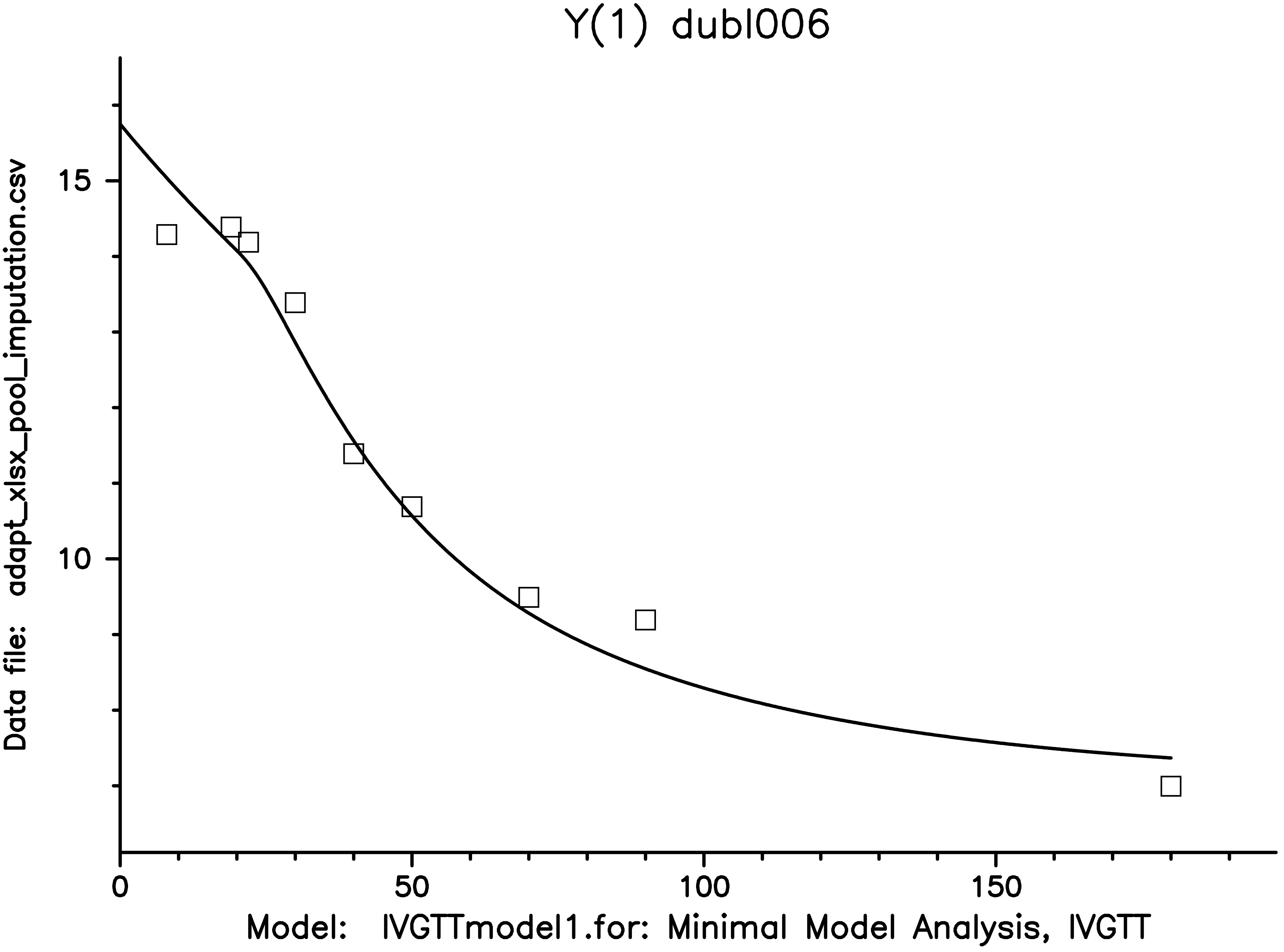
Y(1) dubl006
Data file: adapt_xlsx_pool_imputation.csv
15
10
0
50
100
150
Model: IVGTTmodel1.for: Minimal Model Analysis, IVGTT

Y(1) dubl007

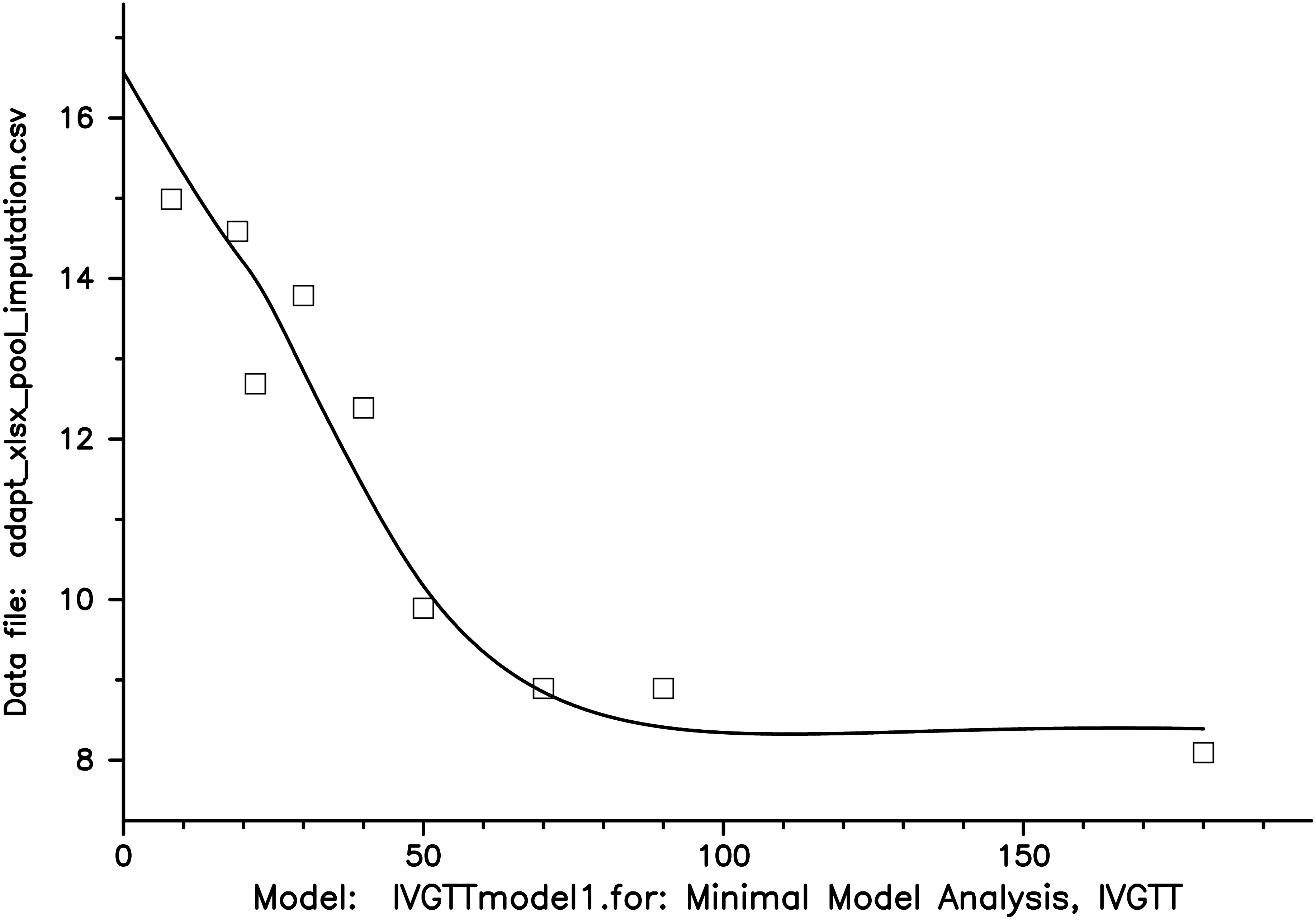
16
14
12
10
8
0
50
100
150
Data file: adapt_xlsx_pool_imputation.csv
Model: IVGTTmodel1.for: Minimal Model Analysis, IVGTT

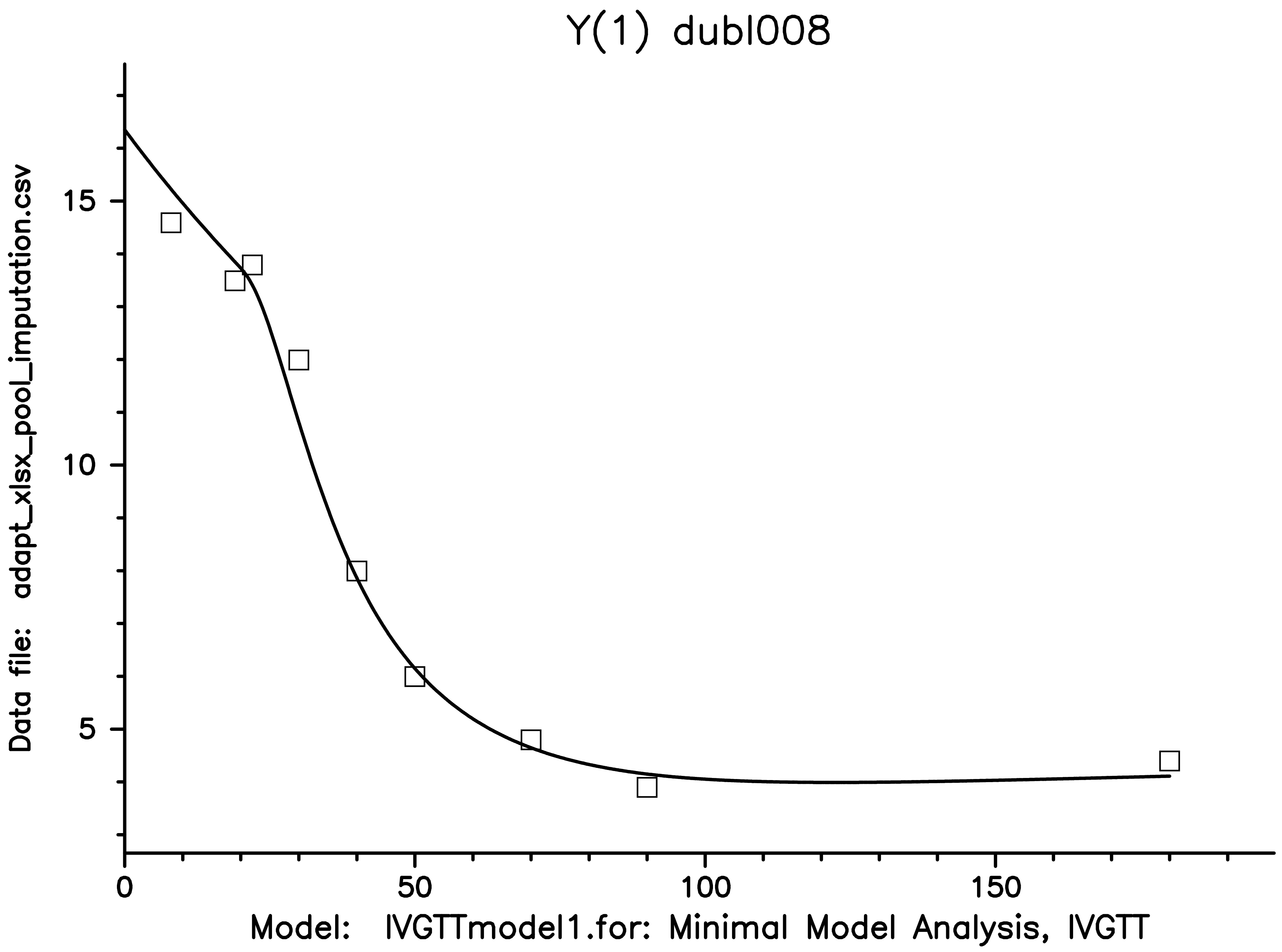

Y(1) dubl008
Data file: adapt_xlsx_pool_imputation.csv
Model: IVGTTmodel1.for: Minimal Model Analysis, IVGTT
15
10
5
0
50
100
150

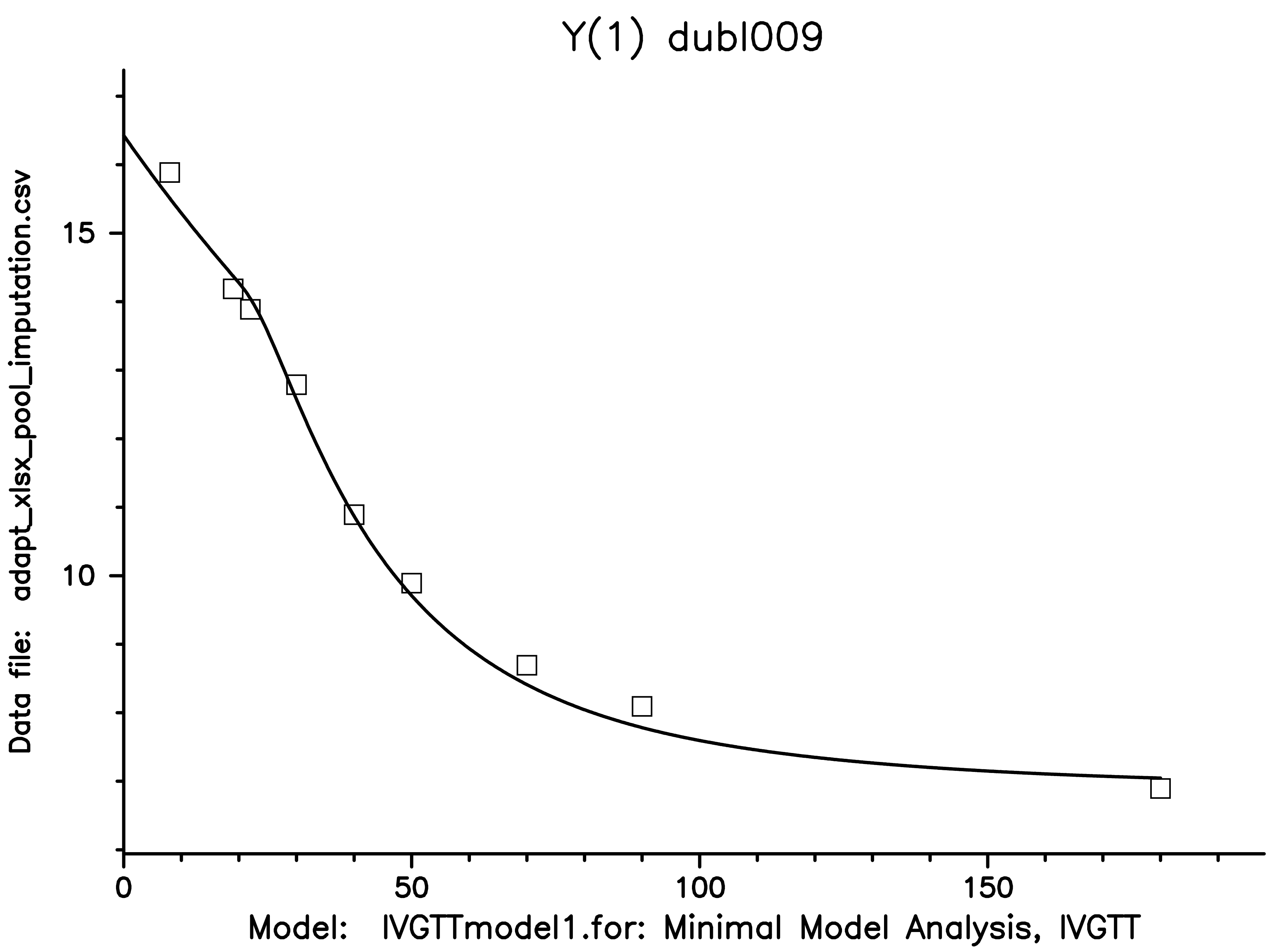

Y(1) dubl009
Data file: adapt_xlsx_pool_imputation.csv
15
10
0
50
100
150
Model: IVGTTmodel1.for: Minimal Model Analysis, IVGTT

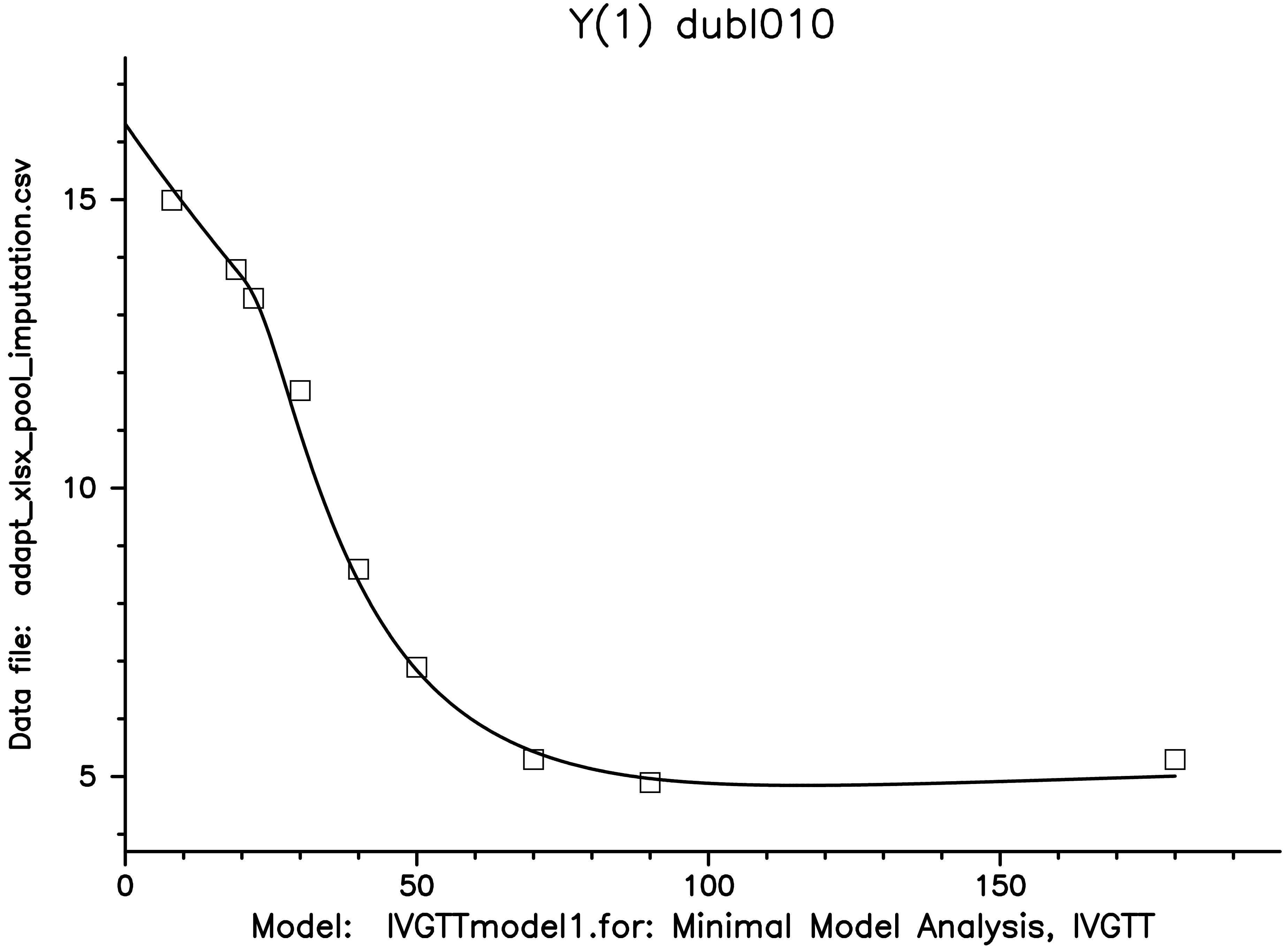

Y(1) dubl010
Data file: adapt_xlsx_pool_imputation.csv
15
10
5
0
50
100
150
Model: IVGTTmodel1.for: Minimal Model Analysis, IVGTT

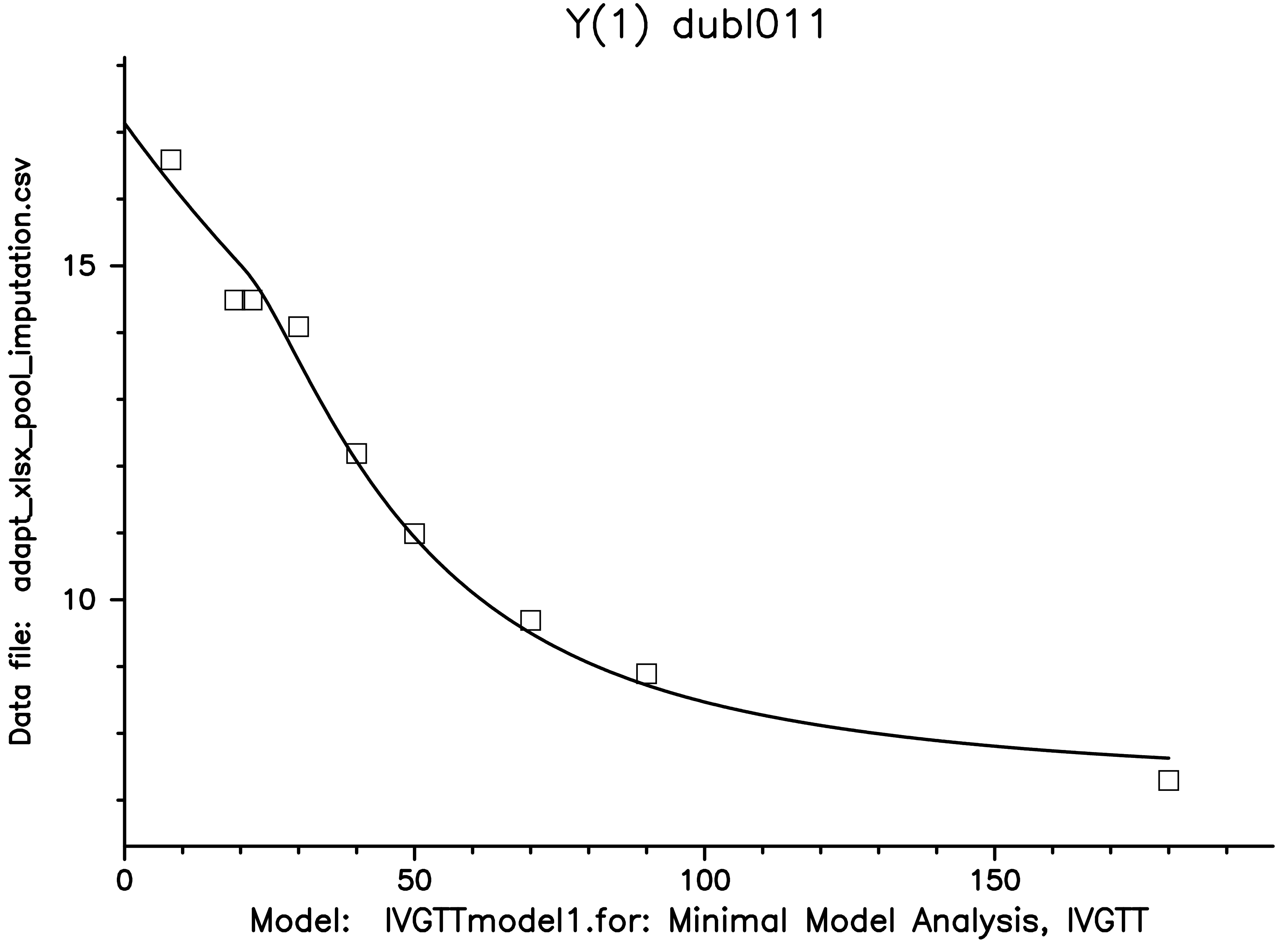

Y(1) dubl011
Data file: adapt_xlsx_pool_imputation.csv
15
10
0
50
100
150
Model: IVGTTmodel1.for: Minimal Model Analysis, IVGTT

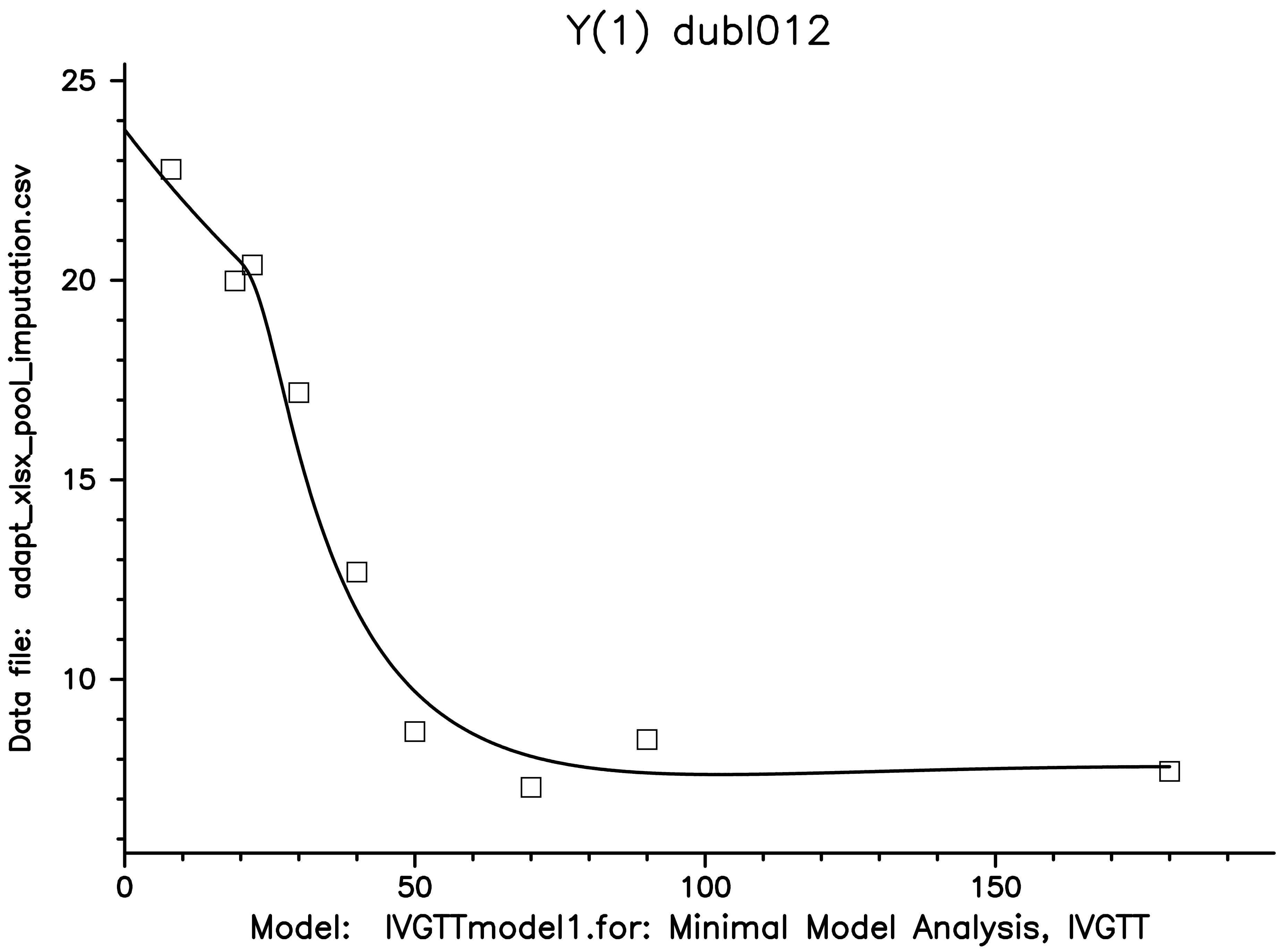

Y(1) dubl012
Data file: adapt_xlsx_pool_imputation.csv
Model: IVGTTmodel1.for: Minimal Model Analysis, IVGTT
25
20
15
10
0
50
100
150

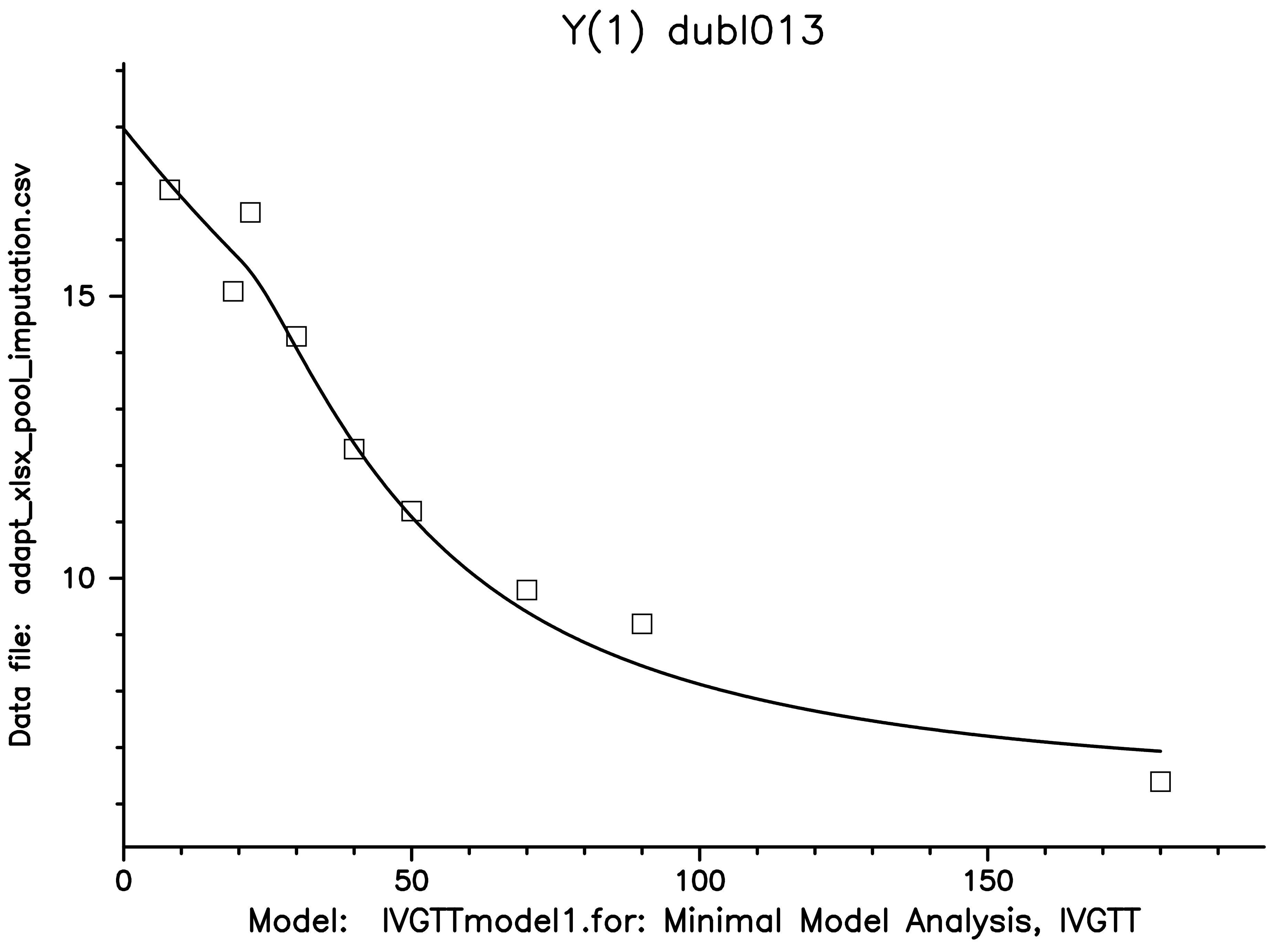

Y(1) dubl013
Data file: adapt_xlsx_pool_imputation.csv
Model: IVGTTmodel1.for: Minimal Model Analysis, IVGTT
15
10
0
50
100
150

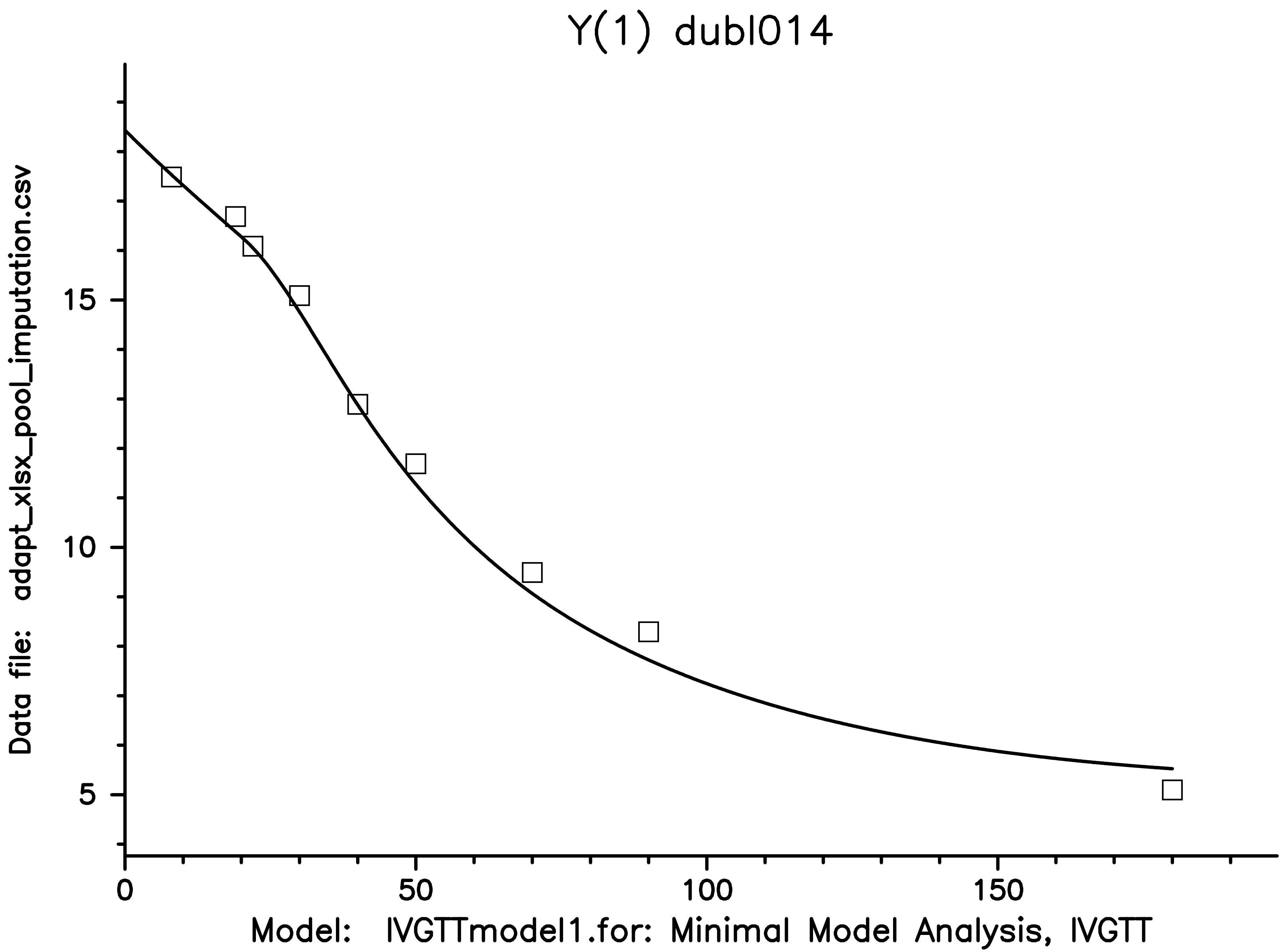

Y(1) dubl014
Data file: adapt_xlsx_pool_imputation.csv
15
10
5
0
50
100
150
Model: IVGTTmodel1.for: Minimal Model Analysis, IVGTT

Y(1) dubl015

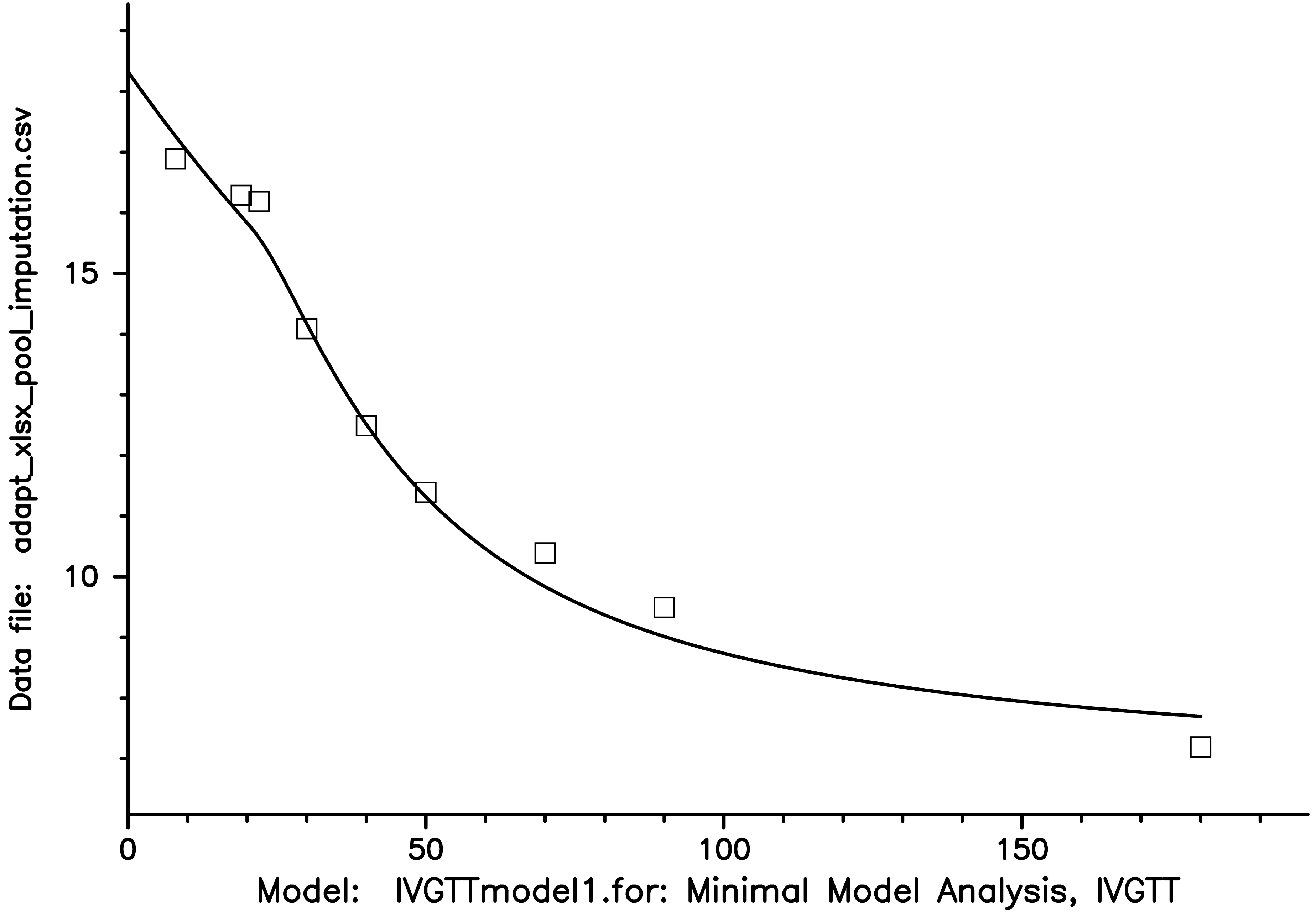
Data file: adapt_xlsx_pool_imputation.csv
15
10
0
50
100
150
Model: IVGTTmodel1.for: Minimal Model Analysis, IVGTT

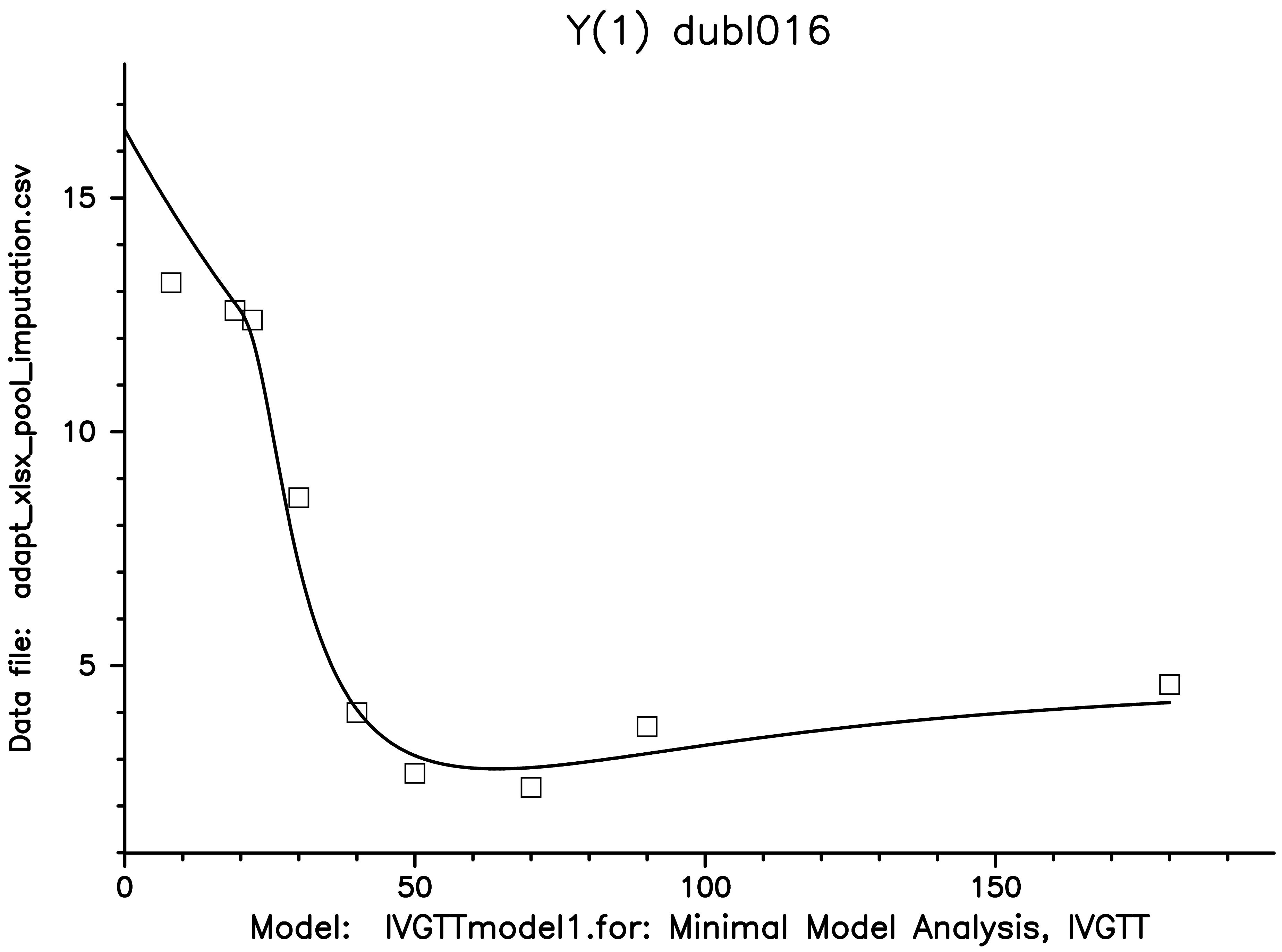
Y(1) dubl016
Data file: adapt_xlsx_pool_imputation.csv
Model: IVGTTmodel1.for: Minimal Model Analysis, IVGTT
15
10
5
0
50
100
150

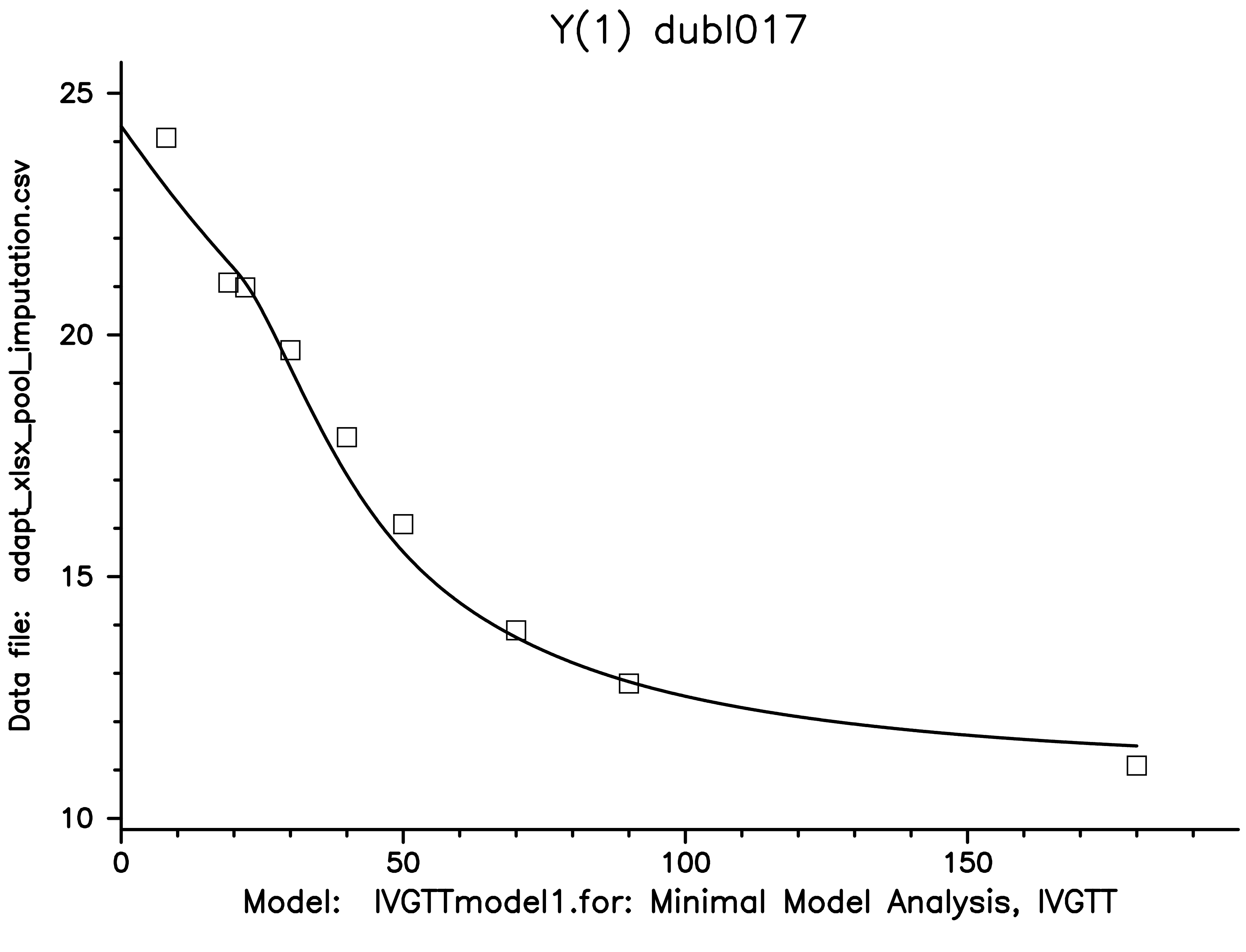

Y(1) dubl017
Data file: adapt_xlsx_pool_imputation.csv
25
20
15
10
0
50
100
150
Model: IVGTTmodel1.for: Minimal Model Analysis, IVGTT

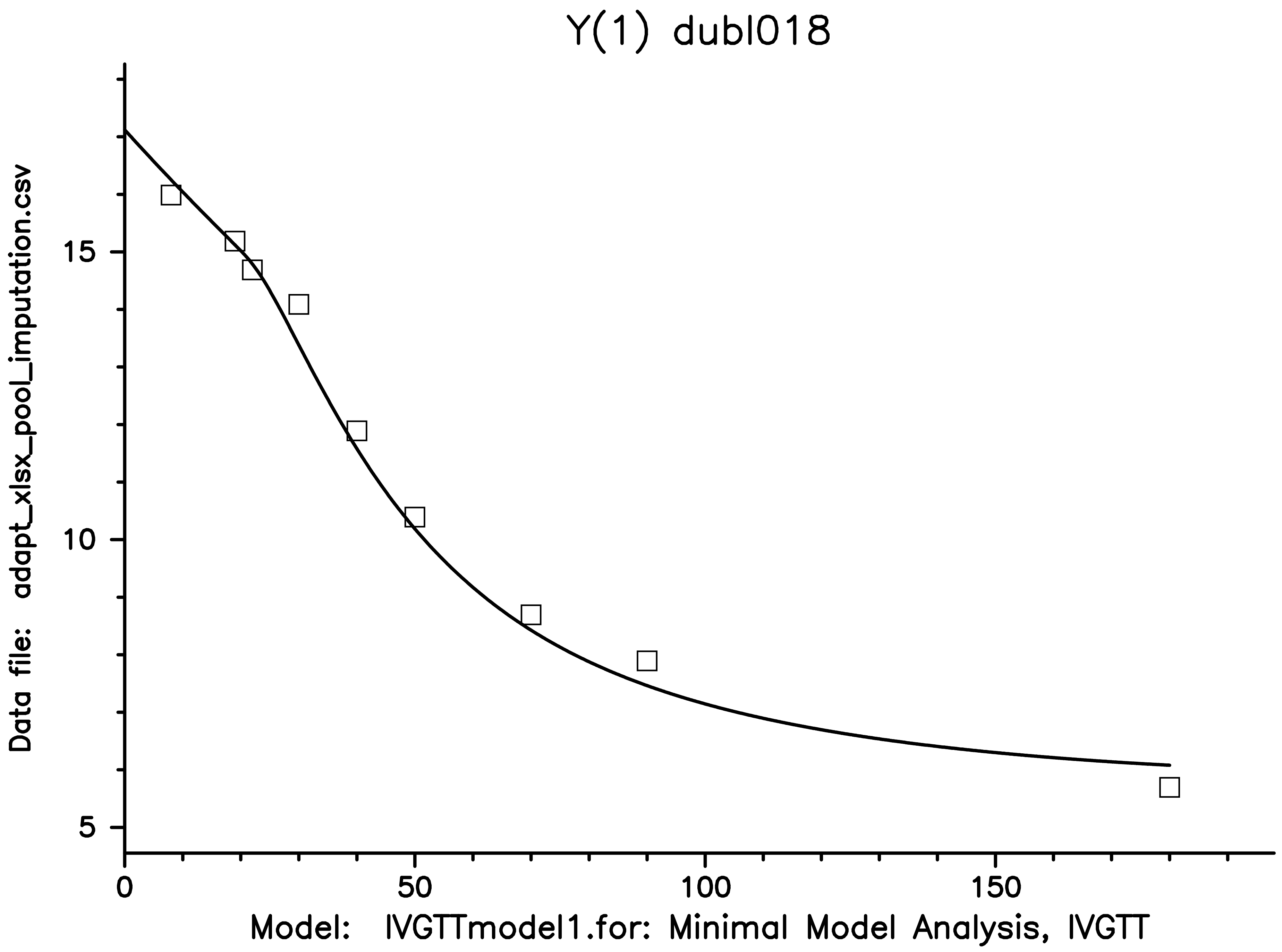
Y(1) dubl018
Data file: adapt_xlsx_pool_imputation.csv
15
10
5
0
50
100
150
Model: IVGTTmodel1.for: Minimal Model Analysis, IVGTT

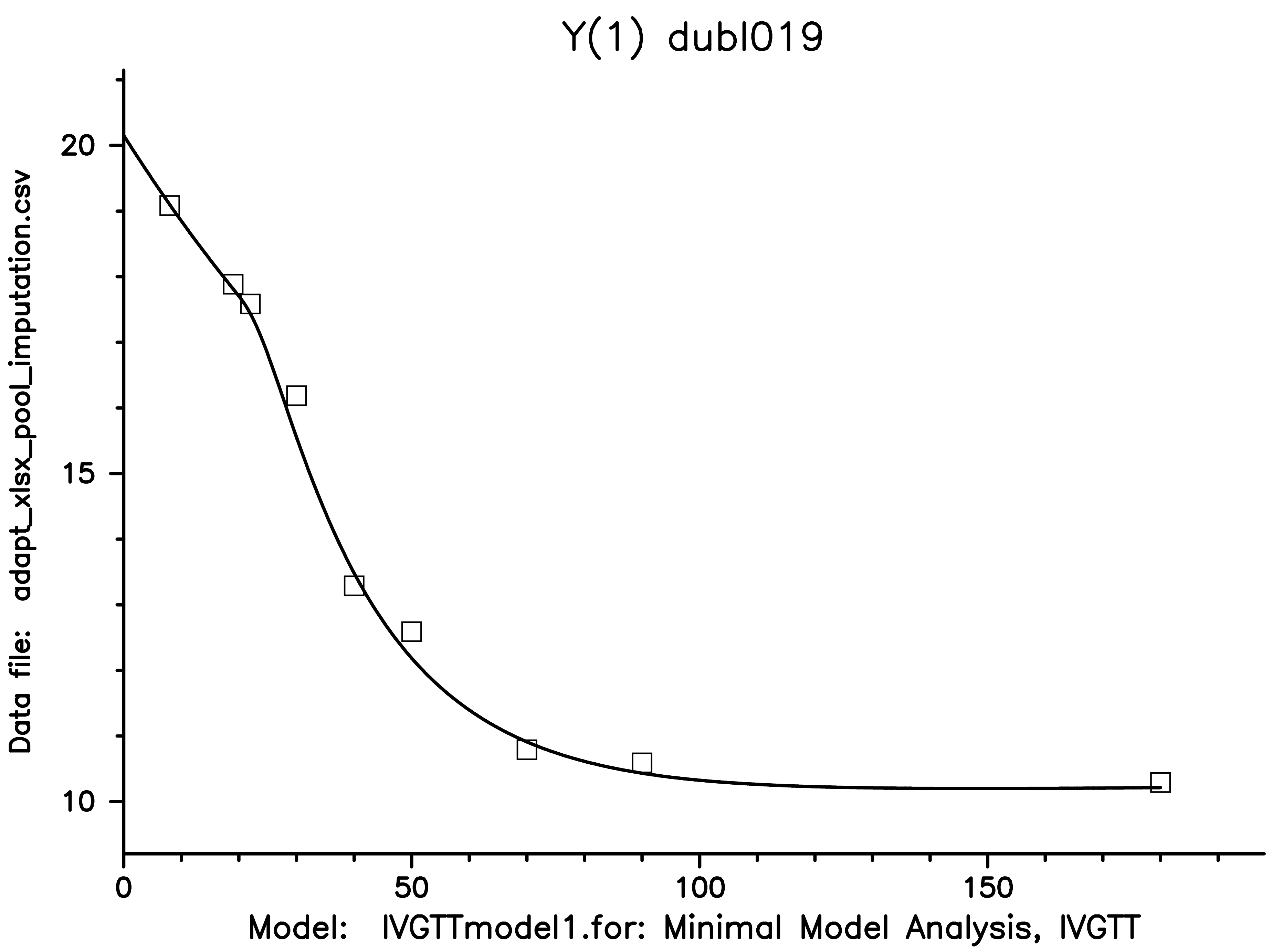
Y(1) dubl019
20
15
10
0
50
100
150
Data file: adapt_xlsx_pool_imputation.csv
Model: IVGTTmodel1.for: Minimal Model Analysis, IVGTT

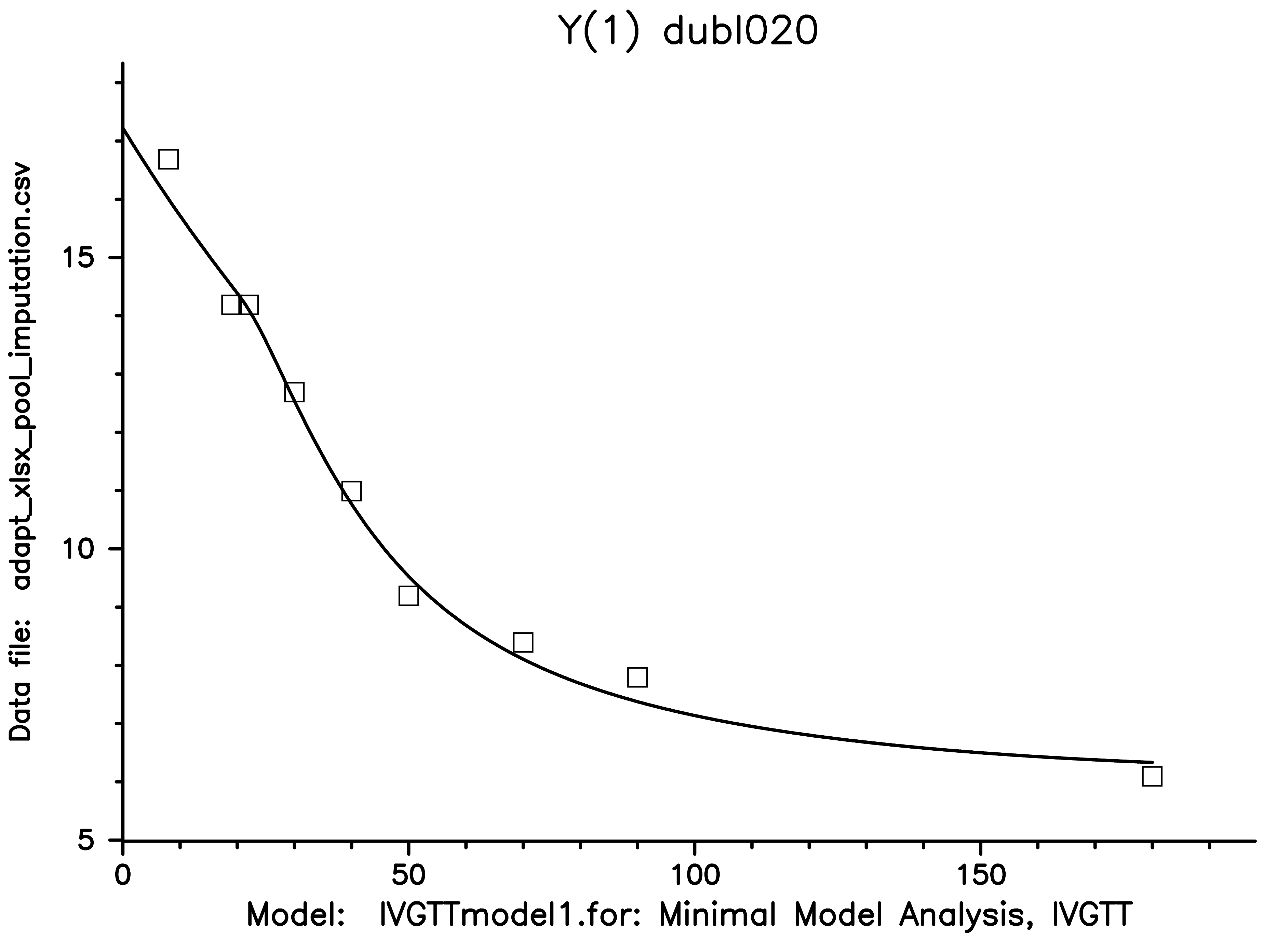

Y(1) dubl020
Data file: adapt_xlsx_pool_imputation.csv
15
10
5
0
50
100
150
Model: IVGTTmodel1.for: Minimal Model Analysis, IVGTT

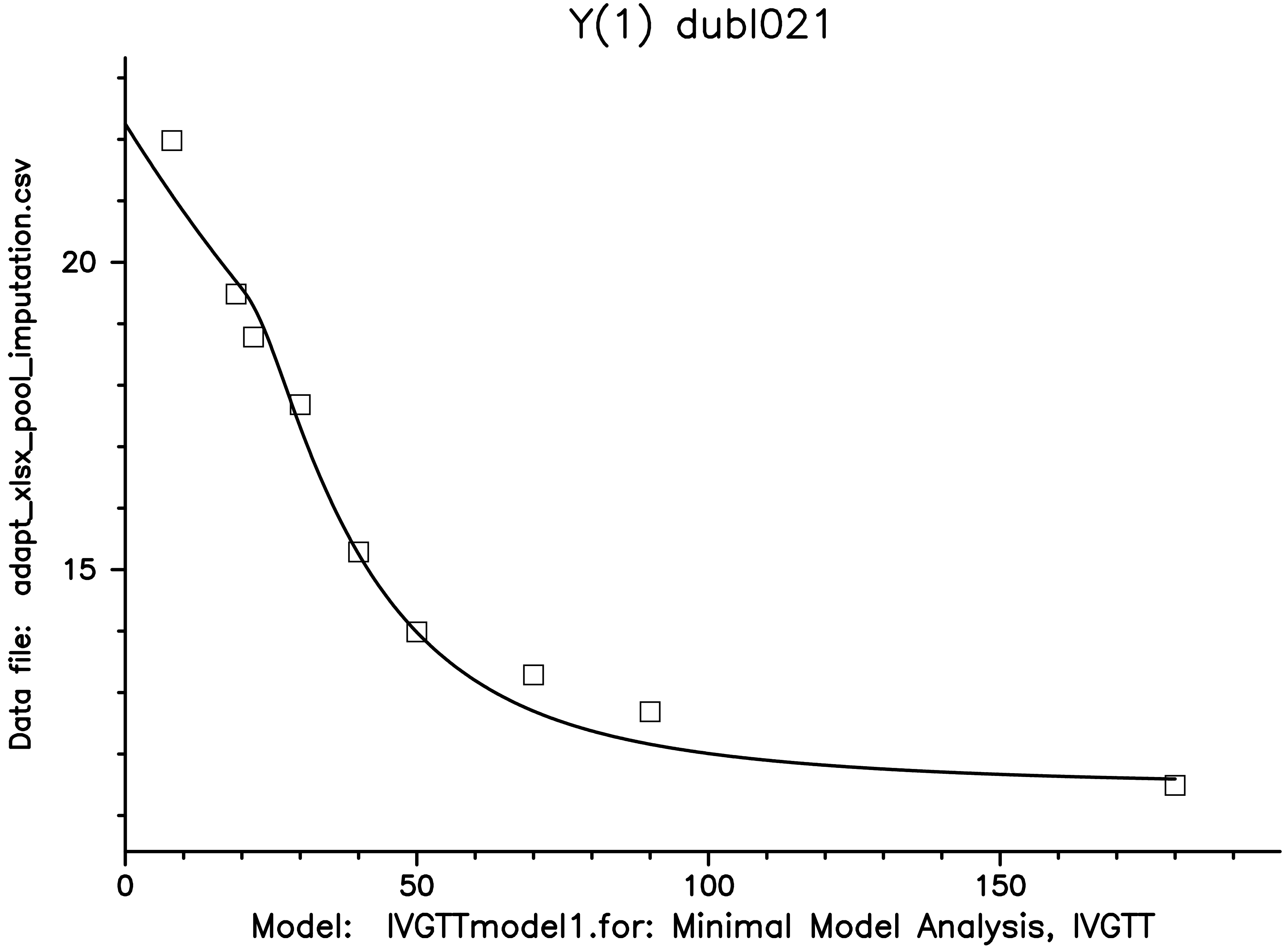
Y(1) dubl021
Data file: adapt_xlsx_pool_imputation.csv
20
15
0
50
100
150
Model: IVGTTmodel1.for: Minimal Model Analysis, IVGTT

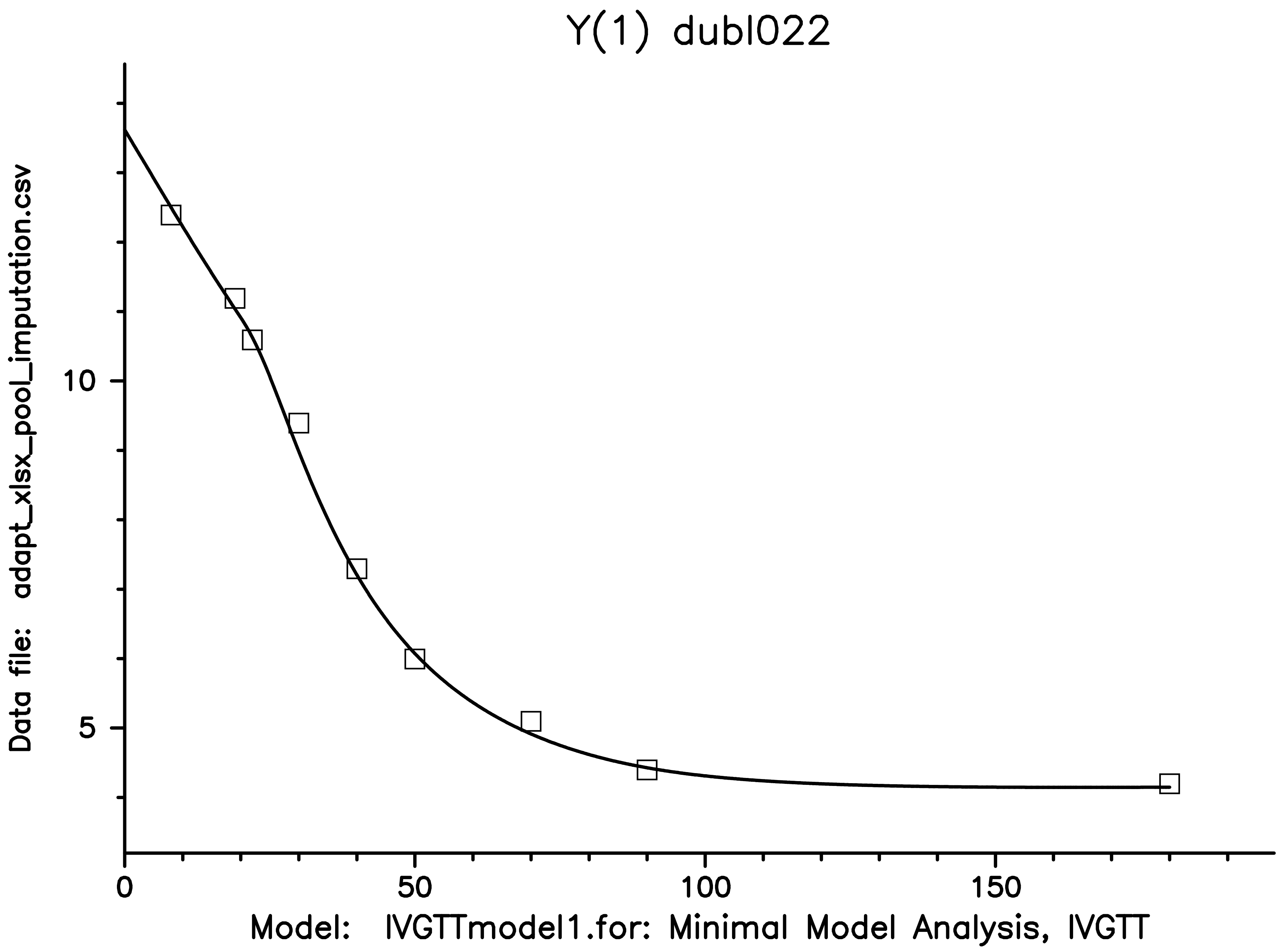

Y(1) dubl022
Data file: adapt_xlsx_pool_imputation.csv
10
5
0
50
100
150
Model: IVGTTmodel1.for: Minimal Model Analysis, IVGTT

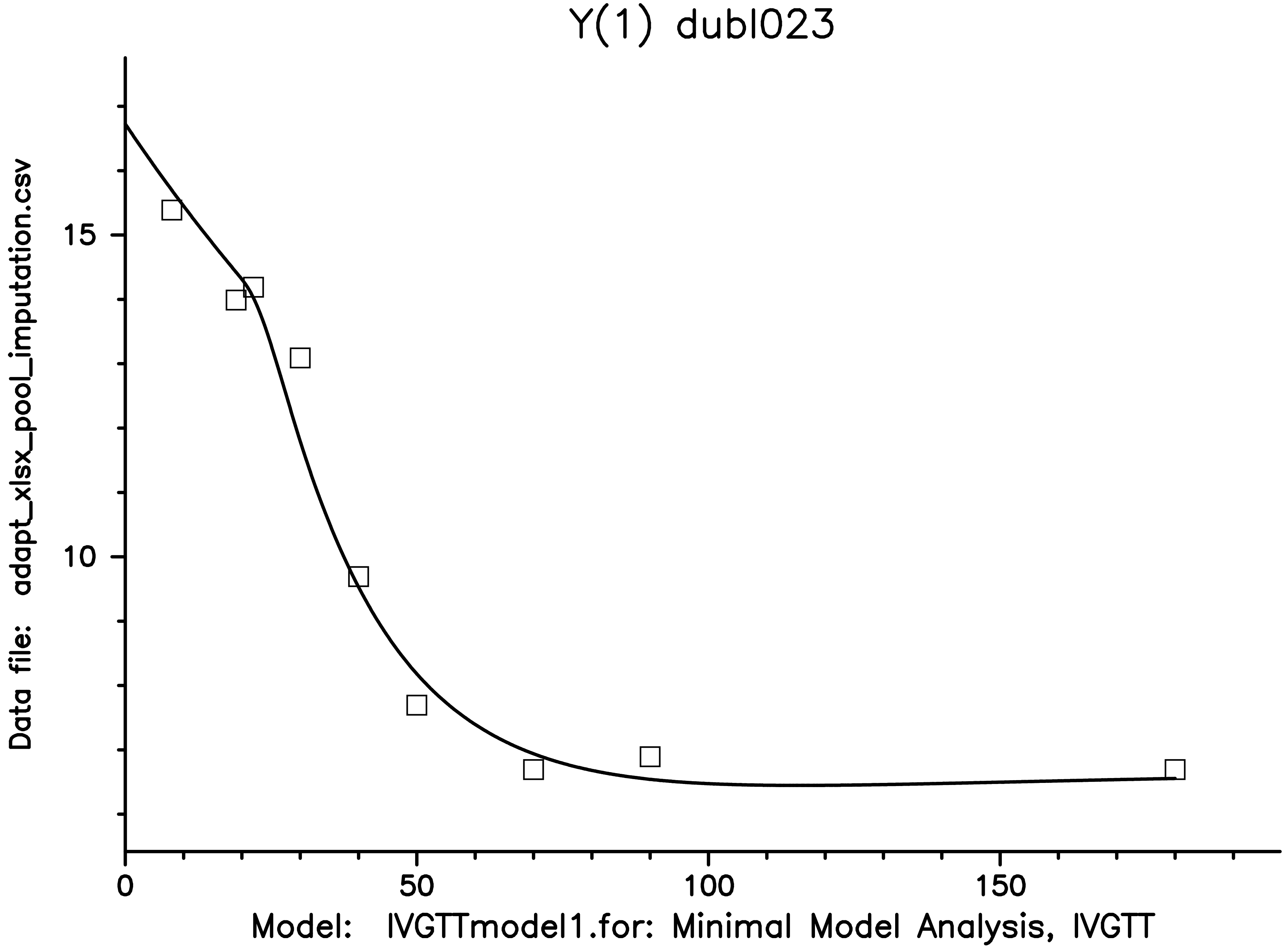
Y(1) dubl023
Data file: adapt_xlsx_pool_imputation.csv
15
10
0
50
100
150
Model: IVGTTmodel1.for: Minimal Model Analysis, IVGTT

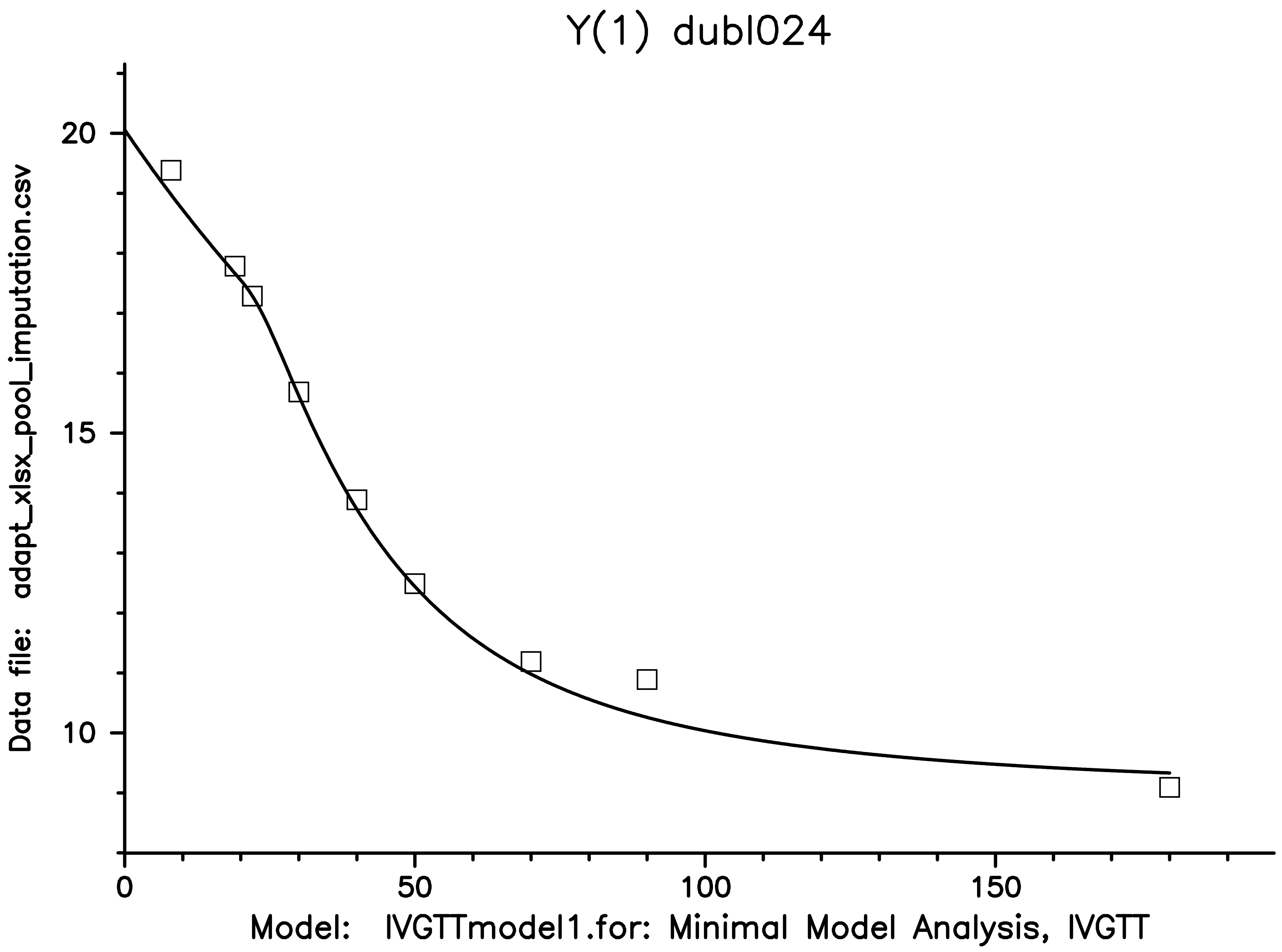
Y(1) dubl024
Data file: adapt_xlsx_pool_imputation.csv
20
15
10
0
50
100
150
Model: IVGTTmodel1.for: Minimal Model Analysis, IVGTT

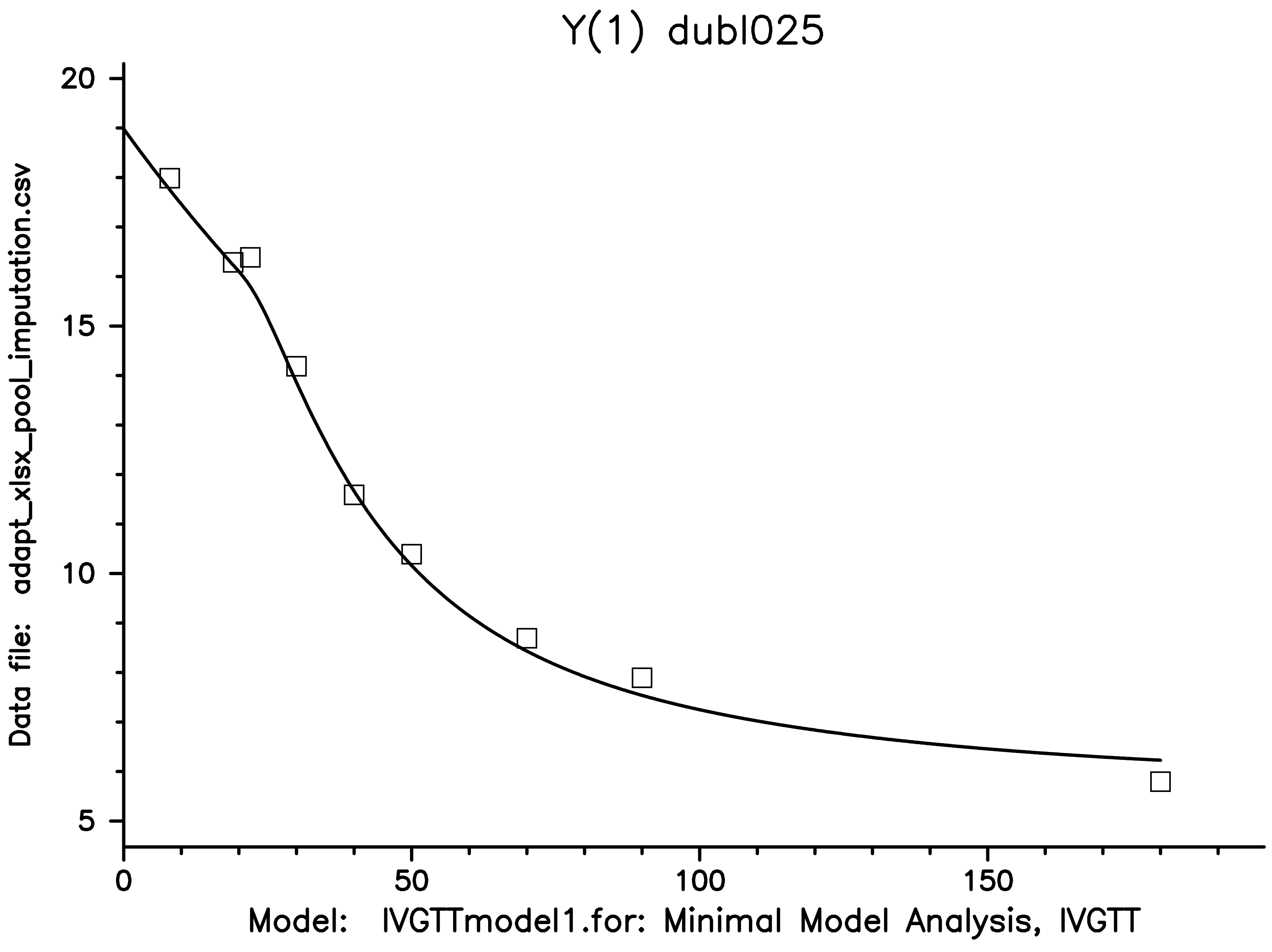
Y(1) dubl025
Data file: adapt_xlsx_pool_imputation.csv
20
15
10
5
0
50
100
150
Model: IVGTTmodel1.for: Minimal Model Analysis, IVGTT

Y(1) dubl026

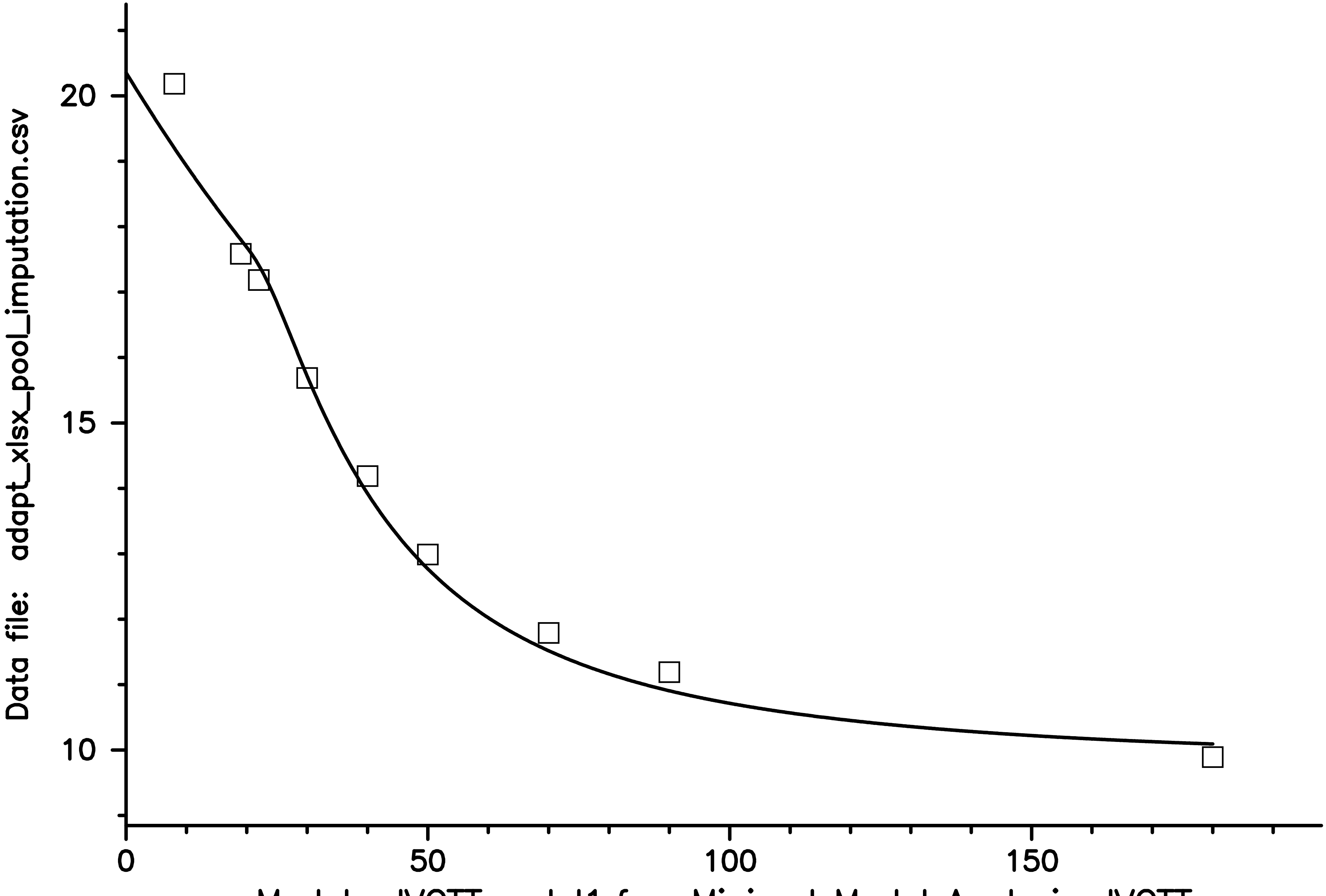

Model: IVGTTmodel1.for: Minimal Model Analysis, IVGTT

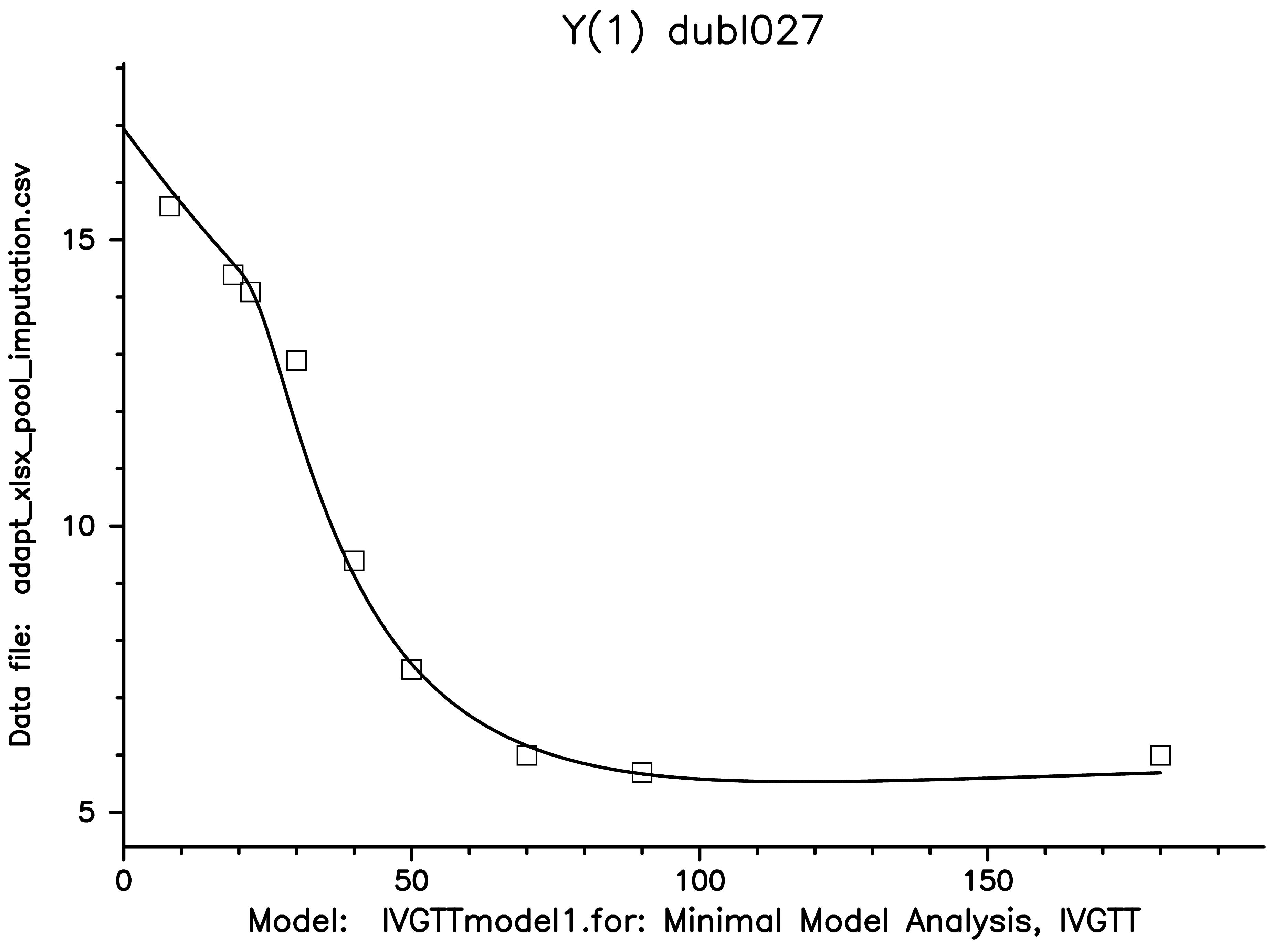

Y(1) dubl027
Data file: adapt_xlsx_pool_imputation.csv
15
10
5
0
50
100
150
Model: IVGTTmodel1.for: Minimal Model Analysis, IVGTT

Y(1) dubl028

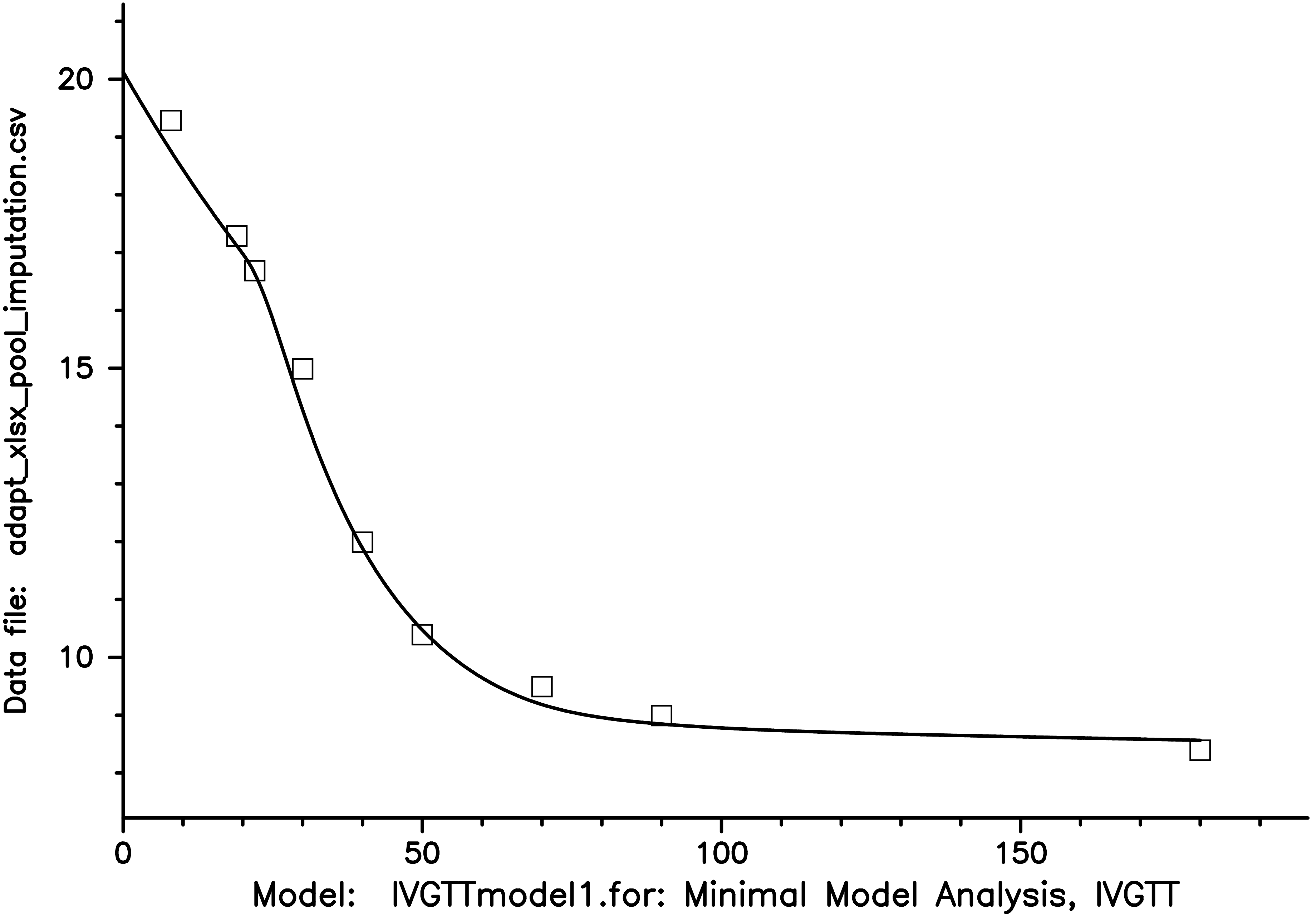

20
15
10
0
50
100
150
Data file: adapt_xlsx_pool_imputation.csv
Model: IVGTTmodel1.for: Minimal Model Analysis, IVGTT

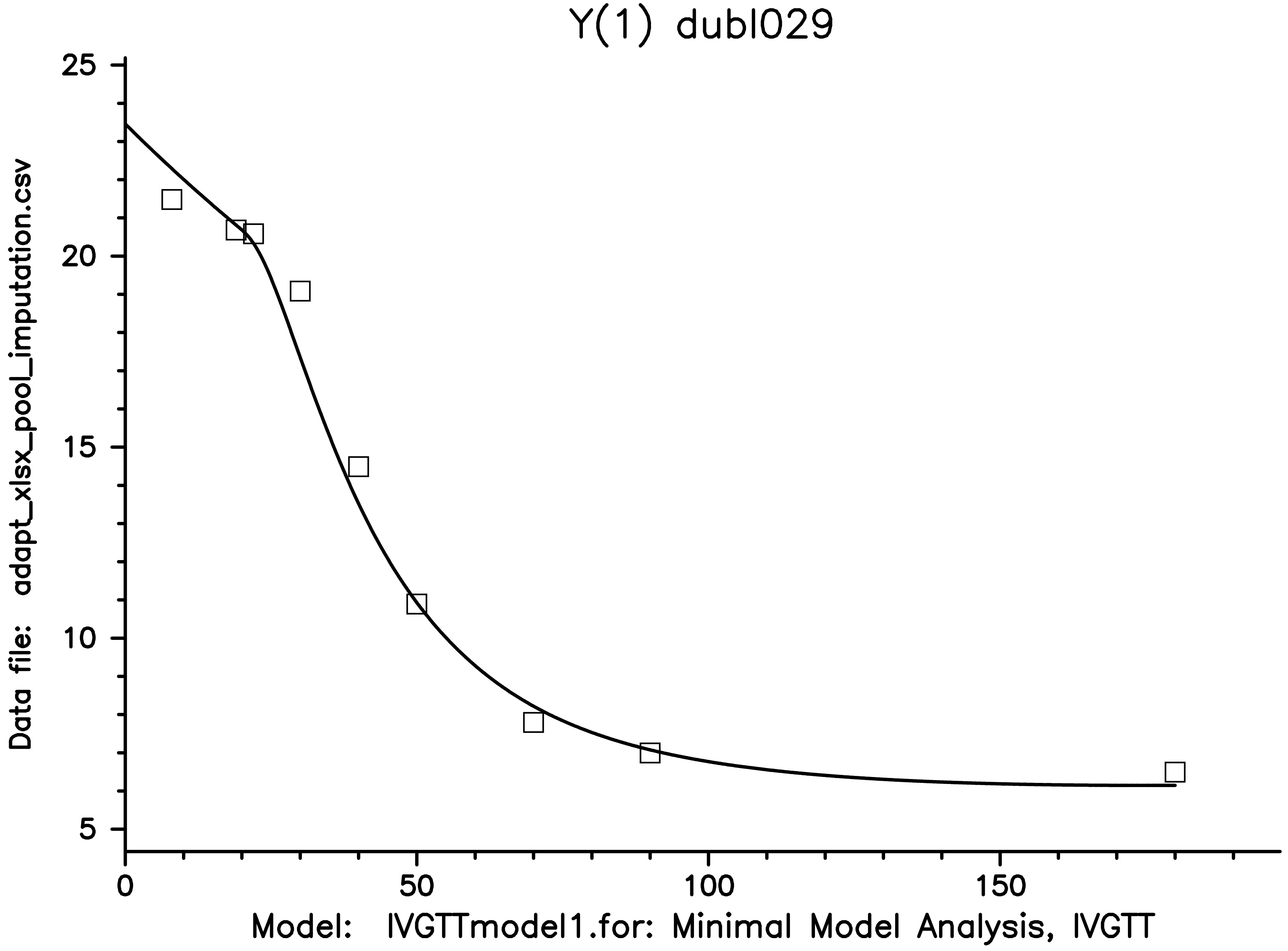
Y(1) dubl029
Data file: adapt_xlsx_pool_imputation.csv
25
20
15
10
5
0
50
100
150
Model: IVGTTmodel1.for: Minimal Model Analysis, IVGTT

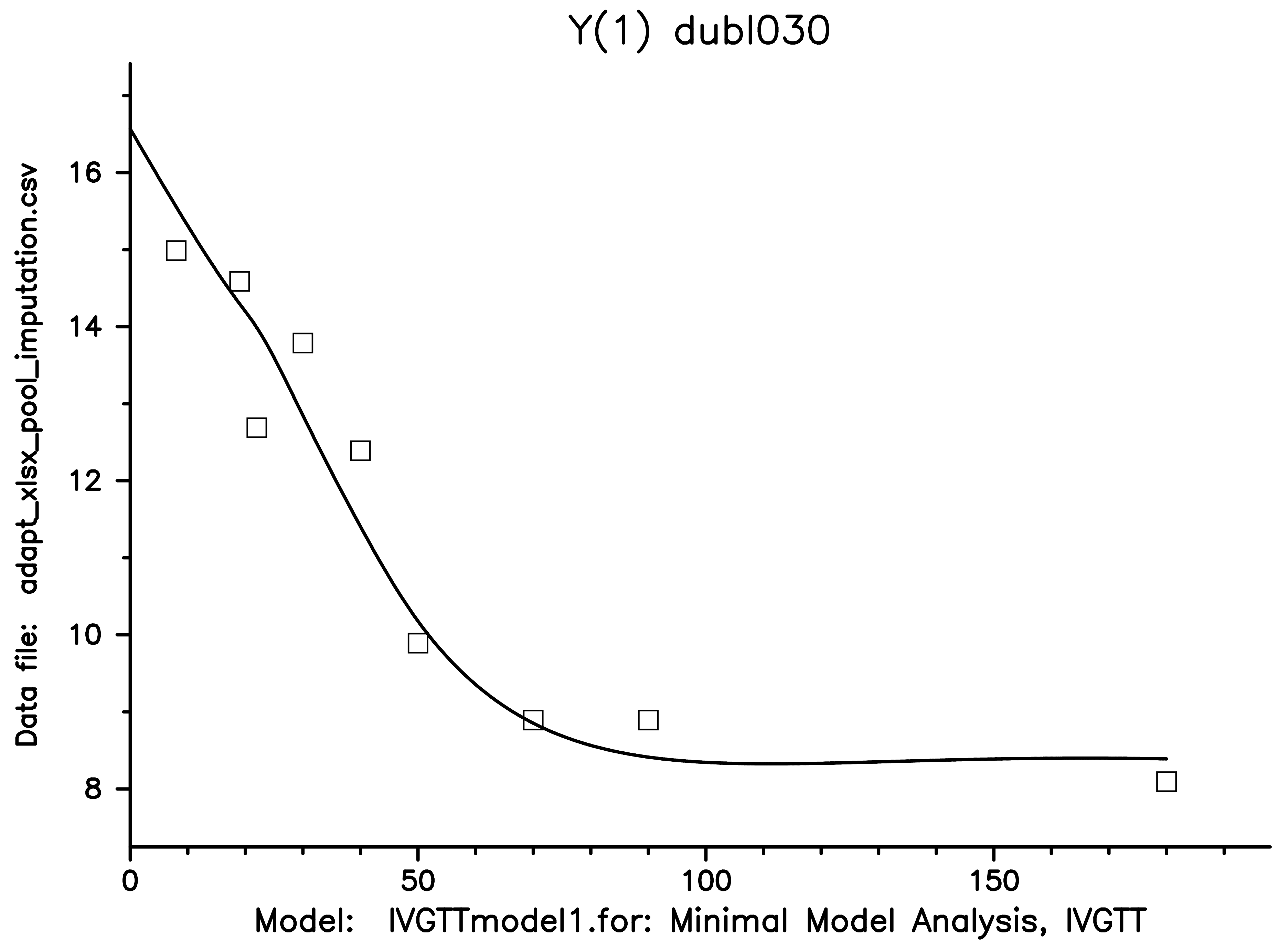
Y(1) dubl030
Data file: adapt_xlsx_pool_imputation.csv
16
14
12
10
8
0
50
100
150
Model: IVGTTmodel1.for: Minimal Model Analysis, IVGTT

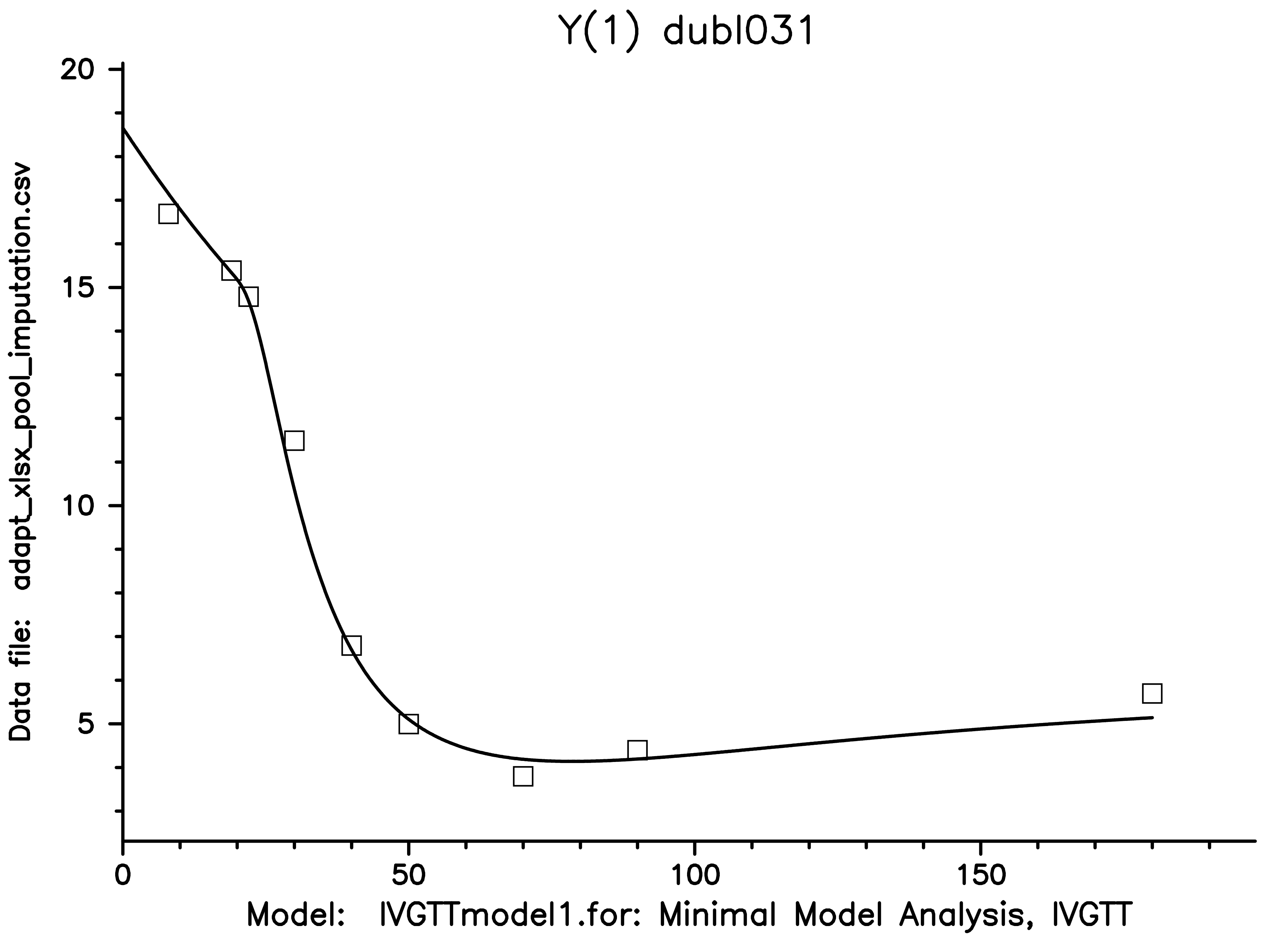

Y(1) dubl031
20
15
10
5
0
50
100
150
Data file: adapt_xlsx_pool_imputation.csv
Model: IVGTTmodel1.for: Minimal Model Analysis, IVGTT

Y(1) du1re01

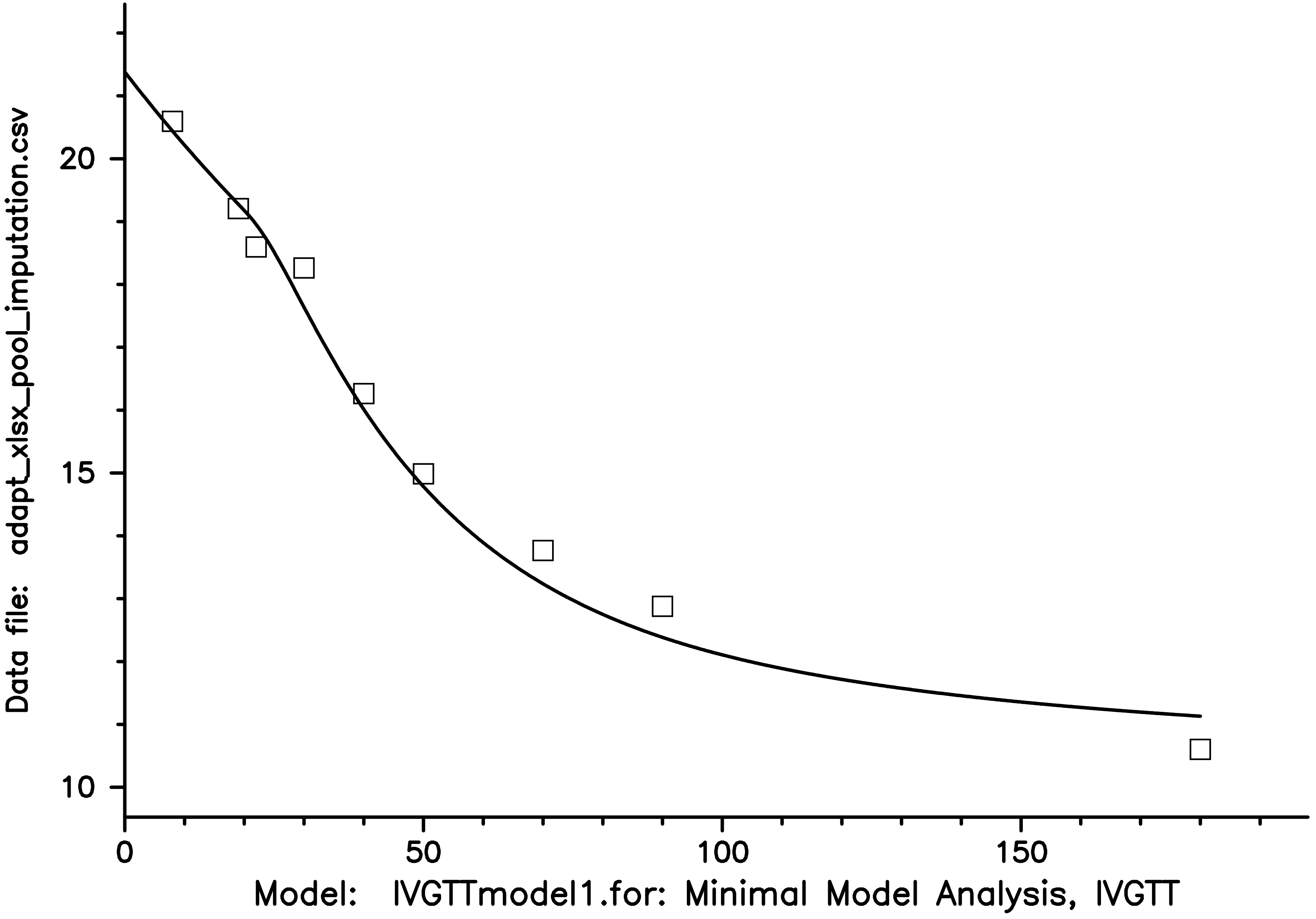

Data file: adapt_xlsx_pool_imputation.csv
20
15
10
0
50
100
150
Model: IVGTTmodel1.for: Minimal Model Analysis, IVGTT

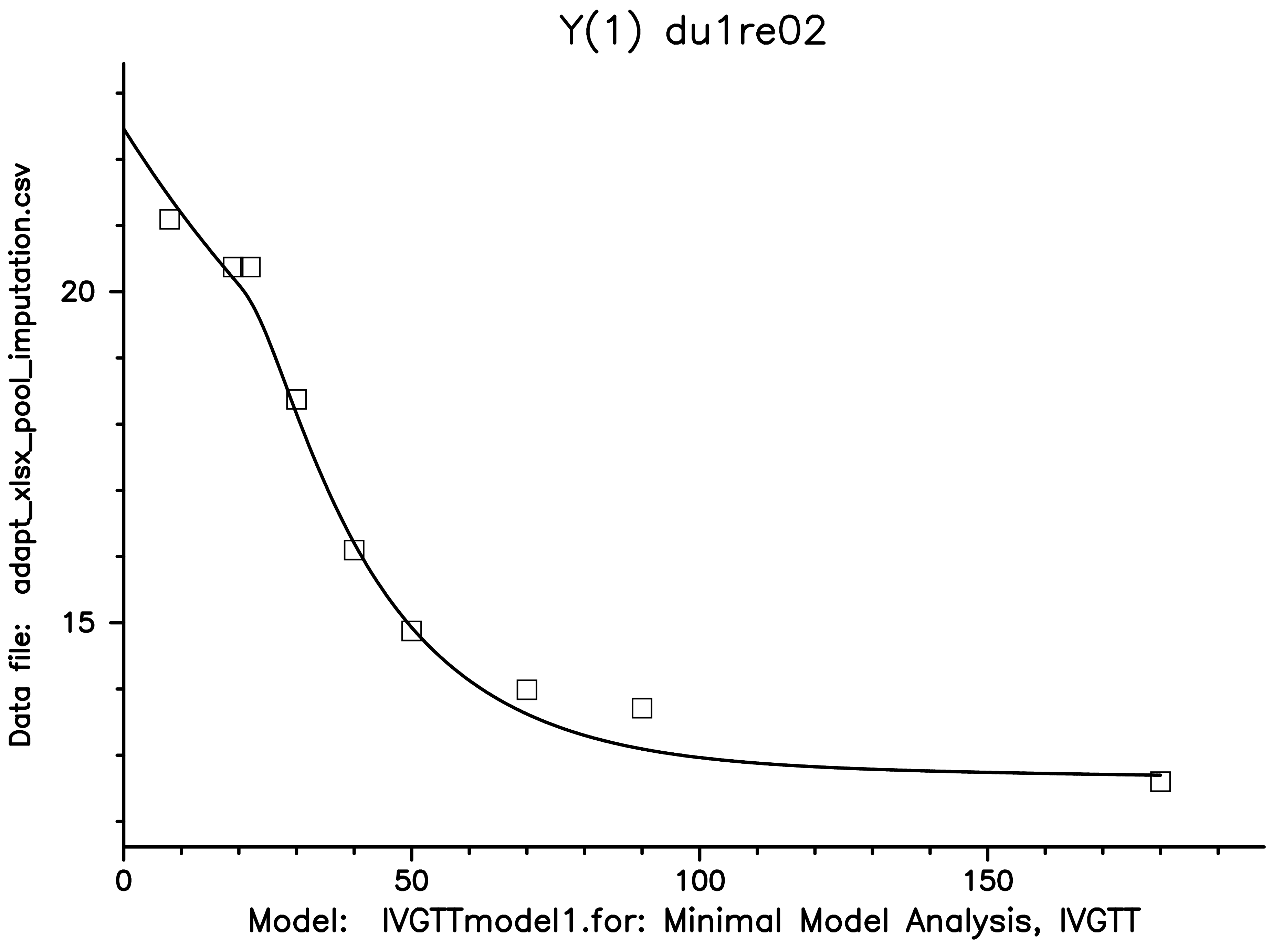
Y(1) du1re02
Data file: adapt_xlsx_pool_imputation.csv
20
15
0
50
100
150
Model: IVGTTmodel1.for: Minimal Model Analysis, IVGTT

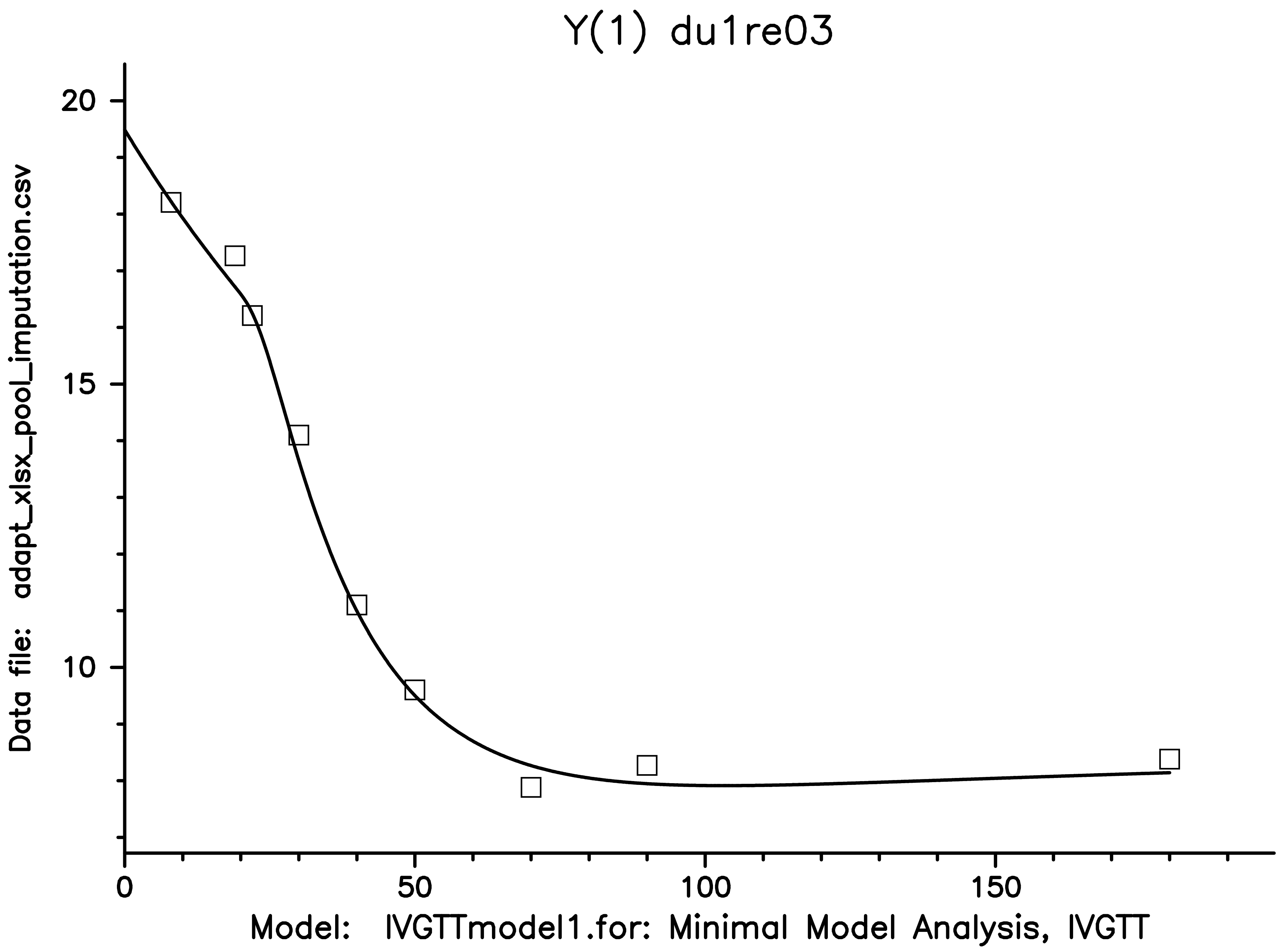

Y(1) du1re03
Data file: adapt_xlsx_pool_imputation.csv
20
15
10
0
50
100
150
Model: IVGTTmodel1.for: Minimal Model Analysis, IVGTT

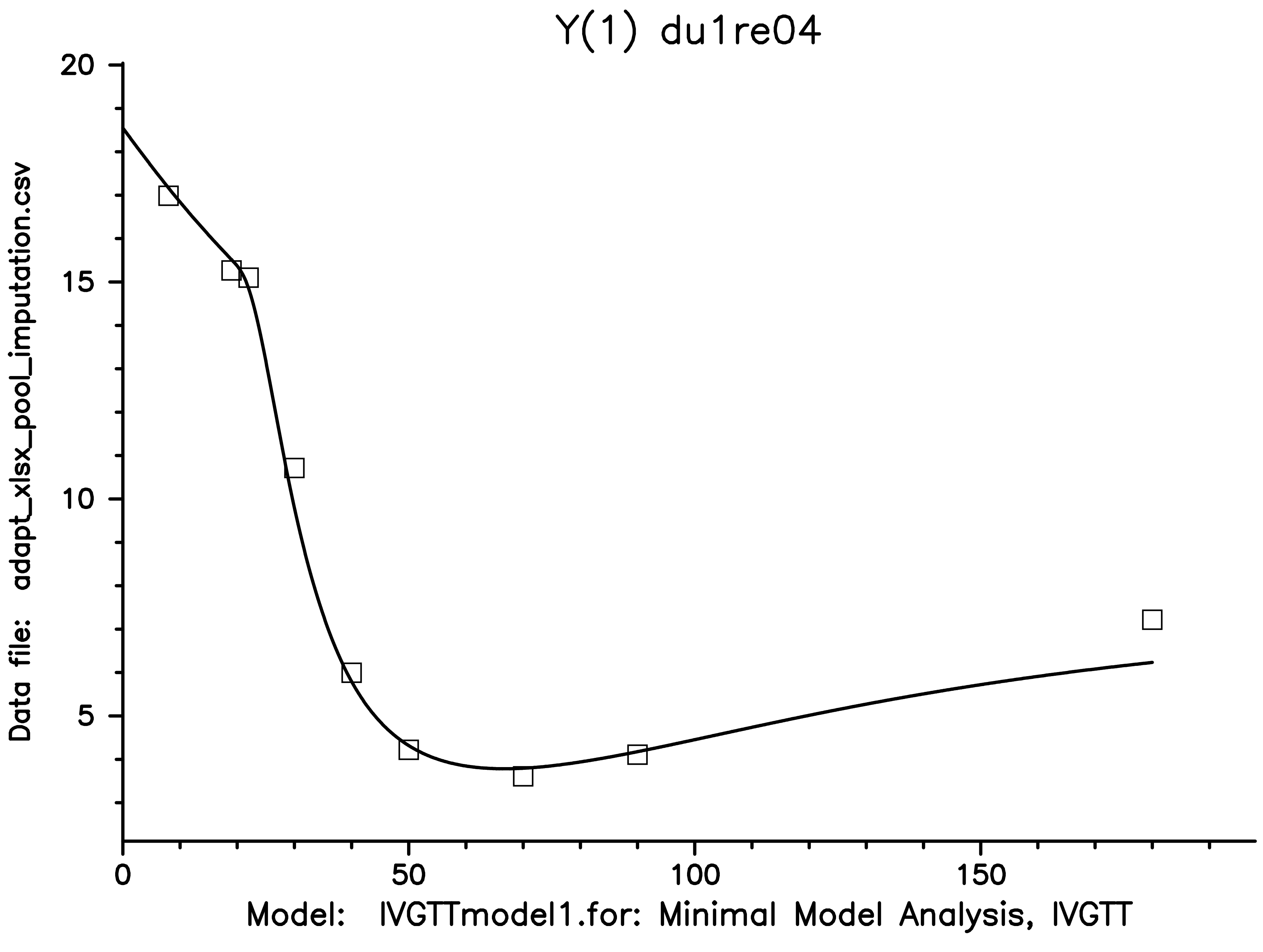
Y(1) du1re04
20
15
10
5
0
50
100
150
Data file: adapt_xlsx_pool_imputation.csv
Model: IVGTTmodel1.for: Minimal Model Analysis, IVGTT

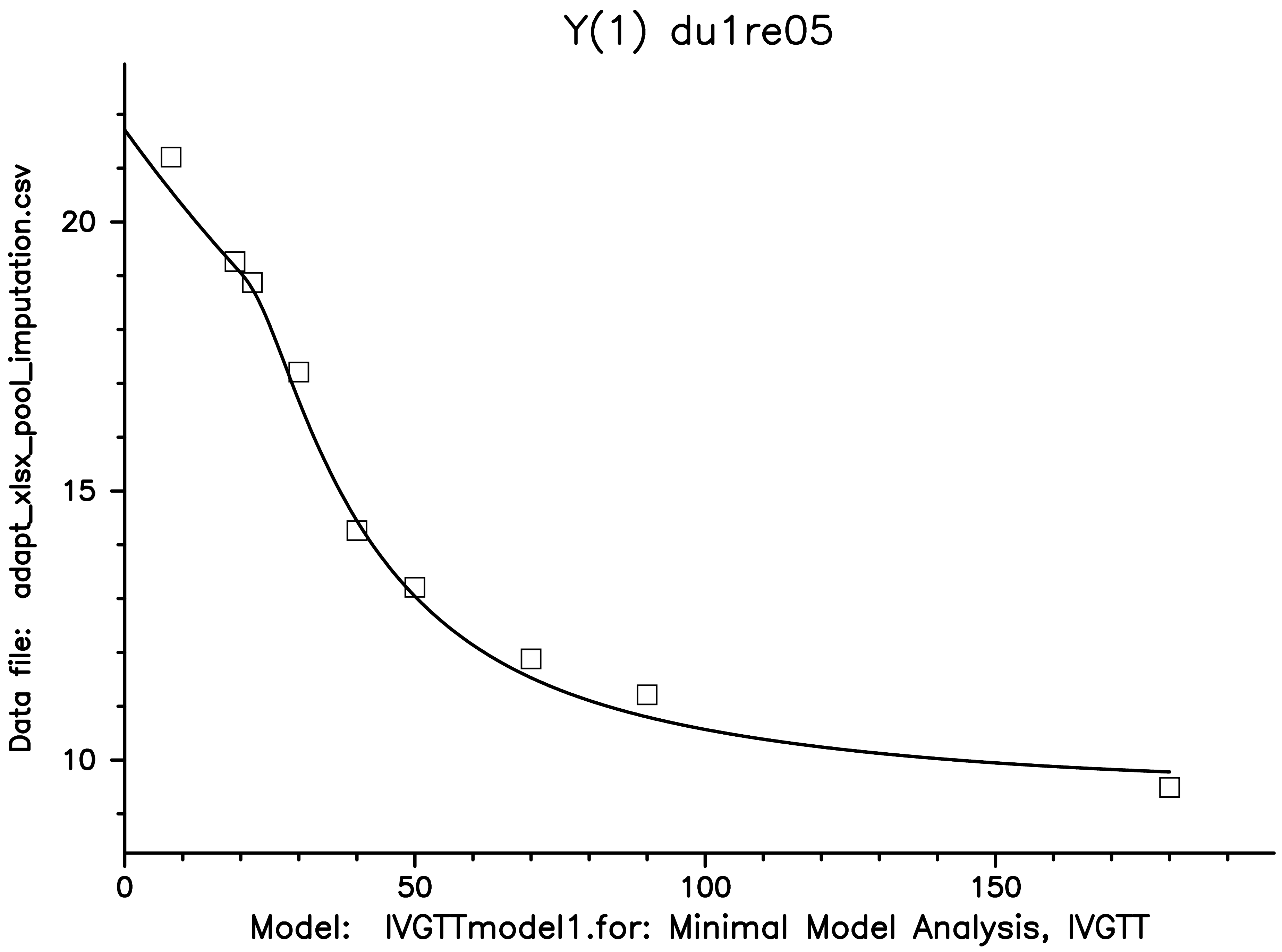
Y(1) du1re05
Data file: adapt_xlsx_pool_imputation.csv
20
15
10
0
50
100
150
Model: IVGTTmodel1.for: Minimal Model Analysis, IVGTT

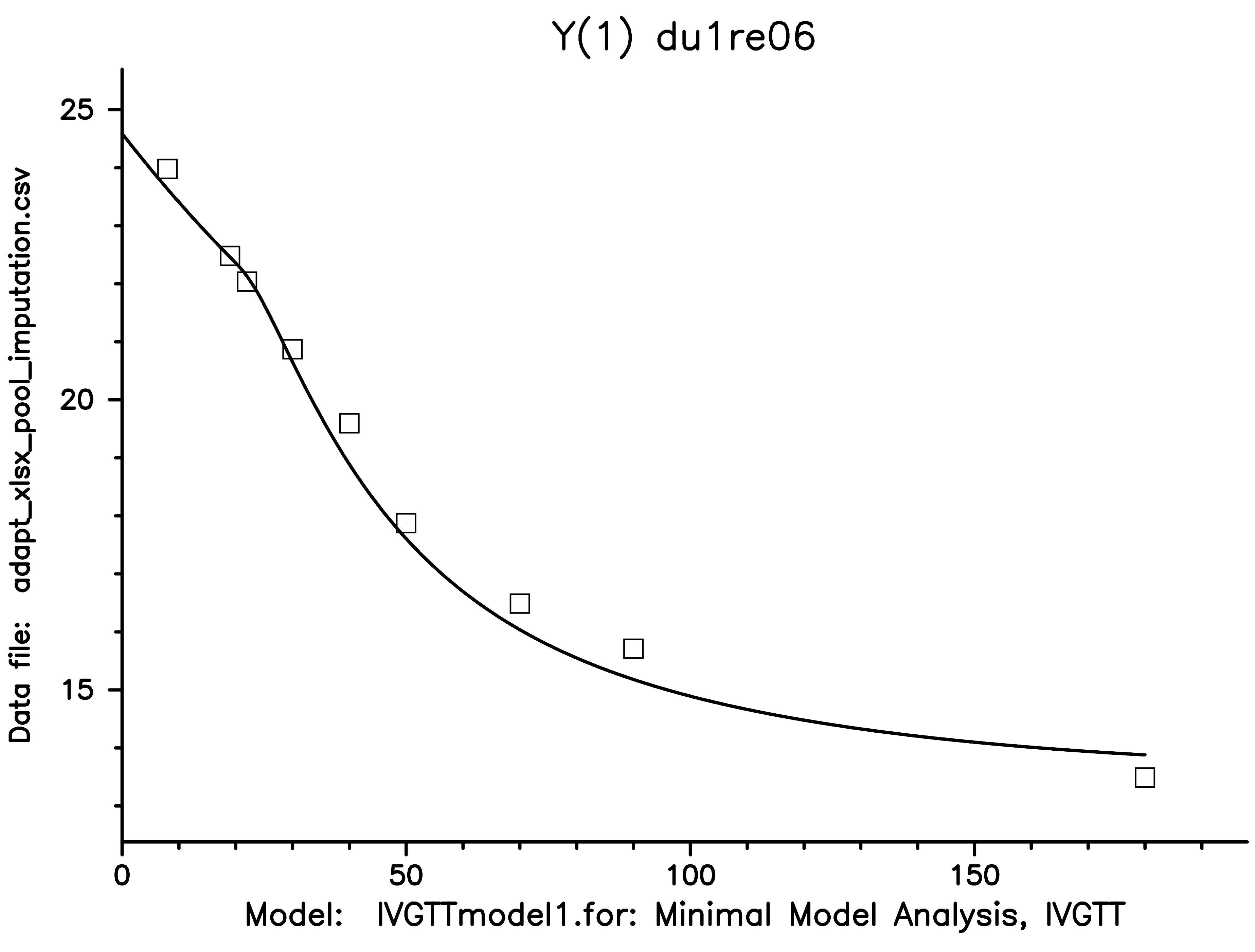

Y(1) du1re06
25
20
15
0
50
100
150
Data file: adapt_xlsx_pool_imputation.csv
Model: IVGTTmodel1.for: Minimal Model Analysis, IVGTT

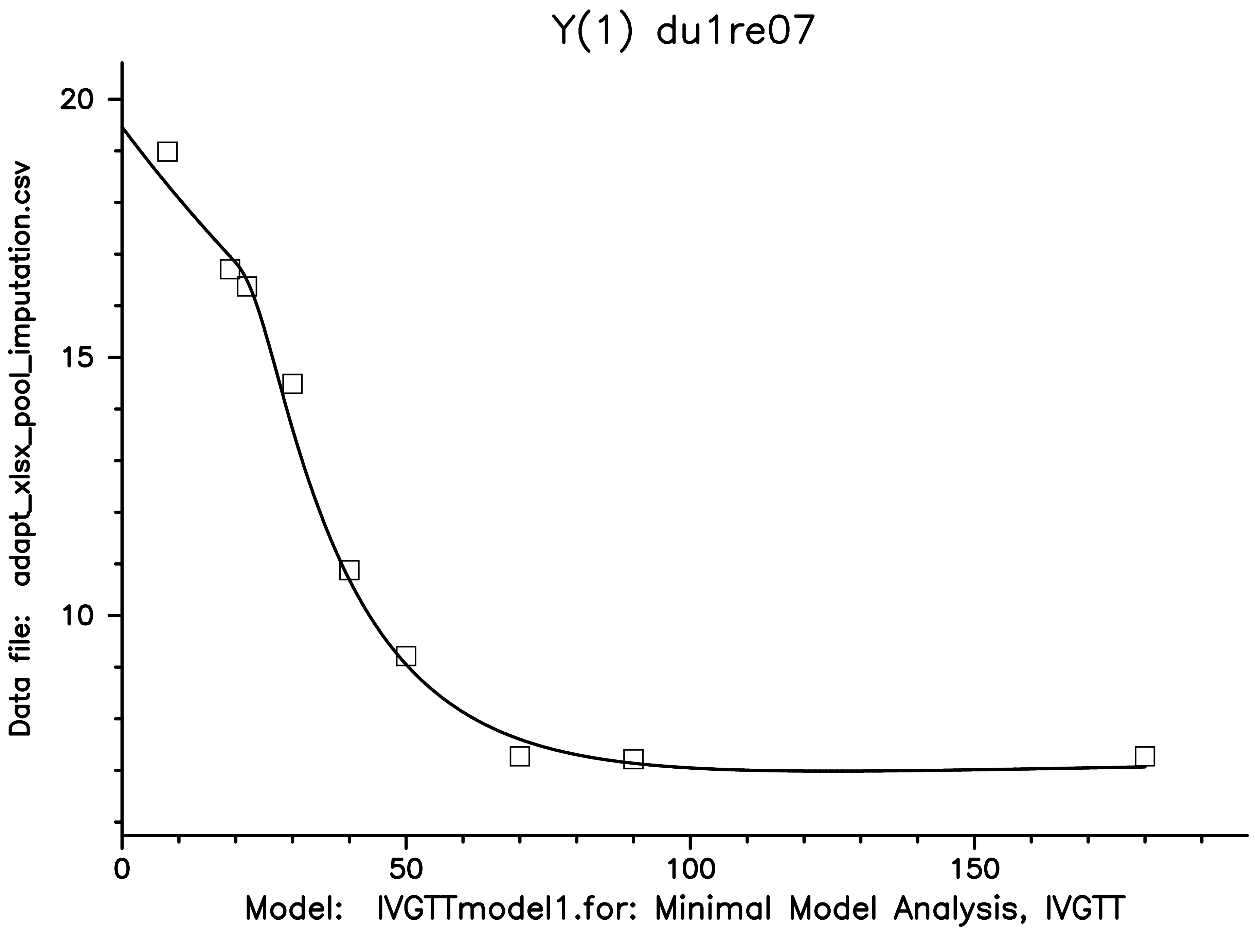

Y(1) du1re07
20
15
10
0
50
100
150
Data file: adapt_xlsx_pool_imputation.csv
Model: IVGTTmodel1.for: Minimal Model Analysis, IVGTT

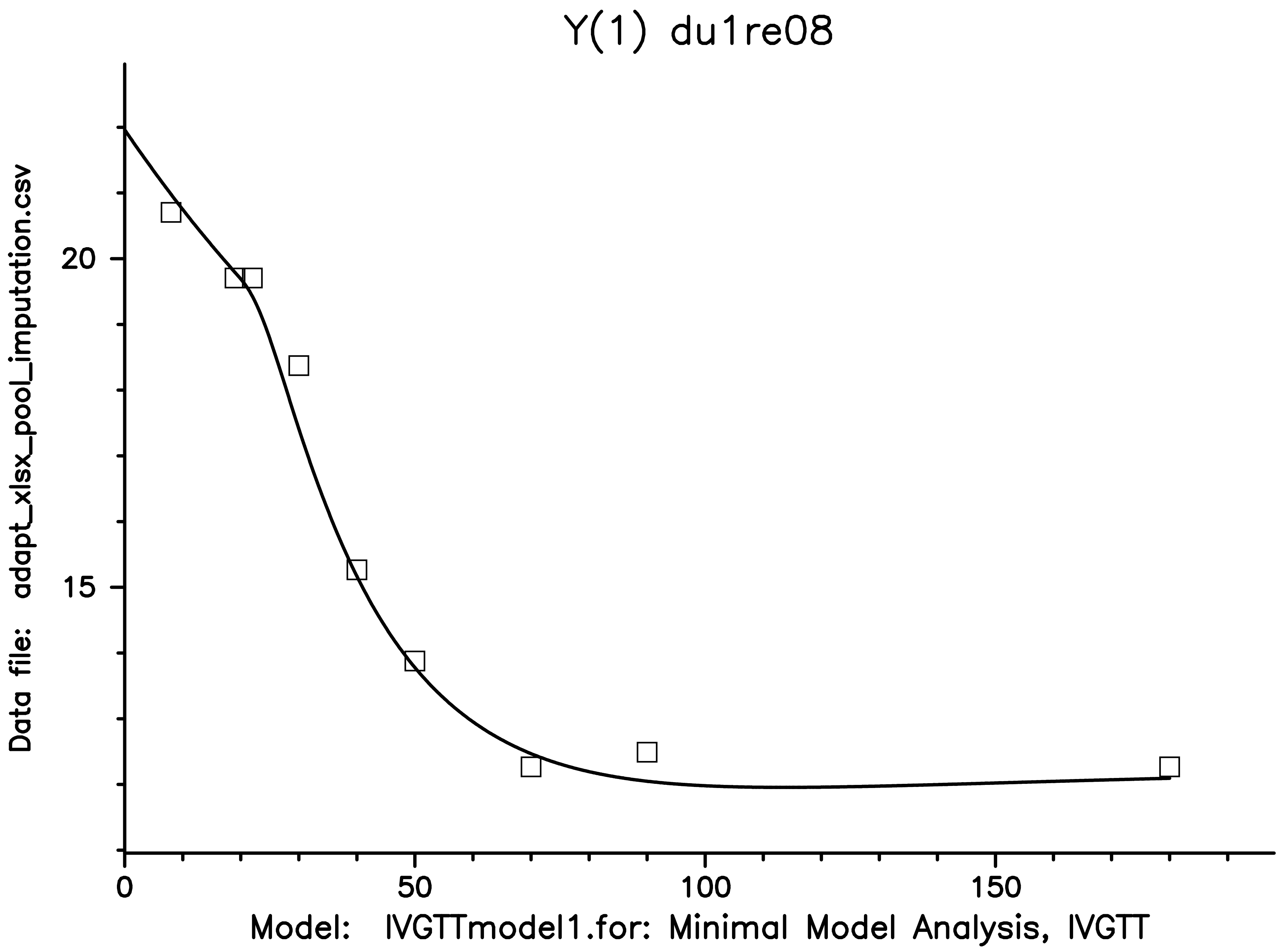
Y(1) du1re08
Data file: adapt_xlsx_pool_imputation.csv
20
15
0
50
100
150
Model: IVGTTmodel1.for: Minimal Model Analysis, IVGTT

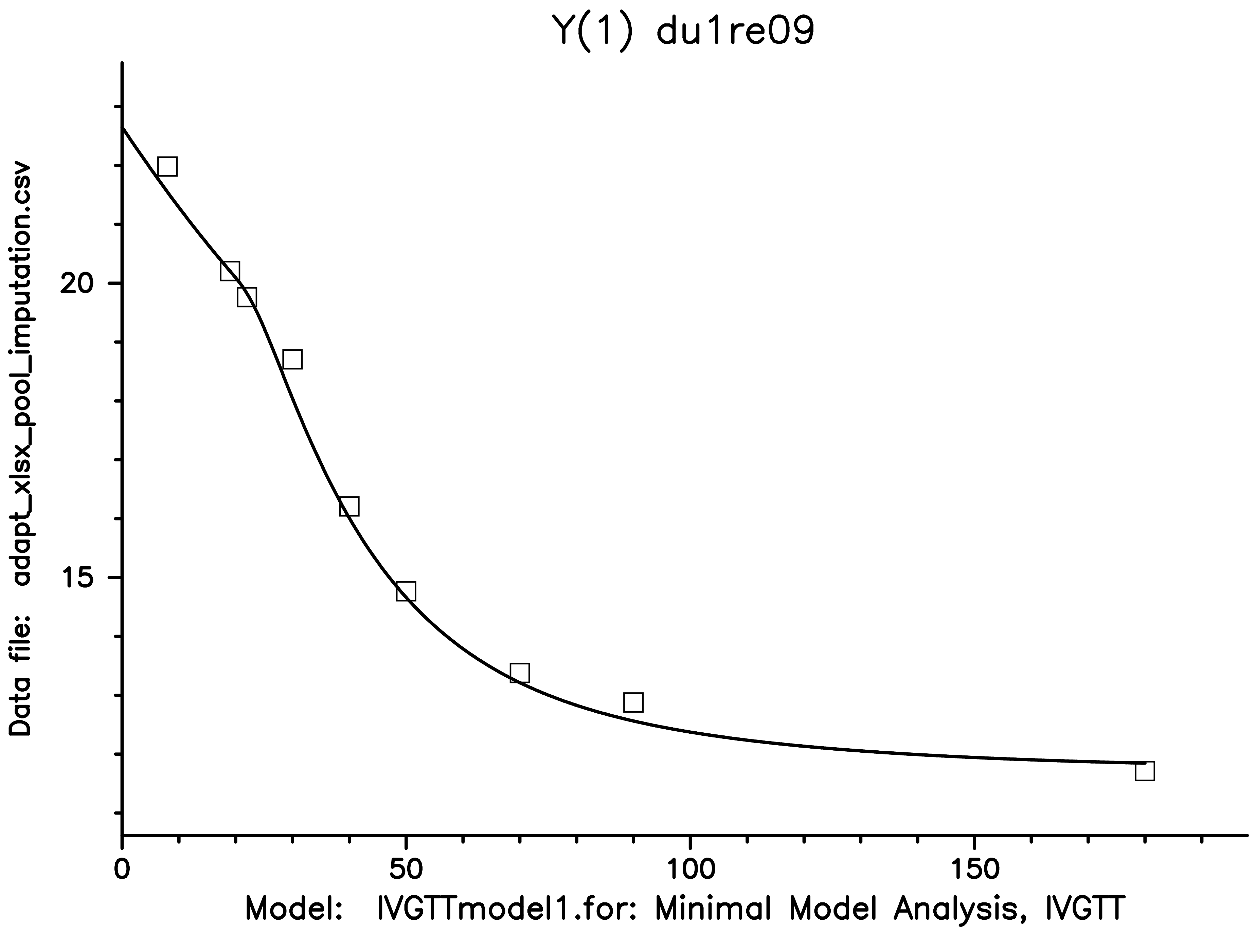

Y(1) du1re09
Data file: adapt_xlsx_pool_imputation.csv
20
15
0
50
100
150
Model: IVGTTmodel1.for: Minimal Model Analysis, IVGTT

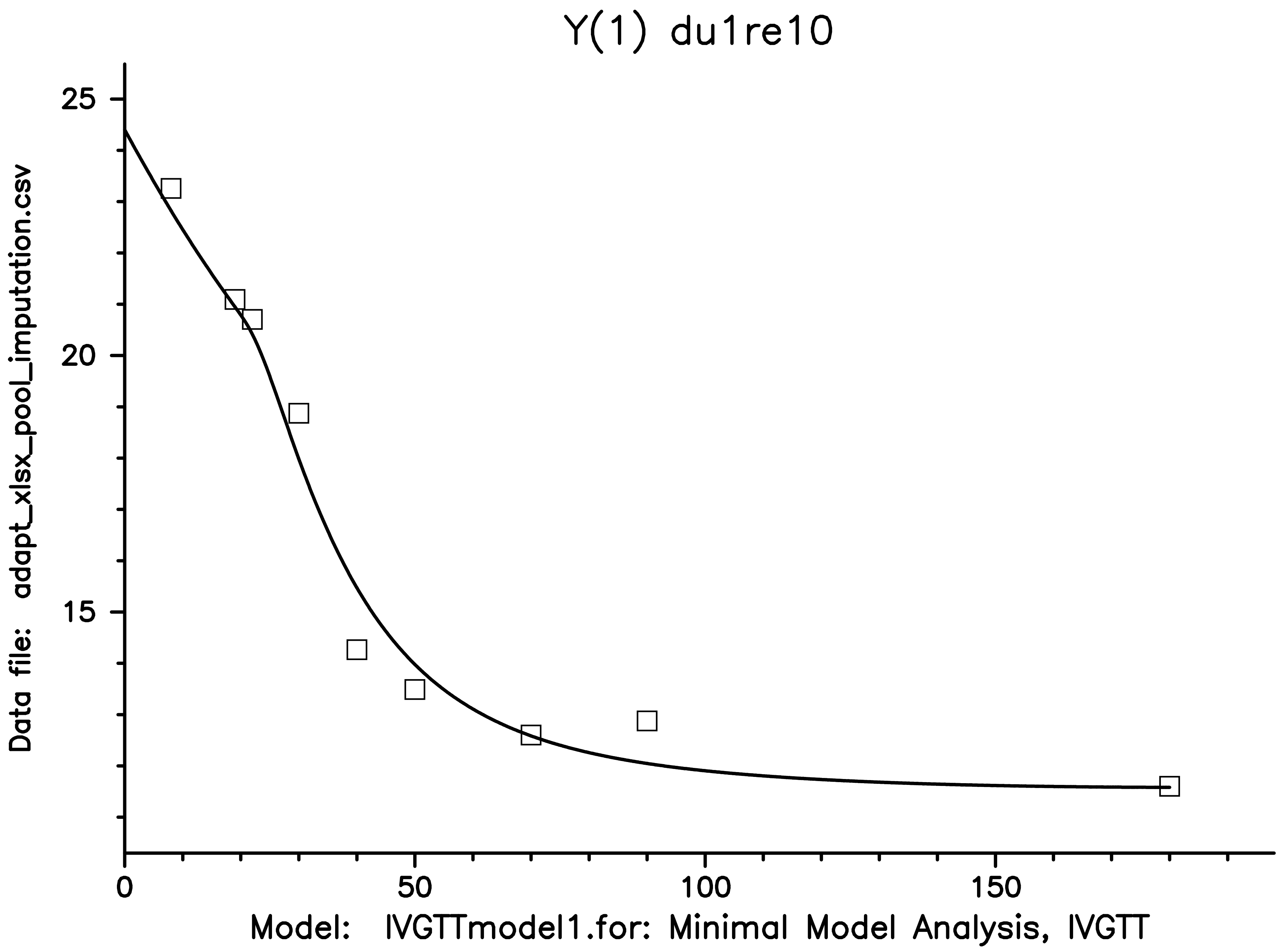

Y(1) du1re10
25
20
15
0
50
100
150
Model: IVGTTmodel1.for: Minimal Model Analysis, IVGTT
Data file: adapt_xlsx_pool_imputation.csv

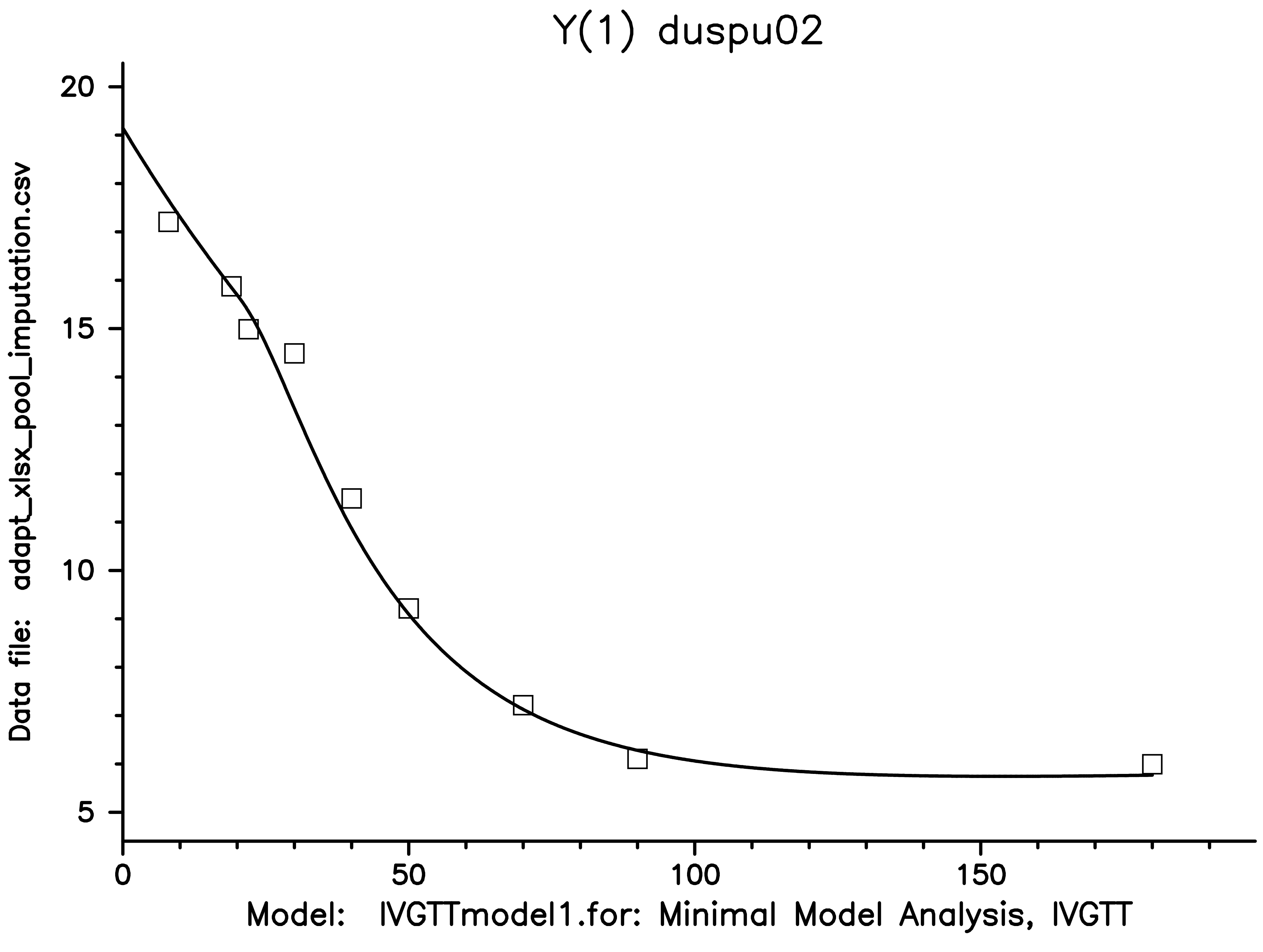
Y(1) duspu02
Data file: adapt_xlsx_pool_imputation.csv
20
15
10
5
0
50
100
150
Model: IVGTTmodel1.for: Minimal Model Analysis, IVGTT

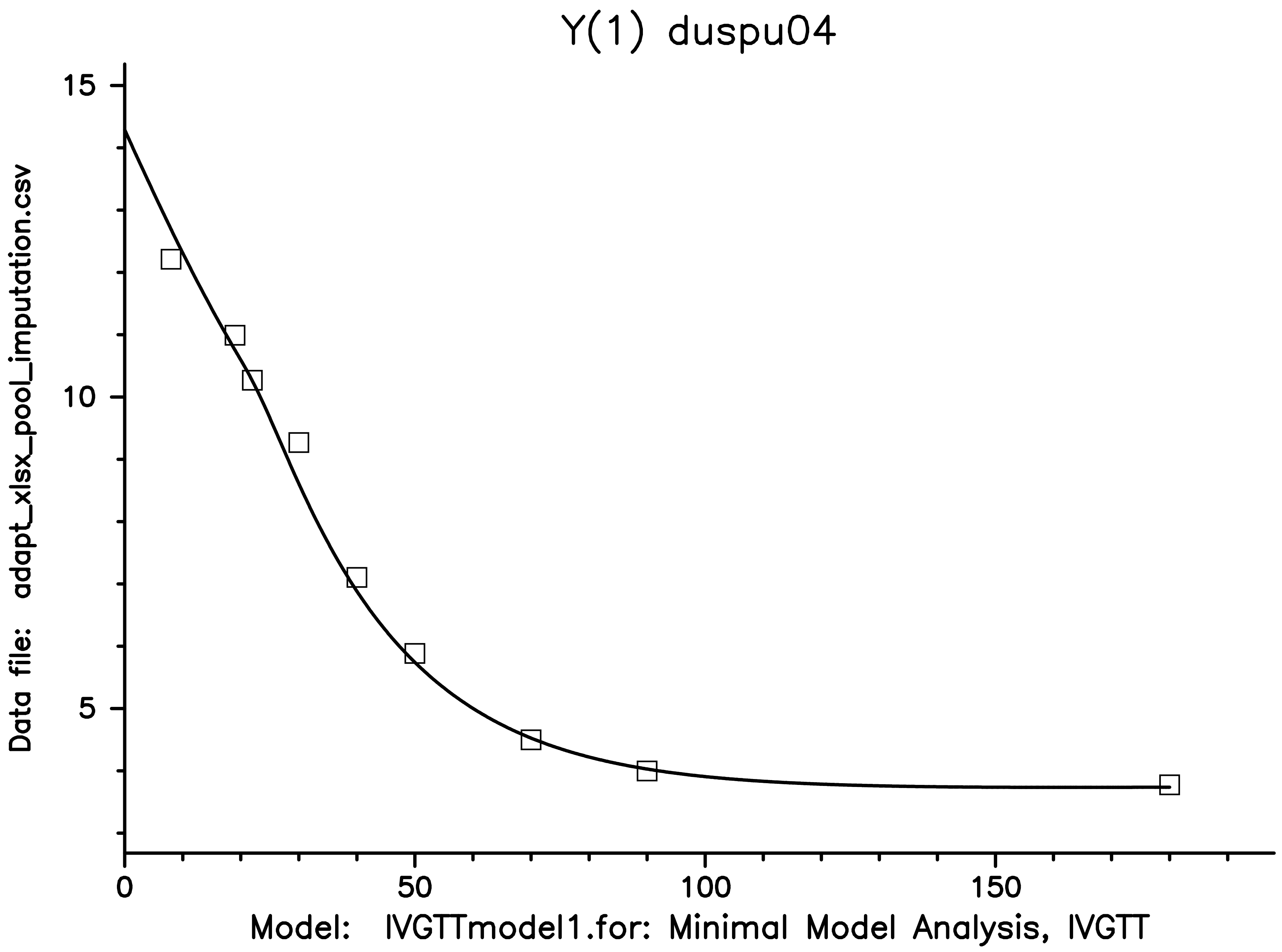
Y(1) duspu04
Data file: adapt_xlsx_pool_imputation.csv
15
10
5
0
50
100
150
Model: IVGTTmodel1.for: Minimal Model Analysis, IVGTT

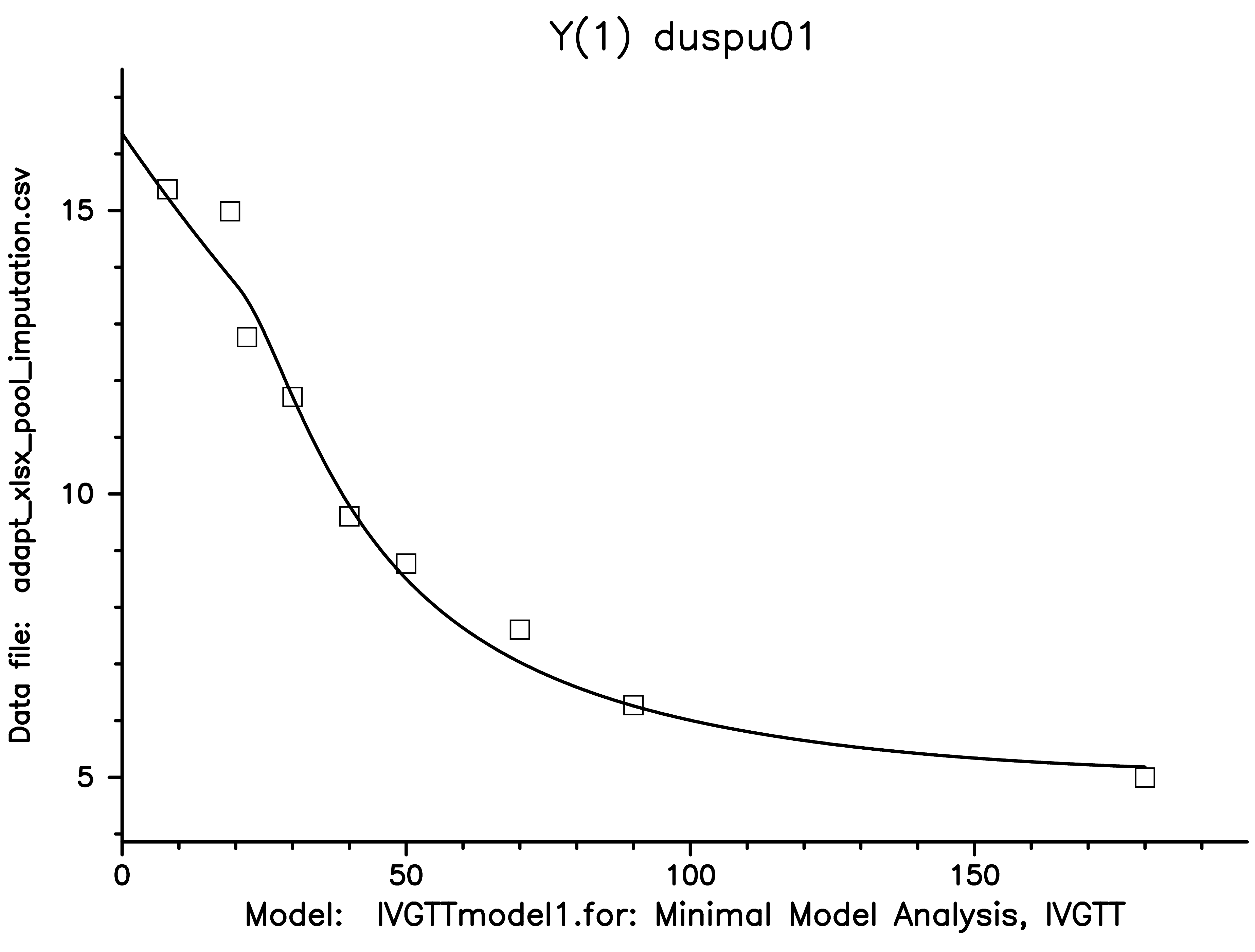
Y(1) duspu01
Data file: adapt_xlsx_pool_imputation.csv
15
10
5
0
50
100
150
Model: IVGTTmodel1.for: Minimal Model Analysis, IVGTT

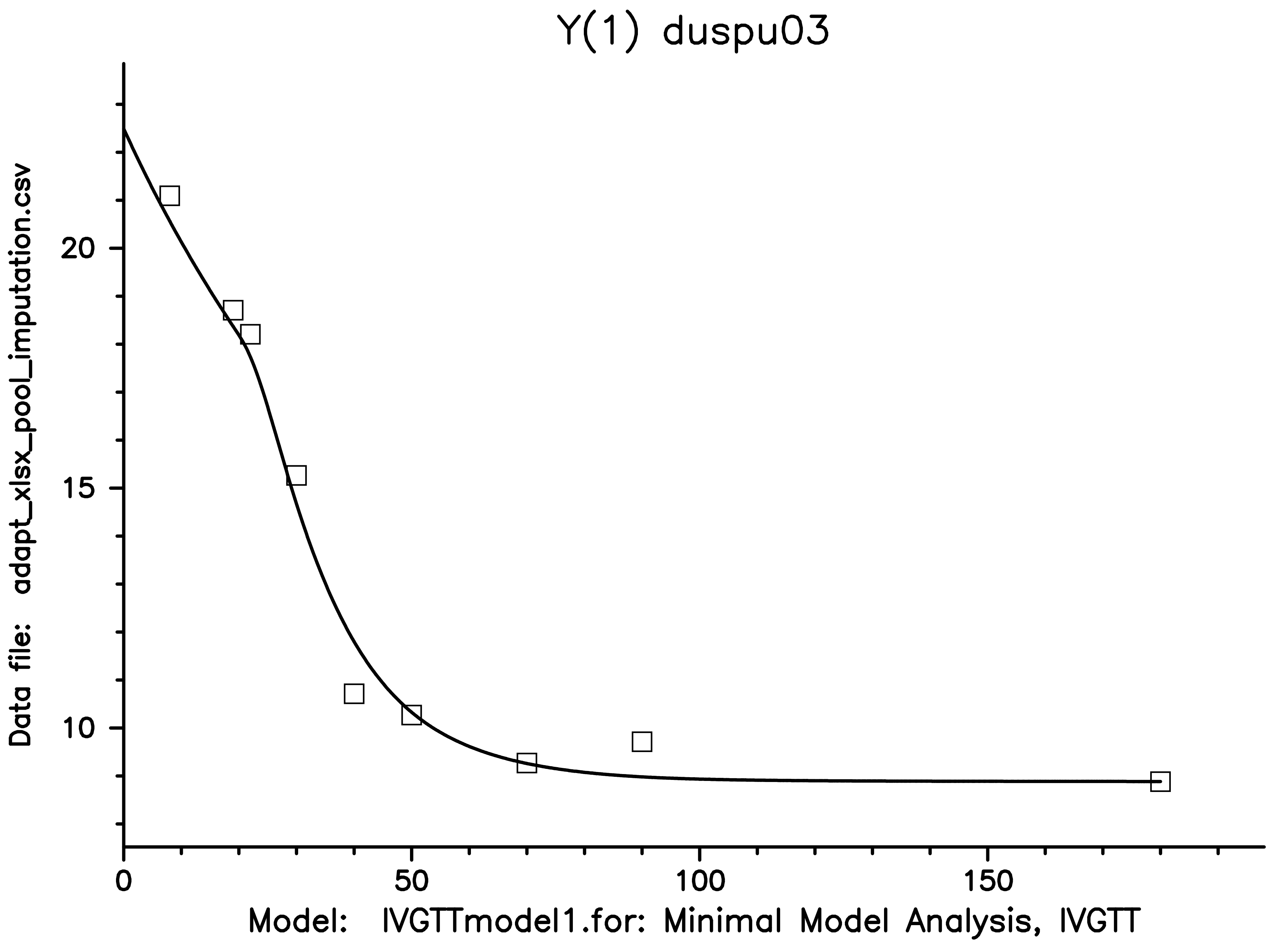
Y(1) duspu03
Data file: adapt_xlsx_pool_imputation.csv
20
15
10
0
50
100
150
Model: IVGTTmodel1.for: Minimal Model Analysis, IVGTT

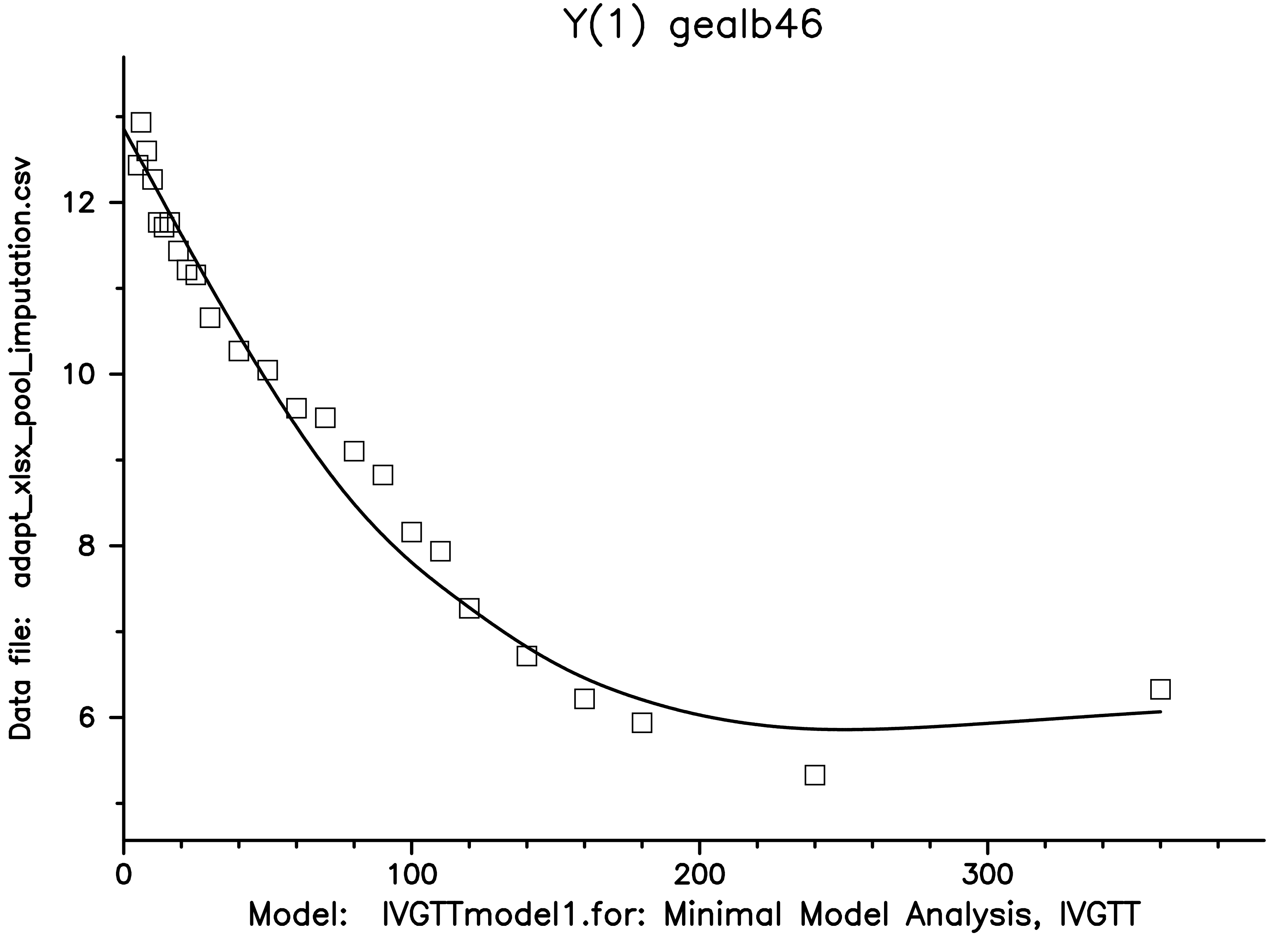
Y(1) gealb46
Data file: adapt_xlsx_pool_imputation.csv
12
10
8
6
0
100
200
300
Model: IVGTTmodel1.for: Minimal Model Analysis, IVGTT

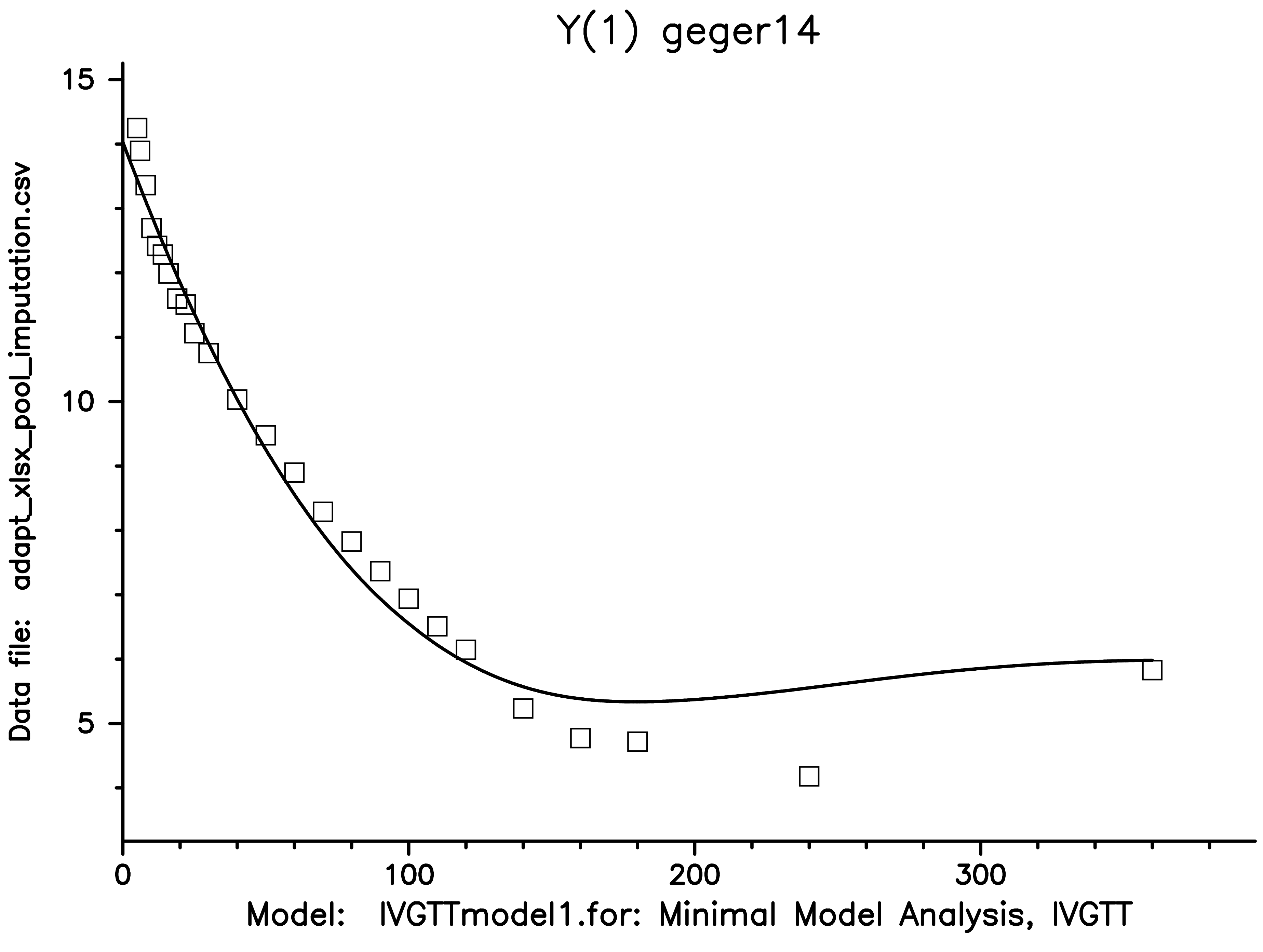

Y(1) geger14
Data file: adapt_xlsx_pool_imputation.csv
15
10
5
0
100
200
300
Model: IVGTTmodel1.for: Minimal Model Analysis, IVGTT

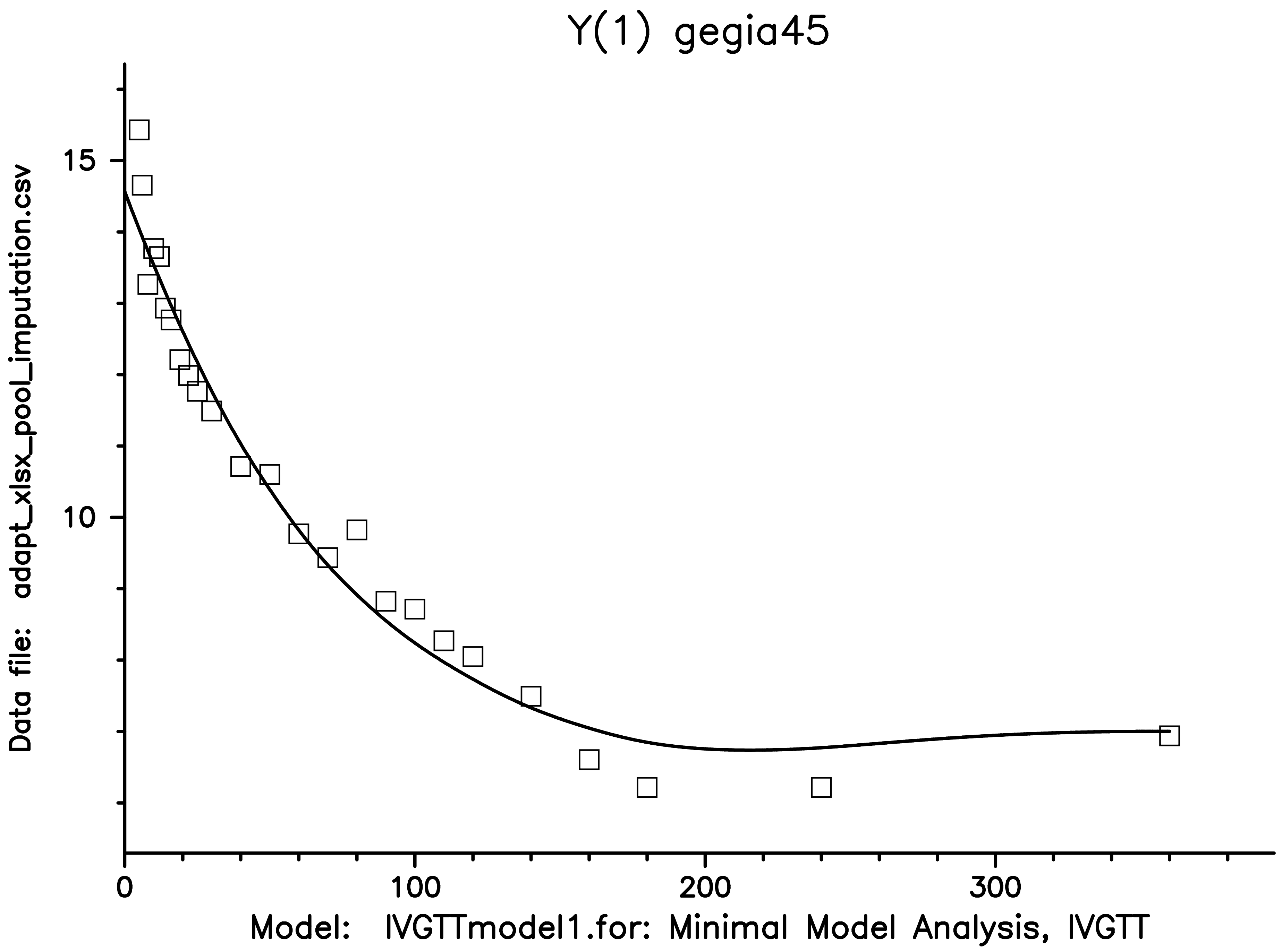

Y(1) gegia45
15
10
0
100
200
300
Data file: adapt_xlsx_pool_imputation.csv
Model: IVGTTmodel1.for: Minimal Model Analysis, IVGTT

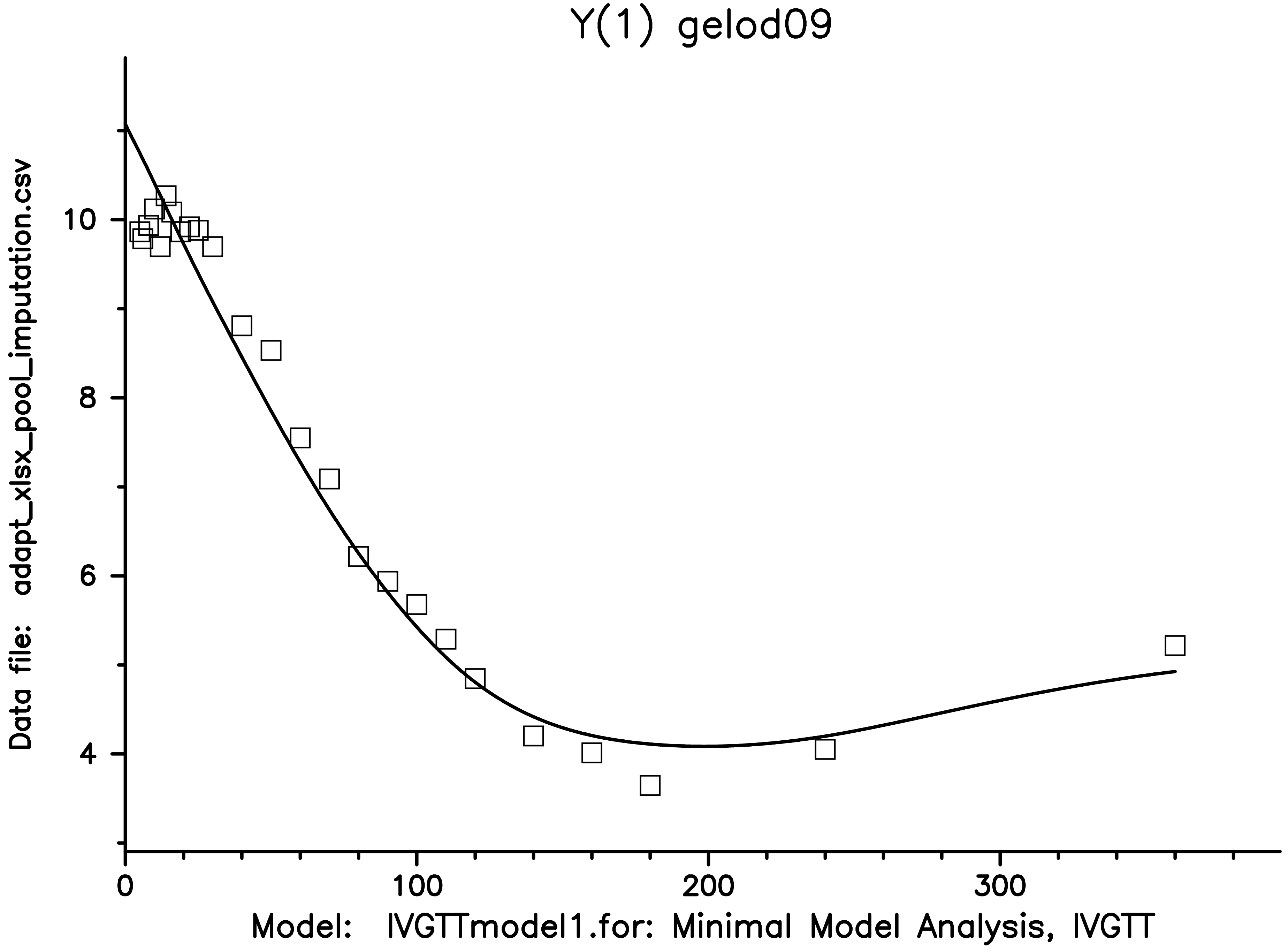
Y(1) gelod09
Data file: adapt_xlsx_pool_imputation.csv
10
8
6
4
0
100
200
300
Model: IVGTTmodel1.for: Minimal Model Analysis, IVGTT

Y(1) gerol48

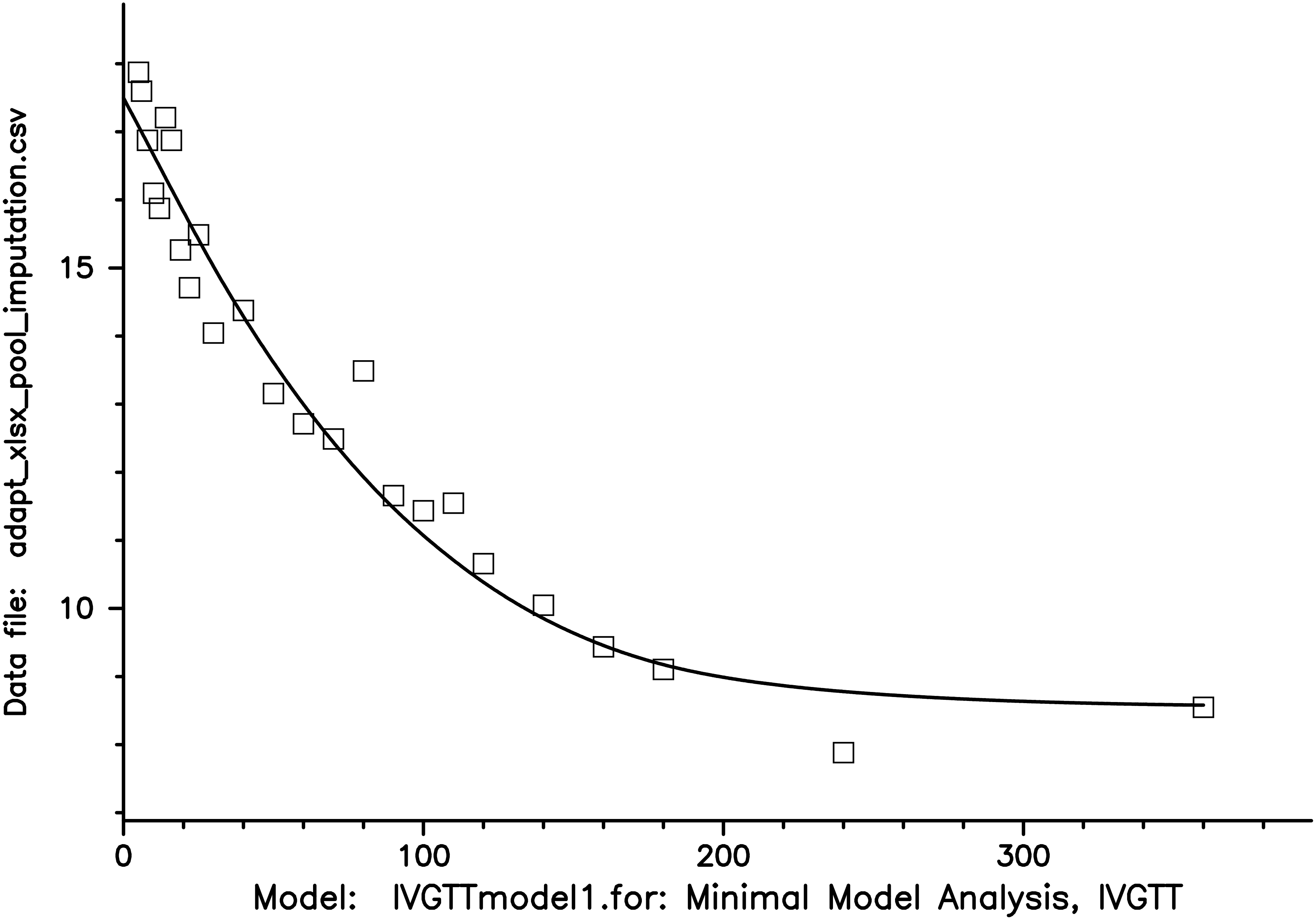

Data file: adapt_xlsx_pool_imputation.csv
15
10
0
100
200
300
Model: IVGTTmodel1.for: Minimal Model Analysis, IVGTT

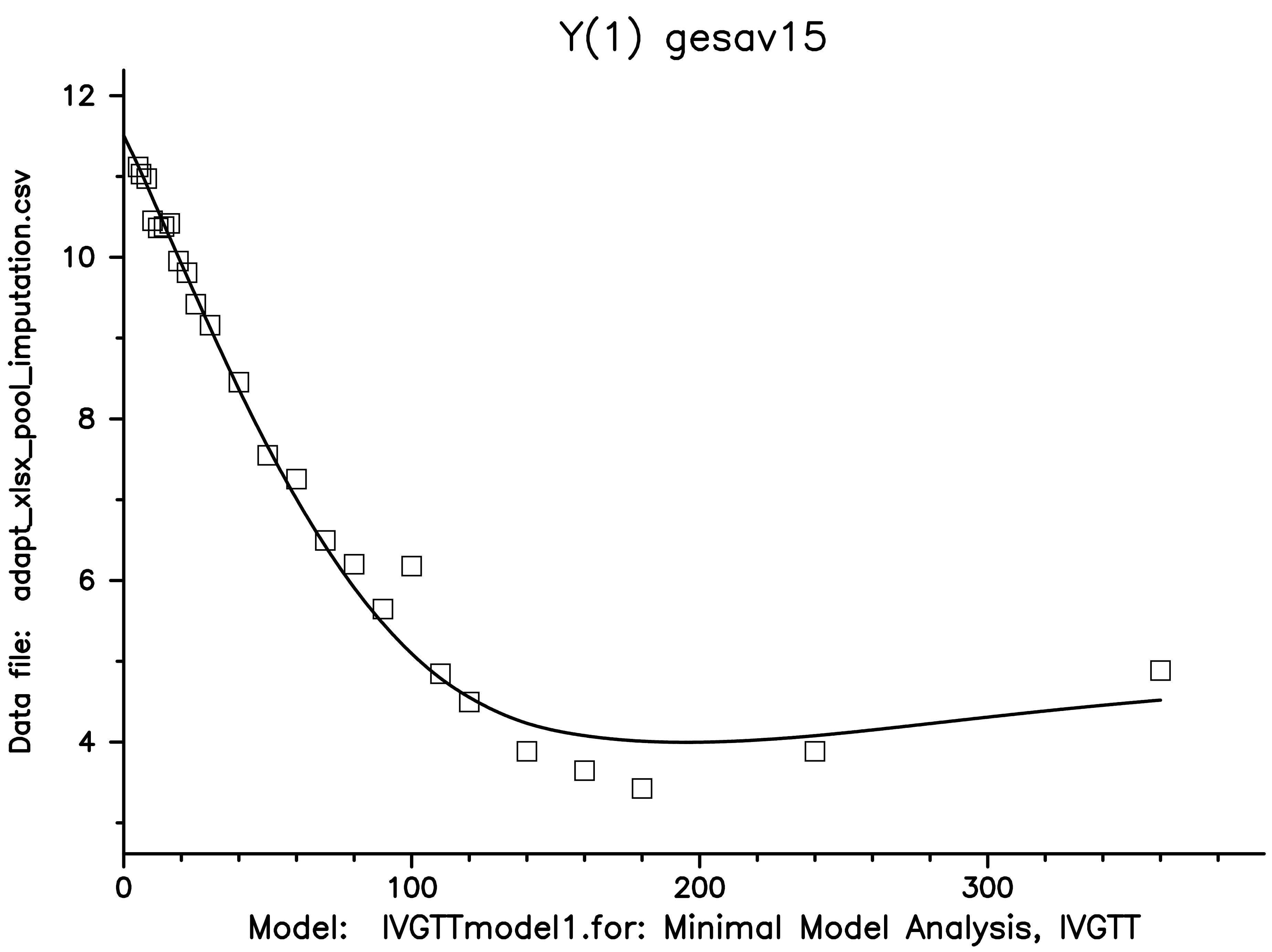

Y(1) gesav15
Data file: adapt_xlsx_pool_imputation.csv
12
10
8
6
4
0
100
200
300
Model: IVGTTmodel1.for: Minimal Model Analysis, IVGTT

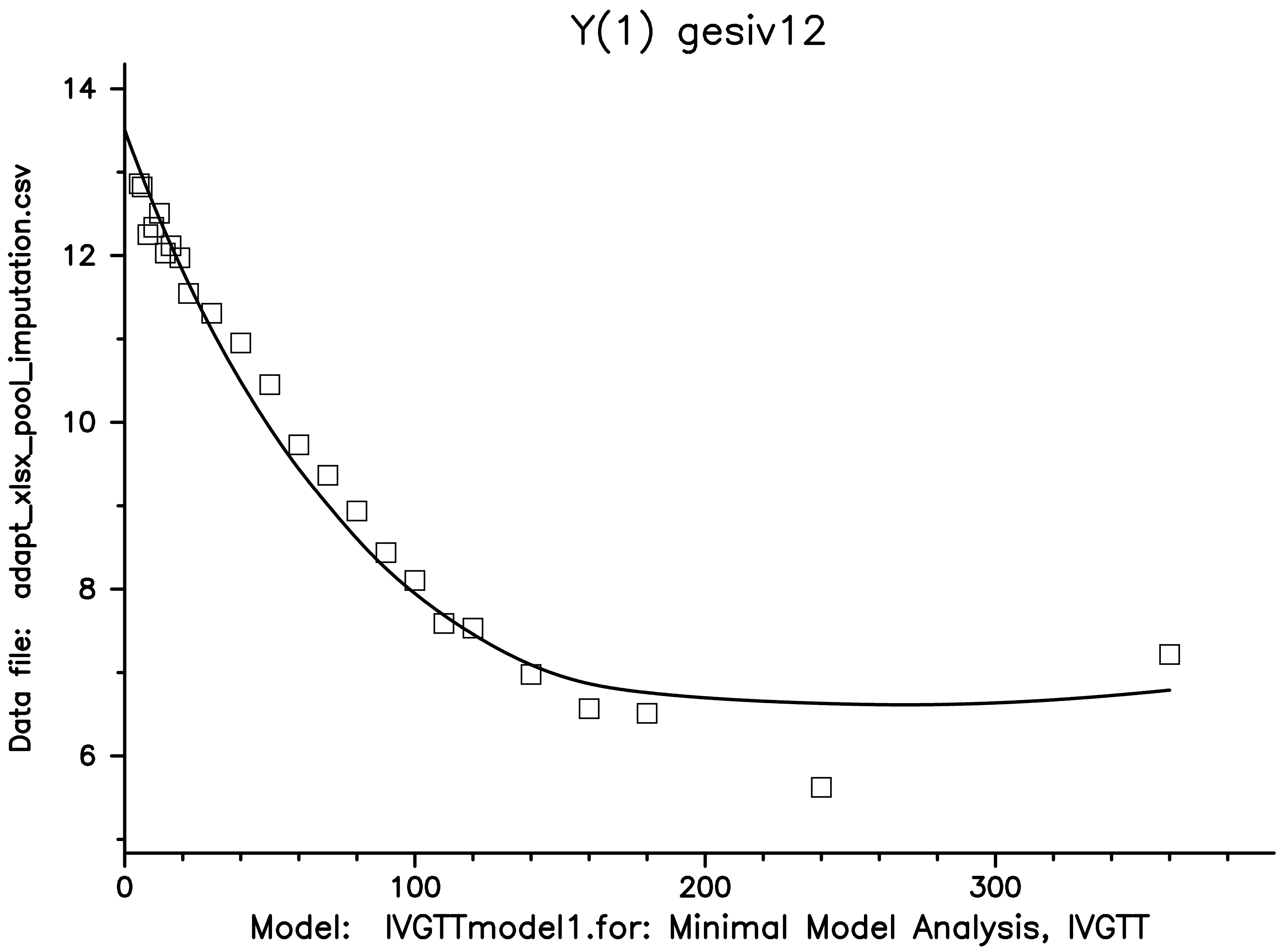
Y(1) gesiv12
Data file: adapt_xlsx_pool_imputation.csv
14
12
10
8
6
0
100
200
300
Model: IVGTTmodel1.for: Minimal Model Analysis, IVGTT

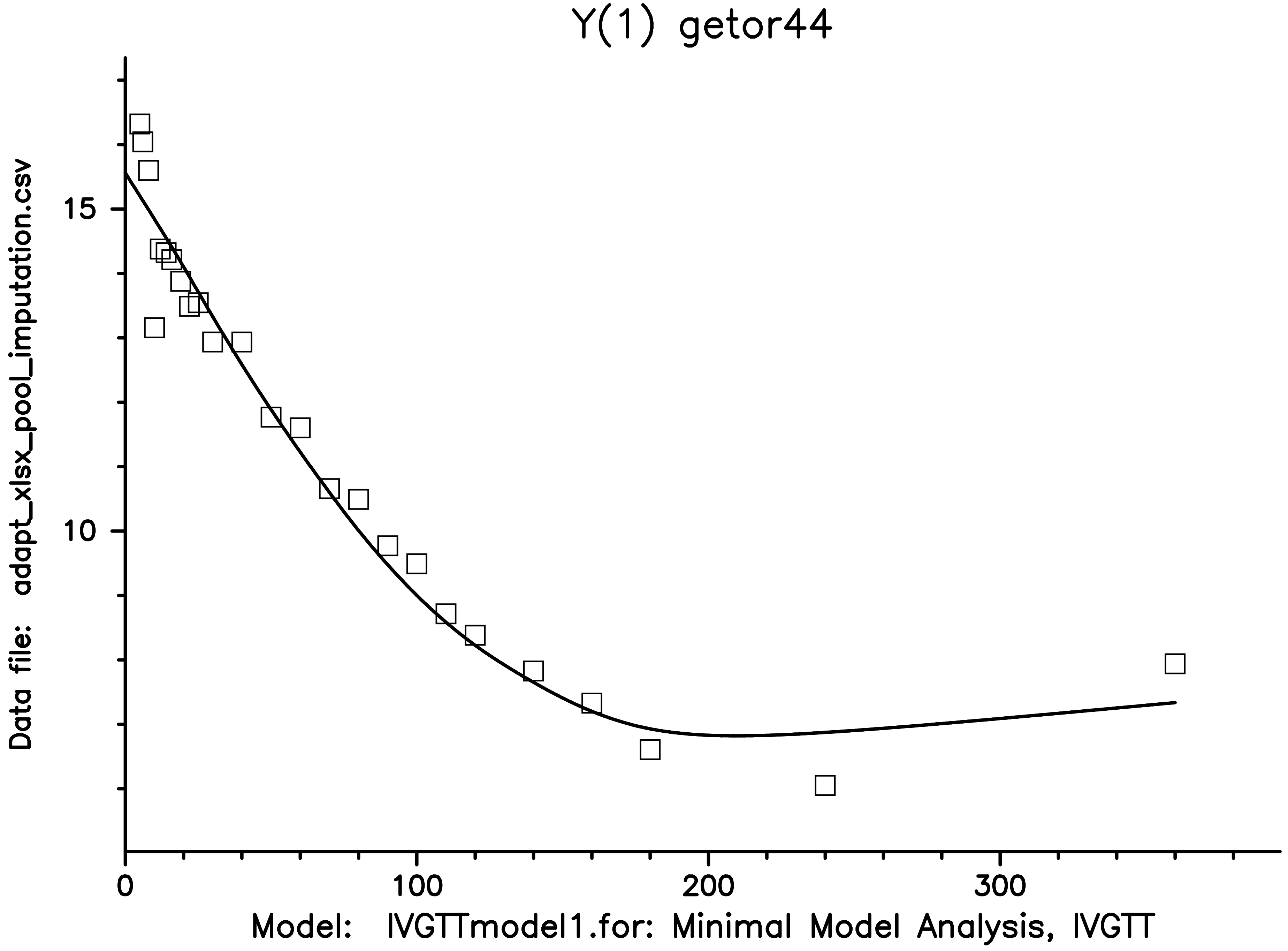
Y(1) getor44
Data file: adapt_xlsx_pool_imputation.csv
15
10
0
100
200
300
Model: IVGTTmodel1.for: Minimal Model Analysis, IVGTT

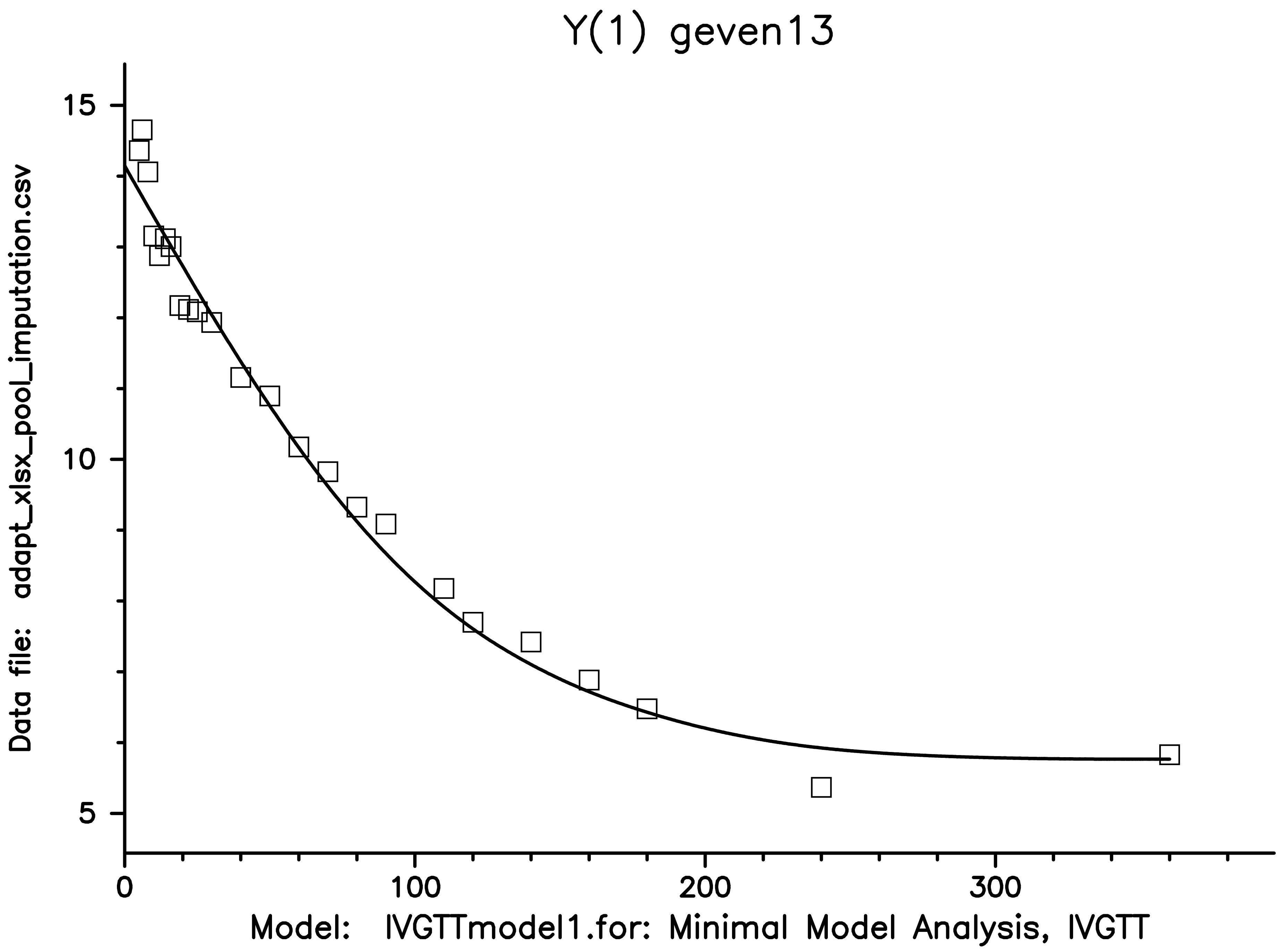
Y(1) geven13
Data file: adapt_xlsx_pool_imputation.csv
15
10
5
0
100
200
300
Model: IVGTTmodel1.for: Minimal Model Analysis, IVGTT

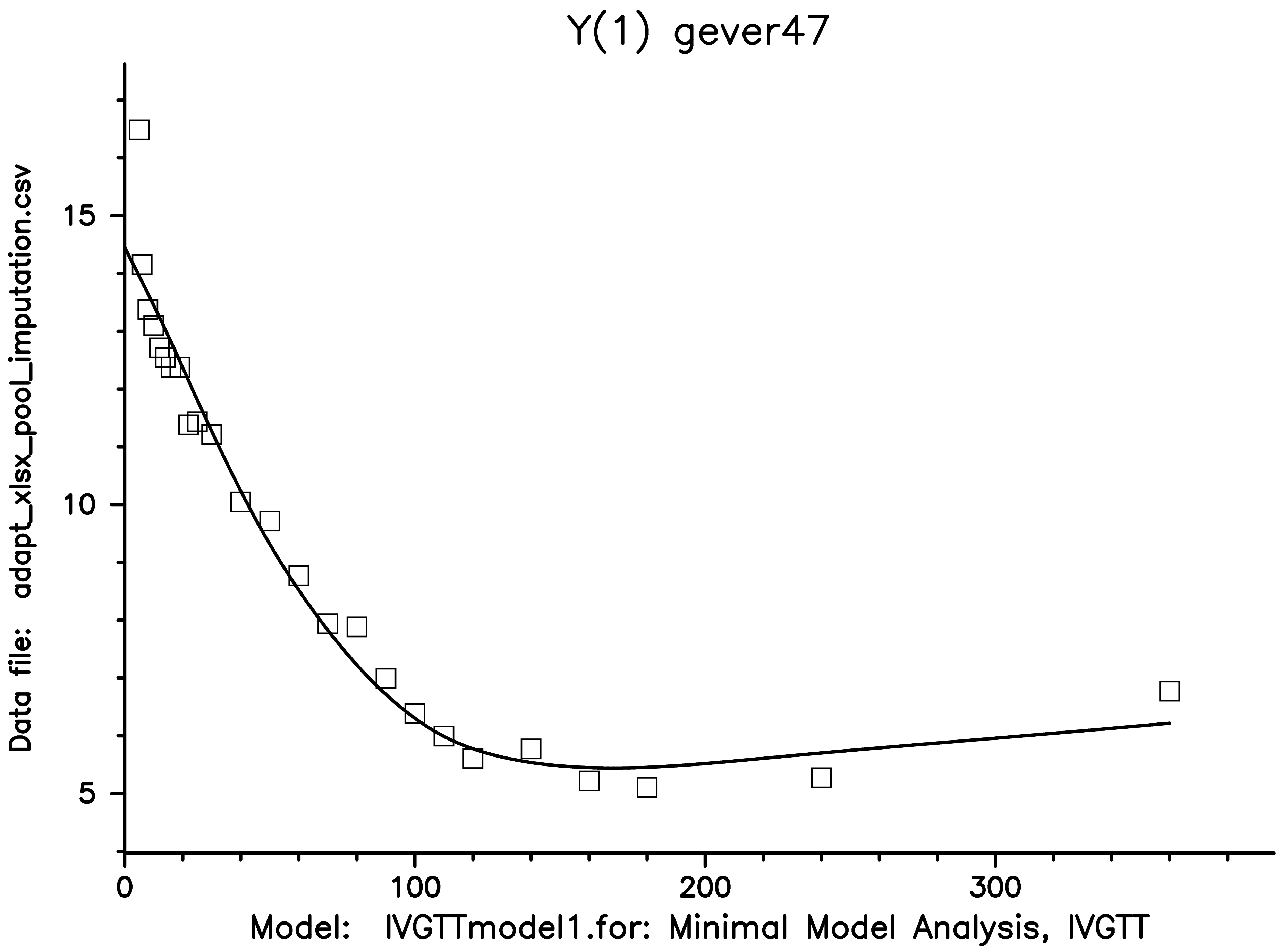
Y(1) gever47
Data file: adapt_xlsx_pool_imputation.csv
15
10
5
0
100
200
300
Model: IVGTTmodel1.for: Minimal Model Analysis, IVGTT

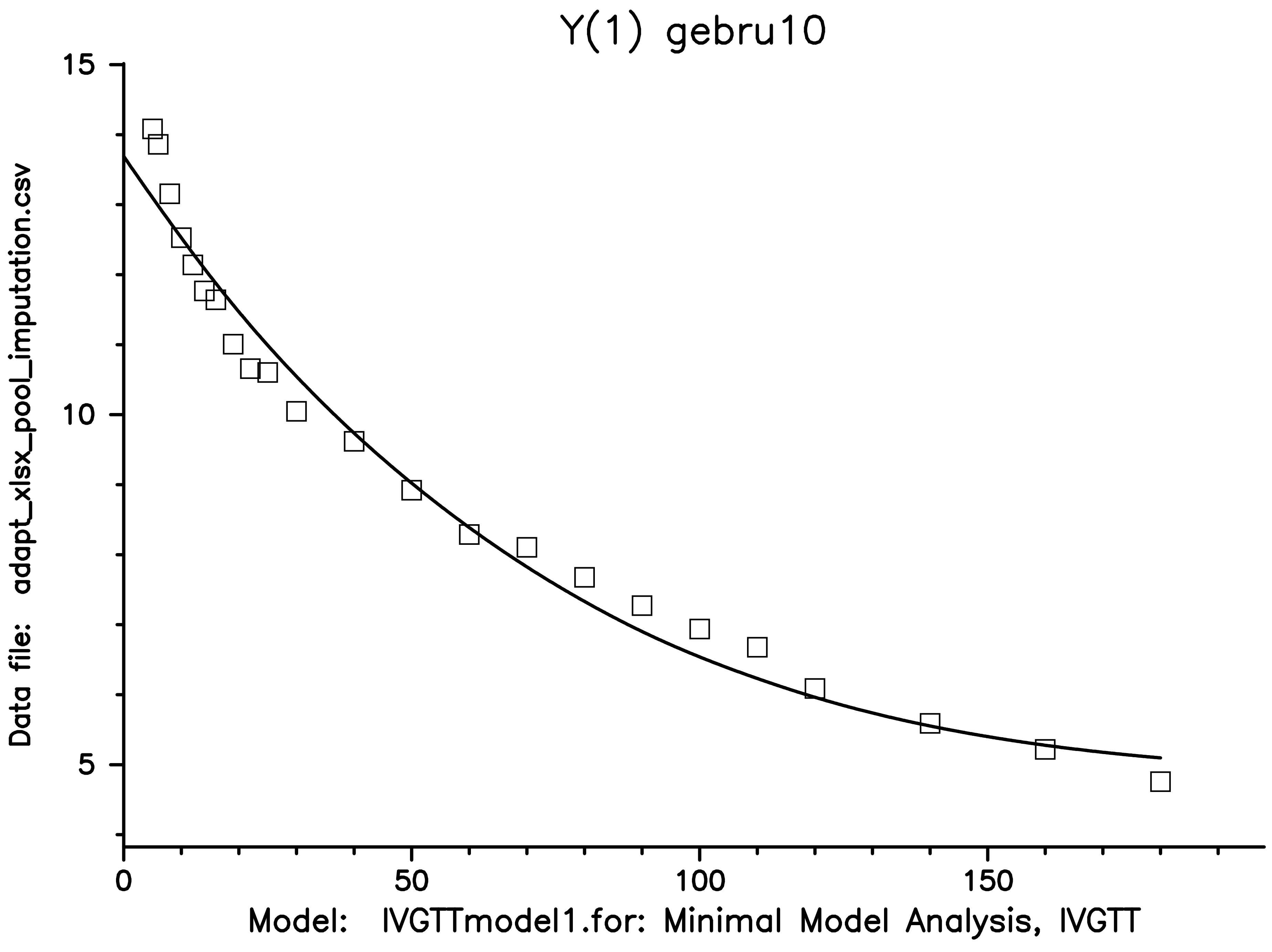
Y(1) gebru10
15
10
5
0
50
100
150
Data file: adapt_xlsx_pool_imputation.csv
Model: IVGTTmodel1.for: Minimal Model Analysis, IVGTT

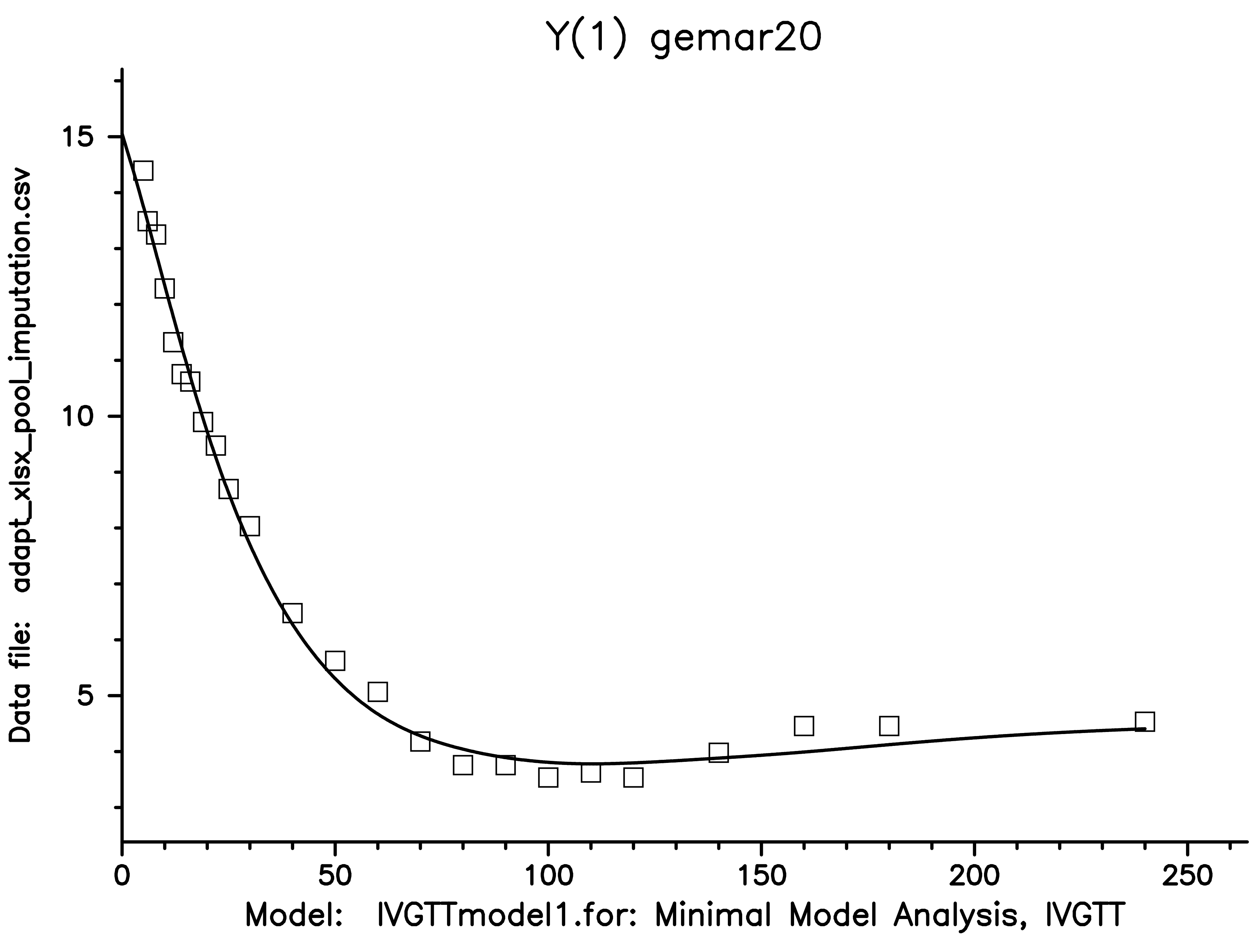

Y(1) gemar20
Data file: adapt_xlsx_pool_imputation.csv
15
10
5
0
50
100
150
200
250
Model: IVGTTmodel1.for: Minimal Model Analysis, IVGTT

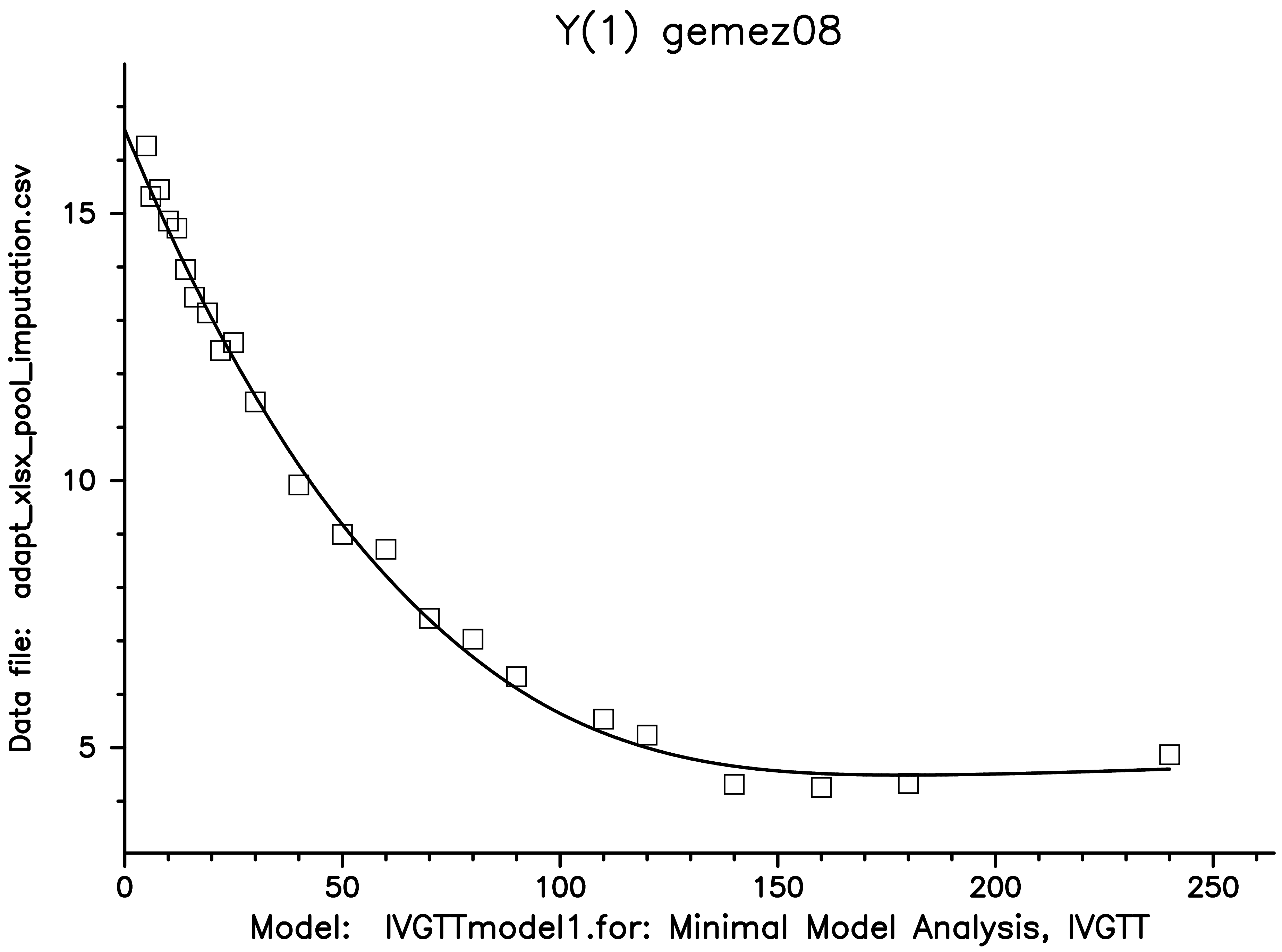
Y(1) gemez08
Data file: adapt_xlsx_pool_imputation.csv
15
10
5
0
50
100
150
200
250
Model: IVGTTmodel1.for: Minimal Model Analysis, IVGTT

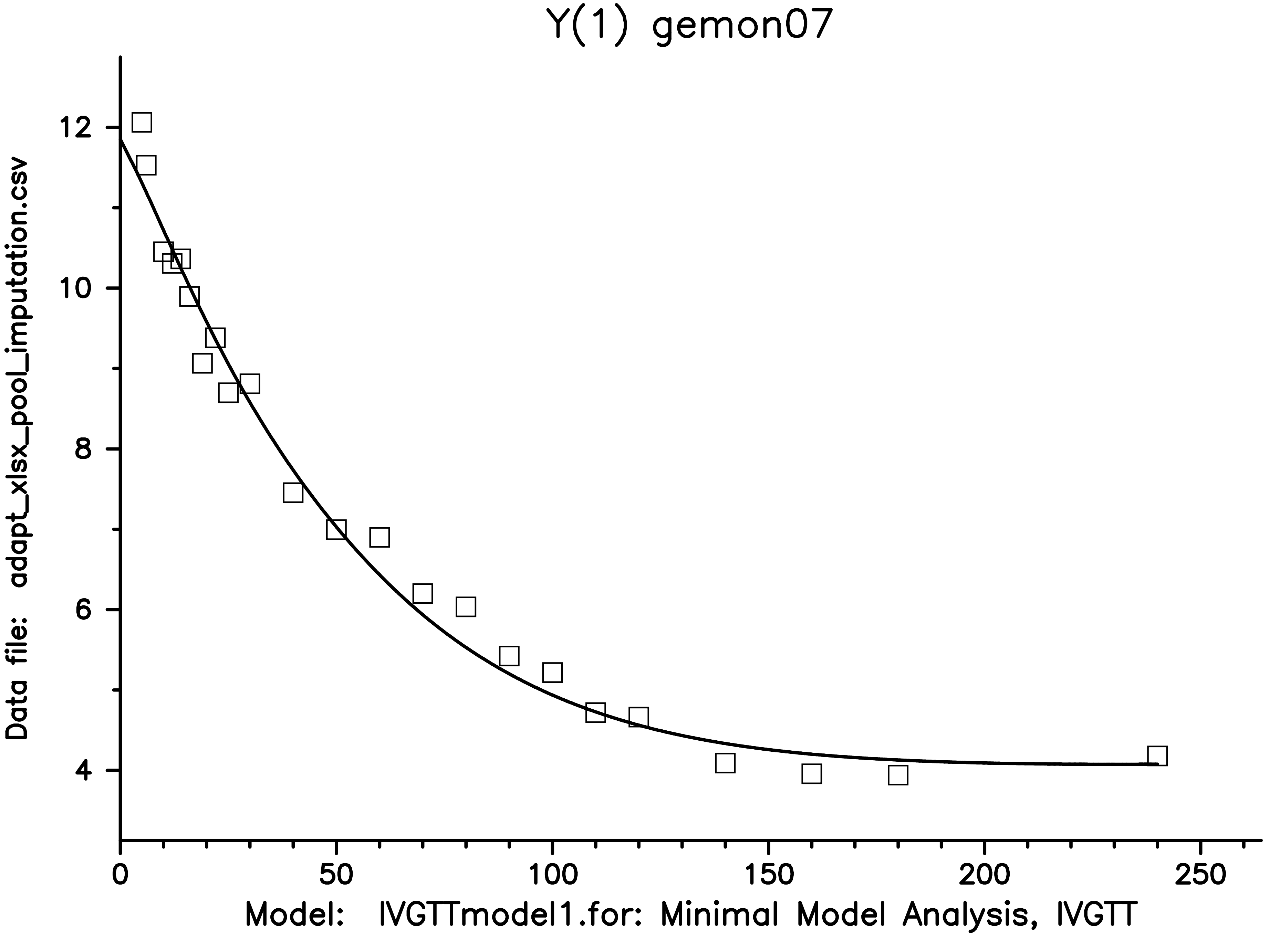
Y(1) gemon07
Data file: adapt_xlsx_pool_imputation.csv
12
10
8
6
4
0
50
100
150
200
250
Model: IVGTTmodel1.for: Minimal Model Analysis, IVGTT

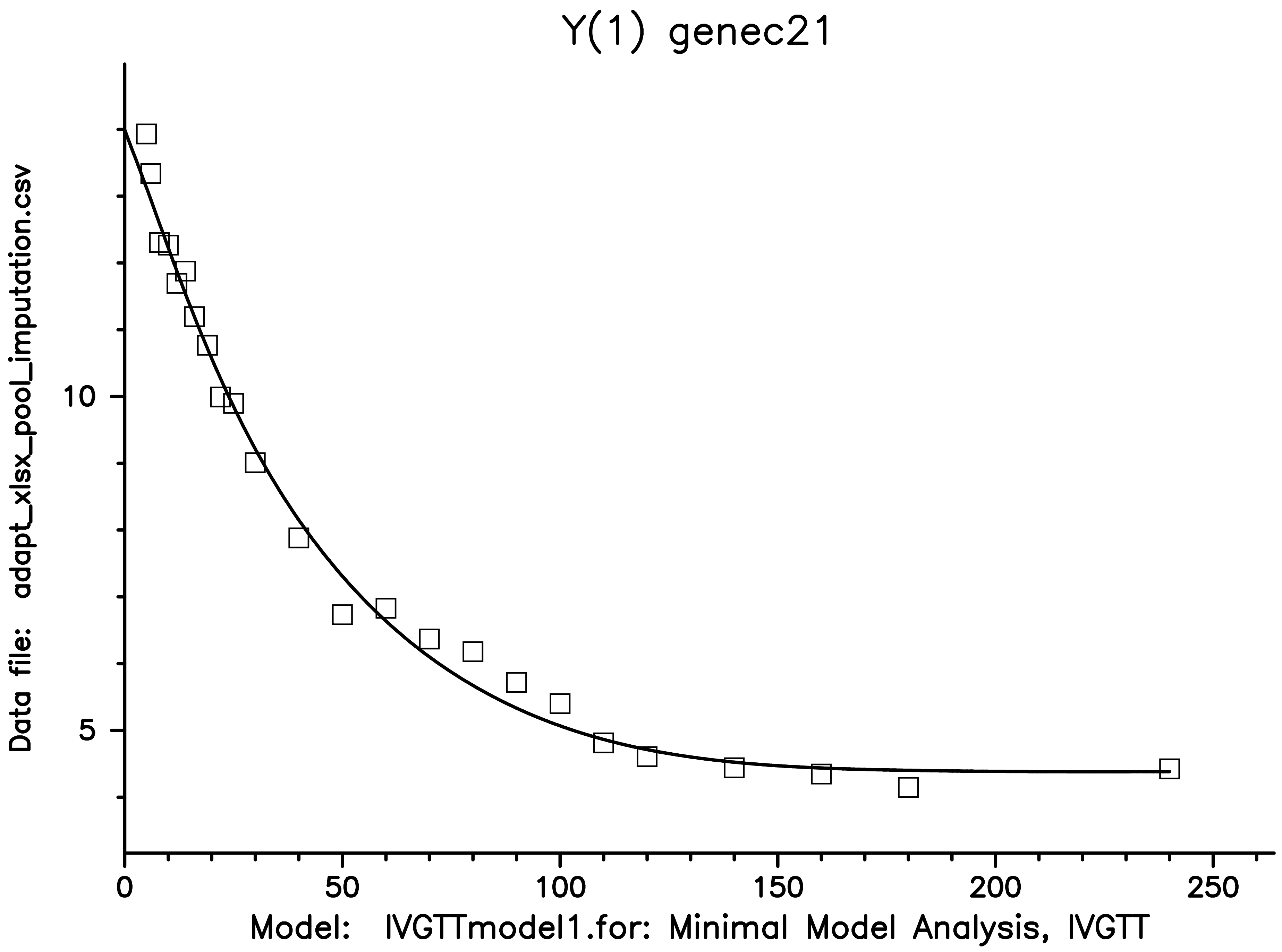
Y(1) genec21
Data file: adapt_xlsx_pool_imputation.csv
10
5
0
50
100
150
200
250
Model: IVGTTmodel1.for: Minimal Model Analysis, IVGTT

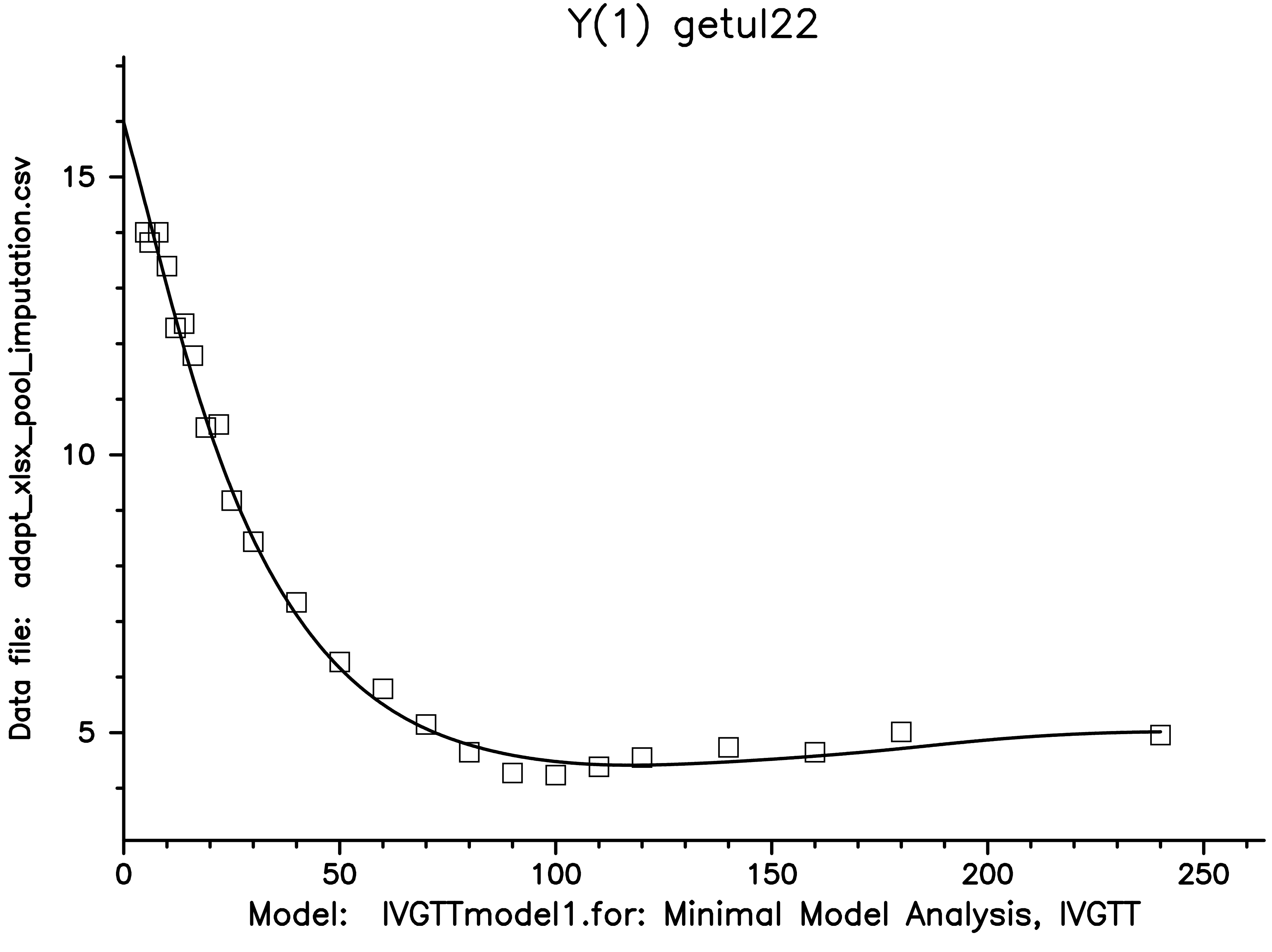
Y(1) getul22
Data file: adapt_xlsx_pool_imputation.csv
Model: IVGTTmodel1.for: Minimal Model Analysis, IVGTT
15
10
5
0
50
100
150
200
250

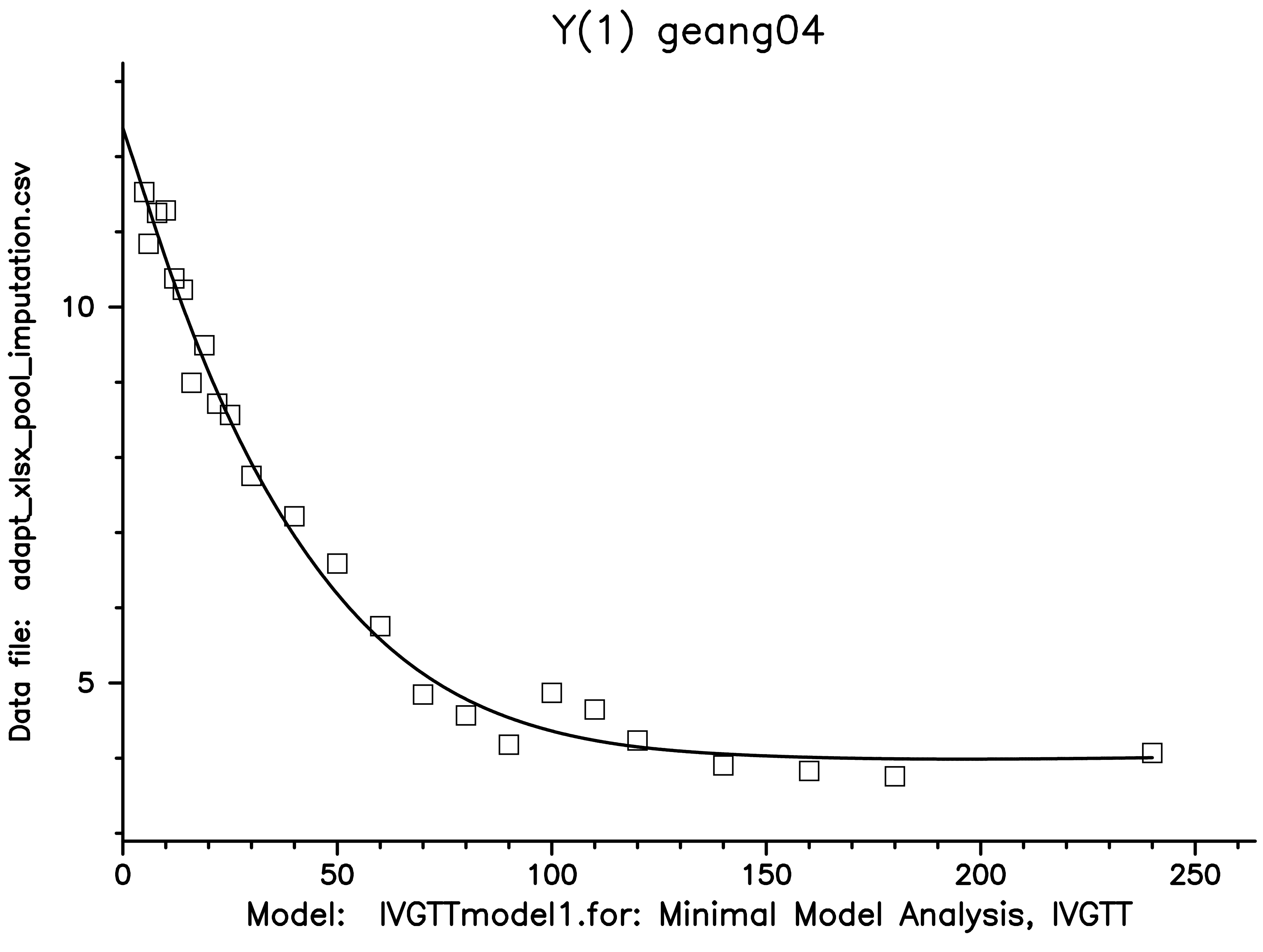
Y(1) geang04
Data file: adapt_xlsx_pool_imputation.csv
10
5
0
50
100
150
200
250
Model: IVGTTmodel1.for: Minimal Model Analysis, IVGTT

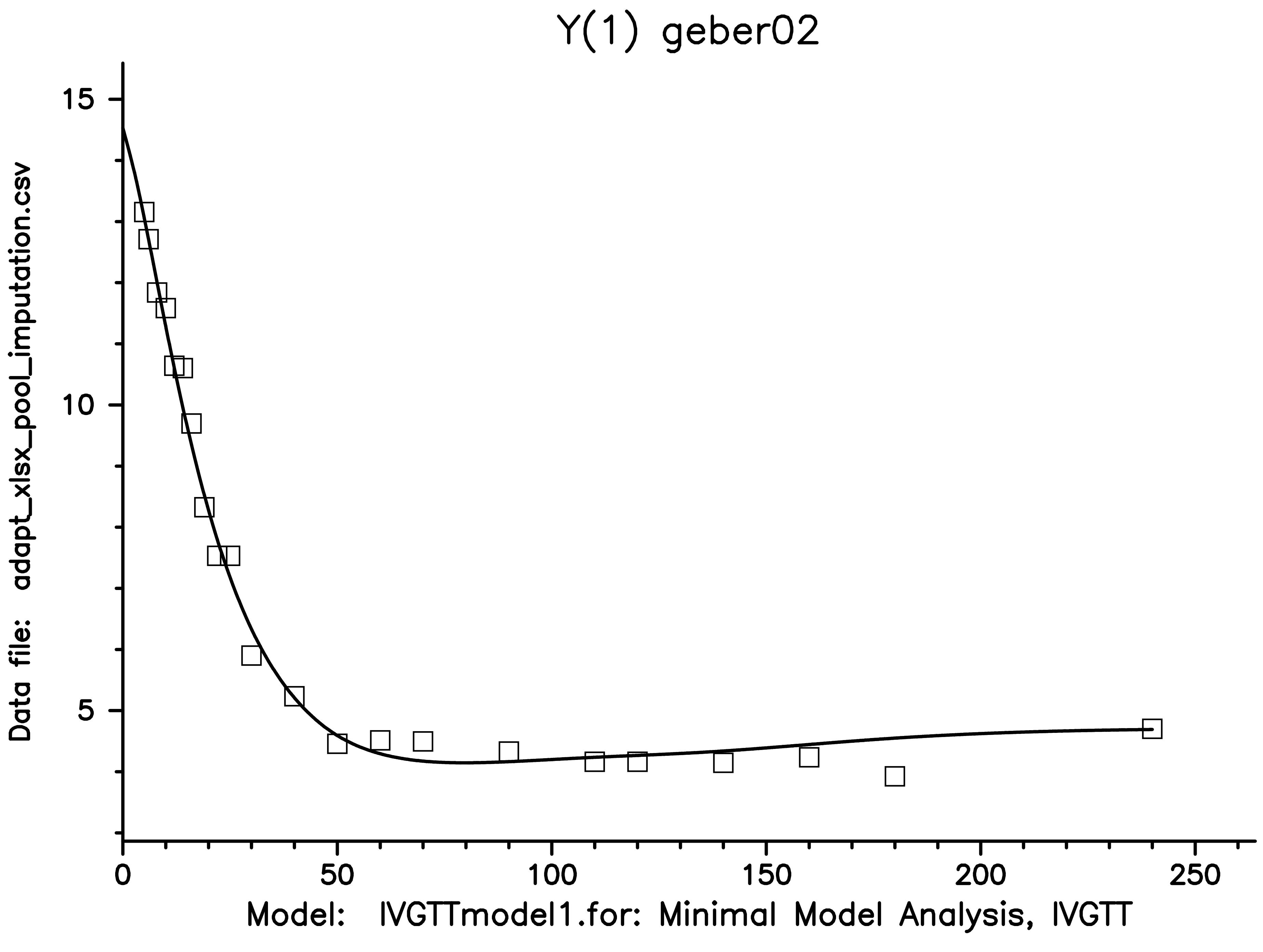
Y(1) geber02
Data file: adapt_xlsx_pool_imputation.csv
15
10
5
0
50
100
150
200
250
Model: IVGTTmodel1.for: Minimal Model Analysis, IVGTT

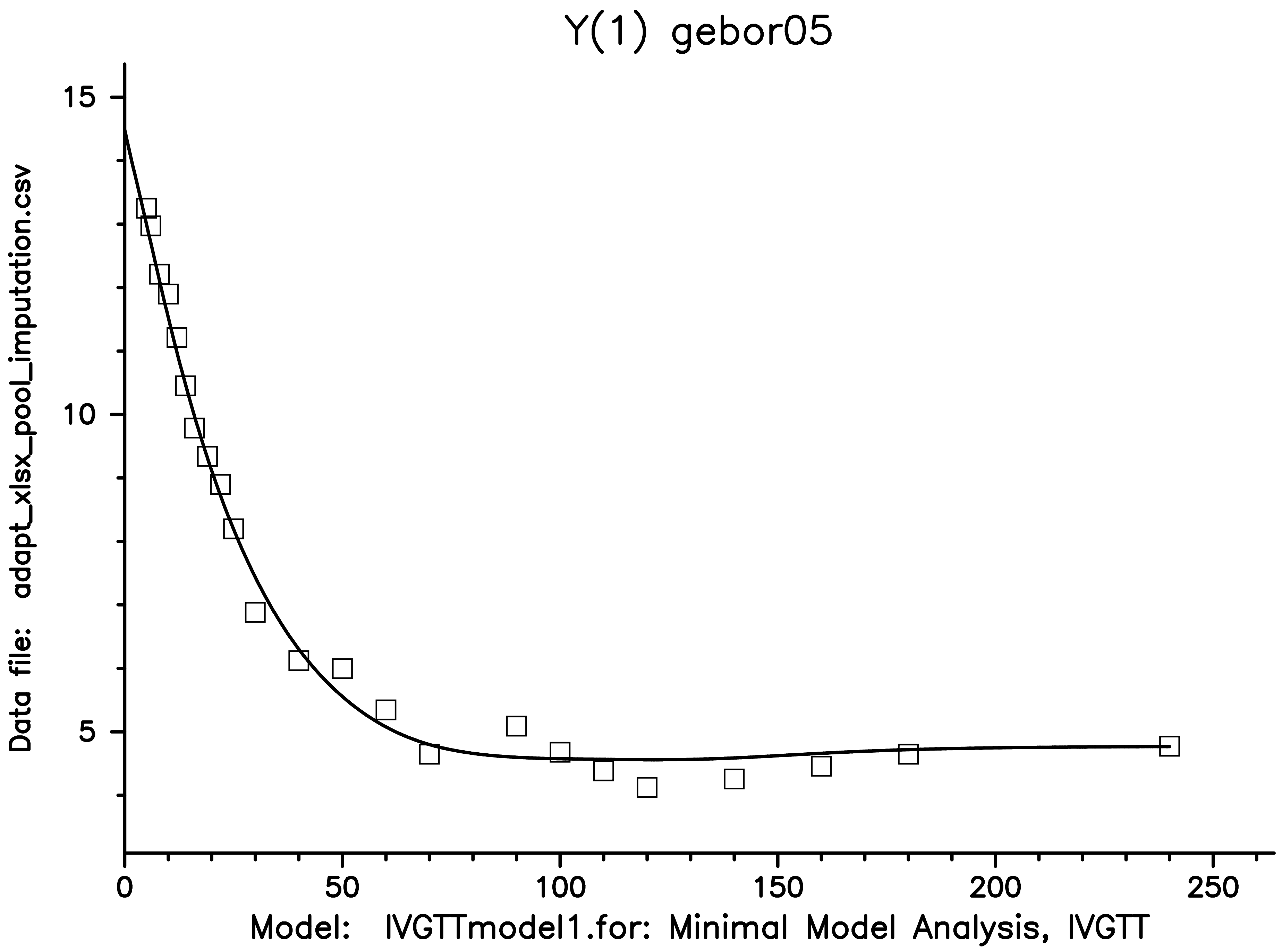

Y(1) gebor05
15
10
5
0
50
100
150
200
250
Data file: adapt_xlsx_pool_imputation.csv
Model: IVGTTmodel1.for: Minimal Model Analysis, IVGTT

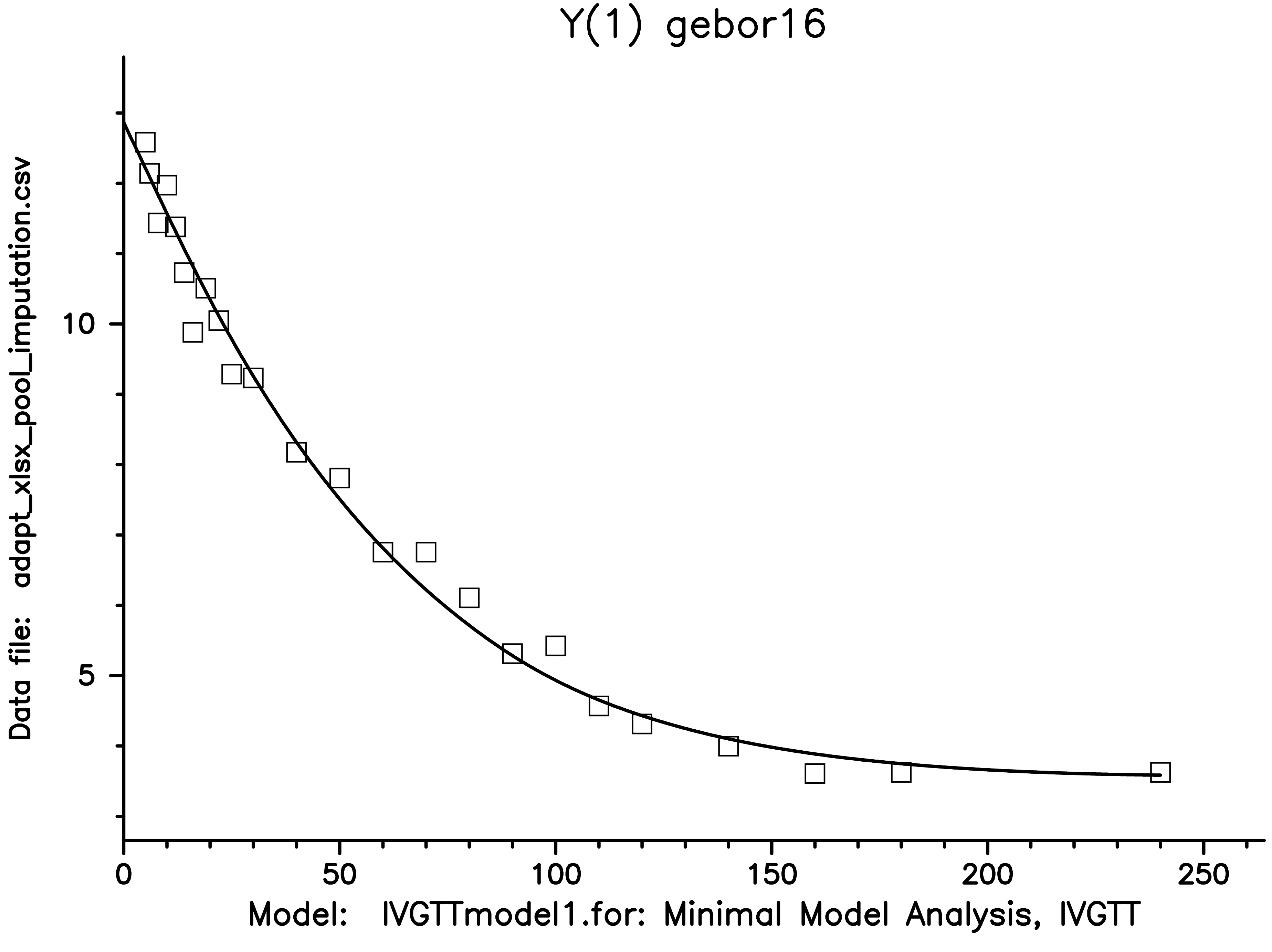
Y(1) gebor16
Data file: adapt_xlsx_pool_imputation.csv
10
5
0
50
100
150
200
250
Model: IVGTTmodel1.for: Minimal Model Analysis, IVGTT

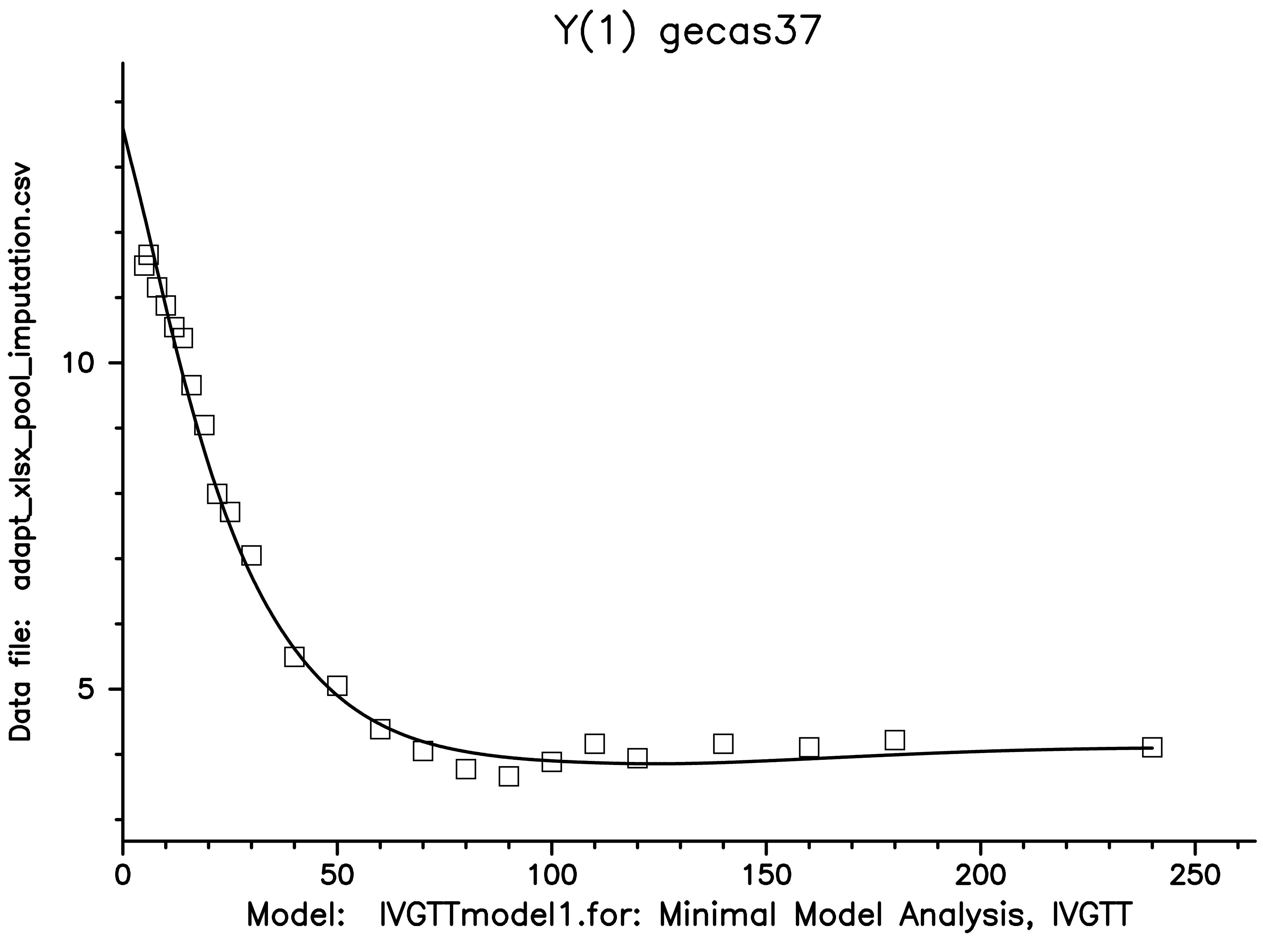
Y(1) gecas37
Data file: adapt_xlsx_pool_imputation.csv
10
5
0
50
100
150
200
250
Model: IVGTTmodel1.for: Minimal Model Analysis, IVGTT

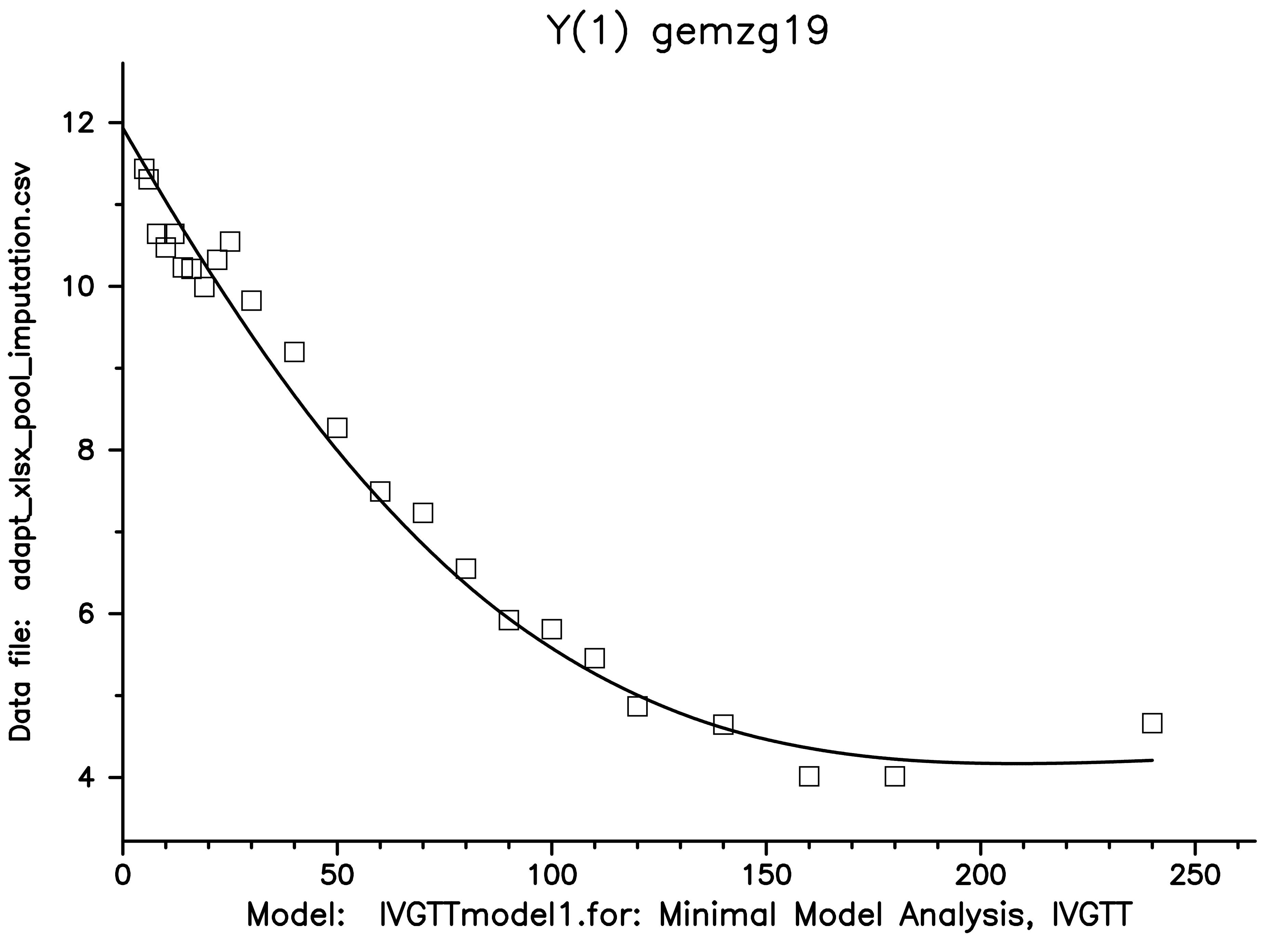
Y(1) gemzg19
Data file: adapt_xlsx_pool_imputation.csv
Model: IVGTTmodel1.for: Minimal Model Analysis, IVGTT
12
10
8
6
4
0
50
100
150
200
250

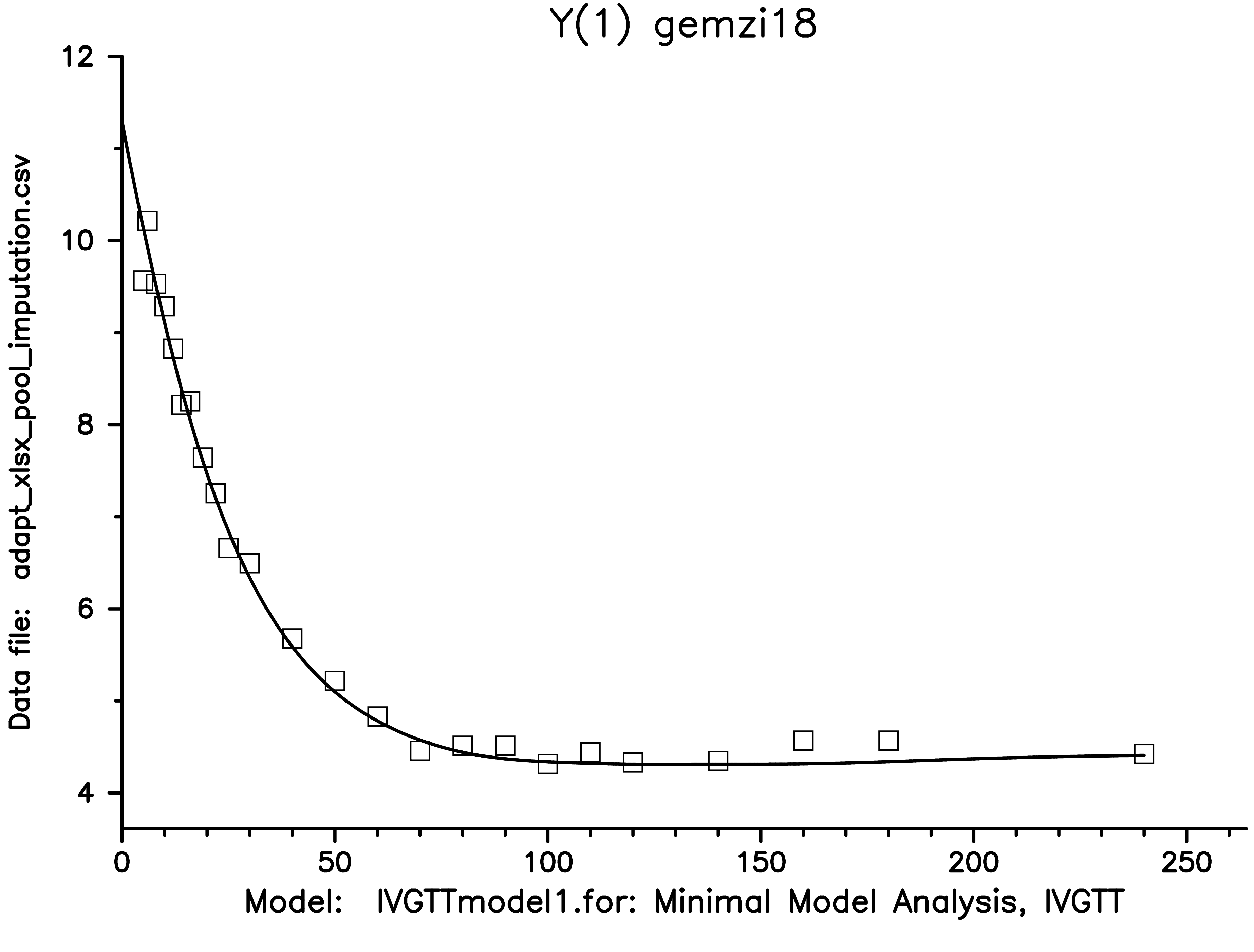
Y(1) gemzi18
Data file: adapt_xlsx_pool_imputation.csv
Model: IVGTTmodel1.for: Minimal Model Analysis, IVGTT
12
10
8
6
4
0
50
100
150
200
250

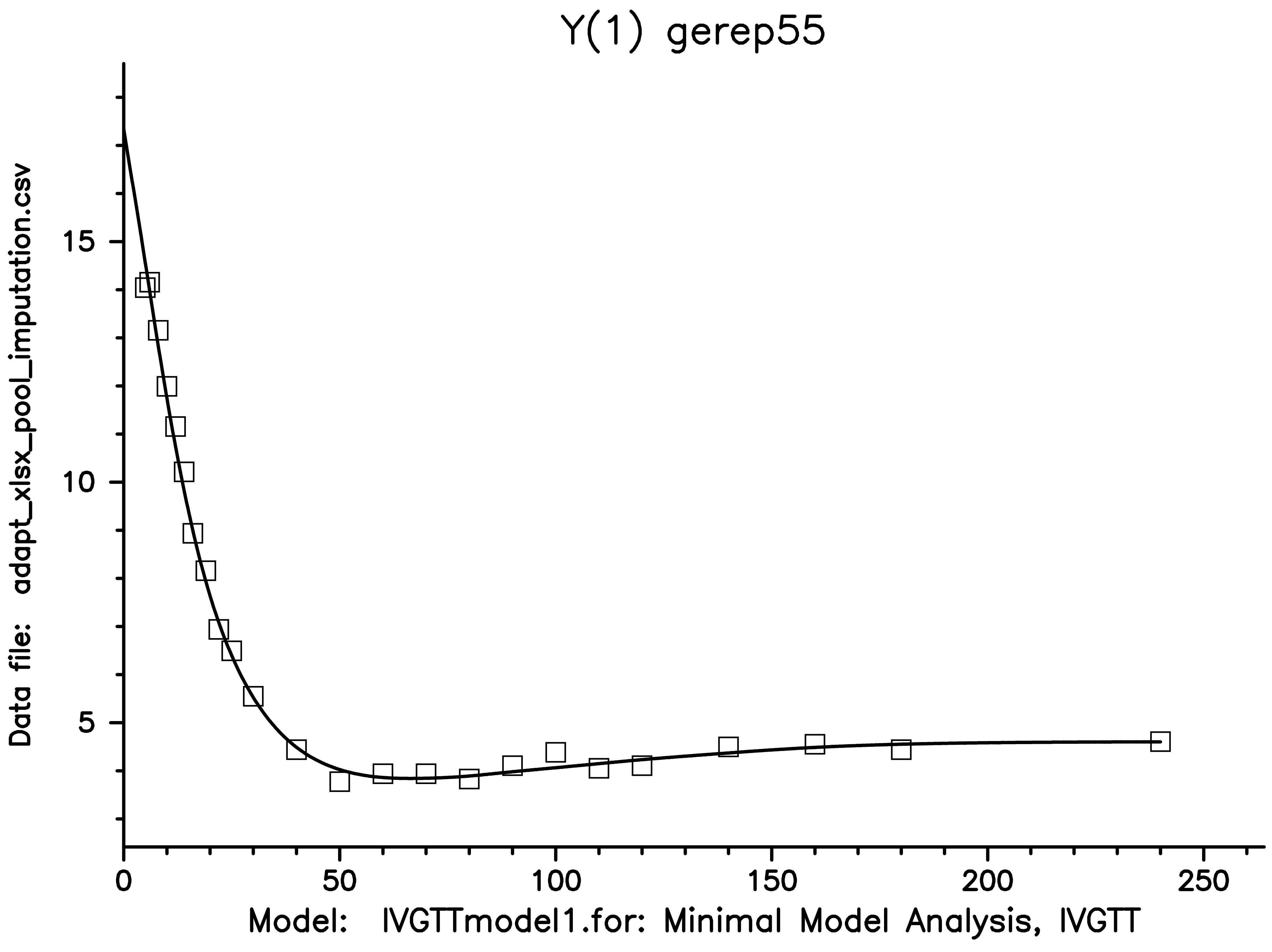
Y(1) gerep55
Data file: adapt_xlsx_pool_imputation.csv
15
10
5
0
50
100
150
200
250
Model: IVGTTmodel1.for: Minimal Model Analysis, IVGTT

Y(1) geros01

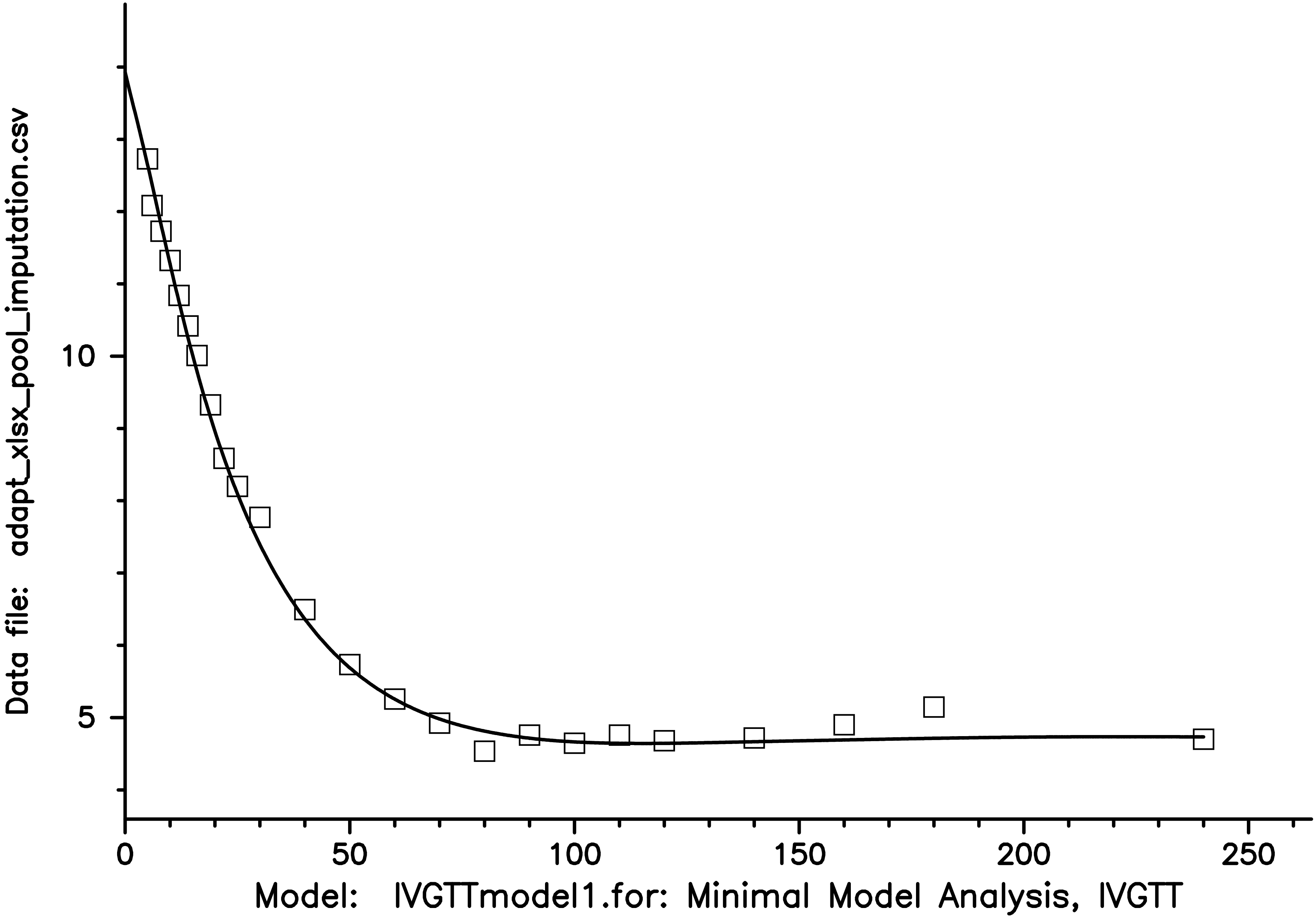

Data file: adapt_xlsx_pool_imputation.csv
10
5
0
50
100
150
200
250
Model: IVGTTmodel1.for: Minimal Model Analysis, IVGTT

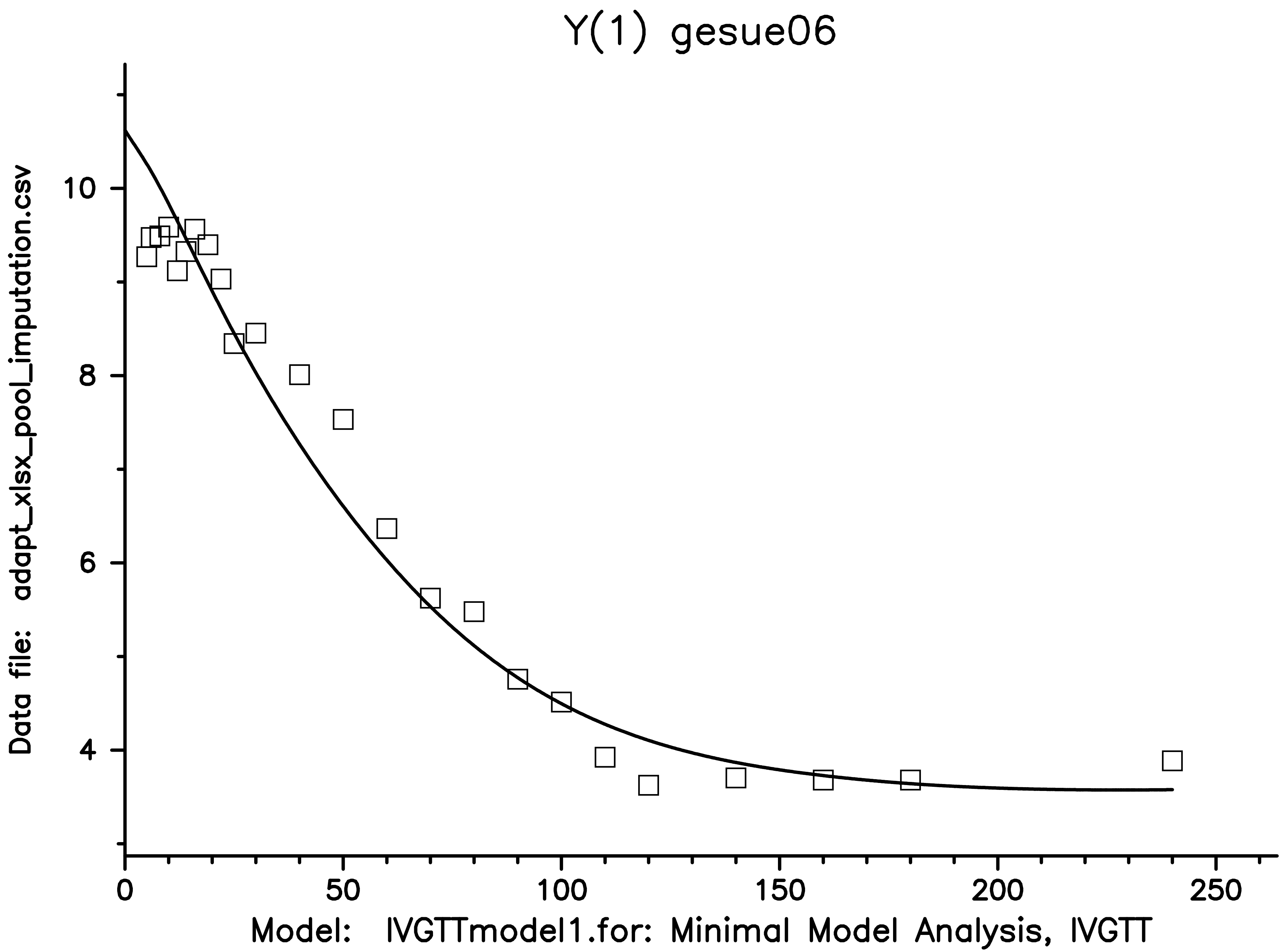
Y(1) gesue06
Data file: adapt_xlsx_pool_imputation.csv
10
8
6
4
0
50
100
150
200
250
Model: IVGTTmodel1.for: Minimal Model Analysis, IVGTT

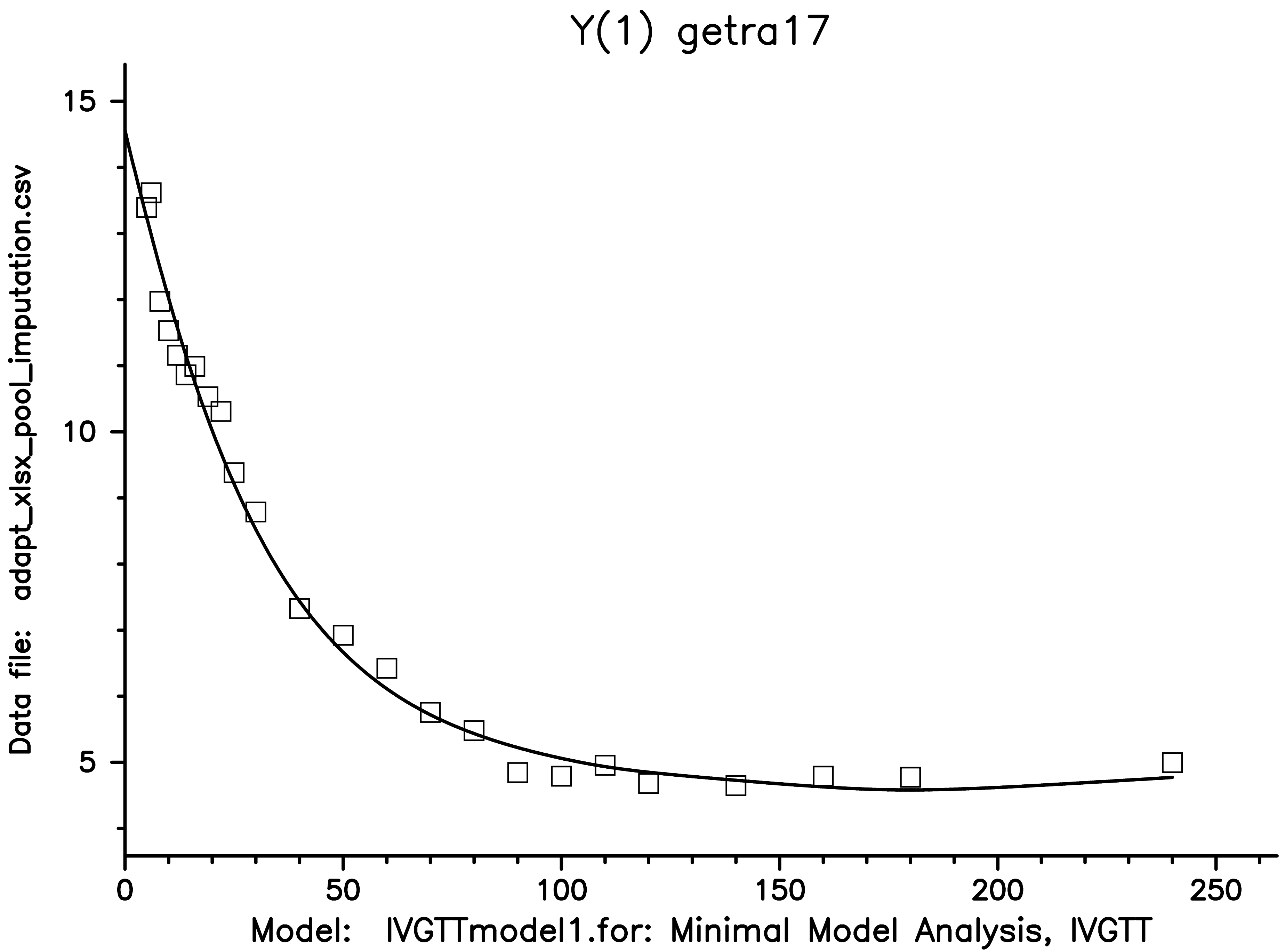
Y(1) getra17
Data file: adapt_xlsx_pool_imputation.csv
Model: IVGTTmodel1.for: Minimal Model Analysis, IVGTT
15
10
5
0
50
100
150
200
250

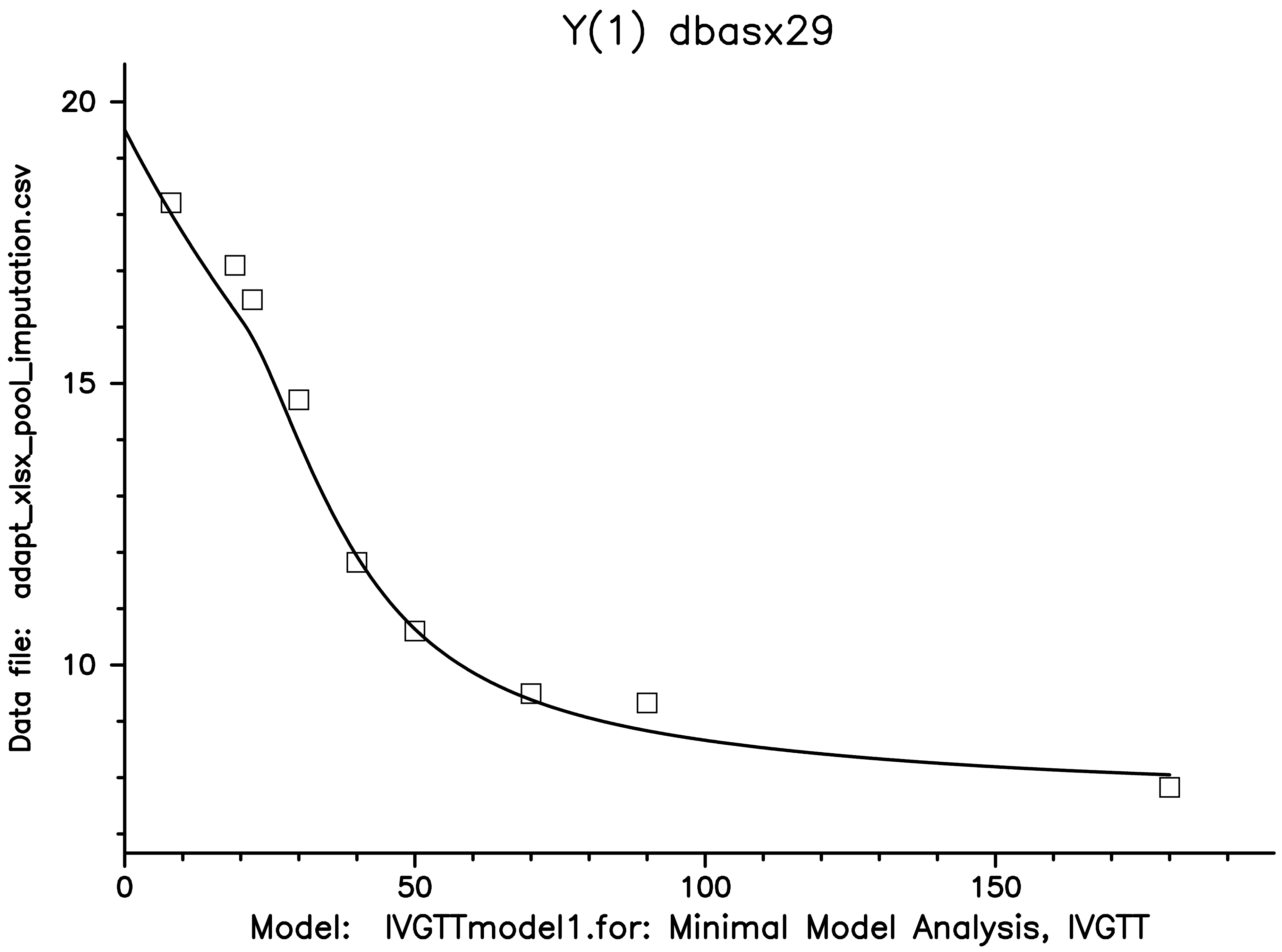
Y(1) dbasx29
Data file: adapt_xlsx_pool_imputation.csv
20
15
10
0
50
100
150
Model: IVGTTmodel1.for: Minimal Model Analysis, IVGTT

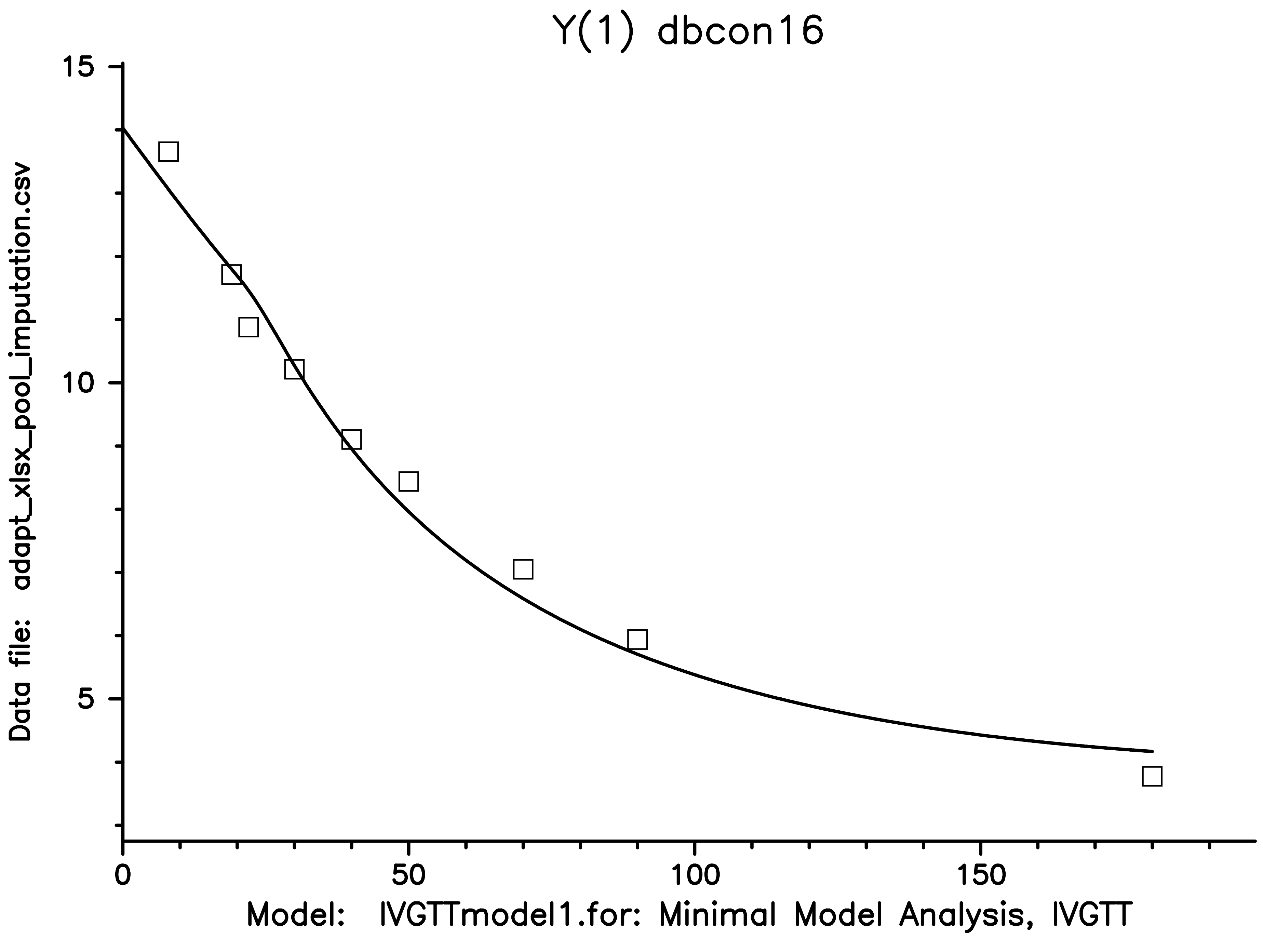

Y(1) dbcon16
Data file: adapt_xlsx_pool_imputation.csv
15
10
5
0
50
100
150
Model: IVGTTmodel1.for: Minimal Model Analysis, IVGTT

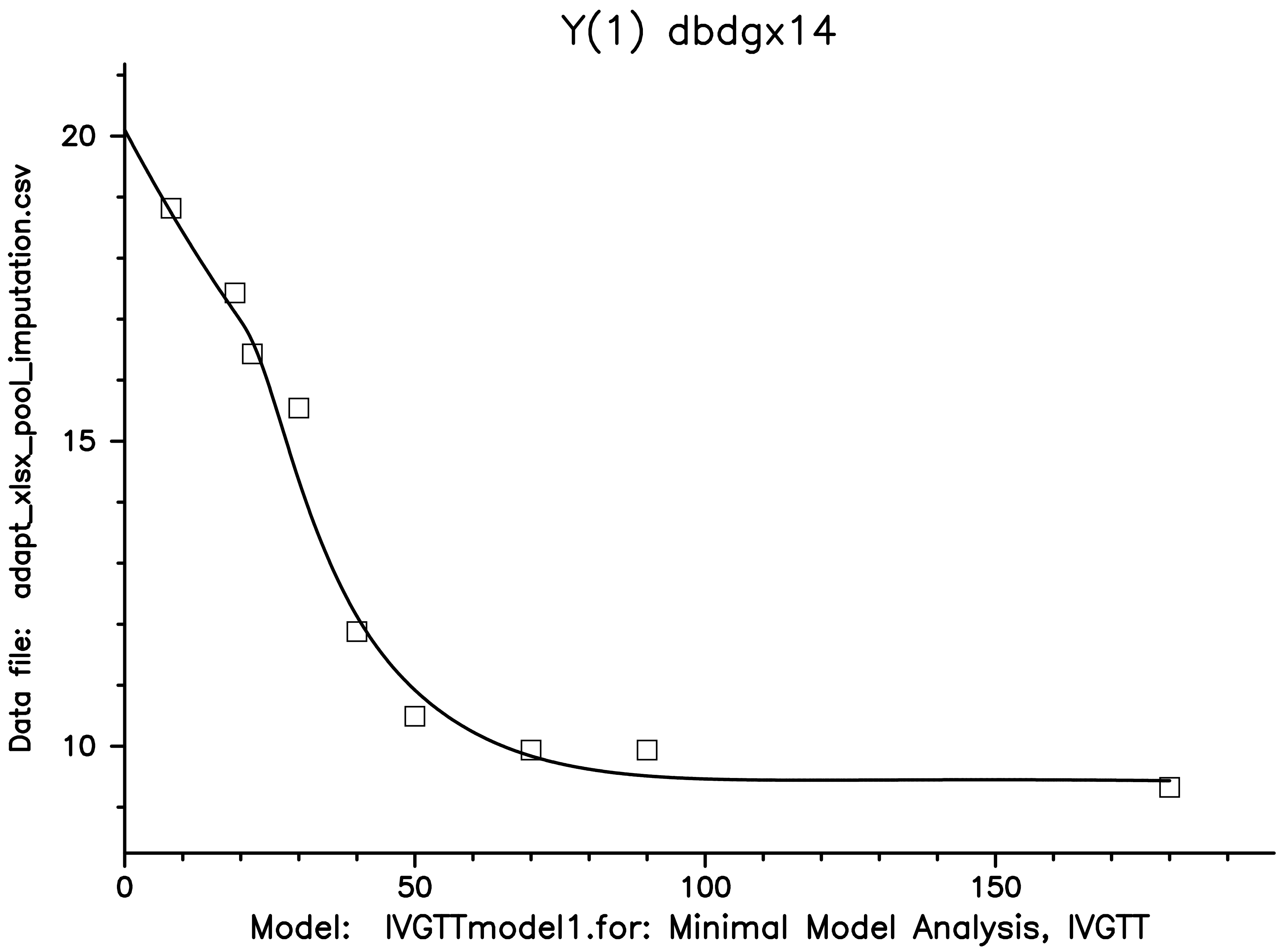
Y(1) dbdgx14
Data file: adapt_xlsx_pool_imputation.csv
Model: IVGTTmodel1.for: Minimal Model Analysis, IVGTT
20
15
10
0
50
100
150

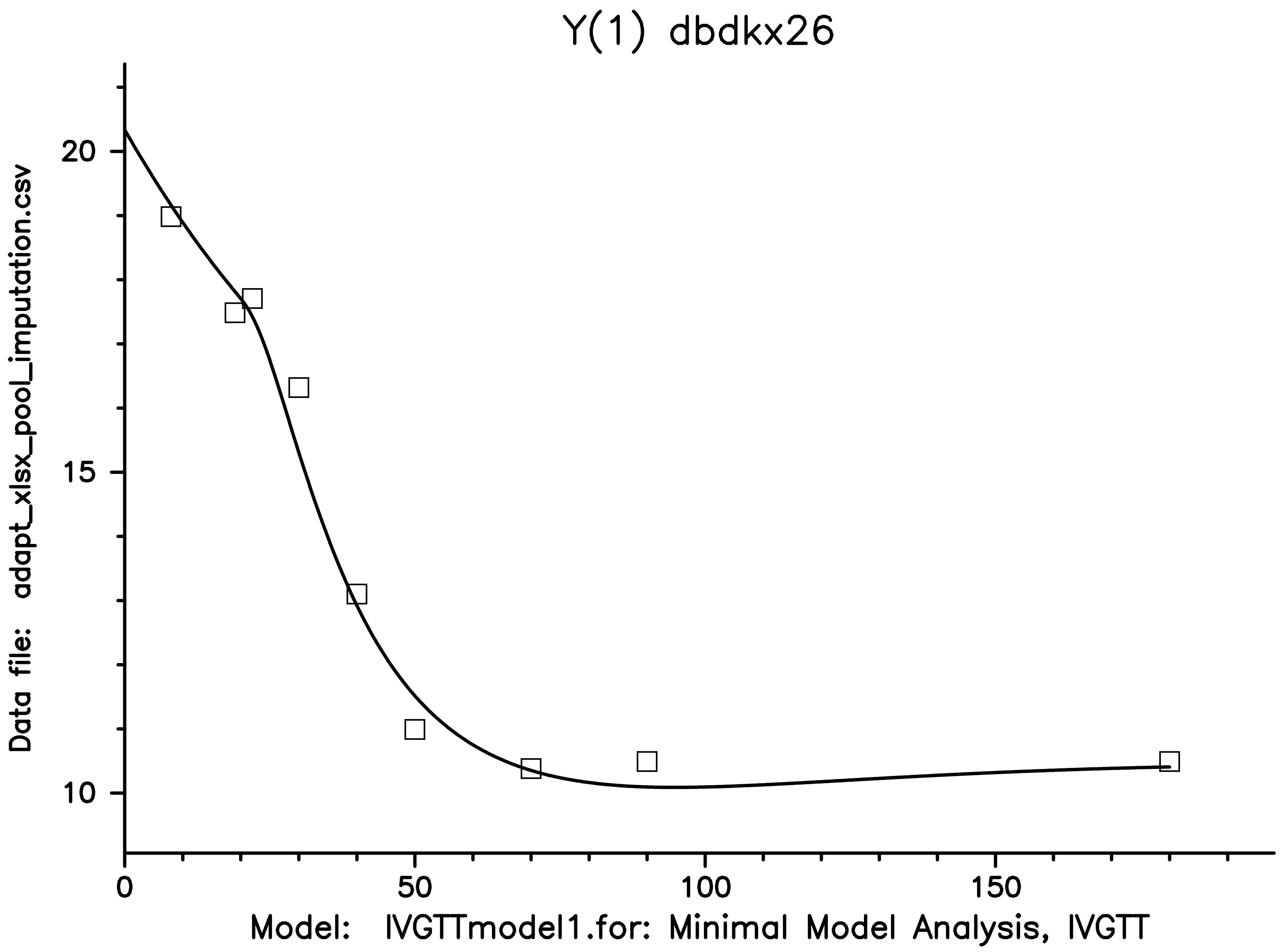

Y(1) dbdkx26
Data file: adapt_xlsx_pool_imputation.csv
20
15
10
0
50
100
150
Model: IVGTTmodel1.for: Minimal Model Analysis, IVGTT

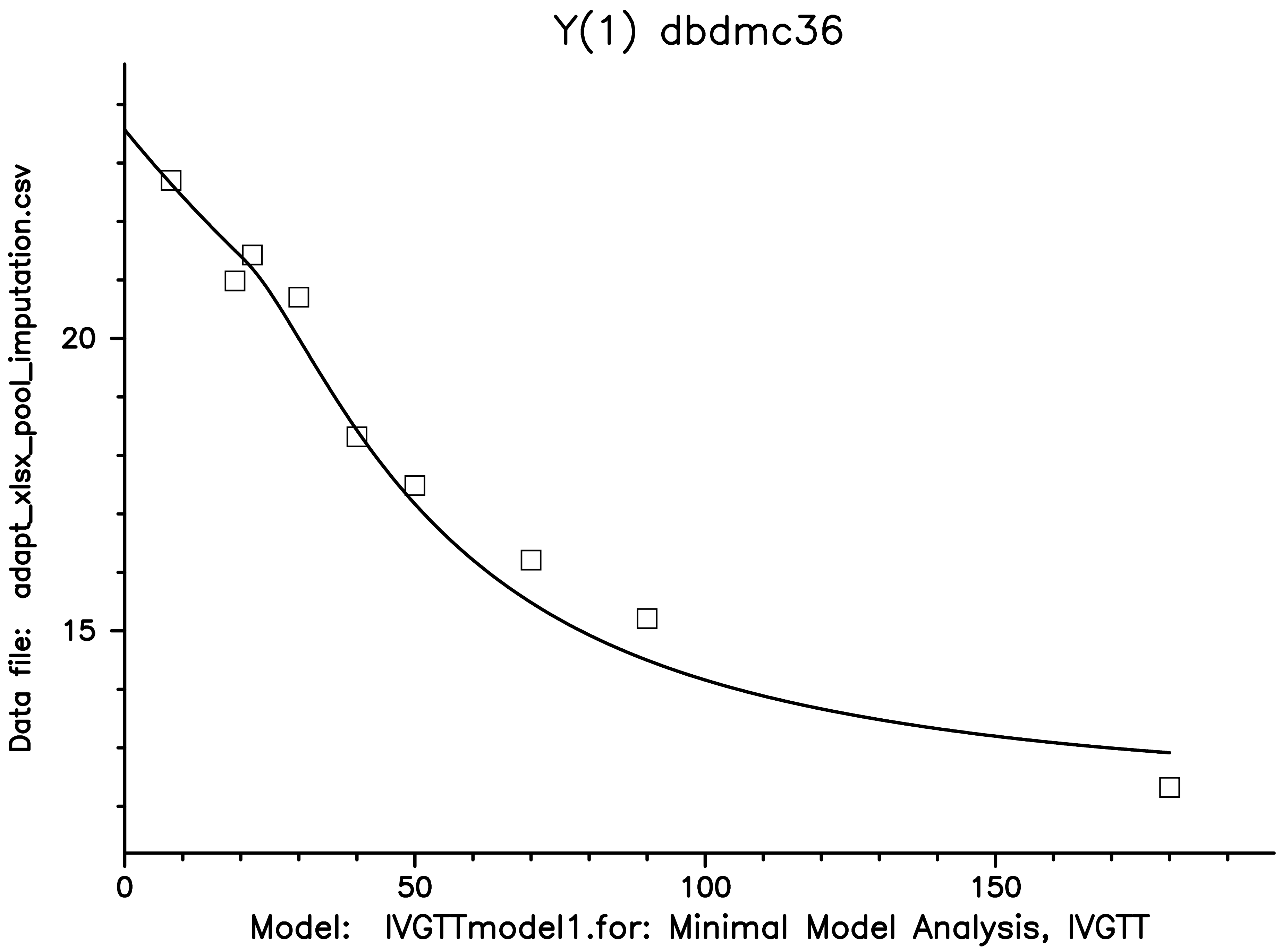

Y(1) dbdmc36
Data file: adapt_xlsx_pool_imputation.csv
20
15
0
50
100
150
Model: IVGTTmodel1.for: Minimal Model Analysis, IVGTT

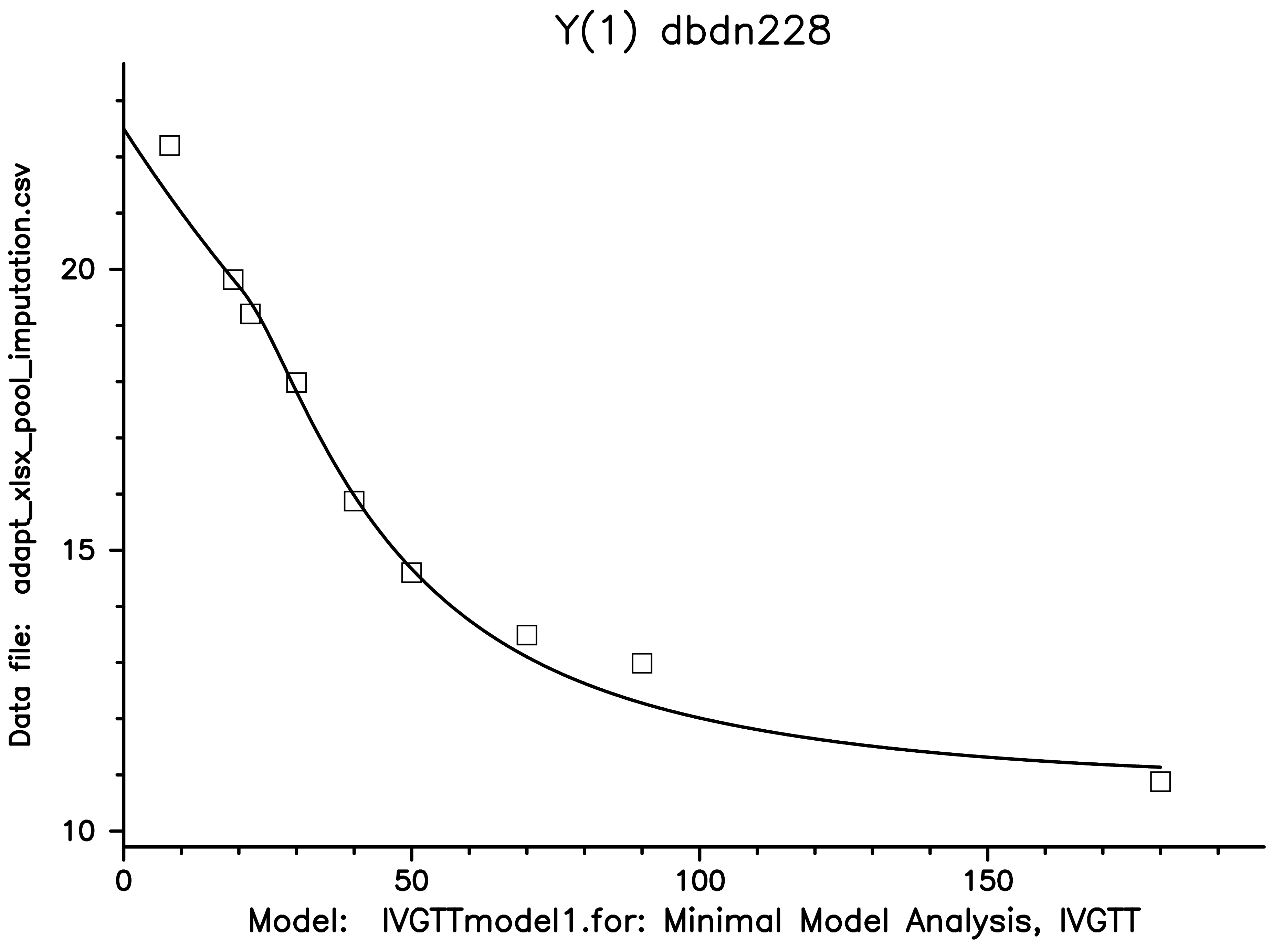
Y(1) dbdn228
Data file: adapt_xlsx_pool_imputation.csv
20
15
10
0
50
100
150
Model: IVGTTmodel1.for: Minimal Model Analysis, IVGTT

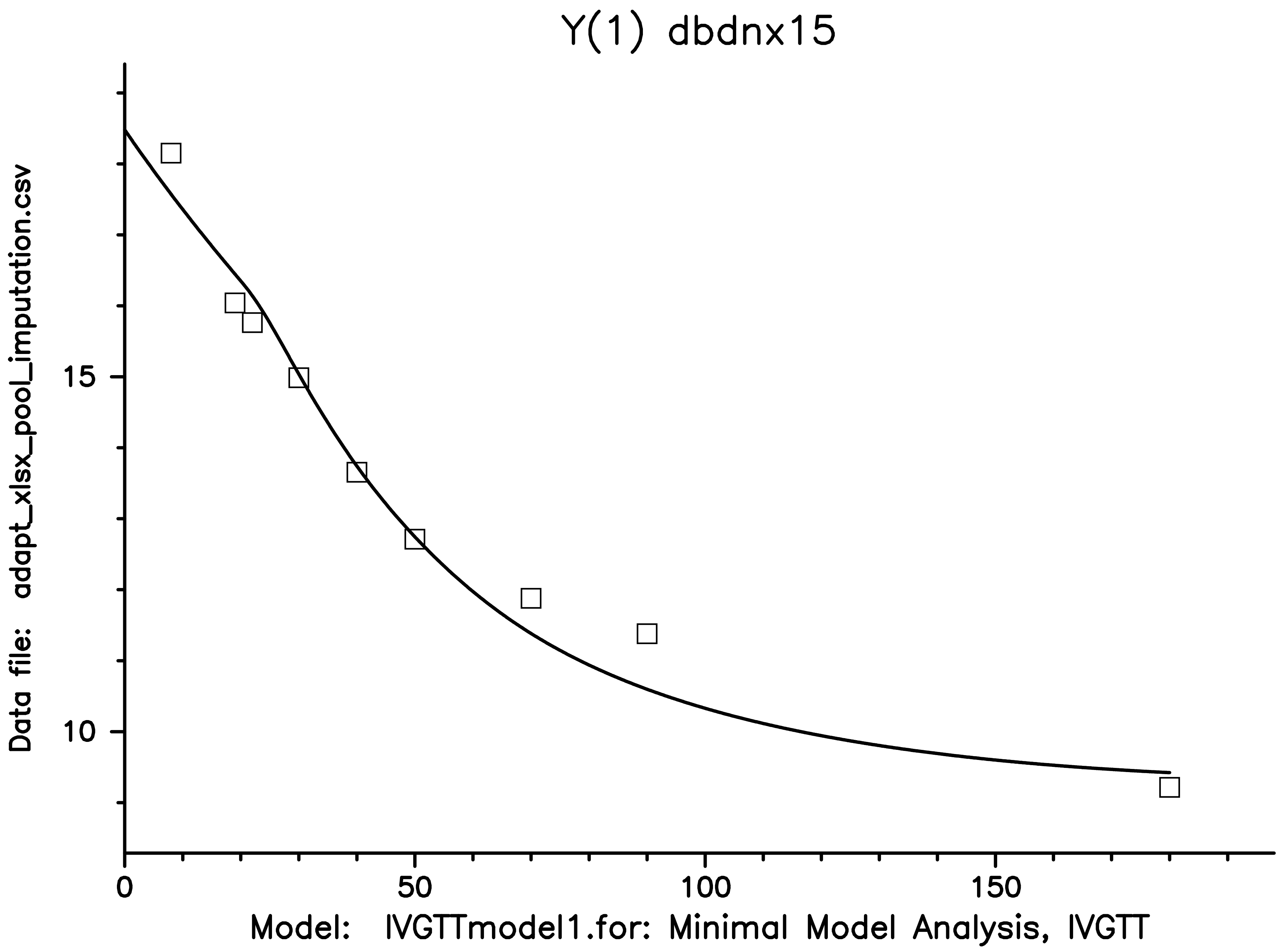

Y(1) dbdnx15
Data file: adapt_xlsx_pool_imputation.csv
15
10
0
50
100
150
Model: IVGTTmodel1.for: Minimal Model Analysis, IVGTT

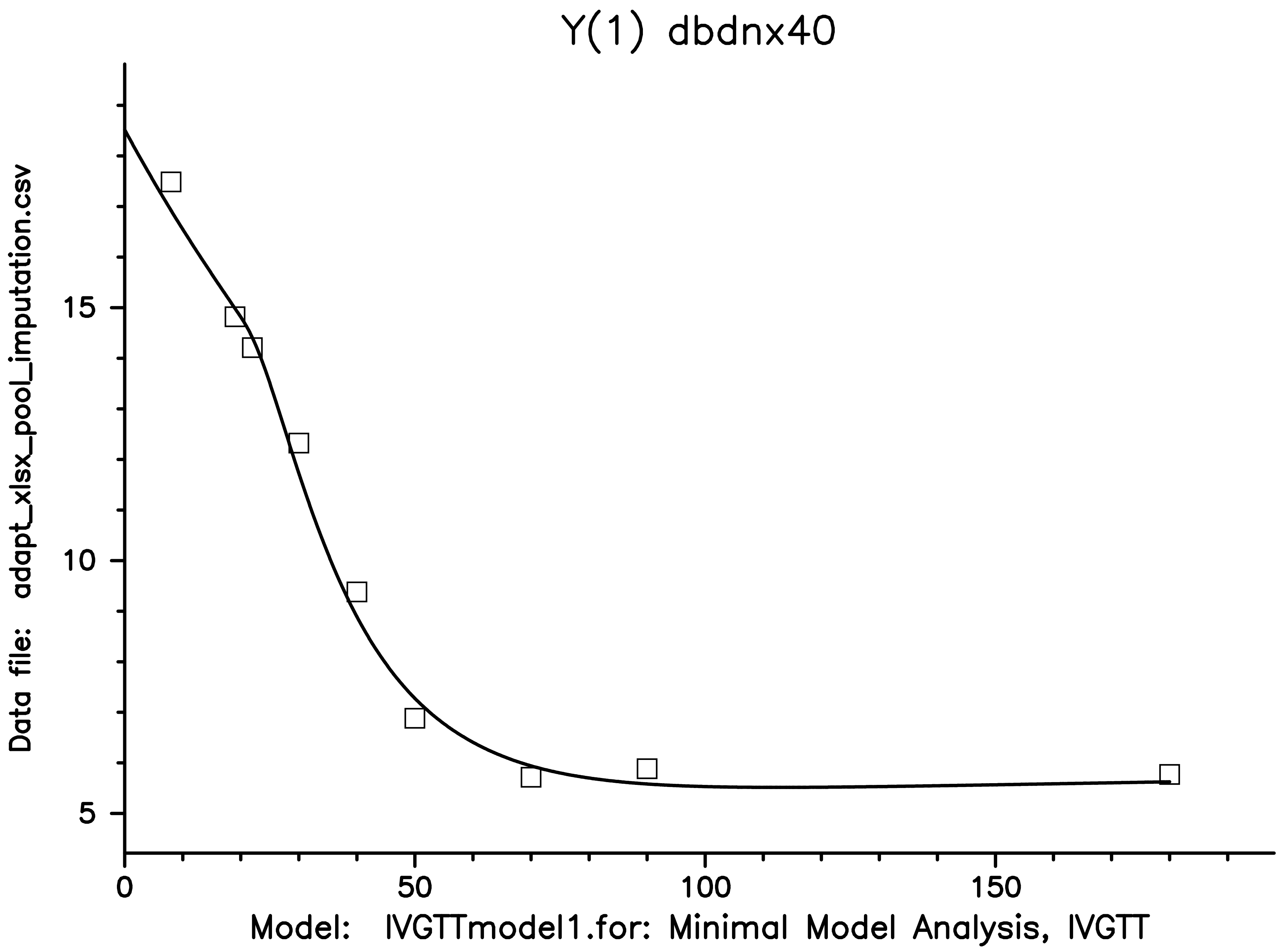

Y(1) dbdnx40
Data file: adapt_xlsx_pool_imputation.csv
15
10
5
0
50
100
150
Model: IVGTTmodel1.for: Minimal Model Analysis, IVGTT

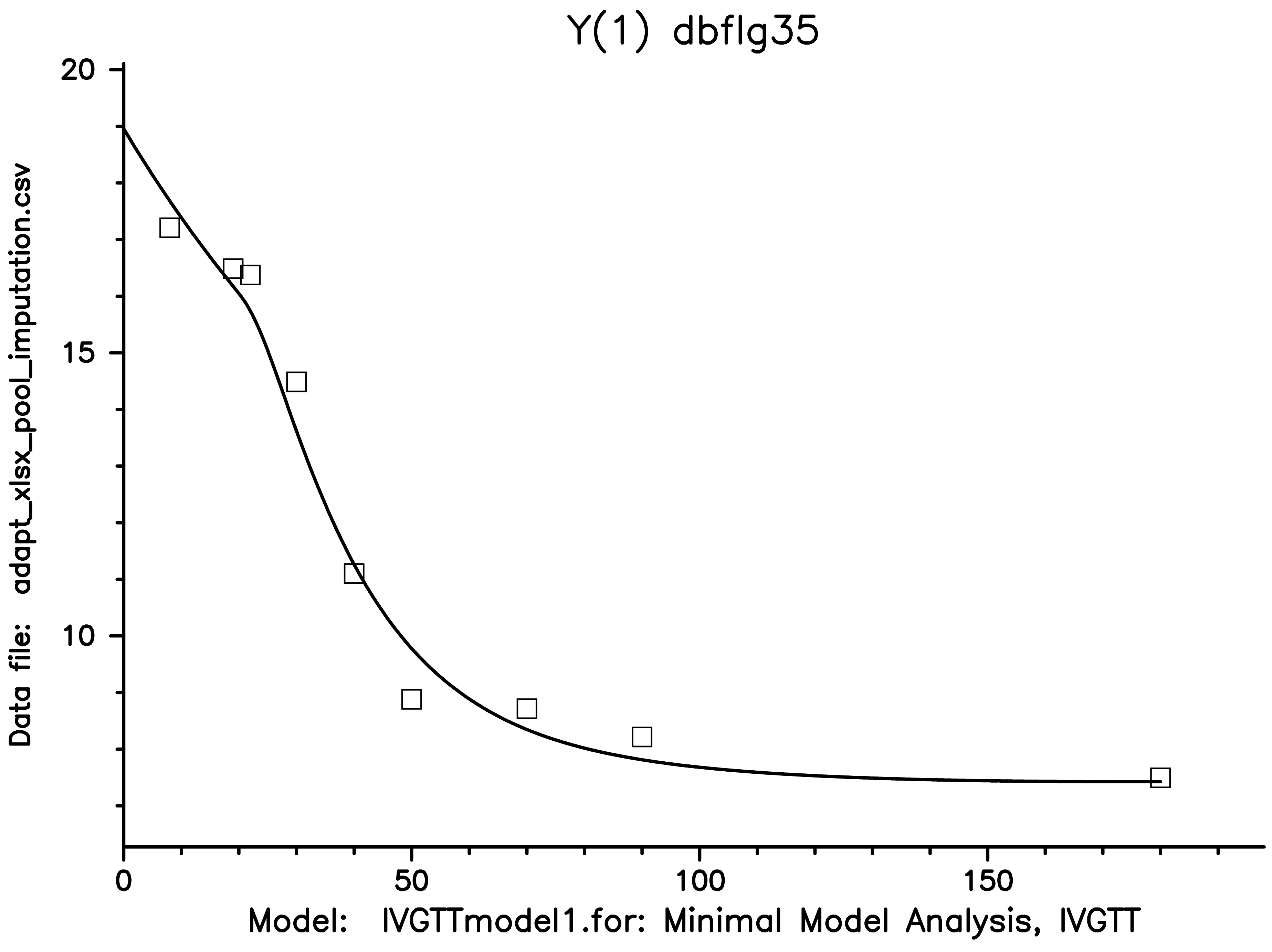

Y(1) dbflg35
20
15
10
0
50
100
150
Data file: adapt_xlsx_pool_imputation.csv
Model: IVGTTmodel1.for: Minimal Model Analysis, IVGTT

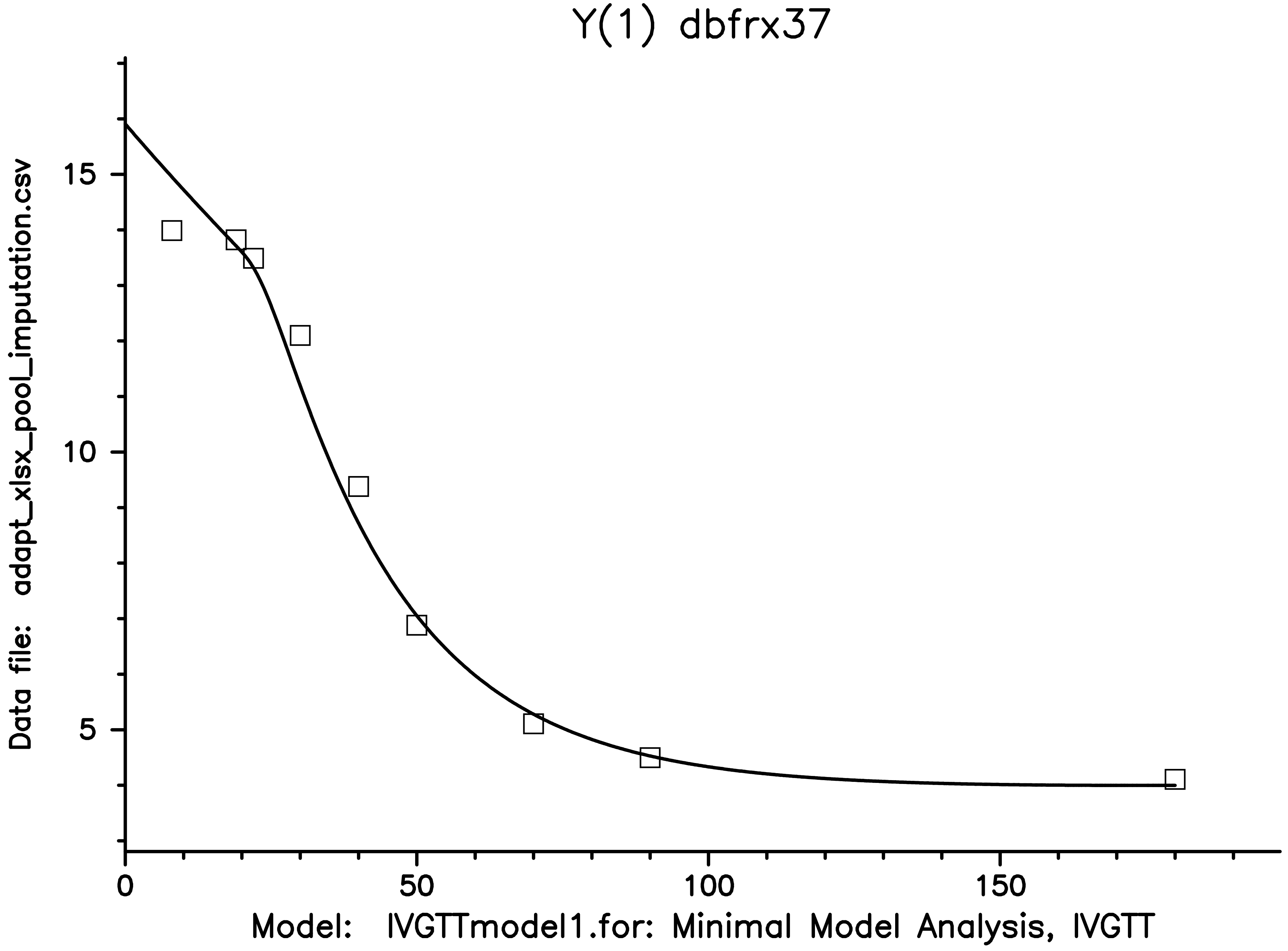
Y(1) dbfrx37
Data file: adapt_xlsx_pool_imputation.csv
15
10
5
0
50
100
150
Model: IVGTTmodel1.for: Minimal Model Analysis, IVGTT

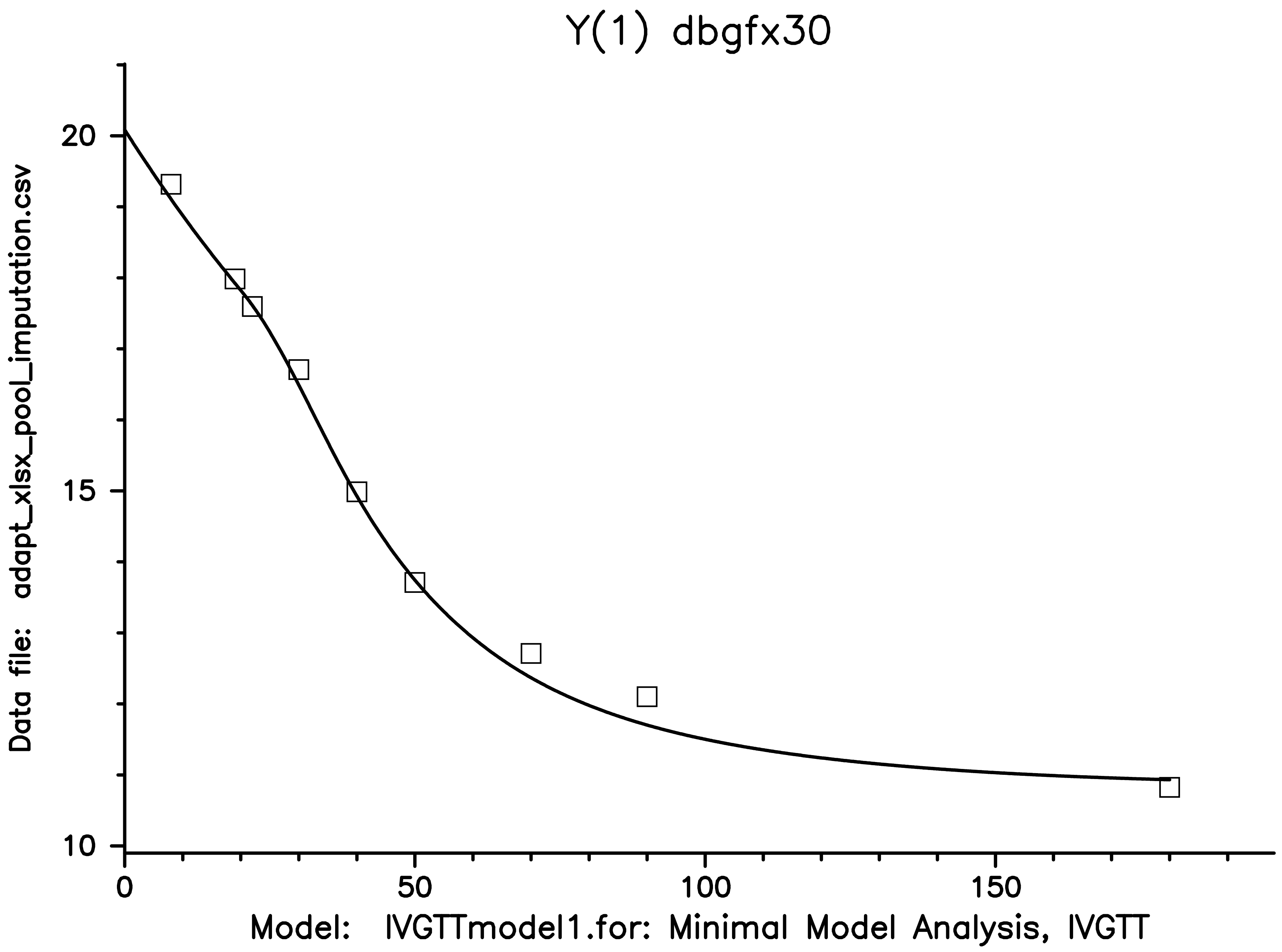

Y(1) dbgfx30
Data file: adapt_xlsx_pool_imputation.csv
20
15
10
0
50
100
150
Model: IVGTTmodel1.for: Minimal Model Analysis, IVGTT

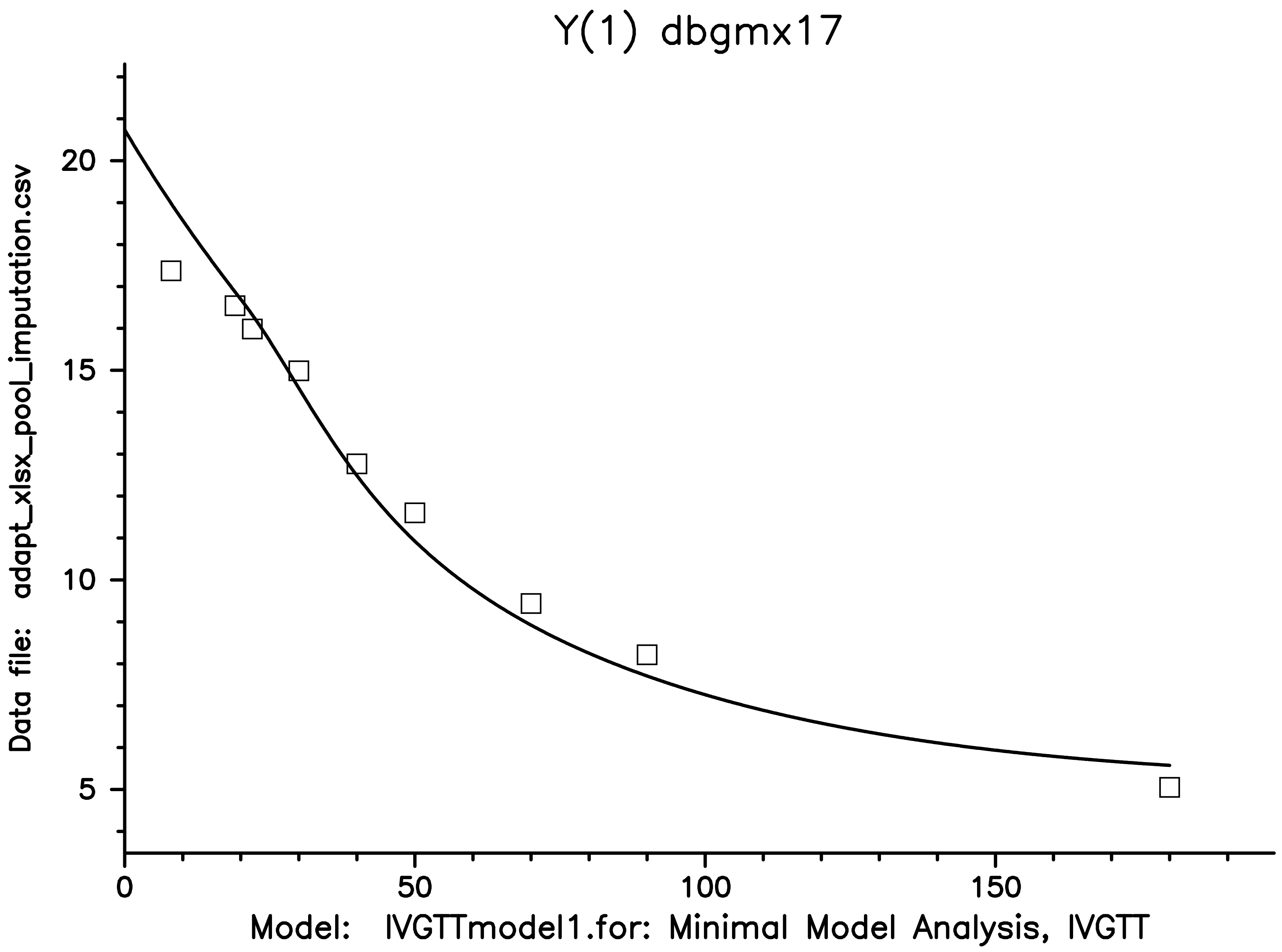
Y(1) dbgmx17
Data file: adapt_xlsx_pool_imputation.csv
20
15
10
5
0
50
100
150
Model: IVGTTmodel1.for: Minimal Model Analysis, IVGTT

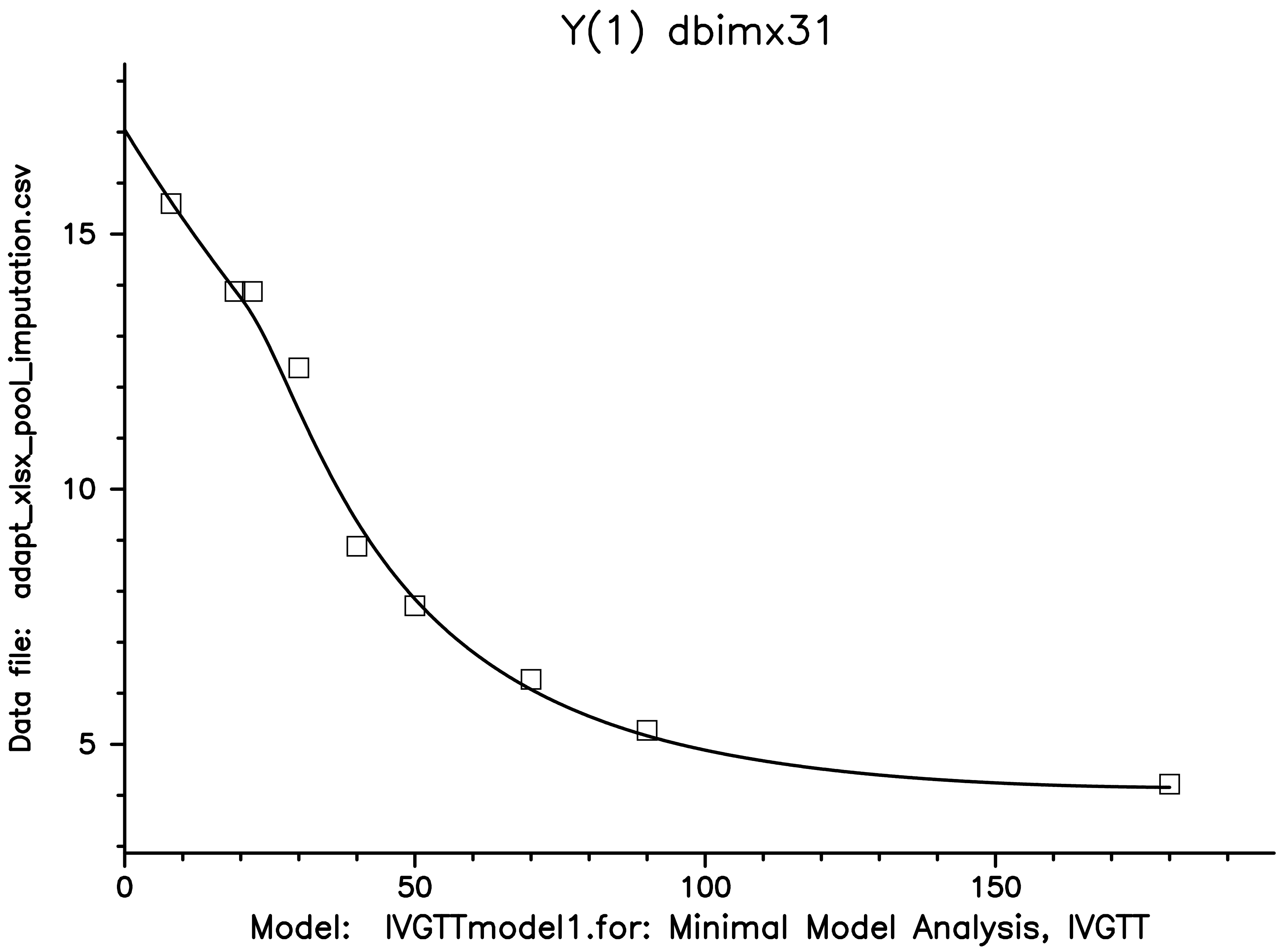
Y(1) dbimx31
Data file: adapt_xlsx_pool_imputation.csv
15
10
5
0
50
100
150
Model: IVGTTmodel1.for: Minimal Model Analysis, IVGTT

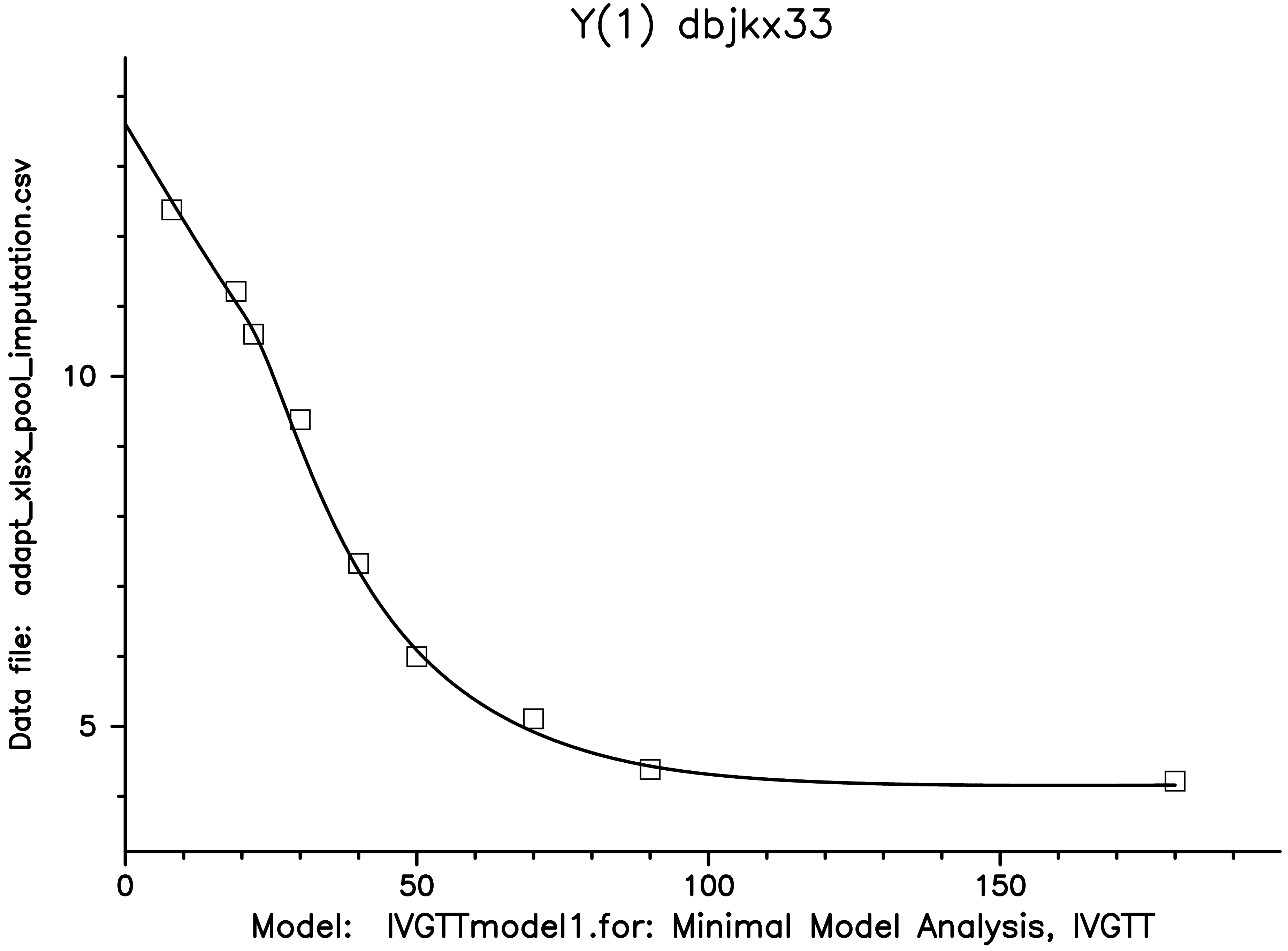

Y(1) dbjkx33
Data file: adapt_xlsx_pool_imputation.csv
10
5
0
50
100
150
Model: IVGTTmodel1.for: Minimal Model Analysis, IVGTT

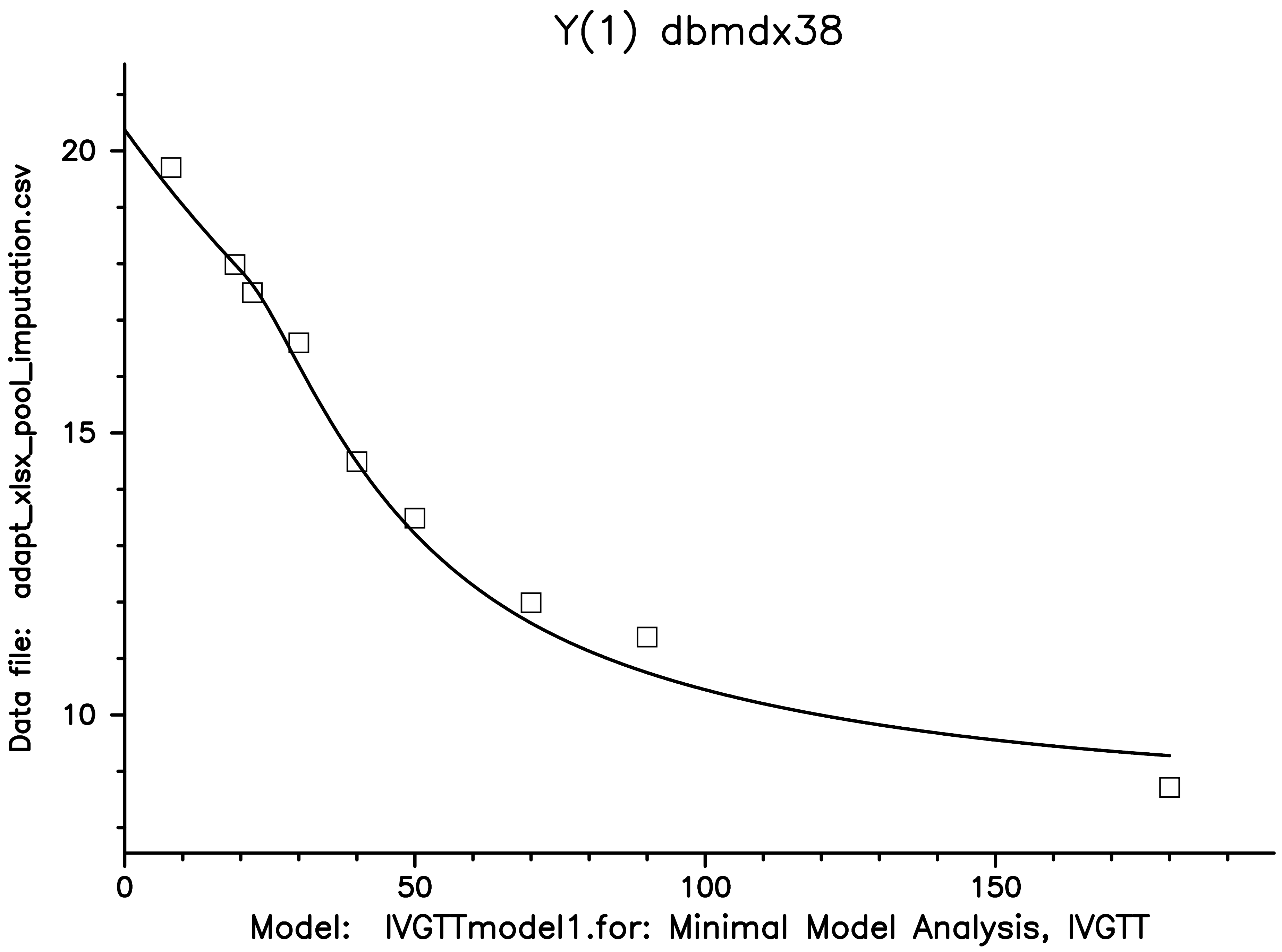
Y(1) dbmdx38
Data file: adapt_xlsx_pool_imputation.csv
20
15
10
0
50
100
150
Model: IVGTTmodel1.for: Minimal Model Analysis, IVGTT

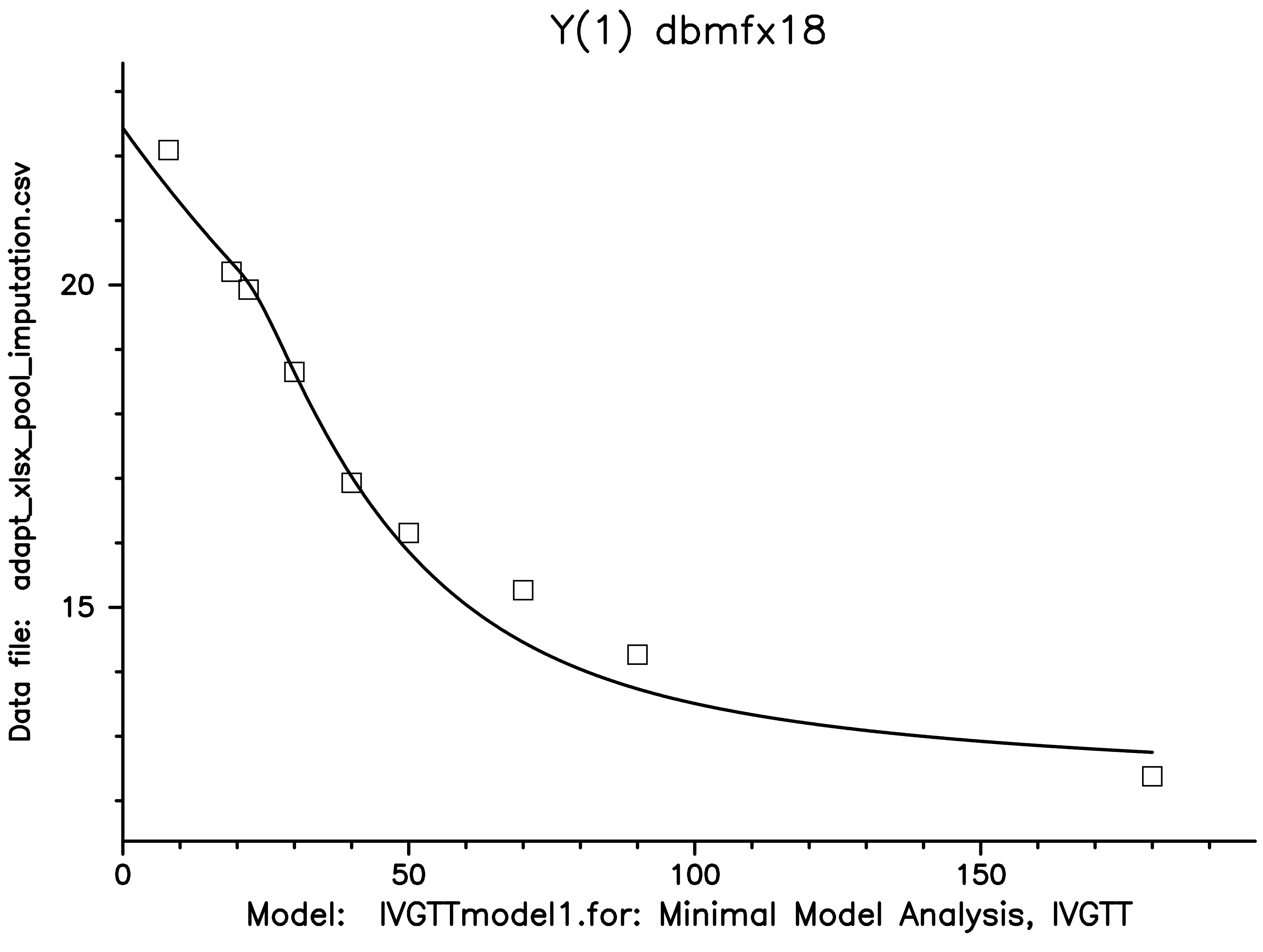

Y(1) dbmfx18
Data file: adapt_xlsx_pool_imputation.csv
20
15
0
50
100
150
Model: IVGTTmodel1.for: Minimal Model Analysis, IVGTT

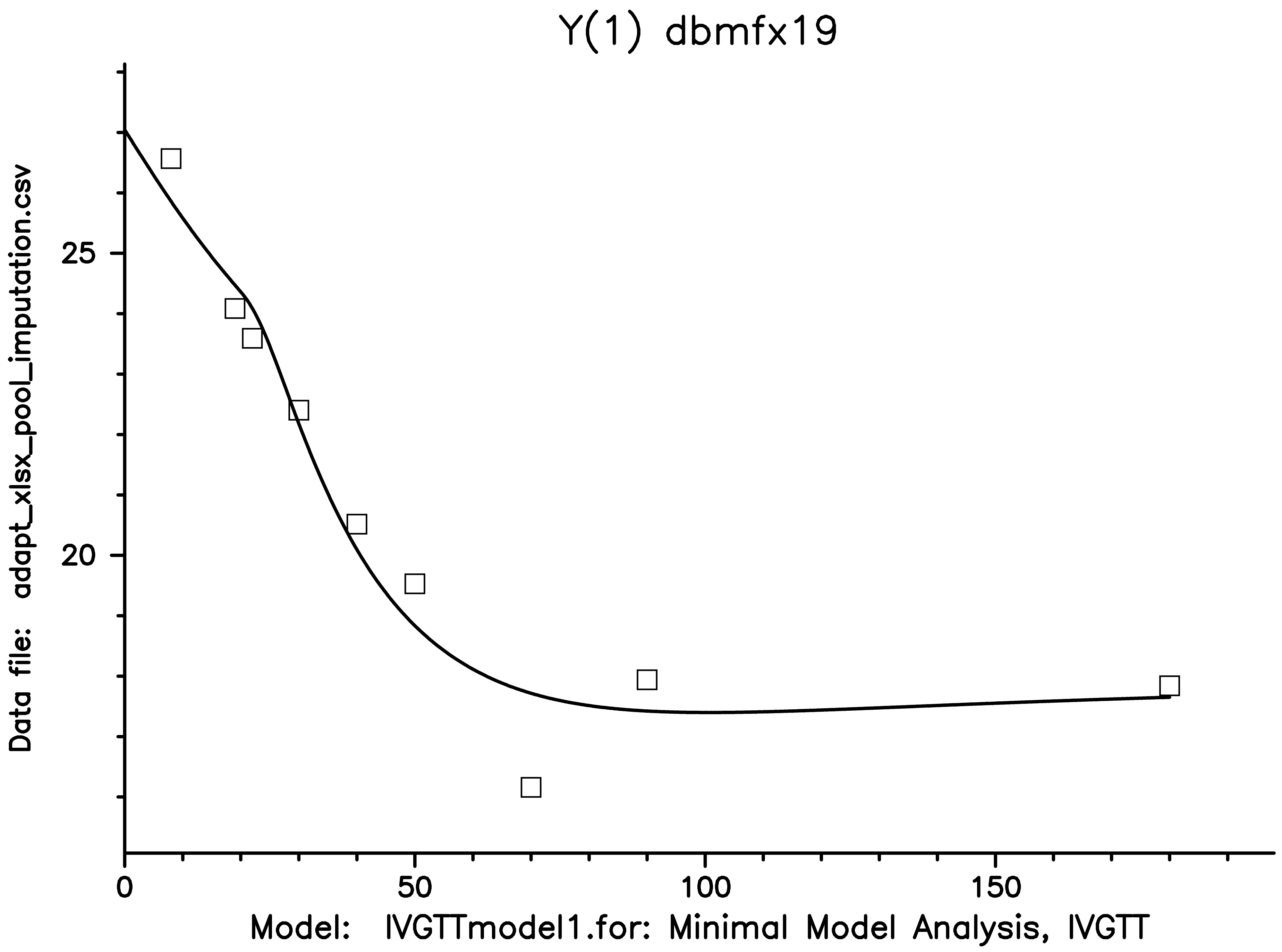
Y(1) dbmfx19
Data file: adapt_xlsx_pool_imputation.csv
25
20
0
50
100
150
Model: IVGTTmodel1.for: Minimal Model Analysis, IVGTT

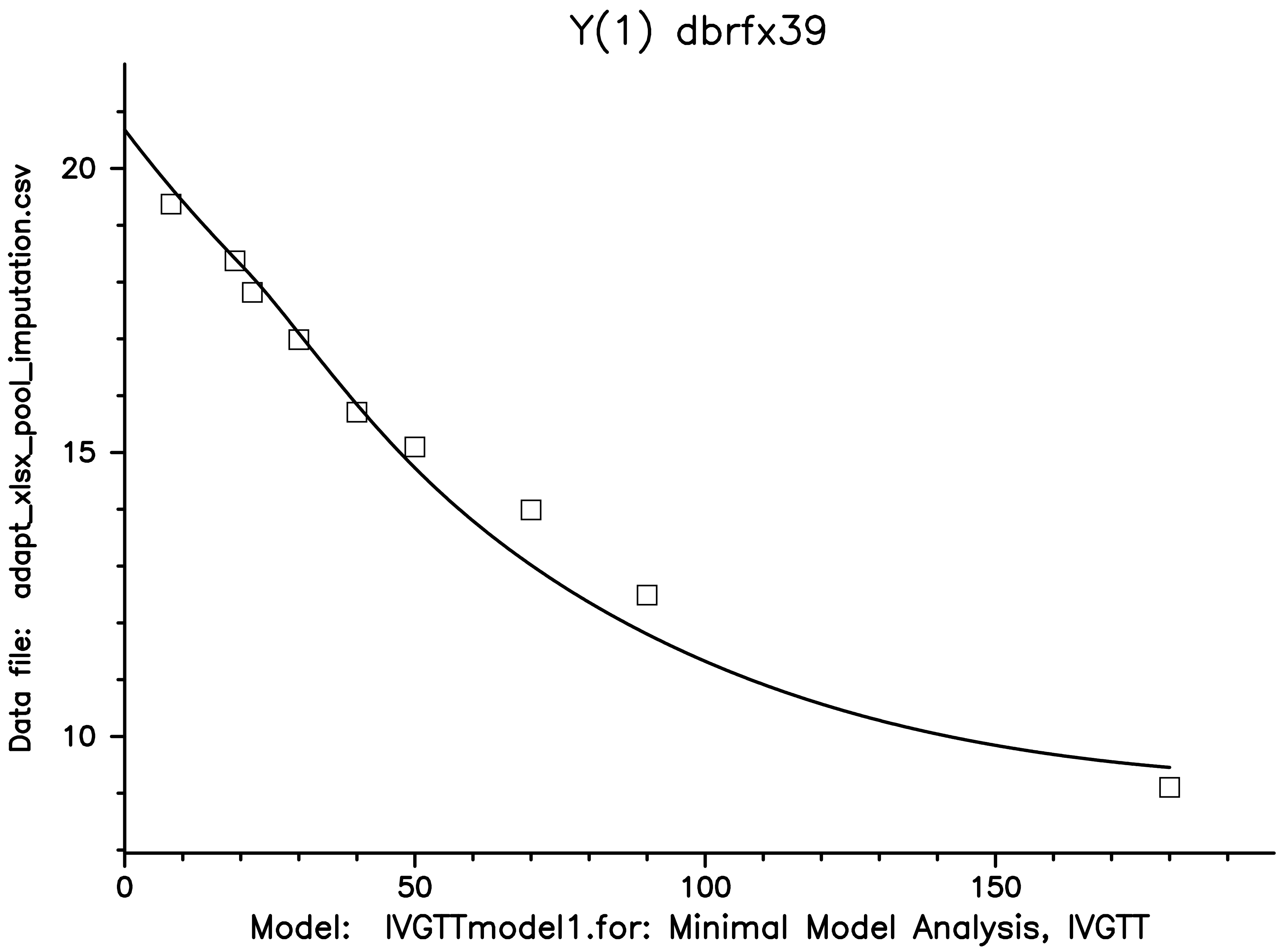
Y(1) dbrfx39
Data file: adapt_xlsx_pool_imputation.csv
20
15
10
0
50
100
150
Model: IVGTTmodel1.for: Minimal Model Analysis, IVGTT

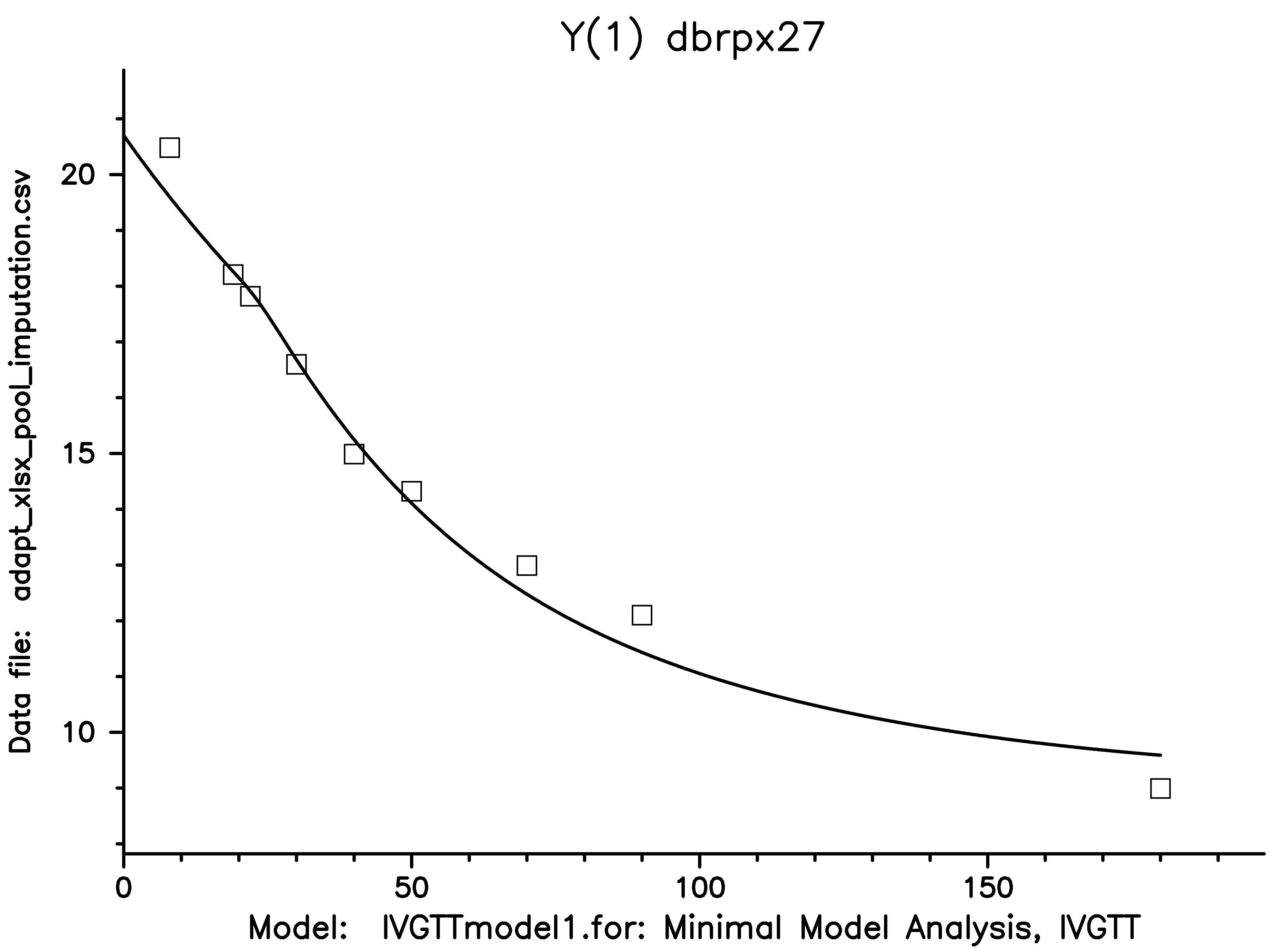

Y(1) dbrpx27
Data file: adapt_xlsx_pool_imputation.csv
20
15
10
0
50
100
150
Model: IVGTTmodel1.for: Minimal Model Analysis, IVGTT

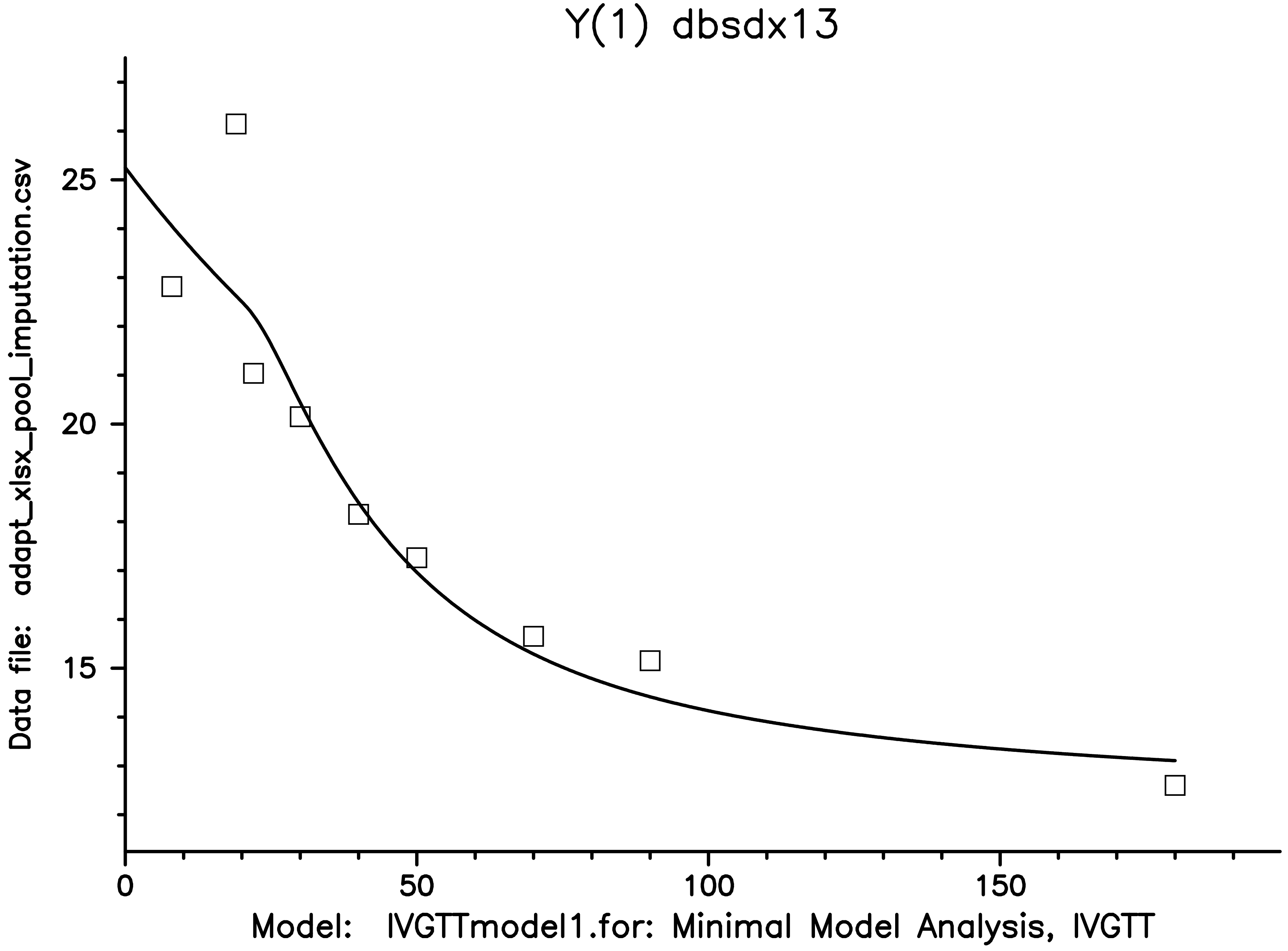
Y(1) dbsdx13
Data file: adapt_xlsx_pool_imputation.csv
25
20
15
0
50
100
150
Model: IVGTTmodel1.for: Minimal Model Analysis, IVGTT

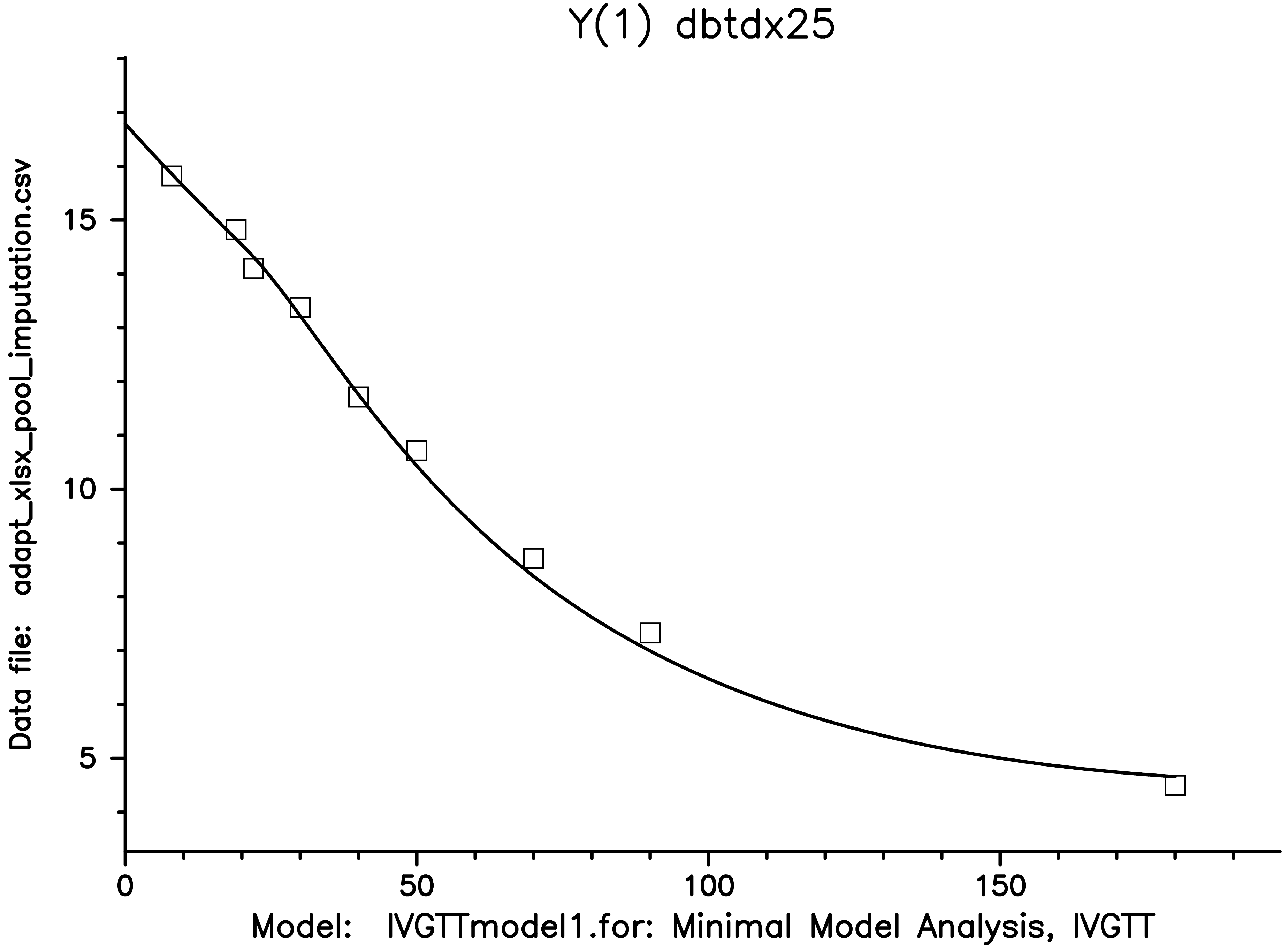

Y(1) dbtdx25
Data file: adapt_xlsx_pool_imputation.csv
15
10
5
0
50
100
150
Model: IVGTTmodel1.for: Minimal Model Analysis, IVGTT

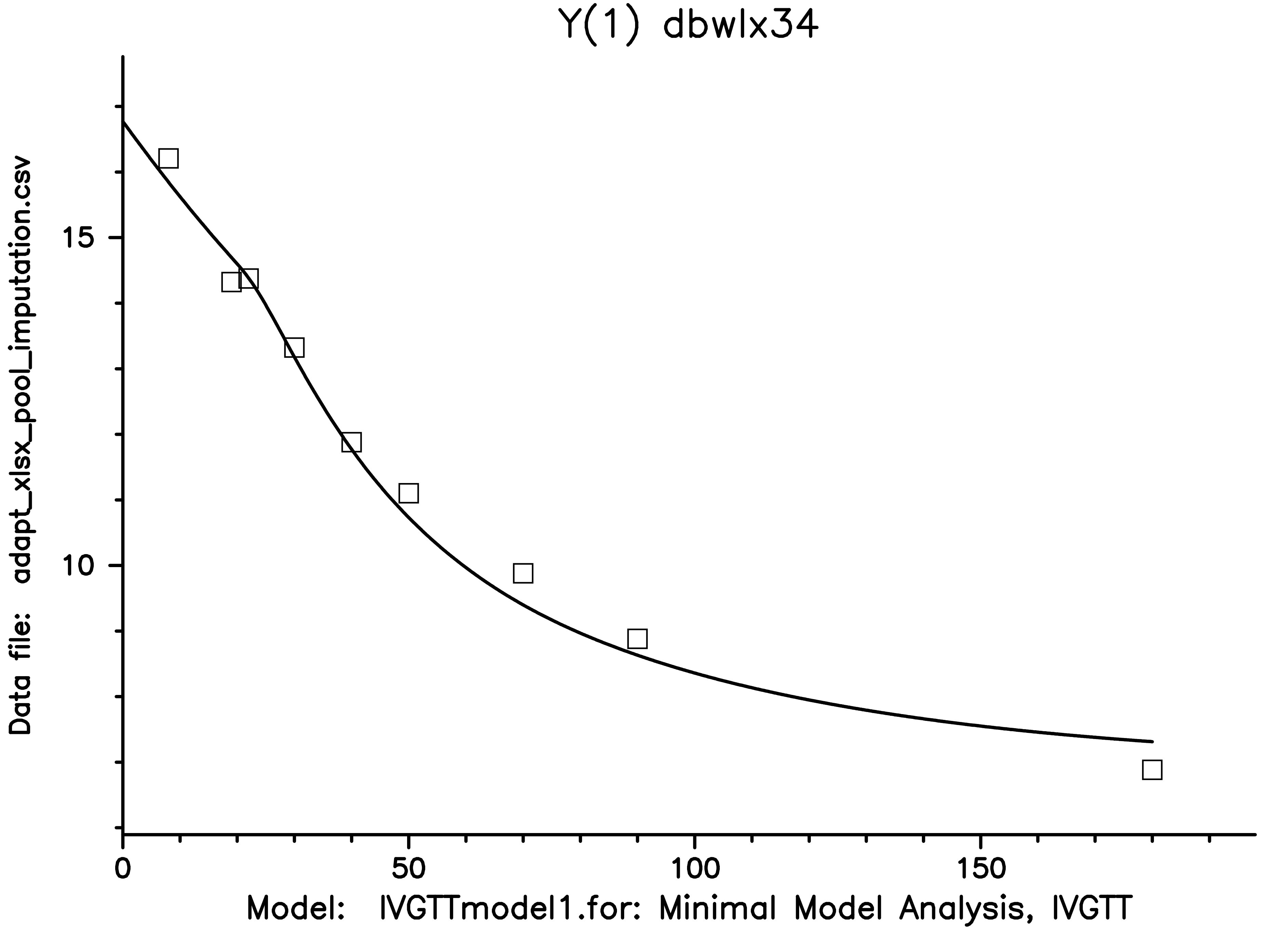
Y(1) dbwlx34
Data file: adapt_xlsx_pool_imputation.csv
15
10
0
50
100
150
Model: IVGTTmodel1.for: Minimal Model Analysis, IVGTT

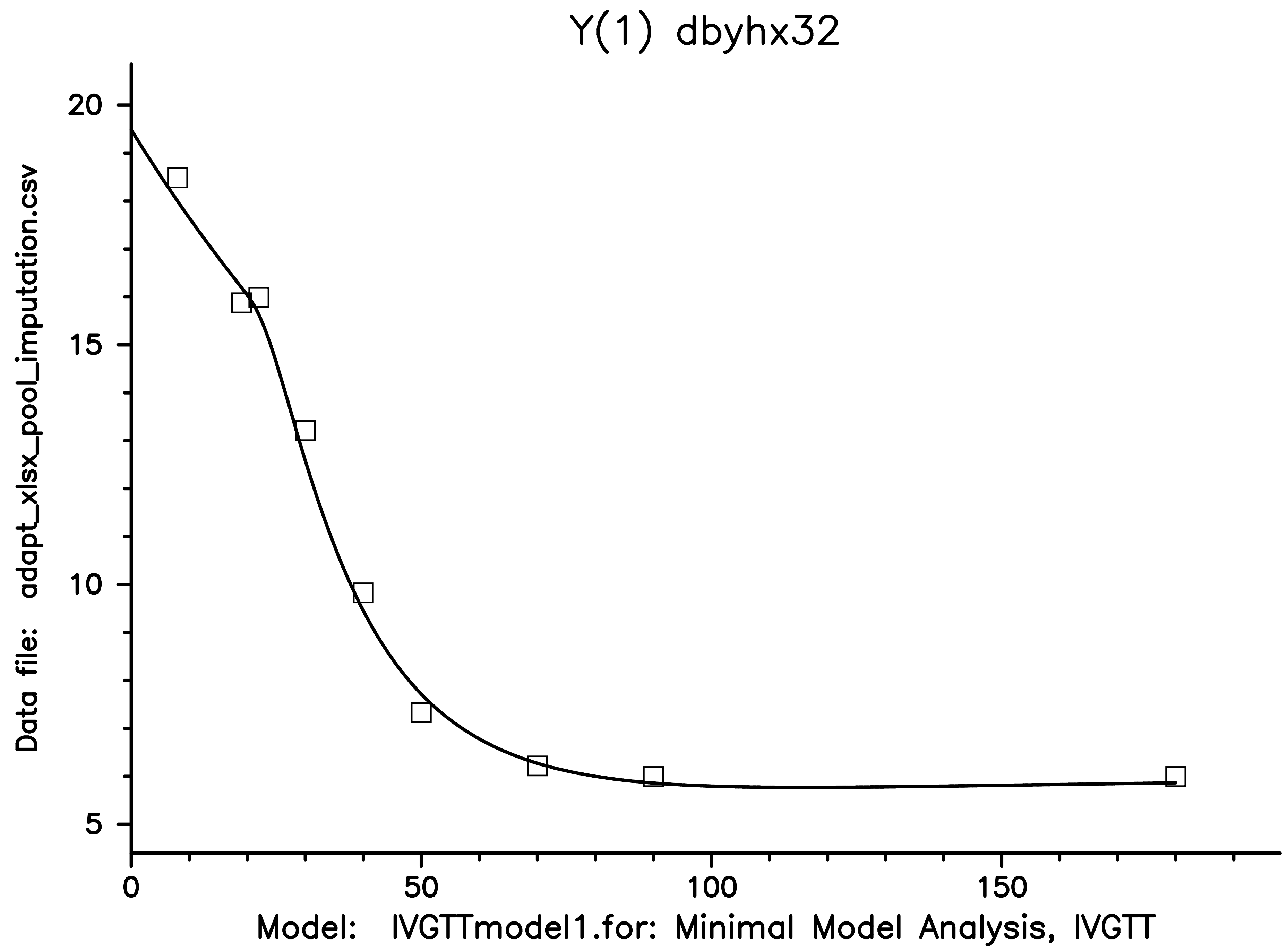
Y(1) dbyhx32
Data file: adapt_xlsx_pool_imputation.csv
20
15
10
5
0
50
100
150
Model: IVGTTmodel1.for: Minimal Model Analysis, IVGTT

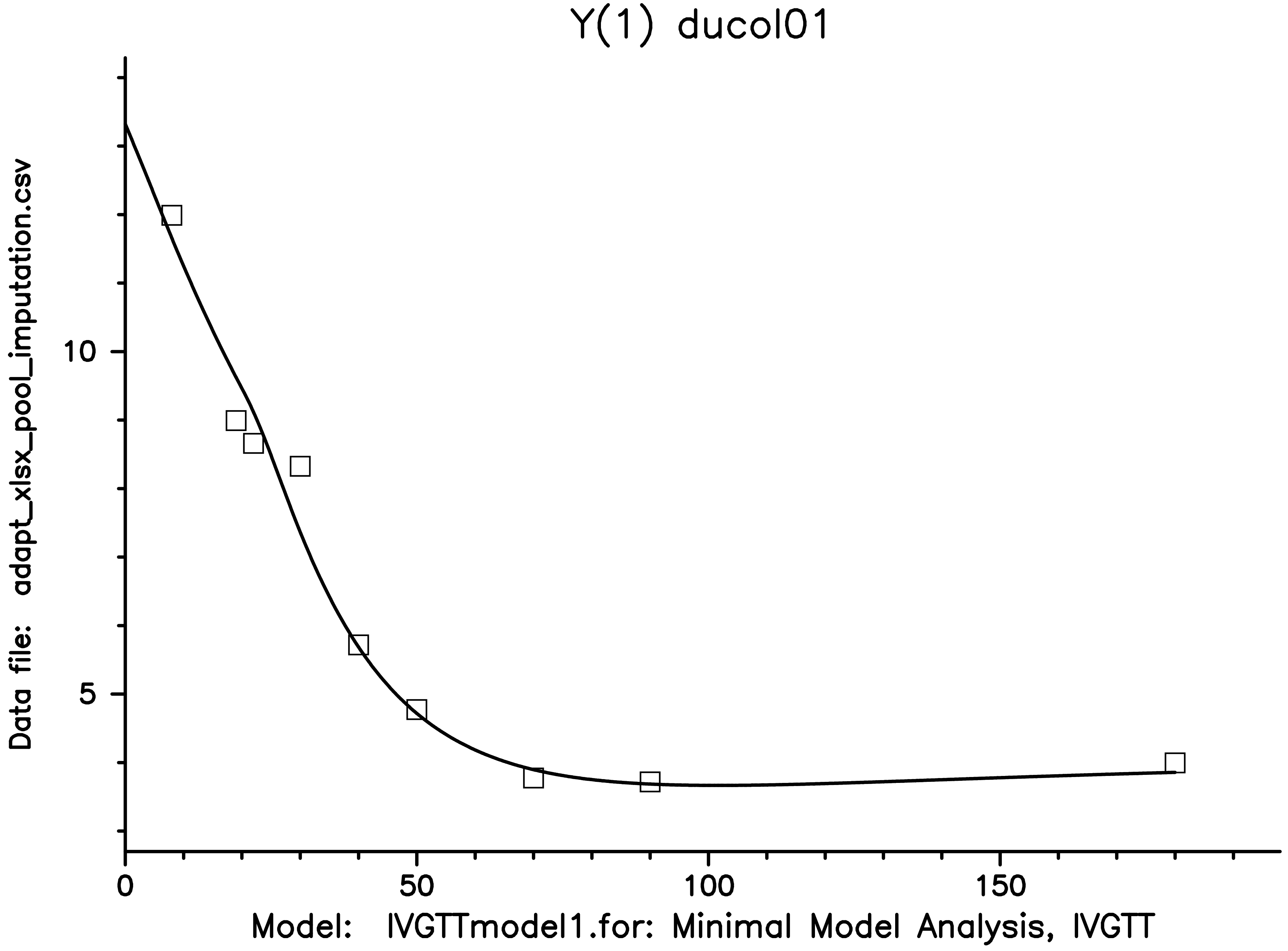
Y(1) ducol01
Data file: adapt_xlsx_pool_imputation.csv
10
5
0
50
100
150
Model: IVGTTmodel1.for: Minimal Model Analysis, IVGTT

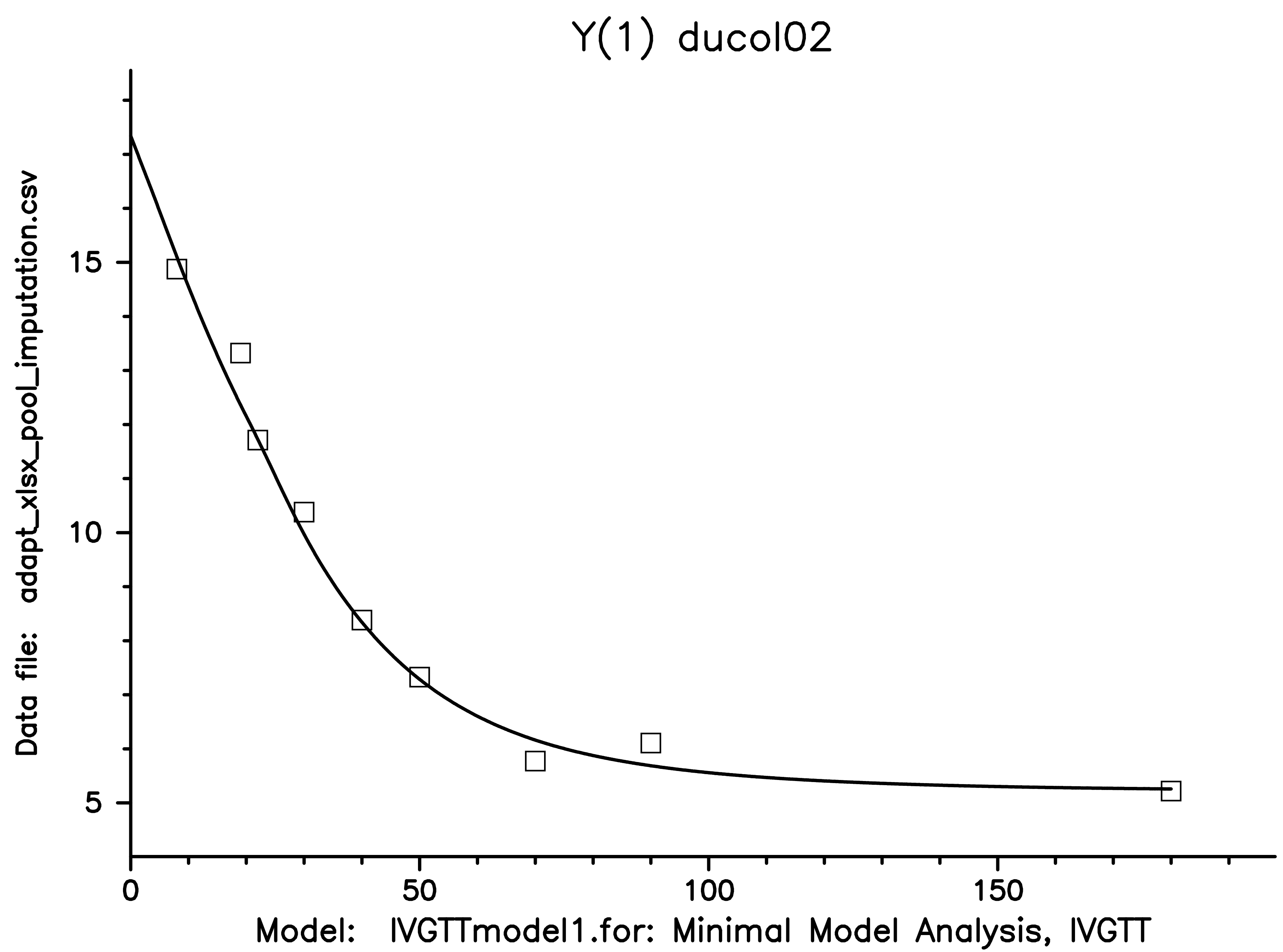

Y(1) ducol02
Data file: adapt_xlsx_pool_imputation.csv
Model: IVGTTmodel1.for: Minimal Model Analysis, IVGTT
15
10
5
0
50
100
150

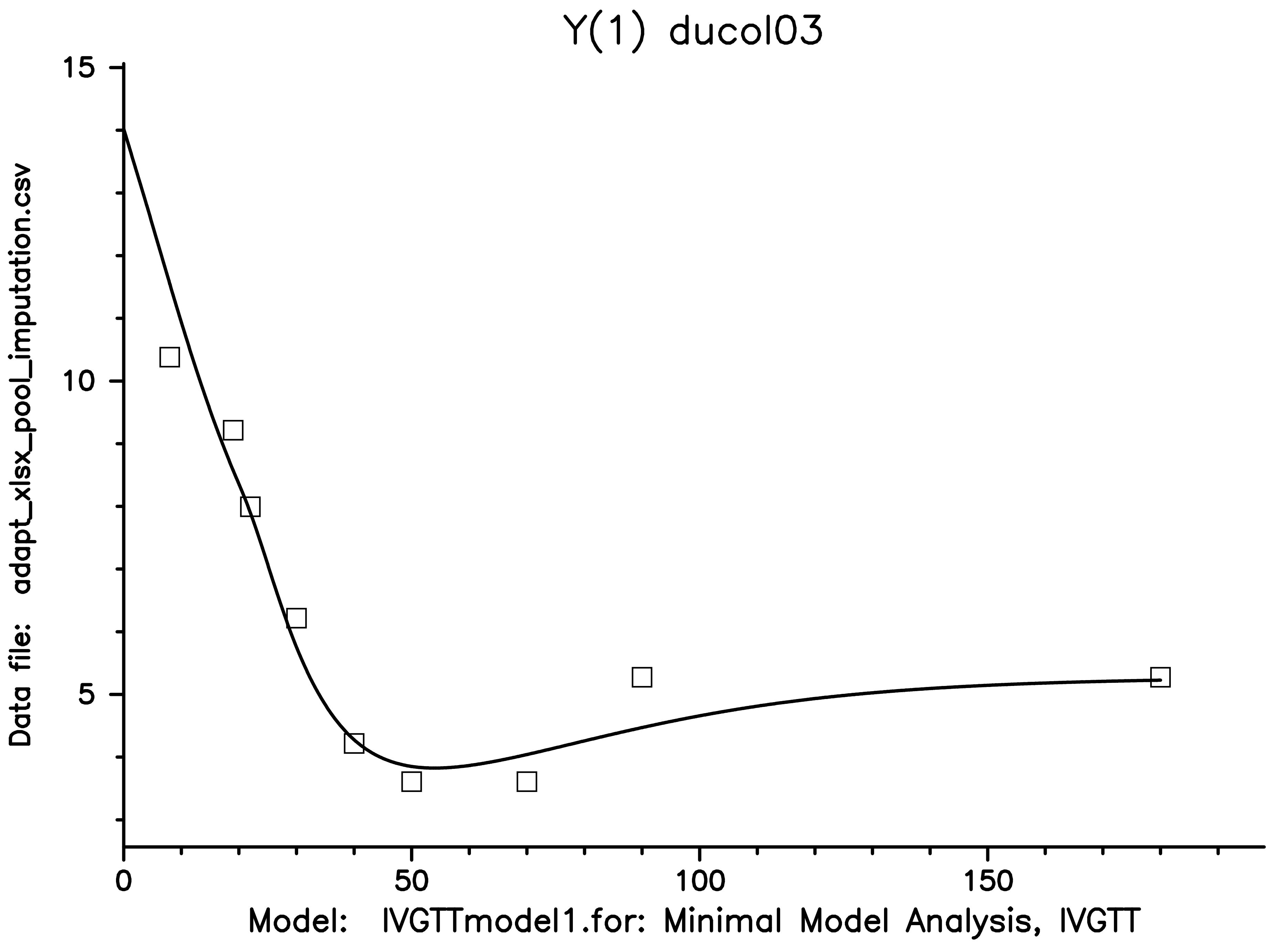

Y(1) ducol03
Data file: adapt_xlsx_pool_imputation.csv
15
10
5
0
50
100
150
Model: IVGTTmodel1.for: Minimal Model Analysis, IVGTT

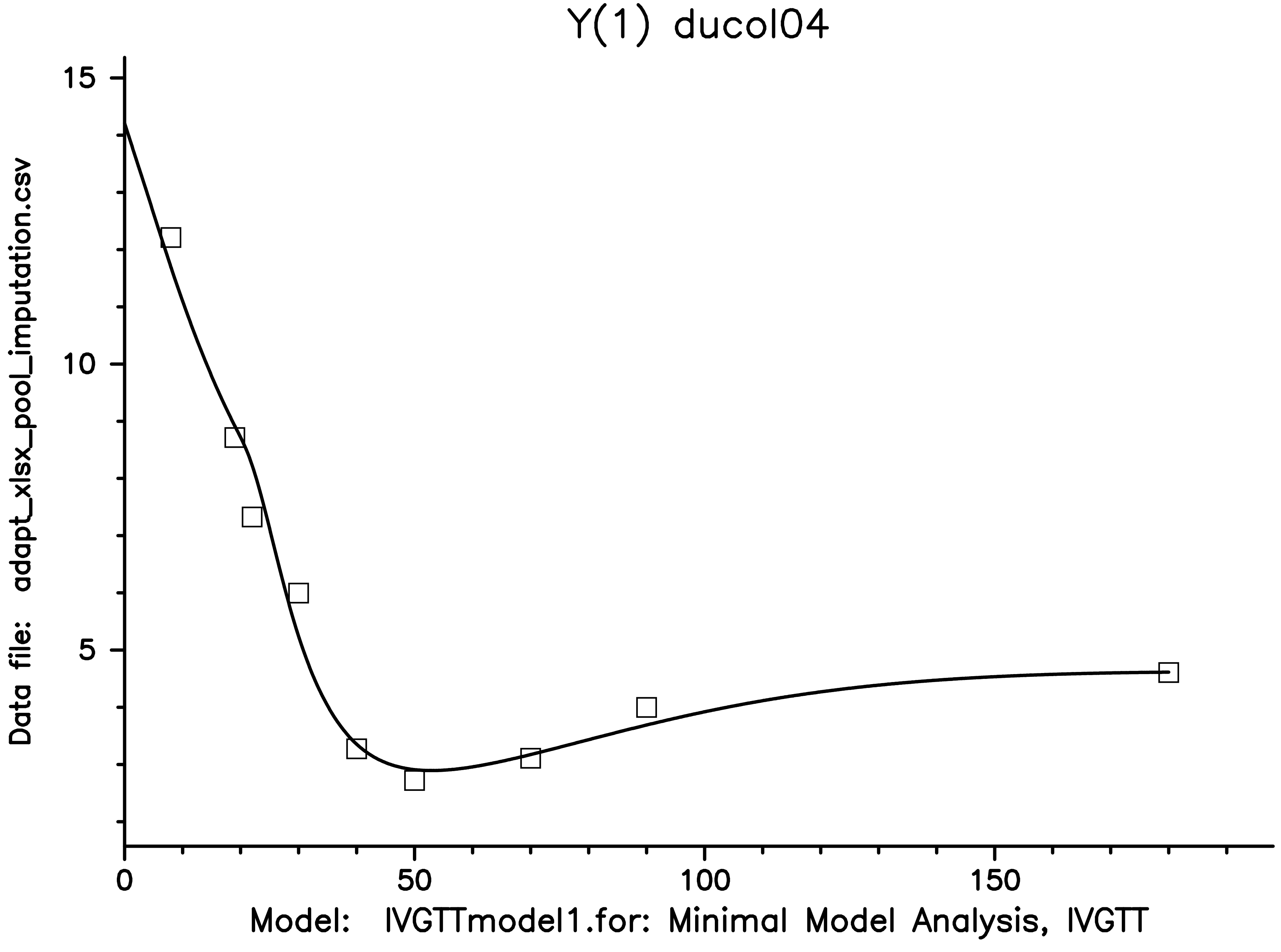
Y(1) ducol04
Data file: adapt_xlsx_pool_imputation.csv
Model: IVGTTmodel1.for: Minimal Model Analysis, IVGTT
15
10
5
0
50
100
150

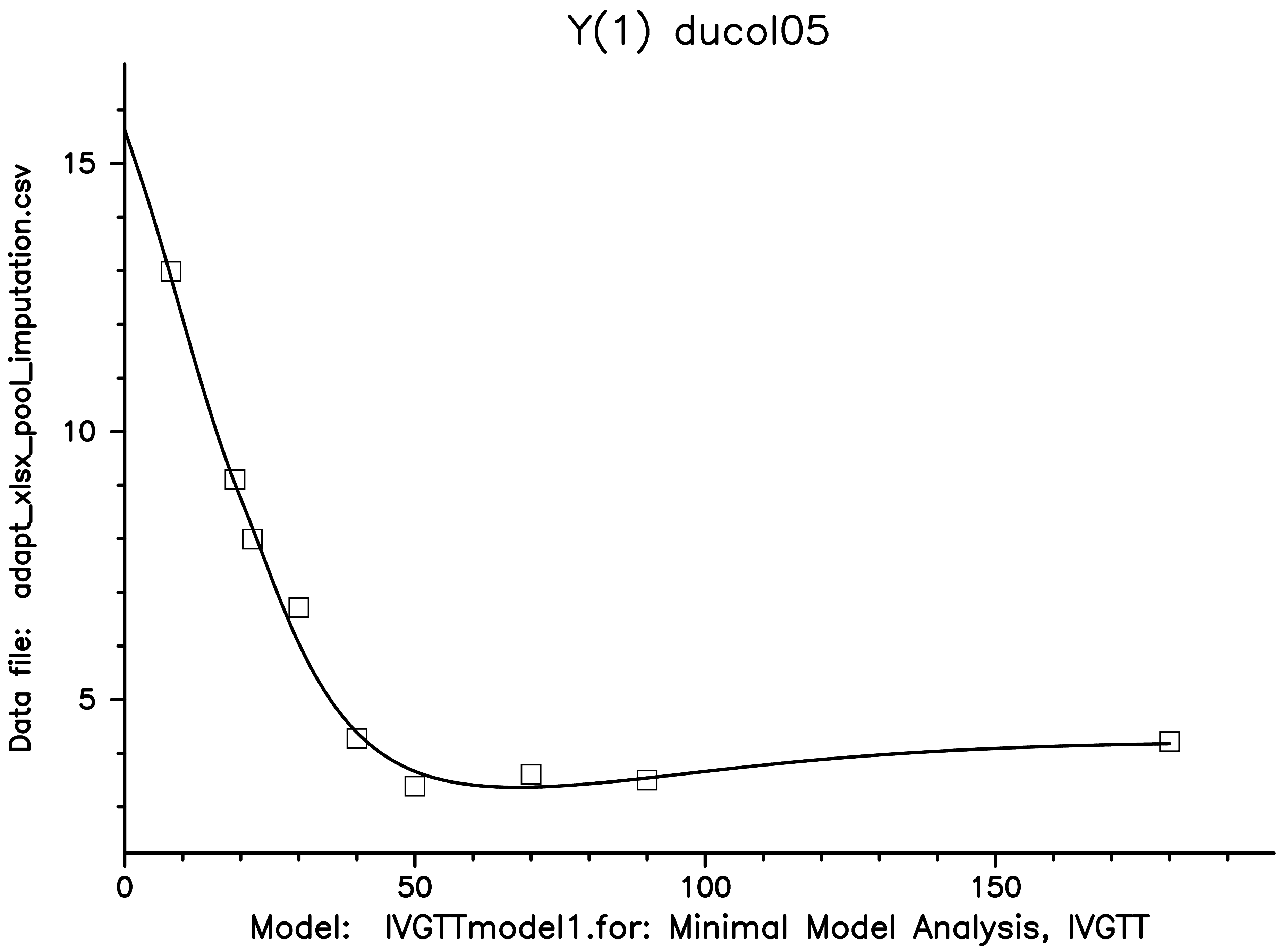

Y(1) ducol05
Data file: adapt_xlsx_pool_imputation.csv
15
10
5
0
50
100
150
Model: IVGTTmodel1.for: Minimal Model Analysis, IVGTT

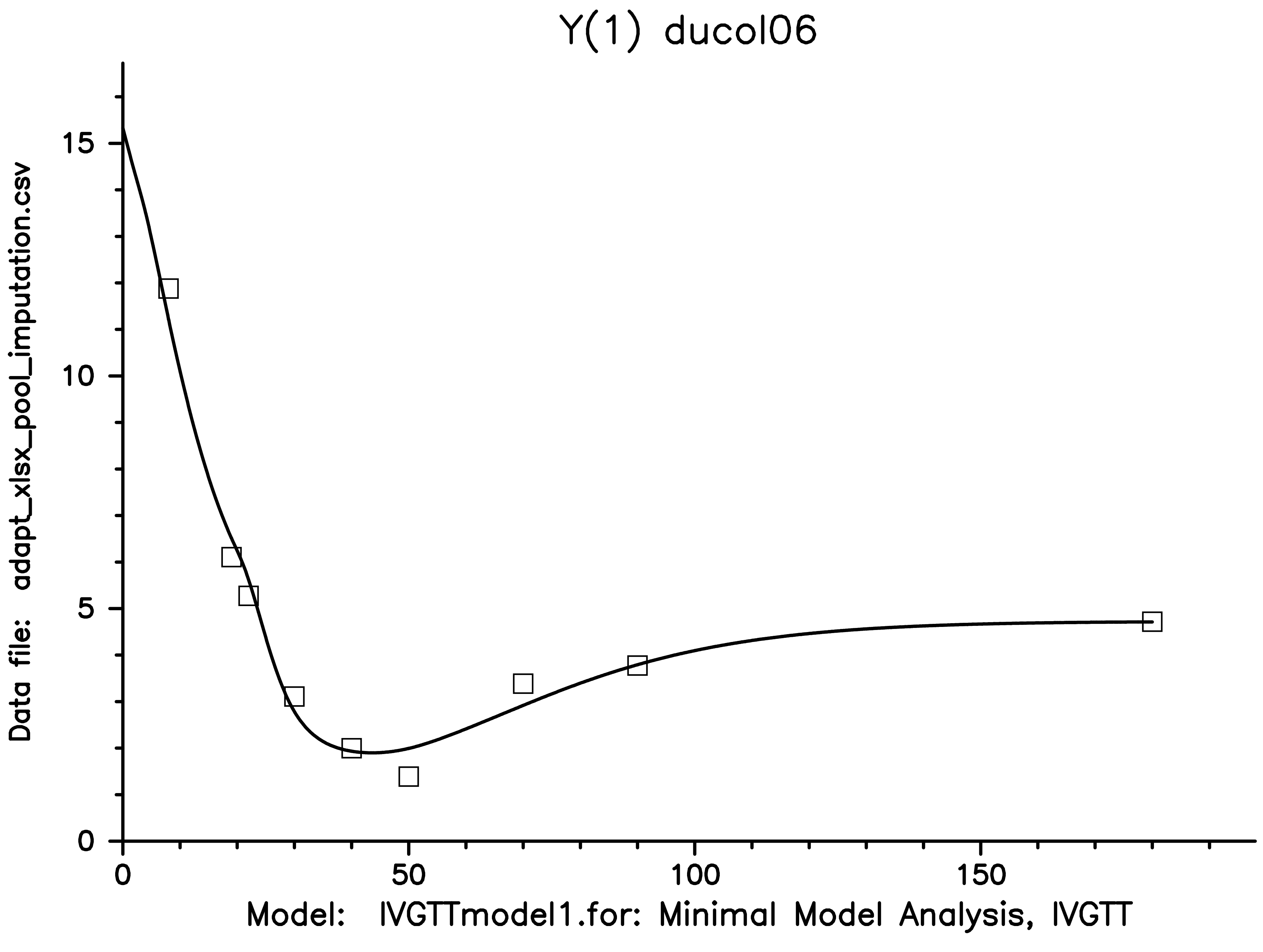
Y(1) ducol06
Data file: adapt_xlsx_pool_imputation.csv
0
5
10
15
0
50
100
150
Model: IVGTTmodel1.for: Minimal Model Analysis, IVGTT

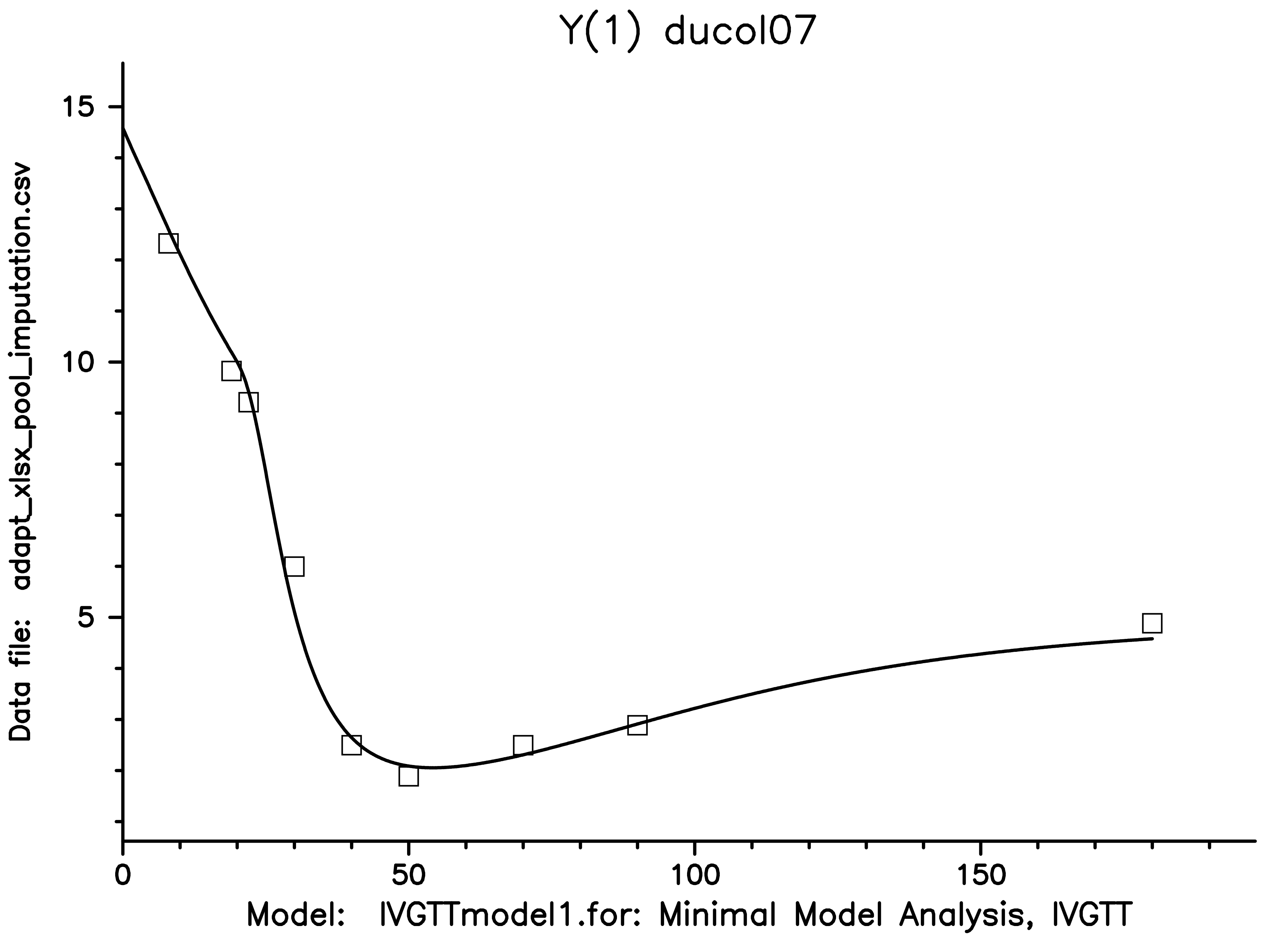

Y(1) ducol07
Data file: adapt_xlsx_pool_imputation.csv
15
10
5
0
50
100
150
Model: IVGTTmodel1.for: Minimal Model Analysis, IVGTT

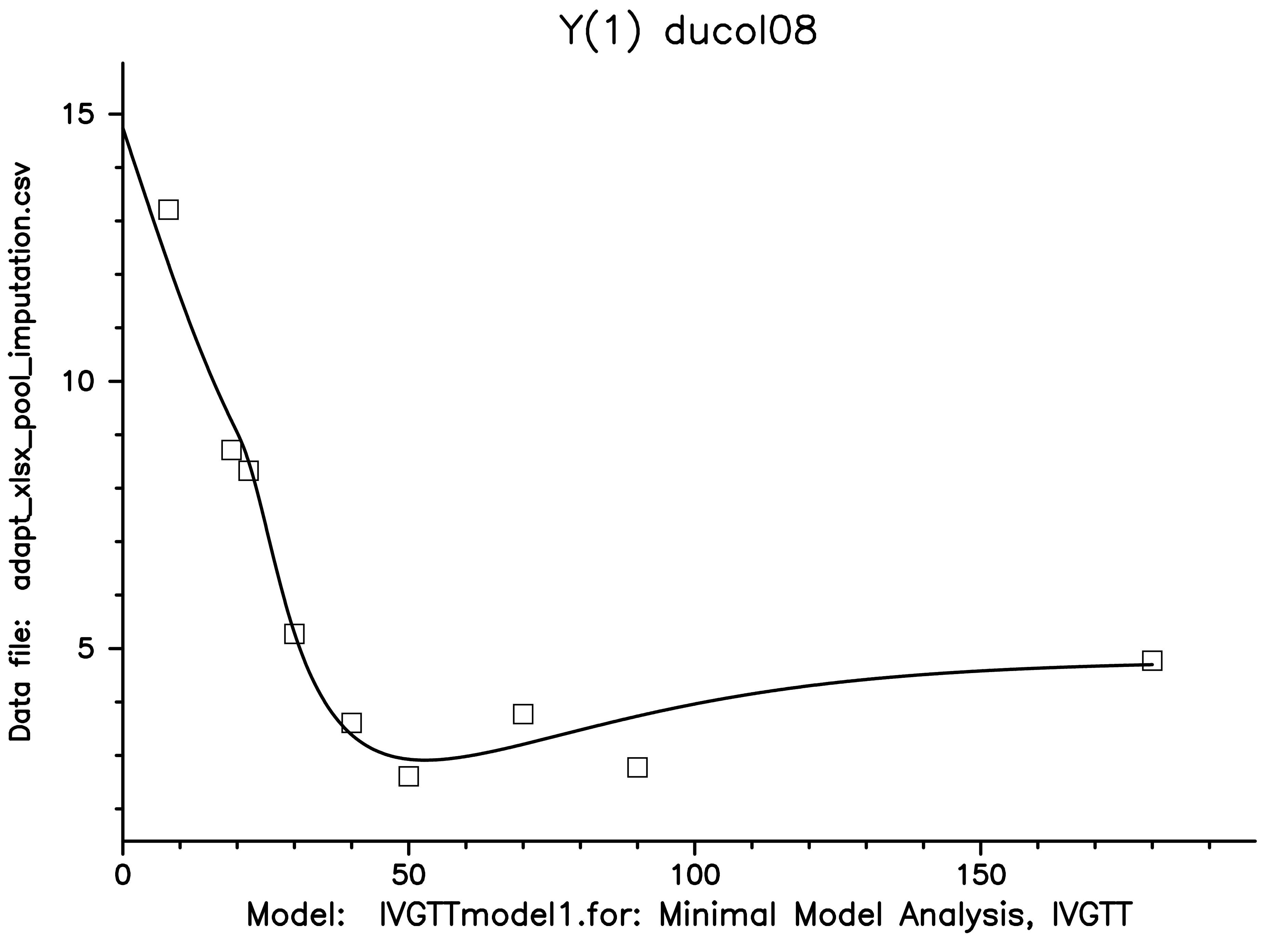
Y(1) ducol08
15
10
5
0
50
100
150
Data file: adapt_xlsx_pool_imputation.csv
Model: IVGTTmodel1.for: Minimal Model Analysis, IVGTT

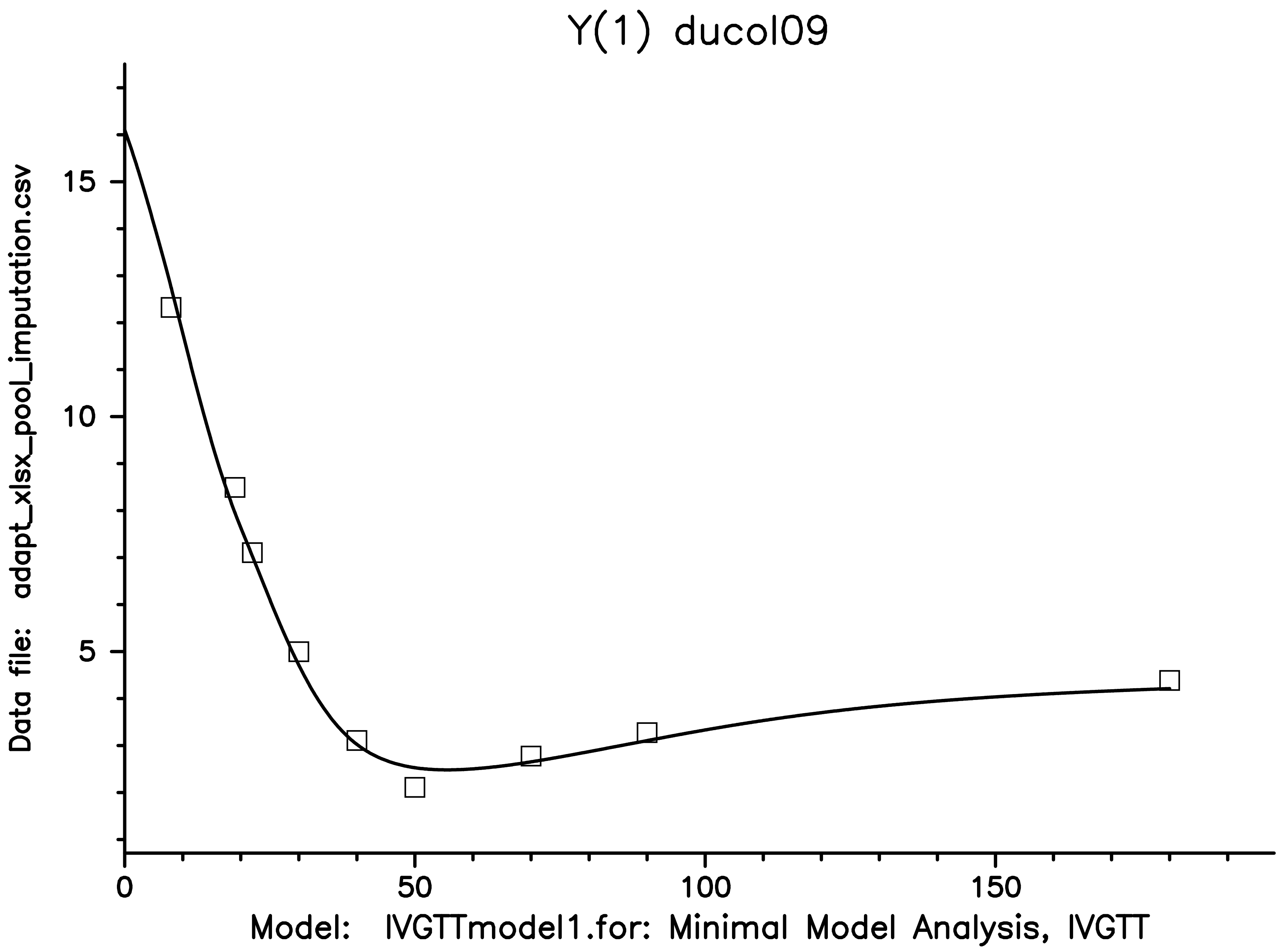
Y(1) ducol09
Data file: adapt_xlsx_pool_imputation.csv
15
10
5
0
50
100
150
Model: IVGTTmodel1.for: Minimal Model Analysis, IVGTT

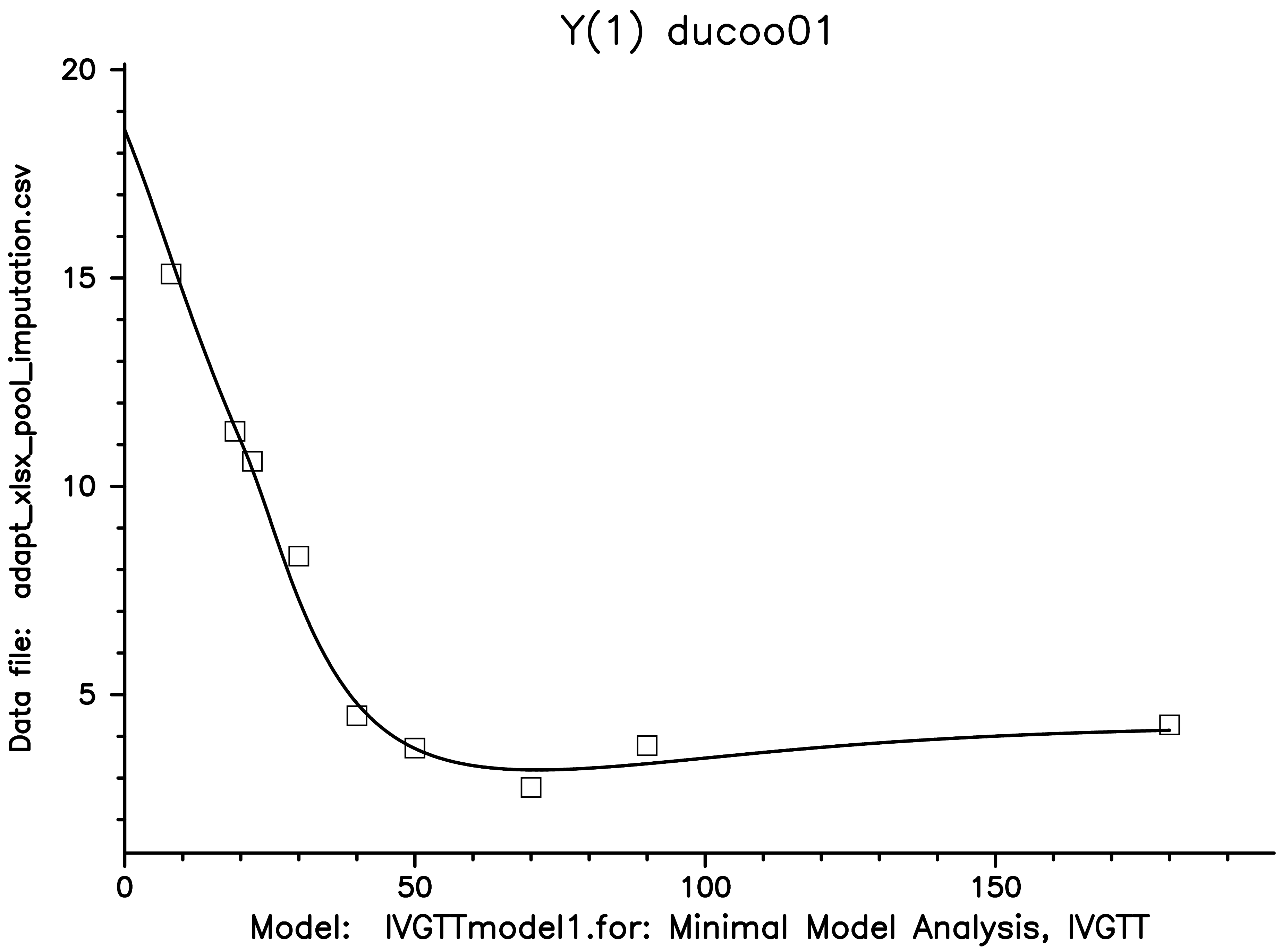
Y(1) ducoo01
20
15
10
5
0
50
100
150
Data file: adapt_xlsx_pool_imputation.csv
Model: IVGTTmodel1.for: Minimal Model Analysis, IVGTT

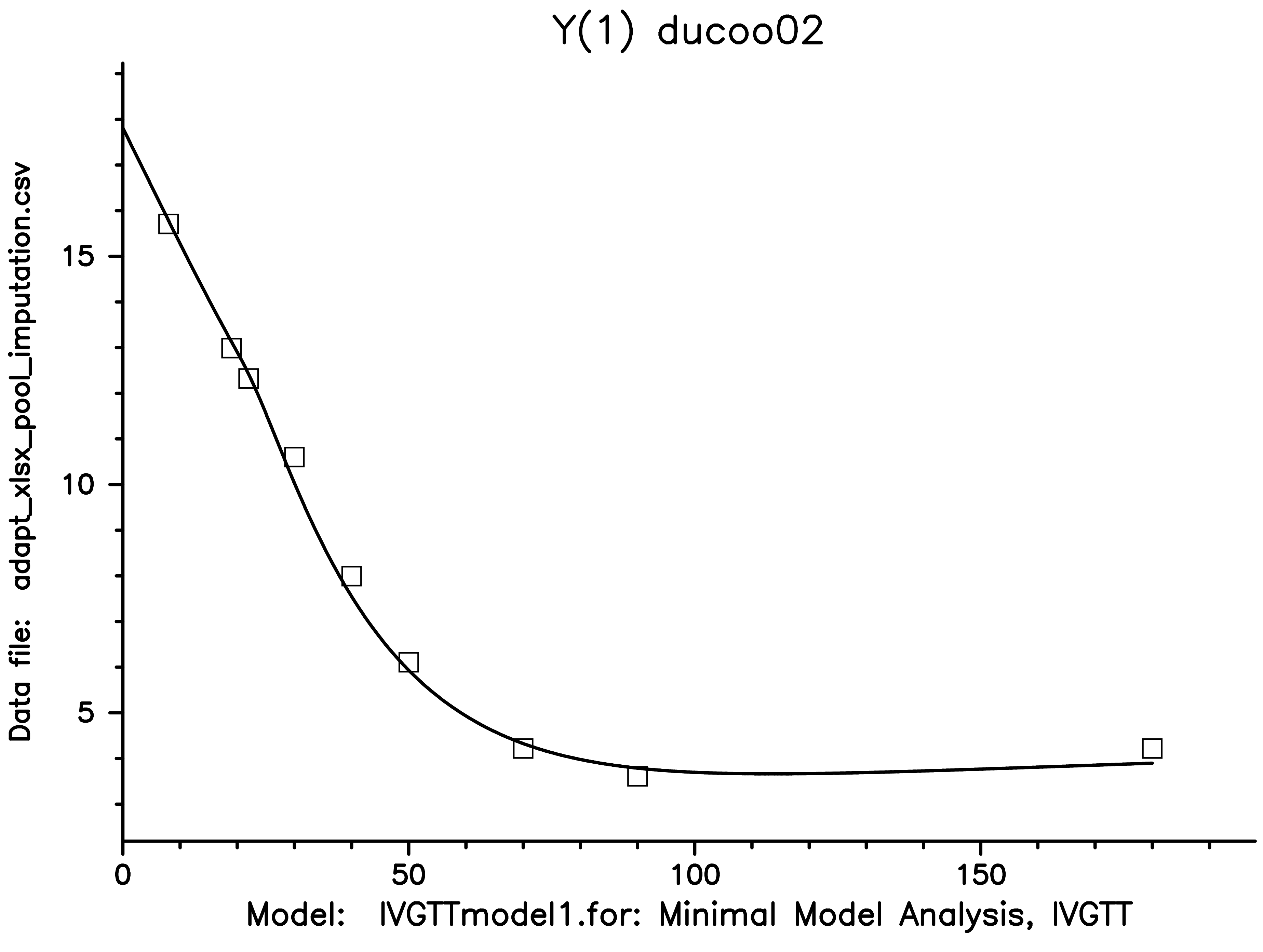
Y(1) ducoo02
Data file: adapt_xlsx_pool_imputation.csv
15
10
5
0
50
100
150
Model: IVGTTmodel1.for: Minimal Model Analysis, IVGTT

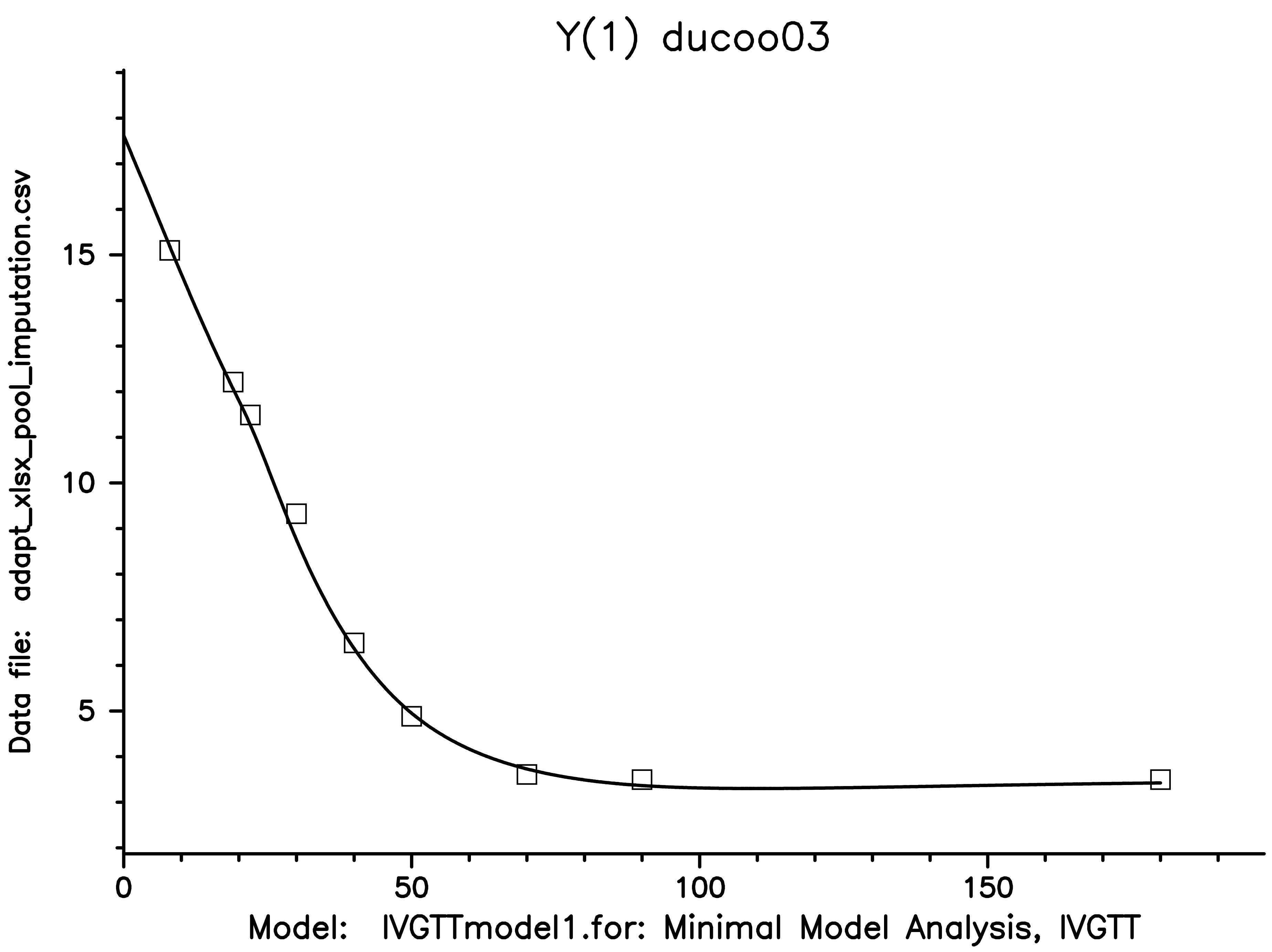
Y(1) ducoo03
Data file: adapt_xlsx_pool_imputation.csv
Model: IVGTTmodel1.for: Minimal Model Analysis, IVGTT
15
10
5
0
50
100
150

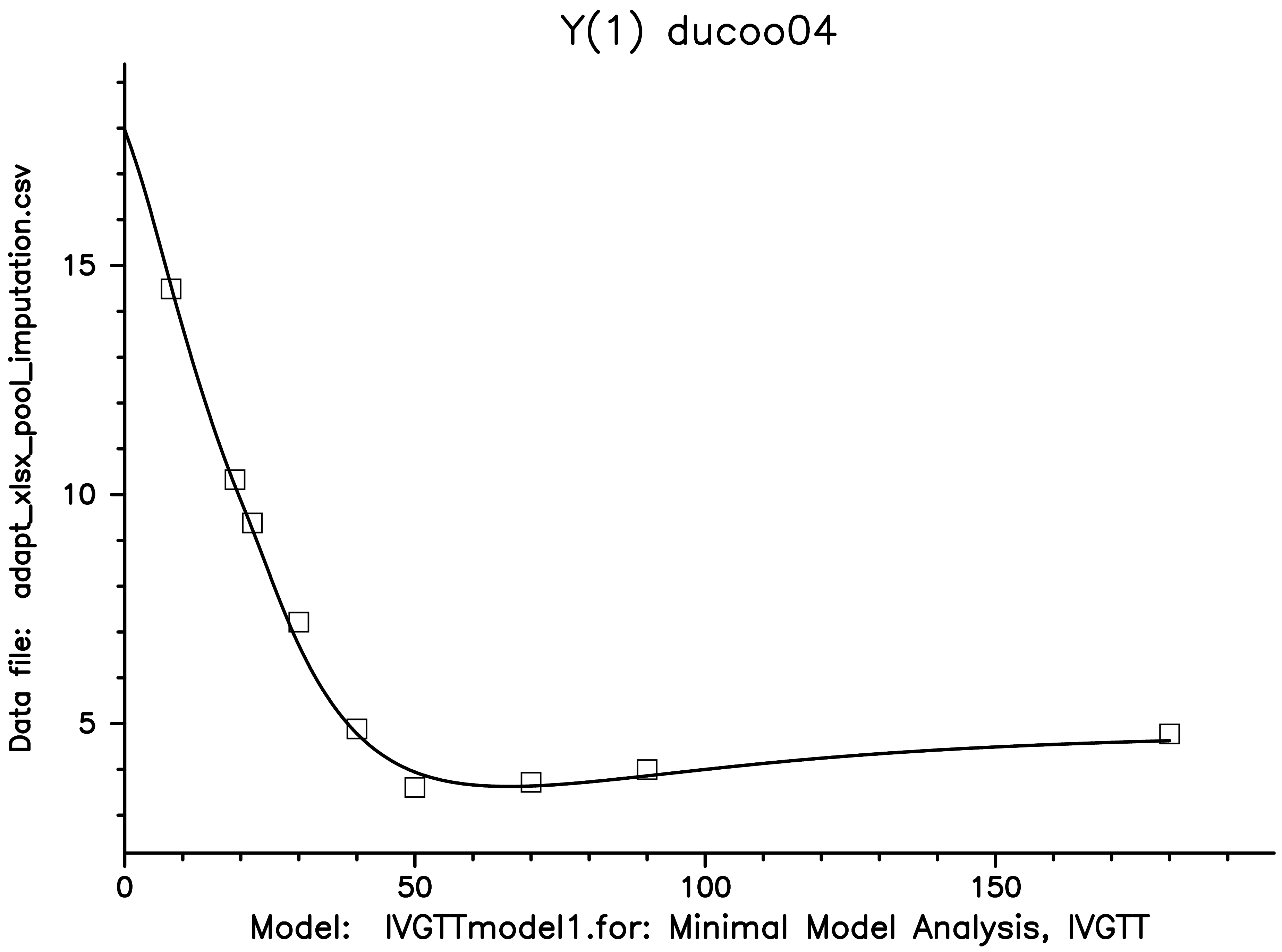
Y(1) ducoo04
Data file: adapt_xlsx_pool_imputation.csv
15
10
5
0
50
100
150
Model: IVGTTmodel1.for: Minimal Model Analysis, IVGTT

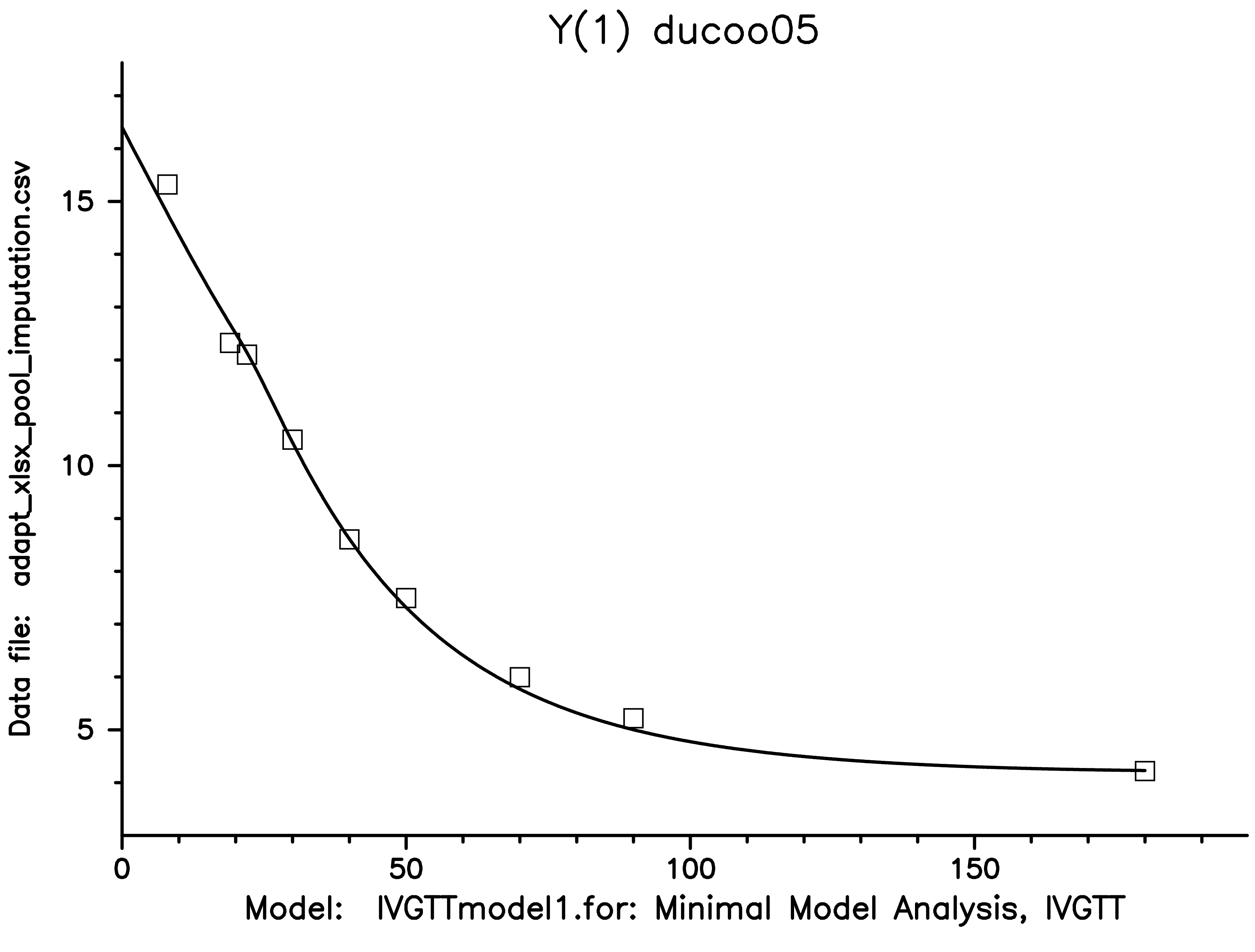
Y(1) ducoo05
Data file: adapt_xlsx_pool_imputation.csv
15
10
5
0
50
100
150
Model: IVGTTmodel1.for: Minimal Model Analysis, IVGTT

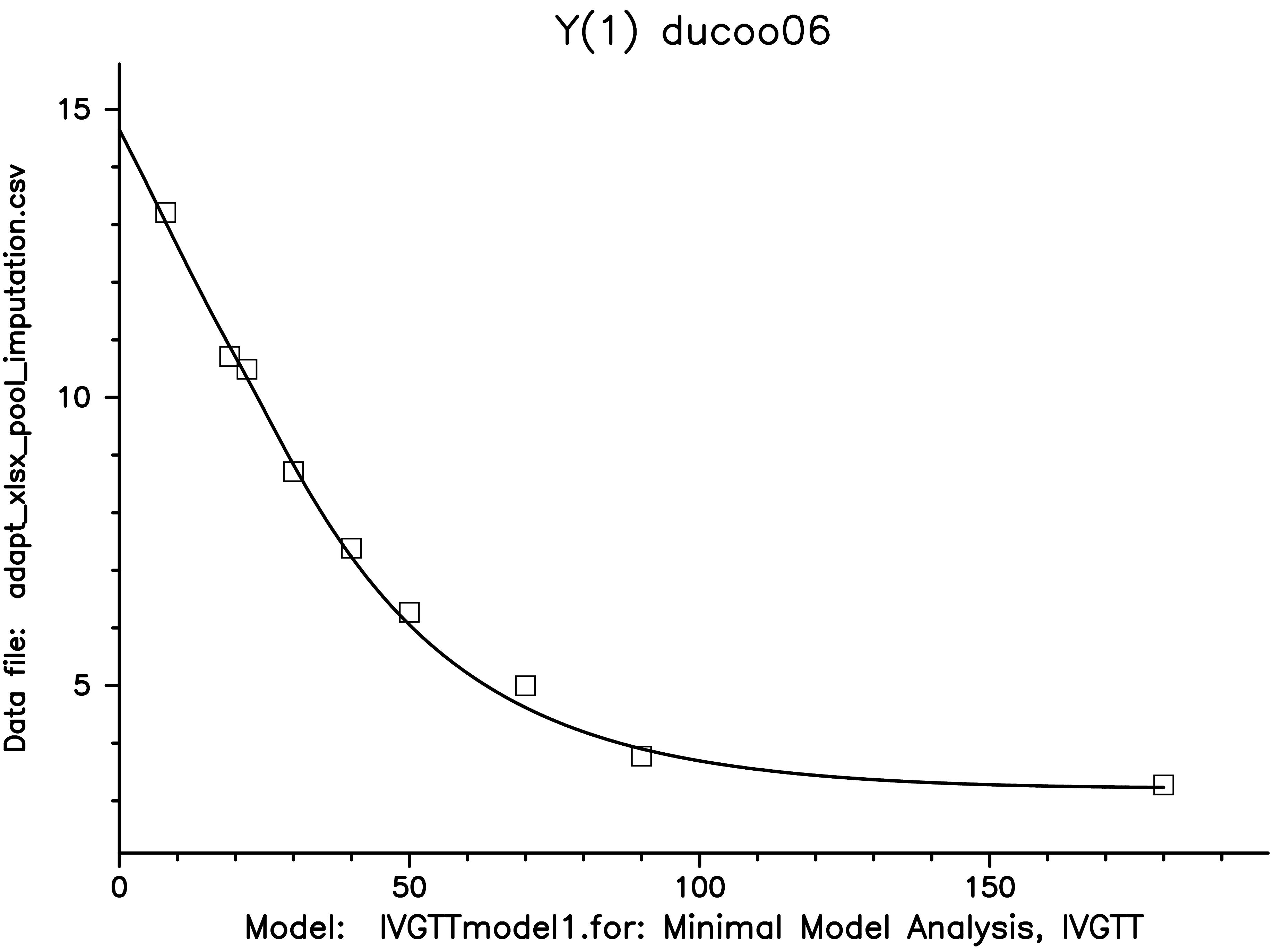

Y(1) ducoo06
15
10
5
0
50
100
150
Data file: adapt_xlsx_pool_imputation.csv
Model: IVGTTmodel1.for: Minimal Model Analysis, IVGTT

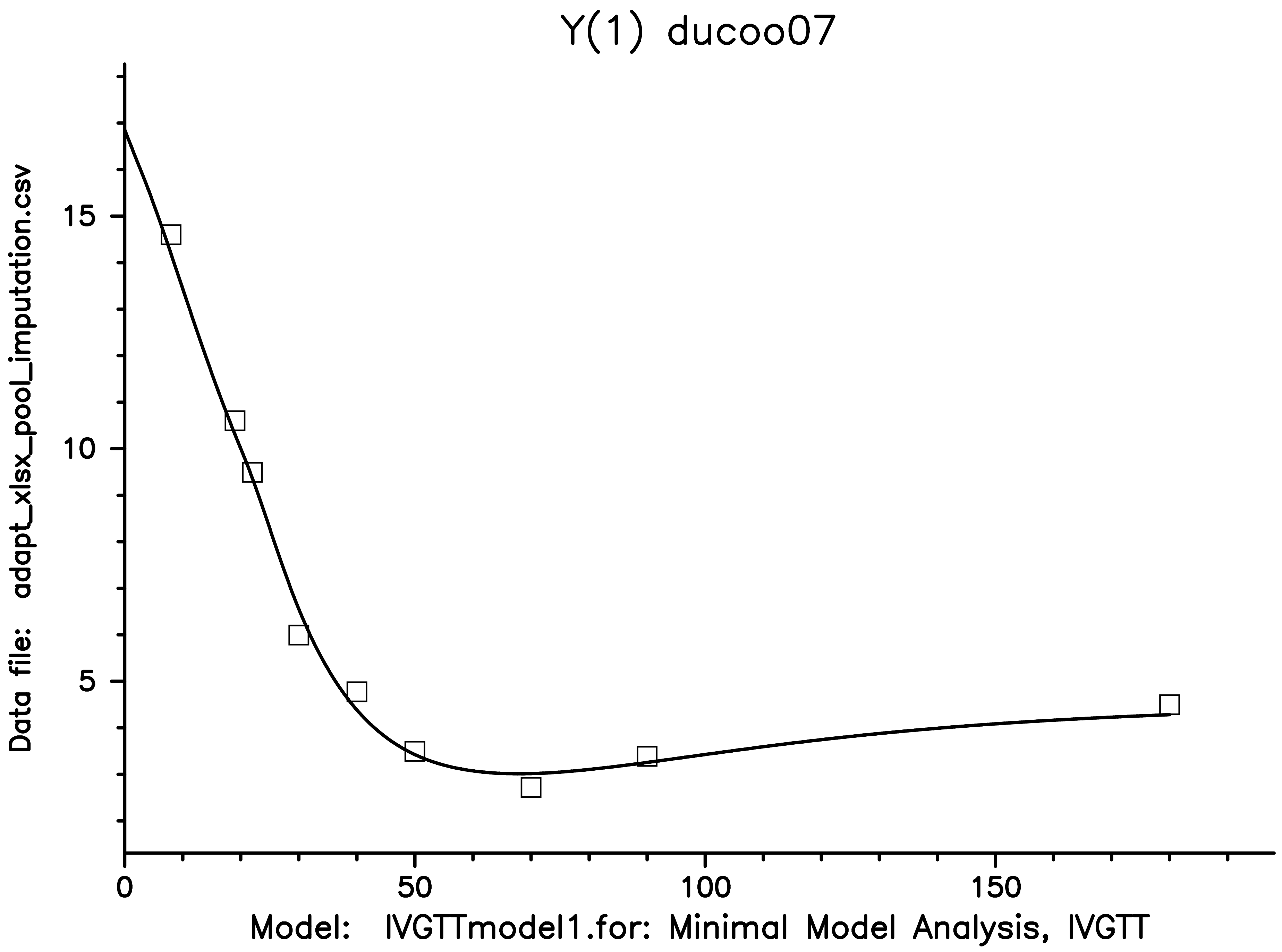
Y(1) ducoo07
Data file: adapt_xlsx_pool_imputation.csv
15
10
5
0
50
100
150
Model: IVGTTmodel1.for: Minimal Model Analysis, IVGTT

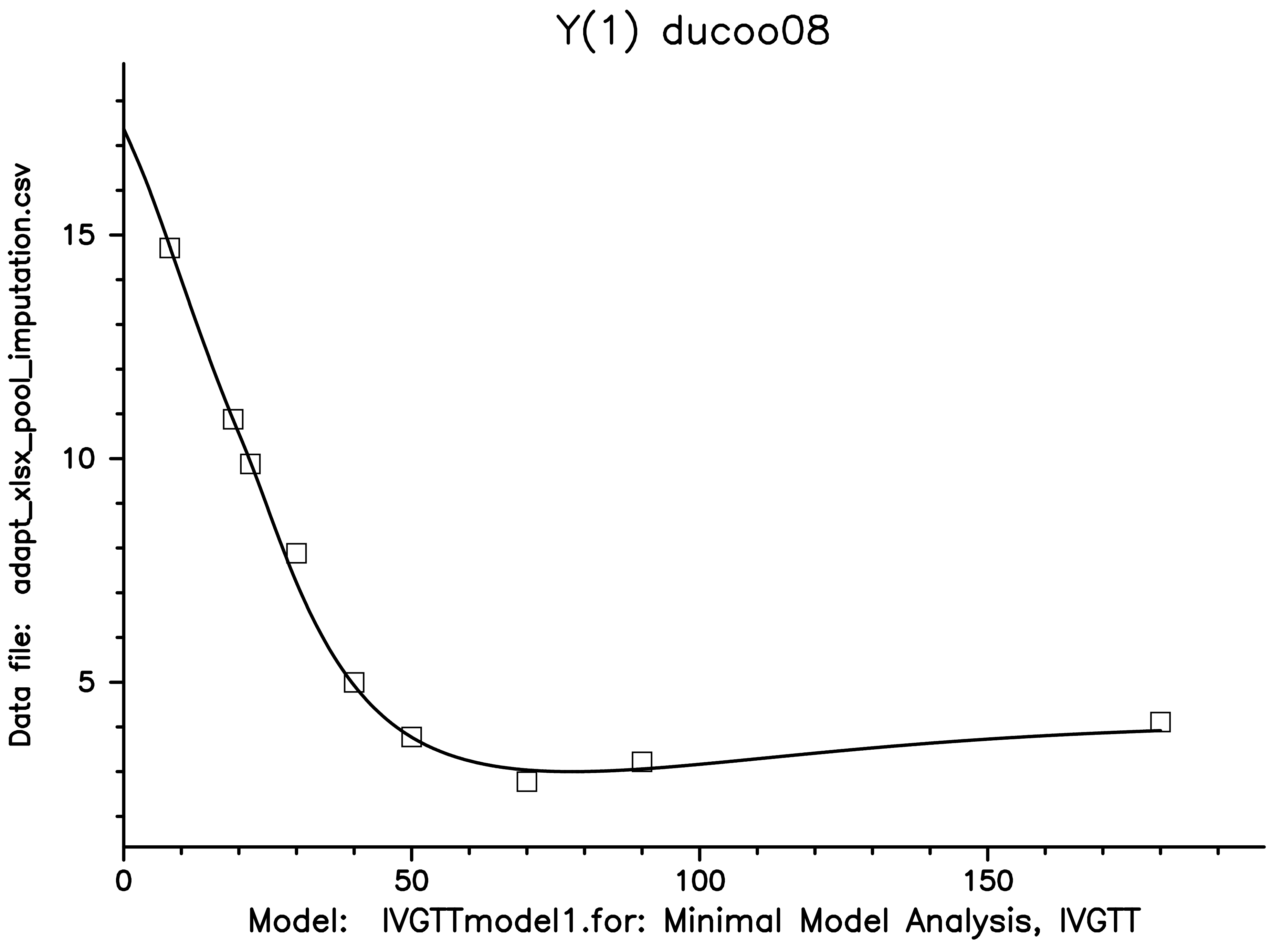
Y(1) ducoo08
Data file: adapt_xlsx_pool_imputation.csv
Model: IVGTTmodel1.for: Minimal Model Analysis, IVGTT
15
10
5
0
50
100
150

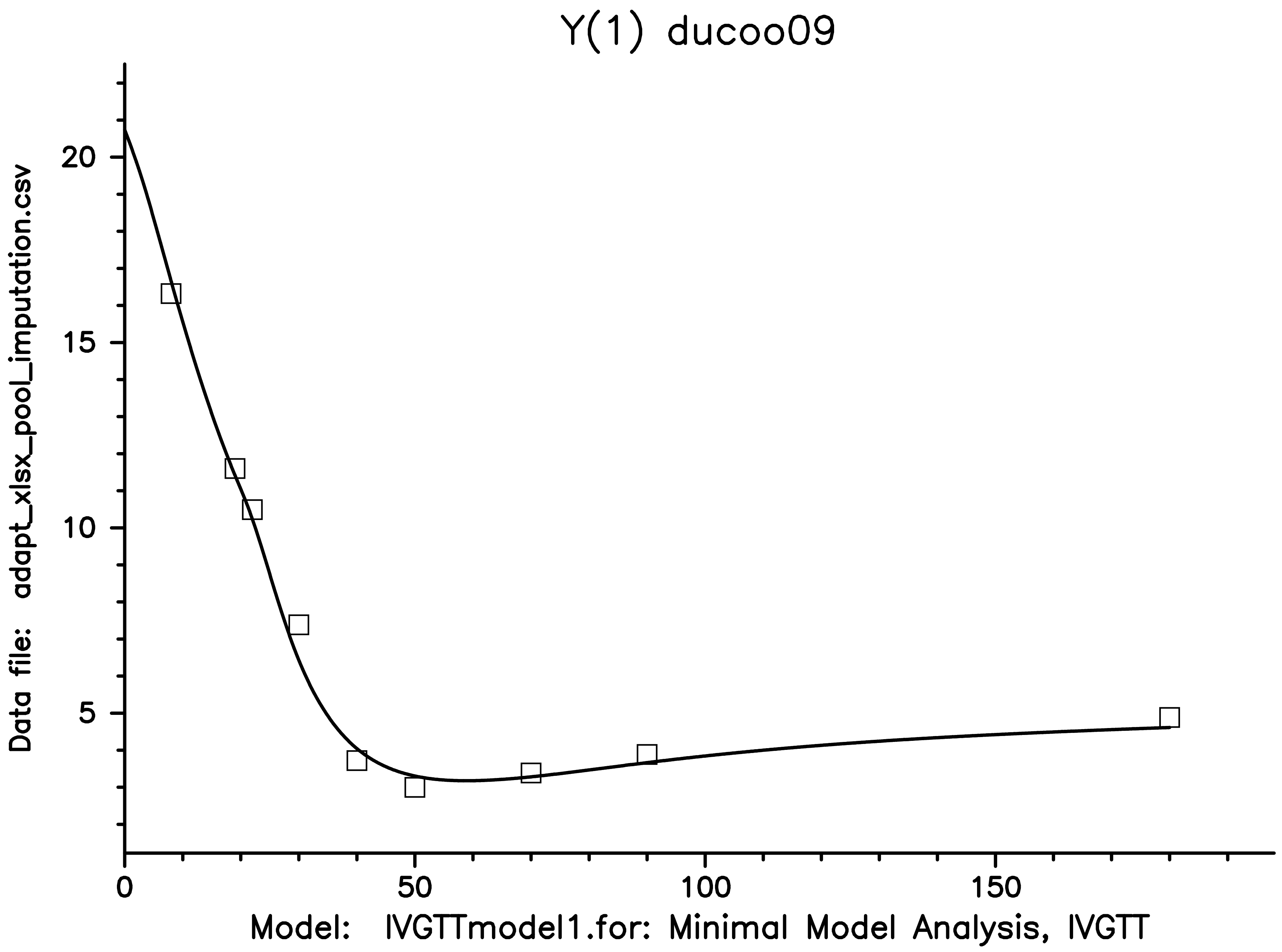
Y(1) ducoo09
Data file: adapt_xlsx_pool_imputation.csv
20
15
10
5
0
50
100
150
Model: IVGTTmodel1.for: Minimal Model Analysis, IVGTT

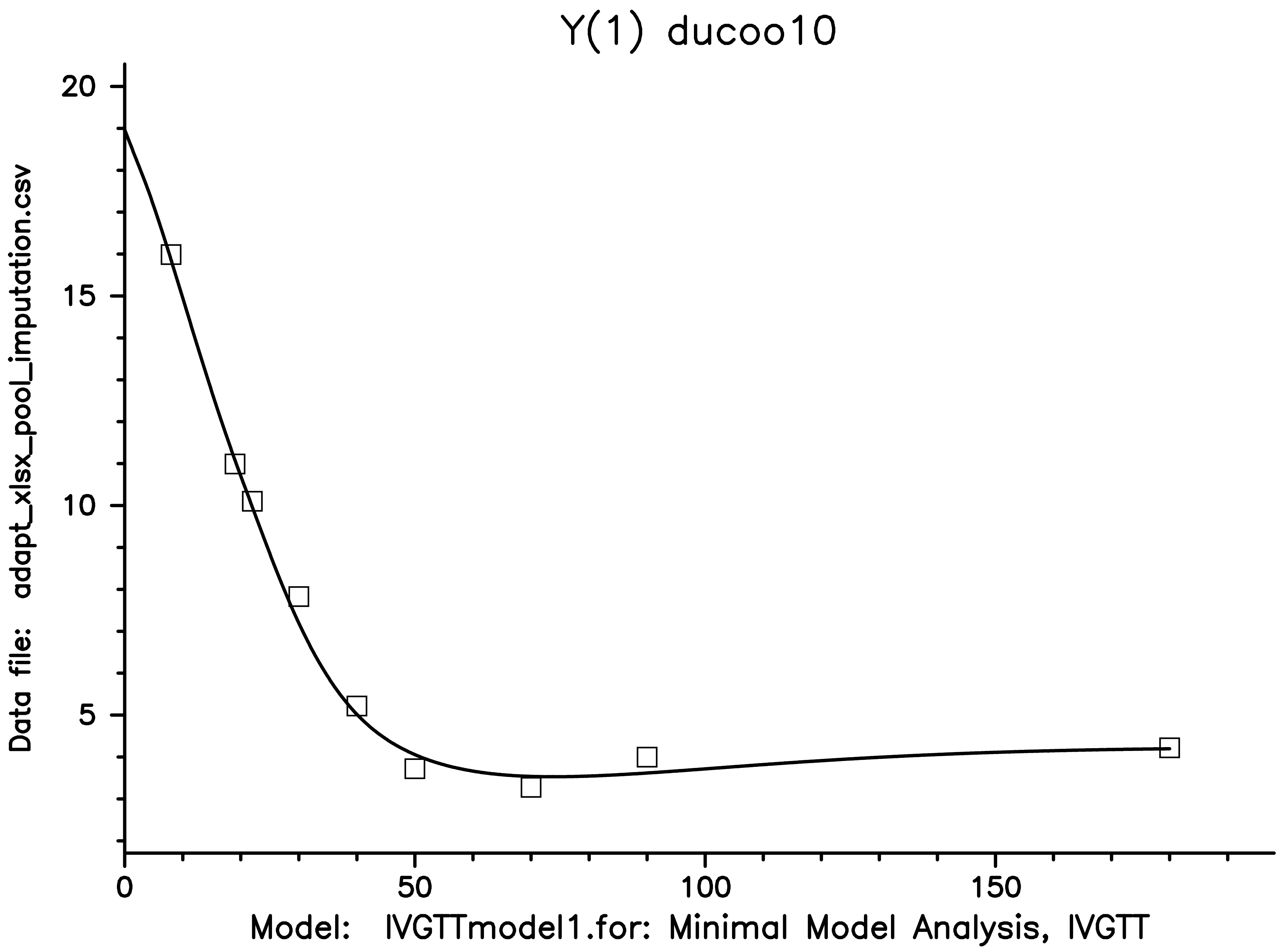
Y(1) ducoo10
Data file: adapt_xlsx_pool_imputation.csv
Model: IVGTTmodel1.for: Minimal Model Analysis, IVGTT
20
15
10
5
0
50
100
150

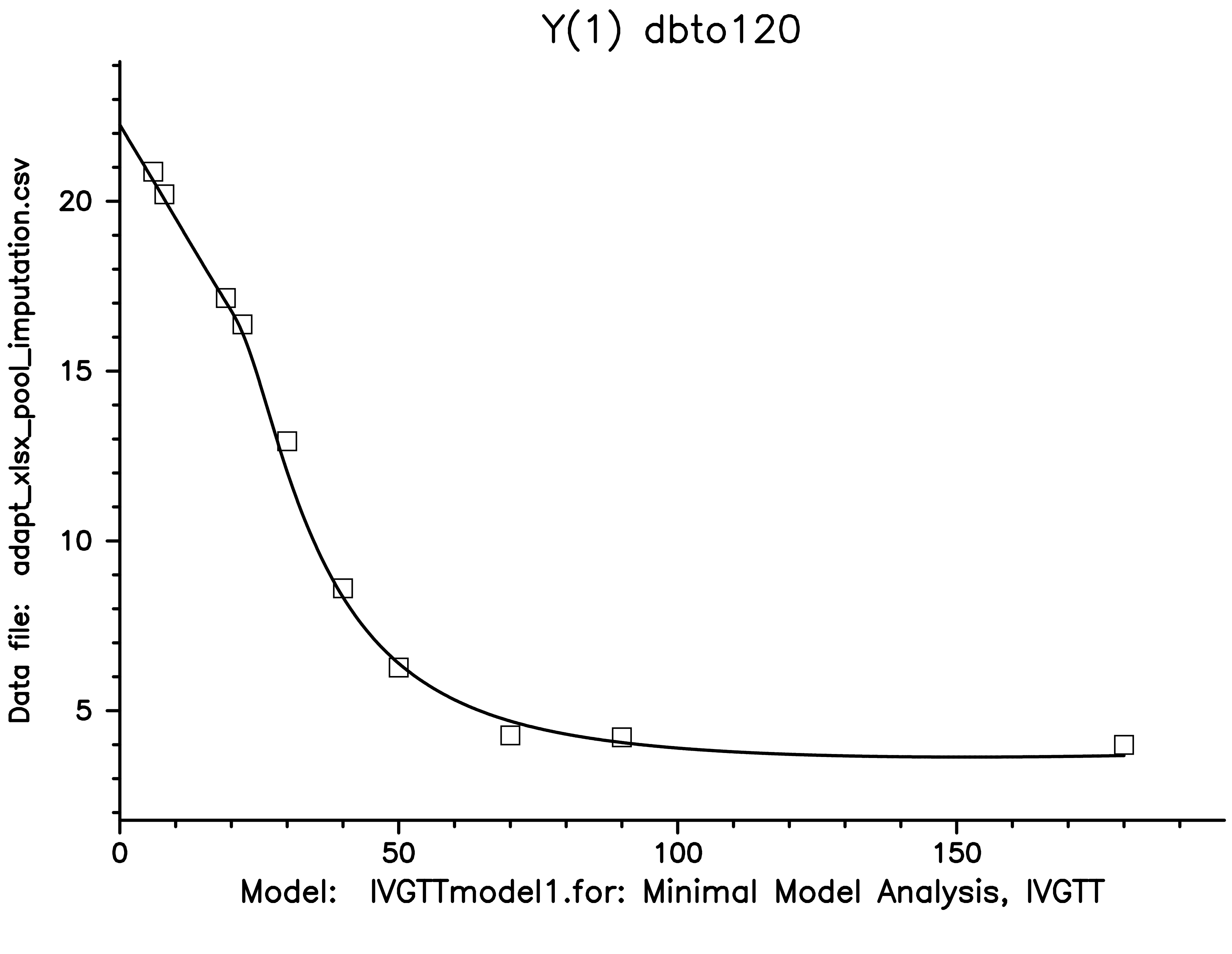
Y(1) dbto120
Data file: adapt_xlsx_pool_imputation.csv
20
15
10
5
0
50
100
150
Model: IVGTTmodel1.for: Minimal Model Analysis, IVGTT

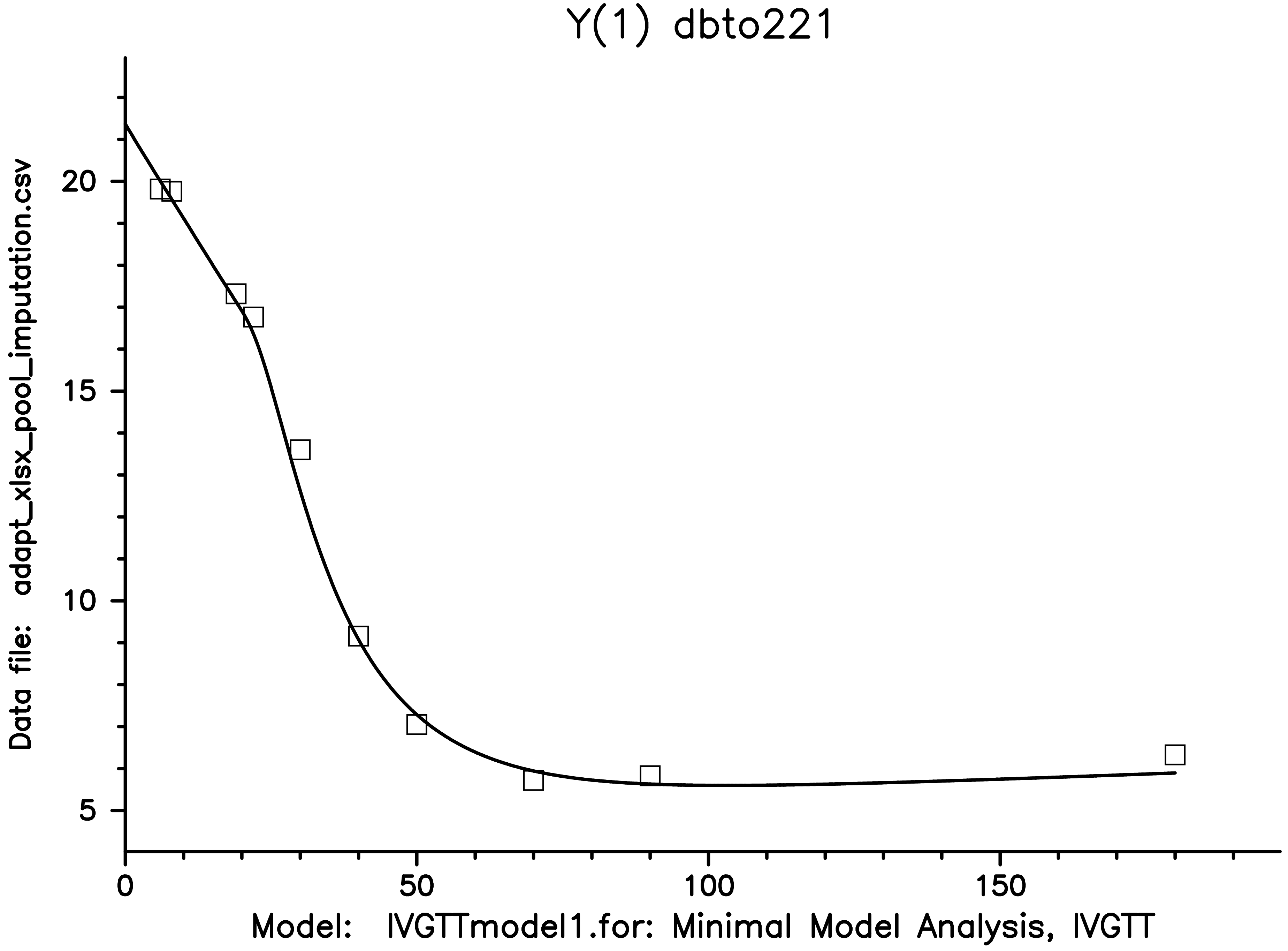
Y(1) dbto221
Data file: adapt_xlsx_pool_imputation.csv
20
15
10
5
0
50
100
150
Model: IVGTTmodel1.for: Minimal Model Analysis, IVGTT

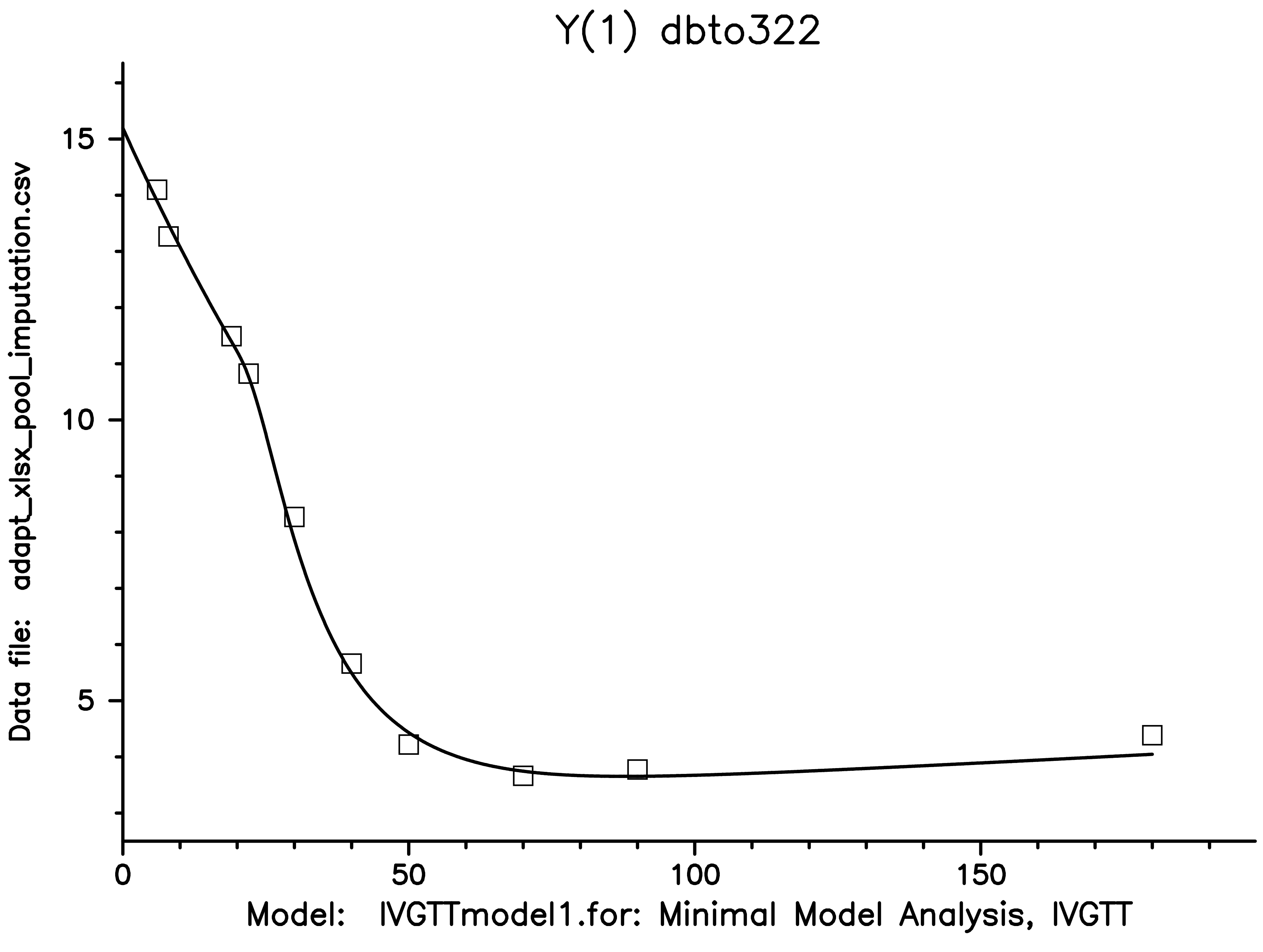
Y(1) dbto322
Data file: adapt_xlsx_pool_imputation.csv
15
10
5
0
50
100
150
Model: IVGTTmodel1.for: Minimal Model Analysis, IVGTT

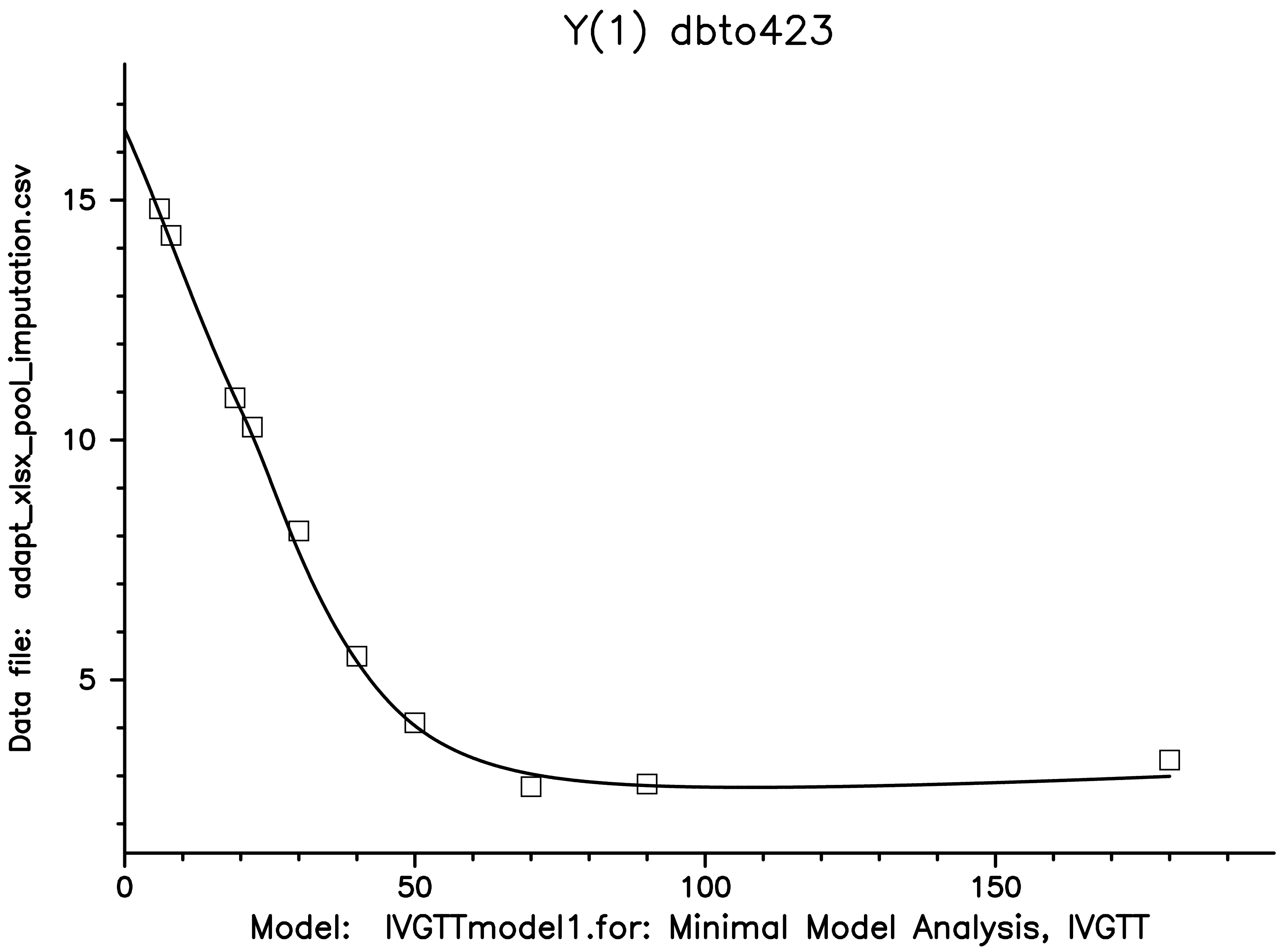
Y(1) dbto423
Data file: adapt_xlsx_pool_imputation.csv
Model: IVGTTmodel1.for: Minimal Model Analysis, IVGTT
15
10
5
0
50
100
150

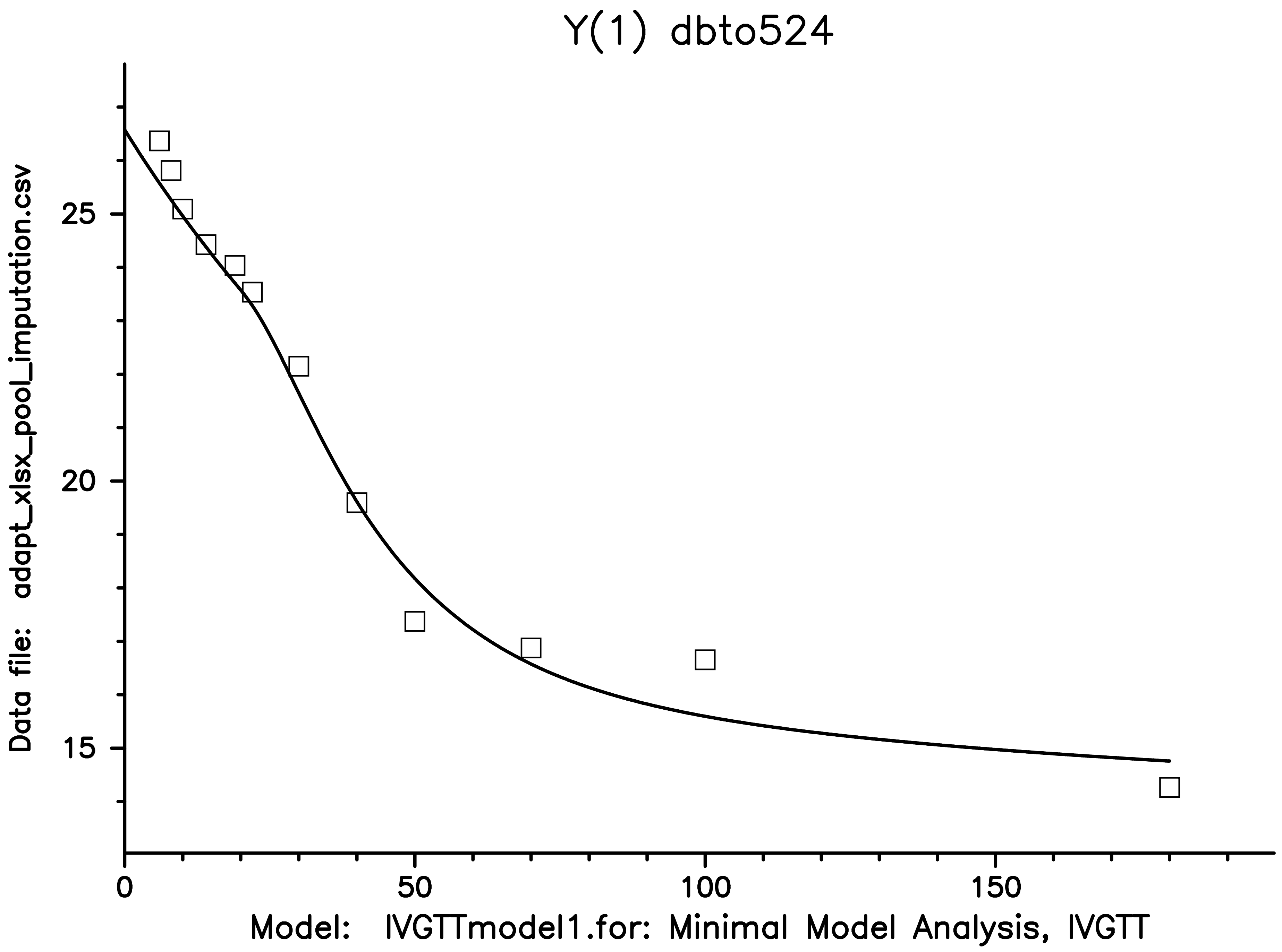
Y(1) dbto524
Data file: adapt_xlsx_pool_imputation.csv
25
20
15
0
50
100
150
Model: IVGTTmodel1.for: Minimal Model Analysis, IVGTT

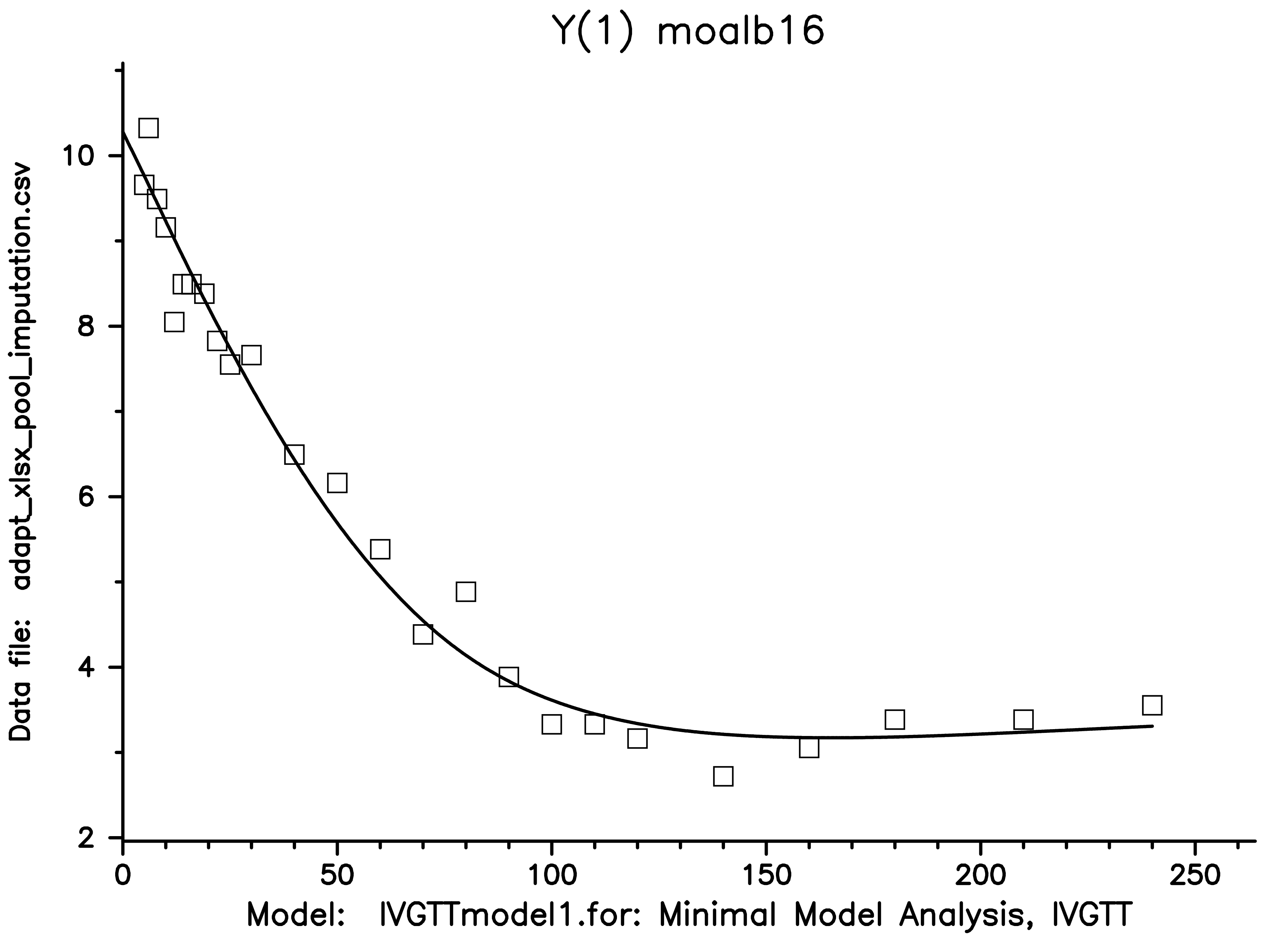

Y(1) moalb16
Data file: adapt_xlsx_pool_imputation.csv
10
8
6
4
2
0
50
100
150
200
250
Model: IVGTTmodel1.for: Minimal Model Analysis, IVGTT

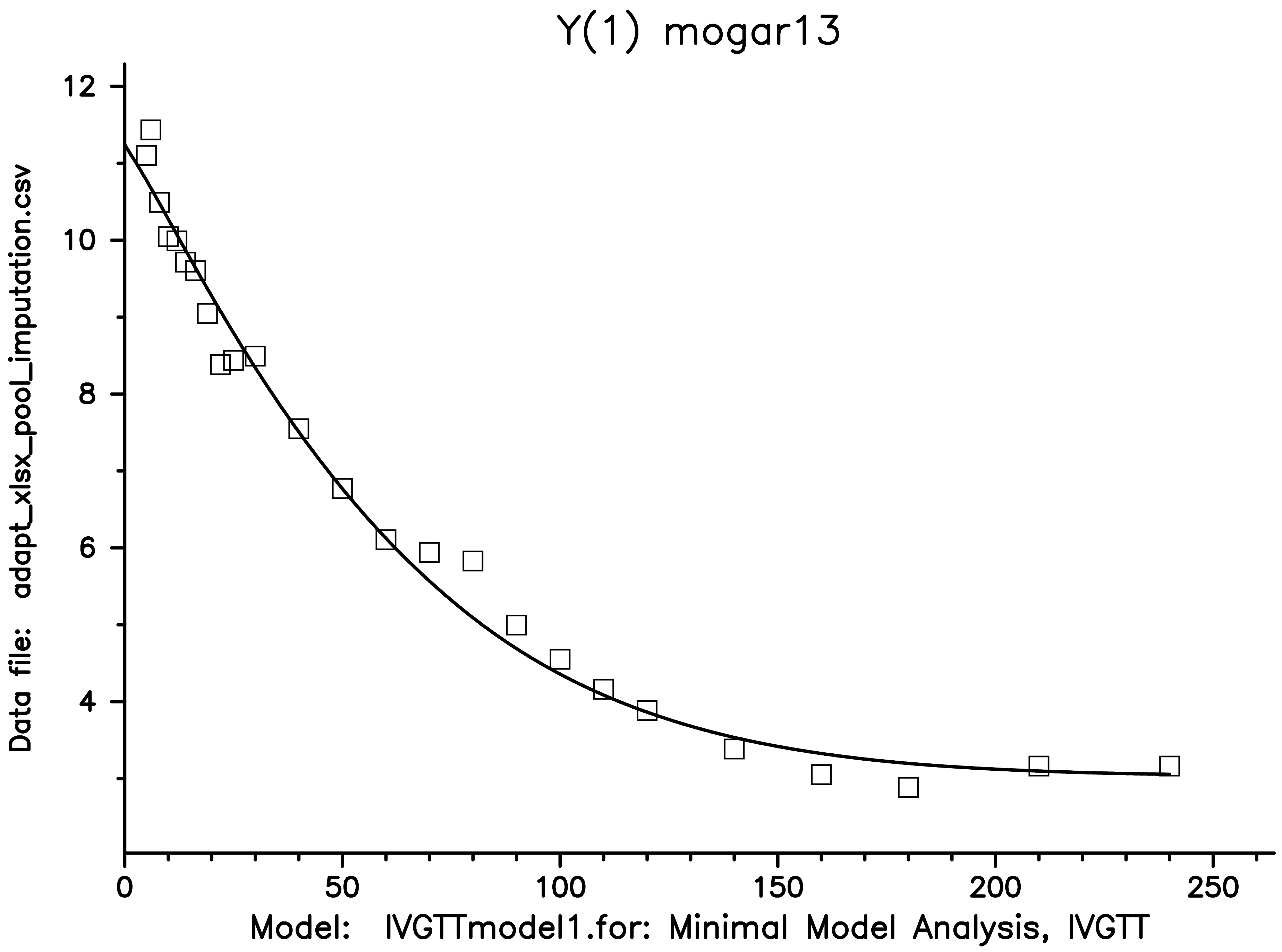
Y(1) mogar13
Data file: adapt_xlsx_pool_imputation.csv
Model: IVGTTmodel1.for: Minimal Model Analysis, IVGTT
12
10
8
6
4
0
50
100
150
200
250

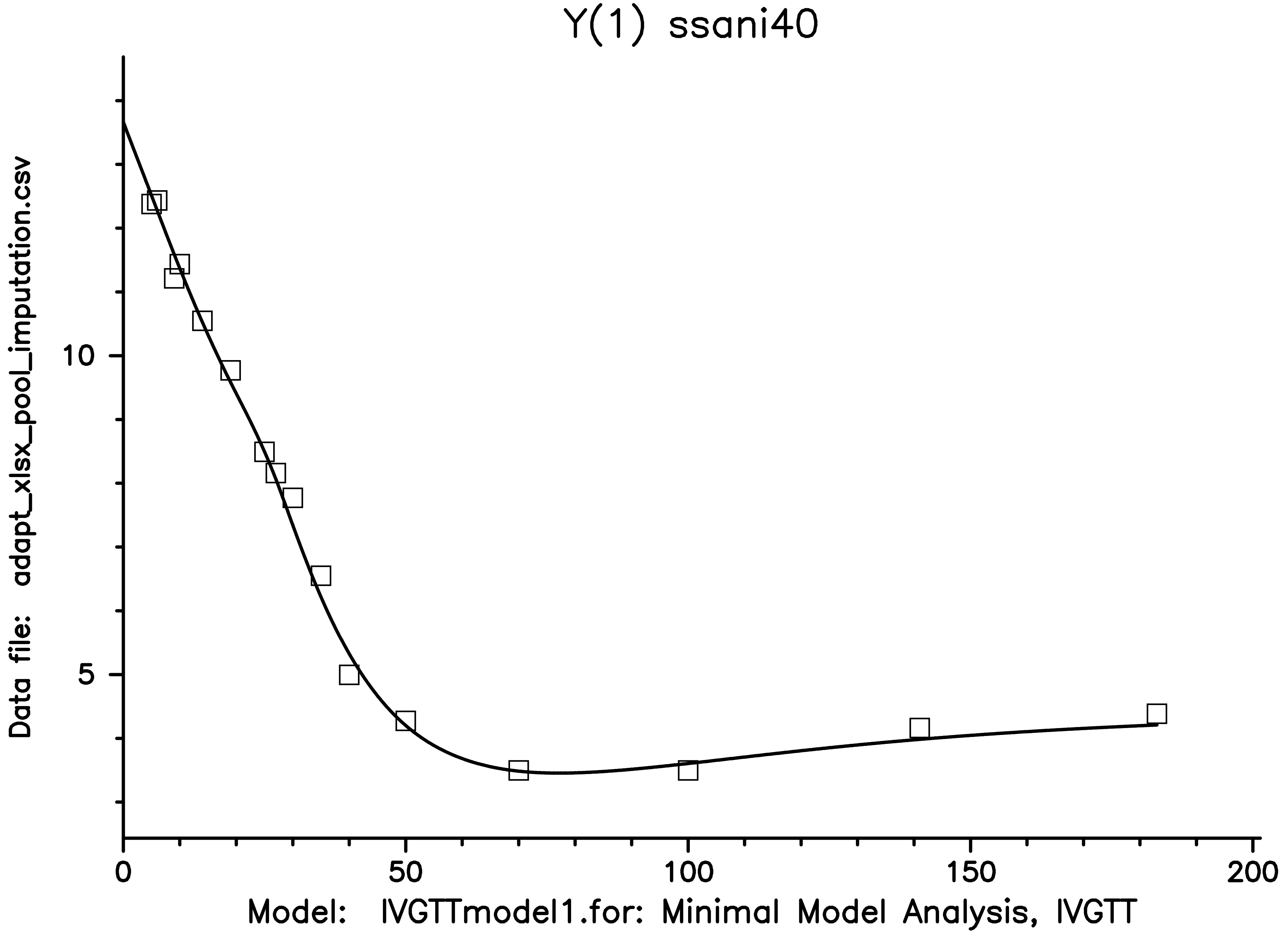

Y(1) ssani40
Data file: adapt_xlsx_pool_imputation.csv
10
5
0
50
100
150
200
Model: IVGTTmodel1.for: Minimal Model Analysis, IVGTT

Y(1) ssati24

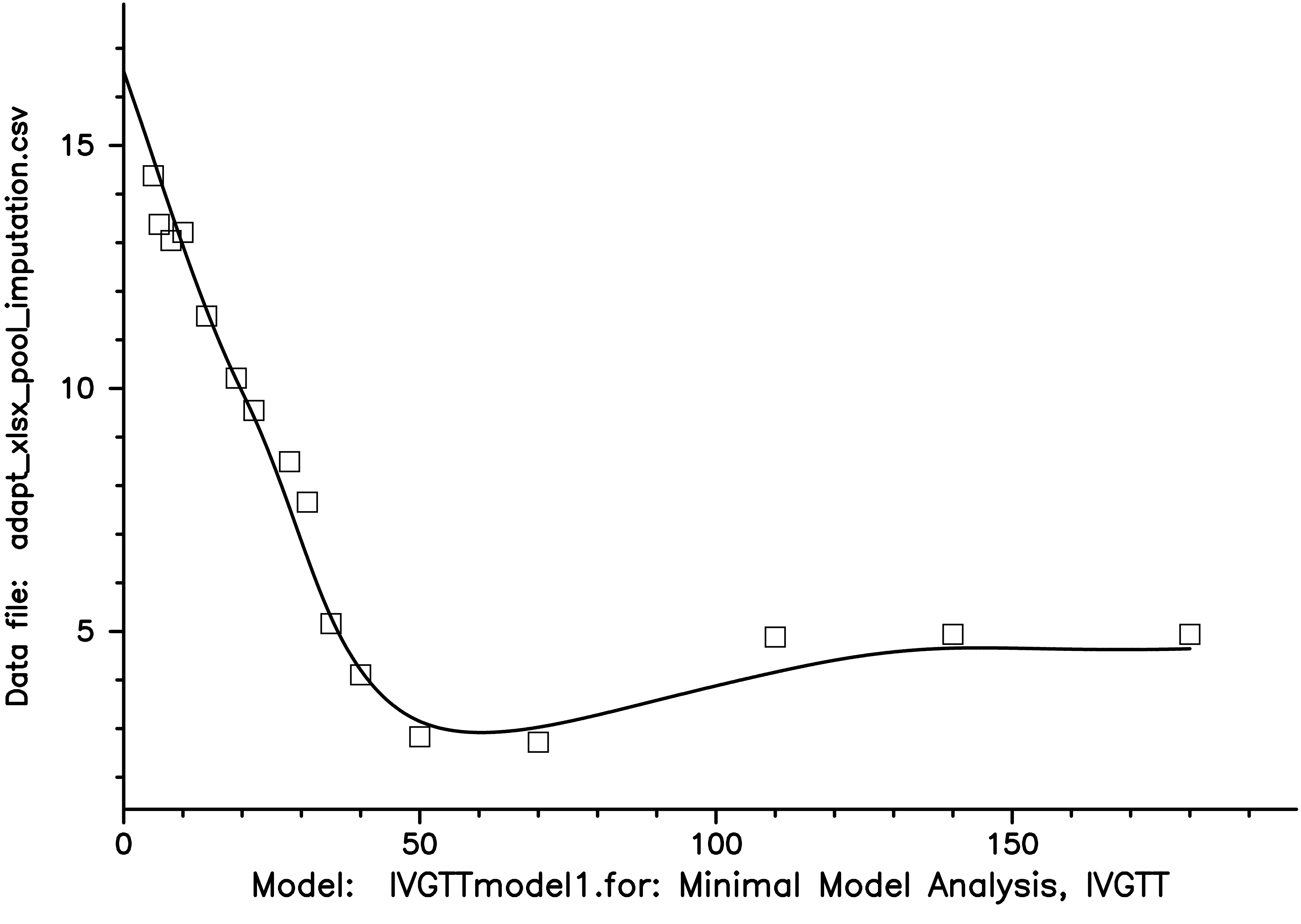

15
10
5
0
50
100
150
Data file: adapt_xlsx_pool_imputation.csv
Model: IVGTTmodel1.for: Minimal Model Analysis, IVGTT

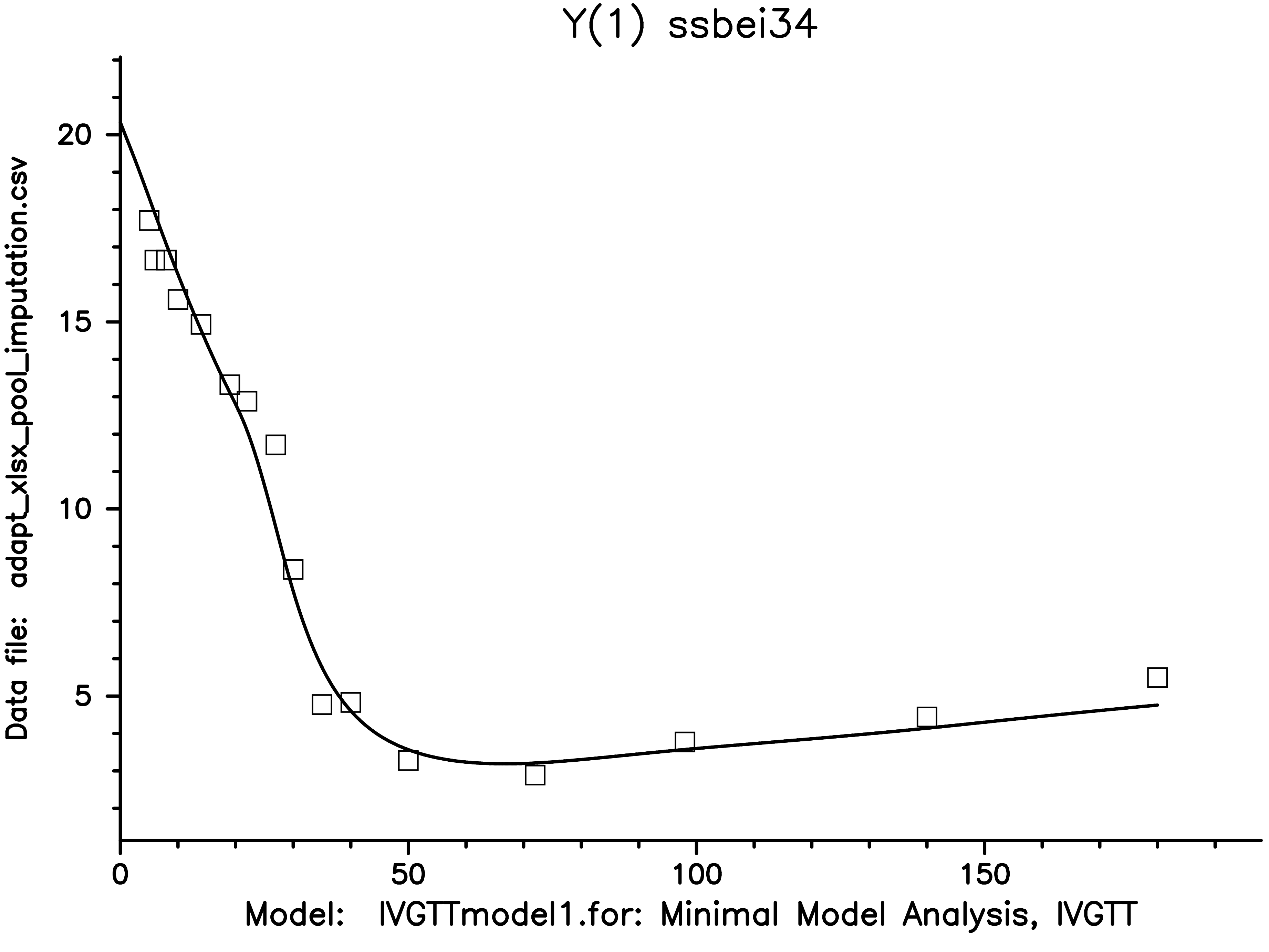
Y(1) ssbei34
Data file: adapt_xlsx_pool_imputation.csv
20
15
10
5
0
50
100
150
Model: IVGTTmodel1.for: Minimal Model Analysis, IVGTT

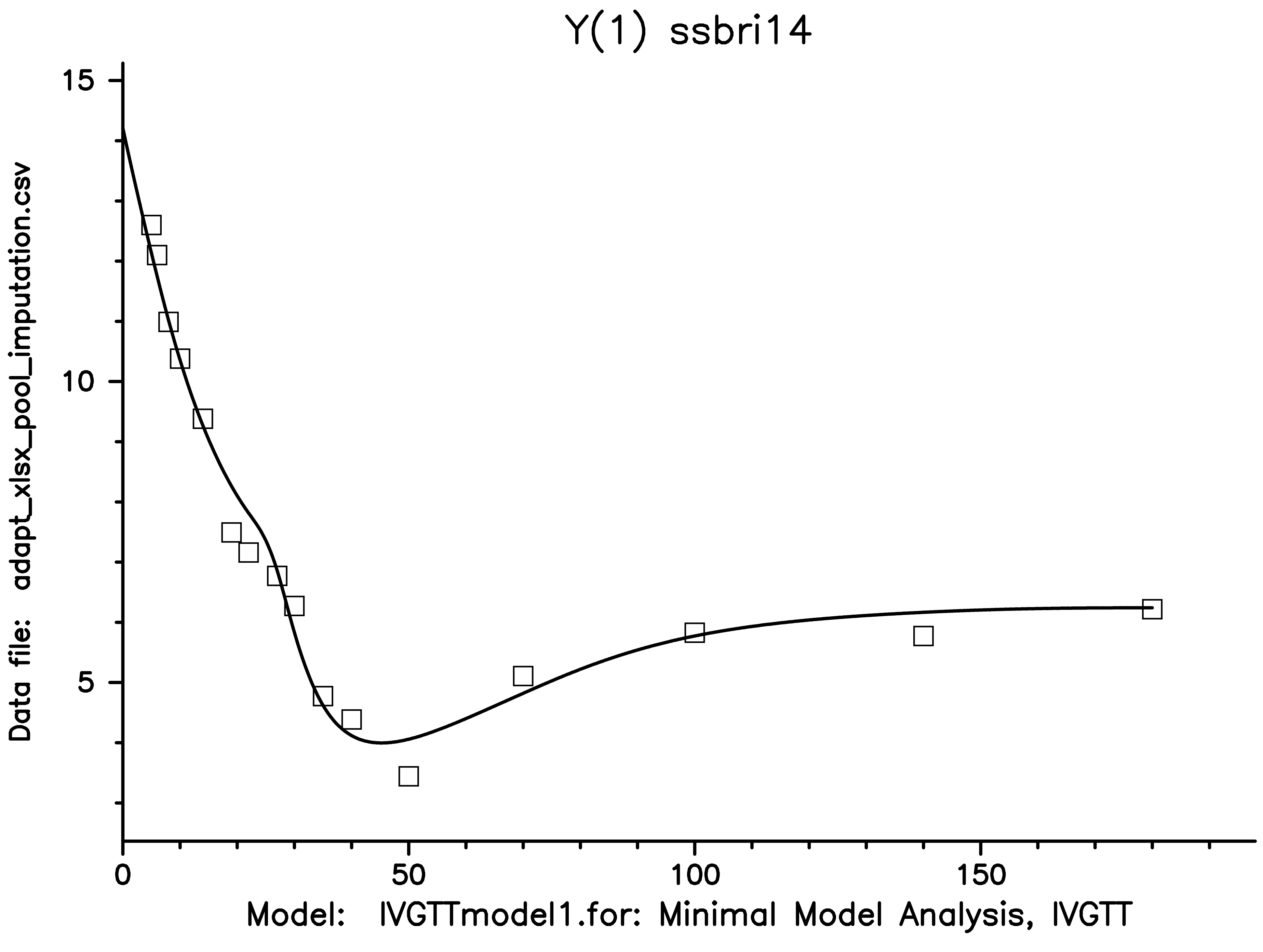
Y(1) ssbri14
Data file: adapt_xlsx_pool_imputation.csv
Model: IVGTTmodel1.for: Minimal Model Analysis, IVGTT
15
10
5
0
50
100
150

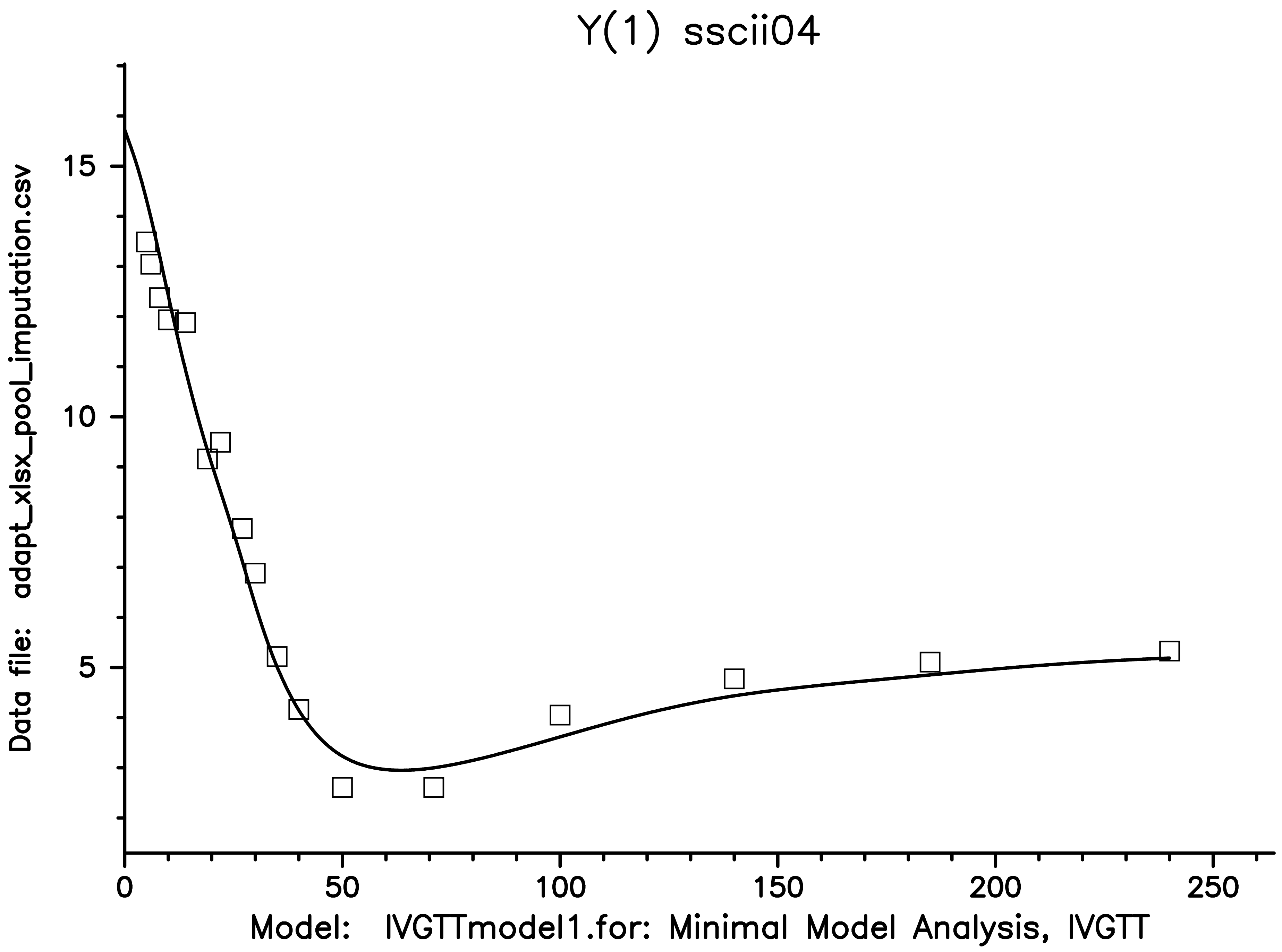
Y(1) sscii04
Data file: adapt_xlsx_pool_imputation.csv
15
10
5
0
50
100
150
200
250
Model: IVGTTmodel1.for: Minimal Model Analysis, IVGTT

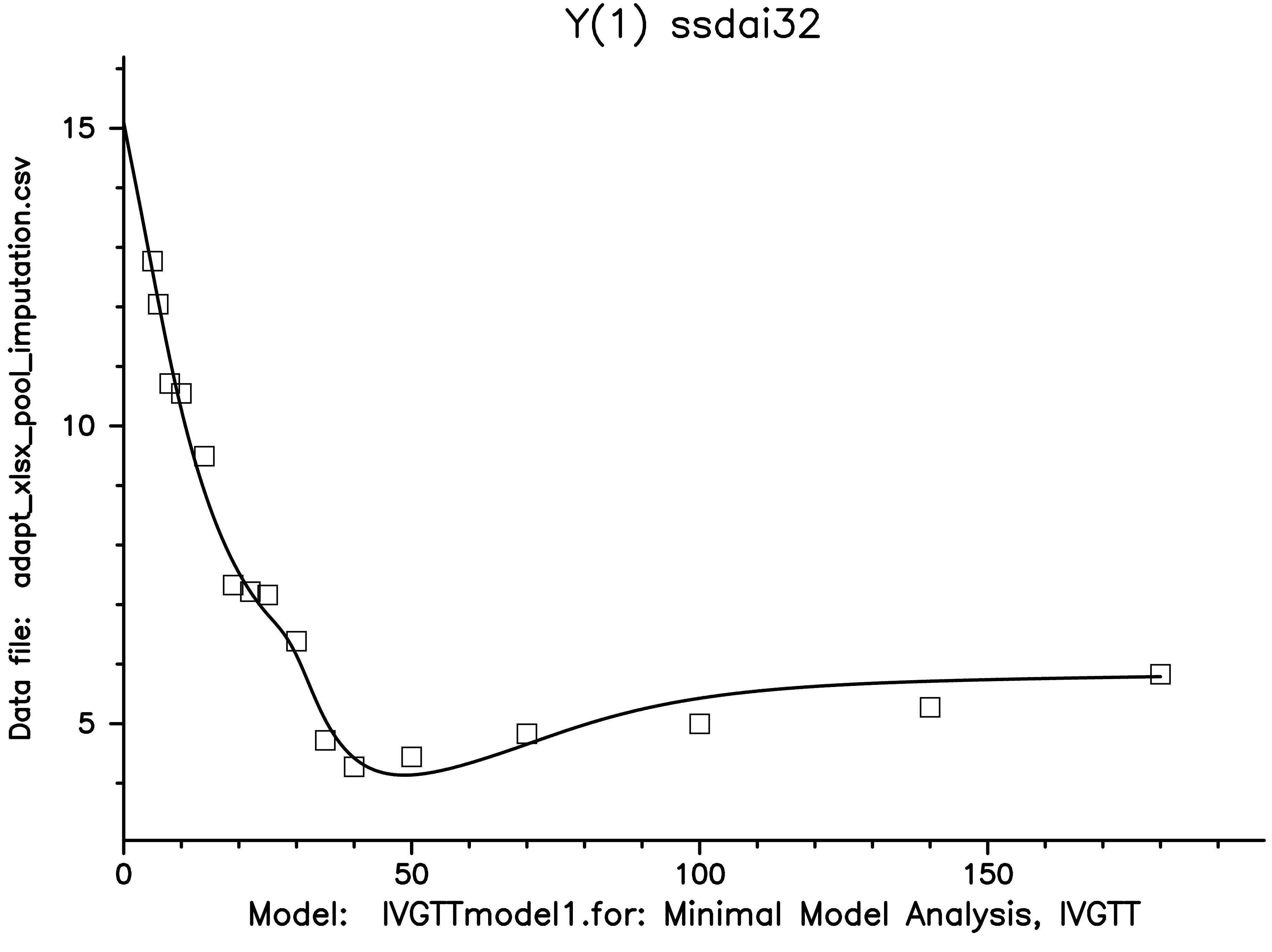

Y(1) ssdai32
Data file: adapt_xlsx_pool_imputation.csv
Model: IVGTTmodel1.for: Minimal Model Analysis, IVGTT
15
10
5
0
50
100
150

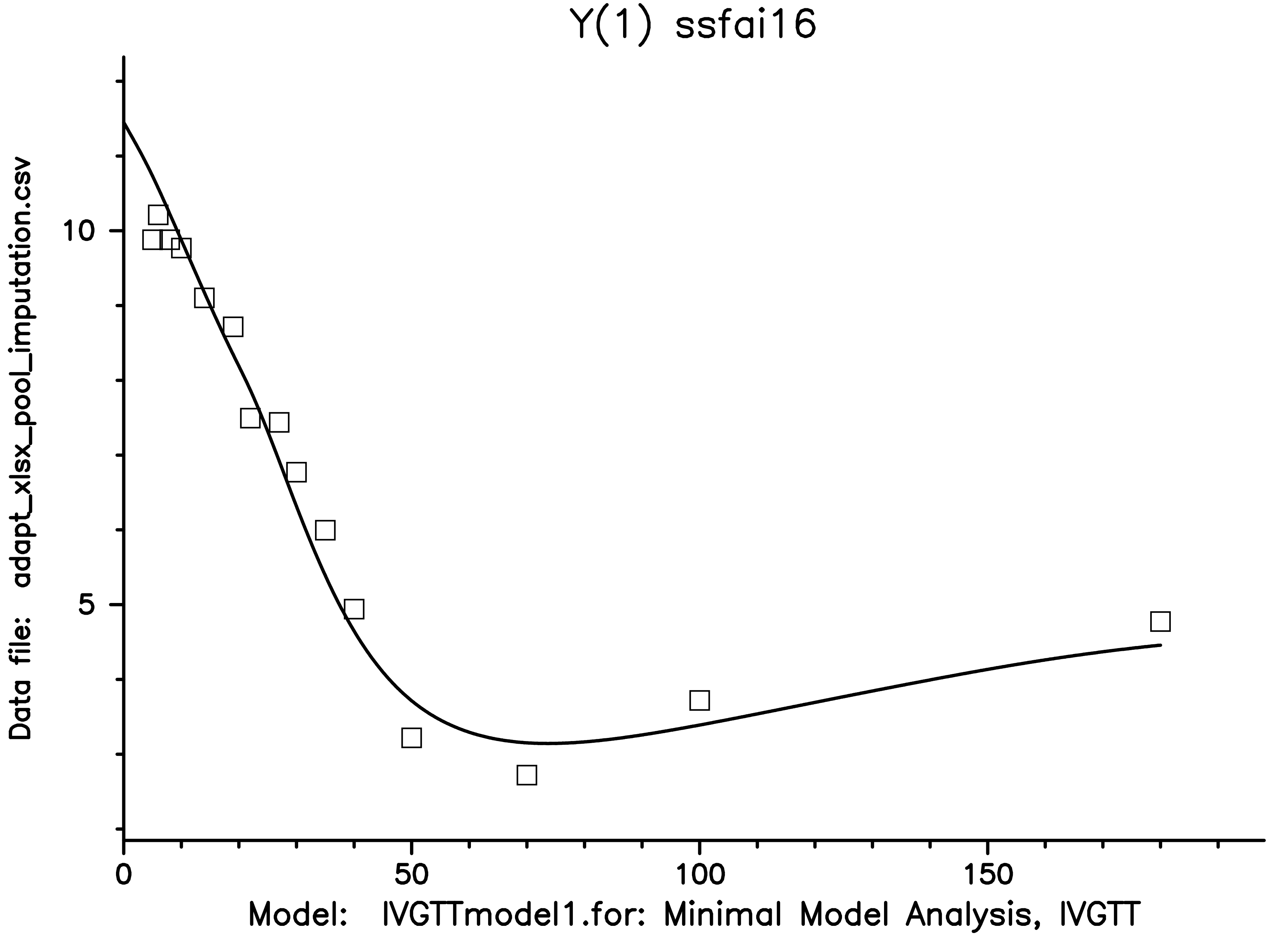
Y(1) ssfai16
Data file: adapt_xlsx_pool_imputation.csv
10
5
0
50
100
150
Model: IVGTTmodel1.for: Minimal Model Analysis, IVGTT

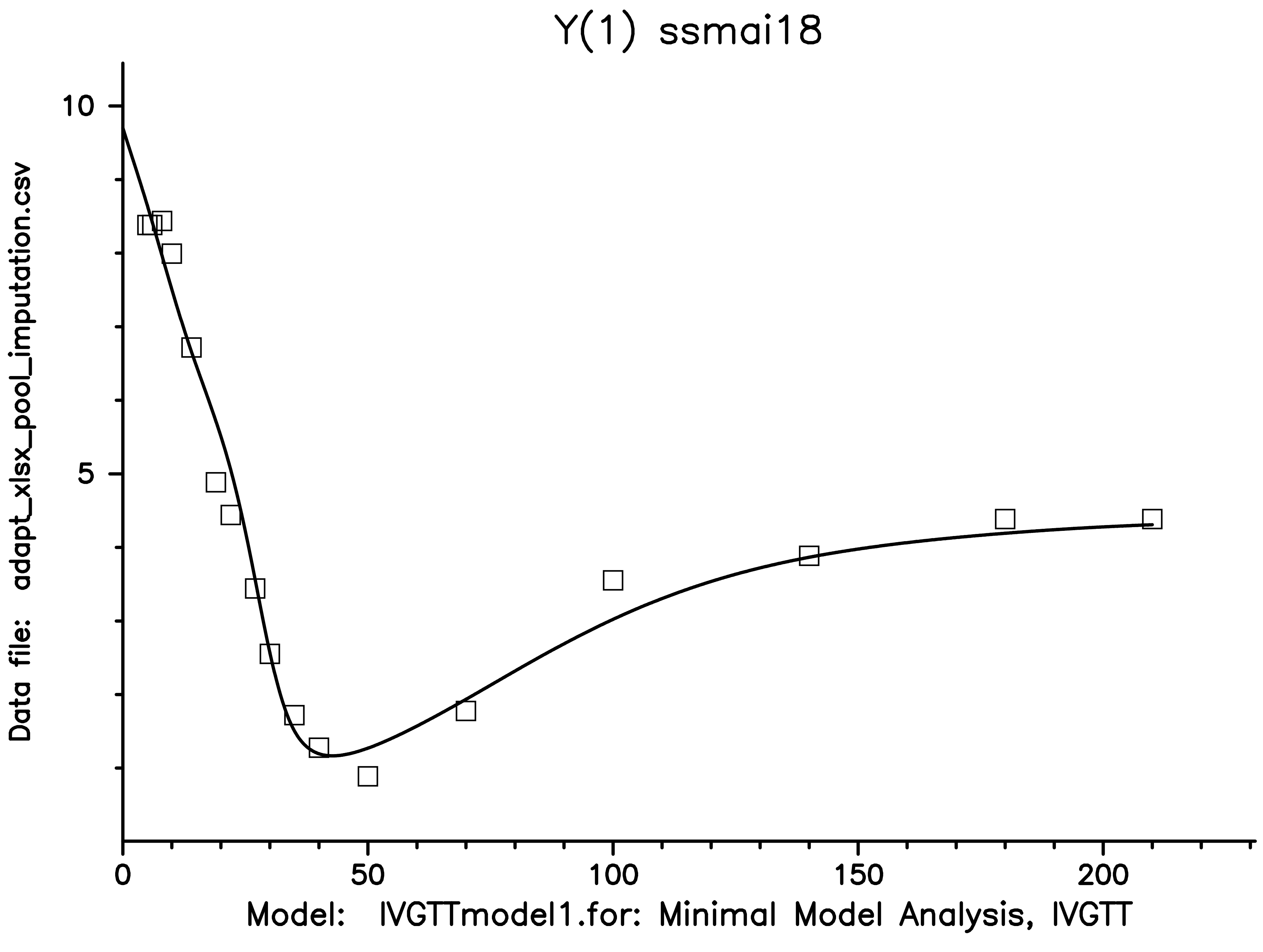
Y(1) ssmai18
10
5
0
50
100
150
200
Data file: adapt_xlsx_pool_imputation.csv
Model: IVGTTmodel1.for: Minimal Model Analysis, IVGTT

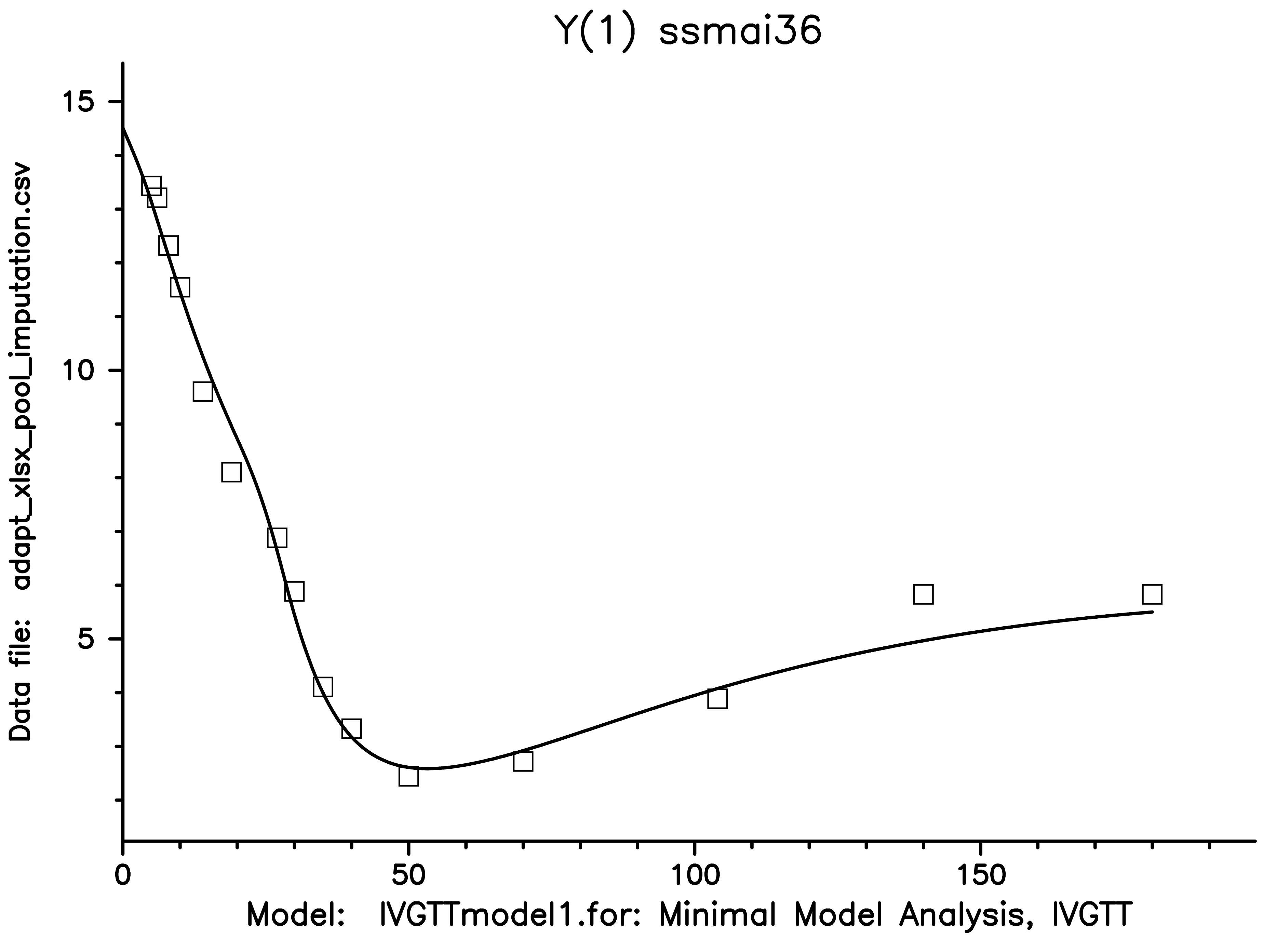
Y(1) ssmai36
Data file: adapt_xlsx_pool_imputation.csv
15
10
5
0
50
100
150
Model: IVGTTmodel1.for: Minimal Model Analysis, IVGTT

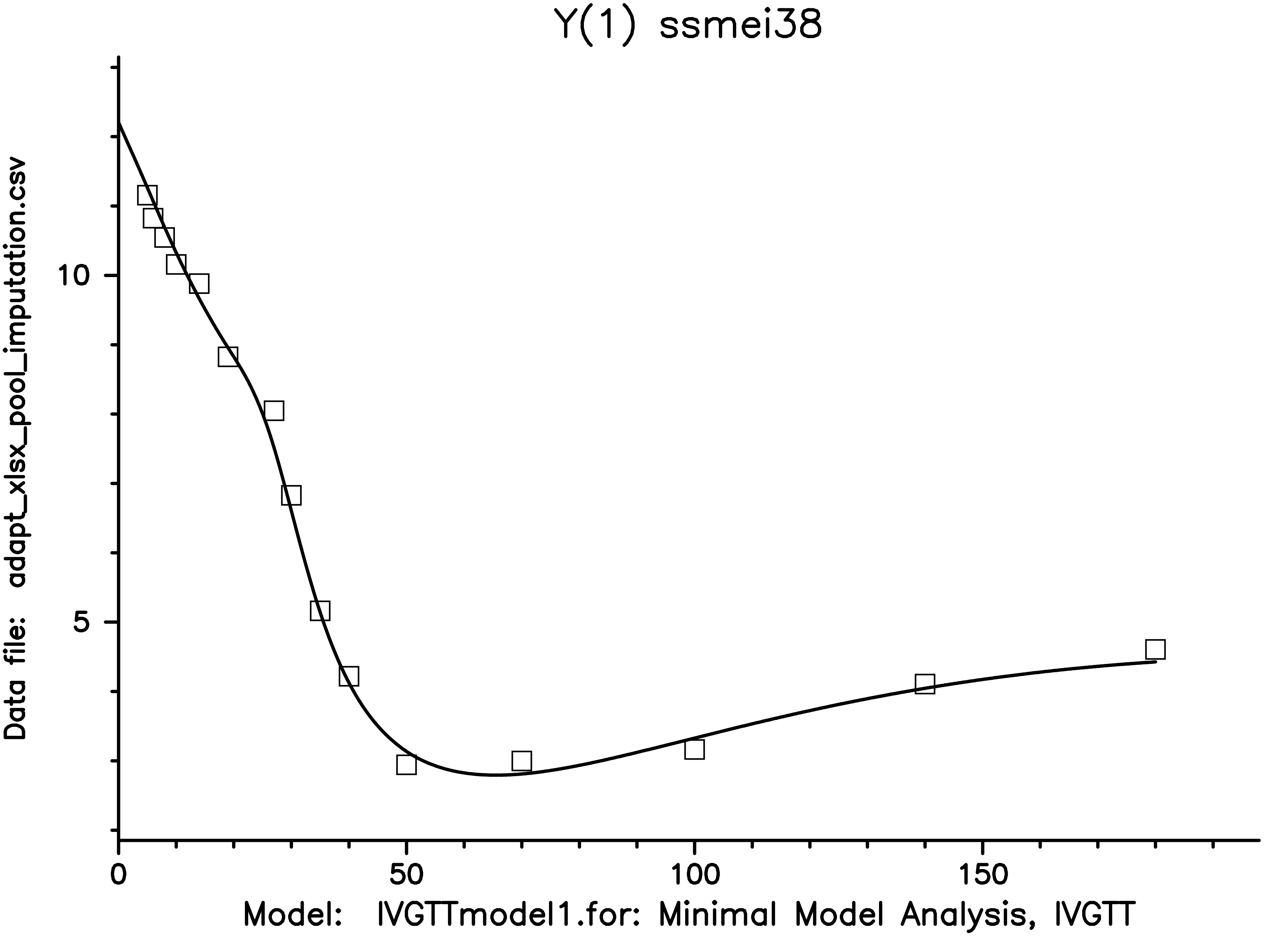

Y(1) ssmei38
Data file: adapt_xlsx_pool_imputation.csv
10
5
0
50
100
150
Model: IVGTTmodel1.for: Minimal Model Analysis, IVGTT

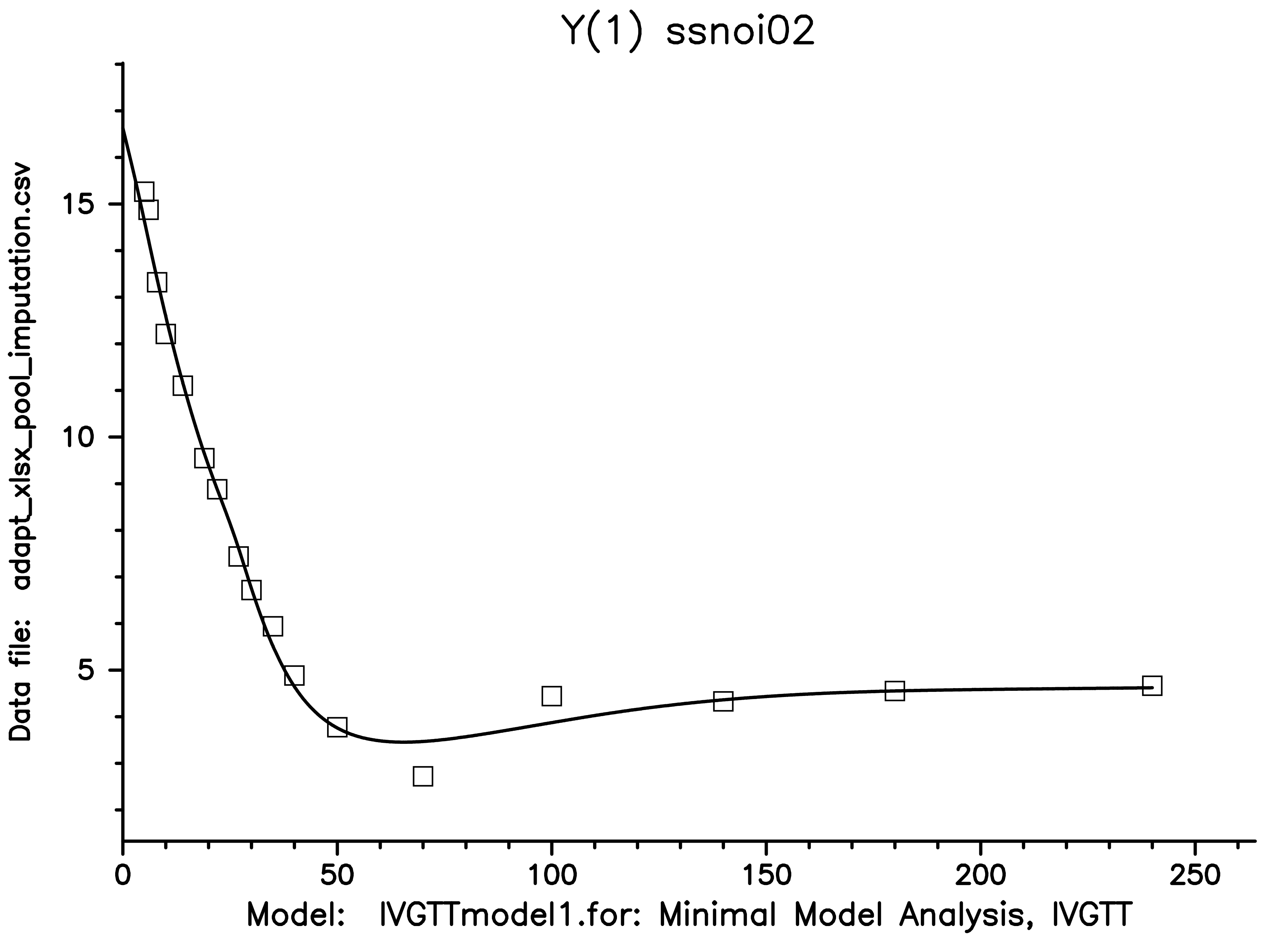
Y(1) ssnoi02
Data file: adapt_xlsx_pool_imputation.csv
Model: IVGTTmodel1.for: Minimal Model Analysis, IVGTT
0
50
100
150
200
250
5
10
15

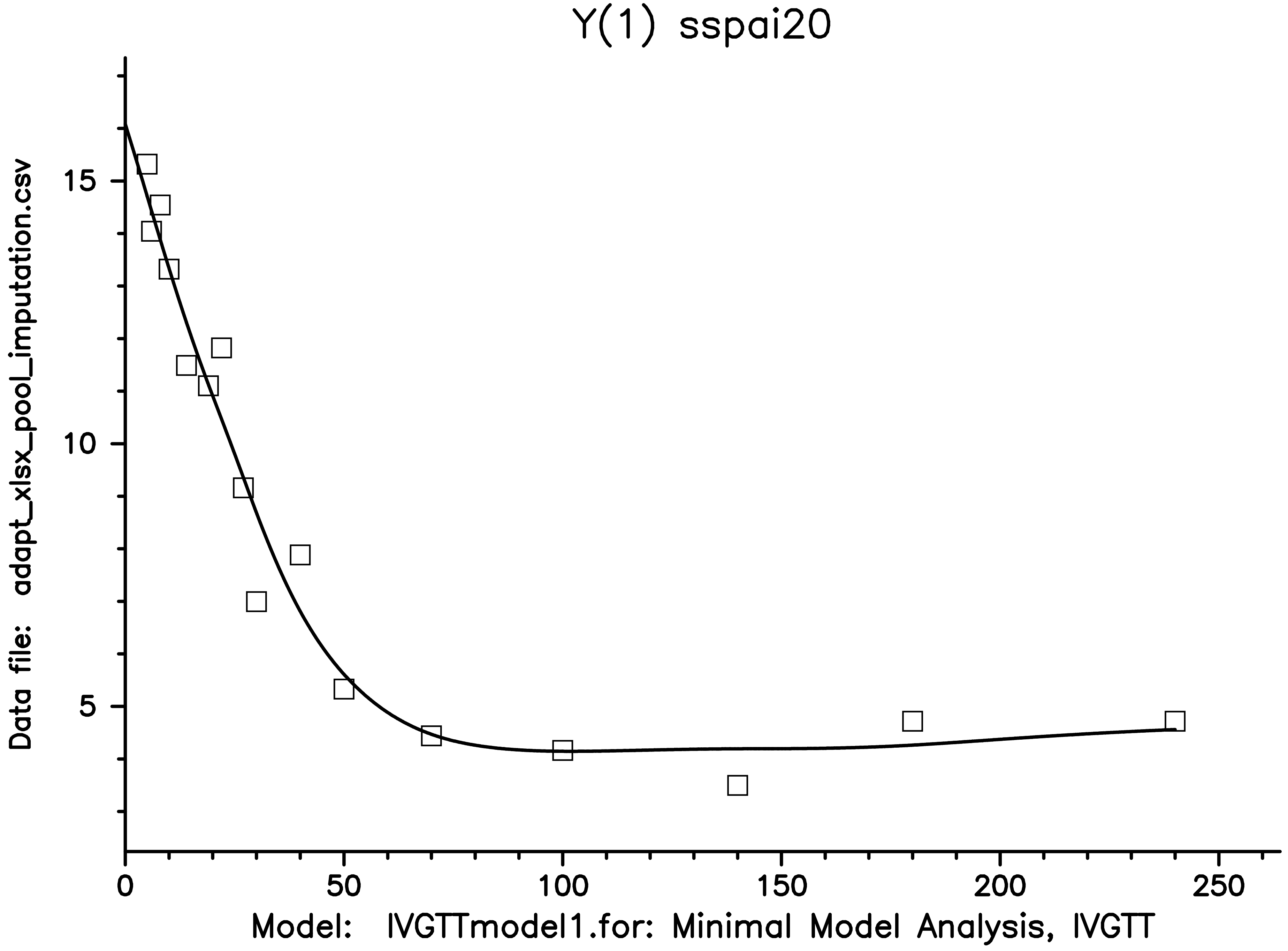
Y(1) sspai20
Data file: adapt_xlsx_pool_imputation.csv
15
10
5
0
50
100
150
200
250
Model: IVGTTmodel1.for: Minimal Model Analysis, IVGTT

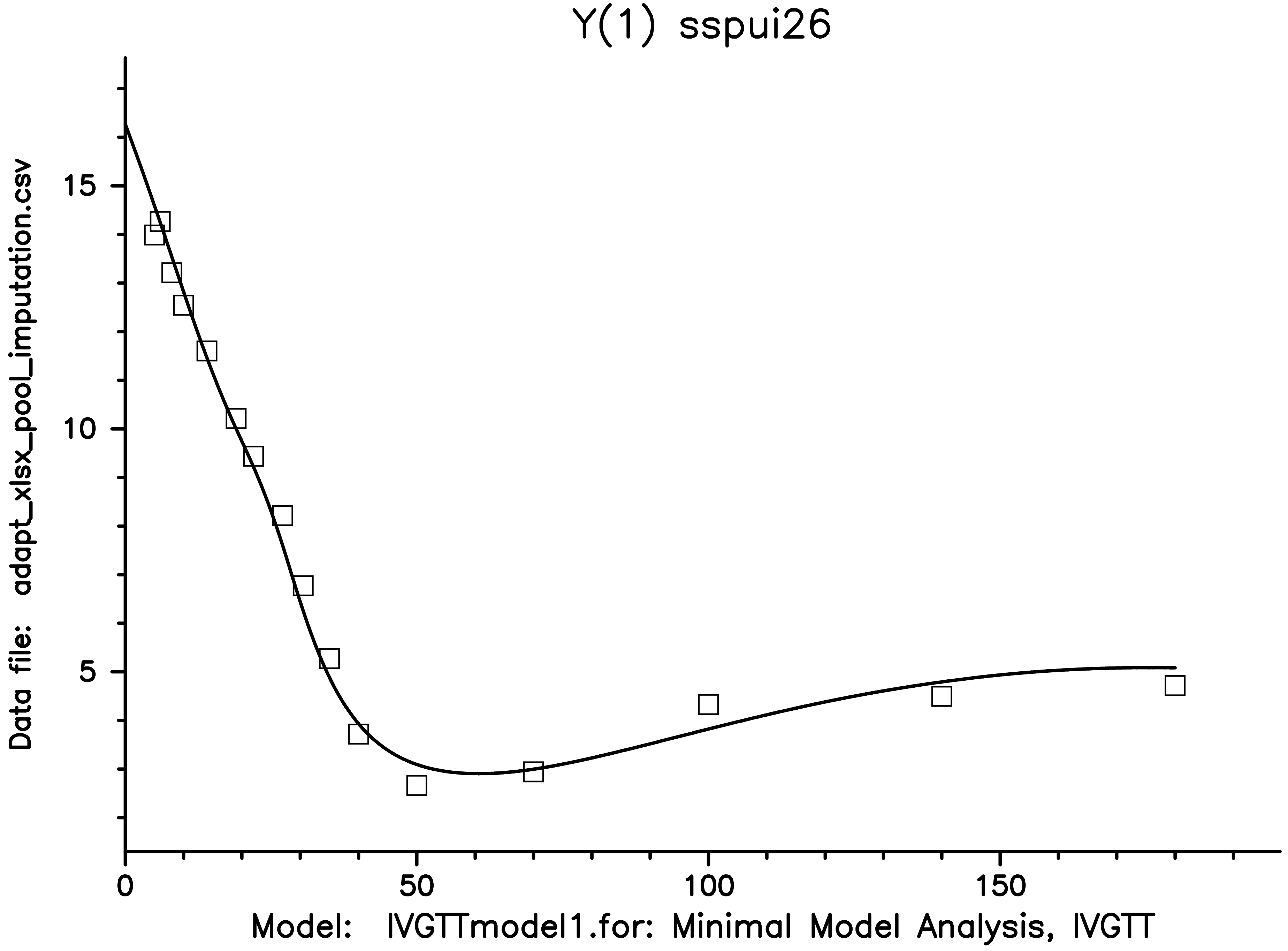

Y(1) sspui26
Data file: adapt_xlsx_pool_imputation.csv
15
10
5
0
50
100
150
Model: IVGTTmodel1.for: Minimal Model Analysis, IVGTT

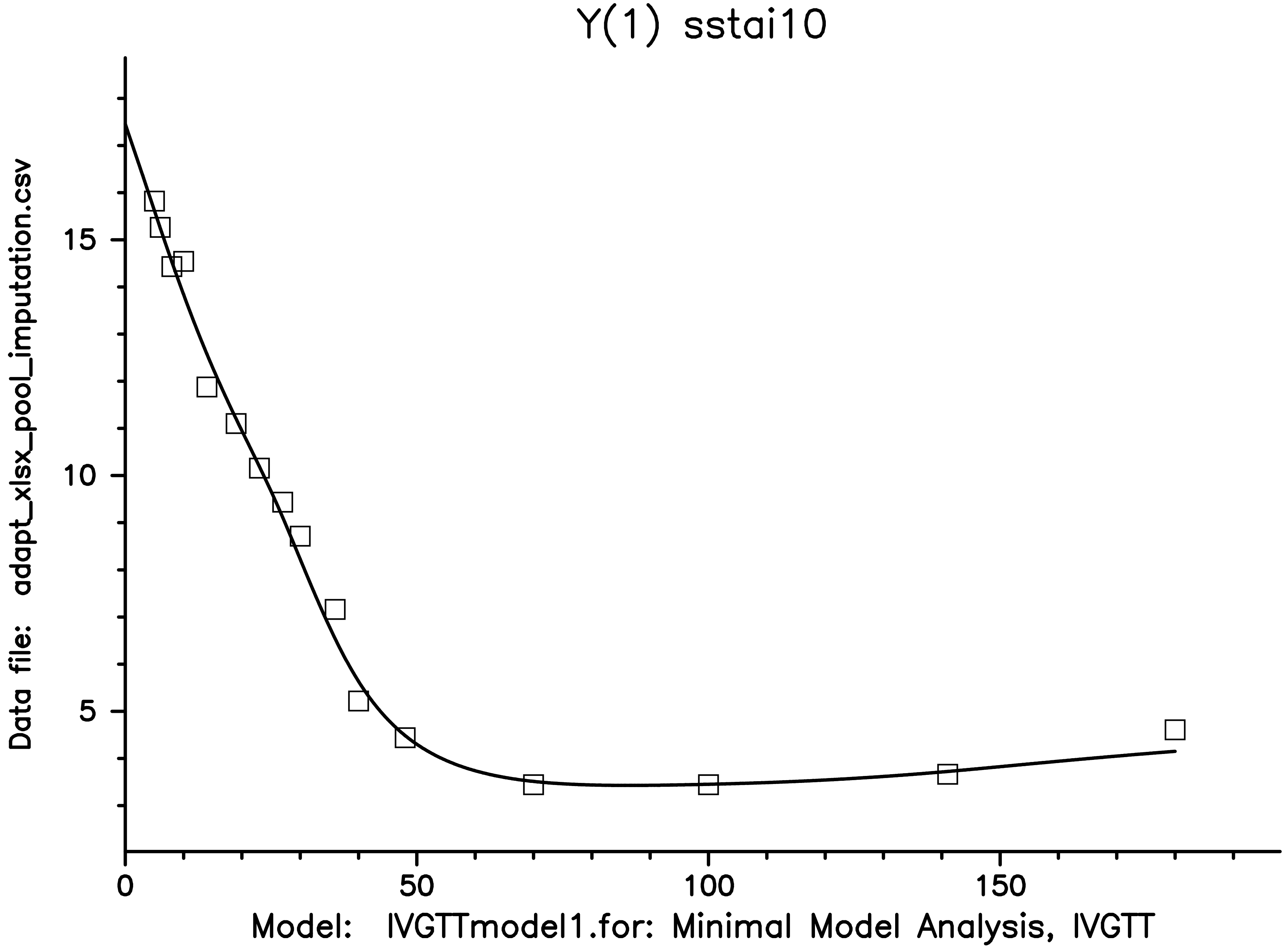
Y(1) sstai10
Data file: adapt_xlsx_pool_imputation.csv
15
10
5
0
50
100
150
Model: IVGTTmodel1.for: Minimal Model Analysis, IVGTT

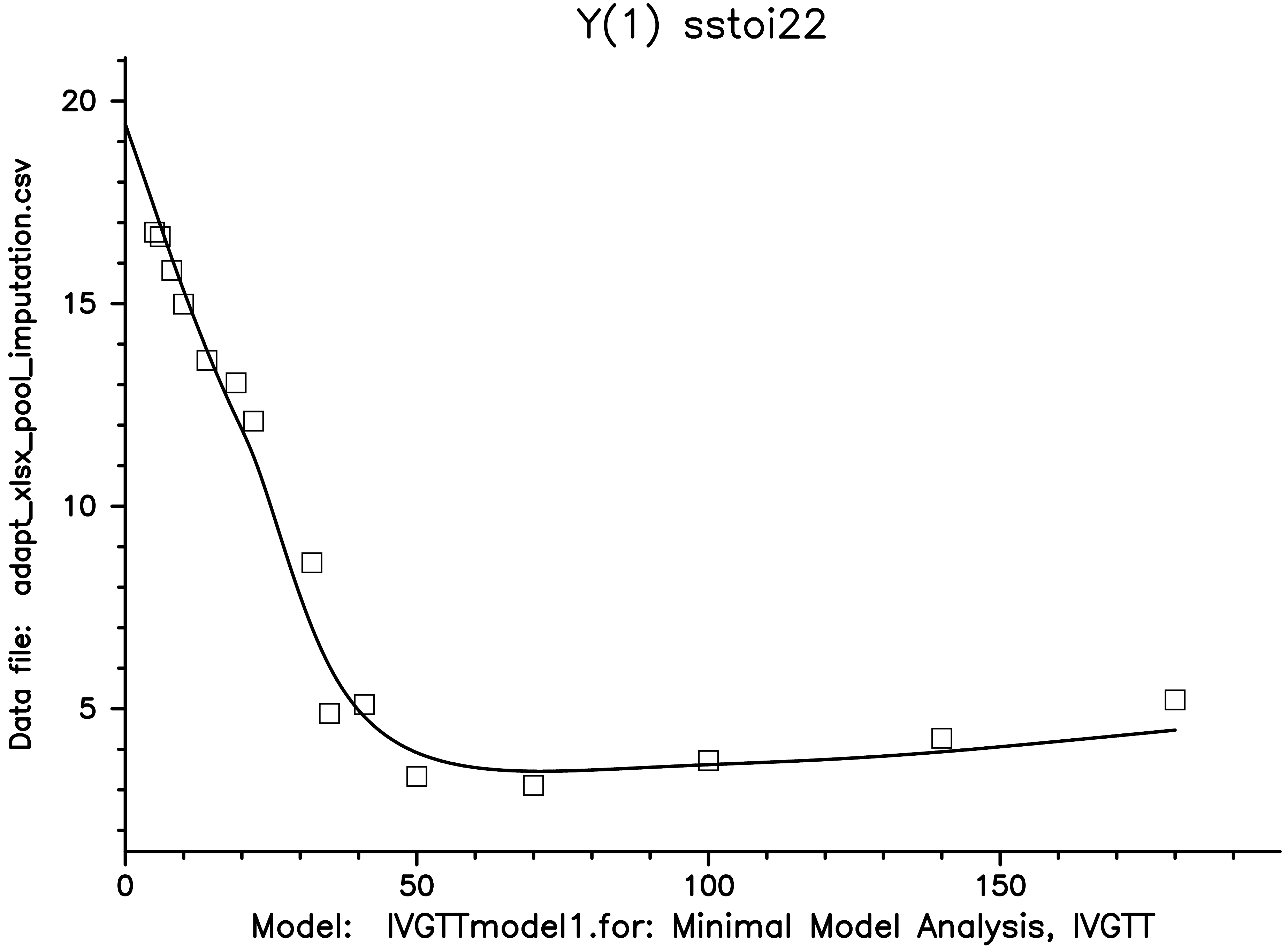
Y(1) sstoi22
Data file: adapt_xlsx_pool_imputation.csv
20
15
10
5
0
50
100
150
Model: IVGTTmodel1.for: Minimal Model Analysis, IVGTT

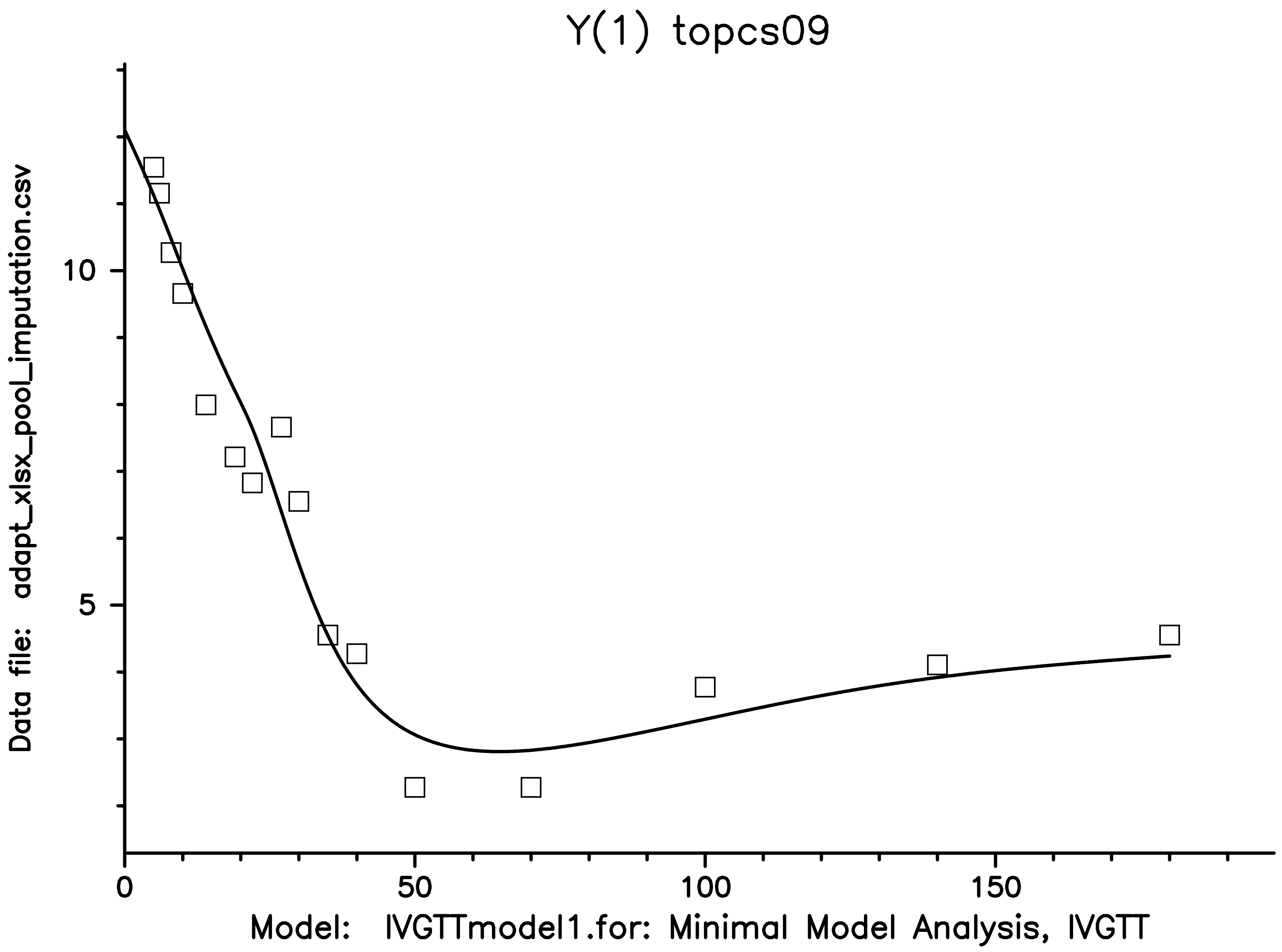
Y(1) topcs09
Data file: adapt_xlsx_pool_imputation.csv
10
5
0
50
100
150
Model: IVGTTmodel1.for: Minimal Model Analysis, IVGTT

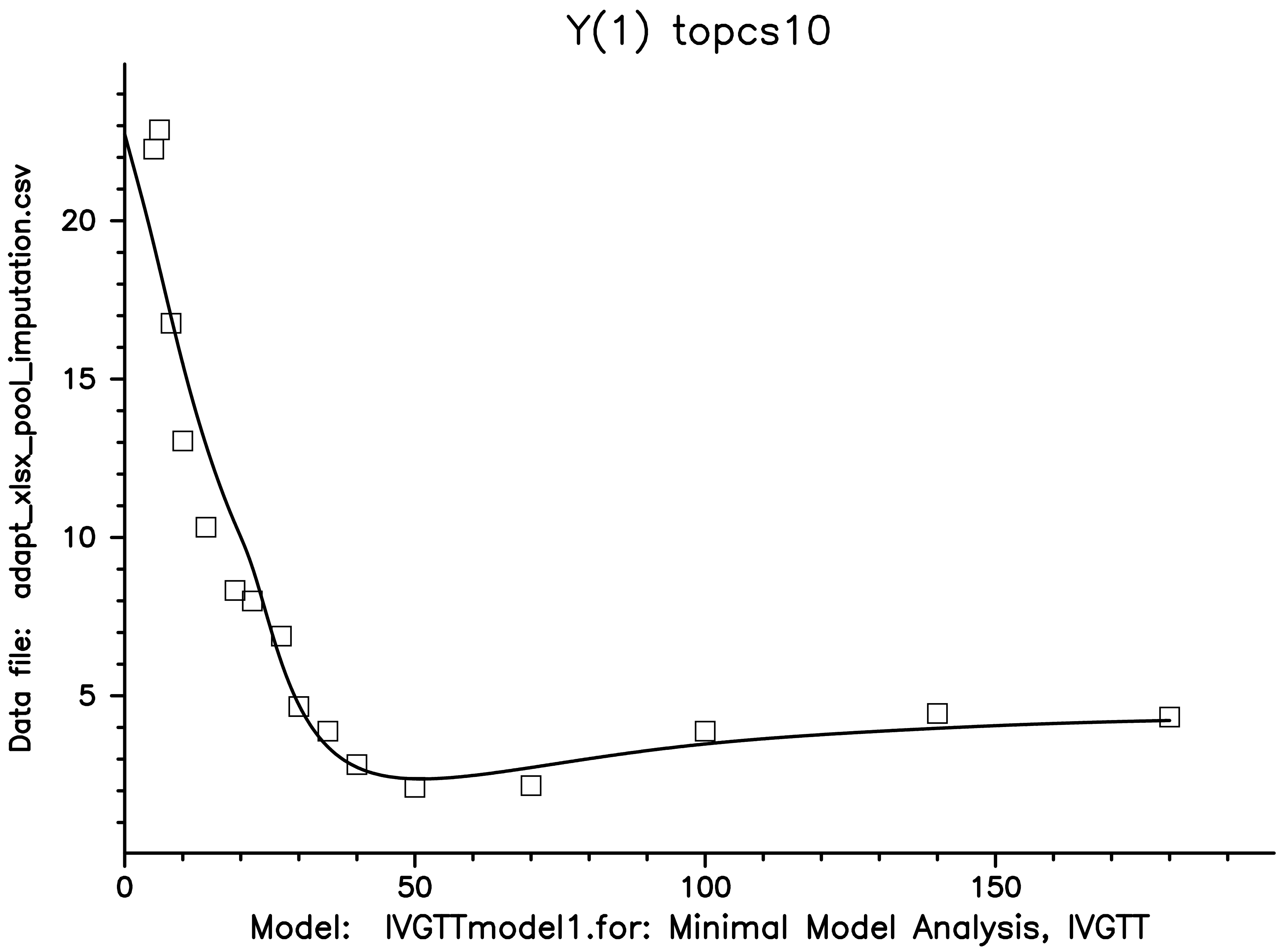

Y(1) topcs10
Data file: adapt_xlsx_pool_imputation.csv
20
15
10
5
0
50
100
150
Model: IVGTTmodel1.for: Minimal Model Analysis, IVGTT

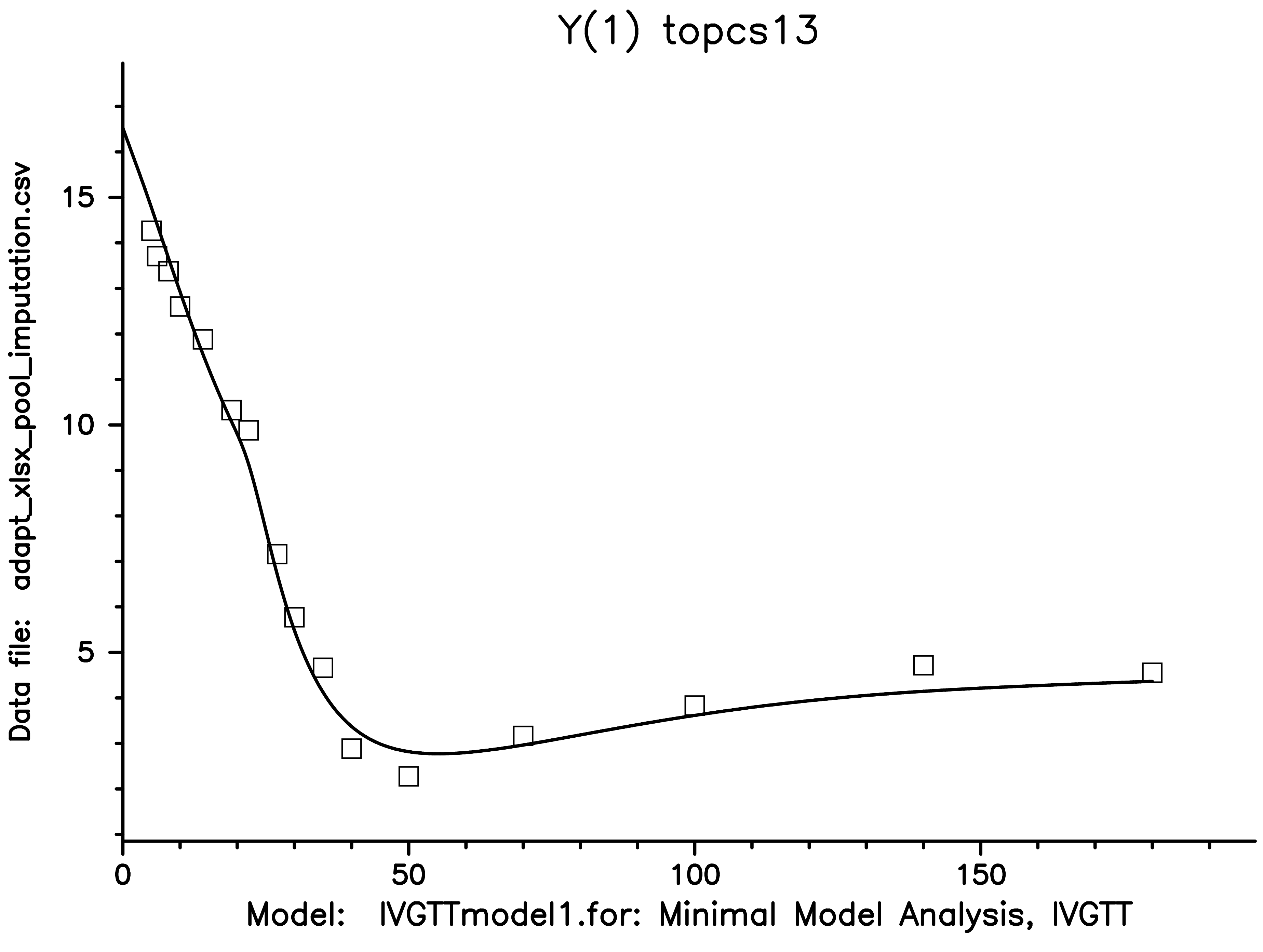

Y(1) topcs13
Data file: adapt_xlsx_pool_imputation.csv
15
10
5
0
50
100
150
Model: IVGTTmodel1.for: Minimal Model Analysis, IVGTT

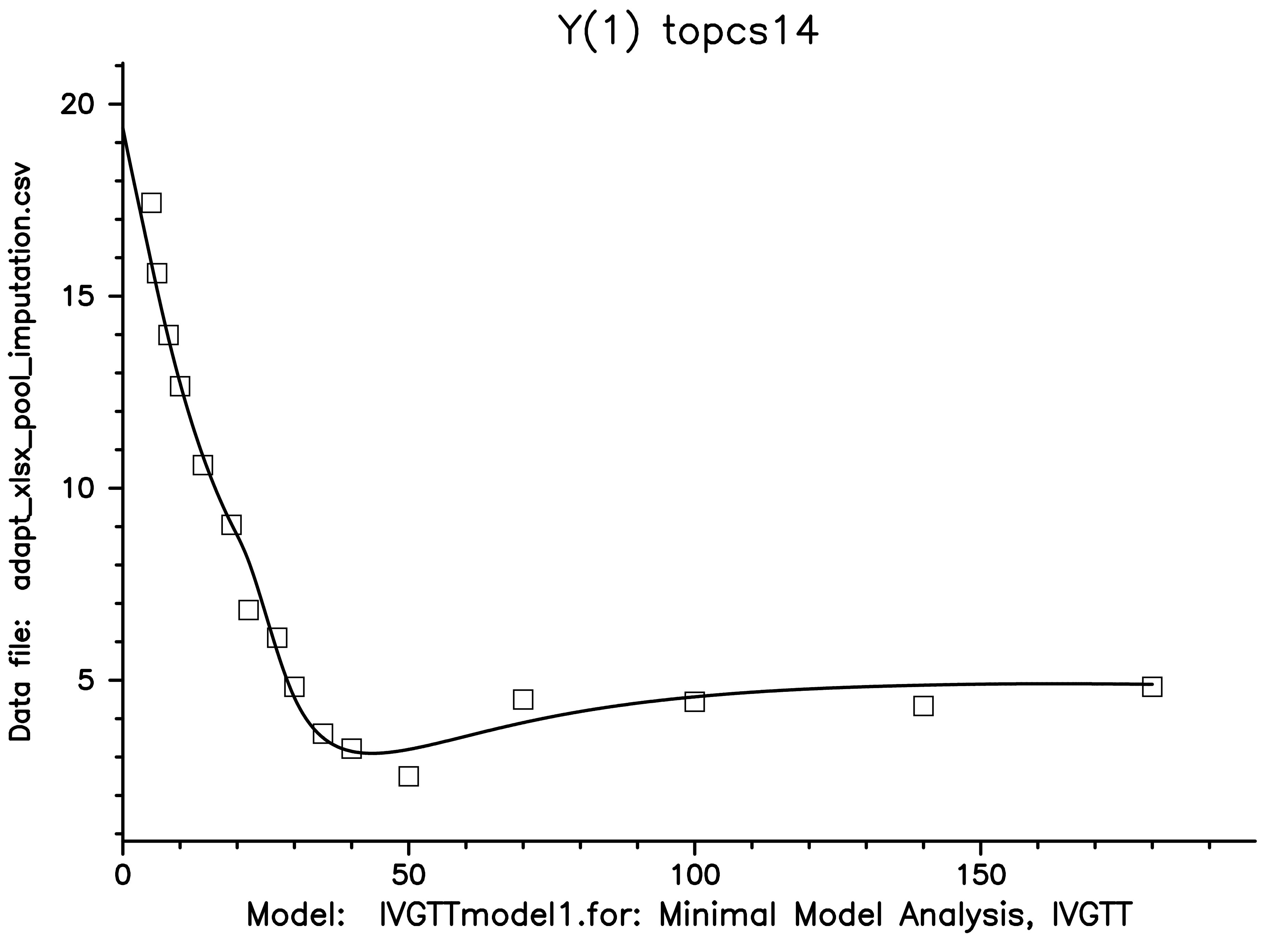

Y(1) topcs14
20
15
10
5
0
50
100
150
Data file: adapt_xlsx_pool_imputation.csv
Model: IVGTTmodel1.for: Minimal Model Analysis, IVGTT

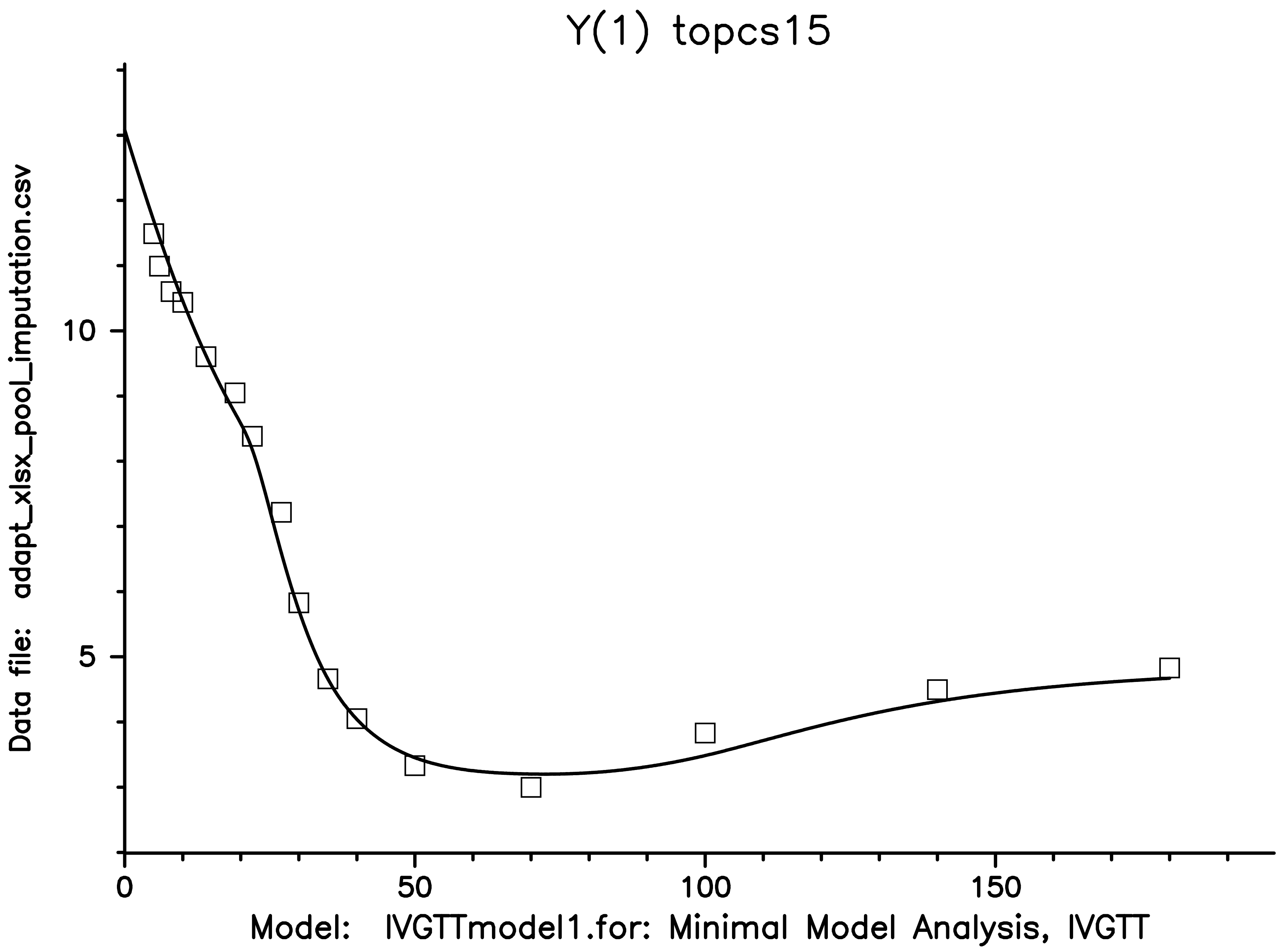
Y(1) topcs15
Data file: adapt_xlsx_pool_imputation.csv
Model: IVGTTmodel1.for: Minimal Model Analysis, IVGTT
10
5
0
50
100
150

Y(1) topcs19

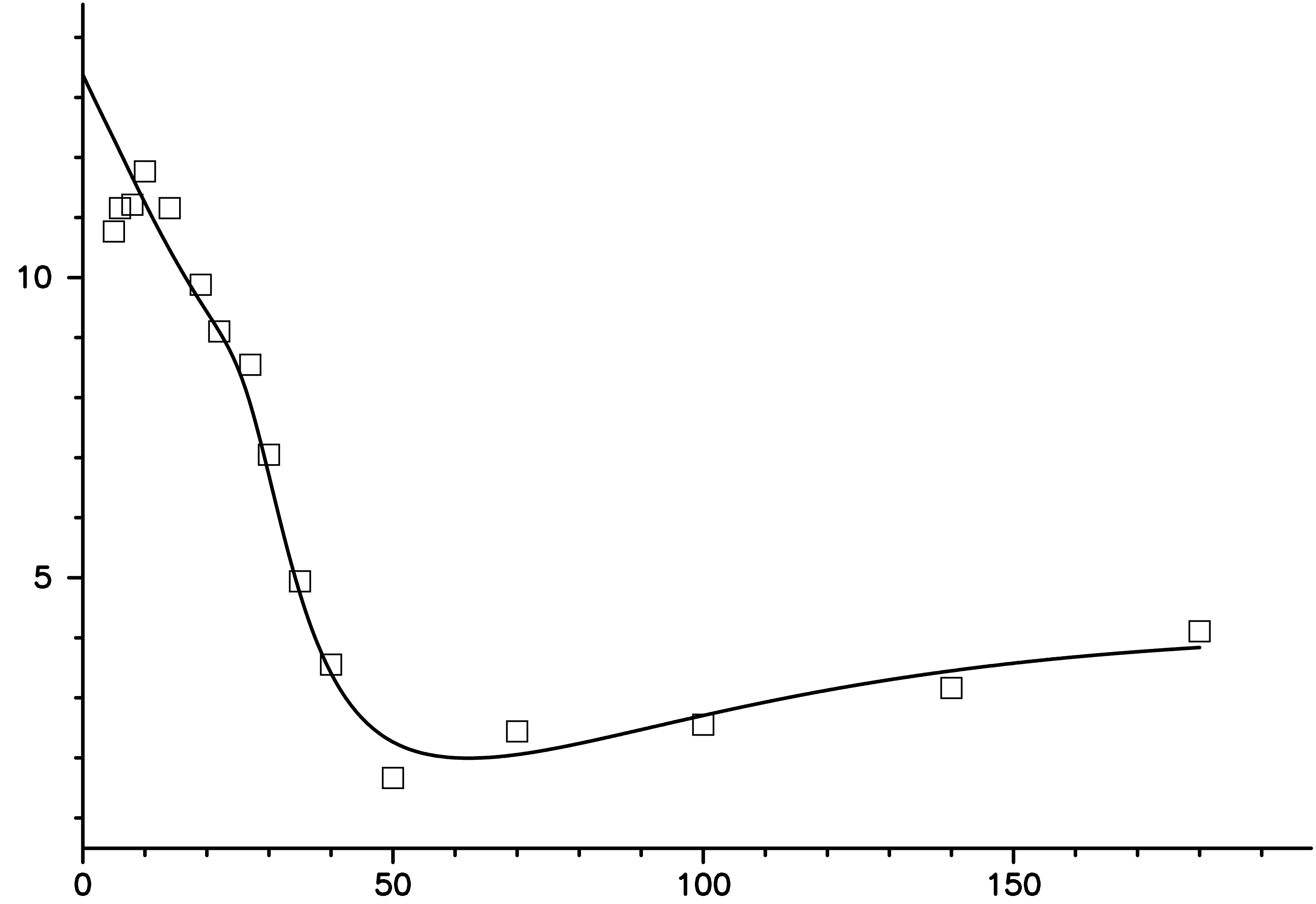

Data file: adapt_xlsx_pool_imputation.csv

Model: IVGTTmodel1.for: Minimal Model Analysis, IVGTT

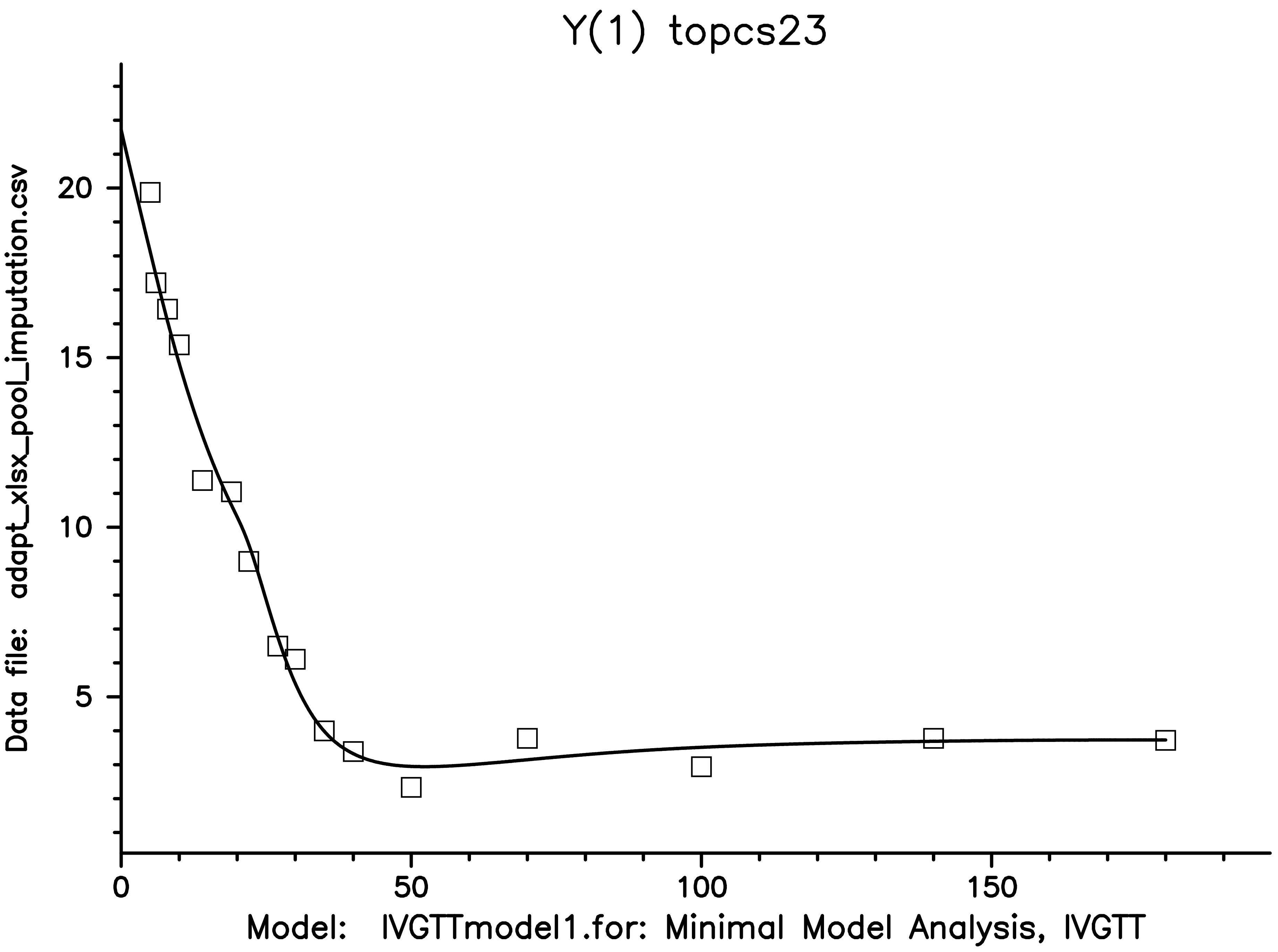
Y(1) topcs23
Data file: adapt_xlsx_pool_imputation.csv
20
15
10
5
0
50
100
150
Model: IVGTTmodel1.for: Minimal Model Analysis, IVGTT

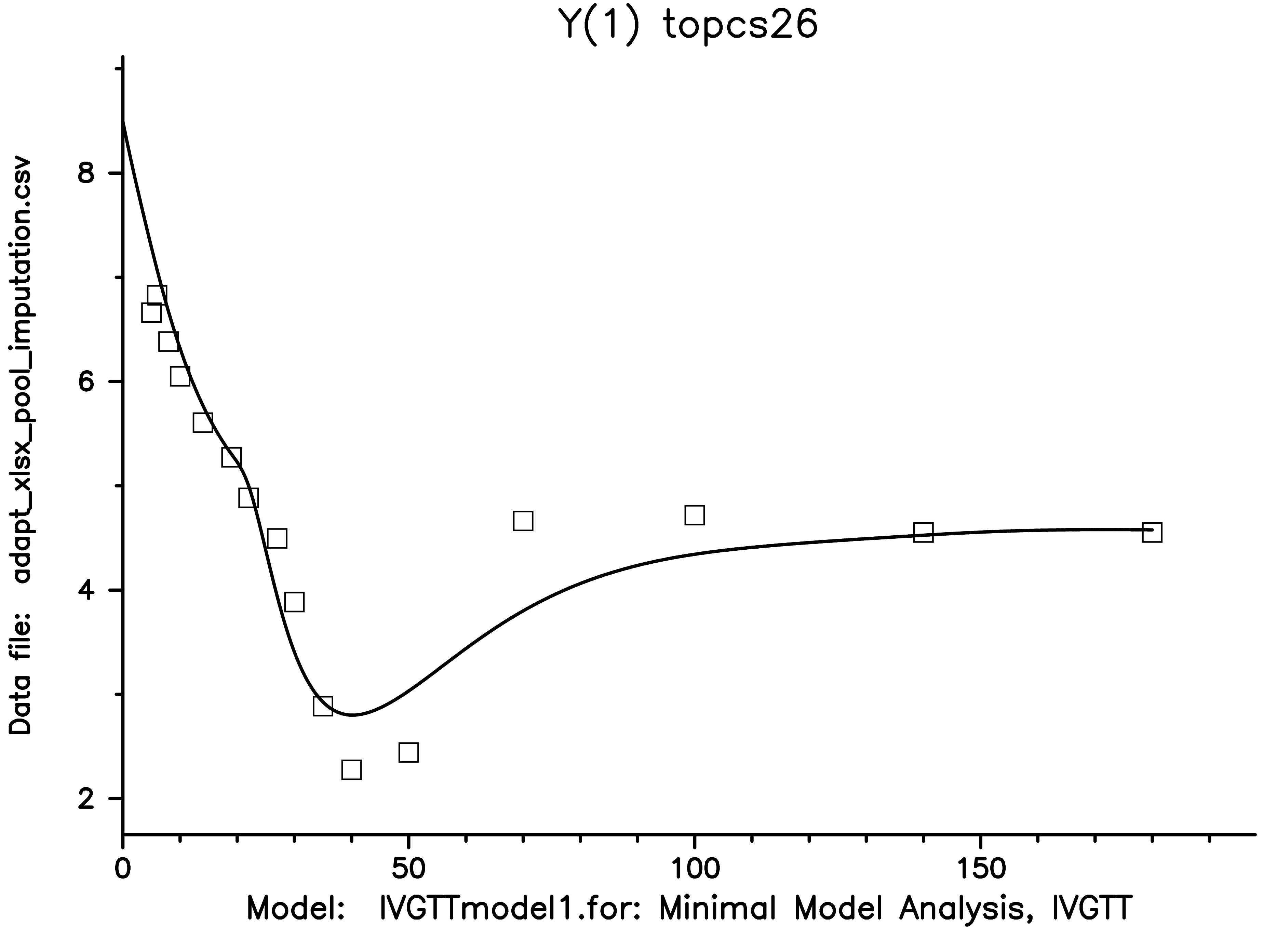
Y(1) topcs26
Data file: adapt_xlsx_pool_imputation.csv
8
6
4
2
0
50
100
150
Model: IVGTTmodel1.for: Minimal Model Analysis, IVGTT

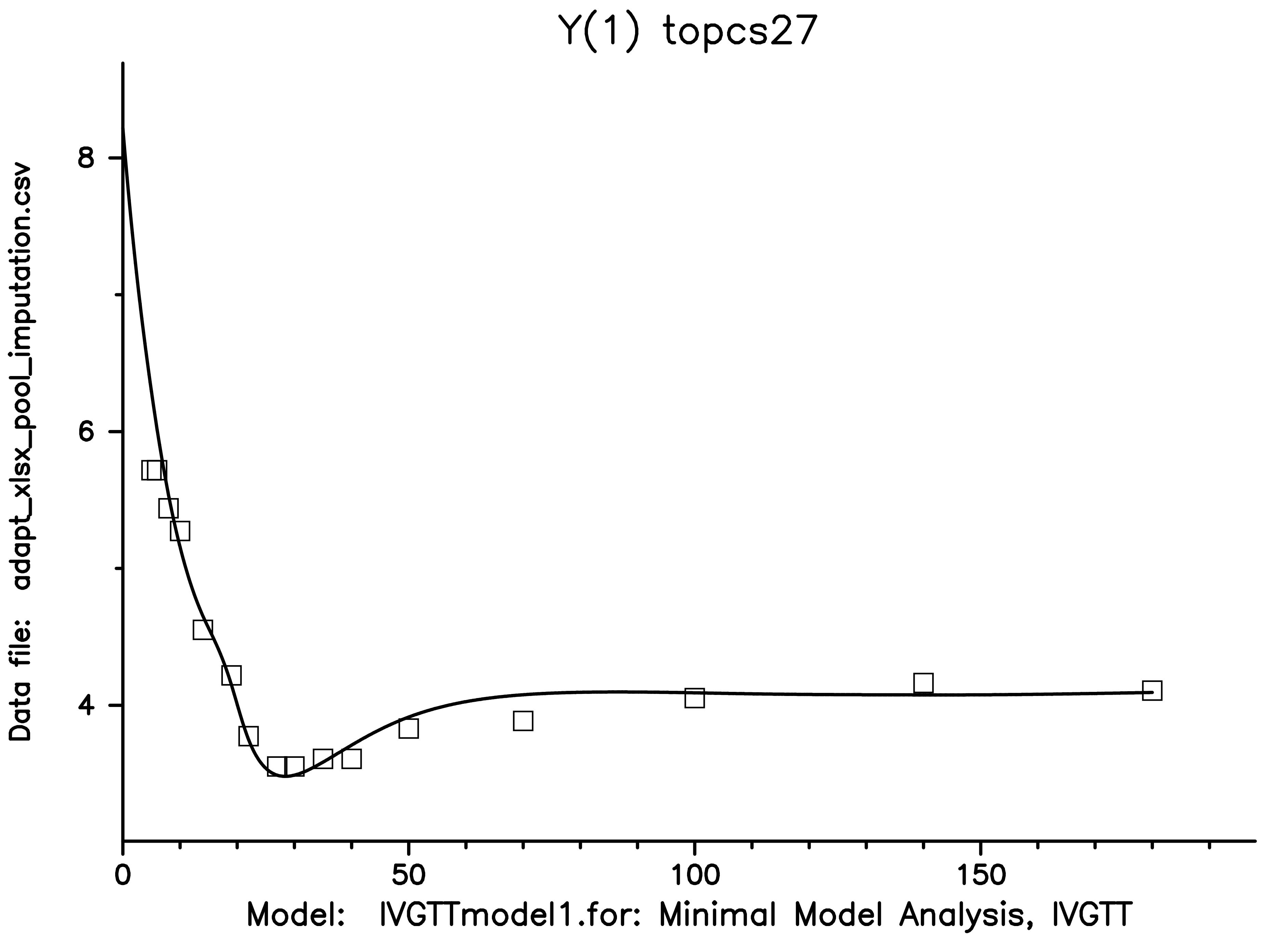
Y(1) topcs27
Data file: adapt_xlsx_pool_imputation.csv
8
6
4
0
50
100
150
Model: IVGTTmodel1.for: Minimal Model Analysis, IVGTT

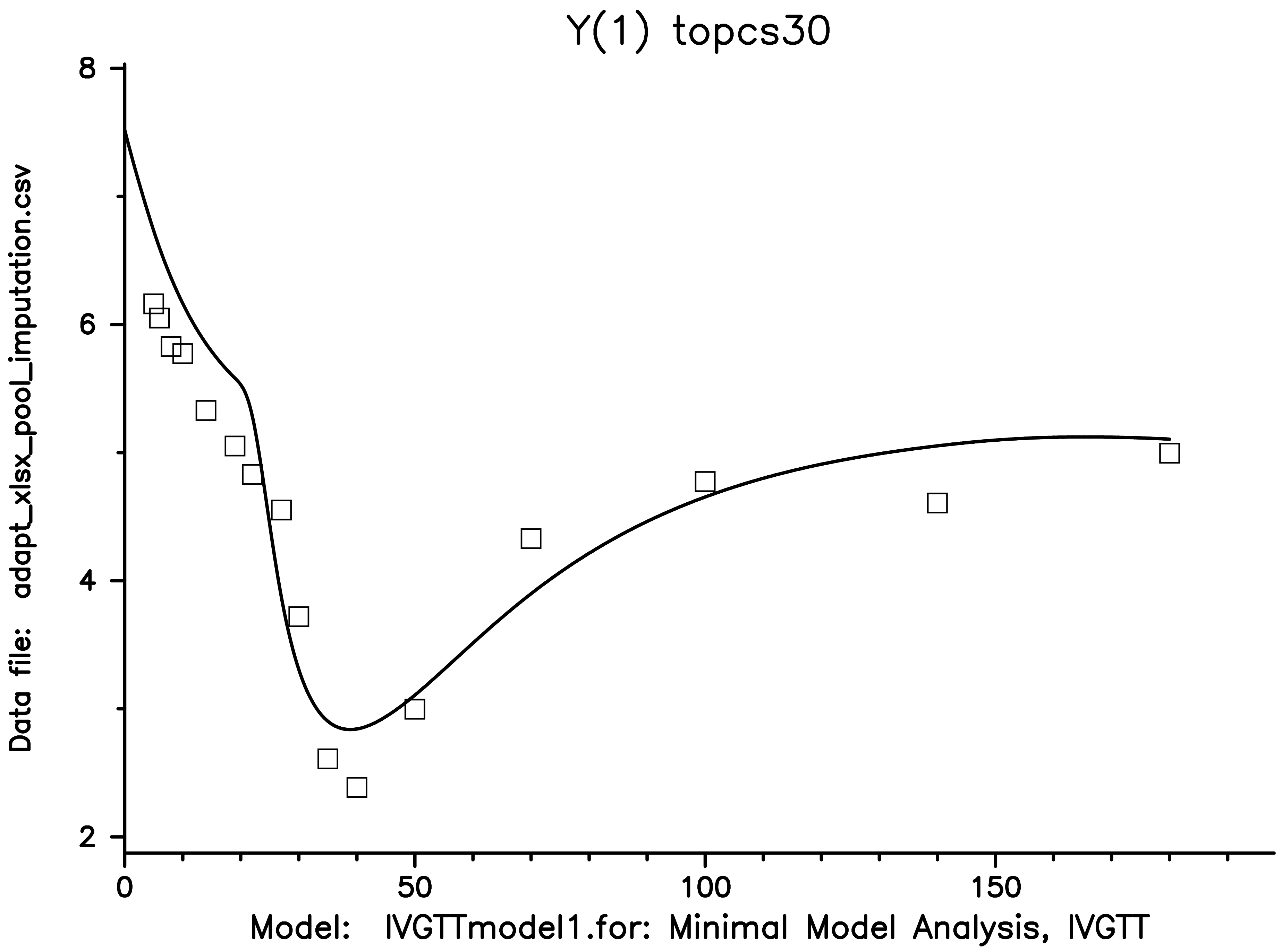
Y(1) topcs30
Data file: adapt_xlsx_pool_imputation.csv
Model: IVGTTmodel1.for: Minimal Model Analysis, IVGTT
8
6
4
2
0
50
100
150

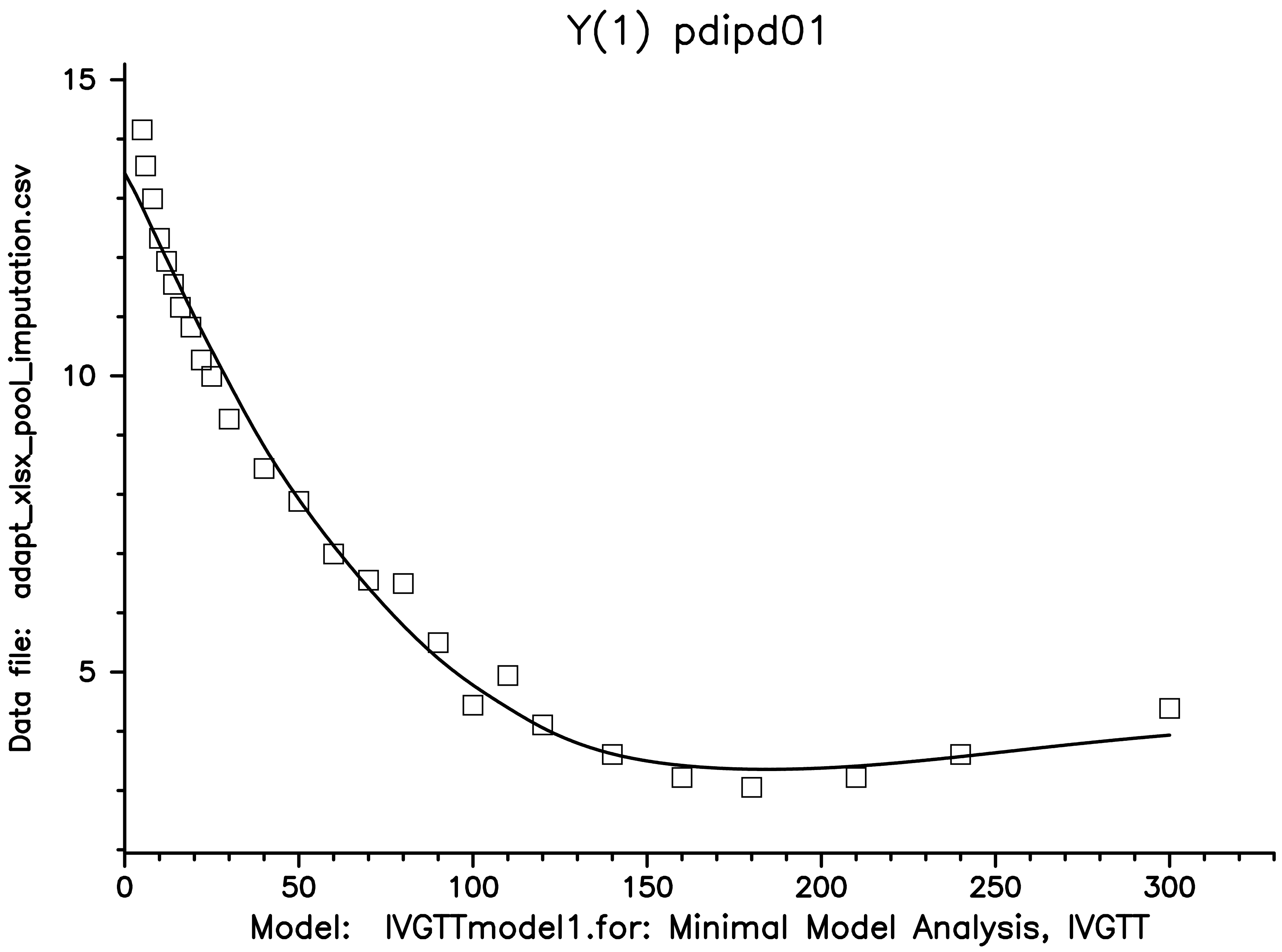
Y(1) pdipd01
Data file: adapt_xlsx_pool_imputation.csv
15
10
5
0
50
100
150
200
250
300
Model: IVGTTmodel1.for: Minimal Model Analysis, IVGTT

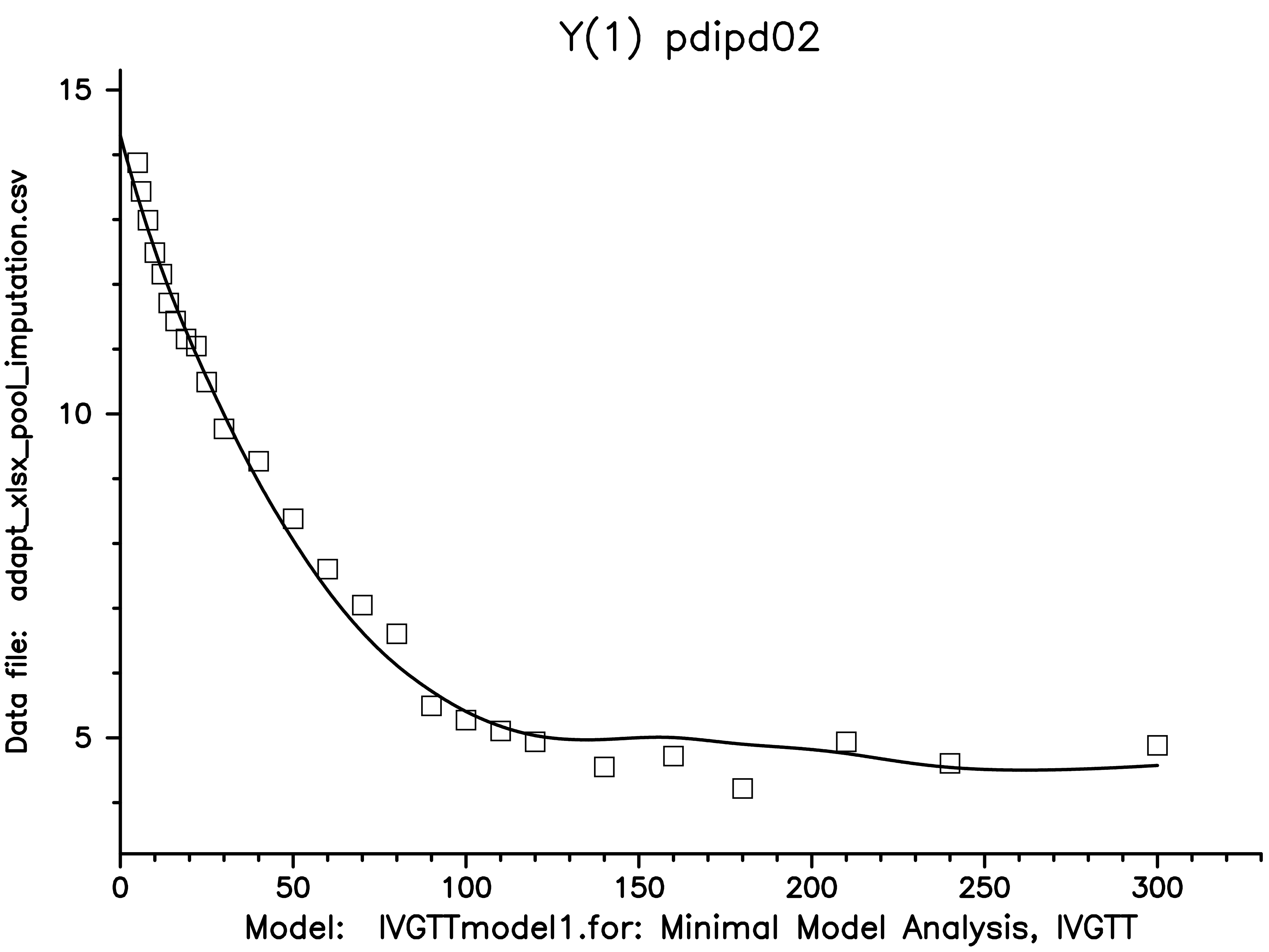

Y(1) pdipd02
Data file: adapt_xlsx_pool_imputation.csv
Model: IVGTTmodel1.for: Minimal Model Analysis, IVGTT
15
10
5
0
50
100
150
200
250
300

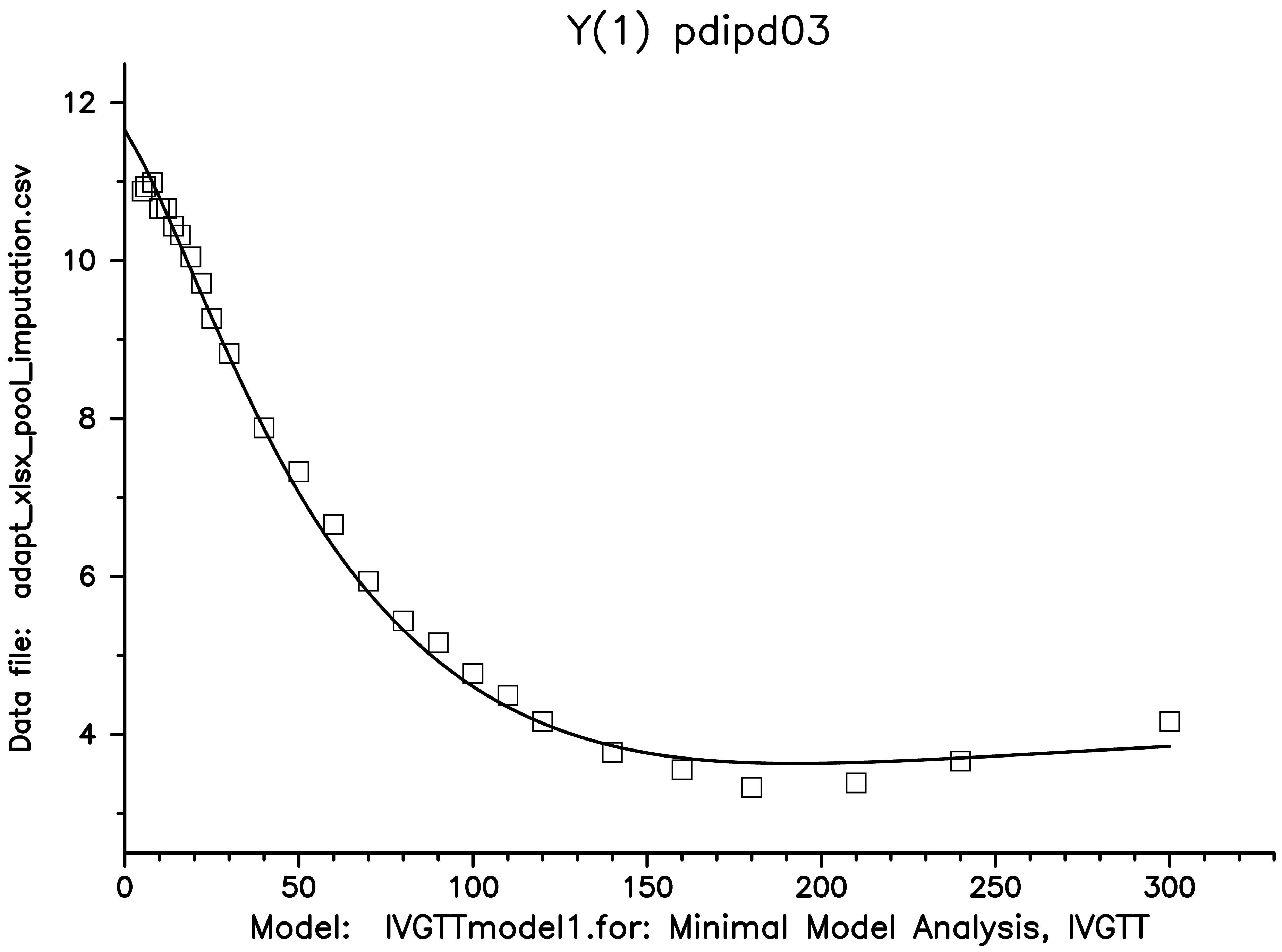
Y(1) pdipd03
Data file: adapt_xlsx_pool_imputation.csv
12
10
8
6
4
0
50
100
150
200
250
300
Model: IVGTTmodel1.for: Minimal Model Analysis, IVGTT

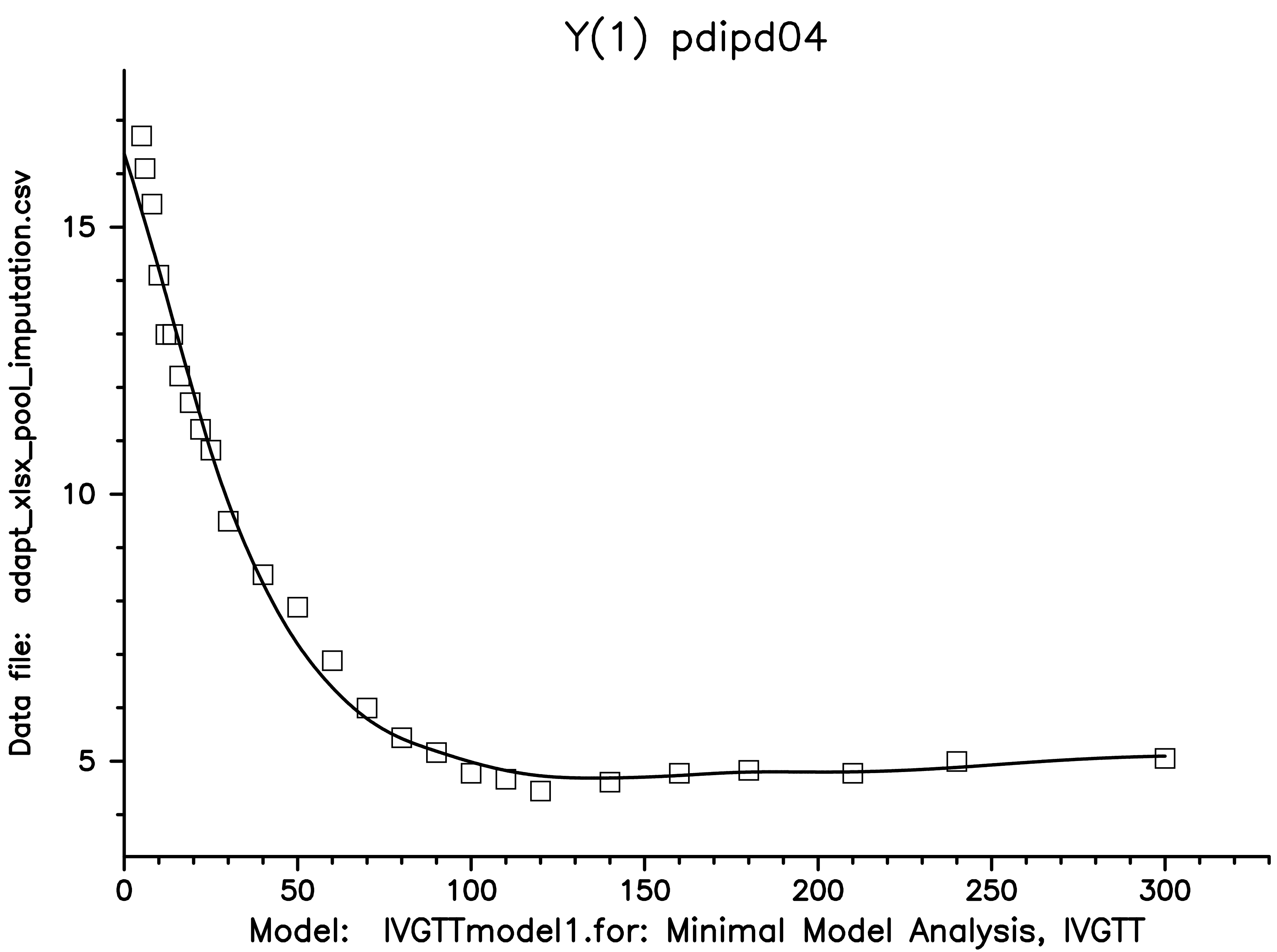

Y(1) pdipd04
Data file: adapt_xlsx_pool_imputation.csv
15
10
5
0
50
100
150
200
250
300
Model: IVGTTmodel1.for: Minimal Model Analysis, IVGTT

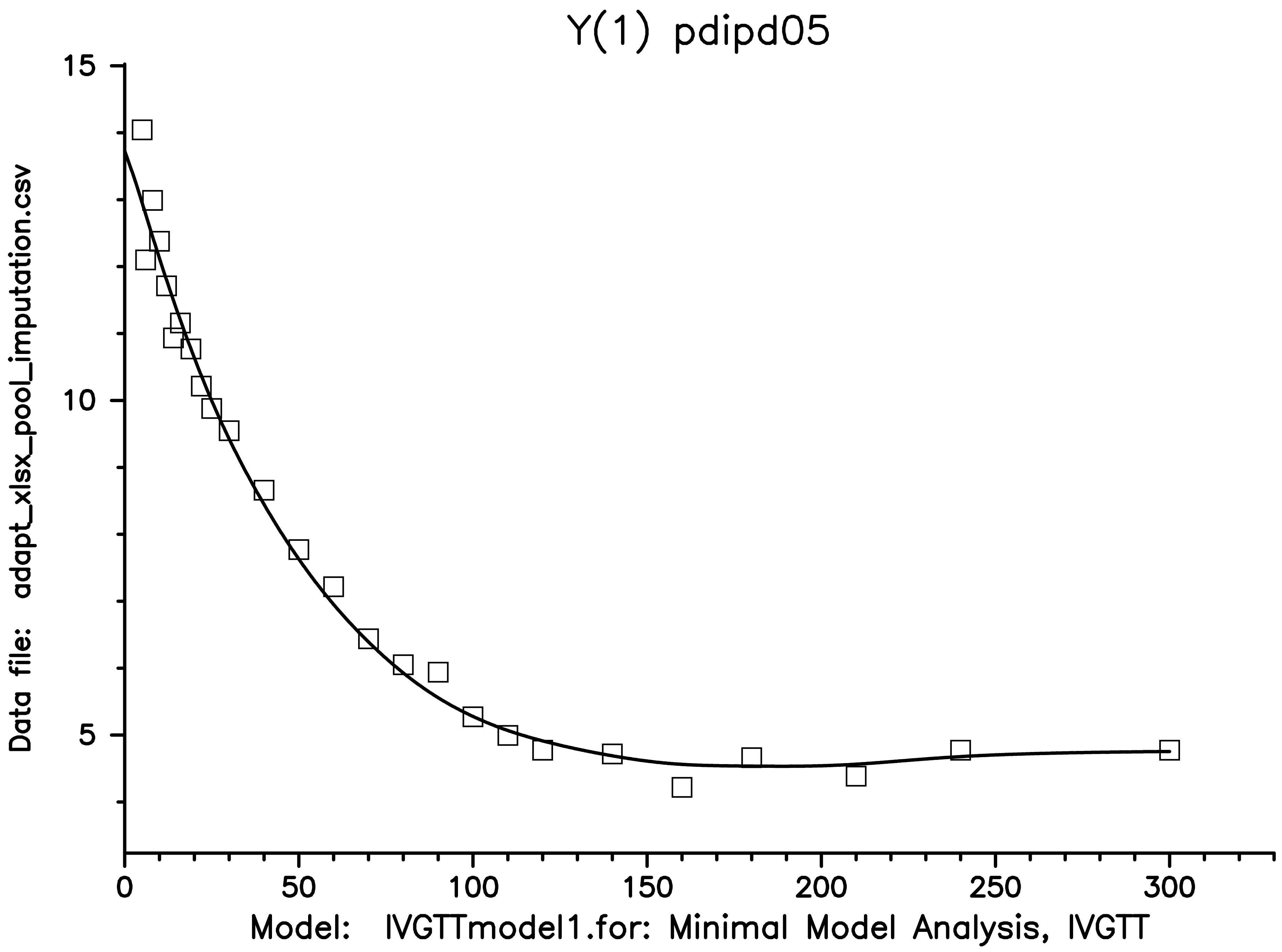
Y(1) pdipd05
15
10
5
0
50
100
150
200
250
300
Data file: adapt_xlsx_pool_imputation.csv
Model: IVGTTmodel1.for: Minimal Model Analysis, IVGTT

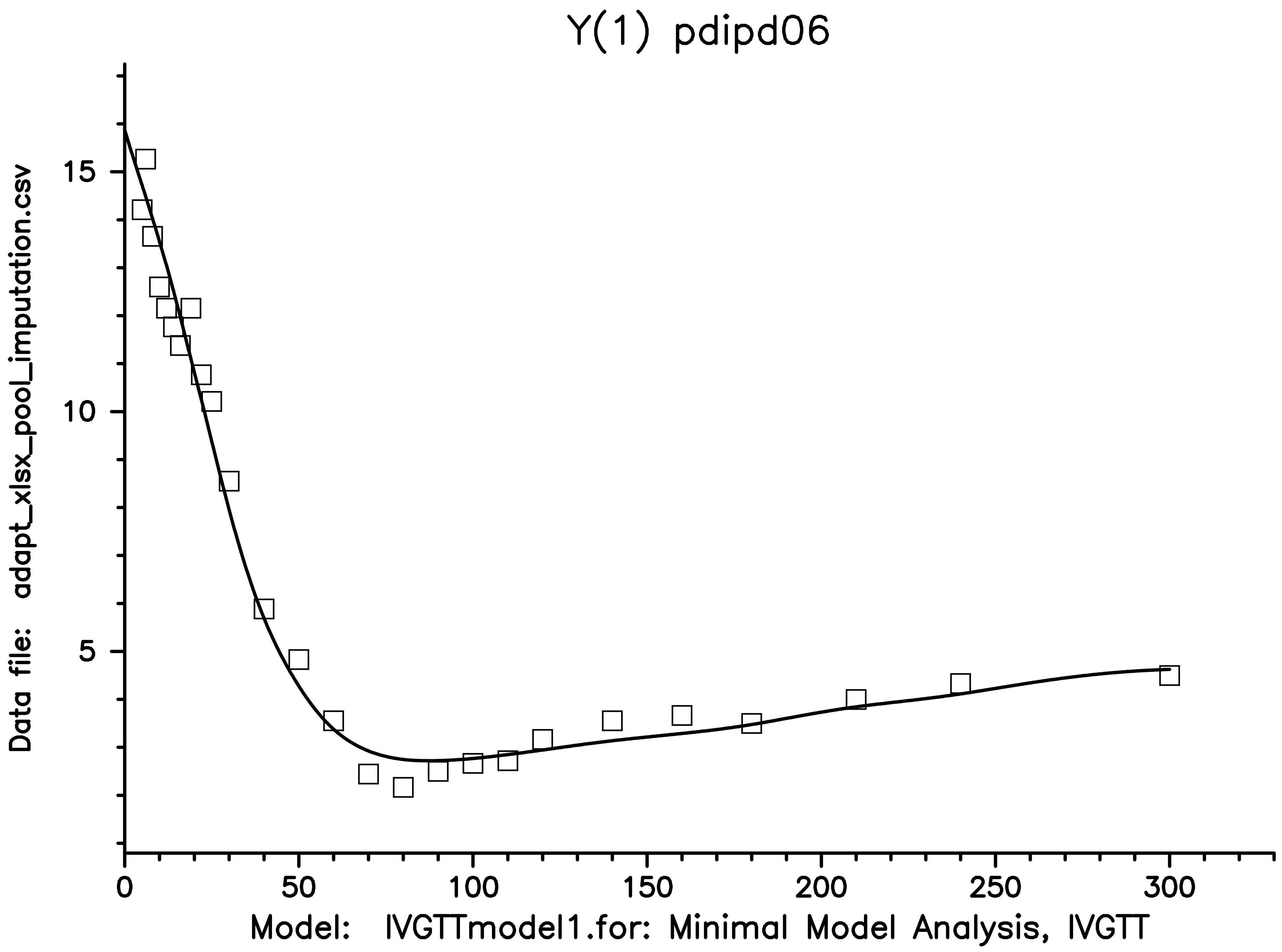

Y(1) pdipd06
15
10
5
0
50
100
150
200
250
300
Data file: adapt_xlsx_pool_imputation.csv
Model: IVGTTmodel1.for: Minimal Model Analysis, IVGTT

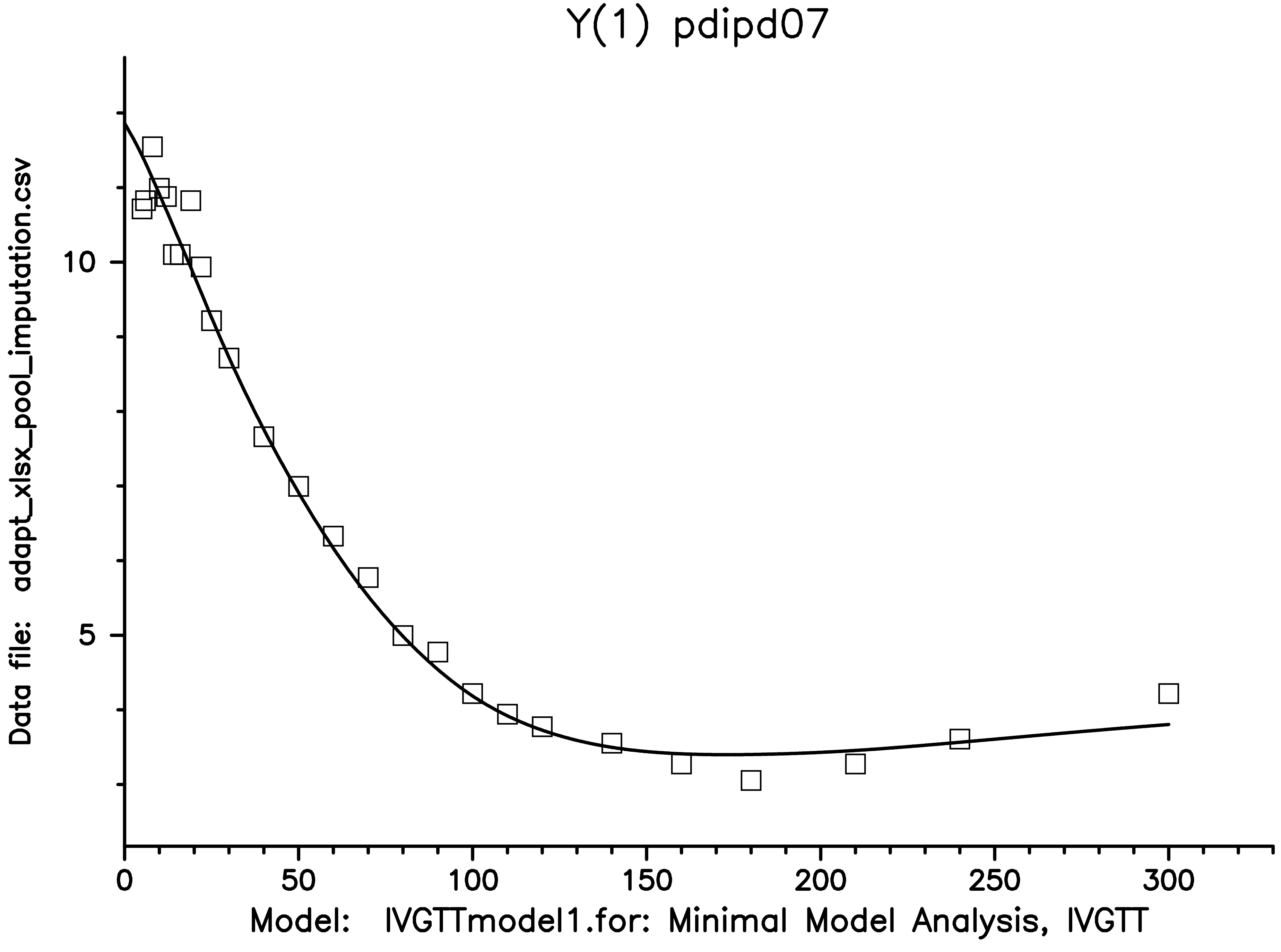
Y(1) pdipd07
Data file: adapt_xlsx_pool_imputation.csv
10
5
0
50
100
150
200
250
300
Model: IVGTTmodel1.for: Minimal Model Analysis, IVGTT

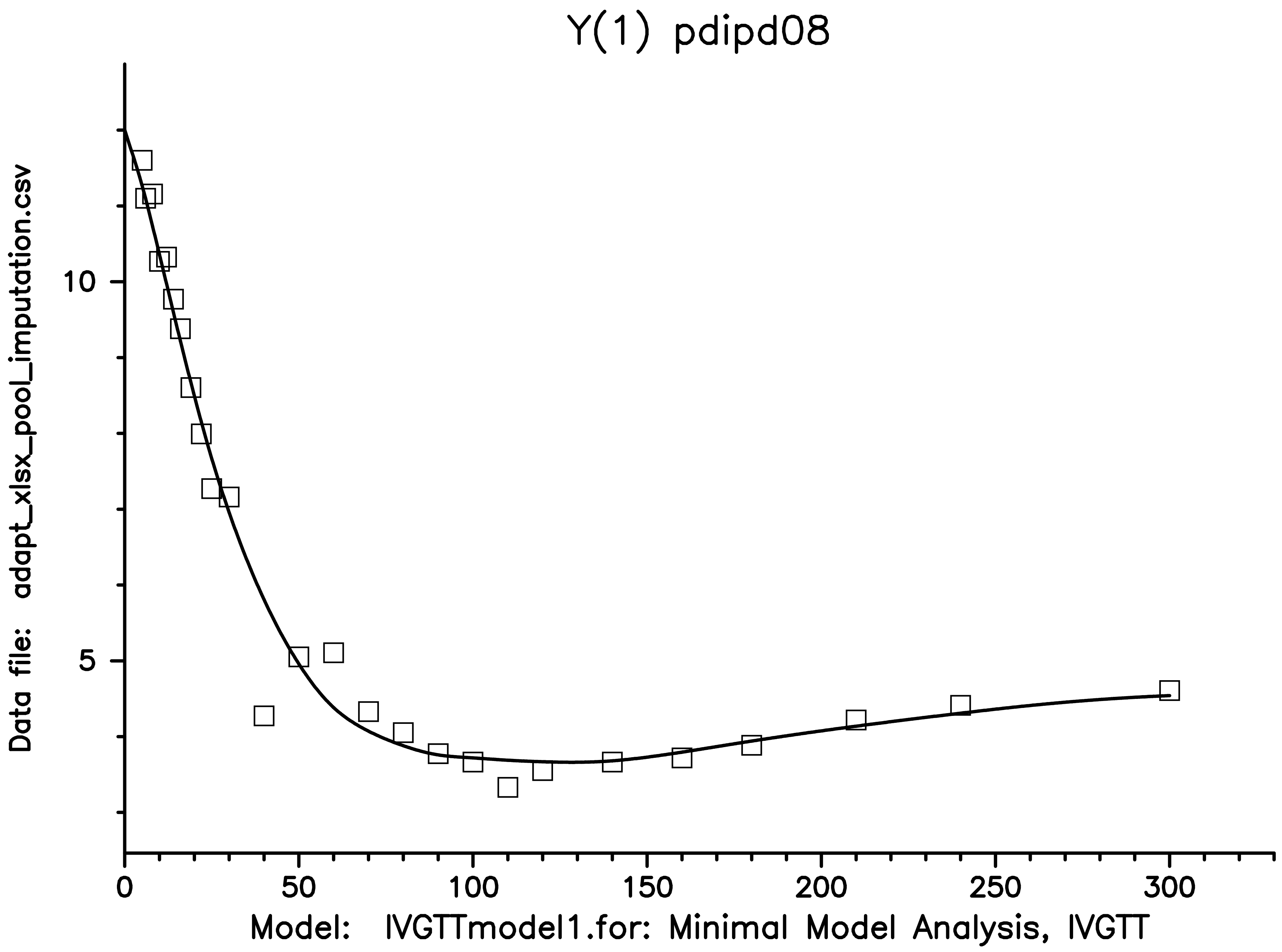

Y(1) pdipd08
Data file: adapt_xlsx_pool_imputation.csv
10
5
0
50
100
150
200
250
300
Model: IVGTTmodel1.for: Minimal Model Analysis, IVGTT

Y(1) pdipd09

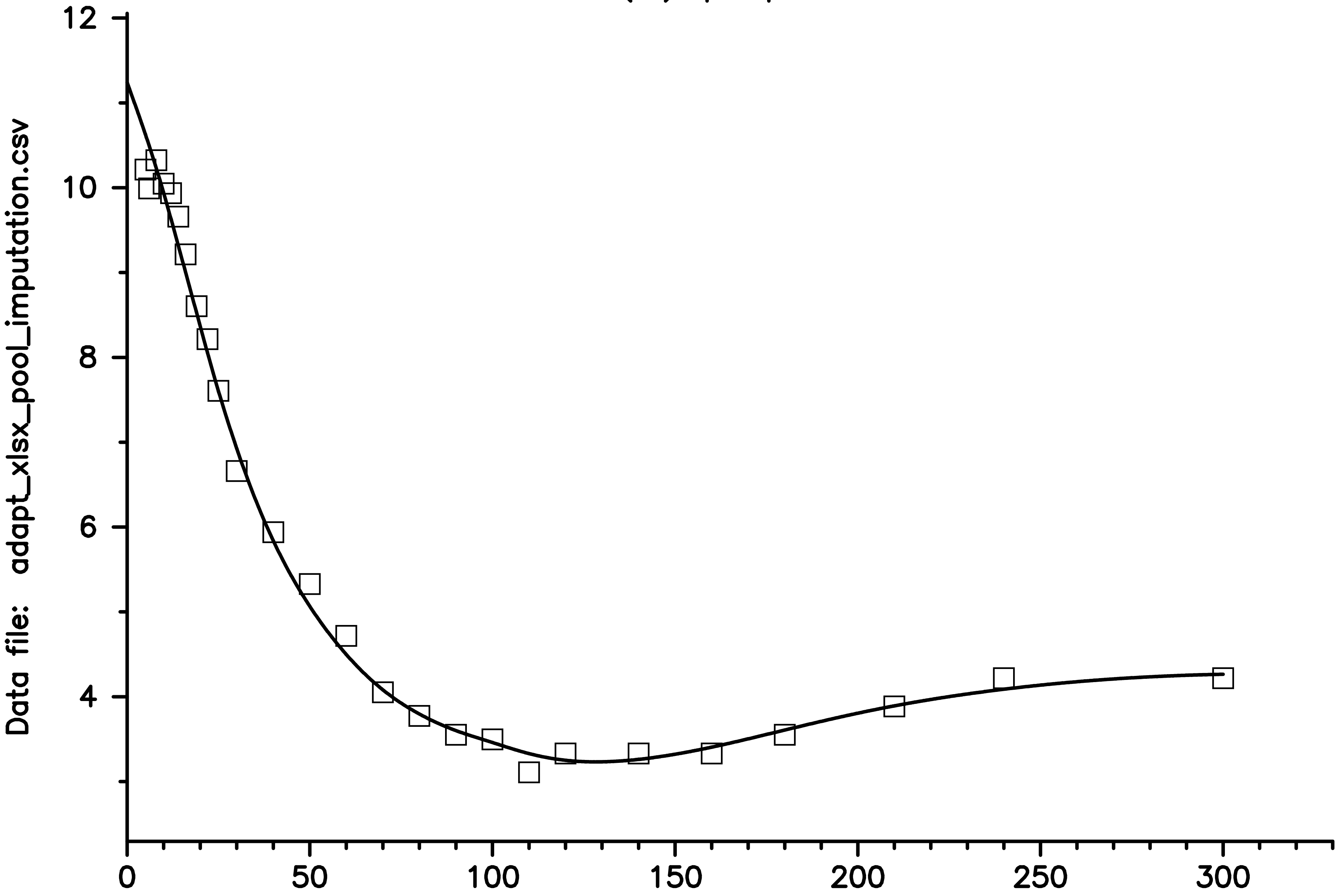

Model: IVGTTmodel1.for: Minimal Model Analysis, IVGTT

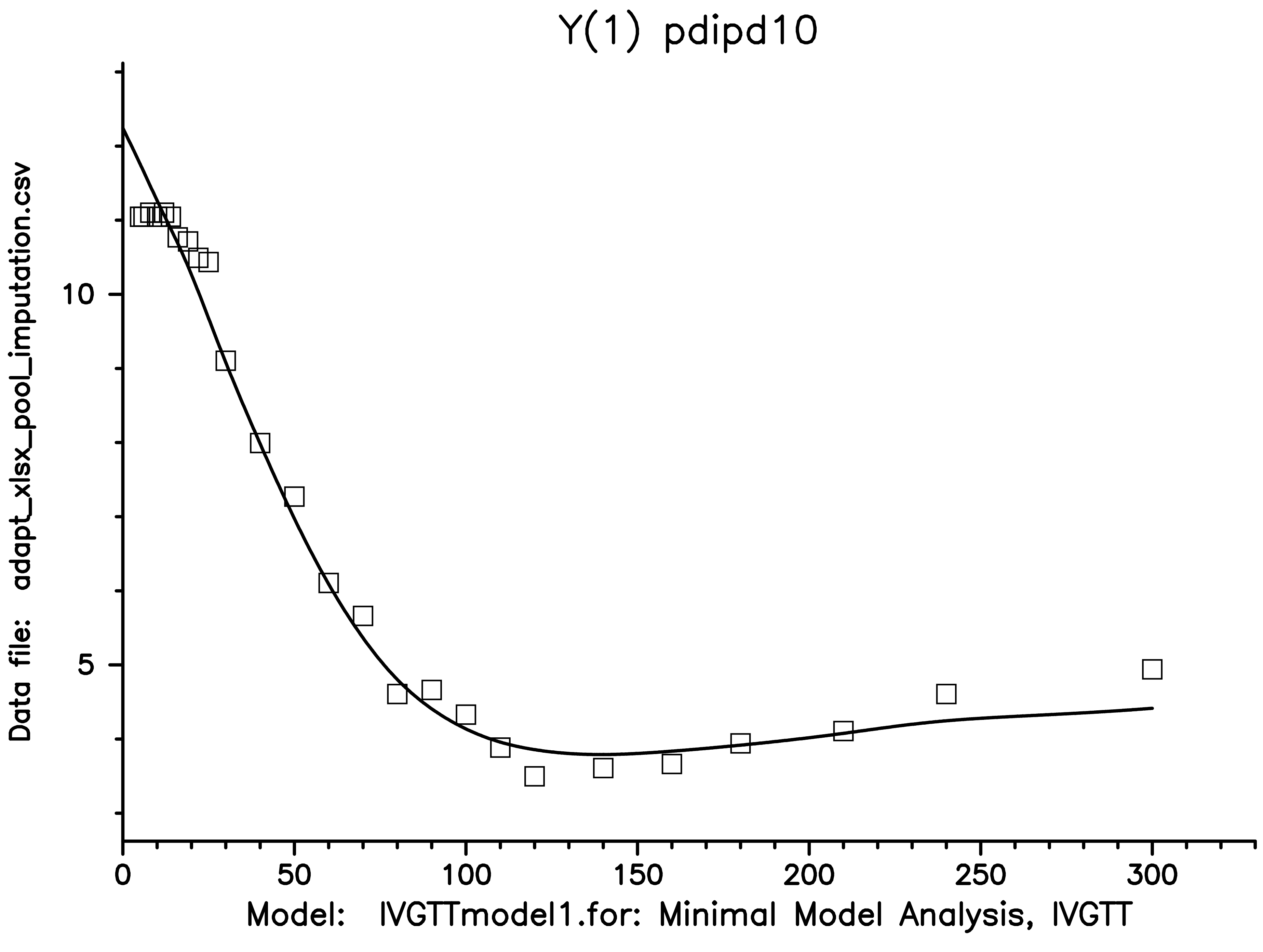
Y(1) pdipd10
Data file: adapt_xlsx_pool_imputation.csv
10
5
0
50
100
150
200
250
300
Model: IVGTTmodel1.for: Minimal Model Analysis, IVGTT

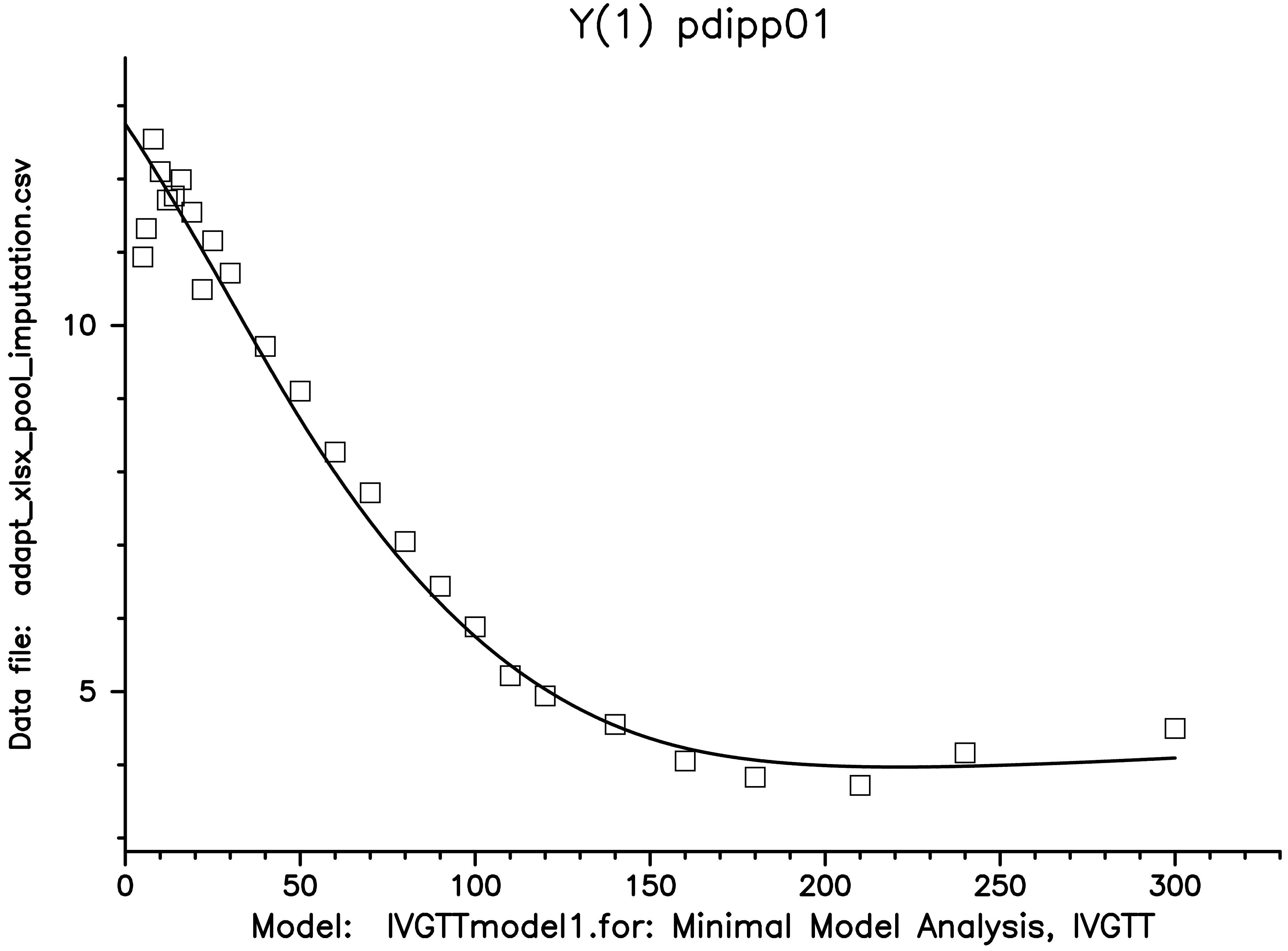
Y(1) pdipp01
Data file: adapt_xlsx_pool_imputation.csv
10
5
0
50
100
150
200
250
300
Model: IVGTTmodel1.for: Minimal Model Analysis, IVGTT

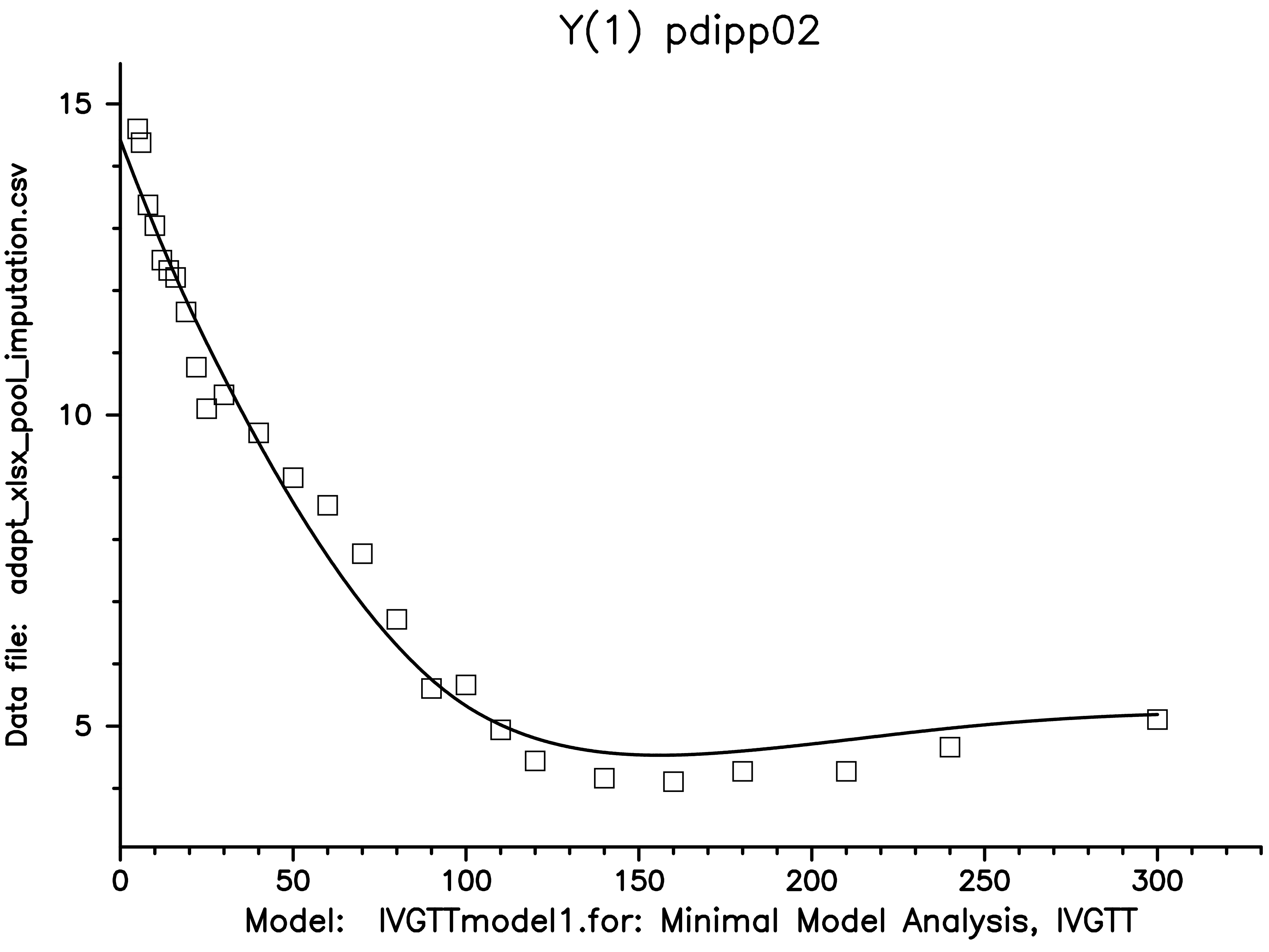
Y(1) pdipp02
Data file: adapt_xlsx_pool_imputation.csv
15
10
5
0
50
100
150
200
250
300
Model: IVGTTmodel1.for: Minimal Model Analysis, IVGTT

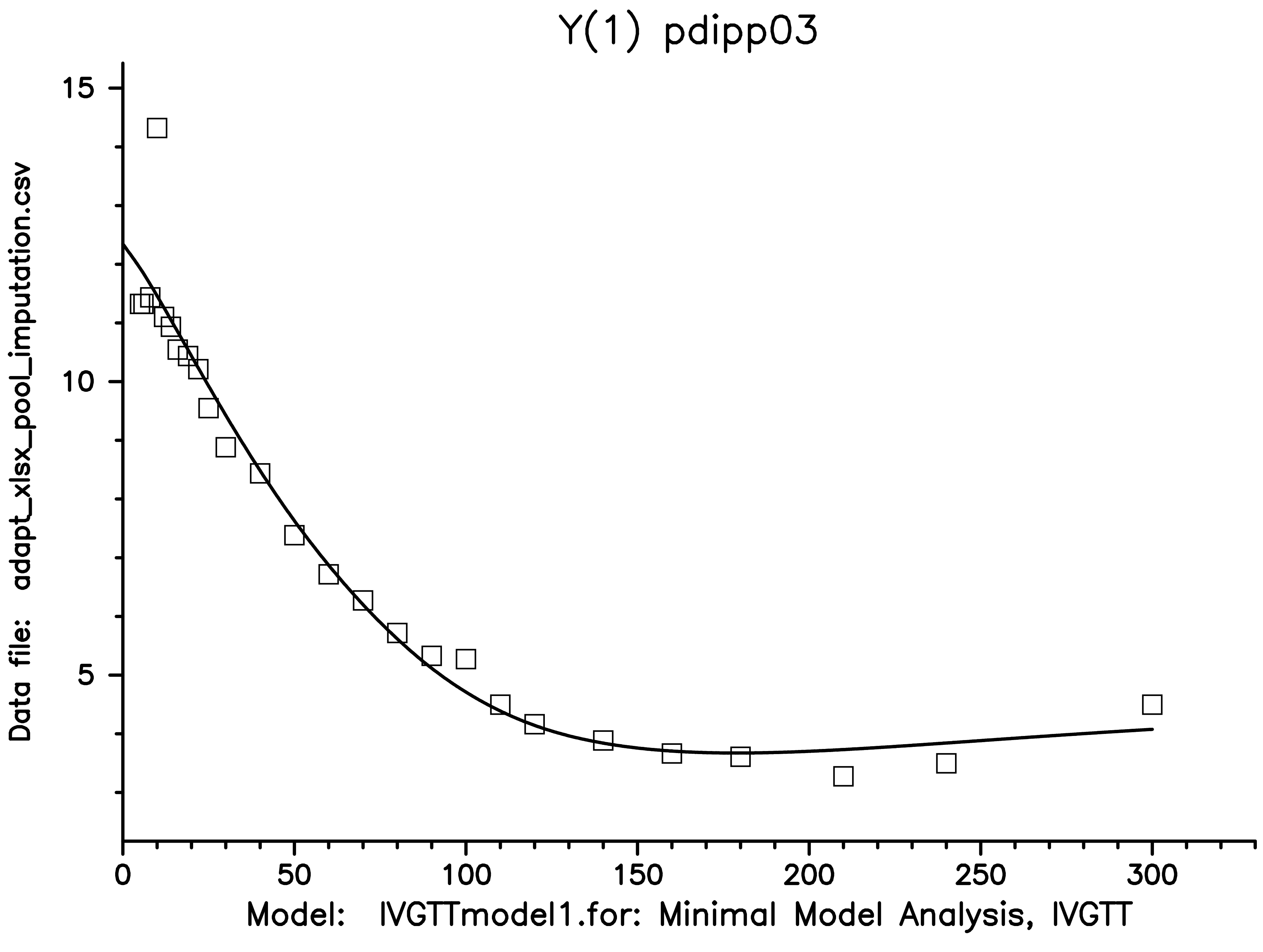
Y(1) pdipp03
Data file: adapt_xlsx_pool_imputation.csv
Model: IVGTTmodel1.for: Minimal Model Analysis, IVGTT
15
10
5
0
50
100
150
200
250
300

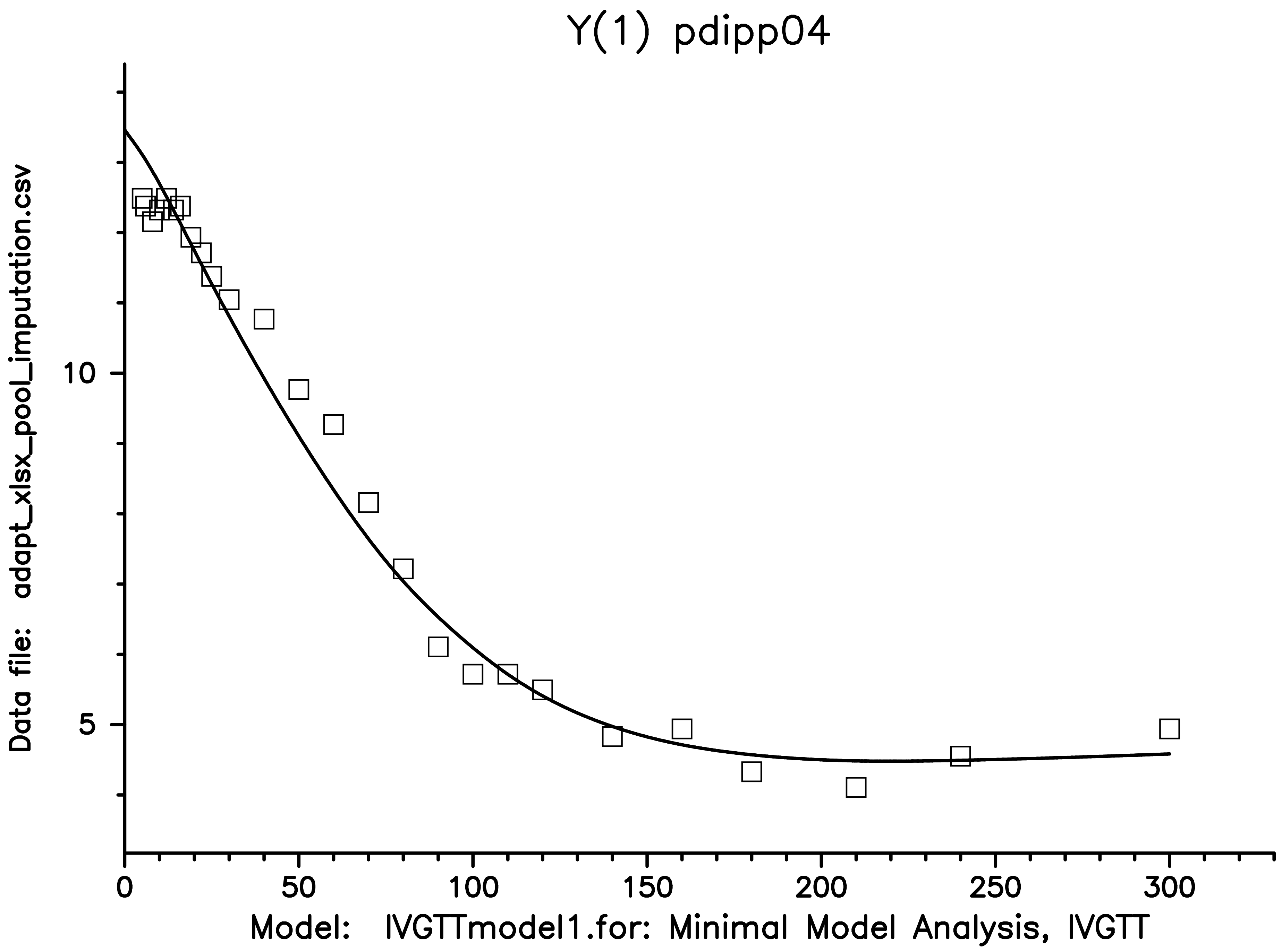
Y(1) pdipp04
Data file: adapt_xlsx_pool_imputation.csv
Model: IVGTTmodel1.for: Minimal Model Analysis, IVGTT
10
5
0
50
100
150
200
250
300

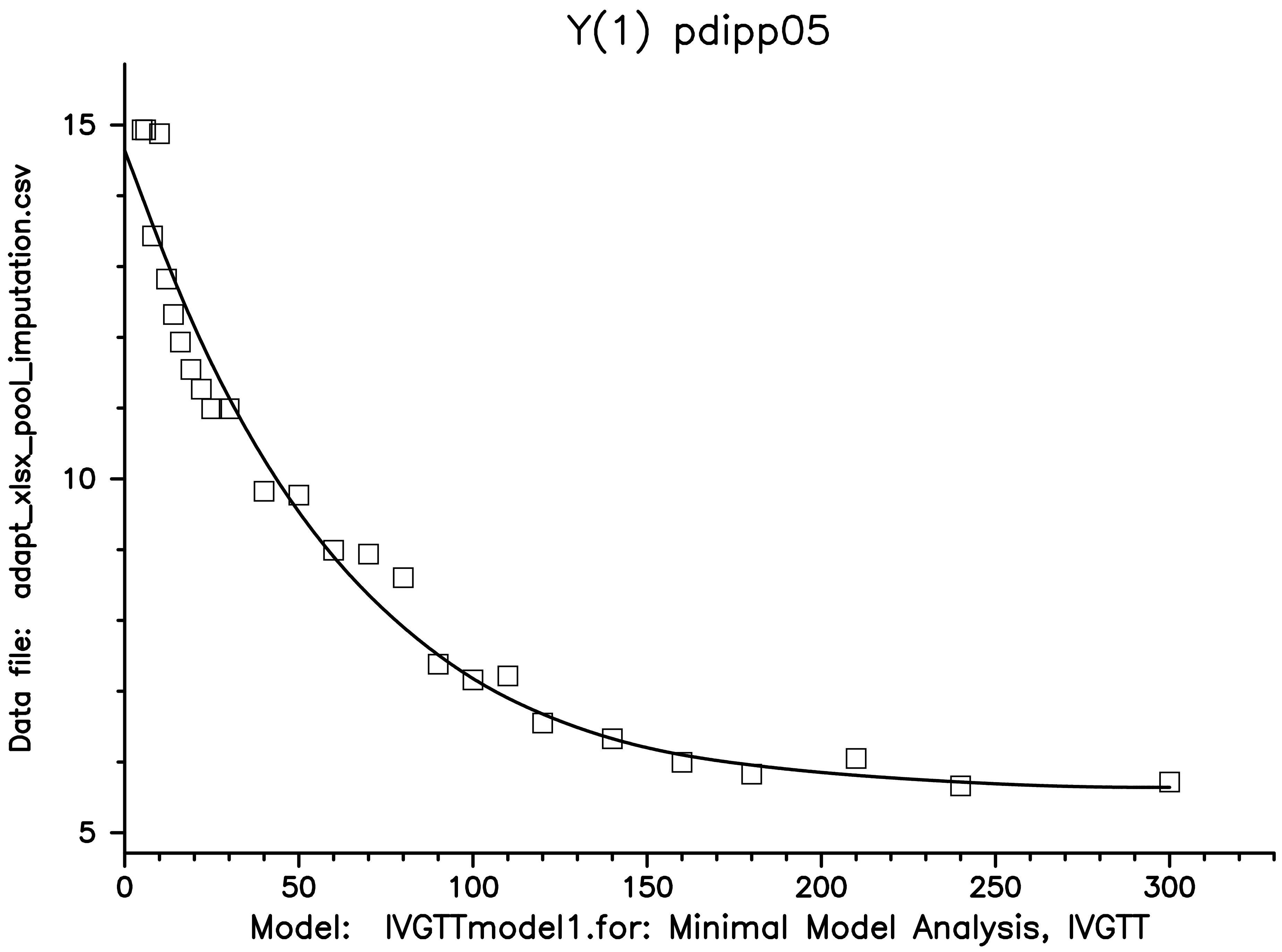
Y(1) pdipp05
Data file: adapt_xlsx_pool_imputation.csv
15
10
5
0
50
100
150
200
250
300
Model: IVGTTmodel1.for: Minimal Model Analysis, IVGTT

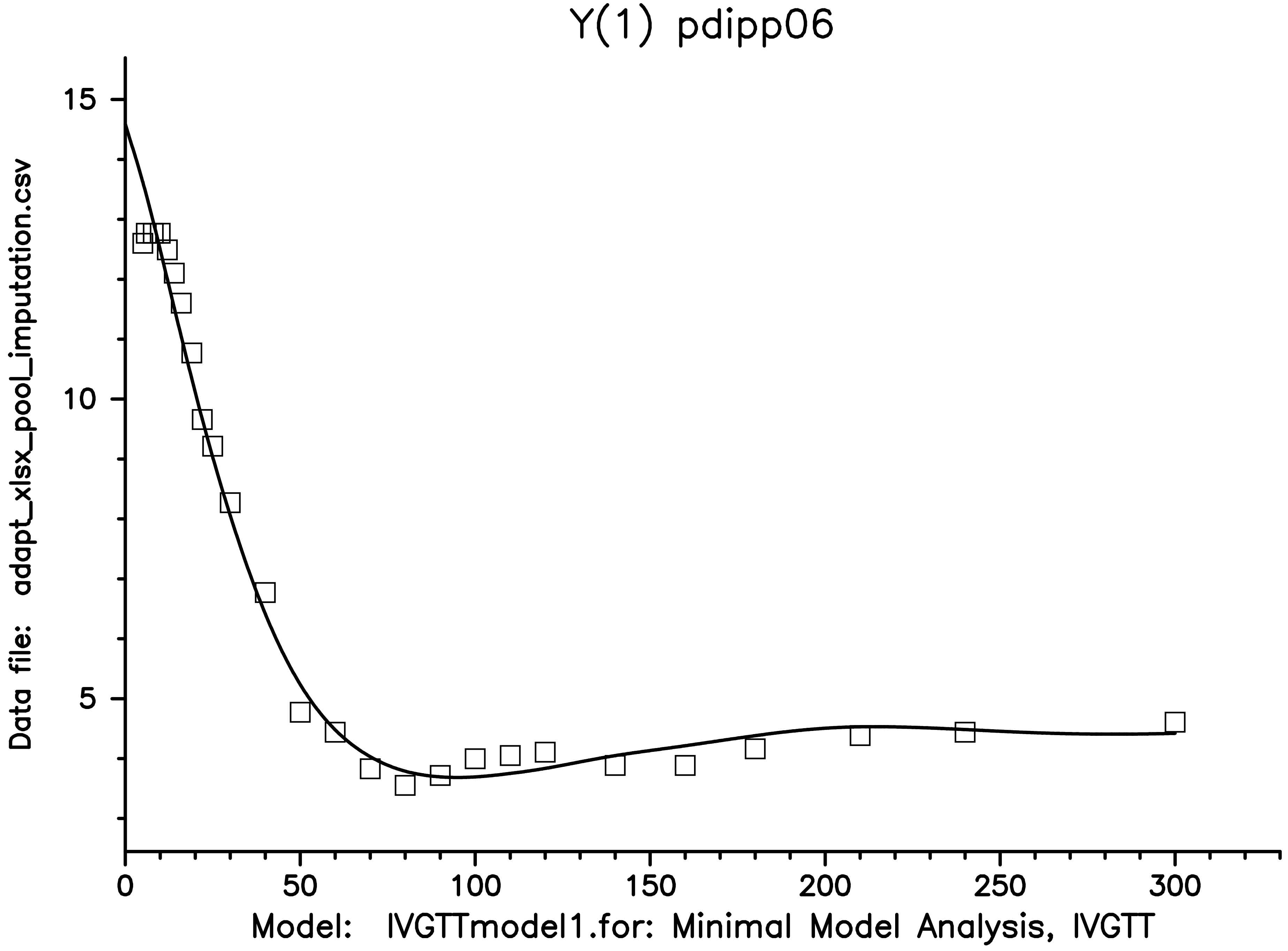
Y(1) pdipp06
Data file: adapt_xlsx_pool_imputation.csv
15
10
5
0
50
100
150
200
250
300
Model: IVGTTmodel1.for: Minimal Model Analysis, IVGTT

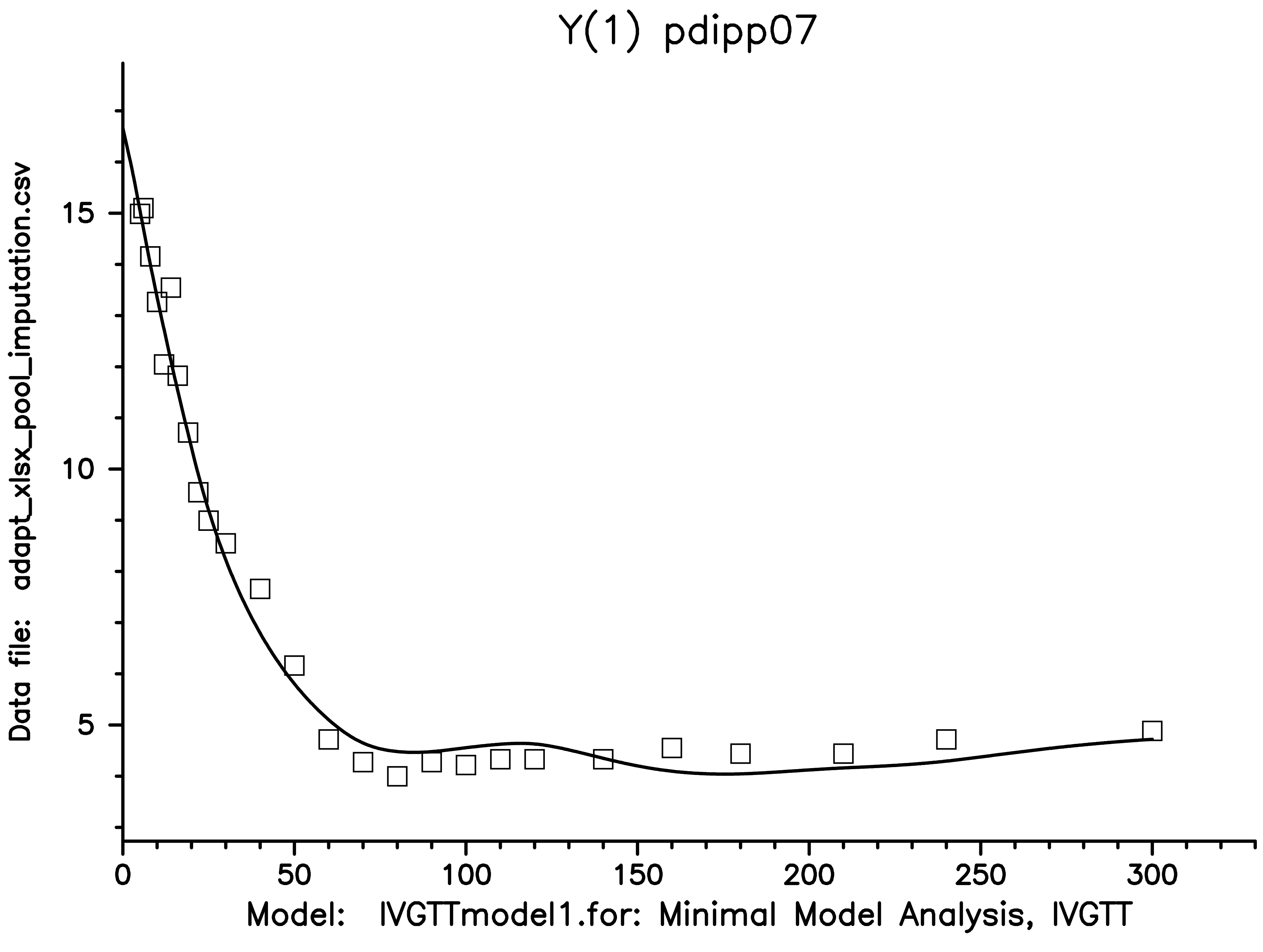
Y(1) pdipp07
Data file: adapt_xlsx_pool_imputation.csv
15
10
5
0
50
100
150
200
250
300
Model: IVGTTmodel1.for: Minimal Model Analysis, IVGTT

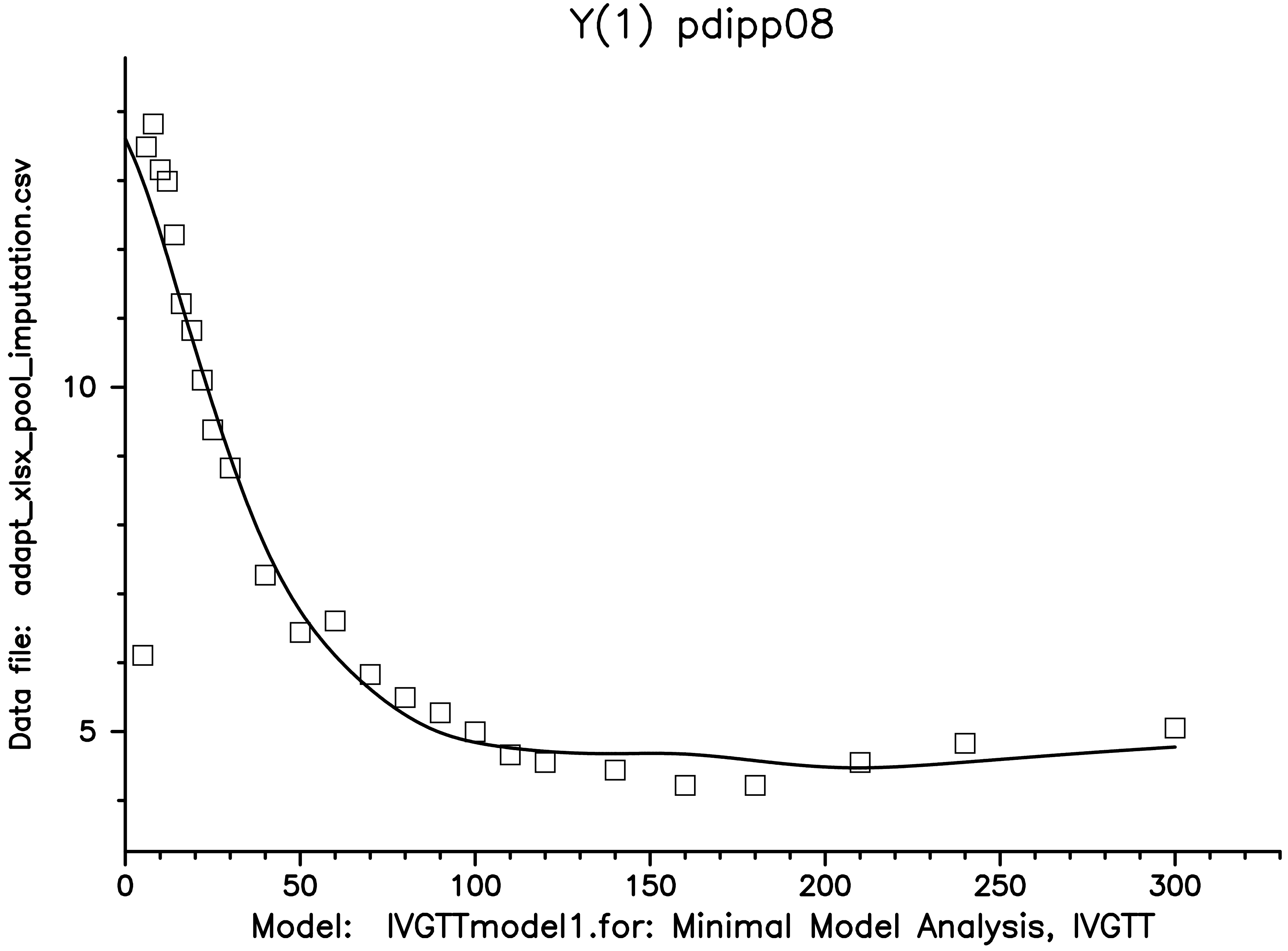
Y(1) pdipp08
Data file: adapt_xlsx_pool_imputation.csv
10
5
0
50
100
150
200
250
300
Model: IVGTTmodel1.for: Minimal Model Analysis, IVGTT

Y(1) pdipp09

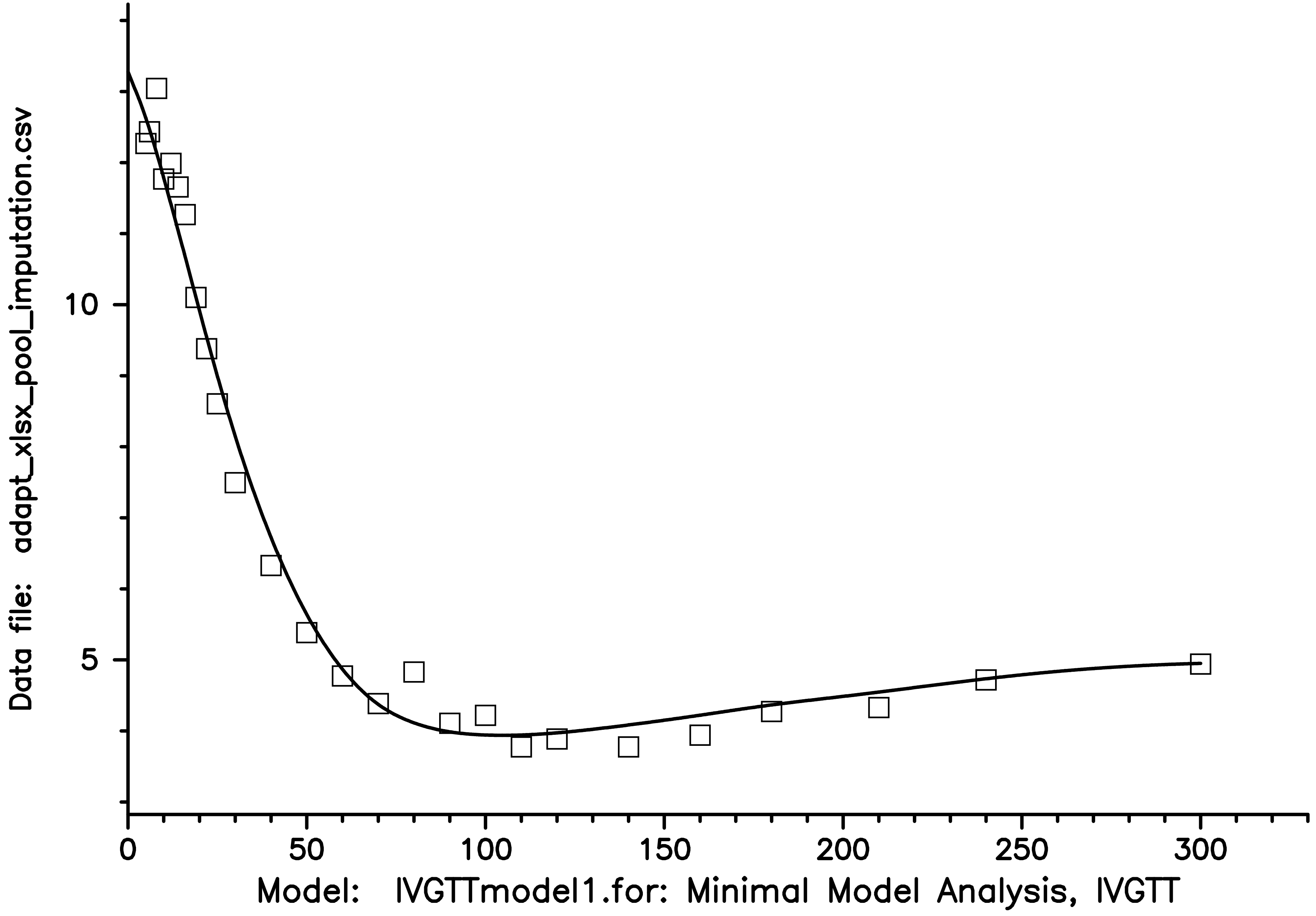

10
5
0
50
100
150
200
250
300
Data file: adapt_xlsx_pool_imputation.csv
Model: IVGTTmodel1.for: Minimal Model Analysis, IVGTT

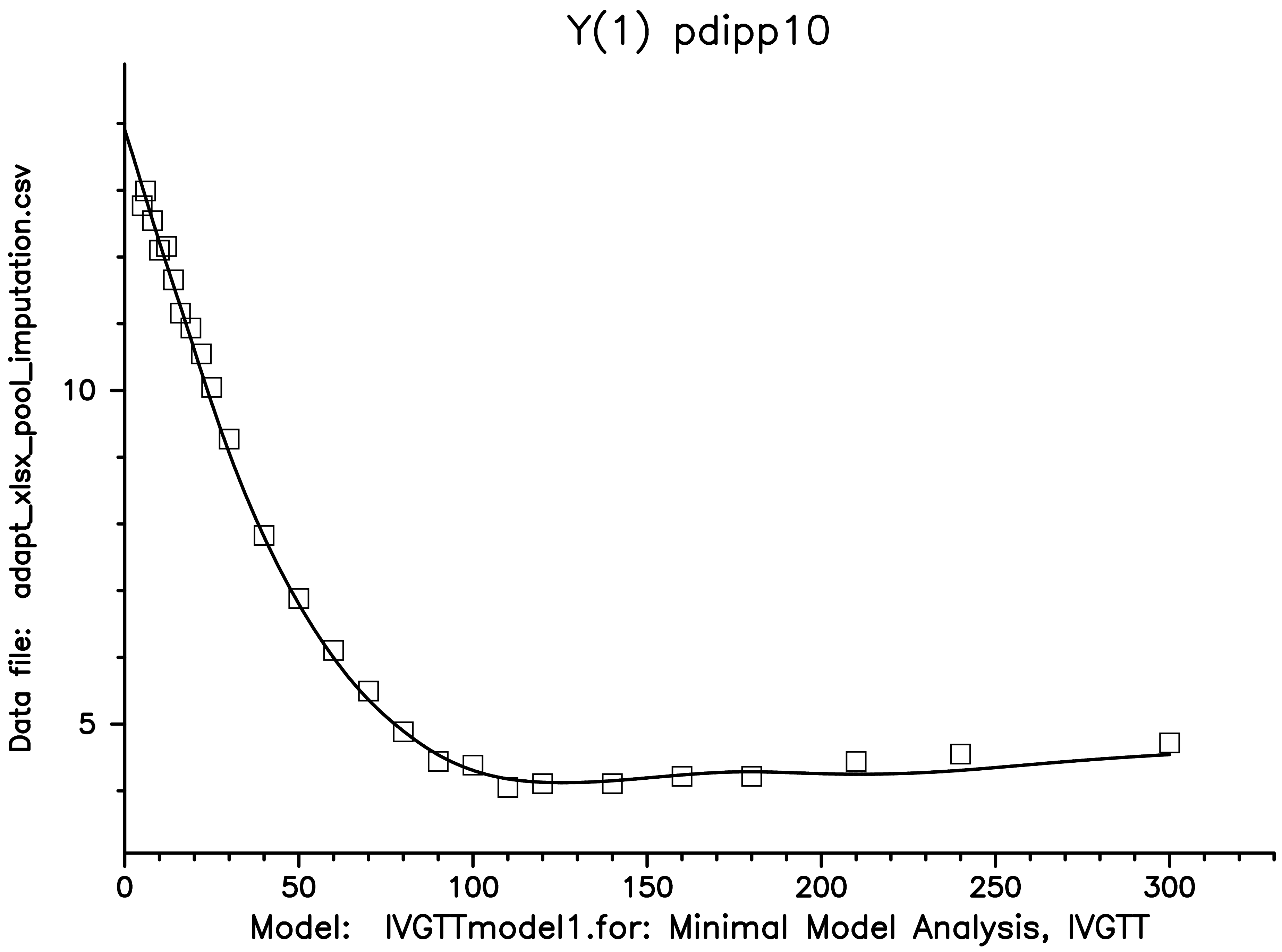

Y(1) pdipp10
Data file: adapt_xlsx_pool_imputation.csv
10
5
0
50
100
150
200
250
300
Model: IVGTTmodel1.for: Minimal Model Analysis, IVGTT

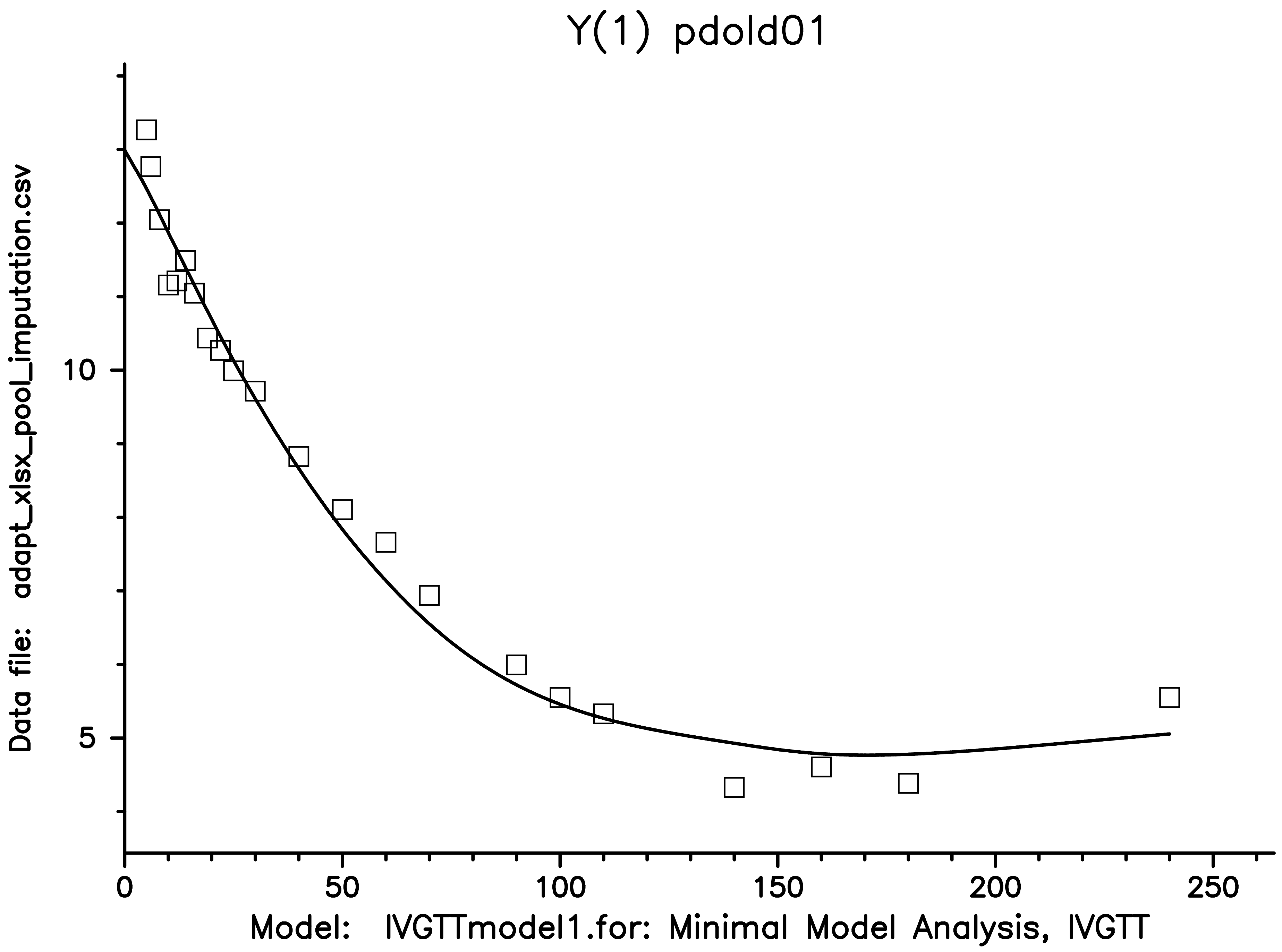
Y(1) pdold01
Data file: adapt_xlsx_pool_imputation.csv
10
5
0
50
100
150
200
250
Model: IVGTTmodel1.for: Minimal Model Analysis, IVGTT

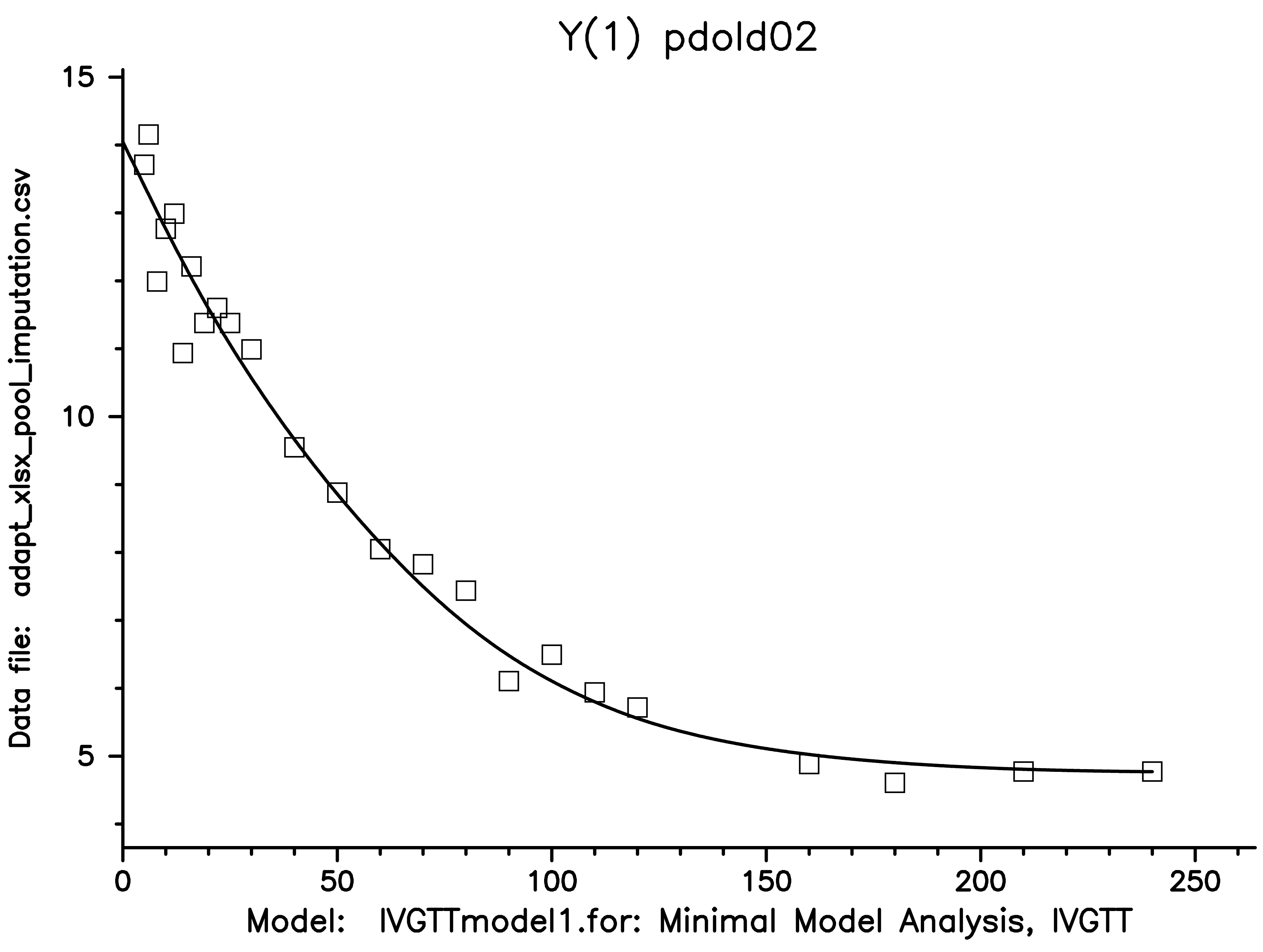
Y(1) pdold02
Data file: adapt_xlsx_pool_imputation.csv
Model: IVGTTmodel1.for: Minimal Model Analysis, IVGTT
15
10
5
0
50
100
150
200
250

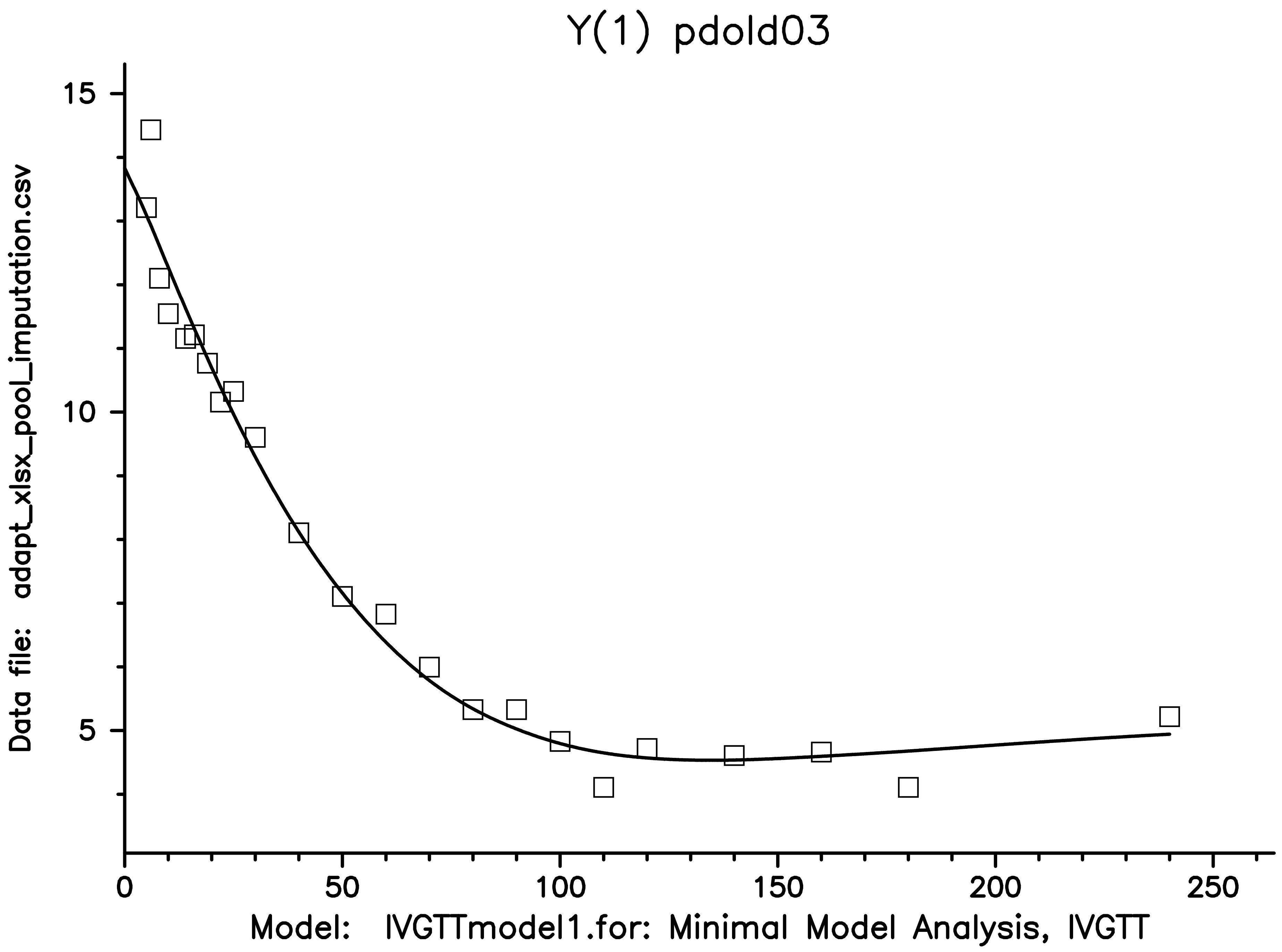
Y(1) pdold03
Data file: adapt_xlsx_pool_imputation.csv
15
10
5
0
50
100
150
200
250
Model: IVGTTmodel1.for: Minimal Model Analysis, IVGTT

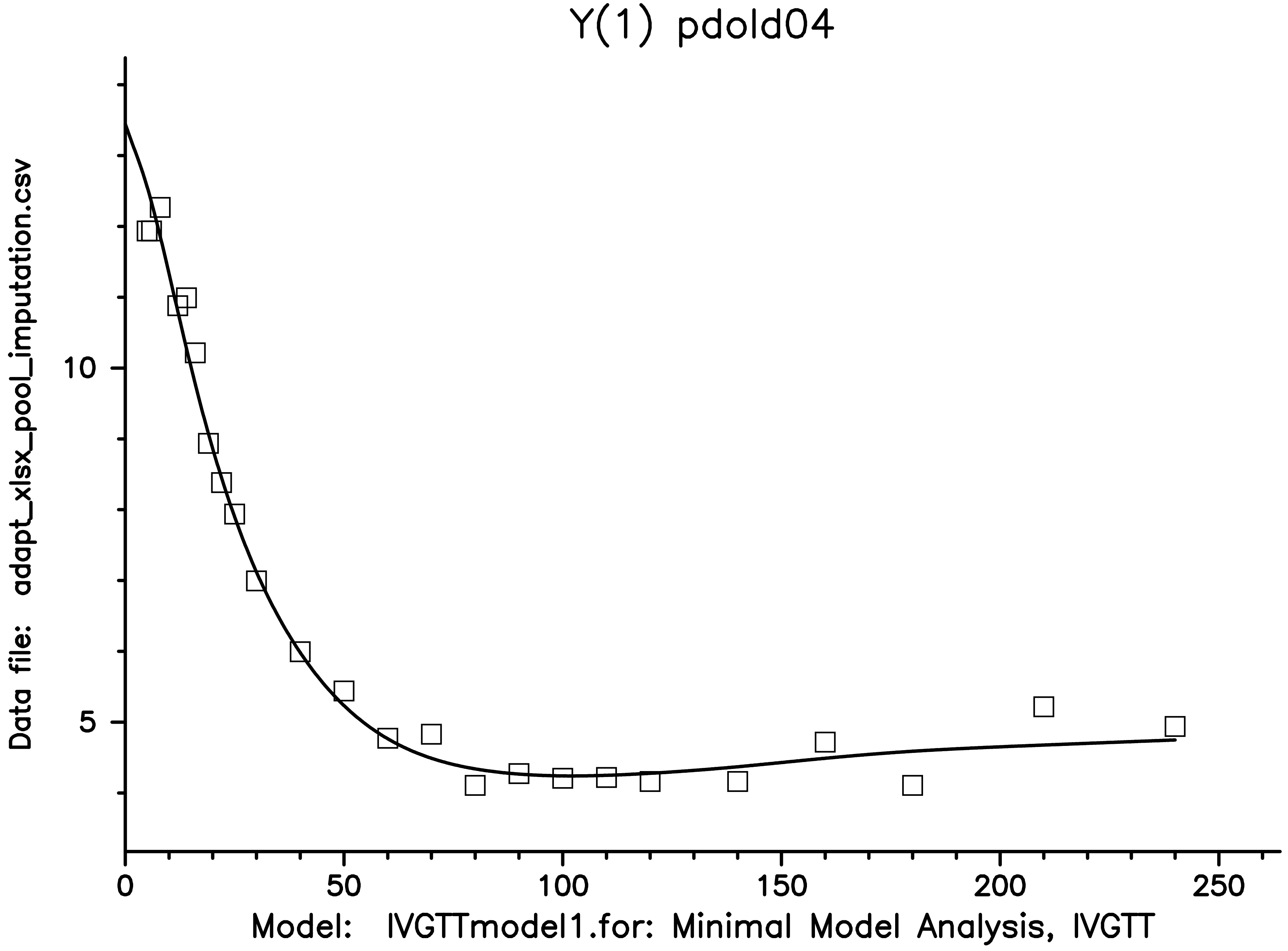

Y(1) pdold04
Data file: adapt_xlsx_pool_imputation.csv
10
5
0
50
100
150
200
250
Model: IVGTTmodel1.for: Minimal Model Analysis, IVGTT

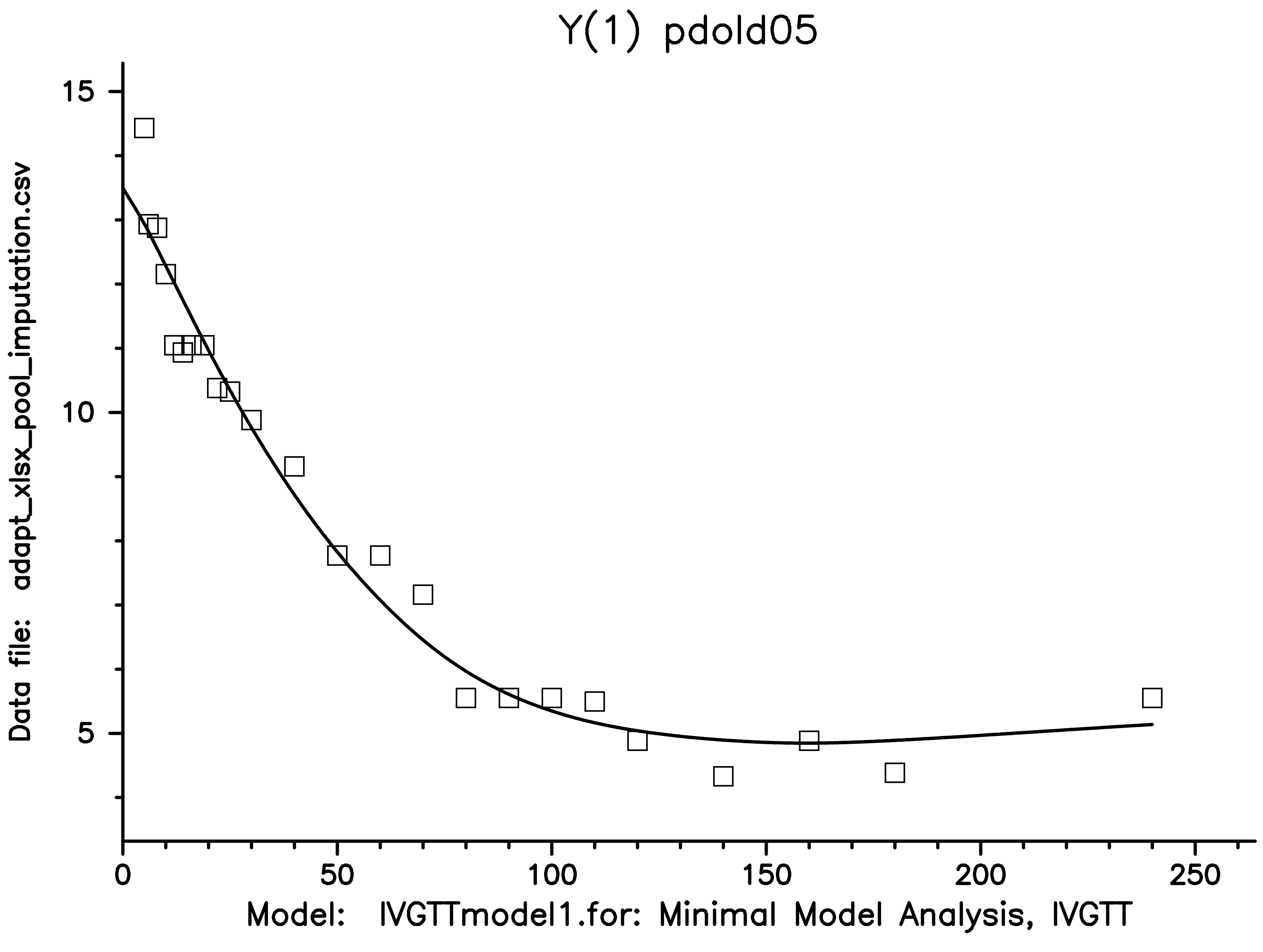
Y(1) pdold05
Data file: adapt_xlsx_pool_imputation.csv
Model: IVGTTmodel1.for: Minimal Model Analysis, IVGTT
15
10
5
0
50
100
150
200
250

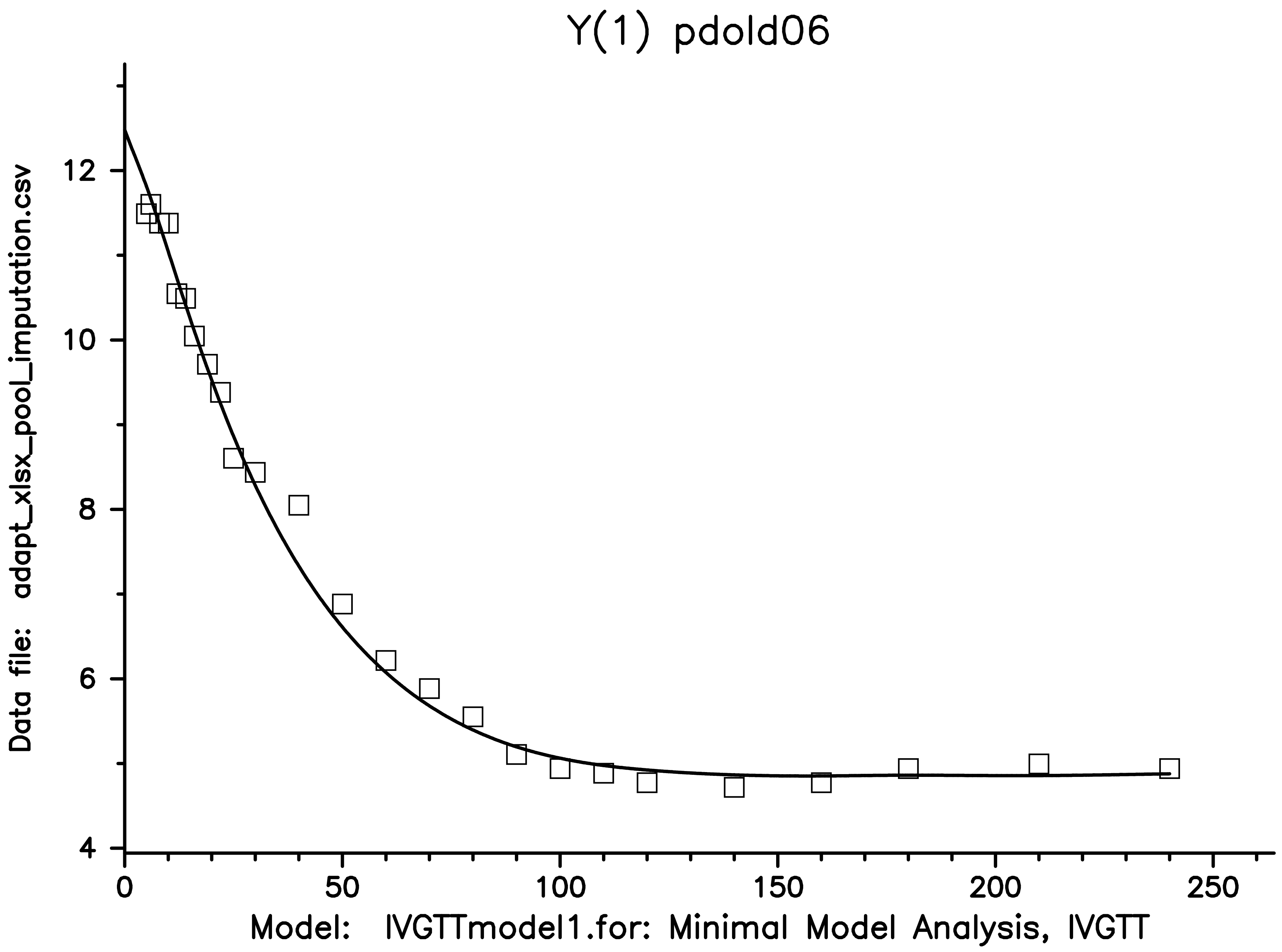
Y(1) pdold06
Data file: adapt_xlsx_pool_imputation.csv
12
10
8
6
4
0
50
100
150
200
250
Model: IVGTTmodel1.for: Minimal Model Analysis, IVGTT

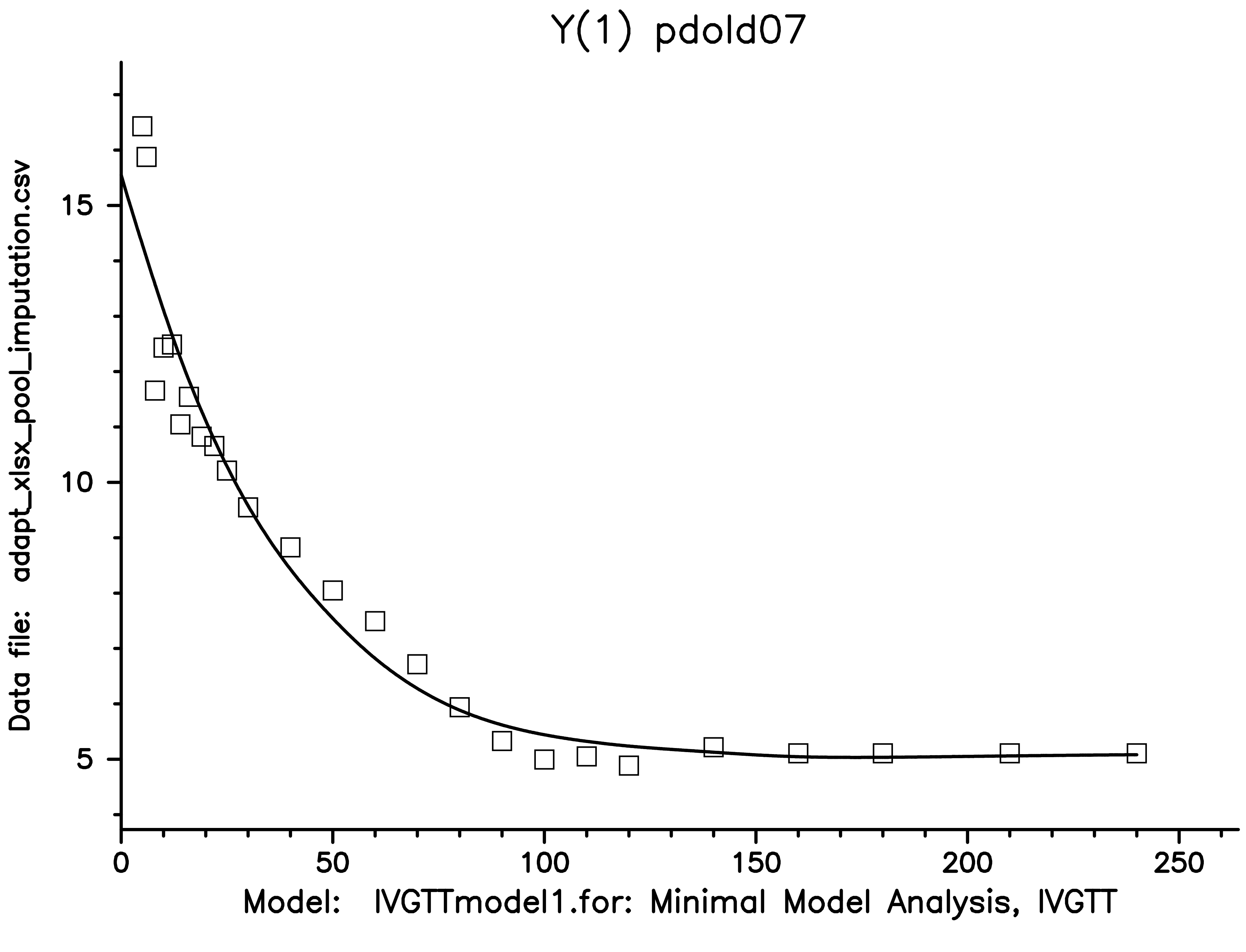

Y(1) pdold07
Data file: adapt_xlsx_pool_imputation.csv
15
10
5
0
50
100
150
200
250
Model: IVGTTmodel1.for: Minimal Model Analysis, IVGTT

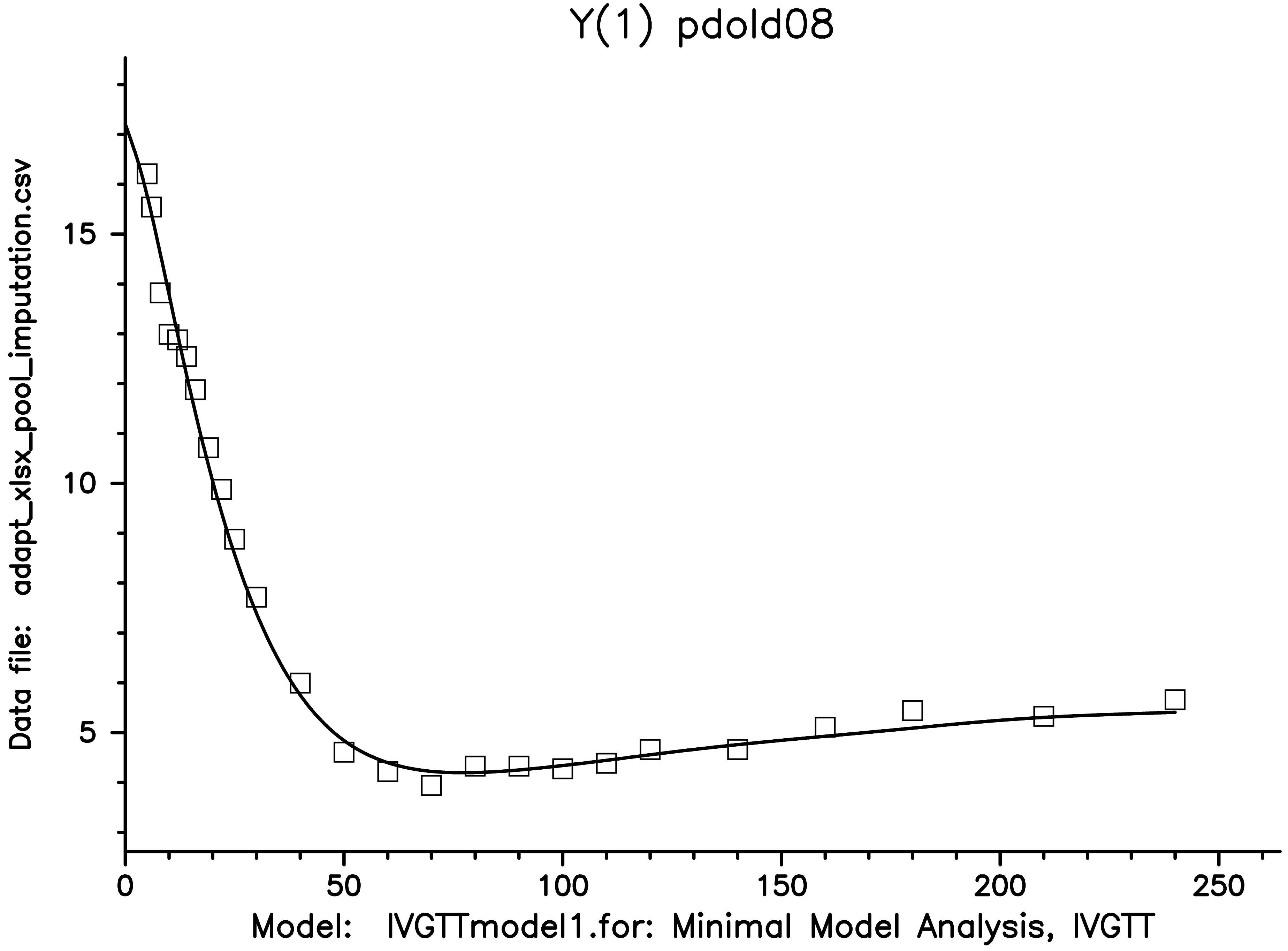

Y(1) pdold08
Data file: adapt_xlsx_pool_imputation.csv
15
10
5
0
50
100
150
200
250
Model: IVGTTmodel1.for: Minimal Model Analysis, IVGTT

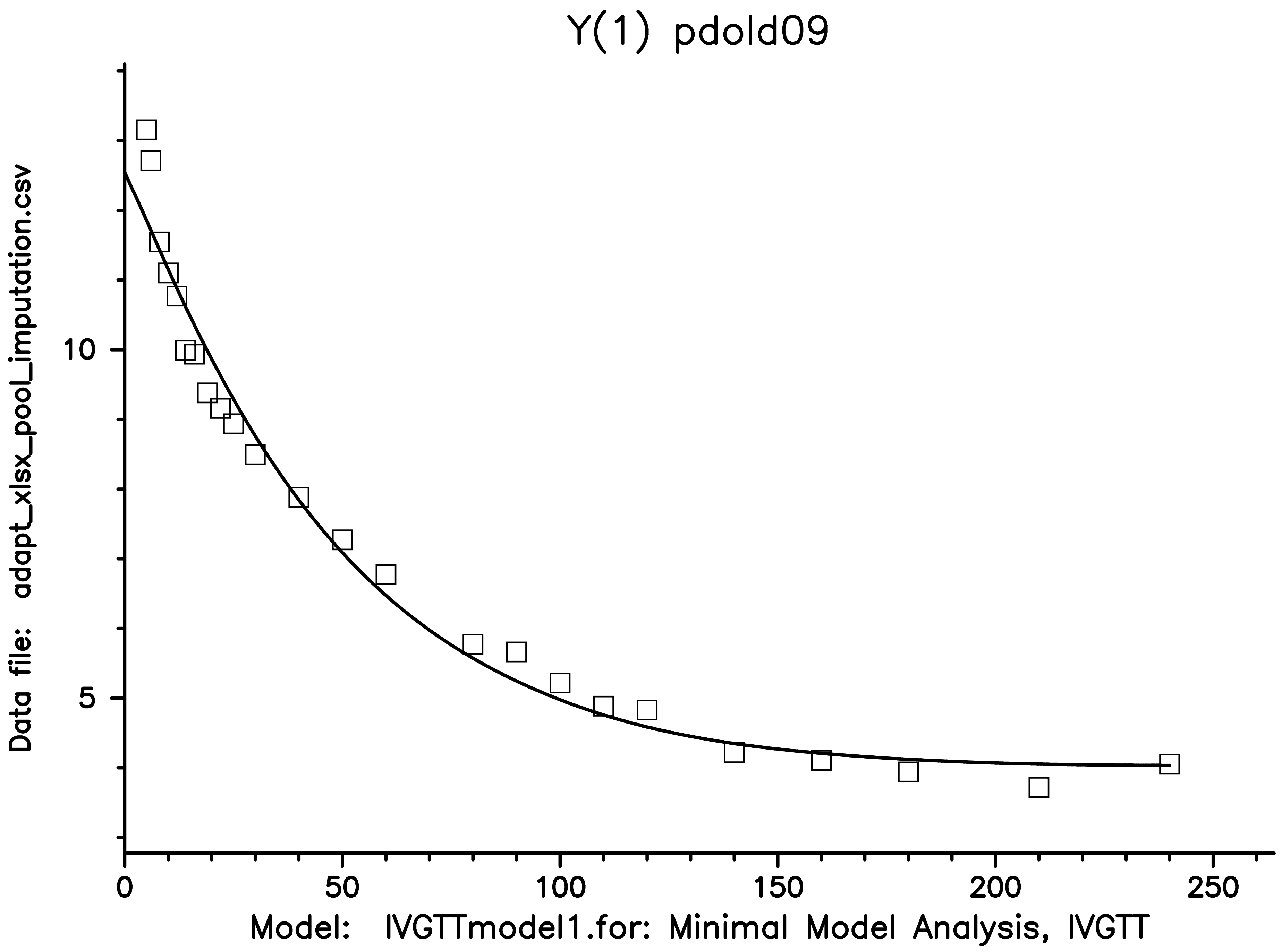

Y(1) pdold09
Data file: adapt_xlsx_pool_imputation.csv
10
5
0
50
100
150
200
250
Model: IVGTTmodel1.for: Minimal Model Analysis, IVGTT

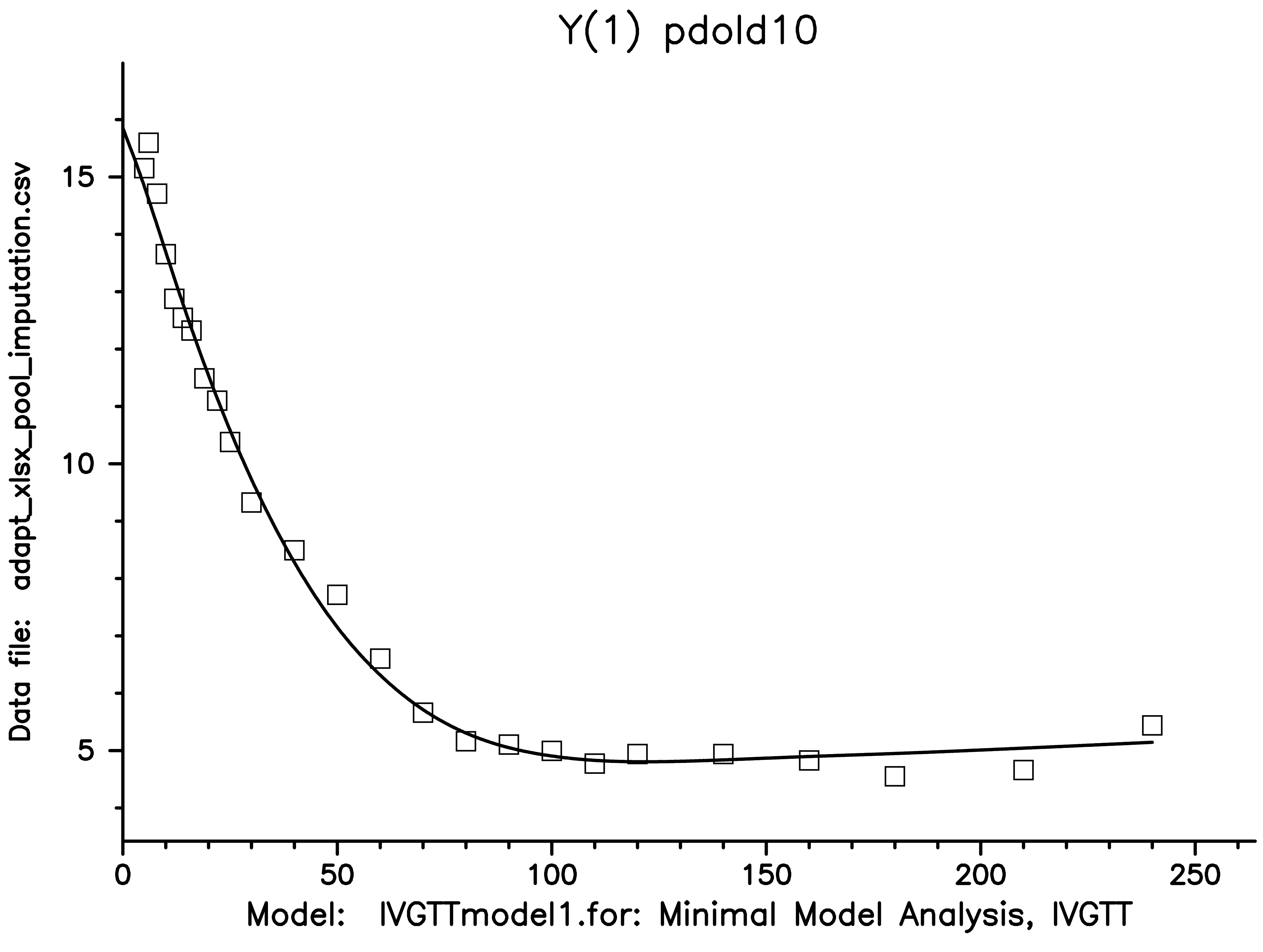

Y(1) pdold10
Data file: adapt_xlsx_pool_imputation.csv
15
10
5
0
50
100
150
200
250
Model: IVGTTmodel1.for: Minimal Model Analysis, IVGTT

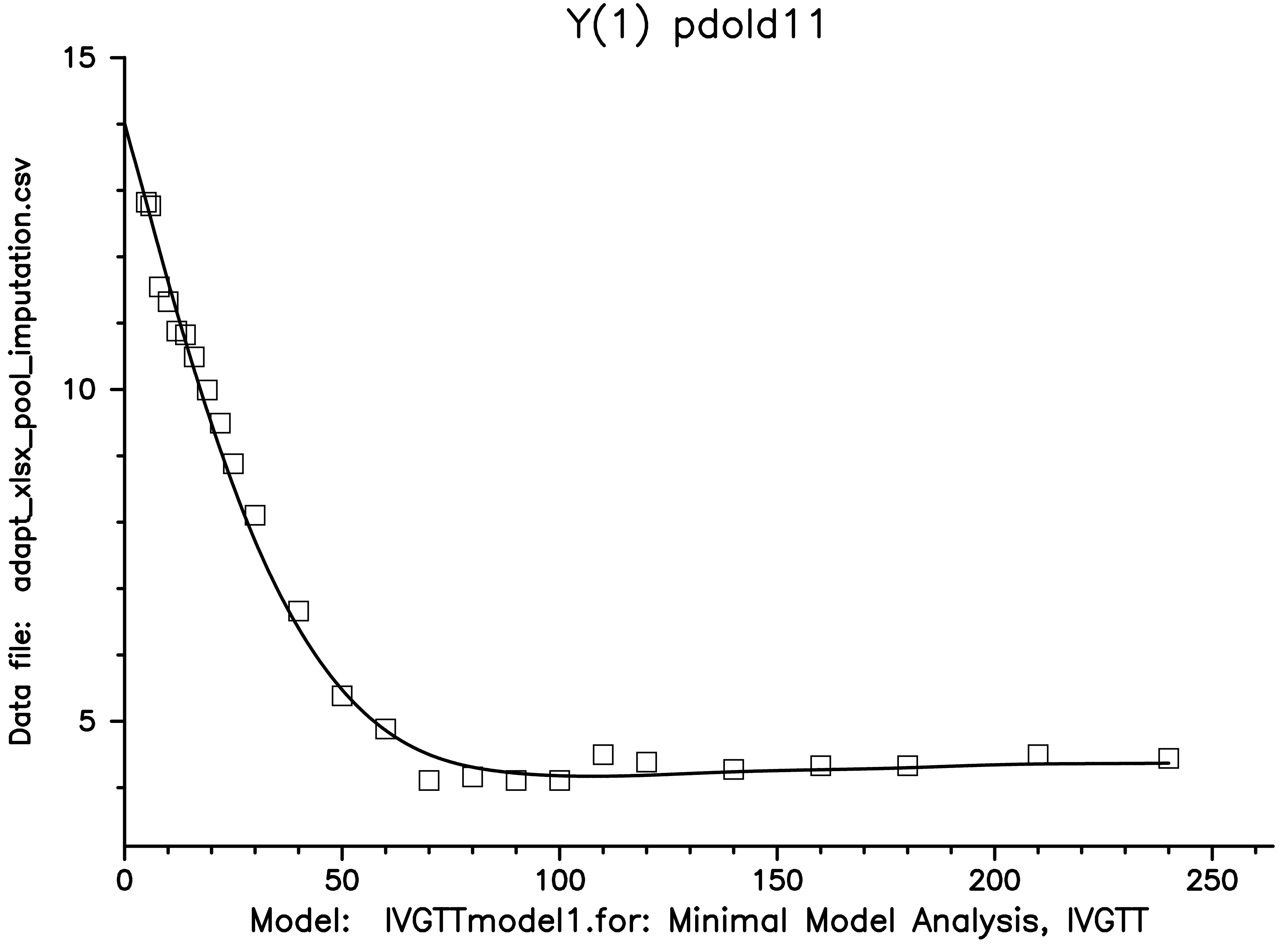
Y(1) pdold11
Data file: adapt_xlsx_pool_imputation.csv
15
10
5
0
50
100
150
200
250
Model: IVGTTmodel1.for: Minimal Model Analysis, IVGTT

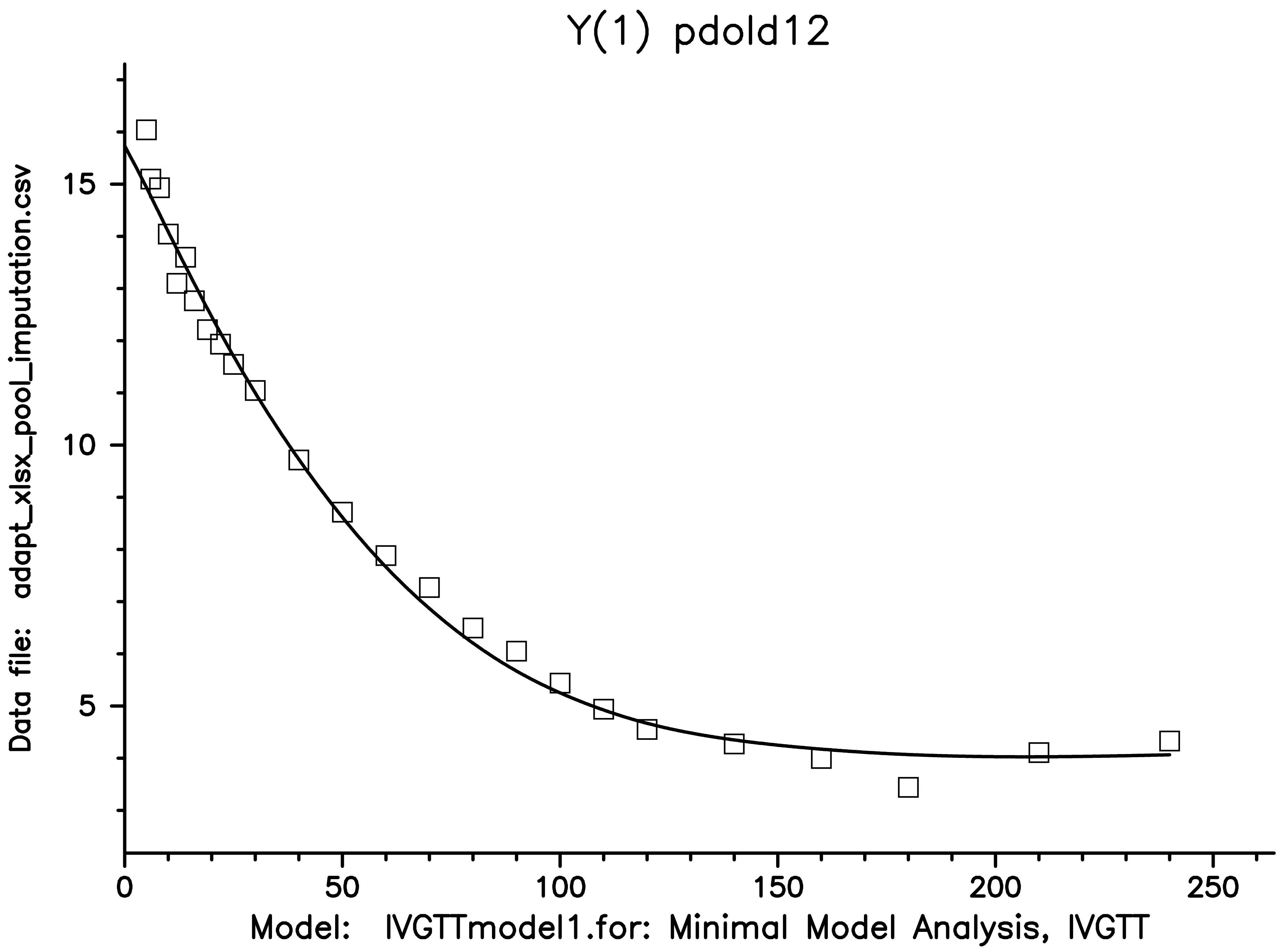
Y(1) pdold12
Data file: adapt_xlsx_pool_imputation.csv
15
10
5
0
50
100
150
200
250
Model: IVGTTmodel1.for: Minimal Model Analysis, IVGTT

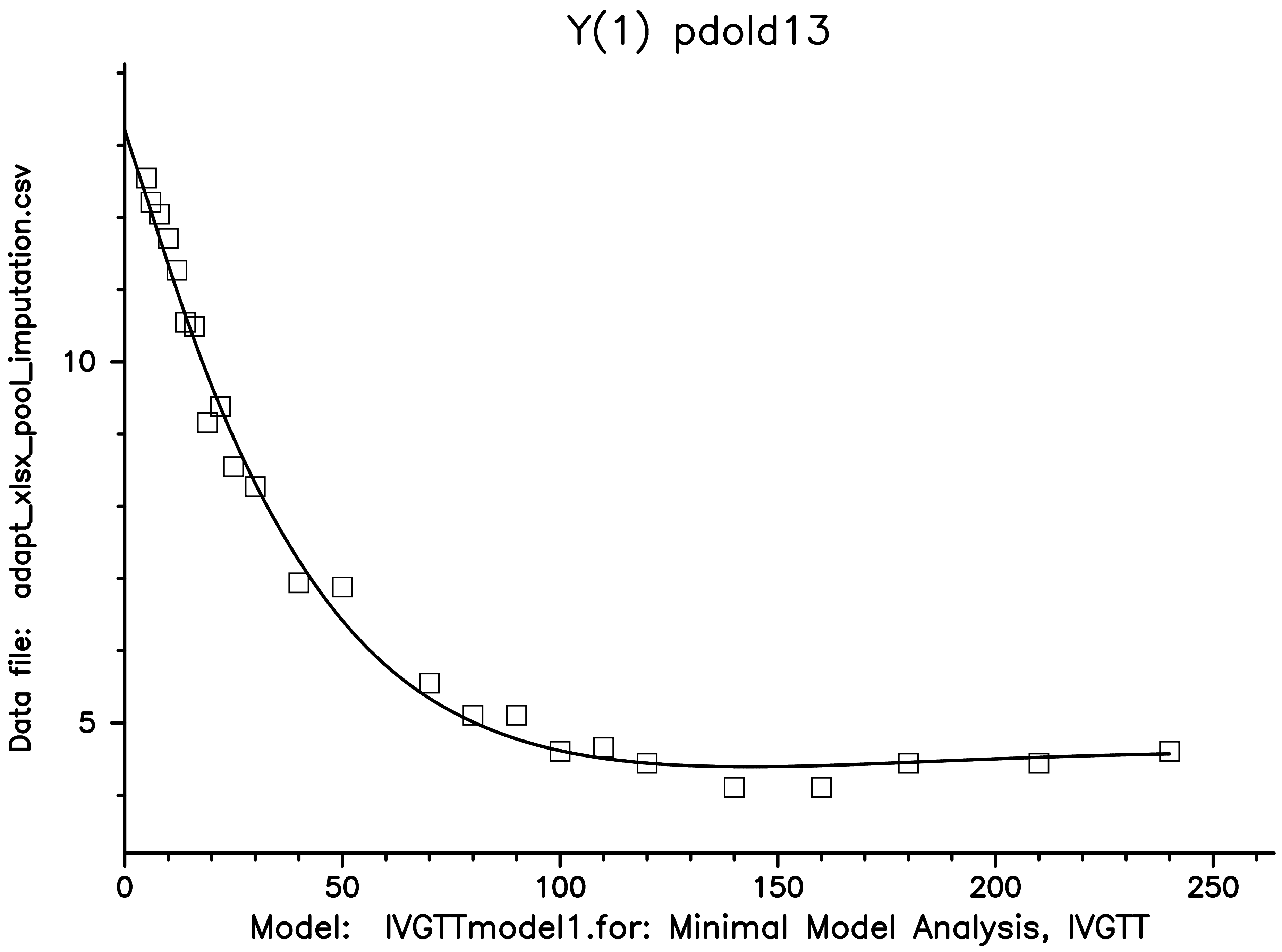
Y(1) pdold13
Data file: adapt_xlsx_pool_imputation.csv
10
5
0
50
100
150
200
250
Model: IVGTTmodel1.for: Minimal Model Analysis, IVGTT

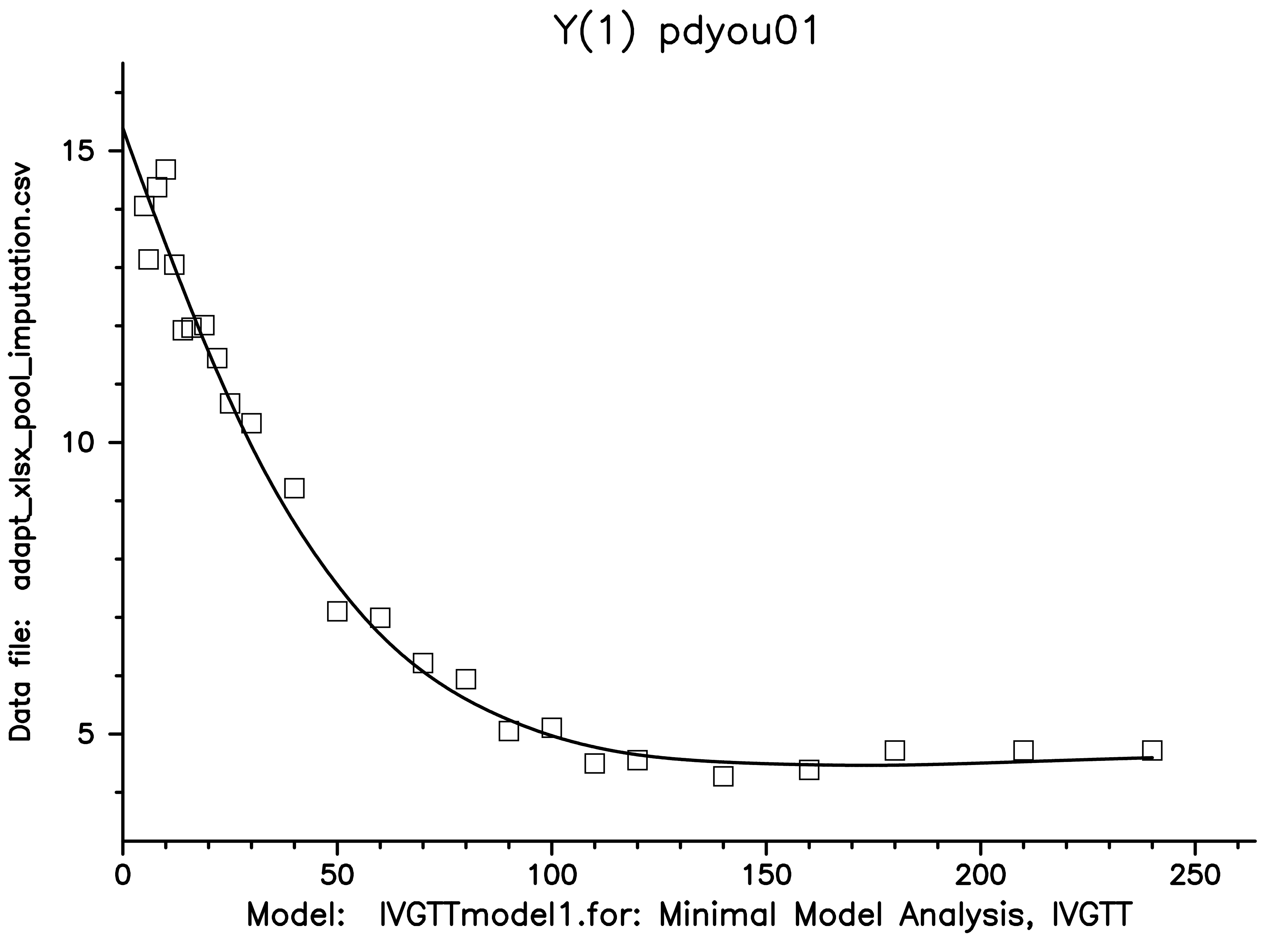
Y(1) pdyou01
Data file: adapt_xlsx_pool_imputation.csv
15
10
5
0
50
100
150
200
250
Model: IVGTTmodel1.for: Minimal Model Analysis, IVGTT

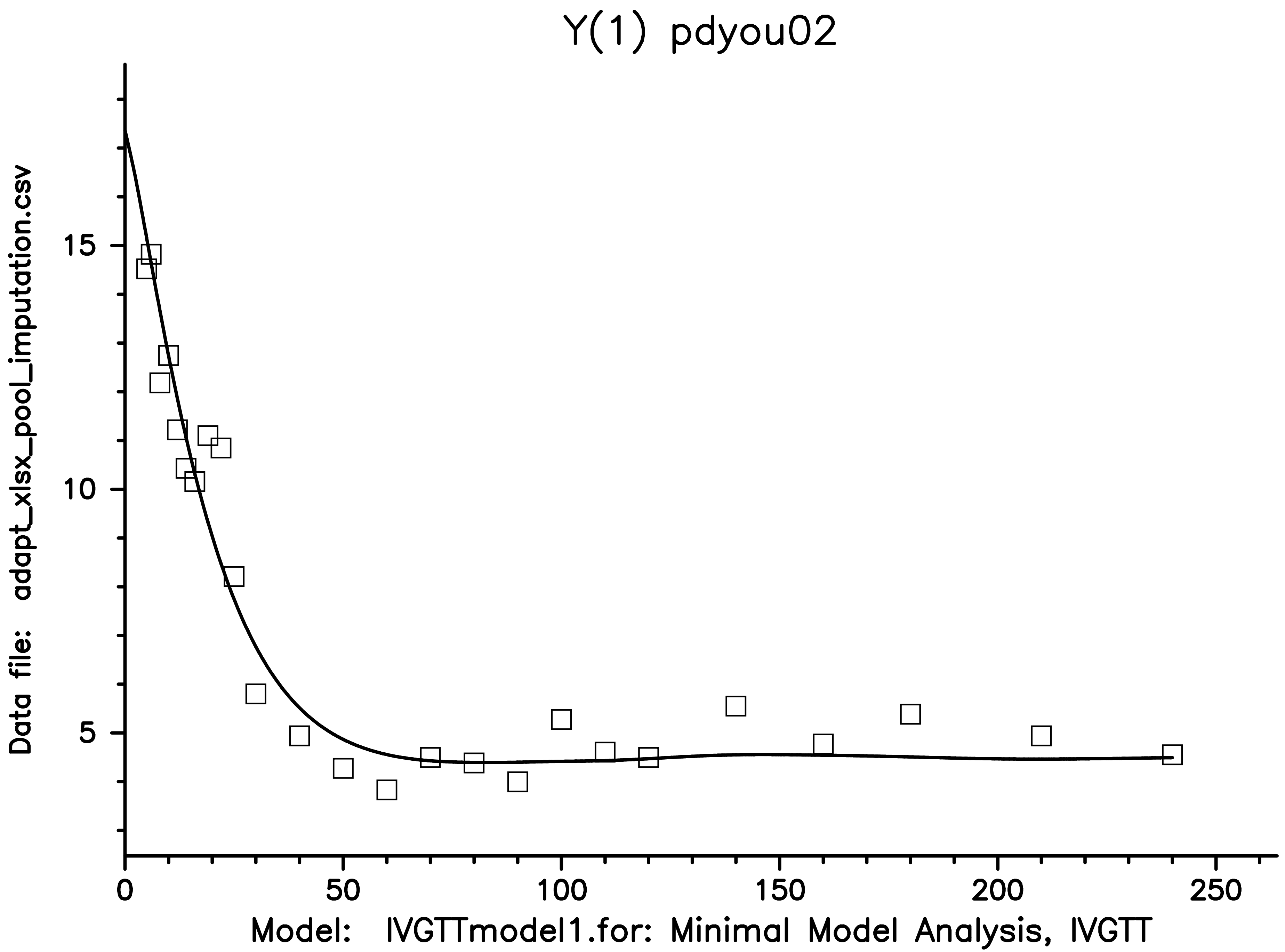
Y(1) pdyou02
Data file: adapt_xlsx_pool_imputation.csv
Model: IVGTTmodel1.for: Minimal Model Analysis, IVGTT
15
10
5
0
50
100
150
200
250

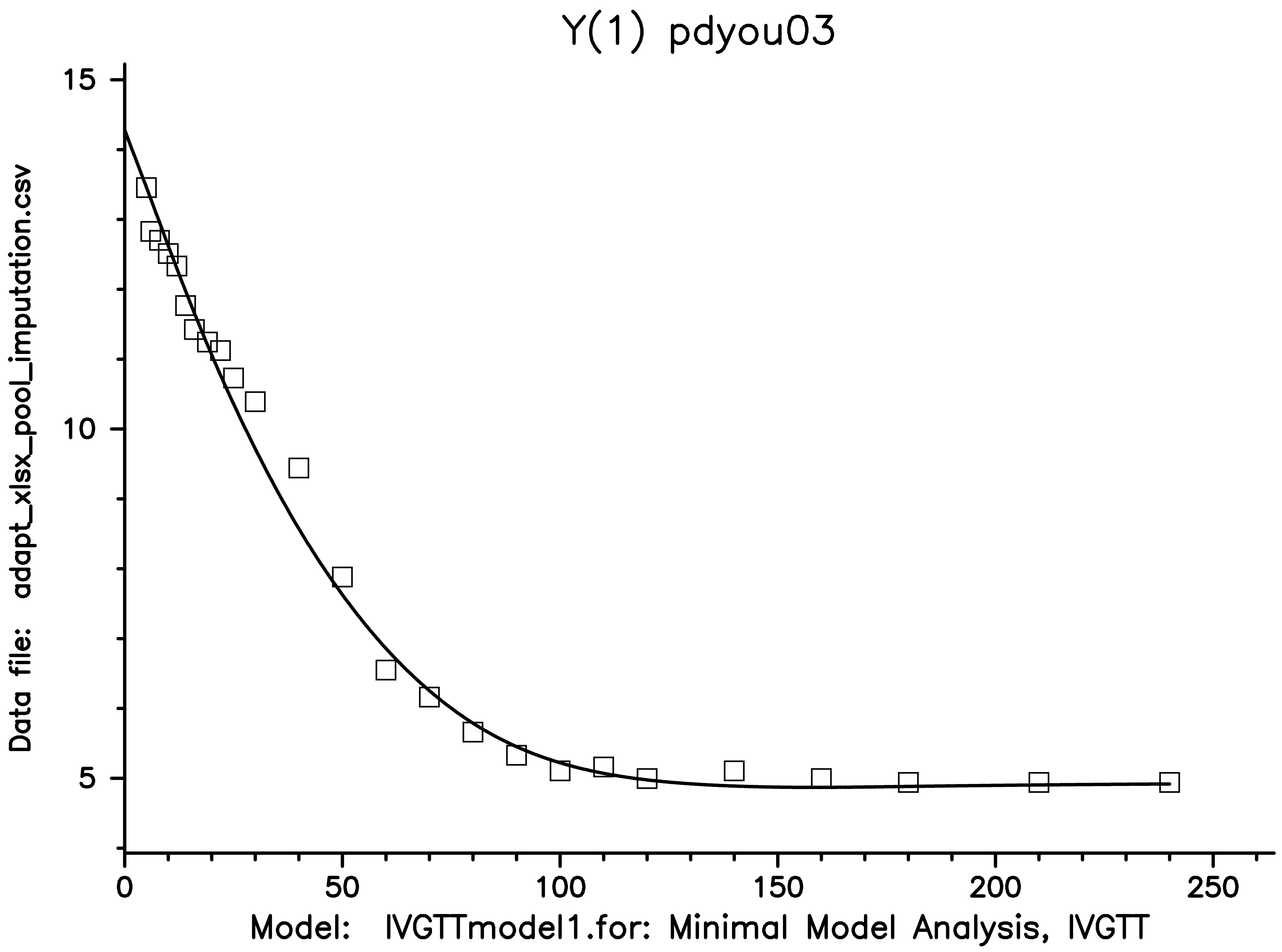
Y(1) pdyou03
Data file: adapt_xlsx_pool_imputation.csv
Model: IVGTTmodel1.for: Minimal Model Analysis, IVGTT
15
10
5
0
50
100
150
200
250

Y(1) pdyou04

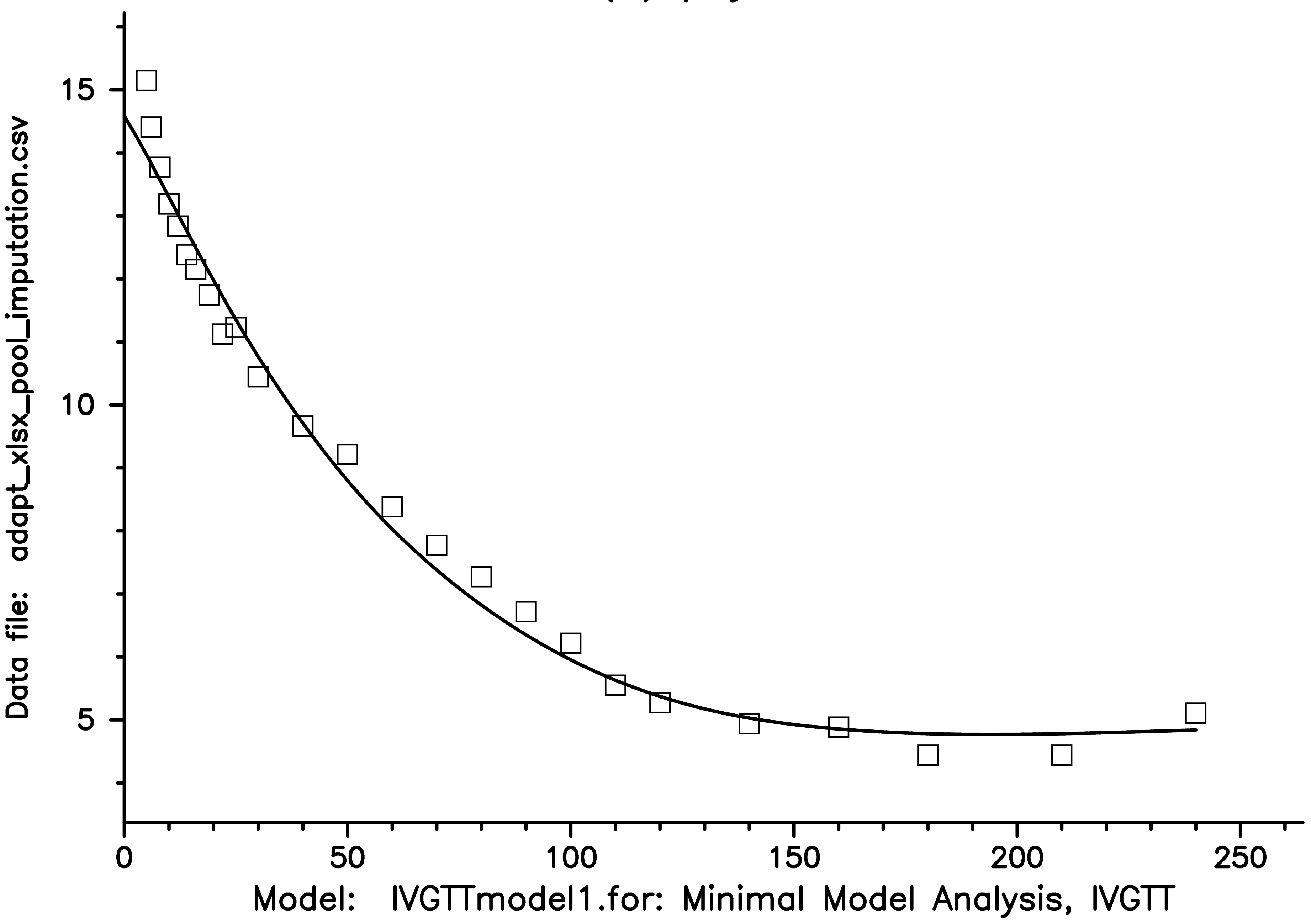

Data file: adapt_xlsx_pool_imputation.csv
15
10
5
0
50
100
150
200
250
Model: IVGTTmodel1.for: Minimal Model Analysis, IVGTT

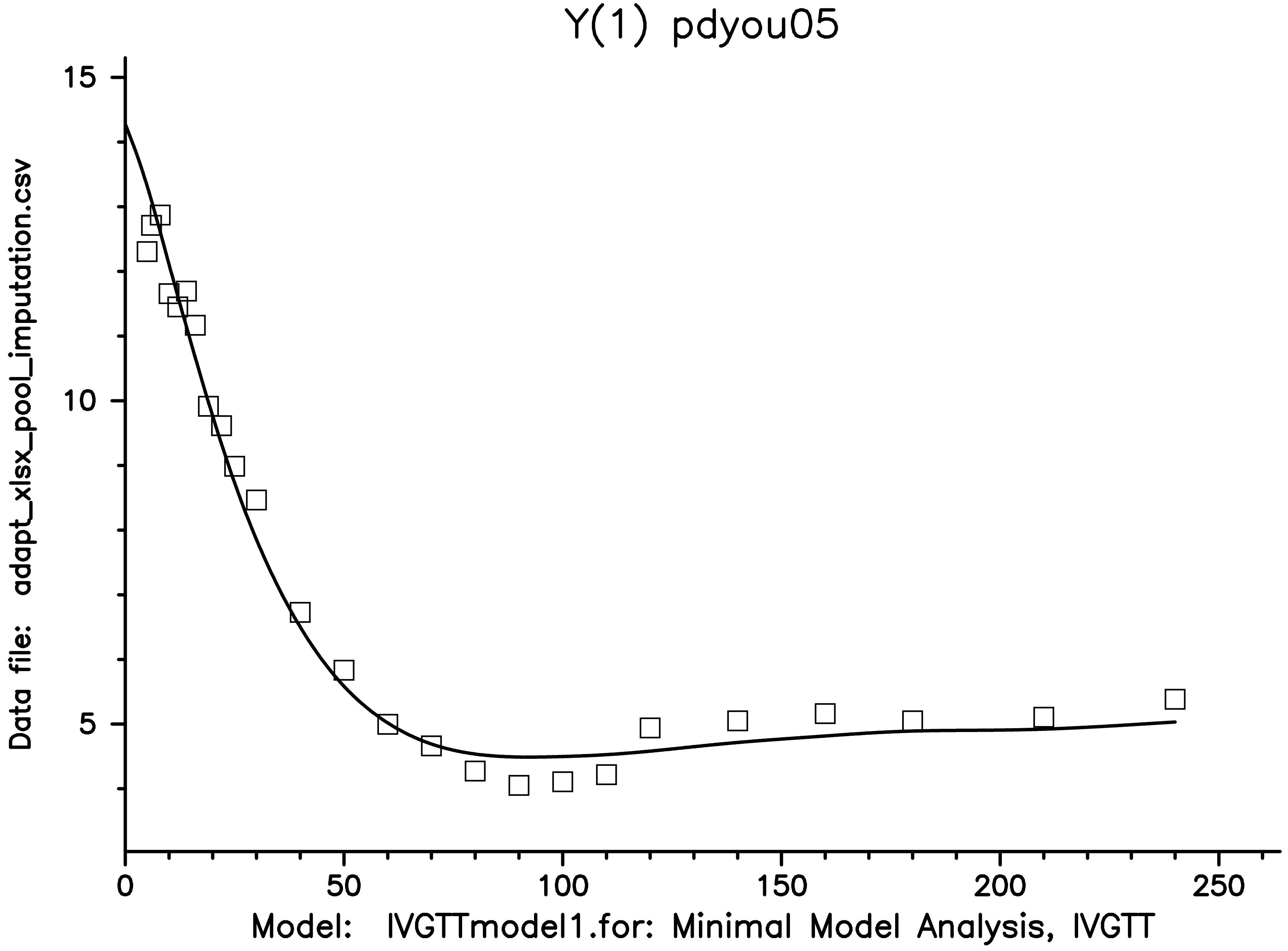
Y(1) pdyou05
Data file: adapt_xlsx_pool_imputation.csv
Model: IVGTTmodel1.for: Minimal Model Analysis, IVGTT
15
10
5
0
50
100
150
200
250

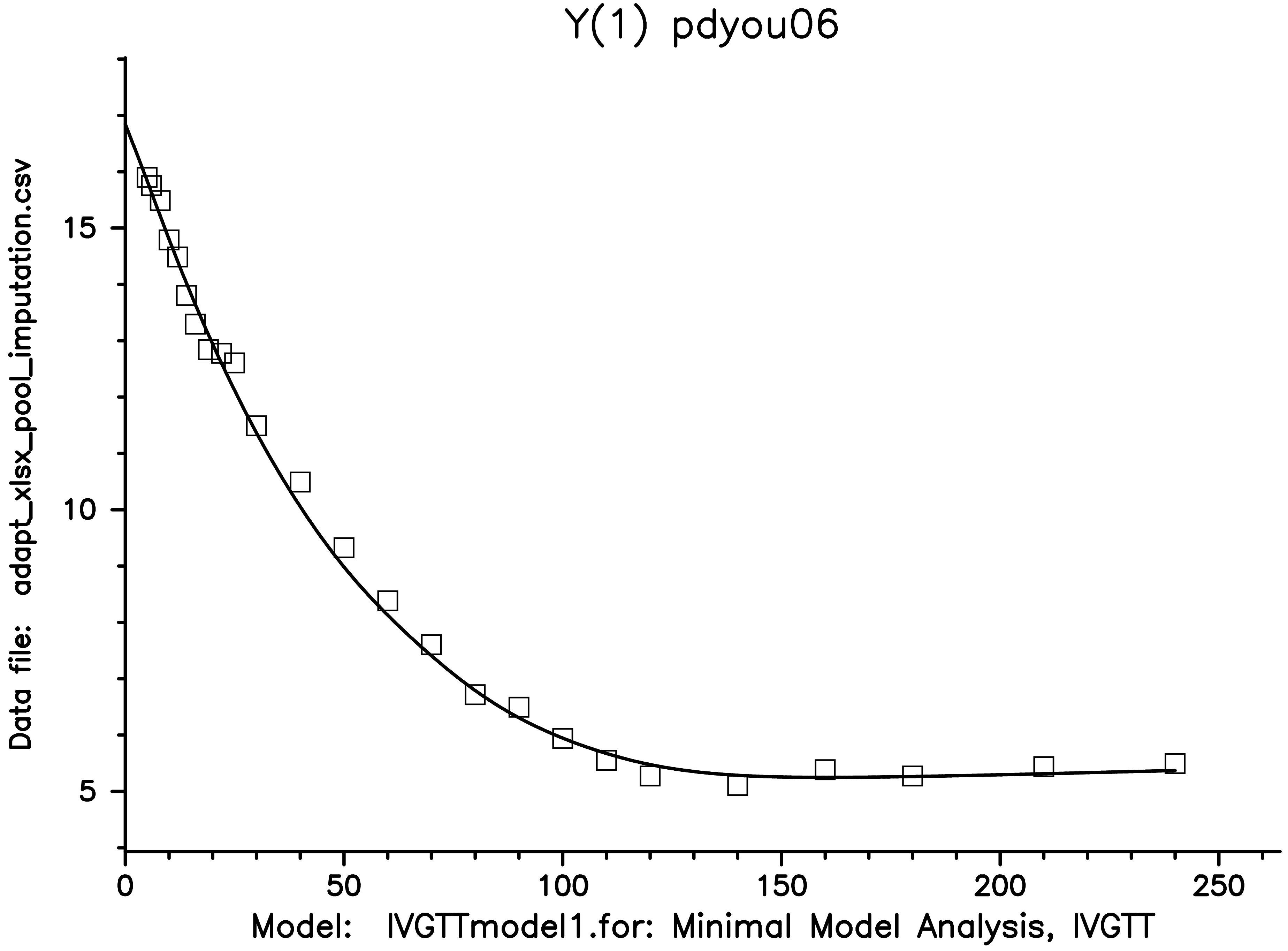
Y(1) pdyou06
Data file: adapt_xlsx_pool_imputation.csv
15
10
5
0
50
100
150
200
250
Model: IVGTTmodel1.for: Minimal Model Analysis, IVGTT

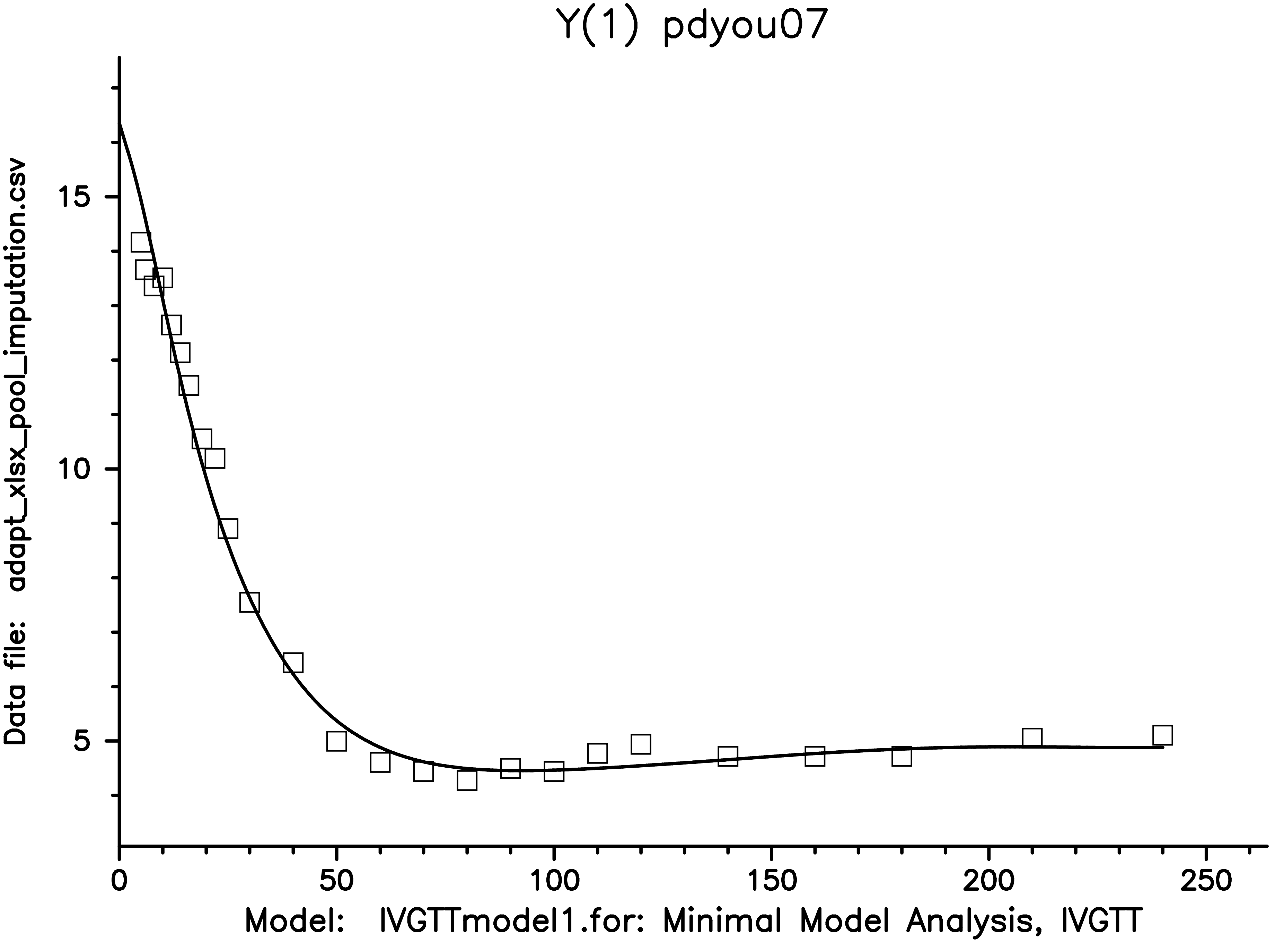
Y(1) pdyou07
Data file: adapt_xlsx_pool_imputation.csv
15
10
5
0
50
100
150
200
250
Model: IVGTTmodel1.for: Minimal Model Analysis, IVGTT

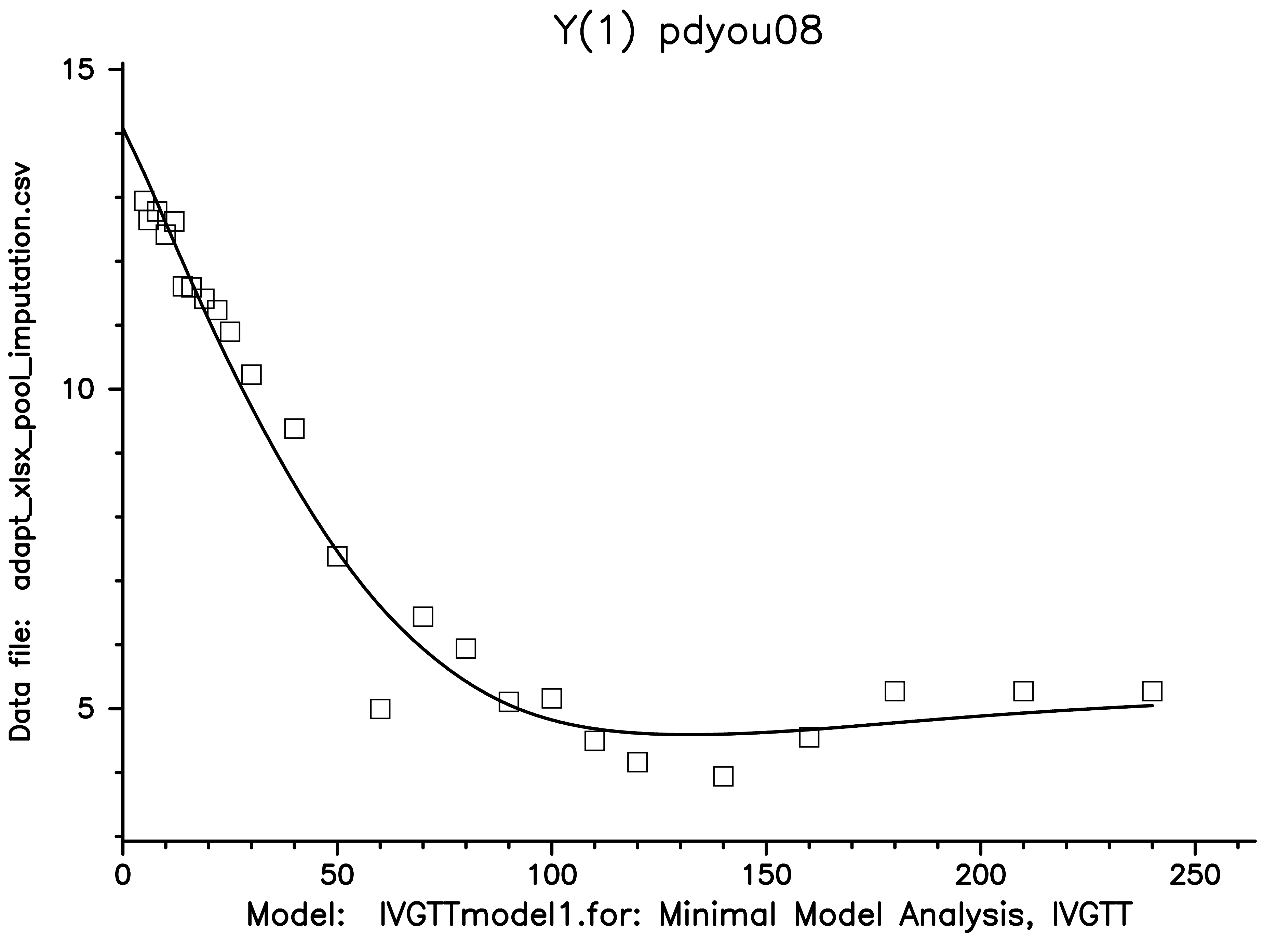
Y(1) pdyou08
15
10
5
0
50
100
150
200
250
Data file: adapt_xlsx_pool_imputation.csv
Model: IVGTTmodel1.for: Minimal Model Analysis, IVGTT

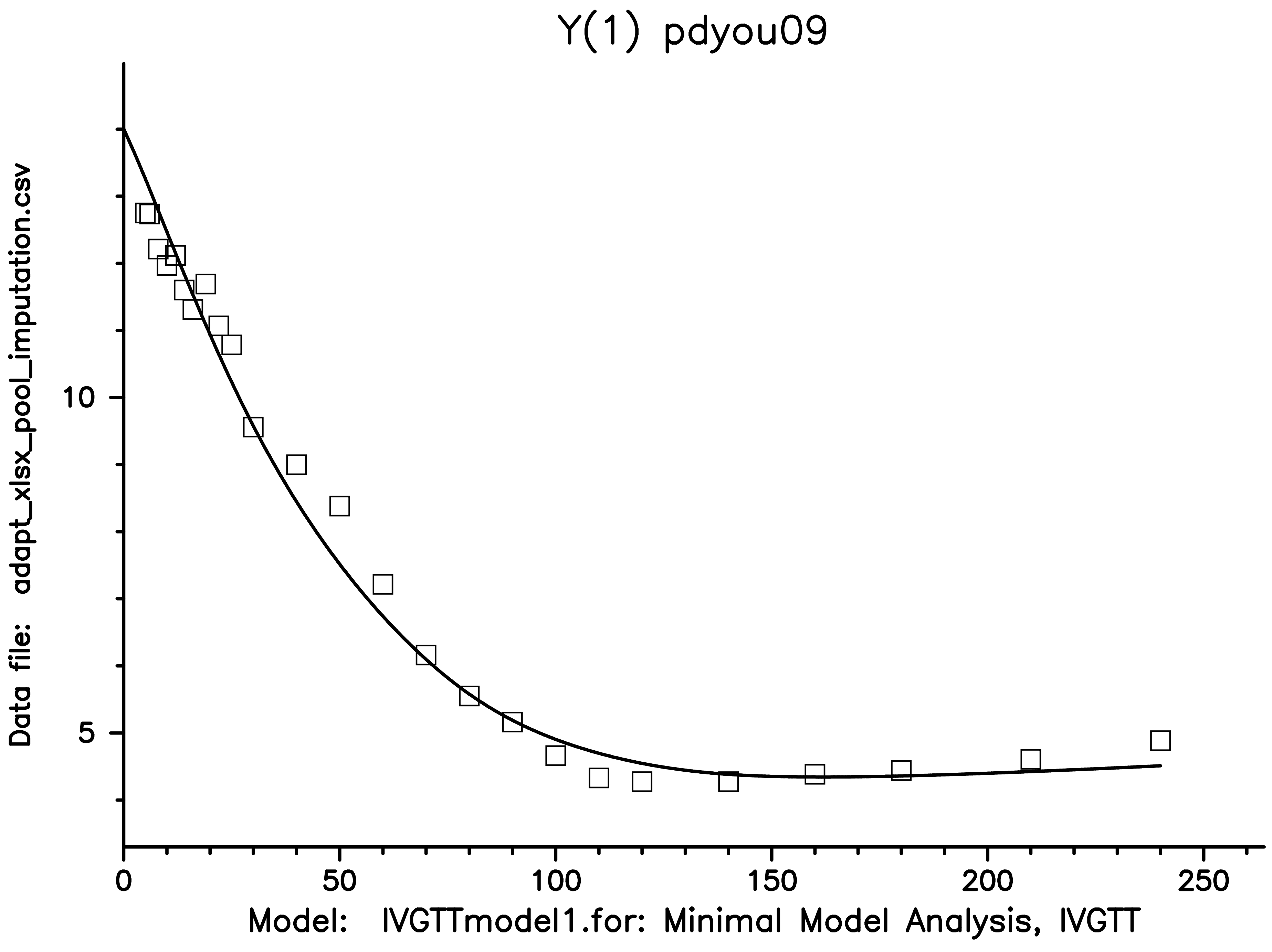
Y(1) pdyou09
Data file: adapt_xlsx_pool_imputation.csv
10
5
0
50
100
150
200
250
Model: IVGTTmodel1.for: Minimal Model Analysis, IVGTT

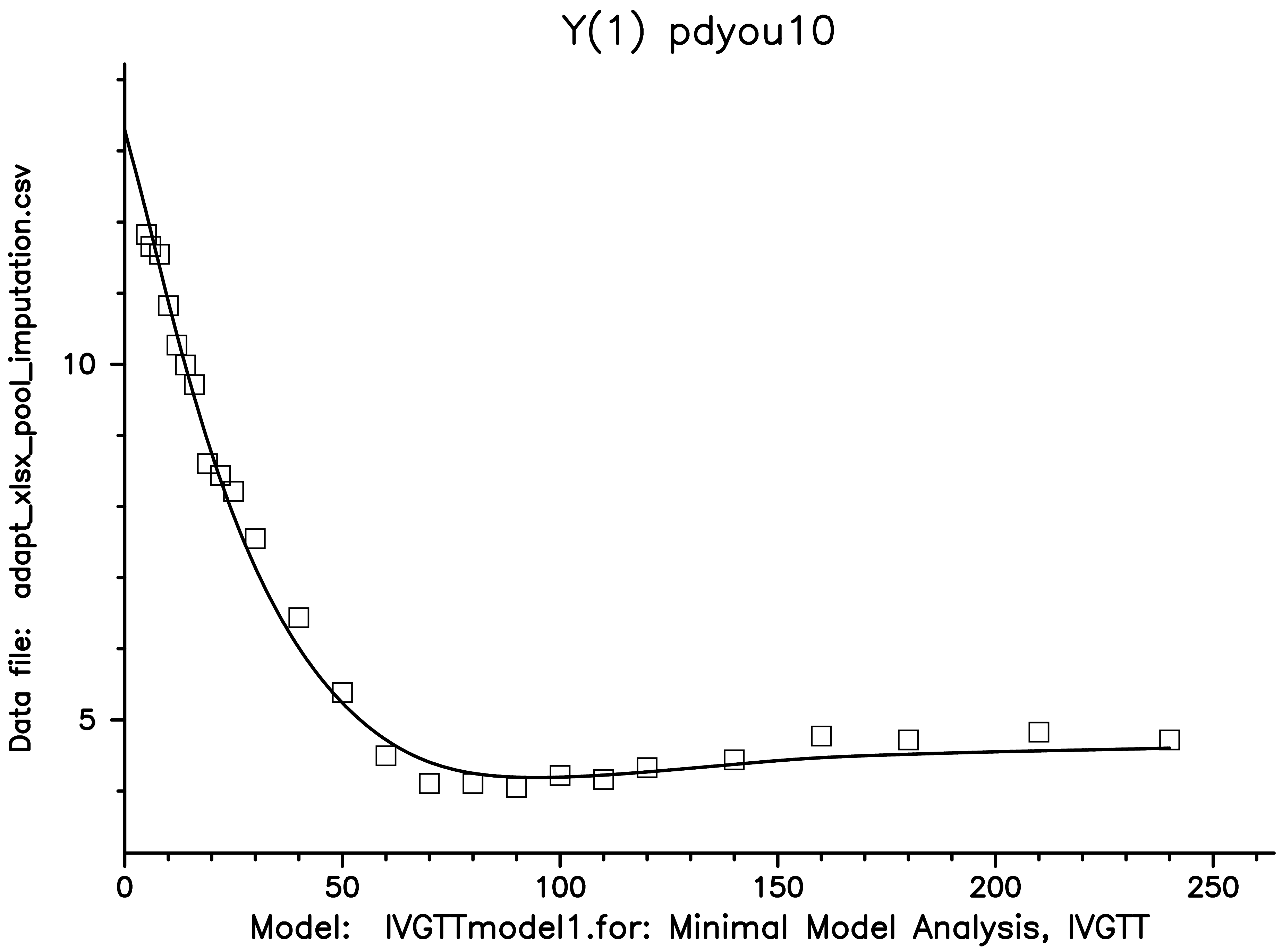
Y(1) pdyou10
Data file: adapt_xlsx_pool_imputation.csv
10
5
0
50
100
150
200
250
Model: IVGTTmodel1.for: Minimal Model Analysis, IVGTT

Y(1) rmcnt01

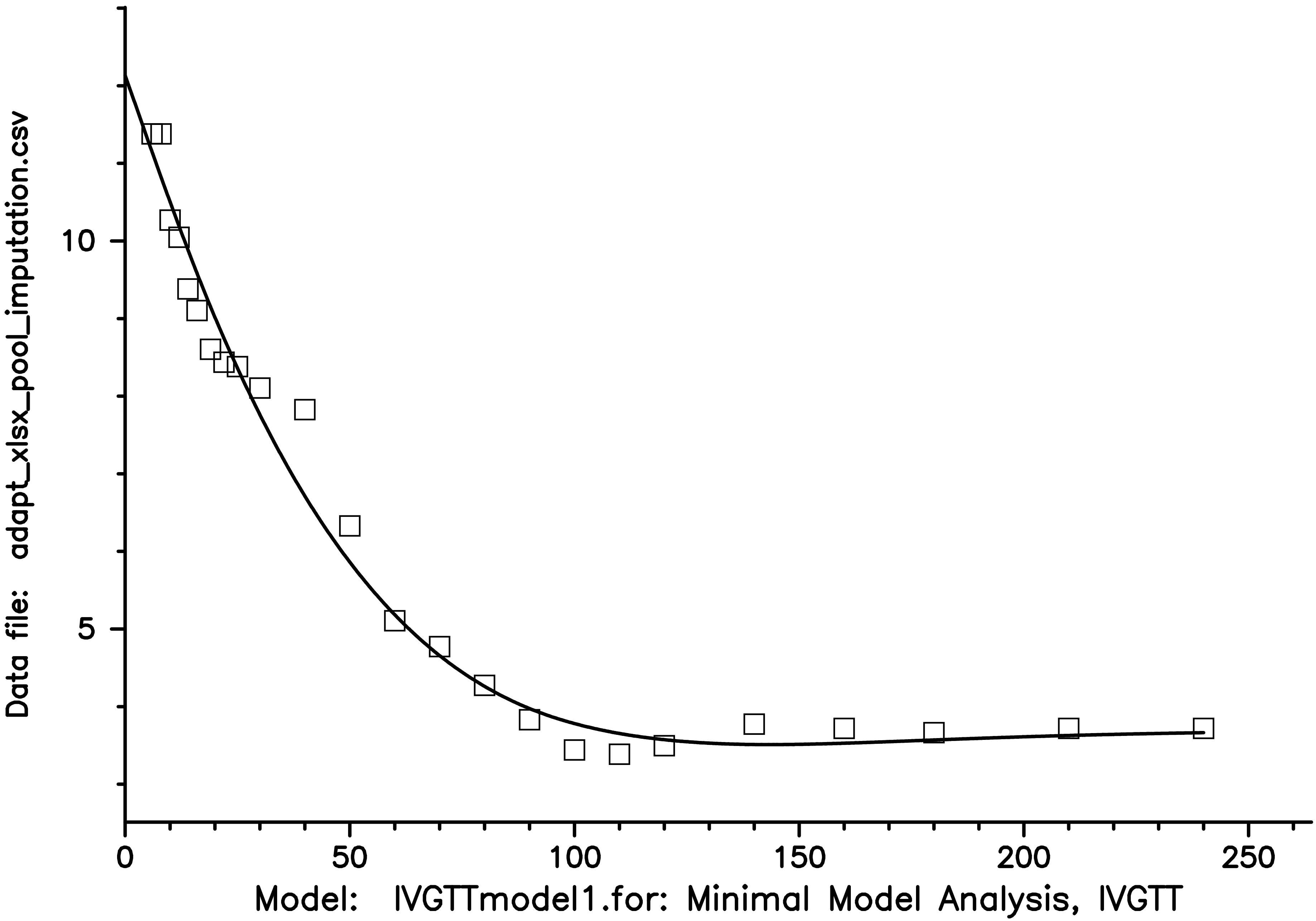

10
5
0
50
100
150
200
250
Data file: adapt_xlsx_pool_imputation.csv
Model: IVGTTmodel1.for: Minimal Model Analysis, IVGTT

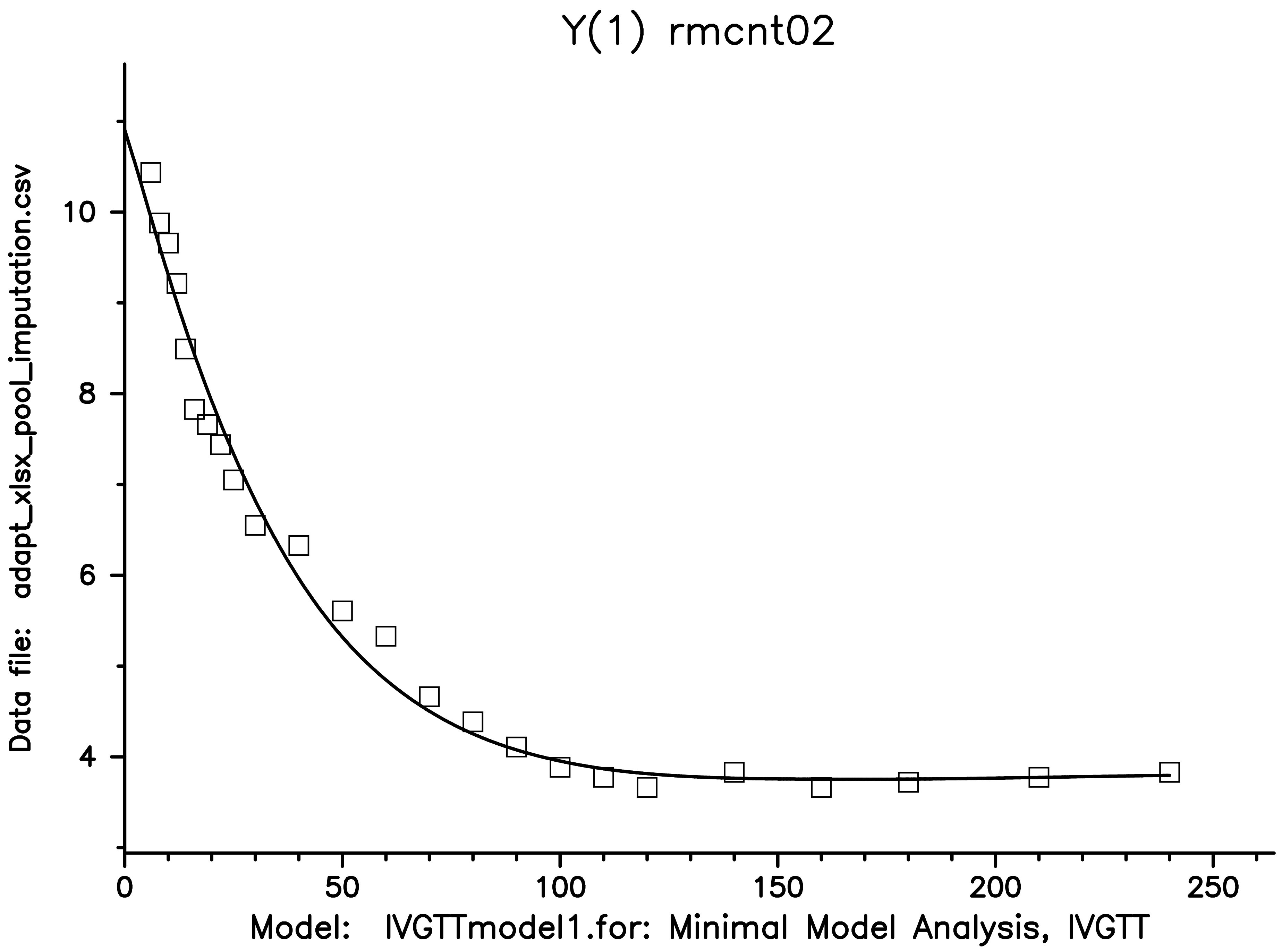

Y(1) rmcnt02
Data file: adapt_xlsx_pool_imputation.csv
10
8
6
4
0
50
100
150
200
250
Model: IVGTTmodel1.for: Minimal Model Analysis, IVGTT

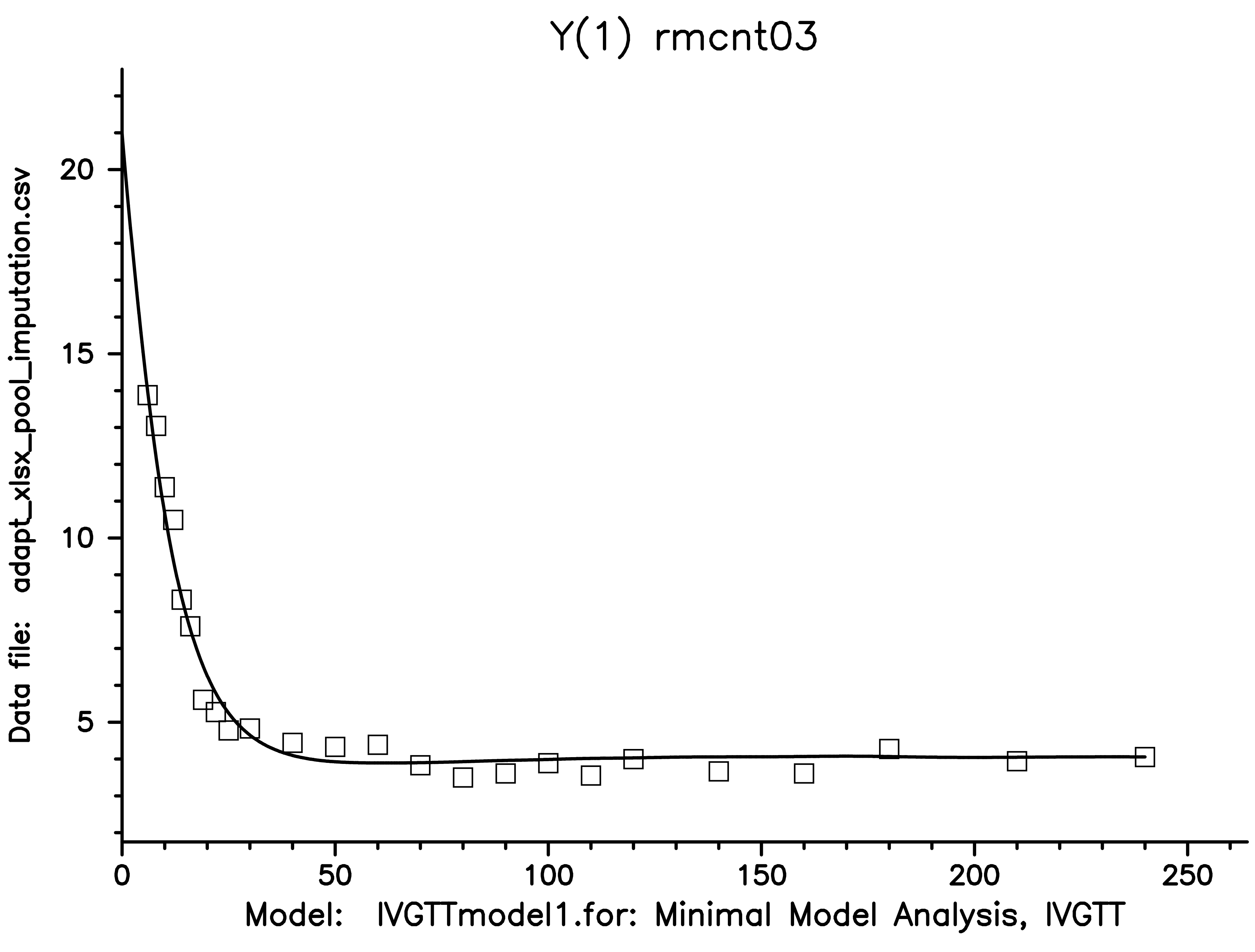
Y(1) rmcnt03
Data file: adapt_xlsx_pool_imputation.csv
20
15
10
5
0
50
100
150
200
250
Model: IVGTTmodel1.for: Minimal Model Analysis, IVGTT

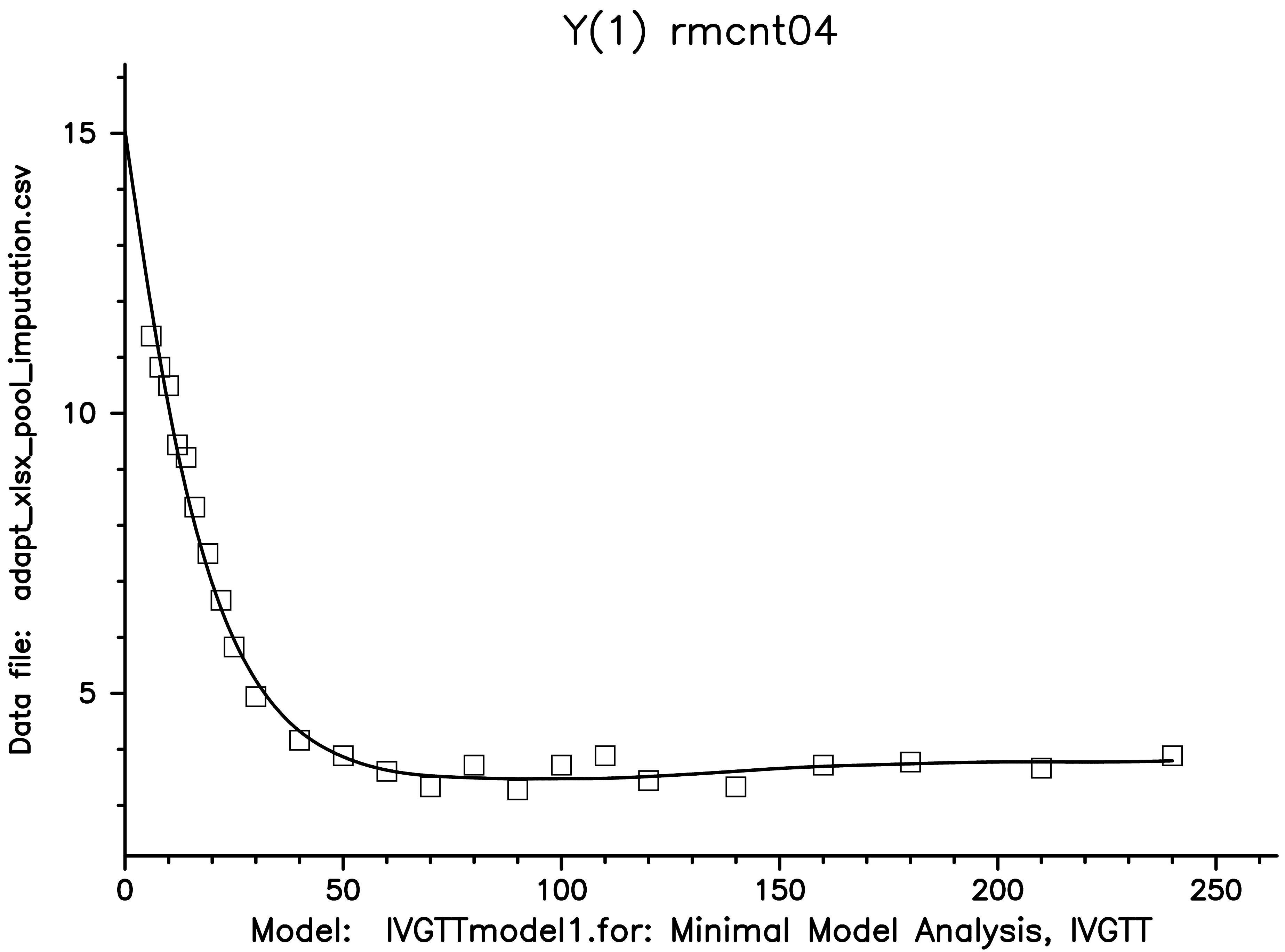
Y(1) rmcnt04
15
10
5
0
50
100
150
200
250
Data file: adapt_xlsx_pool_imputation.csv
Model: IVGTTmodel1.for: Minimal Model Analysis, IVGTT

Y(1) rmcnt05

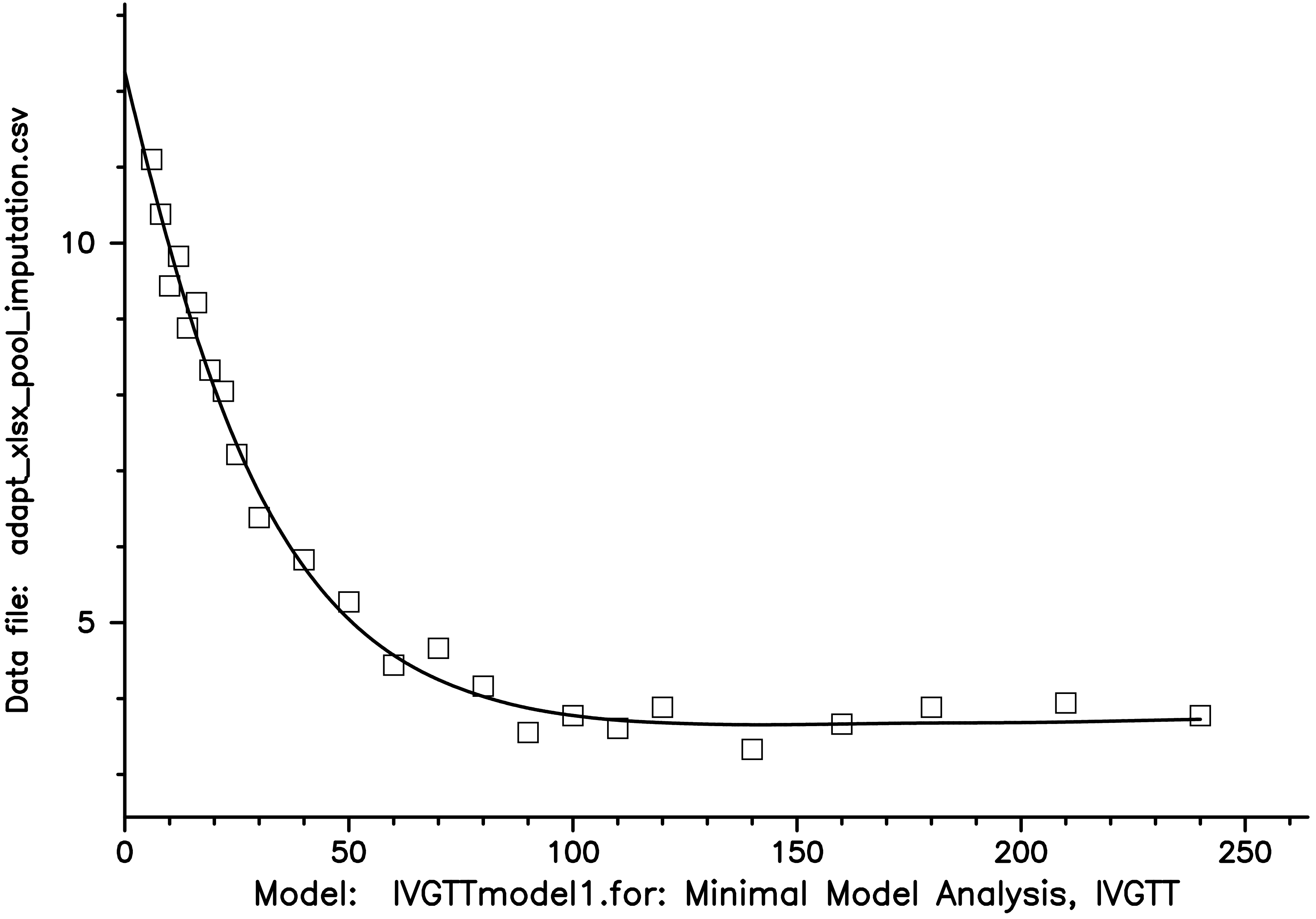

Data file: adapt_xlsx_pool_imputation.csv
10
5
0
50
100
150
200
250
Model: IVGTTmodel1.for: Minimal Model Analysis, IVGTT

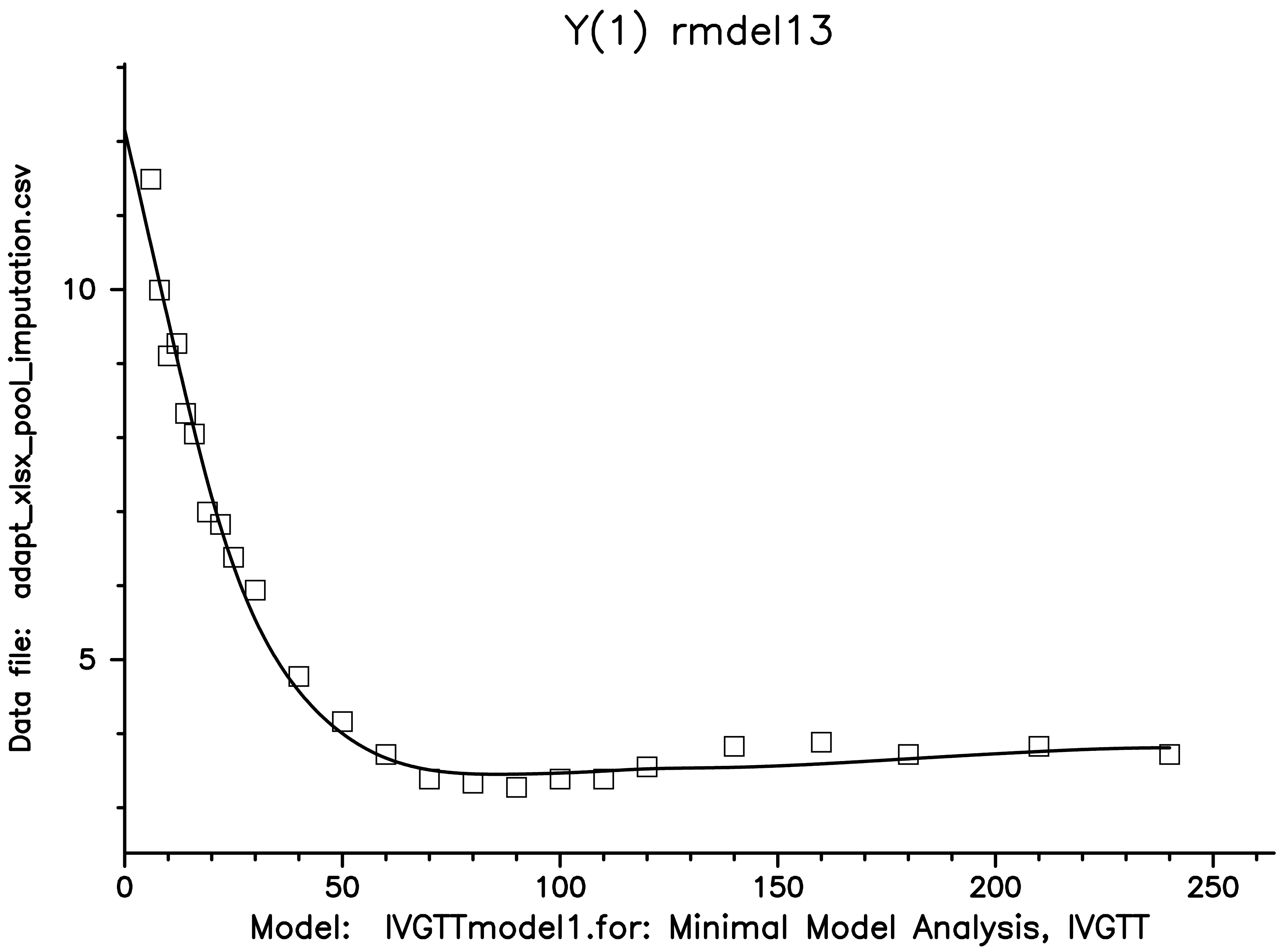
Y(1) rmdel13
Data file: adapt_xlsx_pool_imputation.csv
10
5
0
50
100
150
200
250
Model: IVGTTmodel1.for: Minimal Model Analysis, IVGTT

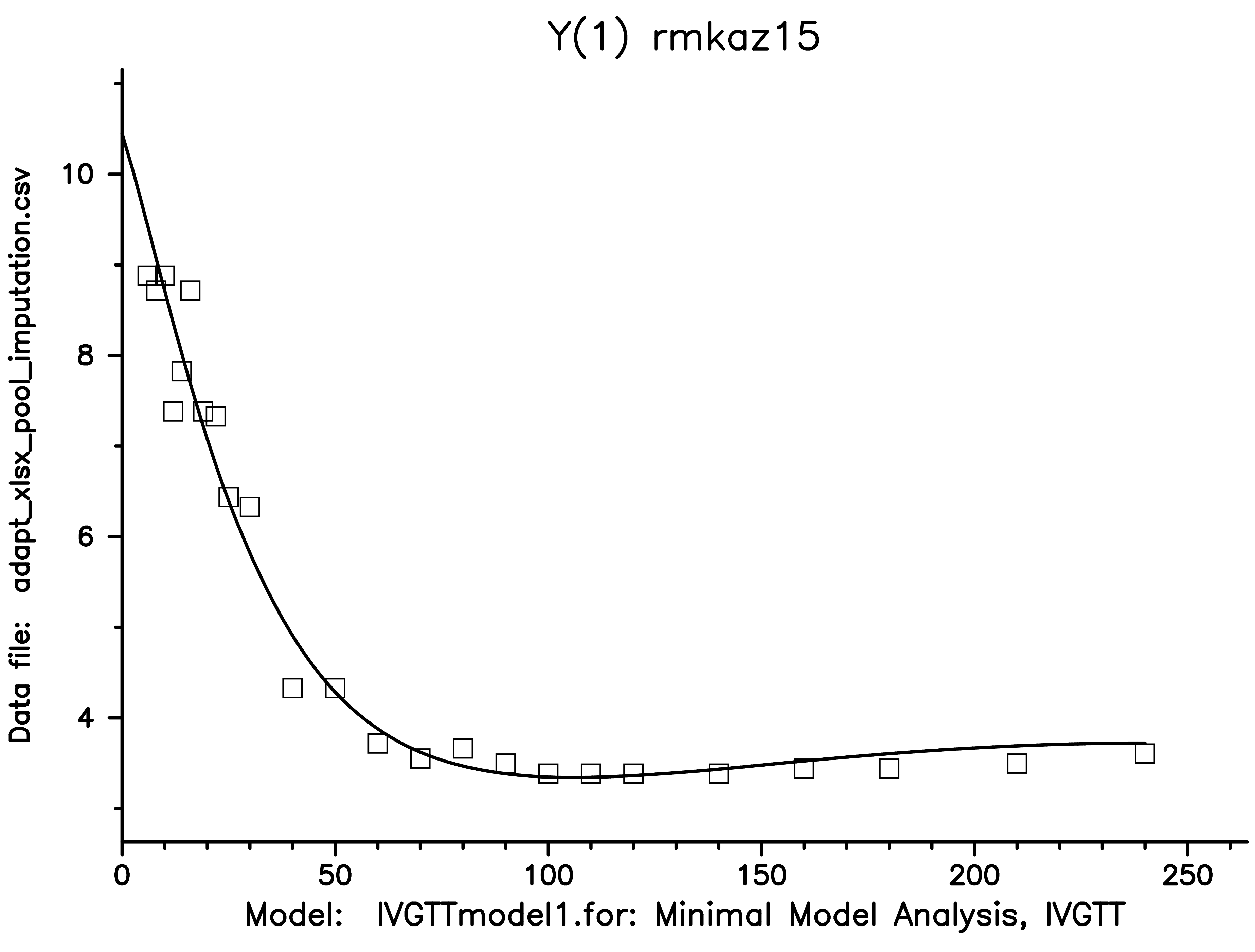
Y(1) rmkaz15
Data file: adapt_xlsx_pool_imputation.csv
10
8
6
4
0
50
100
150
200
250
Model: IVGTTmodel1.for: Minimal Model Analysis, IVGTT

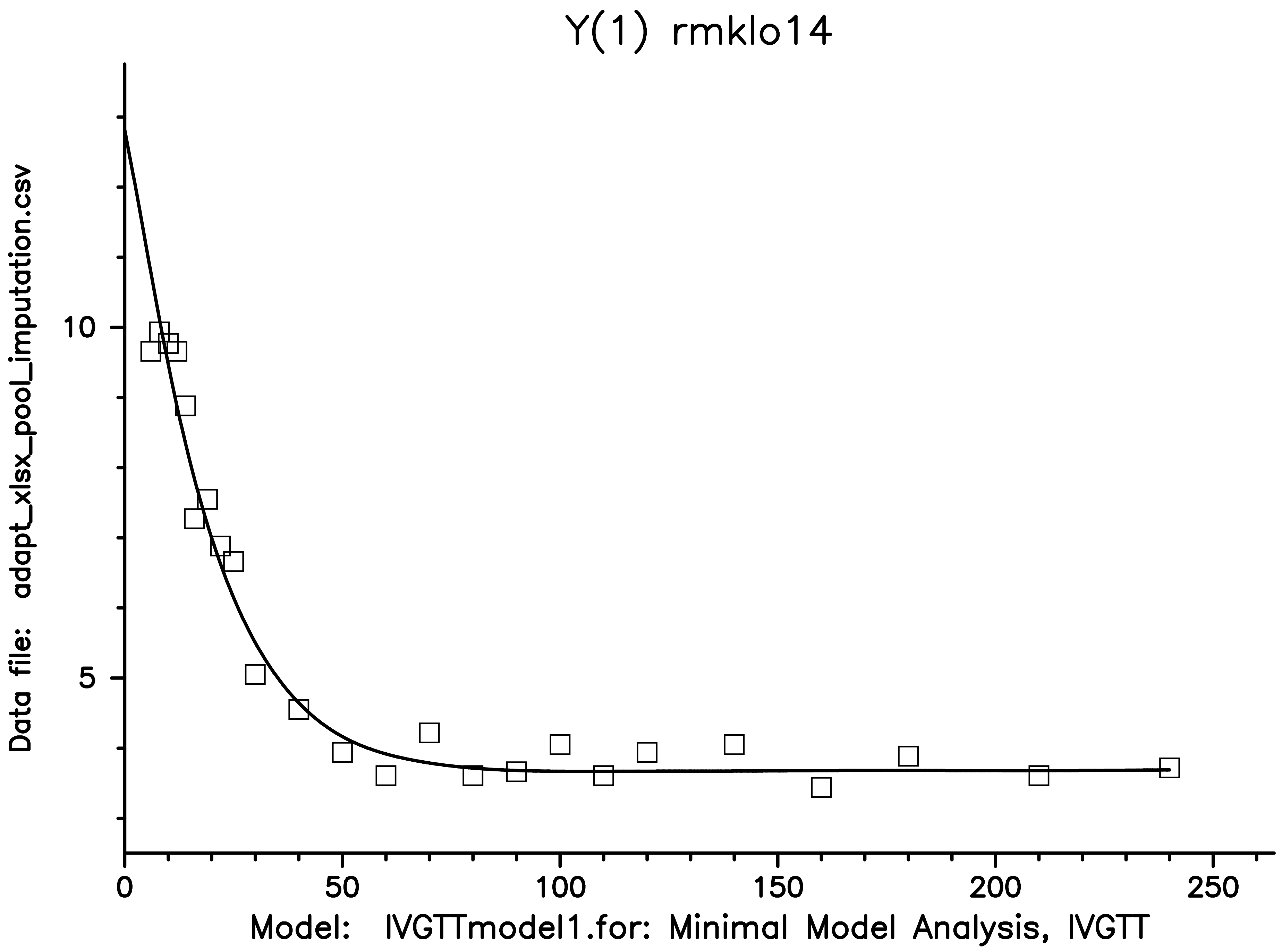

Y(1) rmklo14
Data file: adapt_xlsx_pool_imputation.csv
10
5
0
50
100
150
200
250
Model: IVGTTmodel1.for: Minimal Model Analysis, IVGTT

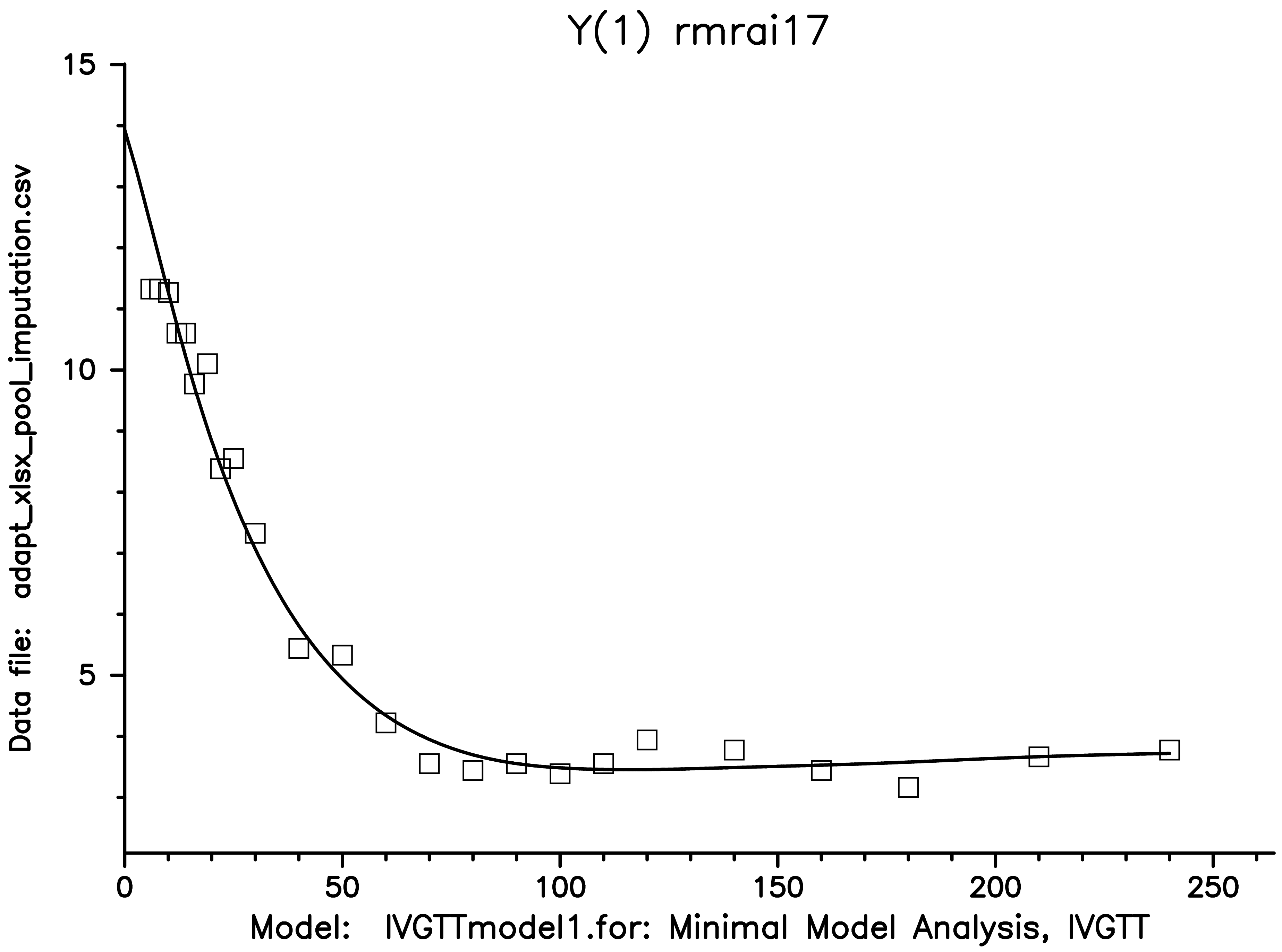

Y(1) rmrai17
Data file: adapt_xlsx_pool_imputation.csv
15
10
5
0
50
100
150
200
250
Model: IVGTTmodel1.for: Minimal Model Analysis, IVGTT

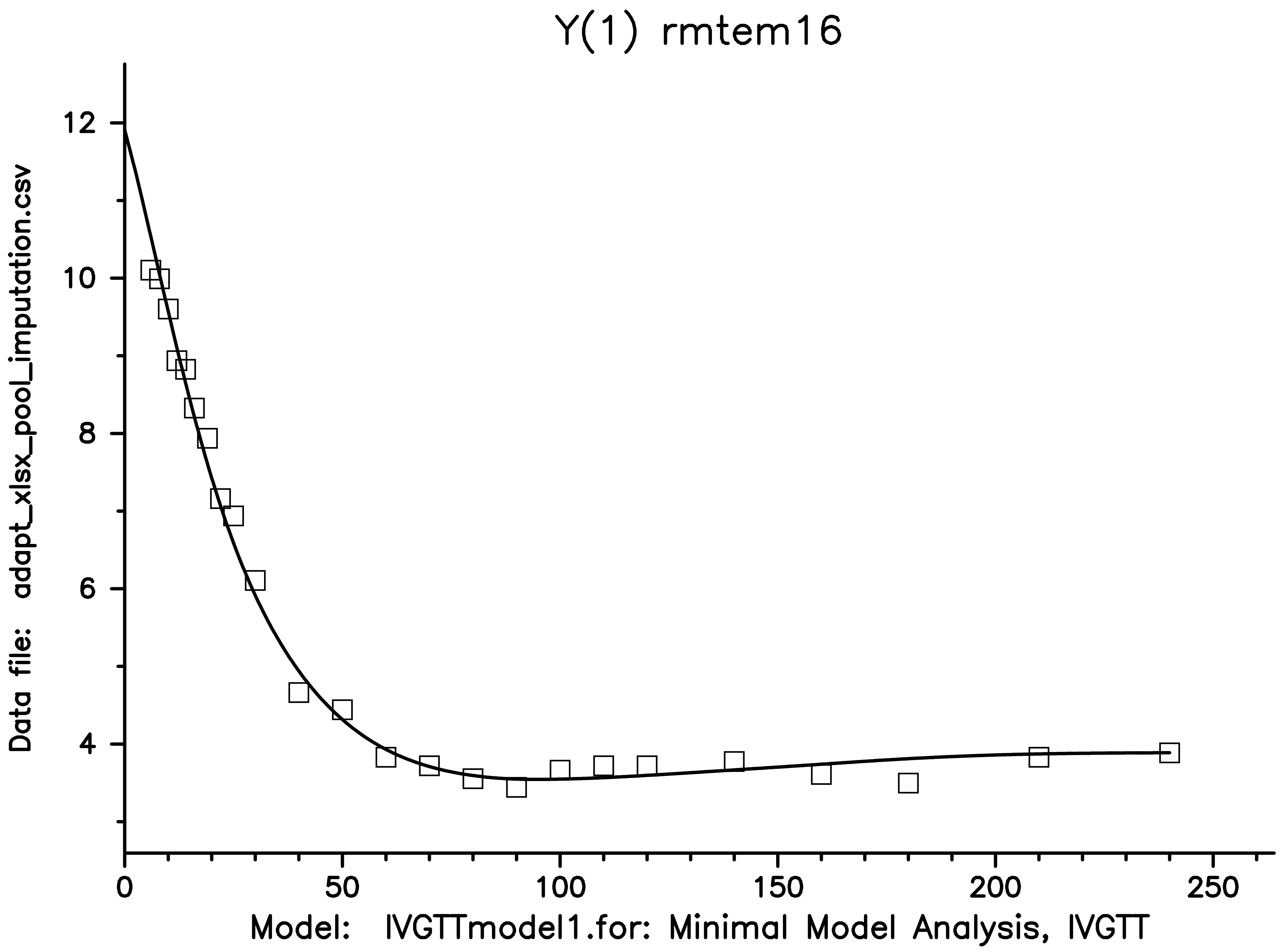
Y(1) rmtem16
Data file: adapt_xlsx_pool_imputation.csv
12
10
8
6
4
0
50
100
150
200
250
Model: IVGTTmodel1.for: Minimal Model Analysis, IVGTT

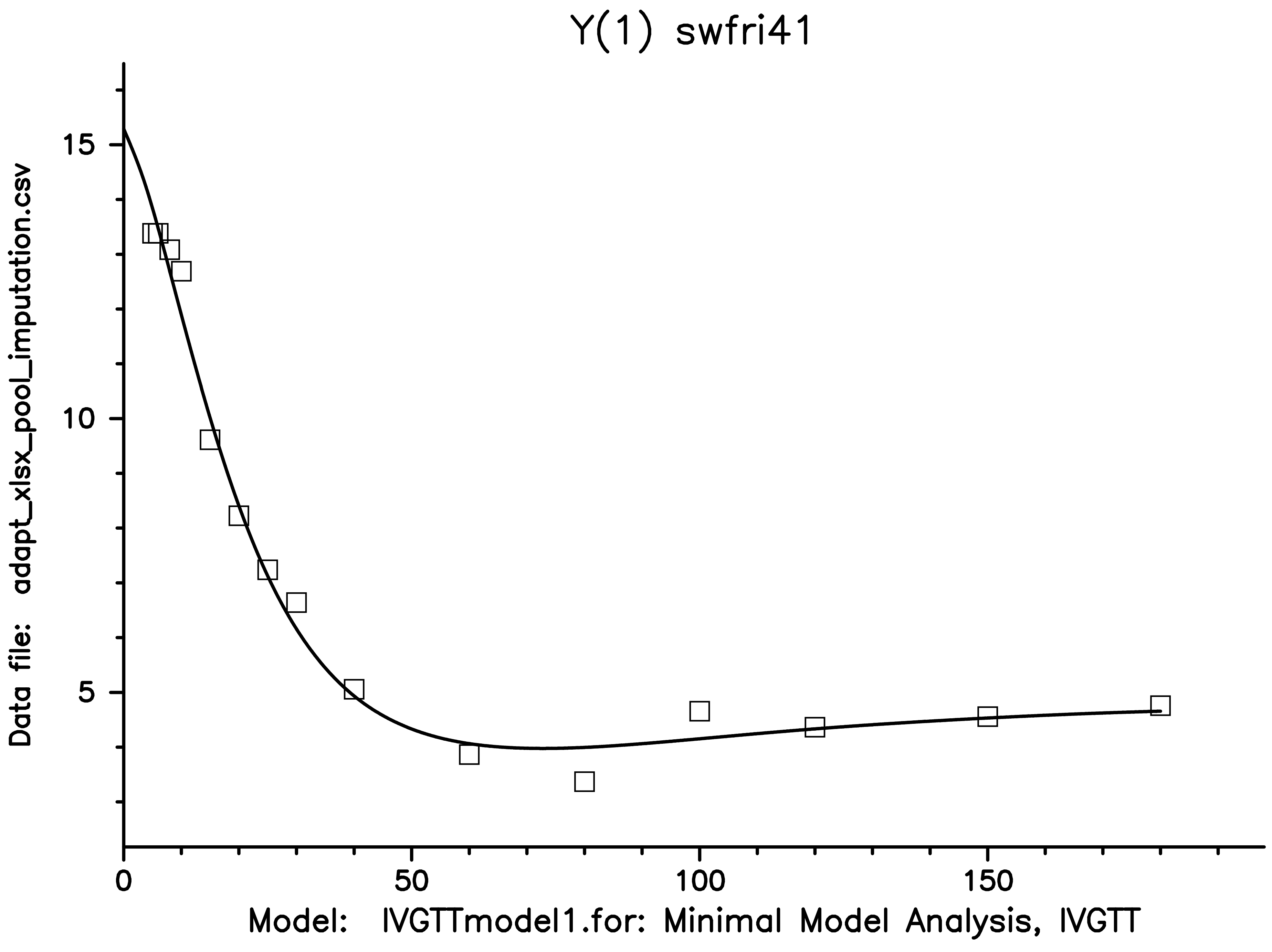
Y(1) swfri41
15
10
5
0
50
100
150
Data file: adapt_xlsx_pool_imputation.csv
Model: IVGTTmodel1.for: Minimal Model Analysis, IVGTT

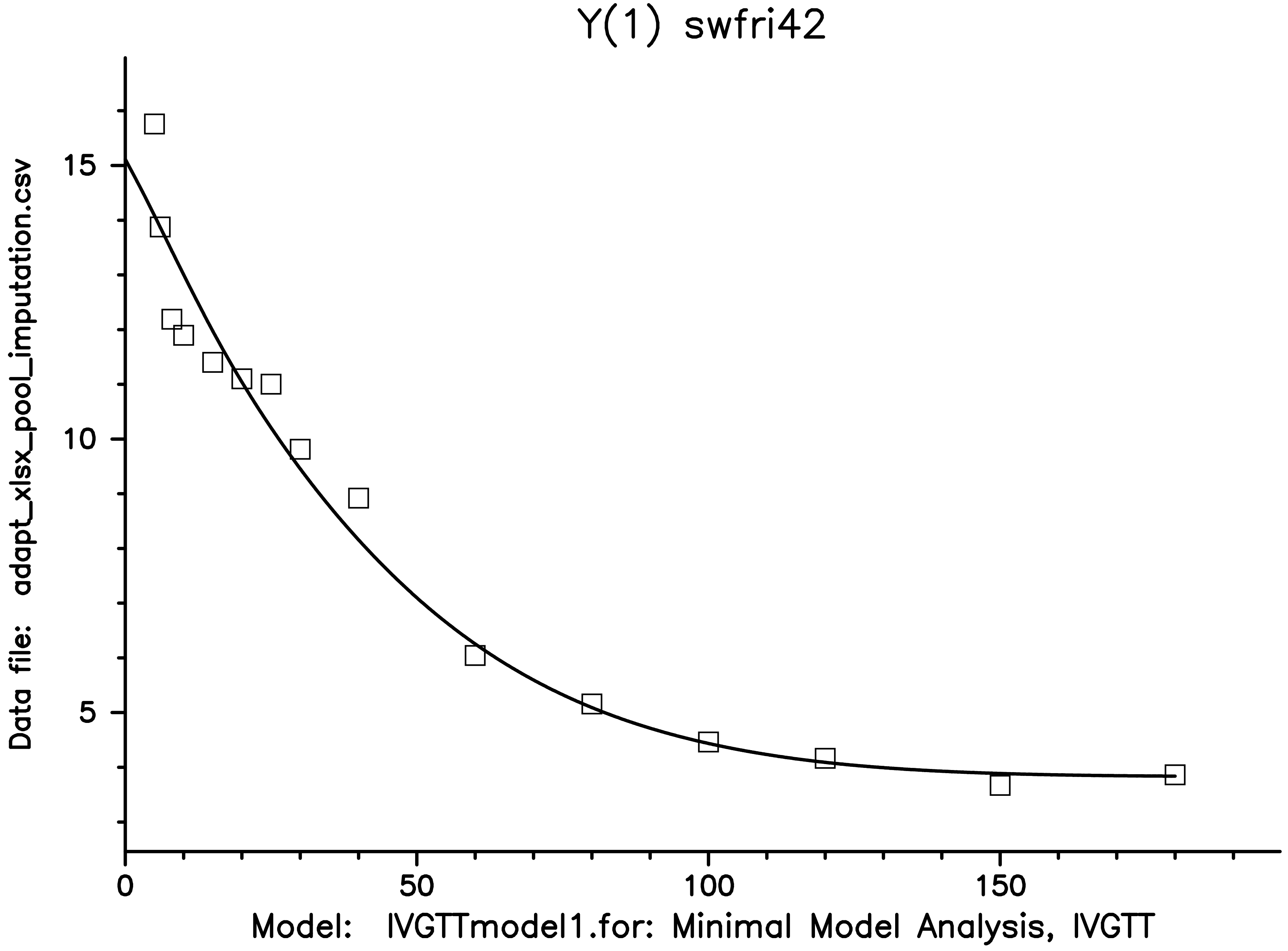
Y(1) swfri42
Data file: adapt_xlsx_pool_imputation.csv
Model: IVGTTmodel1.for: Minimal Model Analysis, IVGTT
15
10
5
0
50
100
150

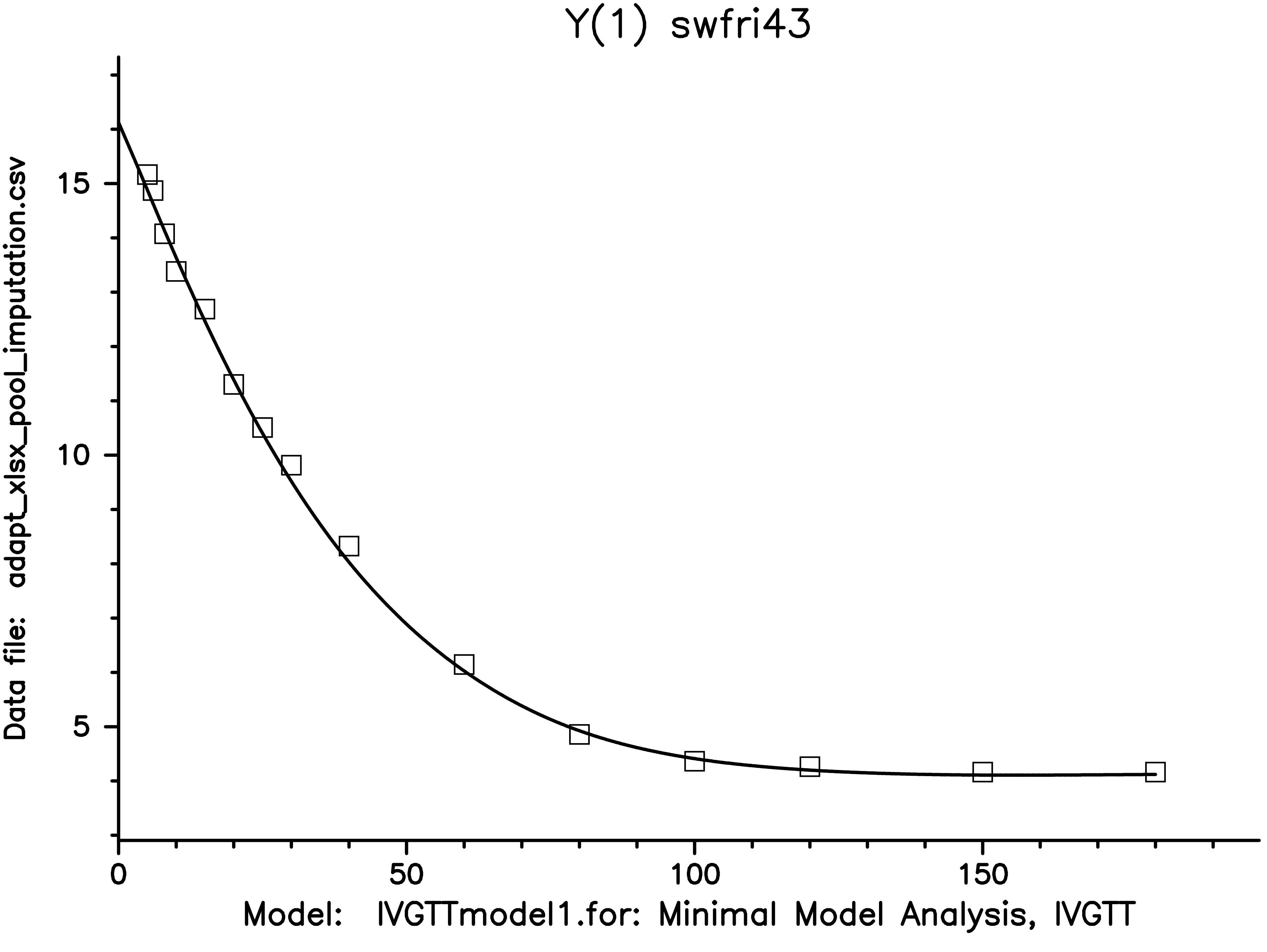

Y(1) swfri43
Data file: adapt_xlsx_pool_imputation.csv
15
10
5
0
50
100
150
Model: IVGTTmodel1.for: Minimal Model Analysis, IVGTT

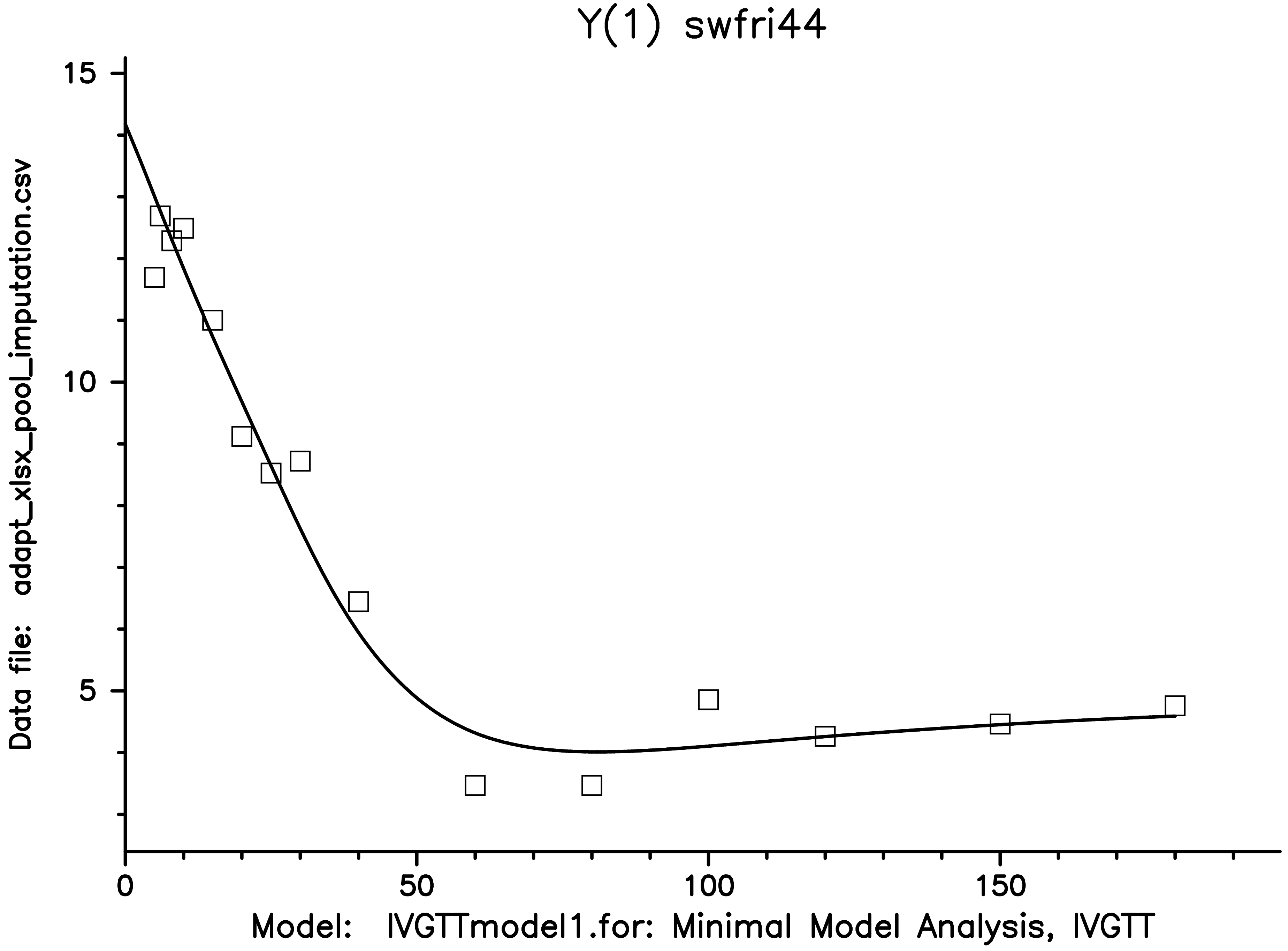

Y(1) swfri44
Data file: adapt_xlsx_pool_imputation.csv
15
10
5
0
50
100
150
Model: IVGTTmodel1.for: Minimal Model Analysis, IVGTT

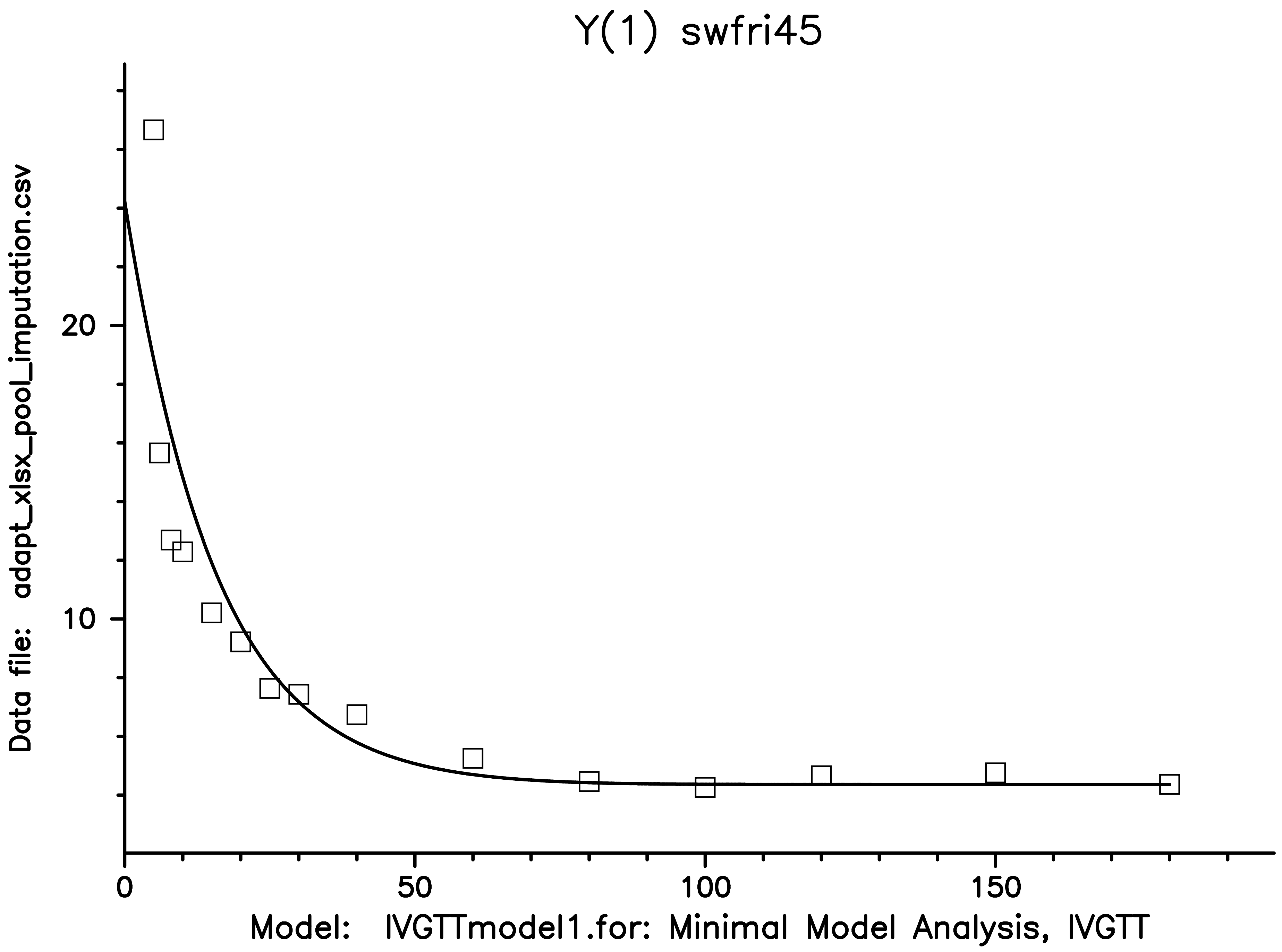
Y(1) swfri45
Data file: adapt_xlsx_pool_imputation.csv
20
10
0
50
100
150
Model: IVGTTmodel1.for: Minimal Model Analysis, IVGTT

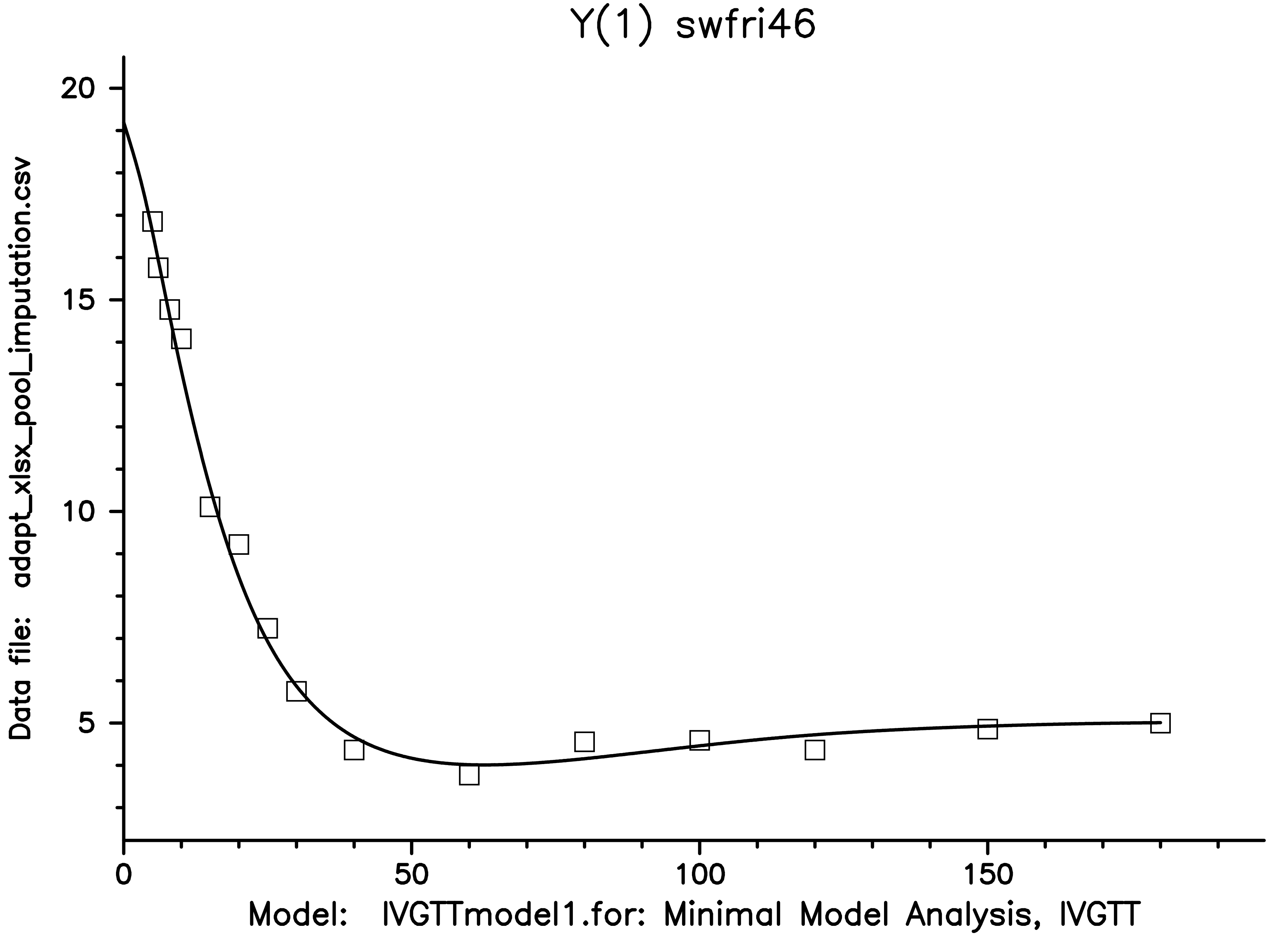

Y(1) swfri46
20
15
10
5
0
50
100
150
Data file: adapt_xlsx_pool_imputation.csv
Model: IVGTTmodel1.for: Minimal Model Analysis, IVGTT

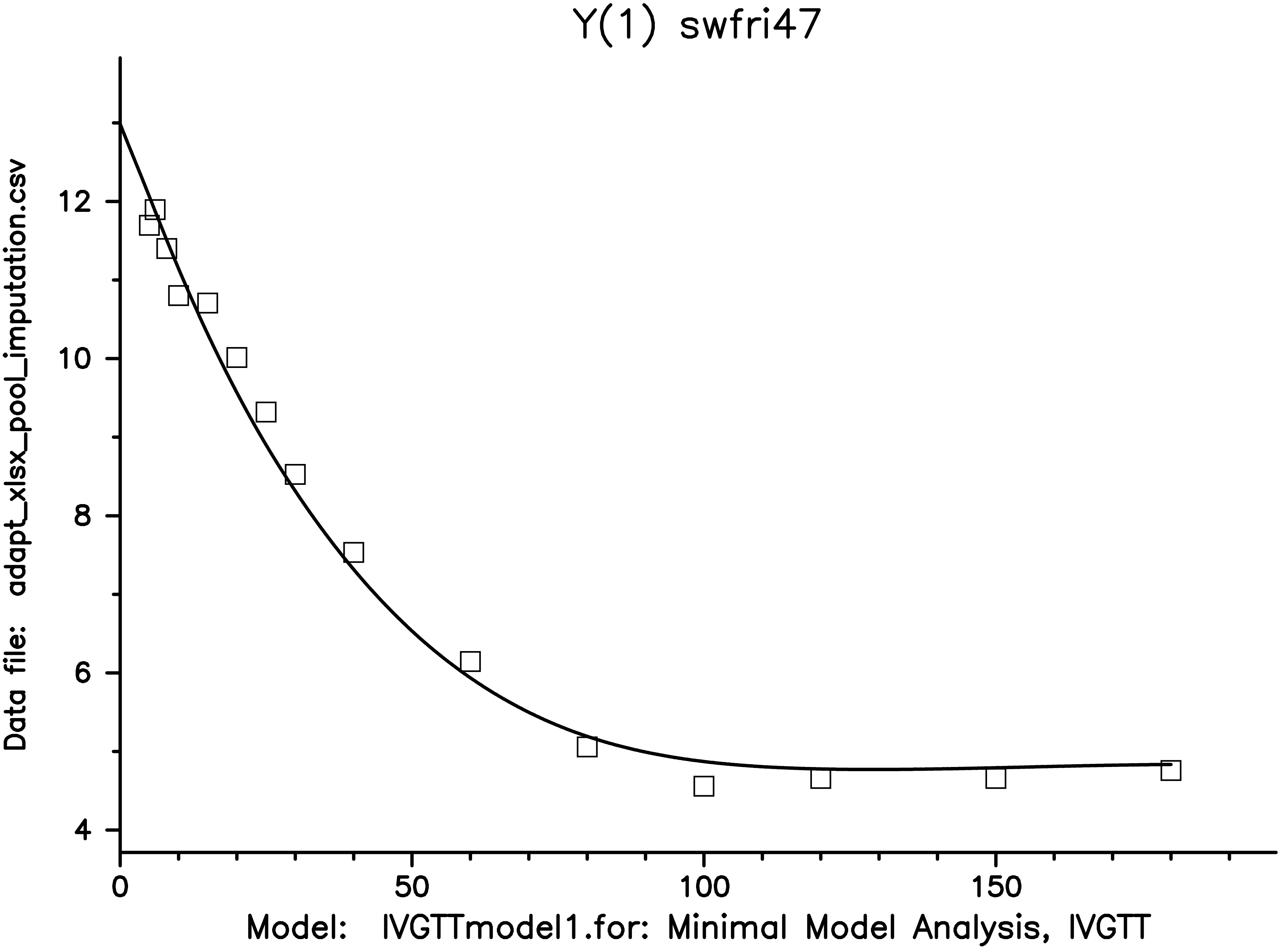

Y(1) swfri47
Data file: adapt_xlsx_pool_imputation.csv
Model: IVGTTmodel1.for: Minimal Model Analysis, IVGTT
12
10
8
6
4
0
50
100
150

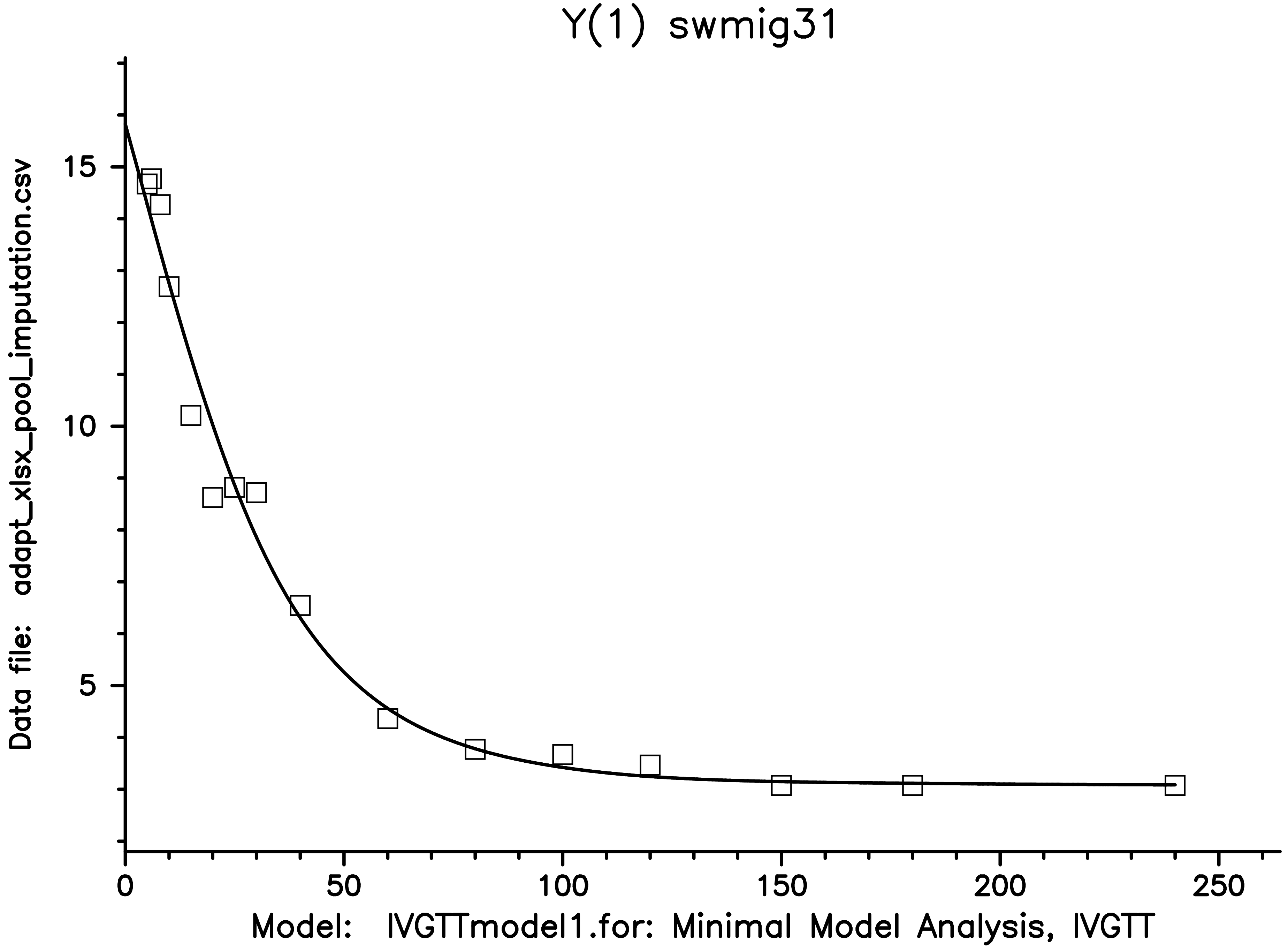

Y(1) swmig31
Data file: adapt_xlsx_pool_imputation.csv
Model: IVGTTmodel1.for: Minimal Model Analysis, IVGTT
15
10
5
0
50
100
150
200
250

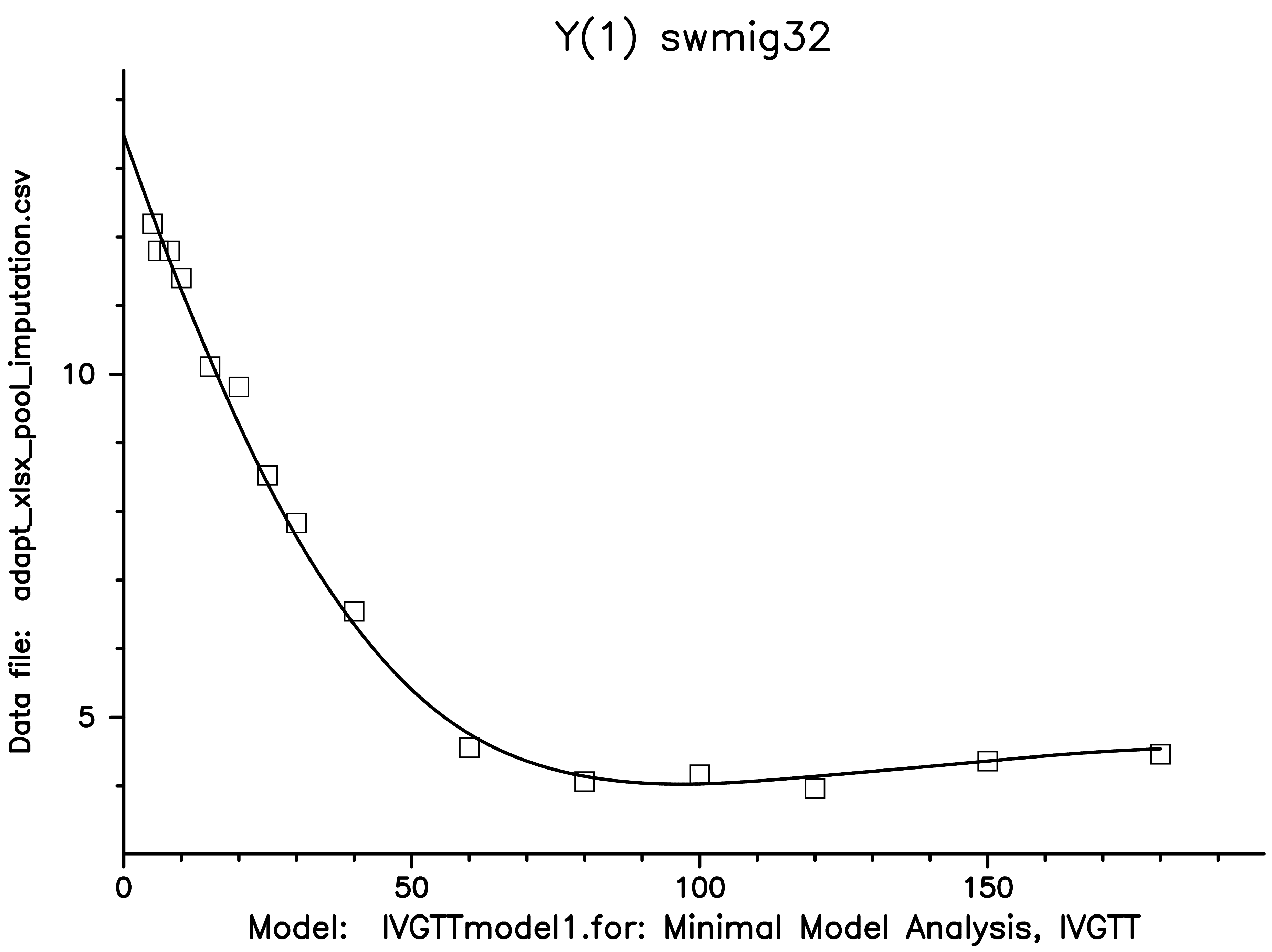

Y(1) swmig32
Data file: adapt_xlsx_pool_imputation.csv
10
5
0
50
100
150
Model: IVGTTmodel1.for: Minimal Model Analysis, IVGTT

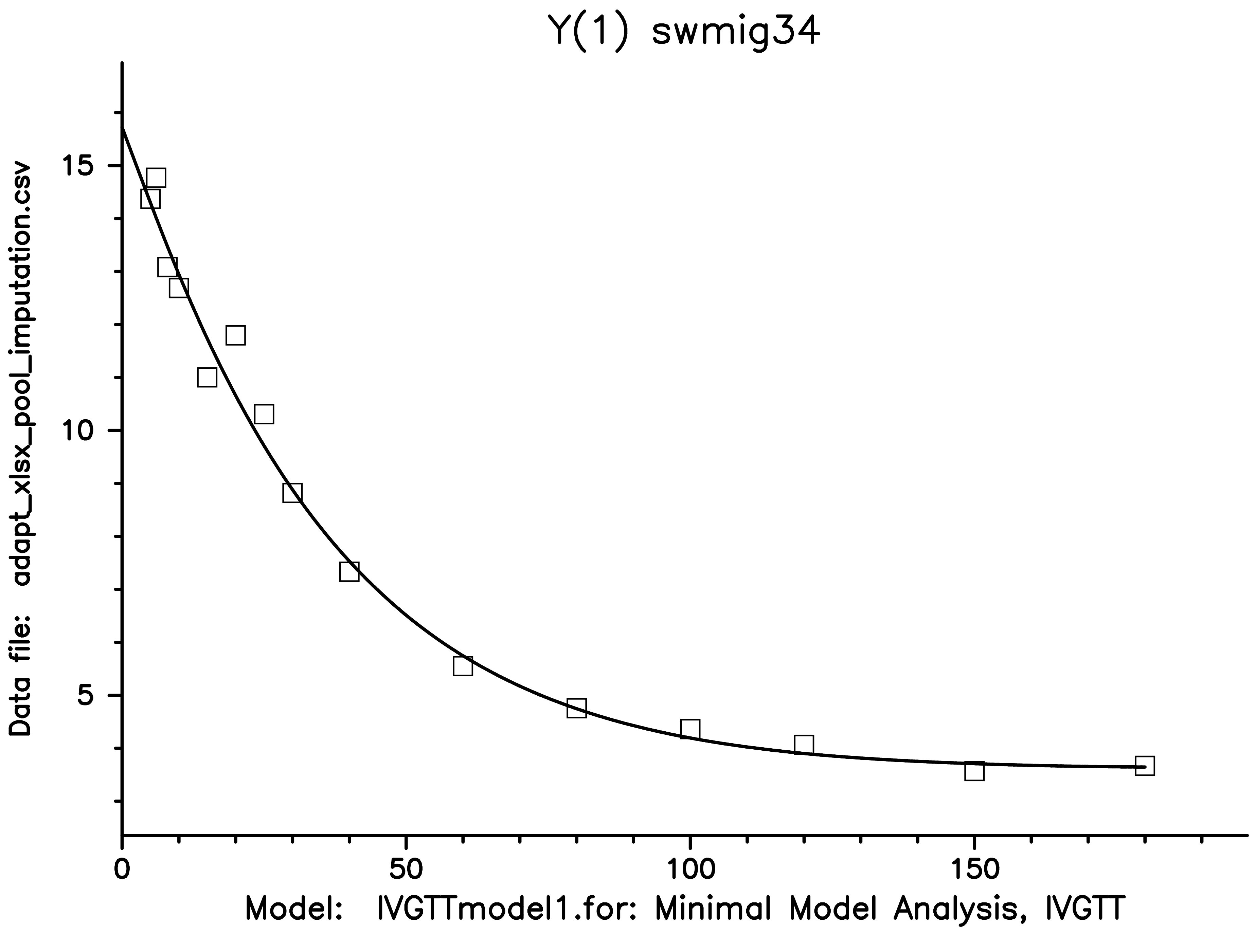
Y(1) swmig34
Data file: adapt_xlsx_pool_imputation.csv
15
10
5
0
50
100
150
Model: IVGTTmodel1.for: Minimal Model Analysis, IVGTT

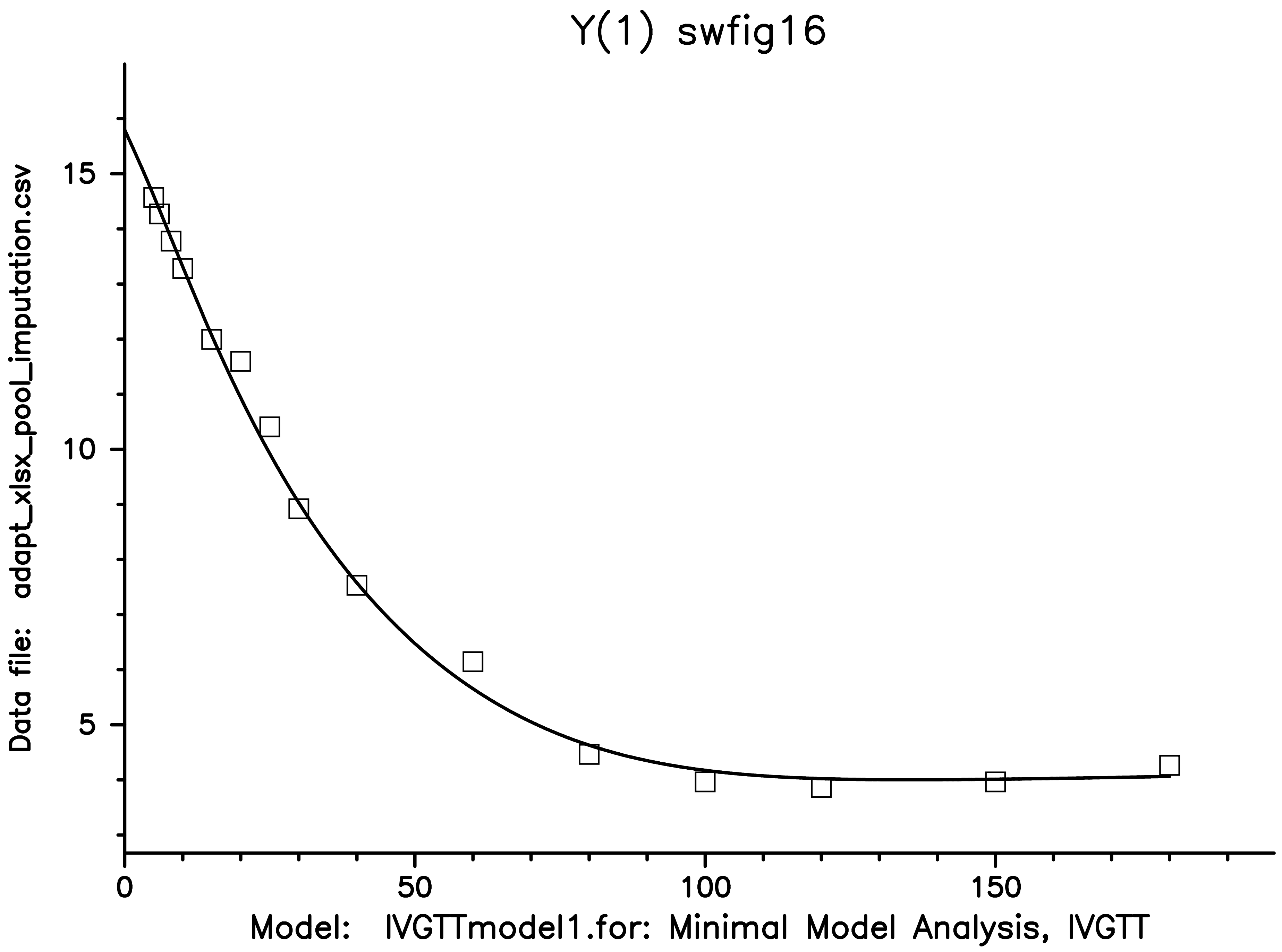
Y(1) swfig16
Data file: adapt_xlsx_pool_imputation.csv
15
10
5
0
50
100
150
Model: IVGTTmodel1.for: Minimal Model Analysis, IVGTT

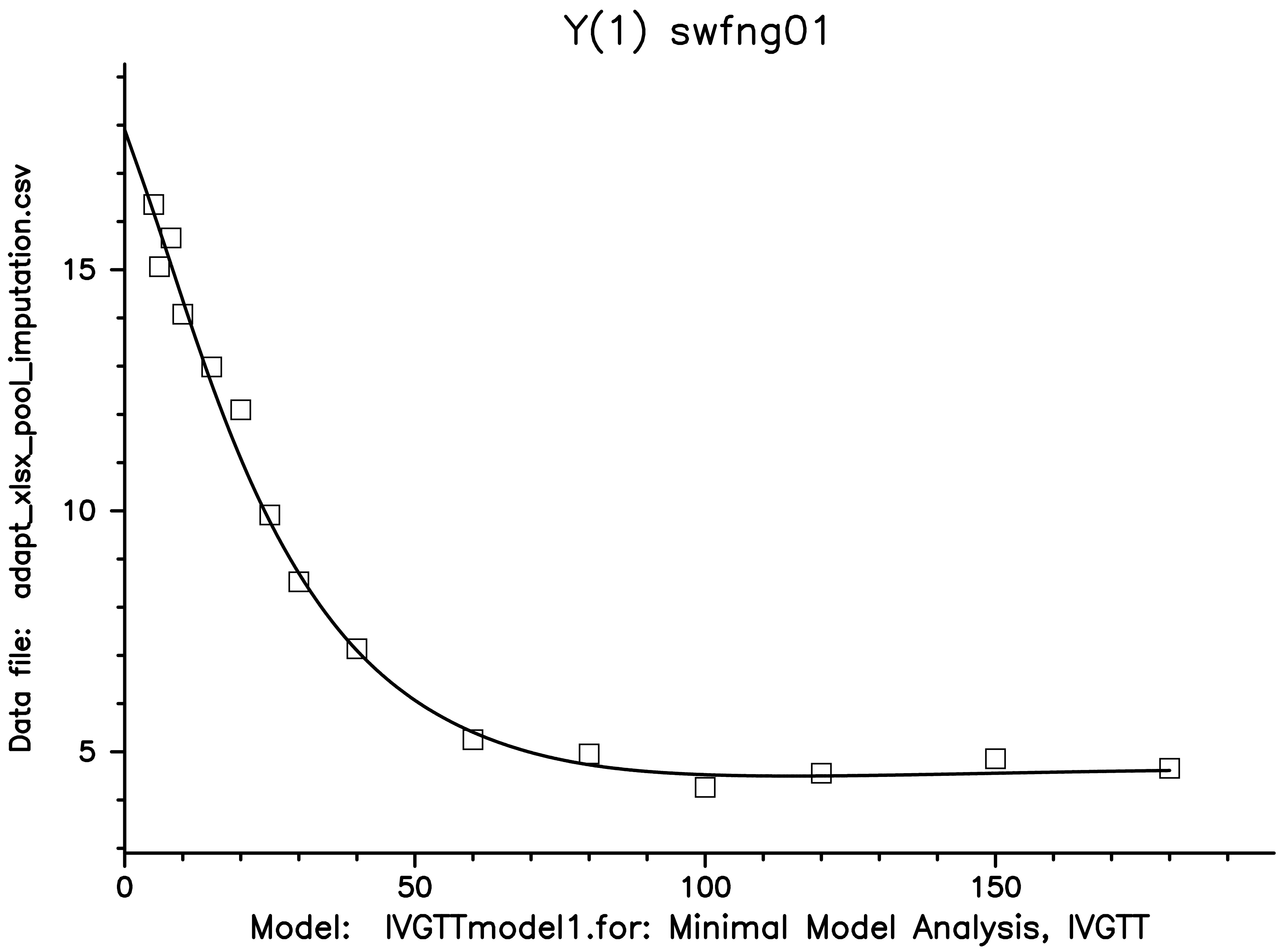

Y(1) swfng01
Data file: adapt_xlsx_pool_imputation.csv
Model: IVGTTmodel1.for: Minimal Model Analysis, IVGTT
0
50
100
150
5
10
15

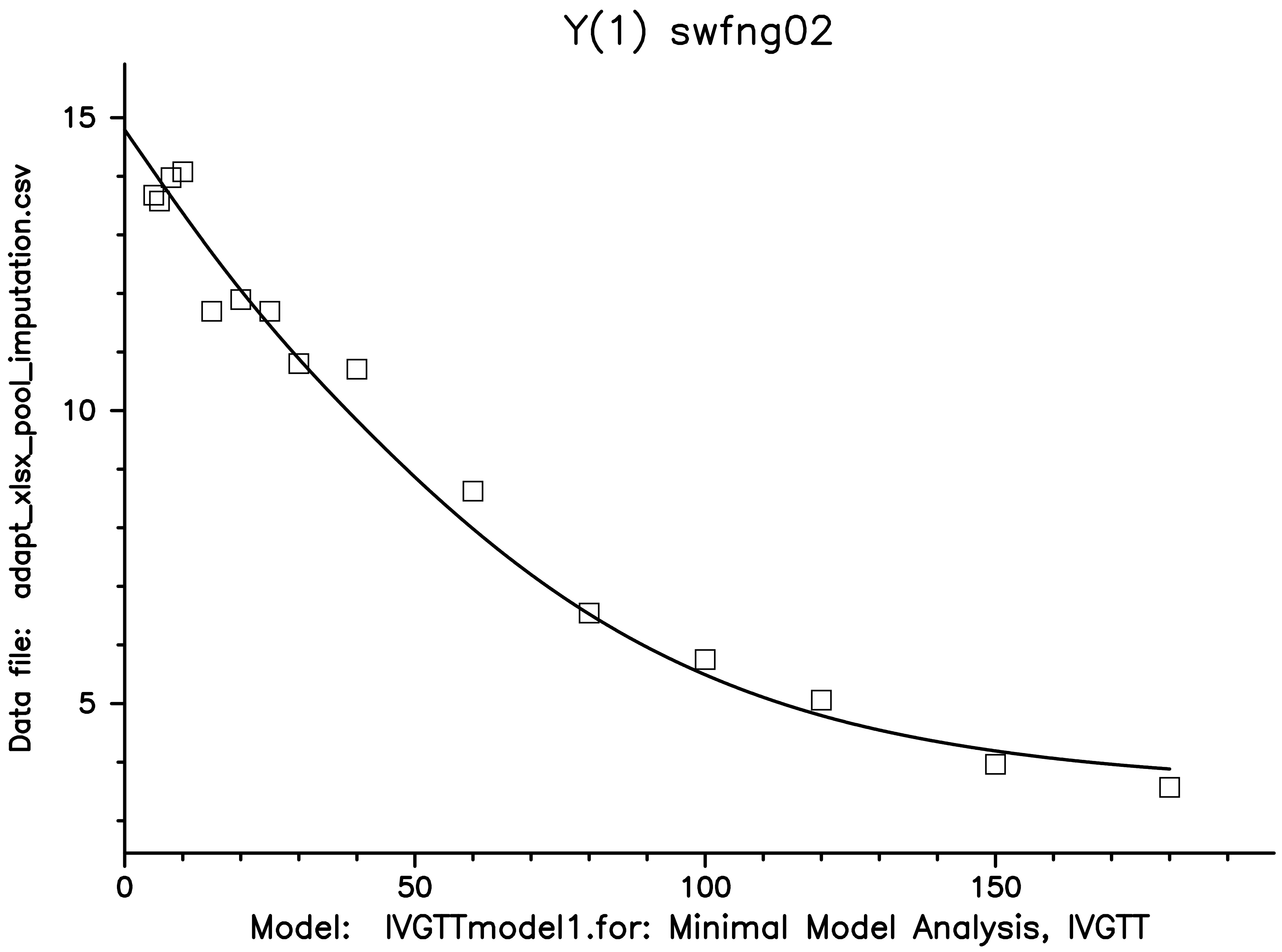
Y(1) swfng02
Data file: adapt_xlsx_pool_imputation.csv
15
10
5
0
50
100
150
Model: IVGTTmodel1.for: Minimal Model Analysis, IVGTT

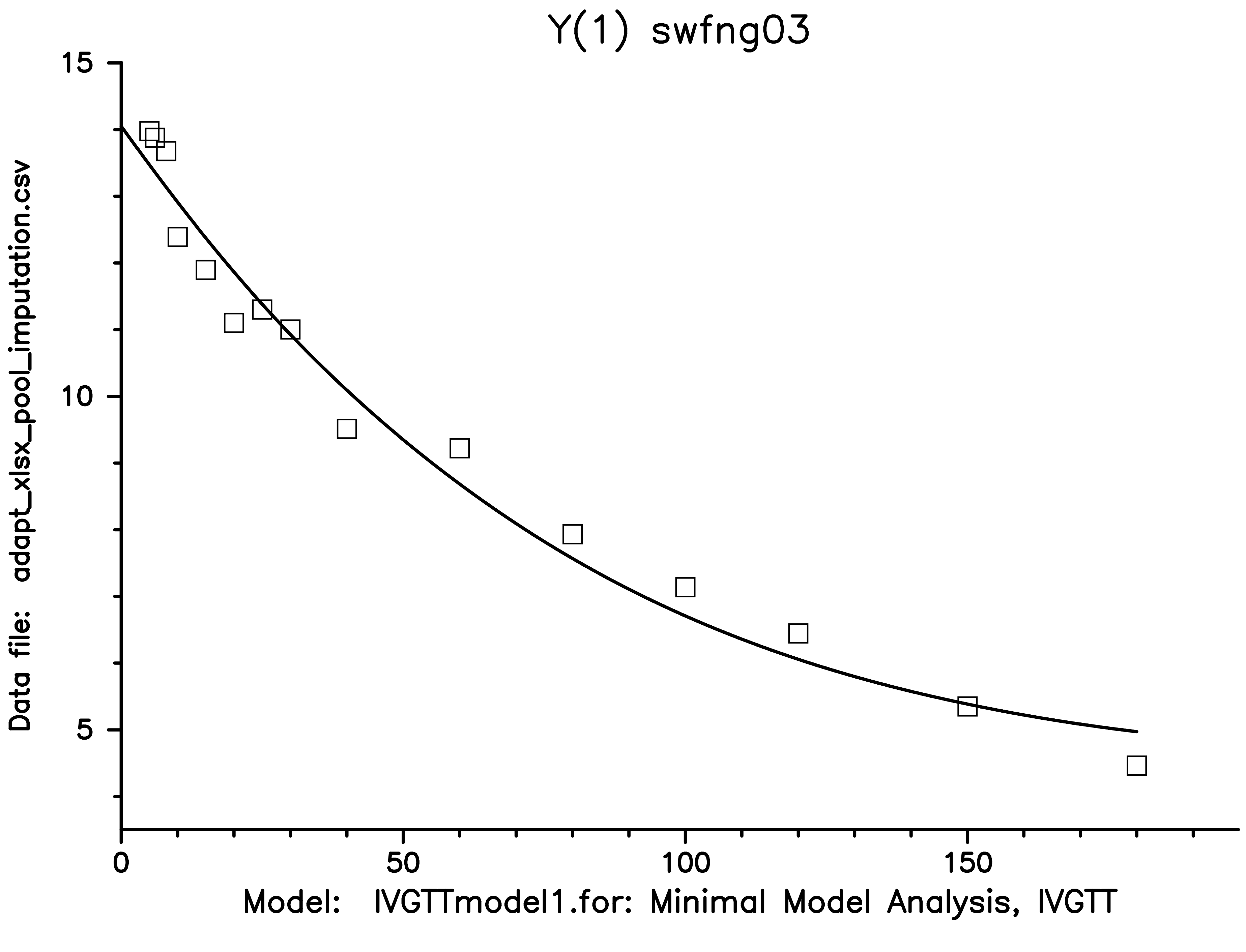
Y(1) swfng03
15
10
5
0
50
100
150
Data file: adapt_xlsx_pool_imputation.csv
Model: IVGTTmodel1.for: Minimal Model Analysis, IVGTT

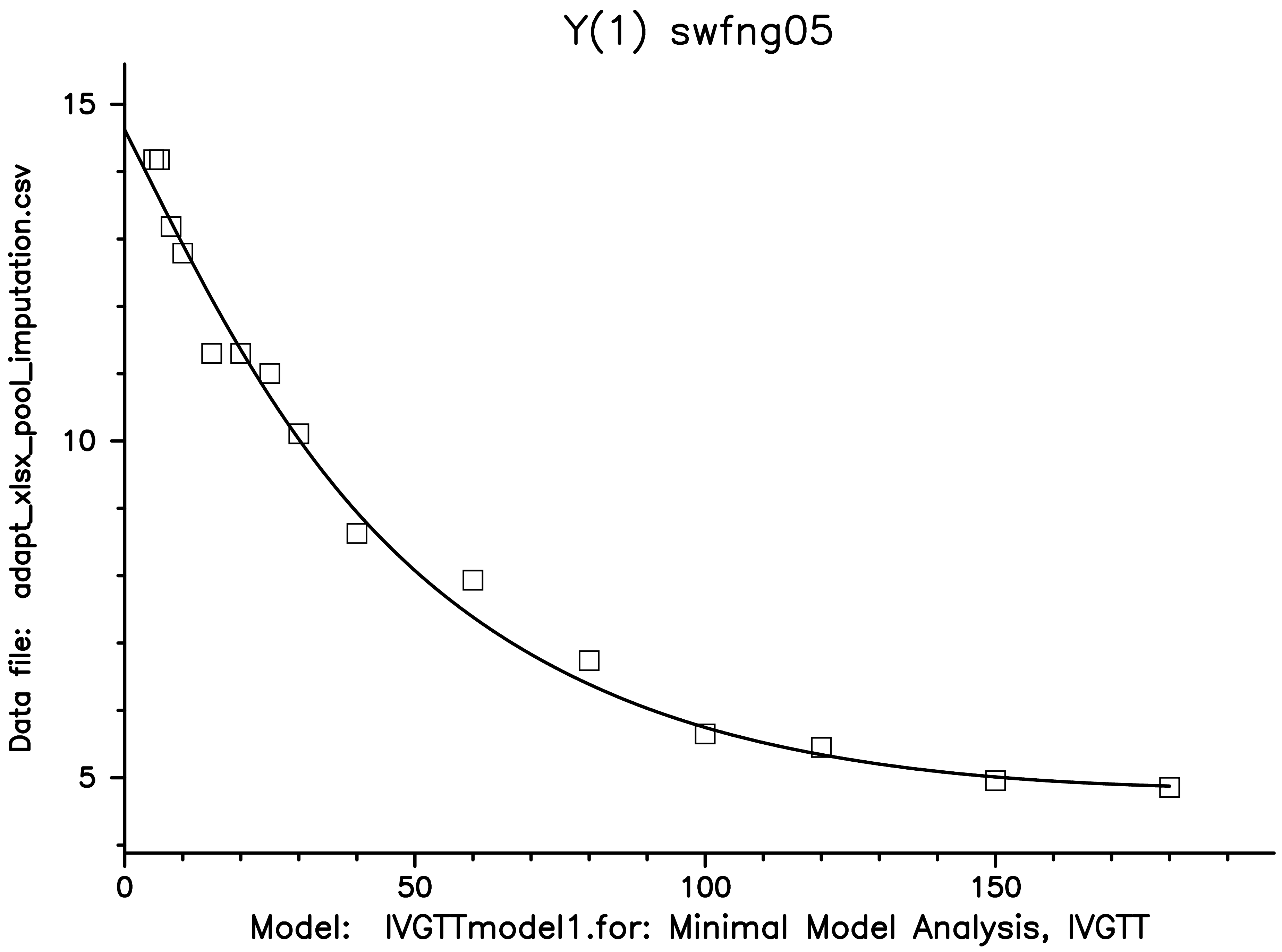

Y(1) swfng05
Data file: adapt_xlsx_pool_imputation.csv
15
10
5
0
50
100
150
Model: IVGTTmodel1.for: Minimal Model Analysis, IVGTT

Y(1) swfng06

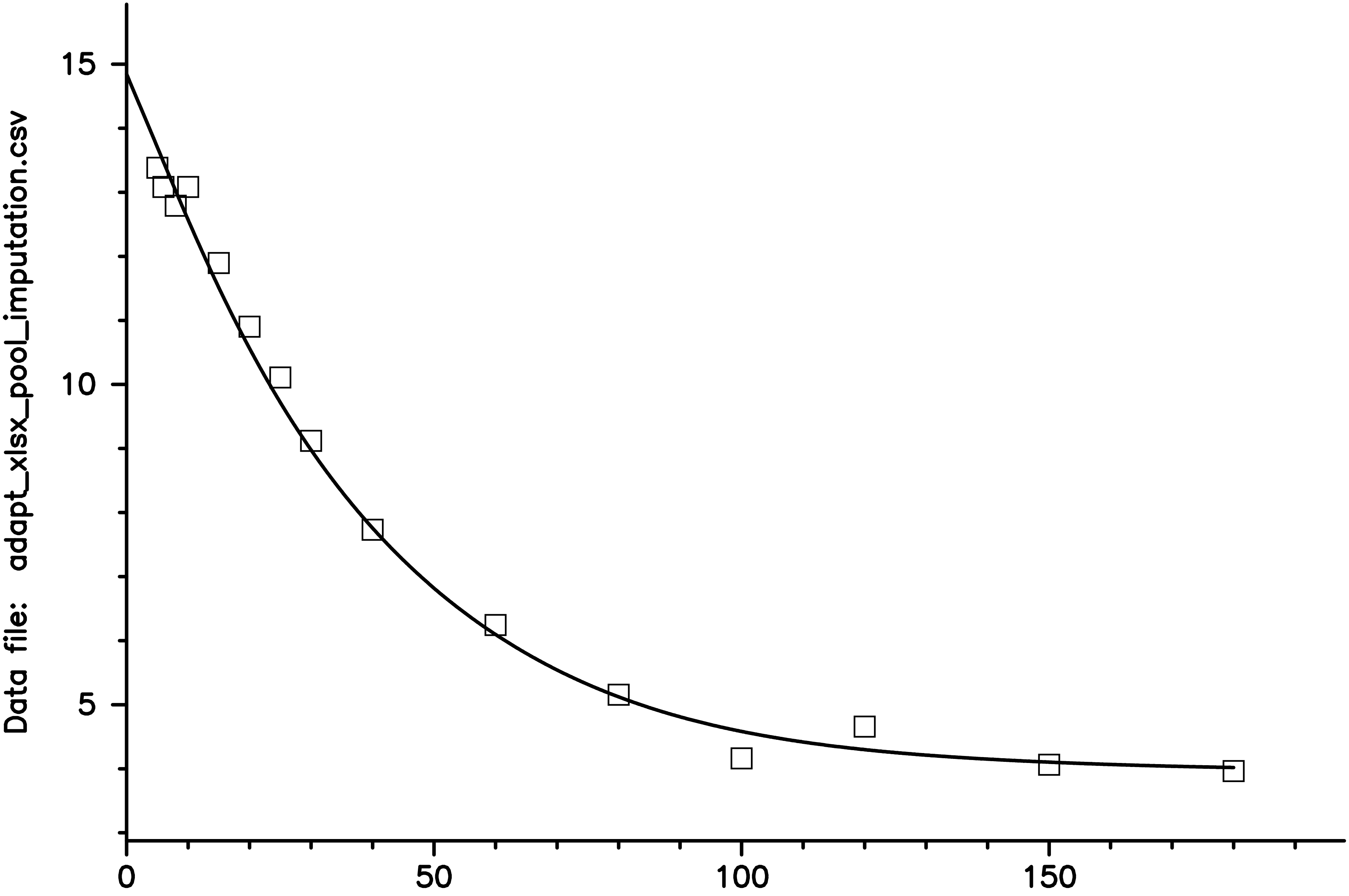

Model: IVGTTmodel1.for: Minimal Model Analysis, IVGTT

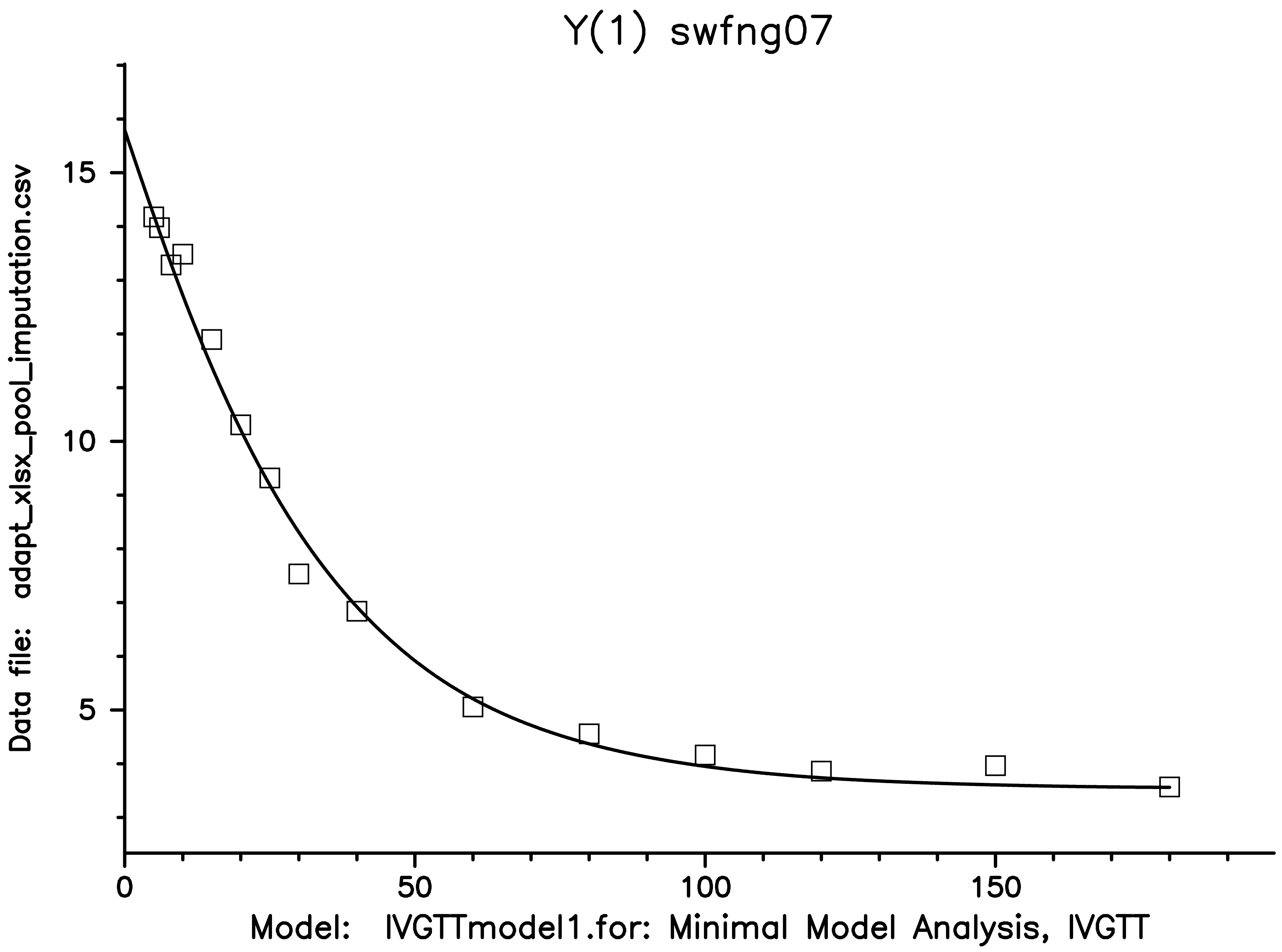
Y(1) swfng07
Data file: adapt_xlsx_pool_imputation.csv
15
10
5
0
50
100
150
Model: IVGTTmodel1.for: Minimal Model Analysis, IVGTT

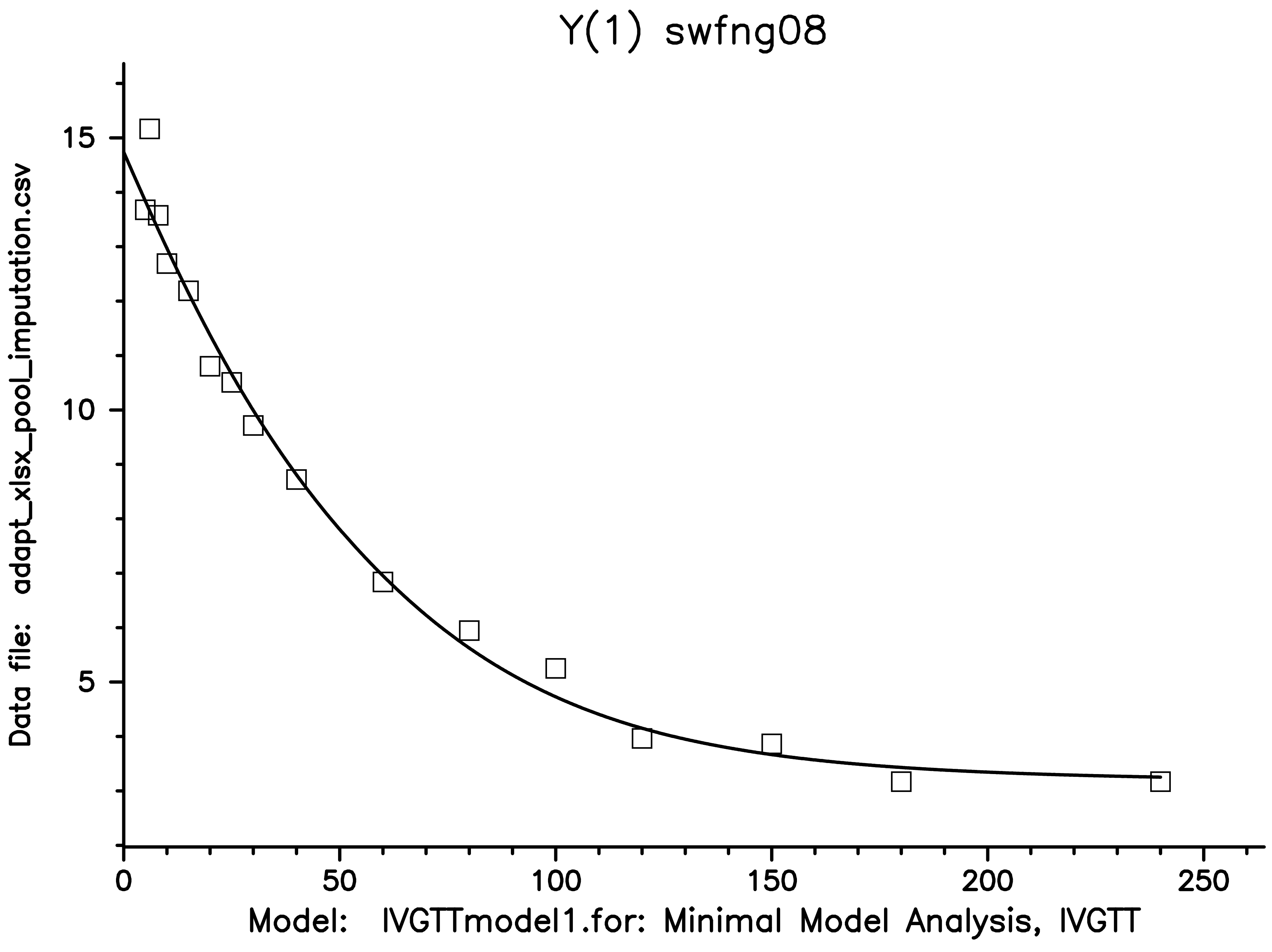
Y(1) swfng08
Data file: adapt_xlsx_pool_imputation.csv
15
10
5
0
50
100
150
200
250
Model: IVGTTmodel1.for: Minimal Model Analysis, IVGTT

Y(1) swfng09

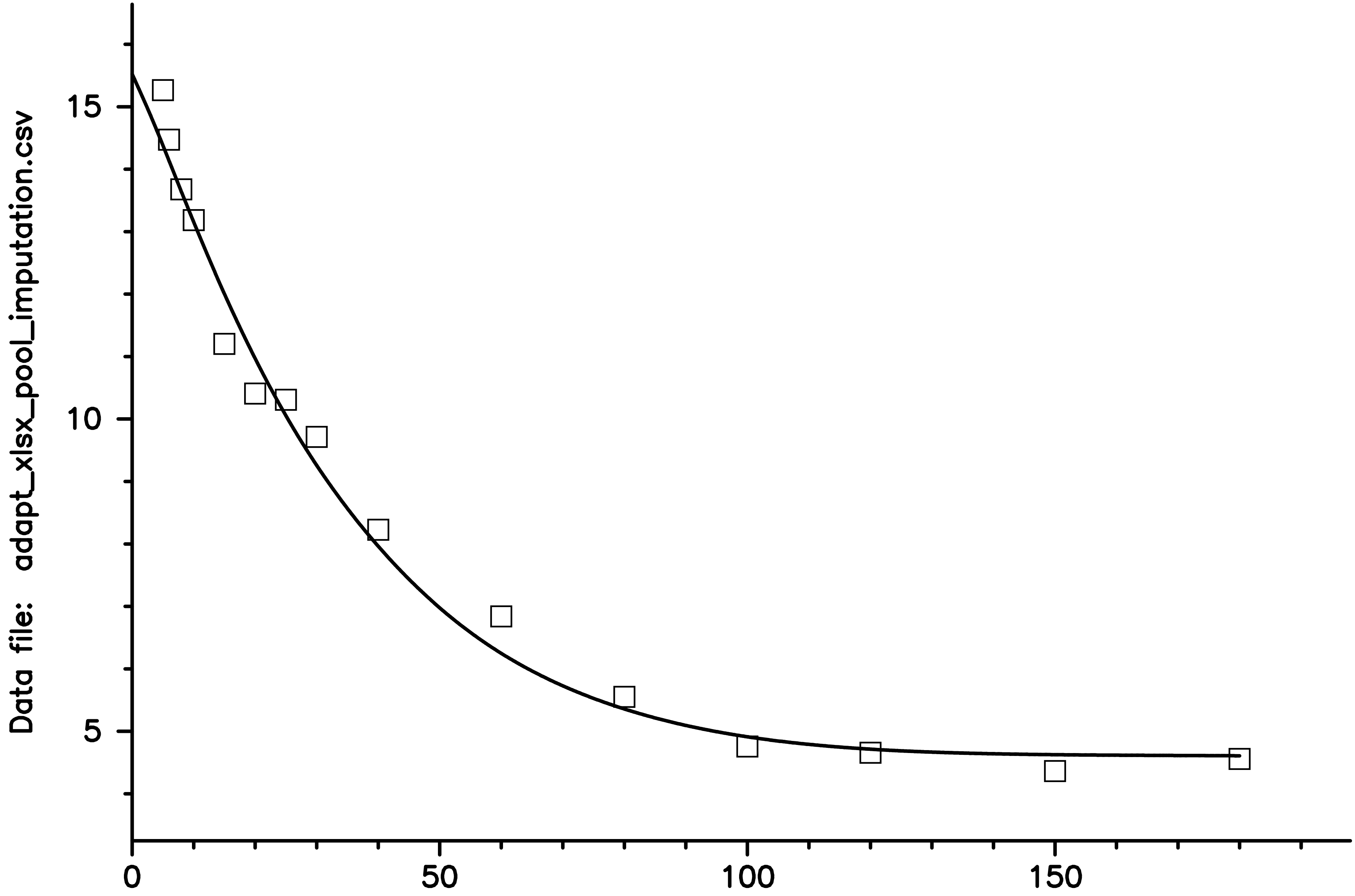

Model: IVGTTmodel1.for: Minimal Model Analysis, IVGTT

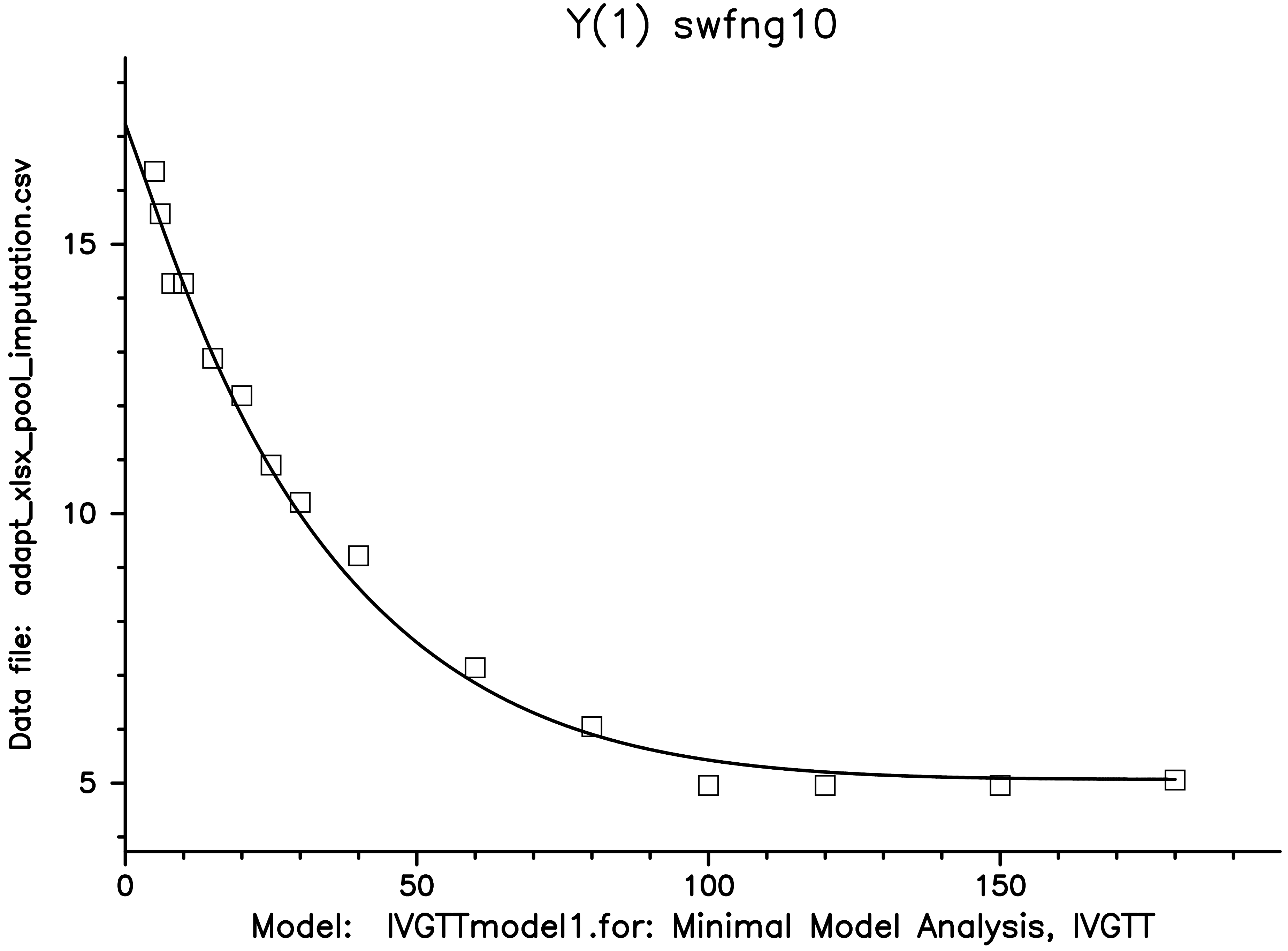
Y(1) swfng10
Data file: adapt_xlsx_pool_imputation.csv
15
10
5
0
50
100
150
Model: IVGTTmodel1.for: Minimal Model Analysis, IVGTT

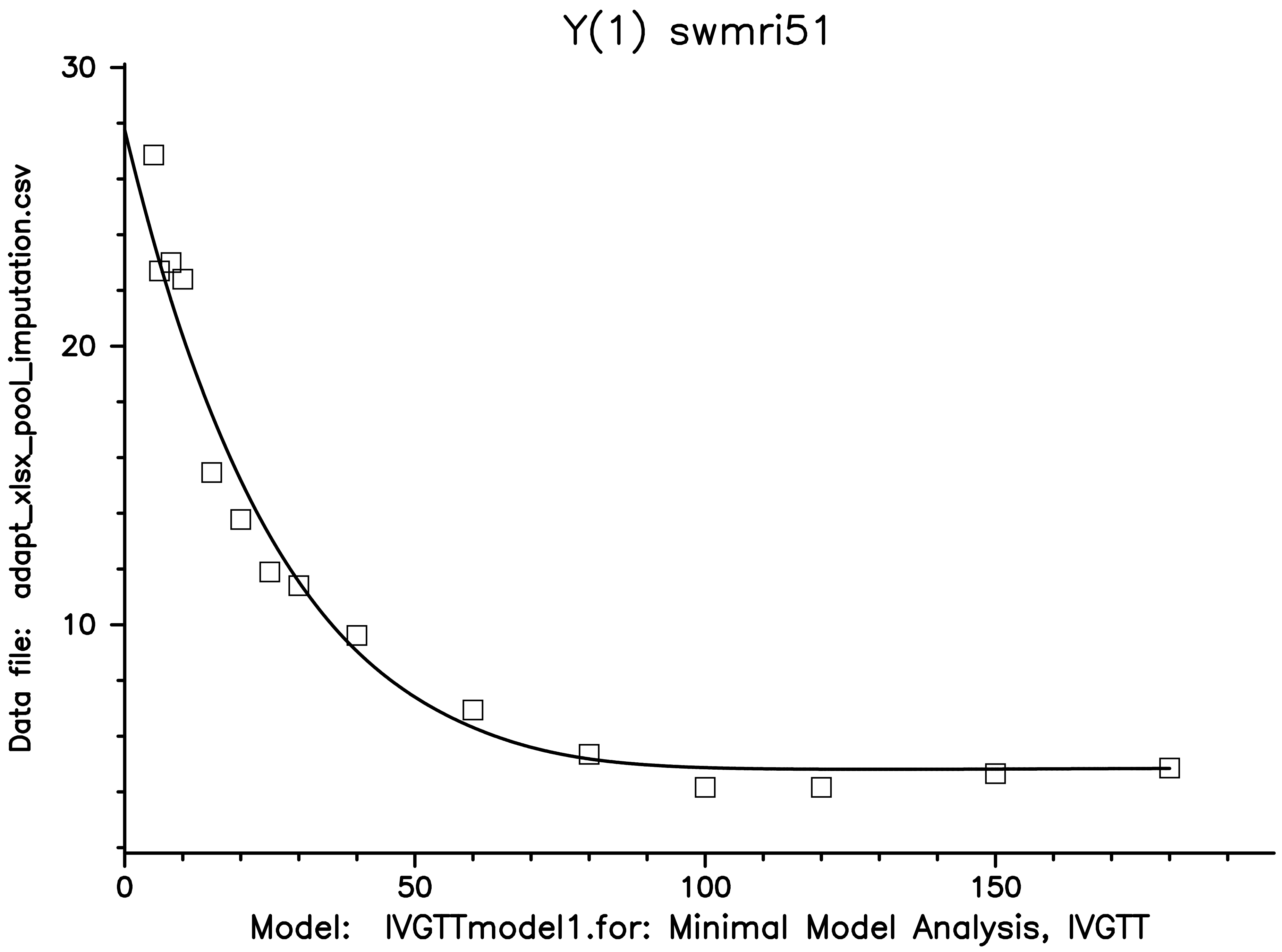

Y(1) swmri51
30
20
10
0
50
100
150
Data file: adapt_xlsx_pool_imputation.csv
Model: IVGTTmodel1.for: Minimal Model Analysis, IVGTT

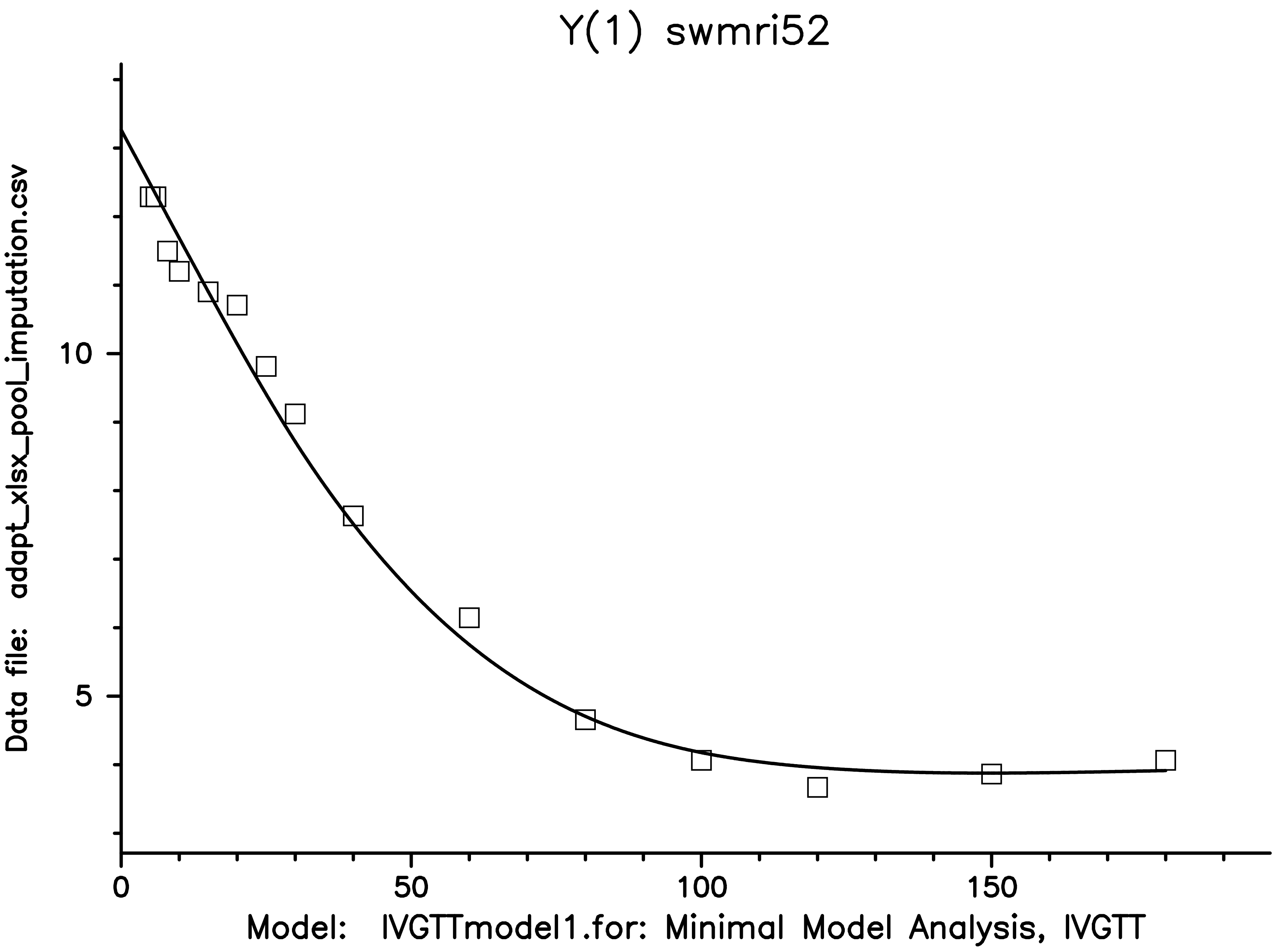

Y(1) swmri52
Data file: adapt_xlsx_pool_imputation.csv
10
5
0
50
100
150
Model: IVGTTmodel1.for: Minimal Model Analysis, IVGTT

Y(1) swmri53

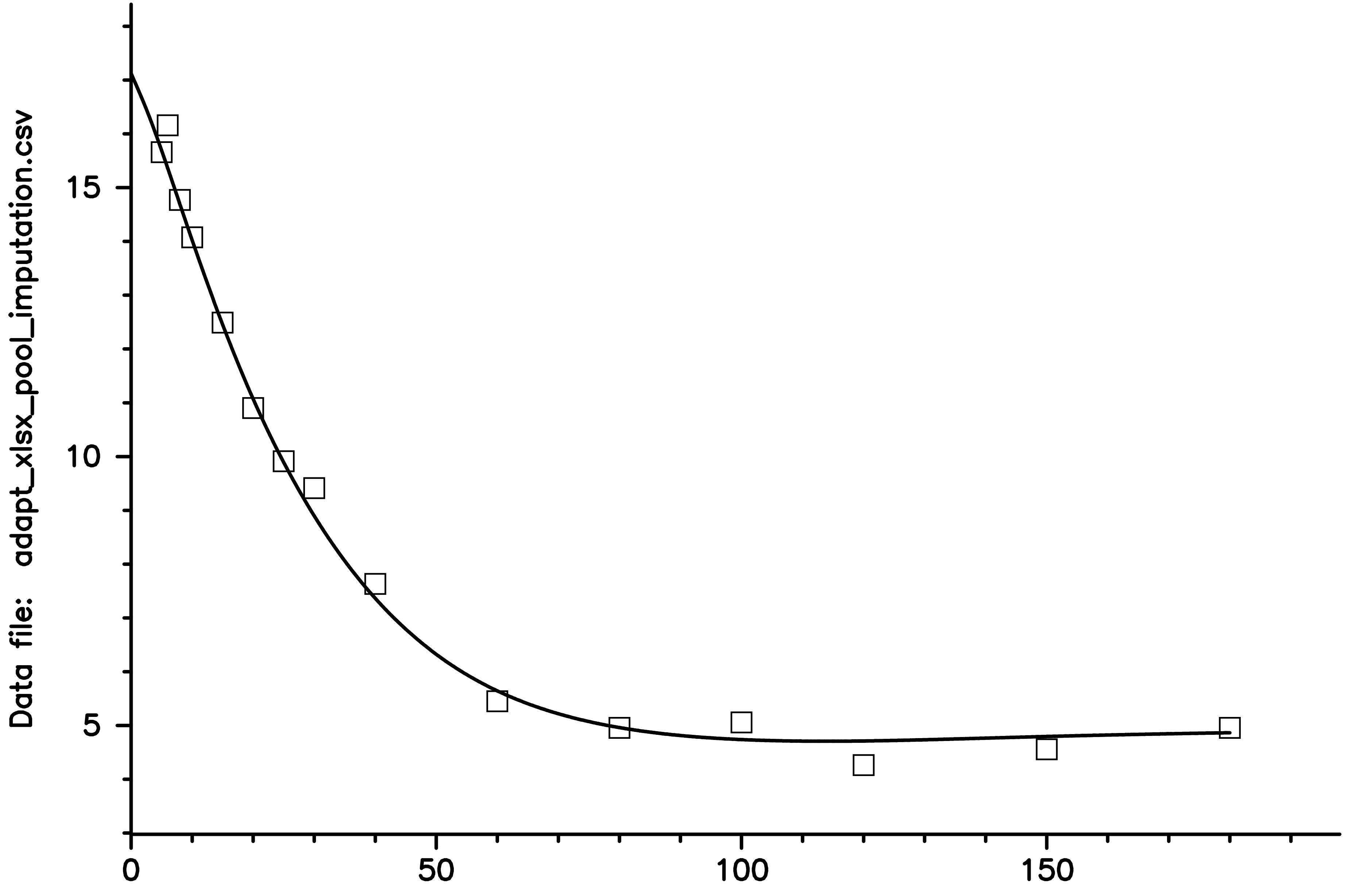

Model: IVGTTmodel1.for: Minimal Model Analysis, IVGTT

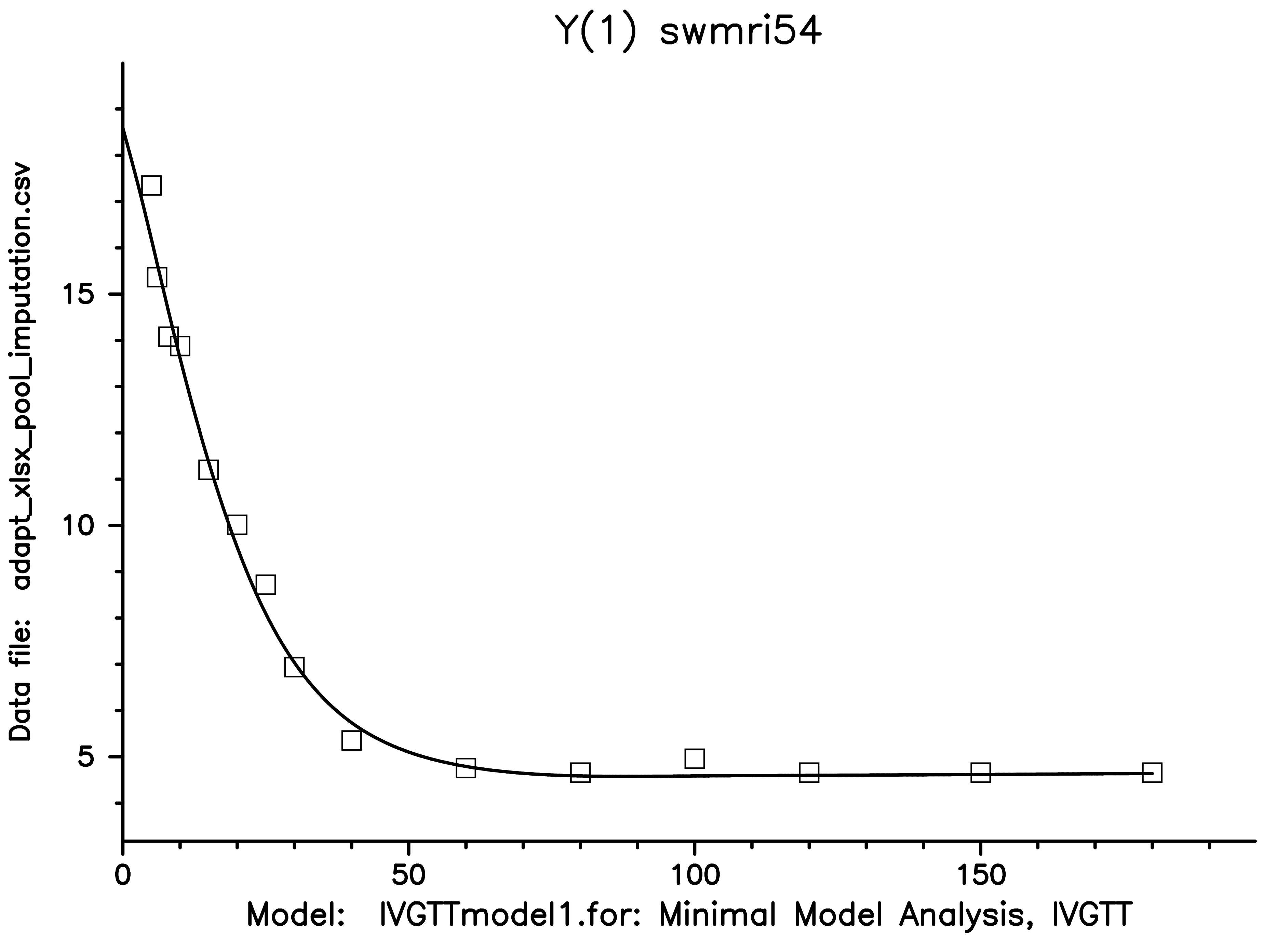

Y(1) swmri54
Data file: adapt_xlsx_pool_imputation.csv
15
10
5
0
50
100
150
Model: IVGTTmodel1.for: Minimal Model Analysis, IVGTT

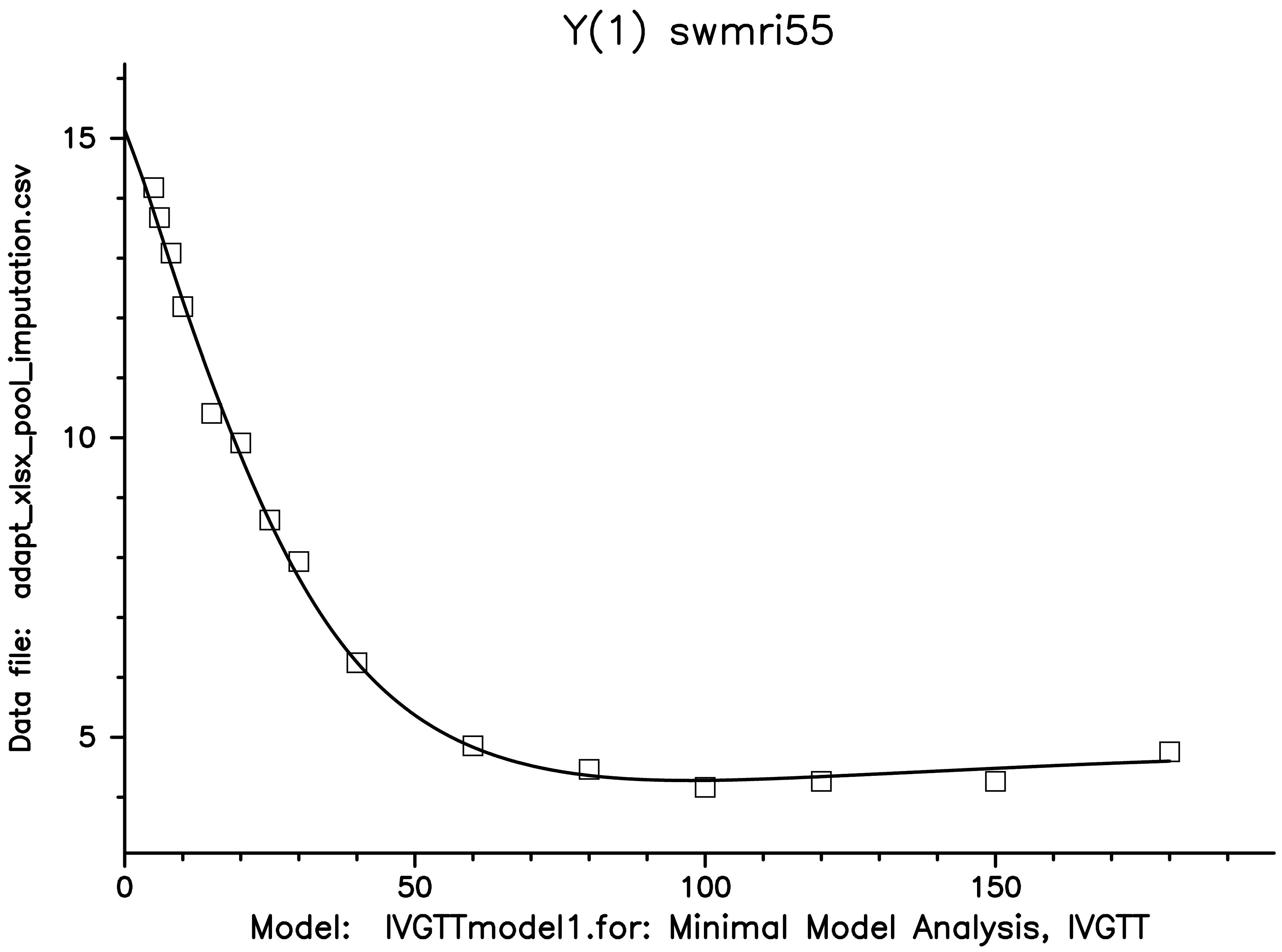
Y(1) swmri55
Data file: adapt_xlsx_pool_imputation.csv
15
10
5
0
50
100
150
Model: IVGTTmodel1.for: Minimal Model Analysis, IVGTT

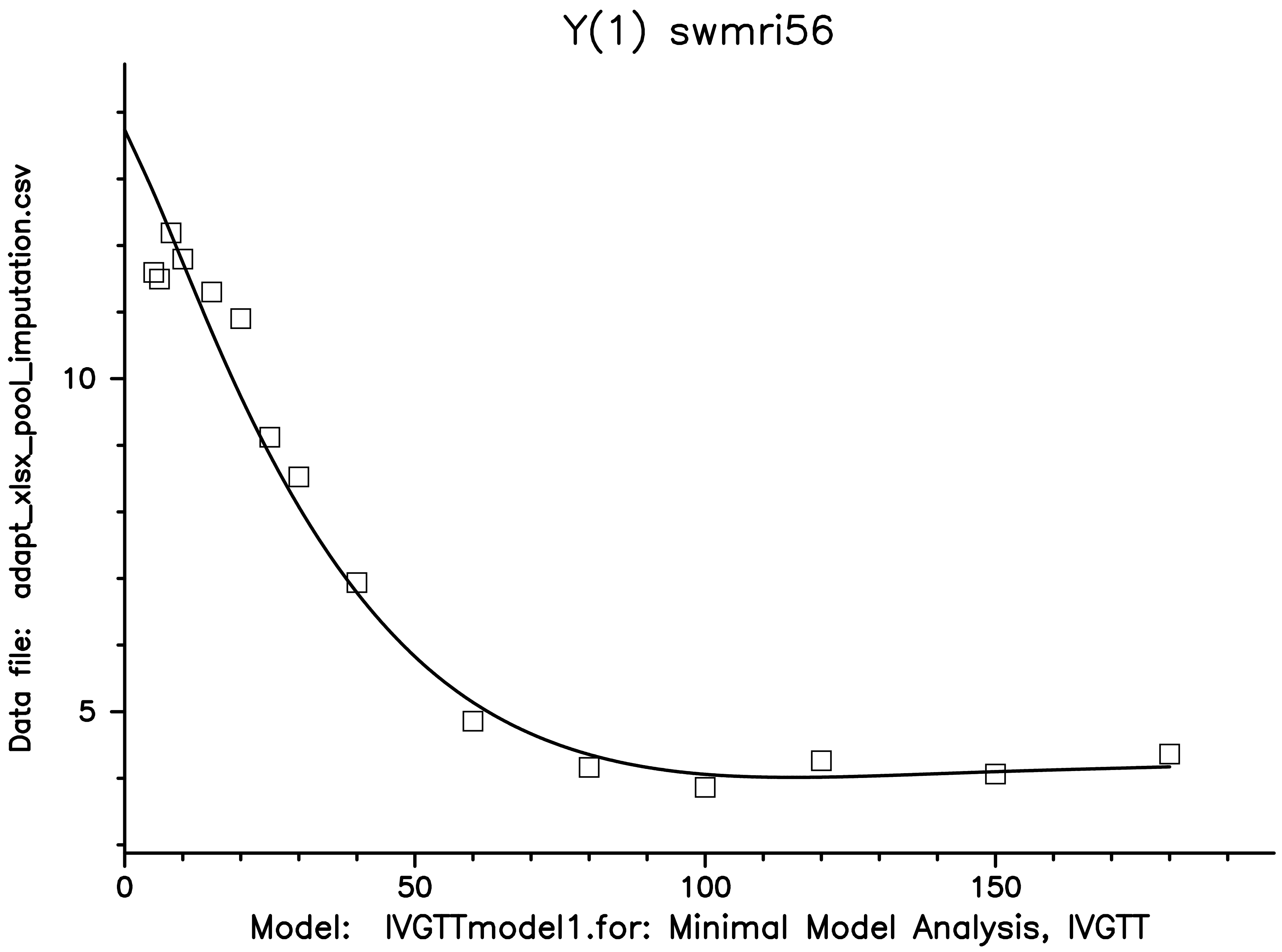

Y(1) swmri56
Data file: adapt_xlsx_pool_imputation.csv
10
5
0
50
100
150
Model: IVGTTmodel1.for: Minimal Model Analysis, IVGTT

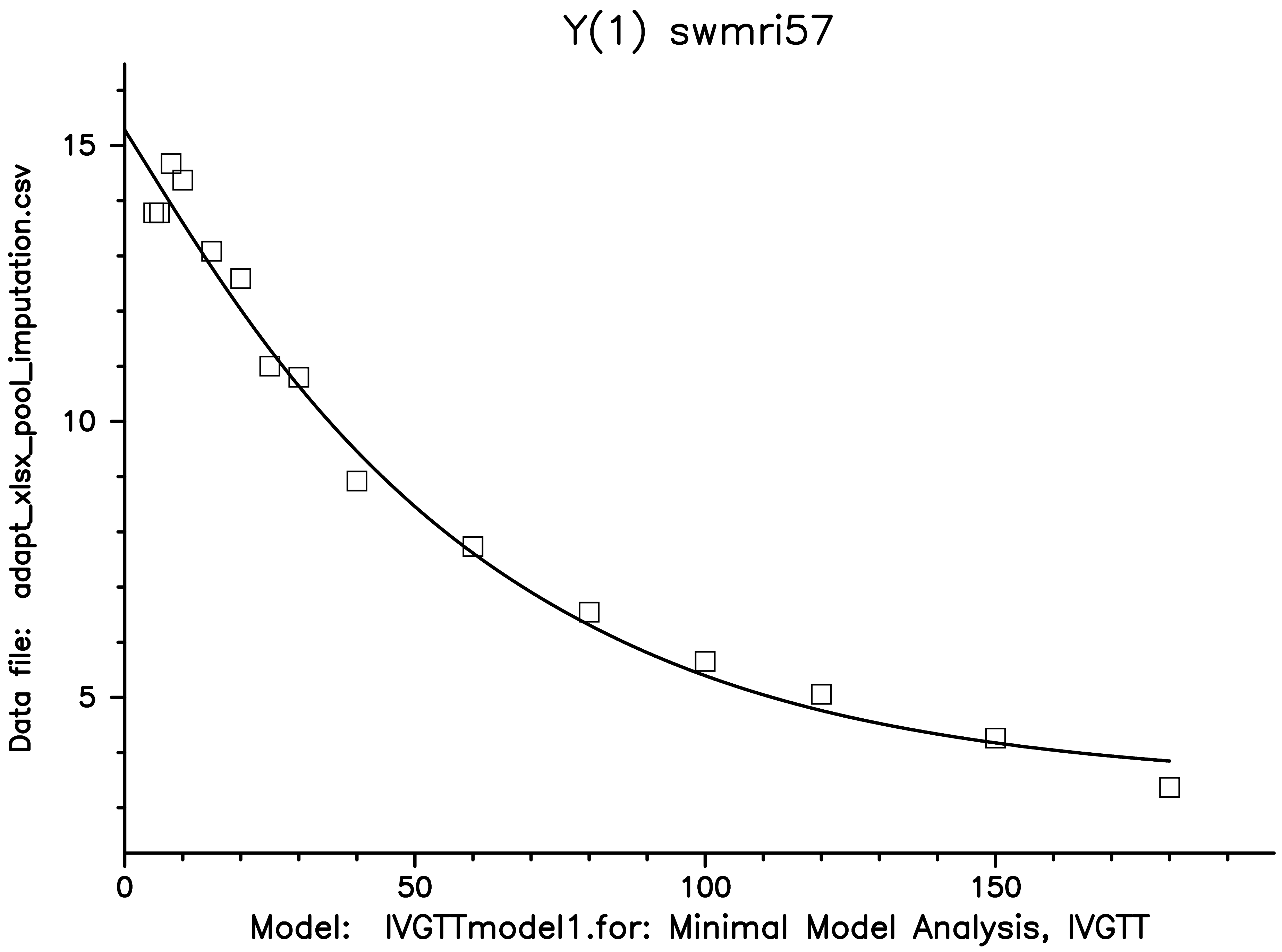
Y(1) swmri57
Data file: adapt_xlsx_pool_imputation.csv
15
10
5
0
50
100
150
Model: IVGTTmodel1.for: Minimal Model Analysis, IVGTT

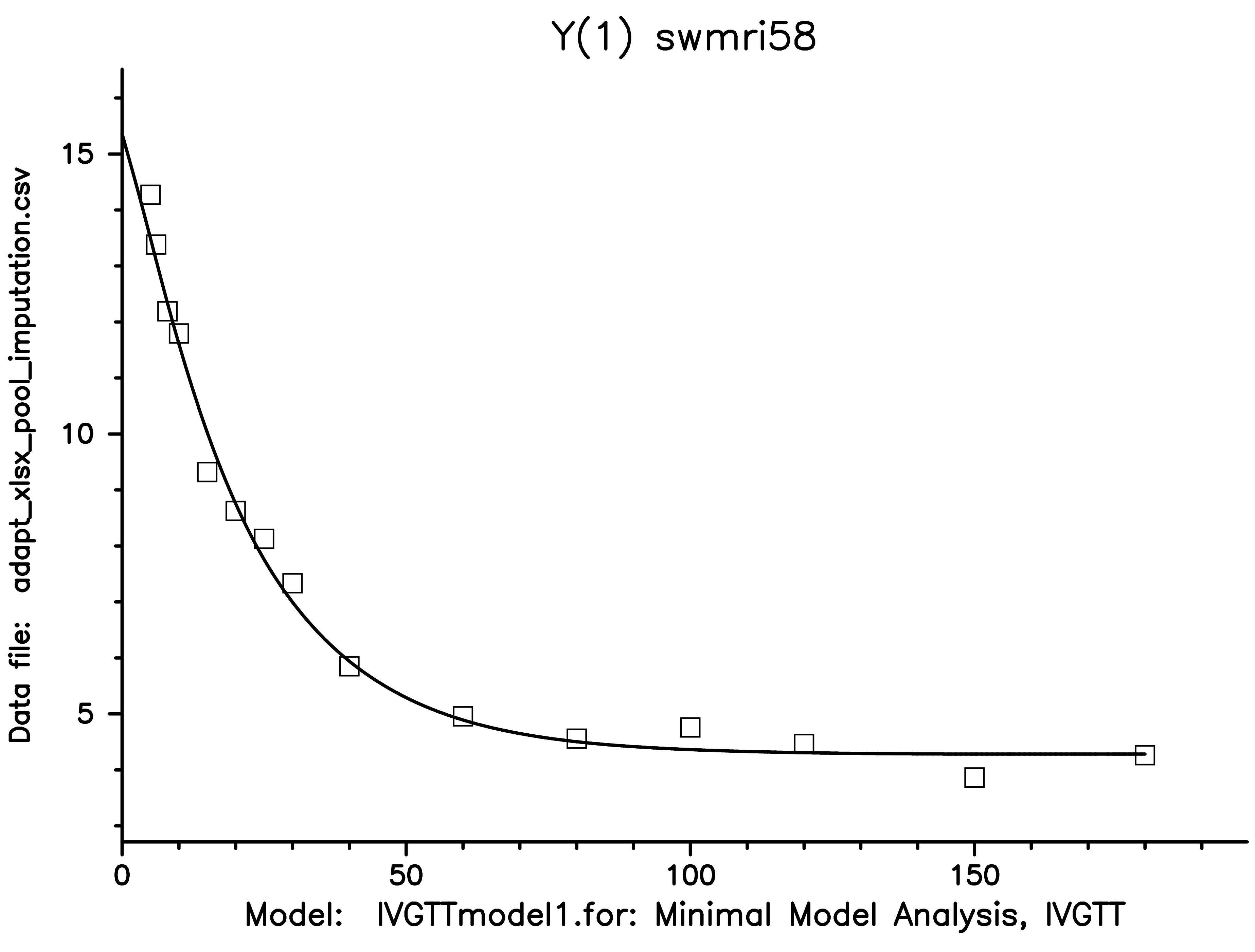

Y(1) swmri58
Data file: adapt_xlsx_pool_imputation.csv
Model: IVGTTmodel1.for: Minimal Model Analysis, IVGTT
15
10
5
0
50
100
150

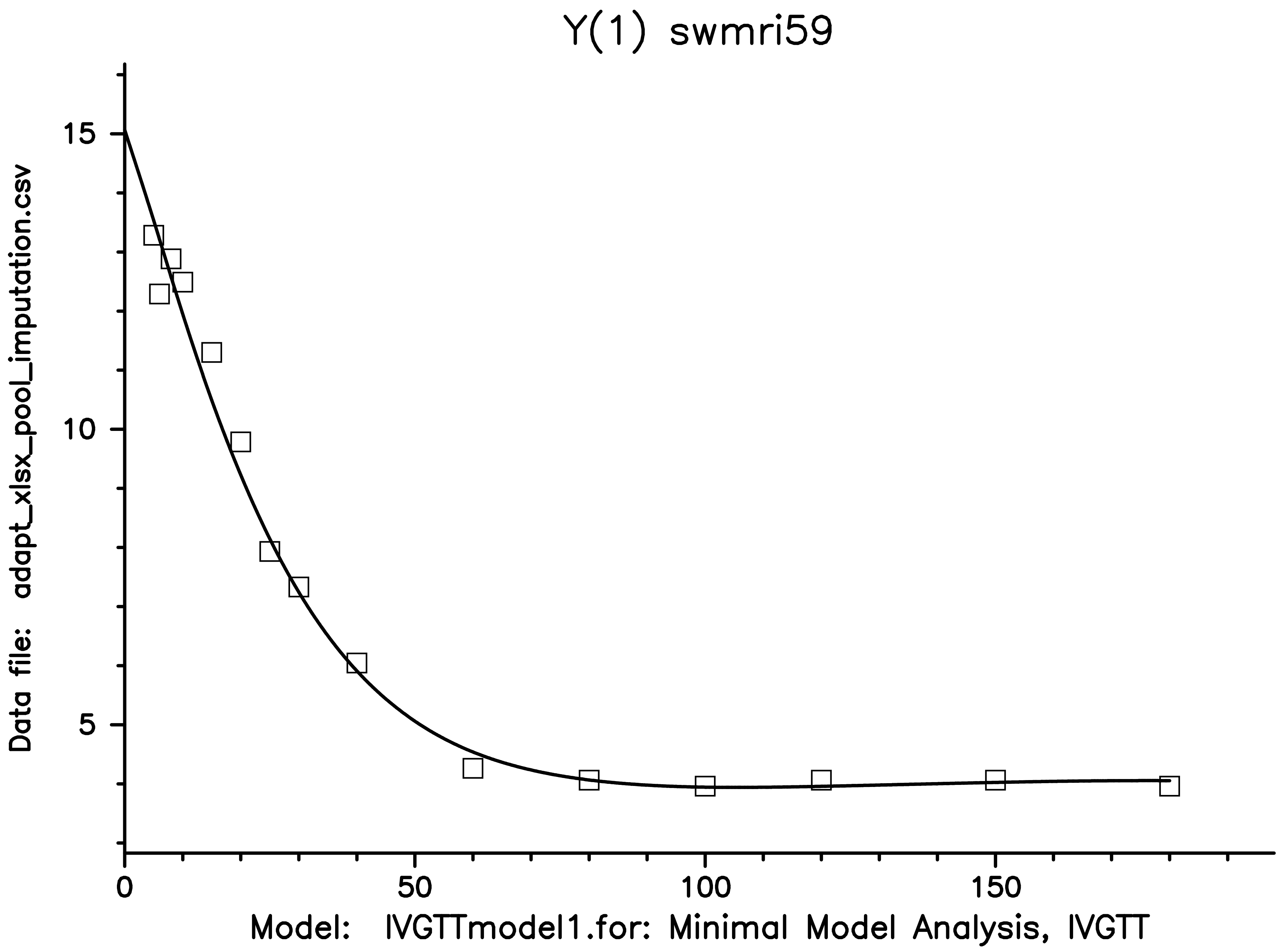
Y(1) swmri59
Data file: adapt_xlsx_pool_imputation.csv
Model: IVGTTmodel1.for: Minimal Model Analysis, IVGTT
15
10
5
0
50
100
150

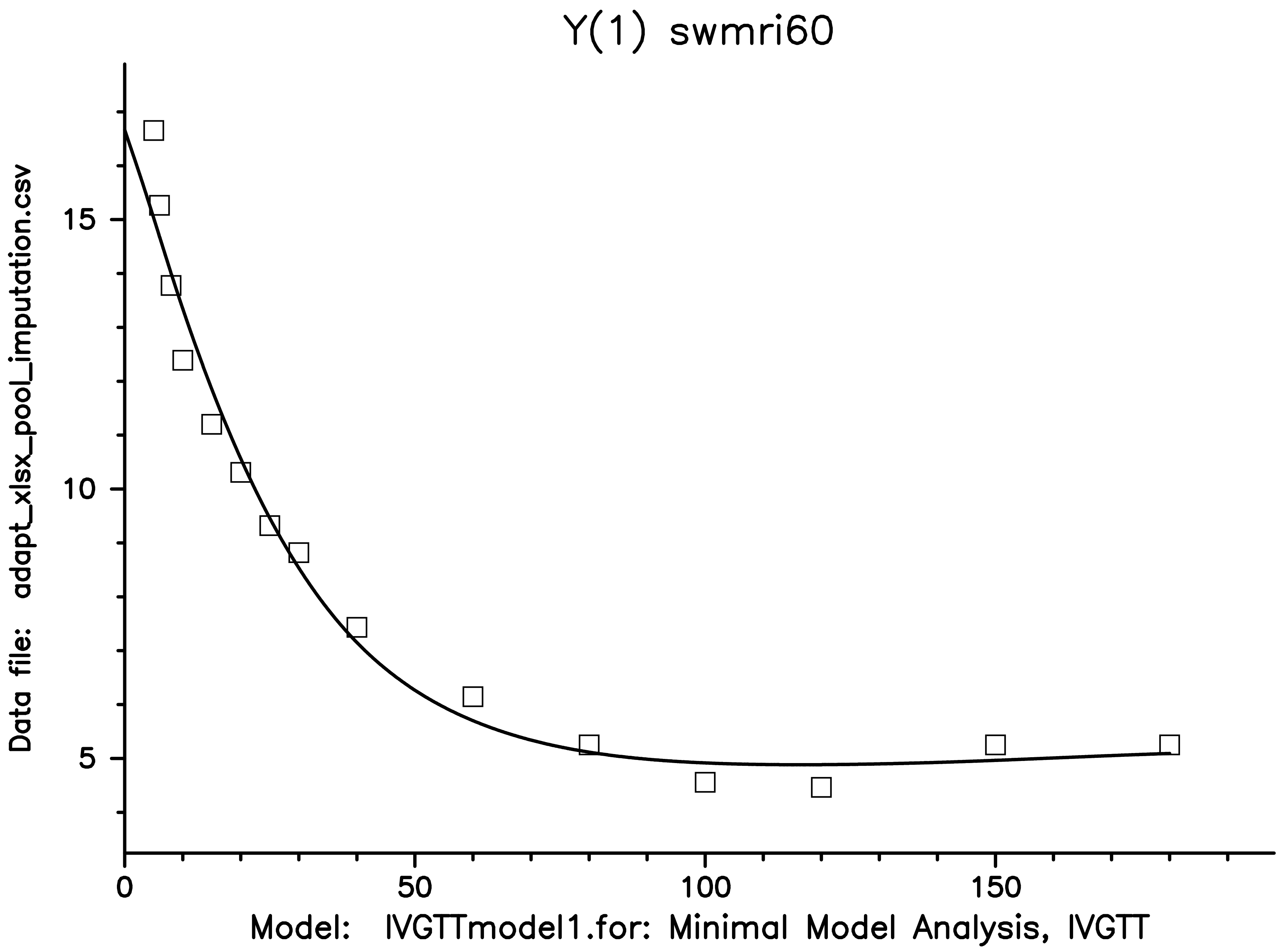

Y(1) swmri60
Data file: adapt_xlsx_pool_imputation.csv
15
10
5
0
50
100
150
Model: IVGTTmodel1.for: Minimal Model Analysis, IVGTT

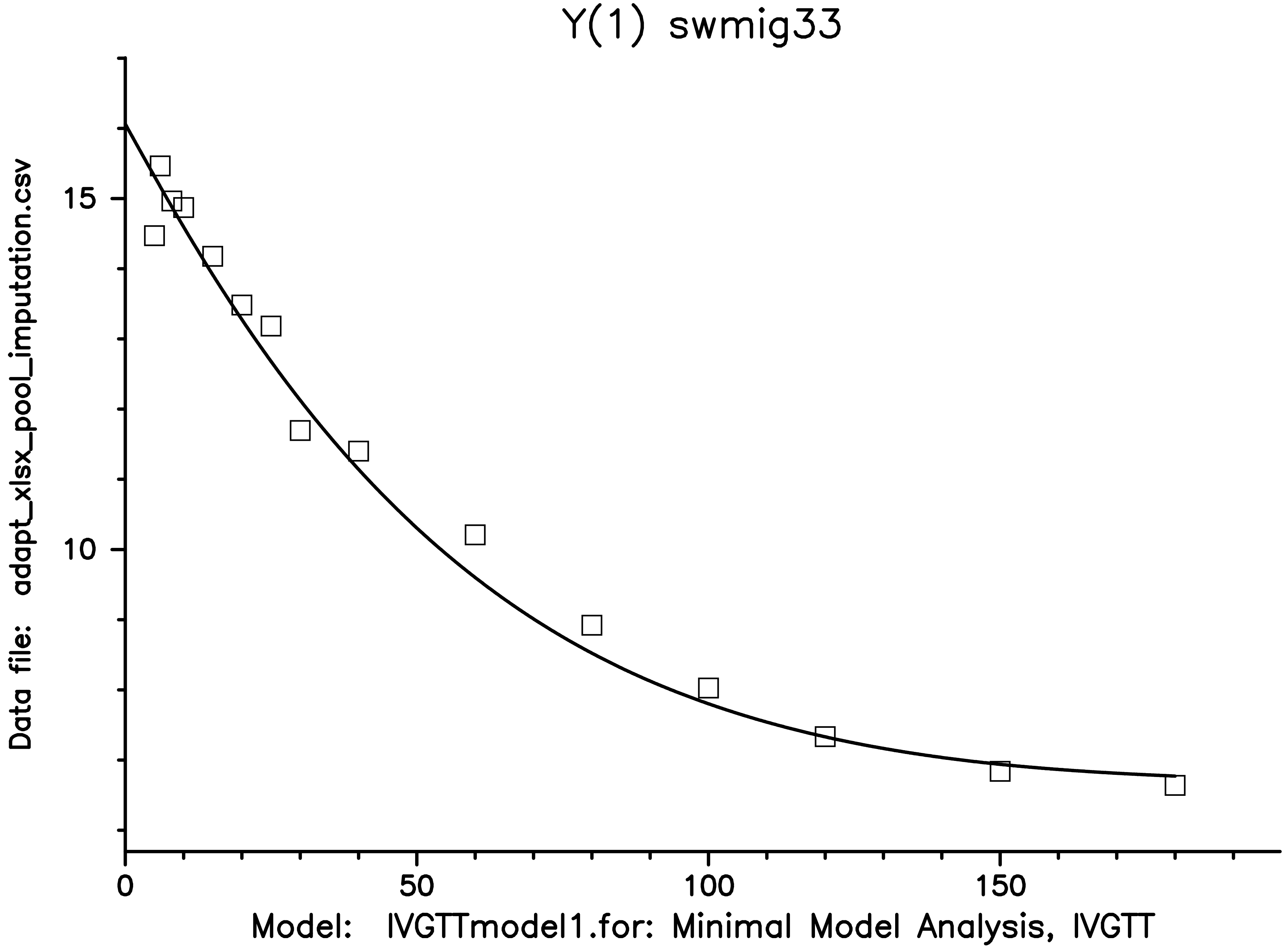
Y(1) swmig33
Data file: adapt_xlsx_pool_imputation.csv
15
10
0
50
100
150
Model: IVGTTmodel1.for: Minimal Model Analysis, IVGTT

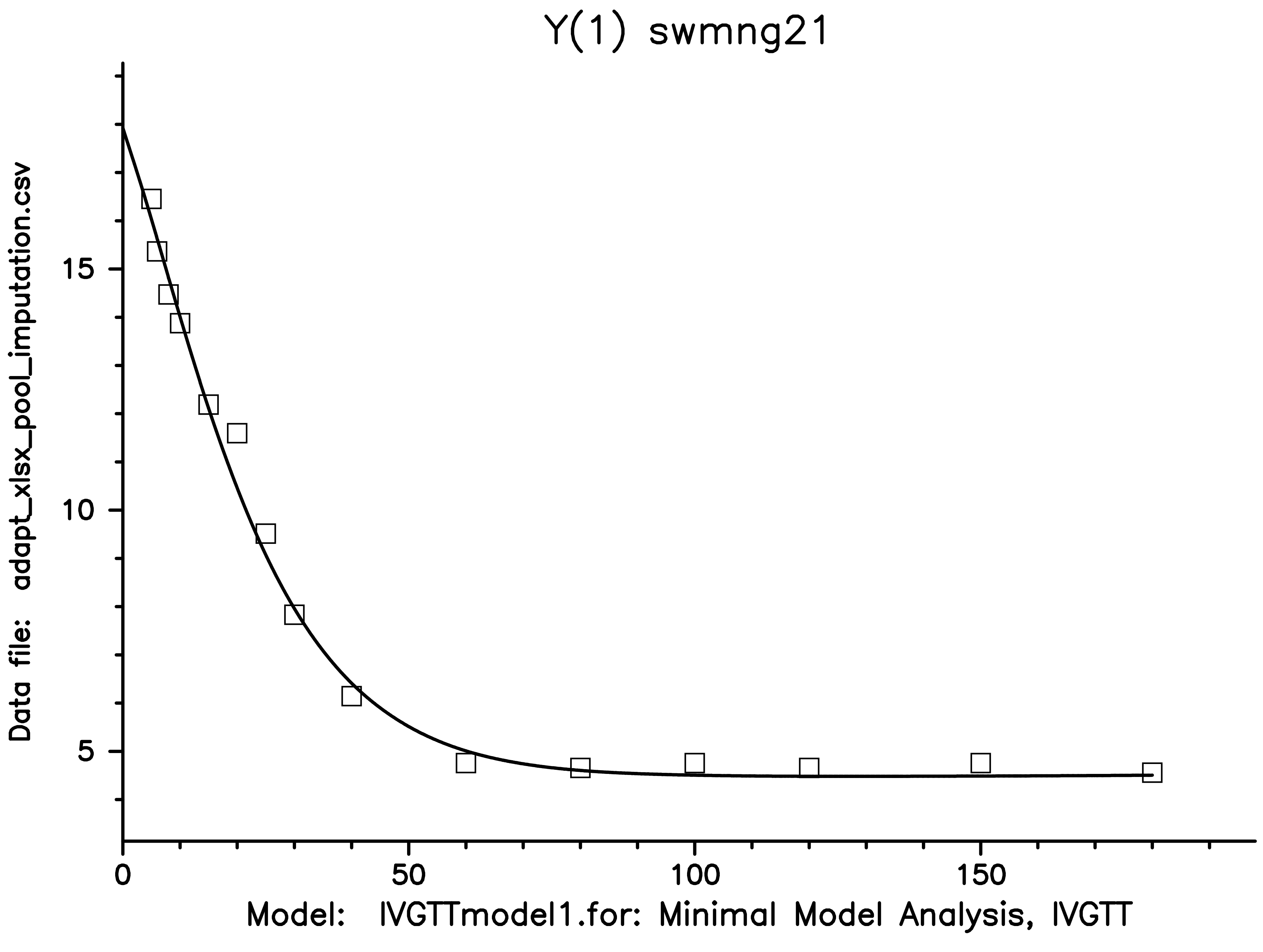
Y(1) swmng21
Data file: adapt_xlsx_pool_imputation.csv
Model: IVGTTmodel1.for: Minimal Model Analysis, IVGTT
15
10
5
0
50
100
150

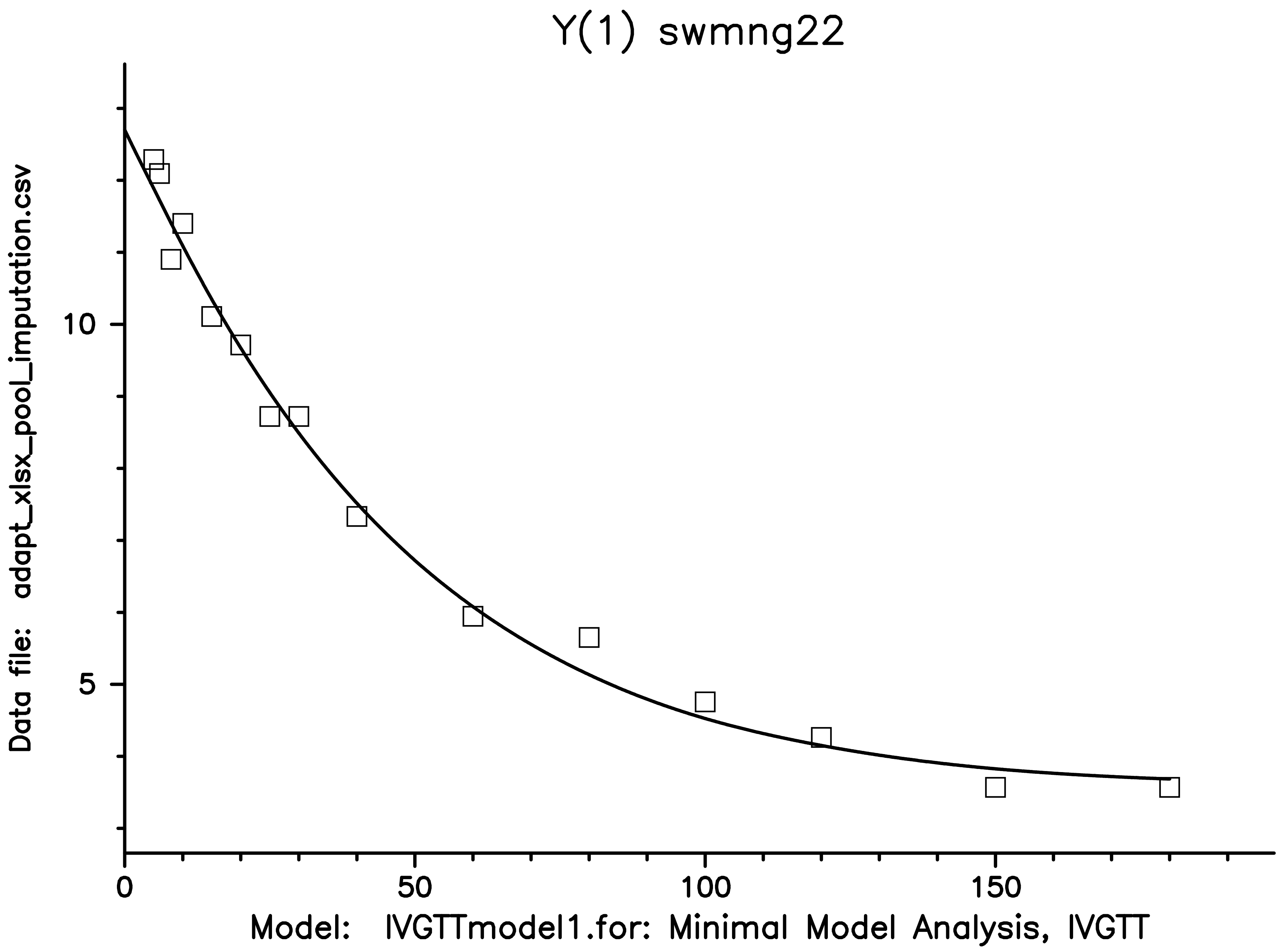

Y(1) swmng22
10
5
0
50
100
150
Data file: adapt_xlsx_pool_imputation.csv
Model: IVGTTmodel1.for: Minimal Model Analysis, IVGTT

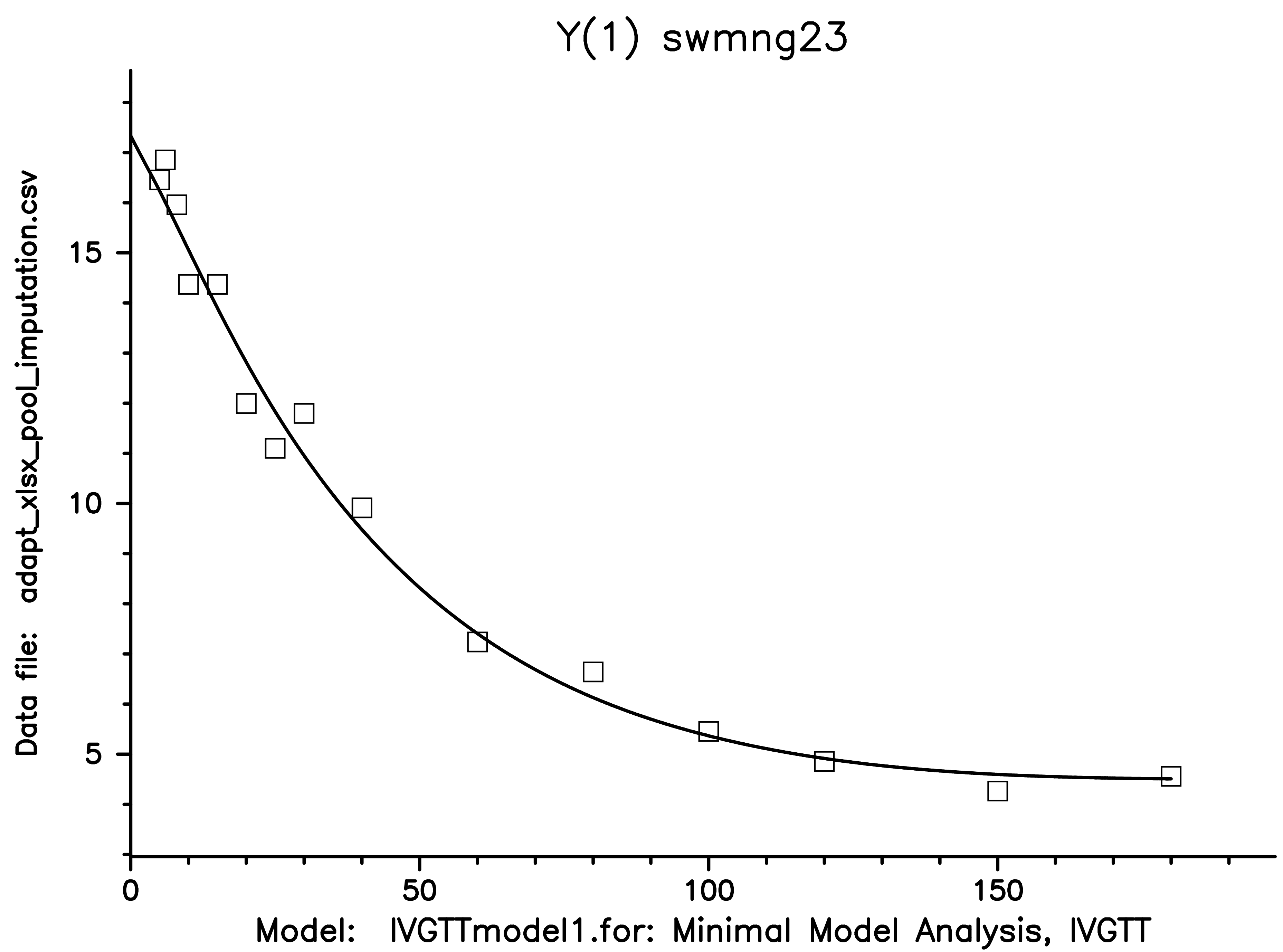
Y(1) swmng23
Data file: adapt_xlsx_pool_imputation.csv
15
10
5
0
50
100
150
Model: IVGTTmodel1.for: Minimal Model Analysis, IVGTT

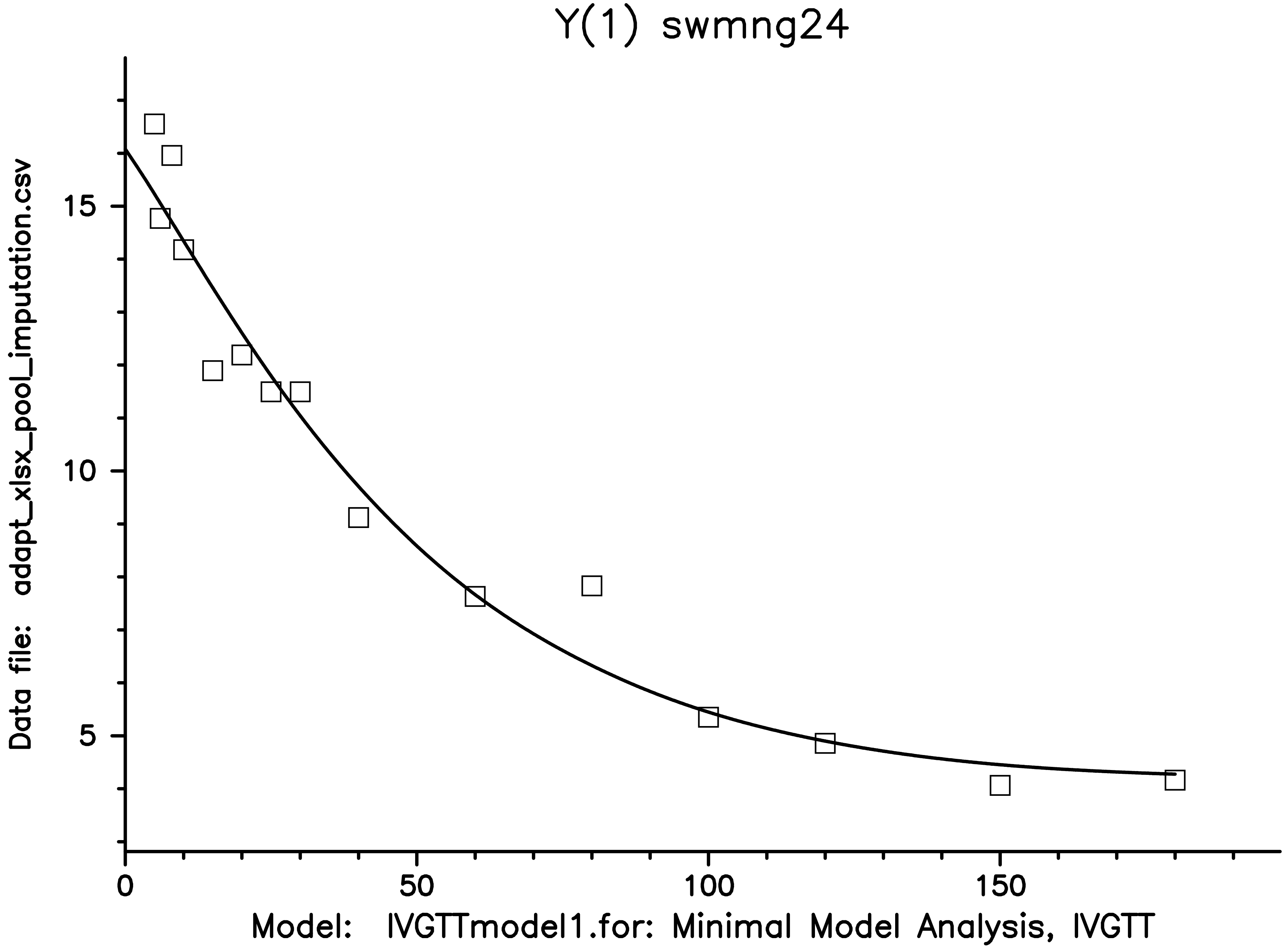
Y(1) swmng24
Data file: adapt_xlsx_pool_imputation.csv
15
10
5
0
50
100
150
Model: IVGTTmodel1.for: Minimal Model Analysis, IVGTT

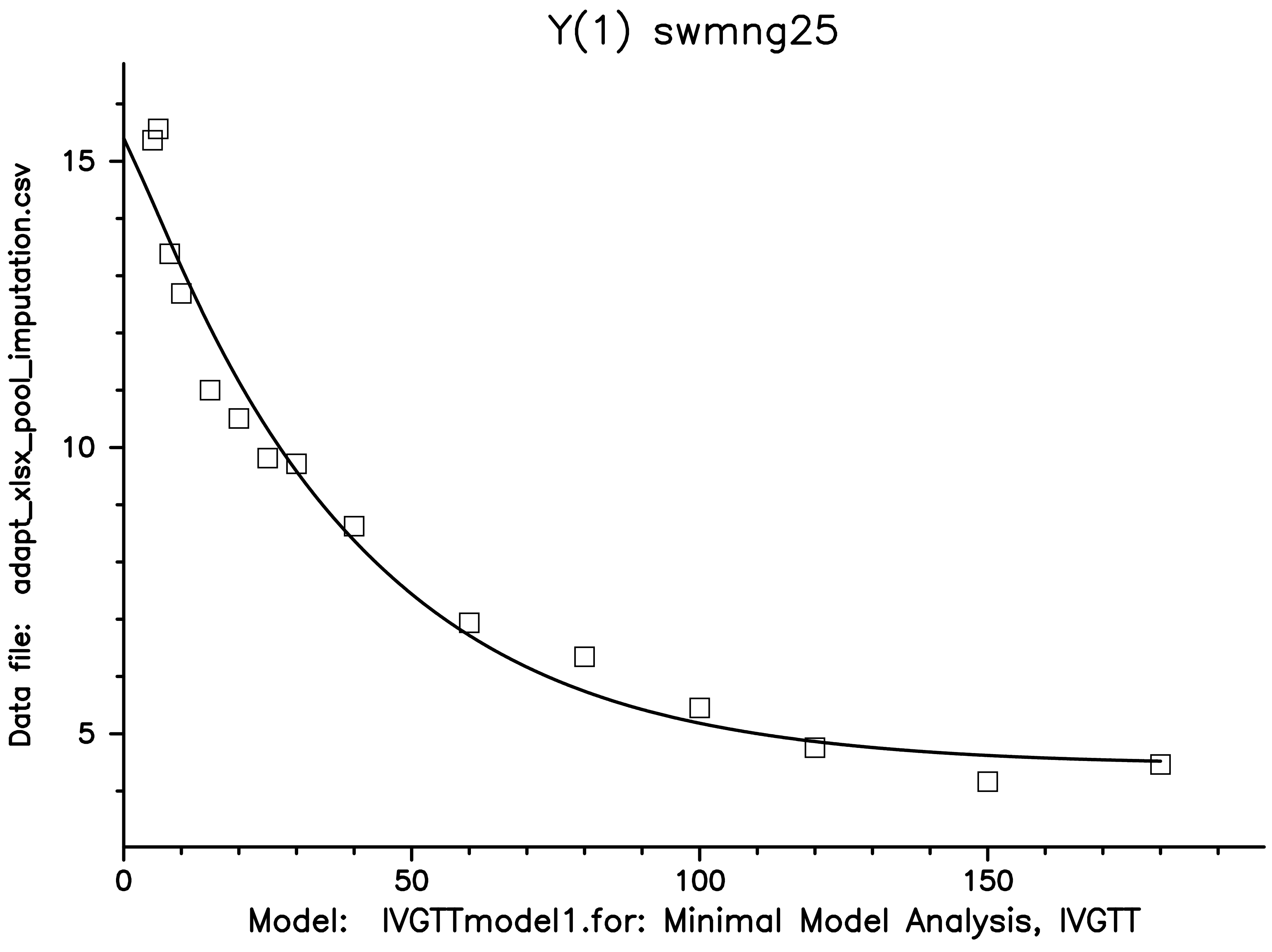

Y(1) swmng25
Data file: adapt_xlsx_pool_imputation.csv
15
10
5
0
50
100
150
Model: IVGTTmodel1.for: Minimal Model Analysis, IVGTT

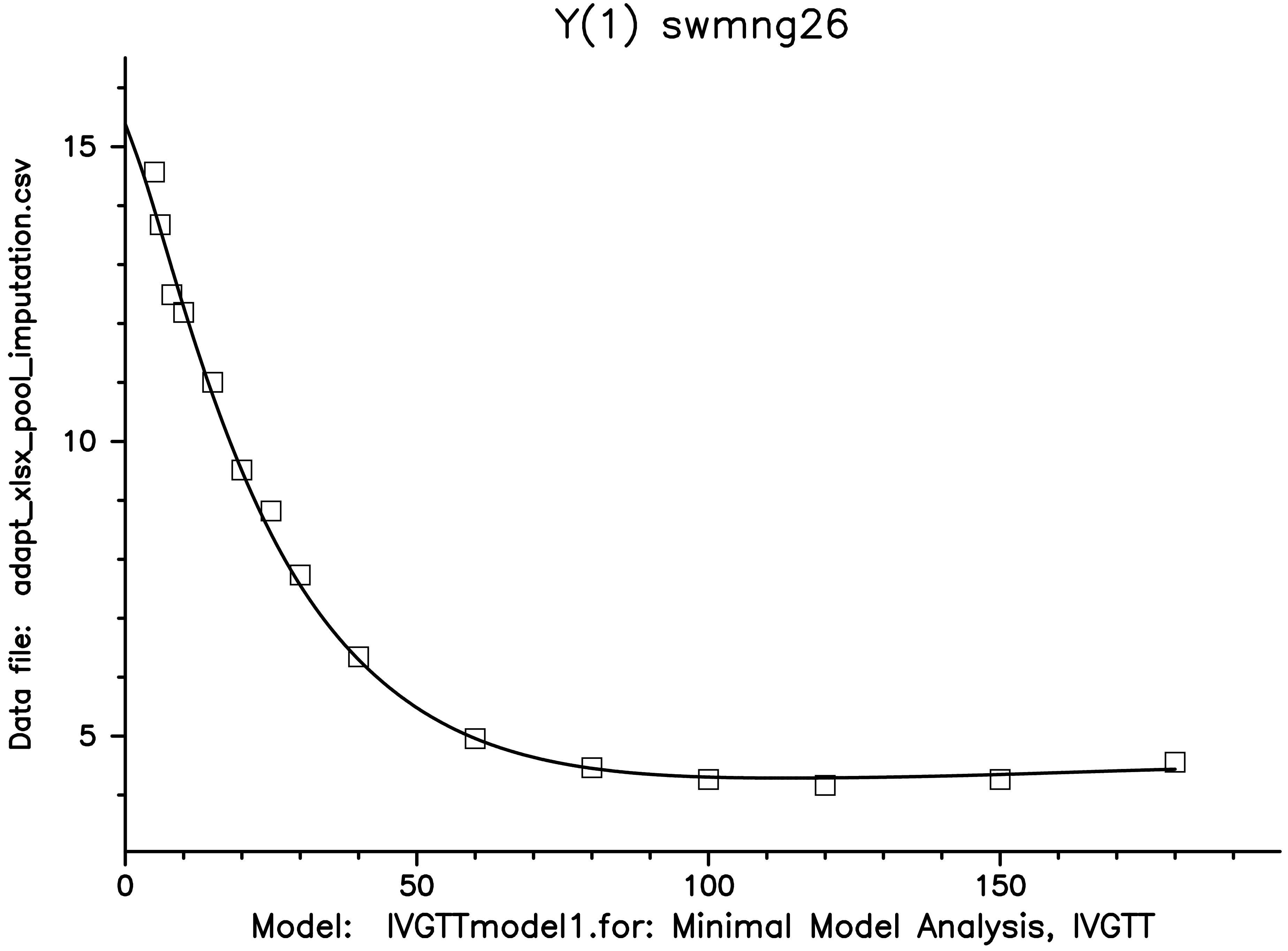
Y(1) swmng26
Data file: adapt_xlsx_pool_imputation.csv
Model: IVGTTmodel1.for: Minimal Model Analysis, IVGTT
15
10
5
0
50
100
150

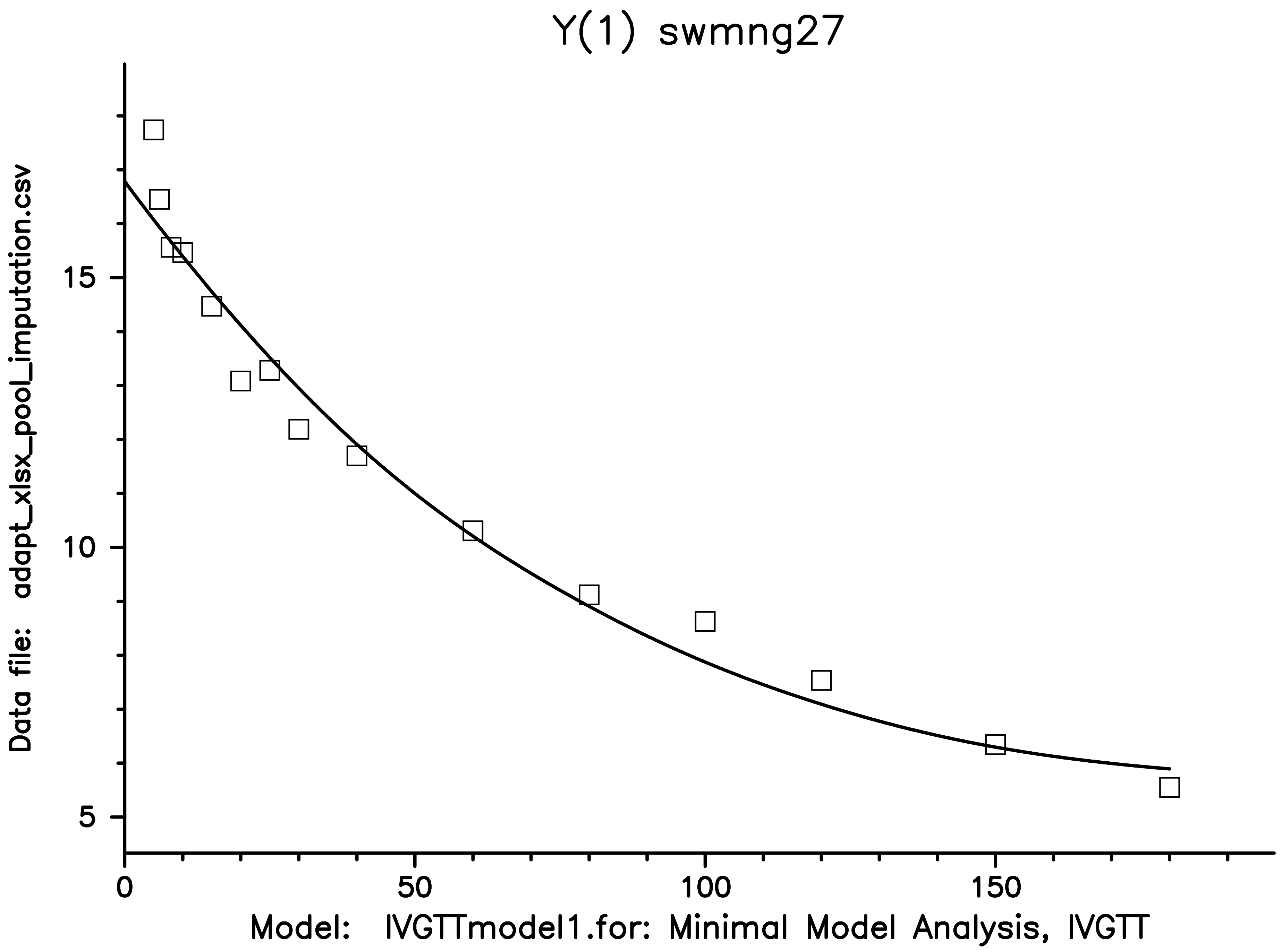

Y(1) swmng27
Data file: adapt_xlsx_pool_imputation.csv
15
10
5
0
50
100
150
Model: IVGTTmodel1.for: Minimal Model Analysis, IVGTT

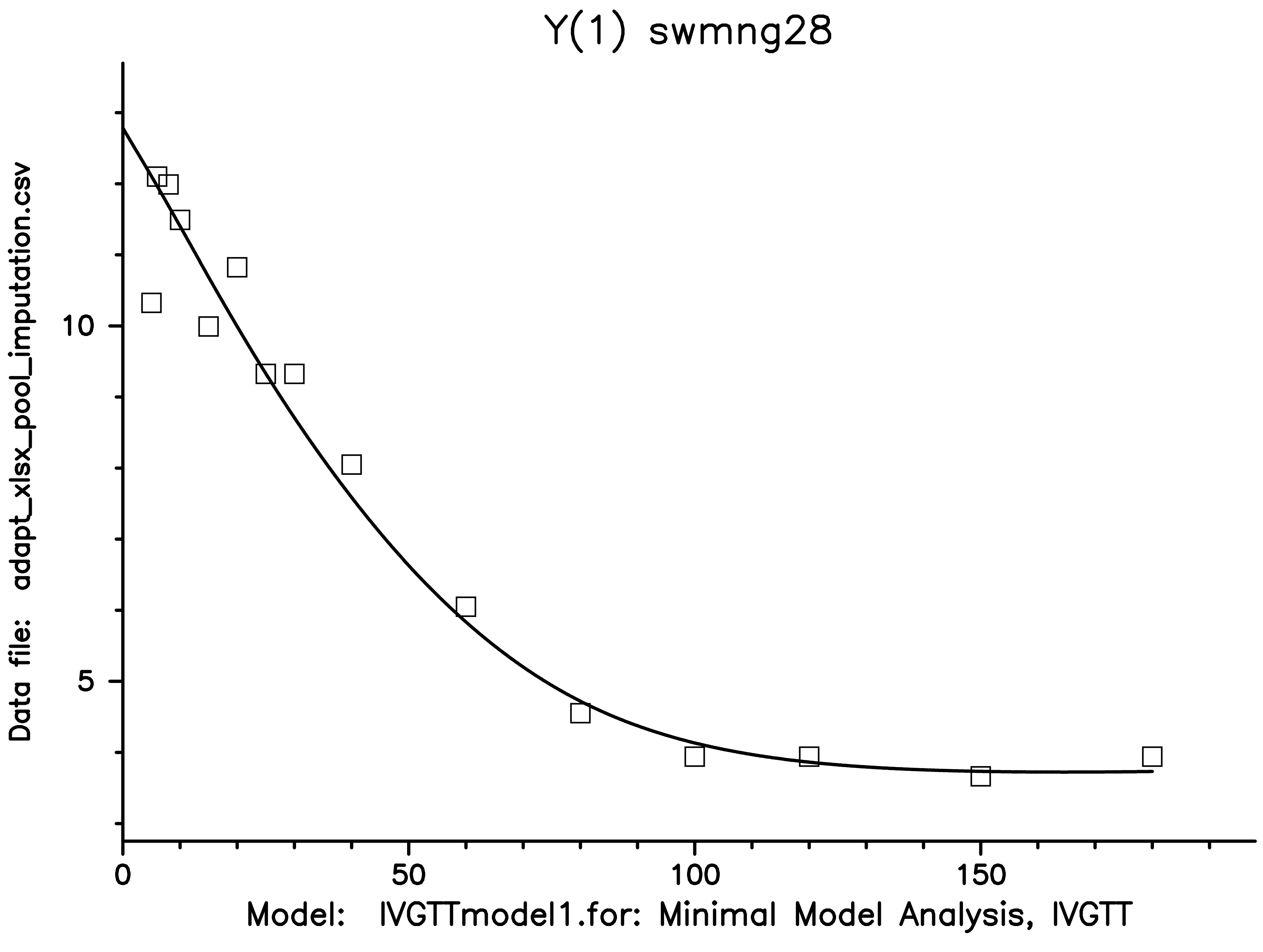

Y(1) swmng28
Data file: adapt_xlsx_pool_imputation.csv
Model: IVGTTmodel1.for: Minimal Model Analysis, IVGTT
10
5
0
50
100
150

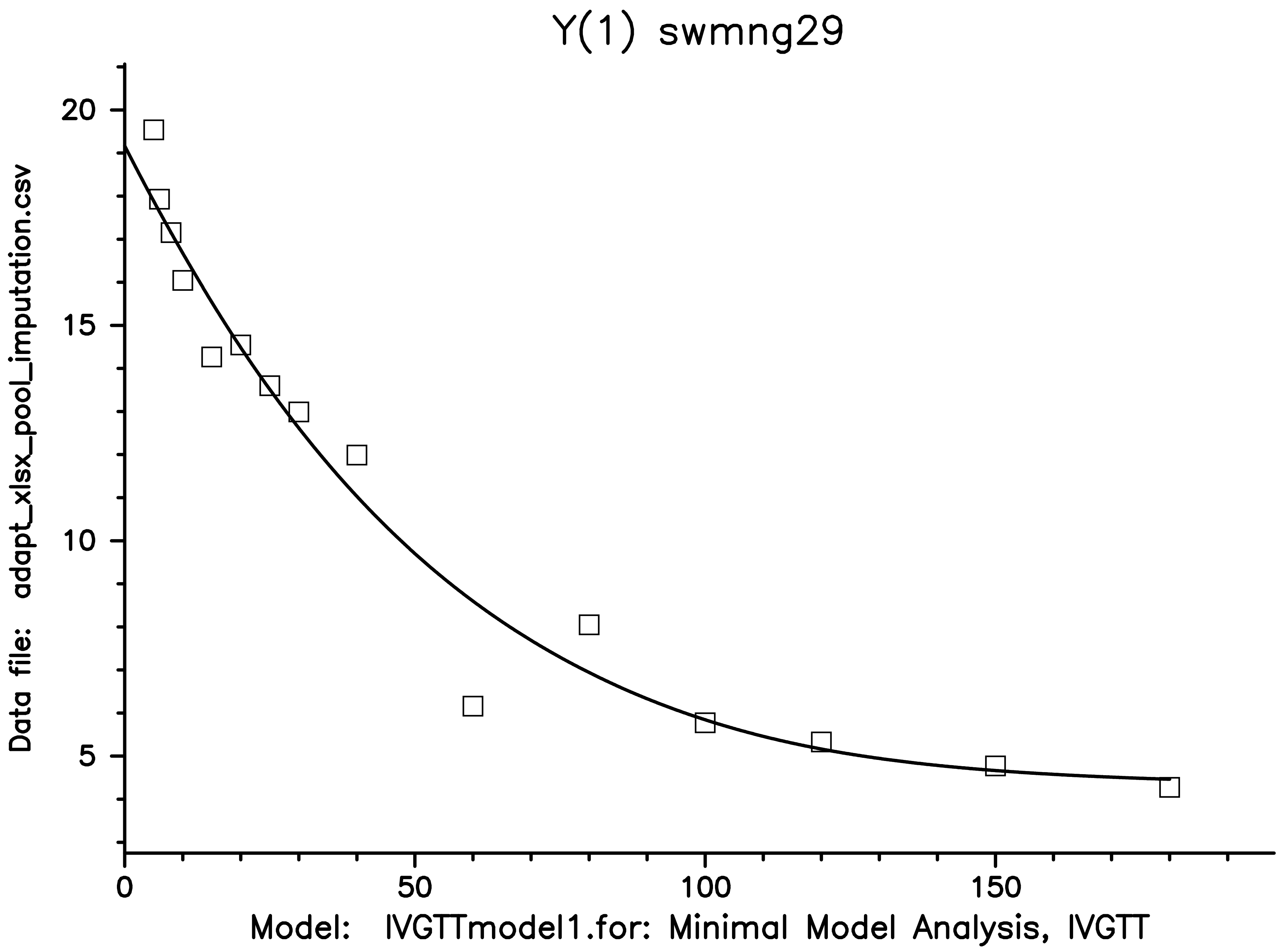
Y(1) swmng29
20
15
10
5
0
50
100
150
Data file: adapt_xlsx_pool_imputation.csv
Model: IVGTTmodel1.for: Minimal Model Analysis, IVGTT

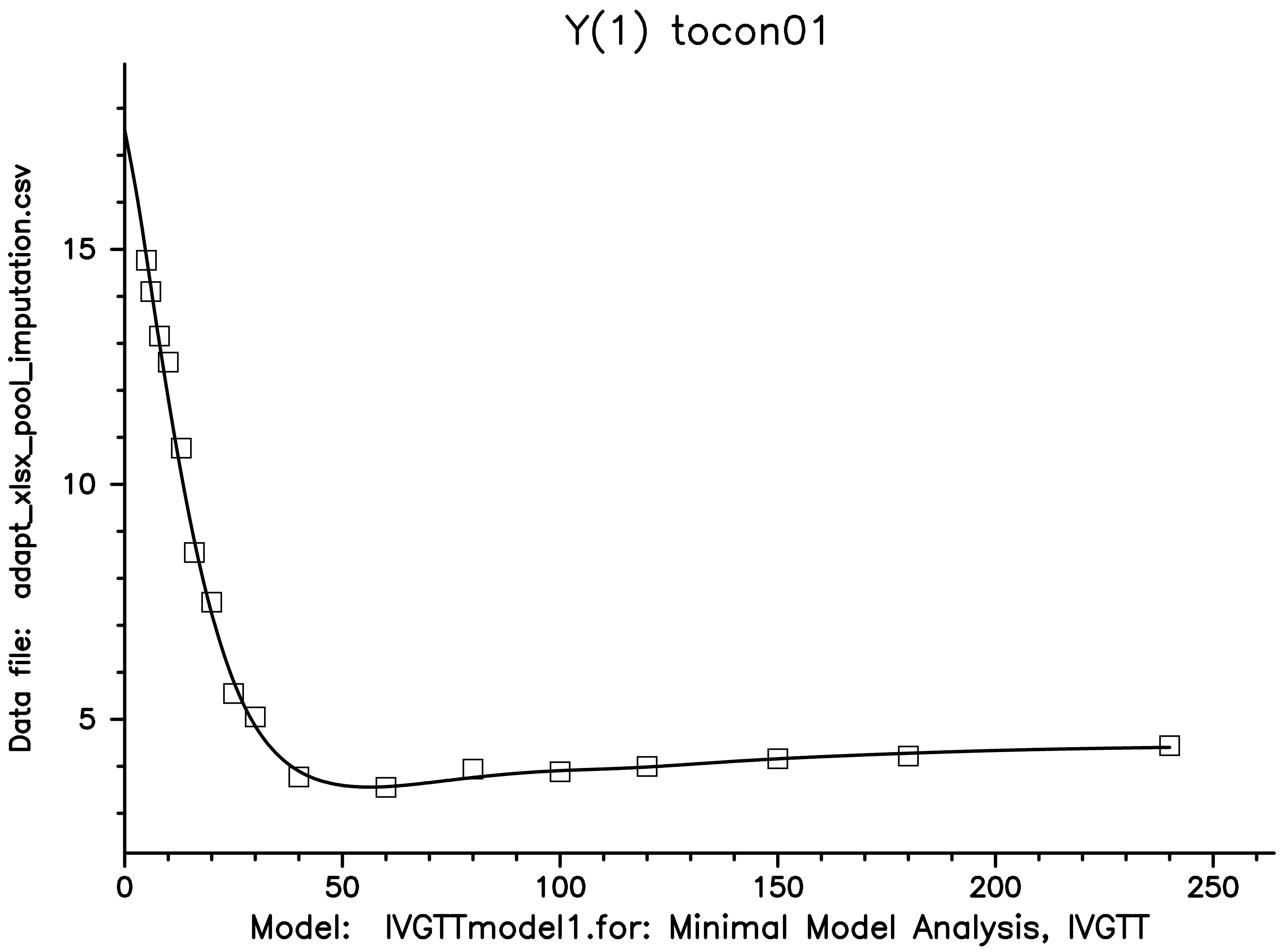

Y(1) tocon01
Data file: adapt_xlsx_pool_imputation.csv
Model: IVGTTmodel1.for: Minimal Model Analysis, IVGTT
15
10
5
0
50
100
150
200
250

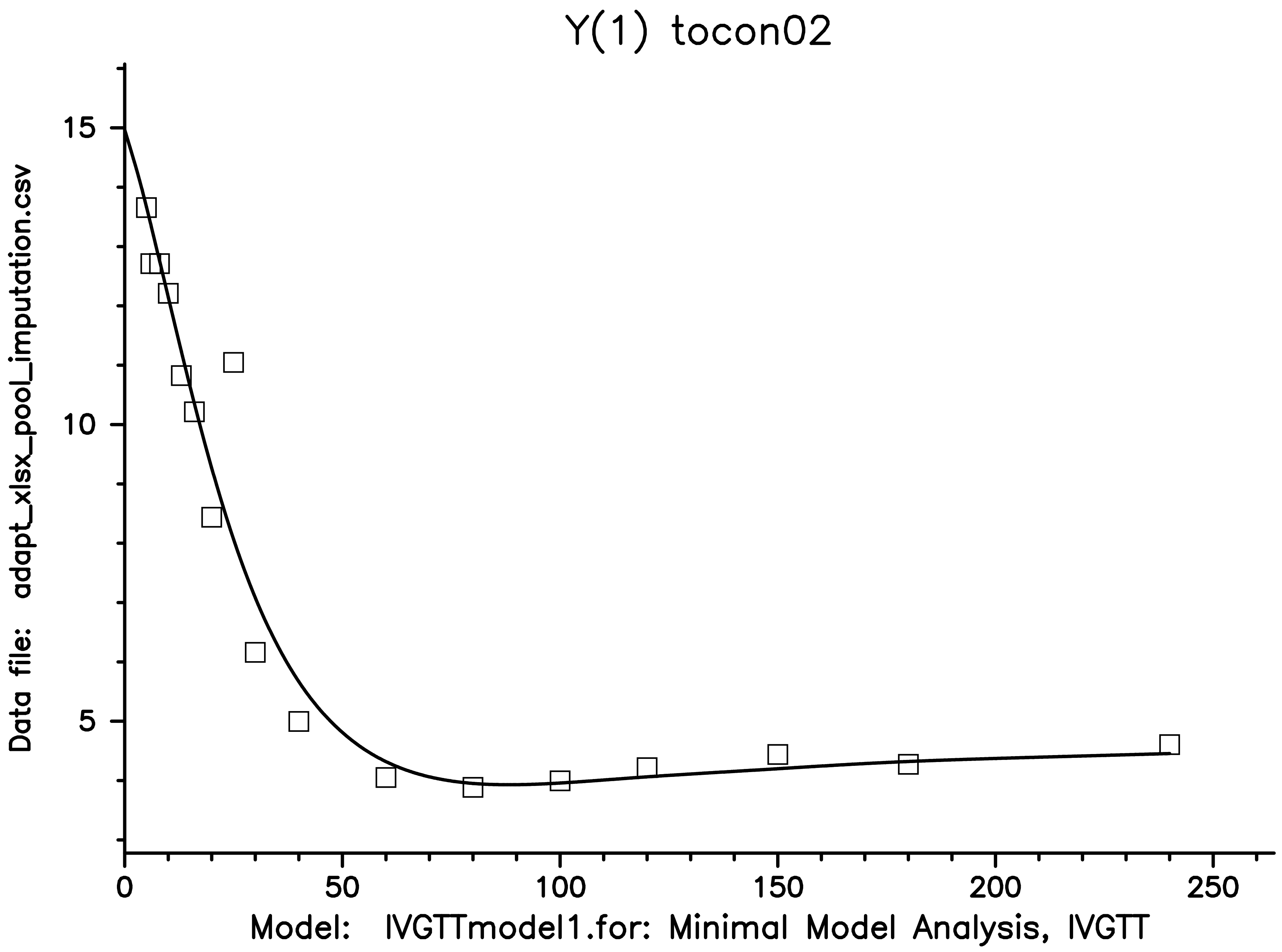
Y(1) tocon02
Data file: adapt_xlsx_pool_imputation.csv
15
10
5
0
50
100
150
200
250
Model: IVGTTmodel1.for: Minimal Model Analysis, IVGTT

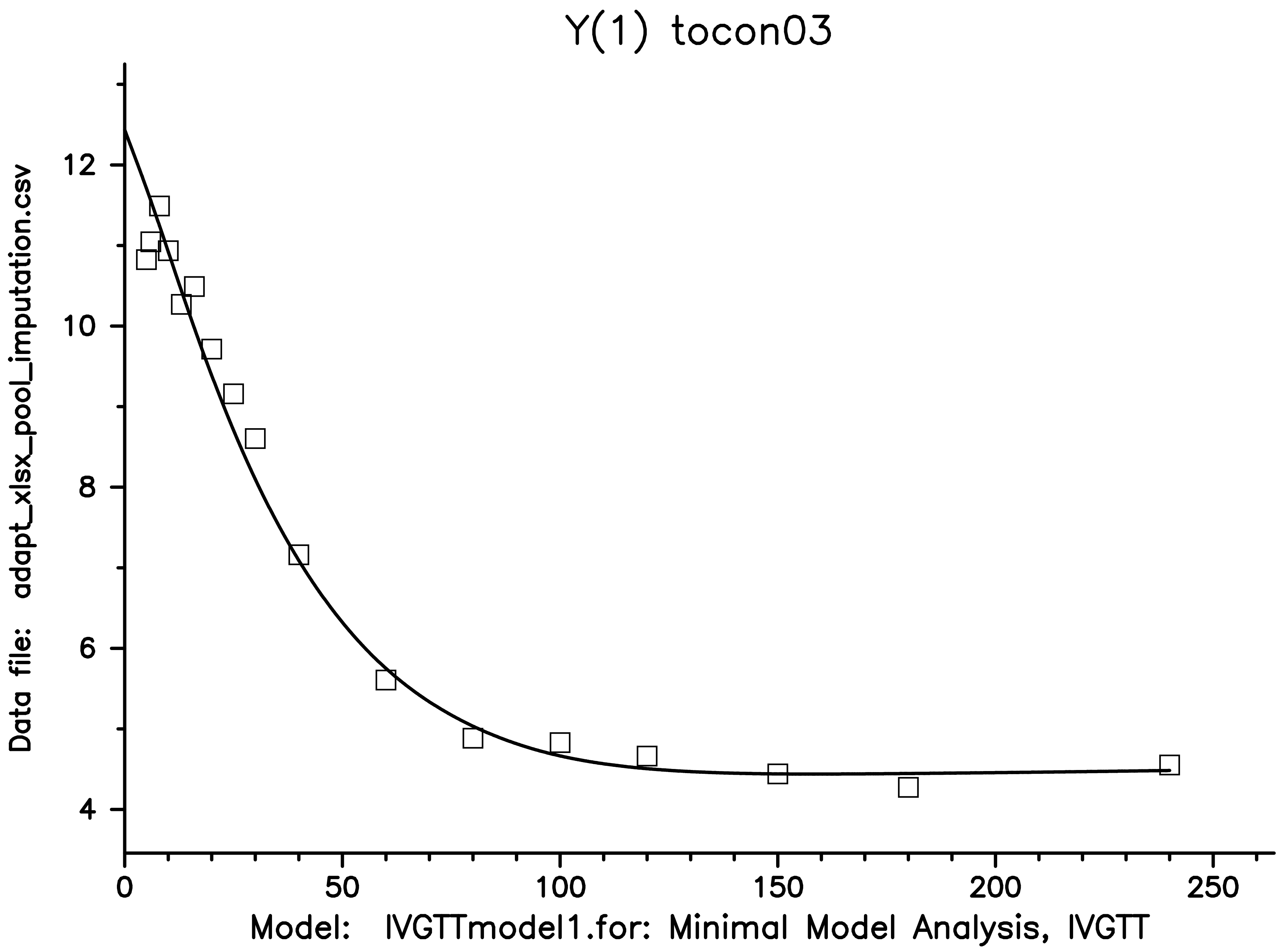

Y(1) tocon03
Data file: adapt_xlsx_pool_imputation.csv
12
10
8
6
4
0
50
100
150
200
250
Model: IVGTTmodel1.for: Minimal Model Analysis, IVGTT

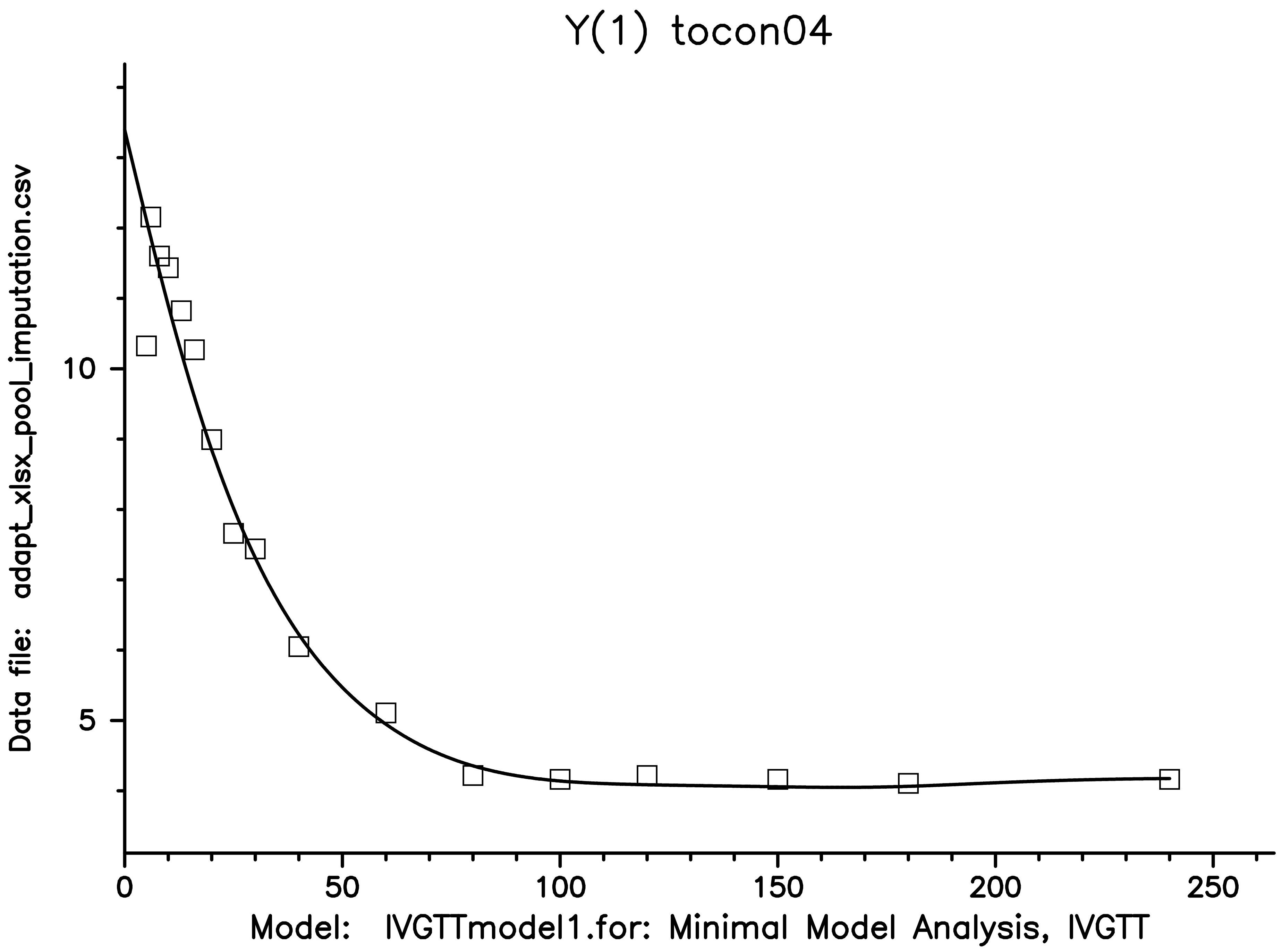
Y(1) tocon04
Data file: adapt_xlsx_pool_imputation.csv
10
5
0
50
100
150
200
250
Model: IVGTTmodel1.for: Minimal Model Analysis, IVGTT

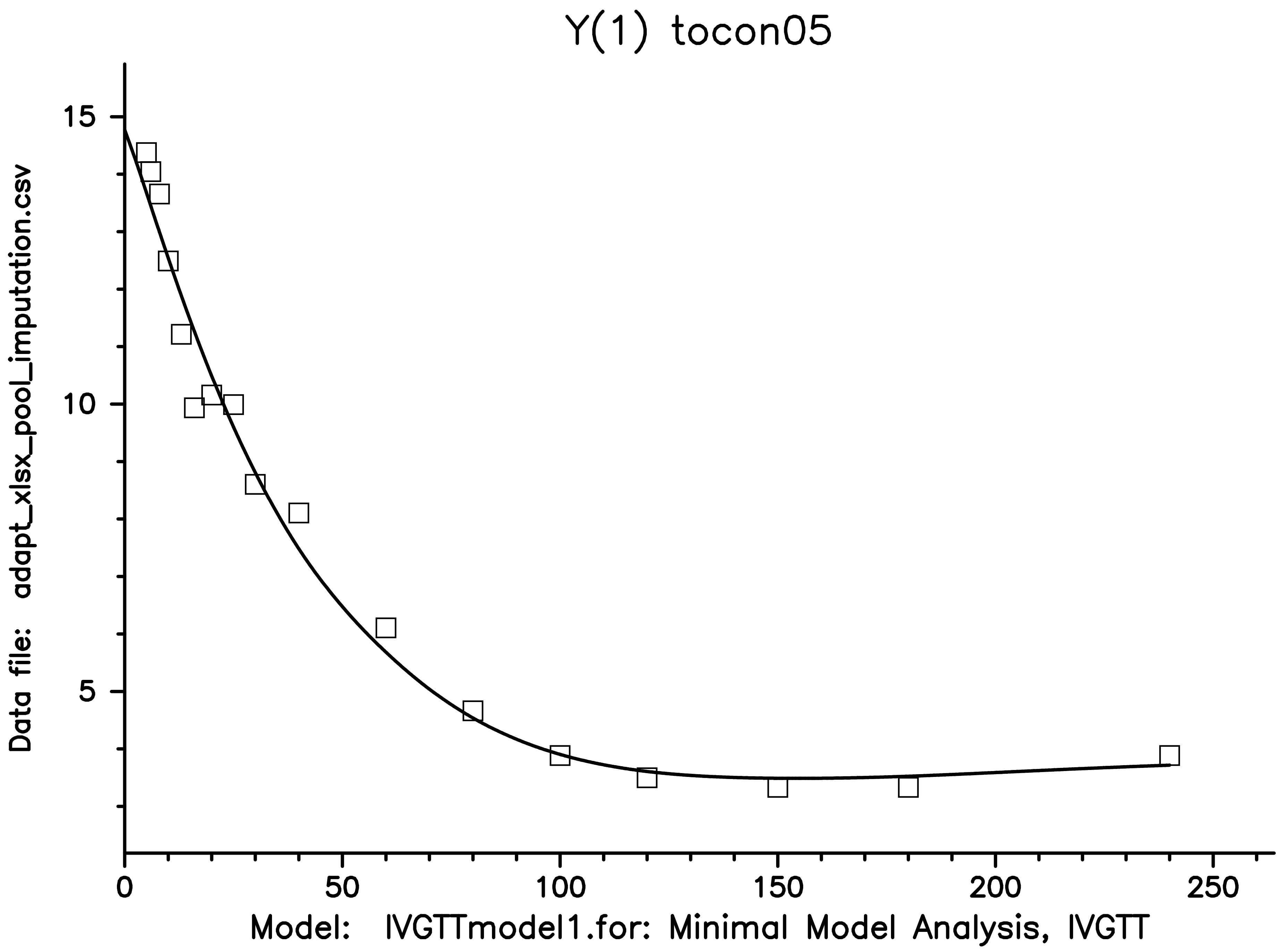
Y(1) tocon05
Data file: adapt_xlsx_pool_imputation.csv
15
10
5
0
50
100
150
200
250
Model: IVGTTmodel1.for: Minimal Model Analysis, IVGTT

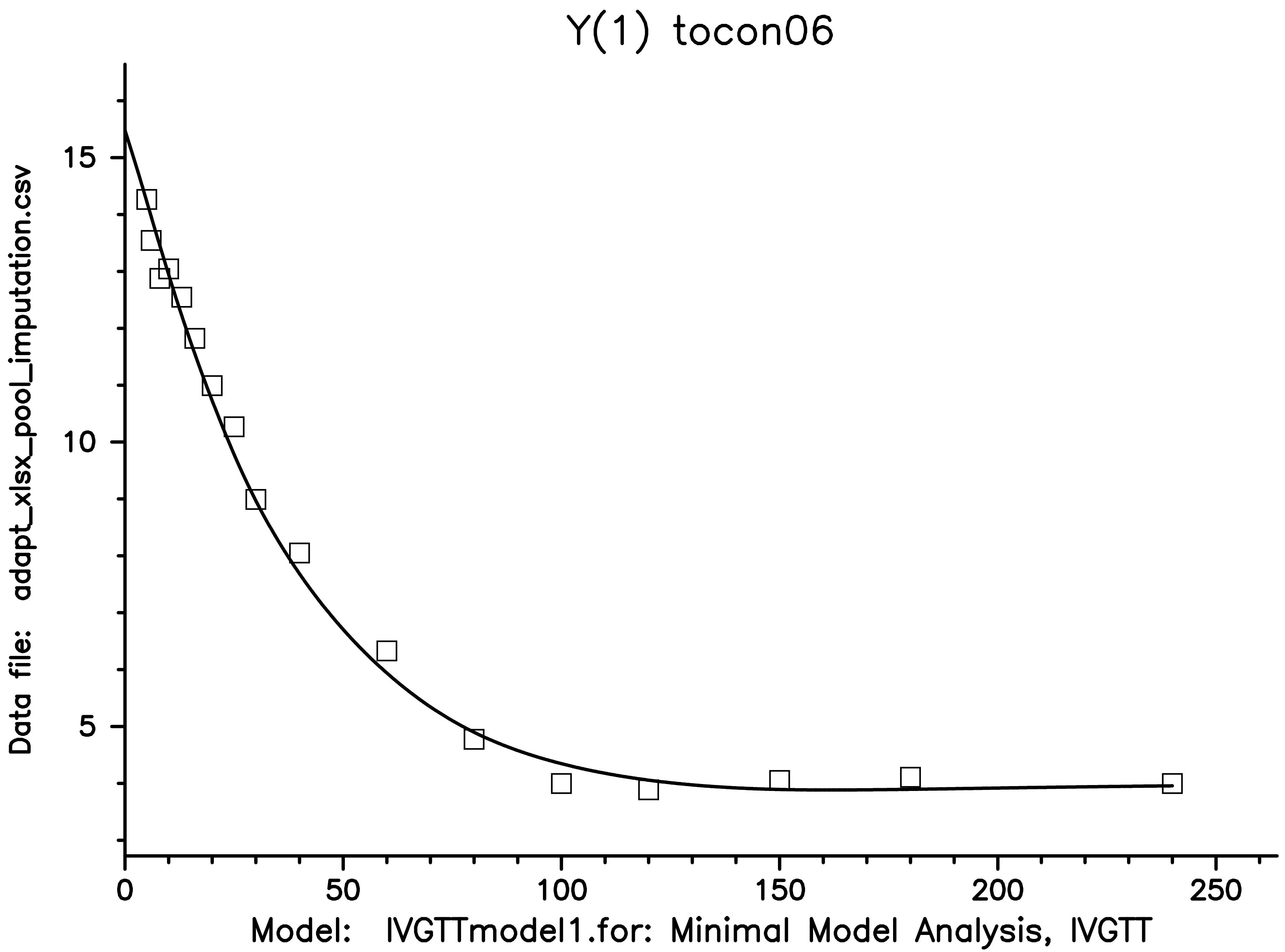
Y(1) tocon06
Data file: adapt_xlsx_pool_imputation.csv
Model: IVGTTmodel1.for: Minimal Model Analysis, IVGTT
15
10
5
0
50
100
150
200
250

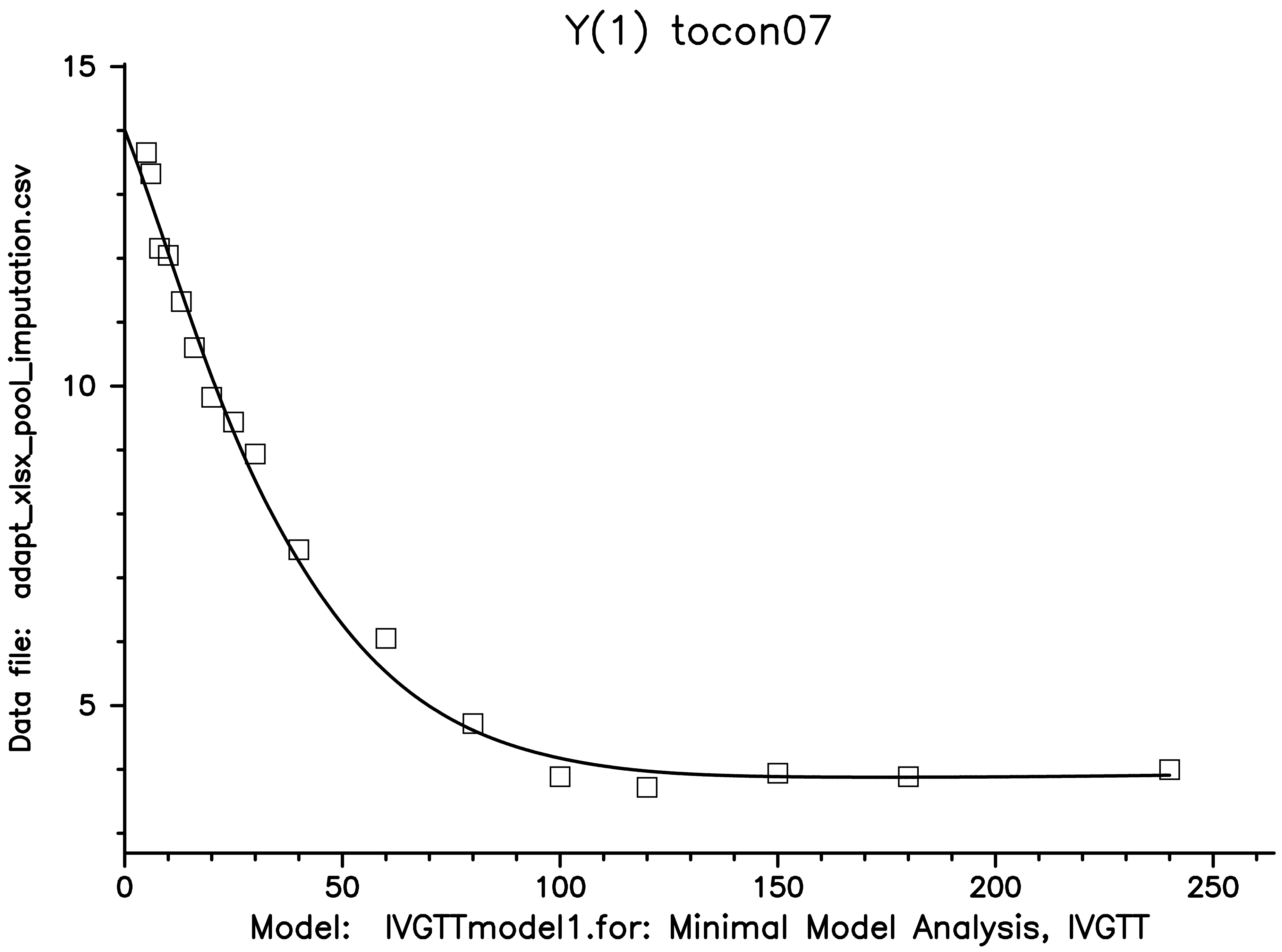
Y(1) tocon07
Data file: adapt_xlsx_pool_imputation.csv
Model: IVGTTmodel1.for: Minimal Model Analysis, IVGTT
15
10
5
0
50
100
150
200
250

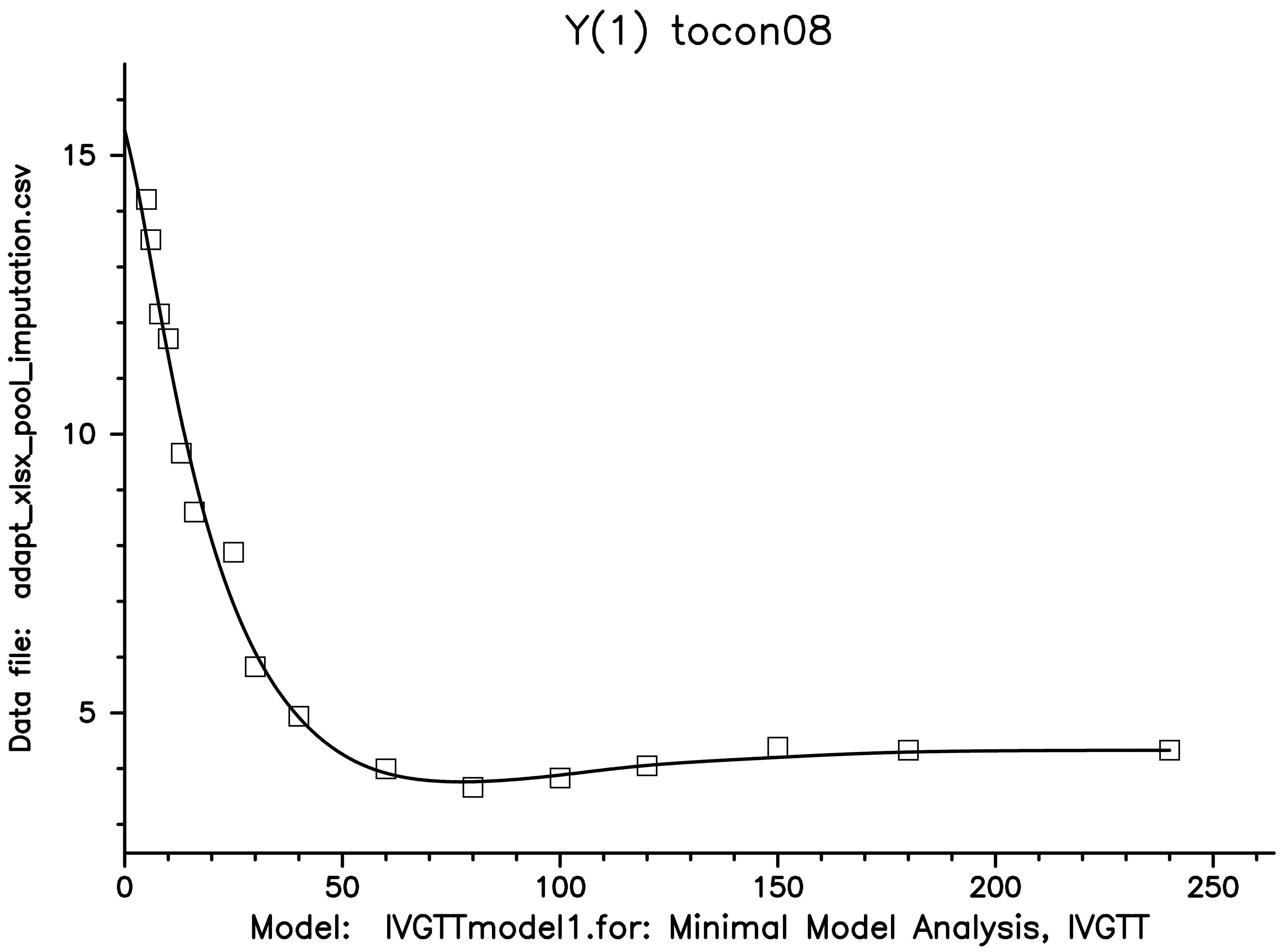
Y(1) tocon08
Data file: adapt_xlsx_pool_imputation.csv
15
10
5
0
50
100
150
200
250
Model: IVGTTmodel1.for: Minimal Model Analysis, IVGTT

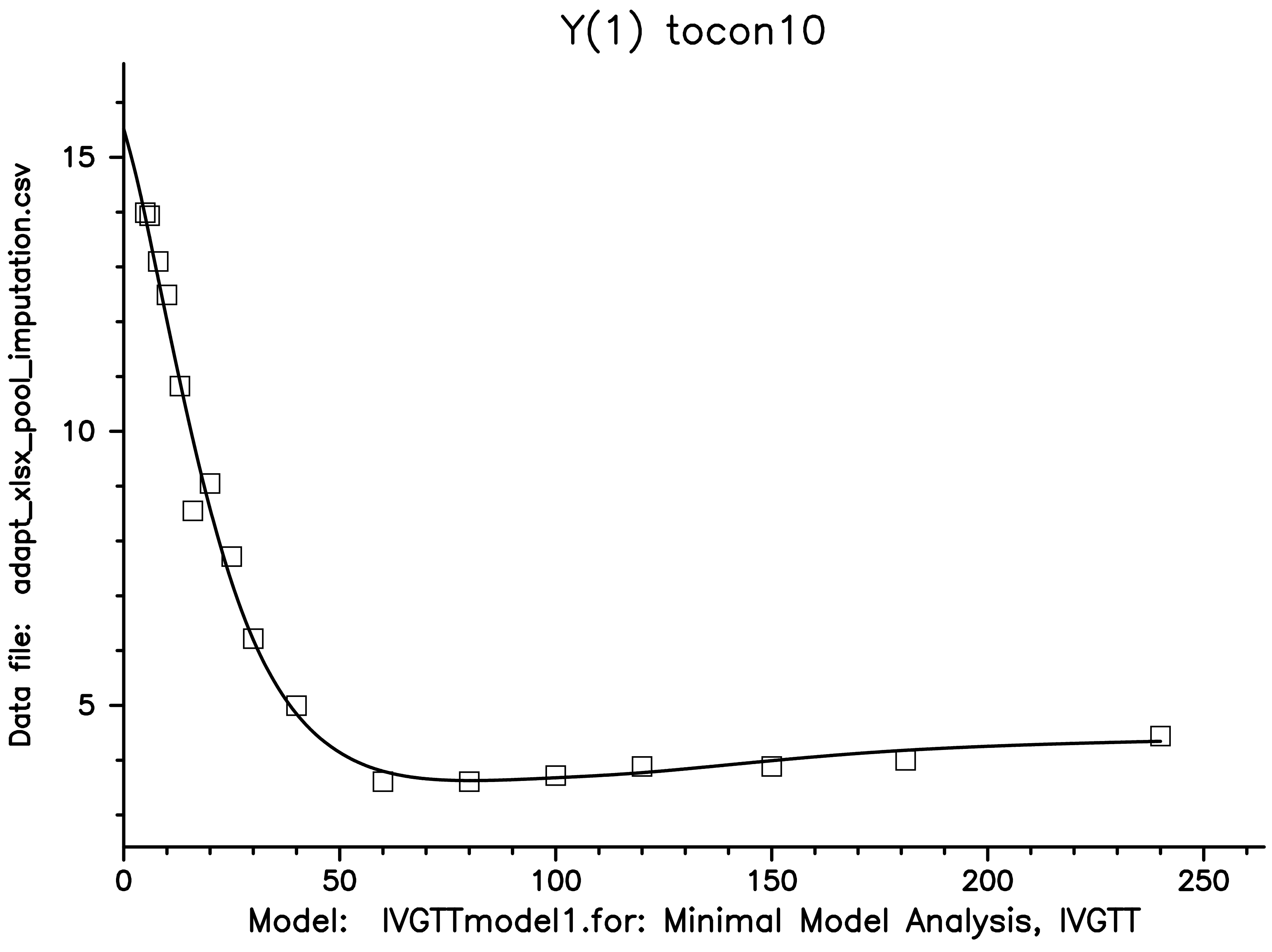
Y(1) tocon10
Data file: adapt_xlsx_pool_imputation.csv
Model: IVGTTmodel1.for: Minimal Model Analysis, IVGTT
15
10
5
0
50
100
150
200
250

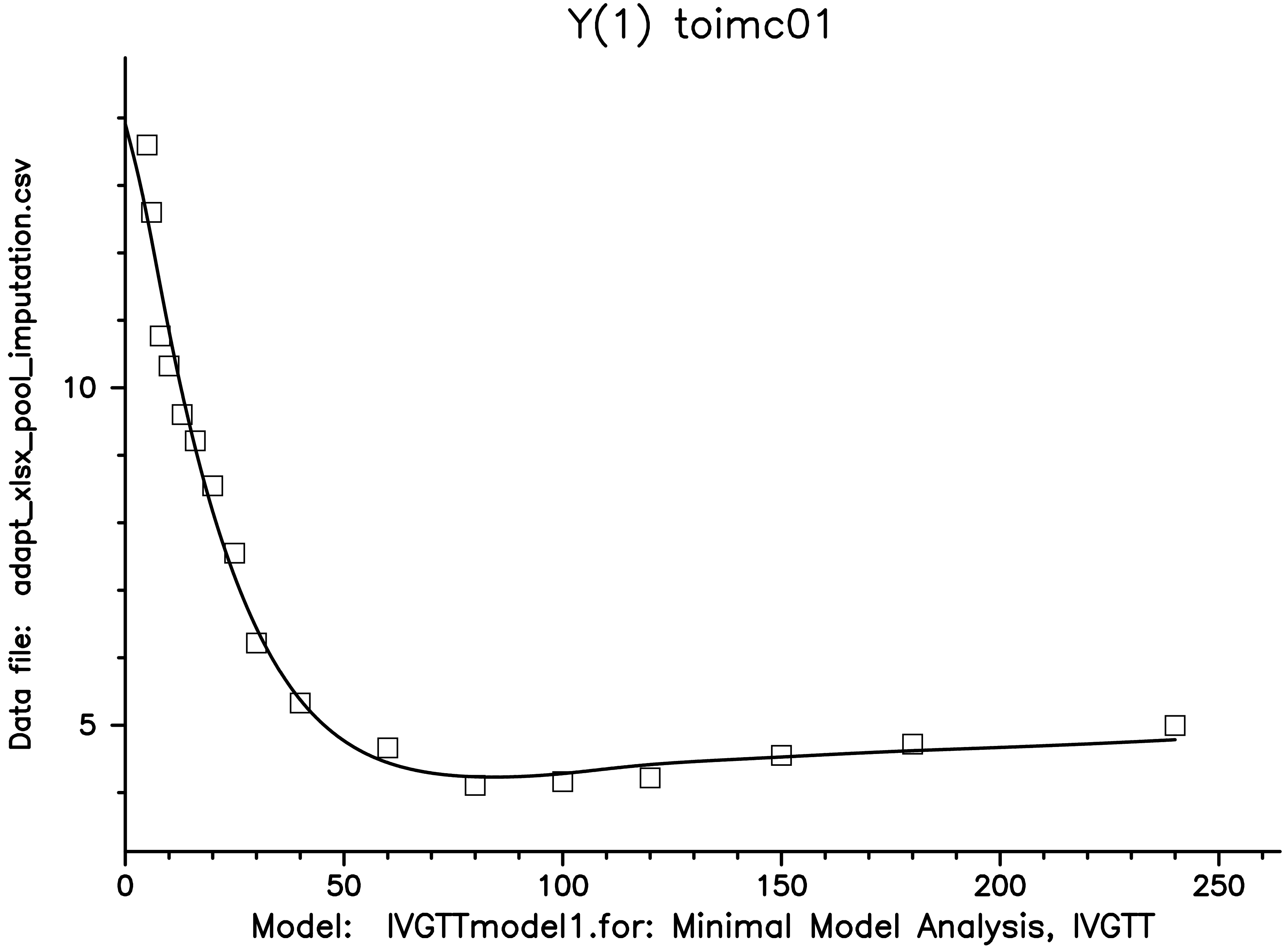

Y(1) toimc01
Data file: adapt_xlsx_pool_imputation.csv
10
5
0
50
100
150
200
250
Model: IVGTTmodel1.for: Minimal Model Analysis, IVGTT

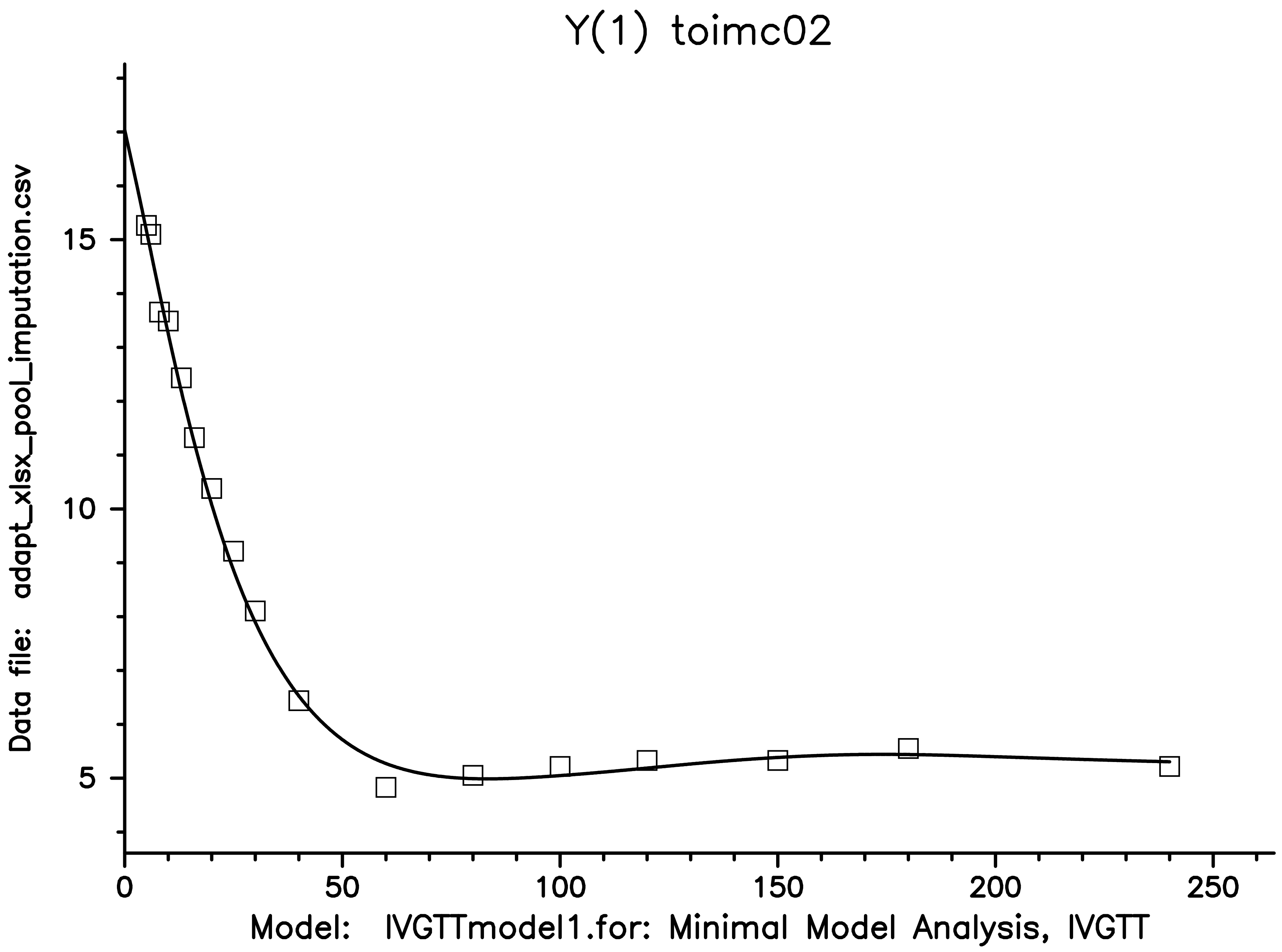

Y(1) toimc02
Data file: adapt_xlsx_pool_imputation.csv
15
10
5
0
50
100
150
200
250
Model: IVGTTmodel1.for: Minimal Model Analysis, IVGTT

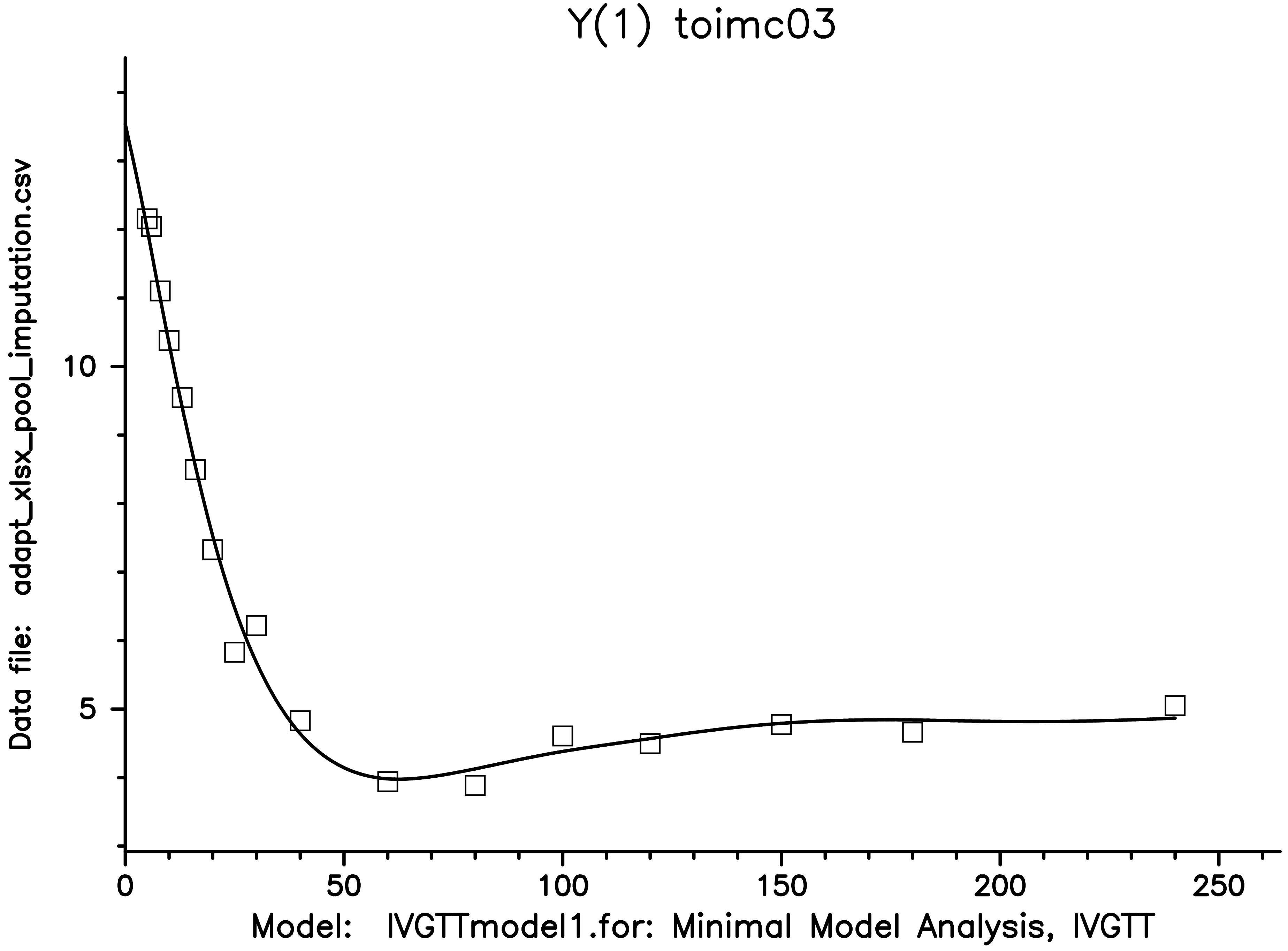
Y(1) toimc03
Data file: adapt_xlsx_pool_imputation.csv
10
5
0
50
100
150
200
250
Model: IVGTTmodel1.for: Minimal Model Analysis, IVGTT

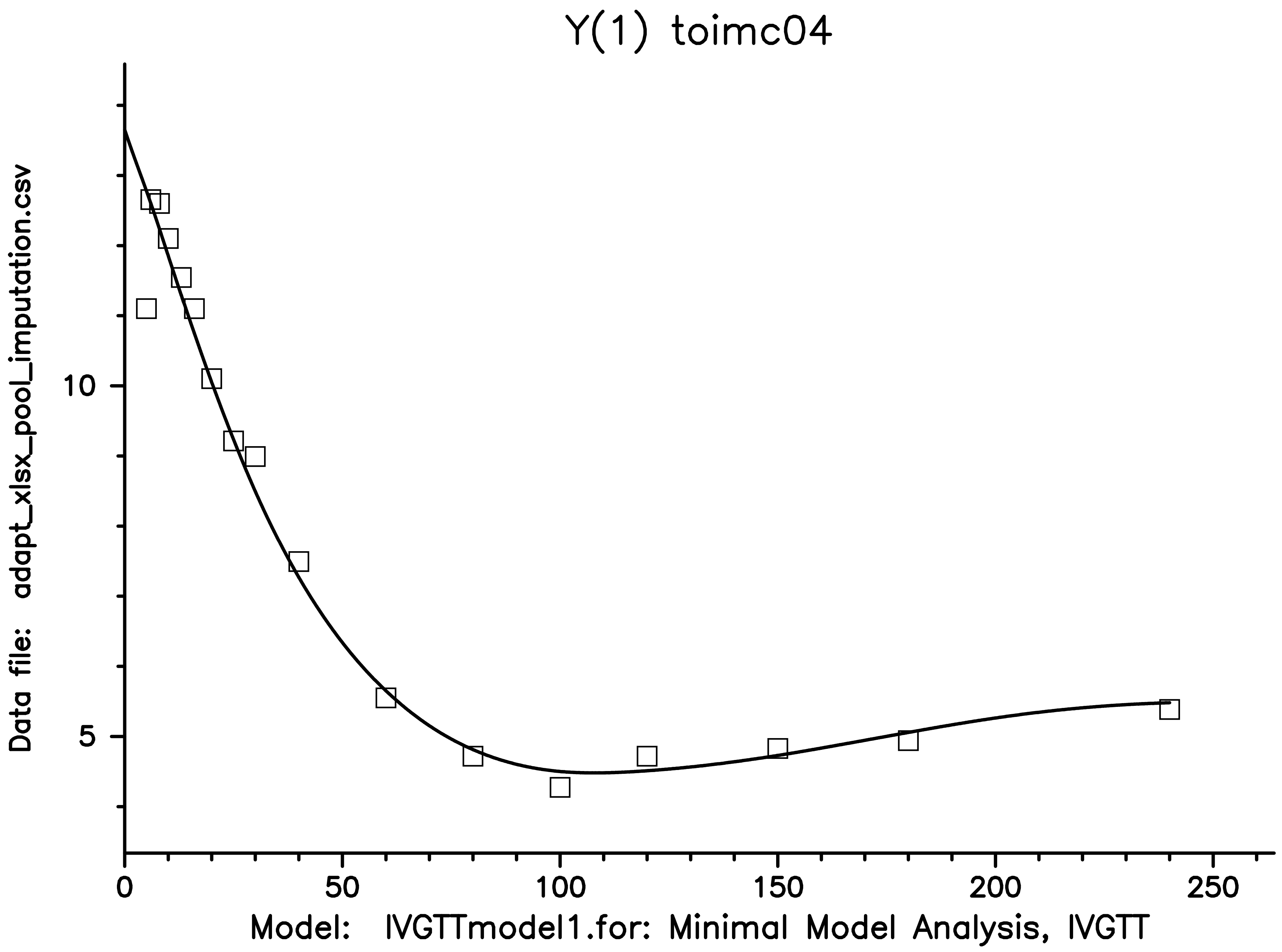
Y(1) toimc04
Data file: adapt_xlsx_pool_imputation.csv
10
5
0
50
100
150
200
250
Model: IVGTTmodel1.for: Minimal Model Analysis, IVGTT

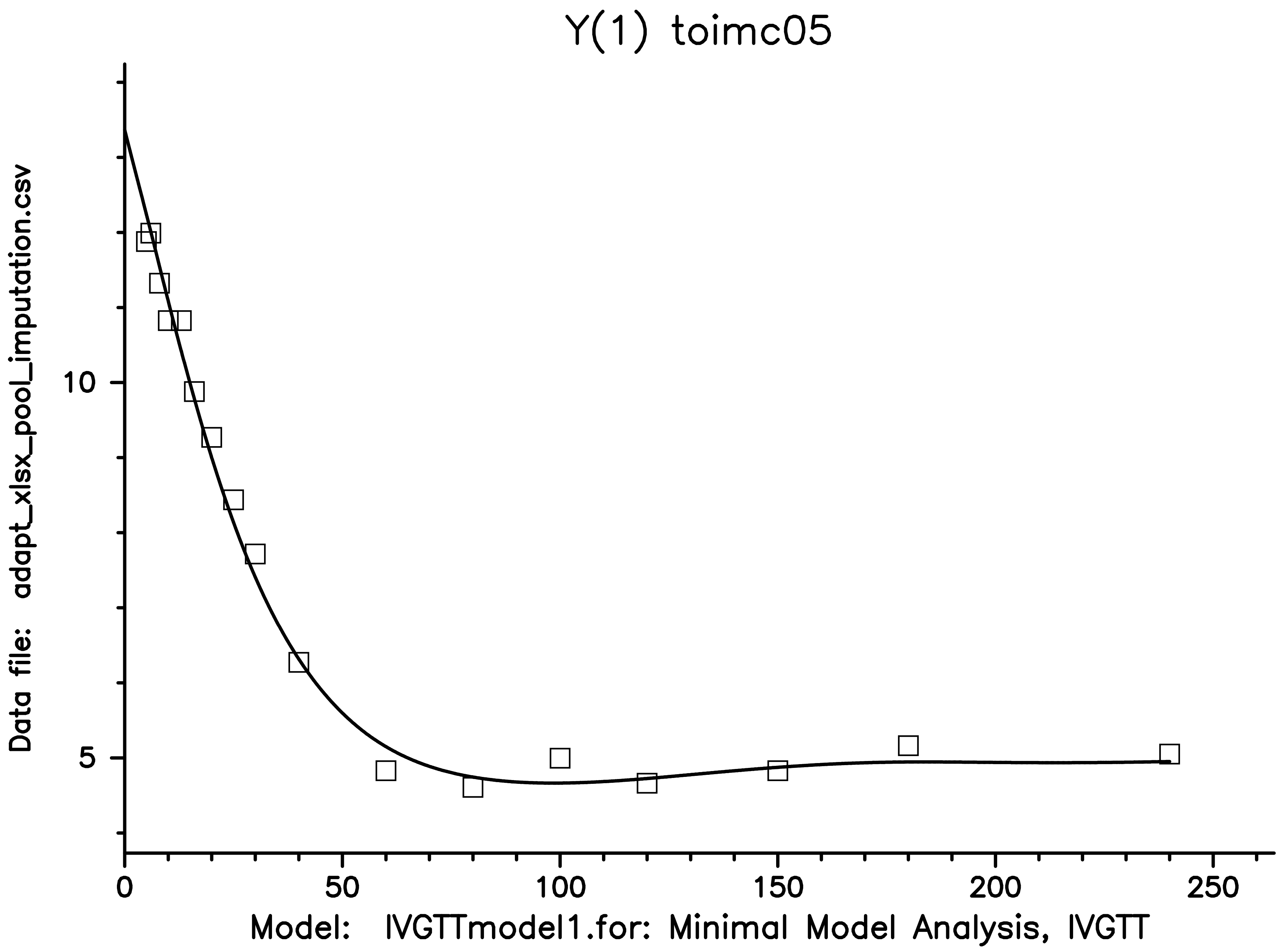

Y(1) toimc05
Data file: adapt_xlsx_pool_imputation.csv
10
5
0
50
100
150
200
250
Model: IVGTTmodel1.for: Minimal Model Analysis, IVGTT

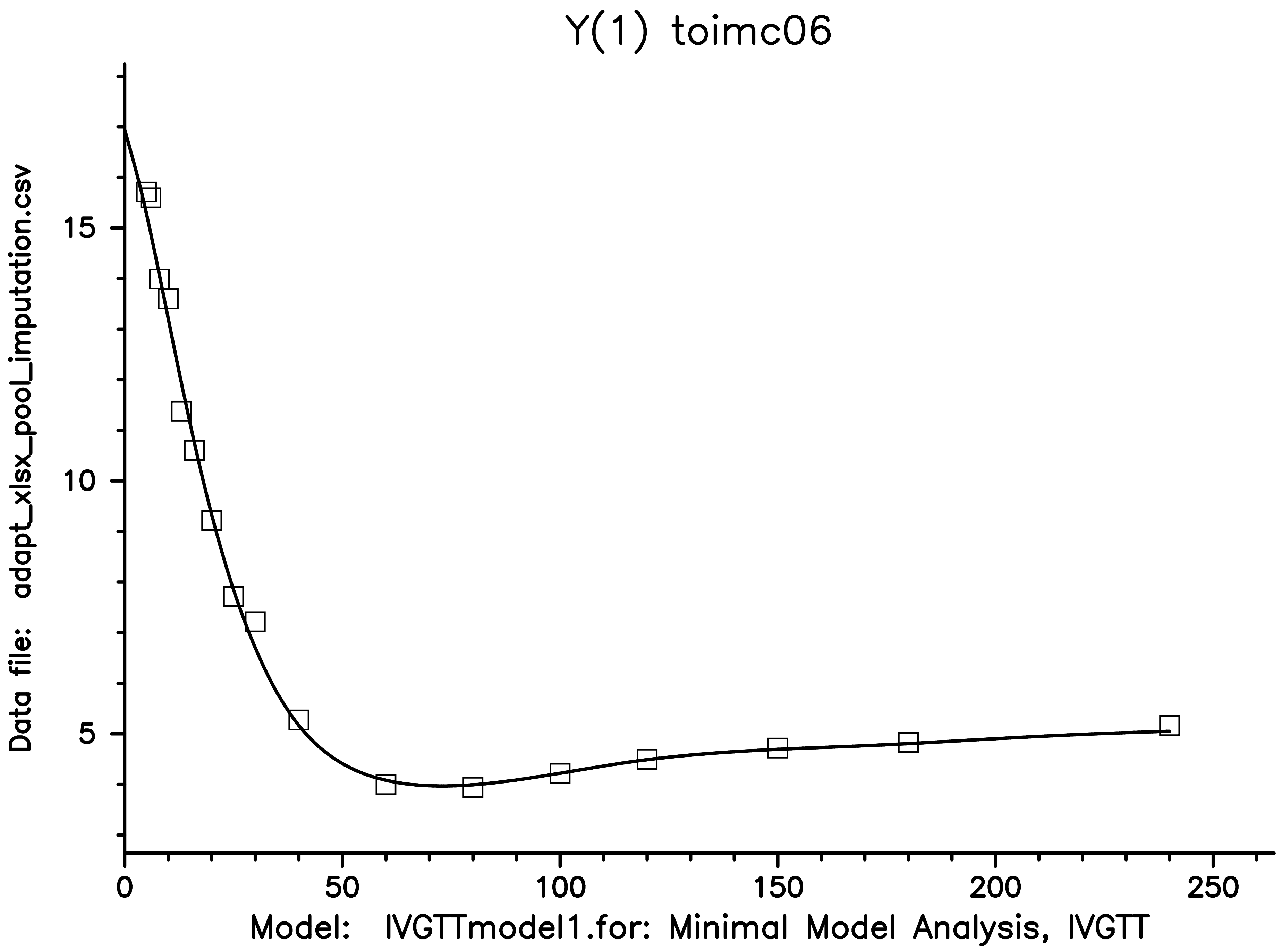
Y(1) toimc06
Data file: adapt_xlsx_pool_imputation.csv
15
10
5
0
50
100
150
200
250
Model: IVGTTmodel1.for: Minimal Model Analysis, IVGTT

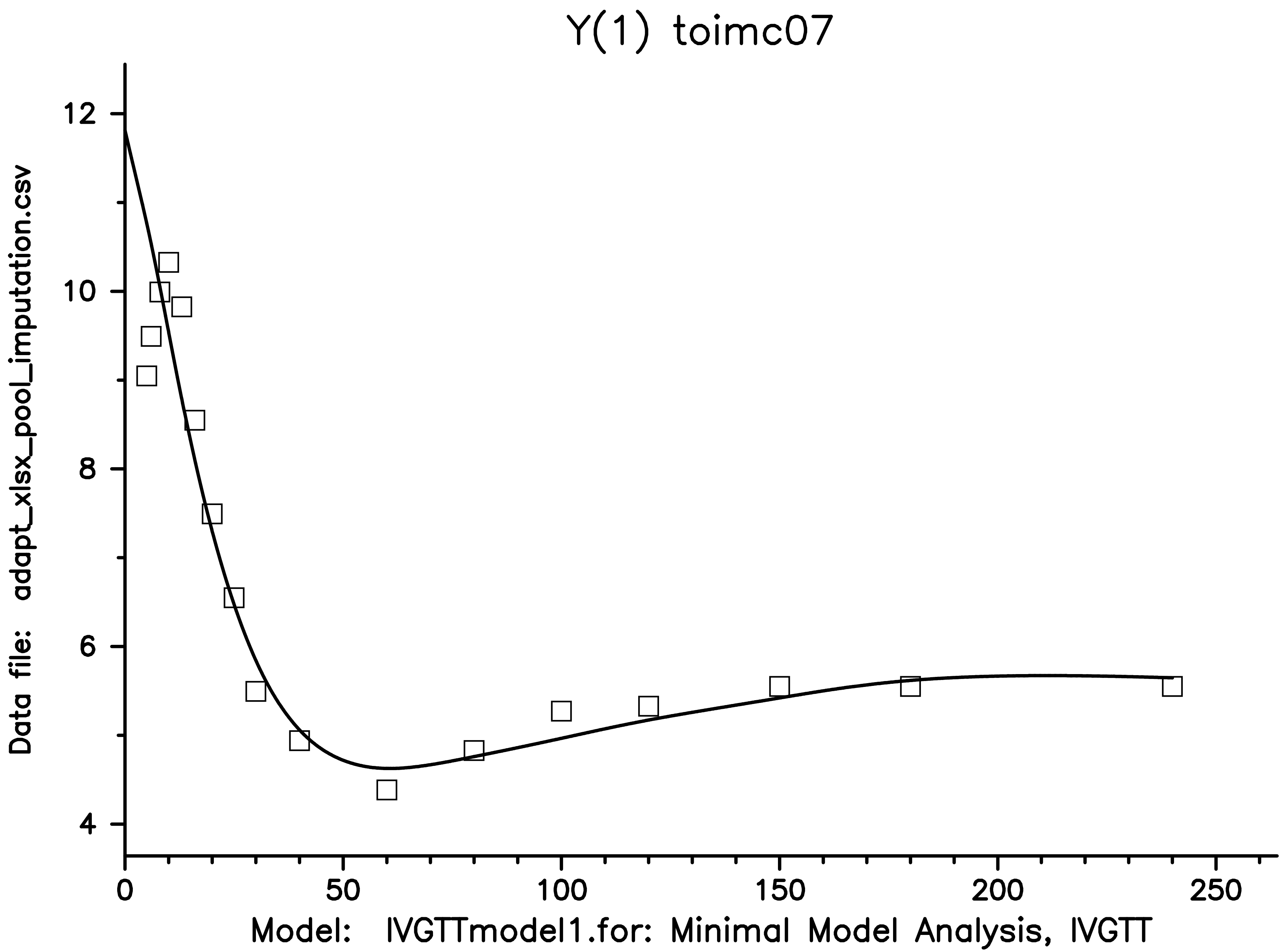
Y(1) toimc07
Data file: adapt_xlsx_pool_imputation.csv
Model: IVGTTmodel1.for: Minimal Model Analysis, IVGTT
12
10
8
6
4
0
50
100
150
200
250

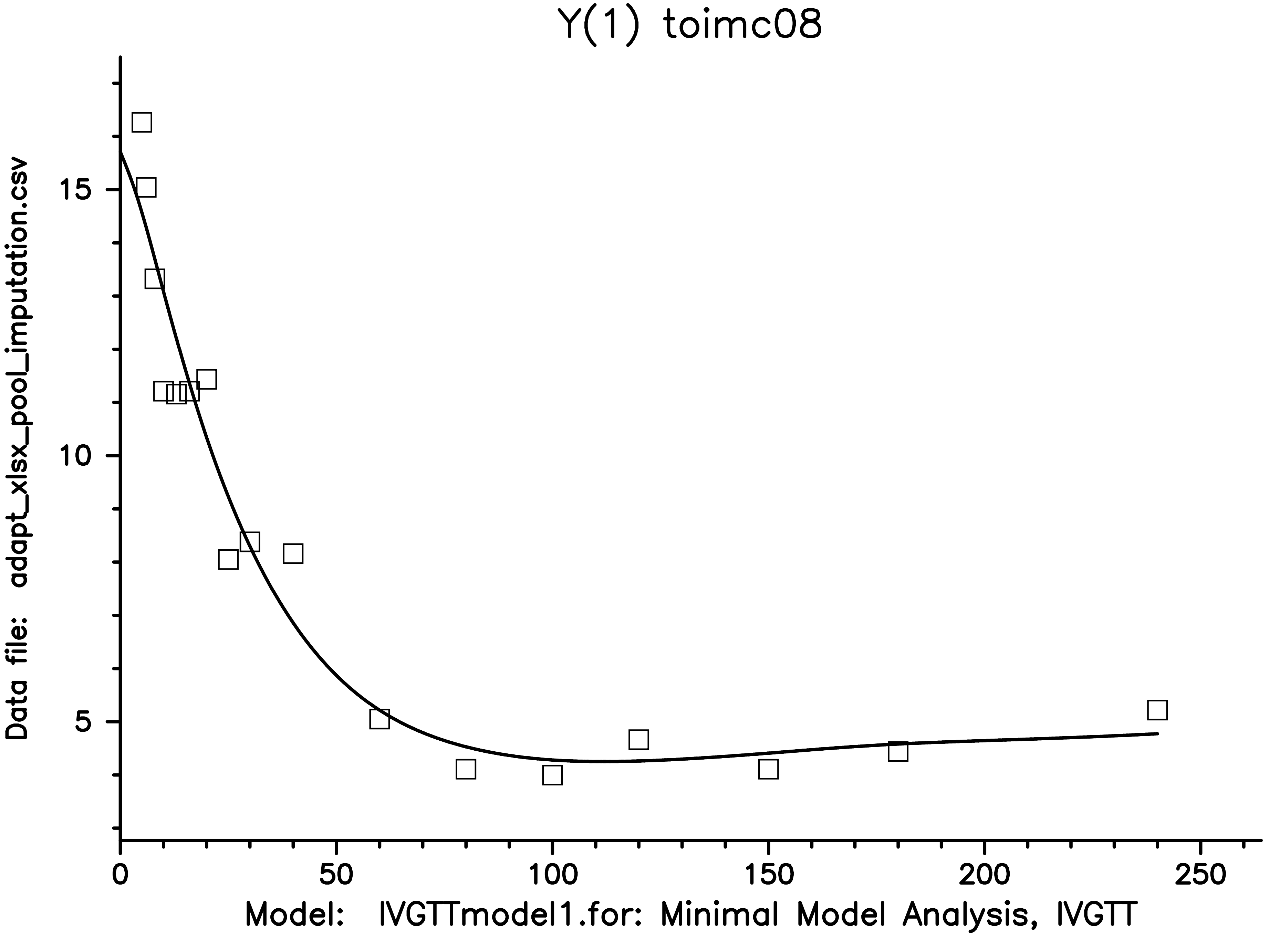

Y(1) toimc08
Data file: adapt_xlsx_pool_imputation.csv
15
10
5
0
50
100
150
200
250
Model: IVGTTmodel1.for: Minimal Model Analysis, IVGTT

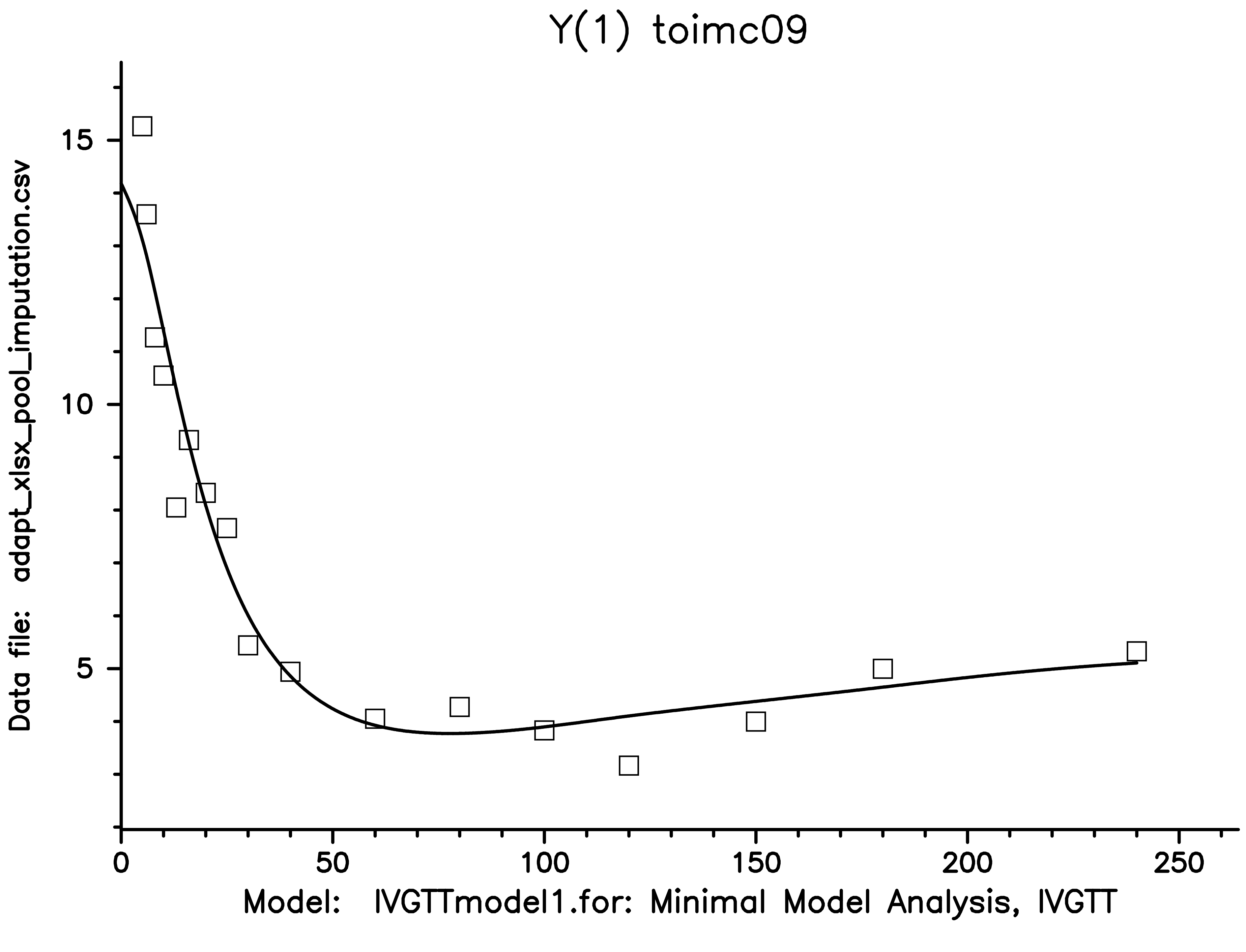

Y(1) toimc09
Data file: adapt_xlsx_pool_imputation.csv
15
10
5
0
50
100
150
200
250
Model: IVGTTmodel1.for: Minimal Model Analysis, IVGTT

Y(1) toimc10

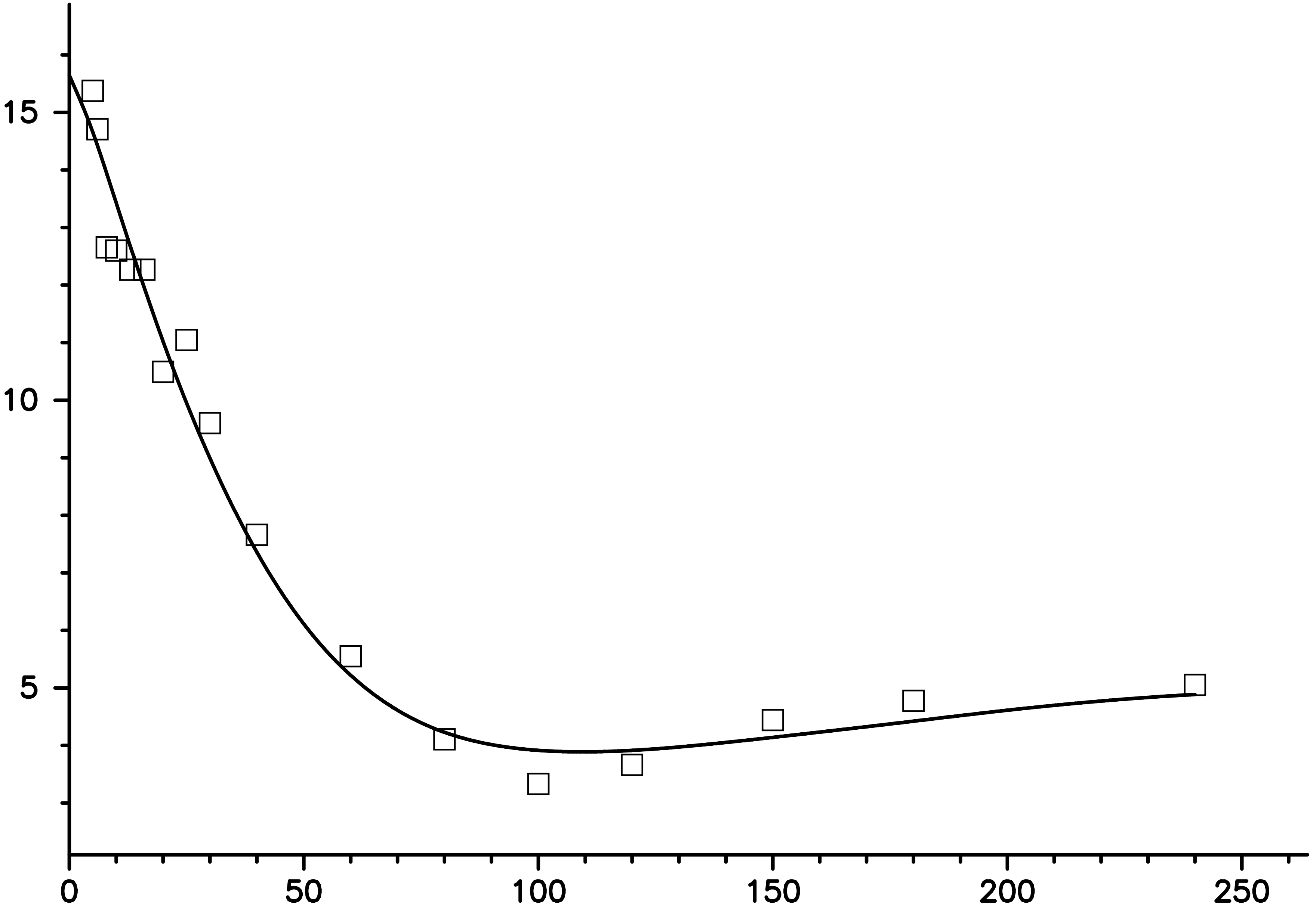

Data file: adapt_xlsx_pool_imputation.csv

Model: IVGTTmodel1.for: Minimal Model Analysis, IVGTT

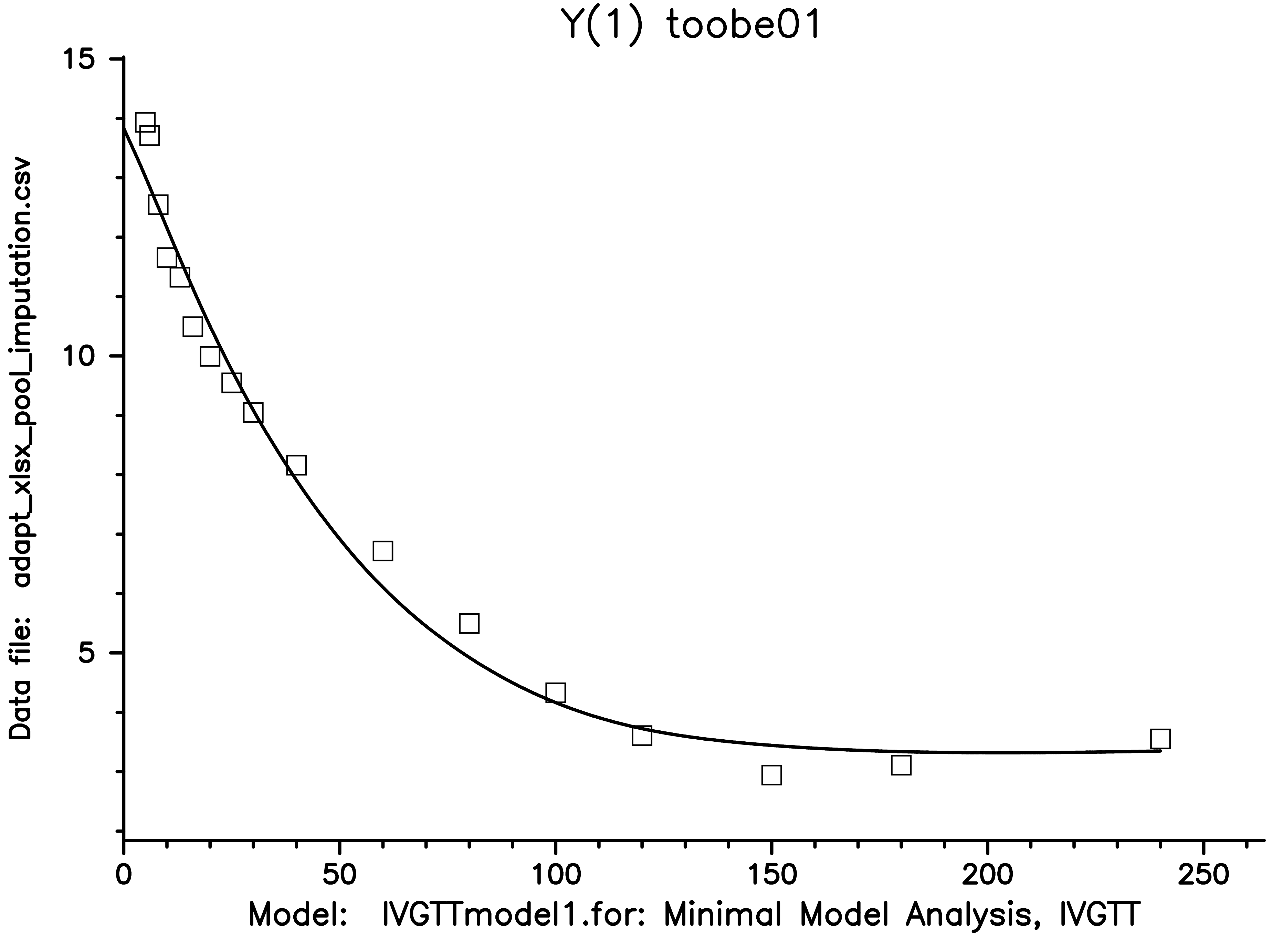

Y(1) toobe01
Data file: adapt_xlsx_pool_imputation.csv
Model: IVGTTmodel1.for: Minimal Model Analysis, IVGTT
15
10
5
0
50
100
150
200
250

Y(1) toobe02

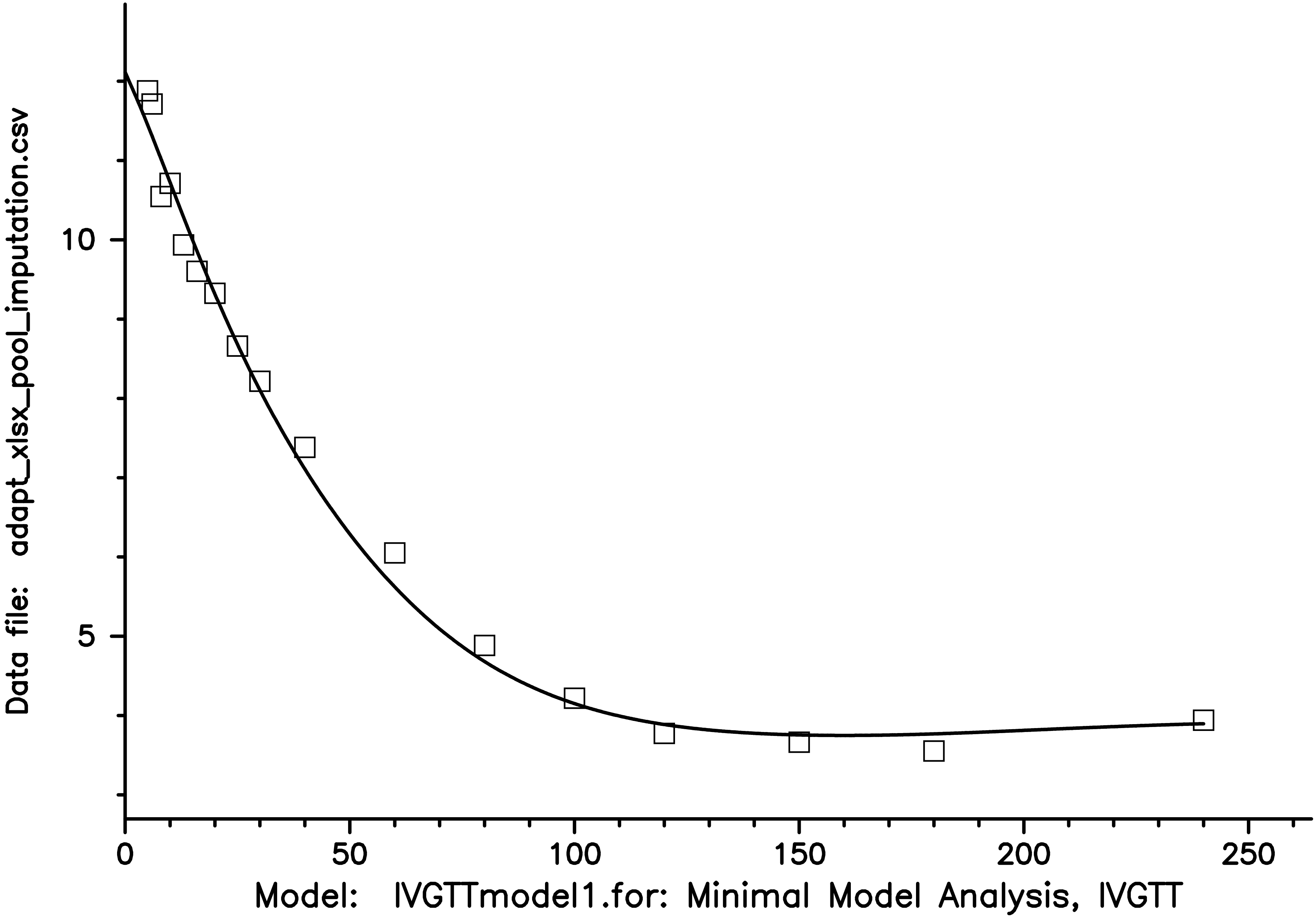
Data file: adapt_xlsx_pool_imputation.csv
10
5
0
50
100
150
200
250
Model: IVGTTmodel1.for: Minimal Model Analysis, IVGTT

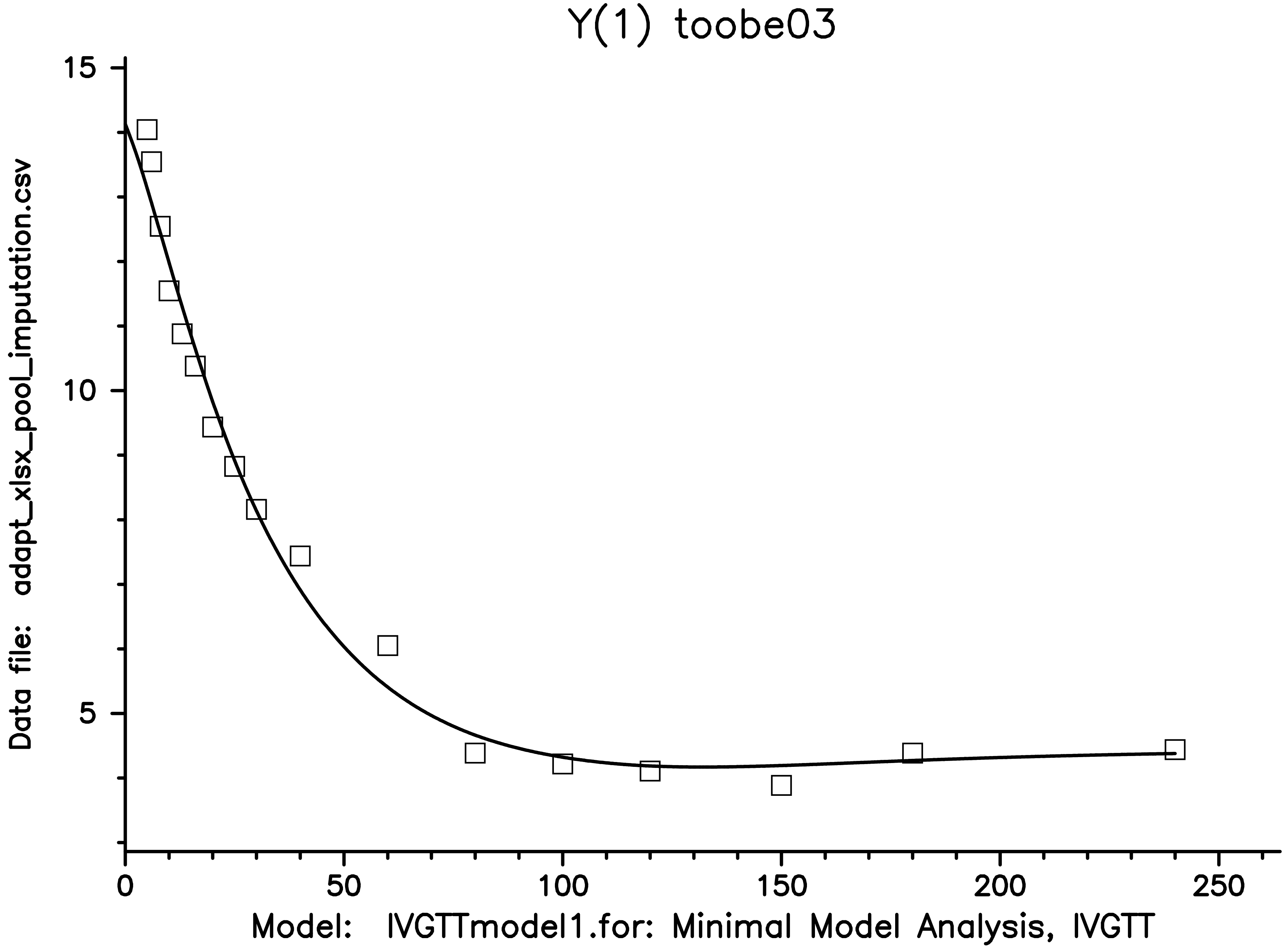

Y(1) toobe03
15
10
5
0
50
100
150
200
250
Data file: adapt_xlsx_pool_imputation.csv
Model: IVGTTmodel1.for: Minimal Model Analysis, IVGTT

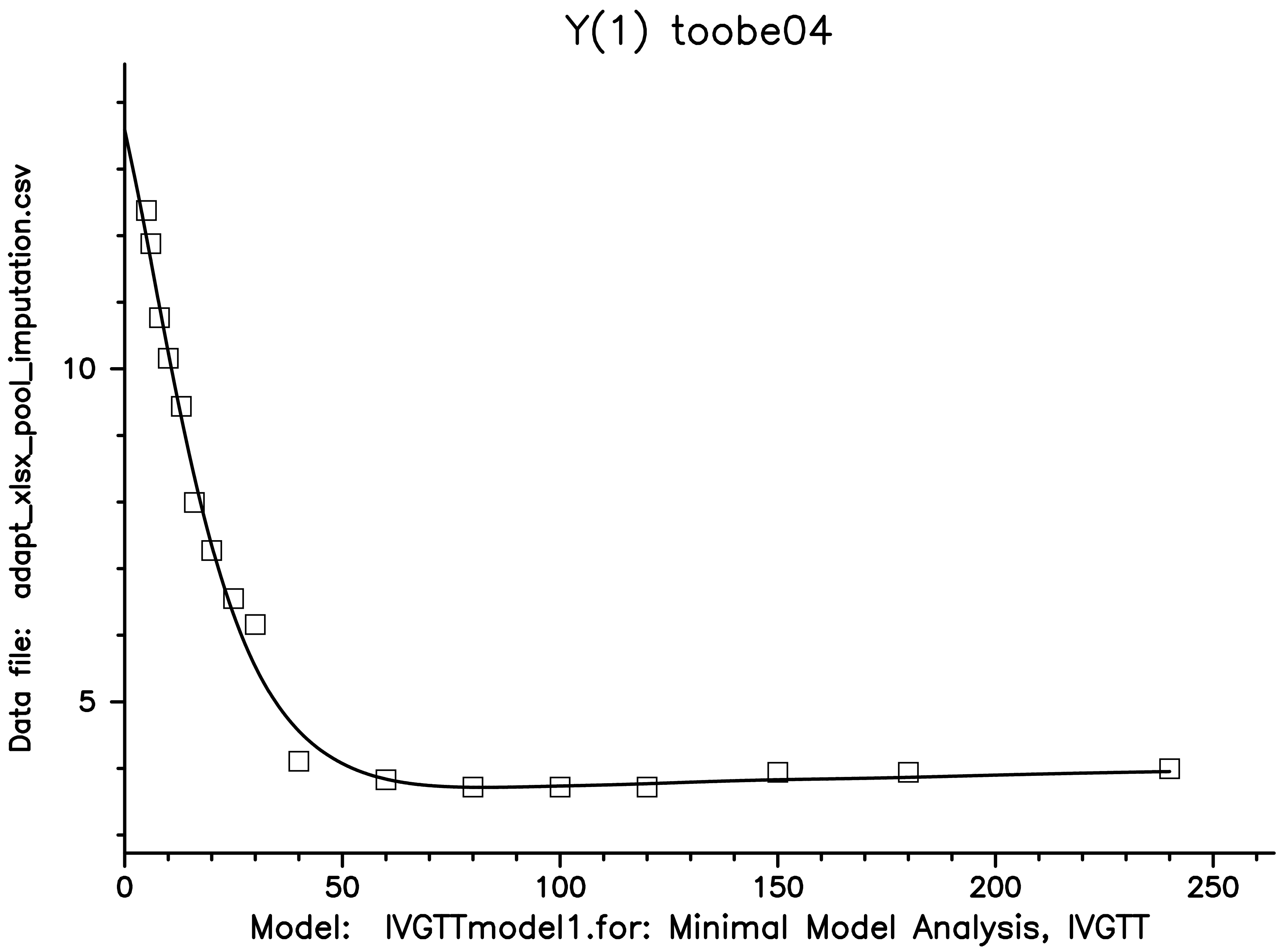

Y(1) toobe04
Data file: adapt_xlsx_pool_imputation.csv
10
5
0
50
100
150
200
250
Model: IVGTTmodel1.for: Minimal Model Analysis, IVGTT

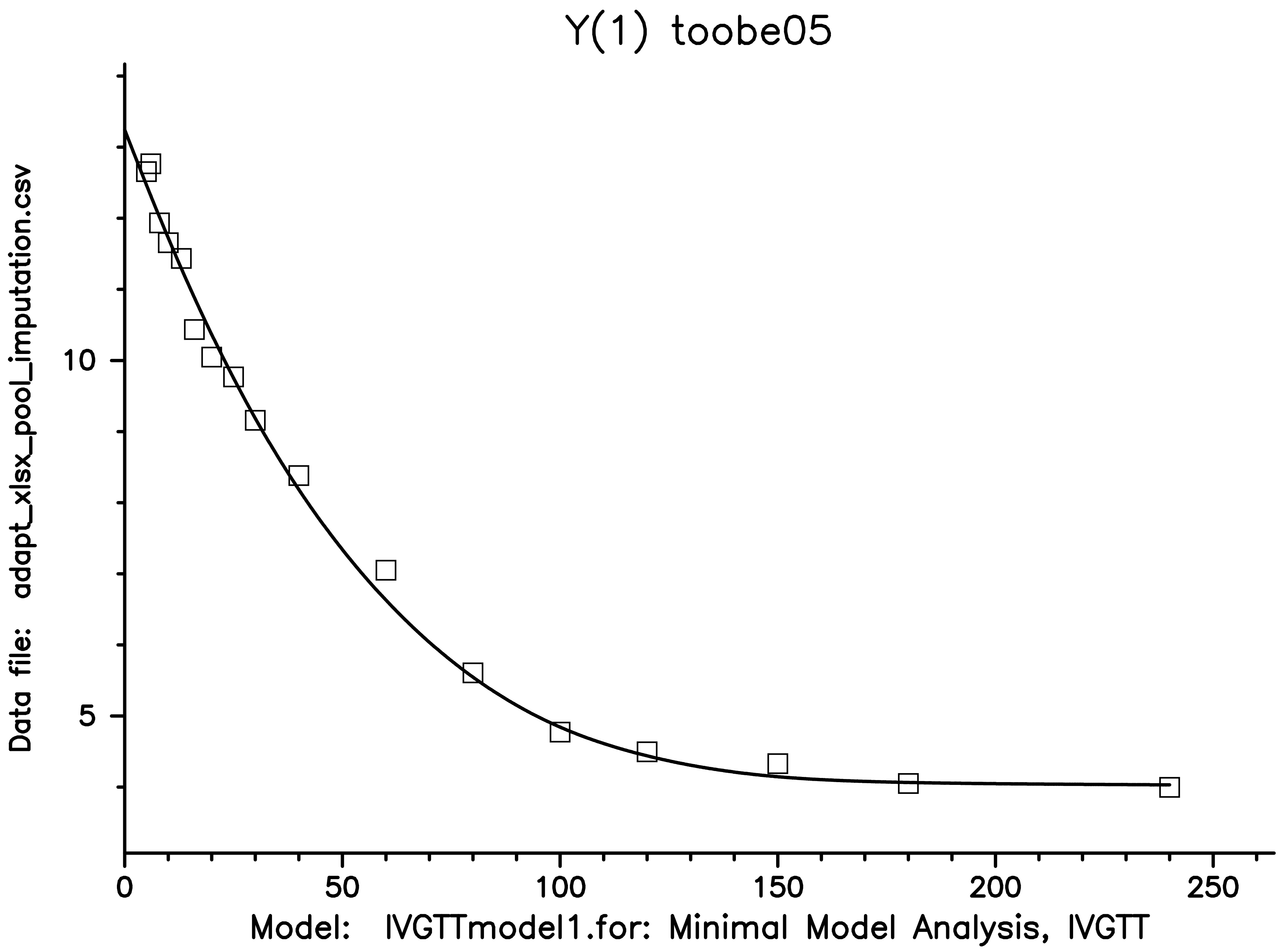
Y(1) toobe05
Data file: adapt_xlsx_pool_imputation.csv
10
5
0
50
100
150
200
250
Model: IVGTTmodel1.for: Minimal Model Analysis, IVGTT

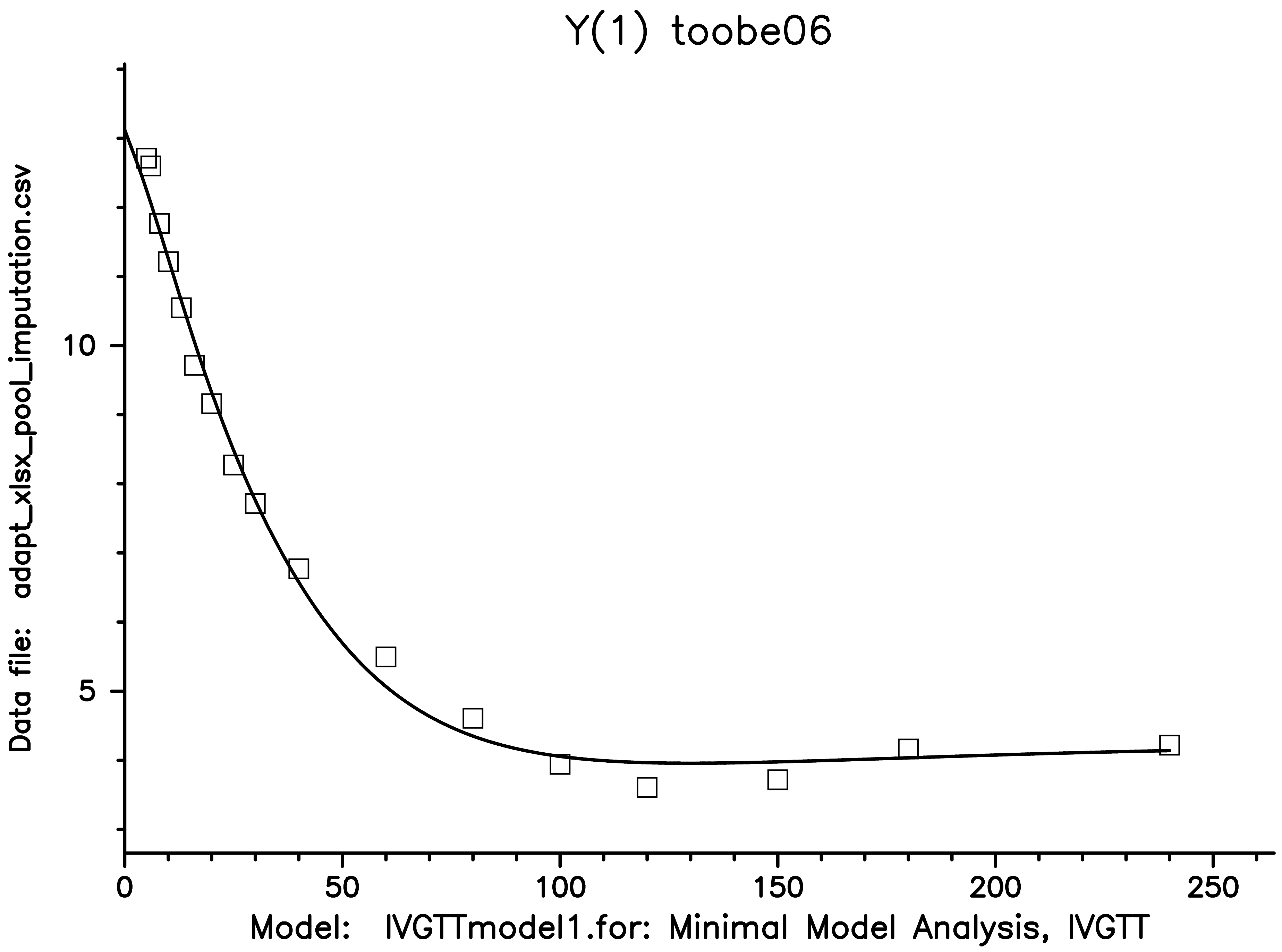

Y(1) toobe06
Data file: adapt_xlsx_pool_imputation.csv
10
5
0
50
100
150
200
250
Model: IVGTTmodel1.for: Minimal Model Analysis, IVGTT

Y(1) toobe07

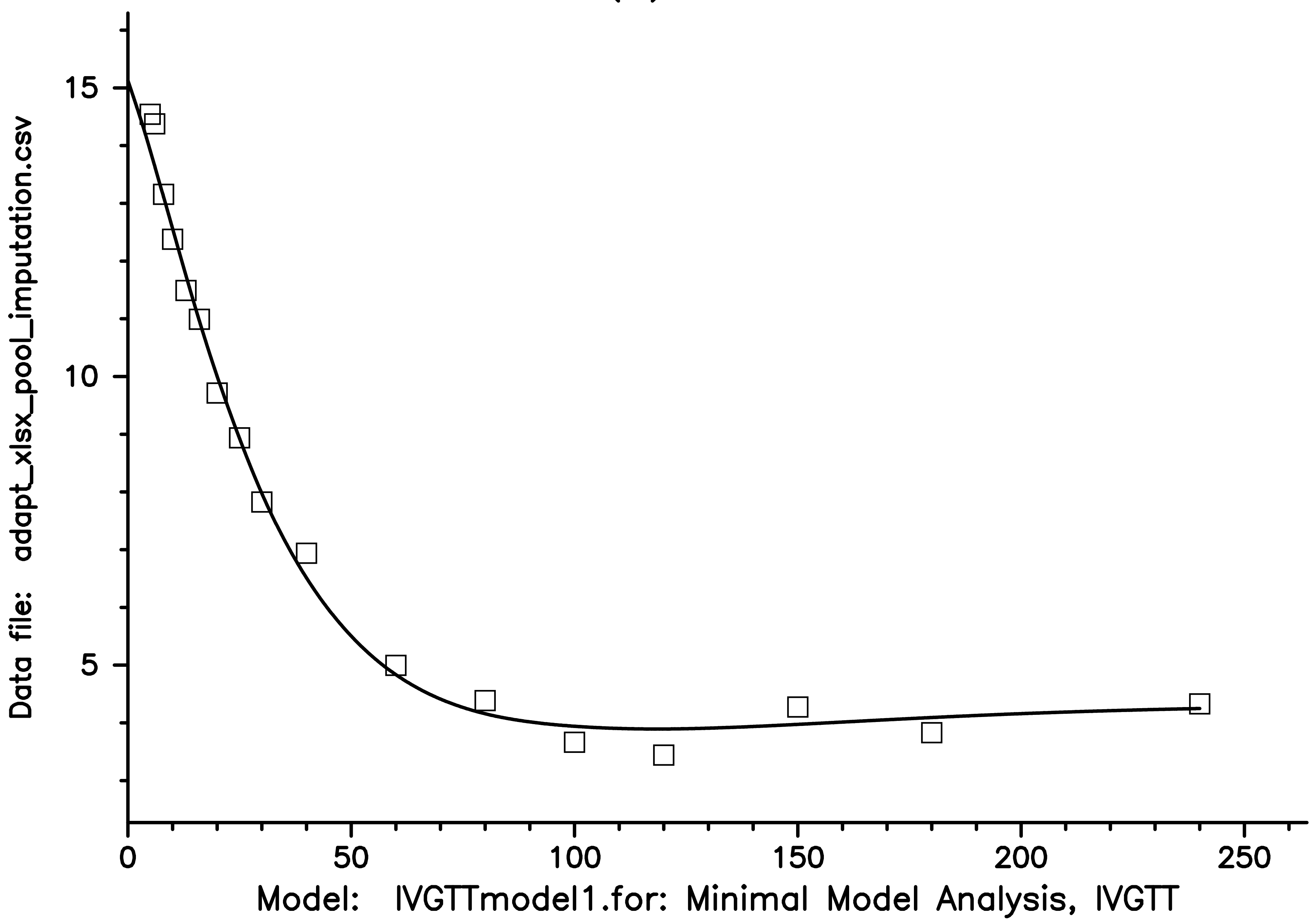
15
10
5
0
50
100
150
200
250
Data file: adapt_xlsx_pool_imputation.csv
Model: IVGTTmodel1.for: Minimal Model Analysis, IVGTT

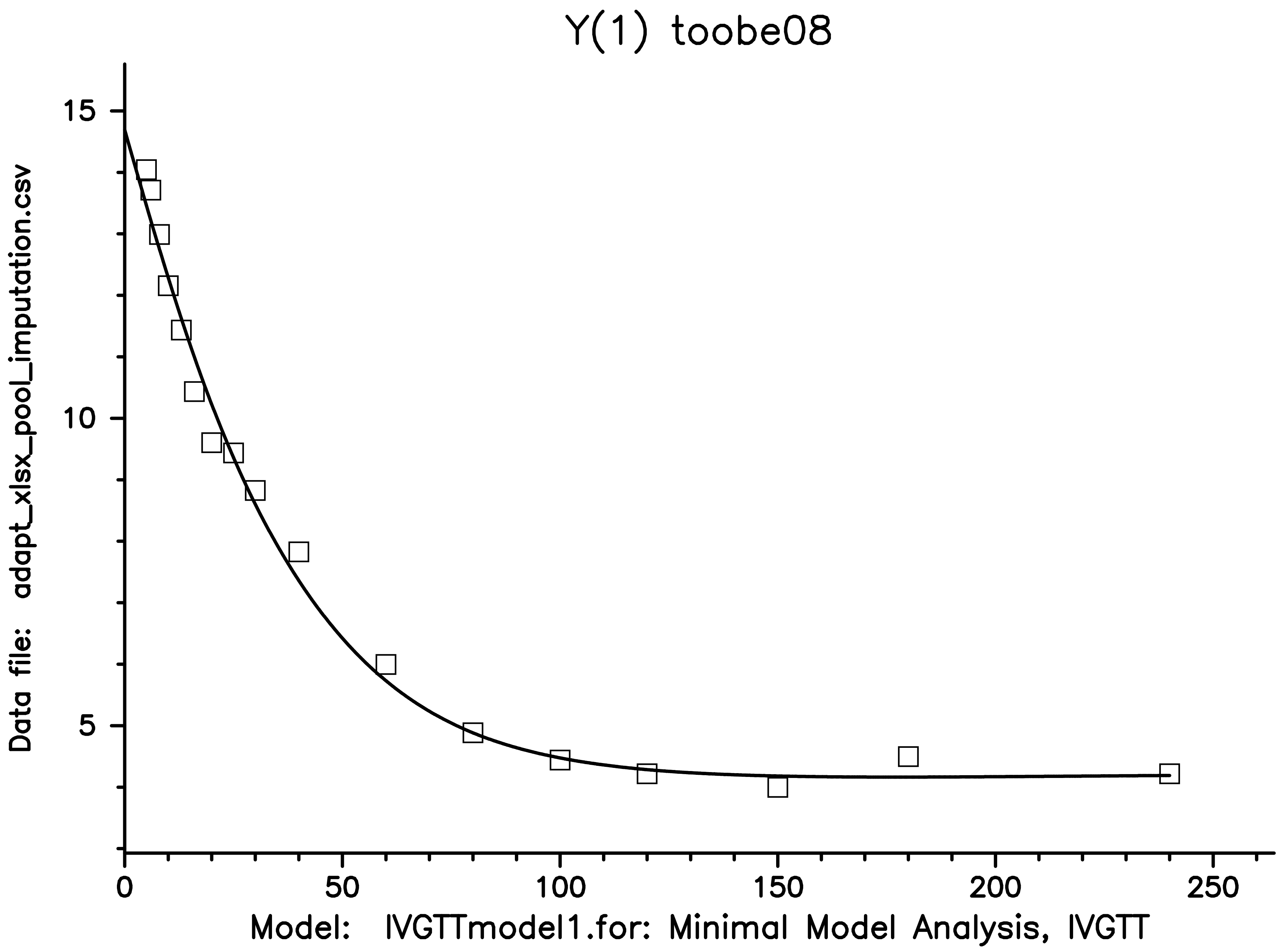
Y(1) toobe08
Data file: adapt_xlsx_pool_imputation.csv
15
10
5
0
50
100
150
200
250
Model: IVGTTmodel1.for: Minimal Model Analysis, IVGTT

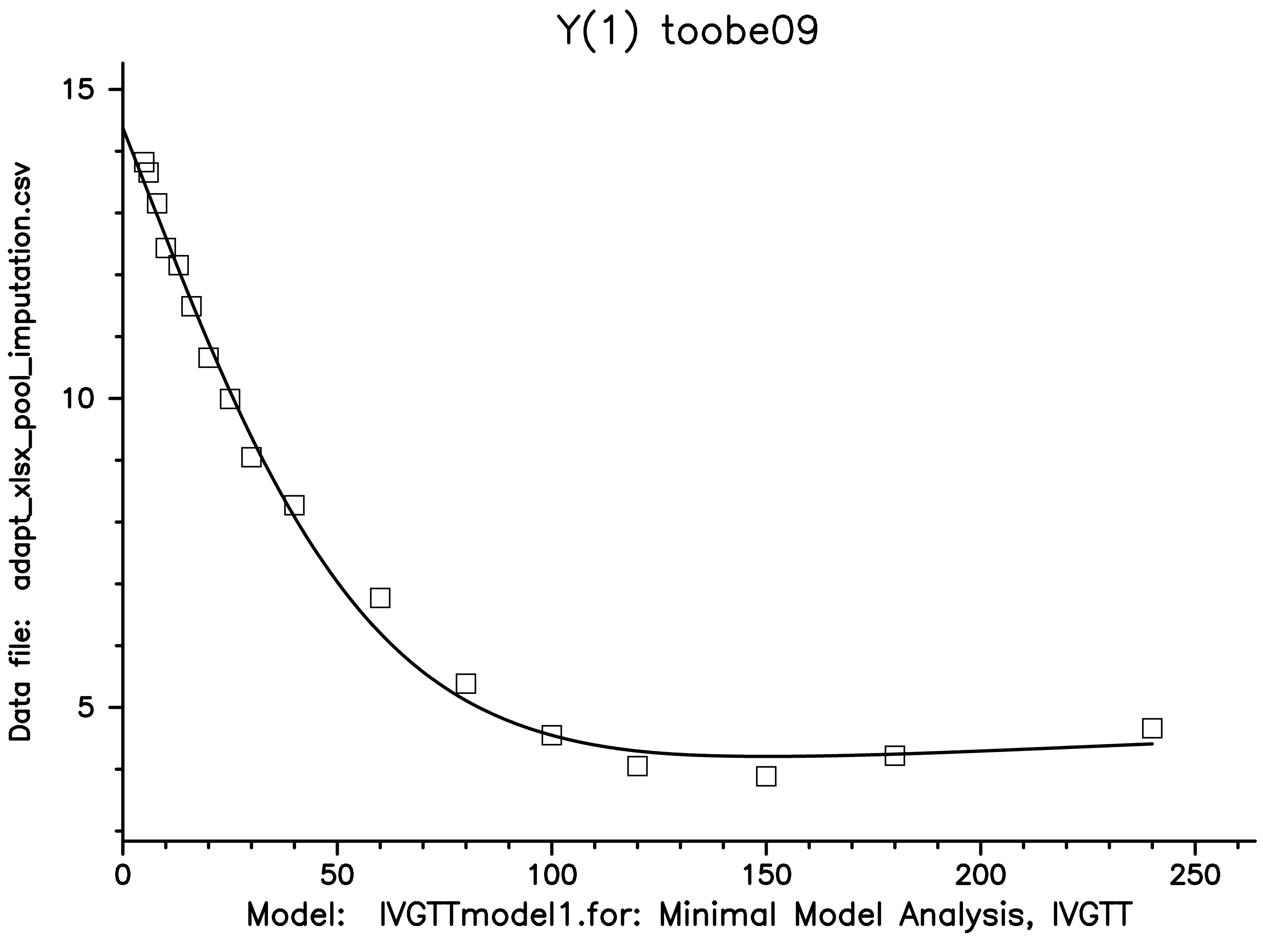

Y(1) toobe09
Data file: adapt_xlsx_pool_imputation.csv
Model: IVGTTmodel1.for: Minimal Model Analysis, IVGTT
15
10
5
0
50
100
150
200
250

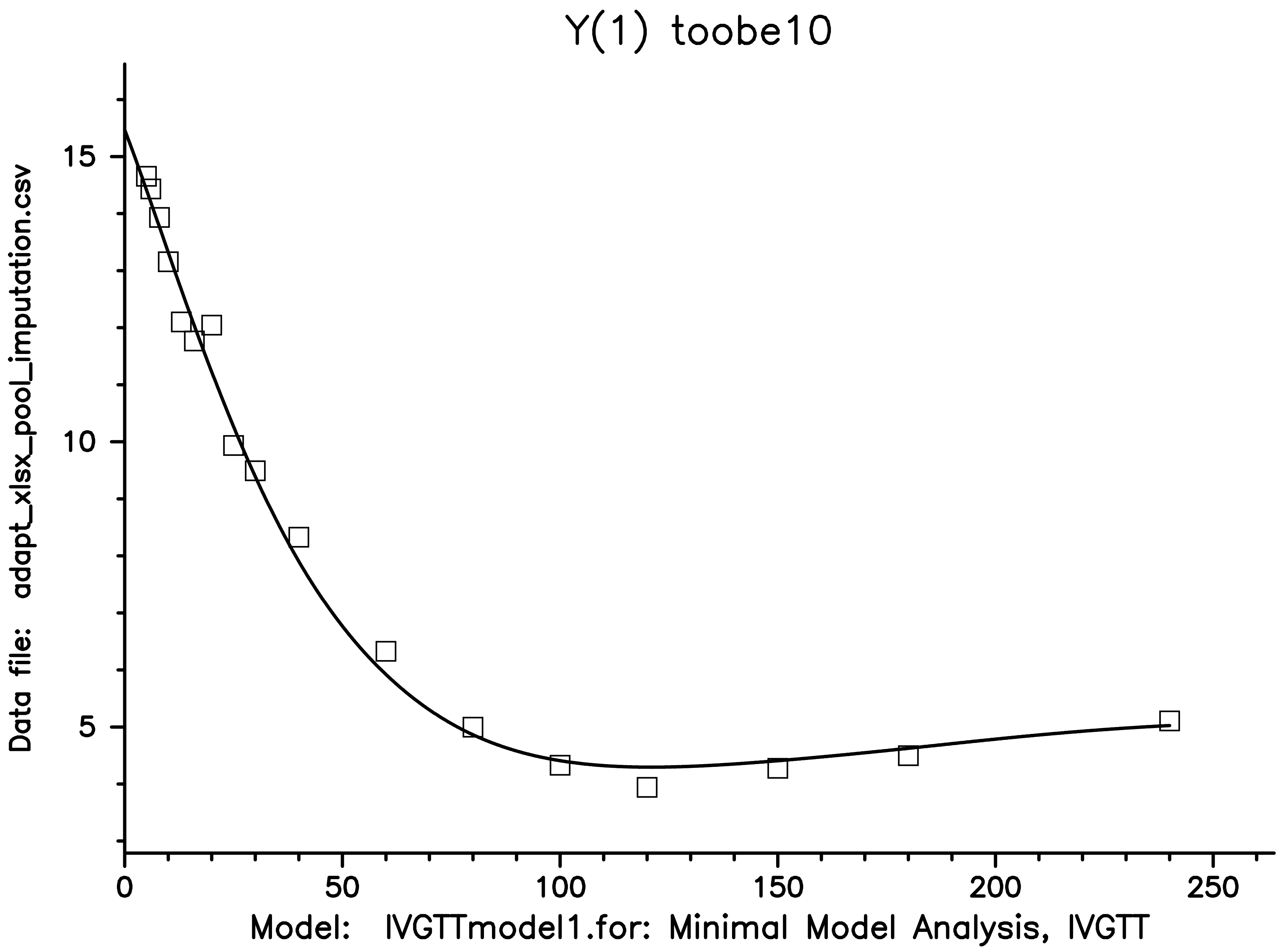
Y(1) toobe10
Data file: adapt_xlsx_pool_imputation.csv
Model: IVGTTmodel1.for: Minimal Model Analysis, IVGTT
15
10
5
0
50
100
150
200
250

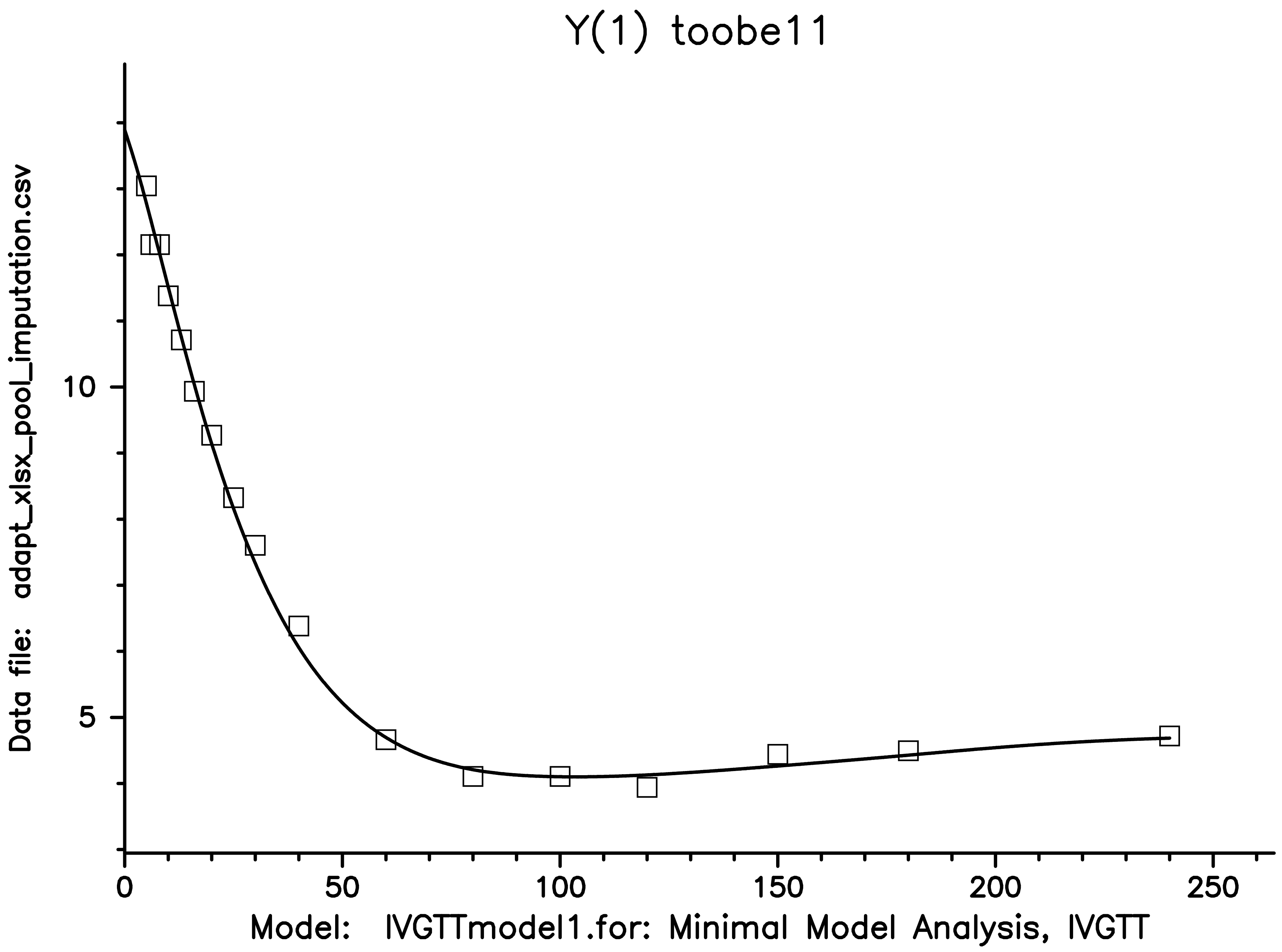

Y(1) toobe11
Data file: adapt_xlsx_pool_imputation.csv
10
5
0
50
100
150
200
250
Model: IVGTTmodel1.for: Minimal Model Analysis, IVGTT

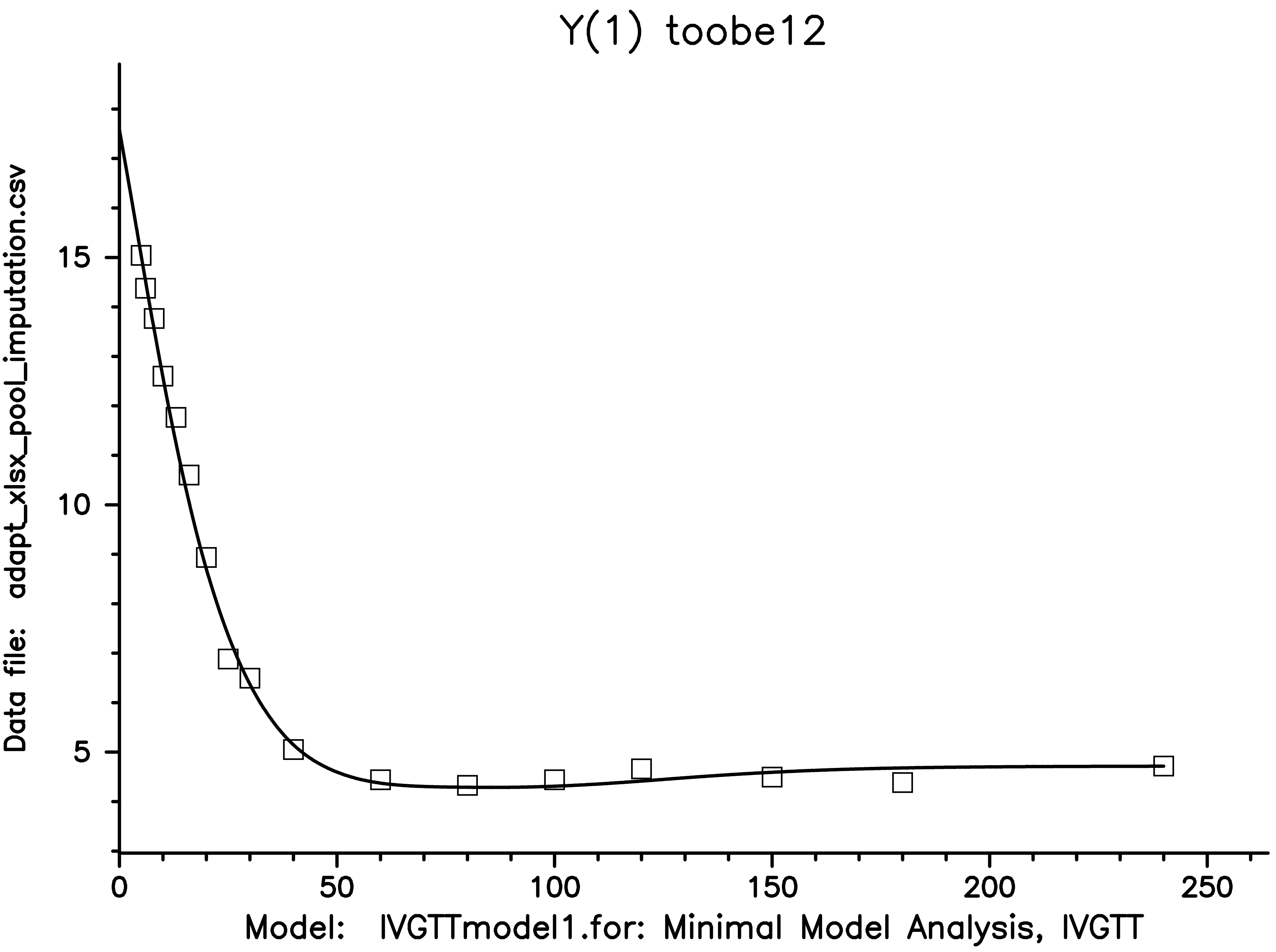
Y(1) toobe12
15
10
5
0
50
100
150
200
250
Data file: adapt_xlsx_pool_imputation.csv
Model: IVGTTmodel1.for: Minimal Model Analysis, IVGTT

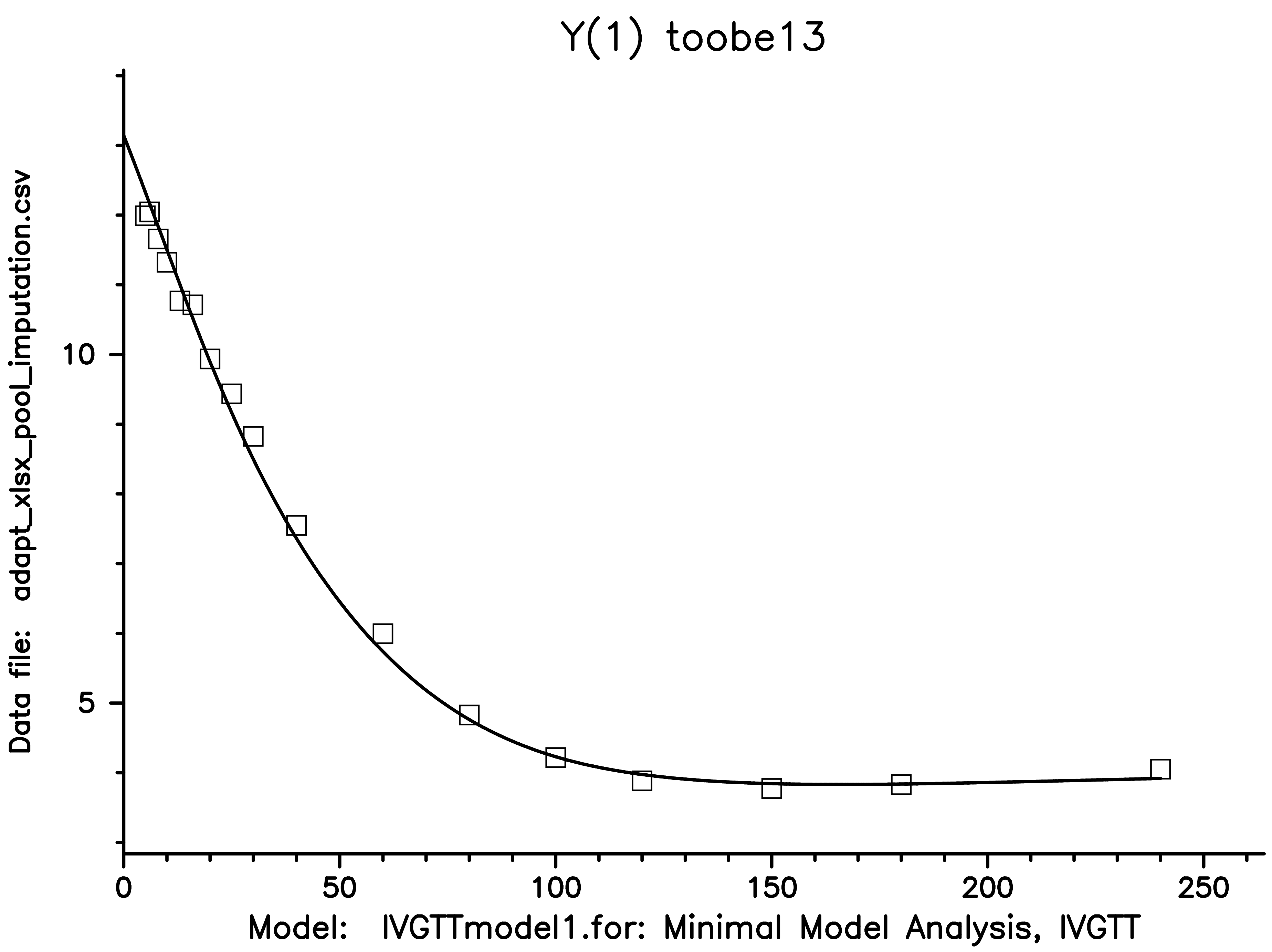
Y(1) toobe13
Data file: adapt_xlsx_pool_imputation.csv
10
5
0
50
100
150
200
250
Model: IVGTTmodel1.for: Minimal Model Analysis, IVGTT

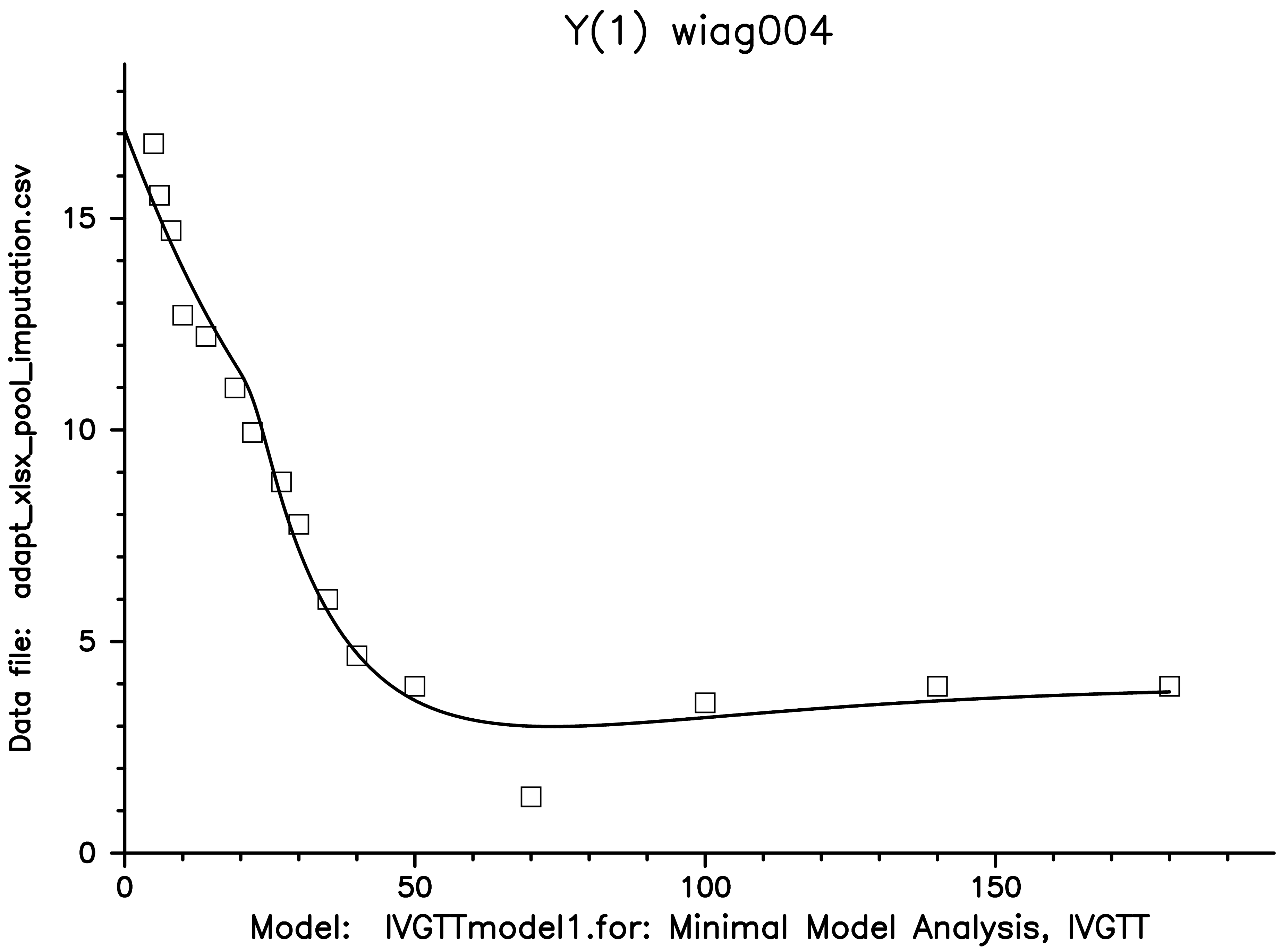

Y(1) wiag004
Data file: adapt_xlsx_pool_imputation.csv
0
5
10
15
0
50
100
150
Model: IVGTTmodel1.for: Minimal Model Analysis, IVGTT

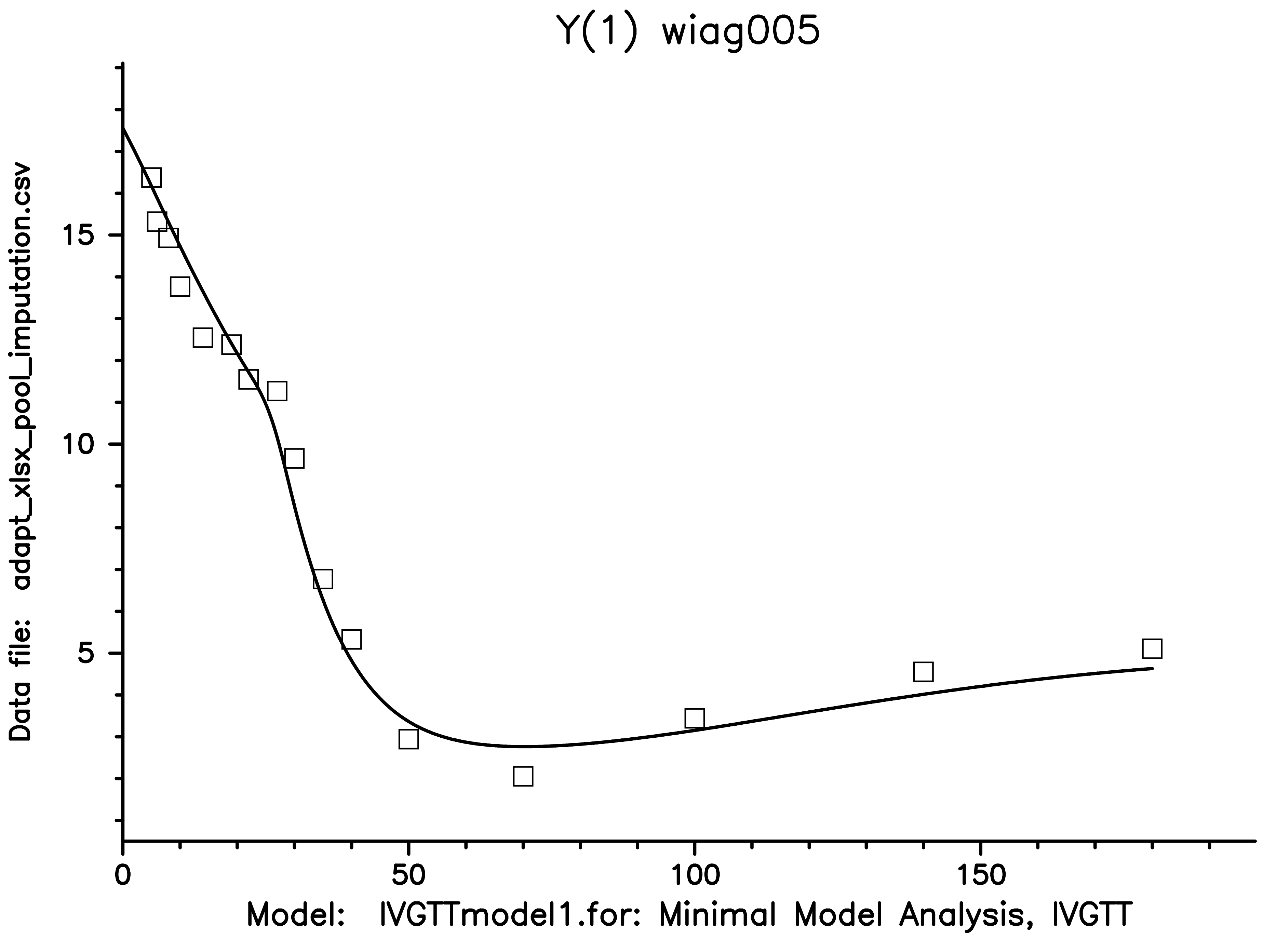
Y(1) wiag005
Data file: adapt_xlsx_pool_imputation.csv
15
10
5
0
50
100
150
Model: IVGTTmodel1.for: Minimal Model Analysis, IVGTT

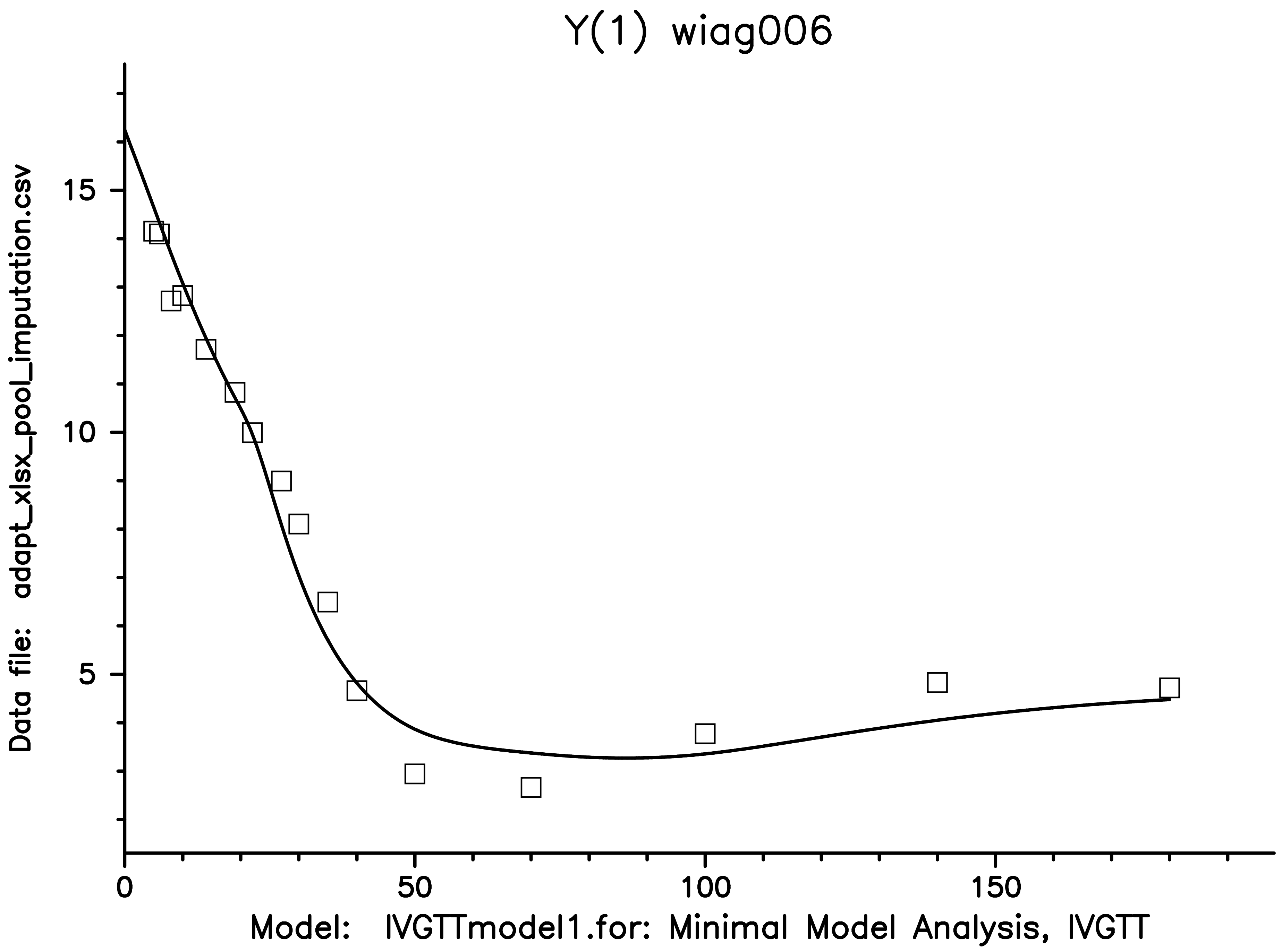
Y(1) wiag006
Data file: adapt_xlsx_pool_imputation.csv
15
10
5
0
50
100
150
Model: IVGTTmodel1.for: Minimal Model Analysis, IVGTT

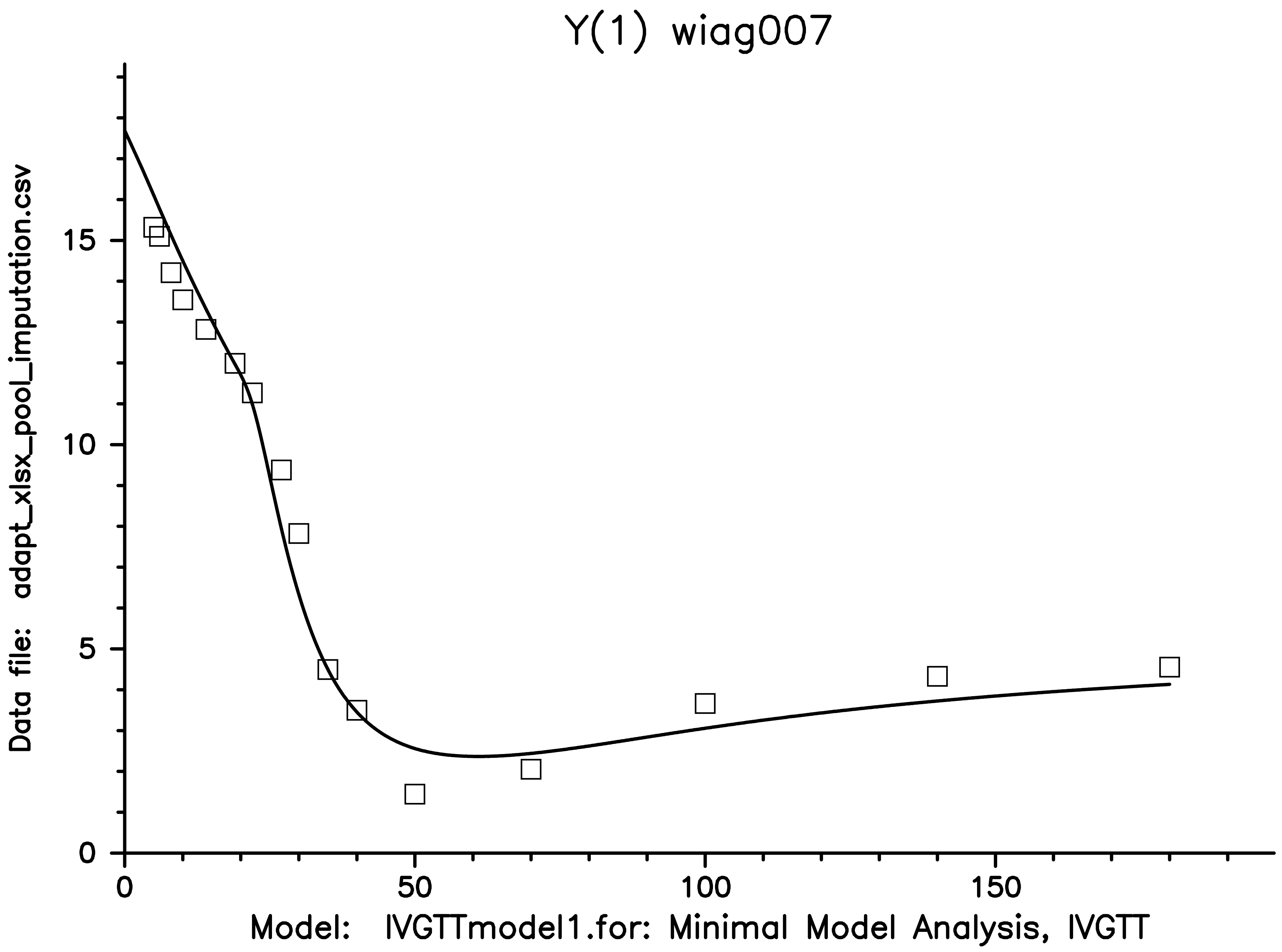

Y(1) wiag007
Data file: adapt_xlsx_pool_imputation.csv
Model: IVGTTmodel1.for: Minimal Model Analysis, IVGTT
0
5
10
15
0
50
100
150

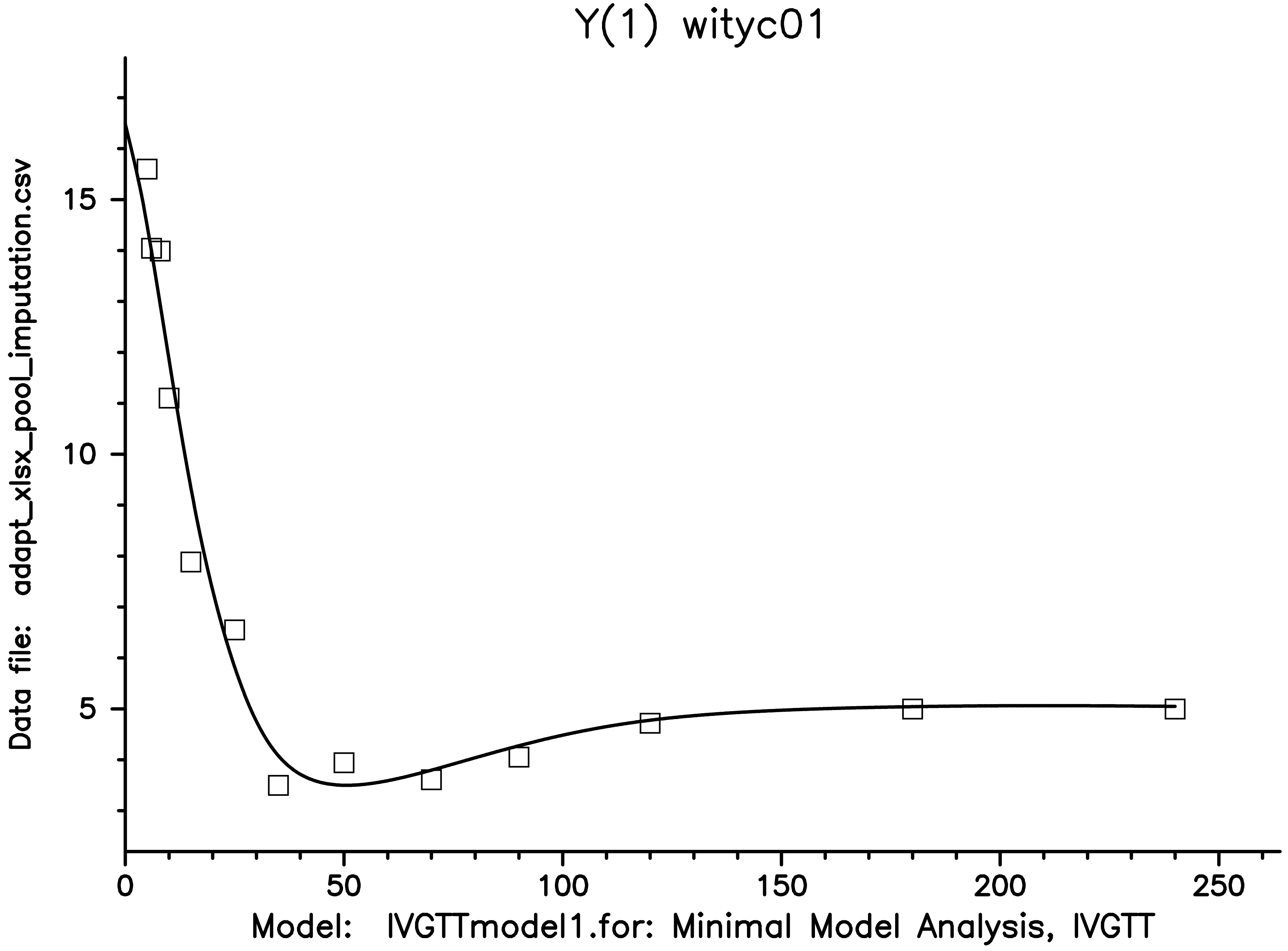
Y(1) wityc01
Data file: adapt_xlsx_pool_imputation.csv
15
10
5
0
50
100
150
200
250
Model: IVGTTmodel1.for: Minimal Model Analysis, IVGTT

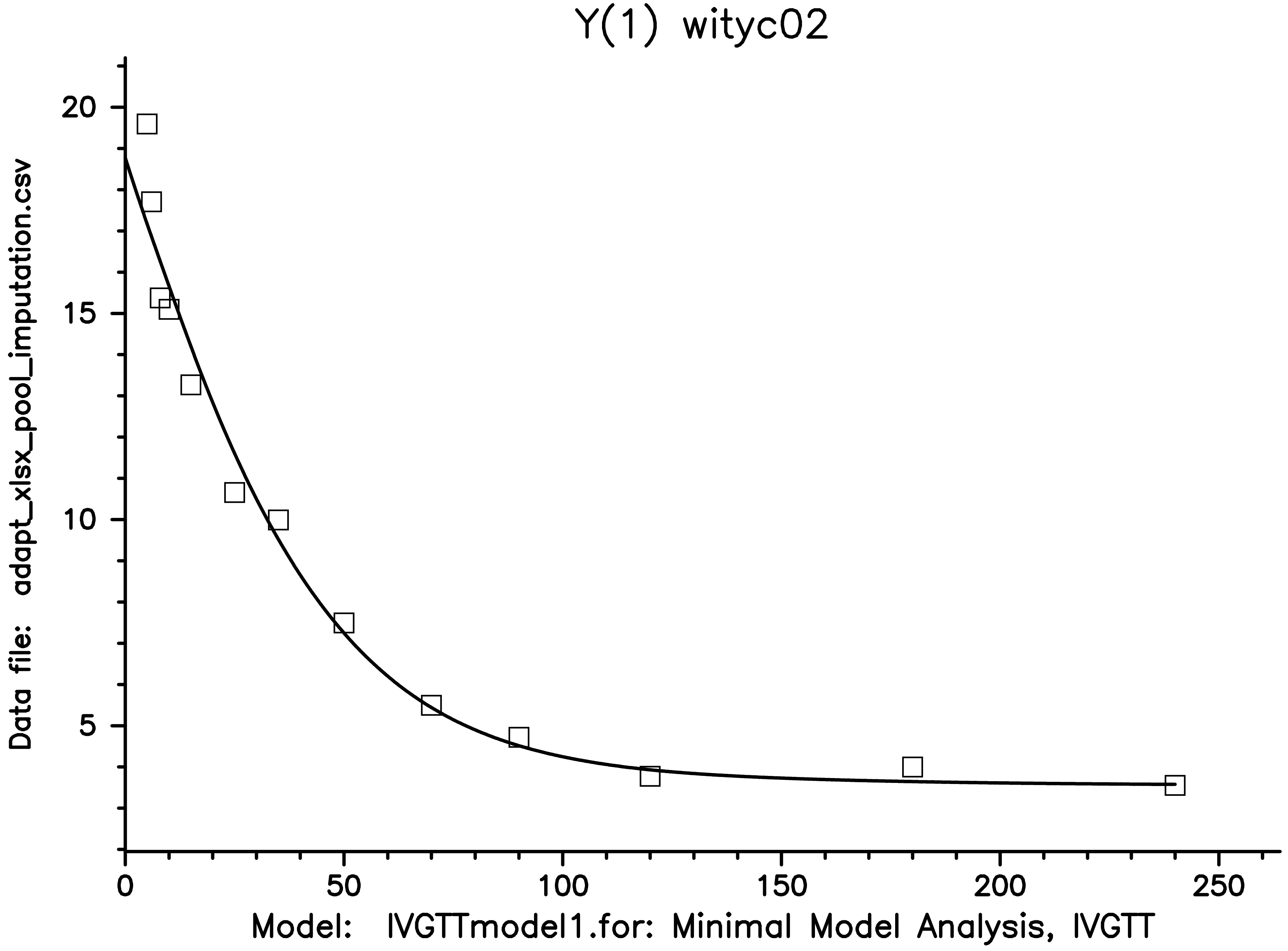
Y(1) wityc02
Data file: adapt_xlsx_pool_imputation.csv
Model: IVGTTmodel1.for: Minimal Model Analysis, IVGTT
20
15
10
5
0
50
100
150
200
250

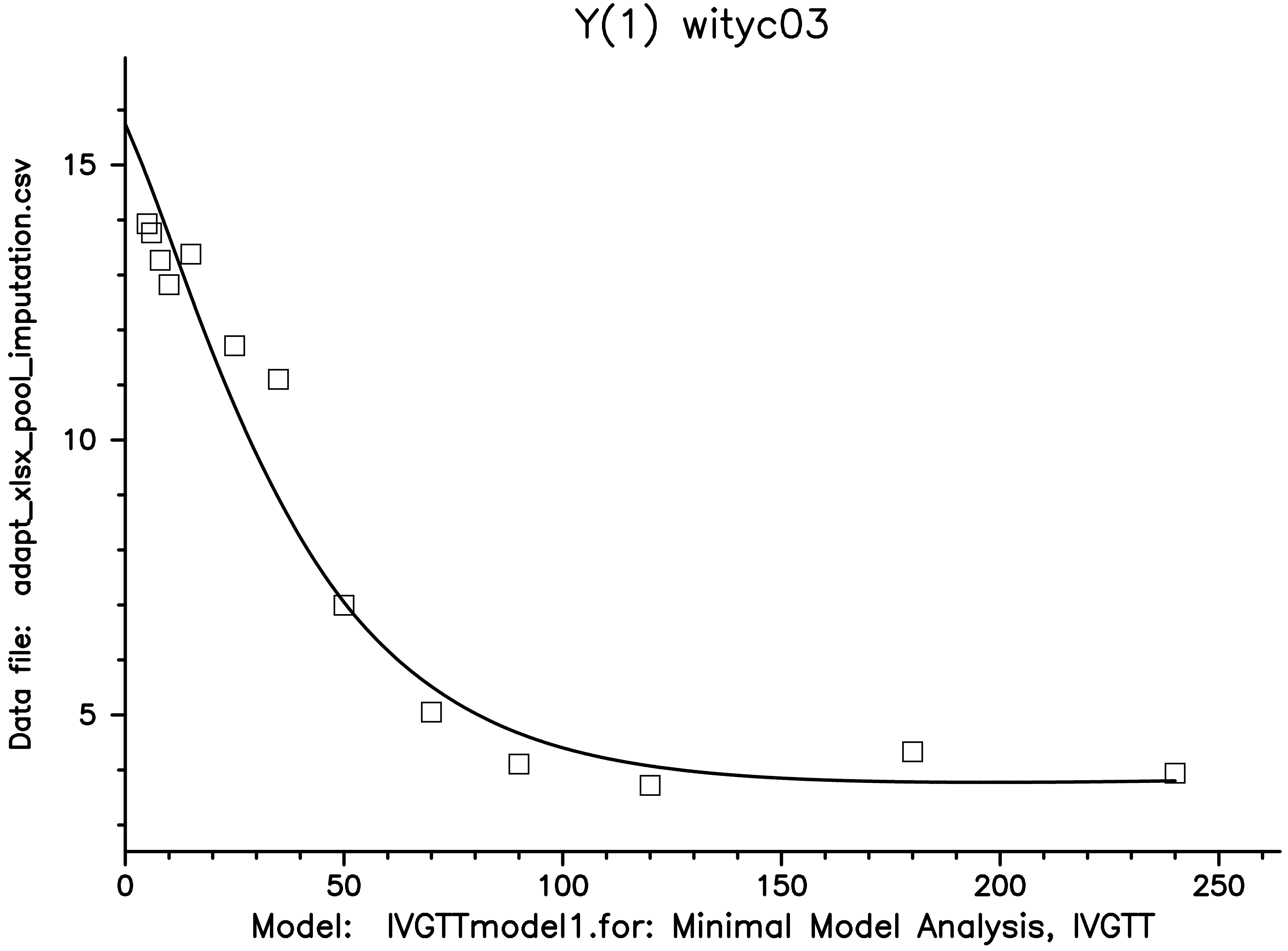
Y(1) wityc03
Data file: adapt_xlsx_pool_imputation.csv
15
10
5
0
50
100
150
200
250
Model: IVGTTmodel1.for: Minimal Model Analysis, IVGTT

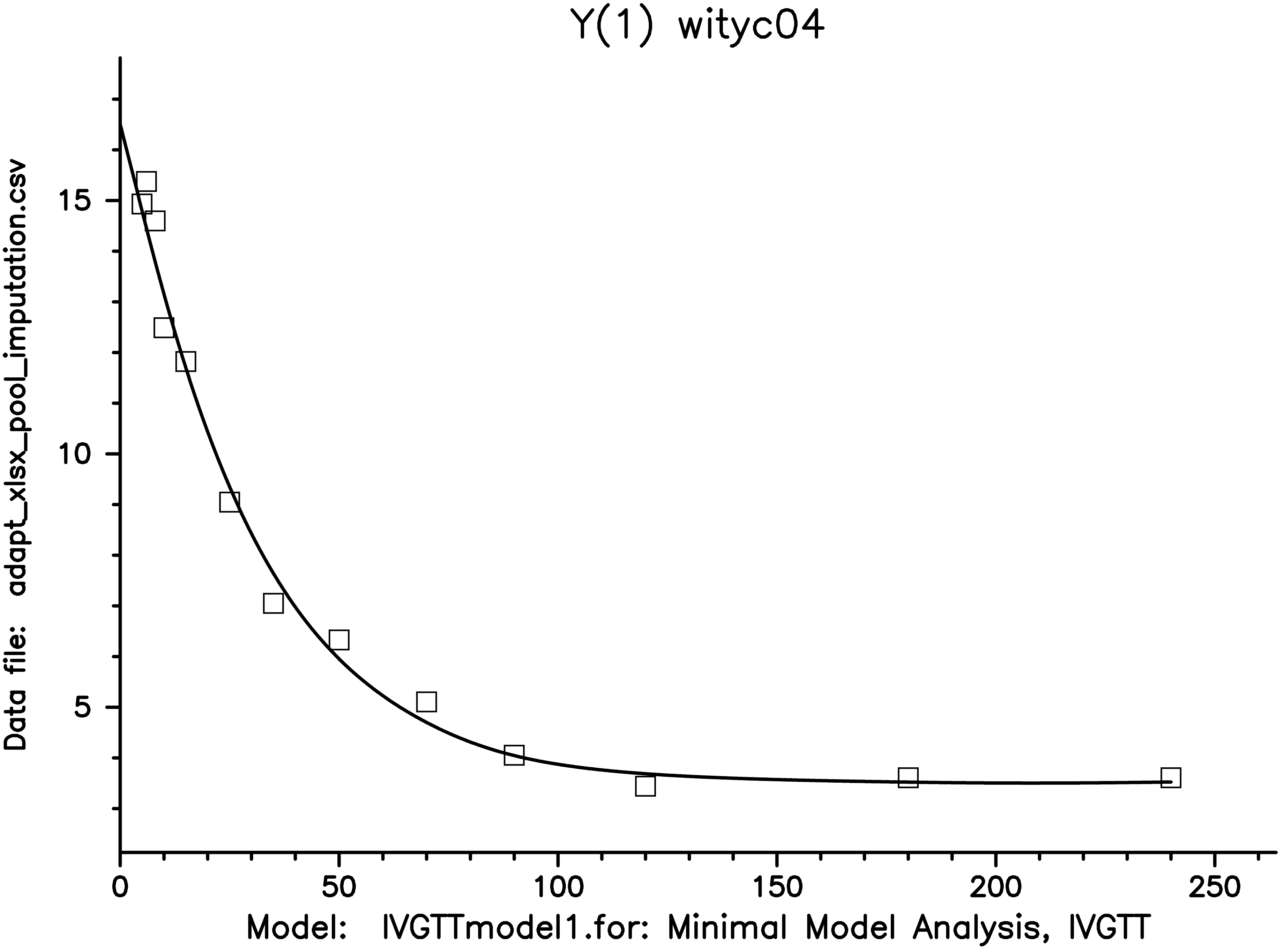

Y(1) wityc04
Data file: adapt_xlsx_pool_imputation.csv
15
10
5
0
50
100
150
200
250
Model: IVGTTmodel1.for: Minimal Model Analysis, IVGTT

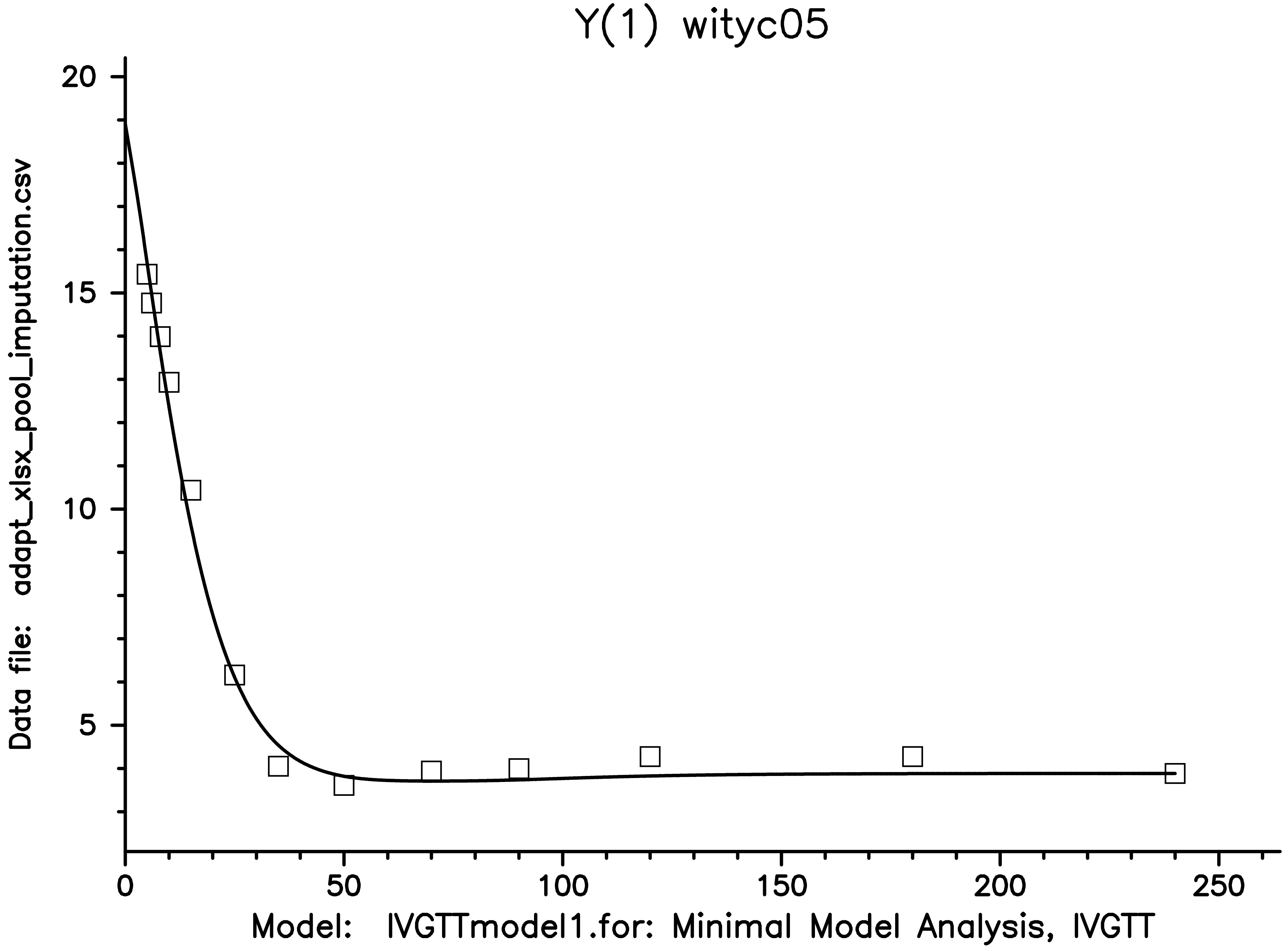
Y(1) wityc05
20
15
10
5
0
50
100
150
200
250
Data file: adapt_xlsx_pool_imputation.csv
Model: IVGTTmodel1.for: Minimal Model Analysis, IVGTT

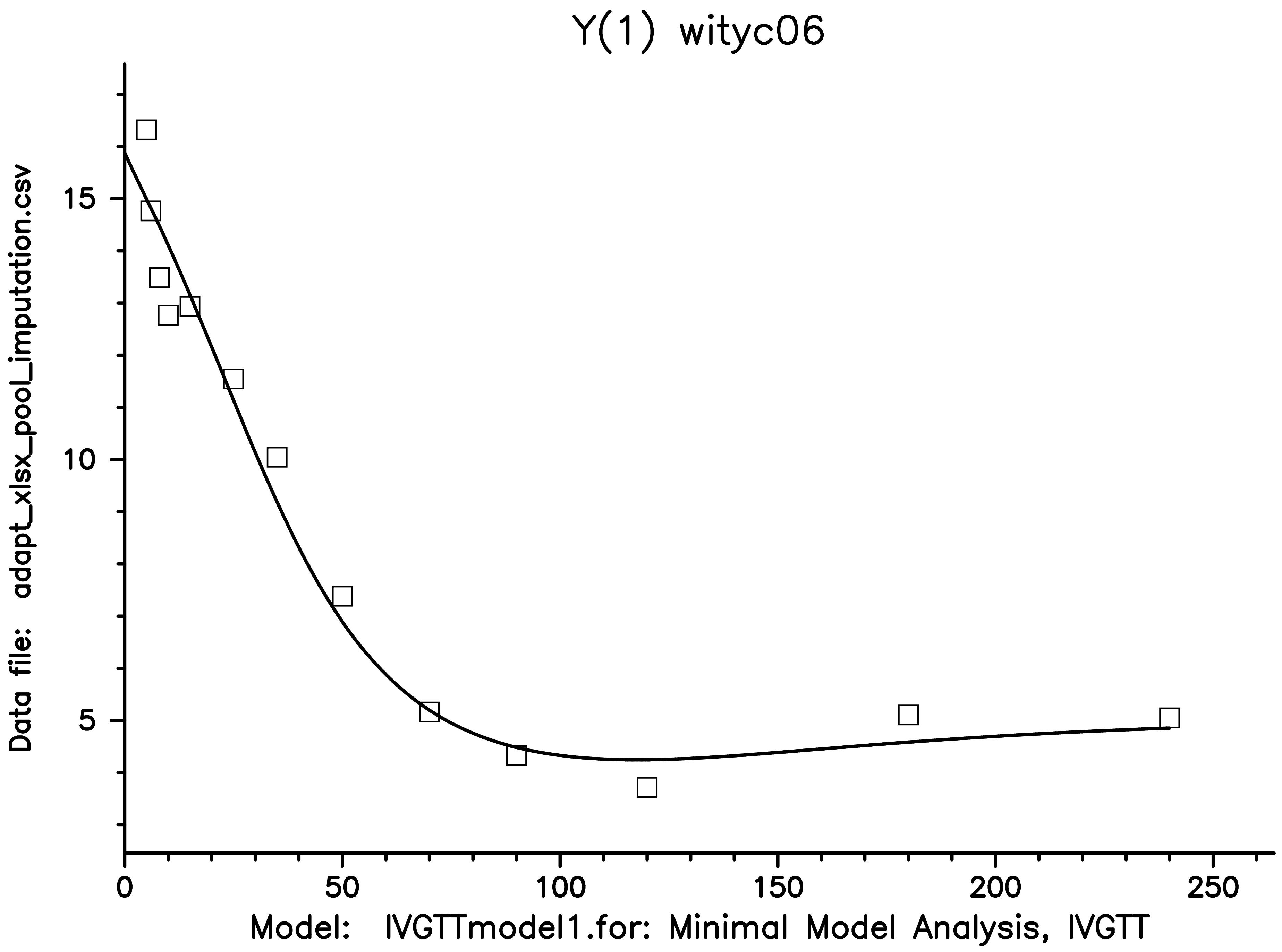

Y(1) wityc06
Data file: adapt_xlsx_pool_imputation.csv
Model: IVGTTmodel1.for: Minimal Model Analysis, IVGTT
15
10
5
0
50
100
150
200
250

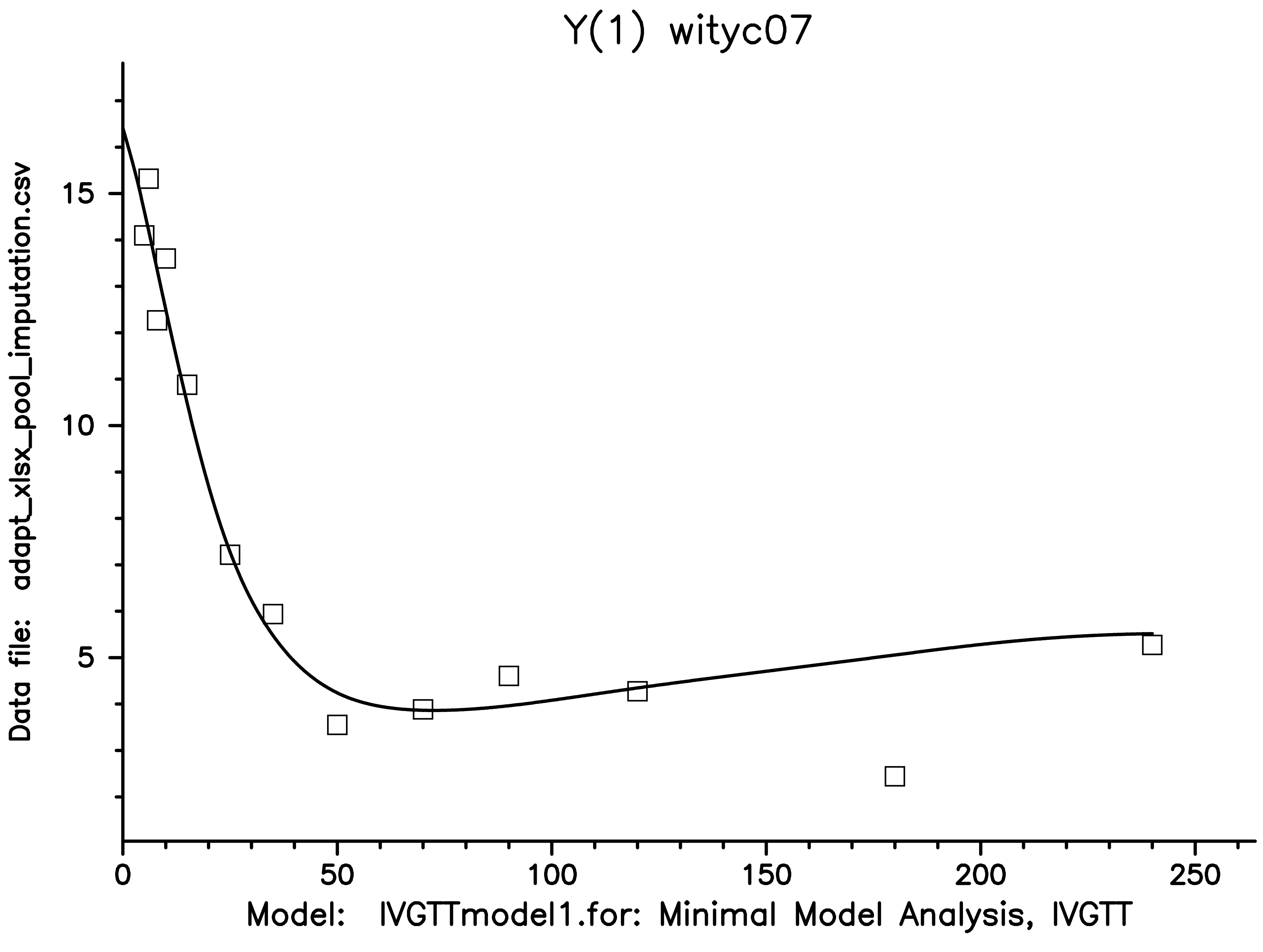

Y(1) wityc07
Data file: adapt_xlsx_pool_imputation.csv
Model: IVGTTmodel1.for: Minimal Model Analysis, IVGTT
15
10
5
0
50
100
150
200
250

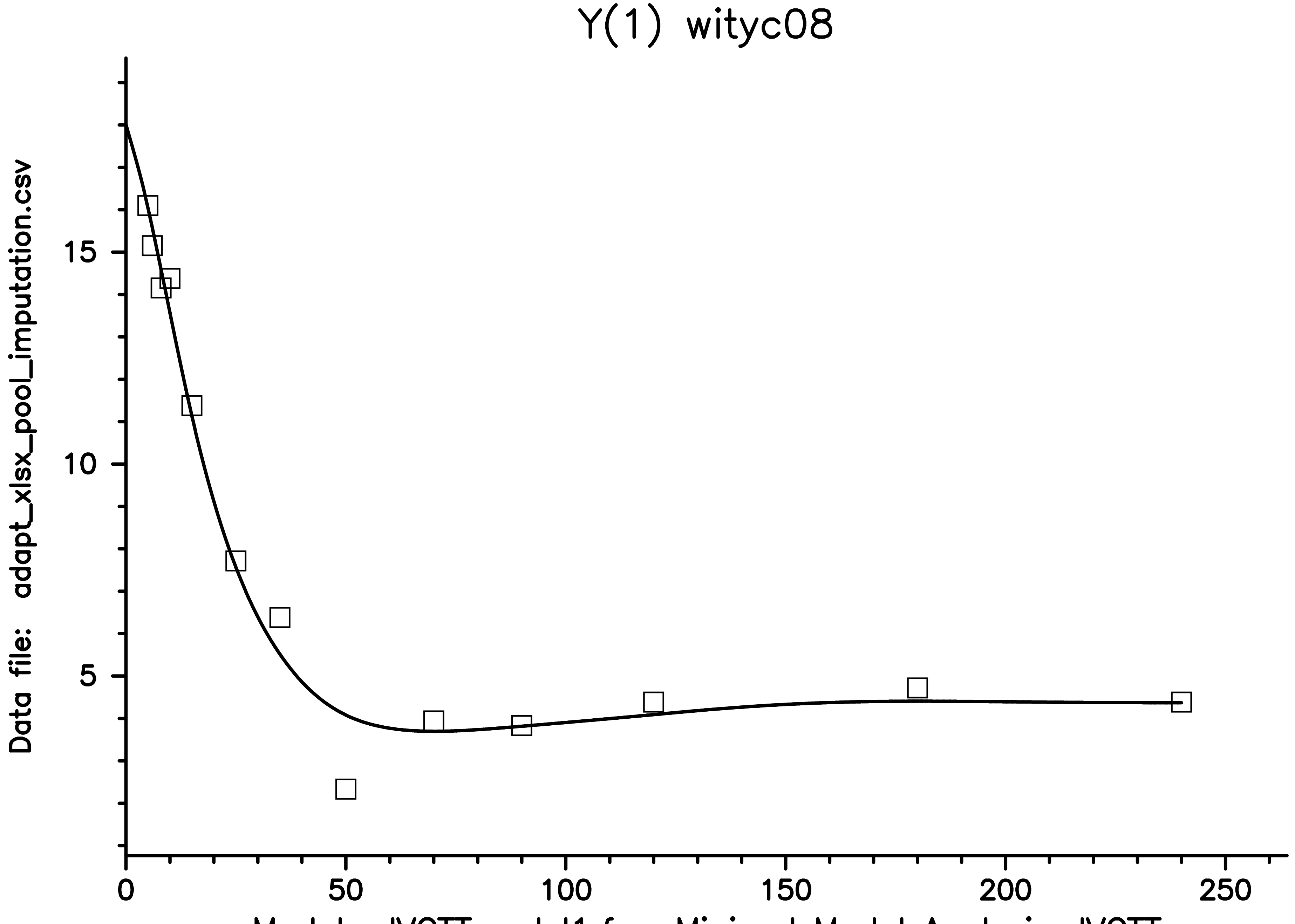

Y(1) wityc08
Data file: adapt_xlsx_pool_imputation.csv
15
10
5
0
50
100
150
200
250
Model: IVGTTmodel1.for: Minimal Model Analysis, IVGTT

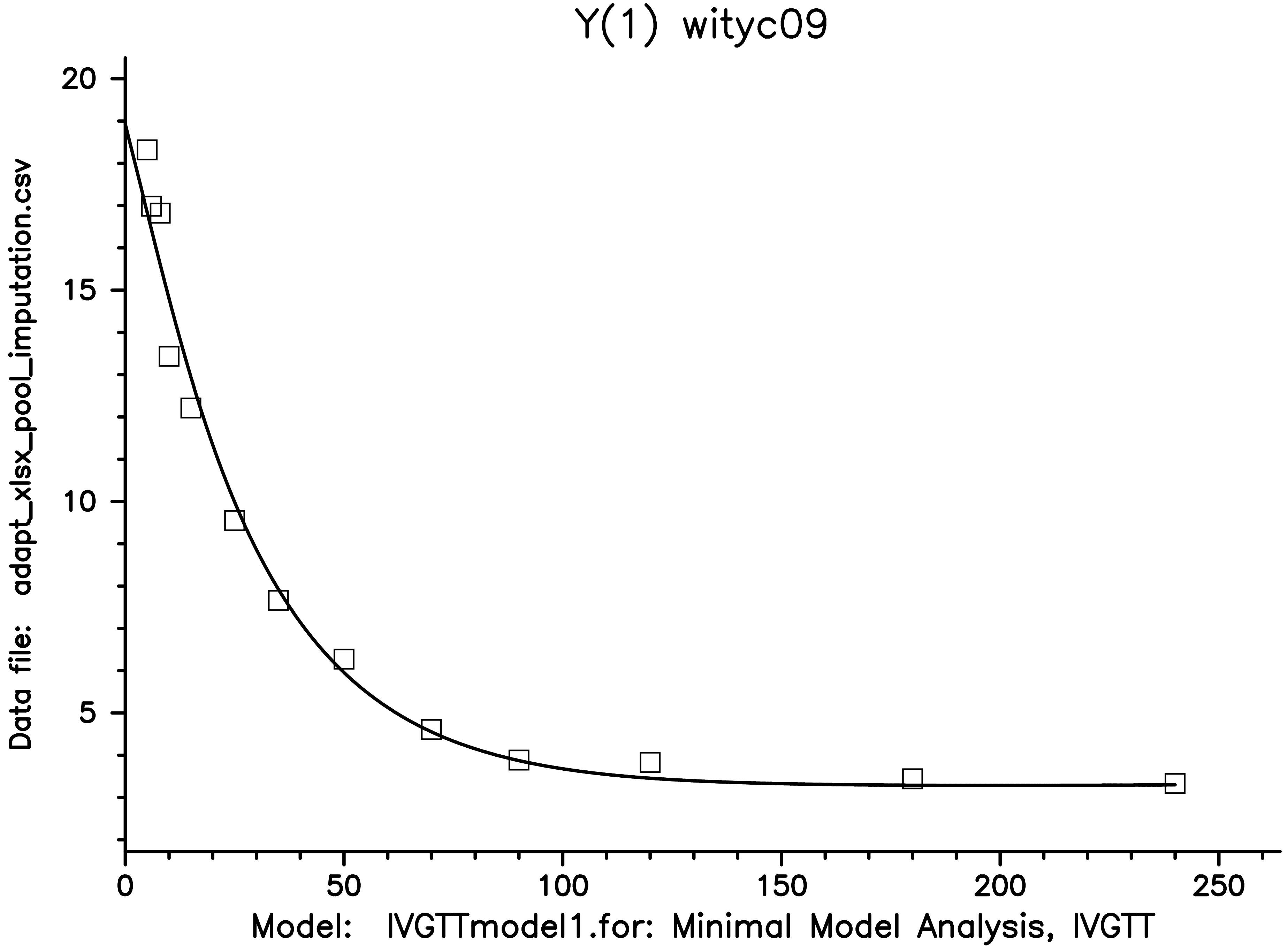
Y(1) wityc09
Data file: adapt_xlsx_pool_imputation.csv
Model: IVGTTmodel1.for: Minimal Model Analysis, IVGTT
20
15
10
5
0
50
100
150
200
250

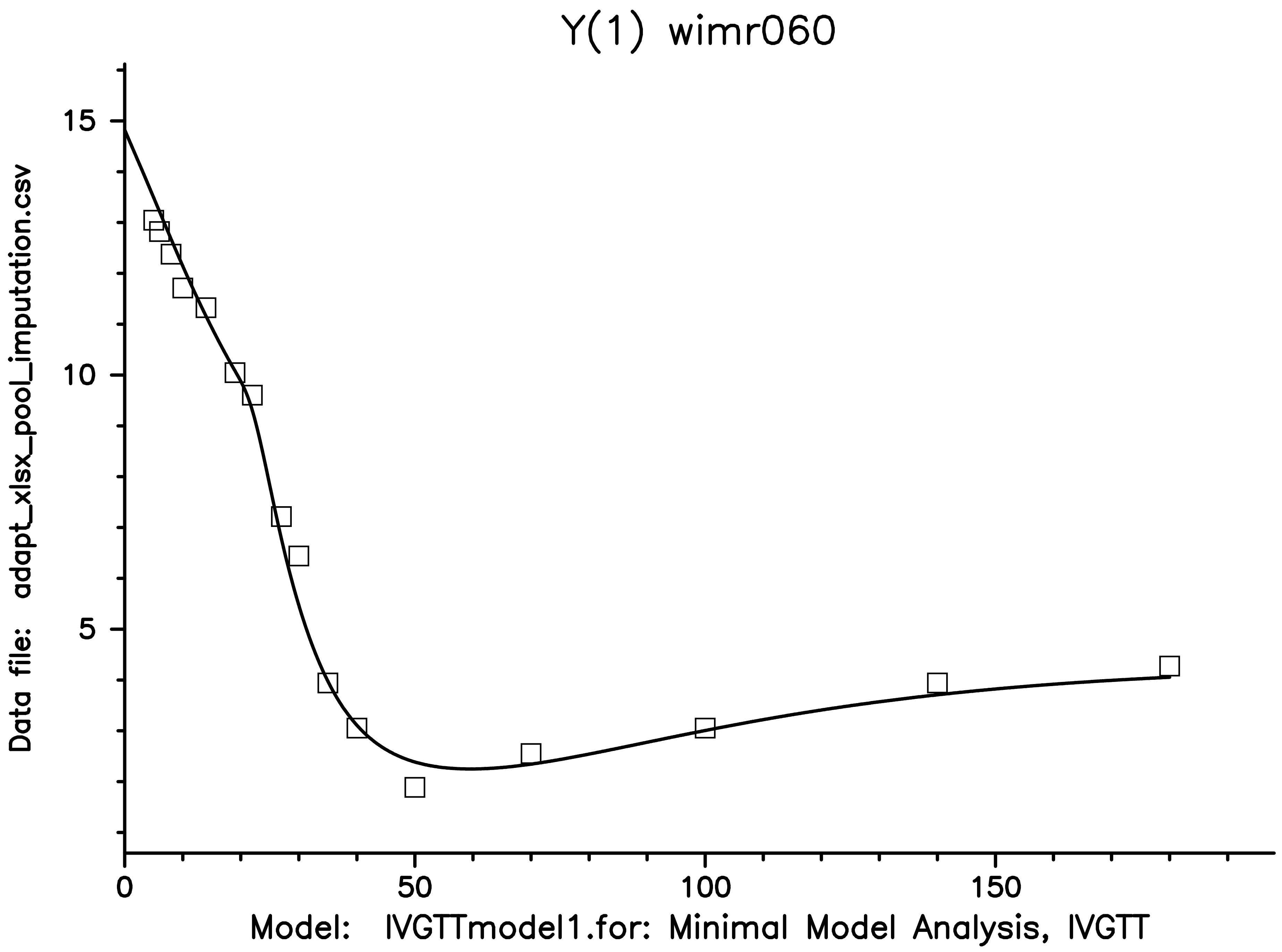

Y(1) wimr060
Data file: adapt_xlsx_pool_imputation.csv
15
10
5
0
50
100
150
Model: IVGTTmodel1.for: Minimal Model Analysis, IVGTT

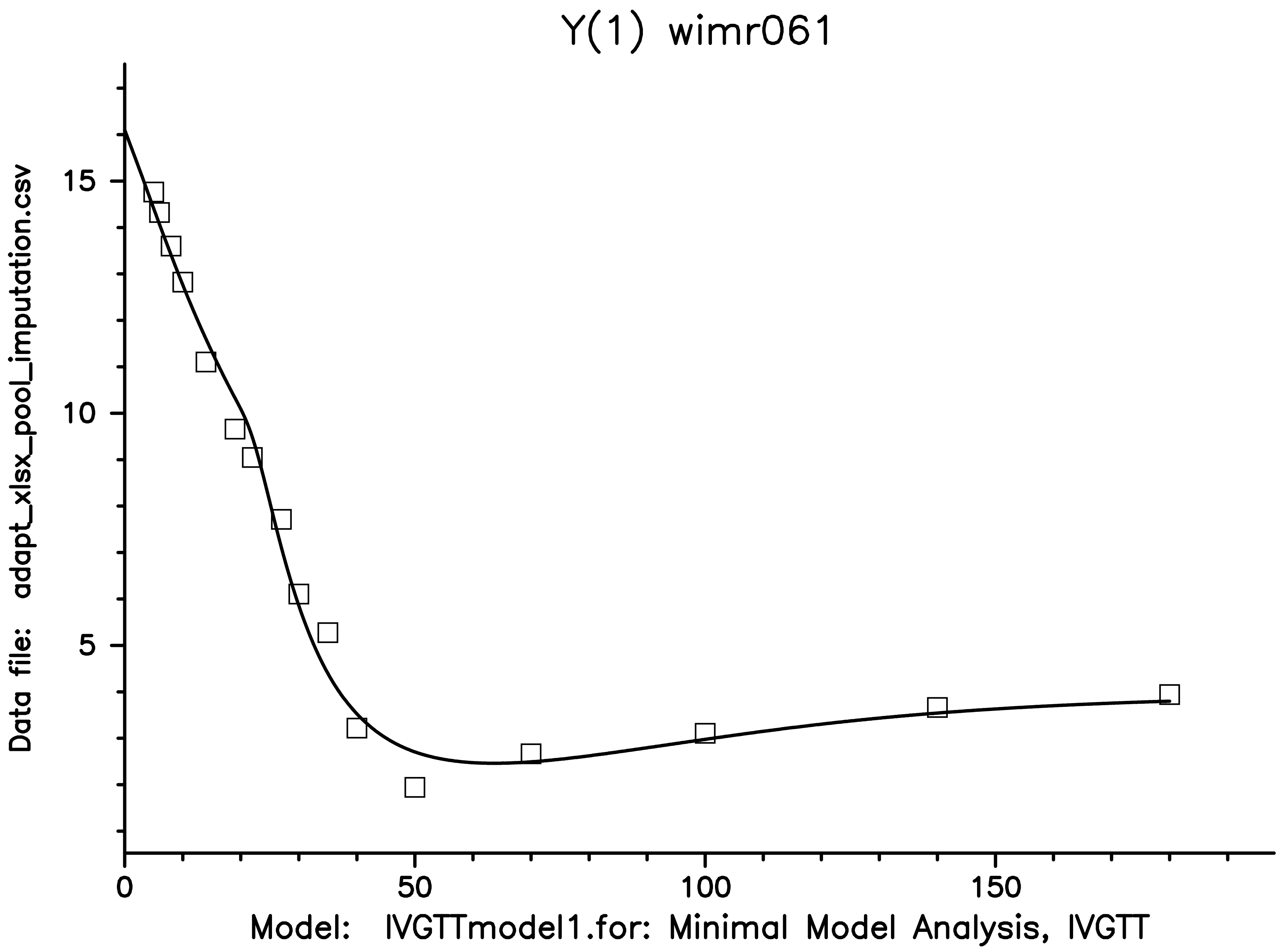

Y(1) wimr061
Data file: adapt_xlsx_pool_imputation.csv
15
10
5
0
50
100
150
Model: IVGTTmodel1.for: Minimal Model Analysis, IVGTT

Y(1) wimr062

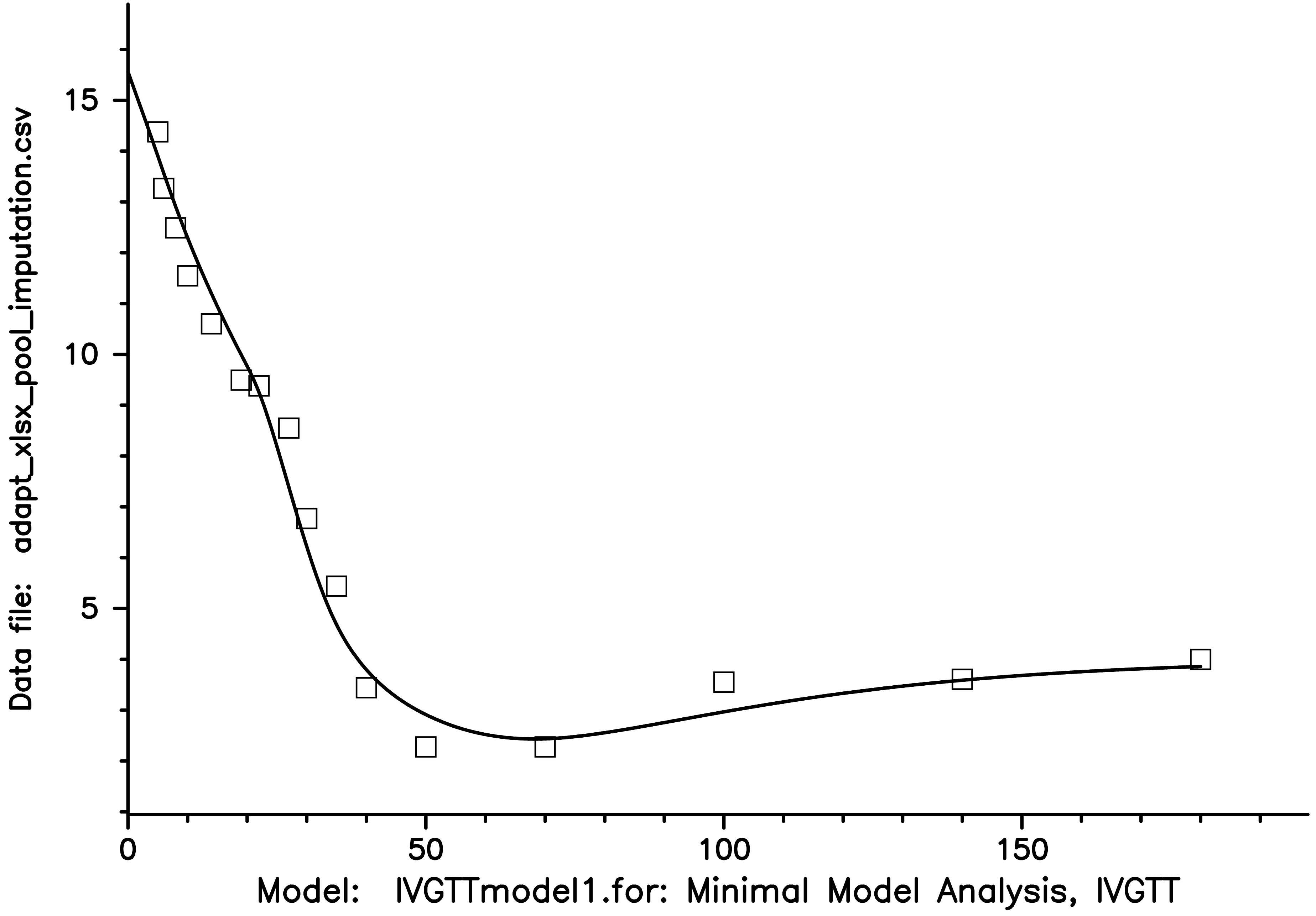

15
10
5
0
50
100
150
Data file: adapt_xlsx_pool_imputation.csv
Model: IVGTTmodel1.for: Minimal Model Analysis, IVGTT

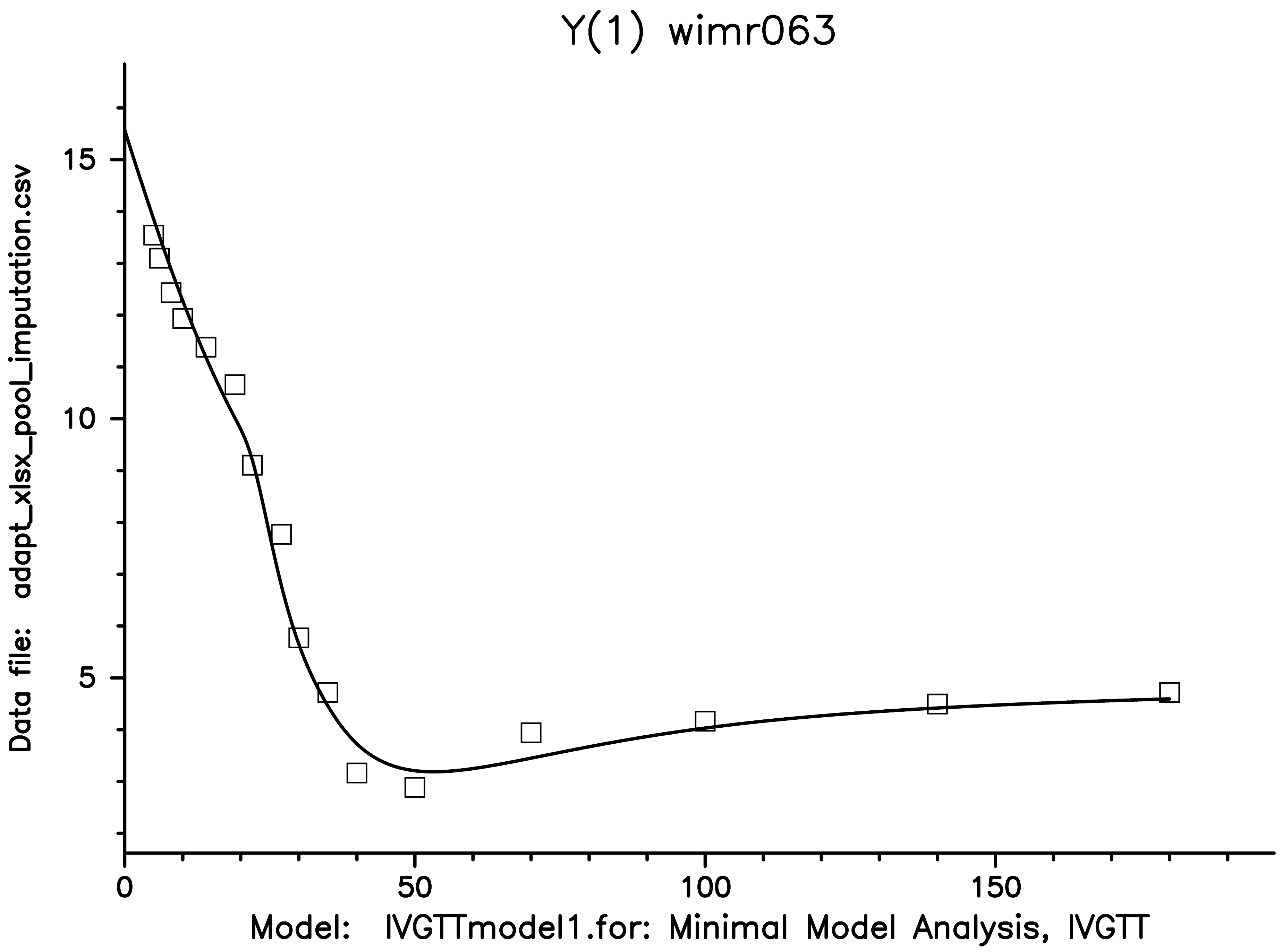
Y(1) wimr063
Data file: adapt_xlsx_pool_imputation.csv
15
10
5
0
50
100
150
Model: IVGTTmodel1.for: Minimal Model Analysis, IVGTT

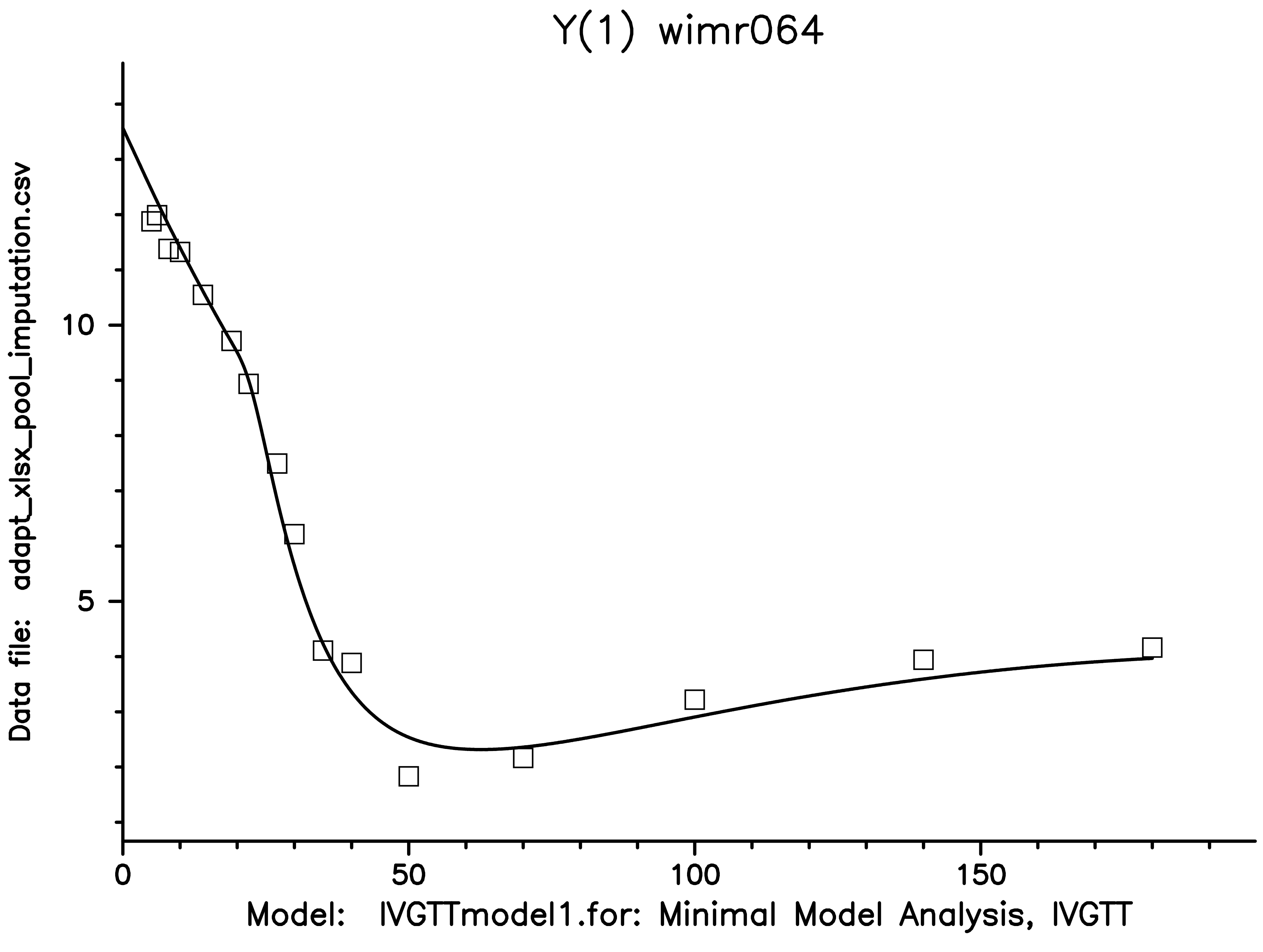
Y(1) wimr064
Data file: adapt_xlsx_pool_imputation.csv
10
5
0
50
100
150
Model: IVGTTmodel1.for: Minimal Model Analysis, IVGTT

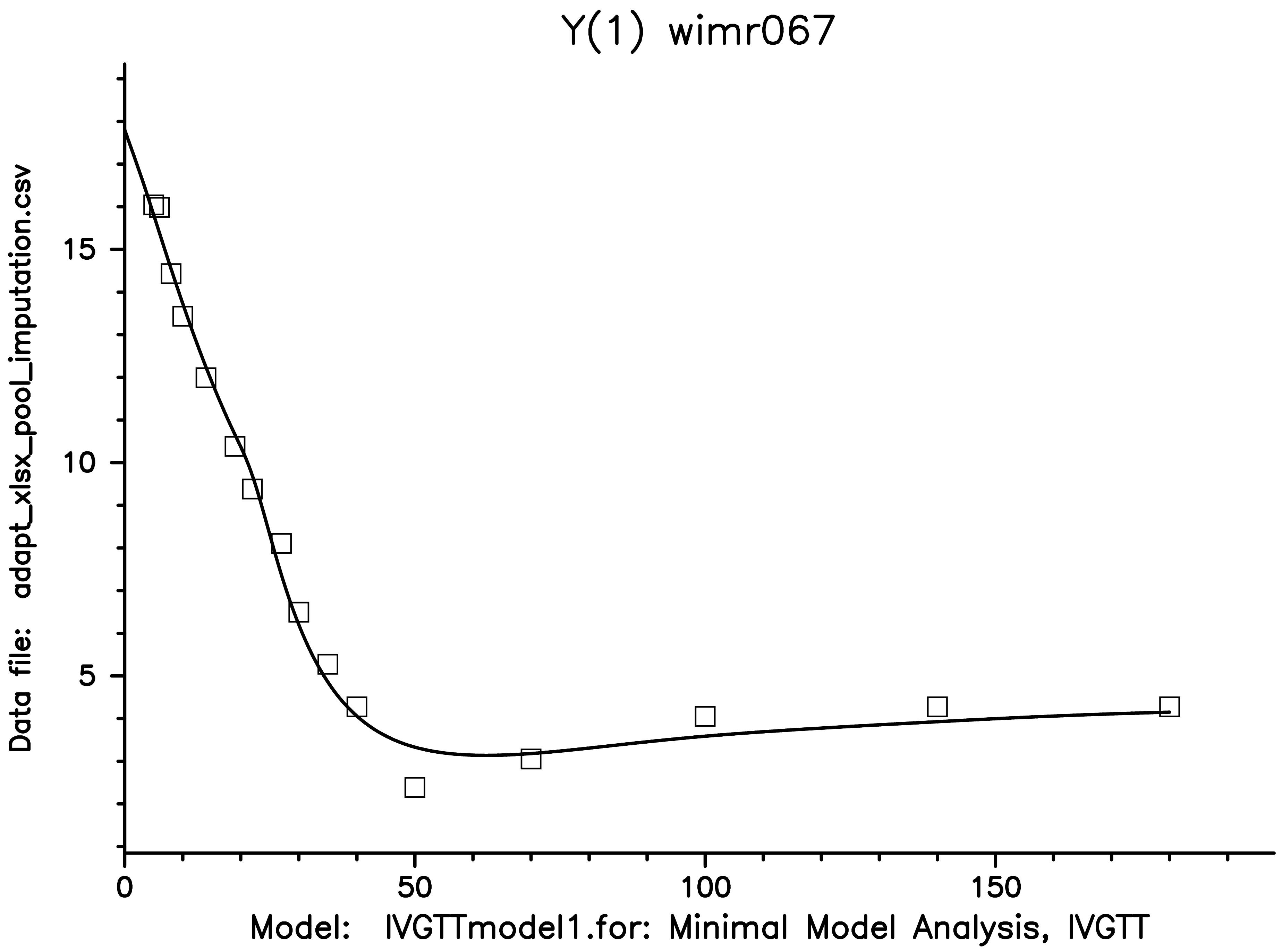
Y(1) wimr067
Data file: adapt_xlsx_pool_imputation.csv
15
10
5
0
50
100
150
Model: IVGTTmodel1.for: Minimal Model Analysis, IVGTT

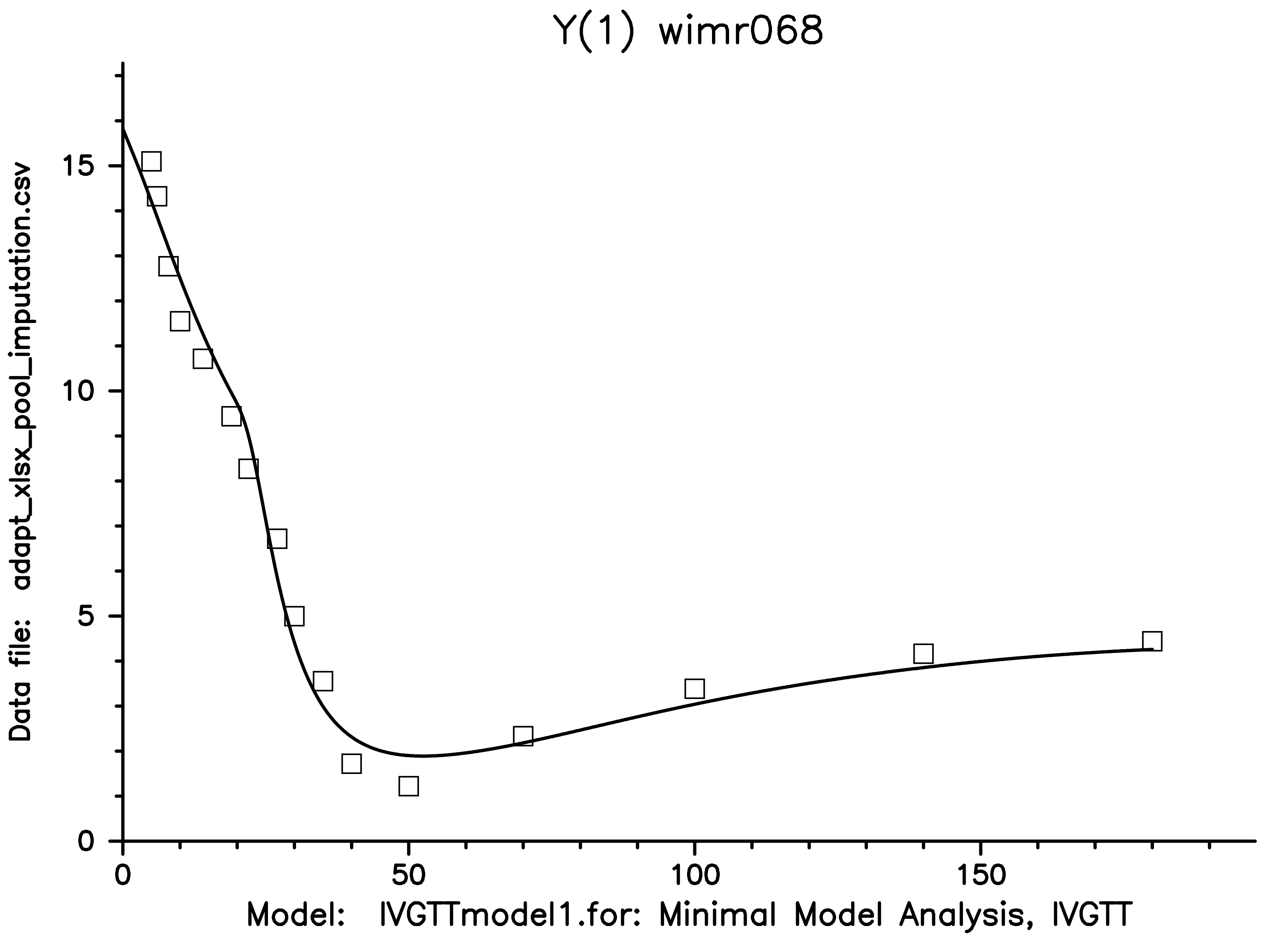
Y(1) wimr068
Data file: adapt_xlsx_pool_imputation.csv
0
5
10
15
0
50
100
150
Model: IVGTTmodel1.for: Minimal Model Analysis, IVGTT

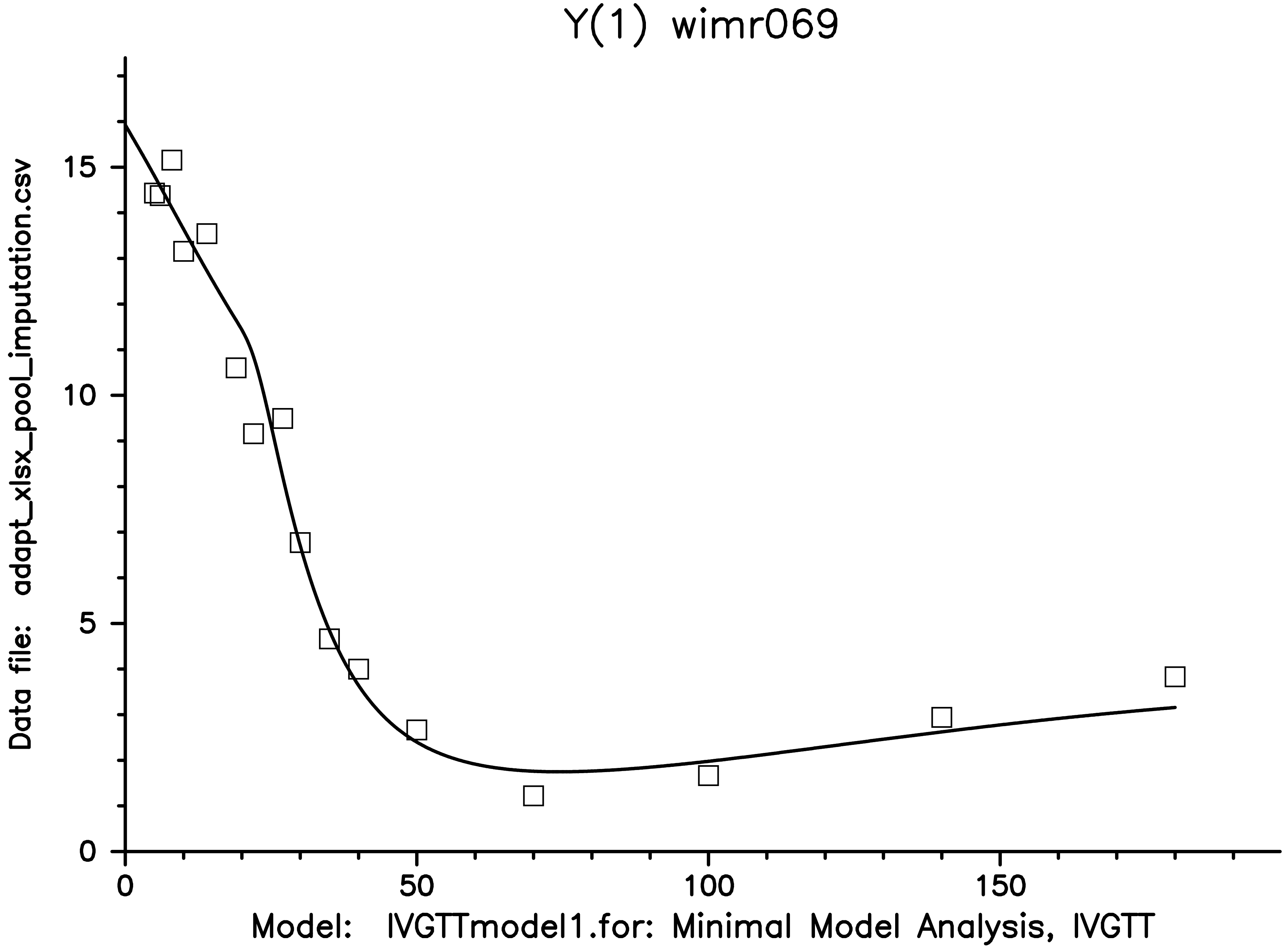

Y(1) wimr069
Data file: adapt_xlsx_pool_imputation.csv
15
10
5
0
0
50
100
150
Model: IVGTTmodel1.for: Minimal Model Analysis, IVGTT

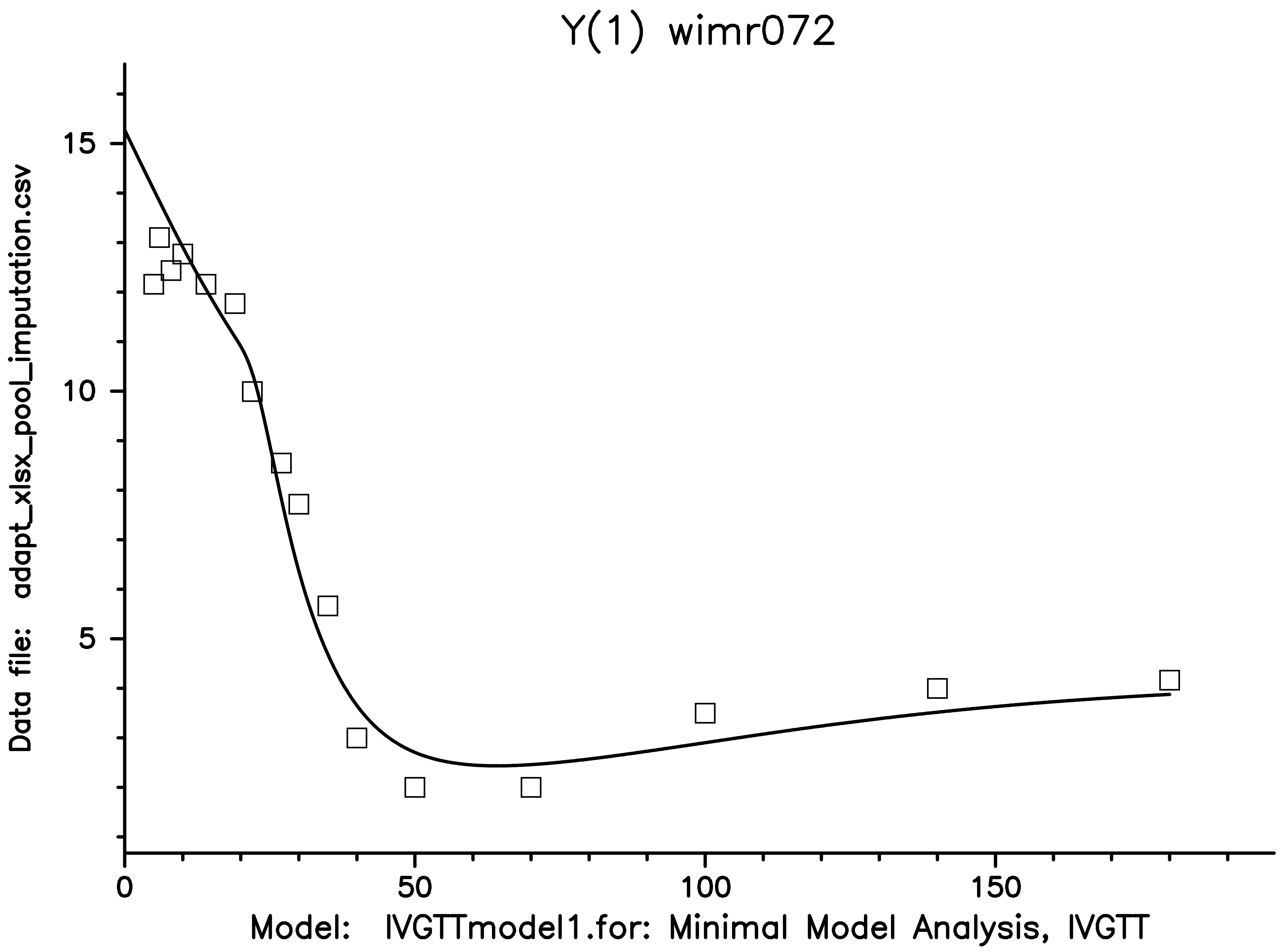

Y(1) wimr072
Data file: adapt_xlsx_pool_imputation.csv
15
10
5
0
50
100
150
Model: IVGTTmodel1.for: Minimal Model Analysis, IVGTT

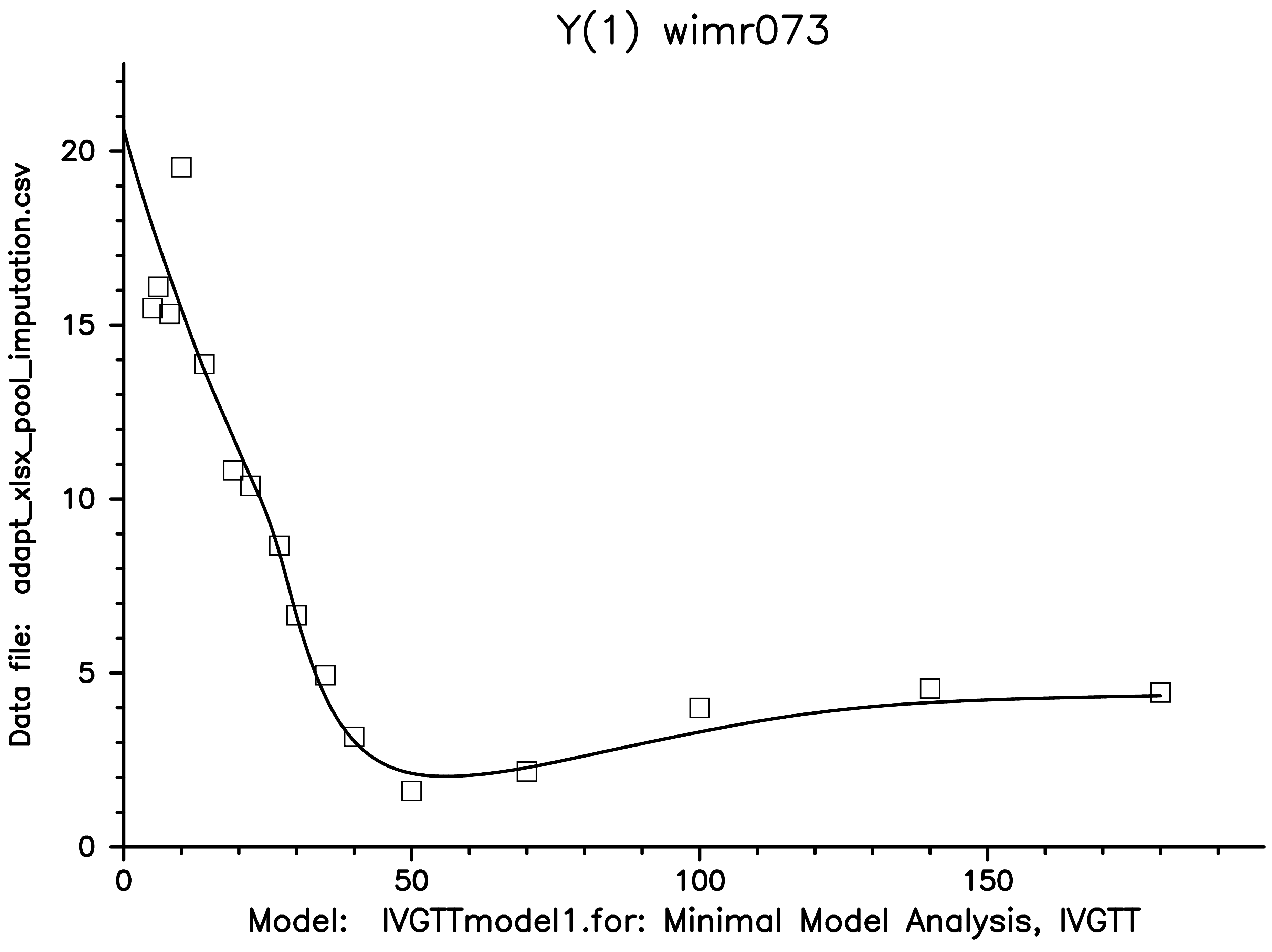
Y(1) wimr073
Data file: adapt_xlsx_pool_imputation.csv
0
5
10
15
20
0
50
100
150
Model: IVGTTmodel1.for: Minimal Model Analysis, IVGTT

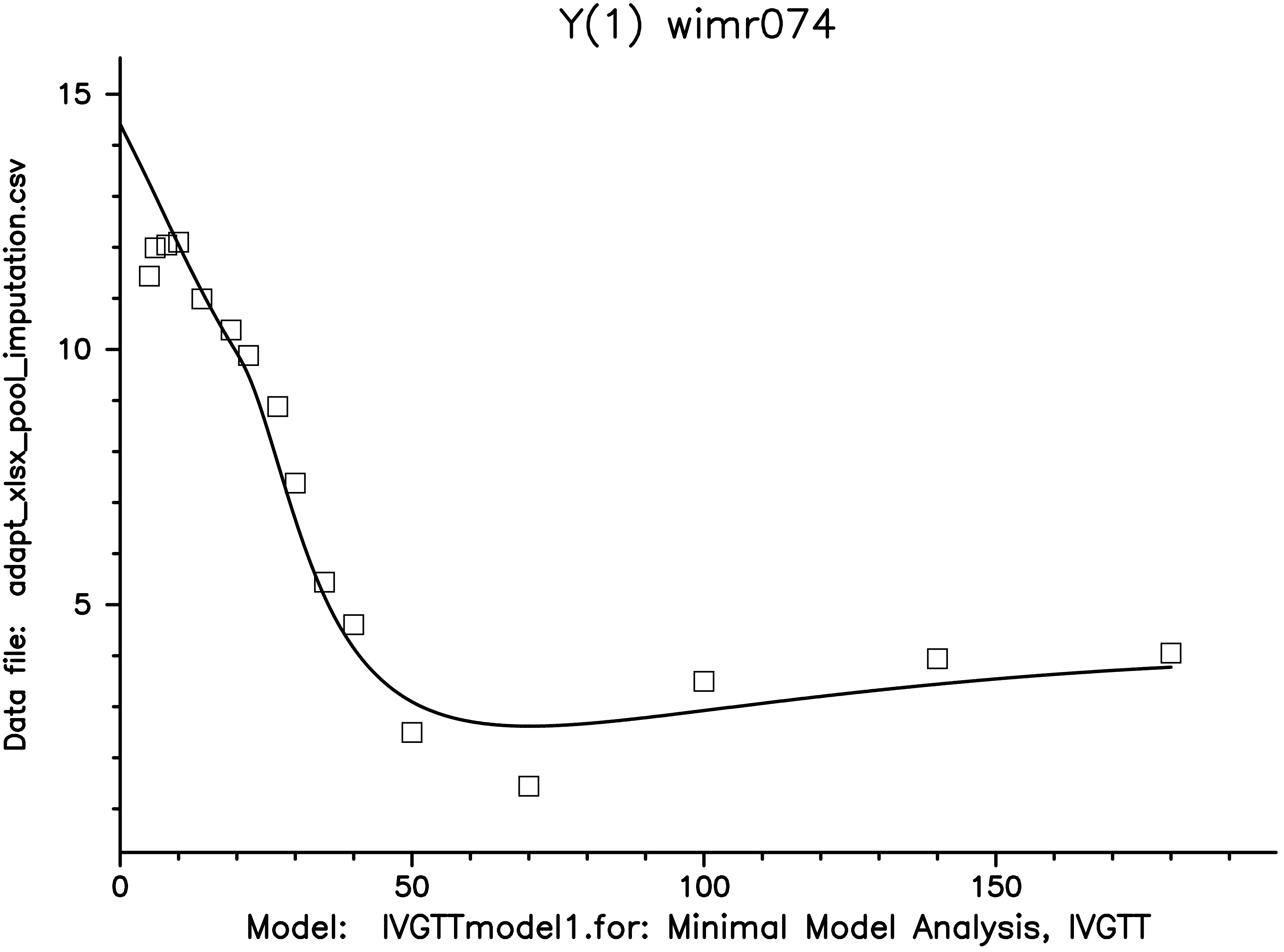
Y(1) wimr074
15
10
5
0
50
100
150
Data file: adapt_xlsx_pool_imputation.csv
Model: IVGTTmodel1.for: Minimal Model Analysis, IVGTT

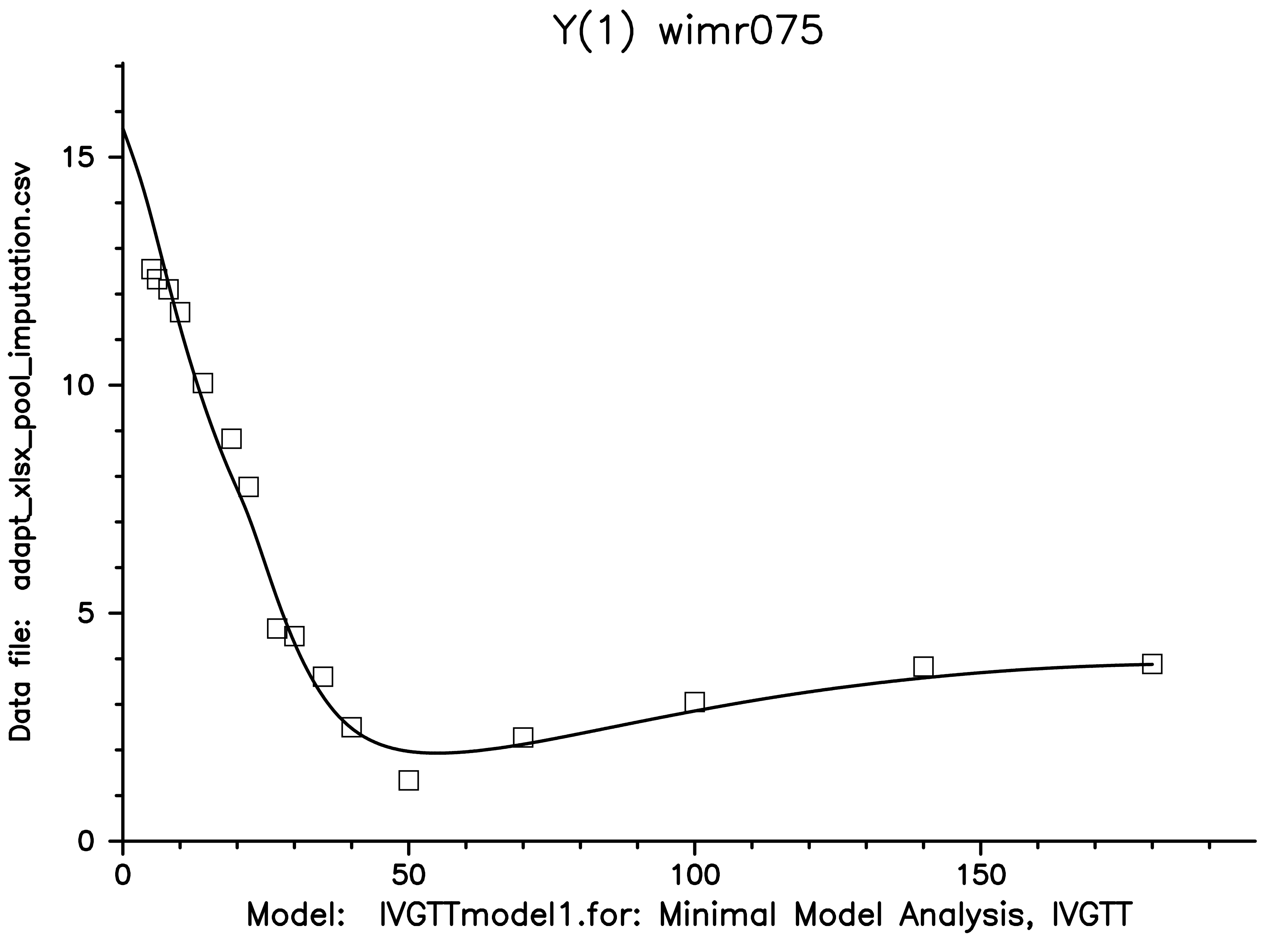

Y(1) wimr075
Data file: adapt_xlsx_pool_imputation.csv
Model: IVGTTmodel1.for: Minimal Model Analysis, IVGTT
0
5
10
15
0
50
100
150

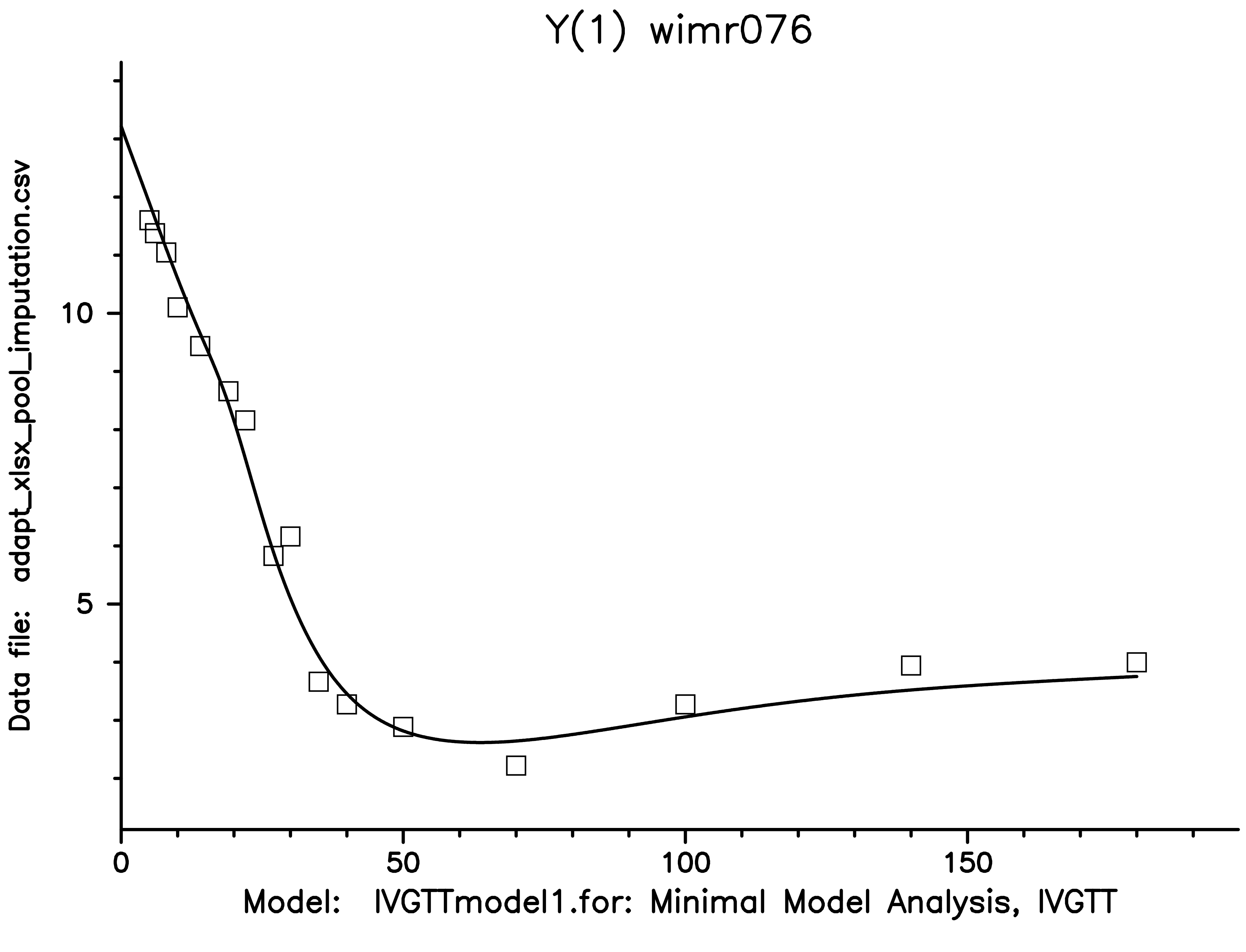

Y(1) wimr076
Data file: adapt_xlsx_pool_imputation.csv
10
5
0
50
100
150
Model: IVGTTmodel1.for: Minimal Model Analysis, IVGTT

Y(1) wimr077

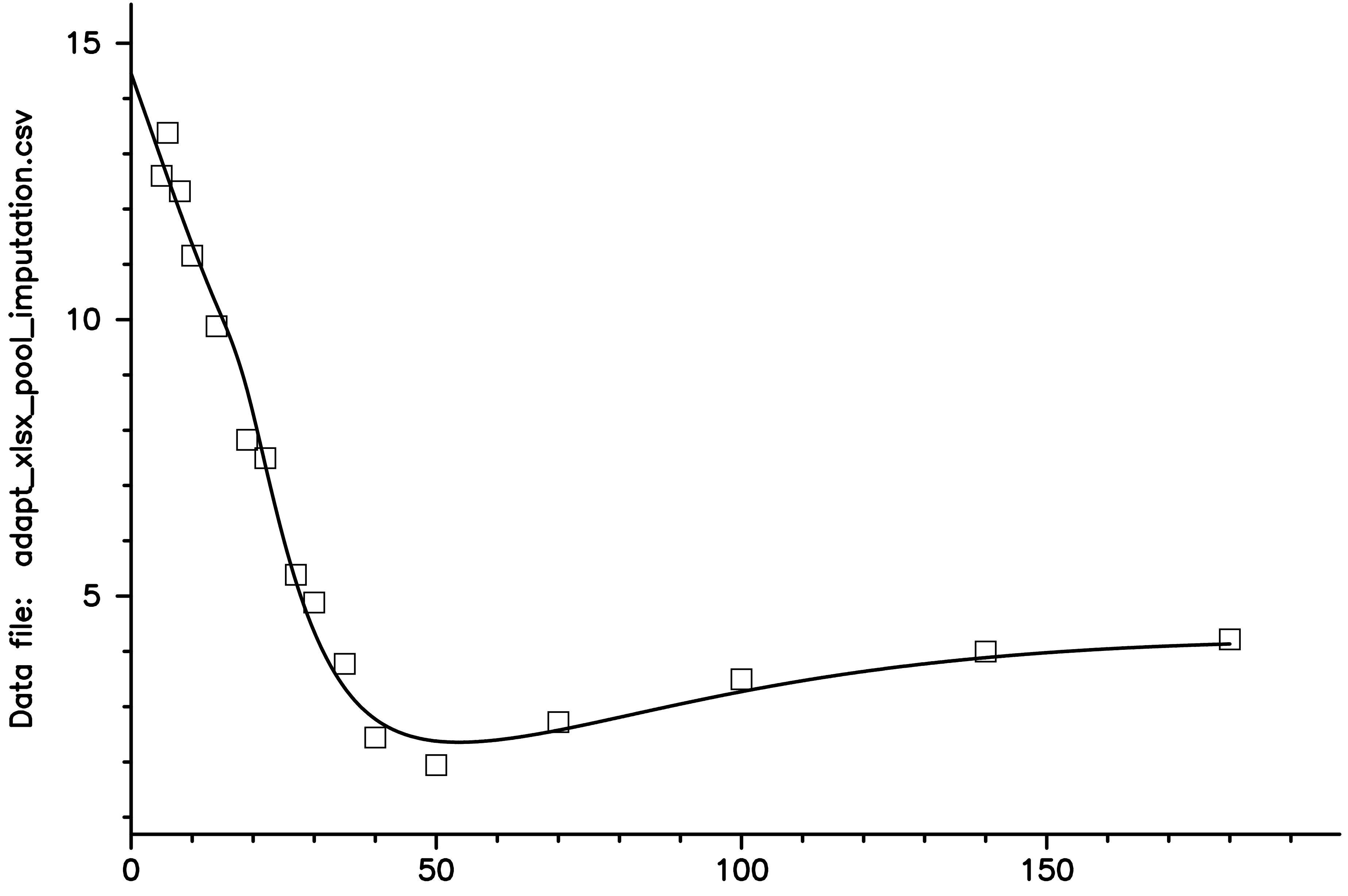

Model: IVGTTmodel1.for: Minimal Model Analysis, IVGTT

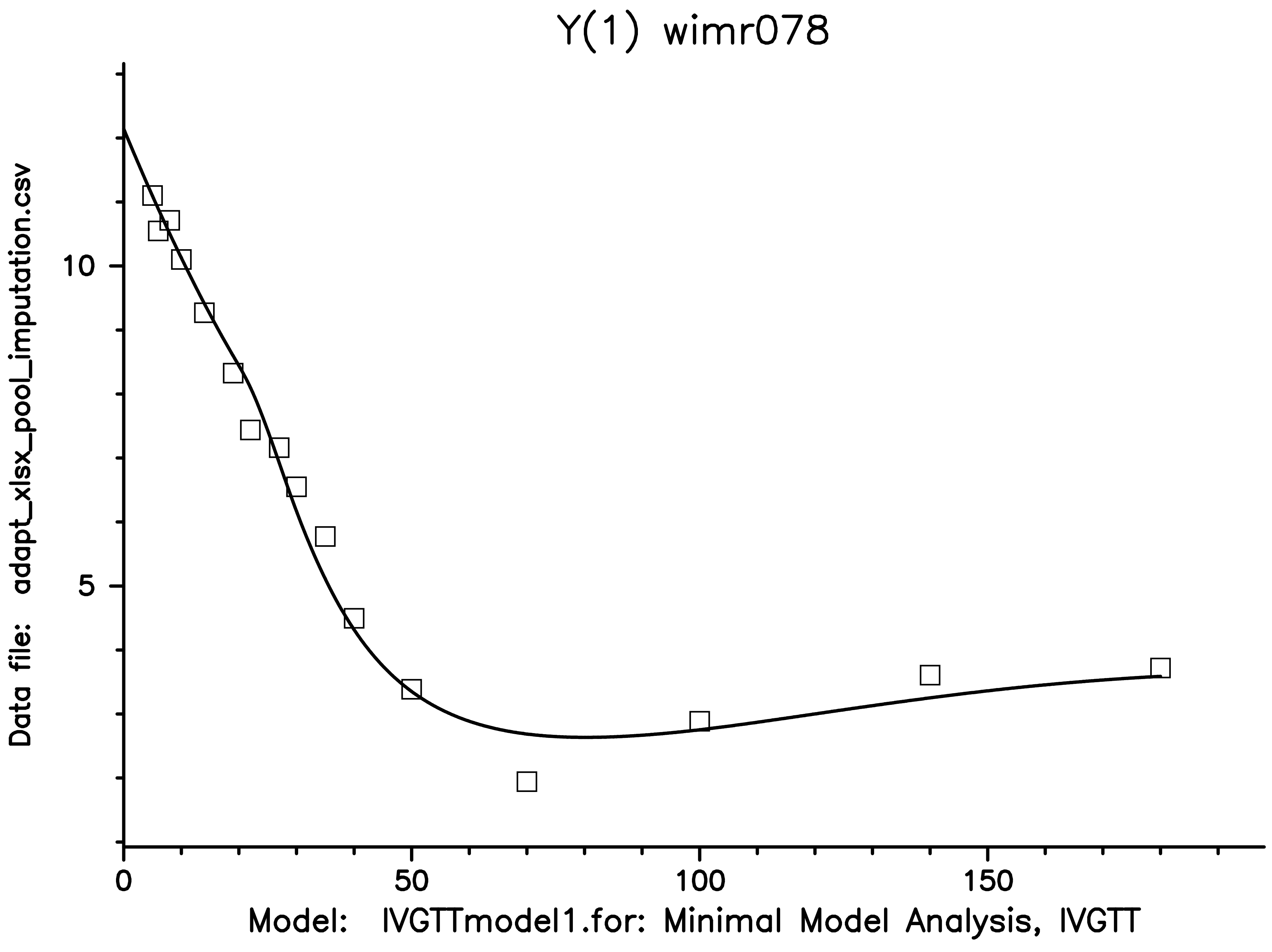
Y(1) wimr078
Data file: adapt_xlsx_pool_imputation.csv
10
5
0
50
100
150
Model: IVGTTmodel1.for: Minimal Model Analysis, IVGTT

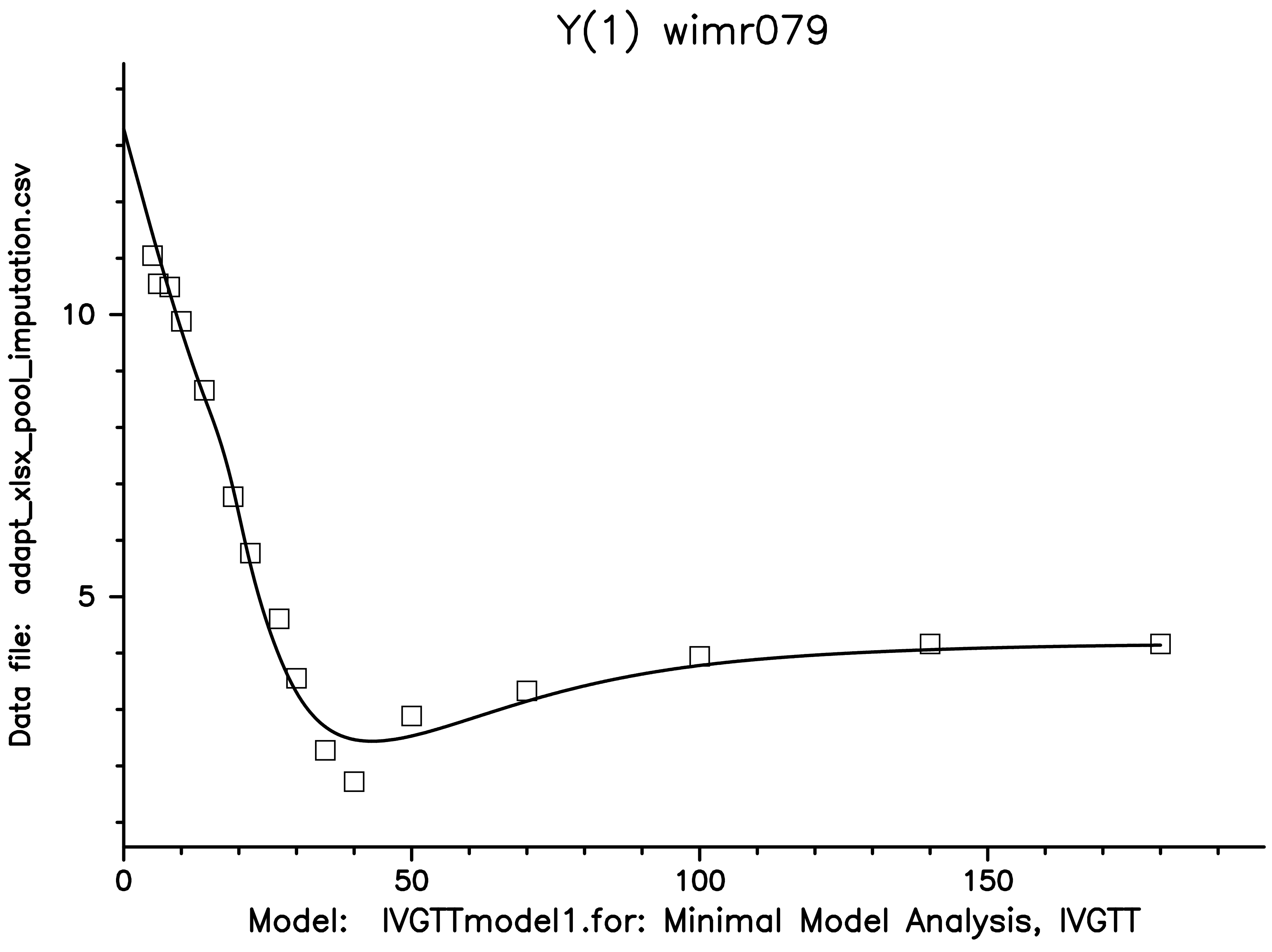

Y(1) wimr079
Data file: adapt_xlsx_pool_imputation.csv
10
5
0
50
100
150
Model: IVGTTmodel1.for: Minimal Model Analysis, IVGTT

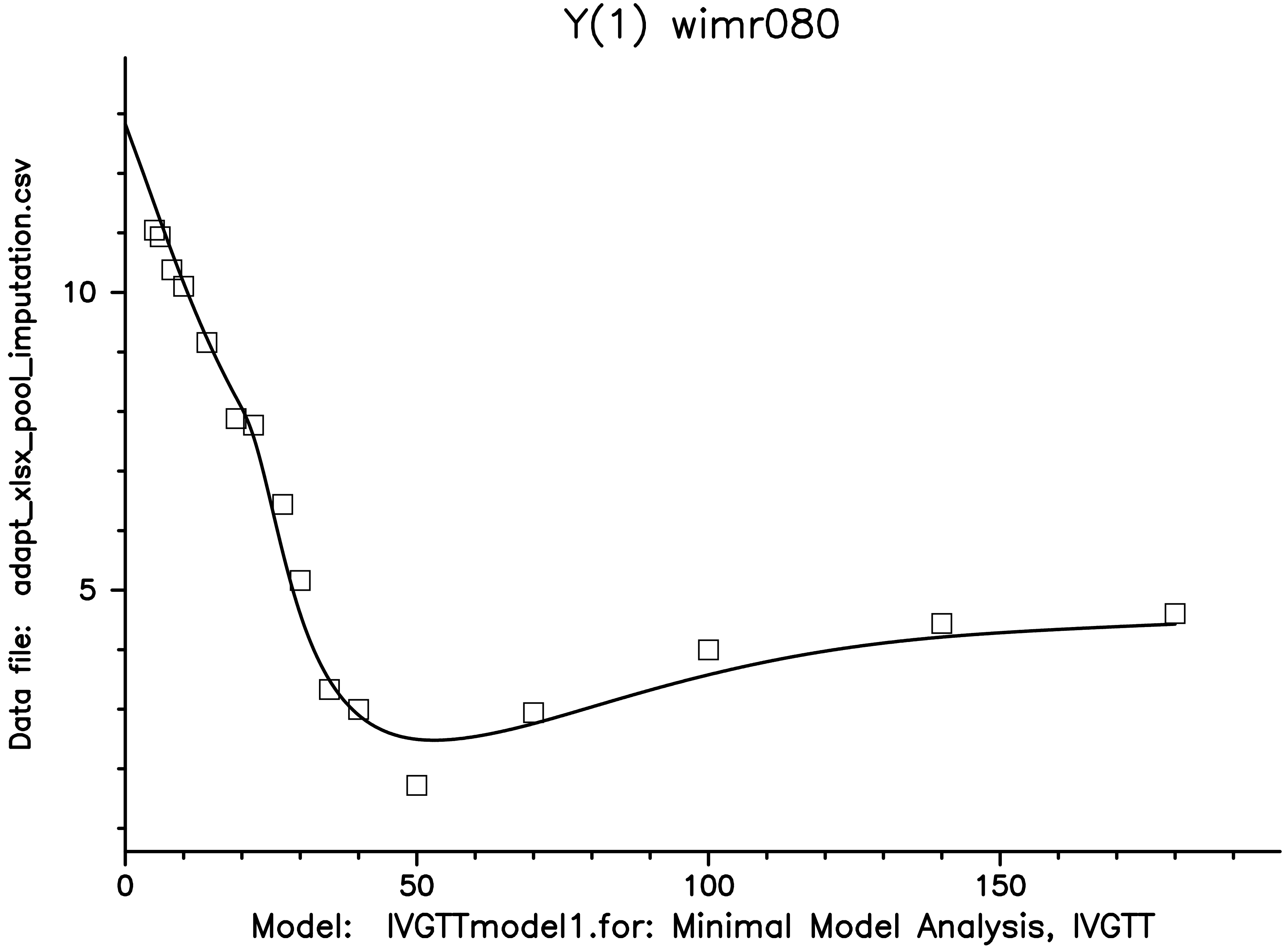

Y(1) wimr080
Data file: adapt_xlsx_pool_imputation.csv
10
5
0
50
100
150
Model: IVGTTmodel1.for: Minimal Model Analysis, IVGTT

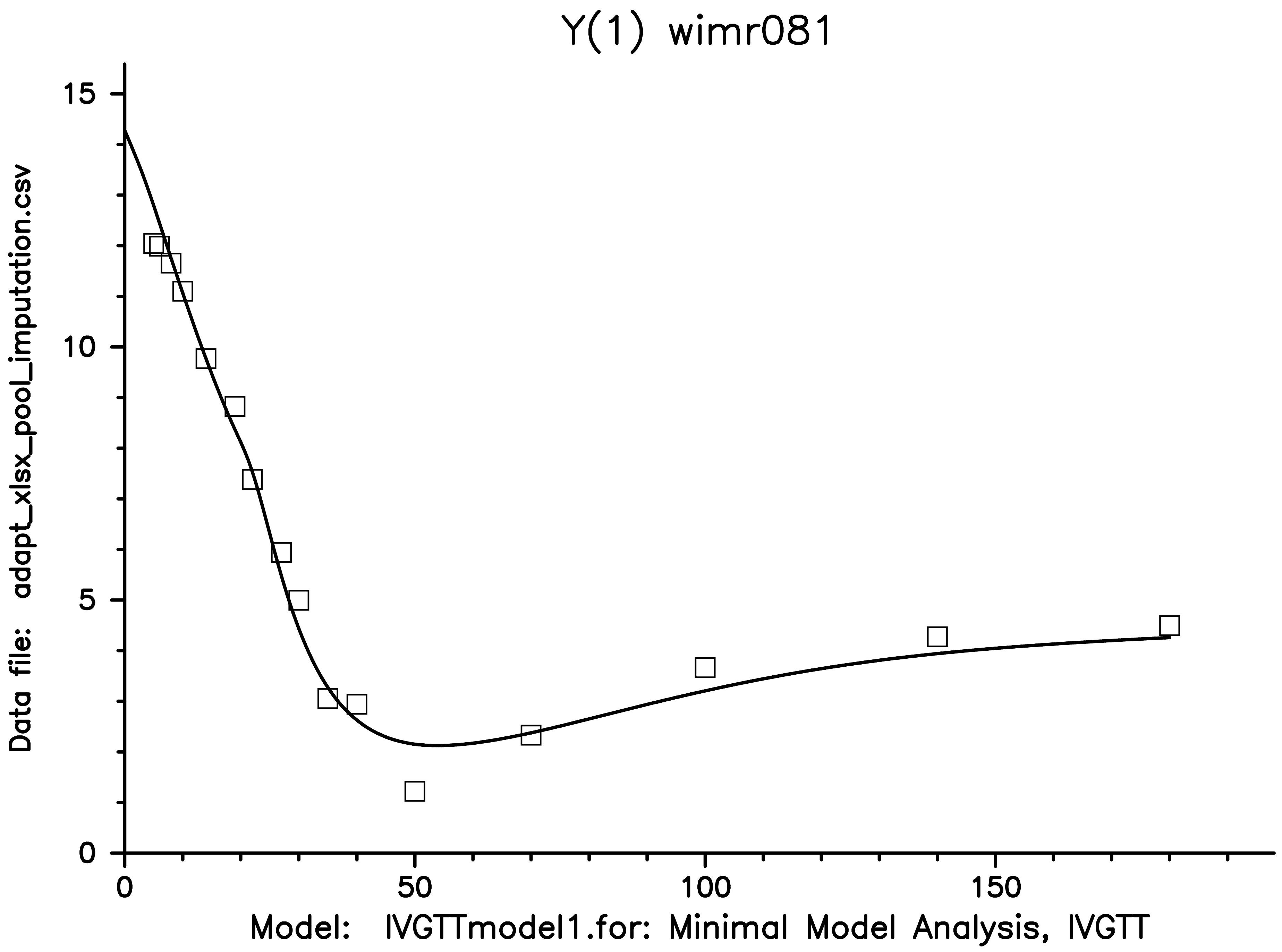

Y(1) wimr081
Data file: adapt_xlsx_pool_imputation.csv
Model: IVGTTmodel1.for: Minimal Model Analysis, IVGTT
15
10
5
0
0
50
100
150

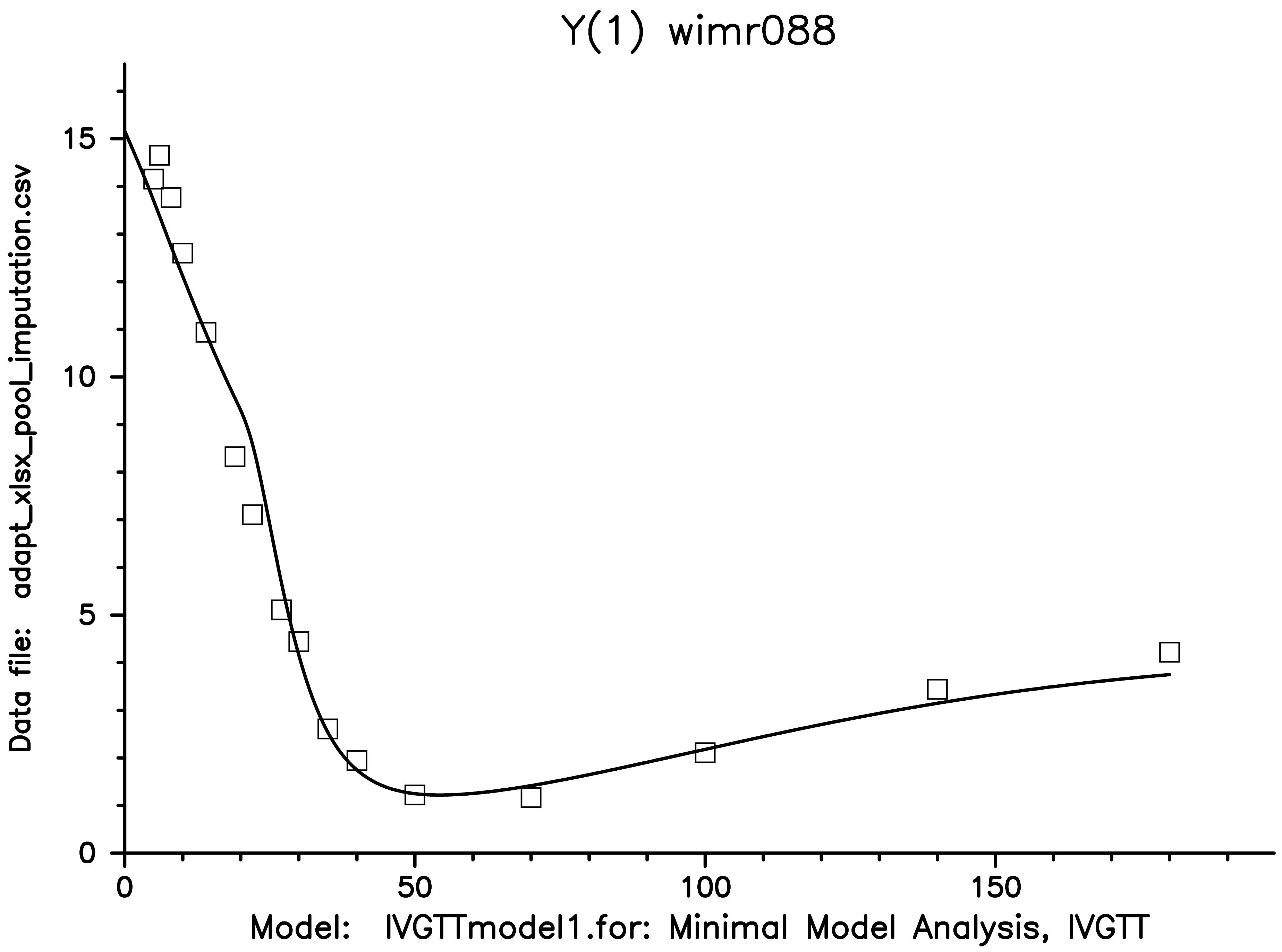
Y(1) wimr088
Data file: adapt_xlsx_pool_imputation.csv
Model: IVGTTmodel1.for: Minimal Model Analysis, IVGTT
0
5
10
15
0
50
100
150

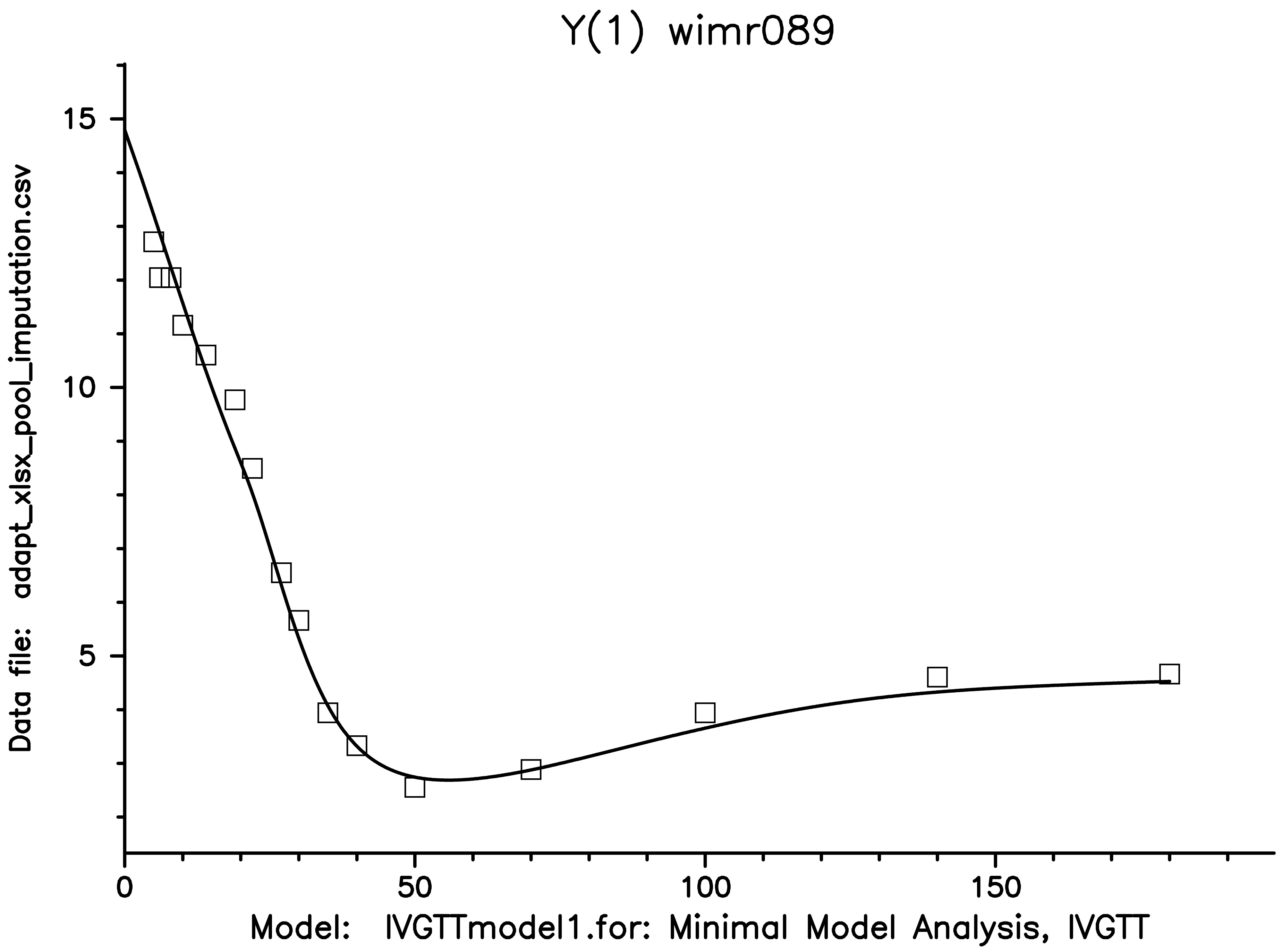

Y(1) wimr089
15
10
5
0
50
100
150
Data file: adapt_xlsx_pool_imputation.csv
Model: IVGTTmodel1.for: Minimal Model Analysis, IVGTT

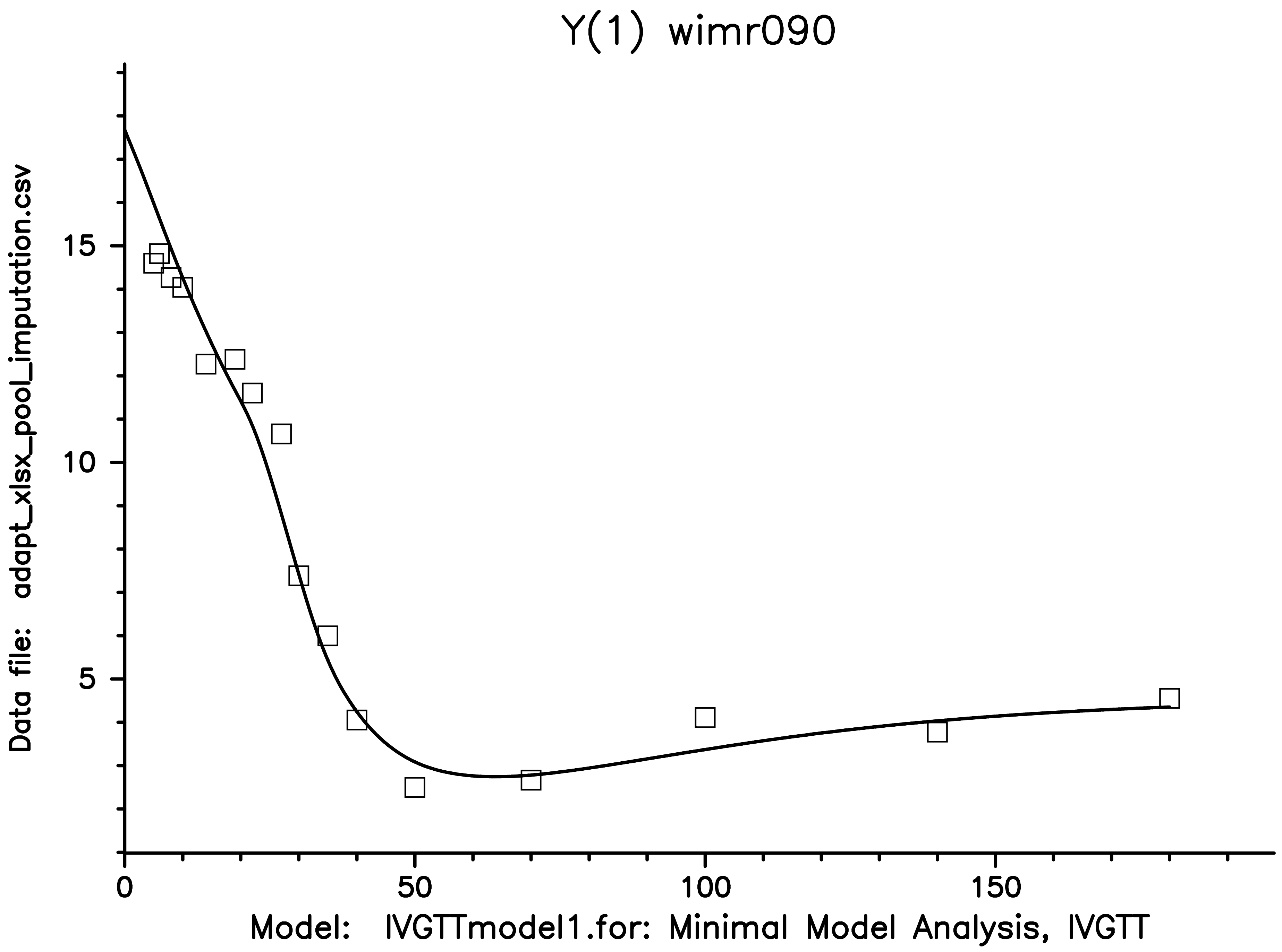
Y(1) wimr090
Data file: adapt_xlsx_pool_imputation.csv
15
10
5
0
50
100
150
Model: IVGTTmodel1.for: Minimal Model Analysis, IVGTT

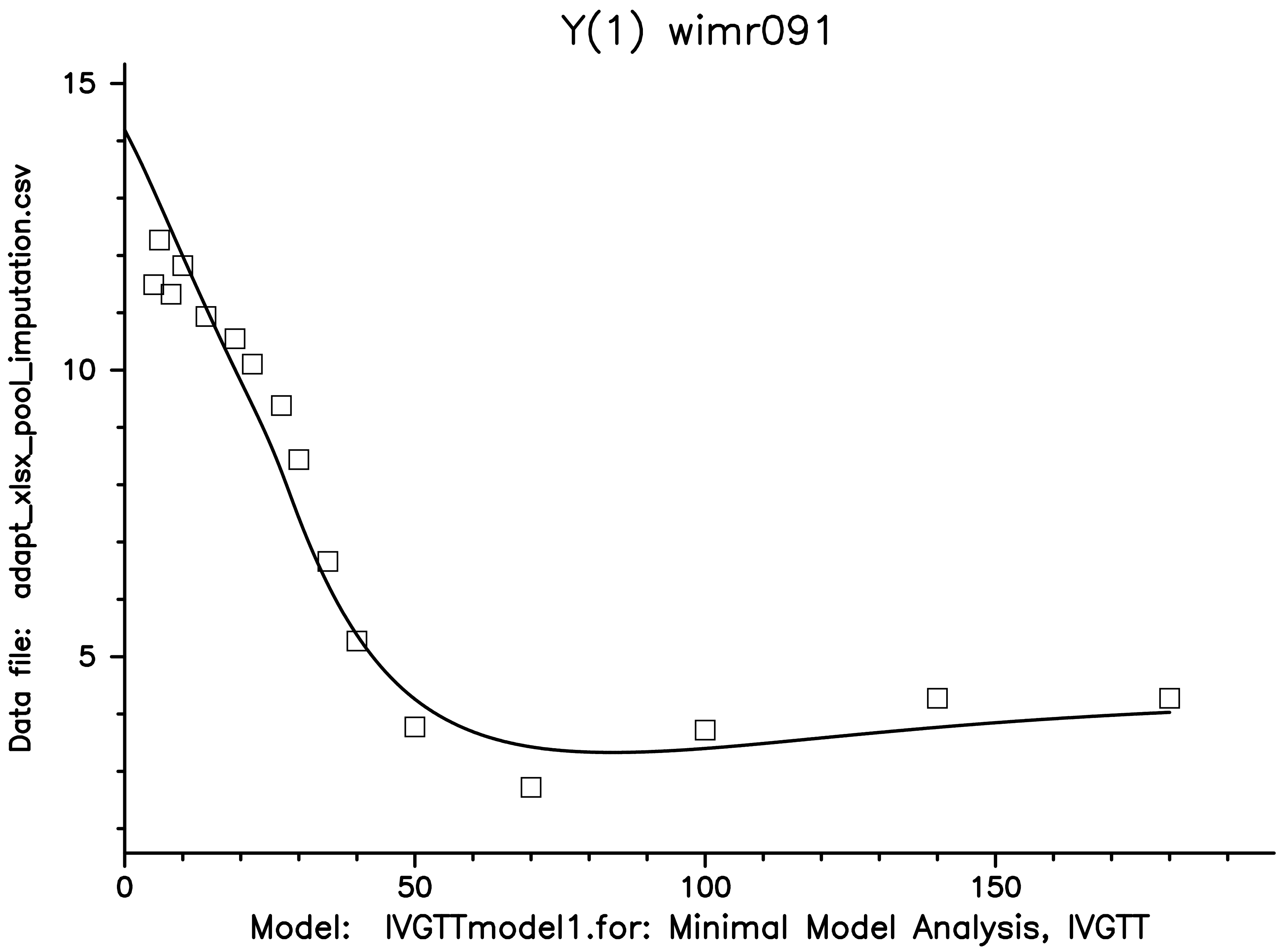

Y(1) wimr091
15
10
5
0
50
100
150
Data file: adapt_xlsx_pool_imputation.csv
Model: IVGTTmodel1.for: Minimal Model Analysis, IVGTT

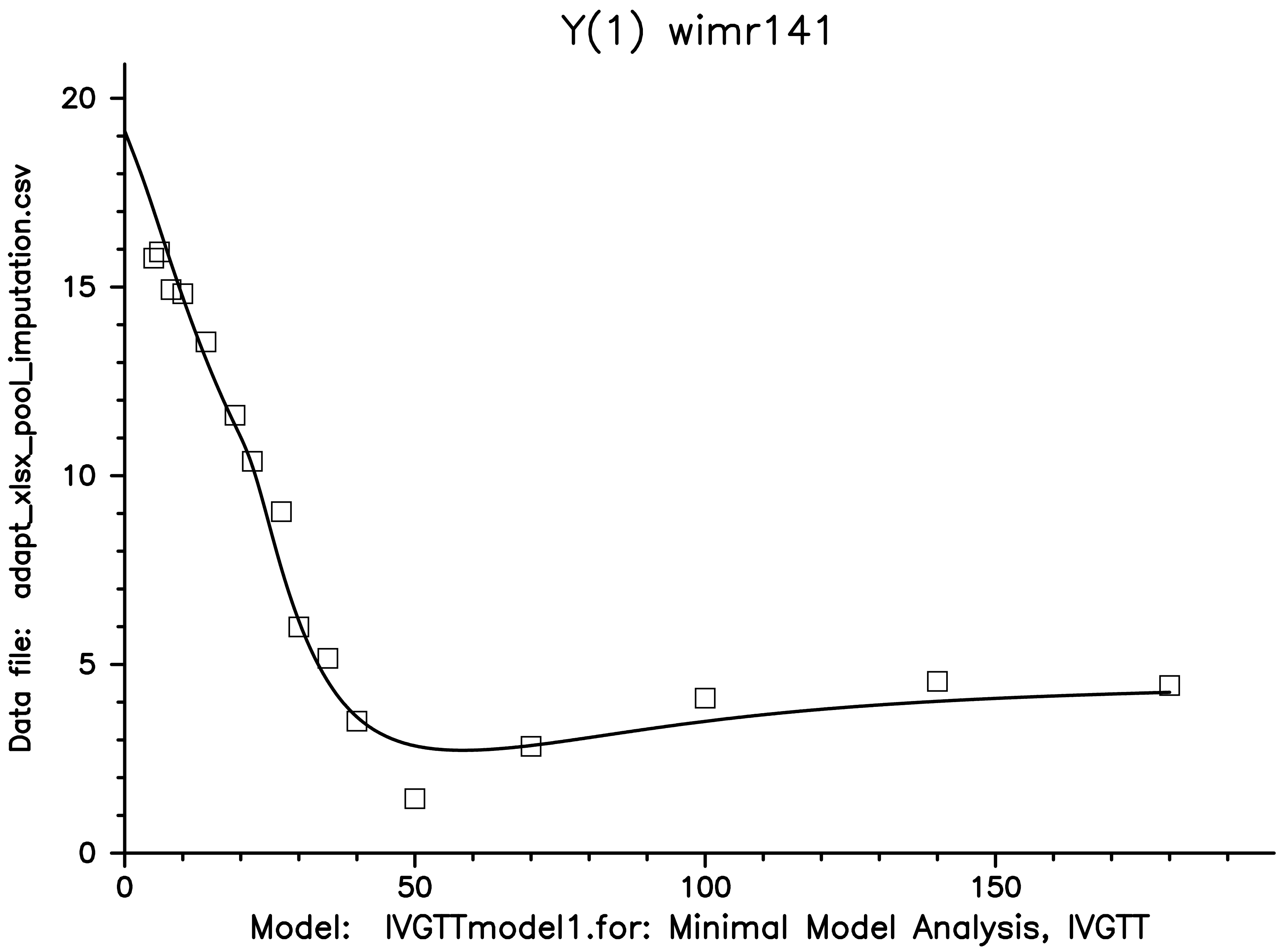
Y(1) wimr141
Data file: adapt_xlsx_pool_imputation.csv
Model: IVGTTmodel1.for: Minimal Model Analysis, IVGTT
0
5
10
15
20
0
50
100
150

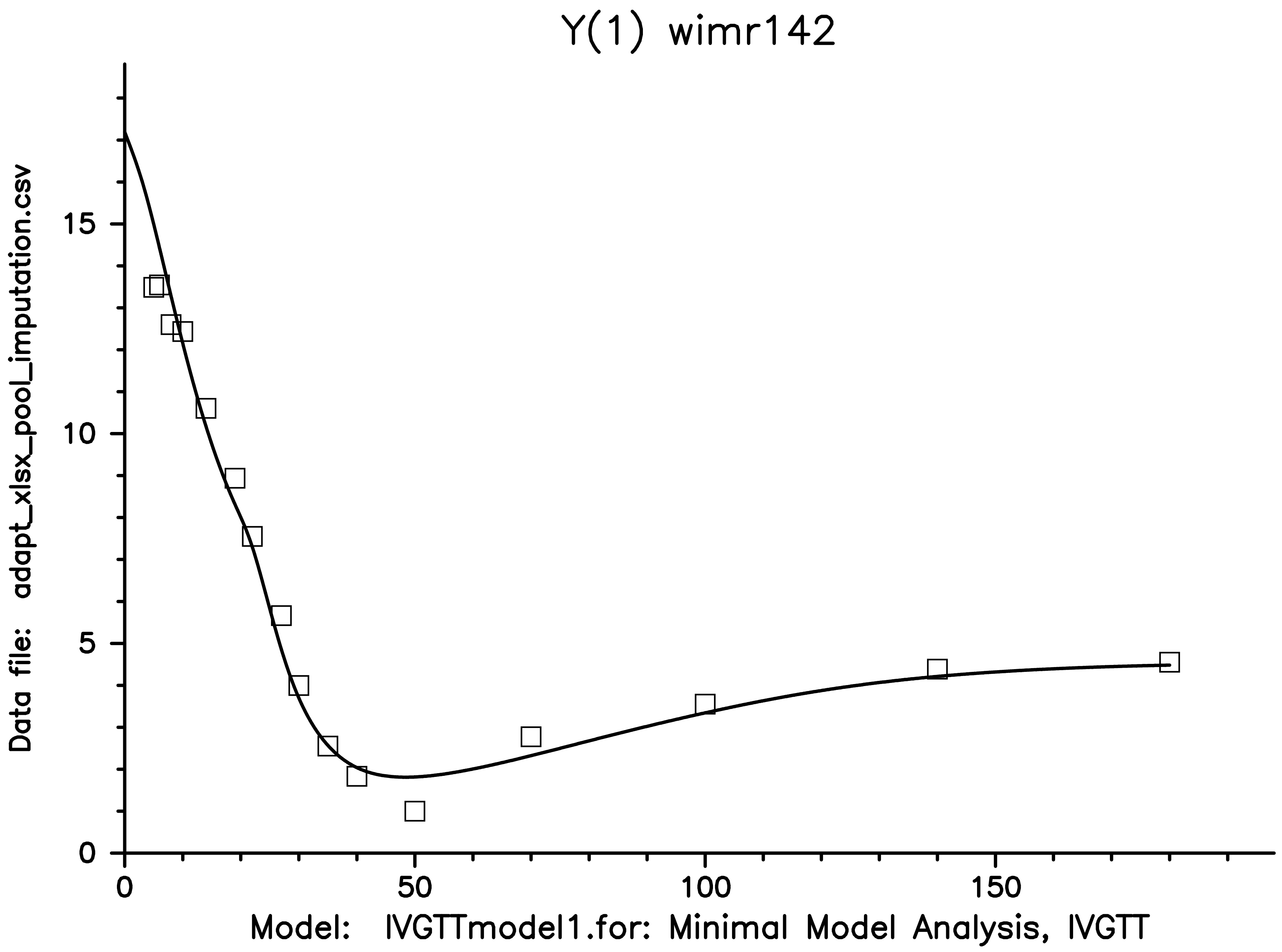

Y(1) wimr142
Data file: adapt_xlsx_pool_imputation.csv
Model: IVGTTmodel1.for: Minimal Model Analysis, IVGTT
0
5
10
15
0
50
100
150

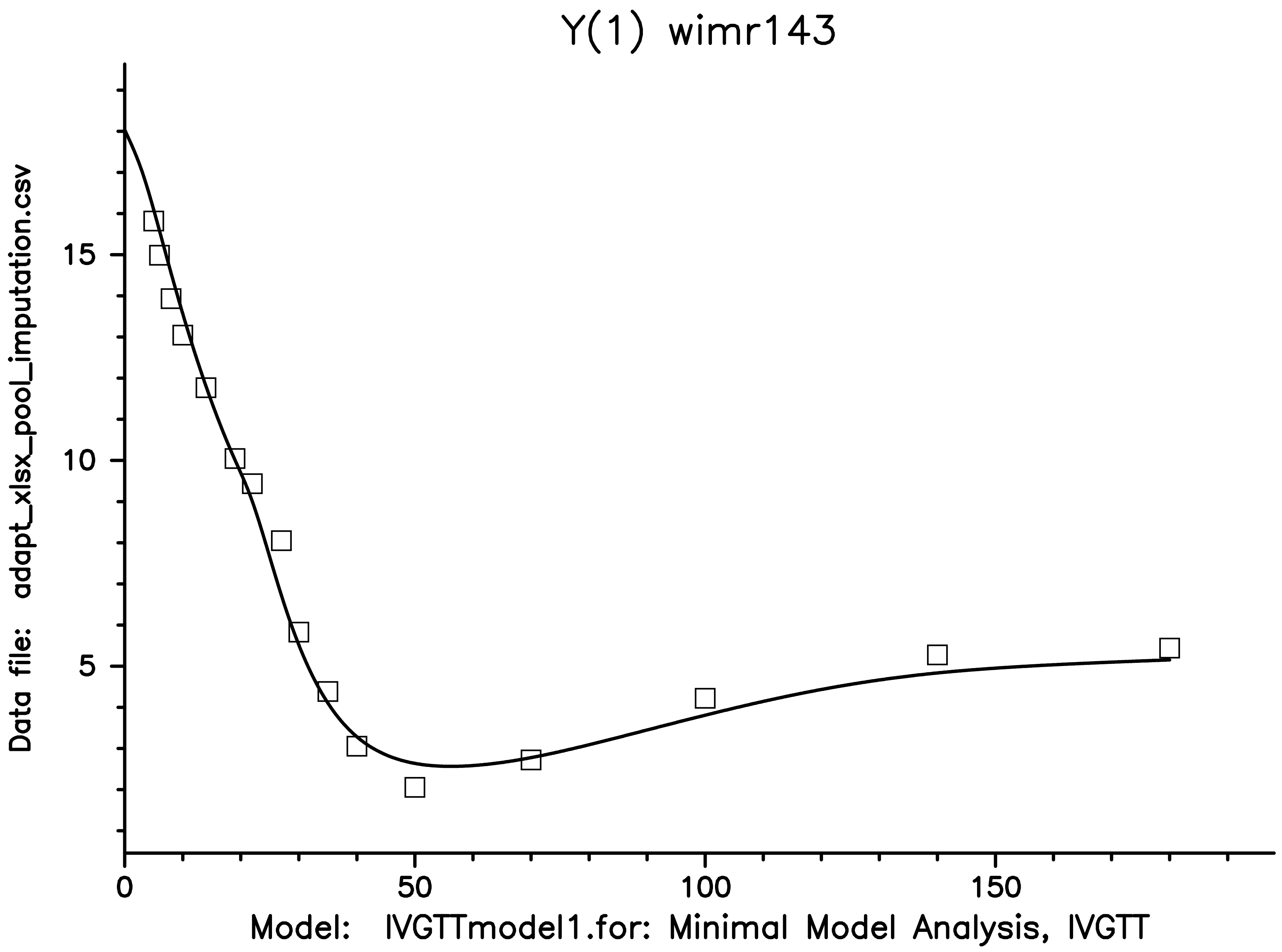
Y(1) wimr143
Data file: adapt_xlsx_pool_imputation.csv
15
10
5
0
50
100
150
Model: IVGTTmodel1.for: Minimal Model Analysis, IVGTT

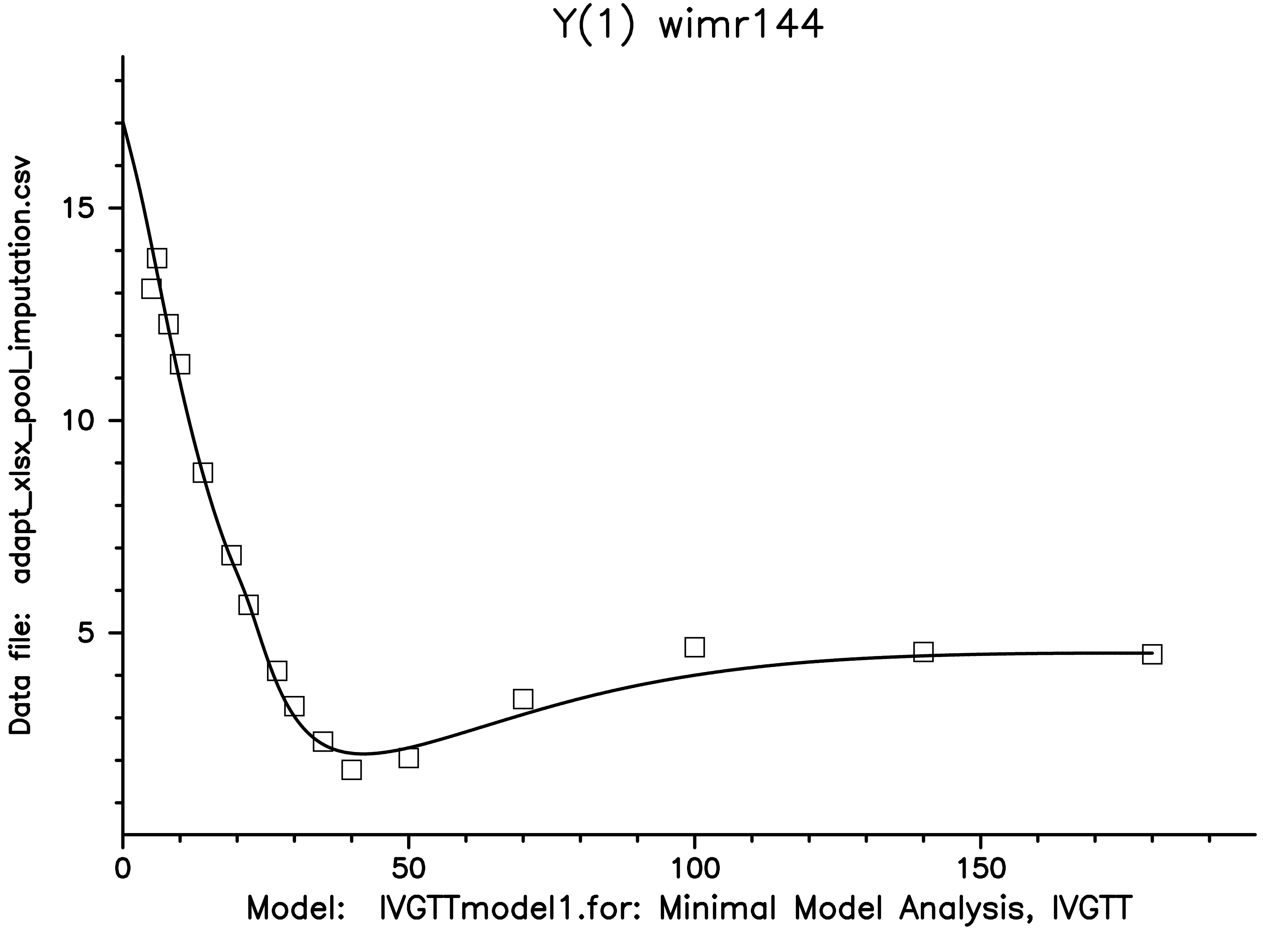

Y(1) wimr144
Data file: adapt_xlsx_pool_imputation.csv
15
10
5
0
50
100
150
Model: IVGTTmodel1.for: Minimal Model Analysis, IVGTT

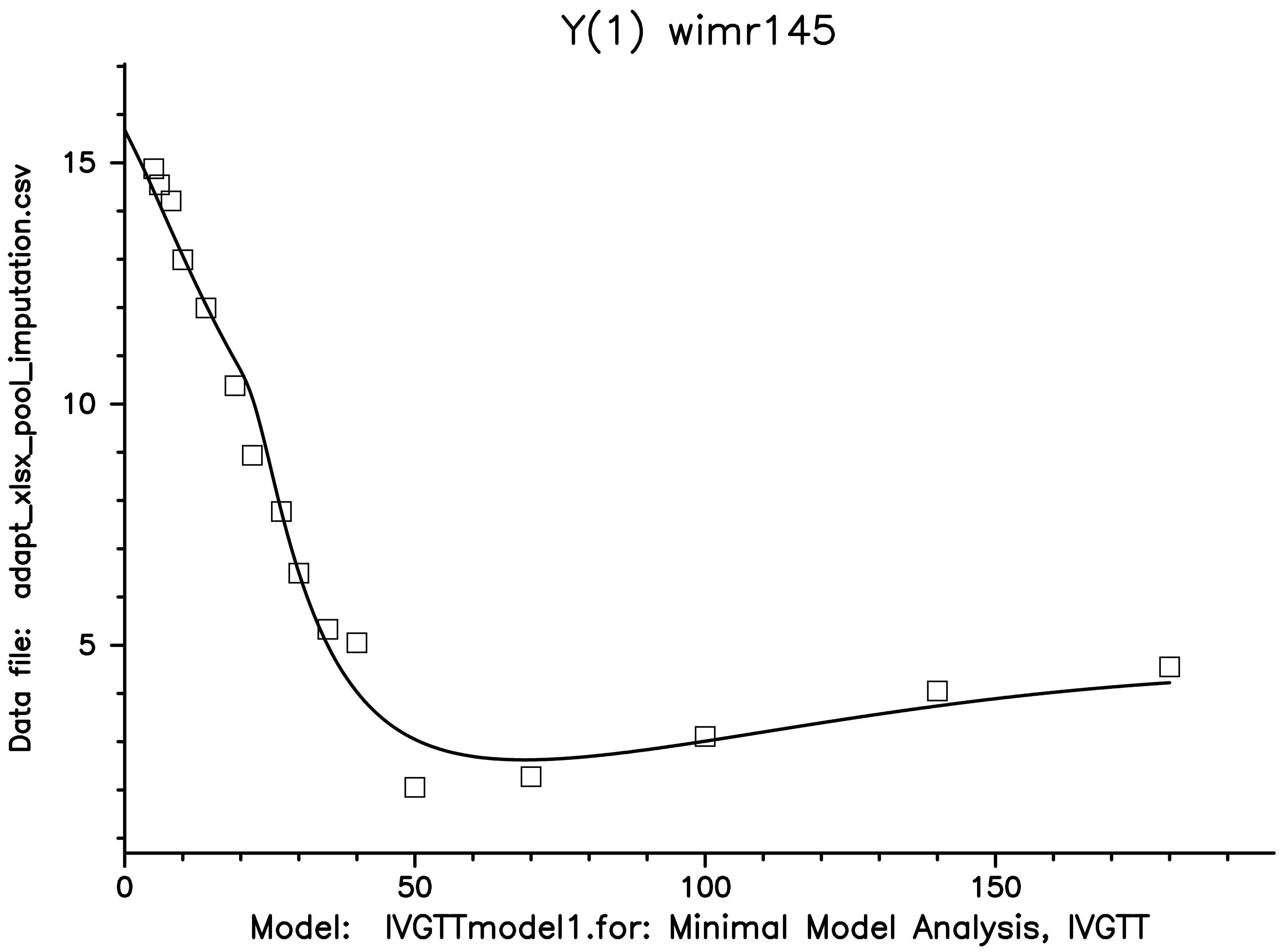
Y(1) wimr145
Data file: adapt_xlsx_pool_imputation.csv
Model: IVGTTmodel1.for: Minimal Model Analysis, IVGTT
15
10
5
0
50
100
150

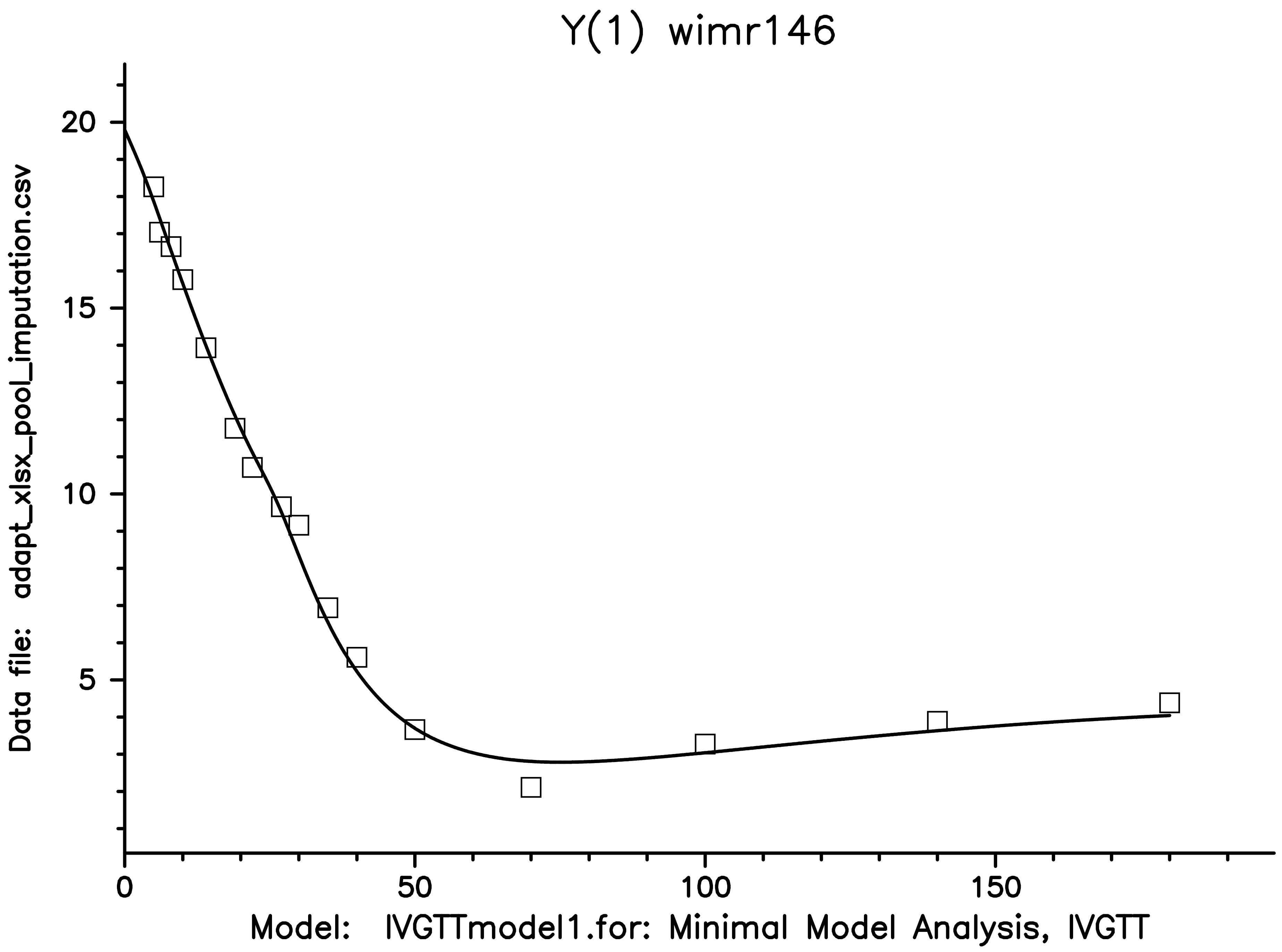

Y(1) wimr146
Data file: adapt_xlsx_pool_imputation.csv
20
15
10
5
0
50
100
150
Model: IVGTTmodel1.for: Minimal Model Analysis, IVGTT

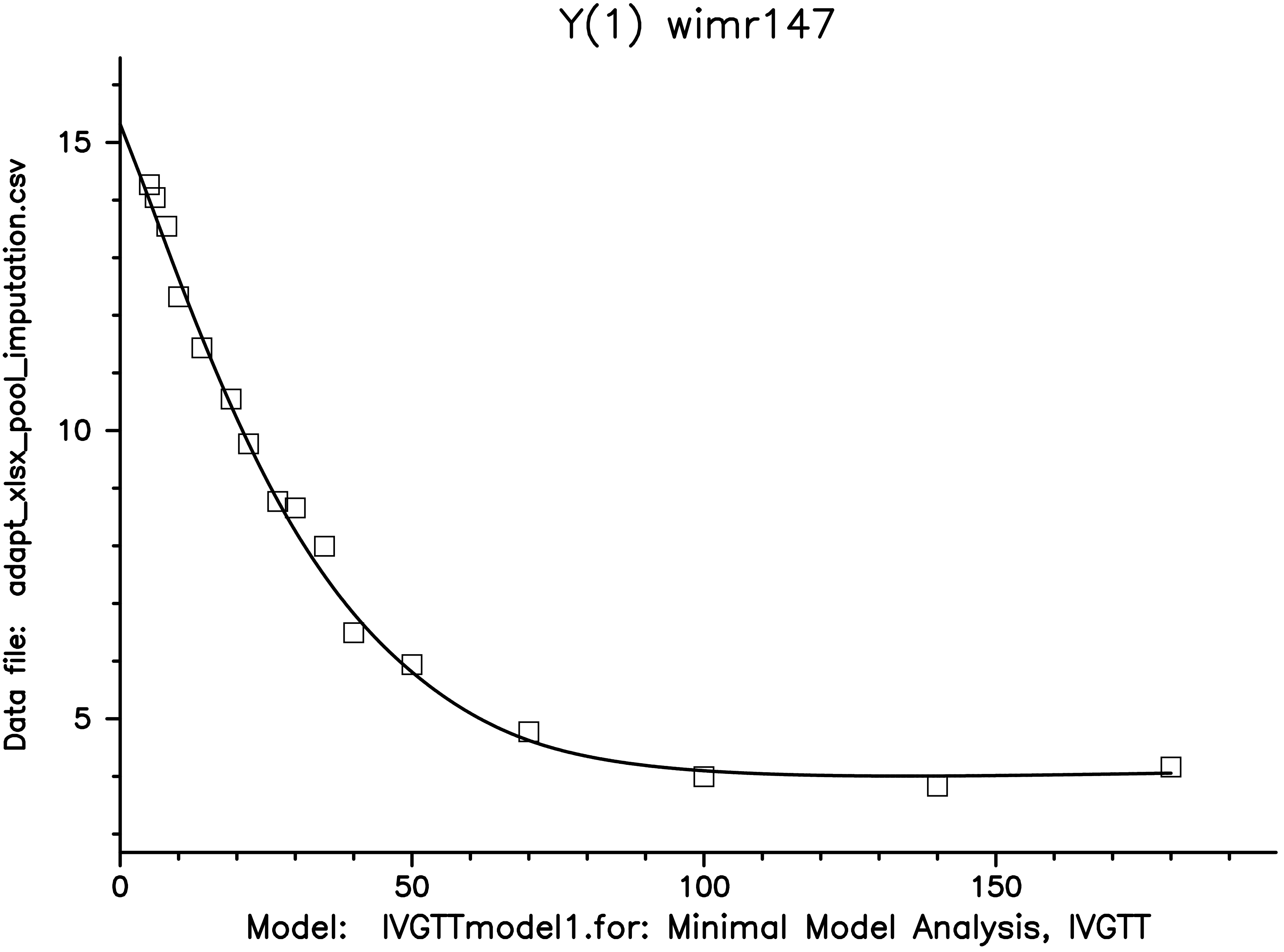
Y(1) wimr147
Data file: adapt_xlsx_pool_imputation.csv
15
10
5
0
50
100
150
Model: IVGTTmodel1.for: Minimal Model Analysis, IVGTT

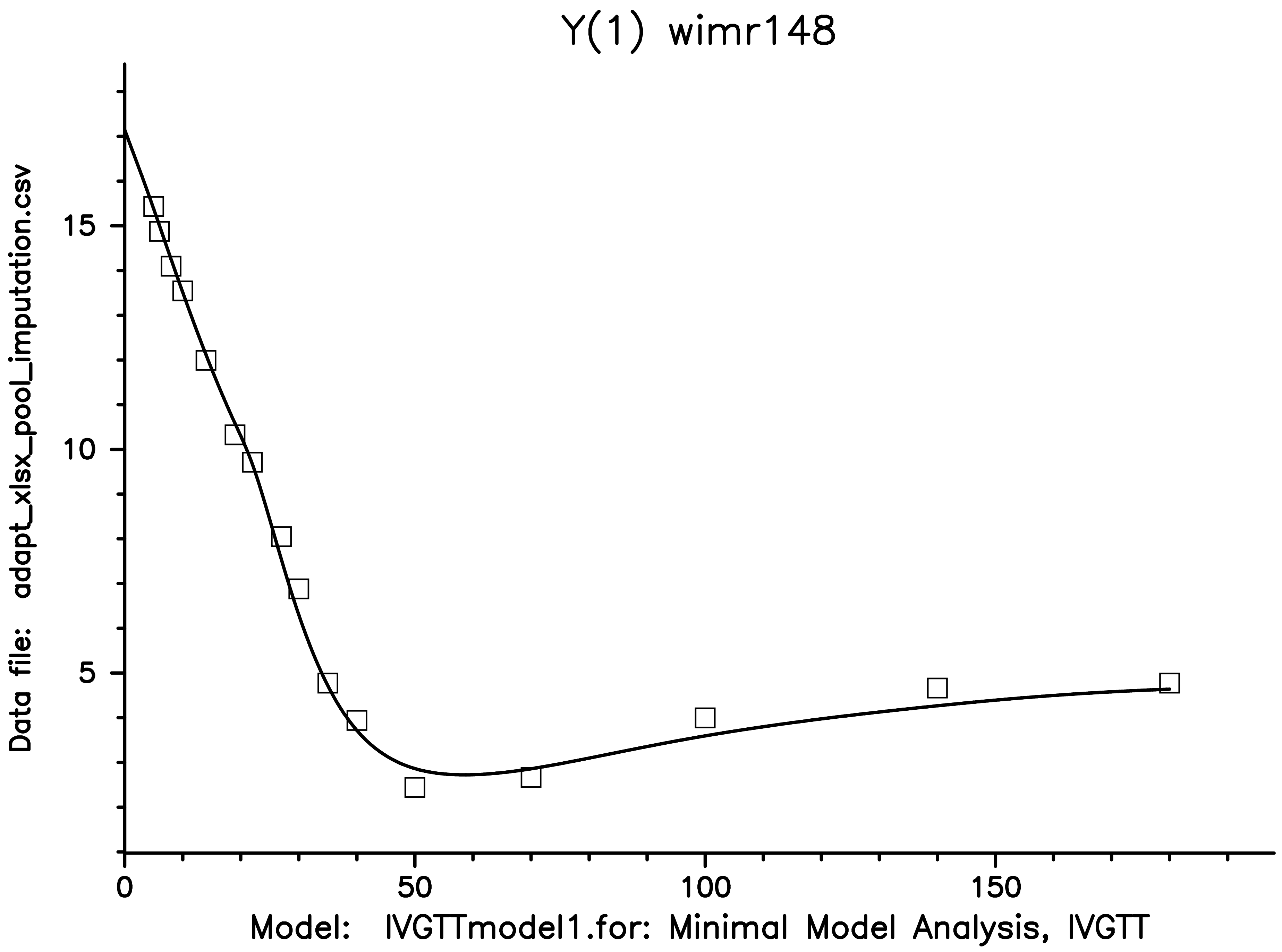

Y(1) wimr148
Data file: adapt_xlsx_pool_imputation.csv
15
10
5
0
50
100
150
Model: IVGTTmodel1.for: Minimal Model Analysis, IVGTT

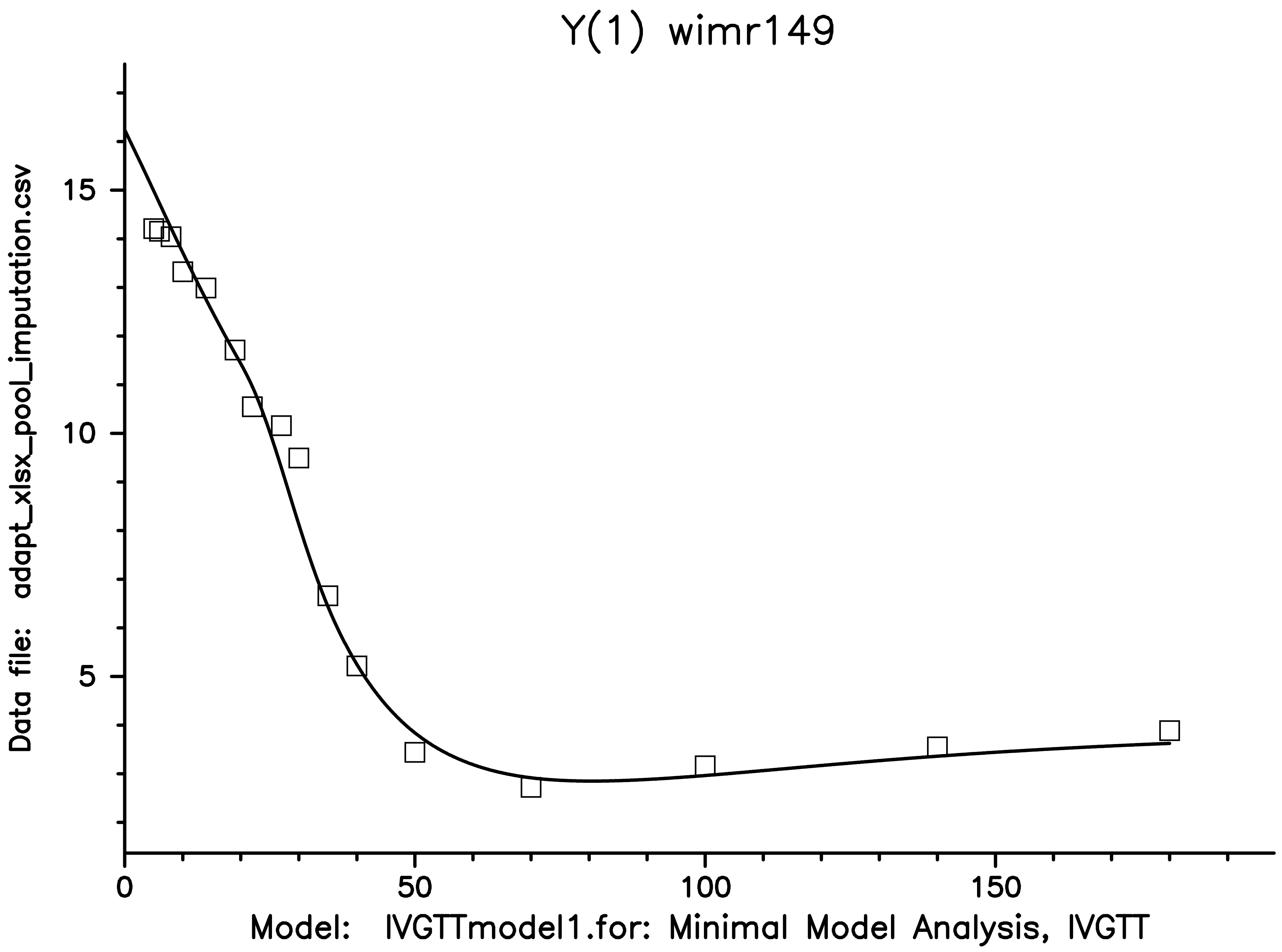
Y(1) wimr149
Data file: adapt_xlsx_pool_imputation.csv
Model: IVGTTmodel1.for: Minimal Model Analysis, IVGTT
15
10
5
0
50
100
150

Y(1) wimr150

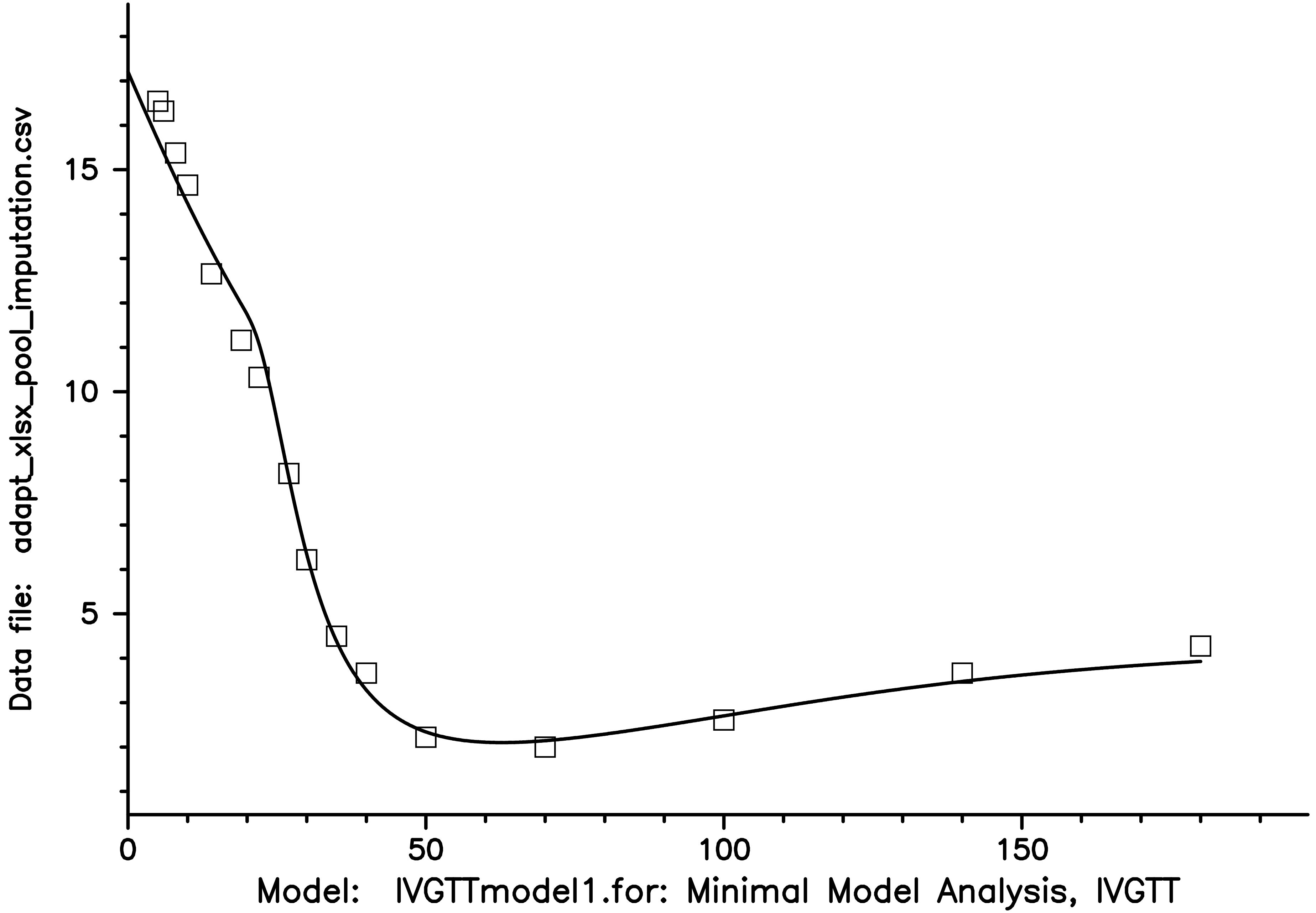

15
10
5
0
50
100
150
Data file: adapt_xlsx_pool_imputation.csv
Model: IVGTTmodel1.for: Minimal Model Analysis, IVGTT

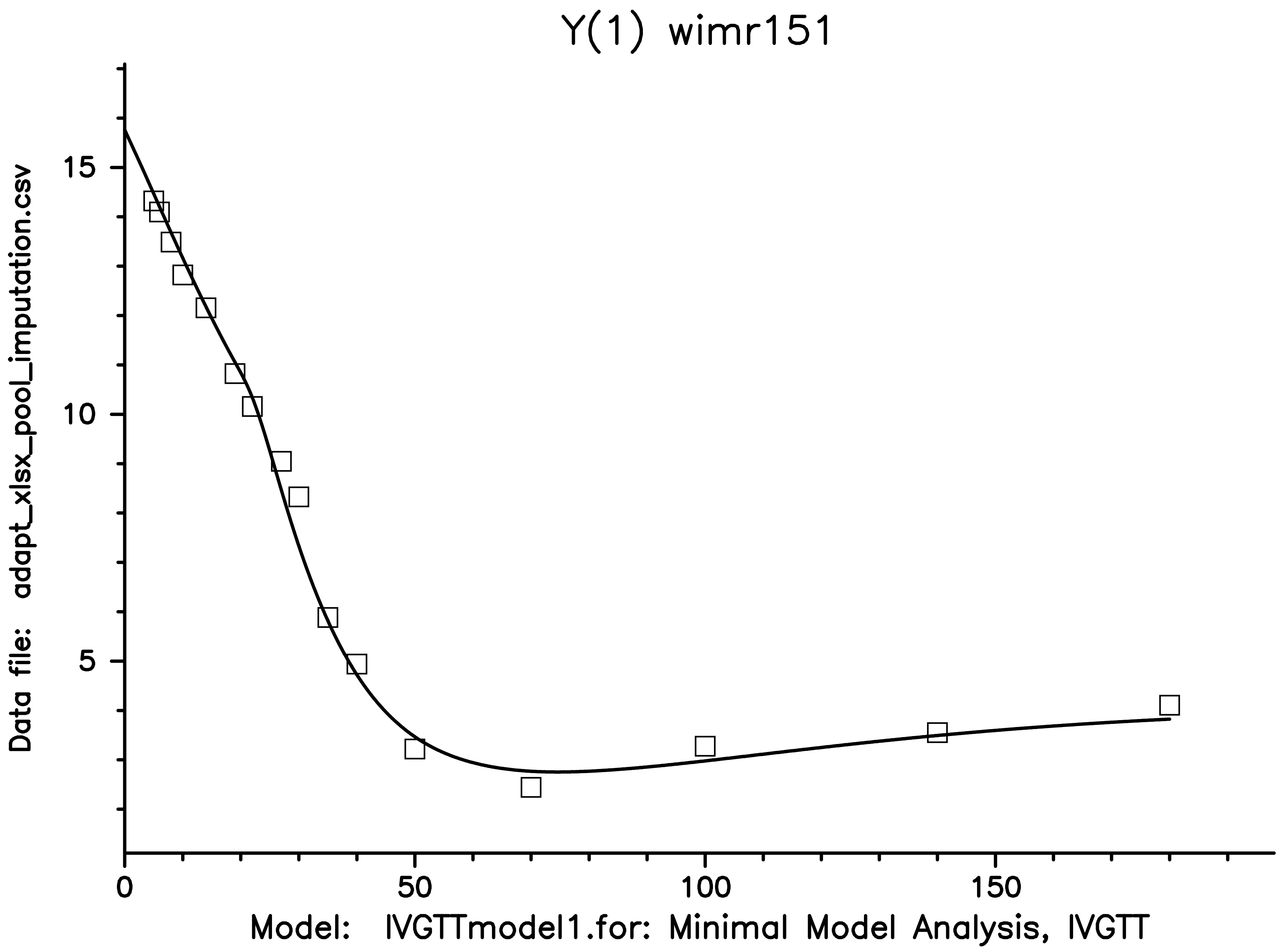
Y(1) wimr151
Data file: adapt_xlsx_pool_imputation.csv
Model: IVGTTmodel1.for: Minimal Model Analysis, IVGTT
0
50
100
150
5
10
15

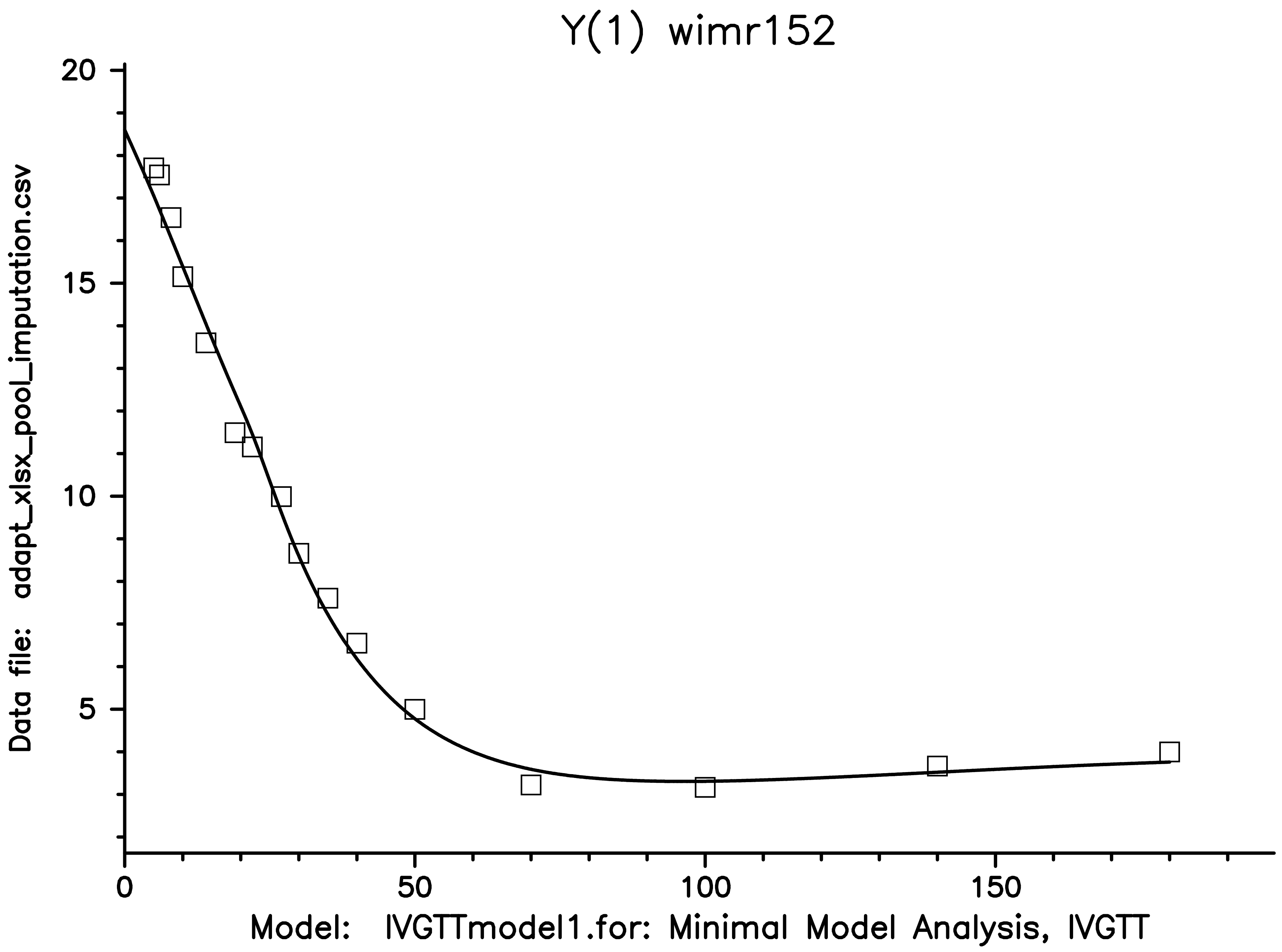
Y(1) wimr152
20
15
10
5
0
50
100
150
Data file: adapt_xlsx_pool_imputation.csv
Model: IVGTTmodel1.for: Minimal Model Analysis, IVGTT

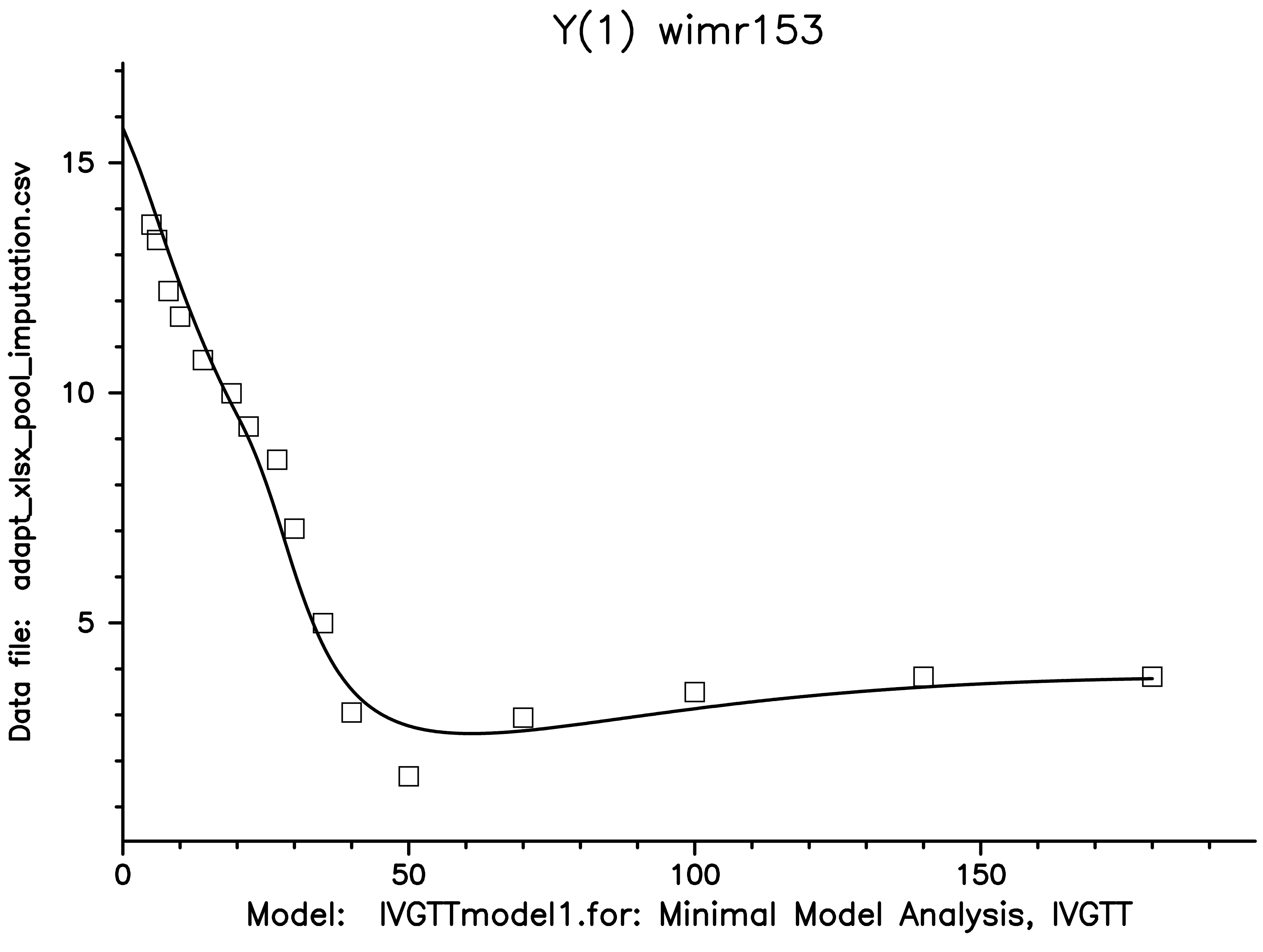

Y(1) wimr153
Data file: adapt_xlsx_pool_imputation.csv
15
10
5
0
50
100
150
Model: IVGTTmodel1.for: Minimal Model Analysis, IVGTT

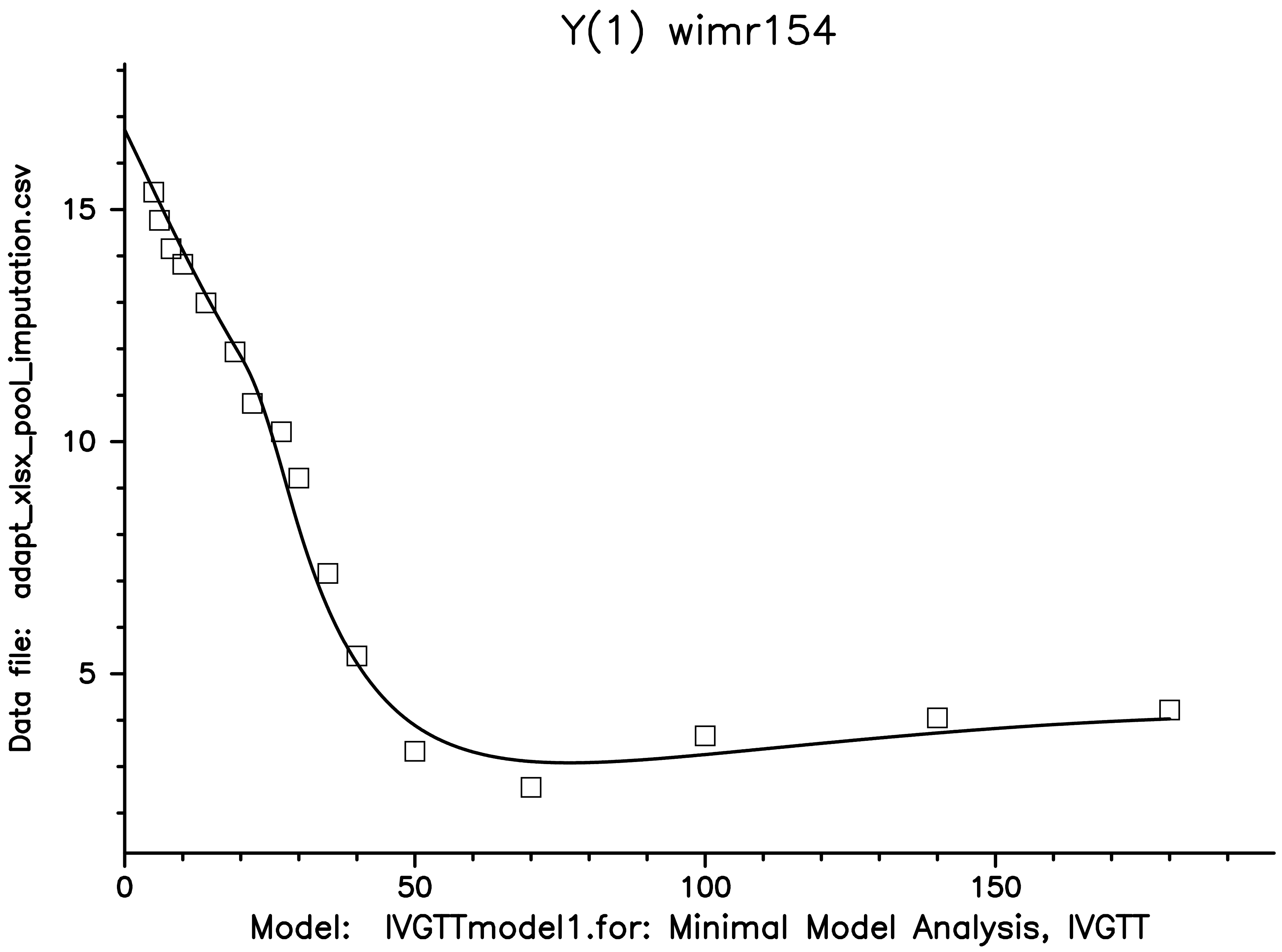
Y(1) wimr154
Data file: adapt_xlsx_pool_imputation.csv
15
10
5
0
50
100
150
Model: IVGTTmodel1.for: Minimal Model Analysis, IVGTT

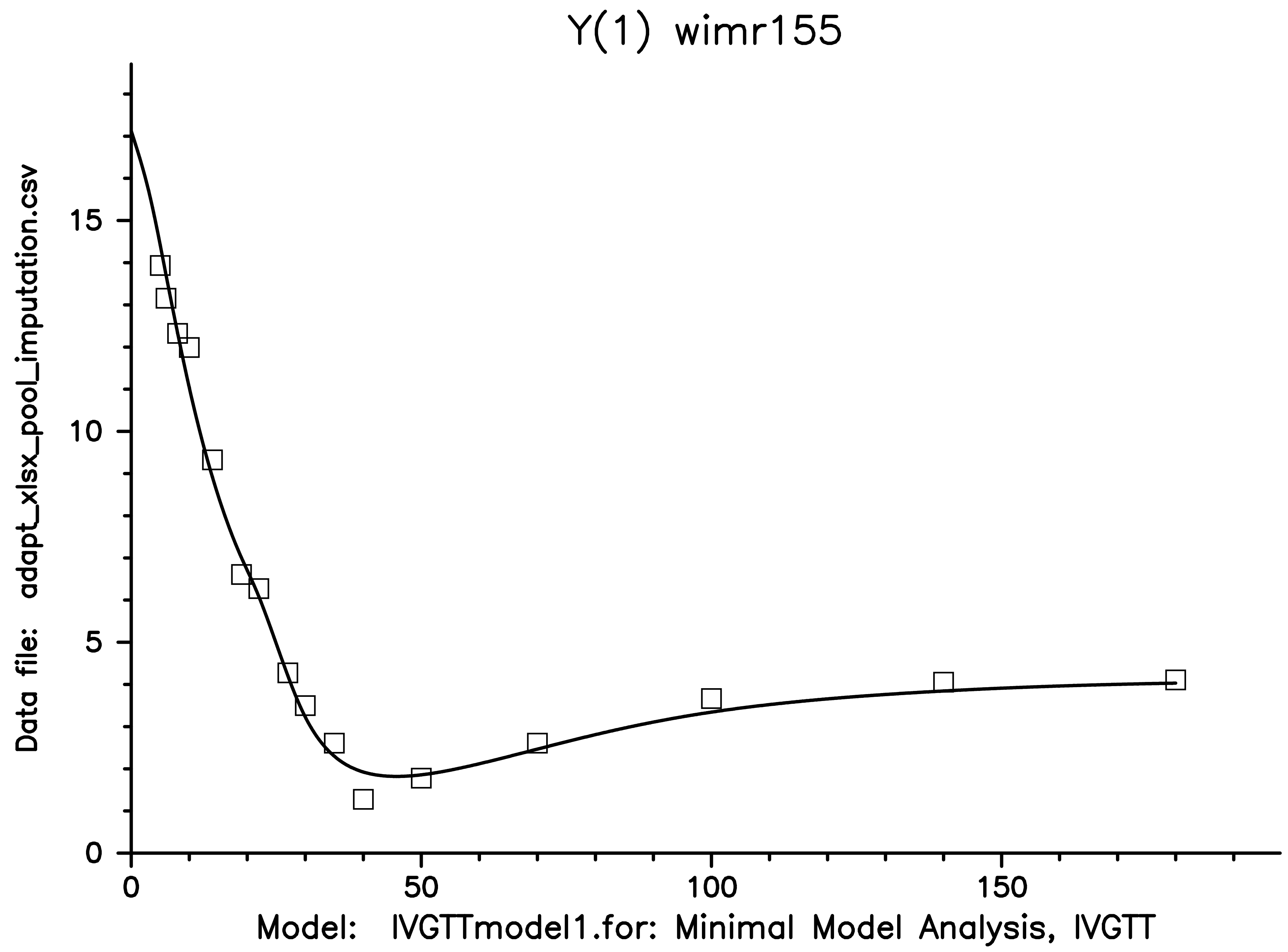

Y(1) wimr155
Data file: adapt_xlsx_pool_imputation.csv
Model: IVGTTmodel1.for: Minimal Model Analysis, IVGTT
0
5
10
15
0
50
100
150

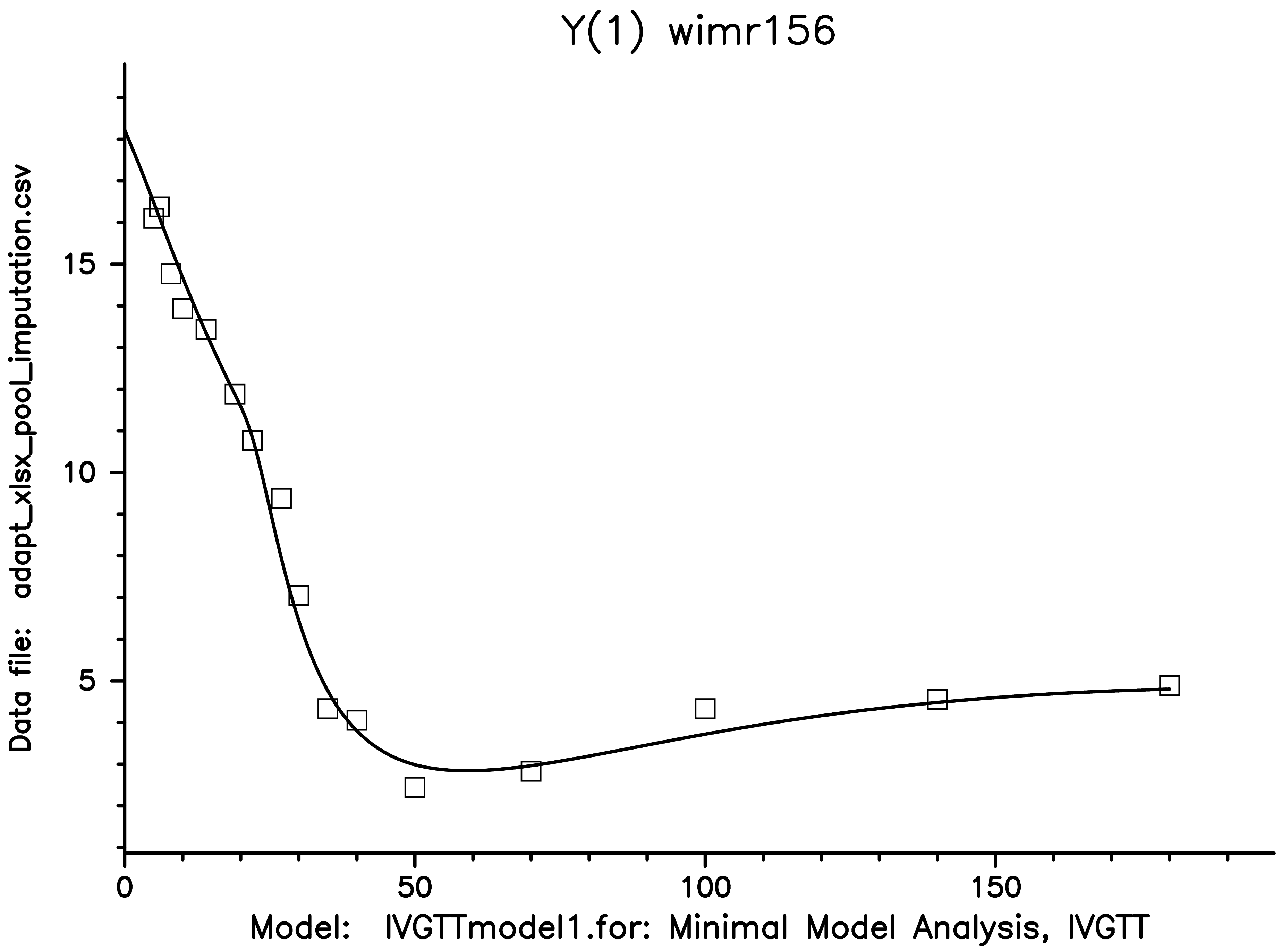
Y(1) wimr156
Data file: adapt_xlsx_pool_imputation.csv
15
10
5
0
50
100
150
Model: IVGTTmodel1.for: Minimal Model Analysis, IVGTT

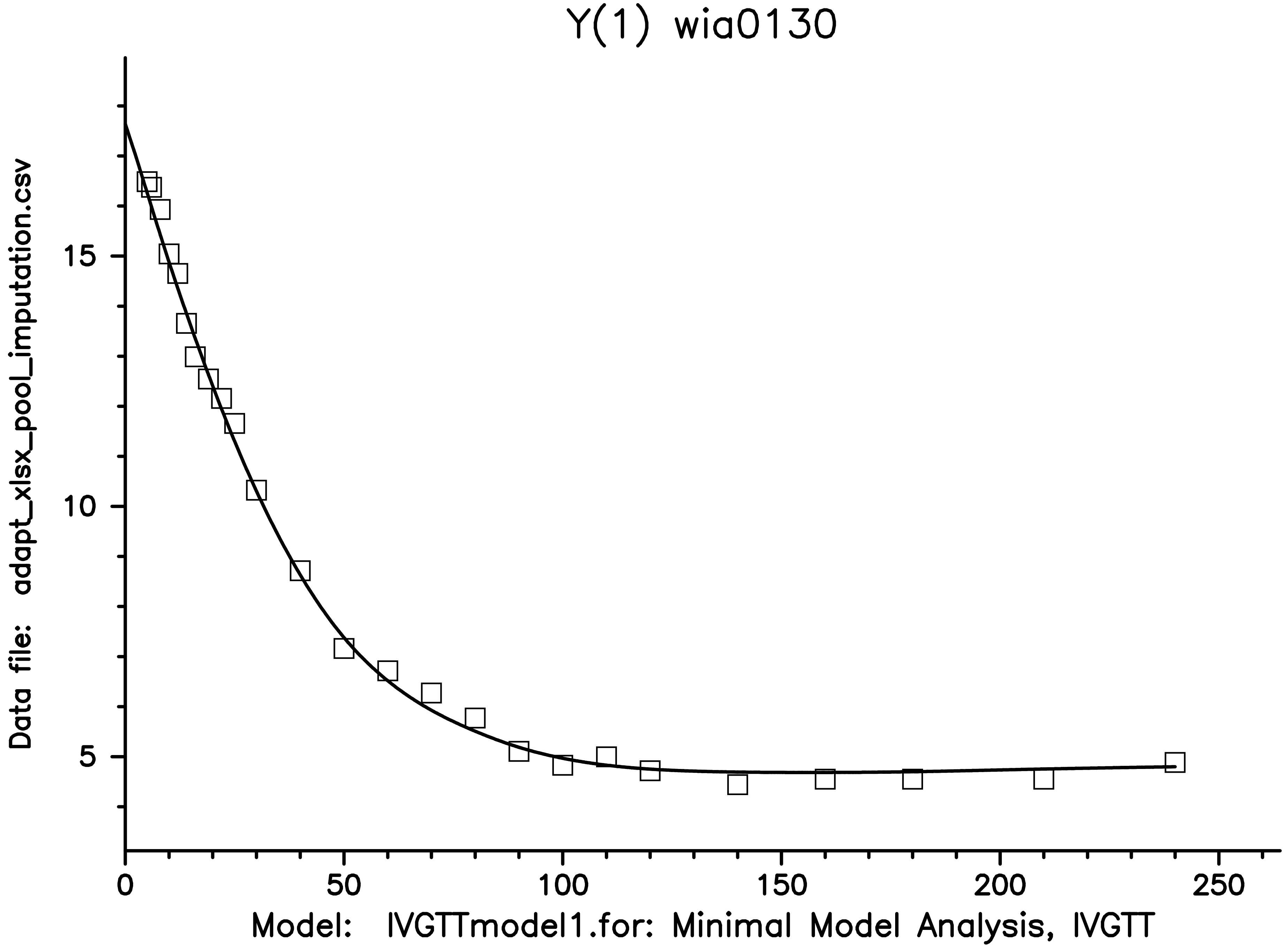

Y(1) wia0130
Data file: adapt_xlsx_pool_imputation.csv
15
10
5
0
50
100
150
200
250
Model: IVGTTmodel1.for: Minimal Model Analysis, IVGTT

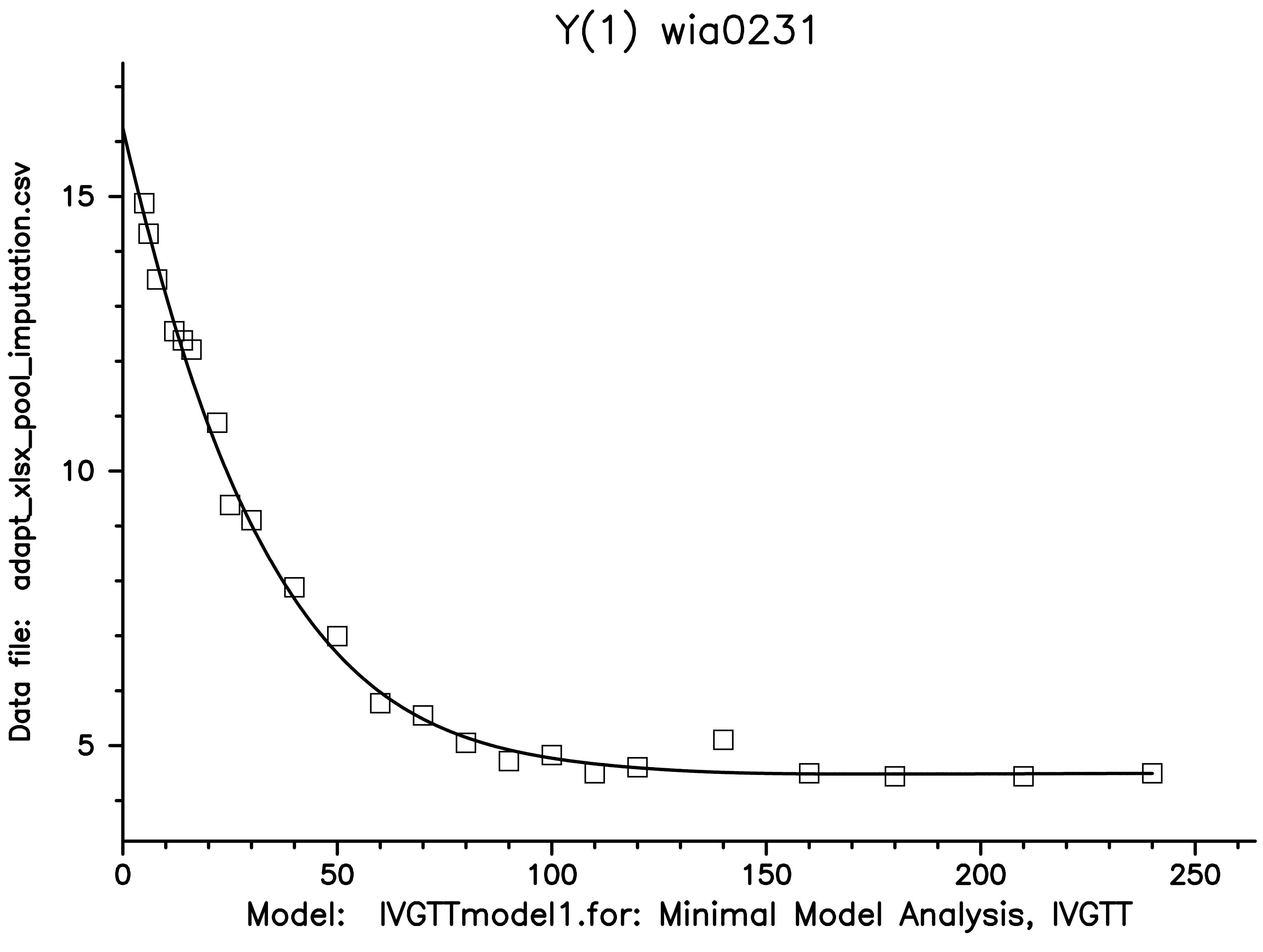
Y(1) wia0231
Data file: adapt_xlsx_pool_imputation.csv
15
10
5
0
50
100
150
200
250
Model: IVGTTmodel1.for: Minimal Model Analysis, IVGTT

Y(1) wia0332

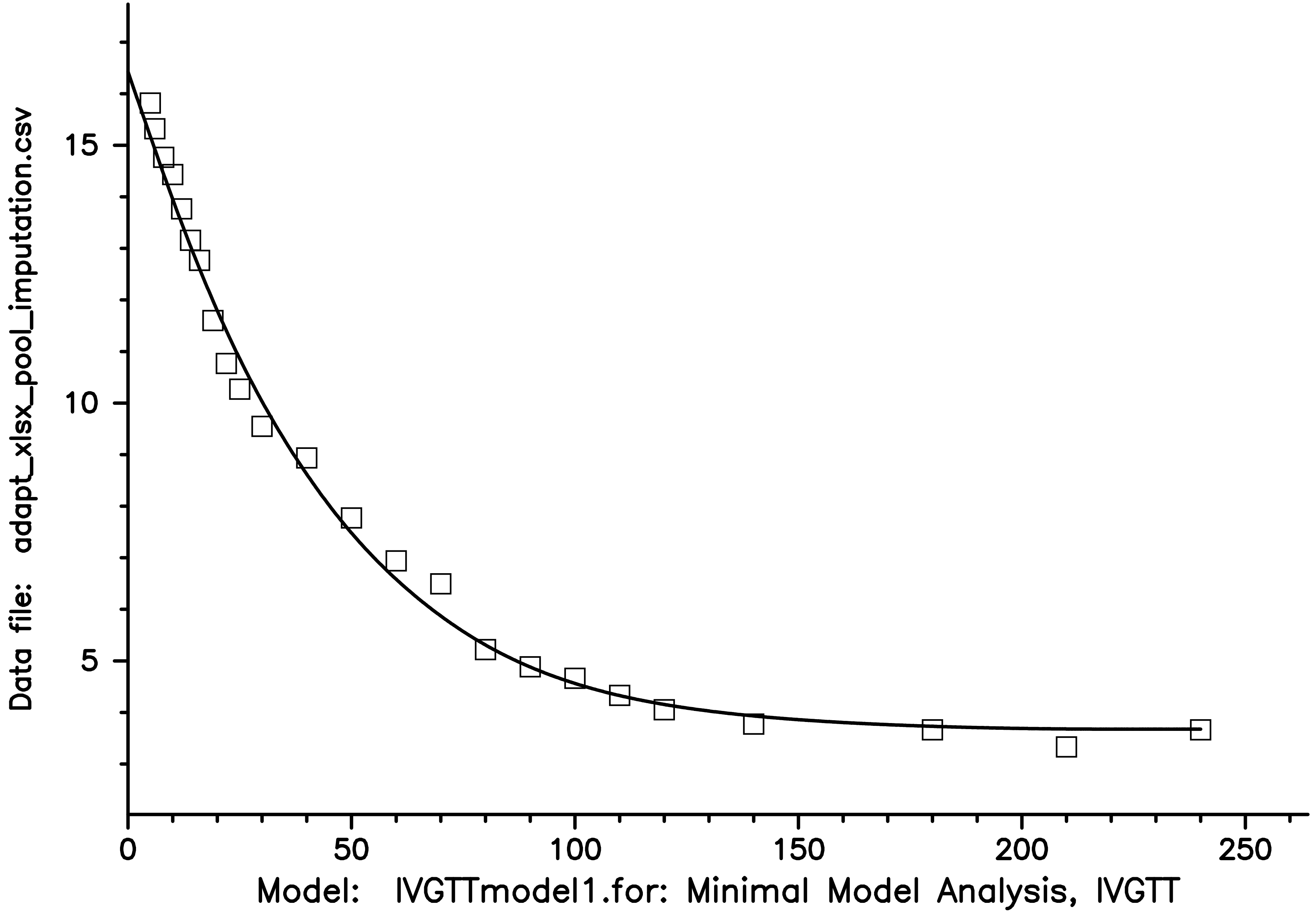

Data file: adapt_xlsx_pool_imputation.csv
15
10
5
0
50
100
150
200
250
Model: IVGTTmodel1.for: Minimal Model Analysis, IVGTT

Y(1) wia0433

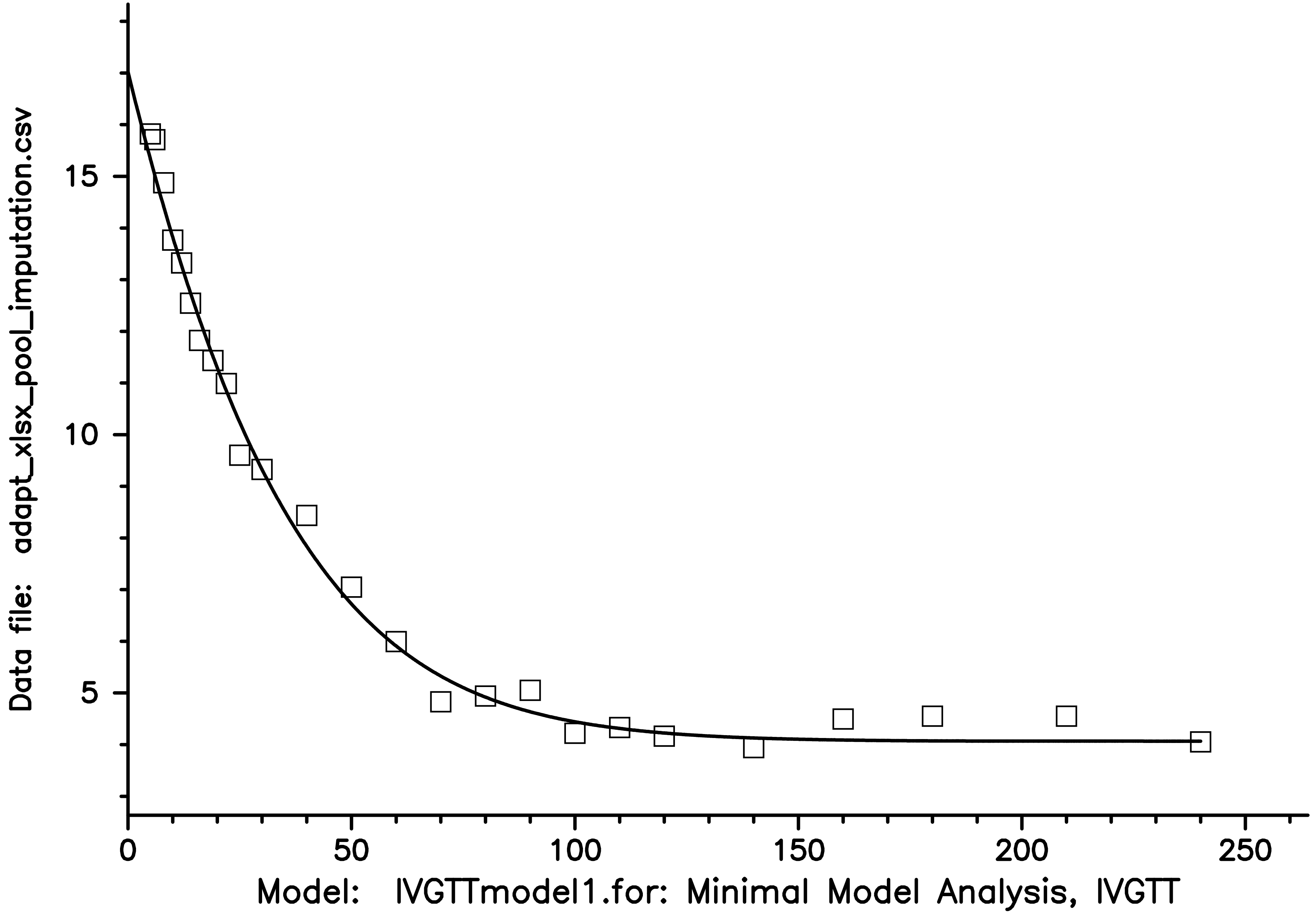

Data file: adapt_xlsx_pool_imputation.csv
15
10
5
0
50
100
150
200
250
Model: IVGTTmodel1.for: Minimal Model Analysis, IVGTT

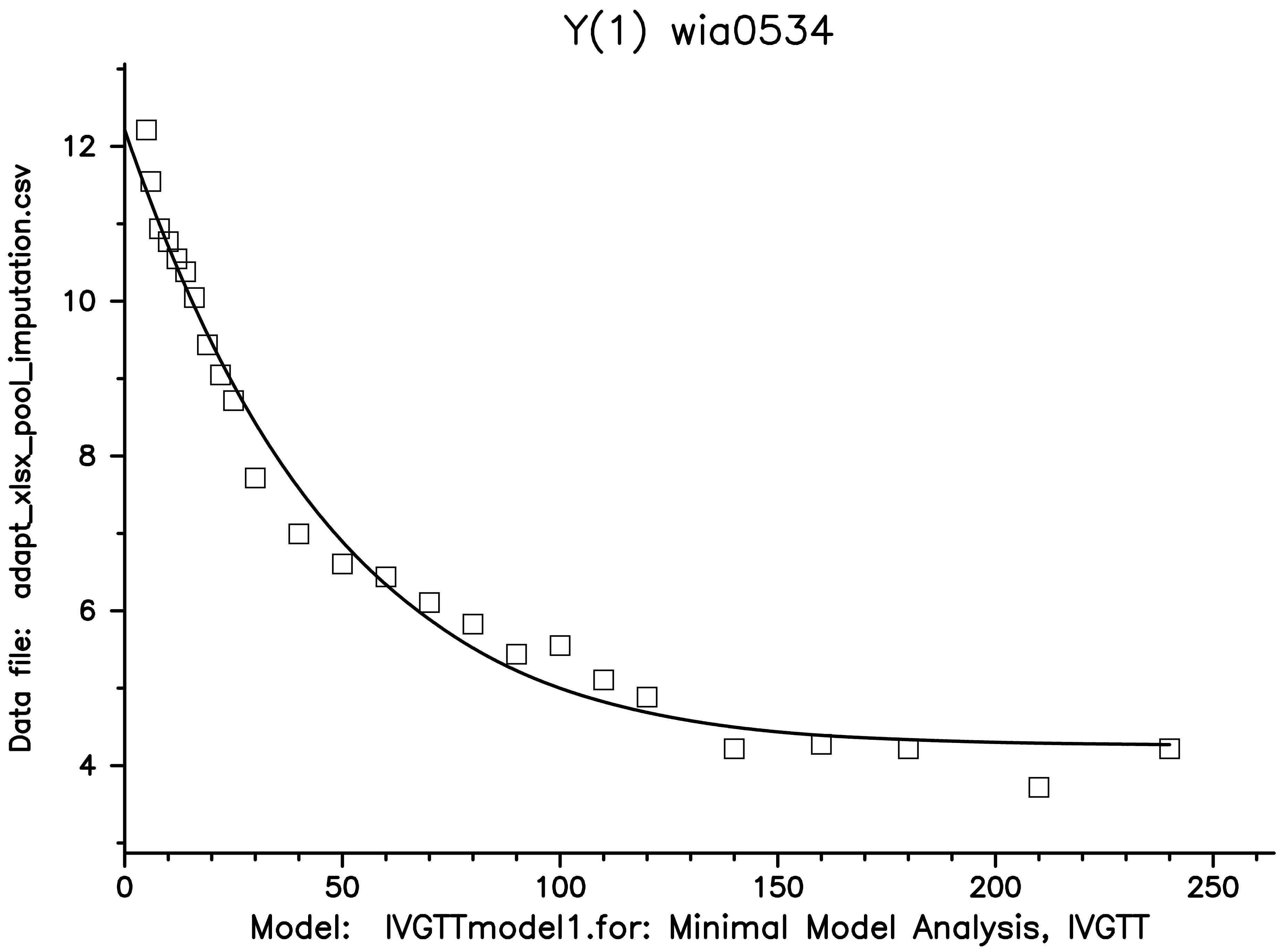

Y(1) wia0534
Data file: adapt_xlsx_pool_imputation.csv
12
10
8
6
4
0
50
100
150
200
250
Model: IVGTTmodel1.for: Minimal Model Analysis, IVGTT

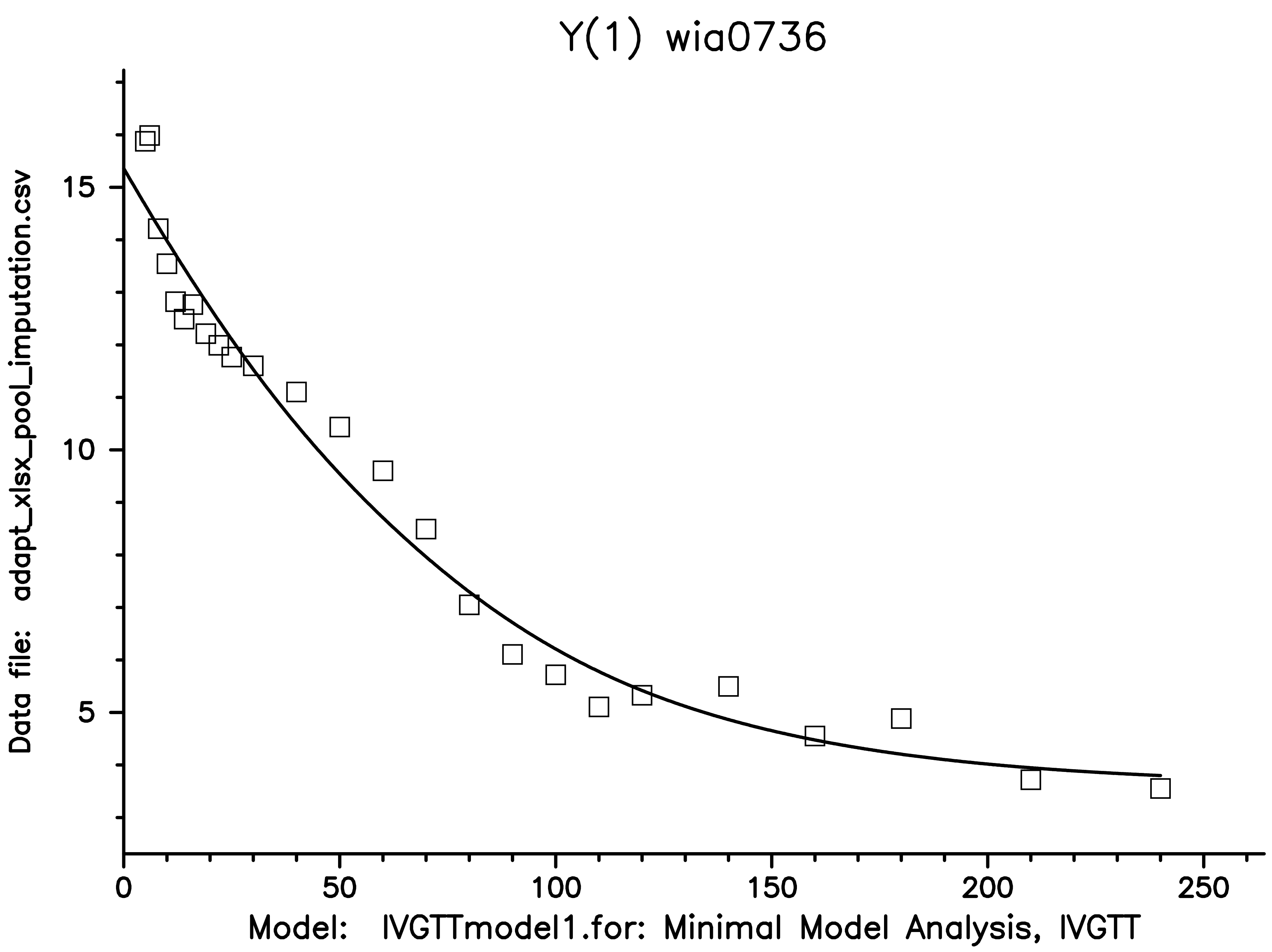

Y(1) wia0736
Data file: adapt_xlsx_pool_imputation.csv
Model: IVGTTmodel1.for: Minimal Model Analysis, IVGTT
15
10
5
0
50
100
150
200
250

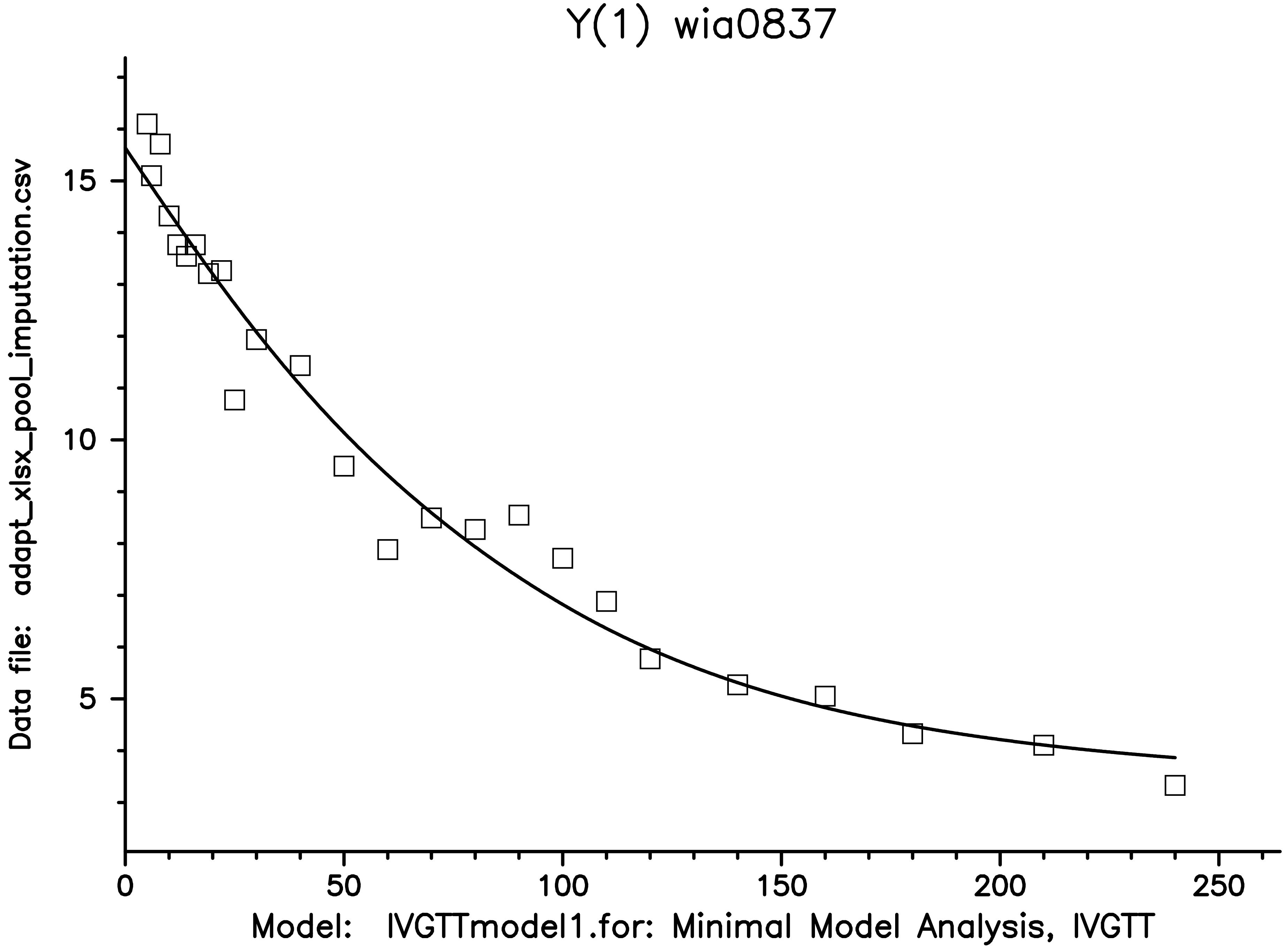

Y(1) wia0837
Data file: adapt_xlsx_pool_imputation.csv
15
10
5
0
50
100
150
200
250
Model: IVGTTmodel1.for: Minimal Model Analysis, IVGTT

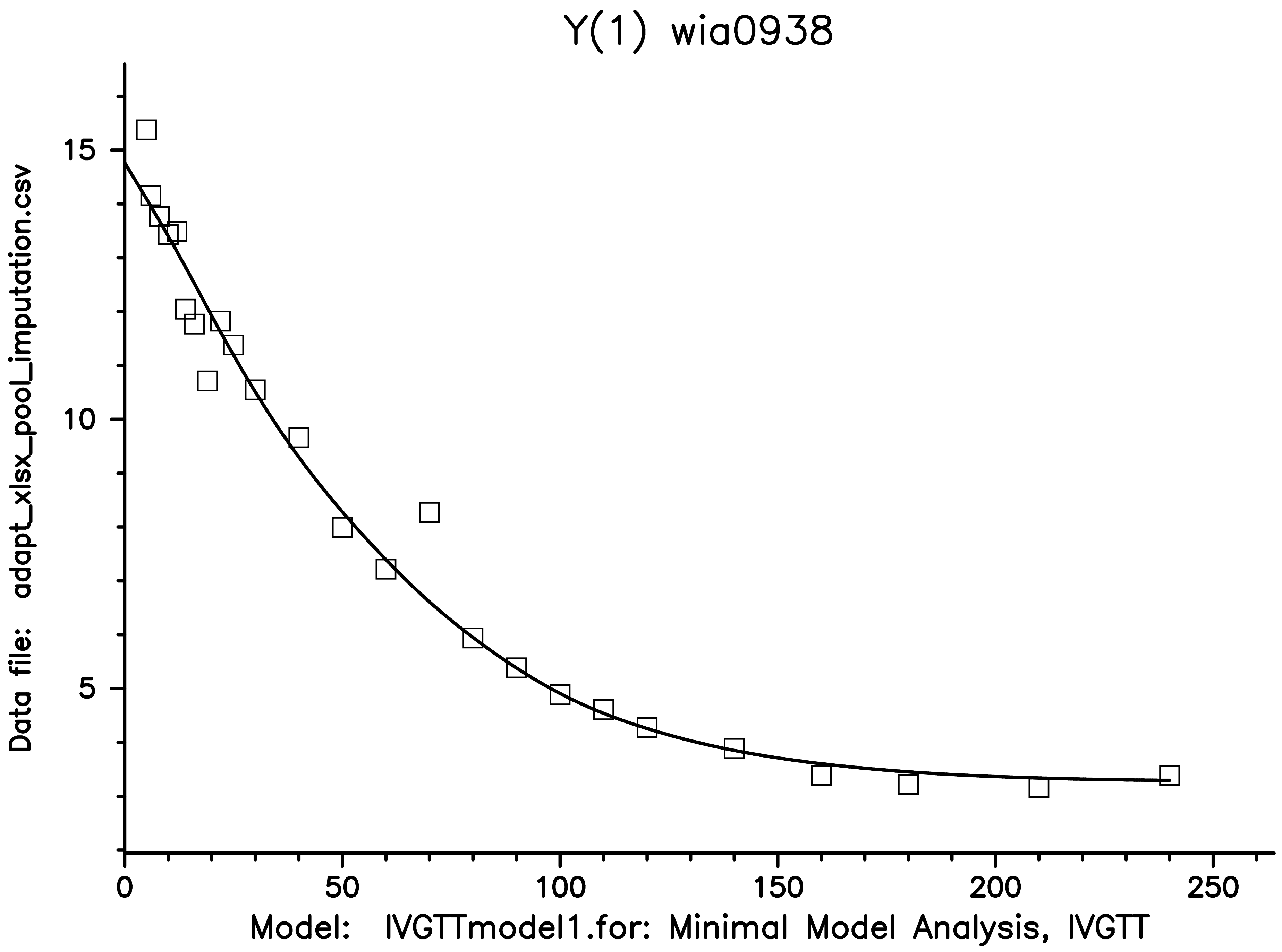
Y(1) wia0938
Data file: adapt_xlsx_pool_imputation.csv
15
10
5
0
50
100
150
200
250
Model: IVGTTmodel1.for: Minimal Model Analysis, IVGTT

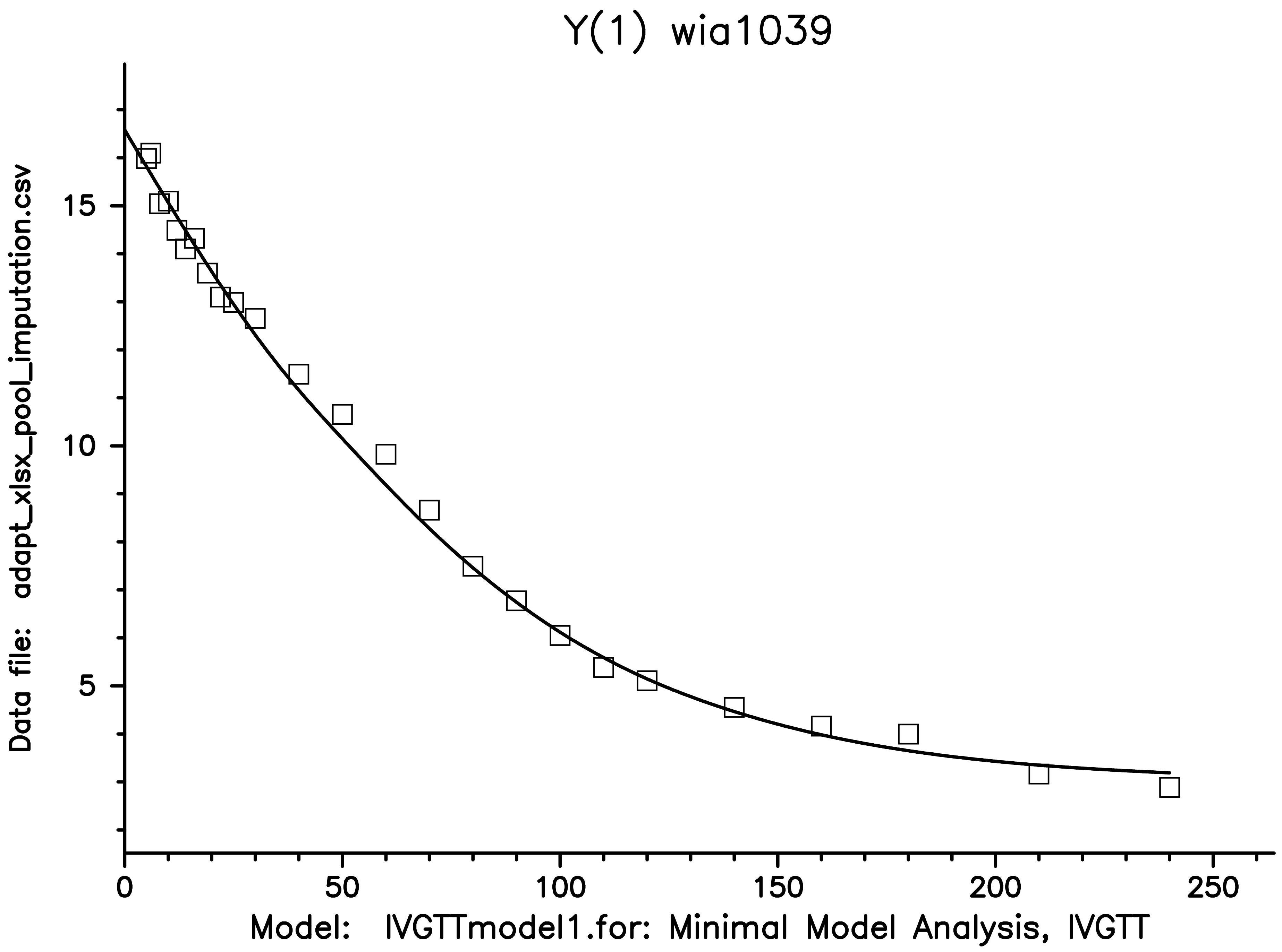
Y(1) wia1039
Data file: adapt_xlsx_pool_imputation.csv
15
10
5
0
50
100
150
200
250
Model: IVGTTmodel1.for: Minimal Model Analysis, IVGTT

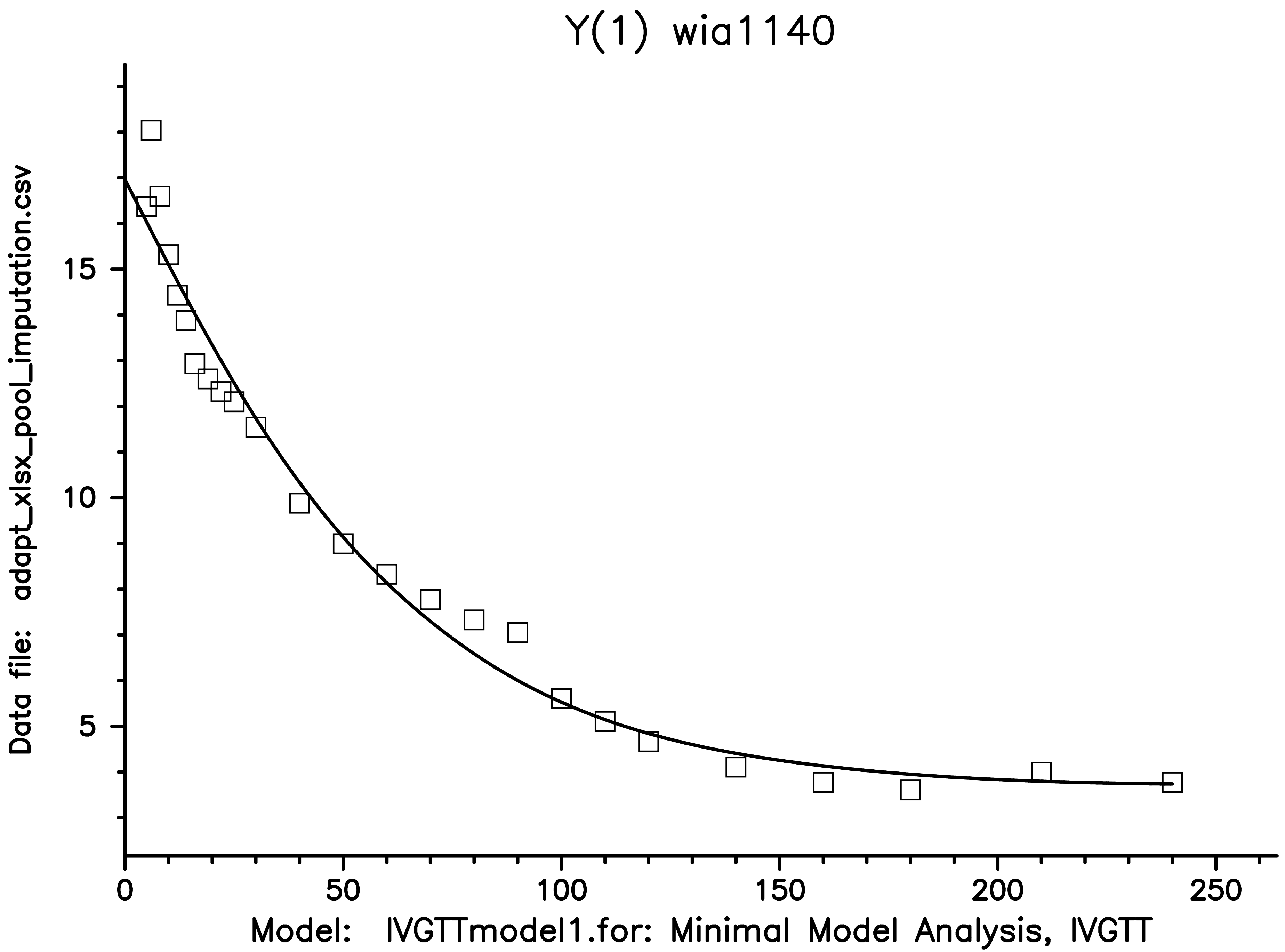
Y(1) wia1140
Data file: adapt_xlsx_pool_imputation.csv
15
10
5
0
50
100
150
200
250
Model: IVGTTmodel1.for: Minimal Model Analysis, IVGTT

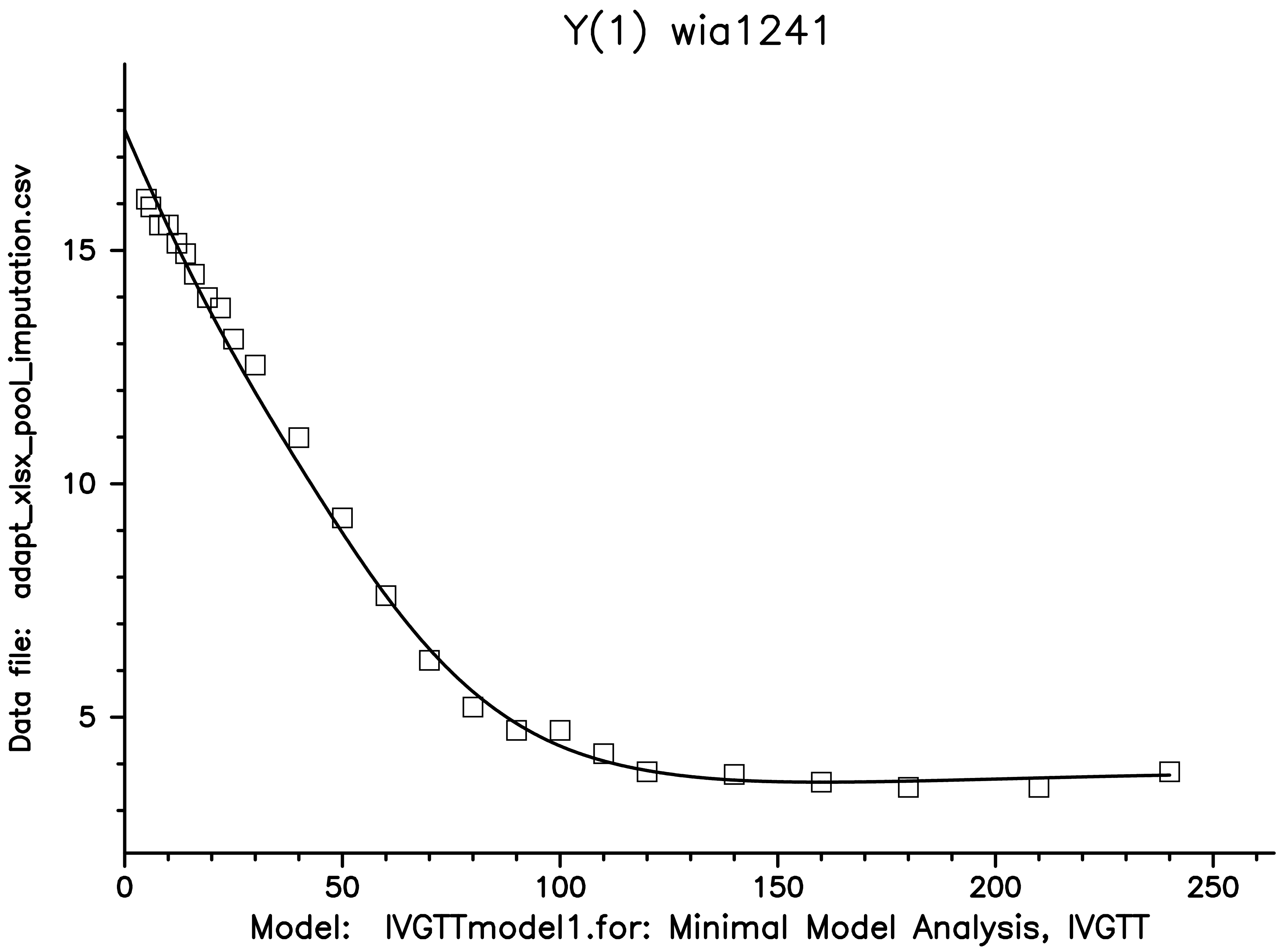
Y(1) wia1241
Data file: adapt_xlsx_pool_imputation.csv
15
10
5
0
50
100
150
200
250
Model: IVGTTmodel1.for: Minimal Model Analysis, IVGTT

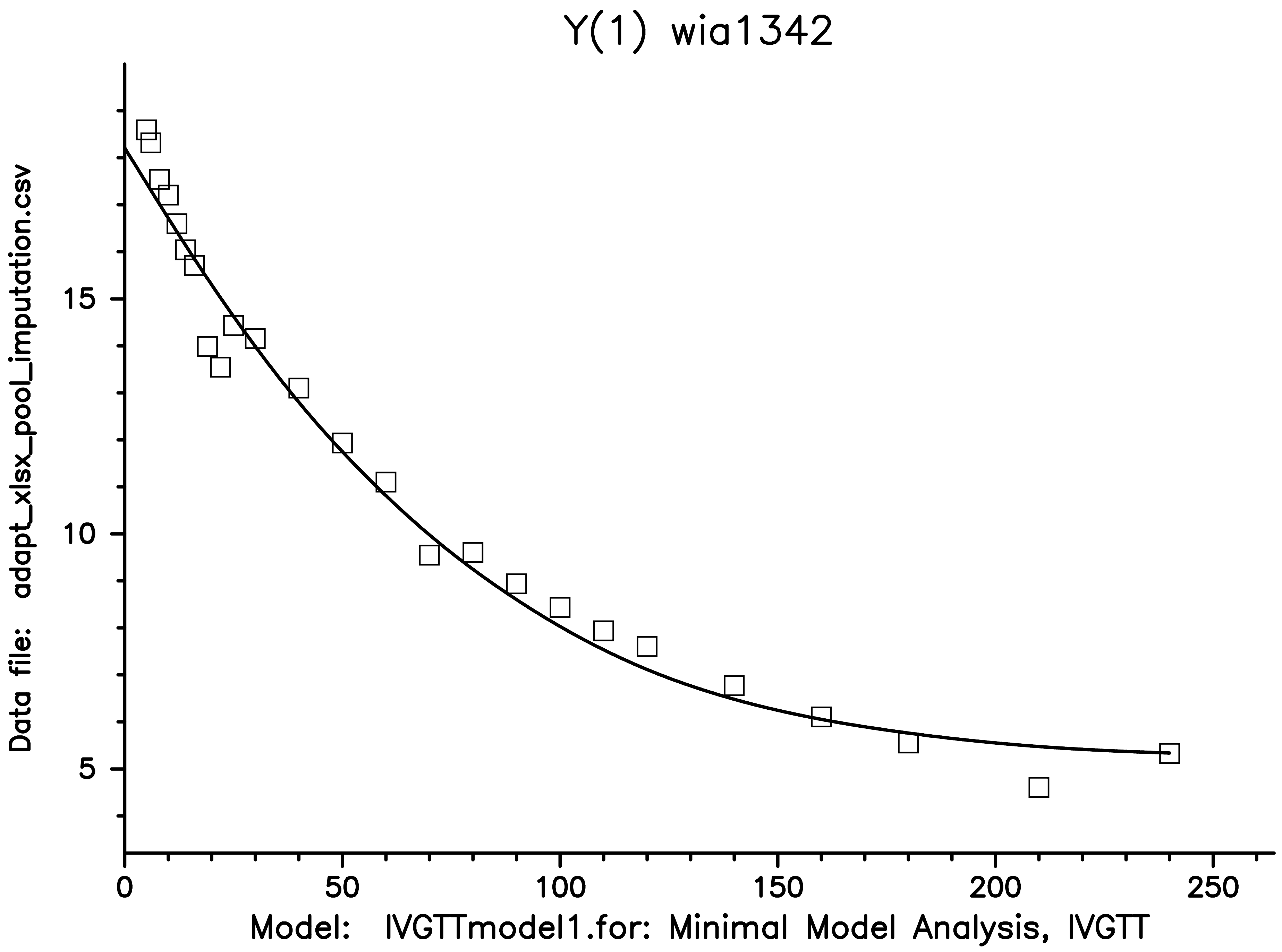
Y(1) wia1342
Data file: adapt_xlsx_pool_imputation.csv
15
10
5
0
50
100
150
200
250
Model: IVGTTmodel1.for: Minimal Model Analysis, IVGTT

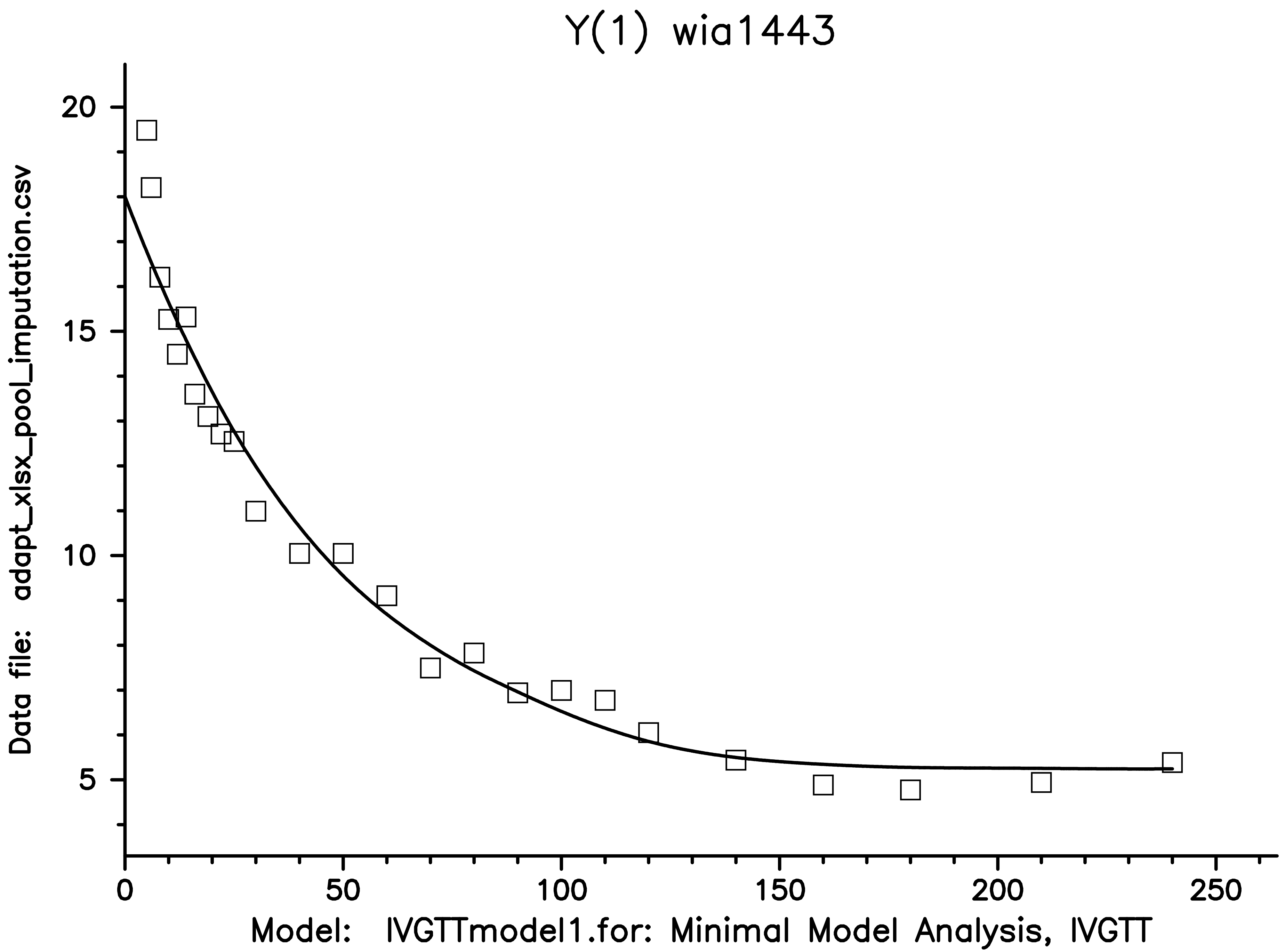

Y(1) wia1443
Data file: adapt_xlsx_pool_imputation.csv
20
15
10
5
0
50
100
150
200
250
Model: IVGTTmodel1.for: Minimal Model Analysis, IVGTT

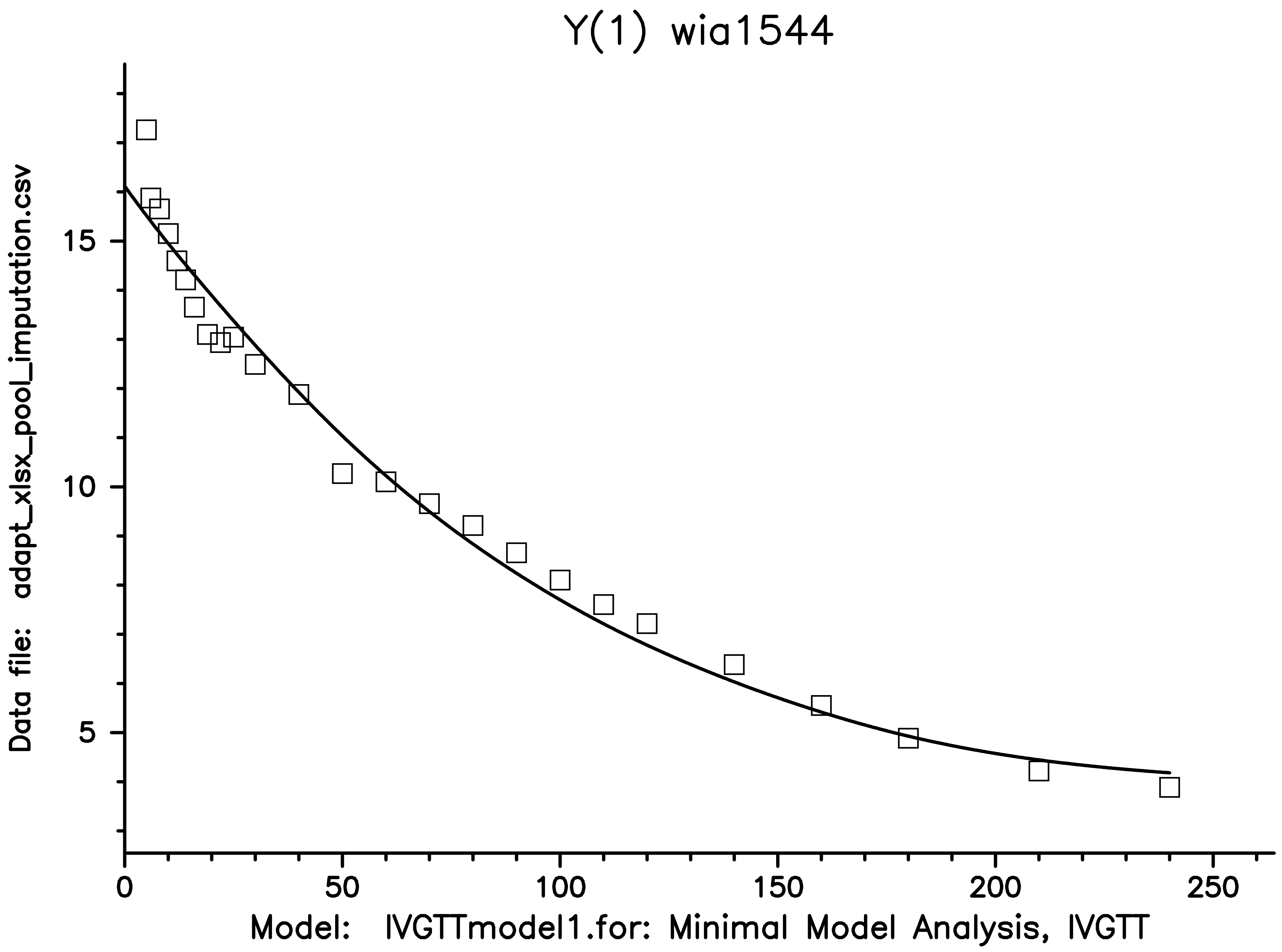
Y(1) wia1544
Data file: adapt_xlsx_pool_imputation.csv
15
10
5
0
50
100
150
200
250
Model: IVGTTmodel1.for: Minimal Model Analysis, IVGTT

Y(1) wia1645

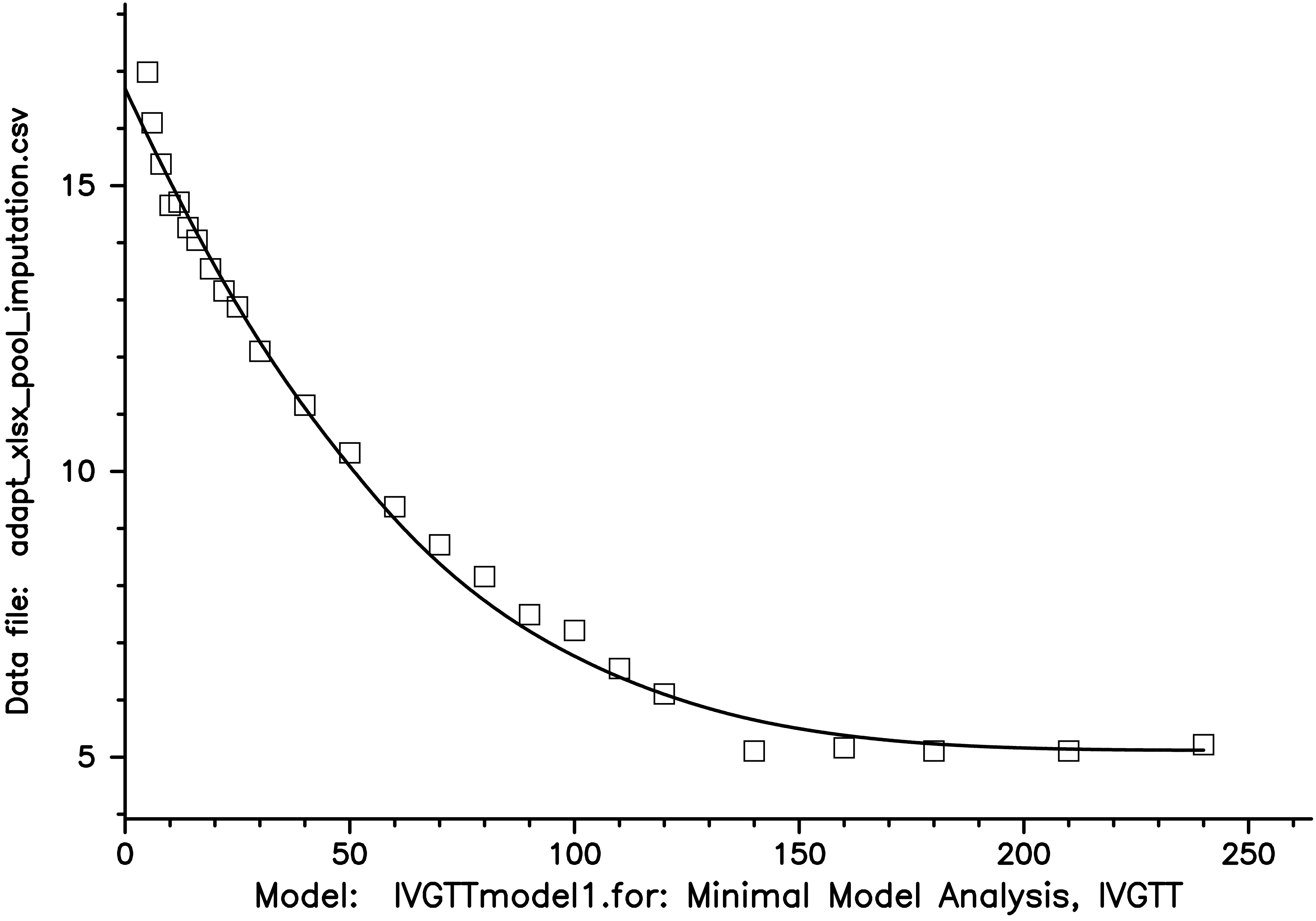

Data file: adapt_xlsx_pool_imputation.csv
15
10
5
0
50
100
150
200
250
Model: IVGTTmodel1.for: Minimal Model Analysis, IVGTT

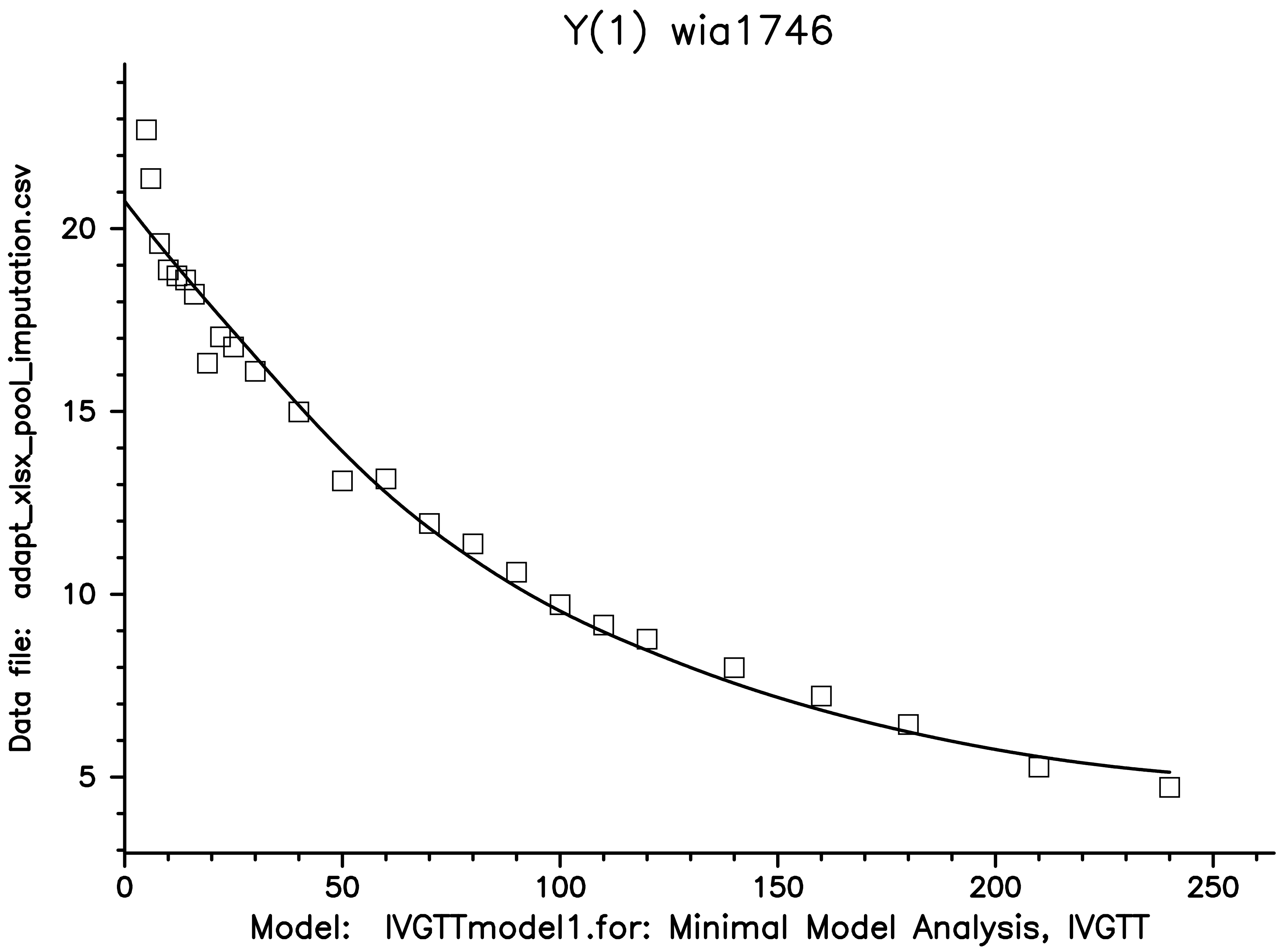

Y(1) wia1746
Data file: adapt_xlsx_pool_imputation.csv
Model: IVGTTmodel1.for: Minimal Model Analysis, IVGTT
20
15
10
5
0
50
100
150
200
250

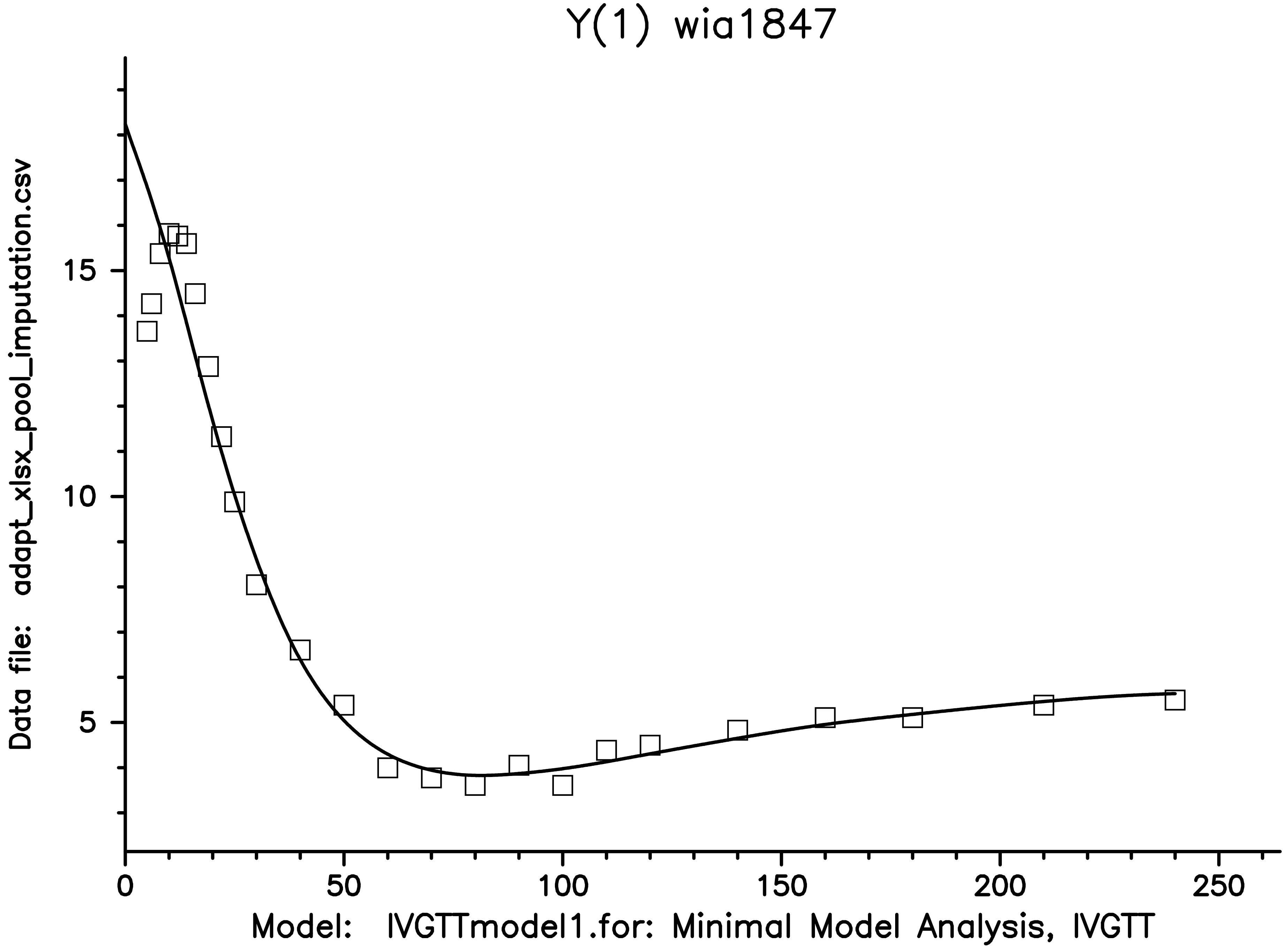

Y(1) wia1847
Data file: adapt_xlsx_pool_imputation.csv
15
10
5
0
50
100
150
200
250
Model: IVGTTmodel1.for: Minimal Model Analysis, IVGTT

Y(1) wia1948

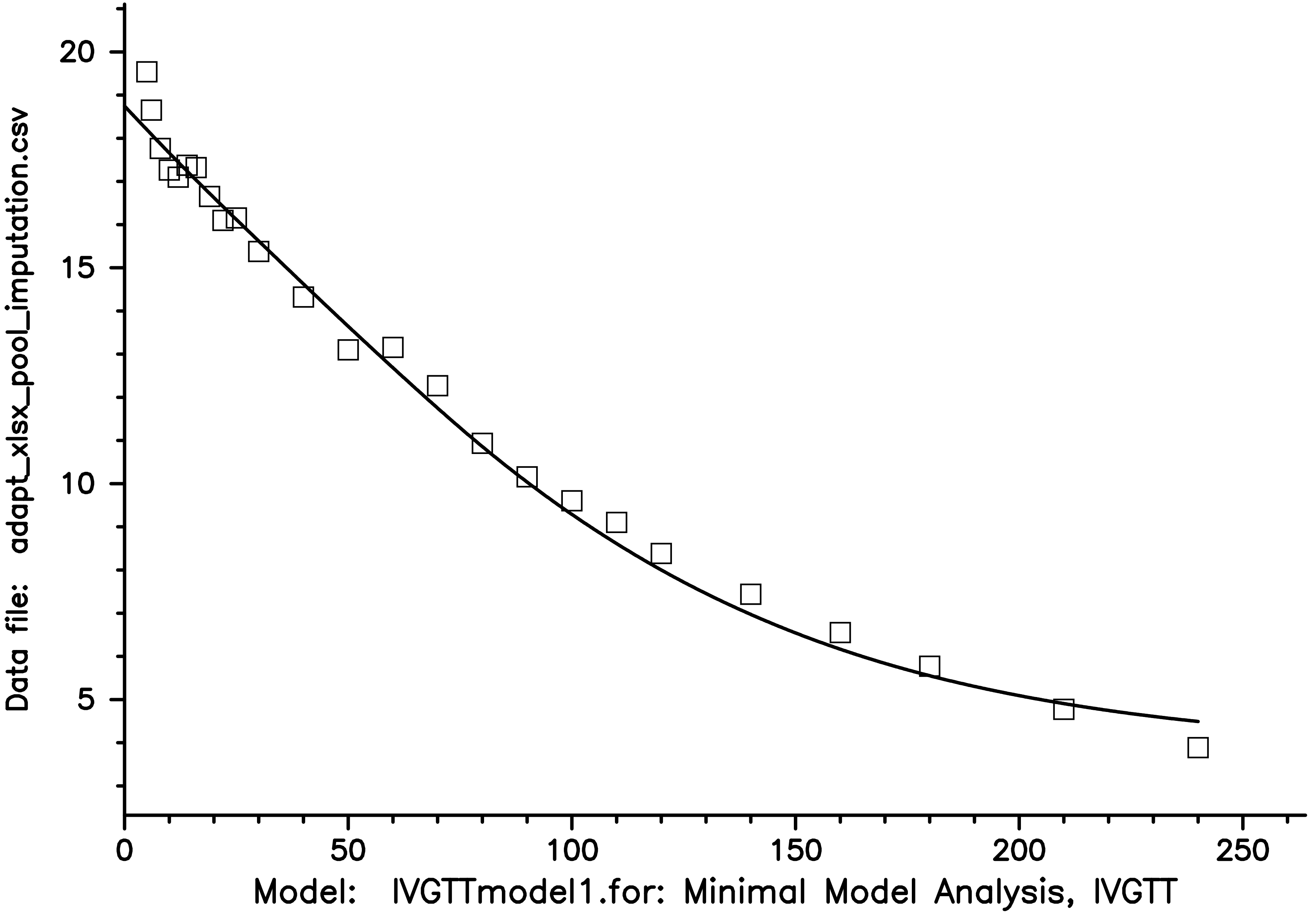

Data file: adapt_xlsx_pool_imputation.csv
20
15
10
5
0
50
100
150
200
250
Model: IVGTTmodel1.for: Minimal Model Analysis, IVGTT

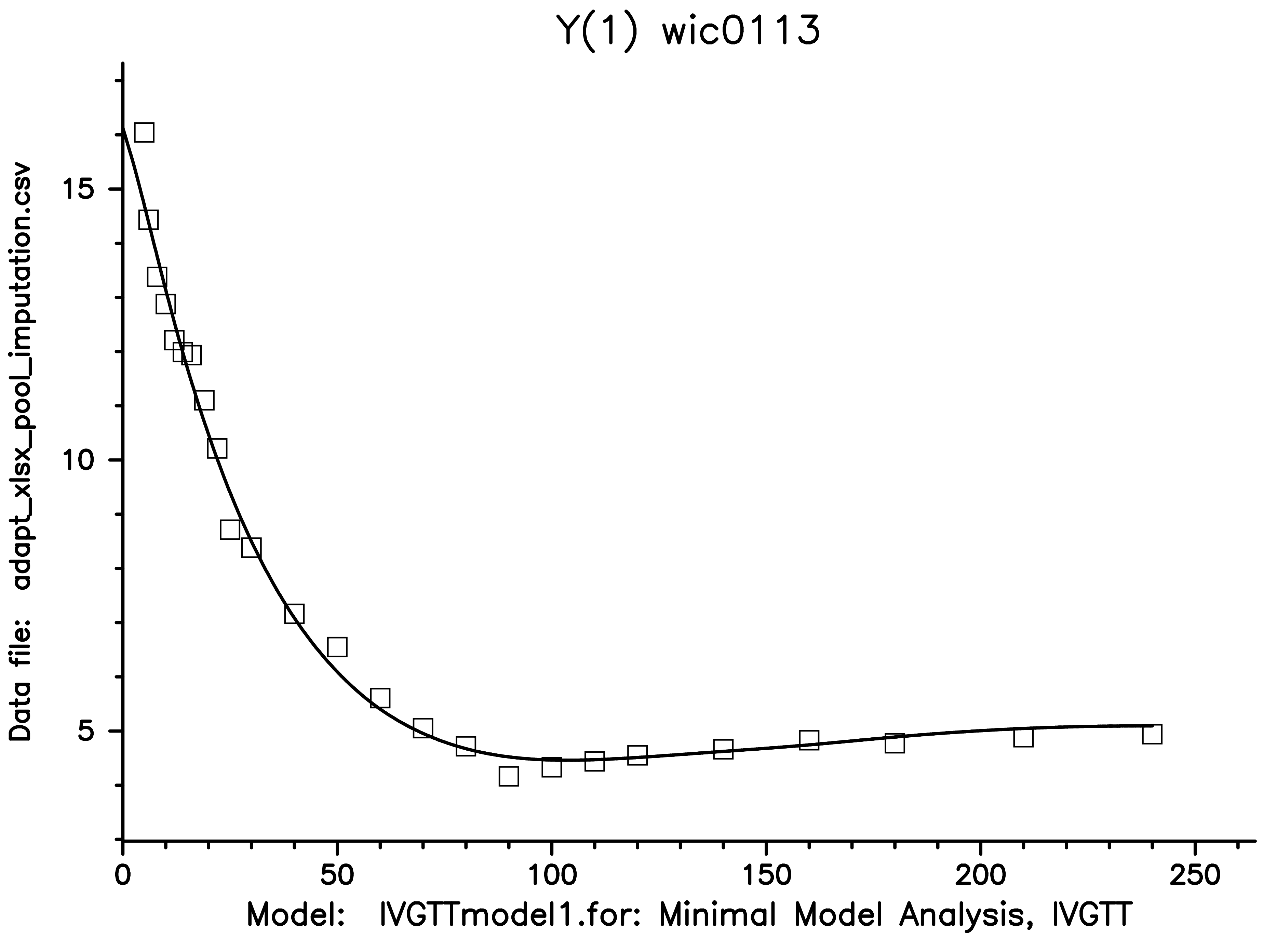
Y(1) wic0113
Data file: adapt_xlsx_pool_imputation.csv
15
10
5
0
50
100
150
200
250
Model: IVGTTmodel1.for: Minimal Model Analysis, IVGTT

Y(1) wic0214

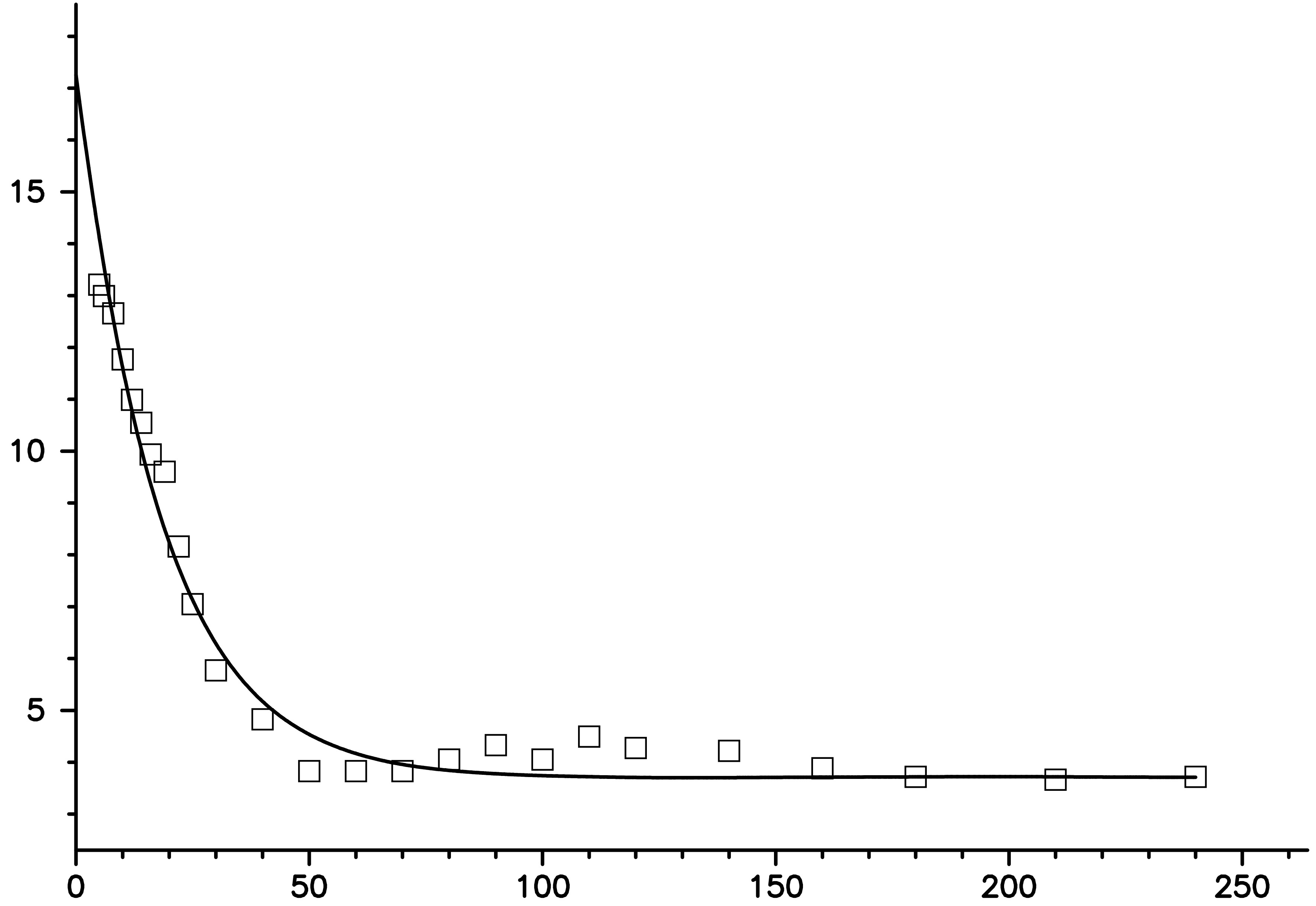

Data file: adapt_xlsx_pool_imputation.csv

Model: IVGTTmodel1.for: Minimal Model Analysis, IVGTT

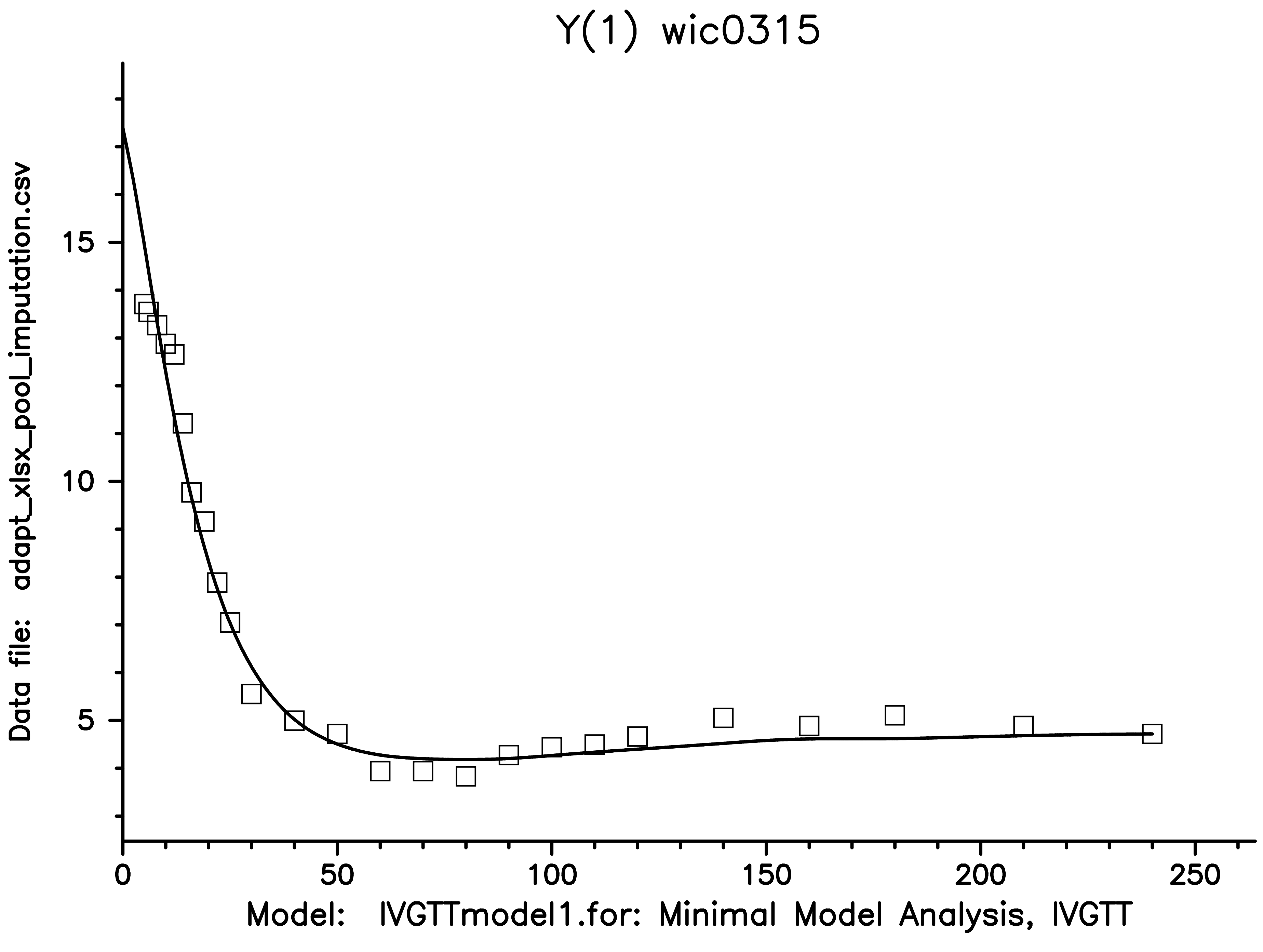

Y(1) wic0315
Data file: adapt_xlsx_pool_imputation.csv
Model: IVGTTmodel1.for: Minimal Model Analysis, IVGTT
15
10
5
0
50
100
150
200
250

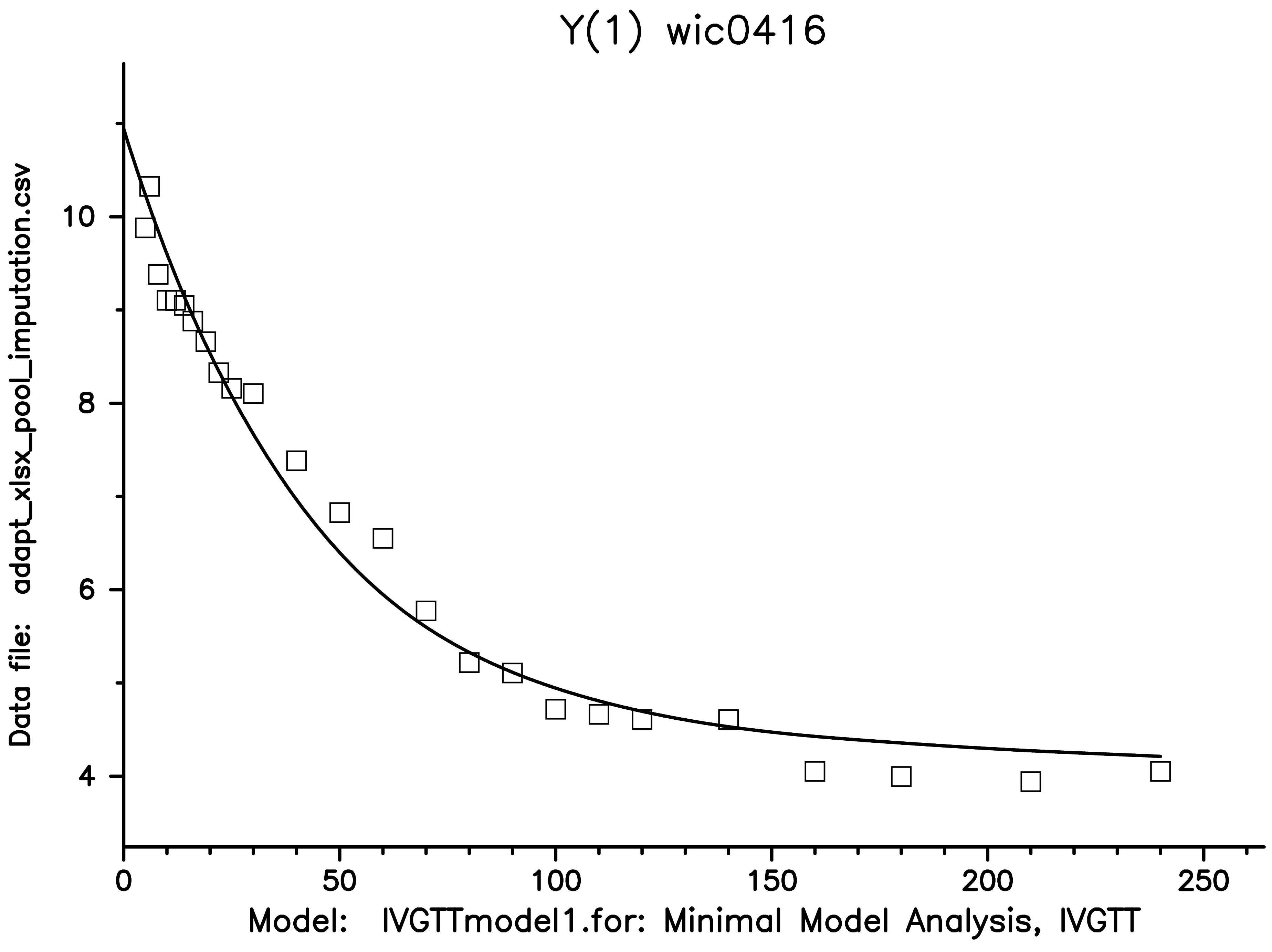
Y(1) wic0416
Data file: adapt_xlsx_pool_imputation.csv
10
8
6
4
0
50
100
150
200
250
Model: IVGTTmodel1.for: Minimal Model Analysis, IVGTT

Y(1) wic0517

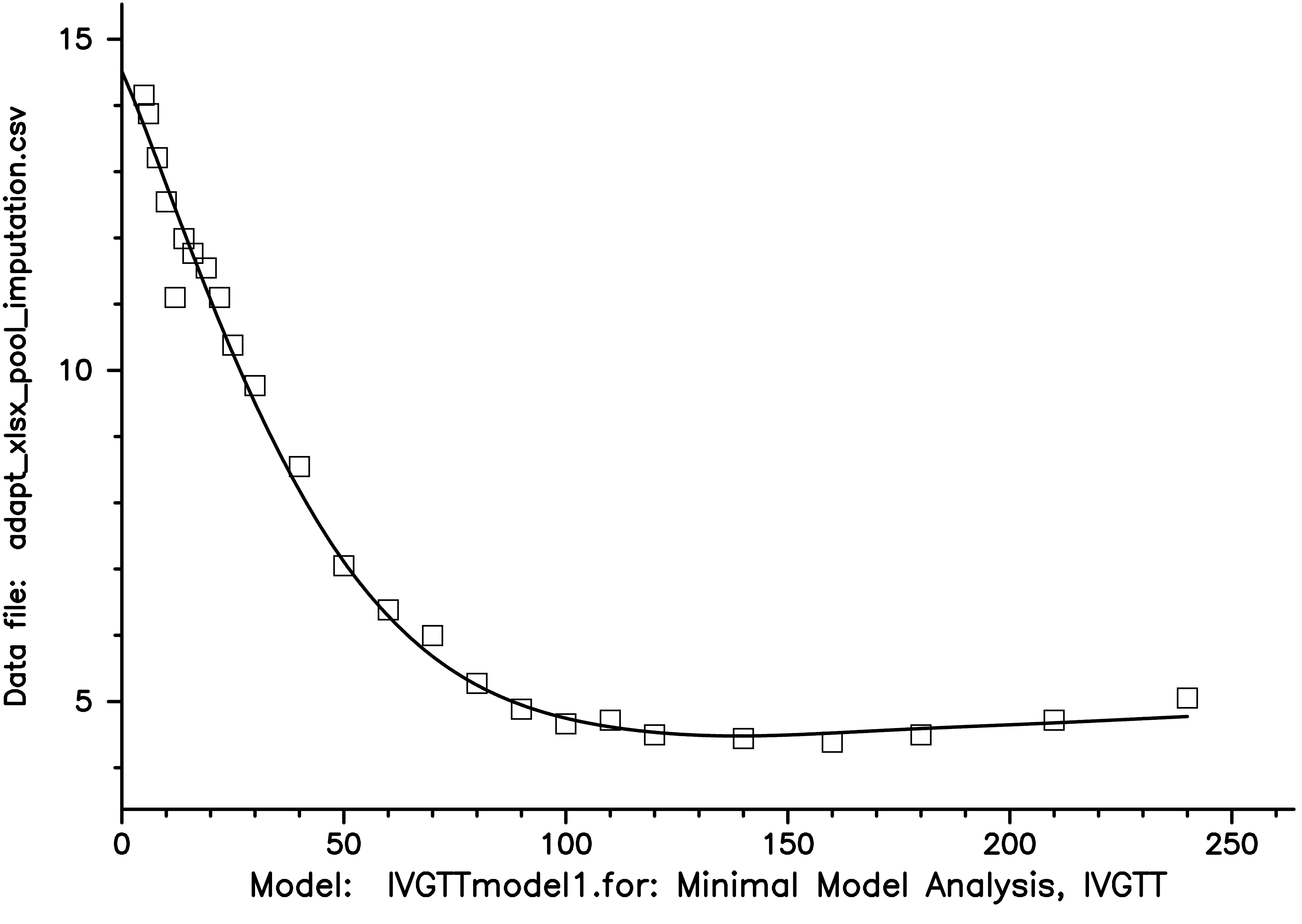

Data file: adapt_xlsx_pool_imputation.csv
15
10
5
0
50
100
150
200
250
Model: IVGTTmodel1.for: Minimal Model Analysis, IVGTT

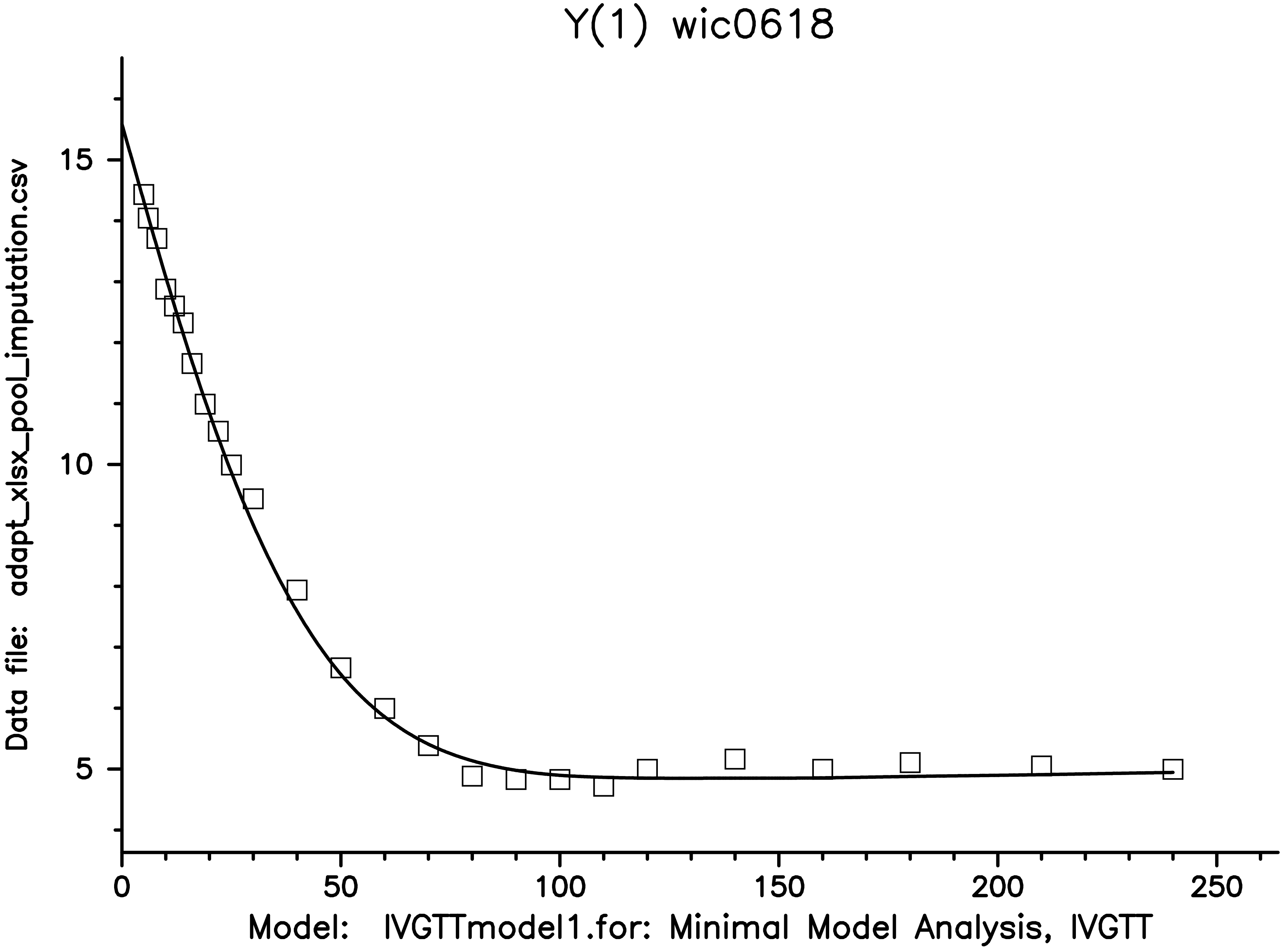
Y(1) wic0618
Data file: adapt_xlsx_pool_imputation.csv
15
10
5
0
50
100
150
200
250
Model: IVGTTmodel1.for: Minimal Model Analysis, IVGTT

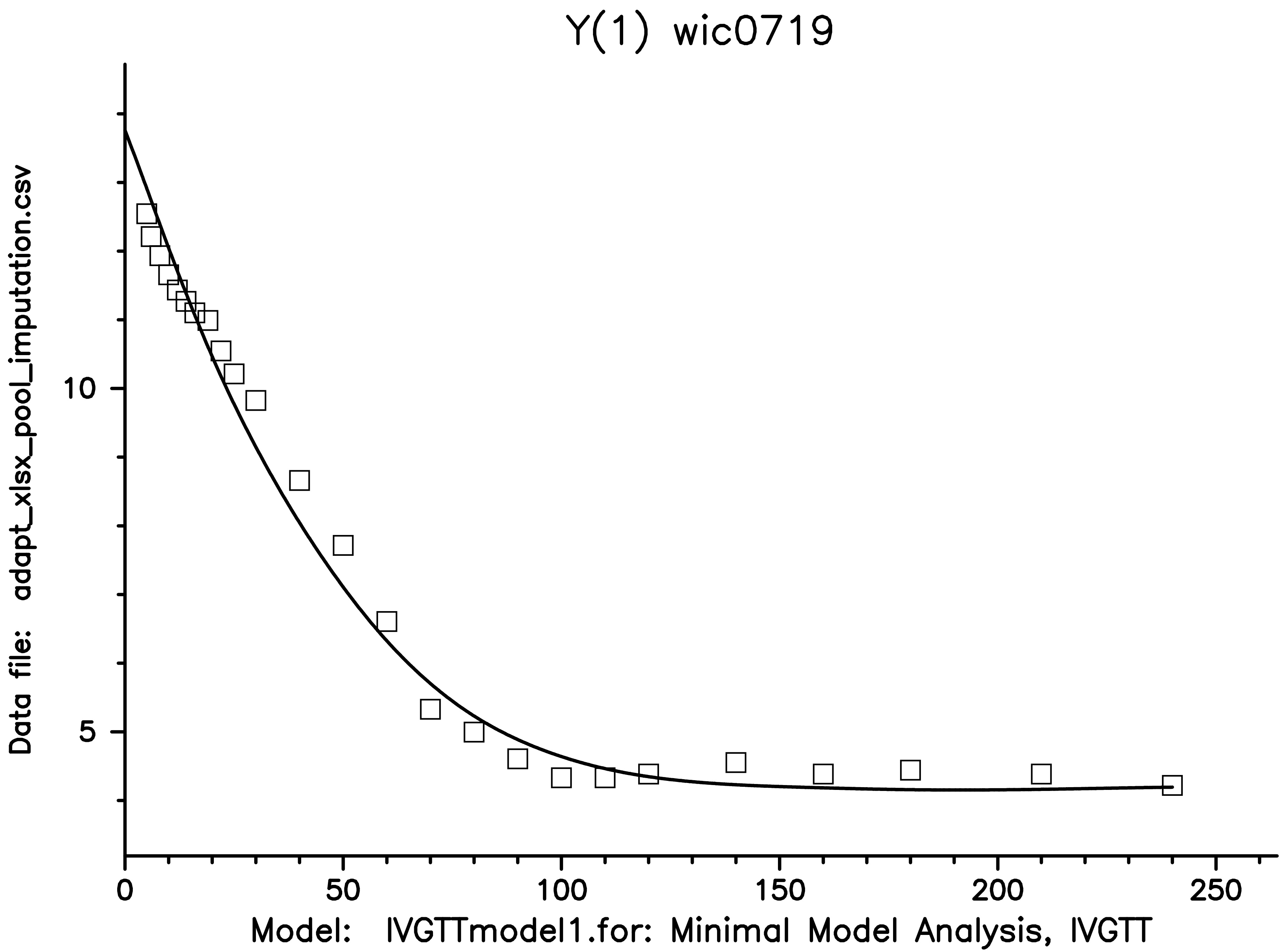

Y(1) wic0719
Data file: adapt_xlsx_pool_imputation.csv
10
5
0
50
100
150
200
250
Model: IVGTTmodel1.for: Minimal Model Analysis, IVGTT

Y(1) wic0820

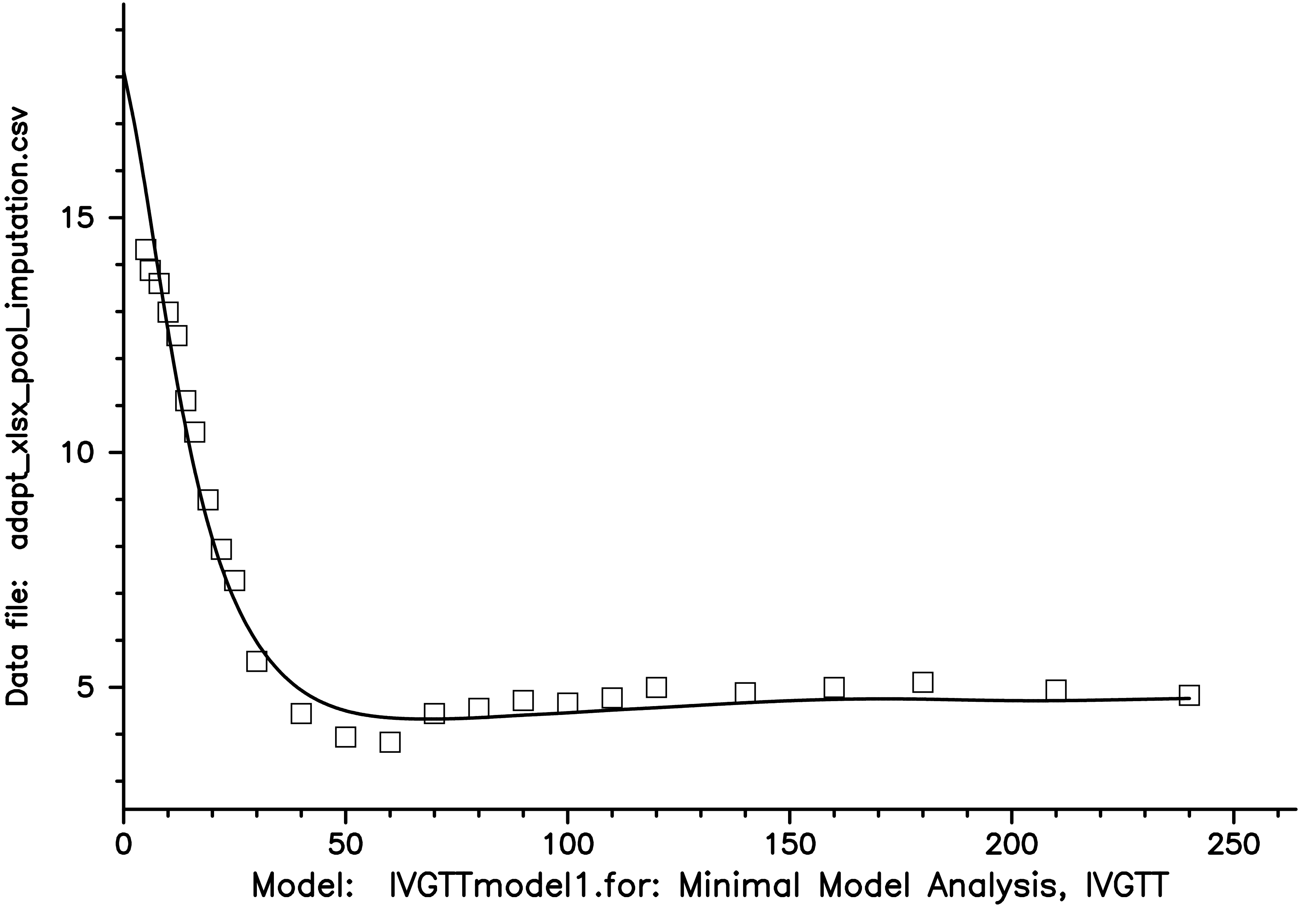

Data file: adapt_xlsx_pool_imputation.csv
15
10
5
0
50
100
150
200
250
Model: IVGTTmodel1.for: Minimal Model Analysis, IVGTT

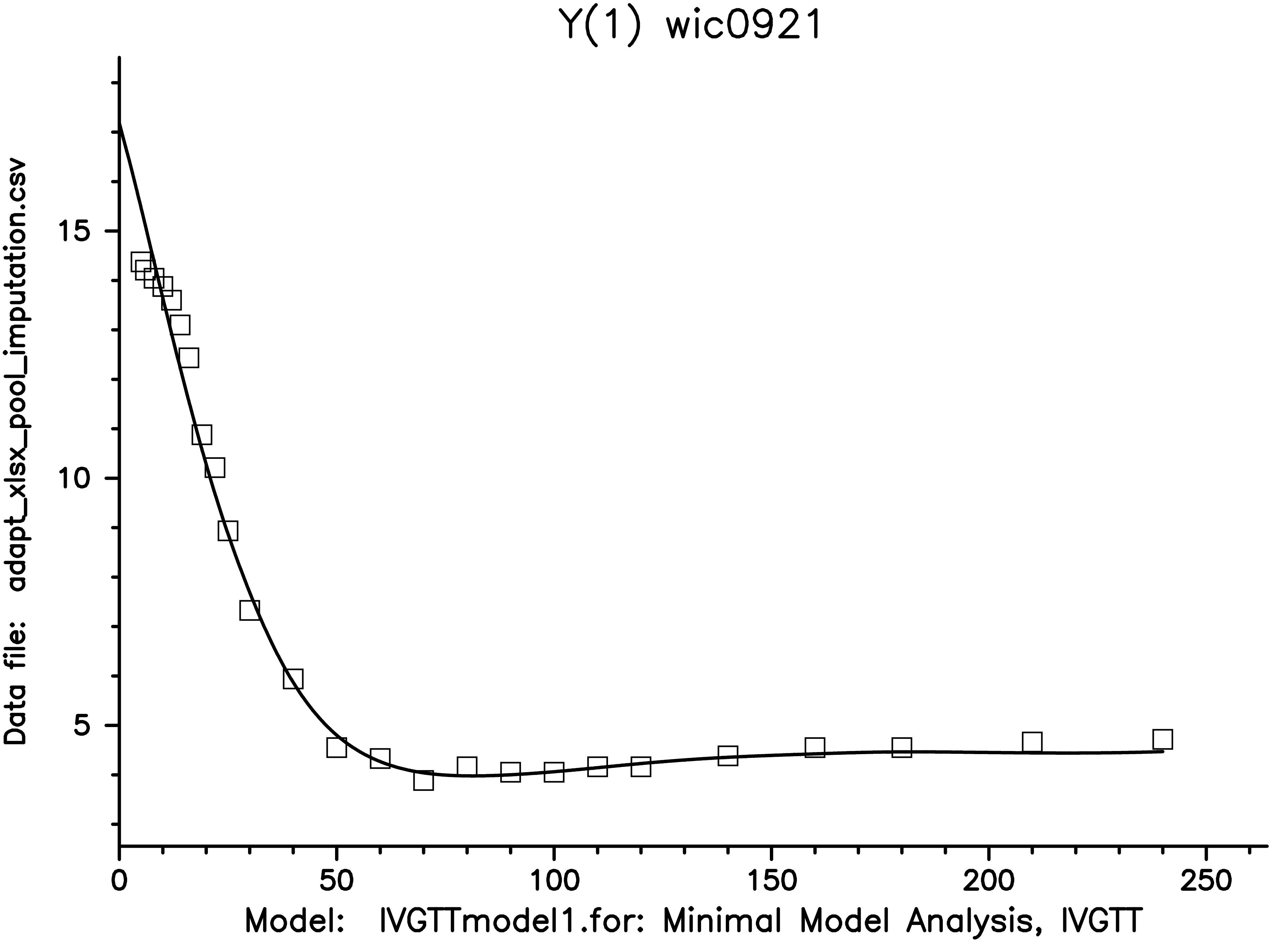
Y(1) wic0921
Data file: adapt_xlsx_pool_imputation.csv
Model: IVGTTmodel1.for: Minimal Model Analysis, IVGTT
15
10
5
0
50
100
150
200
250

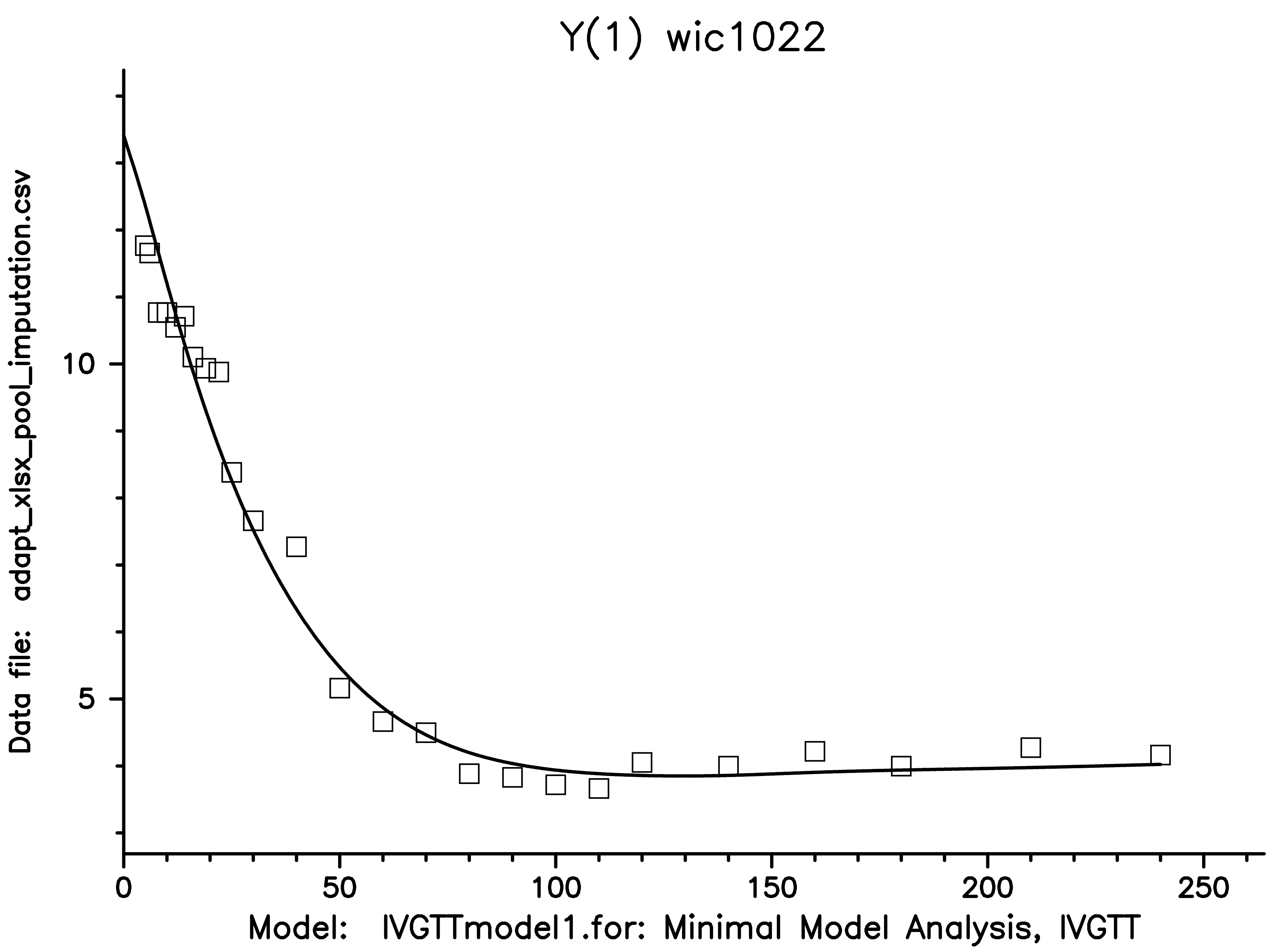

Y(1) wic1022
Data file: adapt_xlsx_pool_imputation.csv
10
5
0
50
100
150
200
250
Model: IVGTTmodel1.for: Minimal Model Analysis, IVGTT

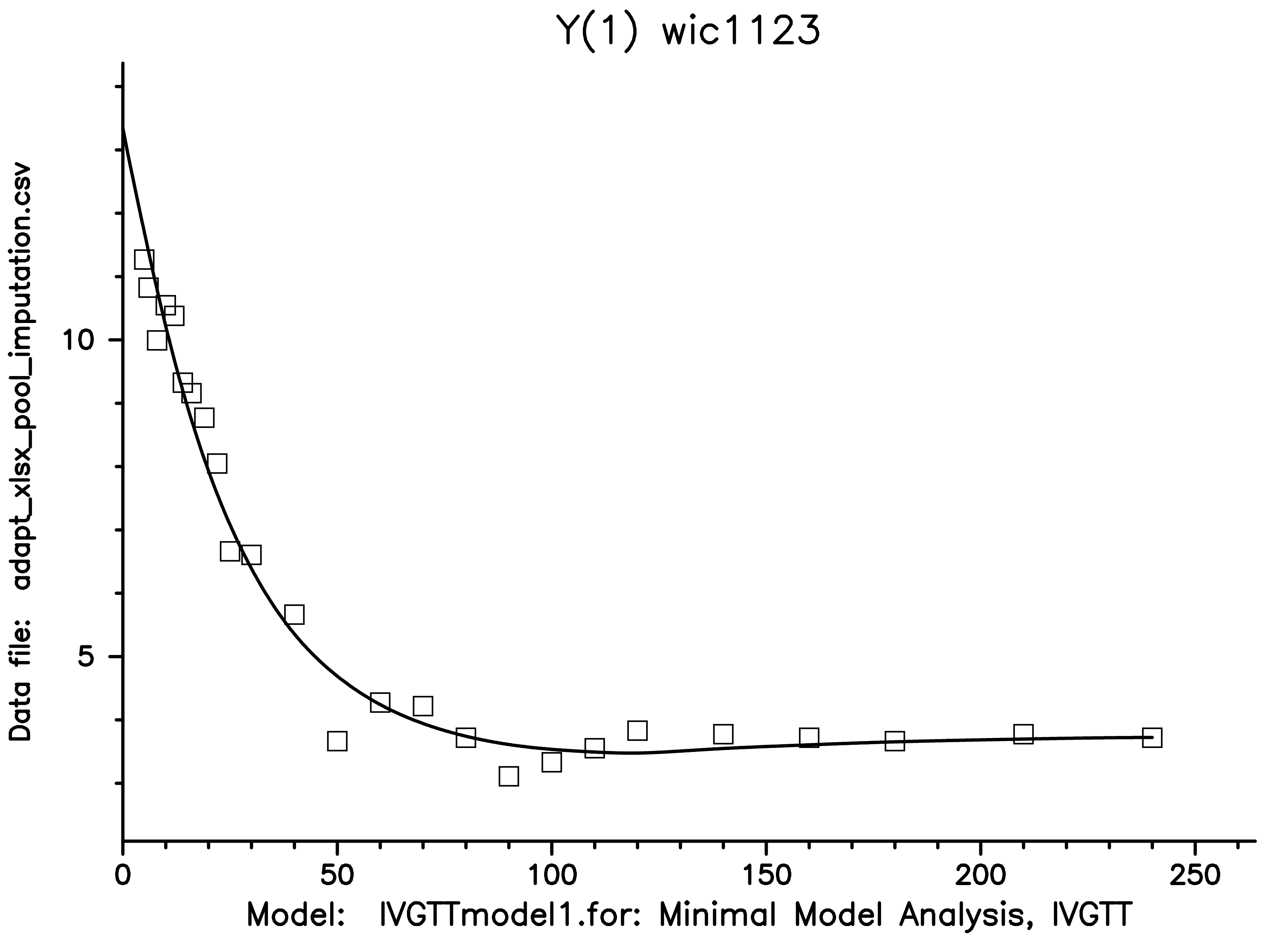
Y(1) wic1123
Data file: adapt_xlsx_pool_imputation.csv
10
5
0
50
100
150
200
250
Model: IVGTTmodel1.for: Minimal Model Analysis, IVGTT

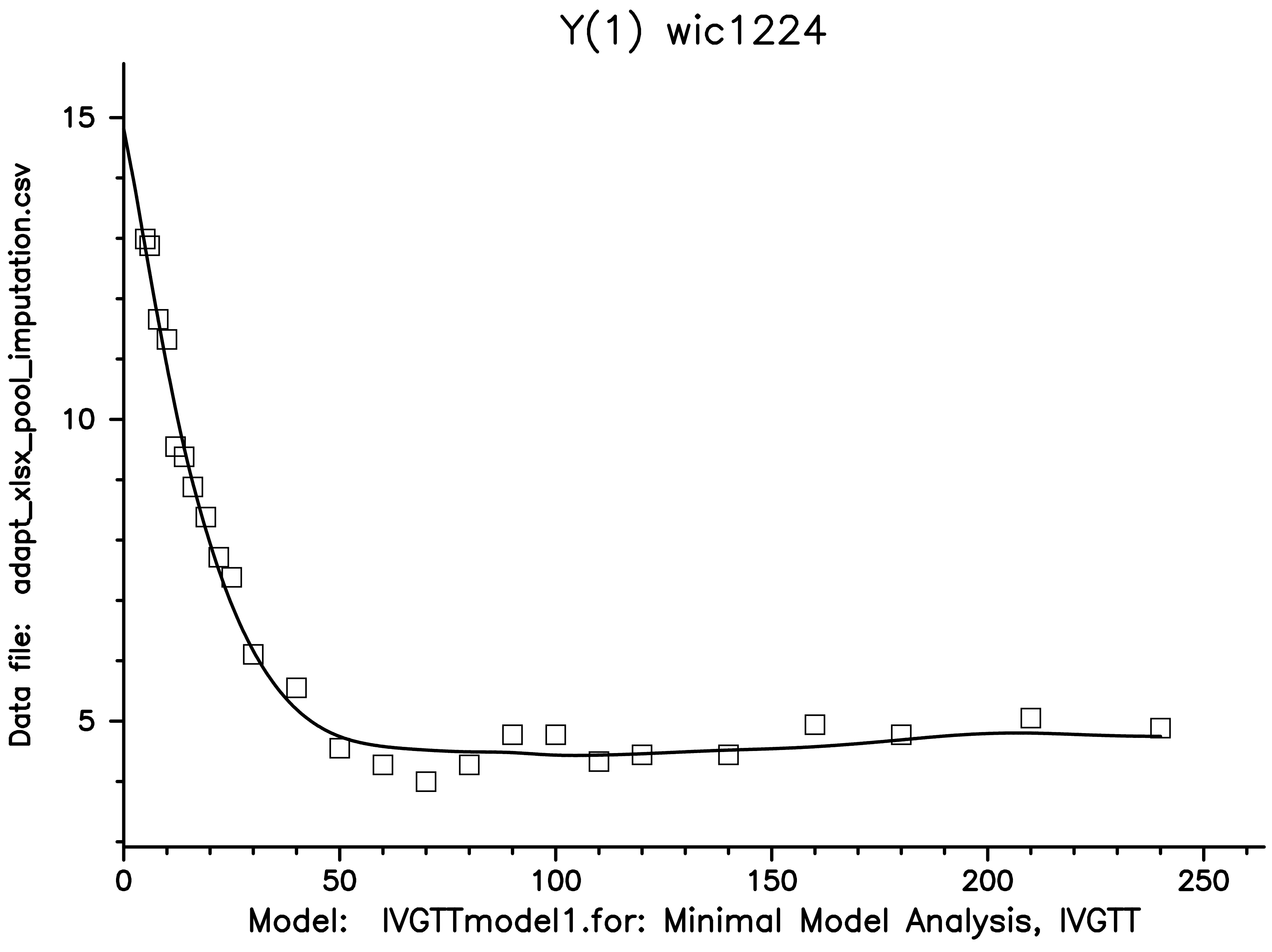

Y(1) wic1224
Data file: adapt_xlsx_pool_imputation.csv
15
10
5
0
50
100
150
200
250
Model: IVGTTmodel1.for: Minimal Model Analysis, IVGTT

Y(1) wic1325

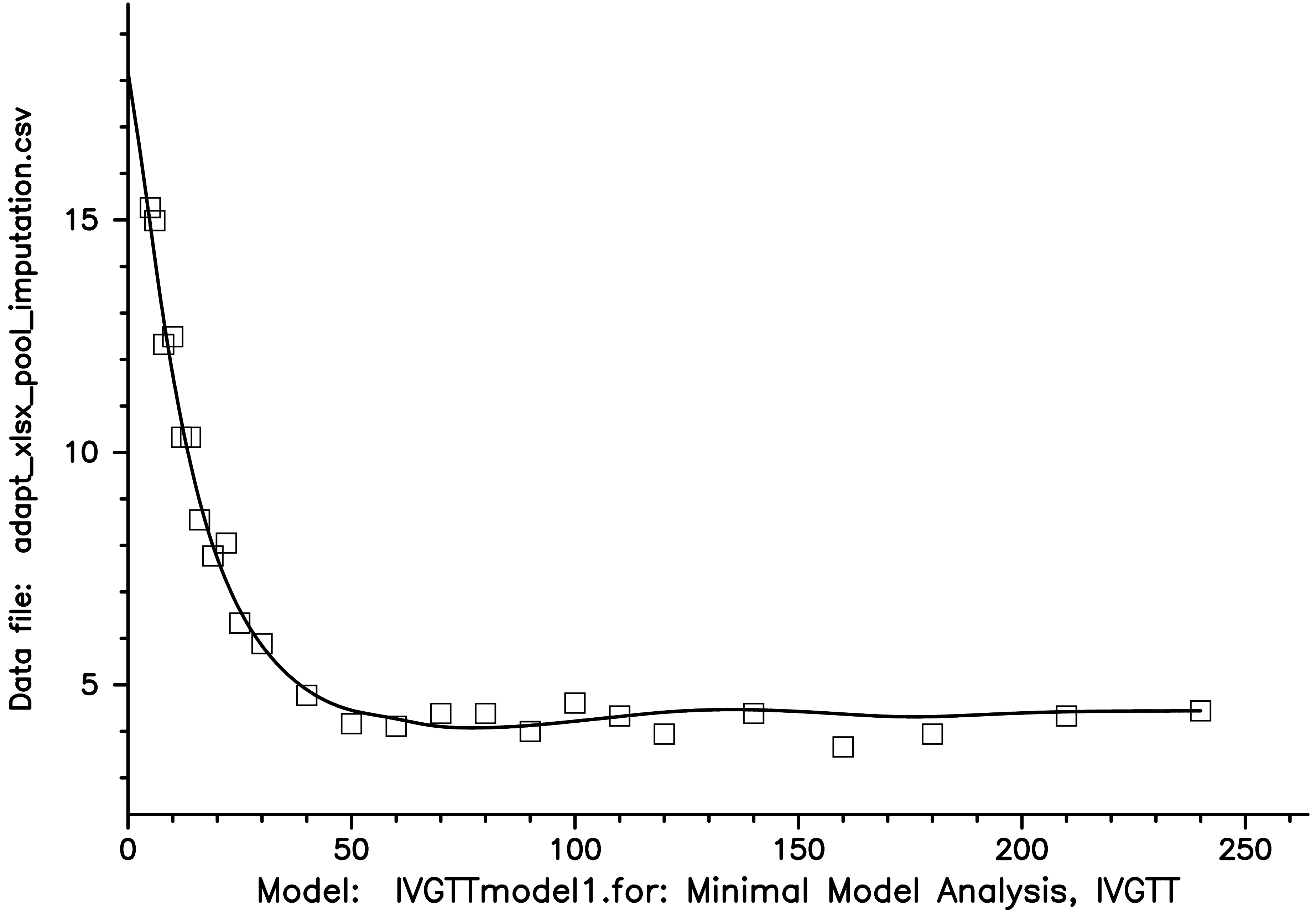

Data file: adapt_xlsx_pool_imputation.csv
15
10
5
0
50
100
150
200
250
Model: IVGTTmodel1.for: Minimal Model Analysis, IVGTT

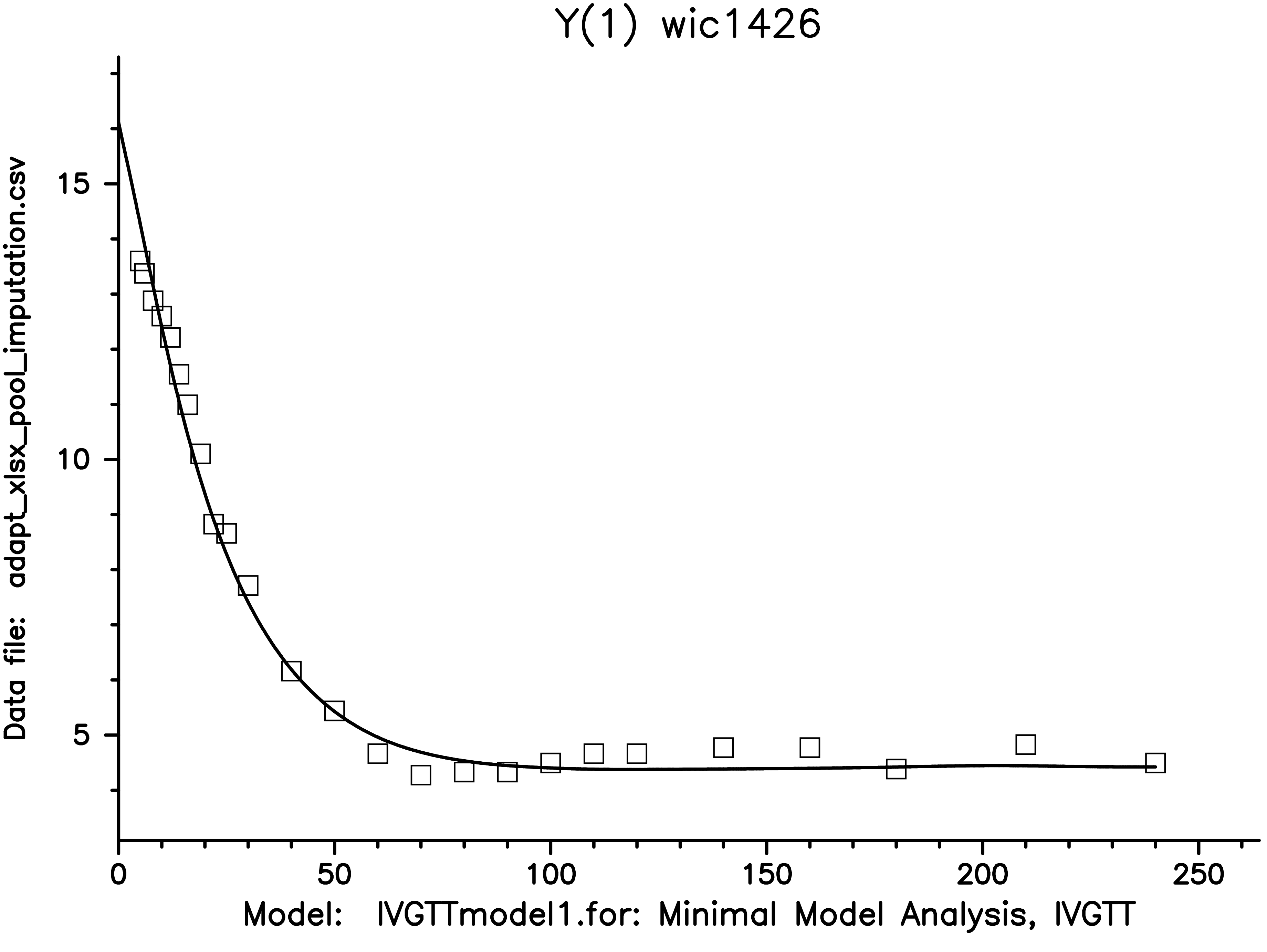
Y(1) wic1426
Data file: adapt_xlsx_pool_imputation.csv
15
10
5
0
50
100
150
200
250
Model: IVGTTmodel1.for: Minimal Model Analysis, IVGTT

Y(1) wic1527

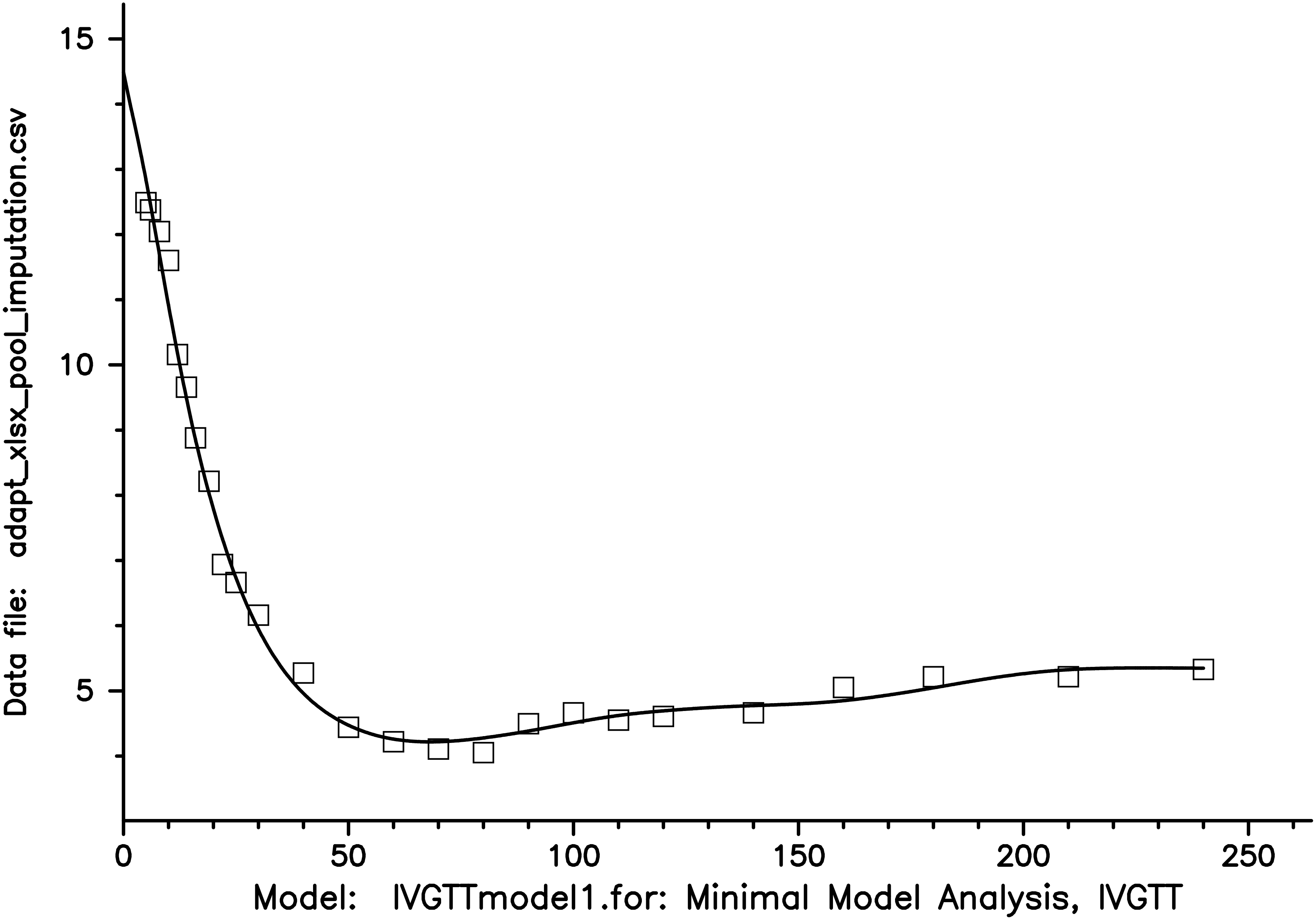

15
10
5
0
50
100
150
200
250
Data file: adapt_xlsx_pool_imputation.csv
Model: IVGTTmodel1.for: Minimal Model Analysis, IVGTT

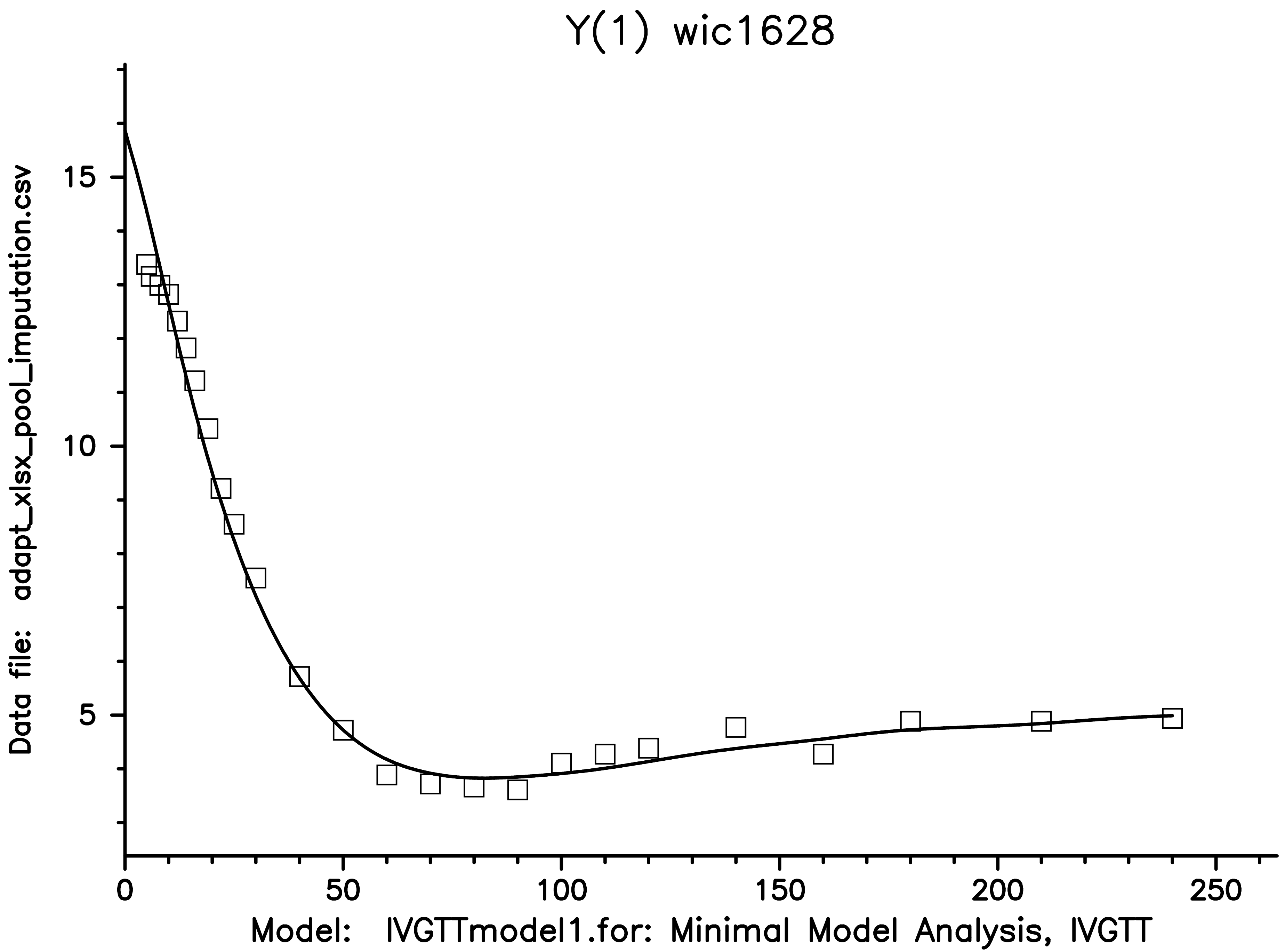
Y(1) wic1628
Data file: adapt_xlsx_pool_imputation.csv
15
10
5
0
50
100
150
200
250
Model: IVGTTmodel1.for: Minimal Model Analysis, IVGTT

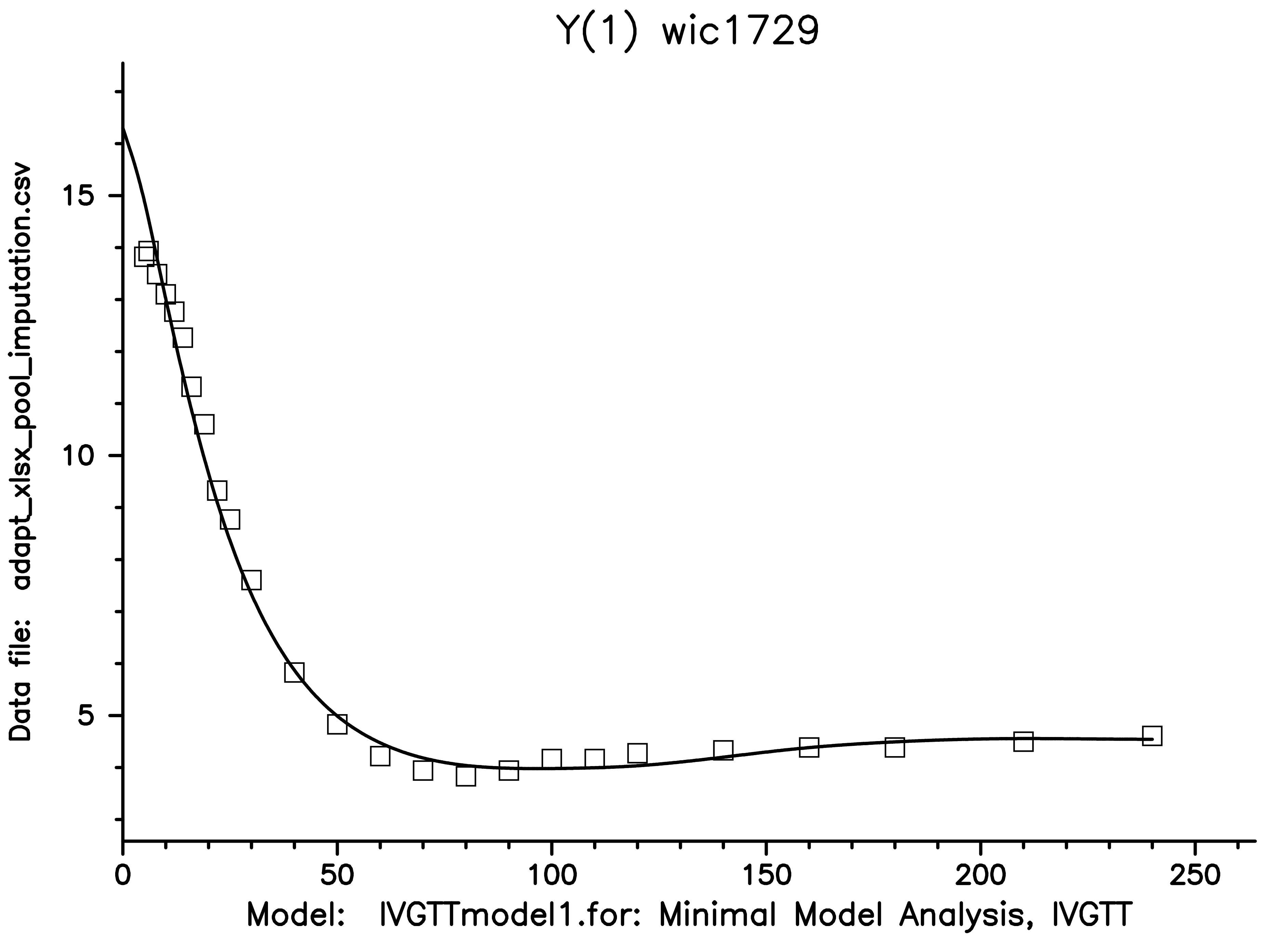

Y(1) wic1729
Data file: adapt_xlsx_pool_imputation.csv
15
10
5
0
50
100
150
200
250
Model: IVGTTmodel1.for: Minimal Model Analysis, IVGTT

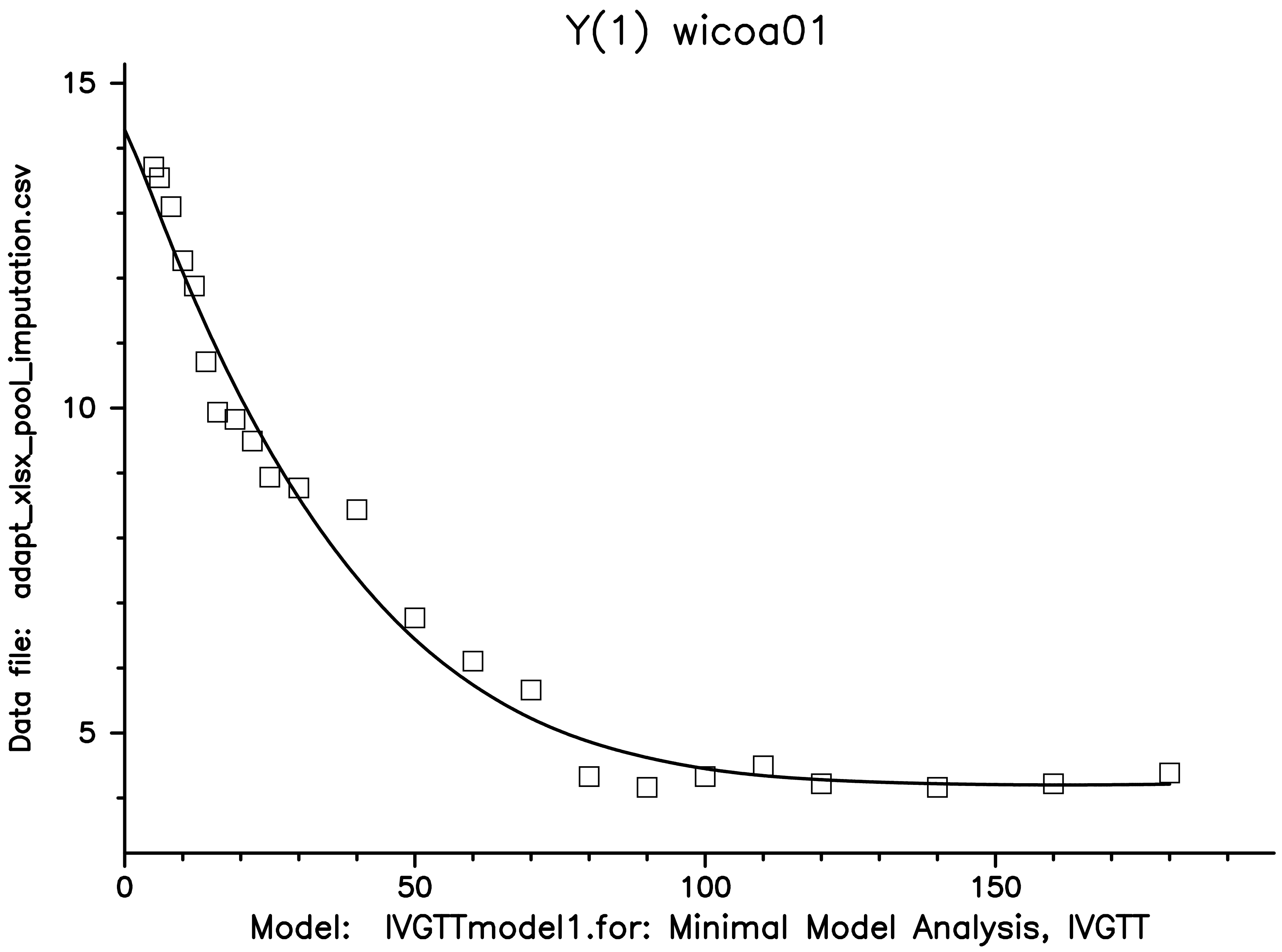
Y(1) wicoa01
15
10
5
0
50
100
150
Data file: adapt_xlsx_pool_imputation.csv
Model: IVGTTmodel1.for: Minimal Model Analysis, IVGTT

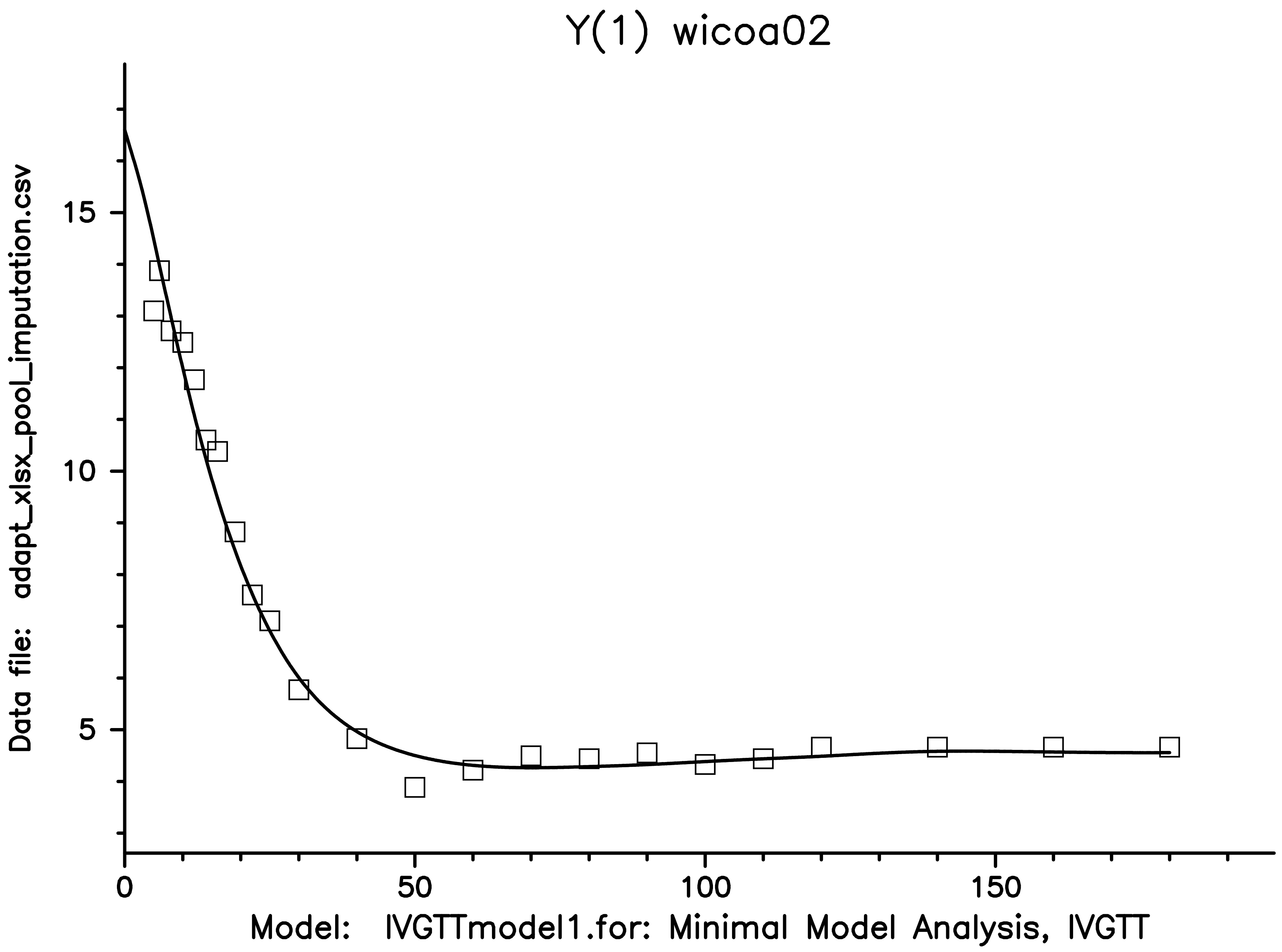
Y(1) wicoa02
Data file: adapt_xlsx_pool_imputation.csv
15
10
5
0
50
100
150
Model: IVGTTmodel1.for: Minimal Model Analysis, IVGTT

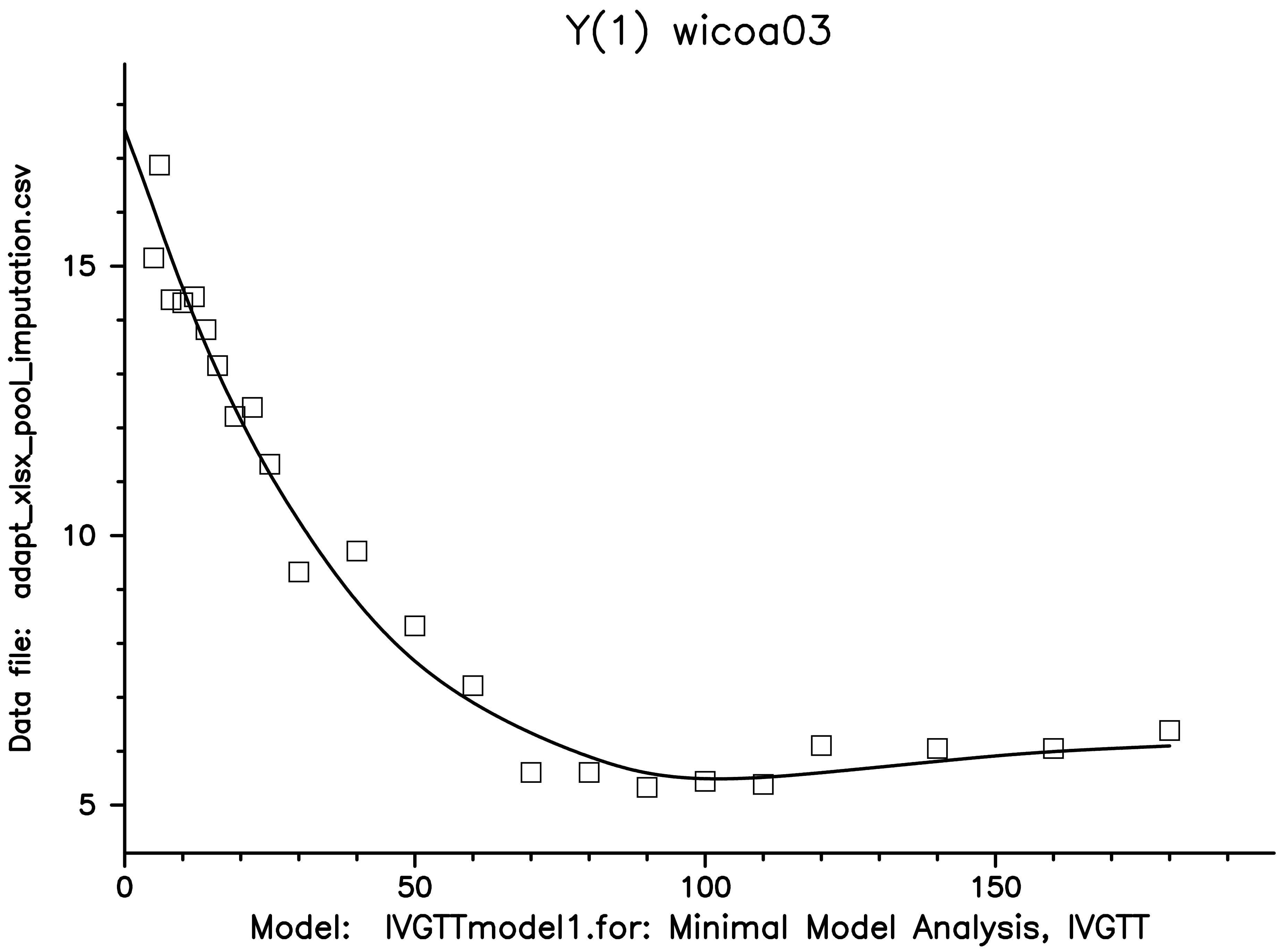
Y(1) wicoa03
Data file: adapt_xlsx_pool_imputation.csv
15
10
5
0
50
100
150
Model: IVGTTmodel1.for: Minimal Model Analysis, IVGTT

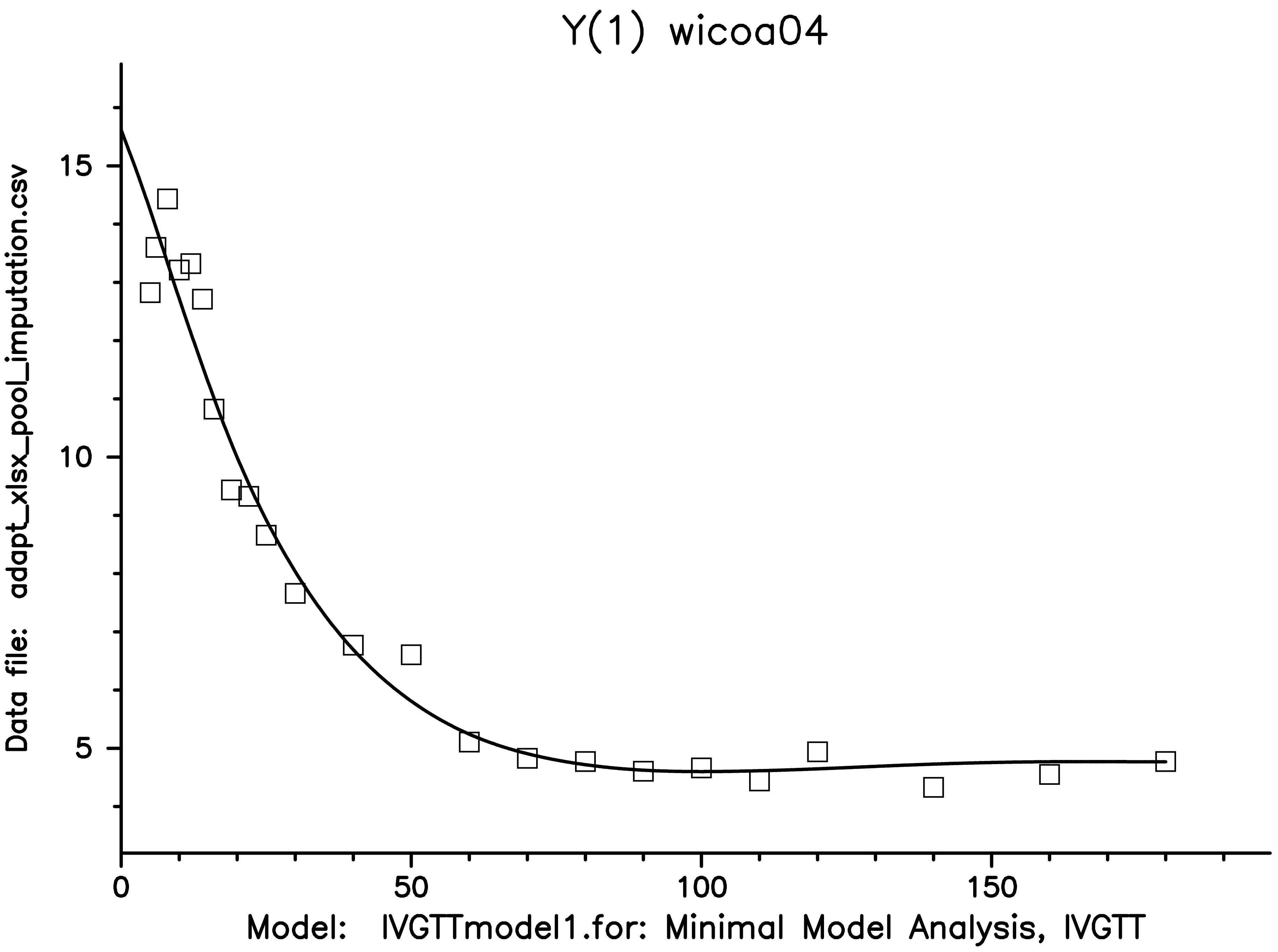

Y(1) wicoa04
Data file: adapt_xlsx_pool_imputation.csv
Model: IVGTTmodel1.for: Minimal Model Analysis, IVGTT
15
10
5
0
50
100
150

Y(1) wicoa05

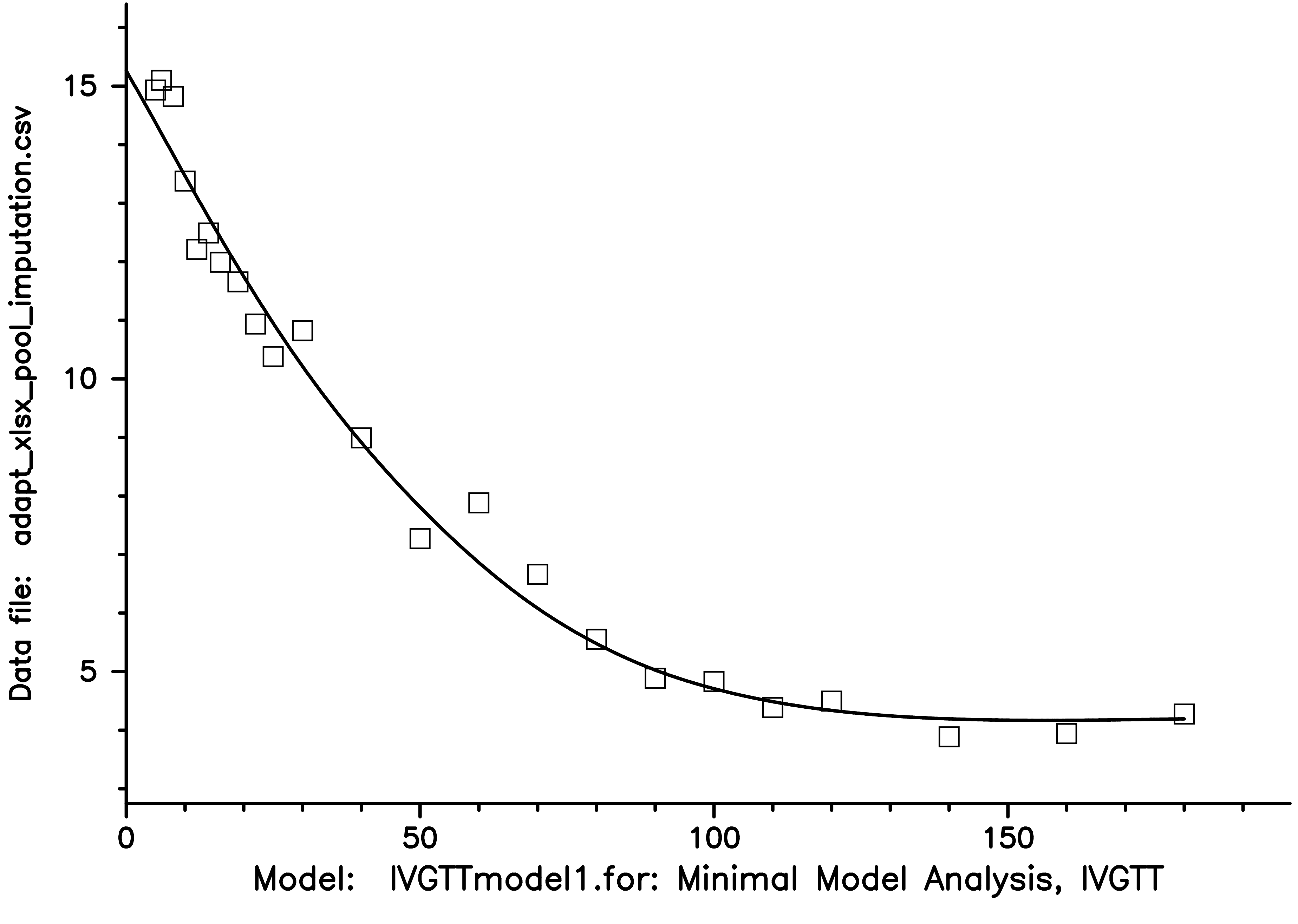
15
10
5
0
50
100
150
Data file: adapt_xlsx_pool_imputation.csv
Model: IVGTTmodel1.for: Minimal Model Analysis, IVGTT

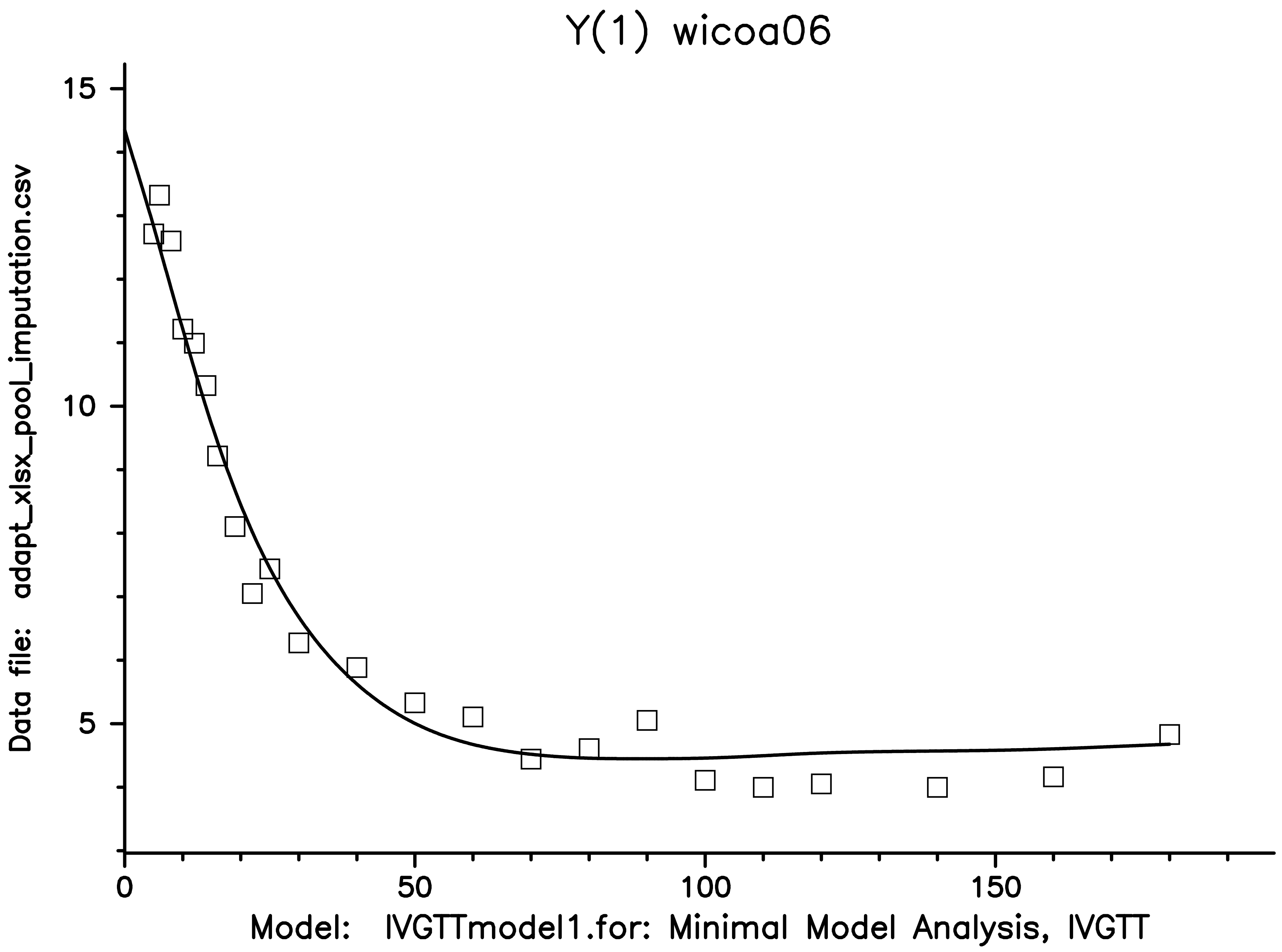

Y(1) wicoa06
15
10
5
0
50
100
150
Data file: adapt_xlsx_pool_imputation.csv
Model: IVGTTmodel1.for: Minimal Model Analysis, IVGTT

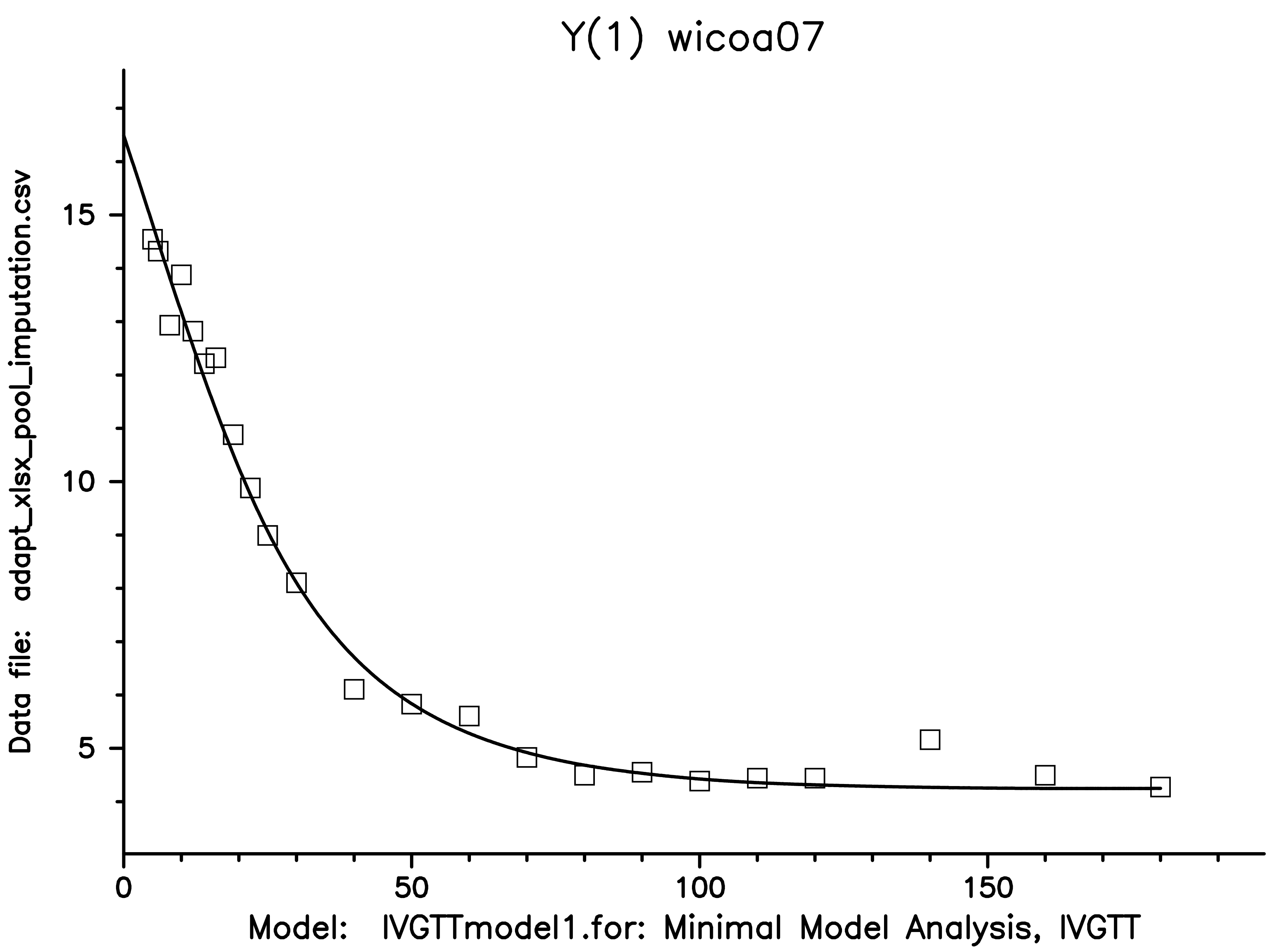

Y(1) wicoa07
Data file: adapt_xlsx_pool_imputation.csv
15
10
5
0
50
100
150
Model: IVGTTmodel1.for: Minimal Model Analysis, IVGTT

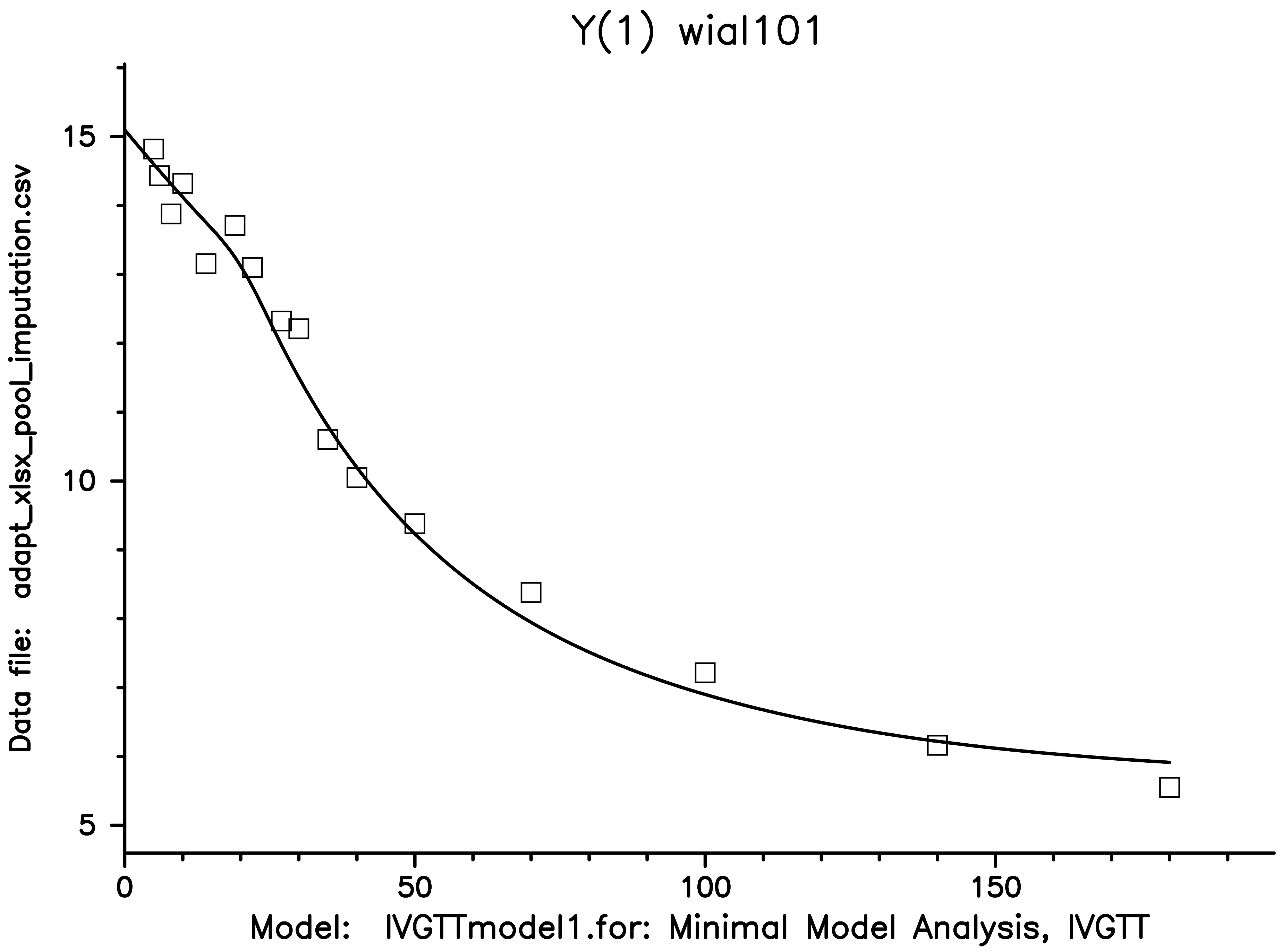
Y(1) wial101
Data file: adapt_xlsx_pool_imputation.csv
15
10
5
0
50
100
150
Model: IVGTTmodel1.for: Minimal Model Analysis, IVGTT

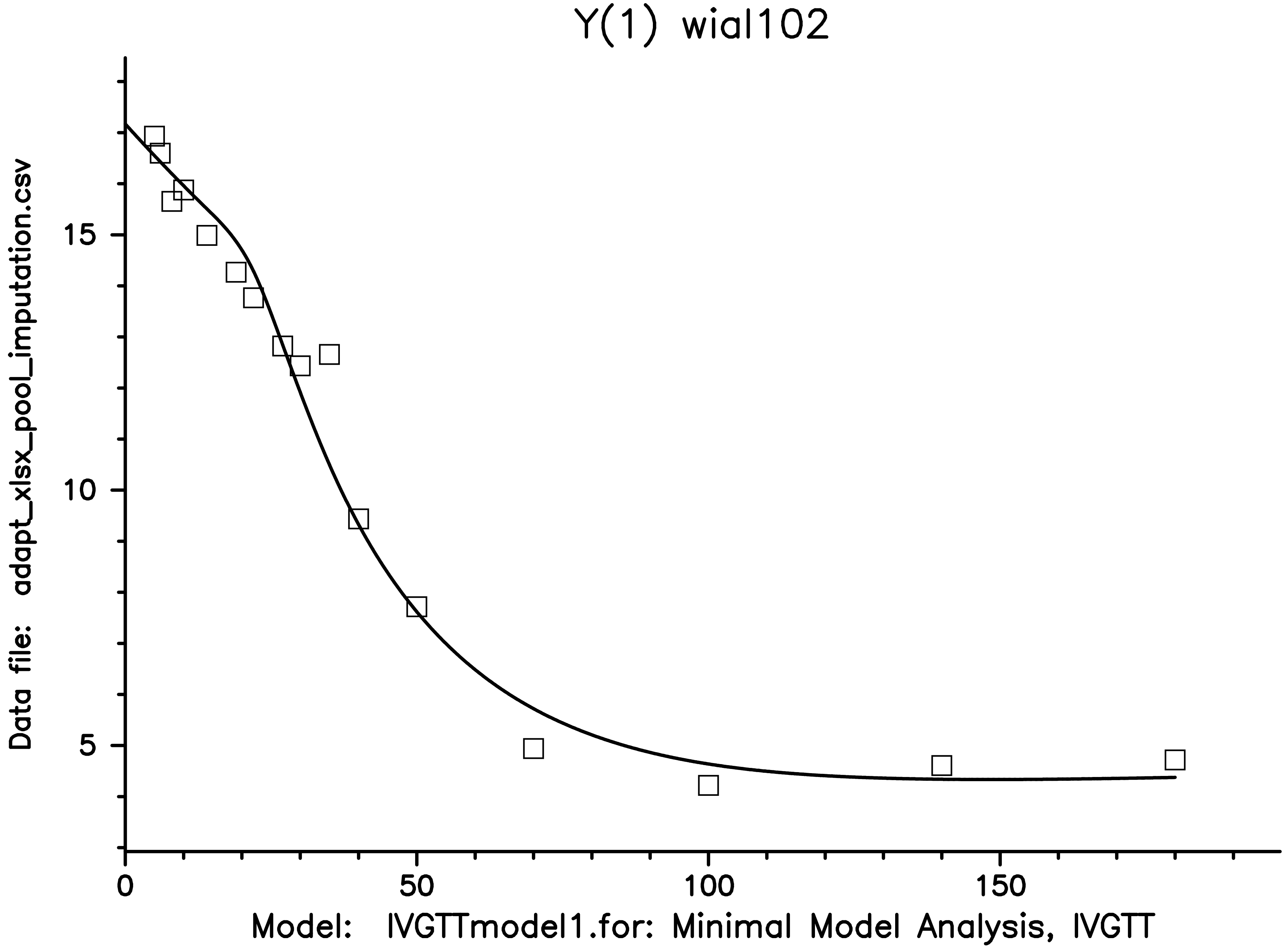
Y(1) wial102
Data file: adapt_xlsx_pool_imputation.csv
15
10
5
0
50
100
150
Model: IVGTTmodel1.for: Minimal Model Analysis, IVGTT

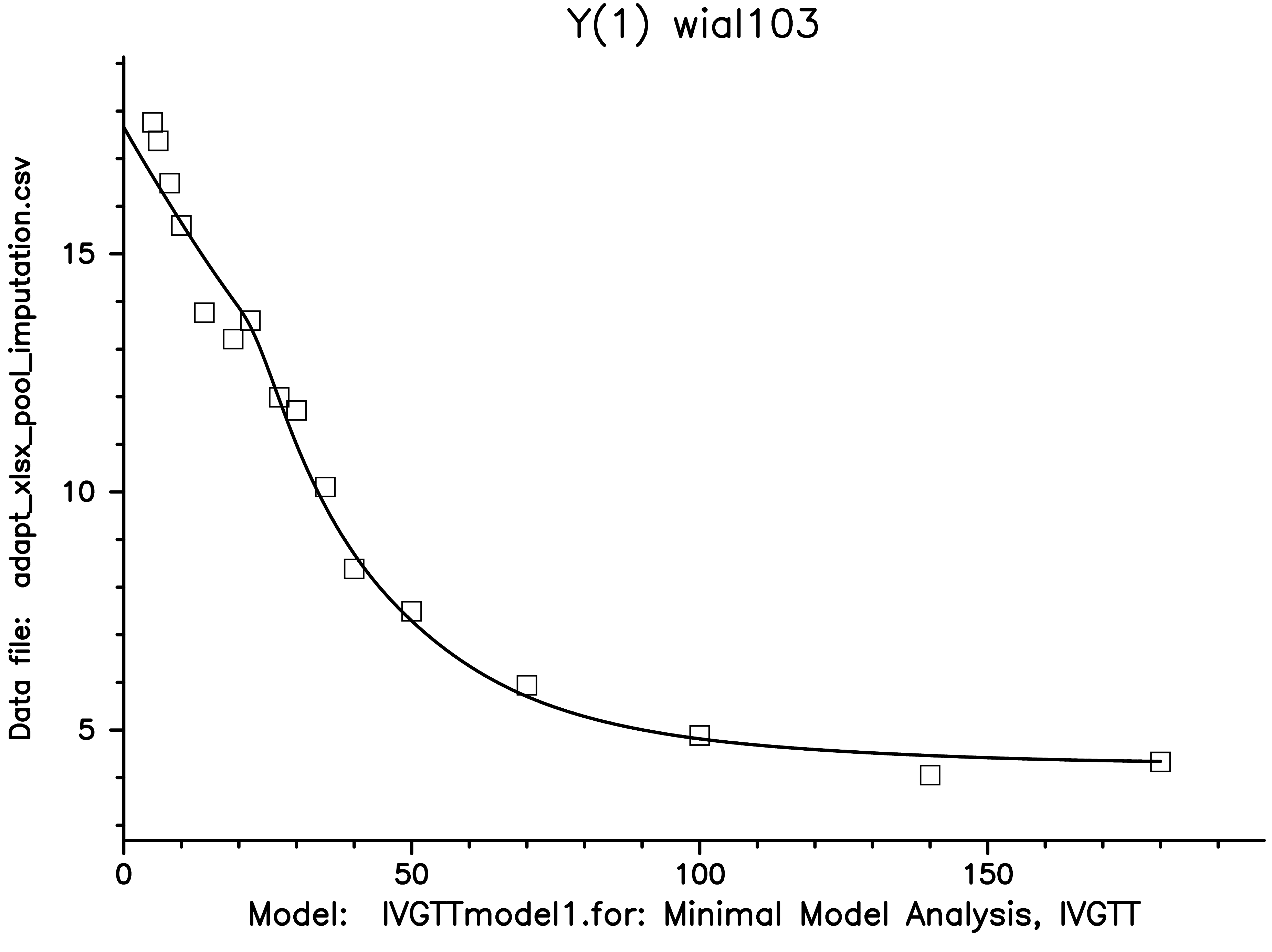

Y(1) wial103
Data file: adapt_xlsx_pool_imputation.csv
15
10
5
0
50
100
150
Model: IVGTTmodel1.for: Minimal Model Analysis, IVGTT

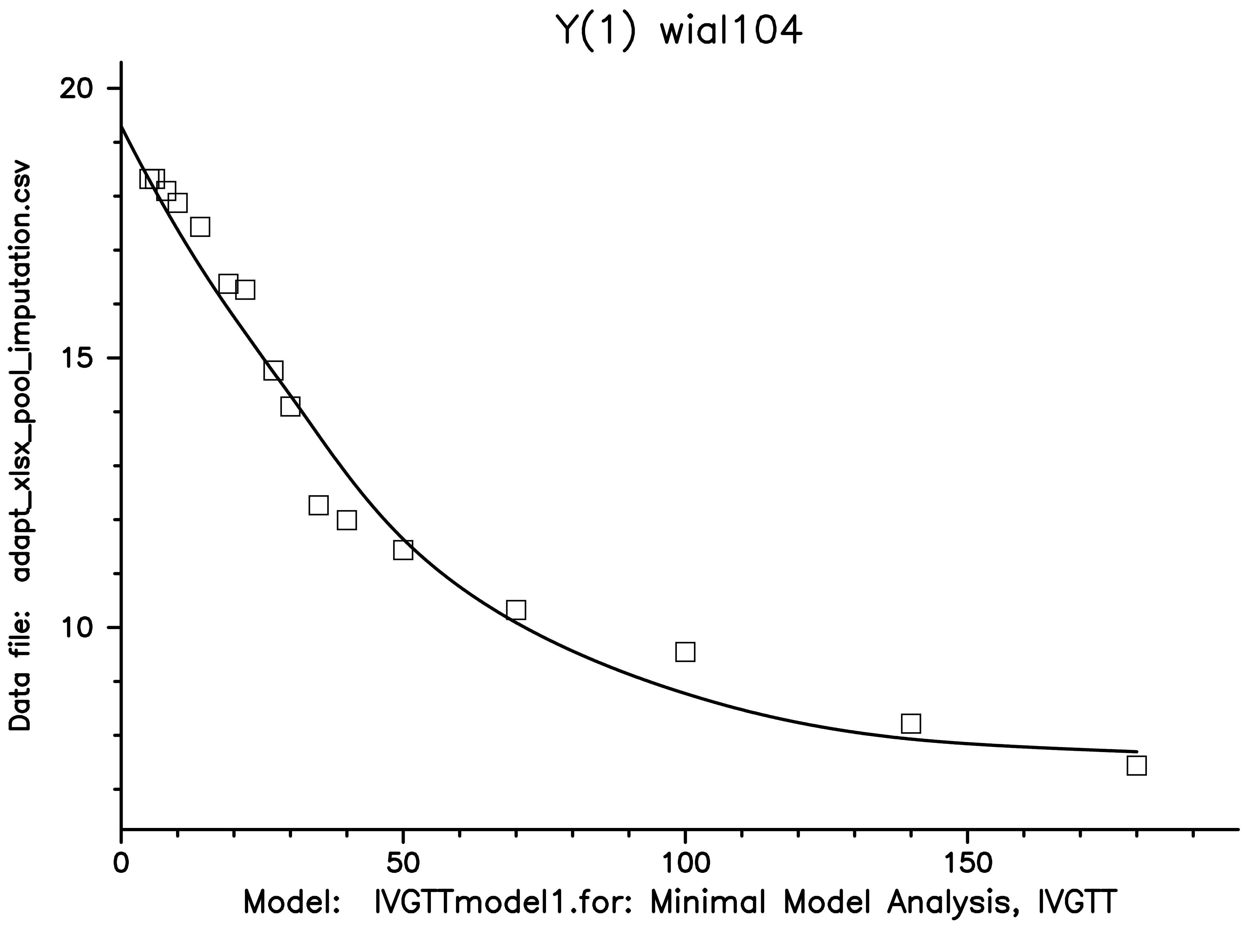
Y(1) wial104
Data file: adapt_xlsx_pool_imputation.csv
20
15
10
0
50
100
150
Model: IVGTTmodel1.for: Minimal Model Analysis, IVGTT

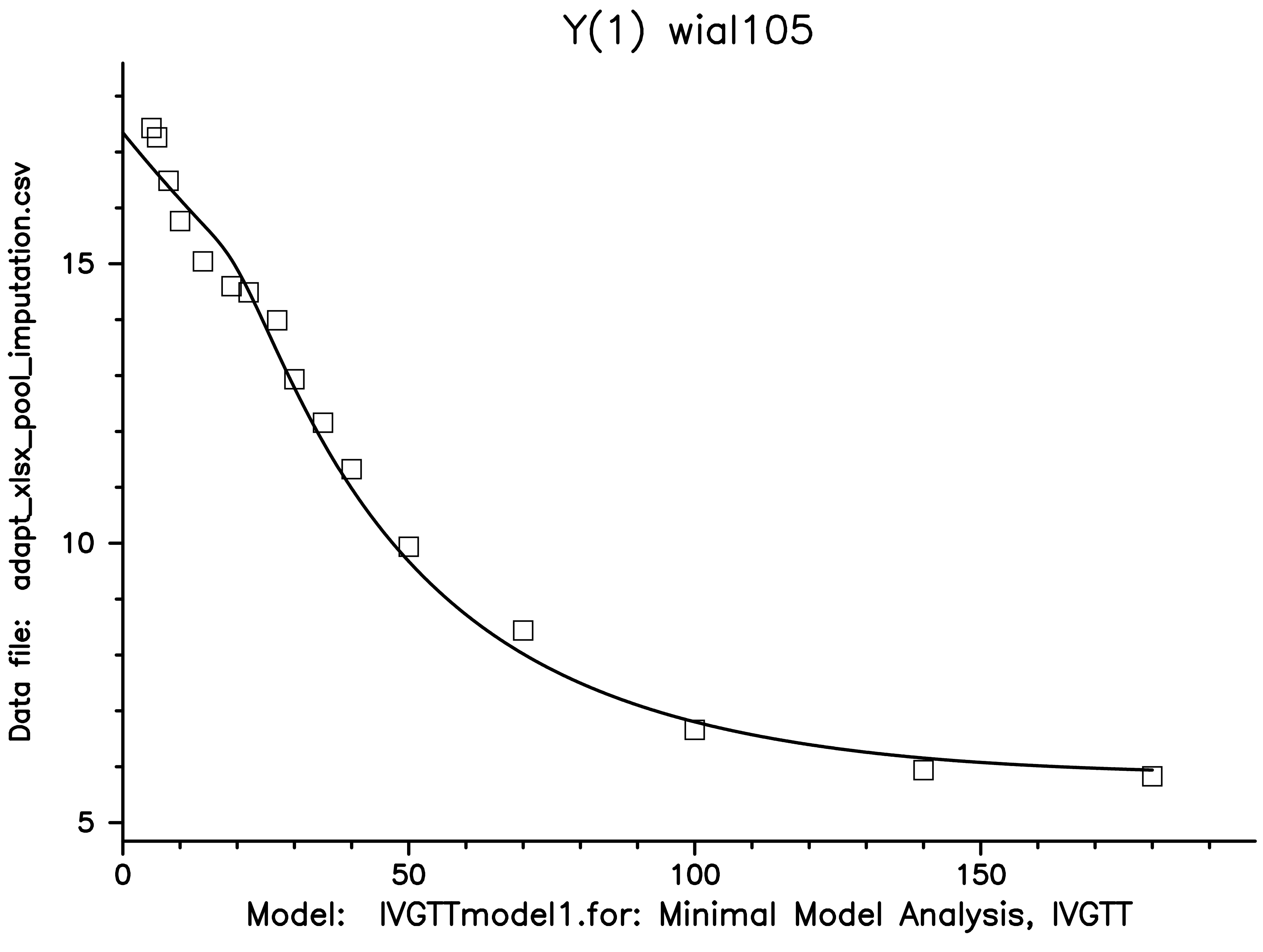

Y(1) wial105
Data file: adapt_xlsx_pool_imputation.csv
Model: IVGTTmodel1.for: Minimal Model Analysis, IVGTT
15
10
5
0
50
100
150

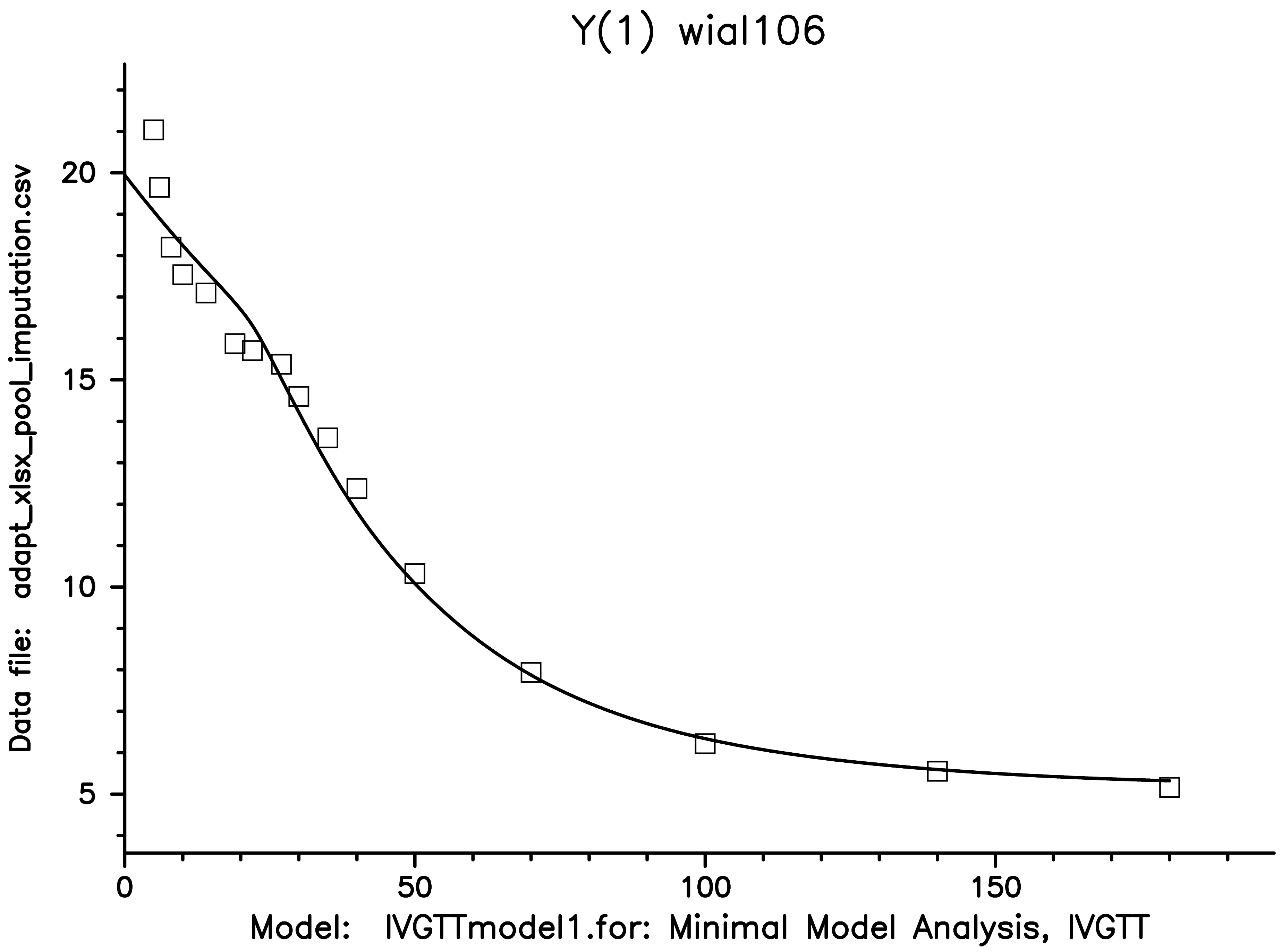

Y(1) wial106
Data file: adapt_xlsx_pool_imputation.csv
20
15
10
5
0
50
100
150
Model: IVGTTmodel1.for: Minimal Model Analysis, IVGTT

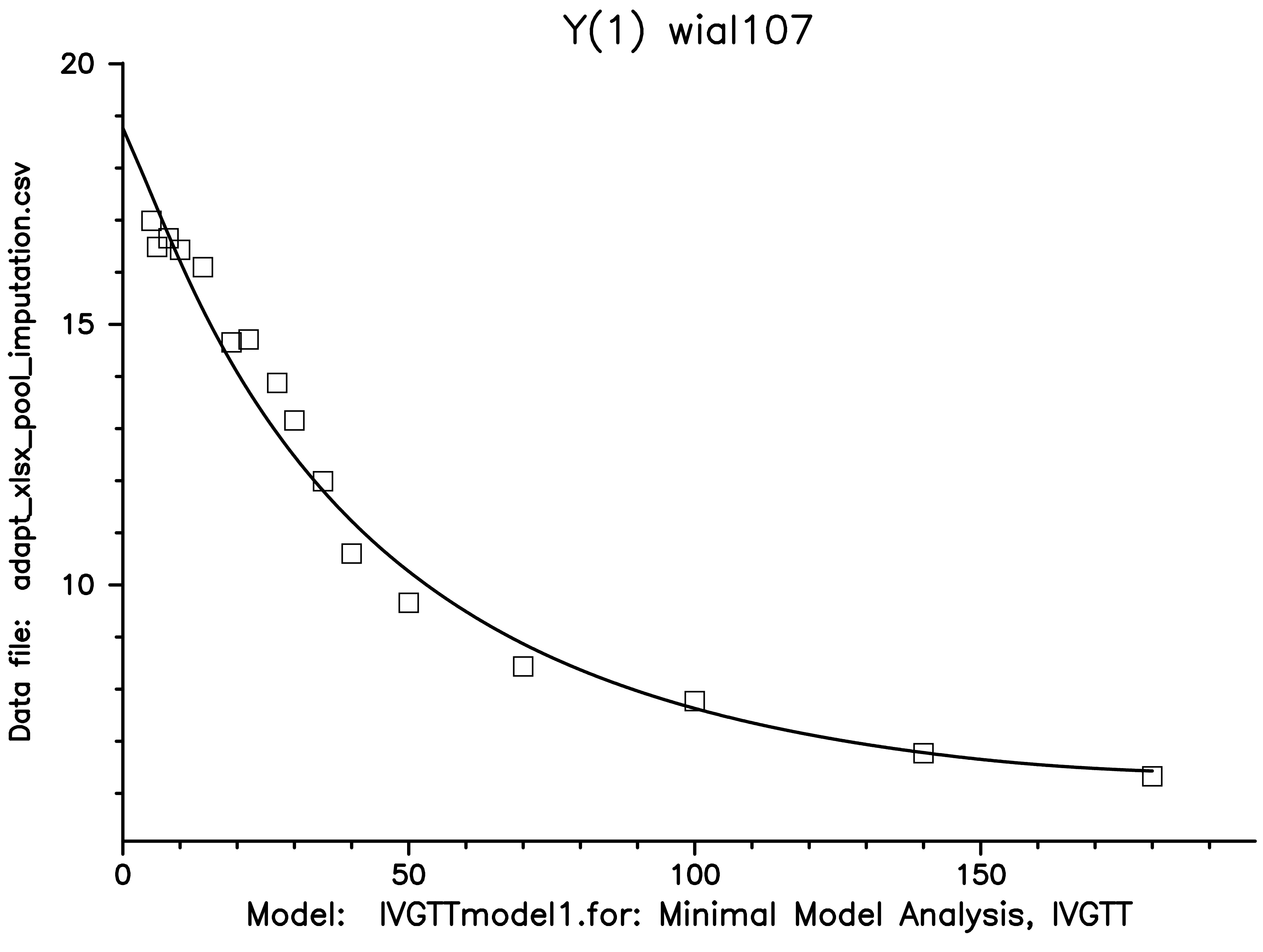
Y(1) wial107
Data file: adapt_xlsx_pool_imputation.csv
20
15
10
0
50
100
150
Model: IVGTTmodel1.for: Minimal Model Analysis, IVGTT

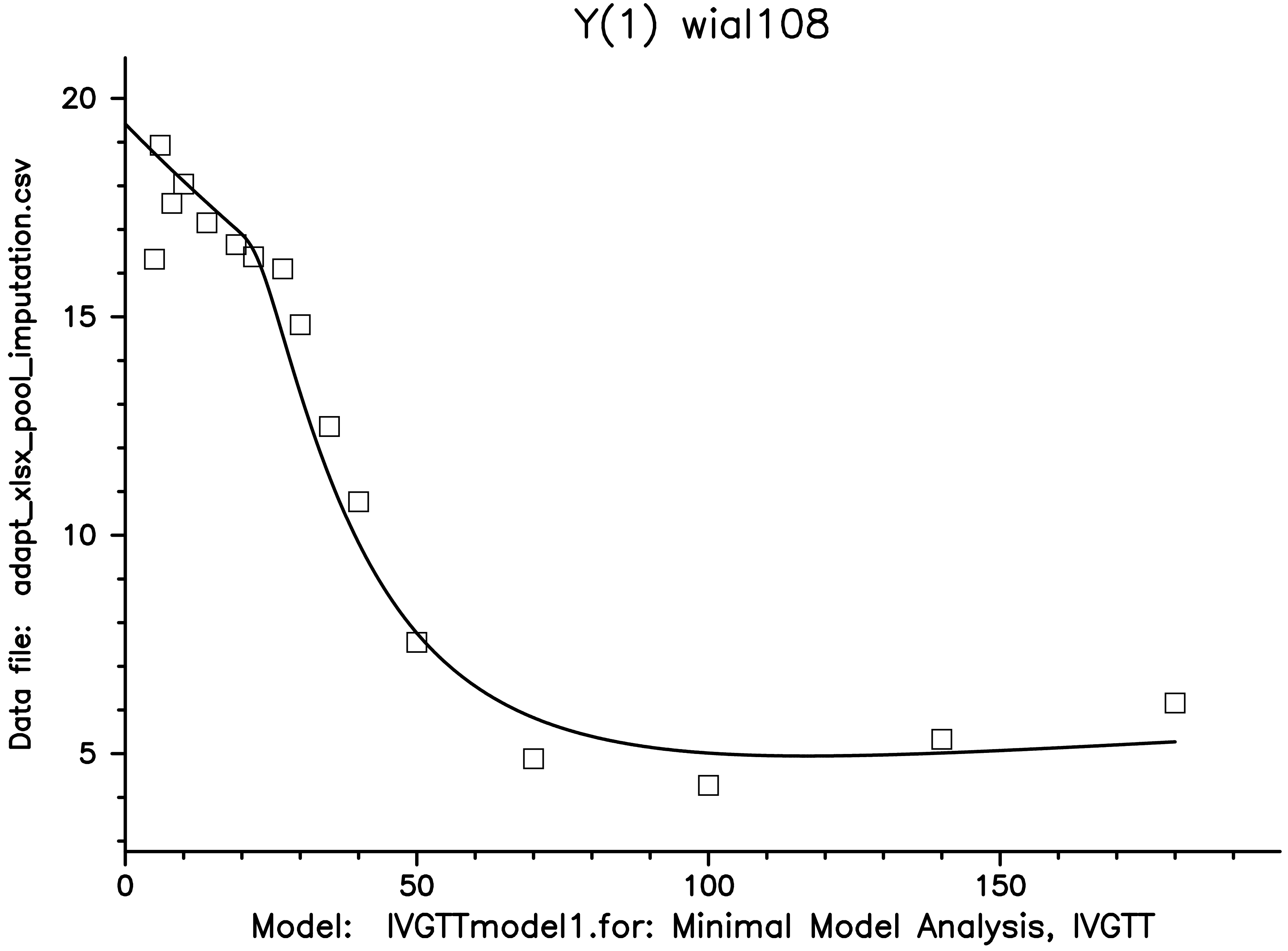

Y(1) wial108
Data file: adapt_xlsx_pool_imputation.csv
Model: IVGTTmodel1.for: Minimal Model Analysis, IVGTT
20
15
10
5
0
50
100
150

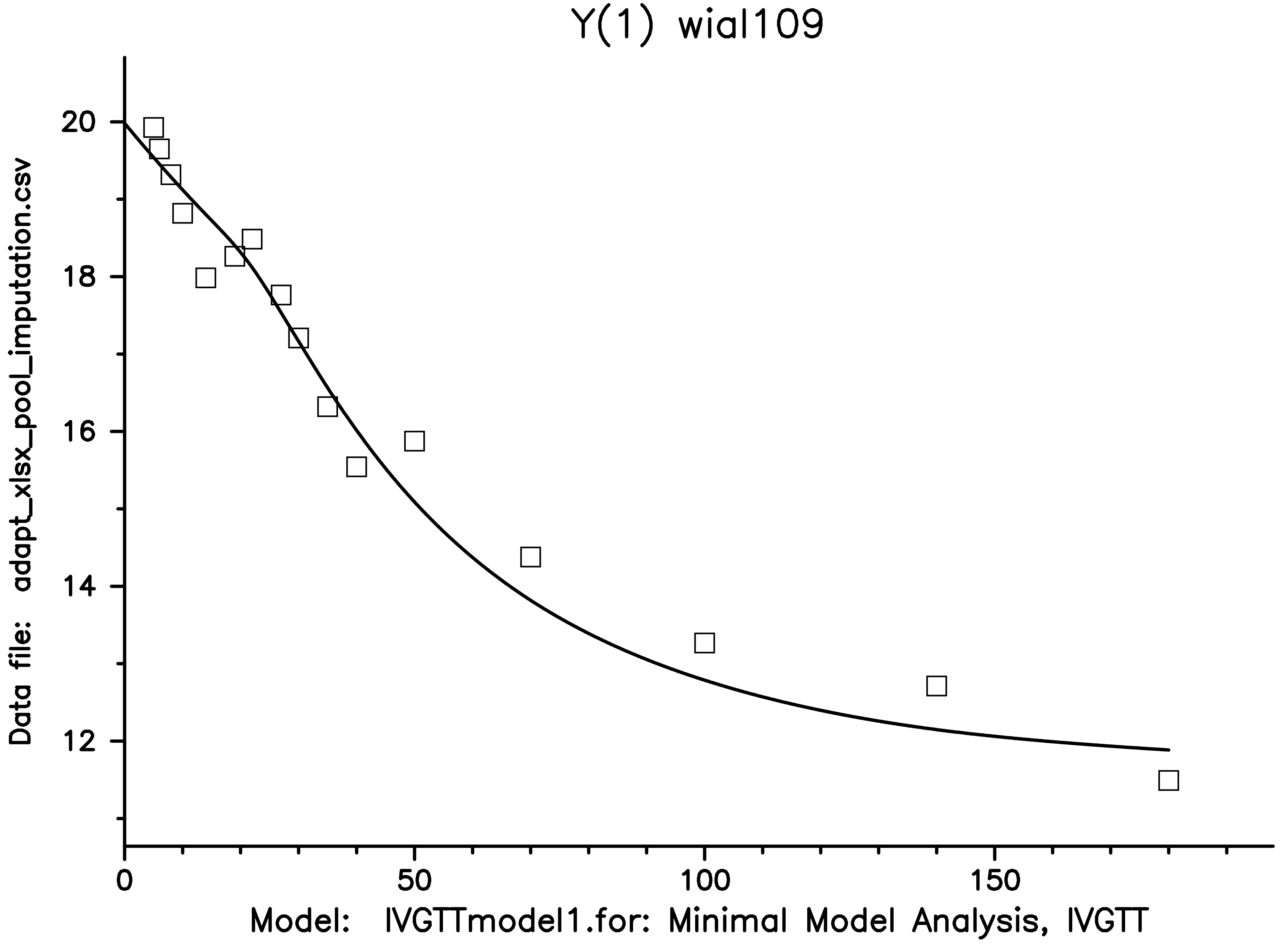
Y(1) wial109
Data file: adapt_xlsx_pool_imputation.csv
20
18
16
14
12
0
50
100
150
Model: IVGTTmodel1.for: Minimal Model Analysis, IVGTT

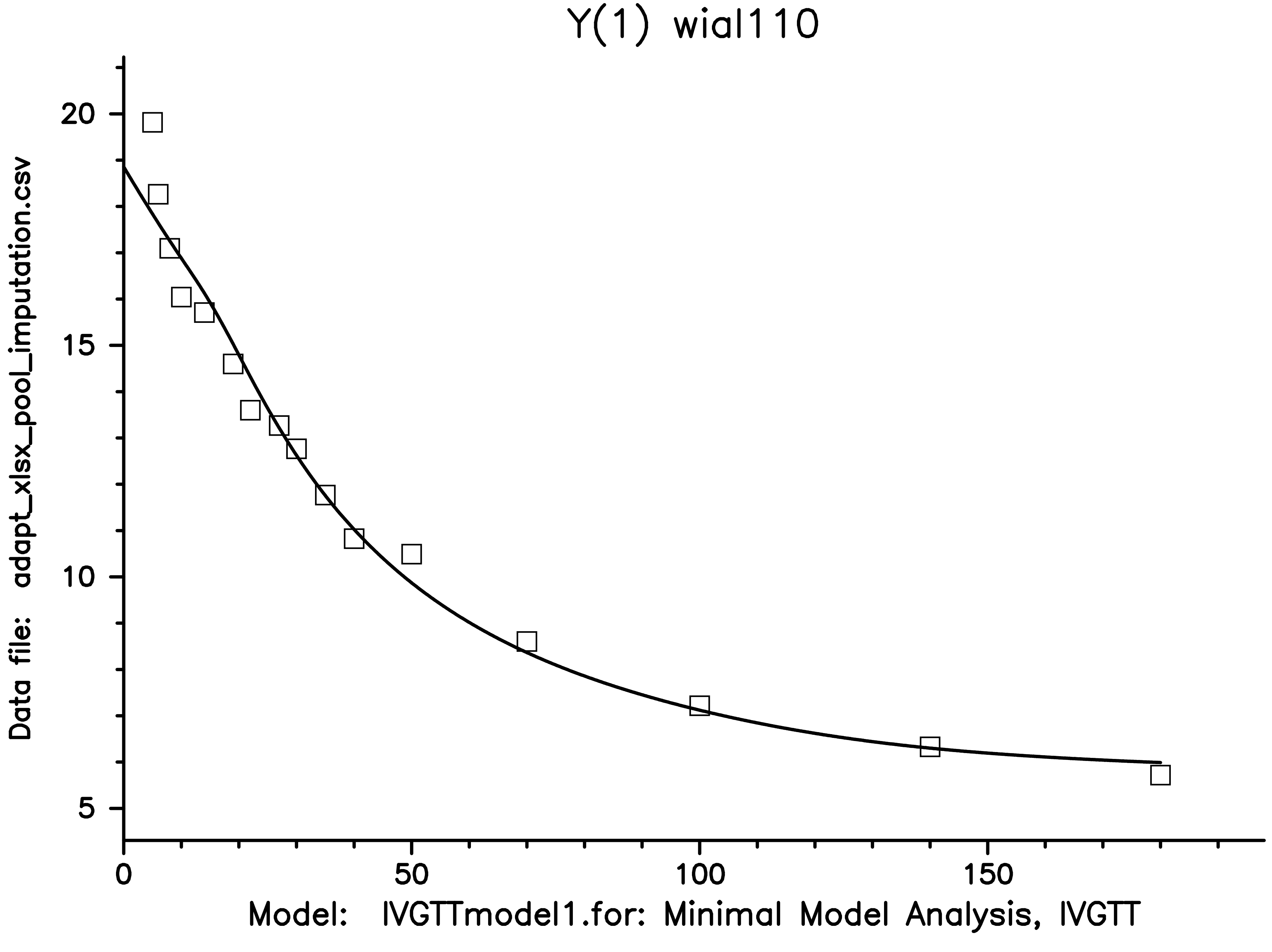
Y(1) wial110
Data file: adapt_xlsx_pool_imputation.csv
20
15
10
5
0
50
100
150
Model: IVGTTmodel1.for: Minimal Model Analysis, IVGTT

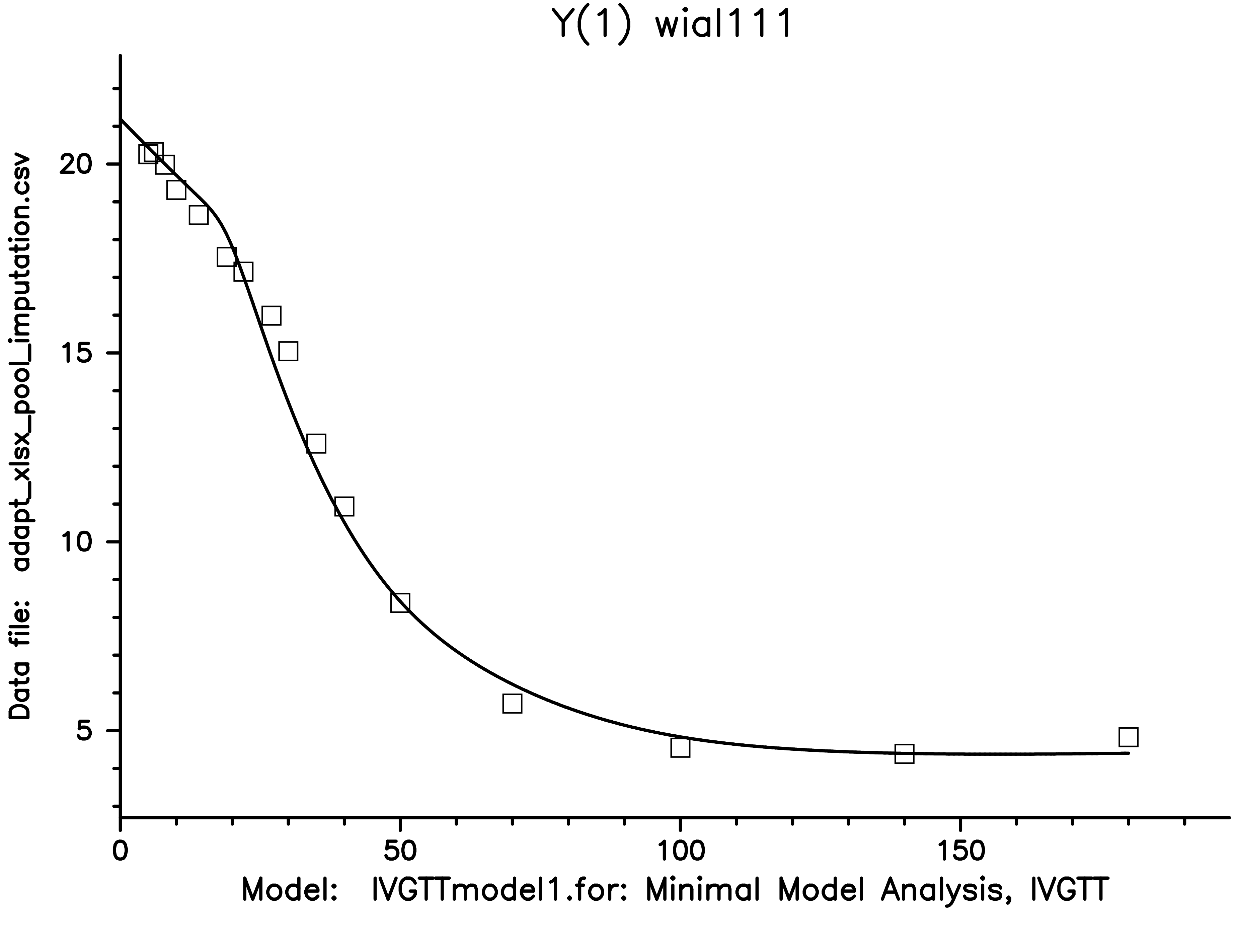
Y(1) wial111
Data file: adapt_xlsx_pool_imputation.csv
Model: IVGTTmodel1.for: Minimal Model Analysis, IVGTT
0
50
100
150
5
10
15
20

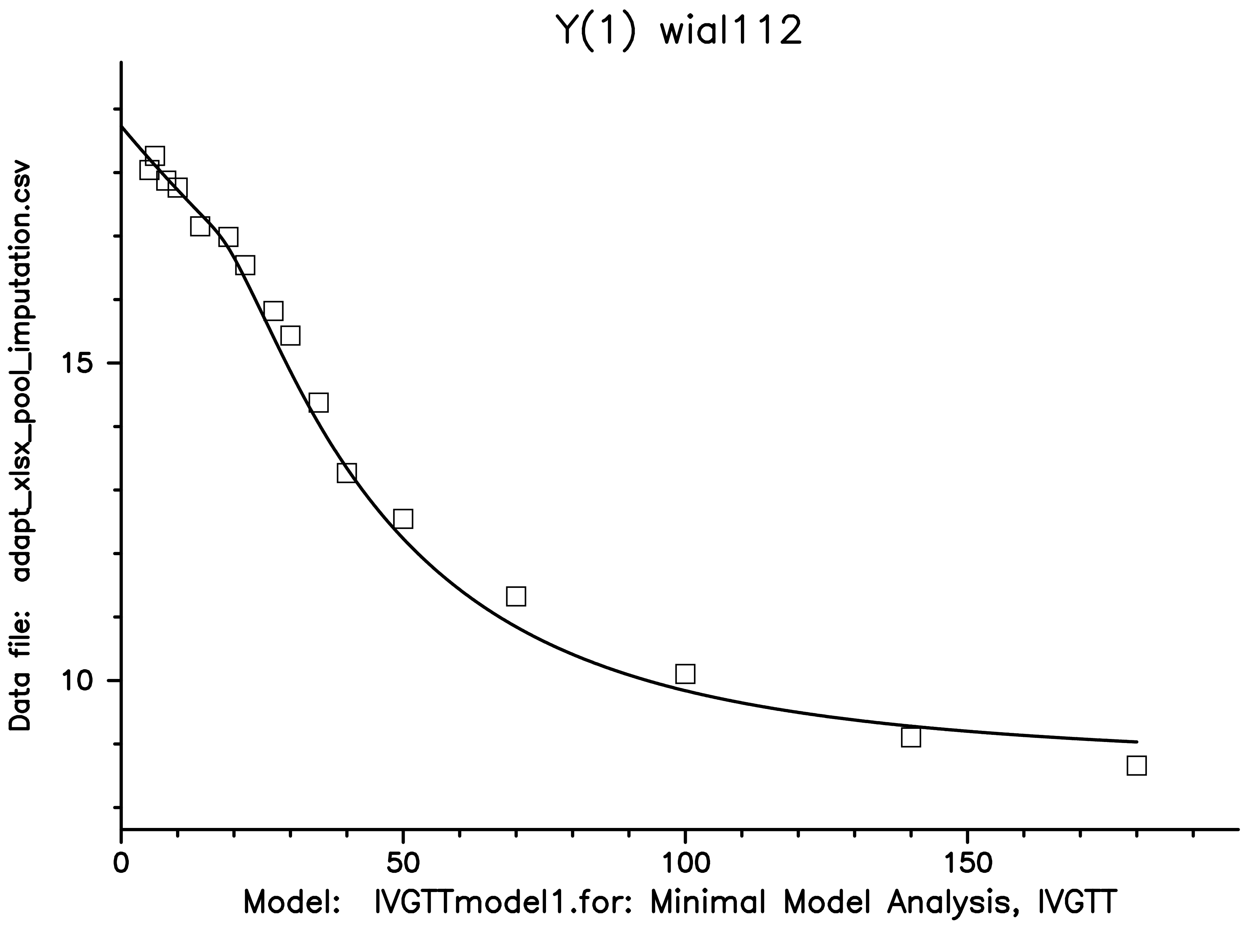

Y(1) wial112
Data file: adapt_xlsx_pool_imputation.csv
15
10
0
50
100
150
Model: IVGTTmodel1.for: Minimal Model Analysis, IVGTT

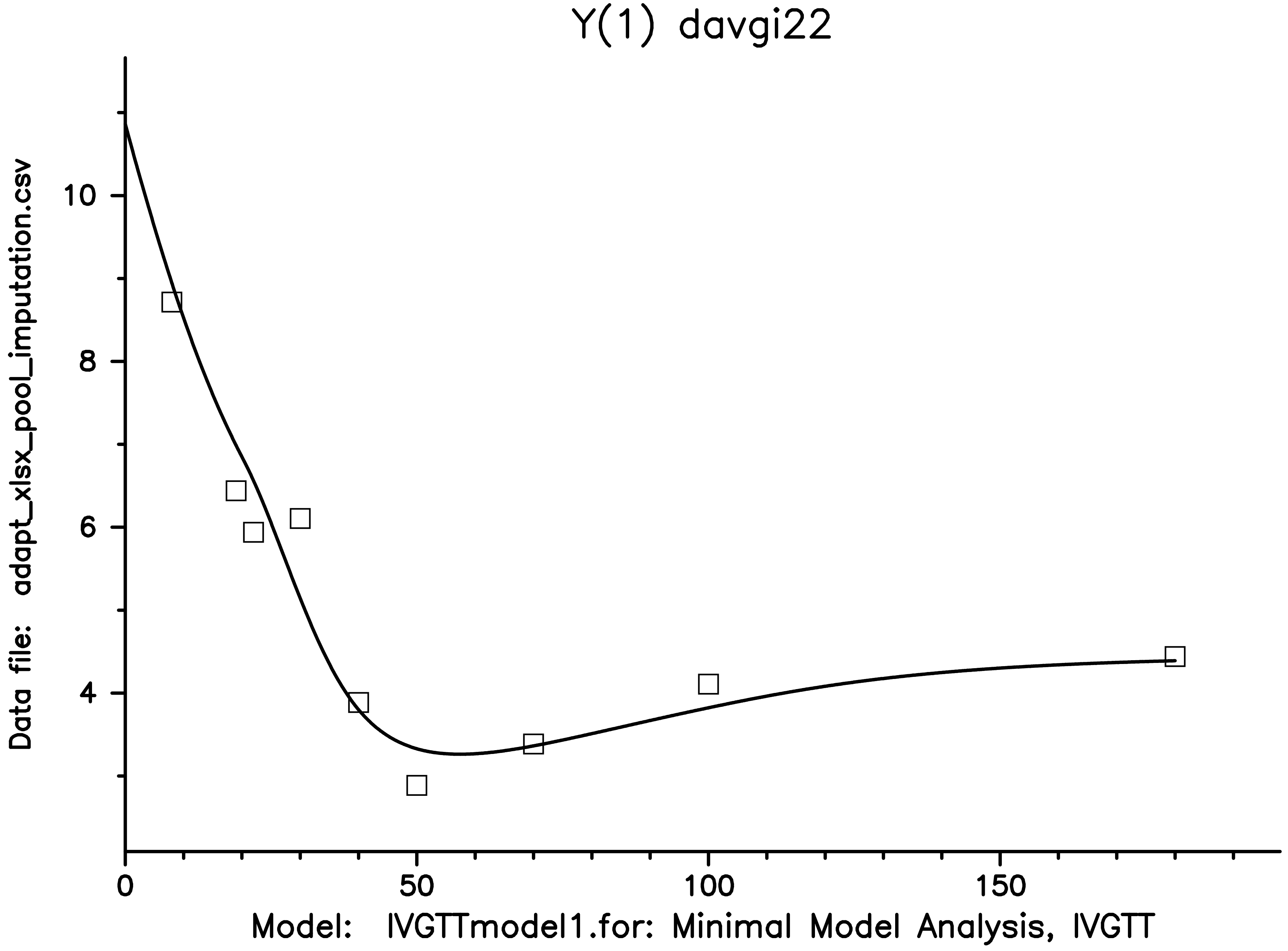

Y(1) davgi22
Data file: adapt_xlsx_pool_imputation.csv
10
8
6
4
0
50
100
150
Model: IVGTTmodel1.for: Minimal Model Analysis, IVGTT

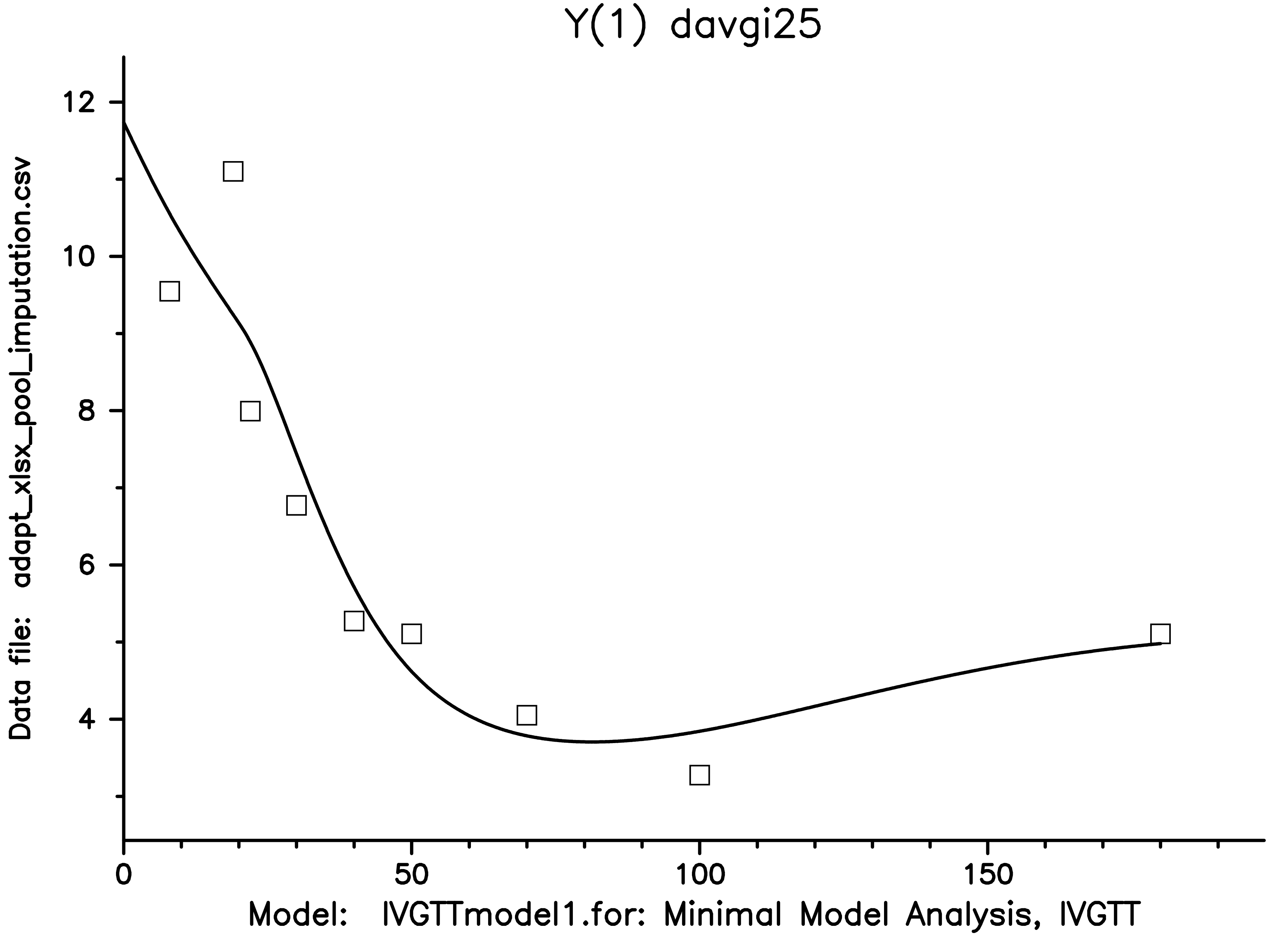
Y(1) davgi25
Data file: adapt_xlsx_pool_imputation.csv
12
10
8
6
4
0
50
100
150
Model: IVGTTmodel1.for: Minimal Model Analysis, IVGTT

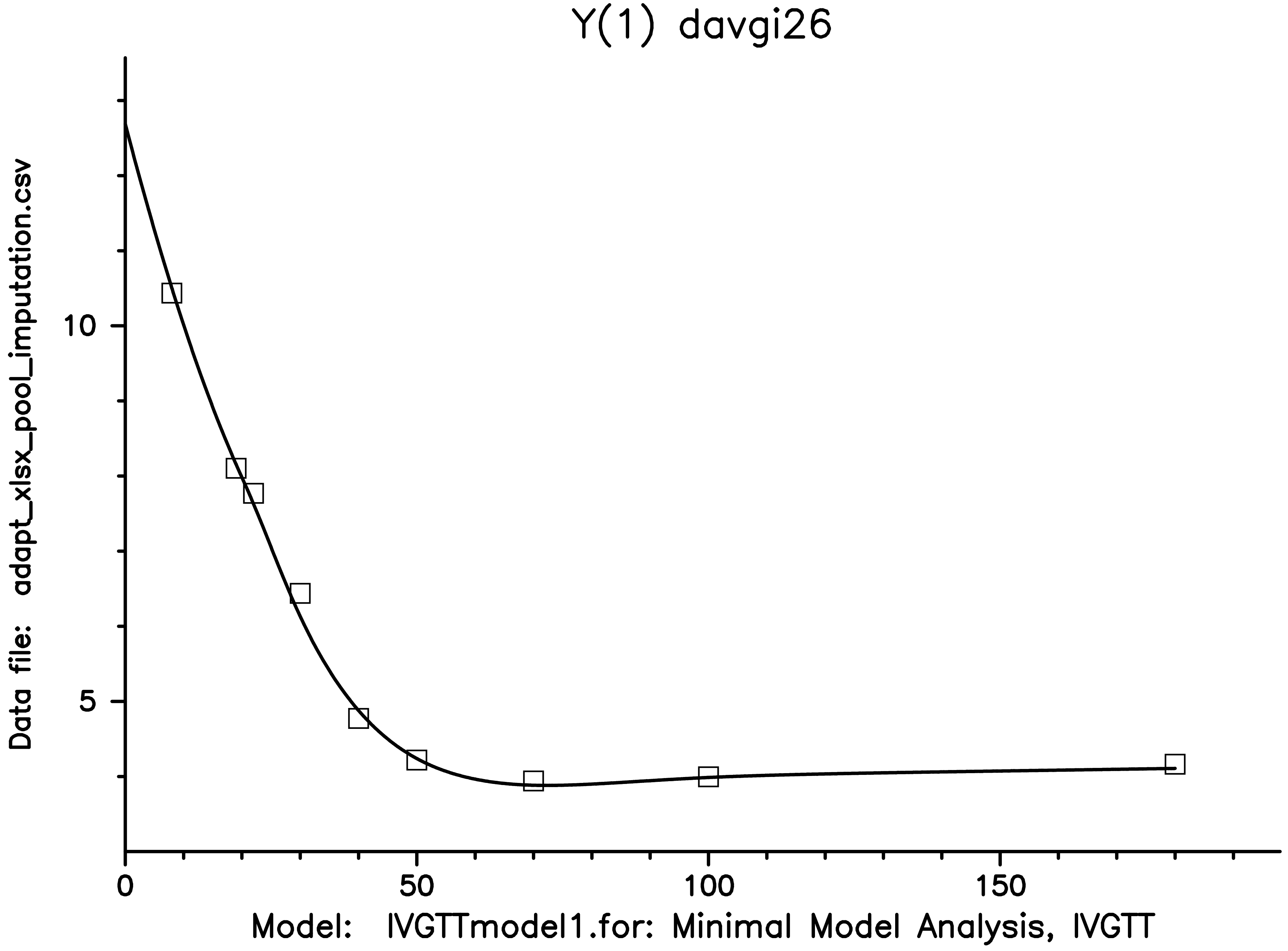

Y(1) davgi26
Data file: adapt_xlsx_pool_imputation.csv
10
5
0
50
100
150
Model: IVGTTmodel1.for: Minimal Model Analysis, IVGTT

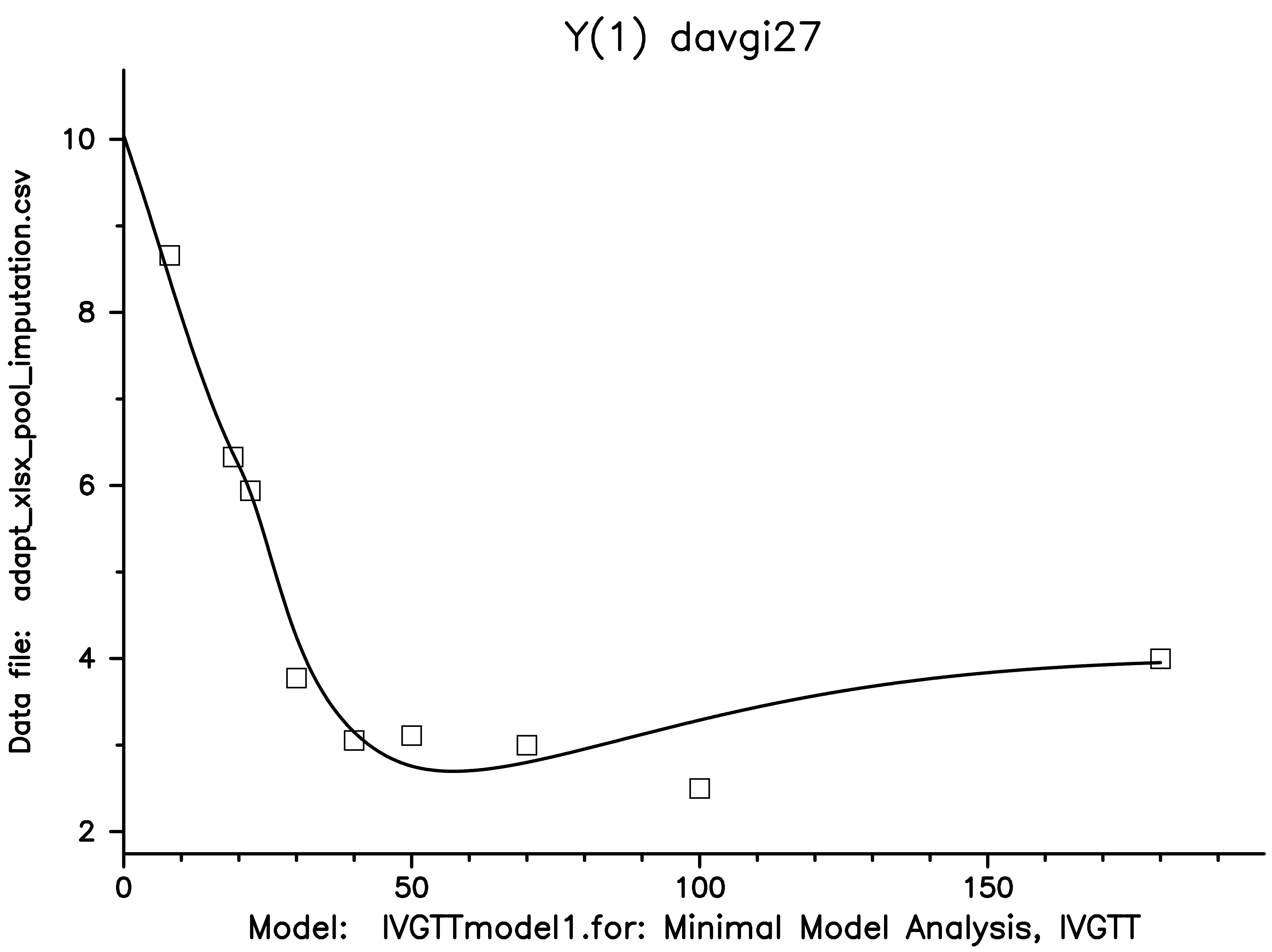
Y(1) davgi27
Data file: adapt_xlsx_pool_imputation.csv
10
8
6
4
2
0
50
100
150
Model: IVGTTmodel1.for: Minimal Model Analysis, IVGTT

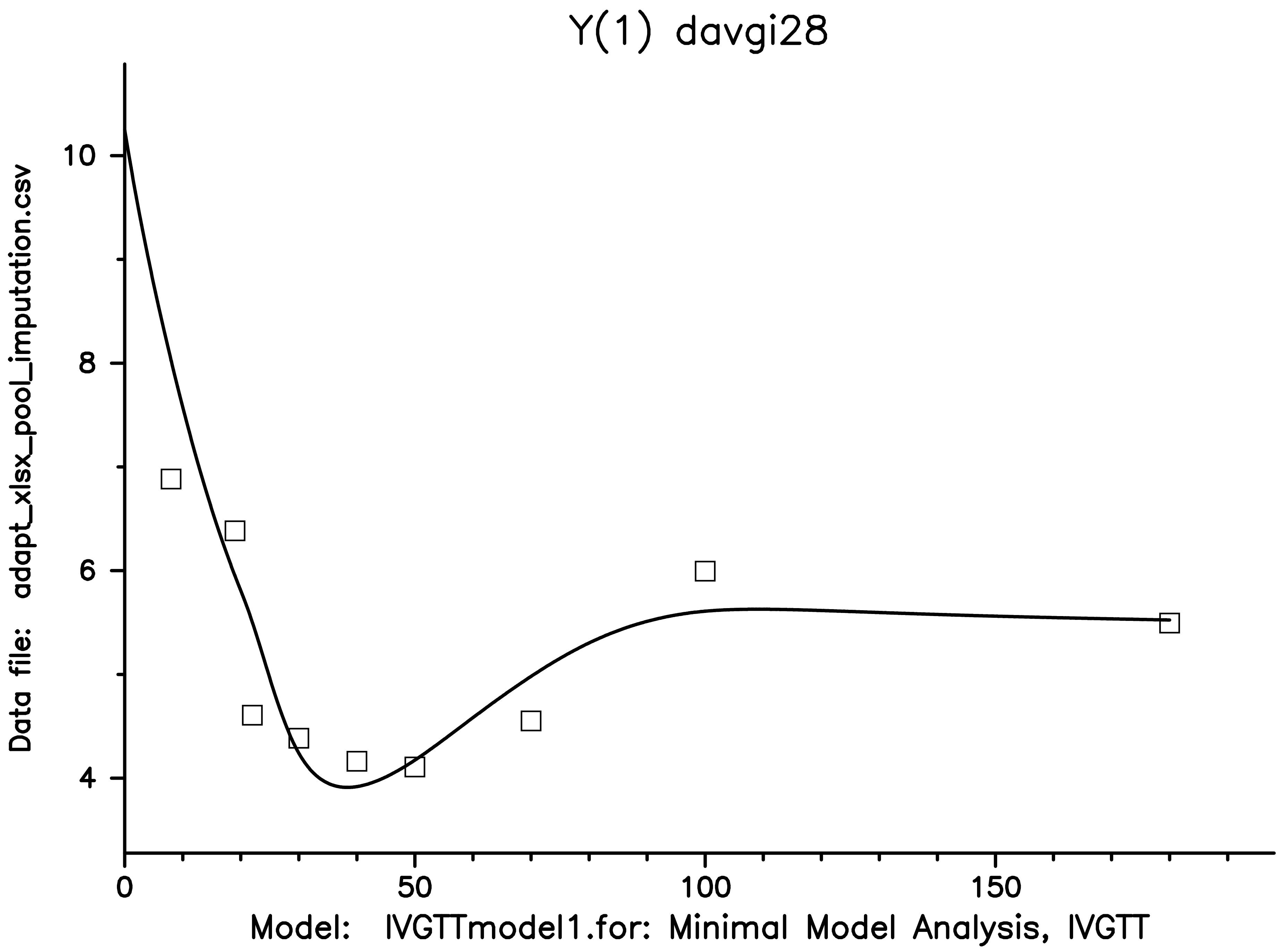
Y(1) davgi28
Data file: adapt_xlsx_pool_imputation.csv
10
8
6
4
0
50
100
150
Model: IVGTTmodel1.for: Minimal Model Analysis, IVGTT

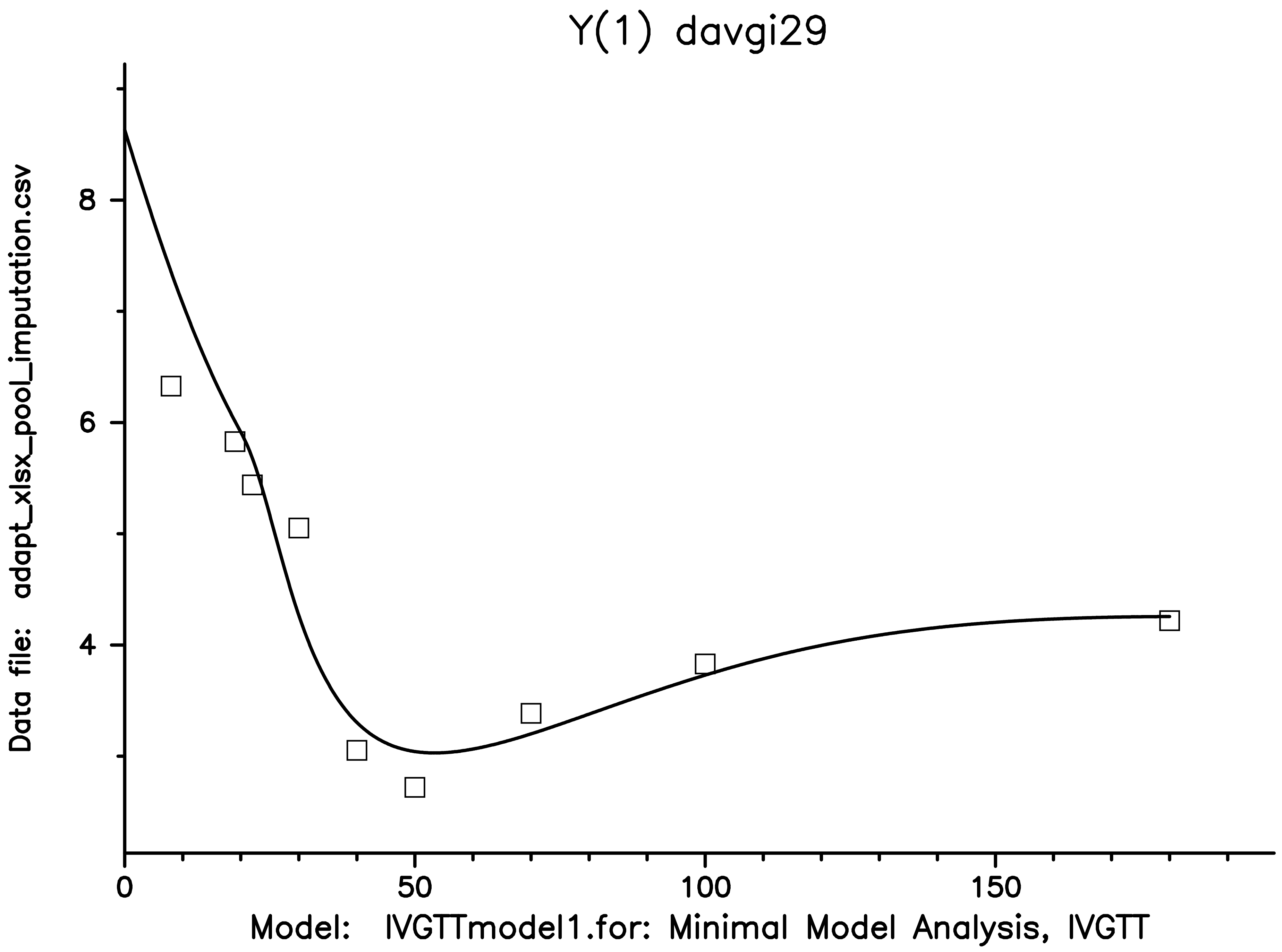
Y(1) davgi29
8
6
4
0
50
100
150
Data file: adapt_xlsx_pool_imputation.csv
Model: IVGTTmodel1.for: Minimal Model Analysis, IVGTT

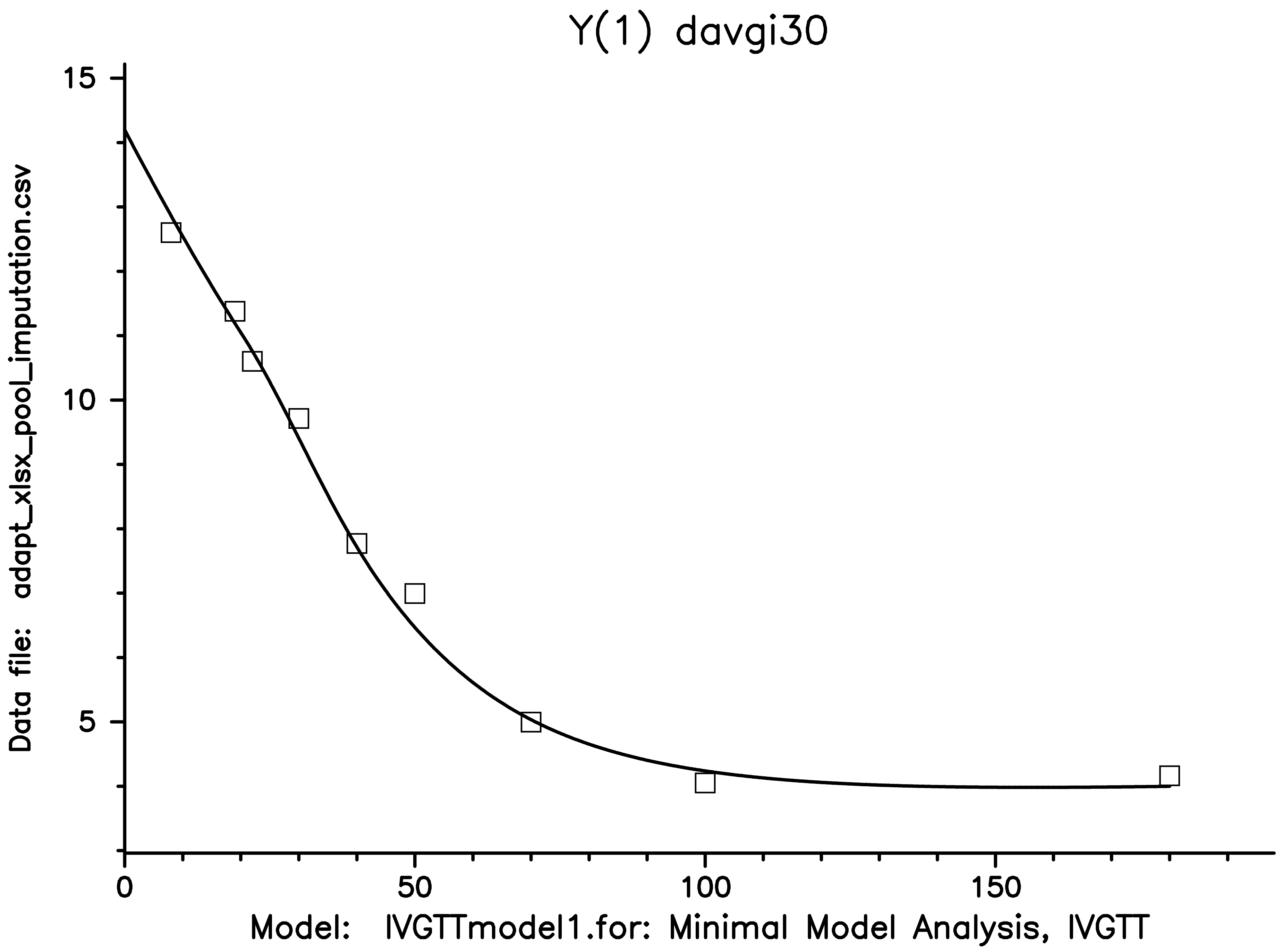

Y(1) davgi30
Data file: adapt_xlsx_pool_imputation.csv
15
10
5
0
50
100
150
Model: IVGTTmodel1.for: Minimal Model Analysis, IVGTT

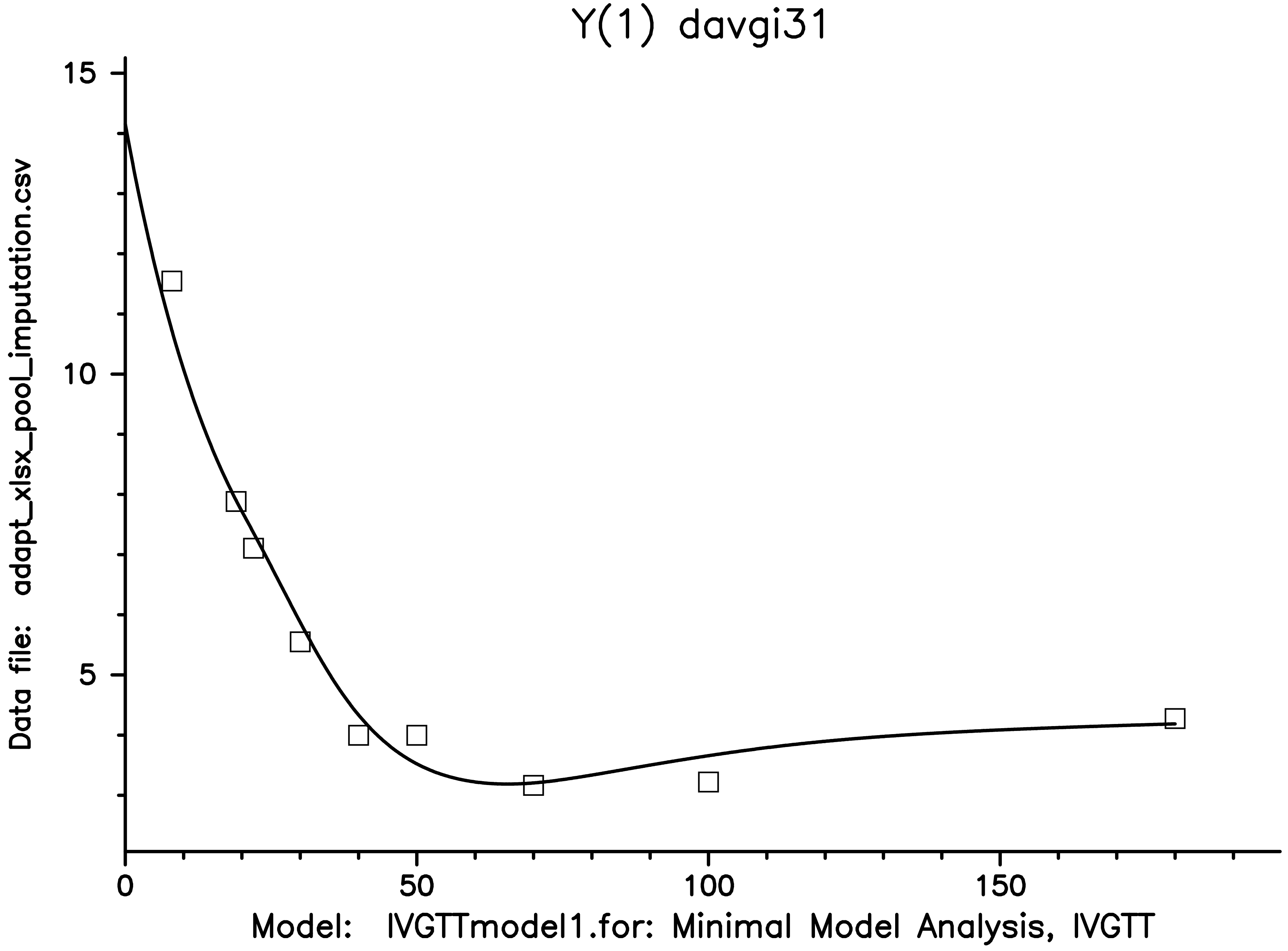

Y(1) davgi31
15
10
5
0
50
100
150
Data file: adapt_xlsx_pool_imputation.csv
Model: IVGTTmodel1.for: Minimal Model Analysis, IVGTT

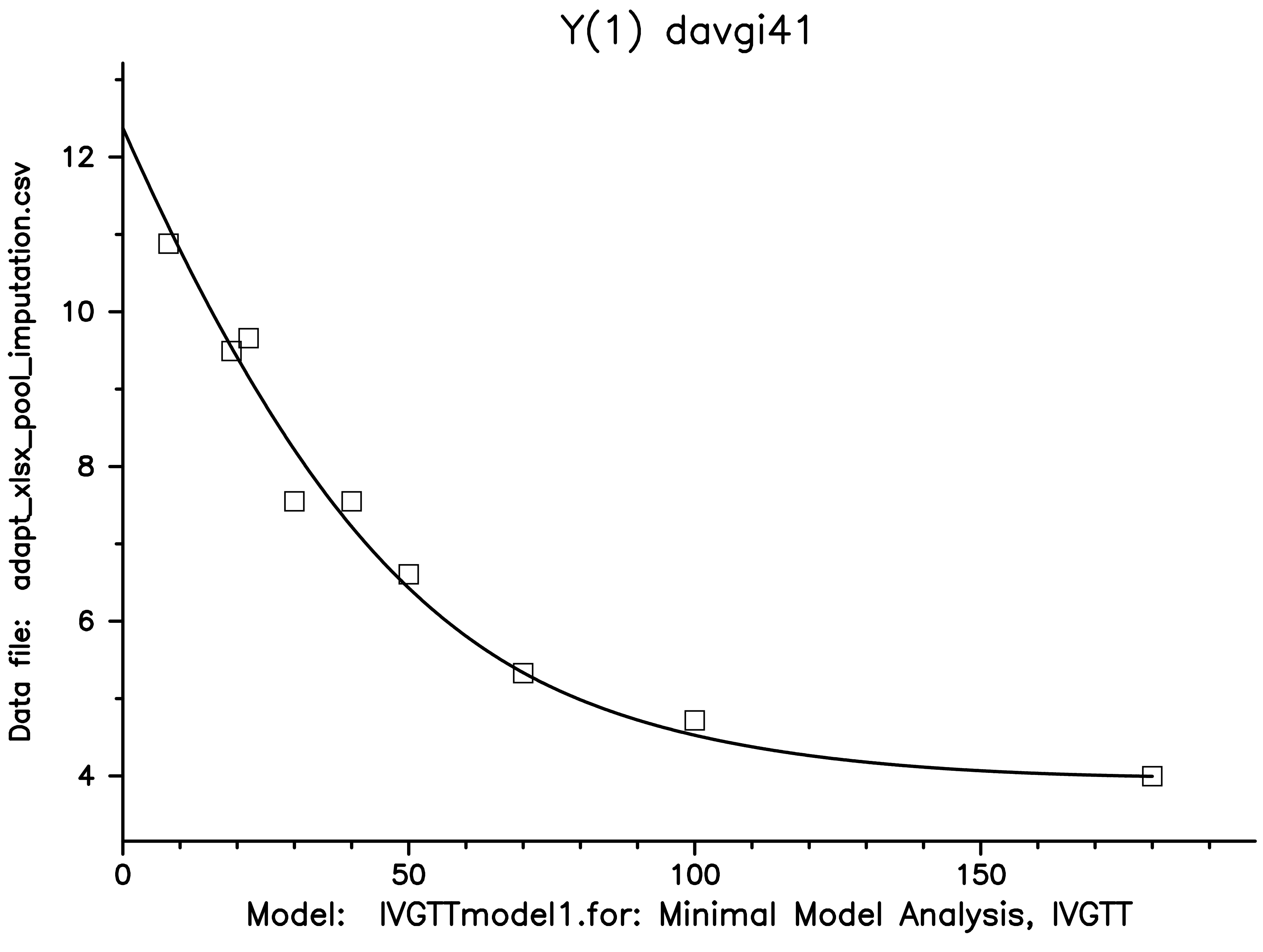
Y(1) davgi41
Data file: adapt_xlsx_pool_imputation.csv
12
10
8
6
4
0
50
100
150
Model: IVGTTmodel1.for: Minimal Model Analysis, IVGTT

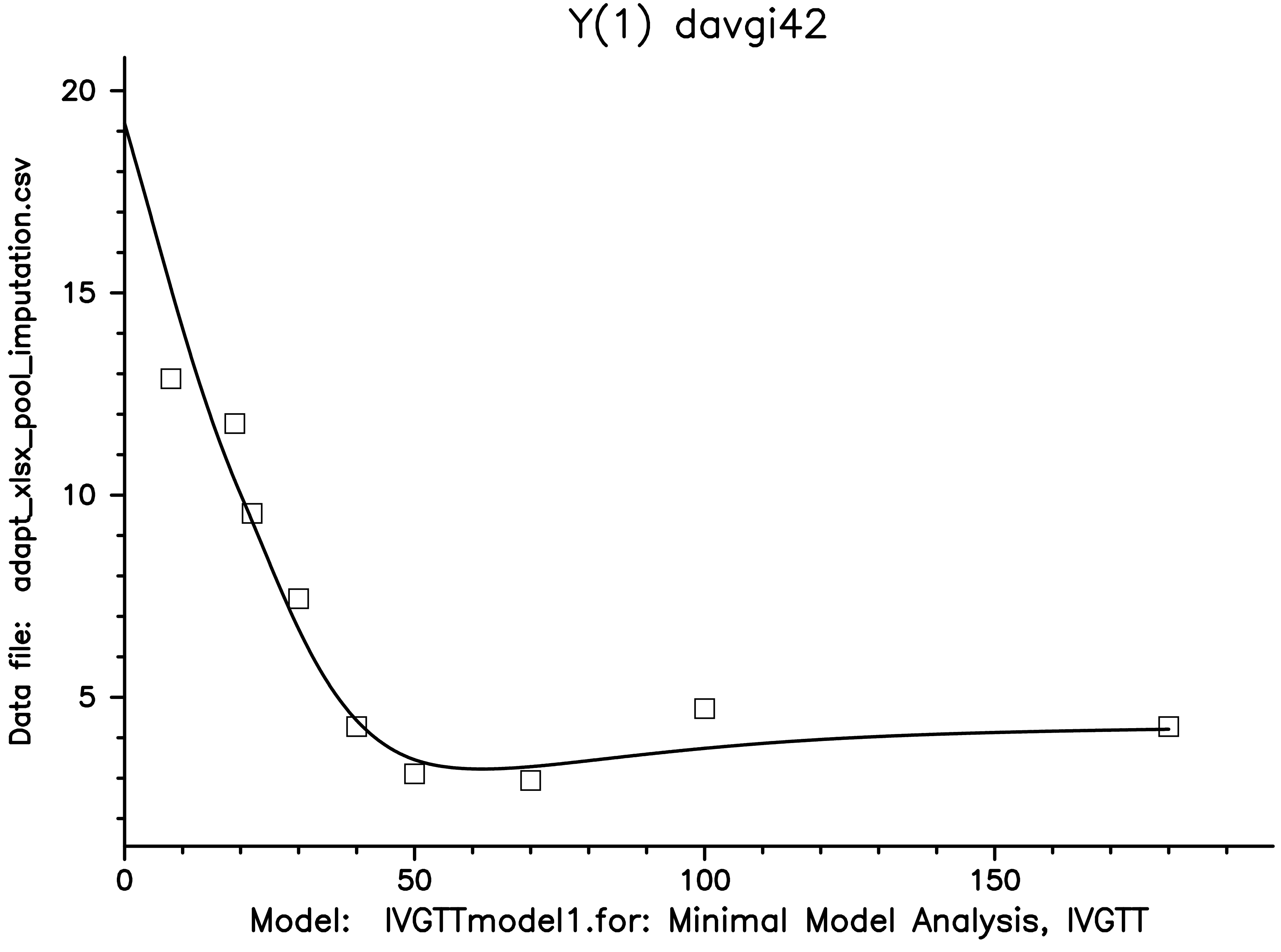
Y(1) davgi42
Data file: adapt_xlsx_pool_imputation.csv
20
15
10
5
0
50
100
150
Model: IVGTTmodel1.for: Minimal Model Analysis, IVGTT

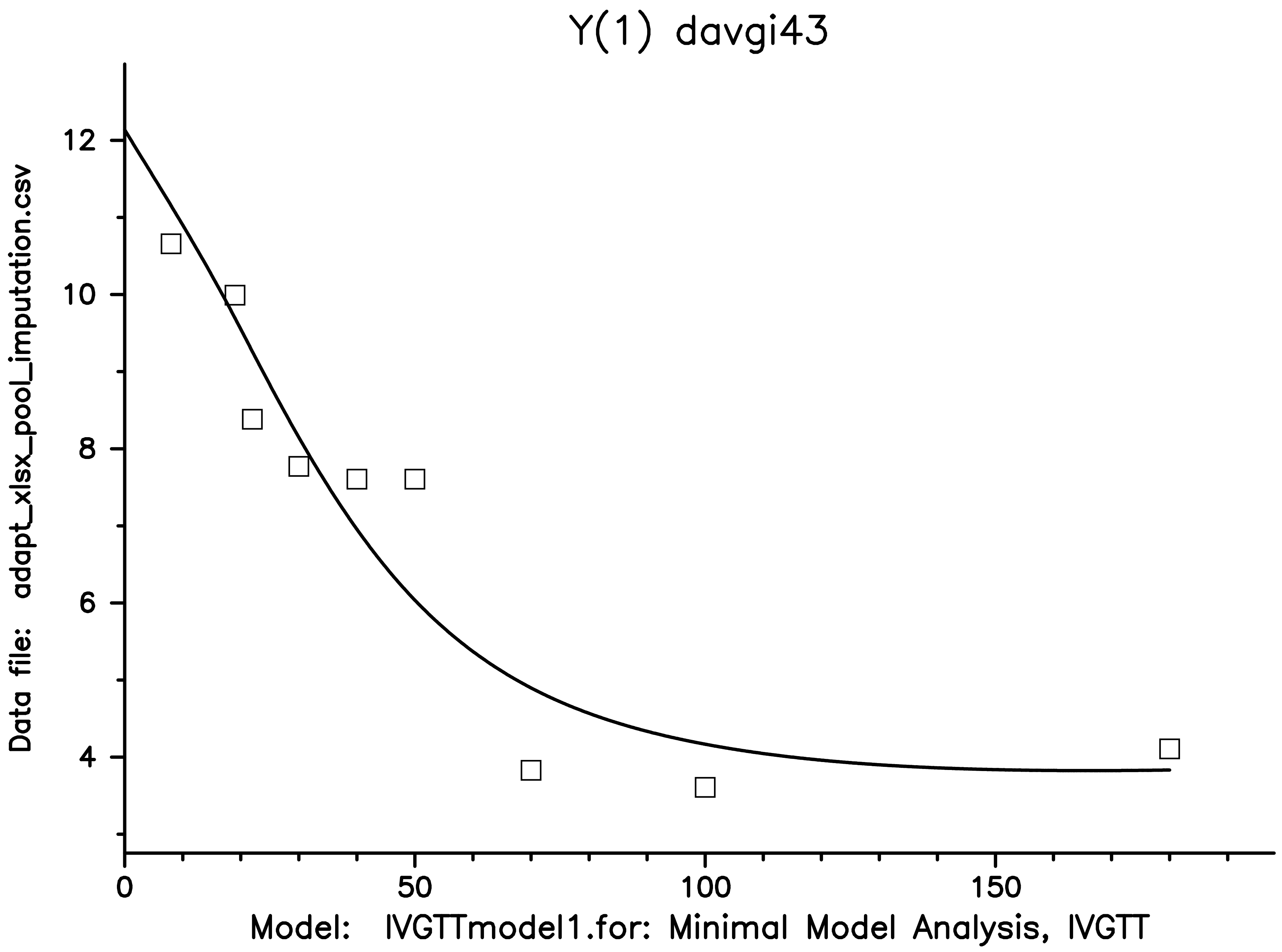
Y(1) davgi43
Data file: adapt_xlsx_pool_imputation.csv
12
10
8
6
4
0
50
100
150
Model: IVGTTmodel1.for: Minimal Model Analysis, IVGTT

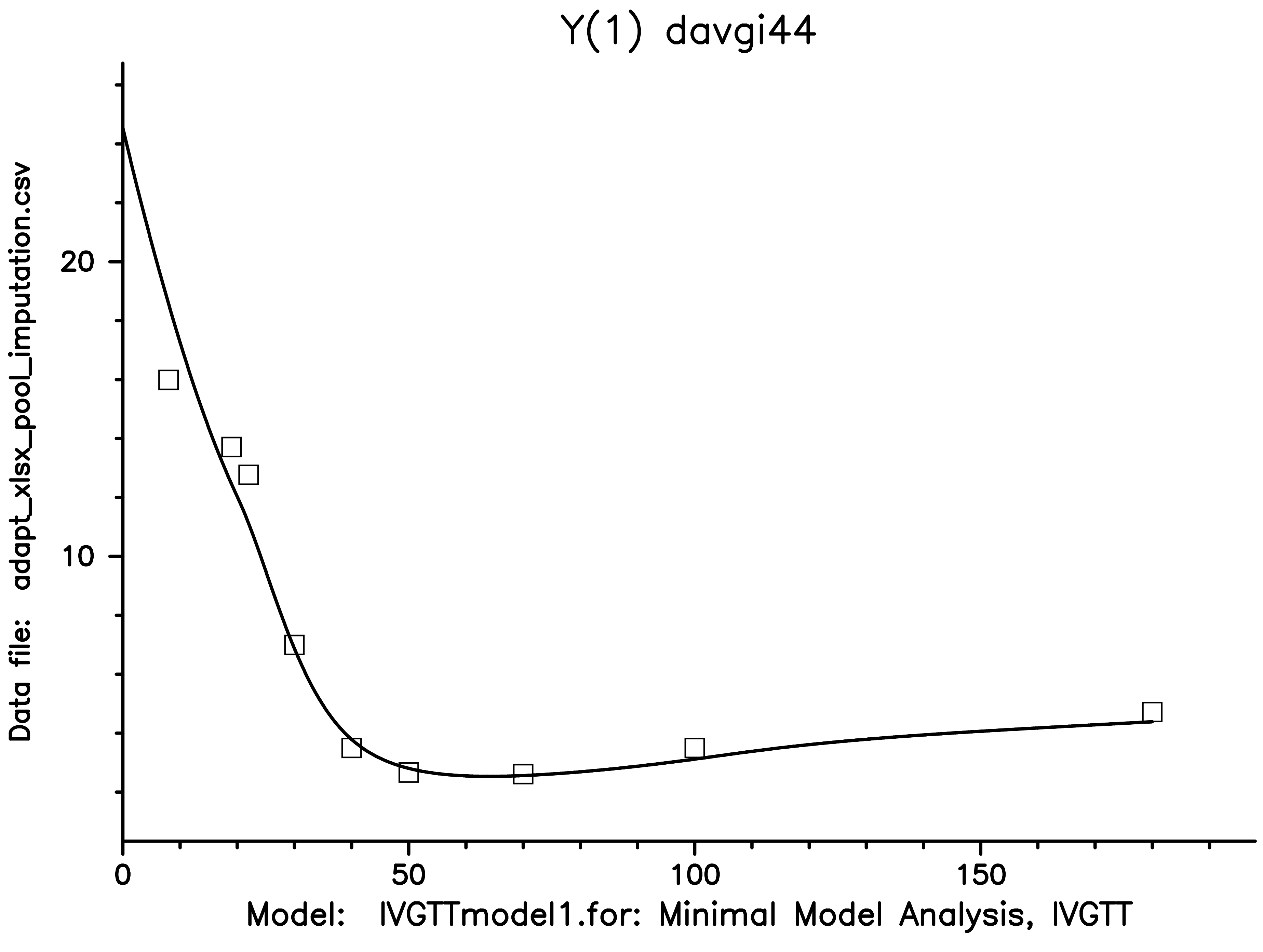
Y(1) davgi44
Data file: adapt_xlsx_pool_imputation.csv
20
10
0
50
100
150
Model: IVGTTmodel1.for: Minimal Model Analysis, IVGTT

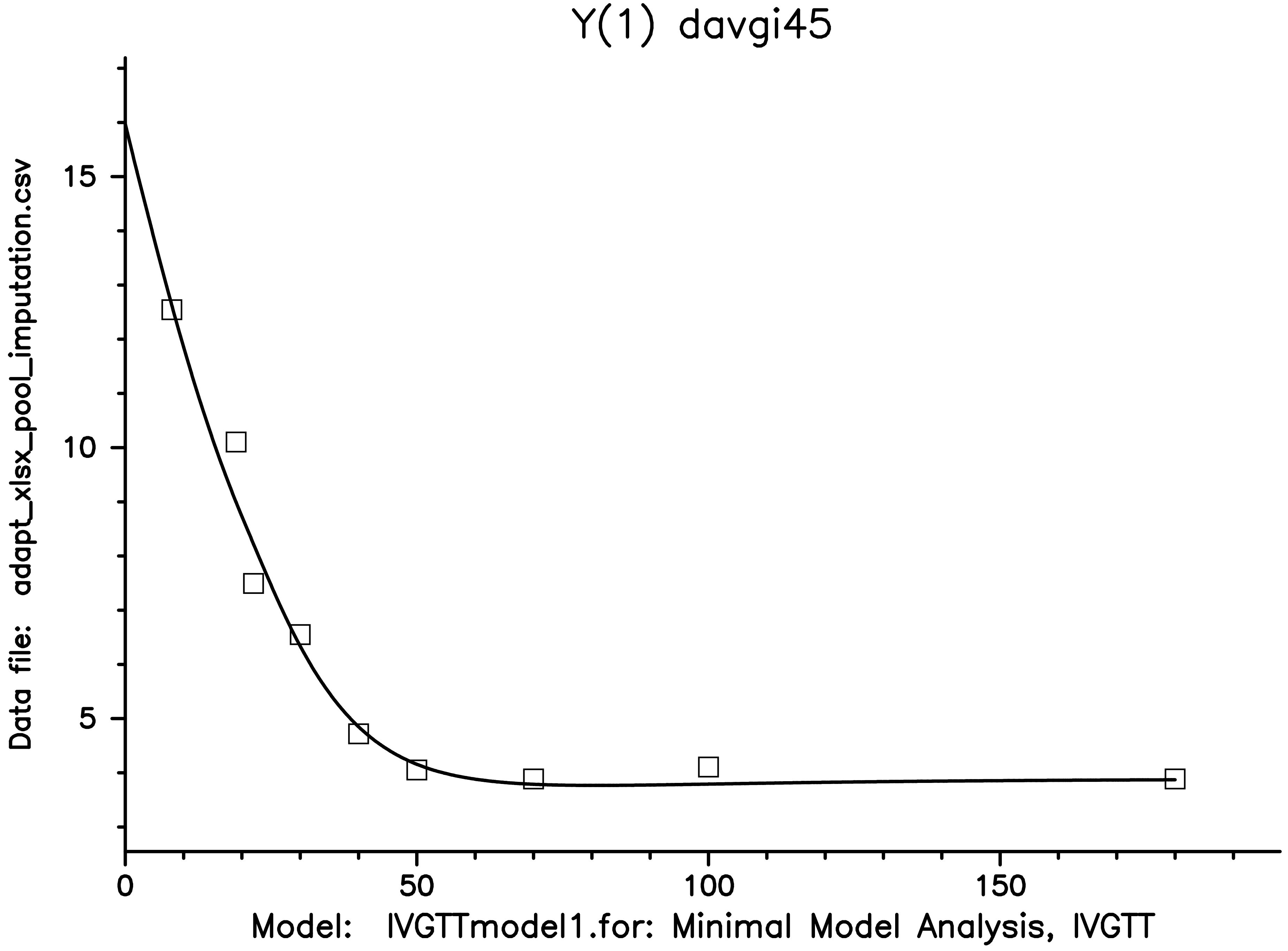
Y(1) davgi45
Data file: adapt_xlsx_pool_imputation.csv
15
10
5
0
50
100
150
Model: IVGTTmodel1.for: Minimal Model Analysis, IVGTT

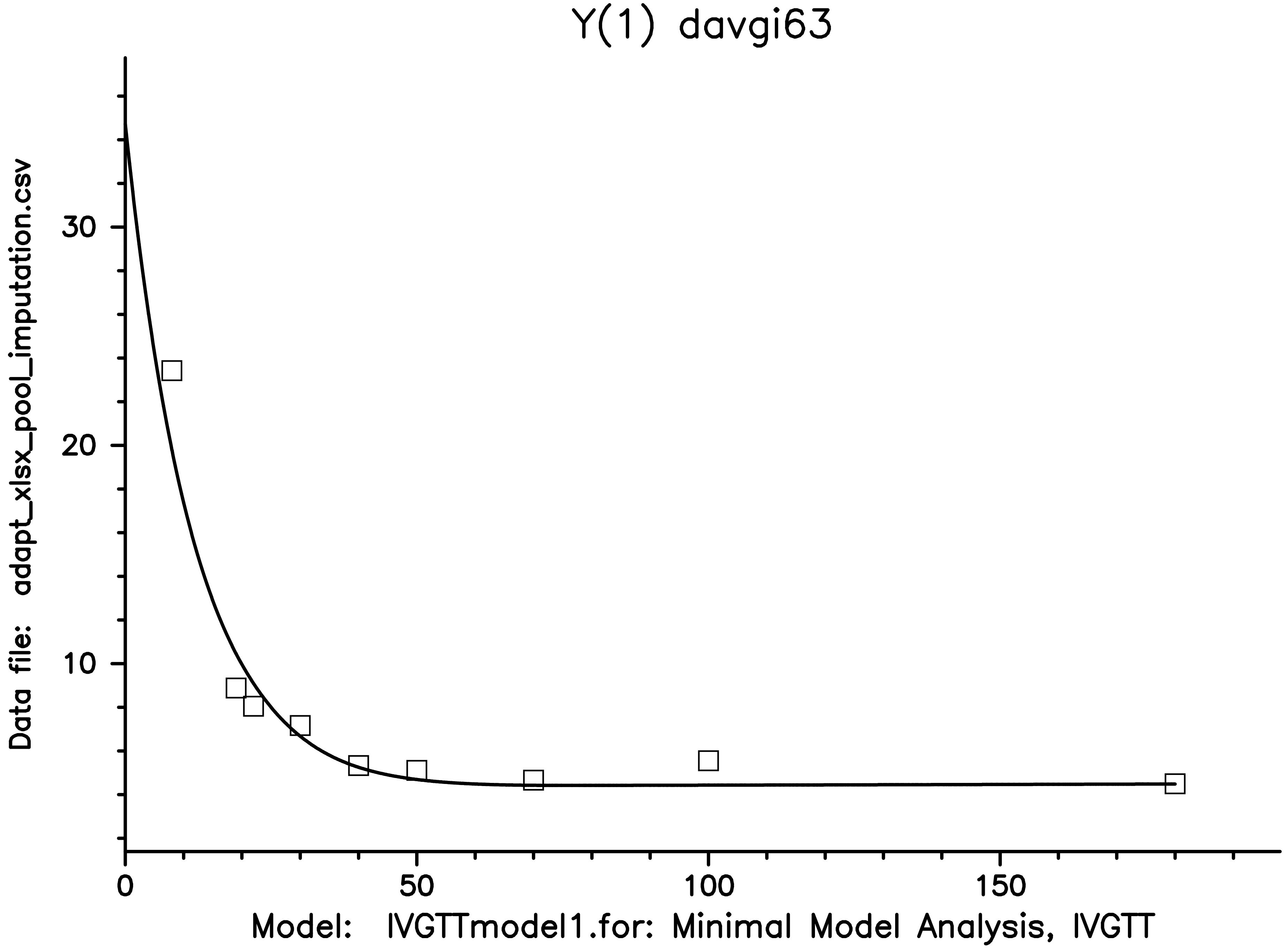
Y(1) davgi63
Data file: adapt_xlsx_pool_imputation.csv
30
20
10
0
50
100
150
Model: IVGTTmodel1.for: Minimal Model Analysis, IVGTT

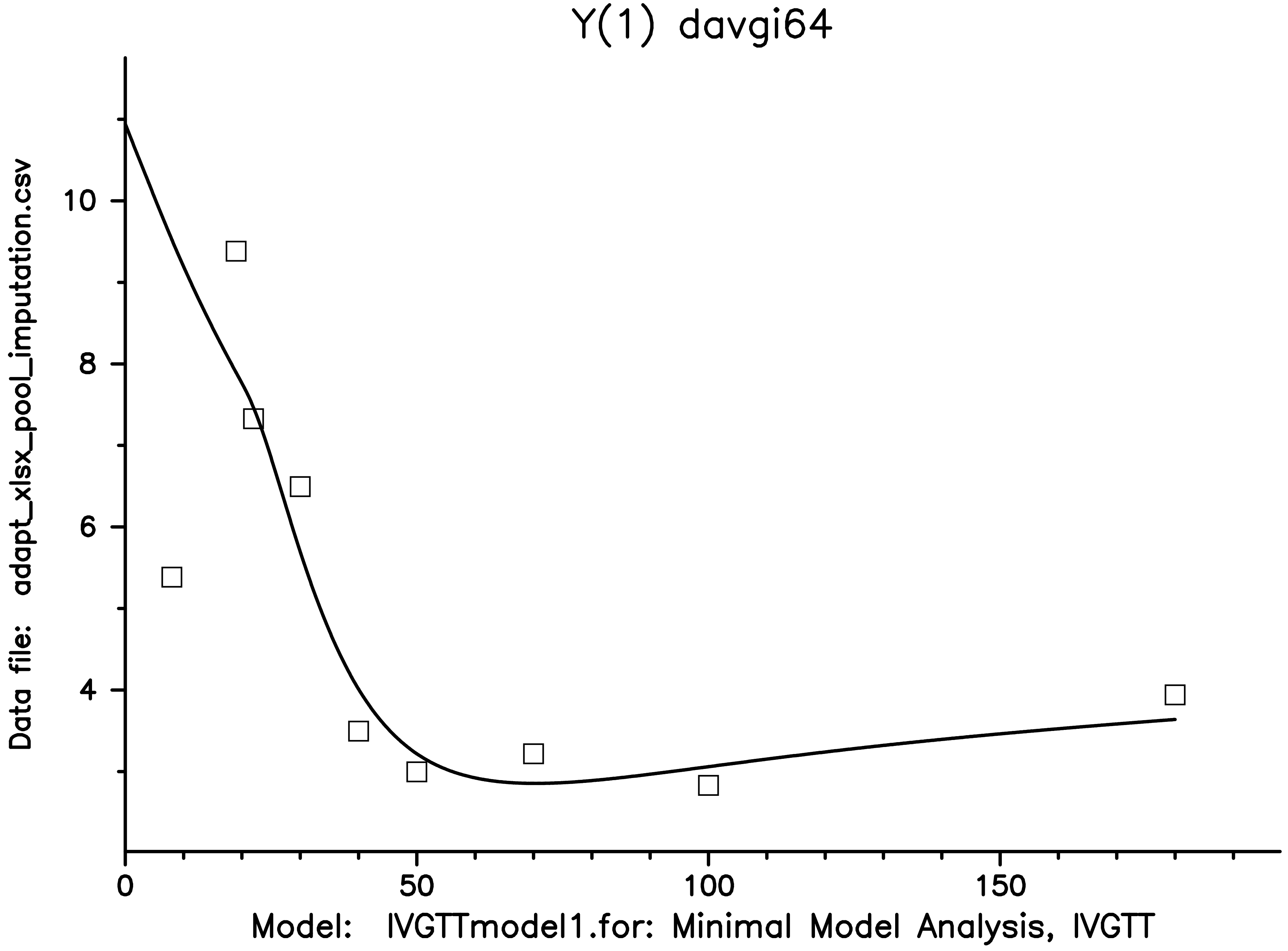
Y(1) davgi64
Data file: adapt_xlsx_pool_imputation.csv
10
8
6
4
0
50
100
150
Model: IVGTTmodel1.for: Minimal Model Analysis, IVGTT

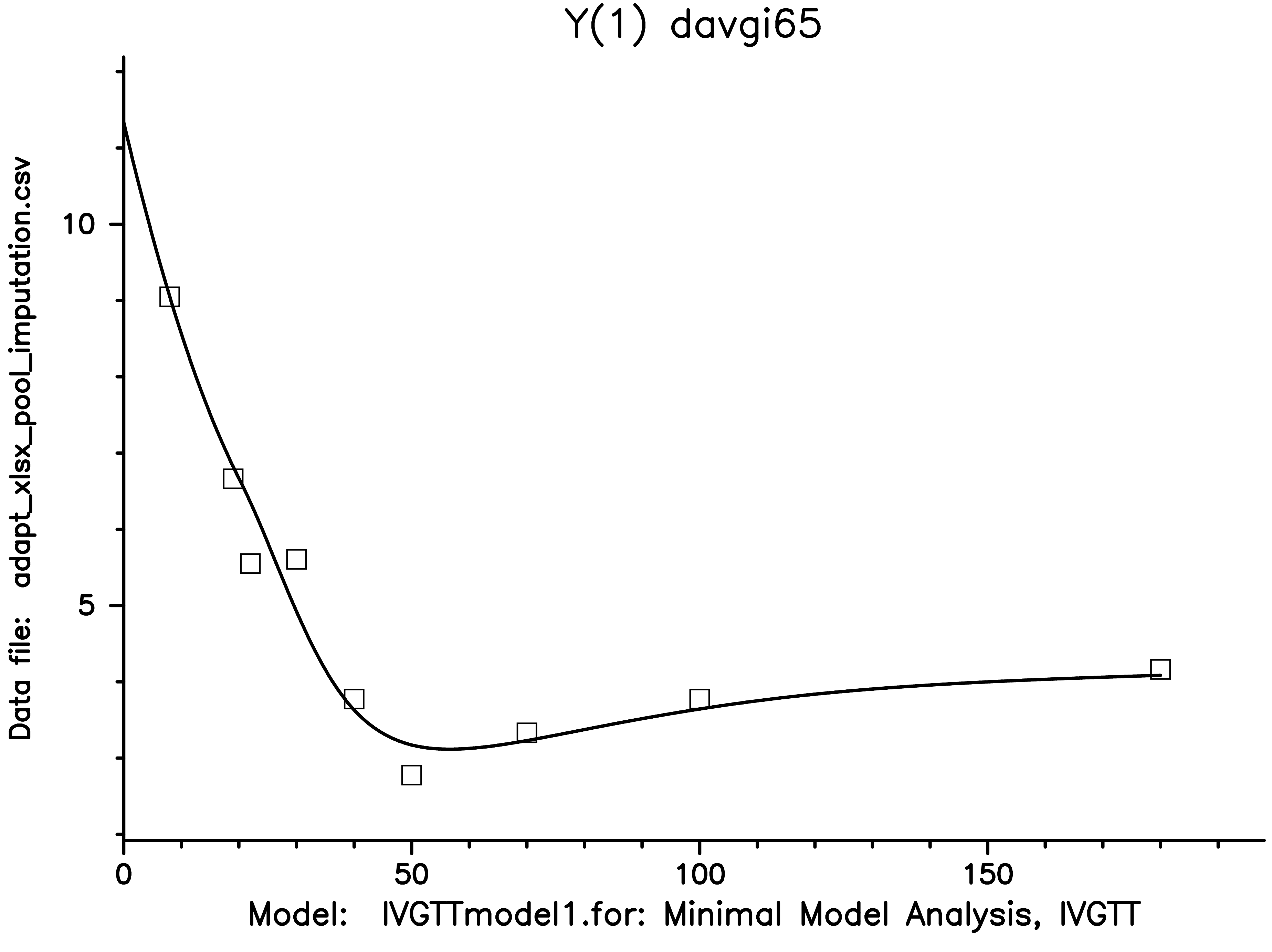
Y(1) davgi65
Data file: adapt_xlsx_pool_imputation.csv
10
5
0
50
100
150
Model: IVGTTmodel1.for: Minimal Model Analysis, IVGTT

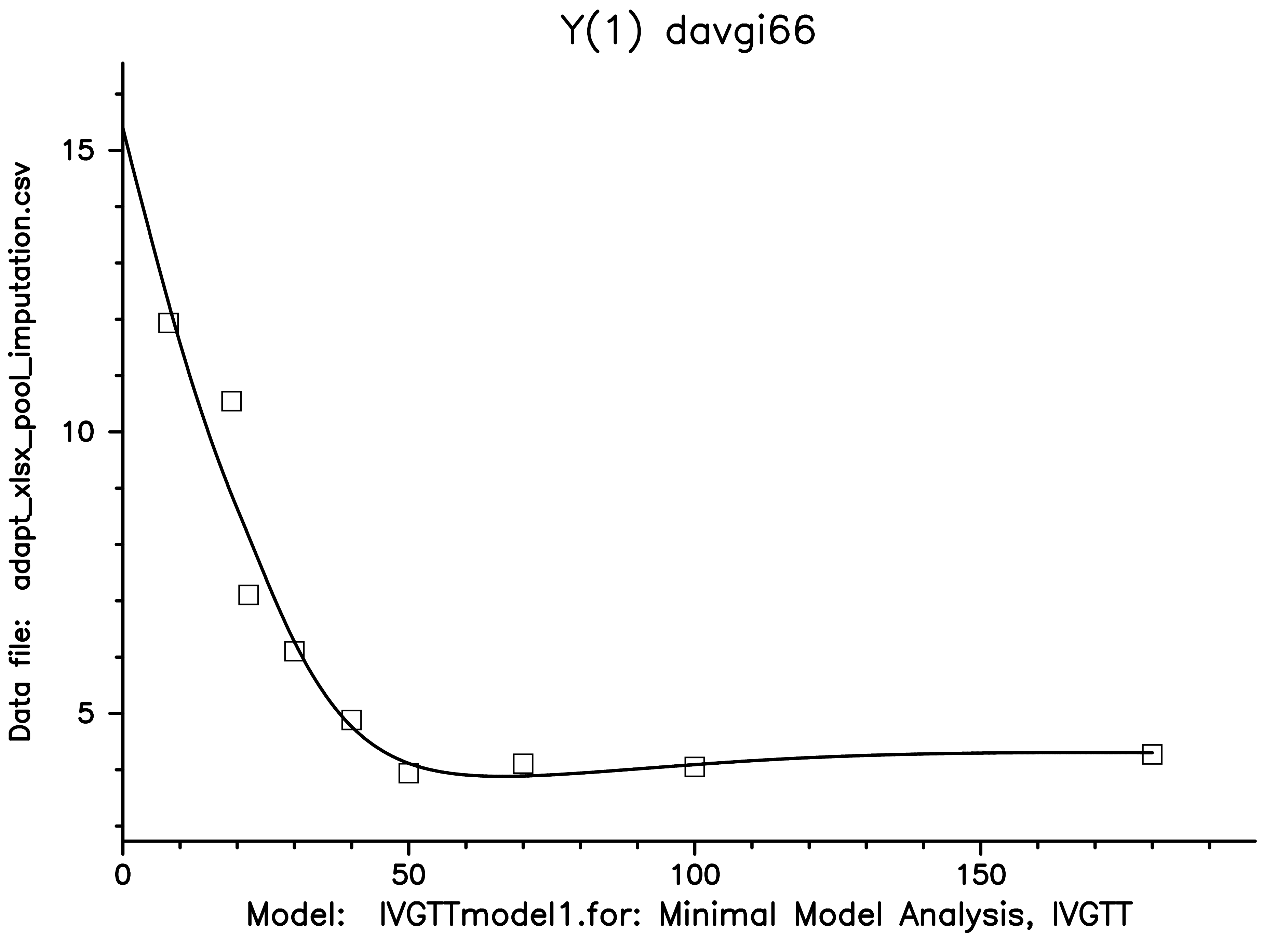
Y(1) davgi66
Data file: adapt_xlsx_pool_imputation.csv
15
10
5
0
50
100
150
Model: IVGTTmodel1.for: Minimal Model Analysis, IVGTT

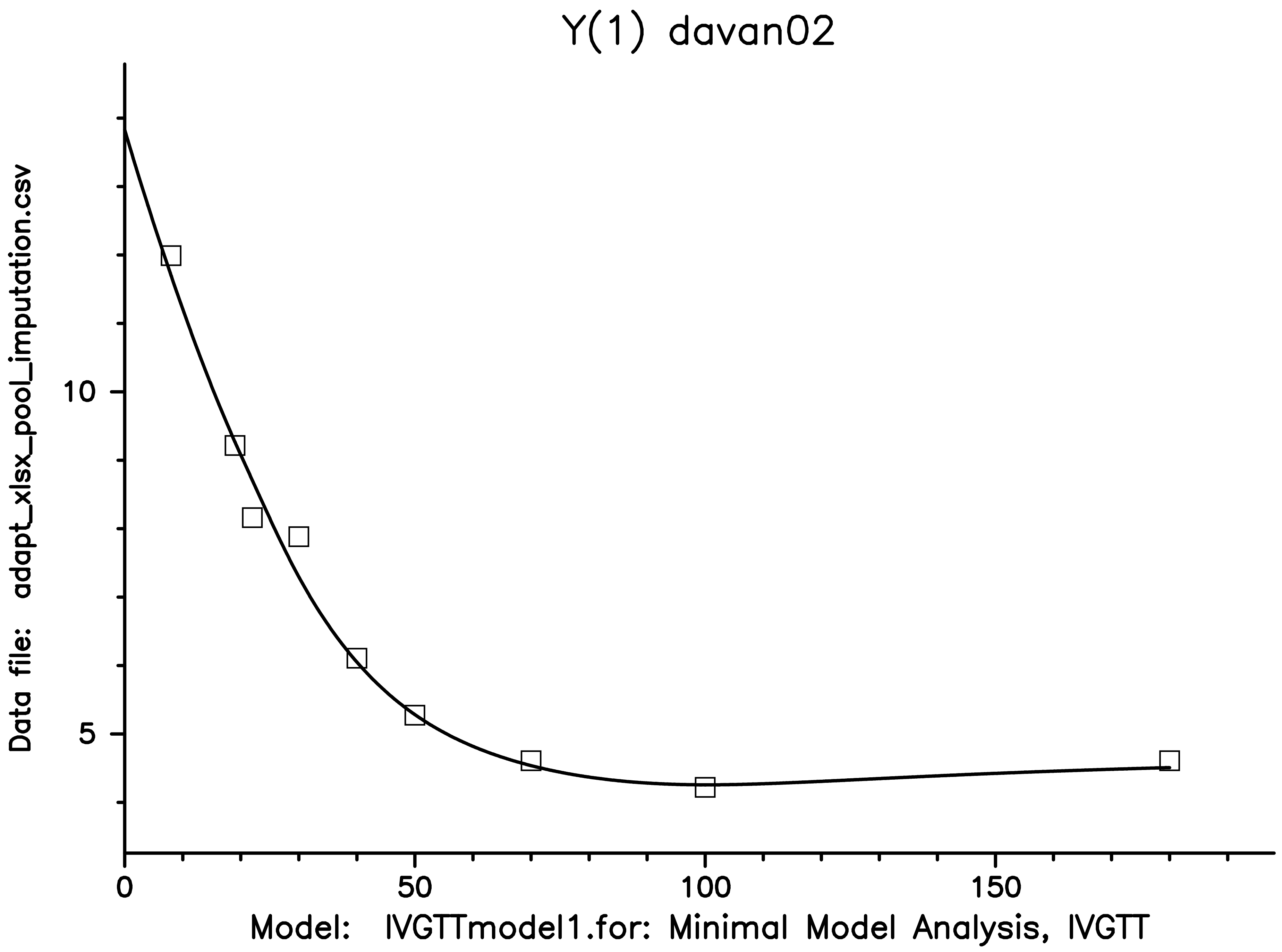
Y(1) davan02
Data file: adapt_xlsx_pool_imputation.csv
10
5
0
50
100
150
Model: IVGTTmodel1.for: Minimal Model Analysis, IVGTT

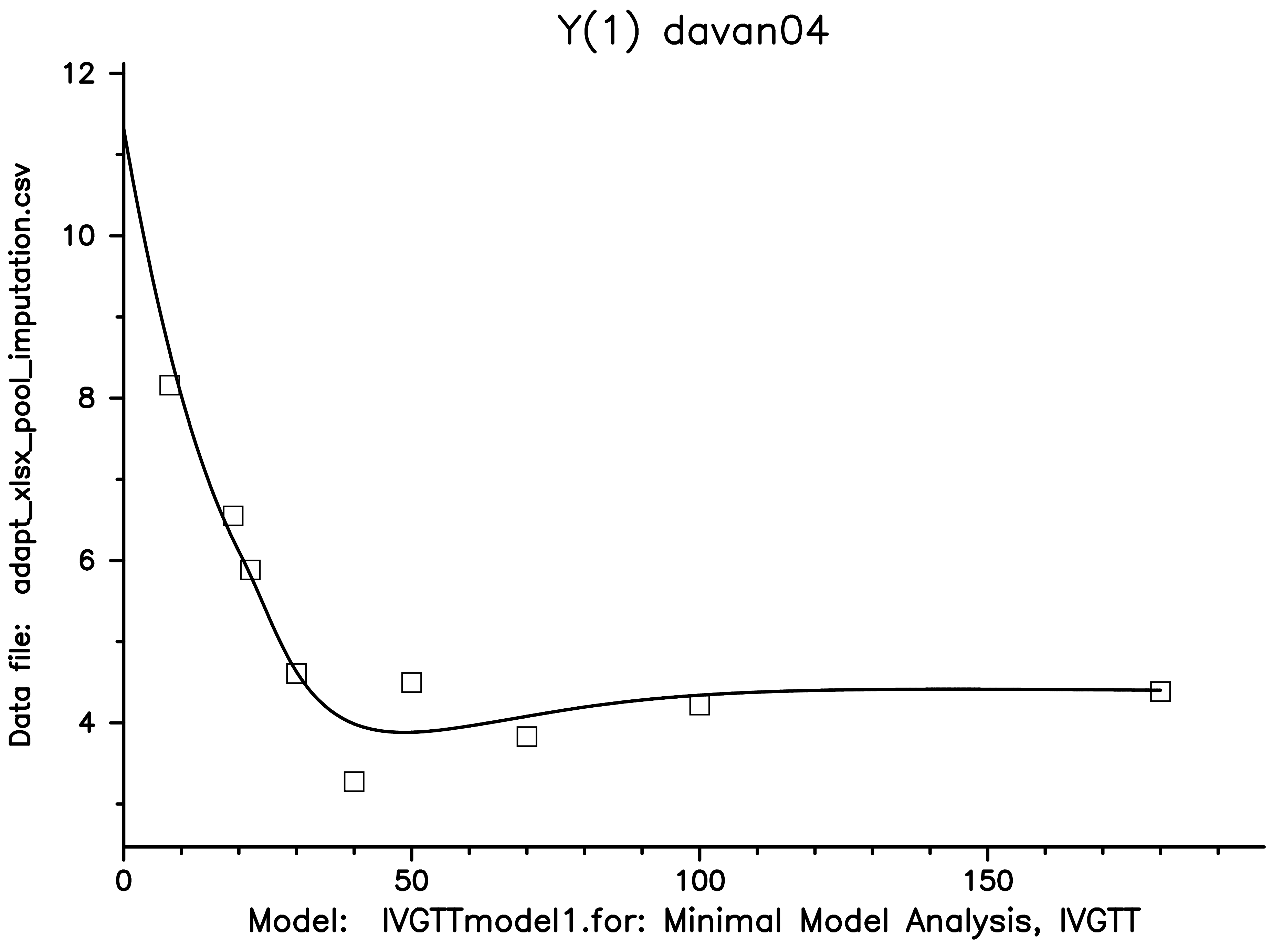
Y(1) davan04
Data file: adapt_xlsx_pool_imputation.csv
12
10
8
6
4
0
50
100
150
Model: IVGTTmodel1.for: Minimal Model Analysis, IVGTT

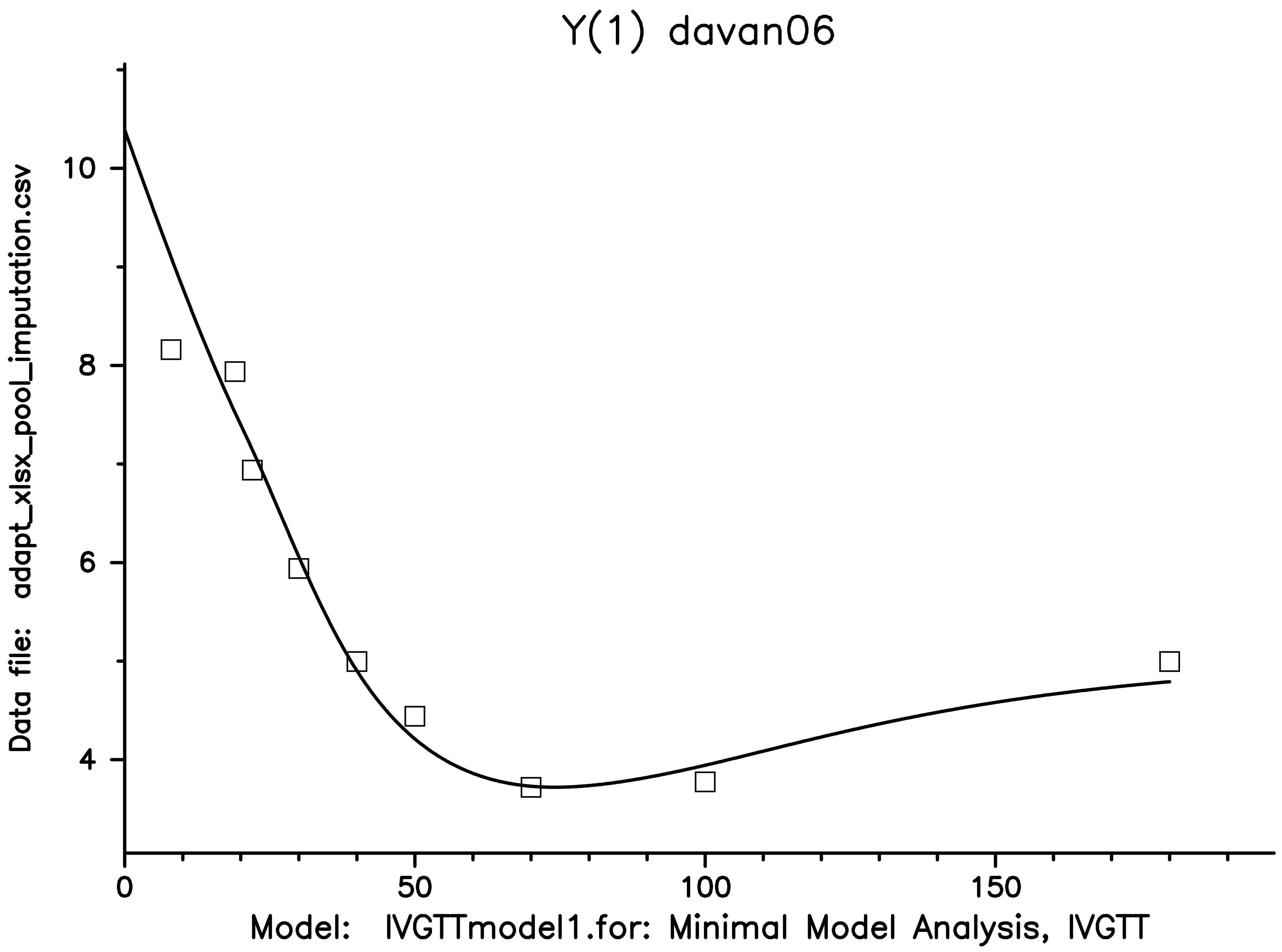
Y(1) davan06
10
8
6
4
0
50
100
150
Data file: adapt_xlsx_pool_imputation.csv
Model: IVGTTmodel1.for: Minimal Model Analysis, IVGTT

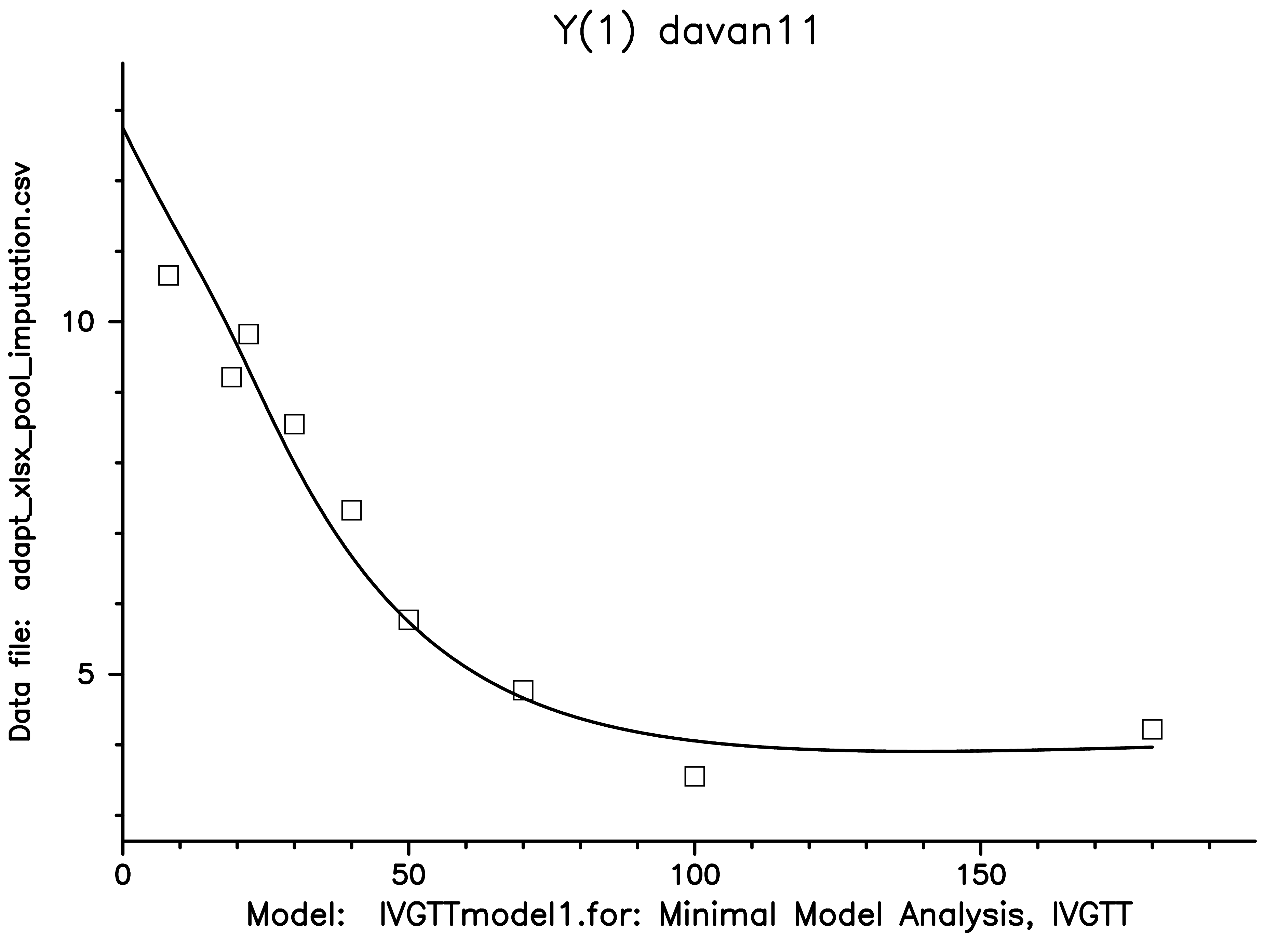

Y(1) davan11
Data file: adapt_xlsx_pool_imputation.csv
10
5
0
50
100
150
Model: IVGTTmodel1.for: Minimal Model Analysis, IVGTT

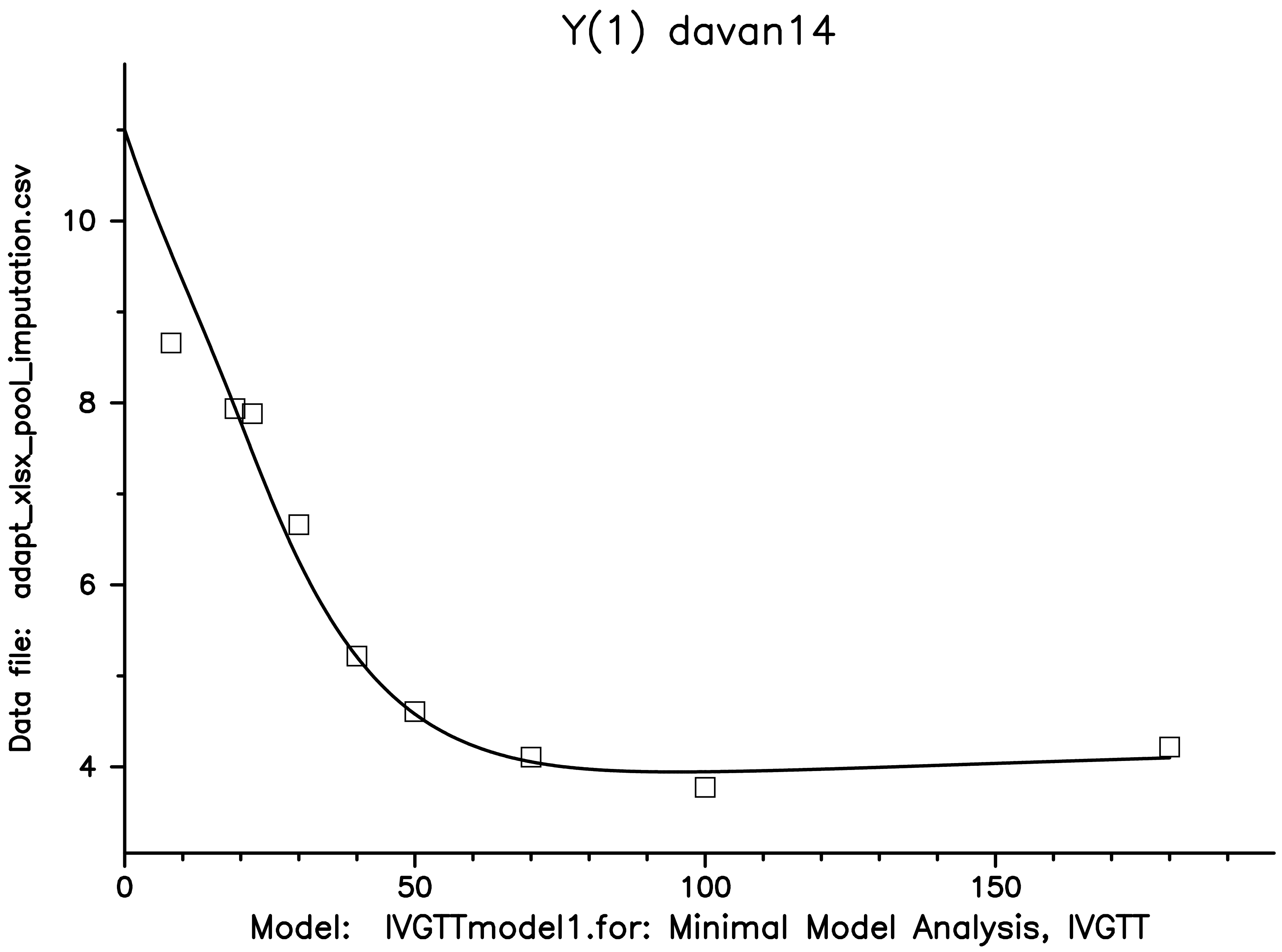
Y(1) davan14
Data file: adapt_xlsx_pool_imputation.csv
10
8
6
4
0
50
100
150
Model: IVGTTmodel1.for: Minimal Model Analysis, IVGTT

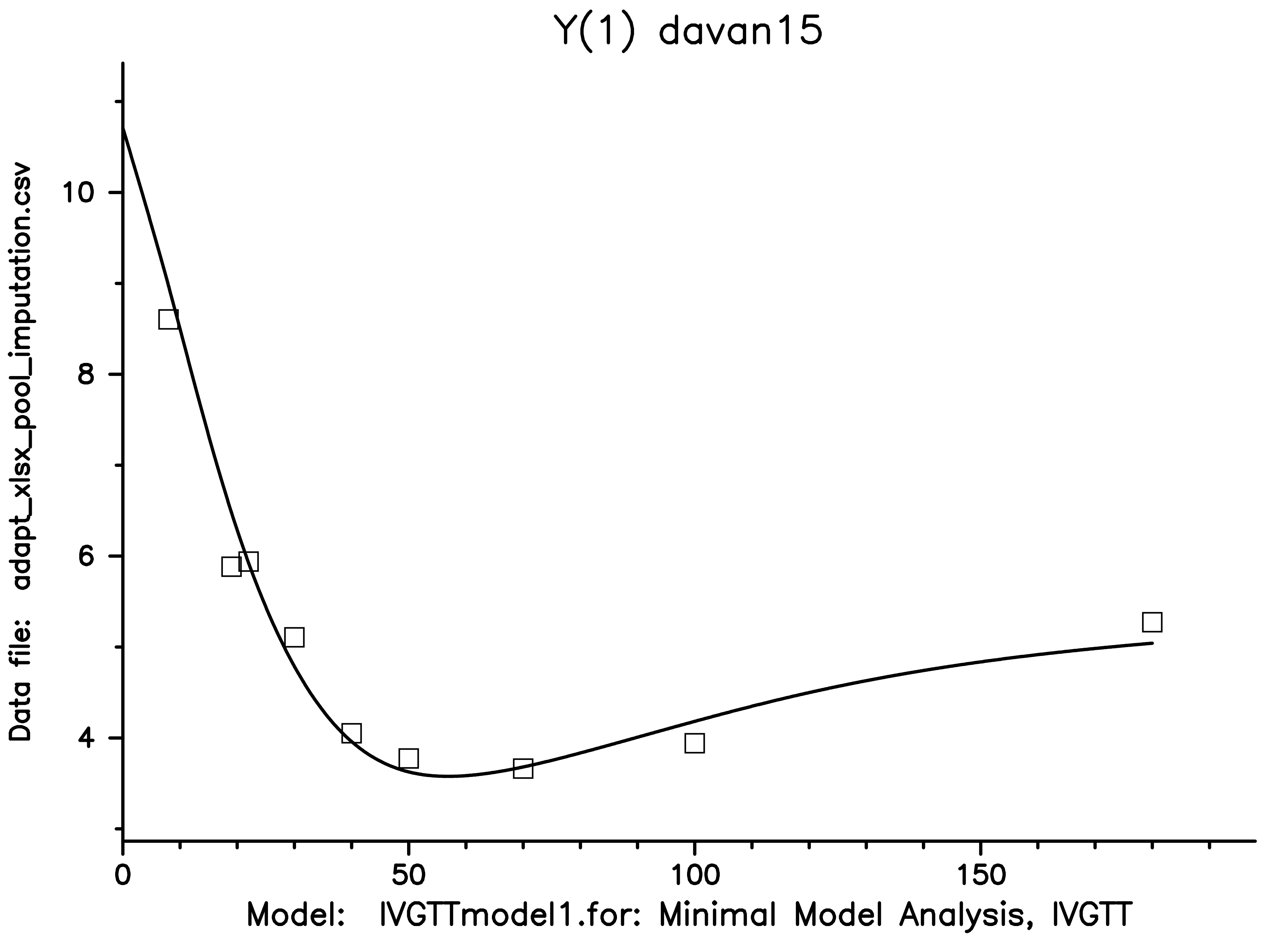
Y(1) davan15
Data file: adapt_xlsx_pool_imputation.csv
10
8
6
4
0
50
100
150
Model: IVGTTmodel1.for: Minimal Model Analysis, IVGTT

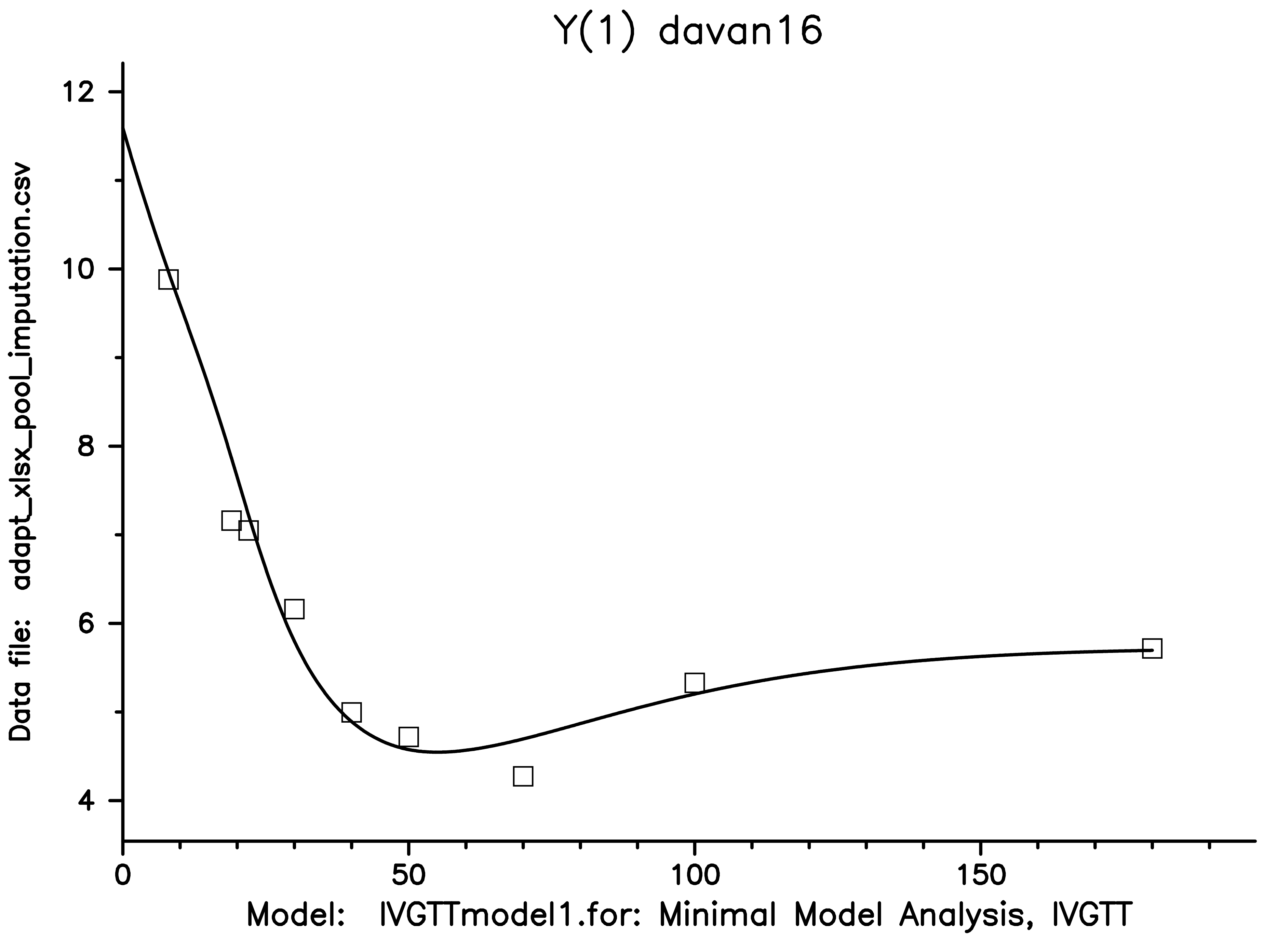
Y(1) davan16
Data file: adapt_xlsx_pool_imputation.csv
12
10
8
6
4
0
50
100
150
Model: IVGTTmodel1.for: Minimal Model Analysis, IVGTT

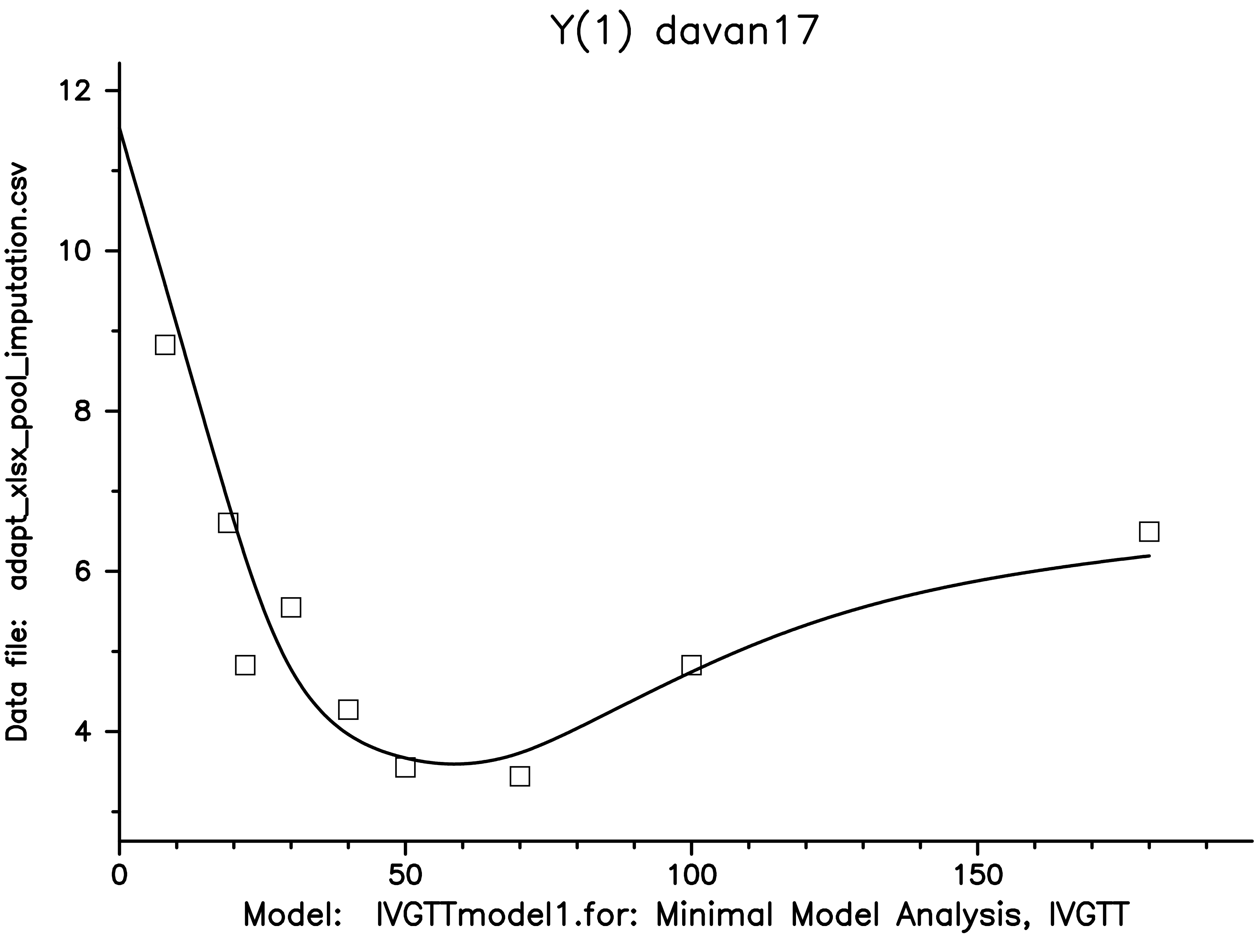

Y(1) davan17
Data file: adapt_xlsx_pool_imputation.csv
12
10
8
6
4
0
50
100
150
Model: IVGTTmodel1.for: Minimal Model Analysis, IVGTT

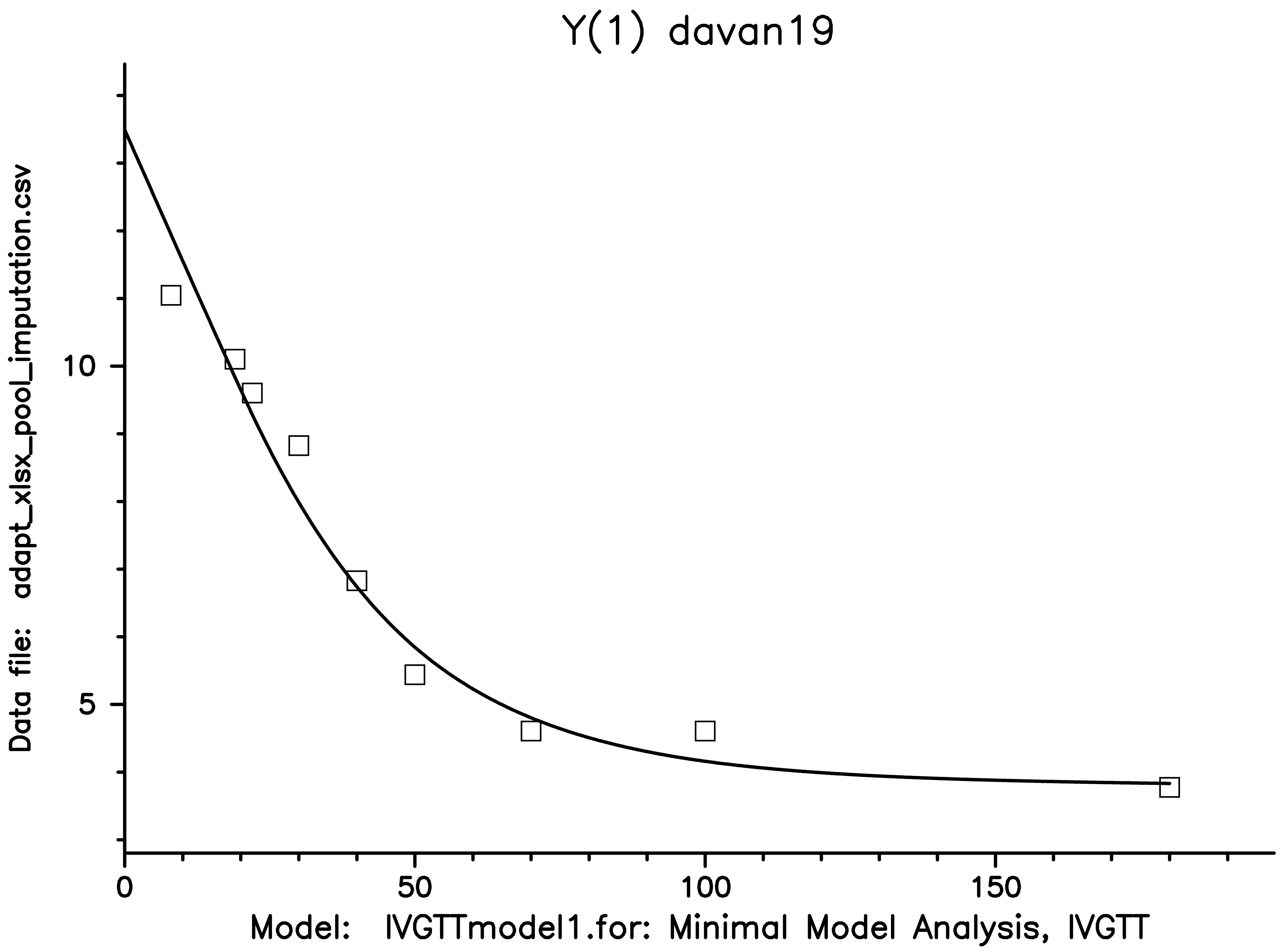
Y(1) davan19
Data file: adapt_xlsx_pool_imputation.csv
10
5
0
50
100
150
Model: IVGTTmodel1.for: Minimal Model Analysis, IVGTT

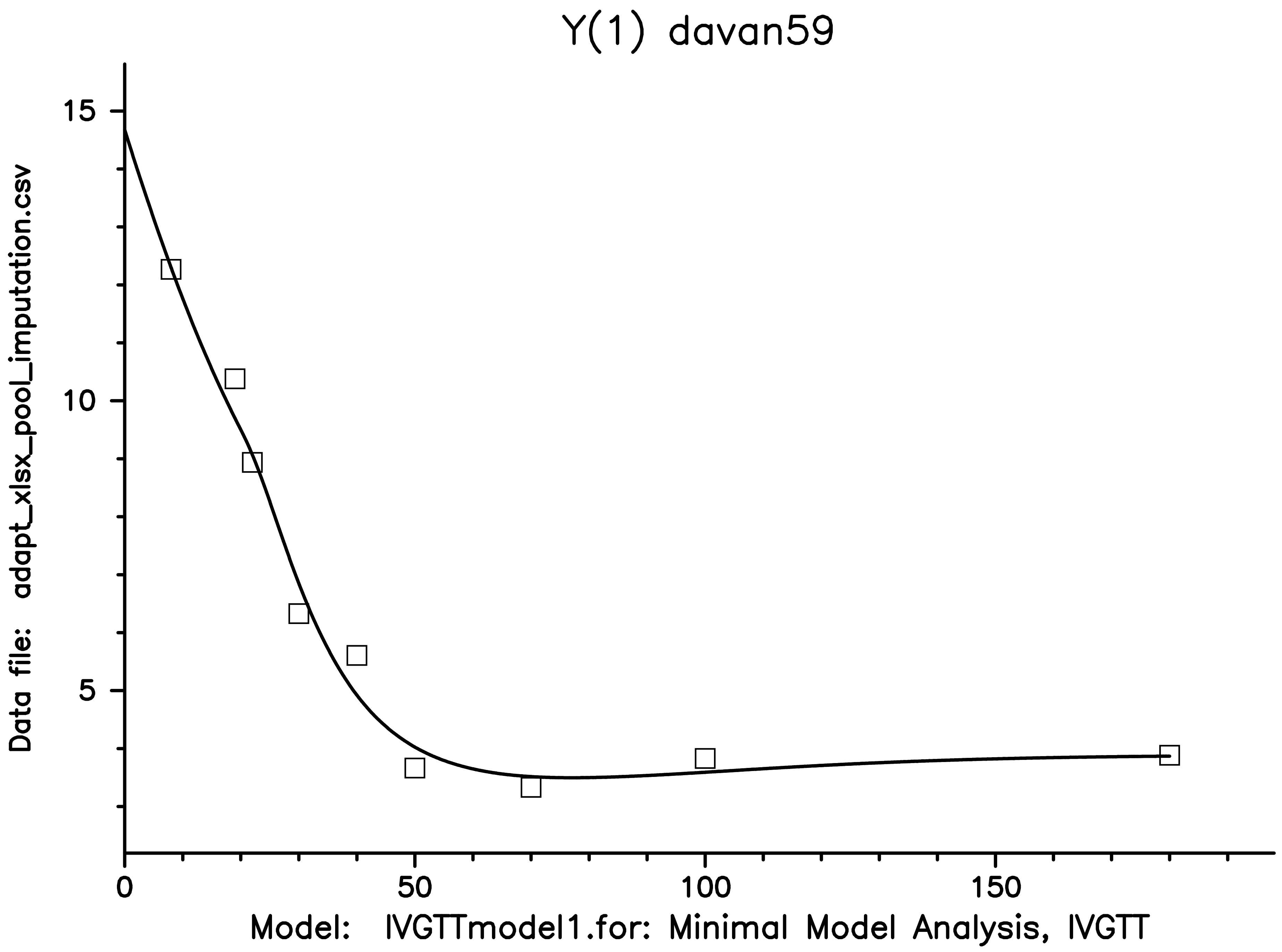
Y(1) davan59
Data file: adapt_xlsx_pool_imputation.csv
15
10
5
0
50
100
150
Model: IVGTTmodel1.for: Minimal Model Analysis, IVGTT

Y(1) davan60

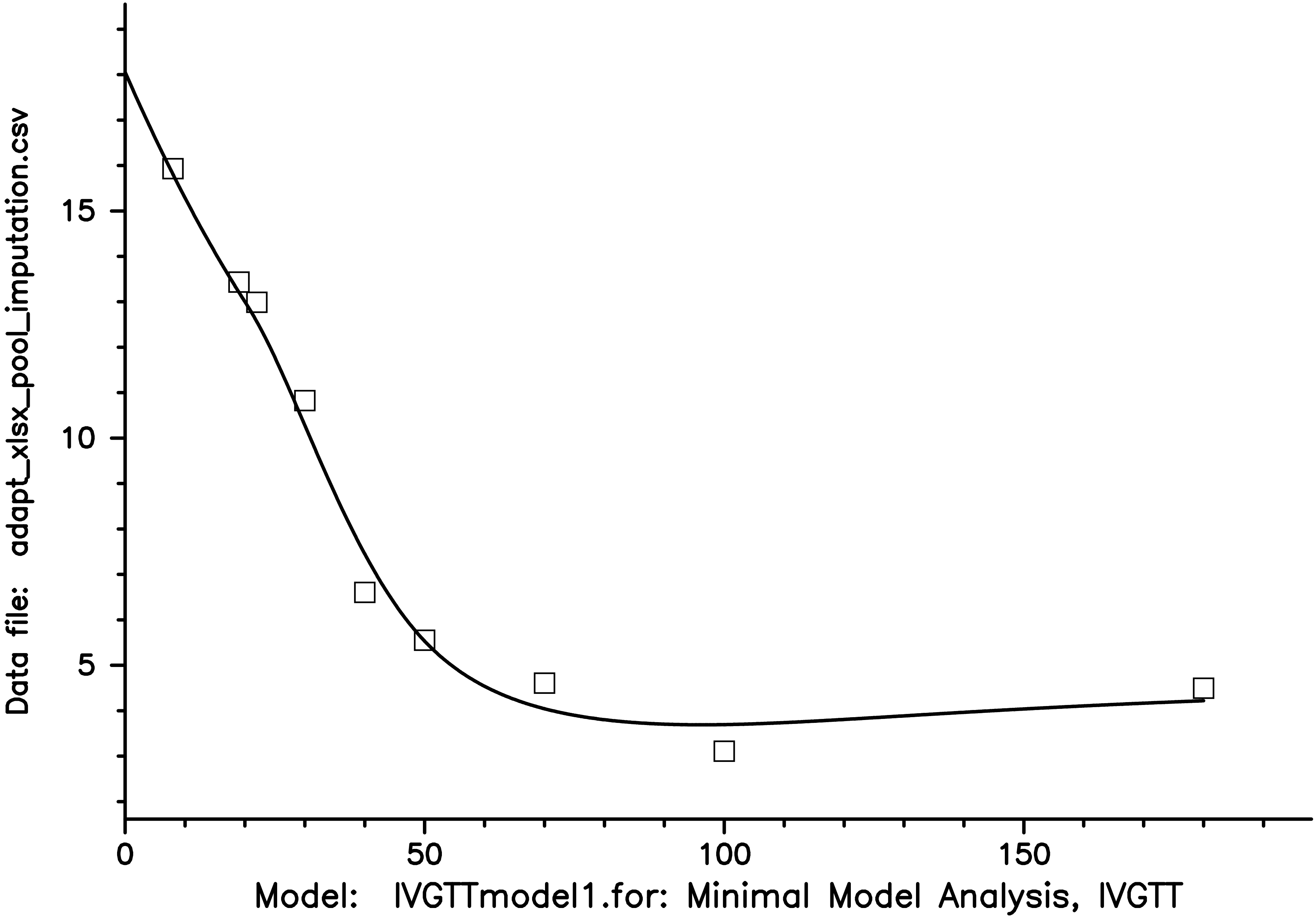

Data file: adapt_xlsx_pool_imputation.csv
15
10
5
0
50
100
150
Model: IVGTTmodel1.for: Minimal Model Analysis, IVGTT

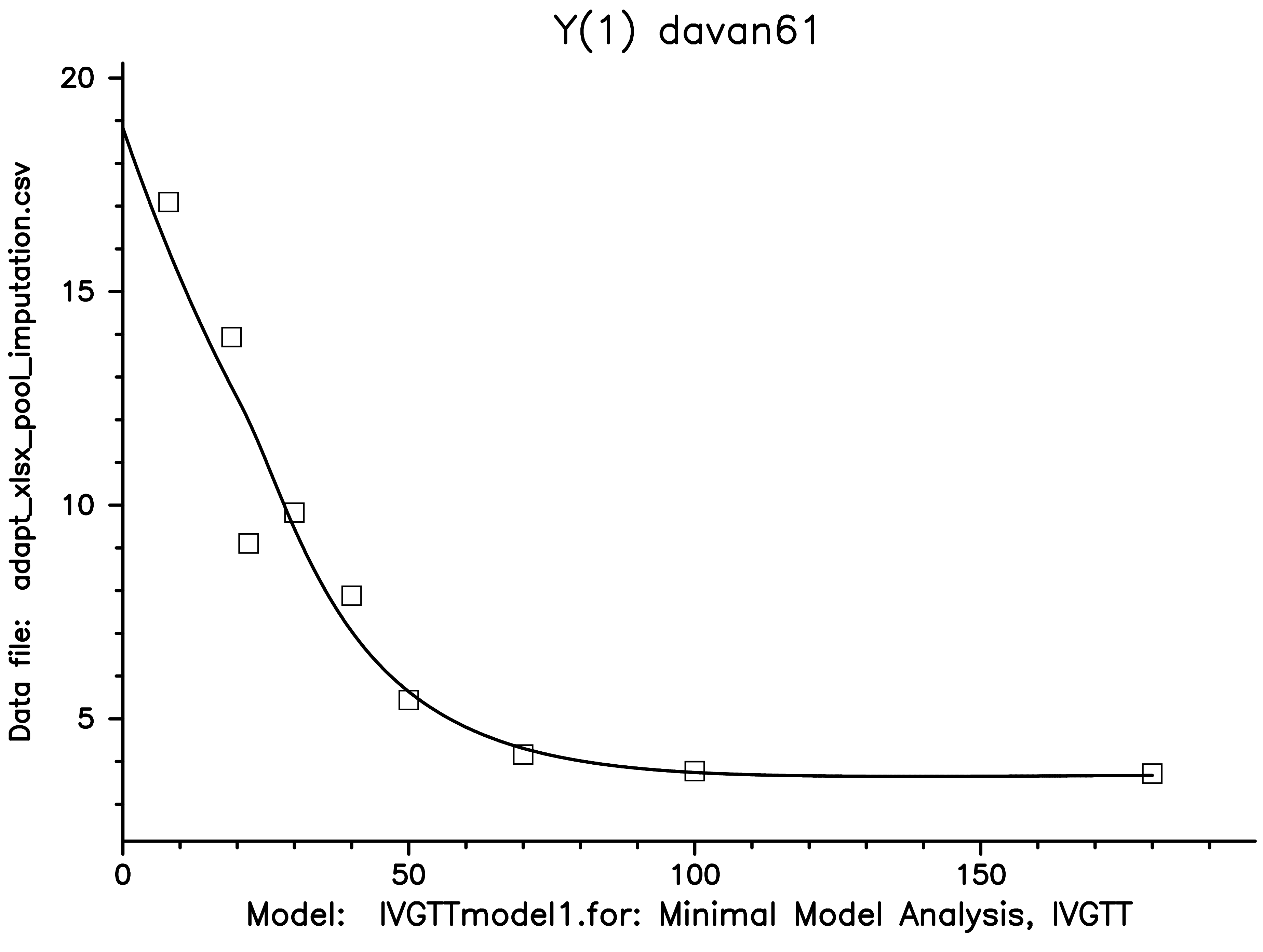
Y(1) davan61
Data file: adapt_xlsx_pool_imputation.csv
Model: IVGTTmodel1.for: Minimal Model Analysis, IVGTT
20
15
10
5
0
50
100
150

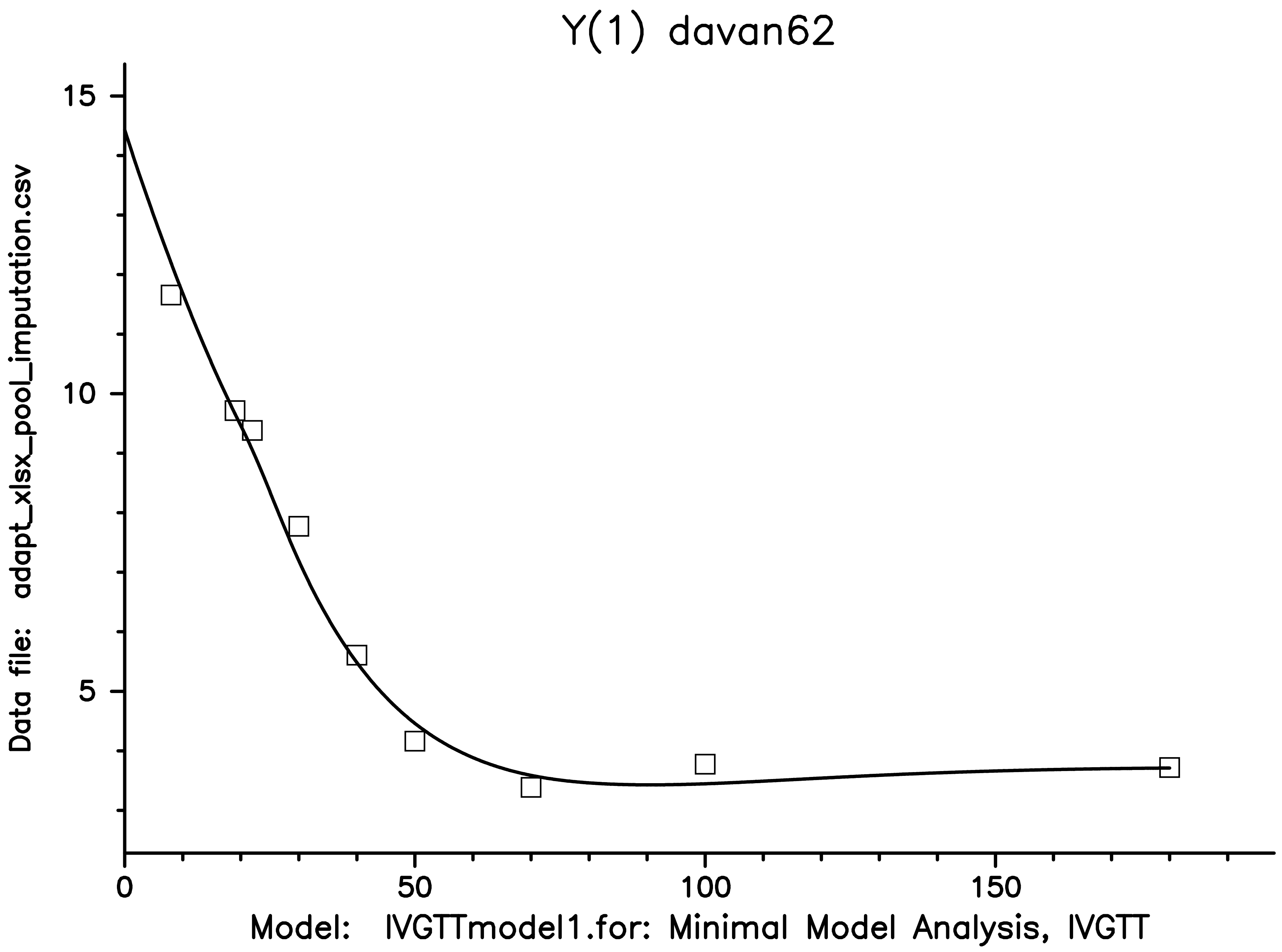
Y(1) davan62
Data file: adapt_xlsx_pool_imputation.csv
15
10
5
0
50
100
150
Model: IVGTTmodel1.for: Minimal Model Analysis, IVGTT

Y(1) davan01

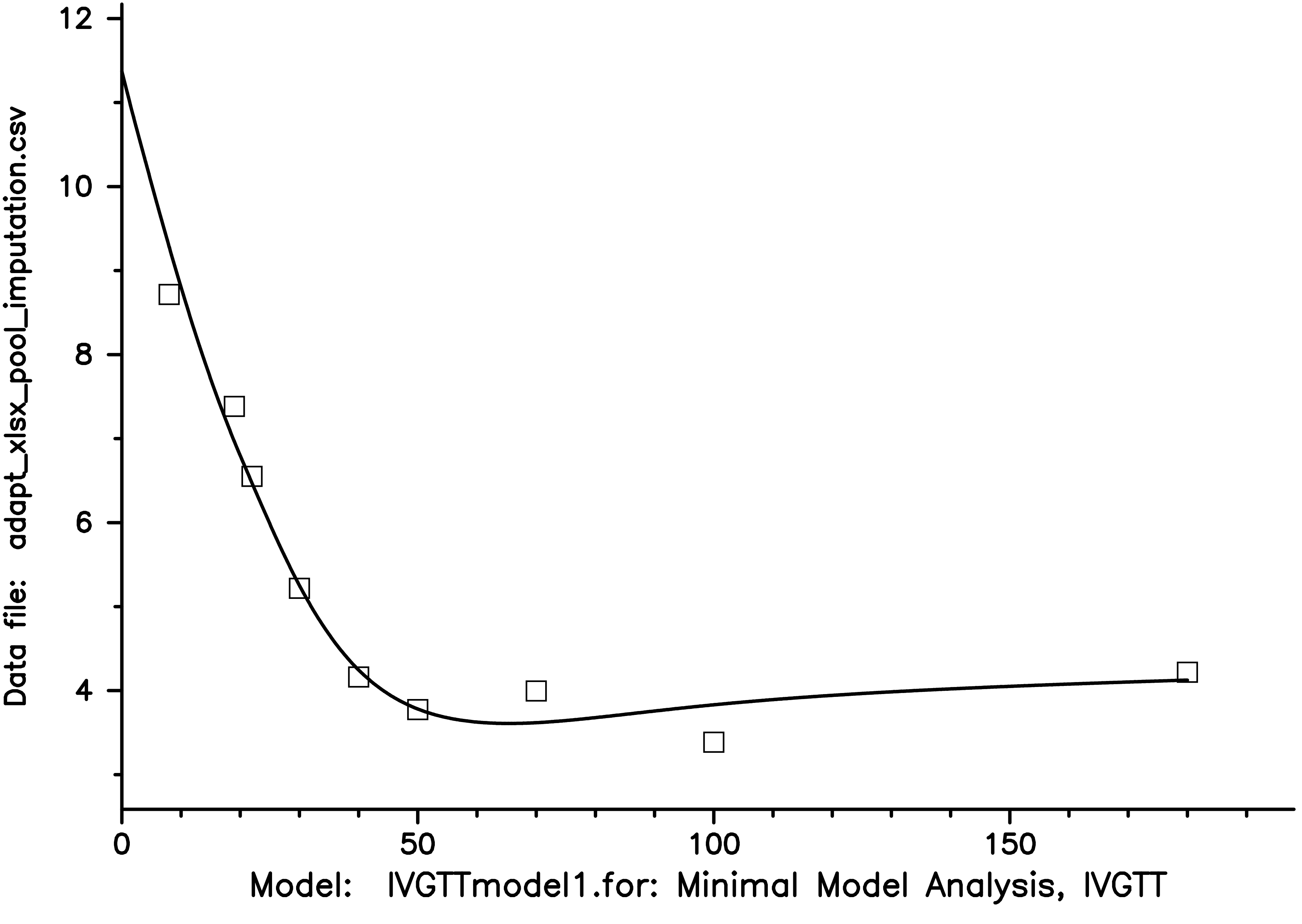

12
10
8
6
4
0
50
100
150
Data file: adapt_xlsx_pool_imputation.csv
Model: IVGTTmodel1.for: Minimal Model Analysis, IVGTT

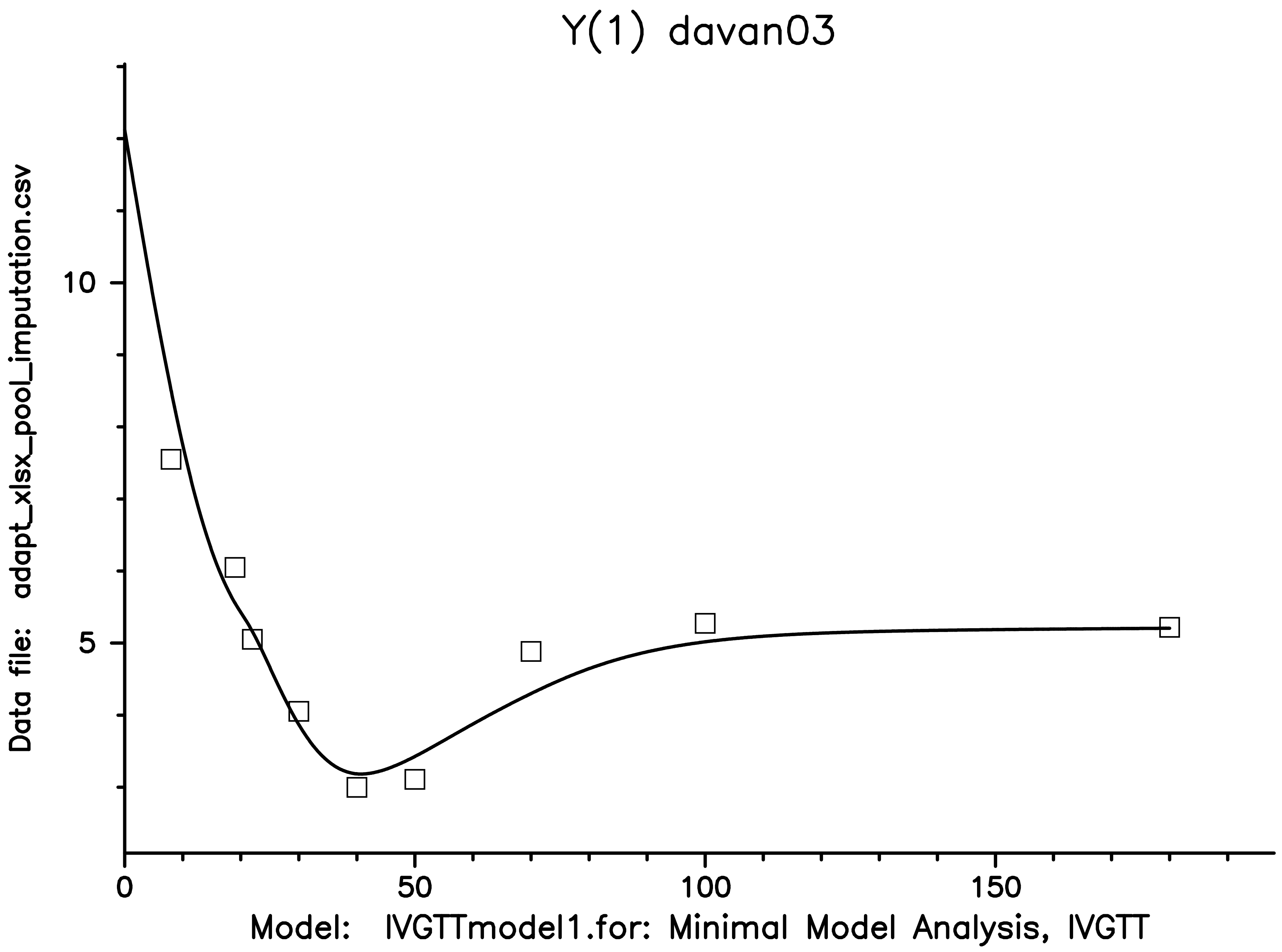
Y(1) davan03
Data file: adapt_xlsx_pool_imputation.csv
10
5
0
50
100
150
Model: IVGTTmodel1.for: Minimal Model Analysis, IVGTT

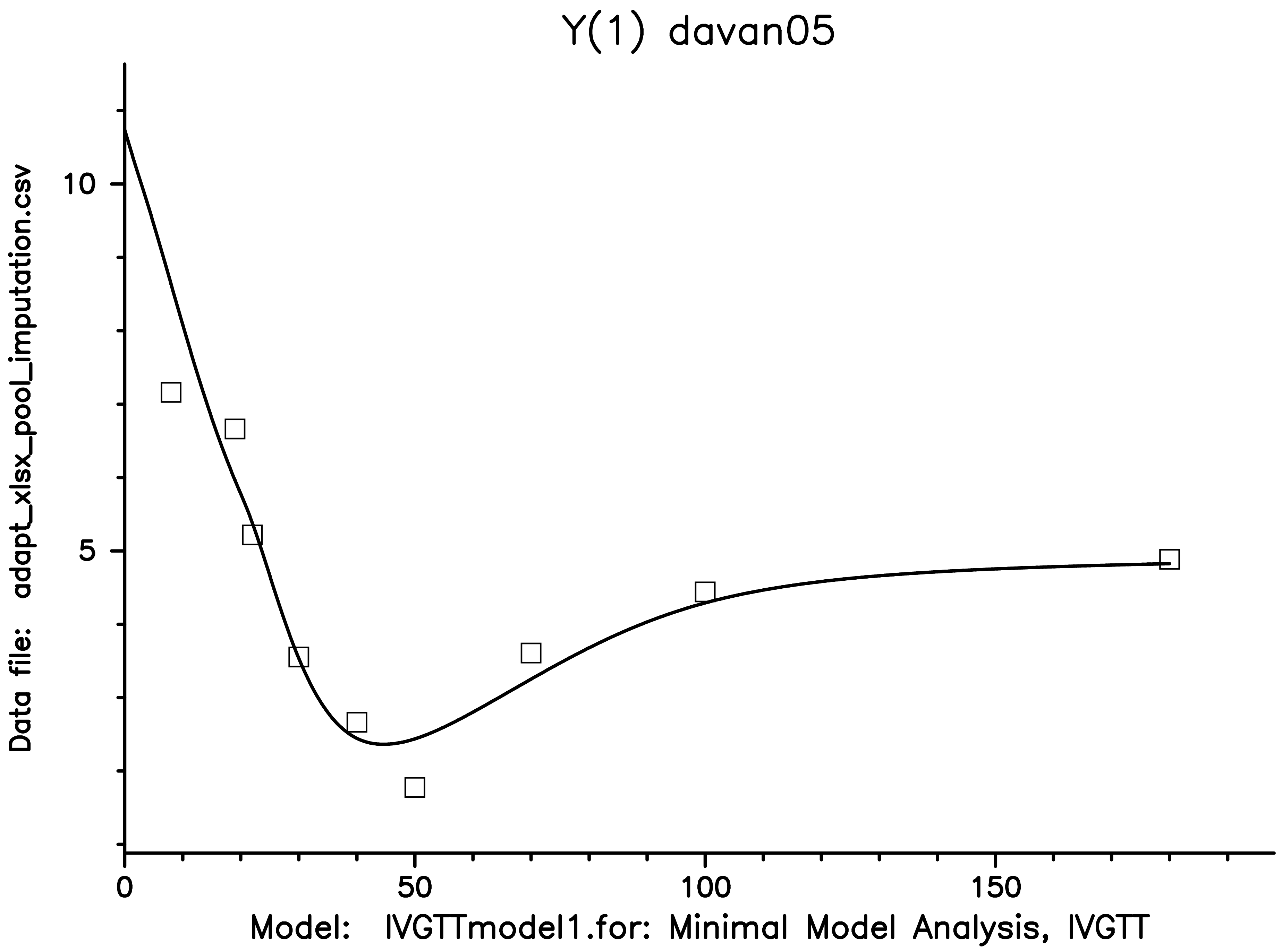
Y(1) davan05
Data file: adapt_xlsx_pool_imputation.csv
10
5
0
50
100
150
Model: IVGTTmodel1.for: Minimal Model Analysis, IVGTT

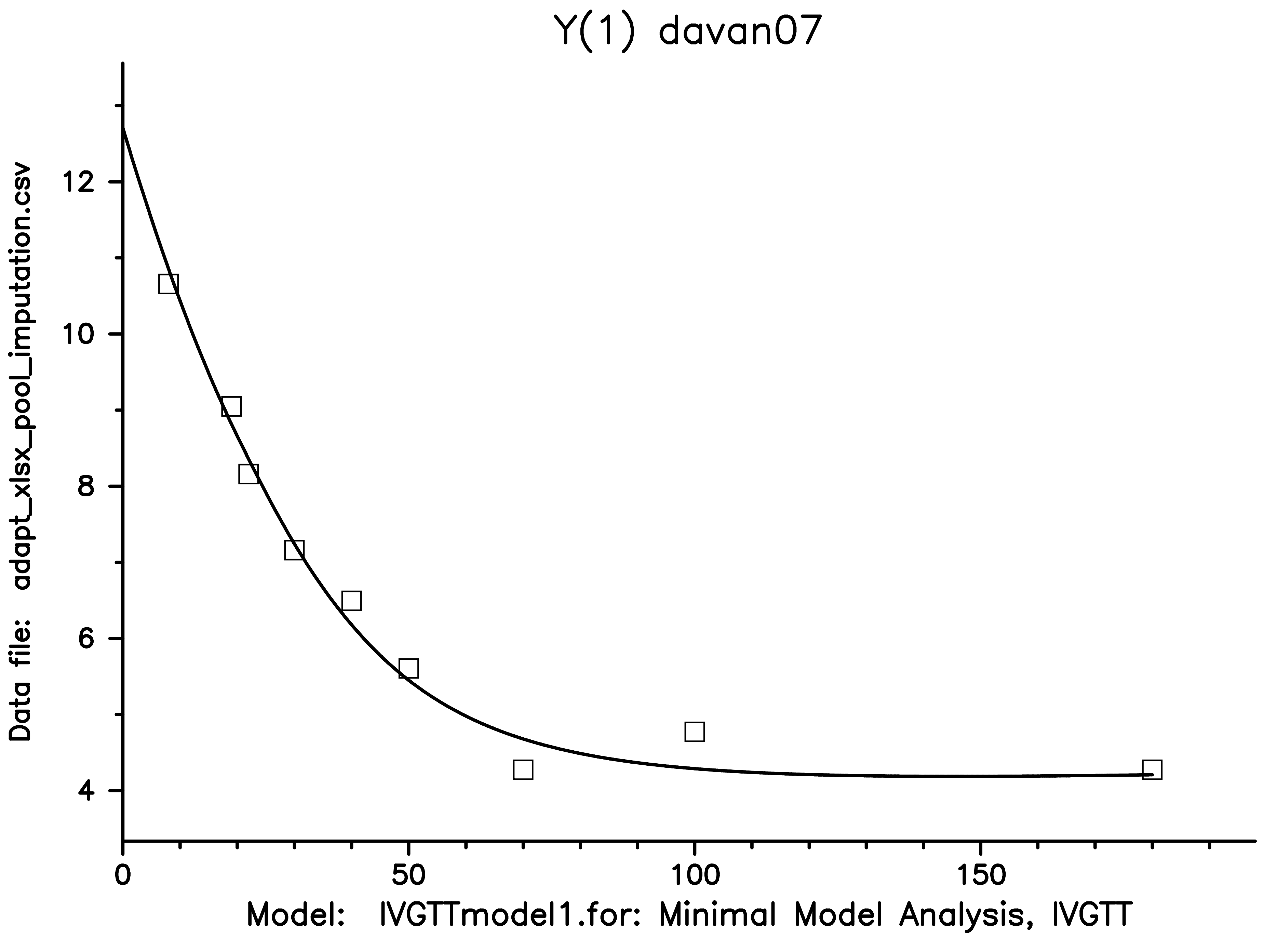
Y(1) davan07
Data file: adapt_xlsx_pool_imputation.csv
Model: IVGTTmodel1.for: Minimal Model Analysis, IVGTT
12
10
8
6
4
0
50
100
150

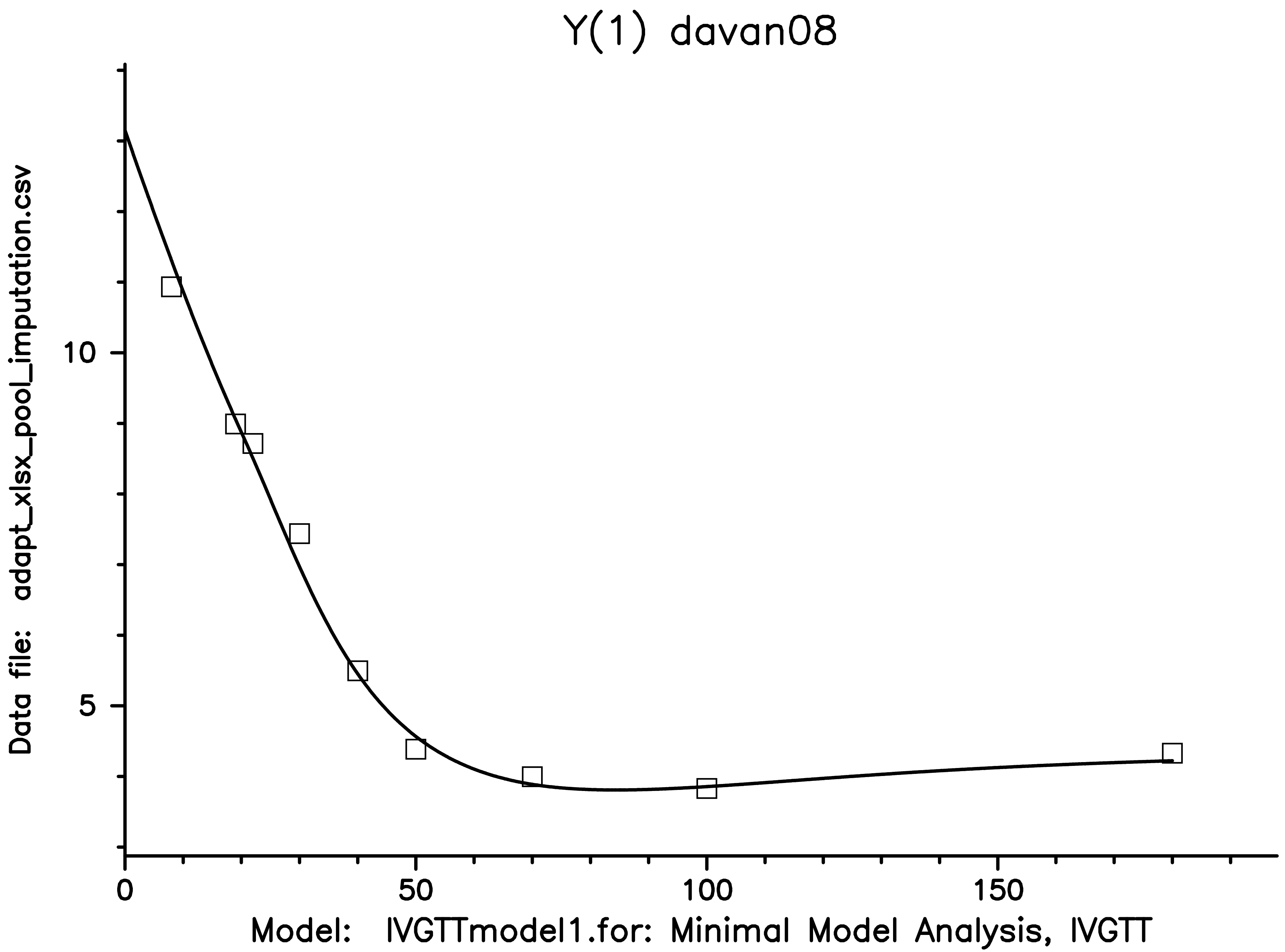

Y(1) davan08
Data file: adapt_xlsx_pool_imputation.csv
10
5
0
50
100
150
Model: IVGTTmodel1.for: Minimal Model Analysis, IVGTT

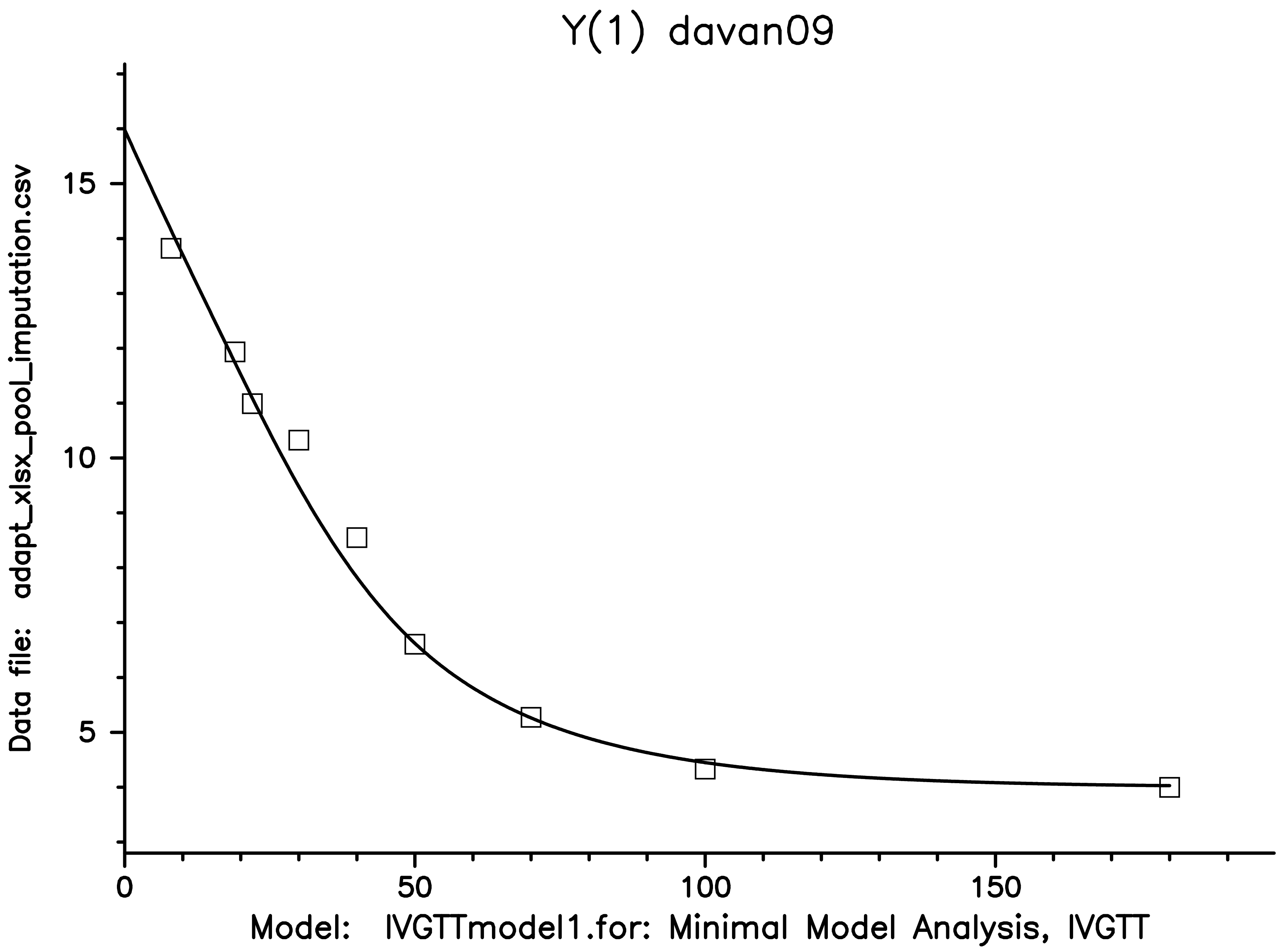

Y(1) davan09
Data file: adapt_xlsx_pool_imputation.csv
15
10
5
0
50
100
150
Model: IVGTTmodel1.for: Minimal Model Analysis, IVGTT

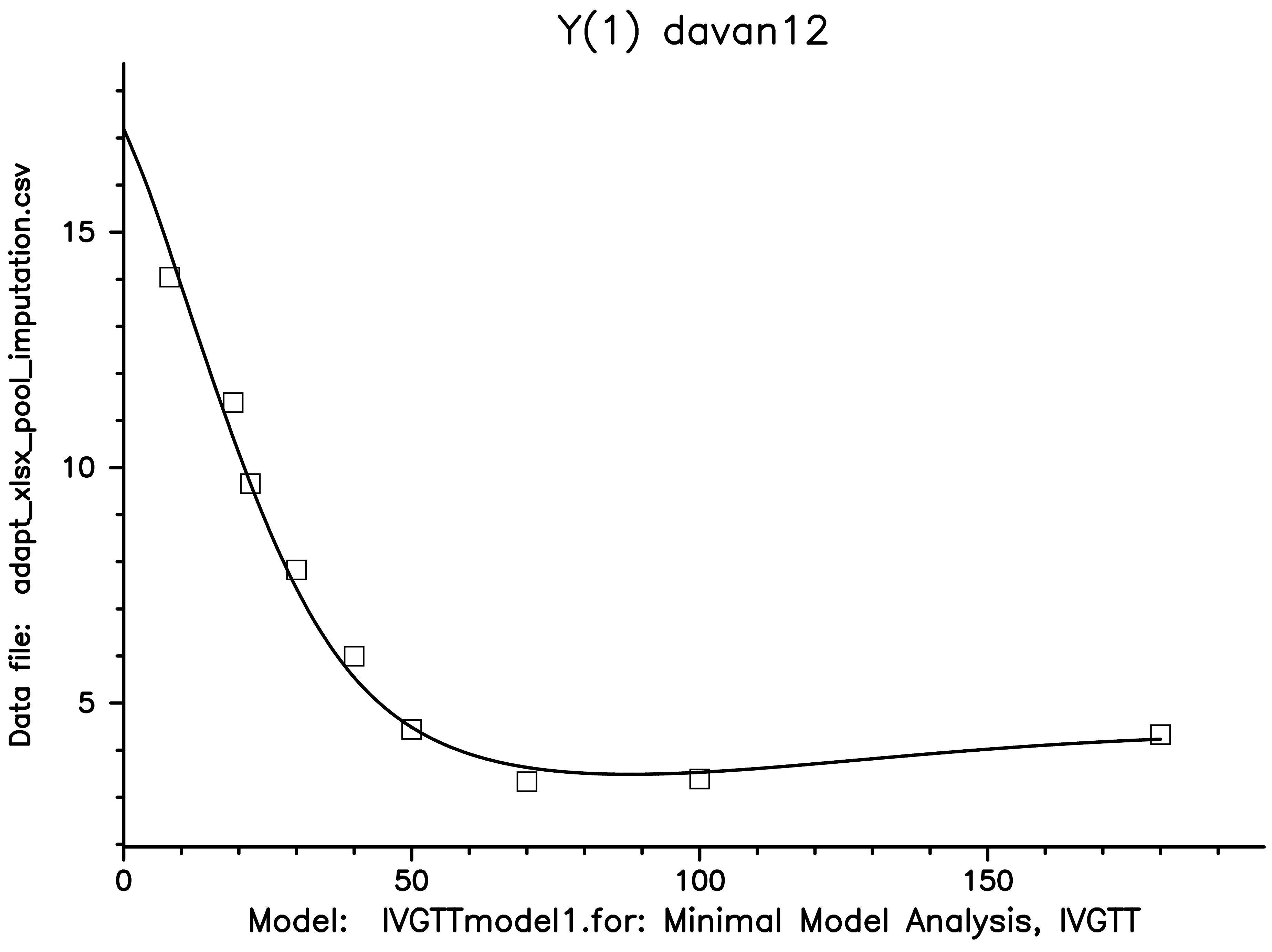
Y(1) davan12
Data file: adapt_xlsx_pool_imputation.csv
15
10
5
0
50
100
150
Model: IVGTTmodel1.for: Minimal Model Analysis, IVGTT

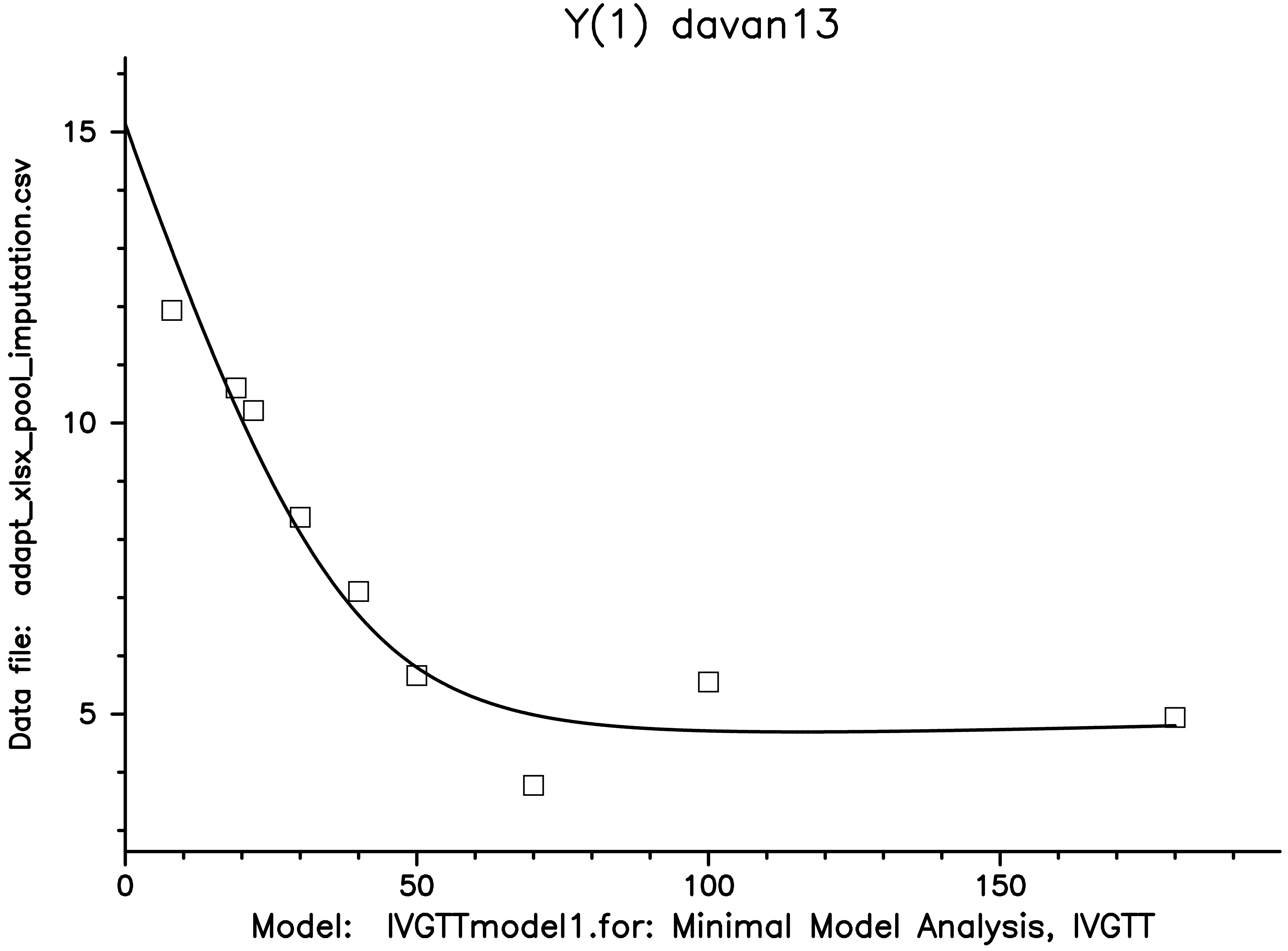

Y(1) davan13
Data file: adapt_xlsx_pool_imputation.csv
15
10
5
0
50
100
150
Model: IVGTTmodel1.for: Minimal Model Analysis, IVGTT

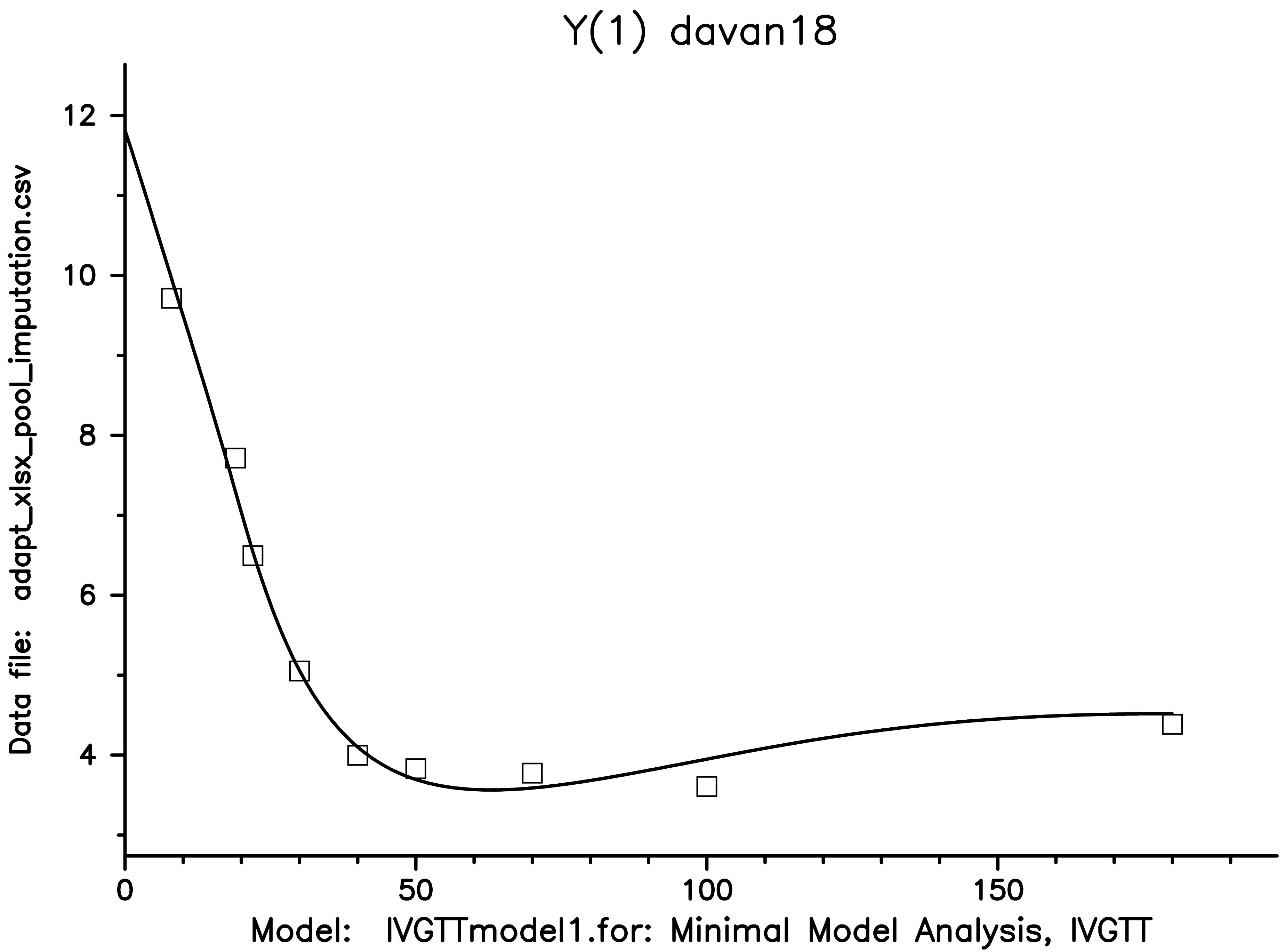
Y(1) davan18
Data file: adapt_xlsx_pool_imputation.csv
12
10
8
6
4
0
50
100
150
Model: IVGTTmodel1.for: Minimal Model Analysis, IVGTT

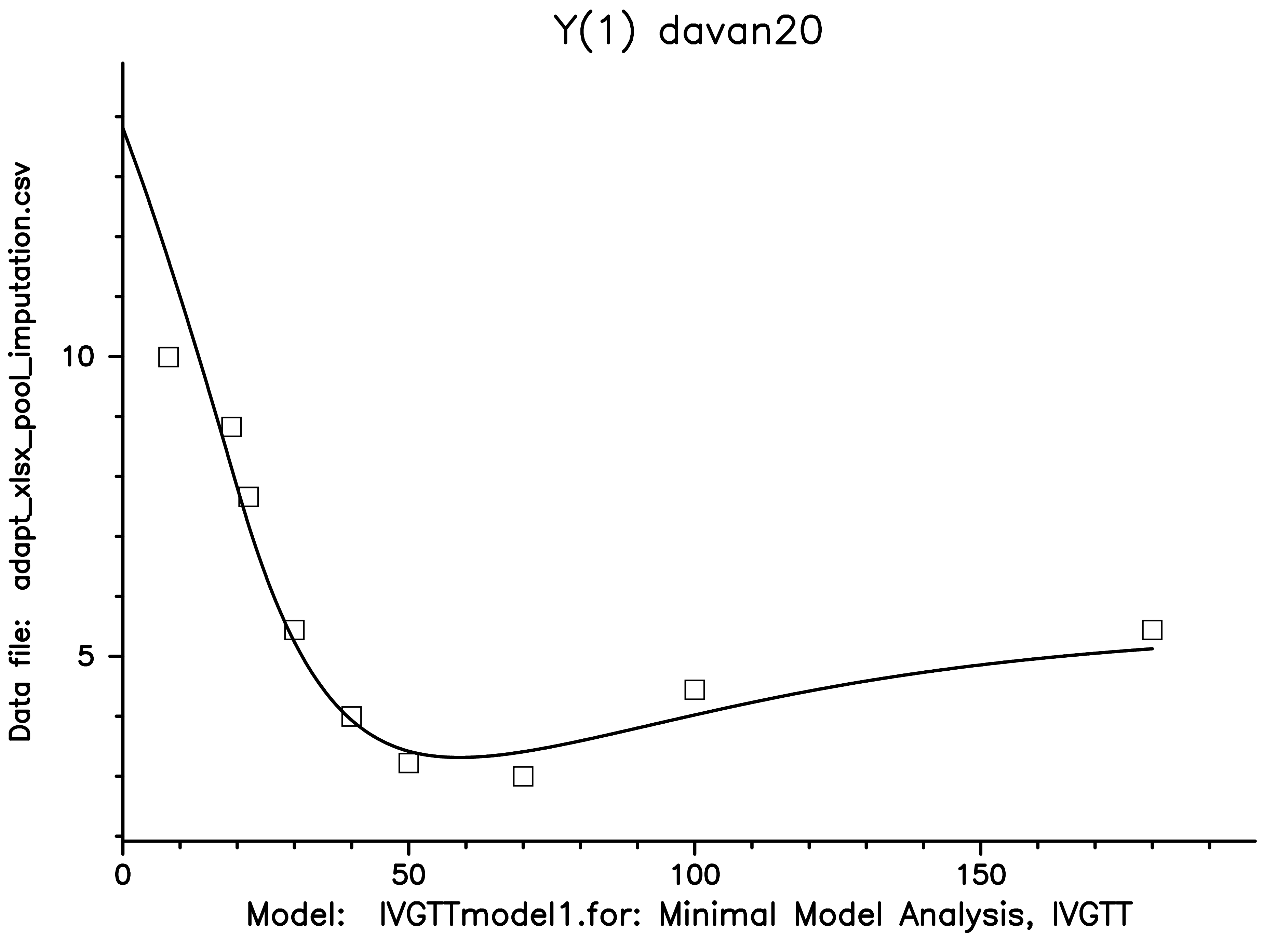
Y(1) davan20
Data file: adapt_xlsx_pool_imputation.csv
10
5
0
50
100
150
Model: IVGTTmodel1.for: Minimal Model Analysis, IVGTT

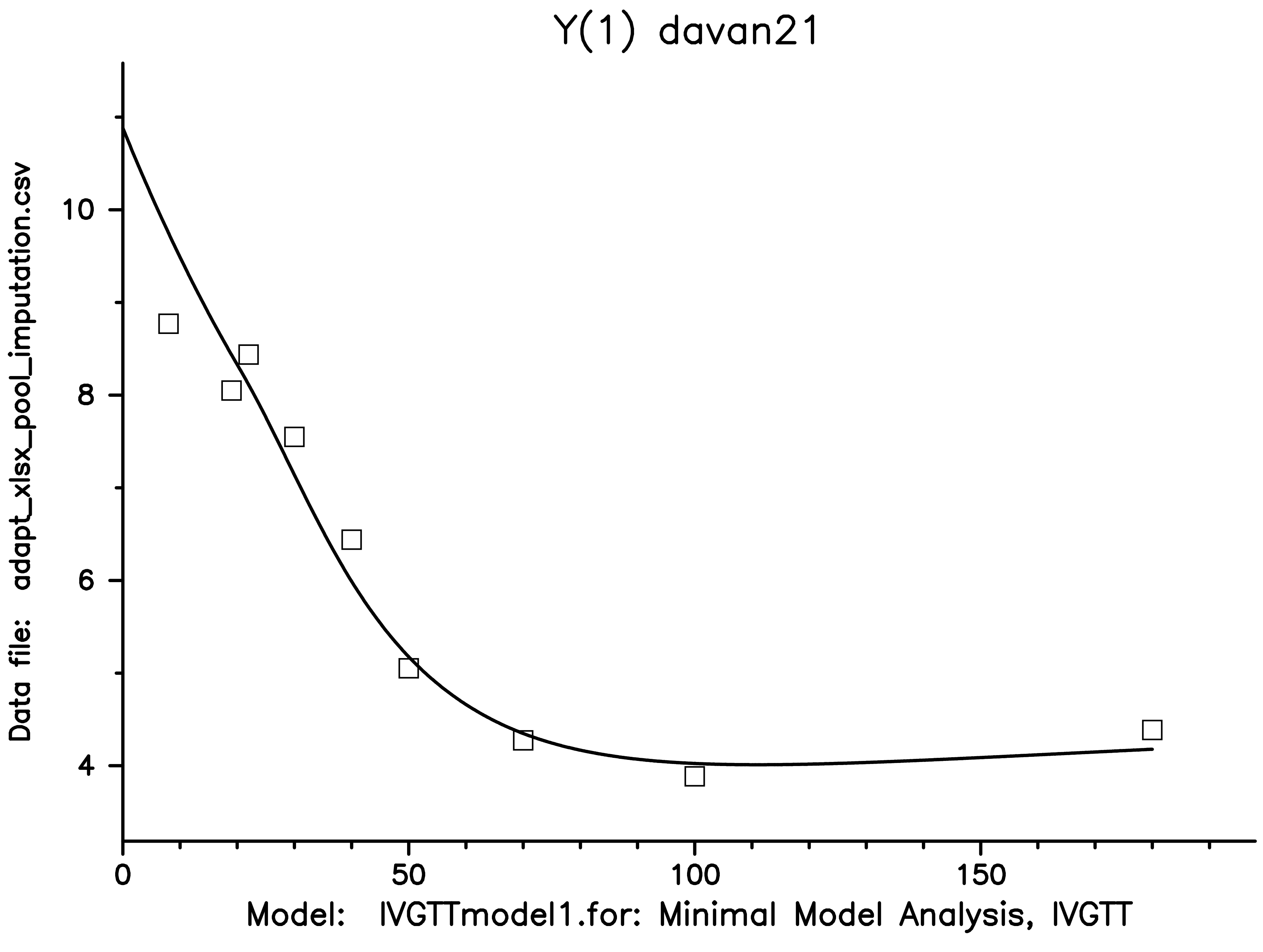

Y(1) davan21
Data file: adapt_xlsx_pool_imputation.csv
10
8
6
4
0
50
100
150
Model: IVGTTmodel1.for: Minimal Model Analysis, IVGTT

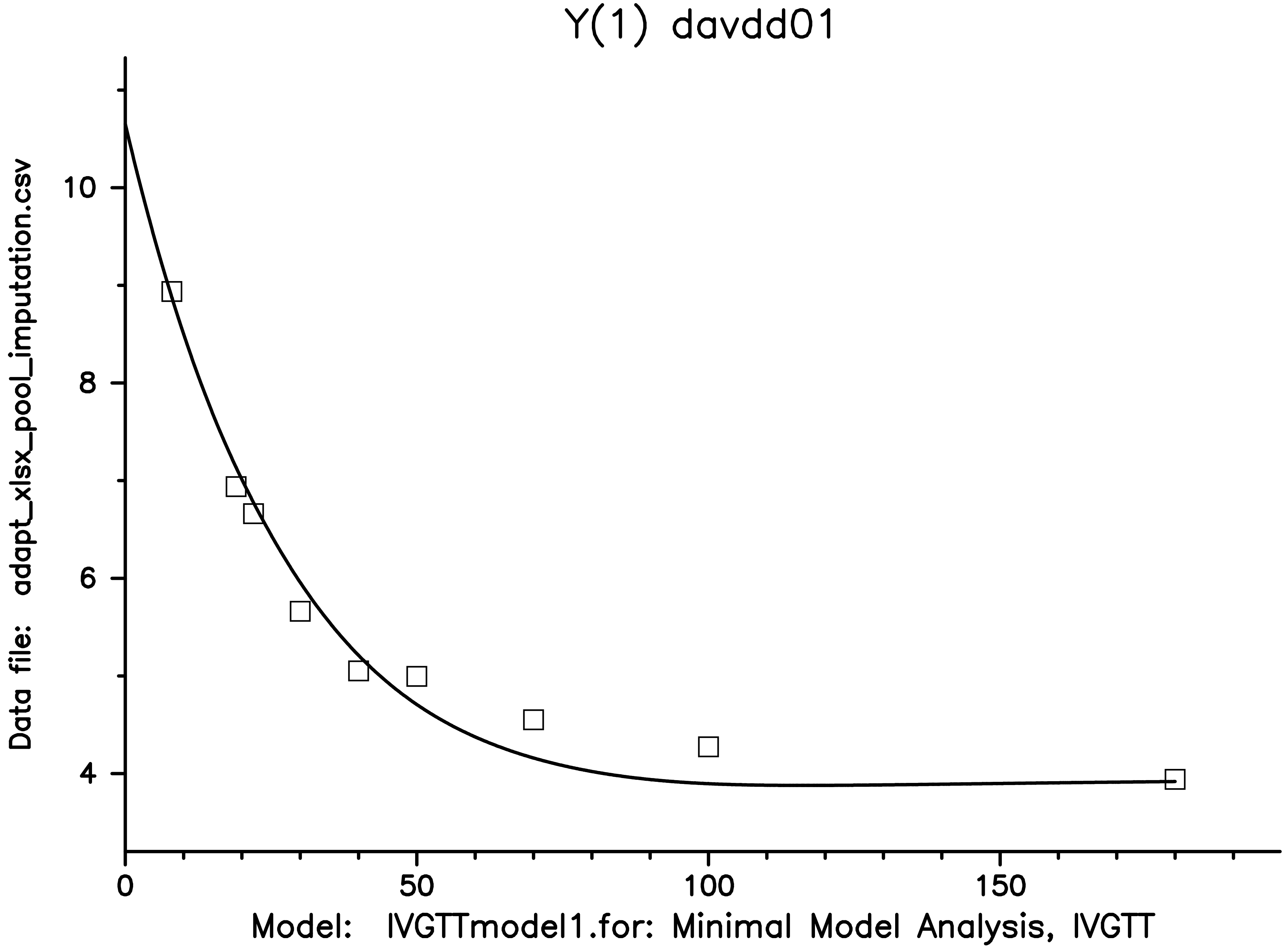
Y(1) davdd01
Data file: adapt_xlsx_pool_imputation.csv
10
8
6
4
0
50
100
150
Model: IVGTTmodel1.for: Minimal Model Analysis, IVGTT

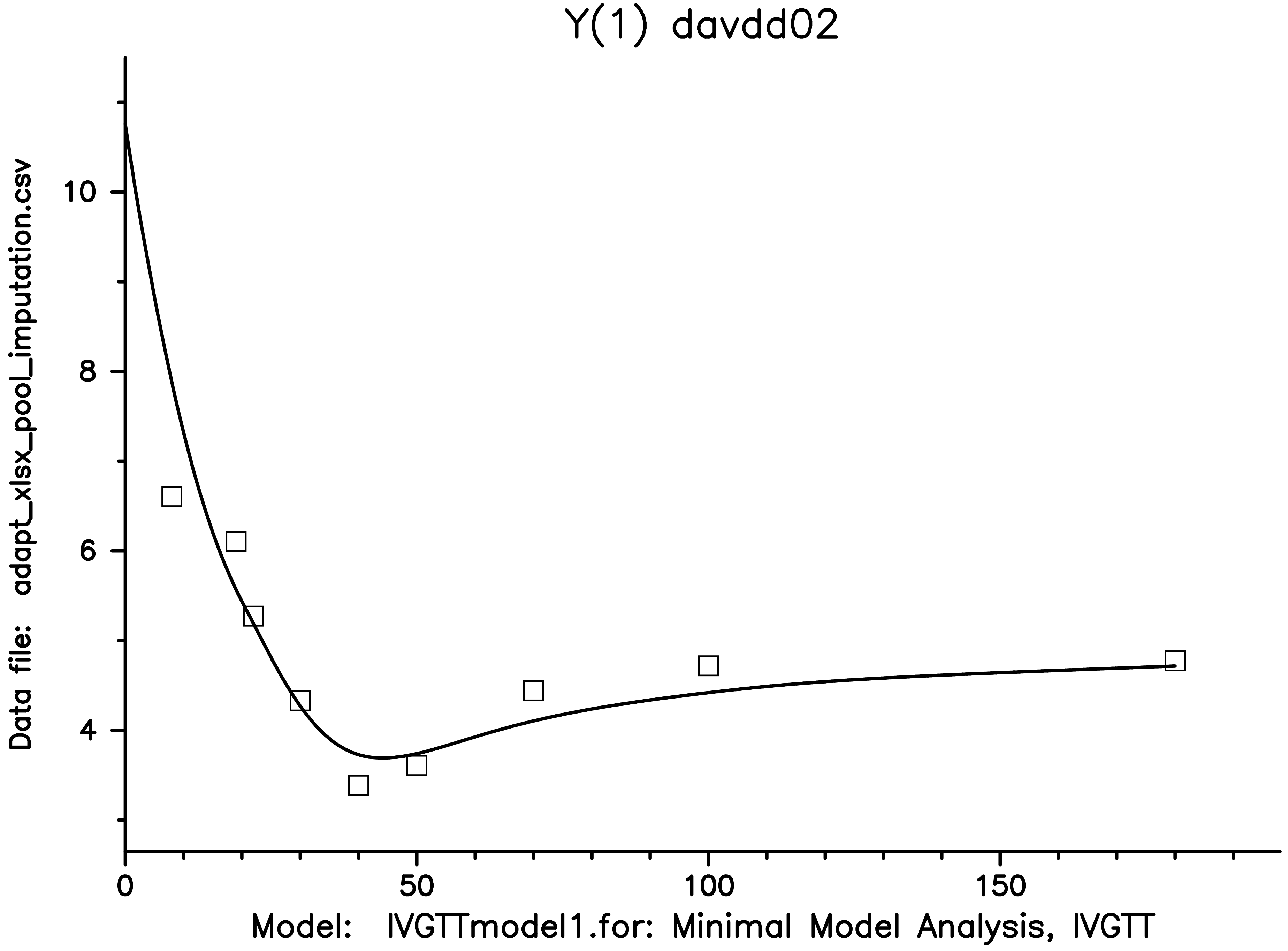
Y(1) davdd02
Data file: adapt_xlsx_pool_imputation.csv
10
8
6
4
0
50
100
150
Model: IVGTTmodel1.for: Minimal Model Analysis, IVGTT

Y(1) davdd03

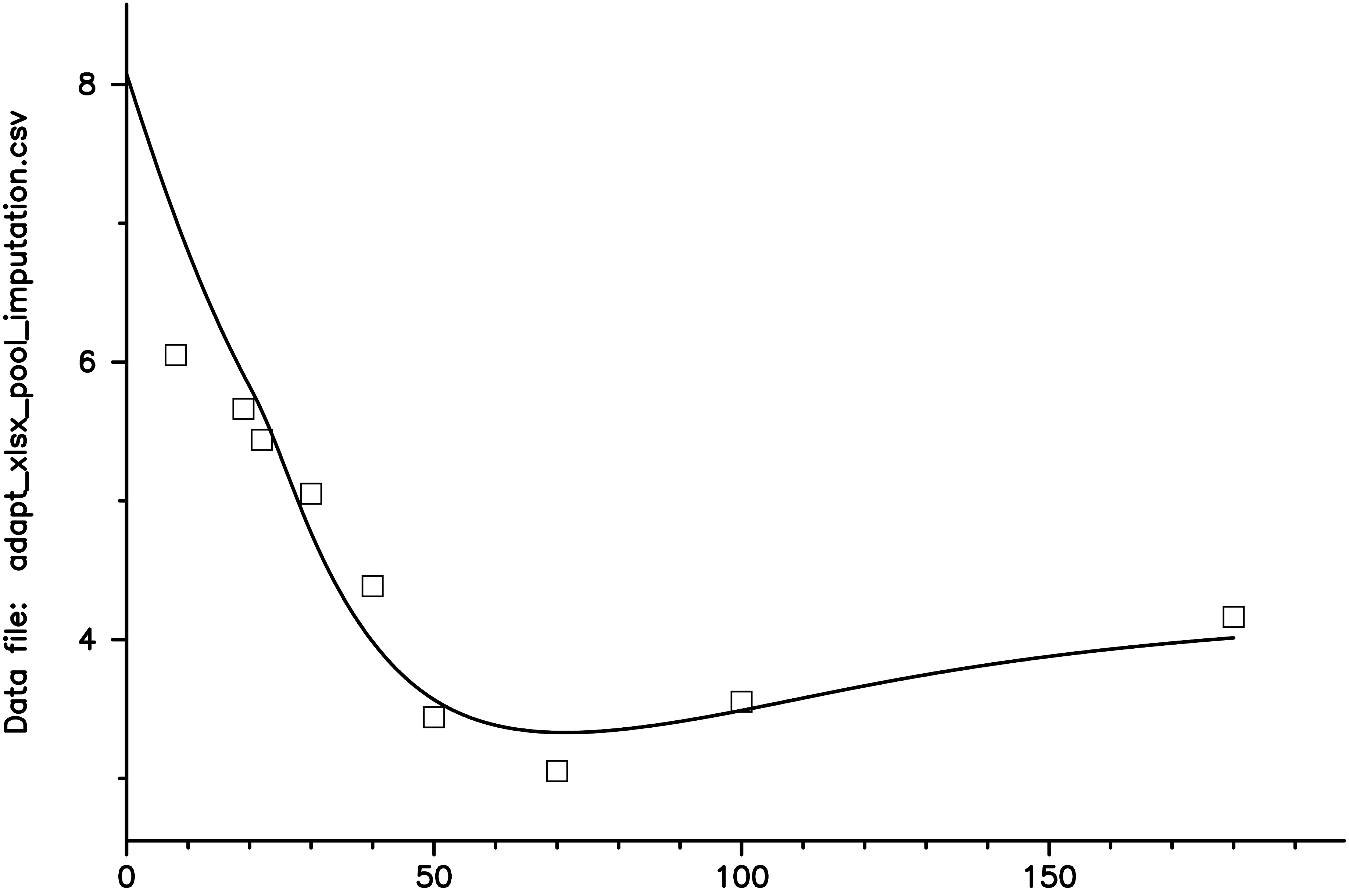

8
6
4
0
50
100
150
Data file: adapt_xlsx_pool_imputation.csv
Model: IVGTTmodel1.for: Minimal Model Analysis, IVGTT

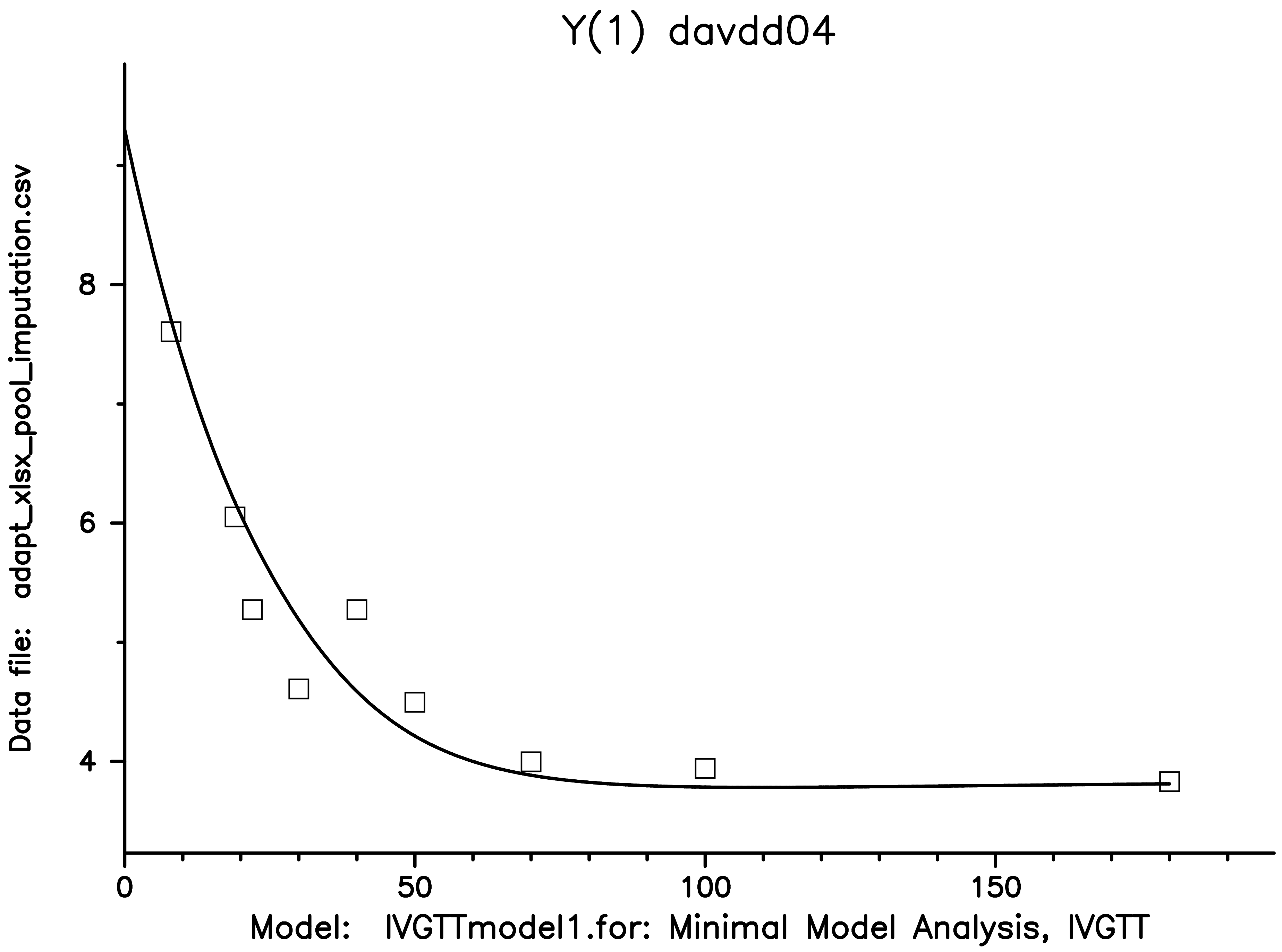

Y(1) davdd04
Data file: adapt_xlsx_pool_imputation.csv
8
6
4
0
50
100
150
Model: IVGTTmodel1.for: Minimal Model Analysis, IVGTT

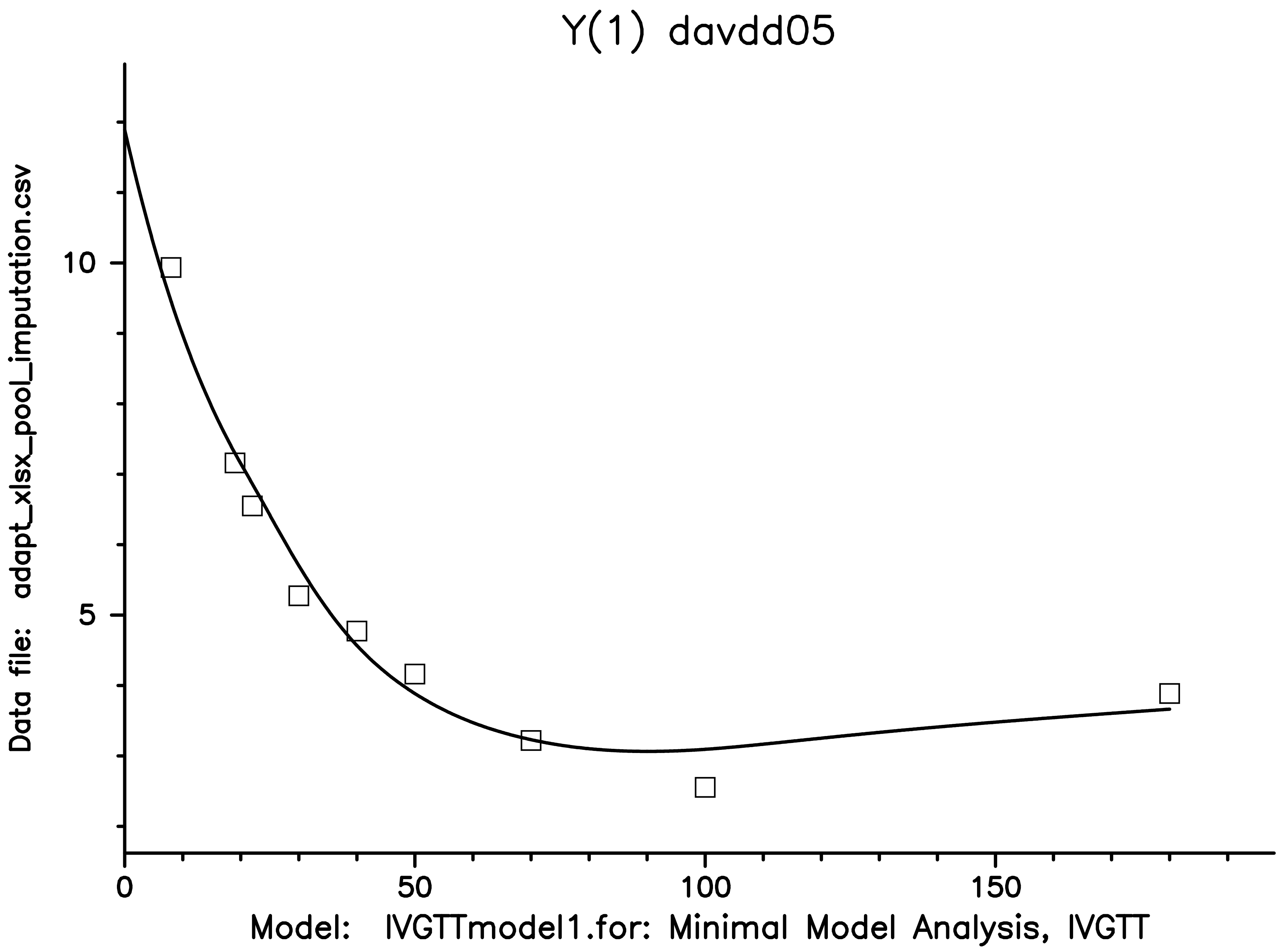

Y(1) davdd05
Data file: adapt_xlsx_pool_imputation.csv
10
5
0
50
100
150
Model: IVGTTmodel1.for: Minimal Model Analysis, IVGTT

Y(1) davdd06

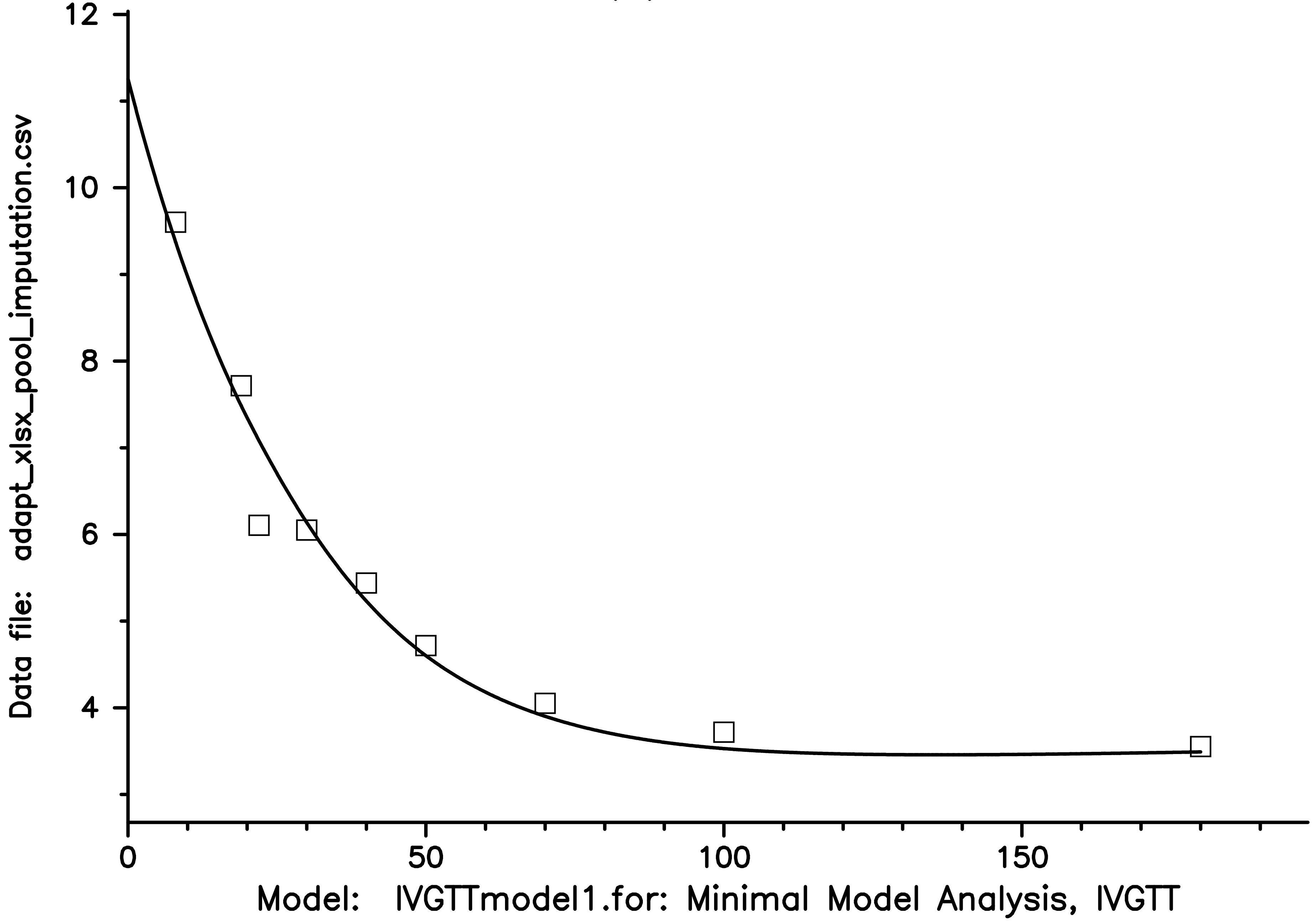

12
10
8
6
4
0
50
100
150
Data file: adapt_xlsx_pool_imputation.csv
Model: IVGTTmodel1.for: Minimal Model Analysis, IVGTT

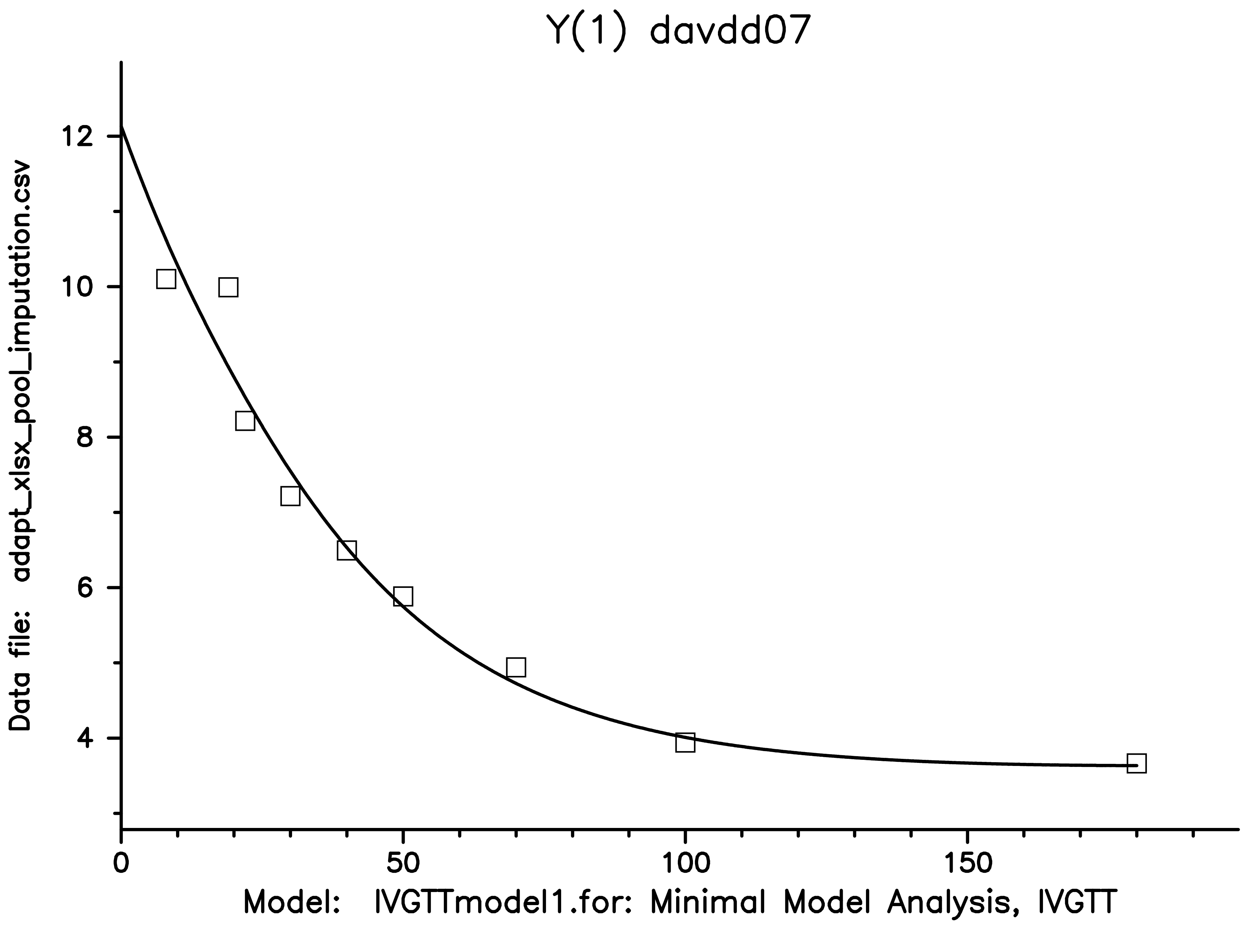

Y(1) davdd07
12
10
8
6
4
0
50
100
150
Data file: adapt_xlsx_pool_imputation.csv
Model: IVGTTmodel1.for: Minimal Model Analysis, IVGTT

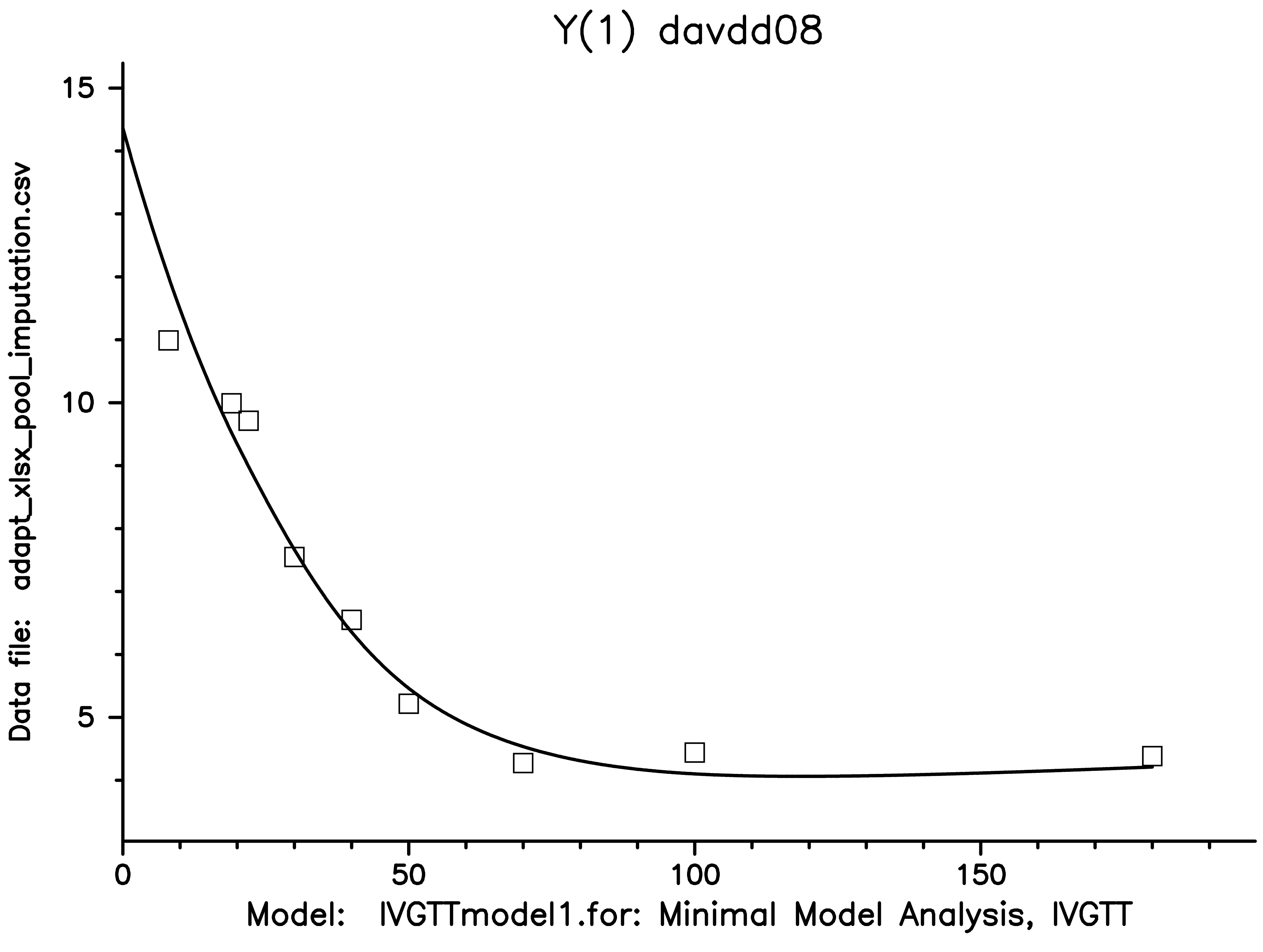
Y(1) davdd08
Data file: adapt_xlsx_pool_imputation.csv
15
10
5
0
50
100
150
Model: IVGTTmodel1.for: Minimal Model Analysis, IVGTT

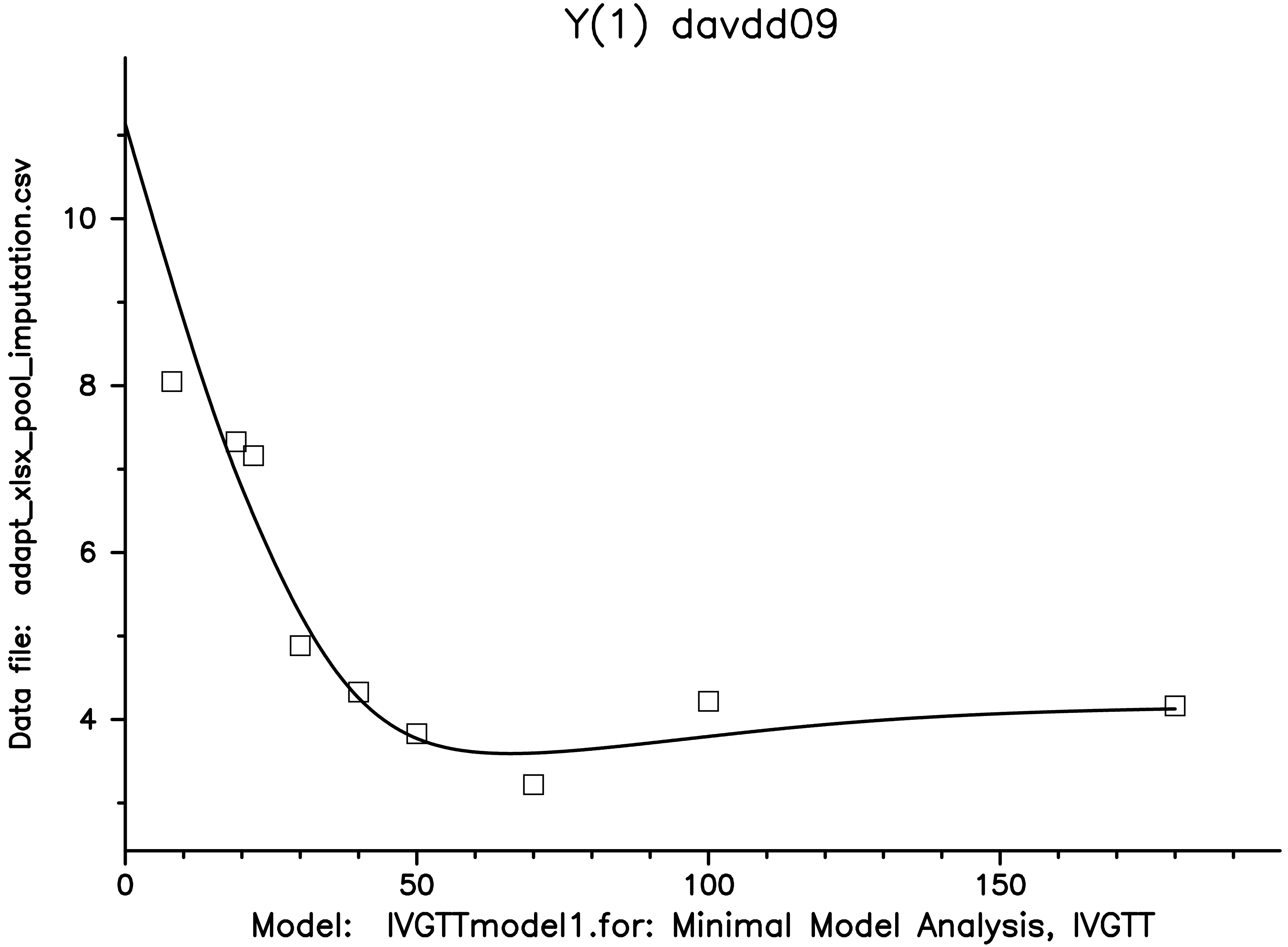
Y(1) davdd09
Data file: adapt_xlsx_pool_imputation.csv
10
8
6
4
0
50
100
150
Model: IVGTTmodel1.for: Minimal Model Analysis, IVGTT

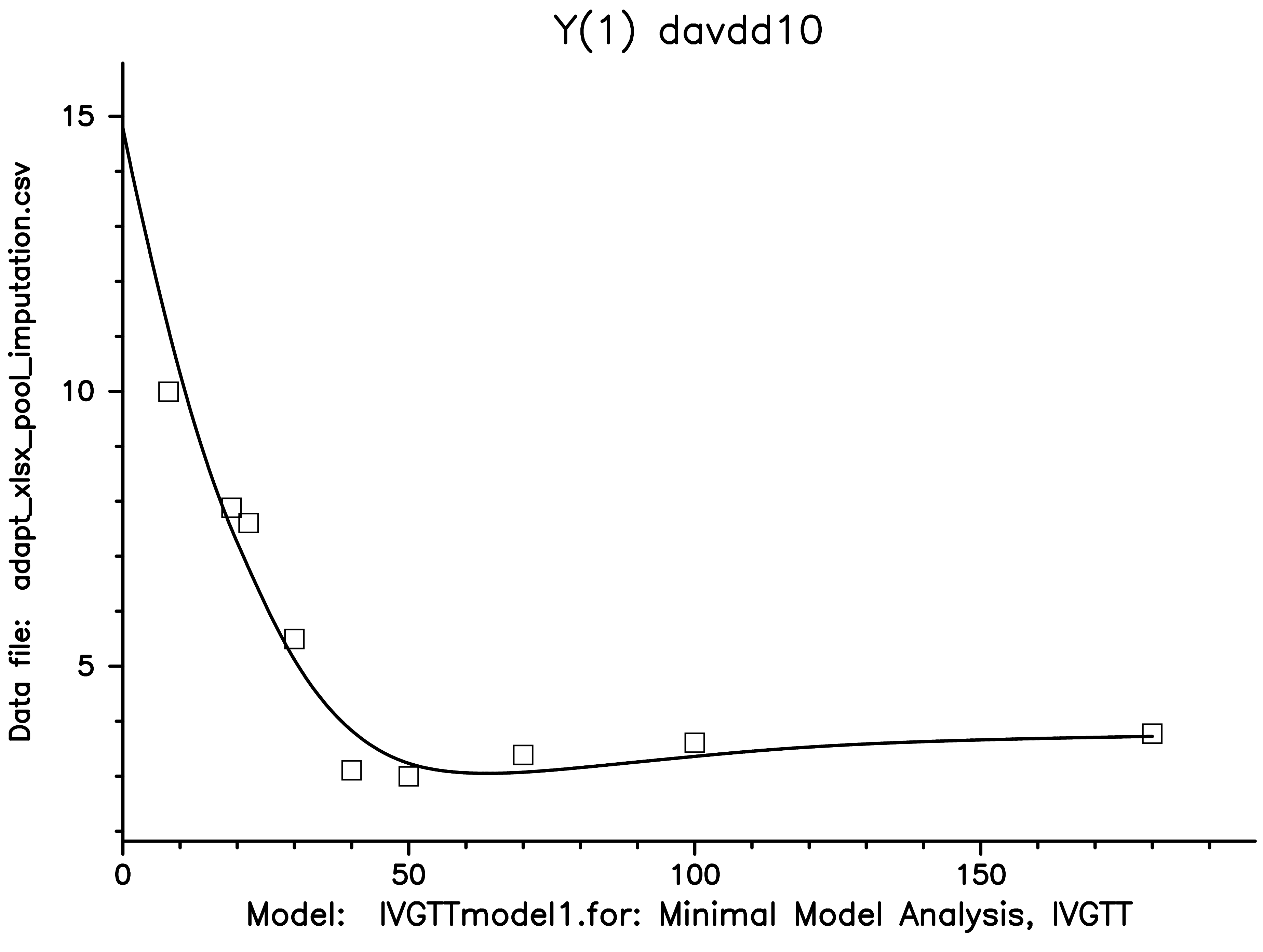
Y(1) davdd10
Data file: adapt_xlsx_pool_imputation.csv
Model: IVGTTmodel1.for: Minimal Model Analysis, IVGTT
15
10
5
0
50
100
150

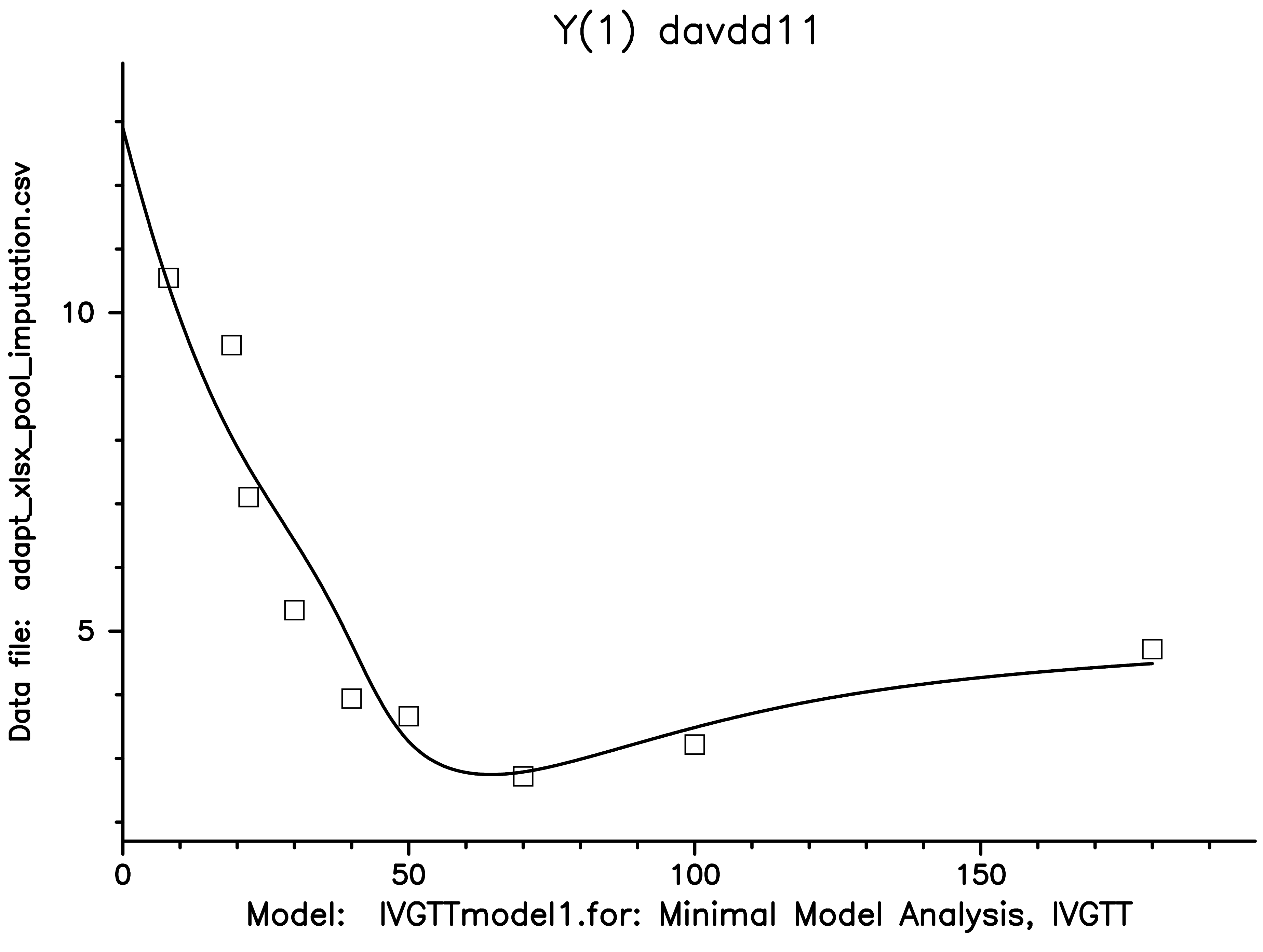
Y(1) davdd11
Data file: adapt_xlsx_pool_imputation.csv
10
5
0
50
100
150
Model: IVGTTmodel1.for: Minimal Model Analysis, IVGTT
